# Comparison of Two Methods for Detecting Alternative Splice Variants Using GeneChip^®^ Exon Arrays

**Published:** 2011-09

**Authors:** Wenhong Fan, Derek L. Stirewalt, Jerald P. Radich, Lueping Zhao

**Affiliations:** 1 *Division of Public Health Sciences, Fred Hutchinson Cancer Research Center, 1100 Fairview Ave. N., Seattle, WA 98109, USA;*; 2 *Clinical Research Division, Fred Hutchinson Cancer Research Center, 1100 Fairview Ave. N., Seattle, WA 98109, USA*

**Keywords:** alternative splicing, exon, gene expression analysis

## Abstract

The Affymetrix GeneChip Exon Array can be used to detect alternative splice variants. Microarray Detection of Alternative Splicing (MIDAS) and Partek^®^ Genomics Suite (Partek^®^ GS) are among the most popular analytical methods used to analyze exon array data. While both methods utilize statistical significance for testing, MIDAS and Partek^®^ GS could produce somewhat different results due to different underlying assumptions. Comparing MIDAS and Partek^®^ GS is quite difficult due to their substantially different mathematical formulations and assumptions regarding alternative splice variants. For meaningful comparison, we have used the previously published generalized probe model (GPM) which encompasses both MIDAS and Partek^®^ GS under different assumptions. We analyzed a colon cancer exon array data set using MIDAS, Partek^®^ GS and GPM. MIDAS and Partek^®^ GS produced quite different sets of genes that are considered to have alternative splice variants. Further, we found that GPM produced results similar to MIDAS as well as to Partek^®^ GS under their respective assumptions. Within the GPM, we show how discoveries relating to alternative variants can be quite different due to different assumptions. MIDAS focuses on relative changes in expression values across different exons within genes and tends to be robust but less efficient. Partek^®^ GS, however, uses absolute expression values of individual exons within genes and tends to be more efficient but more sensitive to the presence of outliers. From our observations, we conclude that MIDAS and Partek^®^ GS produce complementary results, and discoveries from both analyses should be considered.

## INTRODUCTION

The Affymetrix GeneChip^®^ Exon Array (Affymetrix, Santa Clara, CA) is designed to evaluate expression of individual exons within genes and has been used to detect alternative splice variants (1-3). Both Microarray Detection of Alternative Splicing (MIDAS) (4) and Partek^®^ Genomics Suite (5) are among the commonly used methods for analyzing exon array data. When applying both methods to analysis of the same data set (6), we have noted that they produce different results (unpublished data), which could create controversies in practice. Here we intend to compare these two software methods from the perspective of the generalized probe model (GPM) that was published by our group (7). As a general model, GPM can be simplified to be equivalent to either MIDAS or Partek^®^ GS, depending on which assumptions are made.

**Figure 1:**
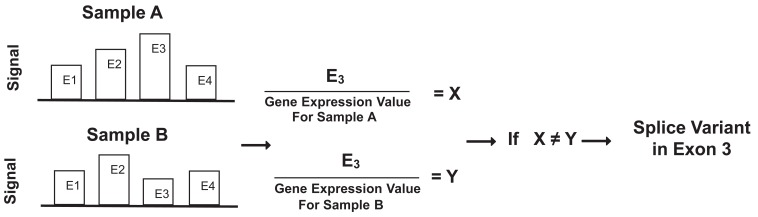
Schematic diagram to illustrate alternative splice variant detection in MIDAS.

MIDAS software assumes that relative changes in exon signals within genes are predictive of alternative splice variants. Under this assumption, MIDAS divides an individual exon signal by its gene expression value. In other words, it creates a gene normalized exon signal by computing ratios between individual exons and whole gene signals. Hereafter, we refer such ratios as “ratio signals.” In MIDAS, the ratio signal for each exon is used to compare exons to one another between comparison groups using analysis of variance (ANOVA). Implicitly such an ANOVA further assumes that the error distribution on the relative scale is normal on the logarithmic scale. Using variations detected by ANOVA, MIDAS predicts which exon is likely to have alternative splicing. Figure 1 illustrates how each exon signal is divided by its gene expression value to obtain the ratio signal. X and Y are the ratio signals for exon 3 in samples A and B, respectively. If X significantly differs from Y, a splice variant exists in exon 3 between samples A and B.

**Figure 2:**
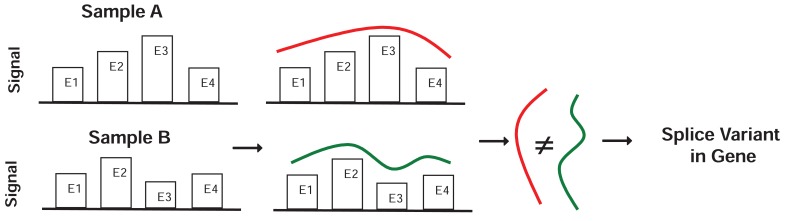
Schematic diagram to illustrate alternative splice variant detection in Partek^®^ GS.

In contrast, Partek^®^ GS directly uses the exon signals to detect differences in the expression of exons in the ANOVA. It then declares the presence of relevant alternative splice variants when one or more exons appear to have different expression mean values between the two comparison groups. Figure 2 illustrates the basic strategy underlying Partek^®^ GS. Essentially it detects significant differences in the exon signals across all exons in the gene between the comparison groups. Implicitly assumed in Partek^®^ GS is the normally distributed error for the raw intensity values on the logarithmic scale.

While both software methods are designed for analyzing exon array data, there are some fundamental differences. Firstly, definitions of alternative splice variants are different at the conceptual level. MIDAS conceptualizes the presence of alternative splice variants via the relative changes of exon-specific expression values within a gene, while Partek^®^ GS assumes that alternative splice variants are absolute changes of exon-specific expression values within a gene. Secondly, MIDAS focuses on the presence of alternative splice variants at the individual exon level so that it generates a *p*-value for each exon in the statistical testing. Partek^®^ GS simply detects the presence of alternative splice variants within a gene, and it therefore produces a *p*-value for each gene. Thirdly, the underlying statistical assumptions on stochastic processes are rather different between these two methods; MIDAS assumes an error distribution for relative changes, and Partek assumes an error distribution for raw intensity values. The software methods are therefore substantially different, presenting challenges to scientists who desire to examine the biological relevance of alternative splice variants. Resolving their differences is the primary motivation for us to consider using GPM to analyze exon array data.

While GPM was recently proposed for analyzing probe-level data from GeneChip^®^ gene expression array data (7), it is rather general and encompasses statistical models used by MIDAS and Partek^®^ GS under their respective assumptions. Fundamentally, GPM is a linear regression model and is directly applicable to exon array data by equating “probe” with “exon.” Because it uses the generalized estimating equation (GEE) technique (8), GPM is particularly suitable for modeling multiple correlated exon expression values within genes. Under the typical multivariate normal distribution assumption, the GPM method produces efficient estimates. Efficiency in the current context refers to statistical efficiency, where a higher efficiency method obtains the same conclusion with relatively fewer samples. GPM encompasses the statistical method in MIDAS because the ANOVA method used in MIDAS is a special case of GPM as long as relative exon-specific expression signals are used in the analysis. Similarly, GPM encompasses Partek^®^ GS. This is also because the multivariate ANOVA used in Partek^®^ GS is a special case of GPM as long as exon-specific expression values are used and a single gene-specific parameter is used to identify alternative splice variants.

In addition to the above reasoning, it is important to demonstrate differences and similarities between these two software methods on an actual data set. For this purpose, we used an empirical data set that was generated from a panel of colon normal and cancerous tissues produced by researchers at Affymetrix (6). We applied MIDAS and Partek^®^ GS, together with GPM, to analyze the colon cancer data set. To eliminate the influence of data pre-processing, we used PLIER in the Affymetrix Power Tools (APT) Software Package (9) to produce both exon signals and gene expression values. We then compared results from MIDAS and Partek^®^ GS with GPM, and results from GPM under different assumptions.

## METHODS

### Data sets

The colon cancer data set includes 20 paired normal/tumor samples from colon tissue (6). Affymetrix Human Exon 1.0 ST array contains over one million exons covering the entire human genome. Exons are categorized into three groups: core content, extended content and full content. For our comparison, the core content exons were used because they had the most supportive evidence in the annotation databases.

### Running MIDAS

MIDAS statistics were calculated using the software provided by Affymetrix in the APT packages. MIDAS uses the two-sample t-test, or 1-way ANOVA, to detect alternative splicing exon by exon. Let Y denote the ratio of the exon signal over the gene expression. MIDAS 1-way ANOVA model can be written as:

(a) $$\log\left(Y\right)\left|T=\mu +T+\varepsilon \right.$$

where *μ* quantifies the baseline mean value, T quantifies the difference between the treatment groups, i.e. tumor versus normal in the colon tumor dataset, and *ε* denotes a normally distributed error. This error is assumed to be independent after accounting for the difference between tumor and normal tissues. The p-values and F-statistics were output into separate files for each exon.

### Running Partek^®^ GS

Partek GS models exon signal directly using mixed ANOVA. For the colon tumor dataset, its model can be written as:

(b) $$\log\left(Y\right)\left|T,E,P=\mu +T+P+E+T^{*}E+S\left(P^{*}T\right)+\varepsilon \right.$$

where *Y* is the exon signal (not the ratio as in MIDAS), *μ* estimates the baseline mean value, *T* estimates the difference between the treatment groups (i.e. differential gene expression parameter), *P* estimates the patient random effect, *E* estimates the Exon effect, *S*(P*T) is the sample random effect which is nested in tissue type and the patient variable, and ε denotes a normally distributed error. Besides the independence across individuals, errors from multiple exons within the same transcript cluster were assumed to be independent. Such conditional independence allows one to derive a joint multivariate normal distribution so that relevant parameters and their standard errors can be estimated. Note that we added the log-stabilizing factor of 8 to the data prior to the logarithmic transformation, to keep raw data as consistent as possible with the MIDAS calculation. We invoked the ALT-SPLICE ANOVA option under the STAT menu per its manual. P-values and F-statistics on the exon and tissue interactions were extracted from Partek^®^ GS outputs.

### Running GPM

GPM was developed for probe-level analysis on the GeneChip^®^ gene expression array data on which multiple probes are used to interrogate one gene. Purely from the modeling perspective, a typical GPM can be written as the linear model used in Partek^®^ GS. To acknowledge the dependence among multiple exons within a transcript cluster, GPM can be formally defined as

(c) $$\left(\begin{array}{c} \log\left(Y_{1}\right)\\ \log\left(Y_{2}\right)\\ ...\\ \log\left(Y_{n}\right) \end{array}\right)=\left(\begin{array}{c} \mu_{1}\\ \mu_{2}\\ ...\\ \mu_{n} \end{array}\right)+\left(\begin{array}{c} \beta_{1}\\ \beta_{2}\\ ...\\ \beta_{n} \end{array}\right)T+\left(\begin{array}{c} \varepsilon_{1}\\ \varepsilon_{2}\\ ...\\ \varepsilon_{n} \end{array}\right)$$

where *n* is used to denote the number of exons, μ’s are mean intensity values for every exon, and β’s are differences between tumor and normal tissues where differences greater than zero imply the presence of alternative splice variants. The residuals *ε’* = (*ε_1_ ε_2_ … ε_n_*) are assumed to be independent across subjects, but are correlated among themselves. Due to using the estimating equation, we did not have to assume a multivariate distribution for these residuals. Once estimates were obtained, we computed their covariance matrix, and hence Wald-statistics to test if all β’s equaled zero.

There are two pertinent remarks regarding use of GPM. In connection with the linear models underlying Partek^®^ GS, one could start with the linear model (b) and compute mean structures. Those random effects are the primary sources contributing to the covariance matrix of multiple exons within the transcript cluster. Specification of their mean structure and covariance matrix allows one to adopt the same estimating equation technique without requiring any normality assumption. The second point is relating to the application of GPM to pair matched observations. The simplest way for GPM to incorporate such a design is to replace the outcome vector with the corresponding differences between exon intensity values (on a log scale), and then to model the association of differences with the tissue type. More generally, if multiple tissue samples are obtained from the same subject, one would expand the above model (c) to include replicates in a straightforward fashion. Again, one does not have to make any unverifiable distributional assumption, resulting in robust estimations and inferences.

## RESULTS

### MIDAS versus Partek^®^ GS

As noted above, MIDAS and Partek^®^ GS are the two most commonly used software methods for detecting alternative splice variants. We ran each analysis using the corresponding default settings, using the Bonferroni corrected *p*-value of 0.05 as the significance level. In this exercise, MIDAS yielded no signals for any alternative splice variants. On the other hand, Partek^®^ GS identified 368 transcript clusters (Table OL1; also see detail information on test statistics and annotation in additional files 1 and 2 in supplemental material online at www.ijbs.org). A transcript cluster includes multiple exons within the same transcriptional locus. It is conceptually equivalent to a gene but may not have the exactly same boundaries. For example, the Akt3 transcript cluster has many probe sets that fall outside the region annotated by the RefSeq database. We use gene and transcript cluster interchangeably in this paper.

Since MIDAS produces statistics on the exon level and Partek^®^ GS on the gene level, it is not feasible to compare their statistics directly. Instead we focused our comparisons on top candidate gene lists selected from both analyses. By ranking *p*-values from MIDAS, we picked the top 462 most significant exons in MIDAS, because these probes are from a list of 363 unique transcript clusters (Table OL2; also see detail information on test statistics and annotation in additional files 3 and 4 in supplemental material online at www.ijbs.org). The choice of 363 transcript clusters is comparable to 368 transcript clusters identified by Partek^®^ GS. Comparing these two lists resulted in a total of 152 transcript clusters that overlap (Table OL3; also see detail information on test statistics and annotation in additional files 5 and 6 in supplemental material online at www.ijbs.org).

**Figure 3:**
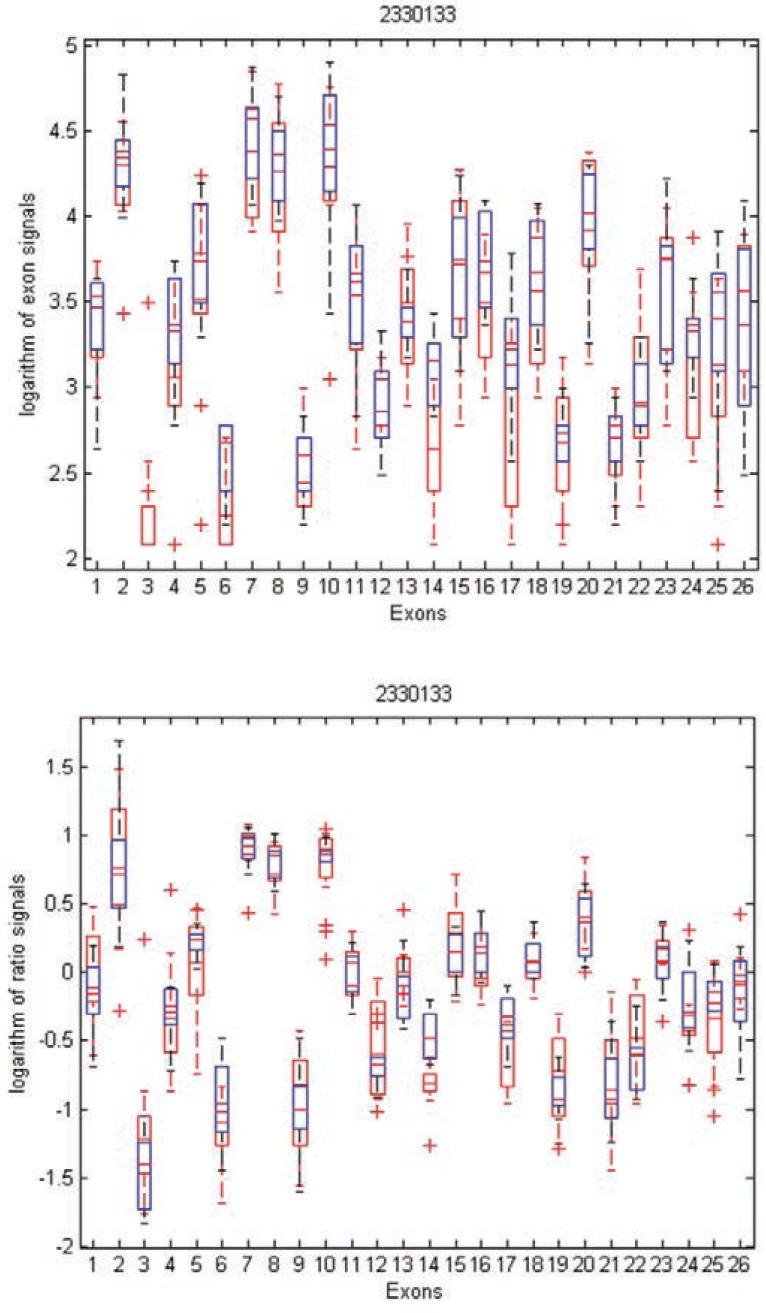
Box plots of exon signals before (top) and after (bottom) normalization by gene expression value for transcript cluster ID 2330133. The x-axis is the individual exons within a gene, and the y-axis is the exon signals (top) or ratio signals (bottom), on logarithm scale.

**Figure 4:**
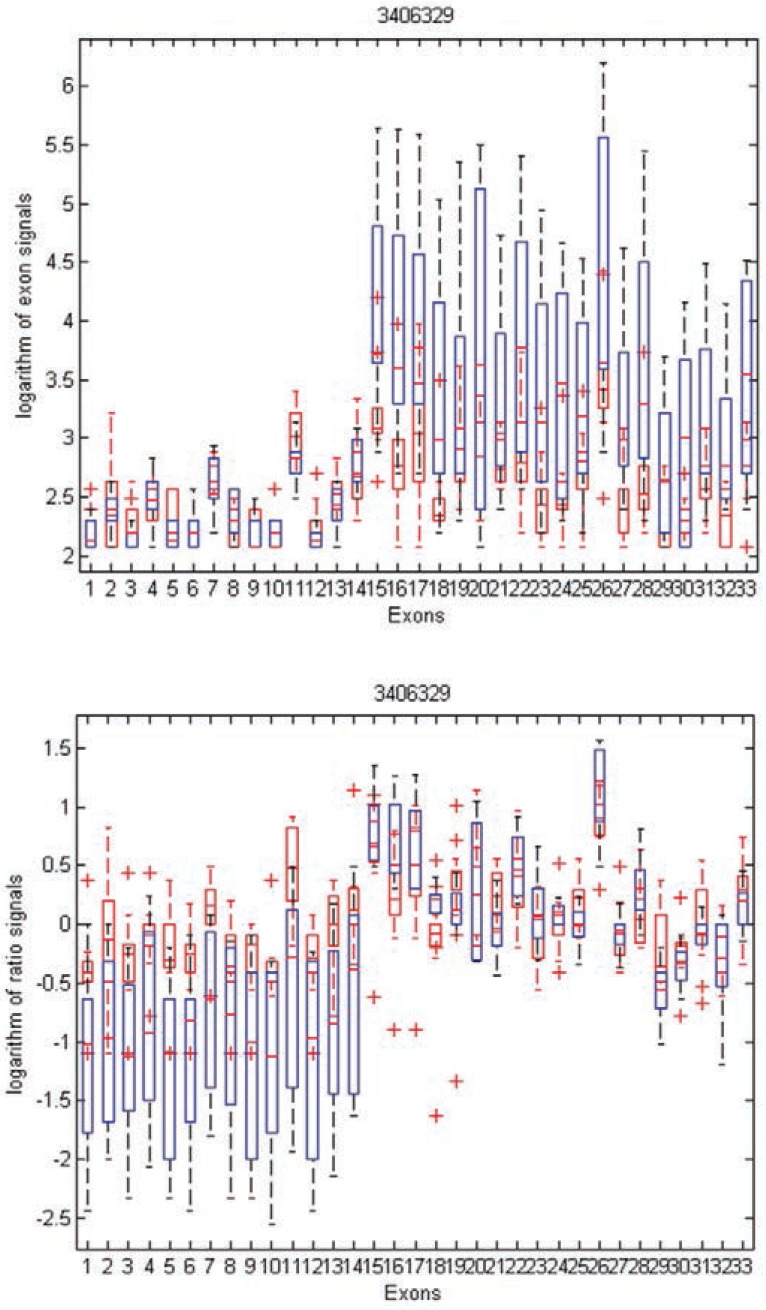
Box plots of exon signals before (top) and after (bottom) normalization by gene expression value for transcript cluster ID 3406329. The axes are the same as in Figure 3.

**Figure 5:**
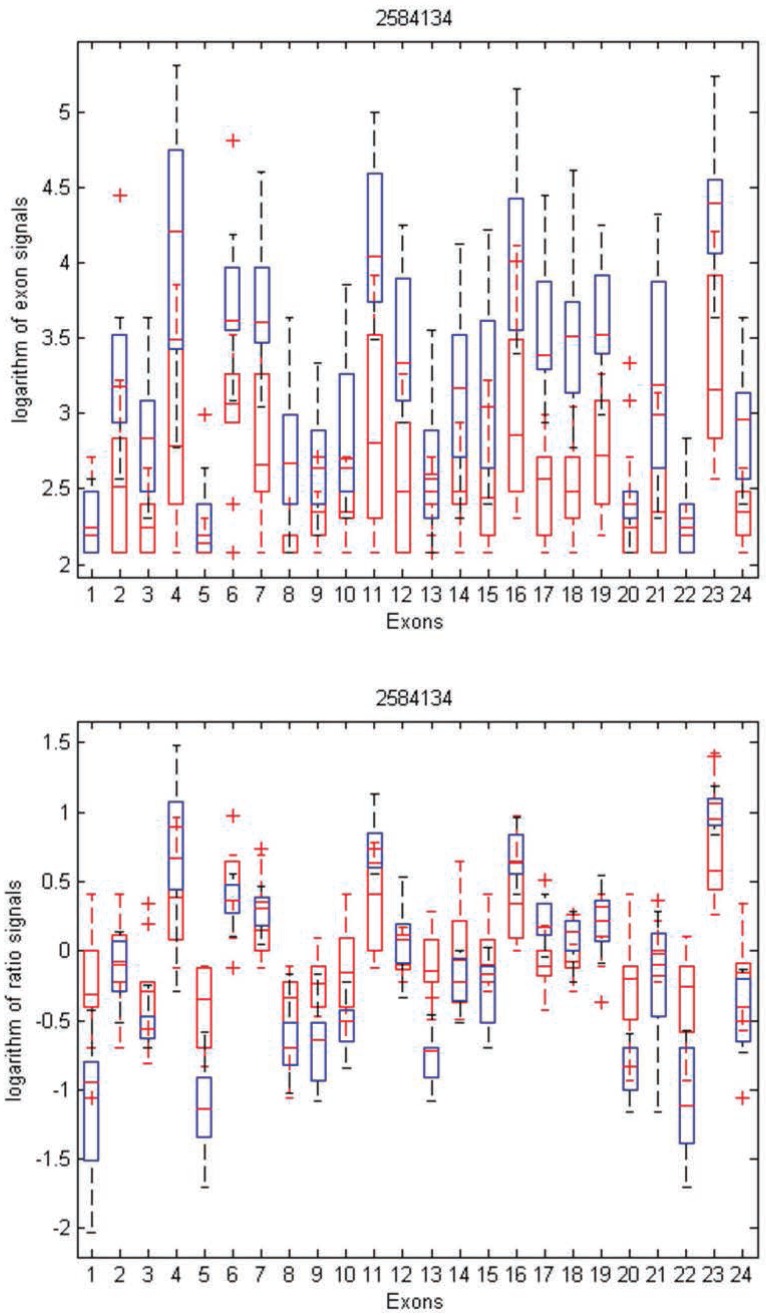
Box plots of exon signals before (top) and after (bottom) normalization by gene expression value for transcript cluster ID 2584134. The axes are the same as in Figure 3.

To gain insight into the differences and similarities between these two methods, we identified three examples in distinct scenarios: transcript clusters selected by MIDAS only, by Partek^®^ GS only, and by both. As shown in Figures 3, 4, and 5, we plotted the ratio signals from MIDAS (bottom panel) and the exon signals from Partek^®^ GS analysis (top panel) for each exon in three transcript clusters. In Figure 3, for transcript cluster 2330133, exon 14 has a *p*-value of 0.04 and had been selected by MIDAS into the top 462 significant exons, but the transcript cluster was not selected by Partek^®^ GS. Exon 14 was selected due to significant ratio signal difference between normal (red) and tumor (blue) tissues. From the top panel, one can see that the exon signals for exon 14 are also quite different between normal and tumor tissue. Transcript cluster 2330133 was not selected by Partek^®^ GS because this method tests the entire set of exons in a transcript cluster, not each individual exon. In Figure 4, transcript cluster 3406329 was selected by Partek^®^ GS, but none of the exons in this transcript cluster had *p*-values sufficiently small to be in the top 462 clusters selected by MIDAS. For this transcript cluster, it seems that expression from the first half of the exons (1-14) was lower than those from the second half (15-33) in both normal and tumor tissues. Expression values for the second half exons were lower in normal than in tumor tissues, but this was not the case for the first half exons. This transcript cluster was selected by Partek^®^ GS because the expression patterns changed from the first half exons to the second half in this transcript cluster between the normal and tumor tissues. In MIDAS, analyzing the ratio signals for each exon in transcript cluster 3406329 did not reveal any significantly different exons between normal and tumor tissues. In Figure 5, transcript cluster 2584134 was selected by both Partek^®^ GS and MIDAS. MIDAS reported that exon 13 had a *p*-value of 0.025. Partek^®^ GS also detected alternative splicing patterns in this transcript cluster.

### MIDAS versus GPM using ratio signals

**Figure 6:**
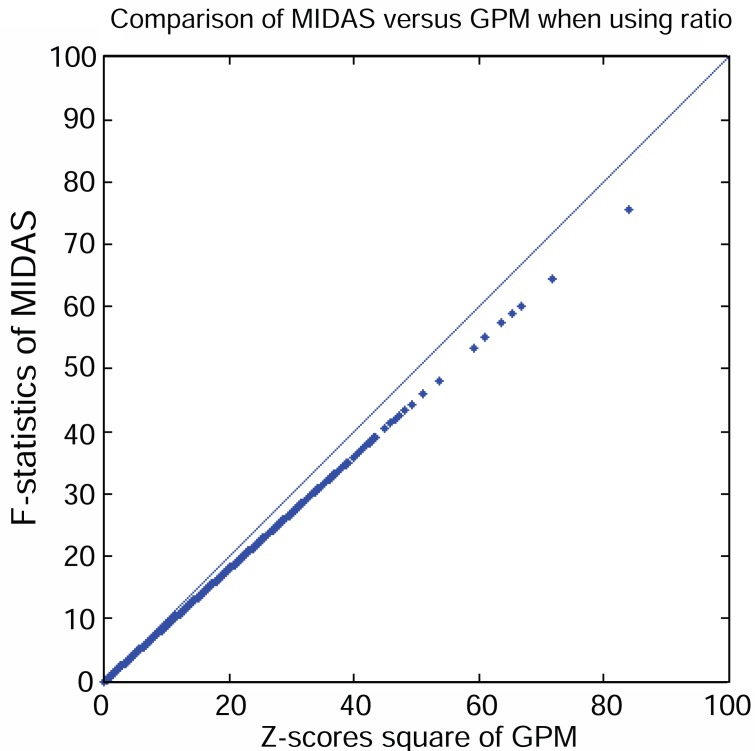
XY plot of square of Z scores from GPM versus F-statistics from MIDAS. Number of data points plotted equal to the number of exons in the core content of the GeneChip^®^ Exon array.

Pertaining to earlier discussion, GPM encompasses MIDAS if the same assumption is made, i.e. if it uses the ratio signals to detect alternative splice variants exon by exon. For comparison, we computed F-statistics for a two-group comparison on each exon using MIDAS. Conceptually, the F-statistics for a two-group comparison are equivalent to the squared Z-scores from GPM analysis, when it is applied to the two-group analysis. After obtaining both F-statistics and squared Z-scores, we plotted the F-statistics from MIDAS against square of Z-scores from GPM, as shown in Figure 6. Both were highly correlated over the range of 0 to 100. However, it is of interest to note that the pairs of observed statistics do not fall on the diagonal line, suggesting a small but systematic difference between their calculations. Without knowing the implementation details, we speculate that the MIDAS calculation may have modified variance estimates in the F-statistics, which has been recommended in the literature in order to avoid “small variances.” In contrast, GPM strictly follows the asymptotic results without incorporating such a factor because adding an arbitrary “variance-stabilizing” constant could reduce the power of detecting subtle differences even when gene expression levels are relatively low.

### Partek^®^ GS versus GPM using exon signals

**Figure 7:**
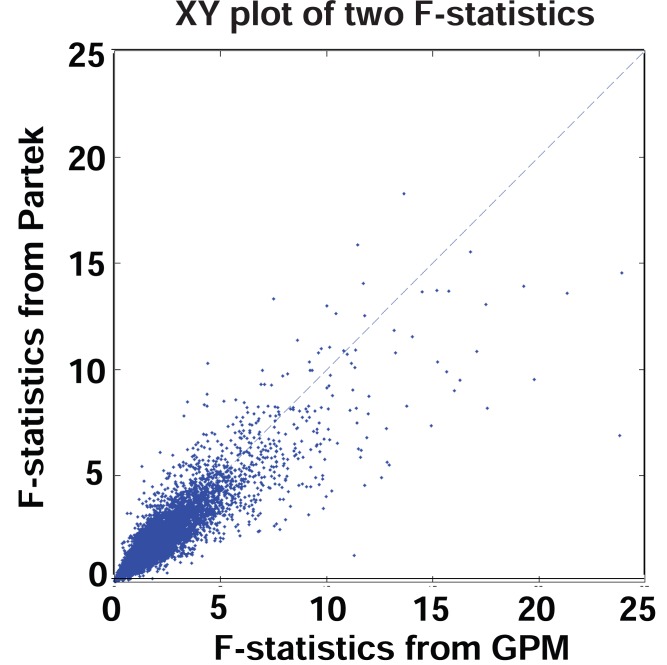
XY plot of F-statistics from GPM versus F-statistics from Partek^®^ GS. Number of data points plotted equal to the number of transcript clusters in the core content of the GeneChip^®^ Exon array.

Instead of detecting alternative splicing exon by exon, Partek^®^ GS interrogates multiple exons within transcript clusters, and tests whether there is evidence for alternative splicing based on all exon-specific expression values within the entire transcript cluster. For the colon cancer data set, Partek^®^ GS tested whether the interaction between tissue type and Exon ID deviated from zero in an ANOVA model based on F-statistics. With the same analytic objective, we also tested overall differences of exon expression levels between tissues via a Wald statistic from the GPM. In the current context of a two-group comparison, our Wald statistic is equivalent to the F-statistic. Figure 7 shows a XY plot for F-statistics generated from Partek^®^ GS and Wald statistics from GPM. While they did not show high correlation as those between MIDAS and GPM, the F- and Wald statistics from Partek^®^ GS and GPM fell on the same diagonal line. This level of consistency indicates that both Partek^®^ GS and GPM captured the same underlying information. Inconsistencies between Partek^®^ GS and GPM are largely associated with the F-statistic calculation. Recall that multivariate ANOVA calculations typically have to assume a certain dependence structure in the multivariate normal distribution, and this could be violated in many transcript clusters. On the other hand, GEE estimates tend to be more sensitive to small sample size. Given respective weaknesses in the two methods, it is appropriate to view that their results are complementary.

### Exon signals versus ratio signals within GPM

**Figure 8:**
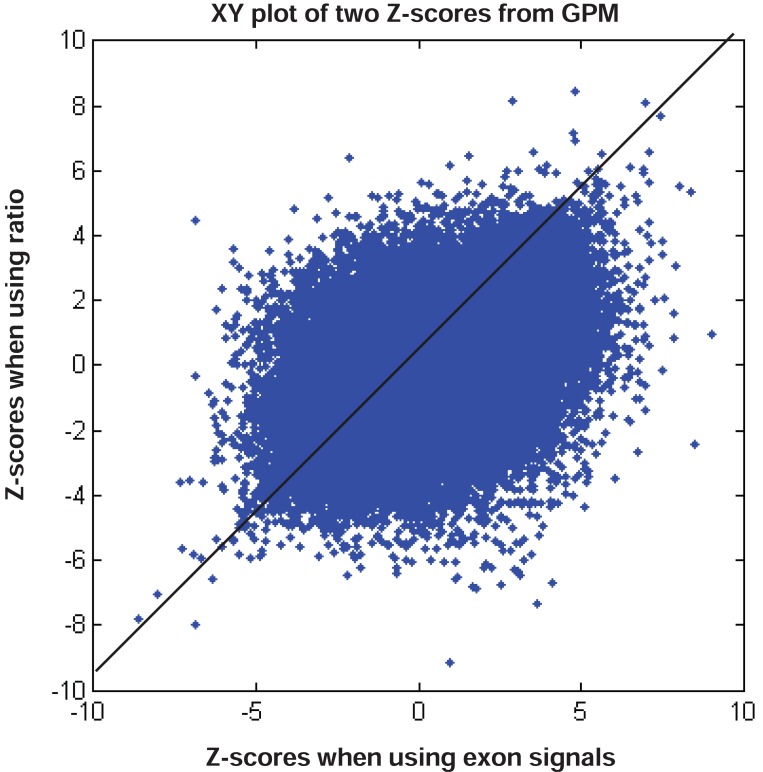
XY plot of Z scores when using exon signals versus Z scores when using ratio signals, both in GPM. Number of data points plotted equal to the number of exons in the core content of the GeneChip^®^ Exon array.

**Figure 9:**
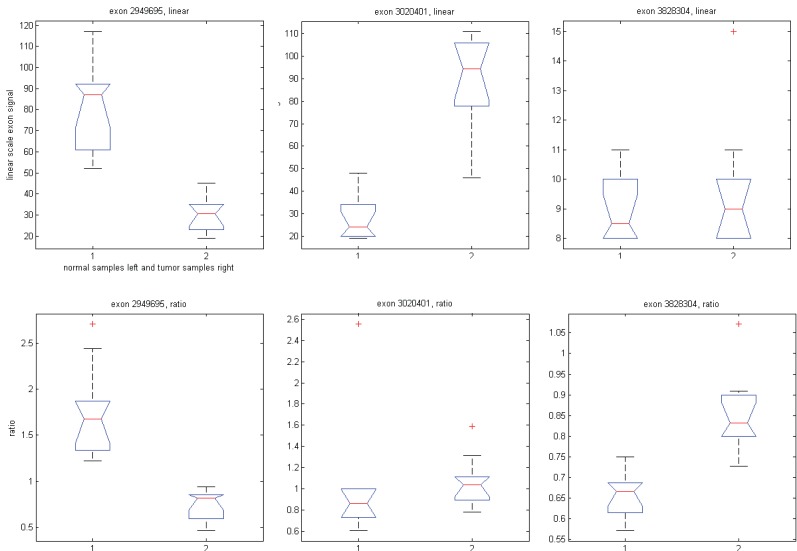
Box plots of exon signals before (top panel) and after (bottom panel) normalization by gene expression value for three exons.

We have shown that GPM produces results similar to MIDAS and Partek^®^ GS under respective assumptions. To shed light on the differences noted when comparing MIDAS and Partek^®^ GS, we used the same GPM to analyze exon array data with both exon signals and ratio signals. Using exon signals, we generated a vector of Z-scores (Z_1_), where each Z-score corresponds to an exon on the exon array. Differentially expressed exons indicate alternative splicing. The larger the absolute Z-scores, the more likely the corresponding exons are alternatively spliced between the normal and tumor tissues. Similarly, we get Z-scores (Z_2_) when using ratio signals. Figure 8 shows a XY plot for (Z_1_, Z_2_) pairs of all exons for the core content in the exon array. The two statistics appear to have limited concordance. To gain insight into their differences, we considered three specific examples, as shown in Figure 9. When exon signals were used, the Z-scores were -8.63, 9.01 and 0.94 for exons 2949695, 3020401 and 3828304, respectively. The Z-scores became -7.82, 0.97 and 6.17 when ratio signals were used as input. The Z-score for exon 2949695 remained similar, but the Z-score for exon 3020401 changed from significant, when exon signals were used, to non-significant, when ratio signal were used. The reverse scenario occurred for exon 3828304. Ratio signals measure the exon signals relative to their gene expression values. The statistics calculated from ratio signals could be different from those that were generated from the original exon signals.

## DISCUSSION

One of the important applications of the GeneChip^®^ Exon Array system is to detect alternative splicing, and this data can be analyzed using novel software methods. In this paper we compared MIDAS and Partek^®^ GS, the two most commonly used software methods for analyzing alternative splice variants using GeneChip^®^ Exon Array data. To ensure an unbiased comparison, we used GPM as a general framework, since it encompasses statistical methods used in both software packages under respective assumptions. We demonstrated the utility of these methods by taking an empirical approach, via analyzing the colon cancer data set. While preliminary, our comparisons have given important insights on some of the similarities and differences between MIDAS and Partek^®^ GS.

From a statistical methods point of view, using exactly the same dataset as input for statistical inference, both methods will produce very similar results because they are both special cases of linear regression models. What makes them different is how the signals are quantified from the probe hybridization intensities. It appears that the single most significant factor that explains the differences between MIDAS and Partek^®^ GS is the difference in scale used to conceptualize the alternative splice variants and the method for quantifying such signals as input data, i.e. individual exon values versus relative changes (ratio signal). The second factor is that different ANOVA models are used to capture the alternative splicing events in MIDAS and Partek^®^ GS. MIDAS detects alternative splicing on the exon level by testing the significance for each exon, while Partek^®^ GS detects alternative splicing on the gene level by testing the significance of the interaction between tissue type and exon ID in the empirical colon cancer data set. The final factor is that MIDAS assumes a distribution for random variations on the logarithmic scale, while Partek^®^ GS assumes a random distribution for raw intensity values from individual exons.

Since exon array technology remains at an early stage, there is no scientific rationale for favoring one methodology over the other. Instead it may be wise for practitioners to appreciate the advantages and disadvantages of both software methods. For MIDAS, the primary advantages of using ratio signals include that the analysis tends to be robust at the level of overall expression values and that the detection is at the level of individual exons. However use of ratio signals tends to be less efficient, neither benefiting from absolute expression values, which have greater variations, nor borrowing information from multiple exons within single genes. On the other hand, Partek^®^ GS uses exon-specific expression values that are potentially more variable, and hence it produces more efficient estimates. Additionally, borrowing information from multiple exons enhances the efficiency in detecting alternative variants. Disadvantages include that raw expression values tend to be influenced by other technical variations, including cross-hybridization and sample-to-sample variation. Also, the statistical assumptions relating to multiple exons tend to be restrictive and are easily violated, where violations could impact the validity of statistical inferences.

One important issue left unaddressed is how to deal with alternative splice signals when the overall gene expression is close to or at background. Theoretically such signals could confound the evaluation with respect to alternative splice variants, since corresponding alternative signals should be treated as “missing” regardless of their intensity values. This issue will impact results from both methods. However it is likely that corresponding signals will be biased towards null by both methods. Hence, the presence of this issue is unlikely to alter the results of our comparisons here. Additionally, the recent recommendation from both Affymetrix and Partek is to filter out those exons, which would reduce the impact of “false alternative splice signals.”

There is an additional issue with our empirical comparison. As documented earlier, the colon cancer data set used for empirical comparison was collected from a pair-matched design with tumor and normal tissue samples taken from 10 individuals. It is desirable to retain such matching for downstream association analysis because matching reduces subject-specific variations. However we performed an unmatched analysis for the purpose of comparing these two methods because matched design was not feasible in the current implementation of MIDAS, even though Partek^®^ GS and GPM could accommodate such a design. If we incorporated the design into Partek^®^ GS and GPM but not MIDAS, the interpretations would be much less intuitive. For the purpose of empirical comparisons, our conclusions could be misled by this design issue. In any scientific exploration, of course, the analysis should acknowledge design.

While introducing new methodologies for analyzing exon array data is not the objective here, our exercise has indirectly supported the potential use of GPM as an alternative software method for many reasons. Firstly, GPM is sufficiently flexible to encompass a range of methods for analyzing exon array data, including those used in MIDAS and Partek^®^ GS. Secondly, it provides the flexibility for practitioners to use ratio signals or expression signals within the same statistical framework. Thirdly, one can perform the analysis at the individual exon level or at the individual gene level. Fourthly, it requires fewer statistical assumptions, which is particularly important if one wants to make inferences for multiple exons within genes.
