# Supplementary material for: Comparison of Two Methods for Detecting Alternative Splice Variants Using GeneChip® Exon Arrays
Source: Int J Biomed Sci. 2011 Sep;7(3):172–80. (PMC3614835)
Supplement: Supplementary file 1 [file IJBS-7-172_SD7.pdf]

Table OL1. Affymetrix Human Exon 1.0 ST Transcription ID for alternative spice variants detected by Partek GS

2425756	2949622	4004044	2961177	3630736	3581637	2730746	2740067
3604147	2652675	3653677	2584134	3296046	2891556	3666366	3102372
2924514	3020343	3023149	3358201	2371139	3406329	3301263	3939470
3422144	3694657	3193482	2970942	3047581	3881443	3610982	3013054
2786322	2999755	3490655	2570616	3768627	2574984	3888133	2746591
3907111	3632806	3023384	2710599	3662808	3569814	2451593	2907671
3105600	2897899	3930360	3292946	2411228	3942681	3046444	3643580
3508330	3049522	2931391	2438282	3110317	3556990	2692447	3855218
2362892	2985781	3497790	2914777	3638607	2599153	3311832	3703885
2605321	2735027	3784208	3910785	3664982	2987632	3881282	3265565
3150844	3682028	2710474	2585476	3385951	2591643	2413203	3388807
3735151	2429556	2709631	3168508	2882834	3428447	3607537	3442641
3845909	2398820	3510066	3911217	3258477	3758510	3773244	2997376
3157385	2692319	3733590	3933566	3913960	2345061	3151534	3304301
3952825	3996667	3474104	2440943	3756193	2487082	2375706	3592755
3933205	3484641	2409104	3326183	2533019	3125571	3388830	3499132
3573870	2889916	3710108	3399545	3891278	3895118	2662020	3853108
3409127	3893520	2852591	2714465	3881786	3148796	3265224	3252036
2345023	3338293	3964049	2461473	3332626	2443120	2531589	3597338
3454892	3472225	3658980	2712236	3394660	3776504	2461037	3783398
3605395	2528476	3494137	2954678	3388673	2398706	2946106	3830216
3985717	3887049	3652902	3134034	2474341	2376168	2633390	2779199
3504434	3750662	3742285	2625793	3903361	3129149	3677752	3871192
2958325	2609347	3092808	3252071	3144973	2816459	3106243	2611848
2409820	3960478	3821263	3726934	3210013	2788926	3069366	2560076
3751859	3581485	2909263	3332424	3748798	3791782	3226883	3868828
3238962	3741800	3815399	3998766	2695941	2796995	3568616	3699634
2728938	2818517	3685329	2358320	3175274	3025545	3728776	3317352
3728964	3079803	2727762	3230760	3428845	3884100	2676009	3888217
2886679	3850069	2842624	3778772	2830638	3222170	3873160	3250237
2447877	3664924	2654023	2676182	3219215	3972093	2604254	3643396
3329099	2379863	3496366	2686458	2610241	3924144	3985169	3900833
3482977	2841964	3590388	2650199	3665029	3726992	3082181	3852381
3315675	3859761	2758043	3923218	3080283	3851651	2449559	3891447
3565571	2438531	2805786	3557851	3064293	2690956	3950872	3923312
2976041	3015911	2624074	3771800	2516023	3414739	2923868	2446567
2458742	2727226	3653123	3935016	3066436	3457101	3629243	2726542
3725572	3497881	3109687	3458248	3764399	3643966	3173880	3174121
2409004	3959388	3595979	3142967	3571944	4018080	3577443	2965206
2951674	3402571	2328868	2321182	2600218	3300597	3152220	2734047
3038065	3644541	3765580	3674199	3590014	3820414	2717857	3262535
3394315	3489020	3026599	3955185	2342738	3923257	3893287	3173974
3421177	2450345	3838425	3201319	3665501	3721452	3257338	3194635
2881860	3756566	3741875	3434012	2570193	3833992	3643143	2496382
3217194	3571667	2886174	4021341	3839346	3599811	2434716	3544678
3771602	3011492	3862167	2366798	2812359	3887017	3651152	3598758
