# Supplementary material for: Comparison of Two Methods for Detecting Alternative Splice Variants Using GeneChip® Exon Arrays
Source: Int J Biomed Sci. 2011 Sep;7(3):172–80. (PMC3614835)
Supplement: Supplementary file 2 [file IJBS-7-172_SD1.pdf]

Additional file 1. Statistics for alternative splice variants selected by Partek® GS

# Probe Sets (# of Exons in the gene)	Transcript ID	*p*-value (tissue types, i.e. two group gene level analysis)	*p*-value (PatientNo)	*p*-value (Exon ID, TIAS indicator)	*p*-value (tissue types * Exon ID, TDAS indicator)	*p*-value (Sample ID(tissue types *PatientNo))	F (tissue types, i.e. two group gene level analysis)	F (PatientNo)	F (Exon ID, TIAS indicactor)	F (tissue types * Exon ID, TDAS indicator)	F (Sample ID (tissue types *PatientNo))	gene_assignment
46	2425756	0.001055	0.540701	0	0	0	22.4958	0.932342	40.1425	13.9066	66.075	NM_080629 // COL11A1 // collagen, type XI, alpha 1 // 1p21 // 1301 /// NM_080630
75	2949622	0.002372	0.655684	0	0	5.08E-25	17.4812	0.759519	87.2859	8.83846	15.9453	NM_032470 // TNXB // tenascin XB // 6p21.3 // 7148 /// NR_001284 // TNXA // tena
99	4004044	0.005123	0.178916	0	3.75E-36	0	13.4984	1.8876	107.127	4.30208	78.8596	NM_004006 // DMD // dystrophin (muscular dystrophy, Duchenne and Becker types) /
70	2961177	0.000781	0.243855	0	3.57E-35	0	24.5946	1.61255	152.942	5.26306	132.831	NM_080645 // COL12A1 // collagen, type XII, alpha 1 // 6q12-q13 // 1303 /// NM_0
40	3630736	0.000818	0.351382	0	1.26E-30	1.50E-33	24.2658	1.29941	79.3582	7.09896	22.3501	NM_001004439 // ITGA11 // integrin, alpha 11 // 15q23 // 22801 /// NM_012211 //
64	3581637	0.178842	0.003901	0	9.33E-30	3.88E-11	2.12568	7.01273	229.434	4.96838	7.75307	XM_496145 // LOC440361 // similar to immunoglobulin M chain // 16p11.2 // 440361
31	2730746	0.000579	0.262781	0	4.77E-28	0	26.8322	1.54769	108.961	8.08872	45.3484	NM_003759 // SLC4A4 // solute carrier family 4, sodium bicarbonate cotransporter
64	2740067	0.005865	0.281337	0	2.12E-27	0	12.8675	1.48889	52.2159	4.69571	29.8112	NM_001148 // ANK2 // ankyrin 2, neuronal // 4q25-q27 // 287 /// NM_020977 // ANK
32	3604147	9.93E-05	0.081243	0	4.03E-24	0	43.557	2.65161	40.0037	6.93928	48.1191	NM_018689 // KIAA1199 // KIAA1199 // 15q24 // 57214
27	2652675	0.000455	0.506058	0	7.14E-23	2.07E-35	28.7461	0.989646	114.83	7.59166	25.2617	NM_018098 // ECT2 // epithelial cell transforming sequence 2 oncogene // 3q26.1-
11	3653677	2.80E-05	0.129805	0	1.30E-22	7.61E-14	60.3394	2.18589	130.538	18.09	11.2191	NM_001169 // AQP8 // aquaporin 8 // 16p12 // 343 /// BC040630 // AQP8 // aquapor
24	2584134	0.000572	0.217723	0	1.97E-22	5.30E-33	26.9259	1.71197	50.4938	8.22571	23.9798	NM_002054 // GCG // glucagon // 2q36-q37 // 2641 /// NM_004460 // FAP // fibrobl
36	3296046	0.014508	0.656767	0	2.87E-22	0	9.11292	0.757982	137.415	5.9607	100.112	NM_002247 // KCNMA1 // potassium large conductance calcium-activated channel, su
12	2891556	0.000532	0.389801	0	4.83E-22	0.000171	27.4968	1.2116	323.822	15.8633	3.83718	NM_033260 // FOXQ1 // forkhead box Q1 // 6p25 // 94234
21	3666366	3.07E-05	0.007745	0	6.54E-22	1.54E-12	58.9394	5.76922	67.4115	9.03181	9.17526	NM_001793 // CDH3 // cadherin 3, type 1, P-cadherin (placental) // 16q22.1 // 10
31	3102372	0.0012	0.538171	0	1.56E-21	0	21.6314	0.936425	173.293	6.48002	73.9985	NM_015170 // SULF1 // sulfatase 1 // 8q13.2-q13.3 // 23213 /// BC068565 // SULF1
26	2924514	0.009067	0.068139	0	5.60E-21	6.03E-17	10.9638	2.837	113.577	7.26625	11.9418	NM_181782 // NCOA7 // nuclear receptor coactivator 7 // 6q22.32 // 135112 /// BC
33	3020343	9.80E-05	0.609592	0	6.93E-21	0	43.7027	0.82625	110.58	6.03875	36.1559	NM_000245 // MET // met proto-oncogene (hepatocyte growth factor receptor) // 7q
48	3023149	0.00135	0.521045	0	2.29E-20	0	20.872	0.964472	78.6857	4.60107	132.761	NM_001458 // FLNC // filamin C, gamma (actin binding protein 280) // 7q32-q35 //
26	3358201	0.001483	0.672506	0	3.45E-20	9.69E-28	20.2758	0.735761	181.766	7.03372	19.5763	NM_031264 // MUCDHL // mucin and cadherin-like // 11p15.5 // 53841 /// NM_021924
31	2371139	0.001746	0.28371	0	4.13E-20	0	19.2705	1.48167	89.8667	6.13509	50.5723	NM_018891 // LAMC2 // laminin, gamma 2 // 1q25-q31 // 3918 /// NM_005562 // LAMC
33	3406329	0.027973	0.543516	0	1.11E-19	5.54E-39	6.84587	0.927818	26.4716	5.76597	26.9639	NM_002848 // PTPRO // protein tyrosine phosphatase, receptor type, O // 12p13.3-
42	3301263	0.018855	0.762086	0	1.24E-19	0	8.16573	0.611967	140.213	4.88424	69.6916	NM_006434 // SORBS1 // sorbin and SH3 domain containing 1 // 10q23.3-q24.1 // 10
15	3939470	0.00049	0.815574	0	2.53E-19	1.40E-45	28.1511	0.53759	42.9593	10.899	42.3121	NM_005940 // MMP11 // matrix metallopeptidase 11 (stromelysin 3) // 22q11.2|22q1
17	3422144	0.000515	0.461095	0	1.63E-18	0	27.7618	1.06925	44.362	9.29852	102.447	NM_003667 // LGR5 // leucine-rich repeat-containing G protein-coupled receptor 5
21	3694657	0.00079	0.178805	0	4.72E-18	4.59E-28	24.5177	1.88816	58.8409	7.57898	20.6702	XM_498645 // LOC440380 // LOC440380 // 16q21 // 440380 /// NM_001797 // CDH11 //
59	3193482	0.025996	0.248506	0	7.44E-18	0	7.0818	1.59611	119.564	3.73974	30.0256	NM_000093 // COL5A1 // collagen, type V, alpha 1 // 9q34.2-q34.3 // 1289 /// AK0
8	2970942	0.000202	0.126	0	5.73E-17	1.81E-06	36.0045	2.21434	220.734	18.7574	5.58287	NM_000493 // COL10A1 // collagen, type X, alpha 1(Schmid metaphyseal chondrodysp
8	3047581	0.000187	0.786518	1.68E-35	1.26E-16	5.50E-18	36.7754	0.578247	55.4693	18.2843	16.884	NM_002192 // INHBA // inhibin, beta A (activin A, activin AB alpha polypeptide)
22	3881443	0.000707	0.532452	0	1.29E-16	8.41E-45	25.3238	0.945709	115.025	6.79714	35.0658	NM_012112 // TPX2 // TPX2, microtubule-associated, homolog (Xenopus laevis) // 2
20	3610982	0.000505	0.242612	0	2.19E-16	2.57E-31	27.9043	1.61701	125.232	7.24206	23.6349	NM_015286 // DMN // desmuslin // 15q26.3 // 23336 /// NM_145728 // DMN // desmus
47	3013054	0.004452	0.26503	0	2.43E-16	0	14.1742	1.54032	305.047	4.02348	265.924	NM_000089 // COL1A2 // collagen, type I, alpha 2 // 7q22.1 // 1278 /// X02488 //
14	2786322	0.001455	0.325878	0	3.70E-16	9.45E-23	20.394	1.36334	44.8734	9.72478	18.1399	NM_014331 // SLC7A11 // solute carrier family 7, (cationic amino acid transporte
27	2999755	0.00999	0.459694	0	3.94E-16	0	10.5656	1.07184	176.617	5.69686	57.276	NM_001129 // AEBP1 // AE binding protein 1 // 7p13 // 165 /// BC047097 // KIAA15
22	3490655	0.000287	0.315549	0	4.02E-16	1.78E-17	32.7271	1.39075	63.622	6.62579	12.5529	NM_018204 // CKAP2 // cytoskeleton associated protein 2 // 13q14 // 26586 /// AK
27	2570616	0.001925	0.64227	0	4.25E-16	5.70E-19	18.6882	0.77867	61.8283	5.68762	13.2641	XM_496582 // LOC339692 // hypothetical protein LOC339692 // 2q13 // 339692 /// N
38	3768627	0.004141	0.1499	0	9.15E-16	3.03E-23	14.5307	2.05022	80.818	4.46283	15.5944	NM_007168 // ABCA8 // ATP-binding cassette, sub-family A (ABC1), member 8 // 17q
20	2574984	0.004684	0.582786	0	9.51E-16	4.30E-11	13.9269	0.866471	176.745	6.99574	8.22684	NM_017980 // LIMS2 // LIM and senescent cell antigen-like domains 2 // 2q14.3 //
35	3888133	0.001015	0.154263	0	1.18E-15	0	22.7578	2.02355	142.567	4.67645	41.8013	XM_047550 // ZNF492 // zinc finger protein 492 // 19p12 // 57615 /// NM_177436 /
12	2746591	0.002227	0.610684	0	1.24E-15	6.93E-19	17.8408	0.824635	73.3418	10.8931	15.3739	NM_001957 // EDNRA // endothelin receptor type A // 4q31.22-q31.23 // 1909 /// S
11	3907111	0.000602	0.300494	0	1.36E-15	9.64E-09	26.5309	1.43245	126.103	11.8541	7.073	NM_006809 // TOMM34 // translocase of outer mitochondrial membrane 34 // // 109
22	3632806	0.001735	0.208378	0	3.15E-15	0.001057	19.3077	1.75082	53.8302	6.31727	3.16725	NM_022369 // STRA6 // stimulated by retinoic acid gene 6 homolog (mouse) // 15q2
22	3023384	0.009555	0.464414	0	4.11E-15	0	10.7475	1.06314	109.397	6.27727	58.331	NM_015328 // KIAA0828 // KIAA0828 protein // 7q32.3 // 23382
10	2710599	5.95E-06	0.008953	0	5.56E-15	5.44E-08	88.4368	5.52936	69.0054	12.528	6.58689	NM_021101 // CLDN1 // claudin 1 // 3q28-q29 // 9076 /// AF134160 // CLDN1 // cla
16	3662808	0.000339	0.64037	0	5.75E-15	1.06E-23	31.2287	0.781398	74.3362	8.02696	18.3086	NM_005682 // GPR56 // G protein-coupled receptor 56 // 16q12.2-q21 // 9289 /// N
22	3569814	0.664416	0.124183	0	5.90E-15	0	0.201126	2.22827	245.342	6.2231	59.9246	NM_004965 // HMGN1 // high-mobility group nucleosome binding domain 1 // 21q22.3
13	2451593	0.002289	0.188053	1.45E-38	1.03E-14	1.86E-25	17.6829	1.84259	28.9302	9.49131	21.1858	NM_004997 // MYBPH // myosin binding protein H // 1q32.1 // 4608 /// NM_001276 /

22	2907671	0.001011	0.625931	0	1.23E-14	3.21E-24	22.7847	0.802281	44.6531	6.11354	17.4875	NM_152880 // PTK7 // PTK7 protein tyrosine kinase 7 // 6p21.1-p12.2 // 5754 ///
9	3105600	0.000116	0.084896	0	1.49E-14	4.27E-19	41.8443	2.6062	290.526	13.5835	17.2512	NM_000067 // CA2 // carbonic anhydrase II // 8q22 // 760 /// BC011949 // CA2 //
16	2897899	8.91E-05	0.454664	0	1.57E-14	0.009174	44.8174	1.0812	231.109	7.81067	2.50231	NM_003107 // SOX4 // SRY (sex determining region Y)-box 4 // 6p22.3 // 6659 ///
25	3930360	0.000232	0.258087	0	1.80E-14	2.35E-09	34.6892	1.56328	155.505	5.52411	6.93164	NM_001001890 // RUNX1 // runt-related transcription factor 1 (acute myeloid leuk
11	3292946	0.002424	0.581751	0	2.40E-14	4.69E-20	17.3611	0.868048	115.155	10.8489	16.9334	NM_001057 // TACR2 // tachykinin receptor 2 // 10q11-q21 // 6865
30	2411228	6.16E-06	0.017136	0	3.28E-14	0.009886	87.7052	4.54128	45.662	4.81653	2.44509	NM_003035 // SIL // TAL1 (SCL) interrupting locus // 1q32|1p32 // 6491
24	3942681	0.014873	0.380663	0	3.78E-14	2.53E-23	9.02083	1.23167	88.7922	5.58554	16.5675	NM_006932 // SMTN // smoothelin // 22q12.2 // 6525 /// NM_134269 // SMTN // smoo
15	3046444	0.000241	0.094664	0	3.84E-14	7.87E-21	34.3182	2.49533	79.5639	8.04554	16.1411	NM_003014 // SFRP4 // secreted frizzled-related protein 4 // 7p14.1 // 6424 ///
41	3643580	0.006375	0.54916	0	5.69E-14	1.15E-23	12.4883	0.918801	105.263	3.93192	15.756	NM_021098 // CACNA1H // calcium channel, voltage-dependent, alpha 1H subunit //
26	3508330	0.00184	0.367707	0	5.82E-14	0	18.9569	1.26096	143.578	5.22877	43.1628	NM_006644 // HSPH1 // heat shock 105kDa/110kDa protein 1 // 13q12.3 // 10808 ///
40	3049522	0.000845	0.496633	0	7.02E-14	2.07E-35	24.0295	1.0058	101.426	3.97116	23.6262	NM_022748 // TNS3 // tensin 3 // 7p13-p12.3 // 64759 /// BC071791 // TNS3 // ten
30	2931391	0.000818	0.777201	0	7.41E-14	4.40E-14	24.2602	0.591139	98.2485	4.7286	9.90429	XM_370560 // ARL4P // ADP-ribosylation factor-like 4 pseudogene // 10q21.2 // 38
40	2438282	0.003018	0.285758	0	7.94E-14	6.44E-22	16.1607	1.4755	60.8623	3.96113	14.6664	NM_178229 // IQGAP3 // IQ motif containing GTPase activating protein 3 // 1q22 /
8	3110317	8.98E-05	0.128856	2.22E-25	8.80E-14	0.010236	44.7264	2.19289	32.4343	14.5505	2.54277	NM_138455 // CTHRC1 // collagen triple helix repeat containing 1 // 8q22.3 // 11
15	3556990	0.000156	0.331878	0	1.85E-13	0.004565	38.6058	1.34784	133.528	7.68431	2.73858	NM_032876 // JUB // jub, ajuba homolog (Xenopus laevis) // 14q11.2 // 84962 ///
39	2692447	0.009226	0.478924	0	1.91E-13	4.75E-43	10.8918	1.03689	142.96	3.9462	29.1338	NM_053026 // MYLK // myosin, light polypeptide kinase // 3q21 // 4638 /// NM_053
24	3855218	0.003671	0.017774	0	2.30E-13	2.25E-15	15.1375	4.48958	19.1556	5.34103	10.973	NM_000095 // COMP // cartilage oligomeric matrix protein // 19p13.1 // 1311 ///
26	2362892	0.000813	0.154536	0	2.32E-13	3.80E-23	24.3073	2.02192	28.2817	5.05626	16.2416	NM_000702 // ATP1A2 // ATPase, Na+/K+ transporting, alpha 2 (+) polypeptide // 1
27	2985781	0.00045	0.167189	0	2.44E-13	0	28.8513	1.94939	85.7387	4.92485	75.8813	NM_003247 // THBS2 // thrombospondin 2 // 6q27 // 7058
33	3497790	0.00704	0.444136	0	2.66E-13	0	12.0462	1.1011	167.798	4.32614	56.6963	NM_002271 // RANBP5 // RAN binding protein 5 // 13q32.2 // 3843
23	2914777	0.00753	0.463306	0	2.82E-13	4.10E-11	11.7521	1.06517	21.5027	5.47319	8.13991	NM_003318 // TTK // TTK protein kinase // 6q13-q21 // 7272 /// X70500 // TTK //
24	3638607	0.001369	0.077974	0	3.12E-13	0	20.7805	2.69434	81.1959	5.29981	80.6682	NM_001150 // ANPEP // alanyl (membrane) aminopeptidase (aminopeptidase N, aminop
40	2599153	0.004006	0.76828	0	3.41E-13	0	14.6955	0.603442	224.07	3.84177	125.601	NM_022648 // TNS1 // tensin 1 // 2q35-q36 // 7145 /// AF116610 // TNS1 // tensin
27	3311832	0.000256	0.009774	0	3.68E-13	9.36E-18	33.7781	5.38767	34.6905	4.87571	12.4398	NM_003474 // ADAM12 // ADAM metallopeptidase domain 12 (meltrin alpha) // 10q26.
18	3703885	0.001869	0.210188	0	3.98E-13	4.85E-32	18.8628	1.74315	96.4812	6.49202	25.0015	NM_003486 // SLC7A5 // solute carrier family 7 (cationic amino acid transporter,
45	2605321	0.071694	0.588427	0	4.13E-13	0	4.1641	0.857905	262.988	3.58464	112.163	NM_057164 // COL6A3 // collagen, type VI, alpha 3 // 2q37 // 1293 /// NM_057165
10	2735027	0.000534	0.145241	1.96E-44	4.65E-13	9.21E-20	27.4732	2.07971	54.3439	10.7954	17.2311	NM_000582 // SPP1 // secreted phosphoprotein 1 (osteopontin, bone sialoprotein I
34	3784208	0.073804	0.440527	0	4.99E-13	4.79E-30	4.09118	1.10803	70.184	4.18826	20.4018	NM_032980 // DTNA // dystrobrevin, alpha // 18q12 // 1837 /// NM_032978 // DTNA
10	3910785	0.000577	0.588563	0	5.50E-13	3.53E-05	26.8588	0.8577	94.3733	10.7317	4.41504	NM_003600 // STK6 // serine/threonine kinase 6 // 20q13.2-q13.3 // 6790 /// NR_0
20	3664982	0.019532	0.47644	0	5.77E-13	1.67E-32	8.04263	1.04133	186.097	5.9357	24.6607	NM_003869 // CES2 // carboxylesterase 2 (intestine, liver) // 16q22.1 // 8824 //
18	2987632	0.006374	0.611931	0	6.87E-13	7.82E-17	12.4894	0.822794	161.361	6.39139	12.4522	NM_025250 // TTYH3 // tweety homolog 3 (Drosophila) // 7p22 // 80727 /// AK12460
21	3881282	0.002155	0.086799	0	1.02E-12	7.24E-23	18.029	2.58346	251.591	5.63942	16.6049	NM_178581 // HM13 // histocompatibility (minor) 13 // 20q11.21 // 81502 /// NM_0
35	3265565	0.002564	0.211593	0	1.39E-12	1.42E-24	17.0463	1.73724	31.8142	4.02101	16.608	NM_207303 // ATRNL1 // attractin-like 1 // 10q26 // 26033 /// BC029592 // ATRNL1
13	3150844	0.000231	0.423969	0	1.62E-12	5.81E-06	34.7169	1.14051	107.794	8.1092	4.88366	NM_021021 // SNTB1 // syntrophin, beta 1 (dystrophin-associated protein A1, 59kD
44	3682028	0.001159	0.546848	0	1.64E-12	0	21.8637	0.922485	175.759	3.52481	189.022	NM_022844 // MYH11 // myosin, heavy polypeptide 11, smooth muscle // 16p13.13-p1
24	2710474	0.004958	0.614287	0	1.88E-12	1.22E-11	13.6541	0.819323	191.364	5.05685	8.46211	NM_018192 // LEPREL1 // leprecan-like 1 // 3q28 // 55214 /// XM_047554 // LOC148
27	2585476	0.003909	0.05732	0	2.52E-12	3.03E-32	14.8197	3.02543	54.7998	4.64492	22.8161	NM_002976 // SCN7A // sodium channel, voltage-gated, type VII, alpha // 2q21-q23
14	3385951	0.002785	0.341604	0	2.72E-12	2.64E-17	16.5928	1.32333	33.7151	7.48619	13.4375	NM_016931 // NOX4 // NADPH oxidase 4 // 11q14.2-q21 // 50507 /// XM_208058 // LO
50	2591643	0.001471	0.271591	0	3.45E-12	0	20.3269	1.51922	116.373	3.2468	69.5644	NM_000393 // COL5A2 // collagen, type V, alpha 2 // 2q14-q32 // 1290 /// BC08687
22	2413203	0.003199	0.257273	0	3.82E-12	1.79E-19	15.8514	1.56602	133.434	5.26222	13.9786	NM_004631 // LRP8 // low density lipoprotein receptor-related protein 8, apolipo
14	3388807	0.000959	0.030363	0	4.23E-12	1.94E-22	23.1424	3.77626	38.8741	7.3782	17.8575	NM_002421 // MMP1 // matrix metallopeptidase 1 (interstitial collagenase) // 11q
44	3735151	0.001469	0.153505	0	4.37E-12	2.72E-42	20.3344	2.02812	117.721	3.45029	28.0508	NM_000213 // ITGB4 // integrin, beta 4 // 17q25 // 3691 /// NM_001005731 // ITGB
16	2429556	0.014637	0.509036	0	4.54E-12	1.66E-36	9.08016	0.984594	64.1789	6.61251	30.5429	NM_001232 // CASQ2 // calsequestrin 2 (cardiac muscle) // 1p13.3-p11 // 845
24	2709631	0.010289	0.700372	0	4.55E-12	8.33E-11	10.4461	0.696904	49.117	4.9373	7.90832	NM_001031849 // MASP1 // mannan-binding lectin serine peptidase 1 (C4/C2 activat
19	3168508	0.001677	0.622388	0	5.81E-12	9.46E-20	19.5136	0.807446	59.7054	5.76603	14.5207	NM_014791 // MELK // maternal embryonic leucine zipper kinase // 9p13.2 // 9833
23	2882834	0.056243	0.250726	0	8.33E-12	2.41E-14	4.79643	1.58838	132.573	4.99473	10.3191	NM_207443 // FLJ45244 // FLJ45244 protein // 14q32.13 // 400242 /// NM_032385 //
67	3428447	0.00817	0.567304	0	8.75E-12	0	11.402	0.890282	46.4197	2.77454	31.7258	NM_014503 // DRIM // down-regulated in metastasis // 12q23 // 27340 /// AK000333
27	3607537	0.000248	0.426846	0	9.20E-12	3.28E-15	34.0759	1.13478	68.3329	4.48917	10.7356	NM_018193 // FLJ10719 // hypothetical protein FLJ10719 // 15q25-q26 // 55215 ///
27	3442641	0.003478	0.162839	0	9.74E-12	2.82E-27	15.4151	1.97358	102.529	4.48233	19.1127	NM_017520 // HSMPP8 // M-phase phosphoprotein, mpp8 // 13q12.11 // 54737 /// NM_
18	3845909	0.003004	0.068582	0	1.07E-11	5.90E-10	16.1857	2.83006	103.675	5.88757	7.52213	NM_032737 // LMNB2 // lamin B2 // 19p13.3 // 84823
18	2398820	0.008209	0.741311	0	1.11E-11	6.79E-42	11.3817	0.640506	124.098	5.88175	34.7045	NM_007365 // PADI2 // peptidyl arginine deiminase, type II // 1p35.2-p35.1 // 11
26	3510066	0.000133	0.037339	0	1.46E-11	2.09E-29	40.3021	3.52123	167.057	4.53963	20.8202	NM_006475 // POSTN // periostin, osteoblast specific factor // 13q13.3 // 10631

18	3911217	0.006287	0.399065	0	1.84E-11	0	12.5512	1.19174	146.857	5.78917	45.403	NM_020182 // TMEPAI // transmembrane, prostate androgen induced RNA // 20q13.31-
38	3258477	0.009596	0.760511	0	2.05E-11	0	10.7297	0.614132	143.928	3.60113	67.1813	NM_016341 // PLCE1 // phospholipase C, epsilon 1 // 10q23 // 51196 /// AF170071
12	3758510	9.35E-05	0.266752	8.53E-40	2.33E-11	1.22E-20	44.2498	1.53473	33.9403	7.9184	17.0073	NM_001986 // ETV4 // ets variant gene 4 (E1A enhancer binding protein, E1AF) //
15	3773244	2.57E-05	0.042017	0	2.91E-11	0.085175	61.6623	3.38041	45.9442	6.54303	1.71835	NM_019020 // TBC1D16 // TBC1 domain family, member 16 // 17q25.3 // 125058 /// A
28	2997376	0.000694	0.430956	0	3.01E-11	1.35E-28	25.4605	1.12666	28.6879	4.25074	19.96	NM_018685 // ANLN // anillin, actin binding protein (scraps homolog, Drosophila)
17	3157385	0.000181	0.335065	0	3.18E-11	1.11E-06	37.1361	1.33973	59.9613	5.9316	5.28716	NM_052963 // TOP1MT // topoisomerase (DNA) I, mitochondrial // 8q24.3 // 116447
22	2692319	0.001283	0.743503	0	3.42E-11	3.09E-17	21.1972	0.637497	48.9325	4.93825	12.3826	NM_183357 // ADCY5 // adenylate cyclase 5 // 3q13.2-q21 // 111 /// AK124691 // A
9	3733590	0.000206	0.286834	0	4.06E-11	1.31E-06	35.8279	1.47228	81.8944	10.0933	5.59119	NM_000346 // SOX9 // SRY (sex determining region Y)-box 9 (campomelic dysplasia,
19	3933566	0.006914	0.381714	0	4.20E-11	4.03E-17	12.1261	1.22933	51.4868	5.42561	12.5588	NM_032404 // TMPRSS3 // transmembrane protease, serine 3 // 21q22.3 // 64699 ///
30	3913960	0.023118	0.346929	0	4.30E-11	5.64E-08	7.46832	1.31021	85.6947	4.03973	5.97154	NM_033405 // PRIC285 // peroxisomal proliferator-activated receptor A interactin
17	2345061	0.004671	0.244125	0	4.60E-11	0	13.9402	1.61159	90.038	5.8601	48.3041	NM_012128 // CLCA4 // chloride channel, calcium activated, family member 4 // 1p
33	3151534	0.00127	0.413148	0	6.31E-11	4.07E-27	21.262	1.1624	49.8383	3.78493	18.4532	NM_014109 // ATAD2 // ATPase family, AAA domain containing 2 // 8q24.13 // 29028
21	3304301	0.05008	0.825101	0	6.61E-11	0.00047	5.11293	0.524046	129.759	4.9932	3.42082	NM_002779 // PSD // pleckstrin and Sec7 domain containing // 10q24 // 5662
21	3952825	0.007042	0.166613	0	7.76E-11	0.005487	12.0453	1.95255	48.7458	4.96827	2.65221	NM_053004 // GNB1L // guanine nucleotide binding protein (G protein), beta polyp
18	3996667	0.001196	0.421434	0	8.05E-11	1.09E-13	21.6567	1.14559	193.016	5.51988	10.1537	NM_001363 // DKC1 // dyskeratosis congenita 1, dyskerin // Xq28 // 1736
55	3474104	0.000362	0.368891	0	8.56E-11	2.21E-14	30.6756	1.25824	79.0398	2.89461	9.75301	NM_007174 // CIT // citron (rho-interacting, serine/threonine kinase 21) // 12q2
11	2440943	0.001123	0.301686	0	8.65E-11	1.34E-12	22.0709	1.42907	91.6867	8.12557	10.1674	NM_000570 // FCGR3B // Fc fragment of IgG, low affinity IIIb, receptor (CD16b) /
37	3756193	0.005259	0.359432	0	8.96E-11	0	13.3749	1.28022	125.402	3.52197	54.2787	NM_001067 // TOP2A // topoisomerase (DNA) II alpha 170kDa // 17q21-q22 // 7153 /
26	2487082	0.003699	0.339775	0	9.14E-11	0	15.0994	1.32788	253.683	4.3108	35.4207	NM_032208 // ANTXR1 // anthrax toxin receptor 1 // 2p13.1 // 84168 /// NM_053034
32	2375706	0.062277	0.429317	0	1.23E-10	0	4.52615	1.12989	121.416	3.78329	107.147	NM_001684 // ATP2B4 // ATPase, Ca++ transporting, plasma membrane 4 // 1q32.1 //
25	3592755	0.00303	0.290936	0	1.25E-10	2.00E-25	16.1397	1.46011	74.7017	4.37639	17.9926	NM_153619 // SEMA6D // sema domain, transmembrane domain (TM), and cytoplasmic d
12	3933205	7.17E-05	0.010917	2.41E-41	1.28E-10	0.235656	47.4326	5.21284	35.8916	7.42712	1.30607	NM_020639 // RIPK4 // receptor-interacting serine-threonine kinase 4 // 21q22.3
38	3484641	0.005338	0.360284	0	1.37E-10	7.64E-22	13.3043	1.27822	35.4154	3.435	14.6848	NM_000059 // BRCA2 // breast cancer 2, early onset // 13q12.3 // 675
14	2409104	0.000376	0.040947	0	1.41E-10	2.37E-28	30.3496	3.41089	271.699	6.53069	23.4929	NM_006516 // SLC2A1 // solute carrier family 2 (facilitated glucose transporter)
27	3326183	0.000518	0.075446	0	1.53E-10	5.74E-23	27.7097	2.72888	218.786	4.15037	16.0294	NM_005898 // M11S1 // membrane component, chromosome 11, surface marker 1 // 11p
21	2533019	0.031958	0.224381	0	1.70E-10	1.54E-24	6.42755	1.68542	75.0491	4.84689	17.8853	NM_019075 // UGT1A10 // UDP glucuronosyltransferase 1 family, polypeptide A10 //
16	3125571	0.005603	0.352538	0	1.74E-10	1.69E-19	13.0781	1.29662	64.5019	5.85633	14.8122	NM_138715 // MSR1 // macrophage scavenger receptor 1 // 8p22 // 4481 /// NM_1387
15	3388830	0.000324	0.108611	0	1.82E-10	7.39E-13	31.6468	2.35852	36.5994	6.13529	9.8035	NM_002422 // MMP3 // matrix metallopeptidase 3 (stromelysin 1, progelatinase) //
16	3499132	0.001308	0.417572	0	1.94E-10	2.77E-11	21.0736	1.15338	37.8627	5.83377	8.56568	NM_004791 // ITGBL1 // integrin, beta-like 1 (with EGF-like repeat domains) // 1
16	3573870	0.003619	0.522347	0	1.95E-10	0.010257	15.2105	0.962314	63.526	5.8322	2.46502	NM_001007023 // DIO2 // deiodinase, iodothyronine, type II // 14q24.2-q24.3 // 1
23	2889916	0.012652	0.710806	0	1.95E-10	2.09E-23	9.63151	0.682477	97.0372	4.54935	16.7451	NM_021599 // ADAMTS2 // ADAM metallopeptidase with thrombospondin type 1 motif,
14	3710108	0.002379	0.112094	0	2.05E-10	0.000135	17.4664	2.32755	40.9949	6.44072	3.87322	NM_004246 // GLP2R // glucagon-like peptide 2 receptor // 17p13.3 // 9340 /// BC
39	3399545	0.000771	0.096578	0	2.29E-10	1.32E-21	24.6937	2.4752	80.3575	3.34407	14.4986	NM_015261 // hCAP-D3 // KIAA0056 protein // 11q25 // 23310 /// AK025667 // hCAP-
19	3891278	0.003714	0.379377	0	3.00E-10	1.17E-21	15.078	1.23453	62.2373	5.08776	15.9833	NM_198976 // TH1L // TH1-like (Drosophila) // 20q13 // 51497 /// NM_016397 // TH
22	3895118	0.0129	0.034981	0	3.21E-10	8.92E-07	9.5569	3.60051	49.8189	4.60741	5.26862	NM_019609 // CPXM // carboxypeptidase X (M14 family) // 20p13-p12.3 // 56265
17	2662020	0.001813	0.096883	0	3.58E-10	1.27E-08	19.0445	2.47204	115.294	5.46362	6.63295	NM_020165 // RAD18 // RAD18 homolog (S. cerevisiae) // 3p25-p24 // 56852 /// AB0
47	3853108	0.010472	0.197824	0	3.59E-10	0	10.375	1.79713	100.466	3.01406	36.1768	NM_000435 // NOTCH3 // Notch homolog 3 (Drosophila) // 19p13.2-p13.1 // 4854 ///
20	3409127	0.000351	0.142111	0	3.86E-10	0.000718	30.9458	2.10014	149.996	4.87385	3.29597	NM_020183 // ARNTL2 // aryl hydrocarbon receptor nuclear translocator-like 2 //
34	3893520	0.07734	0.467392	0	4.21E-10	5.56E-07	3.97464	1.05769	100.307	3.53742	5.30427	NM_003823 // TNFRSF6B // tumor necrosis factor receptor superfamily, member 6b,
31	2852591	0.000535	0.703329	0	4.35E-10	1.14E-27	27.4489	0.69281	34.8371	3.71763	18.9958	NM_030955 // ADAMTS12 // ADAM metallopeptidase with thrombospondin type 1 motif,
13	2714465	0.002529	0.12804	0	4.37E-10	0.007516	17.1237	2.19896	66.4581	6.63253	2.58698	NM_001004356 // FGFRL1 // fibroblast growth factor receptor-like 1 // 4p16 // 53
16	3881786	0.001247	0.676948	0	4.96E-10	2.43E-18	21.3809	0.729529	235.427	5.64035	13.8876	NM_015352 // POFUT1 // protein O-fucosyltransferase 1 // 20q11 // 23509 /// NM_1
17	3148796	0.00083	0.200846	0	5.39E-10	8.05E-12	24.1572	1.78359	117.913	5.38472	8.88567	NM_032869 // NUDCD1 // NudC domain containing 1 // 8q23 // 84955 /// BC031258 //
13	3265224	0.00998	0.693499	0	6.80E-10	7.92E-15	10.5694	0.70644	86.4412	6.5179	11.6121	NM_198496 // AMACO // A-domain containing protein similar to matrilin and collag
14	3252036	0.000103	0.262043	0	7.86E-10	1.44E-17	43.1267	1.55011	82.6019	6.12039	13.6544	NM_002658 // PLAU // plasminogen activator, urokinase // 10q24 // 5328
21	2345023	0.043591	0.489369	0	9.18E-10	0	5.50363	1.01844	137.781	4.58577	79.8779	NM_001285 // CLCA1 // chloride channel, calcium activated, family member 1 // 1p
32	3338293	0.014139	0.213909	0	9.63E-10	3.64E-39	9.20925	1.7276	57.3895	3.57054	27.2586	NM_018043 // TMEM16A // transmembrane protein 16A // 11q13.3 // 55107 /// AK0976
40	3964049	0.005706	0.616658	0	9.78E-10	1.33E-20	12.9939	0.815838	76.0571	3.17789	13.825	NM_014246 // CELSR1 // cadherin, EGF LAG seven-pass G-type receptor 1 (flamingo
34	2461473	0.005317	0.262905	0	1.04E-09	2.44E-37	13.3229	1.54728	70.7943	3.449	25.6073	NM_005646 // TARBP1 // TAR (HIV) RNA binding protein 1 // 1q42.3 // 6894 /// BC0
18	3332626	5.56E-06	0.029094	0	1.07E-09	0.222169	89.9043	3.83027	115.27	5.05038	1.32658	NM_017870 // HSPA5BP1 // heat shock 70kDa protein 5 (glucose-regulated protein,
12	2443120	0.003071	0.526839	0	1.19E-09	8.96E-35	16.0683	0.9549	107.11	6.79117	32.7891	NM_001937 // DPT // dermatopontin // 1q12-q23 // 1805 /// Z22865 // DPT // derma
13	2531589	0.0032	0.533211	0	1.37E-09	4.34E-41	15.8493	0.944473	731.791	6.33647	39.6315	NM_030926 // ITM2C // integral membrane protein 2C // 2q37 // 81618 /// NM_00101

20	3597338	0.026792	0.172066	0	1.44E-09	4.37E-32	6.98416	1.92311	163.761	4.65916	24.2977	NM_001018005 // TPM1 // tropomyosin 1 (alpha) // 15q22.1 // 7168 /// NM_00101800
13	3454892	0.00024	0.021082	0	1.46E-09	1.17E-06	34.376	4.25352	44.1845	6.32119	5.38705	NM_001971 // ELA1 // elastase 1, pancreatic // 12q13 // 1990 /// NM_007210 // GA
24	3472225	2.09E-05	1.11E-05	0	1.65E-09	0.944747	64.9373	30.3817	85.6247	4.13922	0.379567	NM_024072 // DDX54 // DEAD (Asp-Glu-Ala-Asp) box polypeptide 54 // 12q24.13 // 7
18	3658980	0.001025	0.193513	0	1.86E-09	1.62E-11	22.6894	1.81687	57.2862	4.94979	8.61198	NM_133443 // GPT2 // glutamic pyruvate transaminase (alanine aminotransferase) 2
37	2712236	0.011137	0.663777	0	1.92E-09	0	10.1293	0.748055	43.5019	3.24396	92.0207	NM_018406 // MUC4 // mucin 4, tracheobronchial // 3q29 // 4585 /// XM_210543 //
18	3394660	0.005286	0.017611	0	2.23E-09	2.88E-12	13.3501	4.50259	27.4832	4.91745	9.14134	NM_012101 // TRIM29 // tripartite motif-containing 29 // 11q22-q23 // 23650 ///
18	3776504	0.003092	0.238201	0	2.23E-09	9.39E-05	16.0321	1.63302	70.976	4.91678	3.9333	NM_173211 // TGIF // TGFB-induced factor (TALE family homeobox) // 18p11.3 // 70
48	2461037	0.00985	0.186896	0	2.31E-09	1.04E-21	10.6229	1.84815	62.6928	2.84862	14.3298	XM_371511 // LOC388972 // similar to anaphase promoting complex subunit 1; anaph
23	3783398	0.005142	0.005063	0	2.62E-09	0.981818	13.4811	6.51789	117.011	4.18175	0.272301	NM_001942 // DSG1 // desmoglein 1 // 18q12.1 // 1828
35	3605395	0.01542	0.472781	0	2.66E-09	2.19E-38	8.88737	1.04791	71.8783	3.30629	26.2448	NM_207517 // ADAMTSL3 // ADAMTS-like 3 // 15q25.2 // 57188
15	2528476	0.002372	0.548765	0	3.07E-09	0	17.4813	0.919429	77.8013	5.51255	48.7633	NM_005876 // APEG1 // aortic preferentially expressed gene 1 // 2q35 // 10290 //
35	3494137	0.004466	0.320637	0	4.57E-09	5.82E-37	14.1584	1.37713	190.63	3.25396	25.209	NM_005358 // LMO7 // LIM domain 7 // 13q22.2 // 4008 /// AB020665 // LMO7 // LIM
38	2954678	0.004666	0.759905	0	4.58E-09	0	13.9453	0.614966	179.512	3.12284	31.4038	NM_020750 // XPO5 // exportin 5 // 6p21.1 // 57510
7	3388673	0.000526	0.124185	2.07E-27	4.84E-09	4.23E-08	27.5911	2.22826	45.5653	10.3524	7.16965	NM_002423 // MMP7 // matrix metallopeptidase 7 (matrilysin, uterine) // 11q21-q2
8	2398706	0.002077	0.193661	0	5.54E-09	0.000911	18.2411	1.81618	265.759	9.01117	3.39761	NM_002403 // MFAP2 // microfibrillar-associated protein 2 // 1p36.1-p35 // 4237
13	2946106	0.799218	0.70652	0	5.83E-09	0.475194	0.068645	0.688397	52.269	5.96463	0.958852	NM_006632 // SLC17A3 // solute carrier family 17 (sodium phosphate), member 3 //
11	3830216	0.004833	0.26211	0	5.89E-09	8.10E-06	13.7764	1.54989	202.979	6.79424	4.85287	NM_144779 // FXYD5 // FXYD domain containing ion transport regulator 5 // 19q12-
10	3985717	0.000518	0.086747	0	6.20E-09	6.41E-08	27.7087	2.58406	76.8357	7.33573	6.53053	NM_000533 // PLP1 // proteolipid protein 1 (Pelizaeus-Merzbacher disease, spasti
9	3887049	0.002291	0.468448	0	6.57E-09	0.000472	17.6779	1.05577	112.593	8.01693	3.58612	NM_181803 // UBE2C // ubiquitin-conjugating enzyme E2C // 20q13.12 // 11065 ///
16	3652902	0.001836	0.192453	0	6.92E-09	8.72E-16	18.9681	1.8218	70.1393	5.09985	11.9021	NM_000336 // SCNN1B // sodium channel, nonvoltage-gated 1, beta (Liddle syndrome
99	3134034	0.001899	0.275049	0	7.20E-09	0	18.7686	1.50832	192.607	2.09128	123.089	NM_006904 // PRKDC // protein kinase, DNA-activated, catalytic polypeptide // 8q
48	2474341	0.003236	0.104782	0	7.45E-09	1.01E-38	15.7918	2.39392	89.3953	2.7622	25.3008	NM_004341 // CAD // carbamoyl-phosphate synthetase 2, aspartate transcarbamylase
41	2376168	0.011446	0.625354	0	7.54E-09	1.01E-23	10.0211	0.803121	78.8101	2.96785	15.7944	XM_496431 // LOC440711 // similar to mKIAA0756 protein // 1q32.1 // 440711 /// N
17	2633390	0.002331	0.298855	0	8.30E-09	6.98E-06	17.5816	1.43712	67.0074	4.85914	4.73329	NM_001850 // COL8A1 // collagen, type VIII, alpha 1 // 3q12.3 // 1295 /// NM_020
4	2779199	0.002669	0.15855	3.80E-39	8.49E-09	5.87E-06	16.8253	1.99819	490.921	19.8379	6.19931	NM_000667 // ADH1A // alcohol dehydrogenase 1A (class I), alpha polypeptide // 4
28	3504434	0.00554	0.49718	0	8.53E-09	6.96E-27	13.1314	1.00486	80.9893	3.58924	18.7205	NM_022459 // XPO4 // exportin 4 // 13q11 // 64328 /// AK129848 // XPO4 // export
12	3750662	0.202055	0.361256	0	8.54E-09	0.707503	1.8937	1.27594	78.0928	6.23548	0.70106	NM_000638 // VTN // vitronectin (serum spreading factor, somatomedin B, compleme
10	3742285	0.000144	0.016809	0	9.59E-09	9.42E-05	39.4462	4.56869	107.253	7.18434	4.08987	NM_022059 // CXCL16 // chemokine (C-X-C motif) ligand 16 // 17p13 // 58191
25	2625793	0.653173	0.097967	0	9.61E-09	4.61E-17	0.215961	2.46087	82.9317	3.80841	12.0724	XM_498933 // LOC440958 // LOC440958 // 3p14.3 // 440958 /// NM_007159 // SLMAP /
7	3903361	0.005804	0.104174	3.44E-42	9.69E-09	1.19E-07	12.9157	2.39968	102.109	9.96619	6.76409	NM_000687 // AHCY // S-adenosylhomocysteine hydrolase // 20cen-q13.1 // 191 ///
12	3129149	0.001071	0.151264	0	1.17E-08	1.87E-06	22.3921	2.04179	45.1651	6.14849	5.28133	NM_018492 // PBK // PDZ binding kinase // 8p21.2 // 55872
17	3677752	0.001524	0.259611	0	1.19E-08	3.21E-25	20.1029	1.55818	123.852	4.79039	19.3185	NM_016292 // TRAP1 // TNF receptor-associated protein 1 // 16p13.3 // 10131 ///
18	3871192	0.030415	0.815259	0	1.21E-08	3.18E-30	6.58146	0.538036	210.392	4.61093	23.3589	NM_003180 // SYT5 // synaptotagmin V // 19q|11p // 6861 /// NM_014931 // SAPS1 /
125	2958325	0.061622	0.035618	0	1.22E-08	0	4.55384	3.57847	93.4938	1.92363	112.315	NM_001723 // DST // dystonin // 6p12-p11 // 667 /// NM_183380 // DST // dystonin
10	2609347	0.008159	0.137156	0	1.33E-08	6.77E-05	11.4077	2.13359	131.213	7.07119	4.19894	NM_014583 // LMCD1 // LIM and cysteine-rich domains 1 // 3p26-p24 // 29995
35	3092808	0.010759	0.088114	0	1.42E-08	0.010391	10.2666	2.56808	87.5458	3.14425	2.42396	NM_004495 // NRG1 // neuregulin 1 // 8p21-p12 // 3084 /// NM_013964 // NRG1 // n
37	3252071	0.359496	0.341981	0	1.42E-08	0	0.932286	1.32239	182.559	3.05953	48.0159	NM_003373 // VCL // vinculin // 10q22.1-q23 // 7414 /// NM_014000 // VCL // vinc
25	3144973	0.001062	0.181152	0	1.45E-08	2.28E-09	22.4504	1.87635	47.8854	3.75423	6.93956	NM_006550 // FSBP // fibrinogen silencer binding protein // 8q22.1 // 10646 ///
8	2816459	0.000734	0.721741	0	1.48E-08	3.20E-08	25.0509	0.667405	170.379	8.55795	7.0556	NM_001992 // F2R // coagulation factor II (thrombin) receptor // 5q13 // 2149 //
15	3106243	4.31E-05	0.015379	0	1.60E-08	0.004442	54.0888	4.69659	71.8231	5.15147	2.74753	NM_003821 // RIPK2 // receptor-interacting serine-threonine kinase 2 // 8q21 //
12	2611848	0.000107	0.401234	0	1.60E-08	9.46E-28	42.7101	1.18715	80.596	6.05989	24.3206	NM_003043 // SLC6A6 // solute carrier family 6 (neurotransmitter transporter, ta
11	2409820	5.54E-05	0.007656	1.68E-24	1.63E-08	7.80E-06	50.7133	5.78878	19.9548	6.47949	4.86533	NM_153274 // VMD2L2 // vitelliform macular dystrophy 2-like 2 // 1p33-p32.3 // 2
17	3960478	0.001757	0.120736	0	1.67E-08	0.004595	19.2312	2.25539	114.109	4.7249	2.72508	XM_495953 // TPTEps1 // TPTE pseudogene 1 // 13q14.3 // 440140 /// NM_152221 //
12	3821263	0.004792	0.745346	0	1.68E-08	6.45E-44	13.8165	0.634966	55.198	6.04622	46.0545	NM_001299 // CNN1 // calponin 1, basic, smooth muscle // 19p13.2-p13.1 // 1264
9	3726934	0.001286	0.235391	0	1.99E-08	3.78E-07	21.1819	1.6434	156.611	7.5796	6.02243	NM_001018136 // NME2 // non-metastatic cells 2, protein (NM23B) expressed in //
48	3210013	0.036128	0.516459	0	2.01E-08	0	6.05415	0.97211	46.0899	2.68818	48.7817	NM_017662 // TRPM6 // transient receptor potential cation channel, subfamily M,
15	2788926	0.011815	0.427583	0	2.07E-08	6.64E-22	9.89677	1.13332	166.868	5.09511	17.0585	NM_000901 // NR3C2 // nuclear receptor subfamily 3, group C, member 2 // 4q31.1
12	3069366	0.002828	0.69177	2.17E-42	2.18E-08	5.88E-05	16.5097	0.708843	37.2491	5.97336	4.1792	NM_003391 // WNT2 // wingless-type MMTV integration site family member 2 // 7q31
19	2560076	0.000399	0.567135	0	2.64E-08	9.78E-07	29.8412	0.890544	77.7465	4.32005	5.28601	NM_033046 // RTKN // rhotekin // 2p13.1 // 6242 /// NM_001015056 // RTKN // rhot
6	3751859	0.000295	0.177375	6.25E-26	2.66E-08	9.03E-11	32.4716	1.89545	55.1362	11.0313	10.3029	NM_206832 // UNQ9372 // AWKS9372 // 17q11.2 // 388364
8	3581485	0.000806	0.000555	0	2.71E-08	0.932094	24.3707	11.7592	207.773	8.28286	0.401889	NM_177533 // NUDT14 // nudix (nucleoside diphosphate linked moiety X)-type motif
14	2909263	0.005121	0.128294	0	2.77E-08	1.19E-26	13.5001	2.19706	178.751	5.27856	21.8062	NM_005588 // MEP1A // meprin A, alpha (PABA peptide hydrolase) // 6p12-p11 // 42

8	3332424	0.001296	0.10233	1.82E-44	2.80E-08	1.48E-13	21.1327	2.41741	84.3197	8.26811	12.0321	NM_017716 // MS4A12 // membrane-spanning 4-domains, subfamily A, member 12 // 11
9	3748798	0.007604	0.750374	0	3.13E-08	7.19E-20	11.7099	0.628062	405.254	7.40299	18.1216	NM_002404 // MFAP4 // microfibrillar-associated protein 4 // 17p11.2 // 4239 ///
12	3791782	0.00786	0.414822	1.50E-34	3.31E-08	2.32E-13	11.5672	1.15897	27.825	5.85778	10.6024	NM_002639 // SERPINB5 // serpin peptidase inhibitor, clade B (ovalbumin), member
10	3226883	0.045345	0.113571	0	3.38E-08	0.234174	5.3912	2.31477	441.064	6.75022	1.31228	NM_203434 // IER5L // immediate early response 5-like // 9q34.11 // 389792 /// B
8	3868828	0.001664	0.266589	1.50E-11	3.46E-08	2.18E-11	19.5638	1.53526	11.8645	8.17109	9.90683	NM_002776 // KLK10 // kallikrein 10 // 19q13.3-q13.4 // 5655 /// NM_145888 // KL
36	3238962	0.005579	0.304738	0	3.50E-08	1.22E-41	13.0982	1.42047	96.8289	3.01478	28.5013	NM_019590 // KIAA1217 // KIAA1217 // 10p12.31 // 56243 /// BC018764 // KIAA1217
28	3741800	0.001567	0.201167	0	3.57E-08	3.25E-30	19.9305	1.78217	187.221	3.419	21.1467	NM_174955 // ATP2A3 // ATPase, Ca++ transporting, ubiquitous // 17p13.3 // 489 /
5	3815399	0.000674	0.00536	9.20E-42	3.59E-08	0.101989	25.6835	6.41361	256.112	13.3146	1.71146	NM_004368 // CNN2 // calponin 2 // 21q11.1 // 1265 /// NM_201277 // CNN2 // calp
14	3998766	0.000894	0.283202	0	3.68E-08	8.38E-06	23.6334	1.48321	45.9536	5.21185	4.74038	NM_000216 // KAL1 // Kallmann syndrome 1 sequence // Xp22.32 // 3730 /// NM_0529
35	2695941	0.004003	0.021374	0	3.69E-08	1.54E-20	14.6995	4.23488	77.7165	3.05061	13.945	NM_007027 // TOPBP1 // topoisomerase (DNA) II binding protein 1 // 3q22.1 // 110
36	2796995	0.068445	0.25711	0	3.76E-08	4.20E-25	4.28189	1.56657	73.9086	3.00772	16.9077	NM_003603 // SORBS2 // sorbin and SH3 domain containing 2 // 4q35.1 // 8470 ///
19	3568616	0.023304	0.216925	0	4.17E-08	1.10E-09	7.44145	1.71522	52.8114	4.24134	7.29803	NM_198686 // RAB15 // RAB15, member RAS onocogene family // 14q23.3 // 376267 //
14	3699634	0.192604	0.006975	0	4.26E-08	0.051103	1.98363	5.94724	40.6873	5.17719	1.91203	XM_370992 // LOC388298 // hypothetical LOC388298 // 16q23.1 // 388298 /// NM_024
30	2728938	0.000708	0.053132	0	4.34E-08	1.71E-06	25.3179	3.11014	47.6659	3.27625	5.01125	NM_015236 // LPHN3 // latrophilin 3 // 4q13.1 // 23284
26	2818517	0.000453	0.34564	0	4.88E-08	0	28.7807	1.31337	119.632	3.5178	70.1559	NM_004385 // CSPG2 // chondroitin sulfate proteoglycan 2 (versican) // 5q14.3 //
17	3685329	0.001511	0.091114	0	5.28E-08	1.30E-07	20.1593	2.53399	77.5553	4.5039	5.93332	NM_024675 // FLJ21816 // hypothetical protein FLJ21816 // 16p12.1 // 79728
22	2358320	0.006862	0.079402	0	5.53E-08	0.009695	12.1598	2.67542	63.6354	3.84351	2.46461	NM_025150 // TARSL1 // threonyl-tRNA synthetase-like 1 // 1q21.2 // 80222 /// XM
25	3175274	0.009219	0.462824	0	5.54E-08	0	10.8948	1.06606	128.19	3.57699	36.2468	NM_006200 // PCSK5 // proprotein convertase subtilisin/kexin type 5 // 9q21.3 //
29	3025545	0.524914	0.237062	0	5.89E-08	1.06E-41	0.437473	1.63721	104.377	3.29857	29.8275	NM_004342 // CALD1 // caldesmon 1 // 7q33 // 800 /// NM_033139 // CALD1 // calde
12	3728776	0.06346	0.075628	8.41E-44	5.94E-08	0.02504	4.47697	2.72635	39.1356	5.69507	2.17806	NM_058216 // RAD51C // RAD51 homolog C (S. cerevisiae) // 17q22-q23 // 5889 ///
24	3317352	0.033281	0.092229	0	6.33E-08	1.55E-26	6.30283	2.52166	112.159	3.64003	18.9488	NM_181797 // KCNQ1 // potassium voltage-gated channel, KQT-like subfamily, membe
12	3728964	0.001157	0.663066	0	6.76E-08	1.70E-05	21.8748	0.749061	109.888	5.6592	4.57603	NM_174930 // PMS2L5 // postmeiotic segregation increased 2-like 5 // 7q11-q22 //
16	3079803	0.785355	0.027035	0	6.78E-08	0.021553	0.078741	3.92424	43.6679	4.63322	2.2126	NM_016203 // PRKAG2 // protein kinase, AMP-activated, gamma 2 non-catalytic subu
8	2727762	0.006878	0.071252	0	7.51E-08	0.058248	12.1493	2.78932	111.507	7.82206	1.89597	NM_024592 // SRD5A2L // steroid 5 alpha-reductase 2-like // 4q12 // 79644
5	3230760	0.009278	0.146018	1.46E-18	7.77E-08	0.775043	10.8684	2.07471	43.6916	12.6255	0.621402	NM_178448 // C9orf140 // chromosome 9 open reading frame 140 // 9q34.3 // 89958
20	3428845	0.007285	0.444254	0	7.80E-08	6.09E-06	11.8963	1.10088	55.287	4.00841	4.73025	NM_017915 // FLJ20641 // hypothetical protein FLJ20641 // 12q23.3 // 55010 /// B
20	3884100	0.004131	0.260931	0	8.15E-08	1.09E-29	14.5429	1.55379	180.012	4.00107	22.2529	NM_002951 // RPN2 // ribophorin II // 20q12-q13.1 // 6185
15	2676009	0.022277	0.009539	0	8.53E-08	0.00718	7.59262	5.42667	115.246	4.78547	2.58903	NM_007284 // PTK9L // PTK9L protein tyrosine kinase 9-like (A6-related protein)
21	3888217	0.000492	0.283997	0	8.60E-08	4.28E-06	28.1207	1.48081	93.772	3.8797	4.82221	NM_017895 // DDX27 // DEAD (Asp-Glu-Ala-Asp) box polypeptide 27 // 20q13.13 // 5
8	2886679	0.005221	0.517913	0	9.02E-08	2.65E-06	13.4083	0.969683	121.822	7.74032	5.44558	NM_004137 // KCNMB1 // potassium large conductance calcium-activated channel, su
42	3850069	0.002128	0.143991	0	9.03E-08	5.55E-12	18.1023	2.08781	90.6503	2.72627	8.39035	NM_001379 // DNMT1 // DNA (cytosine-5-)-methyltransferase 1 // 19p13.2 // 1786
32	2842624	0.057139	0.935599	0	1.00E-07	0	4.75409	0.345089	64.9157	3.08183	76.7057	NM_017675 // PCLKC // protocadherin LKC // 5q35.2 // 54825
12	3778772	0.056515	0.544871	0	1.01E-07	6.89E-08	4.78348	0.925646	221.423	5.54733	6.34397	NM_153000 // APCDD1 // adenomatosis polyposis coli down-regulated 1 // 18p11.22
19	2830638	0.004919	0.607536	0	1.02E-07	1.96E-16	13.6918	0.829293	29.7993	4.08765	12.056	NM_005733 // KIF20A // kinesin family member 20A // 5q31 // 10112
36	3222170	0.063343	0.677012	0	1.12E-07	0	4.48178	0.729439	146.154	2.90302	173.907	NM_002160 // TNC // tenascin C (hexabrachion) // 9q33 // 3371 /// BX641111 // TN
11	3873160	0.004237	0.061981	5.17E-41	1.14E-07	0.01006	14.4172	2.93944	40.6086	5.8824	2.50515	NM_021158 // TRIB3 // tribbles homolog 3 (Drosophila) // 20p13-p12.2 // 57761 //
27	3250237	0.000708	0.010565	0	1.15E-07	4.36E-12	25.3106	5.26421	51.2057	3.34121	8.67822	NM_025130 // HKDC1 // hexokinase domain containing 1 // 10q22.1 // 80201 /// BX5
20	2447877	0.009698	0.360299	0	1.16E-07	1.26E-43	10.6866	1.27818	69.1073	3.94382	35.0765	NM_022083 // C1orf24 // chromosome 1 open reading frame 24 // 1q25 // 116496 ///
11	3664924	0.003374	0.100896	0	1.17E-07	3.79E-07	15.5729	2.43144	74.6268	5.87551	5.85326	NM_152757 // FLJ30313 // hypothetical protein FLJ30313 // 20q13.33 // 253868 ///
22	2654023	0.006673	0.326644	0	1.24E-07	2.34E-13	12.2835	1.36135	397.093	3.72244	9.69159	NM_004301 // ACTL6A // actin-like 6A // 3q26.33 // 86 /// NM_177989 // ACTL6A //
21	2676182	0.00284	0.055922	0	1.29E-07	4.09E-07	16.4882	3.05286	46.8816	3.81576	5.51024	NM_022908 // FLJ12442 // hypothetical protein FLJ12442 // 3p21.1 // 64943 /// AF
11	3219215	0.010858	0.541153	0	1.39E-07	5.46E-26	10.2304	0.931614	184.547	5.82312	23.2493	XM_498339 // LOC442430 // similar to peptidyl-Pro cis trans isomerase // 9q31.2
38	3972093	0.012712	0.229695	0	1.52E-07	9.15E-38	9.61309	1.66486	50.2072	2.80317	25.4536	NM_016937 // POLA // polymerase (DNA directed), alpha // Xp22.1-p21.3 // 5422
15	2604254	0.003254	0.853262	0	1.53E-07	2.68E-09	15.7614	0.483064	142.378	4.65856	7.19771	NM_018410 // DKFZp762E1312 // hypothetical protein DKFZp762E1312 // 2q37.1 // 55
24	3643396	0.014073	0.436969	0	1.57E-07	5.53E-41	9.22693	1.11491	49.7552	3.51419	30.675	NM_013404 // MSLN // mesothelin // 16p13.3 // 10232 /// NM_005823 // MSLN // mes
18	3329099	0.00377	0.458708	0	1.62E-07	2.84E-09	15.0024	1.07366	26.5749	4.13983	7.04969	NM_152312 // GYLTL1B // glycosyltransferase-like 1B // 11p11.2 // 120071 /// AK0
32	2379863	0.000854	0.196841	0	1.72E-07	2.79E-12	23.9611	1.80159	26.8165	3.02376	8.70294	NM_016343 // CENPF // centromere protein F, 350/400ka (mitosin) // 1q32-q41 // 1
18	3496366	0.003526	0.309874	6.49E-38	1.73E-07	0.003627	15.3437	1.40621	18.7012	4.12797	2.79669	AF339828 // C13orf25 // chromosome 13 open reading frame 25 // 13q31.3 // 407975
35	2686458	0.001644	0.093105	0	1.75E-07	3.77E-24	19.6361	2.5121	53.0657	2.89691	16.3247	NM_015429 // ABI3BP // ABI gene family, member 3 (NESH) binding protein // 3q12
38	2610241	0.00052	0.096417	0	1.84E-07	1.58E-06	27.6787	2.47687	74.6315	2.78532	4.99552	NM_033084 // FANCD2 // Fanconi anemia, complementation group D2 // 3p26 // 2177
38	3924144	0.107212	0.398633	0	1.86E-07	8.61E-25	3.20113	1.19266	102.131	2.78421	16.6062	NM_030582 // COL18A1 // collagen, type XVIII, alpha 1 // 21q22.3 // 80781 /// NM
23	3985169	0.846176	0.104365	0	1.94E-07	0.464366	0.039869	2.39787	66.3144	3.56687	0.969983	NR_002216 // NXF4 // nuclear RNA export factor 4 pseudogene // Xq22 // 55999 ///

8	3900833	0.059062	0.376813	1.38E-35	1.94E-07	0.002318	4.66583	1.24026	55.6971	7.39865	3.07067	NM_153675 // FOXA2 // forkhead box A2 // 20p11 // 3170 /// NM_021784 // FOXA2 //
15	3482977	0.003519	0.863504	0	1.98E-07	3.96E-12	15.355	0.467694	127.018	4.60176	9.26021	NM_015972 // POLR1D // polymerase (RNA) I polypeptide D, 16kDa // 13q12.2 // 510
6	2841964	0.002703	0.219546	0	1.99E-07	0.089847	16.7567	1.70461	407.588	9.69112	1.74678	NM_002449 // MSX2 // msh homeo box homolog 2 (Drosophila) // 5q34-q35 // 4488 //
16	3590388	0.000678	0.343173	0	2.04E-07	0.000212	25.6368	1.31944	65.1343	4.40849	3.70455	NM_018454 // NUSAP1 // nucleolar and spindle associated protein 1 // 15q15.1 //
35	2650199	0.001553	0.034585	0	2.19E-07	1.16E-13	19.9893	3.61444	94.2118	2.87433	9.53154	NM_005496 // SMC4L1 // SMC4 structural maintenance of chromosomes 4-like 1 (yeas
15	3665029	0.022467	0.924063	0	2.26E-07	0	7.56391	0.367368	117.576	4.57245	54.0381	NM_024922 // FLJ21736 // esterase 31 // 16q22.1 // 79984 /// AK097538 // FLJ2173
12	3726992	0.00419	0.263238	0	2.28E-07	0.006531	14.4718	1.54618	79.7439	5.32249	2.64302	NM_016001 // WDR50 // WD repeat domain 50 // 17q21.33 // 51096
33	3082181	0.003333	0.421905	0	2.35E-07	1.93E-24	15.6356	1.14464	70.7943	2.94719	16.6286	BC028618 // LUZP5 // leucine zipper protein 5 // 7q36.3 // 54892
8	3852381	0.002804	0.093337	1.40E-45	2.37E-07	0.00644	16.5572	2.50958	88.0876	7.31084	2.70894	NM_024825 // FLJ23447 // hypothetical protein FLJ23447 // 19p13.12 // 79883 ///
7	3315675	0.000762	0.120022	0	2.64E-07	6.12E-13	24.7787	2.26112	434.691	8.18501	11.9912	NM_003641 // IFITM1 // interferon induced transmembrane protein 1 (9-27) // 11p1
20	3859761	0.16012	0.082833	0	2.68E-07	0.118072	2.34406	2.63155	279.981	3.80567	1.58542	NM_033317 // ZD52F10 // dermokine // 19q13.12 // 93099 /// AY358412 // ZD52F10 /
16	2758043	0.010111	0.17914	0	2.72E-07	8.34E-08	10.5167	1.88647	113.484	4.3493	6.09729	NM_001120 // TETRAN // tetracycline transporter-like protein // 4p16.3 // 10227
21	3923218	0.001714	0.447246	0	2.80E-07	2.82E-06	19.3841	1.09518	107.035	3.69435	4.9445	NM_015056 // KIAA0179 // KIAA0179 // 21q22.3 // 23076 /// AK124620 // KIAA0179 /
7	3080283	0.001909	0.142196	3.66E-35	2.97E-07	0.022372	18.7359	2.09958	70.8044	8.12434	2.27598	CR749256 // XRCC2 // X-ray repair complementing defective repair in Chinese hams
29	3851651	0.011454	0.677952	0	3.07E-07	4.61E-25	10.0186	0.728122	132.891	3.10569	17.3358	NM_001013652 // LOC389791 // hypothetical gene supported by AK094537 // 9q34.11
42	2449559	0.002103	0.147211	0	3.36E-07	5.27E-11	18.1688	2.06711	112.079	2.6138	7.78433	NM_001994 // F13B // coagulation factor XIII, B polypeptide // 1q31-q32.1 // 216
14	3891447	0.000705	0.062319	0	3.38E-07	0.259088	25.3465	2.93351	99.8636	4.6918	1.26114	NM_207034 // EDN3 // endothelin 3 // 20q13.2-q13.3 // 1908 /// NM_207033 // EDN3
27	3565571	0.001103	0.1777	0	3.40E-07	3.62E-09	22.1955	1.89379	73.3567	3.20565	6.77686	NM_001008396 // WDHD1 // WD repeat and HMG-box DNA binding protein 1 // 14q22.2
11	2438531	0.000142	0.038103	0	3.40E-07	0.000849	39.6213	3.49681	117.469	5.55063	3.33641	NM_004494 // HDGF // hepatoma-derived growth factor (high-mobility group protein
21	2805786	0.003866	0.108758	0	3.54E-07	2.62E-24	14.8742	2.35718	194.961	3.65748	17.7083	NM_152295 // TARS // threonyl-tRNA synthetase // 5p13.2 // 6897 /// BC003699 //
34	3557851	0.00536	0.075964	0	3.66E-07	1.02E-15	13.2848	2.7217	131.038	2.86069	10.8591	NM_024658 // IPO4 // importin 4 // 14q11.2 // 79711 /// AK094897 // IPO4 // impo
23	3064293	0.014003	0.079165	0	3.71E-07	4.33E-16	9.24562	2.67853	72.7506	3.47267	11.5168	NM_004444 // EPHB4 // EPH receptor B4 // 7q22 // 2050 /// AB209644 // EPHB4 // E
9	2690956	0.004612	0.228087	0	3.76E-07	4.40E-08	14.0014	1.67102	72.1777	6.44329	6.77908	NM_022135 // POPDC2 // popeye domain containing 2 // 3q13.33 // 64091 /// NM_005
26	3950872	0.323348	0.015473	0	4.00E-07	0.015505	1.09169	4.68771	226.072	3.24676	2.30263	NM_014551 // hCAP-H2 // kleisin beta // 22q13.33 // 29781 /// NM_152299 // hCAP-
17	3923312	0.000103	0.206555	0	4.04E-07	0.1932	43.1279	1.75863	84.434	4.11241	1.38738	NM_003683 // D21S2056E // DNA segment on chromosome 21 (unique) 2056 expressed s
13	2976041	0.004066	0.519065	0	4.08E-07	1.74E-05	14.6214	0.967763	192.066	4.88395	4.53825	NM_014432 // IL20RA // interleukin 20 receptor, alpha // 6q22.33-q23.1 // 53832
13	3015911	0.068285	0.25803	0	4.50E-07	1.49E-05	4.28784	1.56347	116.775	4.85919	4.58698	NM_003302 // TRIP6 // thyroid hormone receptor interactor 6 // 7q22 // 7205 ///
18	2624074	0.007848	0.22393	0	4.53E-07	9.28E-17	11.5736	1.68719	134.046	3.95268	12.397	NM_206826 // GNL3 // guanine nucleotide binding protein-like 3 (nucleolar) // 3p
13	3771800	0.011515	0.109328	0	4.59E-07	4.23E-12	9.99761	2.35205	194.966	4.85407	9.44901	NM_003016 // SFRS2 // splicing factor, arginine/serine-rich 2 // 17q25.1 // 6427
13	2516023	0.009955	0.493199	0	4.65E-07	1.92E-27	10.5797	1.01175	141.613	4.85061	23.2128	NM_031942 // CDCA7 // cell division cycle associated 7 // 2q31 // 83879 /// NM_1
8	3414739	0.010545	0.190135	0	4.71E-07	4.61E-09	10.3473	1.83268	268.432	7.00696	7.78771	XM_375589 // LOC400681 // hypothetical gene supported by BC041864 // 19p12 // 40
8	2923868	0.001605	0.175097	1.77E-41	4.72E-07	0.473146	19.7845	1.9072	73.6225	7.00642	0.963848	NM_181795 // PKIB // protein kinase (cAMP-dependent, catalytic) inhibitor beta /
11	2446567	0.00329	0.102172	0	4.82E-07	0.000181	15.7048	2.41894	116.49	5.44515	3.84293	NM_005819 // STX6 // syntaxin 6 // 1q25.3 // 10228 /// BC039118 // STX6 // synta
18	2458742	0.000706	0.437021	0	5.25E-07	0.334677	25.3332	1.11481	48.5845	3.92565	1.13906	NM_173083 // LIN9 // lin-9 homolog (C. elegans) // 1q42.12 // 286826 /// XM_4977
51	2727226	0.7655	0.024919	0	5.34E-07	1.08E-25	0.094523	4.03025	149.677	2.38079	16.7494	XM_497964 // LOC442108 // similar to LOC200916 protein // 4q12 // 442108 /// XM_
21	3653123	0.016987	0.406632	0	5.62E-07	2.17E-32	8.53589	1.17585	54.7764	3.5846	24.2471	NM_212535 // PRKCB1 // protein kinase C, beta 1 // 16p11.2 // 5579 /// NM_002738
15	3935016	0.006911	0.195922	0	5.79E-07	0.002393	12.1282	1.80578	85.7337	4.36736	2.94885	NM_003056 // SLC19A1 // solute carrier family 19 (folate transporter), member 1
22	3066436	0.00195	0.747477	0	6.00E-07	1.21E-14	18.6118	0.632041	44.2094	3.48487	10.5732	NM_019042 // FLJ20485 // hypothetical protein FLJ20485 // 7q22.3 // 54517
30	3457101	0.006412	0.826864	0	6.02E-07	0	12.4625	0.521525	84.2152	2.9773	33.2542	NM_002206 // ITGA7 // integrin, alpha 7 // 12q13 // 3679 /// AY358882 // ITGA7 /
11	3629243	0.007677	0.979475	0	6.11E-07	2.60E-14	11.6685	0.233102	111.292	5.37342	11.6207	NM_194272 // RBPMS2 // RNA binding protein with multiple splicing 2 // 15q22.31
20	2726542	0.001375	0.12268	0	6.16E-07	3.64E-13	20.7535	2.23999	37.645	3.66829	9.65761	NM_025087 // FLJ21511 // hypothetical protein FLJ21511 // 4p12-p11 // 80157
12	3725572	0.007444	0.617979	1.72E-30	6.30E-07	1.26E-22	11.8024	0.813899	23.5671	5.04268	18.9366	NM_153446 // GALGT2 // UDP-GalNAc:Neu5Acalpha2-3Galbeta-R beta1,4-N-acetylgalact
37	3497881	0.178422	0.84153	0	6.40E-07	0	2.13024	0.500337	65.1176	2.69966	68.6188	NM_005766 // FARP1 // FERM, RhoGEF (ARHGEF) and pleckstrin domain protein 1 (cho
17	3109687	0.00457	0.908011	0	6.41E-07	5.42E-19	14.0463	0.396149	70.3496	4.02302	14.2325	NM_024915 // GRHL2 // grainyhead-like 2 (Drosophila) // 8q22.3 // 79977 /// BC06
30	3458248	0.028678	0.834928	0	6.72E-07	0	6.76665	0.509925	106.467	2.96469	73.915	NM_005379 // MYO1A // myosin IA // 12q13-q15 // 4640
13	3764399	0.017006	0.438142	0	6.76E-07	0	8.53176	1.11263	58.0587	4.75609	60.0202	NM_017763 // FLJ20315 // hypothetical protein FLJ20315 // 17q23.2 // 54894 /// A
31	3643966	0.000231	0.254873	0	6.92E-07	0.1279	34.7151	1.57414	74.5479	2.9159	1.54824	NM_144570 // C16orf34 // chromosome 16 open reading frame 34 // 16p13.3 // 90861
31	3173880	0.028757	0.727454	0	6.96E-07	6.39E-37	6.75797	0.659545	177.988	2.91516	25.7226	NM_201629 // TJP2 // tight junction protein 2 (zona occludens 2) // 9q13-q21 //
16	3174121	0.006912	0.692384	0	7.18E-07	2.72E-31	12.1275	0.70799	36.1458	4.15066	25.2509	NM_153267 // MAMDC2 // MAM domain containing 2 // 9q21.11 // 256691 /// NM_00098
29	2409004	0.006245	0.312436	0	7.46E-07	1.19E-09	12.5812	1.39919	82.3488	3.00057	7.06116	NM_022356 // LEPRE1 // leucine proline-enriched proteoglycan (leprecan) 1 // 1p3
12	3959388	0.019367	0.307556	2.85E-38	7.49E-07	0.074598	8.07219	1.41262	32.0879	4.99498	1.77695	NM_145660 // APOL4 // apolipoprotein L, 4 // 22q11.2-q13.2 // 80832 /// NM_03064
9	3595979	0.000377	0.306923	0	7.56E-07	0.000822	30.3148	1.41437	74.4586	6.17754	3.39746	NM_004701 // CCNB2 // cyclin B2 // 15q22.2 // 9133

10	3142967	0.002732	0.042761	0	7.59E-07	6.82E-10	16.6984	3.35975	68.6355	5.69382	8.11184	NM_006774 // INMT // indolethylamine N-methyltransferase // 7p15.3-p15.2 // 1118
41	3571944	0.016633	0.250833	0	7.68E-07	0	8.61168	1.58801	56.5769	2.56747	35.4772	NM_000428 // LTBP2 // latent transforming growth factor beta binding protein 2 /
12	4018080	0.007979	0.755269	0	7.86E-07	7.64E-18	11.503	0.621337	85.3707	4.98172	14.431	NM_145234 // CHRDL1 // chordin-like 1 // Xq23 // 91851 /// AK092245 // CHRDL1 //
13	3577443	0.006881	0.844755	0	7.97E-07	1.60E-08	12.1474	0.495621	60.4322	4.71462	6.74973	NM_016150 // ASB2 // ankyrin repeat and SOCS box-containing 2 // 14q31-q32 // 51
20	2965206	0.000442	0.010414	0	8.06E-07	2.57E-09	28.9985	5.2869	31.5763	3.6238	7.01665	NM_004440 // EPHA7 // EPH receptor A7 // 6q16.1 // 2045 /// BC027940 // EPHA7 //
18	2951674	0.007621	0.285546	0	8.21E-07	1.53E-28	11.7005	1.47614	99.7084	3.84398	21.8755	NM_003137 // SRPK1 // SFRS protein kinase 1 // 6p21.3-p21.2 // 6732 /// AJ318054
35	3402571	0.002268	0.075393	0	8.47E-07	5.12E-33	17.7351	2.72962	185.231	2.73831	22.3936	NM_014865 // CNAP1 // chromosome condensation-related SMC-associated protein 1 /
13	2328868	0.13397	0.313635	0	8.83E-07	1.17E-13	2.7125	1.39593	367.736	4.68868	10.6736	NM_004964 // HDAC1 // histone deacetylase 1 // 1p34 // 3065 /// BX648055 // HDAC
10	2321182	0.007942	0.535024	0	9.10E-07	5.43E-09	11.5229	0.941525	145.281	5.63283	7.38186	NM_001006624 // PDPN // podoplanin // 1p36.21 // 10630 /// NM_001006625 // PDPN
7	2600218	0.019744	0.241868	0	9.26E-07	0.000726	8.00515	1.61969	144.938	7.53237	3.52738	NM_024536 // CHPF // chondroitin polymerizing factor // 2q35 // 79586 /// BC0088
55	3300597	0.012728	0.073921	0	9.36E-07	0	9.60844	2.75039	143.847	2.27396	96.2545	NM_013451 // FER1L3 // fer-1-like 3, myoferlin (C. elegans) // 10q24 // 26509 //
34	3152220	0.001668	0.072016	0	9.59E-07	2.97E-20	19.5478	2.778	143.557	2.76091	13.7969	NM_014846 // KIAA0196 // KIAA0196 // 8p22 // 9897
13	2734047	0.00222	0.211343	0	9.60E-07	3.29E-11	17.8586	1.73828	59.9971	4.66764	8.76346	NM_032717 // MGC11324 // hypothetical protein MGC11324 // 4q21.23 // 84803 /// A
24	3038065	0.005313	0.300944	0	9.80E-07	1.40E-13	13.3262	1.43117	124.61	3.25899	9.75927	NM_022307 // ICA1 // islet cell autoantigen 1, 69kDa // 7p22 // 3382 /// NM_0049
26	3644541	0.001384	0.008329	0	9.87E-07	4.17E-06	20.7135	5.64802	155.76	3.12895	4.78368	NM_032271 // TRAF7 // TNF receptor-associated factor 7 // 16p13.3 // 84231 /// N
25	3765580	0.00432	0.363225	0	1.01E-06	1.13E-16	14.3215	1.27134	73.9726	3.18728	11.8073	NM_032043 // BRIP1 // BRCA1 interacting protein C-terminal helicase 1 // 17q22-q
20	3674199	0.008489	0.517729	0	1.03E-06	3.11E-08	11.2394	0.96999	54.5711	3.5824	6.28277	NM_014427 // CPNE7 // copine VII // 16q24.3 // 27132 /// NM_153636 // CPNE7 // c
34	3590014	0.00019	0.06889	0	1.05E-06	6.82E-05	36.6246	2.82526	56.0288	2.75128	3.9428	NM_144508 // CASC5 // cancer susceptibility candidate 5 // 15q14 // 57082 /// NM
18	3820414	0.005993	0.003864	0	1.11E-06	0.02526	12.7686	7.03101	163.413	3.78879	2.15191	NM_146388 // MRPL4 // mitochondrial ribosomal protein L4 // // 51073 /// NM_146
14	2717857	0.009391	0.411969	0	1.20E-06	0.000341	10.8187	1.16482	83.4968	4.39517	3.5809	NM_001014447 // CPZ // carboxypeptidase Z // 4p16.1 // 8532 /// NM_001014448 //
12	3262535	0.002741	0.094416	0	1.22E-06	0.224979	16.6795	2.49797	123.926	4.86179	1.32672	NM_183239 // GSTO2 // glutathione S-transferase omega 2 // 10q25.1 // 119391 ///
6	3394315	0.017149	0.17044	8.26E-10	1.24E-06	0.008374	8.50178	1.93177	13.4787	8.51642	2.67834	NM_015645 // C1QTNF5 // C1q and tumor necrosis factor related protein 5 // 11q23
28	3489020	0.004576	0.109486	0	1.31E-06	7.44E-16	14.0402	2.35062	101.244	2.98272	11.1265	NM_000321 // RB1 // retinoblastoma 1 (including osteosarcoma) // 13q14.2 // 5925
26	3026599	0.000366	0.113449	0	1.32E-06	2.46E-07	30.5644	2.31582	59.641	3.09115	5.59396	NM_003852 // TRIM24 // tripartite motif-containing 24 // 7q32-q34 // 8805 /// NM
14	3955185	0.043466	0.19555	0	1.33E-06	1.20E-10	5.51188	1.80748	81.7022	4.37068	8.2482	NM_004121 // GGTLA1 // gamma-glutamyltransferase-like activity 1 // 22q11.23 //
9	2342738	0.953957	0.034117	0	1.36E-06	0.579205	0.003524	3.63119	93.6708	5.95413	0.841458	NM_152996 // ST6GALNAC3 // ST6 (alpha-N-acetyl-neuraminyl-2,3-beta-galactosyl-1,
27	3923257	0.001915	0.349064	0	1.41E-06	1.51E-15	18.7195	1.30501	211.361	3.02572	10.9606	NM_032920 // C21orf124 // chromosome 21 open reading frame 124 // 21q22.3 // 850
23	3893287	0.000602	0.444902	0	1.50E-06	0.009707	26.5326	1.09964	232.505	3.26955	2.46201	NM_018209 // ARFGAP1 // ADP-ribosylation factor GTPase activating protein 1 // 2
14	3173974	0.007892	0.283961	0	1.50E-06	0.000176	11.5498	1.48091	58.1635	4.34169	3.79054	NM_004816 // C9orf61 // chromosome 9 open reading frame 61 // 9q13-q21 // 9413 /
27	3421177	0.00913	0.156783	0	1.57E-06	5.40E-23	10.935	2.00854	67.5399	3.01271	16.0483	NM_020401 // NUP107 // nucleoporin 107kDa // 12q15 // 57122
43	2450345	0.000515	0.085087	0	1.57E-06	0.000966	27.7622	2.60389	38.9601	2.45586	3.15081	NM_031306 // DDX59 // DEAD (Asp-Glu-Ala-Asp) box polypeptide 59 // 1q32.1 // 834
6	3838425	0.409805	0.782692	4.15E-37	1.60E-06	0.025472	0.747219	0.583547	112.141	8.35434	2.25149	NM_014419 // DKKL1 // dickkopf-like 1 (soggy) // 19q13.33 // 27120 /// AF177398
4	3201319	0.040371	0.322183	8.86E-27	1.67E-06	0.391665	5.72553	1.37304	159.024	12.9954	1.08114	NM_000605 // IFNA2 // interferon, alpha 2 // 9p22 // 3440 /// NM_006900 // IFNA1
8	3665501	0.00222	0.913262	0	1.72E-06	4.21E-16	17.8582	0.386971	126.904	6.4425	14.7286	NM_000196 // HSD11B2 // hydroxysteroid (11-beta) dehydrogenase 2 // 16q22 // 329
17	3721452	0.011238	0.233181	0	1.74E-06	3.83E-15	10.0935	1.65166	62.3776	3.83018	11.3029	NM_021939 // FKBP10 // FK506 binding protein 10, 65 kDa // 17q21.2 // 60681 ///
40	3257338	0.001913	0.089023	0	1.81E-06	2.76E-07	18.724	2.55761	14.9797	2.51576	5.46999	NM_016195 // MPHOSPH1 // M-phase phosphoprotein 1 // 10q23.31 // 9585 /// AL1373
45	3194635	0.269313	0.197217	0	1.86E-06	1.48E-10	1.38577	1.79989	111.47	2.39815	7.48642	NM_024718 // C9orf86 // chromosome 9 open reading frame 86 // 9q34.3 // 55684 //
16	2881860	0.009685	0.359575	0	1.87E-06	2.80E-22	10.6919	1.27989	285.668	3.95375	17.1017	NM_015621 // DKFZP434C171 // DKFZP434C171 protein // 5q33.1 // 26112 /// BC01664
10	3756566	0.01234	0.798119	0	1.88E-06	1.82E-35	9.72755	0.562111	192.355	5.39049	37.8189	NM_019010 // KRT20 // keratin 20 // 17q21.2 // 54474
70	3741875	0.009865	0.675667	0	1.90E-06	0	10.6167	0.731324	113.03	2.0481	59.7711	NM_015113 // ZZEF1 // zinc finger, ZZ-type with EF-hand domain 1 // 17p13.2 // 2
5	3434012	0.003537	0.890248	1.26E-29	1.92E-06	9.02E-06	15.3288	0.425851	107.841	9.90585	5.53327	NM_014891 // PDAP1 // PDGFA associated protein 1 // 7q22.1 // 11333 /// NM_01436
6	2570193	0.008382	0.695987	0	2.04E-06	5.53E-13	11.2934	0.702985	401.714	8.20289	12.9157	XM_496582 // LOC339692 // hypothetical protein LOC339692 // 2q13 // 339692 /// N
17	3833992	0.001383	0.016964	0	2.07E-06	5.30E-11	20.7138	4.55565	48.1182	3.79659	8.30357	NM_030622 // CYP2S1 // cytochrome P450, family 2, subfamily S, polypeptide 1 //
34	3643143	0.024361	0.319699	0	2.10E-06	0.00013	7.29441	1.37962	180.774	2.67892	3.75652	NM_032371 // MGC15416 // hypothetical protein MGC15416 // 16p13.3 // 84331 /// N
25	2496382	0.054708	0.302332	0	2.11E-06	0	4.87107	1.42724	170.068	3.08735	49.2249	NM_002518 // NPAS2 // neuronal PAS domain protein 2 // 2q11.2 // 4862 /// AK1285
23	3217194	0.001612	0.216193	0	2.17E-06	8.61E-08	19.7562	1.7182	59.8805	3.21484	5.93158	NM_018421 // TBC1D2 // TBC1 domain family, member 2 // 9q22.33 // 55357 /// AL35
13	3571667	0.072054	0.917754	0	2.18E-06	3.24E-29	4.15147	0.378945	139.293	4.46077	25.0907	NM_001249 // ENTPD5 // ectonucleoside triphosphate diphosphohydrolase 5 // 14q24
40	2886174	0.071166	0.926713	0	2.19E-06	0	4.1828	0.362387	141.19	2.49843	39.2671	NM_003062 // SLIT3 // slit homolog 3 (Drosophila) // 5q35 // 6586 /// XM_496054
16	4021341	0.000281	0.012795	0	2.20E-06	3.04E-16	32.9219	4.96904	181.038	3.92057	12.2527	NM_001008222 // ZDHHC9 // zinc finger, DHHC-type containing 9 // Xq26.1 // 51114
6	3839346	0.006493	0.162081	8.91E-28	2.23E-06	0.090653	12.406	1.97788	62.4566	8.14817	1.74306	NM_003121 // SPIB // Spi-B transcription factor (Spi-1/PU.1 related) // 19q13.3-
21	3599811	0.000343	0.301407	0	2.27E-06	5.42E-09	31.1275	1.42986	101.988	3.36339	6.77127	NM_004856 // KIF23 // kinesin family member 23 // 15q23 // 9493 /// NM_138555 //
15	2434716	0.013424	0.130327	0	2.27E-06	9.34E-13	9.40501	2.18206	358.02	4.06861	9.72753	NM_022075 // LASS2 // LAG1 longevity assurance homolog 2 (S. cerevisiae) // 1q21

42	3544678	0.002988	0.058885	0	2.32E-06	2.98E-16	16.2138	2.99565	167.311	2.44456	11.0423	NM_015072 // TTLL5 // tubulin tyrosine ligase-like family, member 5 // 14q24.3 /
23	3771602	0.003502	0.22366	0	2.34E-06	1.34E-07	15.3791	1.68825	77.7733	3.2037	5.80337	NM_001005498 // RHBDL6 // rhomboid, veinlet-like 6 (Drosophila) // 17q25.1 // 79
34	3011492	0.03681	0.249747	0	2.37E-06	1.90E-06	5.9982	1.59178	73.1276	2.666	4.96005	NM_004194 // ADAM22 // ADAM metallopeptidase domain 22 // 7q21 // 53616 /// NM_0
10	3862167	0.022003	0.472147	0	2.46E-06	1.60E-15	7.63432	1.04906	225.537	5.29982	13.0303	NM_001436 // FBL // fibrillarin // 19q13.1 // 2091 /// X56597 // FBL // fibrilla
9	2366798	0.003835	0.287436	2.74E-34	2.51E-06	1.79E-10	14.9156	1.47048	42.5653	5.72397	8.78933	NM_022716 // PRRX1 // paired related homeobox 1 // 1q24 // 5396 /// NM_006902 //
18	2812359	0.002607	0.379646	0	2.52E-06	1.28E-06	16.9561	1.23393	116.663	3.63804	5.22322	XM_372764 // LOC391004 // novel protein similar to preferentially expressed anti
15	3887017	0.005053	0.718092	0	2.61E-06	5.77E-09	13.5637	0.67243	137.631	4.03781	6.95918	NM_052951 // DNTTIP1 // deoxynucleotidyltransferase, terminal, interacting prote
14	3651152	0.005667	0.152593	0	2.67E-06	1.57E-07	13.0255	2.03365	122.544	4.20755	5.97697	NM_153208 // MGC35048 // hypothetical protein MGC35048 // 16p12.3 // 124152 ///
18	3598758	0.020214	0.436171	0	2.70E-06	0.016132	7.92382	1.11646	160.893	3.62552	2.30538	NM_005585 // SMAD6 // SMAD, mothers against DPP homolog 6 (Drosophila) // 15q21-
