# Supplementary material for: Comparison of Two Methods for Detecting Alternative Splice Variants Using GeneChip® Exon Arrays
Source: Int J Biomed Sci. 2011 Sep;7(3):172–80. (PMC3614835)
Supplement: Supplementary file 3 [file IJBS-7-172_SD2.pdf]

Additional file 2. Annotation for alternative splice variants selected by Partek® GS

Transcript Cluster ID	Exon Cluster ID	Probe Set ID	Probe Set Grade	Current Genome Chromosome	Current Genome Transcript Start	Current Genome Transcript Stop	Current Genome Strand	Transcript ID (Annotated)	Representative Transcript Description
2321182	3732<br>3733<br>3734<br>3735<br>3736<br>3737<br>3738<br>3739<br>3740<br>3741<br>3742<br>3743<br>3744<br>3745<br>3746<br>3747<br>3748<br>3749<br>3750<br>3751<br>3752<br>3753<br>3754<br>3755<br>3756<br>3757<br>3758<br>3759<br>3760<br>3761<br>3762<br>3763	2321183<br>2321184<br>2321185<br>2321186<br>2321187<br>2321188<br>2321189<br>2321190<br>2321191<br>2321192<br>2321193<br>2321194<br>2321195<br>2321196<br>2321197<br>2321198<br>2321199<br>2321200<br>2321201<br>2321202<br>2321203<br>2321204<br>2321205<br>2321206<br>2321207<br>2321208<br>2321209<br>2321210<br>2321211<br>2321212<br>2321213<br>2321214<br>2321215<br>2321216<br>2321217<br>2321218<br>2321219<br>2321220<br>2321221<br>2321222	full<br>full<br>full<br>full<br>full<br>full<br>full<br>extended<br>full<br>full<br>full<br>extended<br>core<br>core<br>core<br>extended<br>extended<br>full<br>extended<br>full<br>core<br>core<br>full<br>core<br>core<br>core<br>core<br>core<br>extended<br>extended<br>extended<br>core<br>core<br>full<br>full<br>full<br>full<br>extended<br>extended<br>full	1	13724708	13852576	+	NM_001006625<br>NM_006474<br>NM_001006624<br>NM_198389<br>AF030427<br>AF030428<br>ENSESTT00000042457<br>ENSESTT00000042458<br>ENST00000376061<br>ENST00000376057<br>ENST00000294489<br>GENSCAN00000063950<br>GENSCAN00000034525	Homo sapiens podoplanin (PDPN), transcript variant 4, mRNA.<br>Homo sapiens podoplanin (PDPN), transcript variant 1, mRNA.<br>Homo sapiens podoplanin (PDPN), transcript variant 3, mRNA.<br>Homo sapiens podoplanin (PDPN), transcript variant 2, mRNA.<br>Homo sapiens lung type-I cell membrane-associated protein hT1a-1 (hT1a-1) mRNA, complete cds.<br>Homo sapiens lung type-I cell membrane-associated protein hT1a-2 (hT1a-2) mRNA, complete cds.<br><br><br>cdna:known chromosome:NCBI36:1:13782814:13817039:1 gene:ENSG00000162493<br>cdna:known chromosome:NCBI36:1:13782814:13817039:1 gene:ENSG00000162493<br>cdna:known chromosome:NCBI36:1:13782839:13817035:1 gene:ENSG00000162493<br>cdna:Genscan chromosome:NCBI36:1:13721668:13800326:1<br>cdna:Genscan chromosome:NCBI36:1:13805801:13865828:1
2328868	8406<br>8407<br>8408<br>8409<br>8410<br>8411<br>8412<br>8413<br>8414	2328869<br>2328870<br>2328871<br>2328872<br>2328873<br>2328874<br>2328875<br>2328876<br>2328877	core<br>core<br>extended<br>core<br>core<br>extended<br>extended<br>full<br>extended	1	32530294	32572813	+	NM_004964<br>AK225555<br>U50079<br>BX648055<br>ENSESTT00000029616<br>ENSESTT00000029617<br>ENSESTT00000029618<br>ENST00000373548<br>ENST00000271095	Homo sapiens histone deacetylase 1 (HDAC1), mRNA.<br>Homo sapiens mRNA for histone deacetylase 1 variant, clone: KDN05801.<br>Human histone deacetylase HD1 mRNA, complete cds.<br>Homo sapiens mRNA; cDNA DKFZp686H12203 (from clone DKFZp686H12203).<br><br><br><br>cdna:known-ccds chromosome:NCBI36:1:32530274:32571823:1 gene:ENSG00000116478 CCDS360.1<br>cdna:known chromosome:NCBI36:1:32530295:32571811:1 gene:ENSG00000116478

	8415<br>8416<br>8417<br>8418<br>8419<br>8420<br>8421<br>8422<br>8423<br>8424<br>8425<br>8426<br>8427<br>8428	2328878<br>2328879<br>2328880<br>2328881<br>2328882<br>2328883<br>2328884<br>2328885<br>2328886<br>2328887<br>2328888<br>2328889<br>2328890<br>2328891<br>2328892<br>2328893<br>2328894<br>2328895<br>2328896<br>2328897<br>2328898<br>2328899<br>2328900<br>2328901	core<br>core<br>extended<br>extended<br>core<br>extended<br>extended<br>core<br>extended<br>extended<br>extended<br>core<br>core<br>core<br>core<br>full<br>core<br>extended<br>core<br>extended<br>core<br>extended<br>core<br>extended					ENST00000373541<br>GENSCAN00000018653	cdna:known chromosome:NCBI36:1:32530295:32571811:1 gene:ENSG00000116478<br>cdna:Genscan chromosome:NCBI36:1:32530358:32571232:1
2342738	17053<br>17054<br>17055<br>17056<br>17057<br>17058<br>17059<br>17060<br>17061<br>17062<br>17063<br>17064<br>17065<br>17066<br>17067<br>17068<br>17069<br>17070<br>17071<br>17072<br>17073<br>17074<br>17075<br>17076<br>17077<br>17078<br>17079<br>17080<br>17081<br>17082	2342739<br>2342740<br>2342741<br>2342742<br>2342743<br>2342744<br>2342745<br>2342746<br>2342747<br>2342748<br>2342749<br>2342750<br>2342751<br>2342752<br>2342753<br>2342754<br>2342755<br>2342756<br>2342757<br>2342758<br>2342759<br>2342760<br>2342761<br>2342762<br>2342763<br>2342764<br>2342765<br>2342766<br>2342767<br>2342768	core<br>core<br>extended<br>extended<br>full<br>full<br>extended<br>extended<br>extended<br>full<br>full<br>extended<br>extended<br>extended<br>extended<br>full<br>full<br>full<br>full<br>extended<br>full<br>full<br>full<br>full<br>full<br>full<br>extended<br>core<br>extended<br>full	1	76313002	76875803	+	NM_152996<br>AK023731<br>AK130749<br>AY358540<br>BC041381<br>BX648274<br>BC039520<br>ENSESTT00000052237<br>ENST00000328299<br>ENST00000370816<br>GENSCAN00000040312<br>GENSCAN00000058732	Homo sapiens ST6 (alpha-N-acetyl-neuraminyl-2,3-beta-galactosyl-1, 3)-N-acetylgalactosaminide alpha-2,6-sialyltransferase 3 (ST6GALNAC3), mRNA.<br>Homo sapiens cDNA FLJ13669 fis, clone PLACE1011719.<br>Homo sapiens cDNA FLJ27239 fis, clone SYN07821.<br>Homo sapiens clone DNA108783 sialyltransferase (UNQ2787) mRNA, complete cds.<br>Homo sapiens cDNA clone IMAGE:5275249.<br>Homo sapiens mRNA; cDNA DKFZp686G18119 (from clone DKFZp686G18119).<br>Homo sapiens, Similar to alpha-N-acetylgalactosaminide alpha-2,6-sialyltransferase III, clone IMAGE:5724478, mRNA.<br><br>cdna:known-ccds chromosome:NCBI36:1:76312992:76872874:1 gene:ENSG00000184005 CCDS672.1<br>cdna:known chromosome:NCBI36:1:76313140:76867079:1 gene:ENSG00000184005<br>cdna:Genscan chromosome:NCBI36:1:76821566:76823901:1<br>cdna:Genscan chromosome:NCBI36:1:76647081:76651606:1

	17083	2342769	full						
	17084	2342770	full						
	17085	2342771	full						
	17086	2342772	full						
	17087	2342773	full						
	17088	2342774	full						
	17089	2342775	full						
	17090	2342776	full						
	17091	2342777	full						
	17092	2342778	full						
	17093	2342779	extended						
	17094	2342780	full						
	17095	2342781	full						
	17096	2342782	full						
	17097	2342783	extended						
	17098	2342784	full						
	17099	2342785	full						
	17100	2342786	full						
	17101	2342787	full						
	17102	2342788	full						
	17103	2342789	core						
	17104	2342790	core						
	17105	2342791	extended						
	17106	2342792	extended						
	17107	2342793	extended						
	17108	2342794	full						
	17109	2342795	extended						
	17110	2342796	extended						
	17111	2342797	full						
	17112	2342798	extended						
	17113	2342799	full						
	17114	2342800	extended						
	17115	2342801	full						
	17116	2342802	full						
	17117	2342803	full						
	17118	2342804	full						
	17119	2342805	full						
	17120	2342806	extended						
	17121	2342807	extended						
	17122	2342808	extended						
	17123	2342809	full						
	17124	2342810	extended						
		2342811	full						
		2342812	core						
		2342813	full						
		2342814	full						
		2342815	extended						
		2342816	extended						
		2342817	extended						
		2342818	extended						
		2342819	full						
		2342820	full						
		2342821	full						
		2342822	core						

		2342823<br>2342824<br>2342825<br>2342826<br>2342827<br>2342828	core<br>core<br>extended<br>extended<br>extended<br>extended						
2345023	18517<br>18518<br>18519<br>18520<br>18521<br>18522<br>18523<br>18524<br>18525<br>18526<br>18527<br>18528<br>18529<br>18530<br>18531<br>18532<br>18533<br>18534<br>18535<br>18536	2345024<br>2345025<br>2345026<br>2345027<br>2345028<br>2345029<br>2345030<br>2345031<br>2345032<br>2345033<br>2345034<br>2345035<br>2345036<br>2345037<br>2345038<br>2345039<br>2345040<br>2345041<br>2345042<br>2345043<br>2345044<br>2345045<br>2345046<br>2345047<br>2345048<br>2345049<br>2345050<br>2345051<br>2345052<br>2345053<br>2345054<br>2345055<br>2345056	core<br>core<br>core<br>core<br>core<br>core<br>core<br>core<br>core<br>core<br>core<br>core<br>core<br>core<br>core<br>full<br>core<br>core<br>core<br>full<br>core<br>core<br>core<br>full<br>core<br>extended<br>core<br>core<br>core<br>core<br>core<br>extended<br>extended	1	86706639	86739141	+	NM_001285<br>ENSESTT00000002539<br>ENSESTT00000002540<br>ENSESTT00000032588<br>ENST00000234701<br>ENST00000353619<br>GENSCAN00000056092	Homo sapiens chloride channel, calcium activated, family member 1 (CLCA1), mRNA.<br><br><br><br>cdna:known-ccds chromosome:NCBI36:1:86706639:86738532:1 gene:ENSG00000016490 CCDS709.1<br>cdna:known chromosome:NCBI36:1:86706639:86738530:1 gene:ENSG00000016490<br>cdna:Genscan chromosome:NCBI36:1:86707243:86738316:1
2345061	18538<br>18539<br>18540<br>18541<br>18542<br>18543<br>18544<br>18545<br>18546<br>18547<br>18548<br>18549<br>18550<br>18551	2345062<br>2345063<br>2345064<br>2345065<br>2345066<br>2345067<br>2345068<br>2345069<br>2345070<br>2345071<br>2345072<br>2345073<br>2345074<br>2345075	full<br>full<br>full<br>full<br>full<br>full<br>core<br>core<br>full<br>full<br>full<br>core<br>core<br>core	1	86744324	86826946	+	NM_012128<br>ENSESTT00000002541<br>ENSESTT00000002542<br>ENSESTT00000002543<br>ENSESTT00000002544<br>ENSESTT00000002545<br>ENSESTT00000002546<br>ENSESTT00000002547<br>ENST00000370563<br>ENST00000263723<br>GENSCAN00000056093	Homo sapiens chloride channel, calcium activated, family member 4 (CLCA4), mRNA.<br><br><br><br><br><br><br><br>cdna:known chromosome:NCBI36:1:86785349:86819025:1 gene:ENSG00000016602<br>cdna:known chromosome:NCBI36:1:86785349:86819018:1 gene:ENSG00000016602<br>cdna:Genscan chromosome:NCBI36:1:86744324:86818616:1

	18552<br>18553<br>18554<br>18555<br>18556<br>18557<br>18558<br>18559<br>18560<br>18561<br>18562	2345076<br>2345077<br>2345078<br>2345079<br>2345080<br>2345081<br>2345082<br>2345083<br>2345084<br>2345085<br>2345086<br>2345087<br>2345088<br>2345089<br>2345090<br>2345091<br>2345092<br>2345093<br>2345094	core<br>core<br>core<br>core<br>core<br>core<br>core<br>extended<br>extended<br>core<br>core<br>full<br>core<br>full<br>core<br>core<br>core<br>core<br>full						
2358320	26546<br>26547<br>26548<br>26549<br>26550<br>26551<br>26552<br>26553<br>26554<br>26555<br>26556<br>26557<br>26558<br>26559<br>26560<br>26561<br>26562<br>26563<br>26564<br>26565<br>26566<br>26567<br>26568	2358321<br>2358322<br>2358323<br>2358324<br>2358325<br>2358326<br>2358327<br>2358328<br>2358329<br>2358330<br>2358331<br>2358332<br>2358333<br>2358334<br>2358335<br>2358336<br>2358337<br>2358338<br>2358339<br>2358340<br>2358341<br>2358342<br>2358343<br>2358344<br>2358345<br>2358346<br>2358347<br>2358348<br>2358349<br>2358350<br>2358351<br>2358352<br>2358353<br>2358354<br>2358355	extended<br>core<br>core<br>core<br>core<br>extended<br>core<br>extended<br>core<br>core<br>core<br>core<br>extended<br>extended<br>core<br>extended<br>extended<br>extended<br>core<br>core<br>core<br>extended<br>core<br>full<br>core<br>core<br>core<br>core<br>extended<br>core<br>core<br>extended<br>core<br>core<br>full	1	148726513	148746702	+	NM_025150<br>AK022590<br>ENSESTT00000021208<br>ENSESTT00000021209<br>ENSESTT00000021210<br>ENSESTT00000021211<br>ENSESTT00000021212<br>ENSESTT00000021213<br>ENSESTT00000021214<br>ENSESTT00000021215<br>ENSESTT00000021216<br>ENST00000369064<br>ENST00000290387<br>ENST00000271635<br>ENST00000369054<br>ENST00000369053<br>ENST00000369052<br>ENST00000369051<br>GENSCAN00000030801	Homo sapiens threonyl-tRNA synthetase-like 1 (TARSL1), mRNA.<br>Homo sapiens cDNA FLJ12528 fis, clone NT2RM4000155, moderately similar to THREONYL-TRNA SYNTHETASE, CYTOPLASMIC (EC 6.1.1.3).<br>cdna:known-ccds chromosome:NCBI36:1:148726517:148746702:1 gene:ENSG00000143374 CCDS952.1<br>cdna:known chromosome:NCBI36:1:148726514:148746358:1 gene:ENSG00000143374<br>cdna:known chromosome:NCBI36:1:148726517:148746369:1 gene:ENSG00000143374<br>cdna:known chromosome:NCBI36:1:148726517:148746369:1 gene:ENSG00000143374<br>cdna:known chromosome:NCBI36:1:148726531:148728408:1 gene:ENSG00000143374<br>cdna:known chromosome:NCBI36:1:148726535:148746702:1 gene:ENSG00000143374<br>cdna:known chromosome:NCBI36:1:148726535:148746698:1 gene:ENSG00000143374<br>cdna:Genscan chromosome:NCBI36:1:148711974:148746164:1

		2358356<br>2358357<br>2358358<br>2358359	core<br>core<br>core<br>core						
2362892	29078<br>29079<br>29080<br>29081<br>29082<br>29083<br>29084<br>29085<br>29086<br>29087<br>29088<br>29089<br>29090<br>29091<br>29092<br>29093<br>29094<br>29095<br>29096<br>29097<br>29098<br>29099<br>29100<br>29101<br>29102<br>29103<br>29104<br>29105<br>29106<br>29107<br>29108<br>29109<br>29110<br>29111<br>29112<br>29113	2362893<br>2362894<br>2362895<br>2362896<br>2362897<br>2362898<br>2362899<br>2362900<br>2362901<br>2362902<br>2362903<br>2362904<br>2362905<br>2362906<br>2362907<br>2362908<br>2362909<br>2362910<br>2362911<br>2362912<br>2362913<br>2362914<br>2362915<br>2362916<br>2362917<br>2362918<br>2362919<br>2362920<br>2362921<br>2362922<br>2362923<br>2362924<br>2362925<br>2362926<br>2362927<br>2362928<br>2362929<br>2362930<br>2362931<br>2362932<br>2362933<br>2362934<br>2362935<br>2362936<br>2362937<br>2362938<br>2362939<br>2362940<br>2362941<br>2362942	full<br>full<br>full<br>core<br>full<br>full<br>full<br>core<br>extended<br>core<br>extended<br>core<br>core<br>core<br>core<br>core<br>extended<br>extended<br>core<br>core<br>core<br>core<br>core<br>core<br>full<br>full<br>core<br>core<br>extended<br>full<br>core<br>core<br>core<br>core<br>core<br>core<br>full<br>full<br>core<br>core<br>core<br>extended<br>extended<br>core<br>extended<br>core<br>core<br>extended<br>extended<br>core	1	158336589	158380853	+	NM_144699<br>NM_000702<br>AK091617<br>AK098076<br>AK125812<br>Y07494<br>ENSESTT00000025415<br>ENSESTT00000025416<br>ENSESTT00000025417<br>ENSESTT00000025418<br>ENST00000361216<br>ENST00000368081<br>ENST00000251207<br>ENST00000368084<br>ENST00000255017<br>ENST00000368080<br>GENSCAN00000041574	Homo sapiens ATPase, Na+/K+ transporting, alpha 4 polypeptide (ATP1A4), transcript variant 1, mRNA.<br>Homo sapiens ATPase, Na+/K+ transporting, alpha 2 (+) polypeptide (ATP1A2), mRNA.<br>Homo sapiens cDNA FLJ34298 fis, clone FEBRA2006357, highly similar to SODIUM/POTASSIUM-TRANSPORTING ATPASE ALPHA-2 CHAIN PRECURSOR (EC 3.6.1.37).<br>Homo sapiens cDNA FLJ40757 fis, clone TRACH2001996, highly similar to SODIUM/POTASSIUM-TRANSPORTING ATPASE ALPHA-1 CHAIN PRECURSOR (EC 3.6.1.37).<br>Homo sapiens cDNA FLJ43824 fis, clone TESTI4002552, highly similar to Sodium/potassium-transporting ATPase alpha-4 chain (EC 3.6.3.9).<br>H. sapiens alpha subunit and regulatory region of Na+, K+ -ATPase.<br><br><br><br><br><br>cdna:known-ccds chromosome:NCBI36:1:158352172:158379996:1 gene:ENSG00000018625 CCDS1196.1<br>cdna:known-ccds chromosome:NCBI36:1:158387984:158423391:1 gene:ENSG00000132681 CCDS1197.1<br>cdna:known chromosome:NCBI36:1:158352187:158380005:1 gene:ENSG00000018625<br>cdna:known chromosome:NCBI36:1:158352191:158377959:1 gene:ENSG00000018625<br>cdna:known chromosome:NCBI36:1:158387987:158423387:1 gene:ENSG00000132681<br>cdna:known chromosome:NCBI36:1:158407818:158419842:1 gene:ENSG00000132681<br>cdna:Genscan chromosome:NCBI36:1:158352276:158423113:1

		2362943<br>2362944<br>2362945<br>2362946<br>2362947<br>2362948<br>2362949	core<br>core<br>core<br>core<br>core<br>core<br>extended						
2366798	31485<br>31486<br>31487<br>31488<br>31489<br>31490<br>31491<br>31492<br>31493<br>31494<br>31495<br>31496<br>31497<br>31498<br>31499<br>31500<br>31501<br>31502<br>31503<br>31504<br>31505<br>31506<br>31507<br>31508<br>31509<br>31510<br>31511<br>31512<br>31513<br>31514<br>31515<br>31516<br>31517<br>31518<br>31519<br>31520<br>31521<br>31522<br>31523<br>31524<br>31525<br>31526<br>31527<br>31528<br>31529<br>31530<br>31531	2366799<br>2366800<br>2366801<br>2366802<br>2366803<br>2366804<br>2366805<br>2366806<br>2366807<br>2366808<br>2366809<br>2366810<br>2366811<br>2366812<br>2366813<br>2366814<br>2366815<br>2366816<br>2366817<br>2366818<br>2366819<br>2366820<br>2366821<br>2366822<br>2366823<br>2366824<br>2366825<br>2366826<br>2366827<br>2366828<br>2366829<br>2366830<br>2366831<br>2366832<br>2366833<br>2366834<br>2366835<br>2366836<br>2366837<br>2366838<br>2366839<br>2366840<br>2366841<br>2366842<br>2366843<br>2366844<br>2366845	full<br>full<br>full<br>full<br>extended<br>extended<br>extended<br>full<br>full<br>core<br>core<br>core<br>full<br>full<br>full<br>full<br>full<br>full<br>full<br>full<br>extended<br>extended<br>free<br>extended<br>extended<br>extended<br>extended<br>full<br>extended<br>extended<br>extended<br>extended<br>full<br>full<br>extended<br>extended<br>full<br>full<br>full<br>core<br>extended<br>extended<br>core<br>extended<br>extended<br>extended<br>extended	1	168872806	169124221	+	NM_022716<br>NM_006902<br>XM_930564<br>XM_942480<br>AK225968<br>AF086400<br>ENSESTT00000036085<br>ENSESTT00000036086<br>ENSESTT00000036087<br>ENST00000239461<br>ENST00000234744<br>ENST00000367761<br>ENST00000367760<br>GENSCAN00000065040<br>GENSCAN00000002994<br>GENSCAN00000002996<br>GENSCAN00000002995	Homo sapiens paired related homeobox 1 (PRRX1), transcript variant pmx-1b, mRNA.<br>Homo sapiens paired related homeobox 1 (PRRX1), transcript variant pmx-1a, mRNA.<br>PREDICTED: Homo sapiens hypothetical protein LOC646669 (LOC646669), mRNA.<br>PREDICTED: Homo sapiens hypothetical protein LOC650595 (LOC650595), mRNA.<br>Homo sapiens mRNA for paired mesoderm homeobox 1 isoform pmx-1b variant, clone: FCC113D08.<br>Homo sapiens full length insert cDNA clone ZD75H02.<br><br><br><br>cdna:known-ccds chromosome:NCBI36:1:168898947:168975184:1 gene:ENSG00000116132 CCDS1291.1<br>cdna:known-ccds chromosome:NCBI36:1:168899671:168972271:1 gene:ENSG00000116132 CCDS1290.1<br>cdna:known chromosome:NCBI36:1:168899937:168975165:1 gene:ENSG00000116132<br>cdna:known chromosome:NCBI36:1:168899937:168975165:1 gene:ENSG00000116132<br>cdna:Genscan chromosome:NCBI36:1:169043188:169124221:1<br>cdna:Genscan chromosome:NCBI36:1:168954621:169001810:1<br>cdna:Genscan chromosome:NCBI36:1:168919765:168936569:1<br>cdna:Genscan chromosome:NCBI36:1:168872806:168905014:1

	31532<br>31533<br>31534<br>31535<br>31536<br>31537<br>31538	2366846<br>2366847<br>2366848<br>2366849<br>2366850<br>2366851<br>2366852<br>2366853<br>2366854<br>2366855<br>2366856<br>2366857<br>2366858<br>2366859<br>2366860<br>2366861<br>2366862<br>2366863<br>2366864<br>2366865<br>2366866<br>2366867<br>2366868<br>2366869<br>2366870	core<br>extended<br>extended<br>core<br>core<br>core<br>extended<br>full<br>full<br>extended<br>extended<br>extended<br>full<br>full<br>full<br>full<br>full<br>full<br>full<br>full<br>full<br>full<br>extended<br>full<br>full						
2371139	34337<br>34338<br>34339<br>34340<br>34341<br>34342<br>34343<br>34344<br>34345<br>34346<br>34347<br>34348<br>34349<br>34350<br>34351<br>34352<br>34353<br>34354<br>34355<br>34356<br>34357<br>34358<br>34359<br>34360<br>34361<br>34362<br>34363<br>34364<br>34365	2371140<br>2371141<br>2371142<br>2371143<br>2371144<br>2371145<br>2371146<br>2371147<br>2371148<br>2371149<br>2371150<br>2371151<br>2371152<br>2371153<br>2371154<br>2371155<br>2371156<br>2371157<br>2371158<br>2371159<br>2371160<br>2371161<br>2371162<br>2371163<br>2371164<br>2371165<br>2371166<br>2371167<br>2371168	full<br>full<br>full<br>full<br>full<br>full<br>core<br>full<br>full<br>full<br>core<br>extended<br>core<br>core<br>extended<br>core<br>full<br>core<br>core<br>core<br>core<br>core<br>core<br>full<br>core<br>core<br>core<br>core<br>extended	1	181400862	181494723	+	NM_005562<br>NM_018891<br>ENSESTT00000017771<br>ENSESTT00000017772<br>ENSESTT00000017773<br>ENST00000264144<br>ENST00000367544<br>GENSCAN00000000951	Homo sapiens laminin, gamma 2 (LAMC2), transcript variant 1, mRNA.<br>Homo sapiens laminin, gamma 2 (LAMC2), transcript variant 2, mRNA.<br><br><br><br>cdna:known-ccds chromosome:NCBI36:1:181422022:181480662:1 gene:ENSG00000058085 CCDS1352.1<br>cdna:known chromosome:NCBI36:1:181422046:181480658:1 gene:ENSG00000058085<br>cdna:Genscan chromosome:NCBI36:1:181400862:181479158:1

	34366<br>34367<br>34368<br>34369<br>34370<br>34371<br>34372<br>34373<br>34374<br>34375<br>34376<br>34377	2371169<br>2371170<br>2371171<br>2371172<br>2371173<br>2371174<br>2371175<br>2371176<br>2371177<br>2371178<br>2371179<br>2371180<br>2371181<br>2371182<br>2371183<br>2371184<br>2371185<br>2371186<br>2371187<br>2371188<br>2371189<br>2371190<br>2371191<br>2371192	core<br>core<br>core<br>core<br>core<br>core<br>core<br>extended<br>core<br>core<br>core<br>extended<br>full<br>core<br>core<br>core<br>core<br>core<br>core<br>core<br>core<br>extended<br>extended<br>extended						
2375706	37286<br>37287<br>37288<br>37289<br>37290<br>37291<br>37292<br>37293<br>37294<br>37295<br>37296<br>37297<br>37298<br>37299<br>37300<br>37301<br>37302<br>37303<br>37304<br>37305<br>37306<br>37307<br>37308<br>37309<br>37310<br>37311<br>37312<br>37313<br>37314<br>37315	2375707<br>2375708<br>2375709<br>2375710<br>2375711<br>2375712<br>2375713<br>2375714<br>2375715<br>2375716<br>2375717<br>2375718<br>2375719<br>2375720<br>2375721<br>2375722<br>2375723<br>2375724<br>2375725<br>2375726<br>2375727<br>2375728<br>2375729<br>2375730<br>2375731<br>2375732<br>2375733<br>2375734<br>2375735<br>2375736	full<br>full<br>full<br>core<br>core<br>extended<br>extended<br>extended<br>extended<br>extended<br>extended<br>extended<br>extended<br>extended<br>extended<br>extended<br>core<br>core<br>full<br>core<br>core<br>extended<br>core<br>core<br>core<br>full<br>core<br>extended<br>core<br>core	1	201851193	201980086	+	NM_001684<br>NR_003019<br>NM_001001396<br>AK026443<br>BC062445<br>BX537745<br>ENSESTT00000019570<br>ENSESTT00000019571<br>ENSESTT00000019572<br>ENSESTT00000019573<br>ENSESTT00000019574<br>ENSESTT00000019575<br>ENSESTT00000019576<br>ENSESTT00000019577<br>ENSESTT00000019578<br>ENST00000357681<br>ENST00000241661<br>ENST00000361344<br>ENST00000356729<br>ENST00000367219<br>ENST00000367218<br>ENST00000341360<br>ENST00000367217<br>ENST00000367216<br>GENSCAN00000039986<br>GENSCAN00000060291<br>GENSCAN00000060289	Homo sapiens ATPase, Ca++ transporting, plasma membrane 4 (ATP2B4), transcript variant 2, mRNA.<br>Homo sapiens small nucleolar RNA, H/ACA box 77 (SNORA77) on chromosome 1.<br>Homo sapiens ATPase, Ca++ transporting, plasma membrane 4 (ATP2B4), transcript variant 1, mRNA.<br>Homo sapiens cDNA: FLJ22790 fis, clone KAIA2176, highly similar to HUMPMCA Human plasma membrane calcium-pumping ATPase (PMCA4) mRNA.<br>Homo sapiens ATPase, Ca++ transporting, plasma membrane 4, mRNA (cDNA clone IMAGE:30318583), partial cds.<br>Homo sapiens mRNA; cDNA DKFZp686E0762 (from clone DKFZp686E0762).<br>cdna:known-ccds chromosome:NCBI36:1:201862312:201979832:1 gene:ENSG00000058668 CCDS1440.1<br>cdna:known-ccds chromosome:NCBI36:1:202001004:202010630:1 gene:ENSG00000122188 CCDS1441.1<br>cdna:known chromosome:NCBI36:1:201862312:201979830:1 gene:ENSG00000058668<br>cdna:known chromosome:NCBI36:1:201862551:201979830:1 gene:ENSG00000058668<br>cdna:known chromosome:NCBI36:1:201862551:201979830:1 gene:ENSG00000058668<br>cdna:known chromosome:NCBI36:1:201862551:201979830:1 gene:ENSG00000058668<br>cdna:known chromosome:NCBI36:1:201918560:201976070:1 gene:ENSG00000058668<br>cdna:known chromosome:NCBI36:1:202000957:202010630:1 gene:ENSG00000122188<br>cdna:known chromosome:NCBI36:1:202001004:202010630:1 gene:ENSG00000122188<br>cdna:Genscan chromosome:NCBI36:1:201933908:202010432:1<br>cdna:Genscan chromosome:NCBI36:1:201851193:201856390:1<br>cdna:Genscan chromosome:NCBI36:1:201918957:201925371:1

	37316<br>37317<br>37318<br>37319<br>37320<br>37321<br>37322<br>37323<br>37324<br>37325<br>37326<br>37327<br>37328<br>37329<br>37330<br>37331<br>37332<br>37333	2375737<br>2375738<br>2375739<br>2375740<br>2375741<br>2375742<br>2375743<br>2375744<br>2375745<br>2375746<br>2375747<br>2375748<br>2375749<br>2375750<br>2375751<br>2375752<br>2375753<br>2375754<br>2375755<br>2375756<br>2375757<br>2375758<br>2375759<br>2375760<br>2375761<br>2375762<br>2375763<br>2375764<br>2375765<br>2375766<br>2375767<br>2375768<br>2375769<br>2375770<br>2375771<br>2375772<br>2375773<br>2375774<br>2375775<br>2375776<br>2375777<br>2375778<br>2375779<br>2375780<br>2375781	extended<br>core<br>core<br>core<br>core<br>core<br>extended<br>full<br>core<br>full<br>core<br>core<br>core<br>core<br>core<br>extended<br>extended<br>extended<br>extended<br>extended<br>extended<br>extended<br>extended<br>extended<br>extended<br>extended<br>extended<br>core<br>core<br>core<br>extended<br>extended<br>extended<br>extended<br>full<br>extended<br>core<br>core<br>core<br>core<br>core<br>core<br>core<br>core<br>full						
2376168	37558<br>37559<br>37560<br>37561<br>37562<br>37563<br>37564<br>37565<br>37566	2376169<br>2376170<br>2376171<br>2376172<br>2376173<br>2376174<br>2376175<br>2376176<br>2376177	extended<br>full<br>full<br>extended<br>extended<br>extended<br>extended<br>full<br>full	1	203063774	203258569	+	NM_015090<br>AB177861<br>AK090639<br>AK127424<br>AK128699<br>AK172728<br>BC040674<br>BX537841<br>BX641048	Homo sapiens neurofascin homolog (chicken) (NFASC), mRNA.<br>Homo sapiens mRNA for KIAA0756 splice variant 1.<br>Homo sapiens cDNA FLJ33320 fis, clone BNGH42007798, highly similar to Rattus norvegicus ankyrin binding cell adhesion molecule neurofascin mRNA.<br>Homo sapiens cDNA FLJ45516 fis, clone BRTHA2022968, moderately similar to NG-CAM related cell adhesion molecule precursor.<br>Homo sapiens cDNA FLJ46866 fis, clone UTERU3011837, moderately similar to NG-CAM related cell adhesion molecule precursor.<br>Homo sapiens cDNA PSEC0044 fis, clone: NT2RP1001007.

	37567	2376178	full					ENSESTT00000016402	Homo sapiens neurofascin, mRNA (cDNA clone IMAGE:4817018).
	37568	2376179	full					ENSESTT00000016403	Homo sapiens mRNA; cDNA DKFZp686E03196 (from clone DKFZp686E03196).
	37569	2376180	full					ENSESTT00000016404	Homo sapiens mRNA; cDNA DKFZp686J0597 (from clone DKFZp686J0597).
	37570	2376181	full					ENSESTT00000016405	
	37571	2376182	full					ENSESTT00000016406	
	37572	2376183	full					ENSESTT00000016407	
	37573	2376184	full					ENST00000367172	
	37574	2376185	extended					ENST00000367171	
	37575	2376186	extended					ENST00000367170	
	37576	2376187	core					ENST00000339876	cdna:known chromosome:NCBI36:1:203064405:203258587:1 gene:ENSG00000163531
	37577	2376188	core					ENST00000367169	cdna:known chromosome:NCBI36:1:203064405:203258587:1 gene:ENSG00000163531
	37578	2376189	full					ENST00000338515	cdna:known chromosome:NCBI36:1:203064405:203258587:1 gene:ENSG00000163531
	37579	2376190	full					ENST00000338586	cdna:known chromosome:NCBI36:1:203064405:203258587:1 gene:ENSG00000163531
	37580	2376191	full					ENST00000295776	cdna:known chromosome:NCBI36:1:203064405:203212895:1 gene:ENSG00000163531
	37581	2376192	full					ENST00000360049	cdna:known chromosome:NCBI36:1:203156423:203258572:1 gene:ENSG00000163531
	37582	2376193	core					ENST00000367173	cdna:known chromosome:NCBI36:1:203156423:203258572:1 gene:ENSG00000163531
	37583	2376194	core					GENSCAN00000038264	cdna:known chromosome:NCBI36:1:203156423:203258572:1 gene:ENSG00000163531
	37584	2376195	core					GENSCAN00000017937	cdna:known chromosome:NCBI36:1:203156423:203258572:1 gene:ENSG00000163531
	37585	2376196	extended						cdna:novel chromosome:NCBI36:1:203064402:203246653:1 gene:ENSG00000163531
	37586	2376197	full						cdna:Genscan chromosome:NCBI36:1:203064427:203126117:1
	37587	2376198	full						cdna:Genscan chromosome:NCBI36:1:203173804:203252290:1
	37588	2376199	full						
	37589	2376200	full						
	37590	2376201	full						
	37591	2376202	extended						
	37592	2376203	extended						
	37593	2376204	extended						
	37594	2376205	full						
	37595	2376206	full						
	37596	2376207	full						
	37597	2376208	extended						
	37598	2376209	core						
	37599	2376210	core						
	37600	2376211	full						
	37601	2376212	core						
	37602	2376213	core						
	37603	2376214	core						
	37604	2376215	full						
	37605	2376216	full						
	37606	2376217	core						
	37607	2376218	core						
	37608	2376219	full						
	37609	2376220	core						
	37610	2376221	full						
	37611	2376222	core						
	37612	2376223	core						
	37613	2376224	core						
	37614	2376225	full						
	37615	2376226	core						
	37616	2376227	core						
	37617	2376228	core						
	37618	2376229	core						
	37619	2376230	core						
	37620	2376231	core						

	37621	2376232	full						
	37622	2376233	core						
	37623	2376234	core						
	37624	2376235	core						
	37625	2376236	core						
	37626	2376237	core						
	37627	2376238	full						
	37628	2376239	full						
	37629	2376240	extended						
	37630	2376241	extended						
	37631	2376242	extended						
	37632	2376243	core						
	37633	2376244	extended						
	37634	2376245	extended						
	37635	2376246	extended						
		2376247	extended						
		2376248	extended						
		2376249	extended						
		2376250	core						
		2376251	core						
		2376252	extended						
		2376253	full						
		2376254	free						
		2376255	full						
		2376256	core						
		2376257	core						
		2376258	extended						
		2376259	extended						
		2376260	extended						
		2376261	extended						
		2376262	extended						
		2376263	extended						
		2376264	extended						
		2376265	extended						
		2376266	extended						
		2376267	extended						
		2376268	core						
		2376269	core						
		2376270	core						
		2376271	full						
		2376272	full						
		2376273	full						
		2376274	core						
		2376275	core						
		2376276	core						
		2376277	core						
		2376278	full						
		2376279	full						
		2376280	core						
		2376281	core						
		2376282	core						
		2376283	core						
		2376284	core						
2379863	39879	2379864	core	1	212843171	212988438	+	NM_016343	Homo sapiens centromere protein F, 350/400ka (mitosin) (CENPF), mRNA.

	39880	2379865	core					ENSESTT00000051499	
	39881	2379866	full					ENSESTT00000051500	
	39882	2379867	extended					ENSESTT00000051501	
	39883	2379868	extended					ENSESTT00000051502	
	39884	2379869	full					ENSESTT00000051503	
	39885	2379870	full					ENSESTT00000051504	
	39886	2379871	full					ENSESTT00000051505	
	39887	2379872	full					ENST00000271778	cdna:known chromosome:NCBI36:1:212843155:212904535:1 gene:ENSG00000117724
	39888	2379873	full					ENST00000366955	cdna:known chromosome:NCBI36:1:212843161:212904554:1 gene:ENSG00000117724
	39889	2379874	full					GENSCAN00000066314	cdna:Genscan chromosome:NCBI36:1:212988212:212988438:1
	39890	2379875	full					GENSCAN00000048419	cdna:Genscan chromosome:NCBI36:1:212892794:212903760:1
	39891	2379876	full						
	39892	2379877	full						
	39893	2379878	core						
	39894	2379879	core						
	39895	2379880	core						
	39896	2379881	extended						
	39897	2379882	core						
	39898	2379883	core						
	39899	2379884	full						
	39900	2379885	core						
	39901	2379886	core						
	39902	2379887	core						
	39903	2379888	core						
	39904	2379889	core						
	39905	2379890	core						
	39906	2379891	full						
	39907	2379892	full						
	39908	2379893	full						
	39909	2379894	full						
	39910	2379895	core						
	39911	2379896	core						
	39912	2379897	core						
	39913	2379898	core						
	39914	2379899	core						
	39915	2379900	core						
	39916	2379901	core						
	39917	2379902	core						
	39918	2379903	core						
	39919	2379904	core						
	39920	2379905	core						
		2379906	full						
		2379907	core						
		2379908	core						
		2379909	core						
		2379910	extended						
		2379911	extended						
		2379912	core						
		2379913	core						
		2379914	core						
		2379915	core						
		2379916	extended						
		2379917	full						
		2379918	core						

		2379919<br>2379920<br>2379921<br>2379922<br>2379923<br>2379924<br>2379925<br>2379926<br>2379927<br>2379928<br>2379929<br>2379930	core<br>core<br>free<br>extended<br>extended<br>full<br>full<br>full<br>full<br>full<br>full<br>full						
2398706	51223<br>51224<br>51225<br>51226<br>51227<br>51228<br>51229<br>51230<br>51231<br>51232<br>51233<br>51234<br>51235<br>51236	2398707<br>2398708<br>2398709<br>2398710<br>2398711<br>2398712<br>2398713<br>2398714<br>2398715<br>2398716<br>2398717<br>2398718<br>2398719<br>2398720<br>2398721<br>2398722<br>2398723<br>2398724<br>2398725<br>2398726<br>2398727<br>2398728<br>2398729<br>2398730<br>2398731	full<br>full<br>full<br>core<br>core<br>core<br>core<br>core<br>full<br>extended<br>core<br>core<br>extended<br>core<br>extended<br>core<br>extended<br>core<br>core<br>full<br>full<br>core<br>extended<br>extended<br>core	1	17173325	17180668	-	NM_022089<br>NM_017459<br>NM_002403<br>BC030267<br>BC028033<br>ENSESTT00000039935<br>ENSESTT00000039936<br>ENSESTT00000039937<br>ENSESTT00000039938<br>ENST00000375535<br>ENST00000326735<br>ENST00000375534<br>ENST00000235772<br>ENST00000341676<br>ENST00000375508<br>GENSCAN00000024160	Homo sapiens ATPase type 13A2 (ATP13A2), mRNA.<br>Homo sapiens microfibrillar-associated protein 2 (MFAP2), transcript variant 1, mRNA.<br>Homo sapiens microfibrillar-associated protein 2 (MFAP2), transcript variant 2, mRNA.<br>Homo sapiens ATPase type 13A2, mRNA (cDNA clone MGC:40082 IMAGE:5240813), complete cds.<br>Homo sapiens microfibrillar-associated protein 2, mRNA (cDNA clone IMAGE:5242233), with apparent retained intron.<br><br><br><br><br>cdna:known-ccds chromosome:NCBI36:1:17173584:17179917:-1 gene:ENSG00000117122 CCDS174.1<br>cdna:known-ccds chromosome:NCBI36:1:17185040:17210854:-1 gene:ENSG00000159363 CCDS175.1<br>cdna:known chromosome:NCBI36:1:17173584:17177358:-1 gene:ENSG00000117122<br>cdna:known chromosome:NCBI36:1:17173590:17179760:-1 gene:ENSG00000117122<br>cdna:known chromosome:NCBI36:1:17185040:17210997:-1 gene:ENSG00000159363<br>cdna:known chromosome:NCBI36:1:17194941:17199373:-1 gene:ENSG00000159363<br>cdna:Genscan chromosome:NCBI36:1:17173325:17210602:-1
2398820	51292<br>51293<br>51294<br>51295<br>51296<br>51297<br>51298<br>51299<br>51300<br>51301<br>51302<br>51303<br>51304<br>51305<br>51306<br>51307	2398821<br>2398822<br>2398823<br>2398824<br>2398825<br>2398826<br>2398827<br>2398828<br>2398829<br>2398830<br>2398831<br>2398832<br>2398833<br>2398834<br>2398835<br>2398836	extended<br>extended<br>core<br>core<br>core<br>extended<br>core<br>extended<br>core<br>full<br>core<br>core<br>full<br>core<br>extended<br>extended	1	17265844	17318517	-	NM_007365<br>AK123164<br>AK127006<br>BC009701<br>ENSESTT00000039930<br>ENST00000375486<br>ENST00000245530<br>ENST00000375481<br>GENSCAN00000024162	Homo sapiens peptidyl arginine deiminase, type II (PADI2), mRNA.<br>Homo sapiens cDNA FLJ41169 fis, clone BRACE2041200, highly similar to PROTEIN-ARGININE DEIMINASE TYPE II (EC 3.5.3.15).<br>Homo sapiens cDNA FLJ45061 fis, clone BRAWH3023421, highly similar to Protein-arginine deiminase type II (EC 3.5.3.15).<br>Homo sapiens peptidyl arginine deiminase, type II, mRNA (cDNA clone MGC:9250 IMAGE:3901790), complete cds.<br><br>cdna:known-ccds chromosome:NCBI36:1:17265843:17318517:-1 gene:ENSG00000117115 CCDS177.1<br>cdna:known chromosome:NCBI36:1:17265844:17318517:-1 gene:ENSG00000117115<br>cdna:known chromosome:NCBI36:1:17278113:17318517:-1 gene:ENSG00000117115<br>cdna:Genscan chromosome:NCBI36:1:17268126:17318453:-1

	51308<br>51309<br>51310<br>51311<br>51312<br>51313<br>51314<br>51315<br>51316<br>51317<br>51318<br>51319<br>51320	2398837<br>2398838<br>2398839<br>2398840<br>2398841<br>2398842<br>2398843<br>2398844<br>2398845<br>2398846<br>2398847<br>2398848<br>2398849<br>2398850<br>2398851<br>2398852<br>2398853<br>2398854<br>2398855<br>2398856<br>2398857<br>2398858	core<br>core<br>extended<br>extended<br>core<br>full<br>core<br>core<br>core<br>full<br>full<br>full<br>full<br>extended<br>extended<br>core<br>core<br>extended<br>full<br>full<br>core<br>core						
2409004	57521<br>57522<br>57523<br>57524<br>57525<br>57526<br>57527<br>57528<br>57529<br>57530<br>57531<br>57532<br>57533<br>57534<br>57535<br>57536<br>57537<br>57538<br>57539<br>57540<br>57541<br>57542<br>57543<br>57544<br>57545<br>57546<br>57547	2409005<br>2409006<br>2409007<br>2409008<br>2409009<br>2409010<br>2409011<br>2409012<br>2409013<br>2409014<br>2409015<br>2409016<br>2409017<br>2409018<br>2409019<br>2409020<br>2409021<br>2409022<br>2409023<br>2409024<br>2409025<br>2409026<br>2409027<br>2409028<br>2409029<br>2409030<br>2409031<br>2409032<br>2409033<br>2409034<br>2409035<br>2409036	extended<br>extended<br>extended<br>core<br>core<br>core<br>core<br>core<br>core<br>core<br>core<br>extended<br>core<br>core<br>extended<br>core<br>extended<br>extended<br>extended<br>extended<br>core<br>extended<br>extended<br>core<br>core<br>core<br>core<br>core<br>core<br>core<br>extended<br>core	1	42983942	43005315	-	NM_022356<br>AF097431<br>BC068461<br>CR616745<br>CR749690<br>ENSESTT00000045958<br>ENSESTT00000045959<br>ENSESTT00000045960<br>ENSESTT00000045961<br>ENSESTT00000045962<br>ENST00000236040<br>ENST00000296388<br>ENST00000372526<br>GENSCAN00000031484	Homo sapiens leucine proline-enriched proteoglycan (leprecan) 1 (LEPRE1), mRNA.<br>Homo sapiens GROS1-S protein mRNA, complete cds.<br>Homo sapiens leucine proline-enriched proteoglycan (leprecan) 1, mRNA (cDNA clone IMAGE:30342539), complete cds.<br>full-length cDNA clone CS0DK007YM05 of HeLa cells Cot 25-normalized of Homo sapiens (human).<br>Homo sapiens mRNA; cDNA DKFZp547C166 (from clone DKFZp547C166).<br><br><br><br><br><br>cdna:known-ccds chromosome:NCBI36:1:42984633:43005270:-1 gene:ENSG00000117385 CCDS472.1<br>cdna:known chromosome:NCBI36:1:42984633:43005281:-1 gene:ENSG00000117385<br>cdna:novel chromosome:NCBI36:1:43000088:43005275:-1 gene:ENSG00000117385<br>cdna:Genscan chromosome:NCBI36:1:42984955:43005229:-1

		2409037<br>2409038<br>2409039<br>2409040<br>2409041<br>2409042<br>2409043<br>2409044<br>2409045<br>2409046<br>2409047<br>2409048<br>2409049<br>2409050<br>2409051<br>2409052	extended<br>extended<br>core<br>core<br>core<br>core<br>core<br>core<br>extended<br>full<br>full<br>core<br>core<br>core<br>core<br>core						
2409104	57577<br>57578<br>57579<br>57580<br>57581<br>57582<br>57583<br>57584<br>57585<br>57586<br>57587<br>57588<br>57589<br>57590<br>57591<br>57592<br>57593<br>57594<br>57595<br>57596<br>57597<br>57598<br>57599<br>57600<br>57601<br>57602<br>57603<br>57604<br>57605<br>57606<br>57607<br>57608<br>57609<br>57610<br>57611<br>57612<br>57613<br>57614	2409105<br>2409106<br>2409107<br>2409108<br>2409109<br>2409110<br>2409111<br>2409112<br>2409113<br>2409114<br>2409115<br>2409116<br>2409117<br>2409118<br>2409119<br>2409120<br>2409121<br>2409122<br>2409123<br>2409124<br>2409125<br>2409126<br>2409127<br>2409128<br>2409129<br>2409130<br>2409131<br>2409132<br>2409133<br>2409134<br>2409135<br>2409136<br>2409137<br>2409138<br>2409139<br>2409140<br>2409141<br>2409142	full<br>full<br>extended<br>full<br>full<br>full<br>full<br>full<br>full<br>full<br>extended<br>core<br>core<br>core<br>core<br>core<br>extended<br>core<br>core<br>core<br>extended<br>full<br>core<br>core<br>extended<br>core<br>extended<br>extended<br>core<br>full<br>extended<br>full<br>extended<br>extended<br>core<br>full<br>extended<br>extended	1	43124794	43338329	-	NM_006516<br>XM_941326<br>XM_927705<br>AB208987<br>ENSESTT00000045954<br>ENSESTT00000045955<br>ENST00000372501<br>ENST00000270929<br>ENST00000372500<br>GENSCAN00000054874<br>GENSCAN00000047316<br>GENSCAN00000031170<br>GENSCAN00000007378	Homo sapiens solute carrier family 2 (facilitated glucose transporter), member 1 (SLC2A1), mRNA.<br>PREDICTED: Homo sapiens similar to EVIN1 (LOC652027), mRNA.<br>PREDICTED: Homo sapiens similar to EVIN1 (LOC644590), mRNA.<br>Homo sapiens mRNA for solute carrier family 2 (facilitated glucose transporter), member 1 variant protein.<br><br><br>cdna:known-ccds chromosome:NCBI36:1:43164106:43197088:-1 gene:ENSG00000117394 CCDS477.1<br>cdna:known chromosome:NCBI36:1:43164115:43197088:-1 gene:ENSG00000117394<br>cdna:novel chromosome:NCBI36:1:43168577:43197117:-1 gene:ENSG00000117394<br>cdna:Genscan supercontig::NT_113873:10431:21686:1<br>cdna:Genscan chromosome:NCBI36:1:43165299:43233077:-1<br>cdna:Genscan chromosome:NCBI36:1:43279531:43338329:-1<br>cdna:Genscan chromosome:NCBI36:1:43124794:43129769:-1

	57615<br>57616<br>57617<br>57618<br>57619<br>57620<br>57621<br>57622<br>57623<br>57624<br>57625<br>57626	2409143<br>2409144<br>2409145<br>2409146<br>2409147<br>2409148<br>2409149<br>2409150<br>2409151<br>2409152<br>2409153<br>2409154<br>2409155<br>2409156<br>2409157<br>2409158<br>2409159<br>2409160<br>2409161<br>2409162<br>2409163<br>2409164<br>2409165<br>2409166<br>2409167<br>2409168<br>2409169<br>2409170<br>2409171<br>2409172<br>2409173<br>2409174<br>2409175<br>2409176<br>2409177<br>2409178	extended<br>extended<br>extended<br>full<br>full<br>full<br>full<br>full<br>full<br>full<br>core<br>extended<br>full<br>full<br>full<br>full<br>full<br>full<br>full<br>full<br>full<br>full<br>full<br>full<br>full<br>full<br>full<br>full<br>full<br>full<br>full<br>full<br>full<br>full<br>full<br>full						
2409820	58019<br>58020<br>58021<br>58022<br>58023<br>58024<br>58025<br>58026<br>58027<br>58028	2409821<br>2409822<br>2409823<br>2409824<br>2409825<br>2409826<br>2409827<br>2409828<br>2409829<br>2409830<br>2409831<br>2409832<br>2409833<br>2409834<br>2409835<br>2409836	core<br>core<br>core<br>core<br>extended<br>full<br>core<br>core<br>core<br>core<br>core<br>core<br>full<br>extended<br>core<br>core	1	45021846	45025964	-	NM_153274<br>BC101823<br>ENST00000372207<br>ENST00000271137<br>GENSCAN00000034494	Homo sapiens vitelliform macular dystrophy 2-like 2 (VMD2L2), mRNA.<br>Homo sapiens vitelliform macular dystrophy 2-like 2, mRNA (cDNA clone MGC:126872 IMAGE:8069329), complete cds.<br>cdna:known-ccds chromosome:NCBI36:1:45021844:45025964:-1 gene:ENSG00000142959 CCDS514.1<br>cdna:known chromosome:NCBI36:1:45021846:45025964:-1 gene:ENSG00000142959<br>cdna:Genscan chromosome:NCBI36:1:45022469:45025964:-1
2411228	58794	2411229	core	1	47488403	47552432	-	NM_003035	Homo sapiens SCL/TAL1 interrupting locus (STIL), mRNA.

	58795<br>58796<br>58797<br>58798<br>58799<br>58800<br>58801<br>58802<br>58803<br>58804<br>58805<br>58806<br>58807<br>58808<br>58809<br>58810<br>58811<br>58812<br>58813<br>58814<br>58815	2411230<br>2411231<br>2411232<br>2411233<br>2411234<br>2411235<br>2411236<br>2411237<br>2411238<br>2411239<br>2411240<br>2411241<br>2411242<br>2411243<br>2411244<br>2411245<br>2411246<br>2411247<br>2411248<br>2411249<br>2411250<br>2411251<br>2411252<br>2411253<br>2411254<br>2411255<br>2411256<br>2411257<br>2411258<br>2411259<br>2411260<br>2411261<br>2411262<br>2411263<br>2411264<br>2411265<br>2411266<br>2411267<br>2411268<br>2411269<br>2411270<br>2411271	core<br>core<br>core<br>core<br>core<br>core<br>core<br>core<br>core<br>core<br>core<br>core<br>core<br>core<br>core<br>core<br>core<br>extended<br>core<br>core<br>extended<br>extended<br>core<br>core<br>extended<br>core<br>core<br>core<br>core<br>full<br>core<br>core<br>extended<br>core<br>extended<br>extended<br>core<br>core<br>core<br>core<br>extended<br>full					AK128406<br>ENSESTT00000005471<br>ENSESTT00000005472<br>ENSESTT00000005476<br>ENSESTT00000005478<br>ENSESTT00000005479<br>ENSESTT00000005480<br>ENST00000337817<br>ENST00000371877<br>ENST00000243182<br>ENST00000371874<br>ENST00000360380<br>GENSCAN00000054579<br>GENSCAN00000064736	Homo sapiens cDNA FLJ46549 fis, clone THYMU3038375, highly similar to Homo sapiens TAL1 (SCL) interrupting locus (SIL).<br><br><br><br><br><br><br><br>cdna:known-ccds chromosome:NCBI36:1:47488401:47552382:-1 gene:ENSG00000123473 CCDS548.1<br>cdna:known chromosome:NCBI36:1:47488401:47552382:-1 gene:ENSG00000123473<br>cdna:known chromosome:NCBI36:1:47488401:47552382:-1 gene:ENSG00000123473<br>cdna:known chromosome:NCBI36:1:47488401:47519315:-1 gene:ENSG00000123473<br>cdna:known chromosome:NCBI36:1:47488436:47552406:-1 gene:ENSG00000123473<br>cdna:Genscan chromosome:NCBI36:1:47489395:47510452:-1<br>cdna:Genscan chromosome:NCBI36:1:47518479:47548578:-1
2413203	60072<br>60073<br>60074<br>60075<br>60076<br>60077<br>60078<br>60079<br>60080<br>60081<br>60082<br>60083	2413204<br>2413205<br>2413206<br>2413207<br>2413208<br>2413209<br>2413210<br>2413211<br>2413212<br>2413213<br>2413214<br>2413215	free<br>free<br>extended<br>extended<br>extended<br>core<br>core<br>core<br>core<br>core<br>core<br>extended	1	53482138	53632680	-	NM_017522<br>NM_004631<br>NM_001018054<br>NM_033300<br>AK096482<br>AK122887<br>BC014162<br>ENSESTT00000008245<br>ENSESTT00000008246<br>ENSESTT00000008247<br>ENSESTT00000008248<br>ENSESTT00000008249	Homo sapiens low density lipoprotein receptor-related protein 8, apolipoprotein e receptor (LRP8), transcript variant 3, mRNA.<br>Homo sapiens low density lipoprotein receptor-related protein 8, apolipoprotein e receptor (LRP8), transcript variant 1, mRNA.<br>Homo sapiens low density lipoprotein receptor-related protein 8, apolipoprotein e receptor (LRP8), transcript variant 4, mRNA.<br>Homo sapiens low density lipoprotein receptor-related protein 8, apolipoprotein e receptor (LRP8), transcript variant 2, mRNA.<br>Homo sapiens cDNA FLJ39163 fis, clone OCBBF2002615, highly similar to Human mRNA for apolipoprotein E receptor 2.<br>Homo sapiens cDNA FLJ16536 fis, clone OCBBF2032152, highly similar to Homo sapiens low density lipoprotein receptor-related protein 8, apolipoprotein e receptor (LRP8).<br>Homo sapiens cDNA clone IMAGE:4547814, partial cds.

	60084	2413216	extended					ENST00000347547	
	60085	2413217	extended					ENST00000371456	cdna:known-ccds chromosome:NCBI36:1:53483800:53566409:-1 gene:ENSG00000157193 CCDS580.1
	60086	2413218	core					ENST00000354412	cdna:known-ccds chromosome:NCBI36:1:53483805:53566274:-1 gene:ENSG00000157193 CCDS578.1
	60087	2413219	core					ENST00000306052	cdna:known-ccds chromosome:NCBI36:1:53483806:53566314:-1 gene:ENSG00000157193 CCDS579.1
	60088	2413220	core					ENST00000371454	cdna:known chromosome:NCBI36:1:53483806:53566314:-1 gene:ENSG00000157193
	60089	2413221	core					ENST00000357488	cdna:known chromosome:NCBI36:1:53483806:53566314:-1 gene:ENSG00000157193
	60090	2413222	extended					ENST00000371452	cdna:known chromosome:NCBI36:1:53484813:53566314:-1 gene:ENSG00000157193
	60091	2413223	extended					GENSCAN00000034038	cdna:known chromosome:NCBI36:1:53484828:53566172:-1 gene:ENSG00000157193
	60092	2413224	core						cdna:Genscan chromosome:NCBI36:1:53485278:53502656:-1
	60093	2413225	core						
	60094	2413226	core						
	60095	2413227	full						
	60096	2413228	core						
	60097	2413229	core						
	60098	2413230	extended						
	60099	2413231	core						
	60100	2413232	core						
	60101	2413233	extended						
	60102	2413234	extended						
	60103	2413235	full						
	60104	2413236	full						
	60105	2413237	full						
	60106	2413238	extended						
	60107	2413239	full						
	60108	2413240	full						
	60109	2413241	core						
	60110	2413242	core						
	60111	2413243	full						
	60112	2413244	full						
	60113	2413245	full						
	60114	2413246	core						
	60115	2413247	extended						
	60116	2413248	extended						
	60117	2413249	extended						
	60118	2413250	extended						
	60119	2413251	extended						
	60120	2413252	extended						
	60121	2413253	core						
	60122	2413254	full						
	60123	2413255	full						
	60124	2413256	full						
	60125	2413257	extended						
	60126	2413258	extended						
	60127	2413259	extended						
	60128	2413260	full						
	60129	2413261	extended						
	60130	2413262	extended						
	60131	2413263	extended						
	60132	2413264	full						
	60133	2413265	extended						
	60134	2413266	extended						
		2413267	extended						
		2413268	full						
		2413269	full						

		2413270<br>2413271<br>2413272<br>2413273<br>2413274<br>2413275<br>2413276<br>2413277<br>2413278<br>2413279<br>2413280<br>2413281<br>2413282<br>2413283<br>2413284<br>2413285<br>2413286<br>2413287<br>2413288<br>2413289<br>2413290	extended<br>extended<br>full<br>extended<br>full<br>full<br>full<br>core<br>full<br>full<br>extended<br>extended<br>core<br>core<br>core<br>full<br>full<br>full<br>full<br>full<br>full						
2425756	68105<br>68106<br>68107<br>68108<br>68109<br>68110<br>68111<br>68112<br>68113<br>68114<br>68115<br>68116<br>68117<br>68118<br>68119<br>68120<br>68121<br>68122<br>68123<br>68124<br>68125<br>68126<br>68127<br>68128<br>68129<br>68130<br>68131<br>68132<br>68133<br>68134<br>68135<br>68136<br>68137	2425757<br>2425758<br>2425759<br>2425760<br>2425761<br>2425762<br>2425763<br>2425764<br>2425765<br>2425766<br>2425767<br>2425768<br>2425769<br>2425770<br>2425771<br>2425772<br>2425773<br>2425774<br>2425775<br>2425776<br>2425777<br>2425778<br>2425779<br>2425780<br>2425781<br>2425782<br>2425783<br>2425784<br>2425785<br>2425786<br>2425787<br>2425788<br>2425789	extended<br>extended<br>core<br>core<br>core<br>core<br>core<br>extended<br>core<br>core<br>core<br>core<br>extended<br>core<br>core<br>extended<br>core<br>core<br>core<br>extended<br>core<br>core<br>core<br>core<br>core<br>core<br>core<br>core<br>core<br>core<br>core<br>core<br>full	1	103114282	103347204	-	NM_001854<br>NM_080630<br>NM_080629<br>ENSESTT00000034465<br>ENSESTT00000034466<br>ENSESTT00000034467<br>ENSESTT00000034468<br>ENSESTT00000034469<br>ENSESTT00000034470<br>ENSESTT00000034471<br>ENST00000370096<br>ENST00000358392<br>ENST00000353414<br>ENST00000193186<br>ENST00000370090<br>GENSCAN00000063565<br>GENSCAN00000010468	Homo sapiens collagen, type XI, alpha 1 (COL11A1), transcript variant A, mRNA.<br>Homo sapiens collagen, type XI, alpha 1 (COL11A1), transcript variant C, mRNA.<br>Homo sapiens collagen, type XI, alpha 1 (COL11A1), transcript variant B, mRNA.<br><br><br><br><br><br><br><br>cdna:known-ccds chromosome:NCBI36:1:103114611:103346635:-1 gene:ENSG00000060718 CCDS778.1<br>cdna:known-ccds chromosome:NCBI36:1:103115583:103346640:-1 gene:ENSG00000060718 CCDS779.1<br>cdna:known-ccds chromosome:NCBI36:1:103115583:103346640:-1 gene:ENSG00000060718 CCDS780.1<br>cdna:known chromosome:NCBI36:1:103115583:103346640:-1 gene:ENSG00000060718<br>cdna:known chromosome:NCBI36:1:103115629:103269357:-1 gene:ENSG00000060718<br>cdna:Genscan chromosome:NCBI36:1:103116163:103161533:-1<br>cdna:Genscan chromosome:NCBI36:1:103172593:103234186:-1

	68138	2425790	core						
	68139	2425791	core						
	68140	2425792	core						
	68141	2425793	core						
	68142	2425794	extended						
	68143	2425795	core						
	68144	2425796	core						
	68145	2425797	core						
	68146	2425798	core						
	68147	2425799	core						
	68148	2425800	core						
	68149	2425801	core						
	68150	2425802	core						
	68151	2425803	core						
	68152	2425804	core						
	68153	2425805	core						
	68154	2425806	extended						
	68155	2425807	core						
	68156	2425808	core						
	68157	2425809	core						
	68158	2425810	core						
	68159	2425811	core						
	68160	2425812	core						
	68161	2425813	extended						
	68162	2425814	extended						
	68163	2425815	core						
	68164	2425816	core						
	68165	2425817	core						
	68166	2425818	core						
	68167	2425819	core						
	68168	2425820	core						
	68169	2425821	core						
	68170	2425822	core						
	68171	2425823	core						
	68172	2425824	core						
	68173	2425825	core						
	68174	2425826	core						
	68175	2425827	core						
	68176	2425828	core						
	68177	2425829	core						
	68178	2425830	core						
	68179	2425831	extended						
	68180	2425832	core						
	68181	2425833	core						
	68182	2425834	extended						
	68183	2425835	core						
	68184	2425836	core						
		2425837	core						
		2425838	extended						
		2425839	core						
		2425840	full						
		2425841	full						
		2425842	core						
		2425843	full						

		2425844<br>2425845<br>2425846<br>2425847<br>2425848<br>2425849<br>2425850<br>2425851	core<br>core<br>full<br>full<br>full<br>core<br>core<br>full						
2429556	70373<br>70374<br>70375<br>70376<br>70377<br>70378<br>70379<br>70380<br>70381<br>70382<br>70383<br>70384<br>70385<br>70386<br>70387<br>70388<br>70389<br>70390<br>70391<br>70392<br>70393<br>70394<br>70395<br>70396<br>70397<br>70398<br>70399<br>70400<br>70401<br>70402<br>70403<br>70404<br>70405<br>70406<br>70407<br>70408<br>70409<br>70410	2429557<br>2429558<br>2429559<br>2429560<br>2429561<br>2429562<br>2429563<br>2429564<br>2429565<br>2429566<br>2429567<br>2429568<br>2429569<br>2429570<br>2429571<br>2429572<br>2429573<br>2429574<br>2429575<br>2429576<br>2429577<br>2429578<br>2429579<br>2429580<br>2429581<br>2429582<br>2429583<br>2429584<br>2429585<br>2429586<br>2429587<br>2429588<br>2429589<br>2429590<br>2429591<br>2429592<br>2429593<br>2429594<br>2429595<br>2429596<br>2429597<br>2429598<br>2429599<br>2429600<br>2429601<br>2429602	full<br>full<br>full<br>extended<br>core<br>core<br>core<br>core<br>core<br>full<br>core<br>core<br>full<br>full<br>full<br>full<br>full<br>full<br>core<br>full<br>full<br>full<br>extended<br>core<br>core<br>full<br>extended<br>core<br>core<br>full<br>core<br>core<br>full<br>full<br>full<br>full<br>full<br>full<br>full<br>full<br>core<br>core<br>core<br>full<br>full<br>full	1	116039193	116178493	-	NM_001232<br>ENSESTT00000036803<br>ENSESTT00000036804<br>ENST00000261448<br>GENSCAN00000062879<br>GENSCAN00000042375	Homo sapiens calsequestrin 2 (cardiac muscle) (CASQ2), mRNA.<br><br><br>cdna:known-ccds chromosome:NCBI36:1:116044151:116112925:-1 gene:ENSG00000118729 CCDS884.1<br>cdna:Genscan chromosome:NCBI36:1:116110864:116134503:-1<br>cdna:Genscan chromosome:NCBI36:1:116045385:116077118:-1

		2429603<br>2429604<br>2429605<br>2429606	full<br>full<br>full<br>full						
2434716	73417<br>73418<br>73419<br>73420<br>73421<br>73422<br>73423<br>73424<br>73425<br>73426<br>73427<br>73428<br>73429<br>73430<br>73431<br>73432<br>73433<br>73434<br>73435<br>73436	2434717<br>2434718<br>2434719<br>2434720<br>2434721<br>2434722<br>2434723<br>2434724<br>2434725<br>2434726<br>2434727<br>2434728<br>2434729<br>2434730<br>2434731<br>2434732<br>2434733<br>2434734<br>2434735<br>2434736<br>2434737<br>2434738<br>2434739<br>2434740<br>2434741<br>2434742<br>2434743	extended<br>extended<br>extended<br>core<br>core<br>core<br>core<br>core<br>core<br>core<br>core<br>extended<br>core<br>core<br>extended<br>core<br>core<br>core<br>core<br>core<br>core<br>extended<br>full<br>extended<br>core<br>core<br>extended	1	149199708	149214083	-	NM_013384<br>NM_181746<br>NM_022075<br>AF189062<br>ENSESTT00000021268<br>ENSESTT00000021269<br>ENST00000368954<br>ENST00000361419<br>ENST00000271688<br>ENST00000368949<br>ENST00000345896<br>GENSCAN00000067571	Homo sapiens LAG1 longevity assurance homolog 2 (S. cerevisiae) (LASS2), transcript variant 3, mRNA.<br>Homo sapiens LAG1 longevity assurance homolog 2 (S. cerevisiae) (LASS2), transcript variant 1, mRNA.<br>Homo sapiens LAG1 longevity assurance homolog 2 (S. cerevisiae) (LASS2), transcript variant 2, mRNA.<br>Homo sapiens tumor metastasis-suppressor (TMSG1) mRNA, complete cds.<br><br><br>cdna:known-ccds chromosome:NCBI36:1:149204286:149214103:-1 gene:ENSG00000143418 CCDS973.1<br>cdna:known-ccds chromosome:NCBI36:1:149204287:149213943:-1 gene:ENSG00000143418 CCDS974.1<br>cdna:known chromosome:NCBI36:1:149204287:149213943:-1 gene:ENSG00000143418<br>cdna:known chromosome:NCBI36:1:149204287:149211007:-1 gene:ENSG00000143418<br>cdna:known chromosome:NCBI36:1:149204363:149213943:-1 gene:ENSG00000143418<br>cdna:Genscan chromosome:NCBI36:1:149205248:149216107:-1
2438282	75406<br>75407<br>75408<br>75409<br>75410<br>75411<br>75412<br>75413<br>75414<br>75415<br>75416<br>75417<br>75418<br>75419<br>75420<br>75421<br>75422<br>75423<br>75424<br>75425<br>75426<br>75427	2438283<br>2438284<br>2438285<br>2438286<br>2438287<br>2438288<br>2438289<br>2438290<br>2438291<br>2438292<br>2438293<br>2438294<br>2438295<br>2438296<br>2438297<br>2438298<br>2438299<br>2438300<br>2438301<br>2438302<br>2438303<br>2438304	core<br>core<br>core<br>core<br>core<br>core<br>core<br>core<br>core<br>core<br>core<br>core<br>core<br>full<br>full<br>core<br>extended<br>extended<br>core<br>core<br>extended<br>extended	1	154761821	154809020	-	NM_178229<br>ENSESTT00000058698<br>ENSESTT00000058699<br>ENSESTT00000058700<br>ENSESTT00000058701<br>ENST00000368238<br>ENST00000368239<br>ENST00000361170<br>ENST00000328017<br>GENSCAN00000010967	Homo sapiens IQ motif containing GTPase activating protein 3 (IQGAP3), mRNA.<br><br><br><br><br>cdna:known-ccds chromosome:NCBI36:1:154761821:154808956:-1 gene:ENSG00000183856 CCDS1144.1<br>cdna:known chromosome:NCBI36:1:154761821:154809020:-1 gene:ENSG00000183856<br>cdna:known chromosome:NCBI36:1:154761822:154809020:-1 gene:ENSG00000183856<br>cdna:novel chromosome:NCBI36:1:154761822:154809020:-1 gene:ENSG00000183856<br>cdna:Genscan chromosome:NCBI36:1:154762902:154806587:-1

	75428<br>75429<br>75430<br>75431<br>75432<br>75433<br>75434<br>75435<br>75436<br>75437<br>75438<br>75439<br>75440<br>75441<br>75442<br>75443<br>75444<br>75445	2438305<br>2438306<br>2438307<br>2438308<br>2438309<br>2438310<br>2438311<br>2438312<br>2438313<br>2438314<br>2438315<br>2438316<br>2438317<br>2438318<br>2438319<br>2438320<br>2438321<br>2438322<br>2438323<br>2438324<br>2438325<br>2438326<br>2438327<br>2438328<br>2438329<br>2438330<br>2438331<br>2438332<br>2438333<br>2438334<br>2438335<br>2438336<br>2438337<br>2438338<br>2438339	core<br>core<br>core<br>core<br>full<br>full<br>core<br>core<br>core<br>core<br>core<br>full<br>core<br>core<br>full<br>core<br>core<br>core<br>extended<br>core<br>core<br>core<br>core<br>core<br>core<br>core<br>full<br>full<br>core<br>core<br>core<br>core<br>core<br>core<br>core						
2438531	75533<br>75534<br>75535<br>75536<br>75537<br>75538<br>75539<br>75540<br>75541<br>75542<br>75543<br>75544<br>75545<br>75546<br>75547<br>75548<br>75549<br>75550<br>75551	2438532<br>2438533<br>2438534<br>2438535<br>2438536<br>2438537<br>2438538<br>2438539<br>2438540<br>2438541<br>2438542<br>2438543<br>2438544<br>2438545<br>2438546<br>2438547<br>2438548<br>2438549<br>2438550	core<br>core<br>core<br>core<br>core<br>core<br>core<br>extended<br>core<br>core<br>core<br>core<br>extended<br>extended<br>core<br>extended<br>core<br>full<br>full	1	154978532	155003331	-	NM_004494<br>AK096411<br>AL833592<br>ENSESTT00000058689<br>ENSESTT00000058690<br>ENSESTT00000058691<br>ENST00000357325<br>ENST00000368209<br>ENST00000305467<br>ENST00000368206<br>GENSCAN00000016274	Homo sapiens hepatoma-derived growth factor (high-mobility group protein 1-like) (HDGF), mRNA.<br>Homo sapiens cDNA FLJ39092 fis, clone NT2RP7019835, highly similar to HEPATOMA-DERIVED GROWTH FACTOR.<br>Homo sapiens mRNA; cDNA DKFZp686J1764 (from clone DKFZp686J1764).<br><br><br><br>cdna:known-ccds chromosome:NCBI36:1:154978523:154988160:-1 gene:ENSG00000143321 CCDS1156.1<br>cdna:known chromosome:NCBI36:1:154978526:154988649:-1 gene:ENSG00000143321<br>cdna:known chromosome:NCBI36:1:154978526:154988160:-1 gene:ENSG00000143321<br>cdna:known chromosome:NCBI36:1:154979761:154988864:-1 gene:ENSG00000143321<br>cdna:Genscan chromosome:NCBI36:1:154973814:154987845:-1

	75552	2438551<br>2438552<br>2438553<br>2438554<br>2438555<br>2438556<br>2438557<br>2438558<br>2438559<br>2438560<br>2438561<br>2438562<br>2438563	full<br>full<br>full<br>full<br>core<br>core<br>core<br>core<br>extended<br>extended<br>extended<br>extended<br>extended						
2440943	76901<br>76902<br>76903<br>76904<br>76905<br>76906<br>76907<br>76908<br>76909<br>76910<br>76911<br>76912<br>76913<br>76914<br>76915<br>76916<br>76917	2440944<br>2440945<br>2440946<br>2440947<br>2440948<br>2440949<br>2440950<br>2440951<br>2440952<br>2440953<br>2440954<br>2440955<br>2440956<br>2440957<br>2440958<br>2440959<br>2440960<br>2440961<br>2440962<br>2440963<br>2440964<br>2440965<br>2440966<br>2440967<br>2440968<br>2440969<br>2440970<br>2440971<br>2440972	full<br>extended<br>full<br>full<br>full<br>full<br>core<br>core<br>core<br>core<br>core<br>core<br>core<br>core<br>full<br>core<br>core<br>extended<br>core<br>core<br>full<br>core<br>core<br>core<br>full<br>full<br>extended<br>extended<br>full	1	159767032	159893765	-	XM_942097<br>NM_000569<br>BC017865<br>ENSESTT00000045029<br>ENSESTT00000045030<br>ENSESTT00000045031<br>ENST00000367969<br>ENST00000367968<br>ENST00000367967<br>GENSCAN00000000209	PREDICTED: Homo sapiens similar to Fc fragment of IgG, low affinity IIIa, receptor for (CD16) (LOC652578), mRNA.<br>Homo sapiens Fc fragment of IgG, low affinity IIIa, receptor (CD16a) (FCGR3A), mRNA.<br>Homo sapiens Fc fragment of IgG, low affinity IIIa, receptor (CD16a), mRNA (cDNA clone MGC:22630 IMAGE:4690249), complete cds.<br><br><br><br>cdna:known chromosome:NCBI36:1:159778173:159786442:-1 gene:ENSG00000203747<br>cdna:known chromosome:NCBI36:1:159778179:159786442:-1 gene:ENSG00000203747<br>cdna:known chromosome:NCBI36:1:159778210:159787005:-1 gene:ENSG00000203747<br>cdna:Genscan chromosome:NCBI36:1:159767032:159786150:-1
2443120	78246<br>78247<br>78248<br>78249<br>78250<br>78251<br>78252<br>78253<br>78254<br>78255<br>78256	2443121<br>2443122<br>2443123<br>2443124<br>2443125<br>2443126<br>2443127<br>2443128<br>2443129<br>2443130<br>2443131	full<br>full<br>full<br>full<br>full<br>full<br>full<br>full<br>full<br>full<br>full	1	166827378	166965116	-	NM_001937<br>Z22865<br>ENSESTT00000055262<br>ENST00000367817<br>ENST00000271418<br>GENSCAN00000052263<br>GENSCAN00000052264	Homo sapiens dermatopontin (DPT), mRNA.<br>H.sapiens dermatopontin mRNA, complete CDS.<br><br>cdna:known-ccds chromosome:NCBI36:1:166931321:166965126:-1 gene:ENSG00000143196 CCDS1275.1<br>cdna:known chromosome:NCBI36:1:166931331:166965052:-1 gene:ENSG00000143196<br>cdna:Genscan chromosome:NCBI36:1:166924151:166931665:-1<br>cdna:Genscan chromosome:NCBI36:1:166932411:166965036:-1

	78257<br>78258<br>78259<br>78260<br>78261<br>78262<br>78263<br>78264<br>78265<br>78266<br>78267<br>78268<br>78269<br>78270<br>78271<br>78272<br>78273<br>78274<br>78275<br>78276<br>78277<br>78278	2443132<br>2443133<br>2443134<br>2443135<br>2443136<br>2443137<br>2443138<br>2443139<br>2443140<br>2443141<br>2443142<br>2443143<br>2443144<br>2443145<br>2443146<br>2443147<br>2443148<br>2443149<br>2443150<br>2443151<br>2443152<br>2443153<br>2443154<br>2443155<br>2443156<br>2443157<br>2443158<br>2443159<br>2443160<br>2443161<br>2443162<br>2443163<br>2443164<br>2443165<br>2443166<br>2443167	full<br>full<br>full<br>full<br>full<br>full<br>full<br>full<br>full<br>full<br>core<br>core<br>core<br>core<br>core<br>core<br>core<br>core<br>core<br>extended<br>core<br>extended<br>full<br>core<br>full<br>full<br>full<br>full<br>full<br>full<br>full<br>full<br>core<br>core<br>core<br>extended						
2446567	80388<br>80389<br>80390<br>80391<br>80392<br>80393<br>80394<br>80395<br>80396<br>80397<br>80398<br>80399<br>80400<br>80401<br>80402<br>80403<br>80404<br>80405	2446568<br>2446569<br>2446570<br>2446571<br>2446572<br>2446573<br>2446574<br>2446575<br>2446576<br>2446577<br>2446578<br>2446579<br>2446580<br>2446581<br>2446582<br>2446583<br>2446584<br>2446585	extended<br>extended<br>extended<br>extended<br>extended<br>extended<br>full<br>full<br>full<br>full<br>full<br>full<br>extended<br>full<br>full<br>full<br>extended<br>full	1	179095658	179258656	-	NM_005819<br>AK056657<br>BC039118<br>ENST00000258301<br>ENST00000358073<br>ENST00000362024<br>GENSCAN00000036701<br>GENSCAN00000051836	Homo sapiens syntaxin 6 (STX6), mRNA.<br>Homo sapiens cDNA FLJ32095 fis, clone OCBBF2000998.<br>Homo sapiens syntaxin 6, mRNA (cDNA clone IMAGE:4831269), complete cds.<br>cdna:known-ccds chromosome:NCBI36:1:179208484:179258670:-1 gene:ENSG00000135823 CCDS1341.1<br>cdna:known chromosome:NCBI36:1:179179798:179212241:-1 gene:ENSG00000135823<br>cdna:known chromosome:NCBI36:1:179208801:179258669:-1 gene:ENSG00000135823<br>cdna:Genscan chromosome:NCBI36:1:179187749:179225868:-1<br>cdna:Genscan chromosome:NCBI36:1:179241012:179258432:-1

	80406<br>80407<br>80408<br>80409<br>80410<br>80411<br>80412<br>80413<br>80414<br>80415<br>80416<br>80417<br>80418<br>80419<br>80420<br>80421<br>80422<br>80423<br>80424<br>80425<br>80426<br>80427<br>80428<br>80429<br>80430<br>80431<br>80432<br>80433<br>80434<br>80435<br>80436<br>80437<br>80438<br>80439<br>80440	2446586<br>2446587<br>2446588<br>2446589<br>2446590<br>2446591<br>2446592<br>2446593<br>2446594<br>2446595<br>2446596<br>2446597<br>2446598<br>2446599<br>2446600<br>2446601<br>2446602<br>2446603<br>2446604<br>2446605<br>2446606<br>2446607<br>2446608<br>2446609<br>2446610<br>2446611<br>2446612<br>2446613<br>2446614<br>2446615<br>2446616<br>2446617<br>2446618<br>2446619<br>2446620<br>2446621<br>2446622<br>2446623<br>2446624<br>2446625<br>2446626<br>2446627<br>2446628<br>2446629<br>2446630<br>2446631<br>2446632<br>2446633<br>2446634<br>2446635	full<br>full<br>full<br>full<br>extended<br>extended<br>extended<br>extended<br>full<br>extended<br>extended<br>full<br>full<br>full<br>extended<br>extended<br>extended<br>extended<br>extended<br>extended<br>core<br>core<br>core<br>extended<br>core<br>extended<br>core<br>extended<br>core<br>full<br>full<br>full<br>full<br>core<br>core<br>full<br>extended<br>extended<br>core<br>core<br>full<br>extended<br>extended<br>full<br>full<br>extended<br>extended<br>full<br>core<br>extended						
2447877	81168<br>81169<br>81170<br>81171	2447878<br>2447879<br>2447880<br>2447881	extended<br>extended<br>extended<br>extended	1	183017539	183210605	-	NM_052966<br>AK095547<br>AK125496<br>ENSESTT00000002188	Homo sapiens chromosome 1 open reading frame 24 (C1orf24), transcript variant 2, mRNA.<br>Homo sapiens cDNA FLJ38228 fis, clone FCBBF2004217, highly similar to Homo sapiens NIBAN mRNA.<br>Homo sapiens cDNA FLJ43507 fis, clone PERIC2001227.

	81172	2447882	full					ENSESTT00000002189	
	81173	2447883	extended					ENST00000367511	cdna:known-ccds chromosome:NCBI36:1:183026787:183210305:-1 gene:ENSG00000135842 CCDS1364.1
	81174	2447884	core					ENST00000308303	cdna:known chromosome:NCBI36:1:183026791:183210305:-1 gene:ENSG00000135842
	81175	2447885	core					GENSCAN00000003823	cdna:Genscan chromosome:NCBI36:1:183030734:183043998:-1
	81176	2447886	core					GENSCAN00000053823	cdna:Genscan chromosome:NCBI36:1:183118511:183171992:-1
	81177	2447887	core						
	81178	2447888	core						
	81179	2447889	core						
	81180	2447890	core						
	81181	2447891	extended						
	81182	2447892	full						
	81183	2447893	full						
	81184	2447894	core						
	81185	2447895	core						
	81186	2447896	extended						
	81187	2447897	core						
	81188	2447898	core						
	81189	2447899	extended						
	81190	2447900	extended						
	81191	2447901	core						
	81192	2447902	extended						
	81193	2447903	core						
	81194	2447904	extended						
	81195	2447905	core						
	81196	2447906	full						
	81197	2447907	full						
	81198	2447908	full						
	81199	2447909	extended						
	81200	2447910	extended						
	81201	2447911	extended						
	81202	2447912	extended						
	81203	2447913	core						
	81204	2447914	full						
	81205	2447915	full						
	81206	2447916	extended						
	81207	2447917	full						
	81208	2447918	extended						
	81209	2447919	full						
	81210	2447920	extended						
	81211	2447921	extended						
	81212	2447922	extended						
	81213	2447923	full						
	81214	2447924	full						
	81215	2447925	full						
	81216	2447926	full						
	81217	2447927	core						
	81218	2447928	extended						
	81219	2447929	extended						
	81220	2447930	core						
	81221	2447931	extended						
	81222	2447932	core						
	81223	2447933	extended						
	81224	2447934	core						
	81225	2447935	full						

	81226<br>81227<br>81228<br>81229<br>81230<br>81231<br>81232<br>81233<br>81234<br>81235<br>81236<br>81237	2447936<br>2447937<br>2447938<br>2447939<br>2447940<br>2447941<br>2447942<br>2447943<br>2447944<br>2447945<br>2447946<br>2447947<br>2447948<br>2447949<br>2447950<br>2447951<br>2447952<br>2447953<br>2447954<br>2447955<br>2447956<br>2447957<br>2447958<br>2447959<br>2447960<br>2447961<br>2447962<br>2447963	extended<br>full<br>full<br>extended<br>extended<br>extended<br>extended<br>full<br>extended<br>full<br>extended<br>extended<br>extended<br>extended<br>extended<br>extended<br>extended<br>extended<br>extended<br>full<br>full<br>full<br>core<br>core<br>core<br>core<br>extended<br>extended						
2449559	82179<br>82180<br>82181<br>82182<br>82183<br>82184<br>82185<br>82186<br>82187<br>82188<br>82189<br>82190<br>82191<br>82192<br>82193<br>82194<br>82195<br>82196<br>82197<br>82198<br>82199<br>82200<br>82201<br>82202<br>82203<br>82204	2449560<br>2449561<br>2449562<br>2449563<br>2449564<br>2449565<br>2449566<br>2449567<br>2449568<br>2449569<br>2449570<br>2449571<br>2449572<br>2449573<br>2449574<br>2449575<br>2449576<br>2449577<br>2449578<br>2449579<br>2449580<br>2449581<br>2449582<br>2449583<br>2449584<br>2449585	core<br>core<br>core<br>core<br>core<br>extended<br>core<br>core<br>core<br>core<br>core<br>core<br>core<br>core<br>core<br>extended<br>core<br>extended<br>core<br>core<br>core<br>core<br>core<br>core<br>core<br>core	1	195319885	195382654	-	NM_018136<br>NM_001994<br>AK226178<br>AY971957<br>BC034607<br>BX648804<br>ENSESTT00000000458<br>ENSESTT00000026540<br>ENSESTT00000026541<br>ENSESTT00000026543<br>ENST00000367412<br>ENST00000367409<br>ENST00000271509<br>ENST00000367408<br>ENST00000318429<br>ENST00000294732<br>ENST00000367406<br>ENST00000367407<br>GENSCAN00000017299	Homo sapiens asp (abnormal spindle)-like, microcephaly associated (Drosophila) (ASPM), mRNA.<br>Homo sapiens coagulation factor XIII, B polypeptide (F13B), mRNA.<br>Homo sapiens mRNA for Splice isoform 2 of Q8IZT6 variant, clone: eh00569.<br>Homo sapiens abnormal spindle-like microcephaly associated splice variant 2 (ASPM) mRNA, complete cds, alternatively spliced.<br>Homo sapiens asp (abnormal spindle)-like, microcephaly associated (Drosophila), mRNA (cDNA clone IMAGE:4419772), complete cds.<br>Homo sapiens mRNA; cDNA DKFZp686N06184 (from clone DKFZp686N06184).<br><br><br><br><br><br>cdna:known-ccds chromosome:NCBI36:1:195274944:195303020:-1 gene:ENSG00000143278 CCDS1388.1<br>cdna:known-ccds chromosome:NCBI36:1:195319881:195382447:-1 gene:ENSG00000066279 CCDS1389.1<br>cdna:known chromosome:NCBI36:1:195274944:195302989:-1 gene:ENSG00000143278<br>cdna:known chromosome:NCBI36:1:195319885:195370897:-1 gene:ENSG00000066279<br>cdna:known chromosome:NCBI36:1:195320077:195382190:-1 gene:ENSG00000066279<br>cdna:known chromosome:NCBI36:1:195320077:195382190:-1 gene:ENSG00000066279<br>cdna:known chromosome:NCBI36:1:195323869:195382190:-1 gene:ENSG00000066279<br>cdna:novel chromosome:NCBI36:1:195320077:195382190:-1 gene:ENSG00000066279<br>cdna:Genscan chromosome:NCBI36:1:195254282:195382065:-1

	82205<br>82206<br>82207<br>82208<br>82209<br>82210<br>82211<br>82212	2449586<br>2449587<br>2449588<br>2449589<br>2449590<br>2449591<br>2449592<br>2449593<br>2449594<br>2449595<br>2449596<br>2449597<br>2449598<br>2449599<br>2449600<br>2449601<br>2449602<br>2449603<br>2449604<br>2449605<br>2449606<br>2449607<br>2449608<br>2449609<br>2449610<br>2449611<br>2449612<br>2449613<br>2449614<br>2449615<br>2449616<br>2449617<br>2449618	extended<br>core<br>core<br>core<br>core<br>core<br>core<br>extended<br>core<br>core<br>core<br>core<br>core<br>core<br>core<br>core<br>core<br>core<br>extended<br>full<br>full<br>core<br>extended<br>core<br>core<br>core<br>core<br>core<br>core<br>extended<br>full<br>full<br>full							
2450345	82639<br>82640<br>82641<br>82642<br>82643<br>82644<br>82645<br>82646<br>82647<br>82648<br>82649<br>82650<br>82651<br>82652<br>82653<br>82654<br>82655<br>82656<br>82657<br>82658<br>82659	2450346<br>2450347<br>2450348<br>2450349<br>2450350<br>2450351<br>2450352<br>2450353<br>2450354<br>2450355<br>2450356<br>2450357<br>2450358<br>2450359<br>2450360<br>2450361<br>2450362<br>2450363<br>2450364<br>2450365<br>2450366	full<br>extended<br>full<br>full<br>full<br>full<br>full<br>full<br>full<br>full<br>extended<br>full<br>full<br>full<br>full<br>full<br>extended<br>core<br>core<br>core<br>core	1	198699087	198856743	-	NM_014875<br>BC098582<br>ENSESTT00000045609<br>ENSESTT00000045610<br>ENSESTT00000045611<br>ENST00000367350<br>ENST00000236917<br>GENSCAN00000032460<br>GENSCAN00000051257	Homo sapiens kinesin family member 14 (KIF14), mRNA.<br>Homo sapiens kinesin family member 14, mRNA (cDNA clone IMAGE:6470912), partial cds.<br><br><br><br>cdna:known chromosome:NCBI36:1:198787251:198856485:-1 gene:ENSG00000118193<br>cdna:novel chromosome:NCBI36:1:198787248:198854474:-1 gene:ENSG00000118193<br>cdna:Genscan chromosome:NCBI36:1:198746100:198762905:-1<br>cdna:Genscan chromosome:NCBI36:1:198789139:198902491:-1

	82660<br>82661<br>82662<br>82663<br>82664<br>82665<br>82666<br>82667<br>82668<br>82669<br>82670<br>82671<br>82672<br>82673<br>82674<br>82675<br>82676<br>82677<br>82678<br>82679<br>82680<br>82681<br>82682<br>82683<br>82684<br>82685<br>82686<br>82687<br>82688	2450367<br>2450368<br>2450369<br>2450370<br>2450371<br>2450372<br>2450373<br>2450374<br>2450375<br>2450376<br>2450377<br>2450378<br>2450379<br>2450380<br>2450381<br>2450382<br>2450383<br>2450384<br>2450385<br>2450386<br>2450387<br>2450388<br>2450389<br>2450390<br>2450391<br>2450392<br>2450393<br>2450394<br>2450395<br>2450396<br>2450397<br>2450398<br>2450399<br>2450400<br>2450401<br>2450402<br>2450403<br>2450404<br>2450405<br>2450406<br>2450407<br>2450408<br>2450409<br>2450410<br>2450411<br>2450412<br>2450413<br>2450414<br>2450415	core<br>core<br>core<br>core<br>extended<br>full<br>core<br>core<br>core<br>core<br>core<br>core<br>core<br>core<br>core<br>core<br>core<br>core<br>core<br>core<br>core<br>full<br>core<br>core<br>core<br>full<br>core<br>core<br>core<br>core<br>core<br>core<br>core<br>core<br>core<br>core<br>core<br>core<br>core<br>core<br>core<br>core<br>core<br>core<br>core<br>core<br>core<br>full<br>full						
2451593	83393<br>83394<br>83395<br>83396<br>83397	2451594<br>2451595<br>2451596<br>2451597<br>2451598	core<br>core<br>core<br>core<br>core	1	201414553	201422500	-	NM_001276<br>NM_004997<br>AB209459<br>BC039132<br>ENSESTT00000019594	Homo sapiens chitinase 3-like 1 (cartilage glycoprotein-39) (CHI3L1), mRNA.<br>Homo sapiens myosin binding protein H (MYBPH), mRNA.<br>Homo sapiens mRNA for chitinase 3-like 1 variant protein.<br>Homo sapiens chitinase 3-like 1 (cartilage glycoprotein-39), mRNA (cDNA clone MGC:17246 IMAGE:4183798), complete cds.

	83398<br>83399<br>83400<br>83401<br>83402<br>83403	2451599<br>2451600<br>2451601<br>2451602<br>2451603<br>2451604<br>2451605<br>2451606<br>2451607<br>2451608<br>2451609<br>2451610<br>2451611<br>2451612<br>2451613<br>2451614<br>2451615	extended<br>core<br>extended<br>core<br>extended<br>core<br>extended<br>core<br>core<br>extended<br>core<br>core<br>full<br>full<br>core<br>core<br>core					ENST00000255409<br>ENST00000255416<br>ENST00000367234<br>ENST00000367232<br>GENSCAN00000038022	cdna:known-ccds chromosome:NCBI36:1:201414553:201422500:-1 gene:ENSG00000133048 CCDS1435.1<br>cdna:known chromosome:NCBI36:1:201403562:201411565:-1 gene:ENSG00000133055<br>cdna:known chromosome:NCBI36:1:201403562:201411564:-1 gene:ENSG00000133055<br>cdna:known chromosome:NCBI36:1:201414682:201422500:-1 gene:ENSG00000133048<br>cdna:Genscan chromosome:NCBI36:1:201403828:201421669:-1
2458742	87855<br>87856<br>87857<br>87858<br>87859<br>87860<br>87861<br>87862<br>87863<br>87864<br>87865<br>87866<br>87867<br>87868<br>87869<br>87870<br>87871<br>87872<br>87873<br>87874<br>87875<br>87876<br>87877<br>87878<br>87879	2458743<br>2458744<br>2458745<br>2458746<br>2458747<br>2458748<br>2458749<br>2458750<br>2458751<br>2458752<br>2458753<br>2458754<br>2458755<br>2458756<br>2458757<br>2458758<br>2458759<br>2458760<br>2458761<br>2458762<br>2458763<br>2458764<br>2458765<br>2458766<br>2458767<br>2458768<br>2458769<br>2458770<br>2458771<br>2458772	core<br>core<br>core<br>full<br>core<br>core<br>core<br>extended<br>extended<br>extended<br>core<br>core<br>core<br>core<br>core<br>core<br>core<br>core<br>extended<br>core<br>core<br>core<br>extended<br>extended<br>extended<br>extended<br>core<br>core<br>extended<br>full	1	224485484	224564128	-	NM_001618<br>NM_173083<br>BC065302<br>ENSESTT00000027276<br>ENSESTT00000027286<br>ENSESTT00000027289<br>ENST00000328205<br>ENST00000366794<br>ENST00000366808<br>ENST00000366807<br>ENST00000366804<br>ENST00000366803<br>ENST00000366801<br>ENST00000359525<br>ENST00000272146<br>GENSCAN00000066338<br>GENSCAN00000013814	Homo sapiens poly (ADP-ribose) polymerase family, member 1 (PARP1), mRNA.<br>Homo sapiens lin-9 homolog (C. elegans) (LIN9), mRNA.<br>Homo sapiens lin-9 homolog (C. elegans), mRNA (cDNA clone IMAGE:6156654), partial cds.<br><br><br><br>cdna:known-ccds chromosome:NCBI36:1:224485488:224563821:-1 gene:ENSG00000183814 CCDS1553.1<br>cdna:known-ccds chromosome:NCBI36:1:224615015:224662397:-1 gene:ENSG00000143799 CCDS1554.1<br>cdna:known chromosome:NCBI36:1:224485481:224564193:-1 gene:ENSG00000183814<br>cdna:known chromosome:NCBI36:1:224485481:224564057:-1 gene:ENSG00000183814<br>cdna:known chromosome:NCBI36:1:224485482:224564057:-1 gene:ENSG00000183814<br>cdna:known chromosome:NCBI36:1:224485488:224563913:-1 gene:ENSG00000183814<br>cdna:known chromosome:NCBI36:1:224485488:224563821:-1 gene:ENSG00000183814<br>cdna:known chromosome:NCBI36:1:224505161:224563913:-1 gene:ENSG00000183814<br>cdna:known chromosome:NCBI36:1:224615129:224662414:-1 gene:ENSG00000143799<br>cdna:Genscan chromosome:NCBI36:1:224555497:224637507:-1<br>cdna:Genscan chromosome:NCBI36:1:224486825:224550270:-1
2461037	89274<br>89275<br>89276<br>89277<br>89278<br>89279	2461038<br>2461039<br>2461040<br>2461041<br>2461042<br>2461043	extended<br>extended<br>core<br>core<br>core<br>core	1	231185398	231498109	-	NM_024938<br>XM_015717<br>NM_014801<br>XM_496441<br>NM_001011<br>XM_941967	Homo sapiens pecanex-like 2 (Drosophila) (PCNXL2), transcript variant 2, mRNA.<br>PREDICTED: Homo sapiens similar to 40S ribosomal protein S7 (S8) (LOC149224), mRNA.<br>Homo sapiens pecanex-like 2 (Drosophila) (PCNXL2), transcript variant 1, mRNA.<br>PREDICTED: Homo sapiens similar to 40S ribosomal protein S7 (S8) (LOC440732), mRNA.<br>Homo sapiens ribosomal protein S7 (RPS7), mRNA.<br>PREDICTED: Homo sapiens similar to 40S ribosomal protein S7 (S8) (LOC149224), mRNA.

	89280	2461044	core						AB007895	Homo sapiens KIAA0435 mRNA, partial cds.
	89281	2461045	core						AB055774	Homo sapiens mRNA for ribosomal protein S7, partial cds.
	89282	2461046	core						AK021445	Homo sapiens cDNA FLJ11383 fis, clone HEMBA1000518, weakly similar to PECANEX PROTEIN.
	89283	2461047	core						AK055374	Homo sapiens cDNA FLJ30812 fis, clone FEBRA2001492, moderately similar to Mus musculus pecanex 1 mRNA.
	89284	2461048	core						AK092353	Homo sapiens cDNA FLJ35034 fis, clone OCBBF2016591, moderately similar to Mus musculus pecanex 1 mRNA.
	89285	2461049	full						AK097229	Homo sapiens cDNA FLJ39910 fis, clone SPLEN2017981, moderately similar to Mus musculus pecanex 1 mRNA.
	89286	2461050	full						ENSESTT00000028544	
	89287	2461051	full						ENSESTT00000036462	
	89288	2461052	full						ENSESTT00000036463	
	89289	2461053	full						ENSESTT00000054810	
	89290	2461054	full						ENSESTT00000054811	
	89291	2461055	full						ENSESTT00000054812	
	89292	2461056	full						ENSESTT00000054813	
	89293	2461057	core						ENSESTT00000054814	
	89294	2461058	core						ENSESTT00000054815	
	89295	2461059	extended						ENSESTT00000054816	
	89296	2461060	core						ENSESTT00000054817	
	89297	2461061	core						ENSESTT00000054818	
	89298	2461062	core						ENSESTT00000054819	
	89299	2461063	core						ENSESTT00000054820	
	89300	2461064	full						ENST00000304921	cdna:known-ccds chromosome:NCBI36:2:3600728:3606383:1 gene:ENSG00000171863 CCDS1648.1
	89301	2461065	full						ENST00000366625	cdna:known chromosome:NCBI36:1:231186505:231342142:-1 gene:ENSG00000135749
	89302	2461066	full						ENST00000344698	cdna:known chromosome:NCBI36:1:231186505:231260887:-1 gene:ENSG00000135749
	89303	2461067	full						ENST00000258229	cdna:known chromosome:NCBI36:1:231337381:231461715:-1 gene:ENSG00000135749
	89304	2461068	full						ENST00000324142	cdna:known chromosome:NCBI36:1:231403861:231455056:-1 gene:ENSG00000135749
	89305	2461069	full						ENST00000312534	cdna:novel chromosome:NCBI36:13:21100552:21101154:-1 gene:ENSG00000174121
	89306	2461070	full						ENST00000294604	cdna:novel chromosome:NCBI36:1:68480745:68481326:-1 gene:ENSG00000162596
	89307	2461071	core						ENST00000238199	cdna:novel chromosome:NCBI36:1:231403861:231455058:-1 gene:ENSG00000135749
	89308	2461072	full						ENST00000328626	cdna:novel chromosome:NCBI36:17:23818941:23819587:1 gene:ENSG00000183405
	89309	2461073	core						GENSCAN00000023786	cdna:Genscan chromosome:NCBI36:2:3587810:3606347:1
	89310	2461074	full						GENSCAN00000031381	cdna:Genscan chromosome:NCBI36:4:4559708:4560195:1
	89311	2461075	full						GENSCAN00000040139	cdna:Genscan chromosome:NCBI36:13:21100552:21101154:-1
	89312	2461076	full						GENSCAN00000011317	cdna:Genscan chromosome:NCBI36:1:231186673:231219523:-1
	89313	2461077	full						GENSCAN00000000039	cdna:Genscan chromosome:NCBI36:1:231224734:231259691:-1
	89314	2461078	full						GENSCAN00000056573	cdna:Genscan chromosome:NCBI36:1:231354273:231383247:-1
	89315	2461079	full						GENSCAN00000056571	cdna:Genscan chromosome:NCBI36:1:231421313:231468970:-1
	89316	2461080	core						GENSCAN00000065553	cdna:Genscan chromosome:NCBI36:1:231480652:231489266:-1
	89317	2461081	core						GENSCAN00000018384	cdna:Genscan chromosome:NCBI36:1:68480745:68481326:-1
	89318	2461082	full						GENSCAN00000009367	cdna:Genscan chromosome:NCBI36:17:42154132:42154683:-1
	89319	2461083	full							
	89320	2461084	full							
	89321	2461085	full							
	89322	2461086	full							
	89323	2461087	full							
	89324	2461088	extended							
	89325	2461089	full							
	89326	2461090	full							
	89327	2461091	full							
	89328	2461092	core							
	89329	2461093	core							
	89330	2461094	core							
	89331	2461095	core							
	89332	2461096	extended							
	89333	2461097	extended							

	89334	2461098	extended						
	89335	2461099	extended						
	89336	2461100	core						
	89337	2461101	full						
	89338	2461102	full						
	89339	2461103	full						
	89340	2461104	extended						
	89341	2461105	extended						
	89342	2461106	extended						
	89343	2461107	extended						
	89344	2461108	extended						
	89345	2461109	extended						
	89346	2461110	extended						
	89347	2461111	extended						
	89348	2461112	extended						
	89349	2461113	core						
	89350	2461114	full						
	89351	2461115	core						
	89352	2461116	full						
	89353	2461117	extended						
	89354	2461118	extended						
	89355	2461119	full						
	89356	2461120	extended						
	89357	2461121	extended						
	89358	2461122	extended						
	89359	2461123	extended						
	89360	2461124	core						
	89361	2461125	full						
	89362	2461126	core						
	89363	2461127	full						
	89364	2461128	full						
	89365	2461129	extended						
	89366	2461130	full						
	89367	2461131	full						
	89368	2461132	core						
	89369	2461133	full						
	89370	2461134	core						
	89371	2461135	extended						
	89372	2461136	extended						
	89373	2461137	full						
	89374	2461138	extended						
	89375	2461139	extended						
	89376	2461140	core						
	89377	2461141	core						
	89378	2461142	extended						
	89379	2461143	extended						
	89380	2461144	extended						
	89381	2461145	extended						
	89382	2461146	extended						
	89383	2461147	full						
	89384	2461148	extended						
	89385	2461149	core						
	89386	2461150	core						
	89387	2461151	extended						

	89388	2461152	extended						
	89389	2461153	core						
	89390	2461154	extended						
	89391	2461155	extended						
	89392	2461156	core						
	89393	2461157	extended						
	89394	2461158	core						
	89395	2461159	core						
	89396	2461160	extended						
	89397	2461161	full						
	89398	2461162	extended						
		2461163	extended						
		2461164	extended						
		2461165	extended						
		2461166	full						
		2461167	full						
		2461168	full						
		2461169	full						
		2461170	full						
		2461171	full						
		2461172	core						
		2461173	full						
		2461174	full						
		2461175	full						
		2461176	extended						
		2461177	full						
		2461178	full						
		2461179	full						
		2461180	full						
		2461181	core						
		2461182	core						
		2461183	core						
		2461184	extended						
		2461185	core						
		2461186	extended						
		2461187	extended						
		2461188	extended						
		2461189	core						
		2461190	core						
		2461191	core						
		2461192	core						
		2461193	core						
		2461194	core						
		2461195	core						
		2461196	core						
		2461197	extended						
		2461198	extended						
		2461199	extended						
		2461200	extended						
		2461201	extended						
		2461202	full						
		2461203	full						
		2461204	full						
		2461205	extended						

		2461206<br>2461207<br>2461208<br>2461209<br>2461210<br>2461211	extended<br>full<br>full<br>extended<br>extended<br>extended						
2461473	89532<br>89533<br>89534<br>89535<br>89536<br>89537<br>89538<br>89539<br>89540<br>89541<br>89542<br>89543<br>89544<br>89545<br>89546<br>89547<br>89548<br>89549<br>89550<br>89551<br>89552<br>89553<br>89554<br>89555<br>89556<br>89557<br>89558<br>89559<br>89560<br>89561<br>89562<br>89563<br>89564<br>89565<br>89566<br>89567<br>89568<br>89569<br>89570<br>89571<br>89572<br>89573<br>89574<br>89575<br>89576<br>89577	2461474<br>2461475<br>2461476<br>2461477<br>2461478<br>2461479<br>2461480<br>2461481<br>2461482<br>2461483<br>2461484<br>2461485<br>2461486<br>2461487<br>2461488<br>2461489<br>2461490<br>2461491<br>2461492<br>2461493<br>2461494<br>2461495<br>2461496<br>2461497<br>2461498<br>2461499<br>2461500<br>2461501<br>2461502<br>2461503<br>2461504<br>2461505<br>2461506<br>2461507<br>2461508<br>2461509<br>2461510<br>2461511<br>2461512<br>2461513<br>2461514<br>2461515<br>2461516<br>2461517<br>2461518<br>2461519<br>2461520<br>2461521	core<br>core<br>core<br>extended<br>core<br>core<br>extended<br>core<br>core<br>extended<br>extended<br>extended<br>core<br>extended<br>full<br>extended<br>full<br>core<br>extended<br>core<br>extended<br>core<br>core<br>core<br>core<br>core<br>core<br>core<br>core<br>core<br>core<br>core<br>core<br>core<br>core<br>core<br>core<br>core<br>core<br>full<br>core<br>core<br>core<br>full<br>core<br>core<br>extended<br>extended	1	232593752	232681472	-	NM_005646<br>AK055044<br>BC016023<br>ENSESTT00000018243<br>ENSESTT00000018244<br>ENSESTT00000018245<br>ENSESTT00000018246<br>ENSESTT00000018247<br>ENSESTT00000018248<br>ENST00000040877<br>GENSCAN00000040475<br>GENSCAN00000040474<br>GENSCAN00000053812	Homo sapiens Tar (HIV-1) RNA binding protein 1 (TARBP1), mRNA.<br>Homo sapiens cDNA FLJ30482 fis, clone BRAWH2000034, moderately similar to TRP-185 protein.<br>Homo sapiens Tar (HIV-1) RNA binding protein 1, mRNA (cDNA clone IMAGE:4721653).<br><br><br><br><br><br><br>cdna:known-ccds chromosome:NCBI36:1:232593682:232681472:-1 gene:ENSG00000059588 CCDS1601.1<br>cdna:Genscan chromosome:NCBI36:1:232623071:232659749:-1<br>cdna:Genscan chromosome:NCBI36:1:232680462:232681472:-1<br>cdna:Genscan chromosome:NCBI36:1:232593946:232605562:-1

		2461522<br>2461523<br>2461524<br>2461525<br>2461526<br>2461527<br>2461528<br>2461529<br>2461530	extended<br>extended<br>full<br>core<br>core<br>core<br>core<br>core<br>core						
2474341	97705<br>97706<br>97707<br>97708<br>97709<br>97710<br>97711<br>97712<br>97713<br>97714<br>97715<br>97716<br>97717<br>97718<br>97719<br>97720<br>97721<br>97722<br>97723<br>97724<br>97725<br>97726<br>97727<br>97728<br>97729<br>97730<br>97731<br>97732<br>97733<br>97734<br>97735<br>97736<br>97737<br>97738<br>97739<br>97740<br>97741<br>97742<br>97743<br>97744<br>97745<br>97746<br>97747<br>97748<br>97749	2474342<br>2474343<br>2474344<br>2474345<br>2474346<br>2474347<br>2474348<br>2474349<br>2474350<br>2474351<br>2474352<br>2474353<br>2474354<br>2474355<br>2474356<br>2474357<br>2474358<br>2474359<br>2474360<br>2474361<br>2474362<br>2474363<br>2474364<br>2474365<br>2474366<br>2474367<br>2474368<br>2474369<br>2474370<br>2474371<br>2474372<br>2474373<br>2474374<br>2474375<br>2474376<br>2474377<br>2474378<br>2474379<br>2474380<br>2474381<br>2474382<br>2474383<br>2474384<br>2474385<br>2474386	core<br>core<br>core<br>core<br>core<br>extended<br>core<br>core<br>core<br>core<br>core<br>core<br>core<br>core<br>core<br>core<br>core<br>core<br>core<br>full<br>full<br>core<br>core<br>core<br>extended<br>core<br>extended<br>core<br>core<br>extended<br>core<br>core<br>extended<br>core<br>core<br>core<br>extended<br>extended<br>core<br>core<br>core<br>core<br>core<br>core<br>core	2	27293772	27322337	+	NM_004341<br>AL832309<br>ENSESTT00000058785<br>ENSESTT00000058786<br>ENSESTT00000058787<br>ENSESTT00000058788<br>ENSESTT00000058789<br>ENSESTT00000058790<br>ENSESTT00000058791<br>ENSESTT00000058792<br>ENSESTT00000058793<br>ENSESTT00000058794<br>ENSESTT00000058795<br>ENSESTT00000058796<br>ENSESTT00000058797<br>ENSESTT00000058798<br>ENSESTT00000058799<br>ENSESTT00000058800<br>ENSESTT00000058801<br>ENSESTT00000058802<br>ENSESTT00000058803<br>ENSESTT00000058804<br>ENSESTT00000058805<br>ENSESTT00000058806<br>ENSESTT00000058807<br>ENSESTT00000058808<br>ENSESTT00000058809<br>ENSESTT00000058810<br>ENSESTT00000058811<br>ENSESTT00000058812<br>ENSESTT00000058813<br>ENSESTT00000058814<br>ENSESTT00000058815<br>ENSESTT00000058816<br>ENSESTT00000058817<br>ENST00000380093<br>ENST00000264705<br>GENSCAN00000014770	Homo sapiens carbamoyl-phosphate synthetase 2, aspartate transcarbamylase, and dihydroorotase (CAD), mRNA.<br>Homo sapiens mRNA; cDNA DKFZp667L1519 (from clone DKFZp667L1519).<br><br><br><br><br><br><br><br><br><br><br><br><br><br><br><br><br><br><br><br><br><br><br><br><br><br><br><br><br><br><br><br><br><br>cdna:known-ccds chromosome:NCBI36:2:27293762:27320164:1 gene:ENSG00000084774 CCDS1742.1<br>cdna:known chromosome:NCBI36:2:27293762:27320158:1 gene:ENSG00000084774<br>cdna:Genscan chromosome:NCBI36:2:27288741:27294388:1

	97750<br>97751	2474387<br>2474388<br>2474389<br>2474390<br>2474391<br>2474392<br>2474393<br>2474394<br>2474395<br>2474396<br>2474397<br>2474398<br>2474399<br>2474400<br>2474401<br>2474402<br>2474403<br>2474404<br>2474405<br>2474406<br>2474407<br>2474408	core<br>core<br>extended<br>extended<br>core<br>core<br>extended<br>core<br>core<br>core<br>core<br>core<br>core<br>extended<br>core<br>core<br>full<br>extended<br>core<br>core<br>extended<br>extended						
2487082	105966<br>105967<br>105968<br>105969<br>105970<br>105971<br>105972<br>105973<br>105974<br>105975<br>105976<br>105977<br>105978<br>105979<br>105980<br>105981<br>105982<br>105983<br>105984<br>105985<br>105986<br>105987<br>105988<br>105989<br>105990<br>105991<br>105992<br>105993<br>105994<br>105995<br>105996<br>105997	2487083<br>2487084<br>2487085<br>2487086<br>2487087<br>2487088<br>2487089<br>2487090<br>2487091<br>2487092<br>2487093<br>2487094<br>2487095<br>2487096<br>2487097<br>2487098<br>2487099<br>2487100<br>2487101<br>2487102<br>2487103<br>2487104<br>2487105<br>2487106<br>2487107<br>2487108<br>2487109<br>2487110<br>2487111<br>2487112<br>2487113<br>2487114	extended<br>core<br>core<br>full<br>full<br>full<br>core<br>full<br>core<br>extended<br>full<br>full<br>full<br>core<br>core<br>full<br>core<br>core<br>full<br>full<br>extended<br>core<br>full<br>full<br>full<br>full<br>full<br>extended<br>extended<br>extended<br>core<br>extended	2	69093826	69378877	+	NM_053034<br>NM_018153<br>NM_032208<br>AF090095<br>AK001463<br>AK002160<br>AK022355<br>AK126783<br>AL110155<br>ENSESTT00000005753<br>ENSESTT00000005754<br>ENSESTT00000005755<br>ENSESTT00000005756<br>ENSESTT00000005757<br>ENSESTT00000005758<br>ENST00000303714<br>ENST00000308181<br>GENSCAN00000045579<br>GENSCAN00000038189	Homo sapiens anthrax toxin receptor 1 (ANTXR1), transcript variant 2, mRNA.<br>Homo sapiens anthrax toxin receptor 1 (ANTXR1), transcript variant 3, mRNA.<br>Homo sapiens anthrax toxin receptor 1 (ANTXR1), transcript variant 1, mRNA.<br>Homo sapiens clone IMAGE 286356.<br>Homo sapiens cDNA FLJ10601 fis, clone NT2RP2005000.<br>Homo sapiens cDNA FLJ11298 fis, clone PLACE1009794.<br>Homo sapiens cDNA FLJ12293 fis, clone MAMMA1001815.<br>Homo sapiens cDNA FLJ44833 fis, clone BRACE3048483.<br>Homo sapiens mRNA; cDNA DKFZp586F1324 (from clone DKFZp586F1324).<br><br><br><br><br><br><br>cdna:known-ccds chromosome:NCBI36:2:69093993:69329804:1 gene:ENSG00000169604 CCDS1892.1<br>cdna:known chromosome:NCBI36:2:69093993:69251040:1 gene:ENSG00000169604<br>cdna:Genscan chromosome:NCBI36:2:69169143:69171532:1<br>cdna:Genscan chromosome:NCBI36:2:69378468:69379251:1

	105998<br>105999<br>106000<br>106001<br>106002<br>106003<br>106004<br>106005<br>106006<br>106007<br>106008<br>106009<br>106010<br>106011<br>106012<br>106013<br>106014<br>106015<br>106016<br>106017<br>106018<br>106019<br>106020<br>106021<br>106022<br>106023<br>106024<br>106025<br>106026<br>106027<br>106028<br>106029<br>106030<br>106031<br>106032<br>106033<br>106034<br>106035<br>106036<br>106037<br>106038<br>106039<br>106040<br>106041<br>106042<br>106043<br>106044<br>106045<br>106046<br>106047<br>106048<br>106049<br>106050<br>106051	2487115<br>2487116<br>2487117<br>2487118<br>2487119<br>2487120<br>2487121<br>2487122<br>2487123<br>2487124<br>2487125<br>2487126<br>2487127<br>2487128<br>2487129<br>2487130<br>2487131<br>2487132<br>2487133<br>2487134<br>2487135<br>2487136<br>2487137<br>2487138<br>2487139<br>2487140<br>2487141<br>2487142<br>2487143<br>2487144<br>2487145<br>2487146<br>2487147<br>2487148<br>2487149<br>2487150<br>2487151<br>2487152<br>2487153<br>2487154<br>2487155<br>2487156<br>2487157<br>2487158<br>2487159<br>2487160<br>2487161<br>2487162<br>2487163<br>2487164<br>2487165<br>2487166<br>2487167<br>2487168	full<br>core<br>extended<br>extended<br>extended<br>full<br>full<br>full<br>extended<br>extended<br>extended<br>full<br>full<br>full<br>full<br>core<br>core<br>full<br>extended<br>extended<br>full<br>full<br>full<br>extended<br>full<br>full<br>core<br>core<br>extended<br>extended<br>full<br>full<br>full<br>extended<br>extended<br>extended<br>full<br>core<br>core<br>full<br>full<br>full<br>full<br>full<br>full<br>full<br>extended<br>core<br>extended<br>extended<br>core<br>full<br>extended<br>full						

	106052	2487169	full						
	106053	2487170	full						
	106054	2487171	extended						
	106055	2487172	full						
	106056	2487173	core						
	106057	2487174	core						
	106058	2487175	full						
	106059	2487176	full						
	106060	2487177	core						
	106061	2487178	full						
	106062	2487179	extended						
	106063	2487180	full						
	106064	2487181	full						
	106065	2487182	full						
	106066	2487183	full						
	106067	2487184	extended						
	106068	2487185	full						
	106069	2487186	full						
	106070	2487187	full						
	106071	2487188	extended						
	106072	2487189	full						
	106073	2487190	full						
	106074	2487191	full						
	106075	2487192	extended						
	106076	2487193	extended						
	106077	2487194	full						
	106078	2487195	extended						
	106079	2487196	full						
	106080	2487197	full						
	106081	2487198	full						
		2487199	full						
		2487200	extended						
		2487201	full						
		2487202	extended						
		2487203	extended						
		2487204	extended						
		2487205	extended						
		2487206	extended						
		2487207	extended						
		2487208	extended						
		2487209	full						
		2487210	core						
		2487211	core						
		2487212	core						
		2487213	core						
		2487214	core						
		2487215	core						
		2487216	core						
		2487217	core						
		2487218	core						
		2487219	extended						
		2487220	full						
		2487221	full						
		2487222	full						

		2487223<br>2487224<br>2487225<br>2487226<br>2487227<br>2487228<br>2487229<br>2487230<br>2487231<br>2487232<br>2487233<br>2487234<br>2487235<br>2487236<br>2487237<br>2487238<br>2487239	full<br>full<br>full<br>full<br>full<br>full<br>full<br>full<br>full<br>full<br>full<br>full<br>full<br>full<br>full<br>full<br>full						
2496382	111617<br>111618<br>111619<br>111620<br>111621<br>111622<br>111623<br>111624<br>111625<br>111626<br>111627<br>111628<br>111629<br>111630<br>111631<br>111632<br>111633<br>111634<br>111635<br>111636<br>111637<br>111638<br>111639<br>111640<br>111641<br>111642<br>111643<br>111644<br>111645<br>111646<br>111647<br>111648<br>111649<br>111650<br>111651<br>111652<br>111653	2496383<br>2496384<br>2496385<br>2496386<br>2496387<br>2496388<br>2496389<br>2496390<br>2496391<br>2496392<br>2496393<br>2496394<br>2496395<br>2496396<br>2496397<br>2496398<br>2496399<br>2496400<br>2496401<br>2496402<br>2496403<br>2496404<br>2496405<br>2496406<br>2496407<br>2496408<br>2496409<br>2496410<br>2496411<br>2496412<br>2496413<br>2496414<br>2496415<br>2496416<br>2496417<br>2496418<br>2496419	full<br>full<br>extended<br>full<br>full<br>full<br>full<br>full<br>full<br>full<br>full<br>full<br>full<br>full<br>full<br>full<br>full<br>full<br>full<br>extended<br>full<br>full<br>full<br>extended<br>extended<br>extended<br>extended<br>extended<br>full<br>extended<br>extended<br>full<br>extended<br>extended<br>full<br>full<br>full	2	100752218	100979719	+	NM_002518<br>AK022013<br>AK026791<br>AK091665<br>AK128586<br>AK130869<br>BX640699<br>ENSESTT00000008758<br>ENSESTT00000008759<br>ENSESTT00000008760<br>ENSESTT00000009840<br>ENSESTT00000009841<br>ENSESTT00000009842<br>ENSESTT00000009843<br>ENSESTT00000009844<br>ENSESTT00000009845<br>ENSESTT00000009846<br>ENSESTT00000009847<br>ENST00000335681<br>ENST00000354182<br>ENST00000341082<br>ENST00000320656<br>GENSCAN00000013960<br>GENSCAN00000002933<br>GENSCAN00000023416	Homo sapiens neuronal PAS domain protein 2 (NPAS2), mRNA.<br>Homo sapiens cDNA FLJ11951 fis, clone HEMBB1000827.<br>Homo sapiens cDNA: FLJ23138 fis, clone LNG08913.<br>Homo sapiens cDNA FLJ34346 fis, clone FEBRA2010768.<br>Homo sapiens cDNA FLJ46745 fis, clone TRACH3021883, moderately similar to Neuronal PAS domain protein 2.<br>Homo sapiens cDNA FLJ27359 fis, clone UBA01020.<br>Homo sapiens mRNA; cDNA DKFZp686F0725 (from clone DKFZp686F0725).<br><br><br><br><br><br><br><br><br><br><br><br>cdna:known-ccds chromosome:NCBI36:2:100803045:100979719:1 gene:ENSG00000170485 CCDS2048.1<br>cdna:novel chromosome:NCBI36:2:100908038:100978946:1 gene:ENSG00000170485<br>cdna:novel chromosome:NCBI36:X:67736666:67737040:1 gene:ENSG00000189128<br>cdna:novel chromosome:NCBI36:14:58331126:58331500:1 gene:ENSG00000177132<br>cdna:Genscan chromosome:NCBI36:2:100685486:100802574:1<br>cdna:Genscan chromosome:NCBI36:2:100859671:100861019:1<br>cdna:Genscan chromosome:NCBI36:2:100896830:100989275:1

	111654	2496420	extended						
	111655	2496421	full						
	111656	2496422	full						
	111657	2496423	core						
	111658	2496424	extended						
	111659	2496425	extended						
	111660	2496426	full						
	111661	2496427	full						
	111662	2496428	full						
	111663	2496429	full						
	111664	2496430	full						
	111665	2496431	full						
	111666	2496432	full						
	111667	2496433	full						
	111668	2496434	full						
	111669	2496435	full						
	111670	2496436	core						
	111671	2496437	full						
	111672	2496438	extended						
	111673	2496439	full						
	111674	2496440	core						
	111675	2496441	full						
	111676	2496442	extended						
	111677	2496443	extended						
	111678	2496444	extended						
	111679	2496445	extended						
	111680	2496446	core						
	111681	2496447	full						
	111682	2496448	core						
	111683	2496449	core						
	111684	2496450	full						
	111685	2496451	extended						
	111686	2496452	extended						
	111687	2496453	full						
	111688	2496454	full						
	111689	2496455	extended						
	111690	2496456	core						
	111691	2496457	full						
	111692	2496458	core						
	111693	2496459	full						
	111694	2496460	core						
	111695	2496461	core						
	111696	2496462	full						
	111697	2496463	extended						
	111698	2496464	extended						
	111699	2496465	core						
	111700	2496466	full						
		2496467	full						
		2496468	full						
		2496469	core						
		2496470	full						
		2496471	core						
		2496472	extended						
		2496473	full						

		2496474<br>2496475<br>2496476<br>2496477<br>2496478<br>2496479<br>2496480<br>2496481<br>2496482<br>2496483<br>2496484<br>2496485<br>2496486<br>2496487<br>2496488<br>2496489<br>2496490<br>2496491<br>2496492<br>2496493<br>2496494<br>2496495<br>2496496<br>2496497<br>2496498<br>2496499<br>2496500<br>2496501<br>2496502<br>2496503<br>2496504<br>2496505<br>2496506<br>2496507<br>2496508<br>2496509<br>2496510<br>2496511<br>2496512<br>2496513<br>2496514<br>2496515<br>2496516<br>2496517	full<br>core<br>full<br>full<br>full<br>full<br>full<br>core<br>extended<br>extended<br>extended<br>extended<br>extended<br>extended<br>extended<br>extended<br>extended<br>extended<br>extended<br>core<br>core<br>extended<br>extended<br>extended<br>extended<br>extended<br>extended<br>extended<br>extended<br>core<br>core<br>core<br>full<br>full<br>core<br>core<br>full<br>extended<br>extended<br>core<br>core<br>core<br>core<br>core						
2516023	124122<br>124123<br>124124<br>124125<br>124126<br>124127<br>124128<br>124129<br>124130<br>124131	2516024<br>2516025<br>2516026<br>2516027<br>2516028<br>2516029<br>2516030<br>2516031<br>2516032<br>2516033	full<br>full<br>full<br>full<br>full<br>full<br>extended<br>full<br>extended<br>core	2	173844494	173960400	+	NM_031942<br>NM_145810<br>AK075134<br>AK223524<br>AL833728<br>AL834186<br>ENSESTT00000013609<br>ENSESTT00000013610<br>ENSESTT00000013611<br>ENSESTT00000013612	Homo sapiens cell division cycle associated 7 (CDCA7), transcript variant 1, mRNA.<br>Homo sapiens cell division cycle associated 7 (CDCA7), transcript variant 2, mRNA.<br>Homo sapiens cDNA FLJ90653 fis, clone PLACE1004519.<br>Homo sapiens mRNA for cell division cycle associated protein 7 isoform 2 variant, clone: FCC123E09.<br>Homo sapiens mRNA; cDNA DKFZp666F035 (from clone DKFZp666F035).<br>Homo sapiens mRNA; cDNA DKFZp667L165 (from clone DKFZp667L165).

	124132<br>124133<br>124134<br>124135<br>124136<br>124137<br>124138<br>124139<br>124140<br>124141<br>124142<br>124143<br>124144<br>124145<br>124146	2516034<br>2516035<br>2516036<br>2516037<br>2516038<br>2516039<br>2516040<br>2516041<br>2516042<br>2516043<br>2516044<br>2516045<br>2516046<br>2516047<br>2516048<br>2516049<br>2516050<br>2516051<br>2516052<br>2516053<br>2516054<br>2516055<br>2516056<br>2516057<br>2516058<br>2516059<br>2516060<br>2516061<br>2516062<br>2516063	full<br>full<br>extended<br>core<br>core<br>core<br>core<br>core<br>core<br>extended<br>core<br>extended<br>extended<br>extended<br>extended<br>core<br>core<br>core<br>full<br>core<br>core<br>core<br>core<br>core<br>core<br>full<br>full<br>full<br>full<br>full					ENST00000306721<br>ENST00000347703<br>GENSCAN00000036481	cdna:known-ccds chromosome:NCBI36:2:173927794:173941965:1 gene:ENSG00000144354 CCDS2252.1<br>cdna:known-ccds chromosome:NCBI36:2:173927794:173941965:1 gene:ENSG00000144354 CCDS2253.1<br>cdna:Genscan chromosome:NCBI36:2:173844494:173960400:1
2528476	131887<br>131888<br>131889<br>131890<br>131891<br>131892<br>131893<br>131894<br>131895<br>131896<br>131897	2528477<br>2528478<br>2528479<br>2528480<br>2528481<br>2528482<br>2528483<br>2528484<br>2528485<br>2528486<br>2528487<br>2528488<br>2528489<br>2528490<br>2528491<br>2528492<br>2528493<br>2528494<br>2528495<br>2528496<br>2528497	extended<br>core<br>core<br>core<br>core<br>core<br>core<br>core<br>core<br>core<br>extended<br>core<br>extended<br>extended<br>core<br>core<br>core<br>core<br>core<br>core<br>core	2	219991301	219999683	+	NM_001927<br>AK126500<br>AK128689<br>AY603755<br>ENSESTT00000029824<br>ENSESTT00000029825<br>ENSESTT00000029826<br>ENSESTT00000029827<br>ENSESTT00000029828<br>ENST00000373960<br>ENST00000273074<br>ENST00000312358<br>ENST00000265327<br>GENSCAN00000040487	Homo sapiens desmin (DES), mRNA.<br>Homo sapiens cDNA FLJ44536 fis, clone UTERU3004992, highly similar to Aortic preferentially expressed protein 1.<br>Homo sapiens cDNA FLJ46856 fis, clone UTERU3010409, highly similar to Aortic preferentially expressed protein 1.<br>Homo sapiens striated muscle preferentially expressed protein mRNA, partial cds.<br><br><br><br><br><br>cdna:known chromosome:NCBI36:2:219991343:219999705:1 gene:ENSG00000175084<br>cdna:known chromosome:NCBI36:2:219991429:219999698:1 gene:ENSG00000175084<br>cdna:known chromosome:NCBI36:2:220007812:220066598:1 gene:ENSG00000072195<br>cdna:known chromosome:NCBI36:2:220017618:220066594:1 gene:ENSG00000072195<br>cdna:Genscan chromosome:NCBI36:2:219991117:220065752:1
2531589	133765<br>133766	2531590<br>2531591	core<br>core	2	231437872	231452176	+	NM_001012516<br>NM_030926	Homo sapiens integral membrane protein 2C (ITM2C), transcript variant 2, mRNA.<br>Homo sapiens integral membrane protein 2C (ITM2C), transcript variant 1, mRNA.

	133767<br>133768<br>133769<br>133770<br>133771<br>133772<br>133773<br>133774<br>133775<br>133776<br>133777	2531592<br>2531593<br>2531594<br>2531595<br>2531596<br>2531597<br>2531598<br>2531599<br>2531600<br>2531601<br>2531602<br>2531603<br>2531604<br>2531605<br>2531606<br>2531607<br>2531608<br>2531609<br>2531610<br>2531611<br>2531612<br>2531613<br>2531614<br>2531615	core<br>core<br>extended<br>full<br>full<br>full<br>full<br>full<br>full<br>core<br>full<br>full<br>full<br>full<br>core<br>full<br>core<br>core<br>core<br>core<br>core<br>core<br>core<br>core					NM_001012514<br>CR749389<br>ENSESTT00000043428<br>ENST00000326427<br>ENST00000326407<br>ENST00000335005<br>GENSCAN00000048898	Homo sapiens integral membrane protein 2C (ITM2C), transcript variant 3, mRNA.<br>Homo sapiens mRNA; cDNA DKFZp686L07102 (from clone DKFZp686L07102).<br><br>cdna:known-ccds chromosome:NCBI36:2:231437865:231452206:1 gene:ENSG00000135916 CCDS2479.1<br>cdna:known chromosome:NCBI36:2:231437865:231452206:1 gene:ENSG00000135916<br>cdna:known chromosome:NCBI36:2:231437865:231452206:1 gene:ENSG00000135916<br>cdna:Genscan chromosome:NCBI36:2:231437985:231451058:1
2533019	134688<br>134689<br>134690<br>134691<br>134692<br>134693<br>134694<br>134695<br>134696<br>134697<br>134698<br>134699<br>134700<br>134701<br>134702<br>134703<br>134704<br>134705<br>134706<br>134707<br>134708<br>134709<br>134710<br>134711<br>134712<br>134713<br>134714<br>134715<br>134716<br>134717	2533016<br>2533017<br>2533018<br>2533020<br>2533021<br>2533022<br>2533023<br>2533024<br>2533025<br>2533026<br>2533027<br>2533028<br>2533029<br>2533030<br>2533031<br>2533032<br>2533033<br>2533034<br>2533035<br>2533036<br>2533037<br>2533038<br>2533039<br>2533040<br>2533041<br>2533042<br>2533043<br>2533044<br>2533045<br>2533046	full<br>full<br>full<br>full<br>full<br>full<br>full<br>core<br>core<br>core<br>core<br>core<br>extended<br>full<br>full<br>full<br>full<br>full<br>core<br>core<br>core<br>core<br>core<br>core<br>core<br>core<br>full<br>full<br>full<br>full	2	234176938	234346674	+	NM_019075<br>NM_019077<br>NM_019076<br>NM_007120<br>NM_001072<br>NM_021027<br>NM_019078<br>NM_000463<br>NM_019093<br>NM_205862<br>AF030310<br>AK124100<br>BC001521<br>BC053576<br>BC121036<br>U89507<br>J04093<br>ENSESTT00000035611<br>ENSESTT00000035612<br>ENSESTT00000035613<br>ENST00000373435<br>ENST00000344644<br>ENST00000373424<br>ENST00000373421<br>ENST00000373409<br>ENST00000373406<br>ENST00000373460<br>ENST00000373450<br>ENST00000373445<br>ENST00000354728	Homo sapiens UDP glucuronosyltransferase 1 family, polypeptide A10 (UGT1A10), mRNA.<br>Homo sapiens UDP glucuronosyltransferase 1 family, polypeptide A7 (UGT1A7), mRNA.<br>Homo sapiens UDP glucuronosyltransferase 1 family, polypeptide A8 (UGT1A8), mRNA.<br>Homo sapiens UDP glucuronosyltransferase 1 family, polypeptide A4 (UGT1A4), mRNA.<br>Homo sapiens UDP glucuronosyltransferase 1 family, polypeptide A6 (UGT1A6), transcript variant 1, mRNA.<br>Homo sapiens UDP glucuronosyltransferase 1 family, polypeptide A9 (UGT1A9), mRNA.<br>Homo sapiens UDP glucuronosyltransferase 1 family, polypeptide A5 (UGT1A5), mRNA.<br>Homo sapiens UDP glucuronosyltransferase 1 family, polypeptide A1 (UGT1A1), mRNA.<br>Homo sapiens UDP glucuronosyltransferase 1 family, polypeptide A3 (UGT1A3), mRNA.<br>Homo sapiens UDP glucuronosyltransferase 1 family, polypeptide A6 (UGT1A6), transcript variant 2, mRNA.<br>Homo sapiens UDP-glucuronosyltransferase 1A8 (UGT1A8) mRNA, complete cds.<br>Homo sapiens cDNA FLJ42106 fis, clone TESOP2007725.<br>Homo sapiens UDP glucuronosyltransferase 1 family, polypeptide A6, mRNA (cDNA clone IMAGE:2988019), partial cds.<br>Homo sapiens UDP glucuronosyltransferase 1 family, polypeptide A10, mRNA (cDNA clone MGC:61718 IMAGE:4452440),<br>complete cds.<br>Homo sapiens UDP glucuronosyltransferase 1 family, polypeptide A3, mRNA (cDNA clone MGC:149704 IMAGE:40117585),<br>complete cds.<br>Human UDP-glucuronosyltransferase 1A7 mRNA, complete cds.<br>Homo sapiens phenol UDP-glucuronosyltransferase (UDPGT) mRNA, complete cds.<br><br><br><br>cdna:known-ccds chromosome:NCBI36:2:234245238:234346685:1 gene:ENSG00000167165 CCDS2505.1<br>cdna:known-ccds chromosome:NCBI36:2:234255323:234346684:1 gene:ENSG00000167165 CCDS2506.1<br>cdna:known-ccds chromosome:NCBI36:2:234264992:234346053:1 gene:ENSG00000167165 CCDS2508.1<br>cdna:known-ccds chromosome:NCBI36:2:234266251:234346685:1 gene:ENSG00000167165 CCDS2507.1<br>cdna:known-ccds chromosome:NCBI36:2:234302493:234346684:1 gene:ENSG00000167165 CCDS2509.1<br>cdna:known-ccds chromosome:NCBI36:2:234333633:234346684:1 gene:ENSG00000167165 CCDS2510.1<br>cdna:known chromosome:NCBI36:2:234191030:234346695:1 gene:ENSG00000167165<br>cdna:known chromosome:NCBI36:2:234209839:234346690:1 gene:ENSG00000167165

	134718	2533047	full					ENST00000287677	cdna:known chromosome:NCBI36:2:234209887:234343664:1 gene:ENSG00000167165
	134719	2533048	full					ENST00000373426	cdna:known chromosome:NCBI36:2:234245277:234346684:1 gene:ENSG00000167165
	134720	2533049	full					ENST00000340399	cdna:known chromosome:NCBI36:2:234245277:234346684:1 gene:ENSG00000167165
	134721	2533050	extended					ENST00000360418	cdna:known chromosome:NCBI36:2:234255323:234346684:1 gene:ENSG00000167165
	134722	2533051	core					ENST00000305139	cdna:known chromosome:NCBI36:2:234255755:234265731:1 gene:ENSG00000167165
	134723	2533052	core					ENST00000373414	cdna:known chromosome:NCBI36:2:234265060:234346684:1 gene:ENSG00000167165
	134724	2533053	core					ENST00000373412	cdna:known chromosome:NCBI36:2:234266294:234346684:1 gene:ENSG00000167165
	134725	2533054	core					ENST00000318990	cdna:known chromosome:NCBI36:2:234286377:234346684:1 gene:ENSG00000167165
	134726	2533055	core					ENST00000305208	cdna:known chromosome:NCBI36:2:234292163:234346684:1 gene:ENSG00000167165
	134727	2533056	core					GENSCAN00000058444	cdna:known chromosome:NCBI36:2:234302512:234346684:1 gene:ENSG00000167165
	134728	2533057	core					GENSCAN00000058447	cdna:known chromosome:NCBI36:2:234333658:234346684:1 gene:ENSG00000167165
	134729	2533058	core					GENSCAN00000058446	cdna:Genscan chromosome:NCBI36:2:234255323:234273588:1
	134730	2533059	core					GENSCAN00000058445	cdna:Genscan chromosome:NCBI36:2:234286377:234291458:1
	134731	2533060	extended					GENSCAN00000058441	cdna:Genscan chromosome:NCBI36:2:234292206:234293270:1
	134732	2533061	full					GENSCAN00000029970	cdna:Genscan chromosome:NCBI36:2:234302512:234304450:1
	134733	2533062	full						cdna:Genscan chromosome:NCBI36:2:234320727:234328723:1
	134734	2533063	full						cdna:Genscan chromosome:NCBI36:2:234333673:234345944:1
	134735	2533064	extended						
	134736	2533065	core						
	134737	2533066	core						
	134738	2533067	core						
	134739	2533068	core						
	134740	2533069	full						
	134741	2533070	full						
	134742	2533071	full						
		2533072	extended						
		2533073	extended						
		2533074	extended						
		2533075	extended						
		2533076	full						
		2533077	core						
		2533078	core						
		2533079	core						
		2533080	core						
		2533081	core						
		2533082	full						
		2533083	extended						
		2533084	full						
		2533085	core						
		2533086	core						
		2533087	core						
		2533088	core						
		2533089	core						
		2533090	core						
		2533091	full						
		2533092	full						
		2533093	full						
		2533094	core						
		2533095	core						
		2533096	core						
		2533097	core						
		2533098	core						
		2533099	extended						
		2533100	full						

		2533101<br>2533102<br>2533103<br>2533104<br>2533105<br>2533106<br>2533107<br>2533108<br>2533109<br>2533110<br>2533111<br>2533112<br>2533113<br>2533114<br>2533115<br>2533116<br>2533117<br>2533118<br>2533119<br>2533120<br>2533121<br>2533122<br>2533123<br>2533124<br>2533125<br>2533126<br>2533127<br>2533128<br>2533129<br>2533130<br>2533131<br>2533132	core<br>core<br>core<br>core<br>full<br>full<br>extended<br>extended<br>extended<br>extended<br>extended<br>extended<br>full<br>full<br>extended<br>extended<br>extended<br>extended<br>core<br>core<br>full<br>full<br>core<br>core<br>core<br>core<br>extended<br>extended<br>full<br>core<br>core<br>core						
2560076	151809<br>151810<br>151811<br>151812<br>151813<br>151814<br>151815<br>151816<br>151817<br>151818<br>151819<br>151820<br>151821<br>151822<br>151823<br>151824<br>151825<br>151826<br>151827<br>151828<br>151829<br>151830	2560077<br>2560078<br>2560079<br>2560080<br>2560081<br>2560082<br>2560083<br>2560084<br>2560085<br>2560086<br>2560087<br>2560088<br>2560089<br>2560090<br>2560091<br>2560092<br>2560093<br>2560094<br>2560095<br>2560096<br>2560097<br>2560098	extended<br>full<br>core<br>core<br>core<br>core<br>core<br>extended<br>extended<br>full<br>extended<br>core<br>core<br>core<br>extended<br>core<br>core<br>core<br>core<br>extended<br>core<br>core	2	74504785	74523034	-	NM_033046<br>NM_001015055<br>NM_001015056<br>ENSESTT00000037314<br>ENSESTT00000037315<br>ENSESTT00000037316<br>ENSESTT00000037317<br>ENSESTT00000037318<br>ENST00000305557<br>ENST00000272430<br>ENST00000233330<br>GENSCAN00000039288	Homo sapiens rhotekin (RTKN), transcript variant 2, mRNA.<br>Homo sapiens rhotekin (RTKN), transcript variant 1, mRNA.<br>Homo sapiens rhotekin (RTKN), transcript variant 3, mRNA.<br><br><br><br><br><br>cdna:known-ccds chromosome:NCBI36:2:74506497:74521218:-1 gene:ENSG00000114993 CCDS1941.1<br>cdna:known chromosome:NCBI36:2:74506497:74522568:-1 gene:ENSG00000114993<br>cdna:known chromosome:NCBI36:2:74506497:74521218:-1 gene:ENSG00000114993<br>cdna:Genscan chromosome:NCBI36:2:74506834:74522451:-1

	151831	2560099<br>2560100<br>2560101<br>2560102<br>2560103<br>2560104<br>2560105<br>2560106<br>2560107<br>2560108<br>2560109<br>2560110<br>2560111<br>2560112<br>2560113<br>2560114<br>2560115<br>2560116<br>2560117<br>2560118<br>2560119<br>2560120<br>2560121	core<br>core<br>extended<br>extended<br>extended<br>extended<br>extended<br>core<br>extended<br>full<br>full<br>core<br>core<br>extended<br>extended<br>extended<br>full<br>full<br>core<br>core<br>extended<br>extended<br>extended						
2570193	158069<br>158070<br>158071<br>158072<br>158073<br>158074<br>158075<br>158076<br>158077<br>158078<br>158079<br>158080<br>158081<br>158082<br>158083<br>158084<br>158085<br>158086<br>158087<br>158088<br>158089<br>158090<br>158091<br>158092<br>158093<br>158094<br>158095<br>158096<br>158097<br>158098<br>158099	2570194<br>2570195<br>2570196<br>2570197<br>2570198<br>2570199<br>2570200<br>2570201<br>2570202<br>2570203<br>2570204<br>2570205<br>2570206<br>2570207<br>2570208<br>2570209<br>2570210<br>2570211<br>2570212<br>2570213<br>2570214<br>2570215<br>2570216<br>2570217<br>2570218<br>2570219<br>2570220<br>2570221<br>2570222<br>2570223<br>2570224	core<br>core<br>core<br>core<br>extended<br>extended<br>core<br>full<br>full<br>full<br>core<br>extended<br>extended<br>full<br>full<br>extended<br>full<br>extended<br>full<br>full<br>extended<br>extended<br>full<br>extended<br>extended<br>full<br>extended<br>extended<br>extended<br>extended<br>extended	2	110198740	110616256	-	NM_005434<br>ENSESTT00000036525<br>ENSESTT00000036526<br>ENST00000272462<br>GENSCAN00000054913	Homo sapiens mal, T-cell differentiation protein-like (MALL), mRNA.<br><br><br>cdna:known-ccds chromosome:NCBI36:2:110198738:110231432:-1 gene:ENSG00000144063 CCDS2085.1<br>cdna:Genscan chromosome:NCBI36:2:110200561:110230658:-1

	158100	2570225<br>2570226<br>2570227<br>2570228<br>2570229<br>2570230<br>2570231<br>2570232<br>2570233	extended<br>extended<br>extended<br>full<br>full<br>extended<br>extended<br>extended<br>extended						
2570616	158325<br>158326<br>158327<br>158328<br>158329<br>158330<br>158331<br>158332<br>158333<br>158334<br>158335<br>158336<br>158337<br>158338<br>158339<br>158340<br>158341<br>158342<br>158343<br>158344<br>158345<br>158346<br>158347<br>158348<br>158349<br>158350<br>158351<br>158352<br>158353<br>158354<br>158355<br>158356<br>158357<br>158358<br>158359<br>158360<br>158361<br>158362<br>158363<br>158364<br>158365<br>158366<br>158367<br>158368<br>158369	2570617<br>2570618<br>2570619<br>2570620<br>2570621<br>2570622<br>2570623<br>2570624<br>2570625<br>2570626<br>2570627<br>2570628<br>2570629<br>2570630<br>2570631<br>2570632<br>2570633<br>2570634<br>2570635<br>2570636<br>2570637<br>2570638<br>2570639<br>2570640<br>2570641<br>2570642<br>2570643<br>2570644<br>2570645<br>2570646<br>2570647<br>2570648<br>2570649<br>2570650<br>2570651<br>2570652<br>2570653<br>2570654<br>2570655<br>2570656<br>2570657<br>2570658<br>2570659<br>2570660<br>2570661	full<br>full<br>extended<br>extended<br>extended<br>extended<br>full<br>extended<br>extended<br>extended<br>extended<br>core<br>core<br>core<br>extended<br>extended<br>core<br>core<br>core<br>full<br>core<br>full<br>full<br>core<br>extended<br>extended<br>core<br>extended<br>extended<br>core<br>core<br>extended<br>core<br>core<br>core<br>core<br>core<br>extended<br>core<br>extended<br>core<br>extended<br>core<br>extended<br>extended	2	111088456	111174950	-	NM_004336<br>AK023540<br>ENSESTT00000019182<br>ENST00000302759<br>ENST00000389945<br>ENST00000389944<br>GENSCAN00000013627	Homo sapiens BUB1 budding uninhibited by benzimidazoles 1 homolog (yeast) (BUB1), mRNA.<br>Homo sapiens cDNA FLJ13478 fis, clone PLACE1003709, highly similar to Homo sapiens mitotic checkpoint kinase Bub1 (BUB1) mRNA.<br><br>cdna:known chromosome:NCBI36:2:111111883:111152135:-1 gene:ENSG00000169679<br>cdna:known chromosome:NCBI36:2:111112013:111152043:-1 gene:ENSG00000169679<br>cdna:known chromosome:NCBI36:2:111112013:111152043:-1 gene:ENSG00000169679<br>cdna:Genscan chromosome:NCBI36:2:111112013:111194776:-1

	158370<br>158371<br>158372	2570662<br>2570663<br>2570664<br>2570665<br>2570666<br>2570667<br>2570668<br>2570669<br>2570670<br>2570671<br>2570672<br>2570673<br>2570674<br>2570675<br>2570676<br>2570677<br>2570678<br>2570679<br>2570680<br>2570681<br>2570682<br>2570683<br>2570684<br>2570685<br>2570686	extended<br>core<br>core<br>core<br>extended<br>extended<br>core<br>core<br>core<br>extended<br>extended<br>core<br>core<br>core<br>core<br>core<br>core<br>full<br>full<br>full<br>full<br>full<br>full<br>full<br>full						
2574984	161021<br>161022<br>161023<br>161024<br>161025<br>161026<br>161027<br>161028<br>161029<br>161030<br>161031<br>161032<br>161033<br>161034<br>161035<br>161036<br>161037<br>161038<br>161039<br>161040<br>161041<br>161042<br>161043<br>161044<br>161045<br>161046<br>161047<br>161048<br>161049	2574985<br>2574986<br>2574987<br>2574988<br>2574989<br>2574990<br>2574991<br>2574992<br>2574993<br>2574994<br>2574995<br>2574996<br>2574997<br>2574998<br>2574999<br>2575000<br>2575001<br>2575002<br>2575003<br>2575004<br>2575005<br>2575006<br>2575007<br>2575008<br>2575009<br>2575010<br>2575011<br>2575012<br>2575013	full<br>full<br>core<br>core<br>core<br>core<br>core<br>core<br>core<br>core<br>core<br>core<br>core<br>extended<br>core<br>core<br>extended<br>core<br>extended<br>extended<br>core<br>extended<br>core<br>core<br>core<br>core<br>core<br>extended<br>extended	2	128112306	128155497	-	NM_017980<br>AF527766<br>AF527767<br>AF527768<br>AF527769<br>AF527770<br>AK055363<br>AK091652<br>AK093692<br>AK094954<br>AK095790<br>AK097026<br>AK123014<br>CR592921<br>ENSESTT00000037033<br>ENSESTT00000037034<br>ENSESTT00000037035<br>ENSESTT00000037036<br>ENSESTT00000037037<br>ENST00000324938<br>ENST00000324864<br>ENST00000342067<br>ENST00000355119<br>GENSCAN00000038207<br>GENSCAN00000008986	Homo sapiens LIM and senescent cell antigen-like domains 2 (LIMS2), mRNA.<br>Homo sapiens tissue-type spleen LIM-like protein 2C mRNA, complete cds.<br>Homo sapiens tissue-type uterus LIM-like protein 2D mRNA, complete cds.<br>Homo sapiens tissue-type brain LIM-like protein 2E mRNA, complete cds.<br>Homo sapiens tissue-type brain LIM-like protein 2F mRNA, complete cds.<br>Homo sapiens tissue-type brain LIM-like protein 2G mRNA, complete cds.<br>Homo sapiens cDNA FLJ30801 fis, clone FEBRA2001217, highly similar to PINCH PROTEIN.<br>Homo sapiens cDNA FLJ34333 fis, clone FEBRA2009276, highly similar to PINCH PROTEIN.<br>Homo sapiens cDNA FLJ36373 fis, clone THYMU2008111, moderately similar to PINCH PROTEIN.<br>Homo sapiens cDNA FLJ37635 fis, clone BRCOC2017856, highly similar to PINCH PROTEIN.<br>Homo sapiens cDNA FLJ38471 fis, clone FEBRA2022055, highly similar to PINCH PROTEIN.<br>Homo sapiens cDNA FLJ39707 fis, clone SMINT2012734.<br>Homo sapiens cDNA FLJ16826 fis, clone UTERU3006687, highly similar to Homo sapiens LIM and senescent cell antigen-like domains 1 (LIMS1).<br>full-length cDNA clone CS0DJ014YK07 of T cells (Jurkat cell line) Cot 10-normalized of Homo sapiens (human).<br><br><br><br><br><br>cdna:known-ccds chromosome:NCBI36:2:128112475:128138590:-1 gene:ENSG00000072163 CCDS2147.1<br>cdna:known chromosome:NCBI36:2:128112473:128132267:-1 gene:ENSG00000072163<br>cdna:known chromosome:NCBI36:2:128112475:128118157:-1 gene:ENSG00000072163<br>cdna:known chromosome:NCBI36:2:128113093:128155507:-1 gene:ENSG00000072163<br>cdna:Genscan chromosome:NCBI36:2:128113326:128119675:-1<br>cdna:Genscan chromosome:NCBI36:2:128127128:128155062:-1

		2575014<br>2575015<br>2575016<br>2575017<br>2575018<br>2575019<br>2575020<br>2575021<br>2575022<br>2575023<br>2575024<br>2575025<br>2575026<br>2575027<br>2575028<br>2575029<br>2575030<br>2575031<br>2575032<br>2575033<br>2575034<br>2575035<br>2575036<br>2575037<br>2575038<br>2575039<br>2575040<br>2575041<br>2575042<br>2575043<br>2575044<br>2575045	full<br>core<br>extended<br>full<br>extended<br>core<br>core<br>core<br>extended<br>extended<br>extended<br>extended<br>full<br>core<br>extended<br>extended<br>core<br>core<br>extended<br>full<br>full<br>core<br>core<br>extended<br>extended<br>extended<br>full<br>full<br>extended<br>extended<br>full<br>core						
2584134	166741<br>166742<br>166743<br>166744<br>166745<br>166746<br>166747<br>166748<br>166749<br>166750<br>166751<br>166752<br>166753<br>166754<br>166755<br>166756<br>166757<br>166758<br>166759<br>166760<br>166761<br>166762	2584135<br>2584136<br>2584137<br>2584138<br>2584139<br>2584140<br>2584141<br>2584142<br>2584143<br>2584144<br>2584145<br>2584146<br>2584147<br>2584148<br>2584149<br>2584150<br>2584151<br>2584152<br>2584153<br>2584154<br>2584155<br>2584156	core<br>core<br>core<br>core<br>core<br>core<br>extended<br>extended<br>extended<br>core<br>extended<br>full<br>full<br>core<br>core<br>core<br>extended<br>core<br>extended<br>extended<br>extended<br>full	2	162735446	162809835	-	NM_022168<br>NM_004460<br>NM_002054<br>AK055327<br>AL832166<br>ENSESTT00000006312<br>ENSESTT00000006315<br>ENSESTT00000050859<br>ENSESTT00000050860<br>ENSESTT00000050861<br>ENSESTT00000050862<br>ENSESTT00000050863<br>ENSESTT00000050864<br>ENST00000263642<br>ENST00000188790<br>GENSCAN00000033018	Homo sapiens interferon induced with helicase C domain 1 (IFIH1), mRNA.<br>Homo sapiens fibroblast activation protein, alpha (FAP), mRNA.<br>Homo sapiens glucagon (GCG), mRNA.<br>Homo sapiens cDNA FLJ30765 fis, clone FEBRA2000659, highly similar to Human fibroblast activation protein mRNA.<br>Homo sapiens mRNA; cDNA DKFZp686G13158 (from clone DKFZp686G13158).<br><br><br><br><br><br><br><br><br>cdna:known-ccds chromosome:NCBI36:2:162831836:162883285:-1 gene:ENSG00000115267 CCDS2217.1<br>cdna:known chromosome:NCBI36:2:162735446:162808291:-1 gene:ENSG00000078098<br>cdna:Genscan chromosome:NCBI36:2:162692879:162846301:-1

	166763<br>166764<br>166765<br>166766<br>166767<br>166768<br>166769<br>166770<br>166771<br>166772<br>166773<br>166774<br>166775<br>166776<br>166777<br>166778<br>166779<br>166780<br>166781<br>166782<br>166783<br>166784<br>166785<br>166786<br>166787	2584157<br>2584158<br>2584159<br>2584160<br>2584161<br>2584162<br>2584163<br>2584164<br>2584165<br>2584166<br>2584167<br>2584168<br>2584169<br>2584170<br>2584171<br>2584172<br>2584173<br>2584174<br>2584175<br>2584176<br>2584177<br>2584178<br>2584179<br>2584180<br>2584181<br>2584182<br>2584183<br>2584184<br>2584185<br>2584186<br>2584187<br>2584188<br>2584189<br>2584190<br>2584191<br>2584192<br>2584193<br>2584194<br>2584195	core<br>full<br>extended<br>extended<br>extended<br>full<br>extended<br>core<br>full<br>core<br>full<br>core<br>full<br>core<br>extended<br>extended<br>extended<br>extended<br>extended<br>extended<br>core<br>core<br>core<br>core<br>core<br>extended<br>core<br>extended<br>core<br>core<br>core<br>full<br>core<br>extended<br>core<br>core<br>full<br>full<br>full						
2585476	167645<br>167646<br>167647<br>167648<br>167649<br>167650<br>167651<br>167652<br>167653<br>167654<br>167655<br>167656<br>167657<br>167658<br>167659	2585477<br>2585478<br>2585479<br>2585480<br>2585481<br>2585482<br>2585483<br>2585484<br>2585485<br>2585486<br>2585487<br>2585488<br>2585489<br>2585490<br>2585491	extended<br>extended<br>core<br>full<br>core<br>core<br>extended<br>core<br>core<br>core<br>extended<br>extended<br>core<br>full<br>core	2	166968343	167104259	-	NM_002976<br>BC062699<br>ENSESTT00000052675<br>ENSESTT00000052676<br>ENSESTT00000052677<br>ENST00000259060<br>GENSCAN00000000539<br>GENSCAN00000018541	Homo sapiens sodium channel, voltage-gated, type VII, alpha (SCN7A), mRNA.<br>Homo sapiens sodium channel, voltage-gated, type VII, alpha, mRNA (cDNA clone IMAGE:4703945).<br><br><br><br>cdna:known chromosome:NCBI36:2:166970135:167051727:-1 gene:ENSG00000136546<br>cdna:Genscan chromosome:NCBI36:2:167058566:167104259:-1<br>cdna:Genscan chromosome:NCBI36:2:166970336:167042498:-1

	167660<br>167661<br>167662<br>167663<br>167664<br>167665<br>167666<br>167667<br>167668<br>167669<br>167670<br>167671<br>167672<br>167673<br>167674<br>167675<br>167676<br>167677<br>167678<br>167679<br>167680<br>167681<br>167682<br>167683<br>167684	2585492<br>2585493<br>2585494<br>2585495<br>2585496<br>2585497<br>2585498<br>2585499<br>2585500<br>2585501<br>2585502<br>2585503<br>2585504<br>2585505<br>2585506<br>2585507<br>2585508<br>2585509<br>2585510<br>2585511<br>2585512<br>2585513<br>2585514<br>2585515<br>2585516<br>2585517<br>2585518<br>2585519<br>2585520<br>2585521<br>2585522<br>2585523<br>2585524<br>2585525<br>2585526<br>2585527<br>2585528<br>2585529<br>2585530<br>2585531<br>2585532<br>2585533<br>2585534<br>2585535<br>2585536<br>2585537<br>2585538<br>2585539	core<br>core<br>core<br>extended<br>core<br>core<br>core<br>extended<br>extended<br>core<br>core<br>extended<br>core<br>core<br>core<br>core<br>core<br>core<br>extended<br>extended<br>extended<br>extended<br>core<br>core<br>core<br>core<br>core<br>core<br>core<br>core<br>core<br>core<br>core<br>full<br>core<br>extended<br>extended<br>extended<br>full<br>extended<br>extended<br>full<br>full<br>full<br>full<br>full<br>full<br>full						
2591643	171666<br>171667<br>171668<br>171669<br>171670<br>171671	2591644<br>2591645<br>2591646<br>2591647<br>2591648<br>2591649	extended<br>extended<br>core<br>core<br>core<br>core	2	189604867	189798263	-	NM_000393<br>BC086874<br>ENSESTT00000025116<br>ENSESTT00000025117<br>ENSESTT00000025118<br>ENSESTT00000025119	Homo sapiens collagen, type V, alpha 2 (COL5A2), mRNA.<br>Homo sapiens collagen, type V, alpha 2, mRNA (cDNA clone MGC:105115 IMAGE:30708812), complete cds.

	171672	2591650	core					ENSESTT00000025120	
	171673	2591651	core					ENSESTT00000025121	
	171674	2591652	core					ENSESTT00000025122	
	171675	2591653	core					ENSESTT00000025123	
	171676	2591654	core					ENST00000360859	cdna:known chromosome:NCBI36:2:189605486:189752733:-1 gene:ENSG00000204262
	171677	2591655	extended					ENST00000374866	cdna:known chromosome:NCBI36:2:189605486:189752712:-1 gene:ENSG00000204262
	171678	2591656	core					GENSCAN00000033723	cdna:Genscan chromosome:NCBI36:2:189607041:189689427:-1
	171679	2591657	extended						
	171680	2591658	core						
	171681	2591659	core						
	171682	2591660	core						
	171683	2591661	core						
	171684	2591662	core						
	171685	2591663	core						
	171686	2591664	core						
	171687	2591665	core						
	171688	2591666	core						
	171689	2591667	core						
	171690	2591668	core						
	171691	2591669	extended						
	171692	2591670	core						
	171693	2591671	extended						
	171694	2591672	core						
	171695	2591673	core						
	171696	2591674	core						
	171697	2591675	core						
	171698	2591676	extended						
	171699	2591677	extended						
	171700	2591678	extended						
	171701	2591679	core						
	171702	2591680	core						
	171703	2591681	core						
	171704	2591682	core						
	171705	2591683	extended						
	171706	2591684	extended						
	171707	2591685	extended						
	171708	2591686	core						
	171709	2591687	extended						
	171710	2591688	core						
	171711	2591689	extended						
	171712	2591690	core						
	171713	2591691	extended						
	171714	2591692	core						
	171715	2591693	core						
	171716	2591694	core						
	171717	2591695	core						
	171718	2591696	extended						
	171719	2591697	full						
	171720	2591698	core						
	171721	2591699	extended						
	171722	2591700	extended						
	171723	2591701	core						
	171724	2591702	core						
	171725	2591703	core						

	171726<br>171727<br>171728<br>171729<br>171730<br>171731<br>171732<br>171733<br>171734<br>171735<br>171736<br>171737<br>171738<br>171739<br>171740<br>171741<br>171742<br>171743<br>171744<br>171745<br>171746<br>171747<br>171748<br>171749<br>171750<br>171751	2591704<br>2591705<br>2591706<br>2591707<br>2591708<br>2591709<br>2591710<br>2591711<br>2591712<br>2591713<br>2591714<br>2591715<br>2591716<br>2591717<br>2591718<br>2591719<br>2591720<br>2591721<br>2591722<br>2591723<br>2591724<br>2591725<br>2591726<br>2591727<br>2591728<br>2591729<br>2591730<br>2591731<br>2591732<br>2591733<br>2591734<br>2591735<br>2591736<br>2591737<br>2591738<br>2591739<br>2591740<br>2591741<br>2591742<br>2591743<br>2591744<br>2591745<br>2591746<br>2591747<br>2591748<br>2591749	core<br>extended<br>extended<br>core<br>core<br>core<br>core<br>core<br>extended<br>core<br>extended<br>core<br>extended<br>core<br>extended<br>core<br>core<br>full<br>core<br>core<br>core<br>extended<br>core<br>core<br>extended<br>full<br>full<br>extended<br>extended<br>extended<br>full<br>extended<br>extended<br>extended<br>extended<br>extended<br>core<br>core<br>core<br>extended<br>full<br>full<br>full<br>full<br>extended<br>extended						
2599153	176493<br>176494<br>176495<br>176496<br>176497<br>176498<br>176499<br>176500	2599154<br>2599155<br>2599156<br>2599157<br>2599158<br>2599159<br>2599160<br>2599161	extended<br>extended<br>core<br>core<br>core<br>core<br>core<br>extended	2	218372767	218606341	-	NM_022648<br>AF225896<br>AK001785<br>AK023812<br>BC107758<br>AF116610<br>BC033065<br>ENSESTT00000005143	Homo sapiens tensin 1 (TNS1), mRNA.<br>Homo sapiens tensin mRNA, complete cds.<br>Homo sapiens cDNA FLJ10923 fis, clone OVARC1000437, moderately similar to TENSIN.<br>Homo sapiens cDNA FLJ13750 fis, clone PLACE3000331.<br>Homo sapiens cDNA clone IMAGE:4254181.<br>Homo sapiens PRO0929 mRNA, complete cds.<br>Homo sapiens tensin 1, mRNA (cDNA clone IMAGE:4869823), with apparent retained intron.

	176501	2599162	core					ENSESTT00000005144	
	176502	2599163	core					ENSESTT00000005145	
	176503	2599164	extended					ENSESTT00000005146	
	176504	2599165	core					ENSESTT00000005147	
	176505	2599166	core					ENSESTT00000005148	
	176506	2599167	extended					ENSESTT00000005149	
	176507	2599168	core					ENST00000171887	cdna:known-ccds chromosome:NCBI36:2:218375757:218517006:-1 gene:ENSG00000079308 CCDS2407.1
	176508	2599169	full					ENST00000310858	cdna:known chromosome:NCBI36:2:218432200:218476781:-1 gene:ENSG00000079308
	176509	2599170	core					GENSCAN00000059944	cdna:Genscan chromosome:NCBI36:2:218377427:218432082:-1
	176510	2599171	extended					GENSCAN00000059940	cdna:Genscan chromosome:NCBI36:2:218445126:218509107:-1
	176511	2599172	extended					GENSCAN00000059936	cdna:Genscan chromosome:NCBI36:2:218518916:218519125:-1
	176512	2599173	core					GENSCAN00000055038	cdna:Genscan chromosome:NCBI36:2:218526073:218606341:-1
	176513	2599174	extended						
	176514	2599175	full						
	176515	2599176	core						
	176516	2599177	core						
	176517	2599178	core						
	176518	2599179	extended						
	176519	2599180	full						
	176520	2599181	extended						
	176521	2599182	full						
	176522	2599183	full						
	176523	2599184	core						
	176524	2599185	core						
	176525	2599186	full						
	176526	2599187	core						
	176527	2599188	core						
	176528	2599189	extended						
	176529	2599190	full						
	176530	2599191	full						
	176531	2599192	full						
	176532	2599193	extended						
	176533	2599194	core						
	176534	2599195	core						
	176535	2599196	core						
	176536	2599197	core						
	176537	2599198	core						
	176538	2599199	core						
	176539	2599200	core						
	176540	2599201	core						
	176541	2599202	core						
	176542	2599203	core						
	176543	2599204	full						
	176544	2599205	extended						
	176545	2599206	core						
	176546	2599207	core						
	176547	2599208	full						
	176548	2599209	extended						
	176549	2599210	extended						
	176550	2599211	extended						
	176551	2599212	core						
	176552	2599213	core						
	176553	2599214	core						
	176554	2599215	extended						

	176555	2599216	core						
	176556	2599217	full						
	176557	2599218	full						
	176558	2599219	core						
	176559	2599220	core						
	176560	2599221	core						
	176561	2599222	core						
	176562	2599223	extended						
	176563	2599224	core						
	176564	2599225	core						
	176565	2599226	core						
	176566	2599227	core						
	176567	2599228	extended						
	176568	2599229	extended						
	176569	2599230	extended						
	176570	2599231	core						
	176571	2599232	core						
	176572	2599233	extended						
	176573	2599234	extended						
	176574	2599235	extended						
	176575	2599236	extended						
	176576	2599237	extended						
	176577	2599238	extended						
	176578	2599239	extended						
	176579	2599240	core						
	176580	2599241	extended						
	176581	2599242	extended						
	176582	2599243	extended						
	176583	2599244	full						
	176584	2599245	core						
	176585	2599246	core						
	176586	2599247	full						
	176587	2599248	full						
	176588	2599249	core						
	176589	2599250	core						
	176590	2599251	full						
	176591	2599252	full						
	176592	2599253	full						
		2599254	full						
		2599255	core						
		2599256	extended						
		2599257	full						
		2599258	full						
		2599259	extended						
		2599260	full						
		2599261	core						
		2599262	full						
		2599263	full						
		2599264	full						
		2599265	full						
		2599266	full						
		2599267	full						
		2599268	full						
		2599269	full						

		2599270<br>2599271<br>2599272<br>2599273<br>2599274<br>2599275<br>2599276<br>2599277<br>2599278<br>2599279<br>2599280<br>2599281<br>2599282<br>2599283<br>2599284<br>2599285<br>2599286<br>2599287<br>2599288	full<br>full<br>full<br>extended<br>full<br>extended<br>full<br>full<br>full<br>full<br>full<br>full<br>full<br>full<br>full<br>full<br>full<br>full<br>full						
2600218	177129<br>177130<br>177131<br>177132<br>177133<br>177134	2600219<br>2600220<br>2600221<br>2600222<br>2600223<br>2600224<br>2600225<br>2600226<br>2600227<br>2600228<br>2600229<br>2600230	extended<br>core<br>core<br>core<br>core<br>core<br>core<br>extended<br>core<br>extended<br>core<br>extended	2	220111746	220117247	-	NM_024536<br>BC008878<br>CR625437<br>ENSESTT00000029865<br>ENST00000373891<br>ENST00000243776<br>GENSCAN00000050943	Homo sapiens chondroitin polymerizing factor (CHPF), mRNA.<br>Homo sapiens chondroitin polymerizing factor, mRNA (cDNA clone IMAGE:4298587), partial cds.<br>full-length cDNA clone CS0DK004YN21 of HeLa cells Cot 25-normalized of Homo sapiens (human).<br><br>cdna:known-ccds chromosome:NCBI36:2:220111913:220116753:-1 gene:ENSG00000123989 CCDS2443.1<br>cdna:known chromosome:NCBI36:2:220111914:220116740:-1 gene:ENSG00000123989<br>cdna:Genscan chromosome:NCBI36:2:220112349:220116504:-1
2604254	179728<br>179729<br>179730<br>179731<br>179732<br>179733<br>179734<br>179735<br>179736<br>179737<br>179738<br>179739<br>179740<br>179741<br>179742<br>179743<br>179744<br>179745	2604255<br>2604256<br>2604257<br>2604258<br>2604259<br>2604260<br>2604261<br>2604262<br>2604263<br>2604264<br>2604265<br>2604266<br>2604267<br>2604268<br>2604269<br>2604270<br>2604271<br>2604272<br>2604273<br>2604274<br>2604275<br>2604276	extended<br>full<br>extended<br>core<br>core<br>core<br>core<br>full<br>core<br>core<br>core<br>core<br>core<br>core<br>core<br>core<br>extended<br>extended<br>core<br>core<br>extended<br>core	2	234406876	234427931	-	NM_018410<br>ENSESTT00000035627<br>ENSESTT00000035628<br>ENST00000373395<br>ENST00000243201<br>GENSCAN00000015883<br>GENSCAN00000015881	Homo sapiens hypothetical protein DKFZp762E1312 (DKFZp762E1312), mRNA.<br><br><br>cdna:known chromosome:NCBI36:2:234410225:234427951:-1 gene:ENSG00000123485<br>cdna:known chromosome:NCBI36:2:234410746:234427917:-1 gene:ENSG00000123485<br>cdna:Genscan chromosome:NCBI36:2:234412902:234415492:-1<br>cdna:Genscan chromosome:NCBI36:2:234427149:234427864:-1

		2604277<br>2604278<br>2604279<br>2604280	full<br>core<br>core<br>extended						
2605321	180399<br>180400<br>180401<br>180402<br>180403<br>180404<br>180405<br>180406<br>180407<br>180408<br>180409<br>180410<br>180411<br>180412<br>180413<br>180414<br>180415<br>180416<br>180417<br>180418<br>180419<br>180420<br>180421<br>180422<br>180423<br>180424<br>180425<br>180426<br>180427<br>180428<br>180429<br>180430<br>180431<br>180432<br>180433<br>180434<br>180435<br>180436<br>180437<br>180438<br>180439<br>180440<br>180441<br>180442<br>180443<br>180444<br>180445<br>180446<br>180447<br>180448	2605322<br>2605323<br>2605324<br>2605325<br>2605326<br>2605327<br>2605328<br>2605329<br>2605330<br>2605331<br>2605332<br>2605333<br>2605334<br>2605335<br>2605336<br>2605337<br>2605338<br>2605339<br>2605340<br>2605341<br>2605342<br>2605343<br>2605344<br>2605345<br>2605346<br>2605347<br>2605348<br>2605349<br>2605350<br>2605351<br>2605352<br>2605353<br>2605354<br>2605355<br>2605356<br>2605357<br>2605358<br>2605359<br>2605360<br>2605361<br>2605362<br>2605363<br>2605364<br>2605365<br>2605366<br>2605367<br>2605368<br>2605369<br>2605370<br>2605371	full<br>full<br>full<br>core<br>core<br>core<br>core<br>core<br>core<br>extended<br>extended<br>core<br>core<br>core<br>core<br>core<br>core<br>core<br>core<br>full<br>core<br>extended<br>extended<br>extended<br>core<br>core<br>extended<br>full<br>core<br>core<br>full<br>core<br>core<br>core<br>core<br>full<br>core<br>core<br>extended<br>extended<br>extended<br>extended<br>core<br>extended<br>core<br>core<br>core<br>core<br>core<br>core	2	237897089	238019668	-	NM_057164<br>NM_004369<br>NM_057165<br>NM_057167<br>NM_057166<br>BX641155<br>BX647361<br>S49432<br>BX647500<br>ENSESTT00000018422<br>ENSESTT00000018423<br>ENSESTT00000018424<br>ENSESTT00000018425<br>ENSESTT00000018426<br>ENSESTT00000018427<br>ENSESTT00000018428<br>ENSESTT00000018429<br>ENST00000295550<br>ENST00000346358<br>ENST00000347401<br>ENST00000353578<br>GENSCAN00000024616	Homo sapiens collagen, type VI, alpha 3 (COL6A3), transcript variant 2, mRNA.<br>Homo sapiens collagen, type VI, alpha 3 (COL6A3), transcript variant 1, mRNA.<br>Homo sapiens collagen, type VI, alpha 3 (COL6A3), transcript variant 3, mRNA.<br>Homo sapiens collagen, type VI, alpha 3 (COL6A3), transcript variant 5, mRNA.<br>Homo sapiens collagen, type VI, alpha 3 (COL6A3), transcript variant 4, mRNA.<br>Homo sapiens mRNA; cDNA DKFZp686D23123 (from clone DKFZp686D23123).<br>Homo sapiens mRNA; cDNA DKFZp686N0262 (from clone DKFZp686N0262).<br>type VI collagen alpha 3 chain {5' region, alternatively spliced, exon A9/N10} [human, mRNA, 653 nt].<br>Homo sapiens mRNA; cDNA DKFZp686K04147 (from clone DKFZp686K04147).<br><br><br><br><br><br><br><br><br>cdna:known chromosome:NCBI36:2:237897401:237987559:-1 gene:ENSG00000163359<br>cdna:known chromosome:NCBI36:2:237897401:237987559:-1 gene:ENSG00000163359<br>cdna:known chromosome:NCBI36:2:237897401:237987559:-1 gene:ENSG00000163359<br>cdna:known chromosome:NCBI36:2:237897401:237987559:-1 gene:ENSG00000163359<br>cdna:Genscan chromosome:NCBI36:2:237914246:238019586:-1

	180449<br>180450<br>180451<br>180452<br>180453<br>180454<br>180455<br>180456<br>180457<br>180458<br>180459<br>180460<br>180461<br>180462<br>180463<br>180464<br>180465<br>180466<br>180467<br>180468<br>180469	2605372<br>2605373<br>2605374<br>2605375<br>2605376<br>2605377<br>2605378<br>2605379<br>2605380<br>2605381<br>2605382<br>2605383<br>2605384<br>2605385<br>2605386<br>2605387<br>2605388<br>2605389<br>2605390<br>2605391<br>2605392<br>2605393<br>2605394<br>2605395<br>2605396<br>2605397<br>2605398<br>2605399<br>2605400<br>2605401<br>2605402<br>2605403<br>2605404<br>2605405<br>2605406<br>2605407<br>2605408<br>2605409<br>2605410<br>2605411<br>2605412<br>2605413<br>2605414<br>2605415<br>2605416<br>2605417<br>2605418<br>2605419	full<br>core<br>core<br>core<br>core<br>core<br>core<br>core<br>core<br>extended<br>core<br>core<br>core<br>core<br>core<br>core<br>extended<br>extended<br>core<br>core<br>full<br>extended<br>full<br>core<br>core<br>extended<br>core<br>core<br>core<br>full<br>extended<br>extended<br>extended<br>extended<br>extended<br>extended<br>core<br>full<br>extended<br>full<br>full<br>full<br>full<br>full<br>full<br>full<br>full<br>full						
2609347	183004<br>183005<br>183006<br>183007<br>183008<br>183009	2609336<br>2609338<br>2609340<br>2609342<br>2609344<br>2609346	full<br>free<br>free<br>free<br>free<br>free	3	8400652	8585276	+	NM_014583<br>AK074448<br>ENSESTT00000013540<br>ENSESTT00000013541<br>ENSESTT00000013542<br>ENSESTT00000013543	Homo sapiens LIM and cysteine-rich domains 1 (LMCD1), mRNA.<br>Homo sapiens cDNA FLJ23868 fis, clone LNG09855.

	183010	2609348	full					ENST00000157600	cdna:known chromosome:NCBI36:3:8518511:8584805:1 gene:ENSG00000071282
	183011	2609349	full					GENSCAN00000017097	cdna:Genscan chromosome:NCBI36:3:8518322:8584284:1
	183012	2609350	full						
	183013	2609351	full						
	183014	2609352	full						
	183015	2609353	full						
	183016	2609354	full						
	183017	2609355	full						
	183018	2609356	extended						
	183019	2609357	full						
	183020	2609358	full						
	183021	2609359	full						
	183022	2609360	full						
	183023	2609361	full						
	183024	2609362	full						
	183025	2609363	full						
	183026	2609364	full						
	183027	2609365	full						
	183028	2609366	full						
	183029	2609367	full						
	183030	2609368	core						
	183031	2609369	core						
	183032	2609370	core						
	183033	2609371	full						
	183034	2609372	full						
	183035	2609373	full						
	183036	2609374	full						
	183037	2609375	full						
	183038	2609376	extended						
	183039	2609377	extended						
	183040	2609378	extended						
	183041	2609379	extended						
	183042	2609380	full						
	183043	2609381	extended						
	183044	2609382	extended						
	183045	2609383	extended						
	183046	2609384	full						
	183047	2609385	core						
	183048	2609386	full						
	183049	2609387	full						
	183050	2609388	full						
	183051	2609389	full						
		2609390	core						
		2609391	extended						
		2609392	extended						
		2609393	core						
		2609394	core						
		2609395	core						
		2609396	core						
		2609397	extended						
		2609398	extended						
		2609399	extended						
		2609400	full						
		2609401	full						

		2609402<br>2609403<br>2609404<br>2609405<br>2609406<br>2609407<br>2609408<br>2609409<br>2609410<br>2609411	extended<br>full<br>extended<br>extended<br>extended<br>core<br>extended<br>core<br>core<br>extended						
2610241	183499<br>183500<br>183501<br>183502<br>183503<br>183504<br>183505<br>183506<br>183507<br>183508<br>183509<br>183510<br>183511<br>183512<br>183513<br>183514<br>183515<br>183516<br>183517<br>183518<br>183519<br>183520<br>183521<br>183522<br>183523<br>183524<br>183525<br>183526<br>183527<br>183528<br>183529<br>183530<br>183531<br>183532<br>183533<br>183534<br>183535<br>183536<br>183537<br>183538<br>183539<br>183540<br>183541<br>183542	2610242<br>2610243<br>2610244<br>2610245<br>2610246<br>2610247<br>2610248<br>2610249<br>2610250<br>2610251<br>2610252<br>2610253<br>2610254<br>2610255<br>2610256<br>2610257<br>2610258<br>2610259<br>2610260<br>2610261<br>2610262<br>2610263<br>2610264<br>2610265<br>2610266<br>2610267<br>2610268<br>2610269<br>2610270<br>2610271<br>2610272<br>2610273<br>2610274<br>2610275<br>2610276<br>2610277<br>2610278<br>2610279<br>2610280<br>2610281<br>2610282<br>2610283<br>2610284<br>2610285	full<br>extended<br>full<br>core<br>core<br>core<br>core<br>full<br>core<br>core<br>core<br>full<br>core<br>core<br>core<br>core<br>core<br>core<br>core<br>core<br>full<br>core<br>core<br>core<br>core<br>core<br>core<br>core<br>extended<br>core<br>core<br>core<br>core<br>core<br>extended<br>core<br>core<br>extended<br>core<br>core<br>full<br>core<br>core<br>core	3	10043080	10120436	+	NM_001018115<br>NM_033084<br>BC013582<br>ENSESTT00000022914<br>ENSESTT00000022915<br>ENSESTT00000022916<br>ENSESTT00000022917<br>ENSESTT00000022918<br>ENSESTT00000022919<br>ENST00000287647<br>ENST00000383807<br>ENST00000383806	Homo sapiens Fanconi anemia, complementation group D2 (FANCD2), transcript variant 2, mRNA.<br>Homo sapiens Fanconi anemia, complementation group D2 (FANCD2), transcript variant 1, mRNA.<br>Homo sapiens Fanconi anemia, complementation group D2, mRNA (cDNA clone IMAGE:3876190), complete cds.<br><br><br><br><br><br><br>cdna:known-ccds chromosome:NCBI36:3:10043113:10116344:1 gene:ENSG00000144554 CCDS2595.1<br>cdna:known chromosome:NCBI36:3:10043113:10118614:1 gene:ENSG00000144554<br>cdna:known chromosome:NCBI36:3:10043113:10118614:1 gene:ENSG00000144554

	183543<br>183544<br>183545<br>183546<br>183547<br>183548<br>183549<br>183550<br>183551<br>183552<br>183553<br>183554<br>183555	2610286<br>2610287<br>2610288<br>2610289<br>2610290<br>2610291<br>2610292<br>2610293<br>2610294<br>2610295<br>2610296<br>2610297<br>2610298<br>2610299<br>2610300<br>2610301<br>2610302<br>2610303<br>2610304<br>2610305<br>2610306<br>2610307<br>2610308<br>2610309<br>2610310<br>2610311<br>2610312<br>2610313<br>2610314<br>2610315<br>2610316	core<br>core<br>core<br>core<br>core<br>core<br>core<br>core<br>core<br>full<br>core<br>core<br>core<br>core<br>extended<br>core<br>extended<br>core<br>core<br>core<br>extended<br>core<br>core<br>core<br>core<br>core<br>extended<br>extended<br>extended<br>extended<br>extended						
2611848	184517<br>184518<br>184519<br>184520<br>184521<br>184522<br>184523<br>184524<br>184525<br>184526<br>184527<br>184528<br>184529<br>184530<br>184531<br>184532<br>184533<br>184534<br>184535<br>184536<br>184537<br>184538<br>184539	2611849<br>2611850<br>2611851<br>2611852<br>2611853<br>2611854<br>2611855<br>2611856<br>2611857<br>2611858<br>2611859<br>2611860<br>2611861<br>2611862<br>2611863<br>2611864<br>2611865<br>2611866<br>2611867<br>2611868<br>2611869<br>2611870<br>2611871	full<br>full<br>extended<br>full<br>full<br>full<br>full<br>full<br>full<br>full<br>full<br>full<br>full<br>full<br>full<br>extended<br>extended<br>full<br>full<br>full<br>full<br>full<br>full	3	14249186	14505858	+	NM_003043<br>AK023516<br>BC038790<br>BC111489<br>U16120<br>ENSESTT00000045726<br>ENSESTT00000045727<br>ENSESTT00000045728<br>ENST00000253707<br>ENST00000360861<br>ENST00000388984<br>ENST00000388983<br>GENSCAN00000039280	Homo sapiens solute carrier family 6 (neurotransmitter transporter, taurine), member 6 (SLC6A6), mRNA.<br>Homo sapiens cDNA FLJ13454 fis, clone PLACE1003249.<br>Homo sapiens cDNA clone IMAGE:5271875.<br>Homo sapiens solute carrier family 6 (neurotransmitter transporter, taurine), member 6, mRNA (cDNA clone IMAGE:5755891), complete cds.<br>Human placental taurine transporter mRNA, complete cds.<br><br><br><br>cdna:known chromosome:NCBI36:3:14419110:14503971:1 gene:ENSG00000131389<br>cdna:known chromosome:NCBI36:3:14419154:14505859:1 gene:ENSG00000131389<br>cdna:known chromosome:NCBI36:3:14460147:14501519:1 gene:ENSG00000131389<br>cdna:known chromosome:NCBI36:3:14460147:14501519:1 gene:ENSG00000131389<br>cdna:Genscan chromosome:NCBI36:3:14198139:14263413:1

	184540	2611872	full						
	184541	2611873	full						
	184542	2611874	full						
	184543	2611875	full						
	184544	2611876	full						
	184545	2611877	full						
	184546	2611878	full						
	184547	2611879	full						
	184548	2611880	full						
	184549	2611881	extended						
	184550	2611882	extended						
	184551	2611883	full						
	184552	2611884	extended						
	184553	2611885	extended						
	184554	2611886	full						
	184555	2611887	full						
	184556	2611888	full						
	184557	2611889	extended						
	184558	2611890	full						
	184559	2611891	core						
	184560	2611892	full						
	184561	2611893	full						
	184562	2611894	extended						
	184563	2611895	extended						
	184564	2611896	extended						
	184565	2611897	extended						
	184566	2611898	extended						
	184567	2611899	extended						
	184568	2611900	extended						
	184569	2611901	extended						
	184570	2611902	full						
	184571	2611903	full						
	184572	2611904	full						
	184573	2611905	full						
	184574	2611906	full						
	184575	2611907	full						
	184576	2611908	full						
	184577	2611909	full						
	184578	2611910	extended						
	184579	2611911	extended						
	184580	2611912	extended						
	184581	2611913	full						
	184582	2611914	full						
	184583	2611915	full						
	184584	2611916	full						
	184585	2611917	core						
	184586	2611918	extended						
	184587	2611919	core						
	184588	2611920	extended						
	184589	2611921	core						
	184590	2611922	extended						
	184591	2611923	extended						
	184592	2611924	extended						
	184593	2611925	full						

	184594<br>184595<br>184596<br>184597<br>184598<br>184599<br>184600	2611926<br>2611927<br>2611928<br>2611929<br>2611930<br>2611931<br>2611932<br>2611933<br>2611934<br>2611935<br>2611936<br>2611937<br>2611938<br>2611939<br>2611940<br>2611941<br>2611942<br>2611943<br>2611944<br>2611945<br>2611946	core<br>full<br>full<br>extended<br>extended<br>extended<br>extended<br>core<br>core<br>full<br>core<br>core<br>core<br>full<br>core<br>core<br>core<br>full<br>extended<br>core<br>extended						
2624074	192044<br>192045<br>192046<br>192047<br>192048<br>192049<br>192050<br>192051<br>192052<br>192053<br>192054<br>192055<br>192056<br>192057<br>192058<br>192059<br>192060<br>192061<br>192062<br>192063<br>192064	2624075<br>2624076<br>2624077<br>2624078<br>2624079<br>2624080<br>2624081<br>2624082<br>2624083<br>2624084<br>2624085<br>2624086<br>2624087<br>2624088<br>2624089<br>2624090<br>2624091<br>2624092<br>2624093<br>2624094<br>2624095<br>2624096<br>2624097<br>2624098<br>2624099<br>2624100<br>2624101<br>2624102<br>2624103<br>2624104<br>2624105<br>2624106<br>2624107	core<br>core<br>core<br>core<br>extended<br>extended<br>core<br>core<br>core<br>core<br>extended<br>extended<br>core<br>extended<br>extended<br>extended<br>core<br>extended<br>core<br>extended<br>core<br>core<br>extended<br>core<br>extended<br>core<br>extended<br>core<br>extended<br>core<br>extended<br>core<br>core	3	52694976	52705212	+	NM_206826<br>NM_206825<br>NM_014366<br>CR598596<br>ENSESTT00000004457<br>ENSESTT00000004458<br>ENSESTT00000004459<br>ENSESTT00000004460<br>ENSESTT00000004461<br>ENSESTT00000004462<br>ENSESTT00000004463<br>ENST00000354540<br>GENSCAN00000069012	Homo sapiens guanine nucleotide binding protein-like 3 (nucleolar) (GNL3), transcript variant 3, mRNA.<br>Homo sapiens guanine nucleotide binding protein-like 3 (nucleolar) (GNL3), transcript variant 2, mRNA.<br>Homo sapiens guanine nucleotide binding protein-like 3 (nucleolar) (GNL3), transcript variant 1, mRNA.<br>full-length cDNA clone CS0DJ002YB18 of T cells (Jurkat cell line) Cot 10-normalized of Homo sapiens (human).<br><br><br><br><br><br><br><br>cdna:known-ccds chromosome:NCBI36:3:52695149:52703548:1 gene:ENSG00000163938 CCDS2861.1<br>cdna:Genscan chromosome:NCBI36:3:52695809:52703331:1

		2624108<br>2624109	extended<br>extended						
2625793	193063<br>193064<br>193065<br>193066<br>193067<br>193068<br>193069<br>193070<br>193071<br>193072<br>193073<br>193074<br>193075<br>193076<br>193077<br>193078<br>193079<br>193080<br>193081<br>193082<br>193083<br>193084<br>193085<br>193086<br>193087<br>193088<br>193089<br>193090<br>193091<br>193092<br>193093<br>193094<br>193095<br>193096<br>193097<br>193098<br>193099<br>193100<br>193101<br>193102<br>193103<br>193104<br>193105<br>193106<br>193107<br>193108<br>193109<br>193110<br>193111<br>193112<br>193113<br>193114	2625794<br>2625795<br>2625796<br>2625797<br>2625798<br>2625799<br>2625800<br>2625801<br>2625802<br>2625803<br>2625804<br>2625805<br>2625806<br>2625807<br>2625808<br>2625809<br>2625810<br>2625811<br>2625812<br>2625813<br>2625814<br>2625815<br>2625816<br>2625817<br>2625818<br>2625819<br>2625820<br>2625821<br>2625822<br>2625823<br>2625824<br>2625825<br>2625826<br>2625827<br>2625828<br>2625829<br>2625830<br>2625831<br>2625832<br>2625833<br>2625834<br>2625835<br>2625836<br>2625837<br>2625838<br>2625839<br>2625840<br>2625841<br>2625842<br>2625843<br>2625844<br>2625845	full<br>extended<br>extended<br>core<br>core<br>core<br>extended<br>full<br>extended<br>extended<br>extended<br>full<br>extended<br>extended<br>full<br>extended<br>extended<br>full<br>full<br>full<br>core<br>extended<br>extended<br>extended<br>full<br>core<br>core<br>core<br>extended<br>extended<br>core<br>core<br>core<br>extended<br>core<br>core<br>core<br>core<br>extended<br>extended<br>extended<br>extended<br>extended<br>extended<br>extended<br>extended<br>extended<br>core<br>core<br>core<br>extended<br>extended	3	57716908	57933826	+	NM_007159<br>AF304450<br>AK022561<br>AK124200<br>AY358410<br>BC029462<br>CR627321<br>AF100750<br>ENSESTT00000002082<br>ENSESTT00000002083<br>ENSESTT00000002084<br>ENSESTT00000002085<br>ENSESTT00000002086<br>ENSESTT00000038019<br>ENSESTT00000038020<br>ENSESTT00000038021<br>ENST00000383719<br>ENST00000295952<br>ENST00000383718<br>ENST00000295951<br>ENST00000383717<br>GENSCAN00000032567<br>GENSCAN00000032566<br>GENSCAN00000032562	Homo sapiens sarcolemma associated protein (SLMAP), mRNA.<br>Homo sapiens sarcolemmal associated protein 1 mRNA, complete cds, alternatively spliced.<br>Homo sapiens cDNA FLJ12499 fis, clone NT2RM2001671, highly similar to Oryctolagus cuniculus sarcolemmal associated protein (SLAP1) mRNA.<br>Homo sapiens cDNA FLJ42206 fis, clone THYMU2035735, highly similar to Oryctolagus cuniculus sarcolemmal associated protein-3 mRNA.<br>Homo sapiens clone DNA53991 SLAP (UNQ1847) mRNA, complete cds.<br>Homo sapiens, clone IMAGE:4663772, mRNA.<br>Homo sapiens mRNA; cDNA DKFZp779I1058 (from clone DKFZp779I1058).<br>Homo sapiens SLAP-2 homolog mRNA, complete cds.<br><br><br><br><br><br><br><br><br>cdna:known chromosome:NCBI36:3:57718068:57851973:1 gene:ENSG00000163681<br>cdna:known chromosome:NCBI36:3:57718214:57889934:1 gene:ENSG00000163681<br>cdna:known chromosome:NCBI36:3:57718321:57858460:1 gene:ENSG00000163681<br>cdna:known chromosome:NCBI36:3:57825353:57888326:1 gene:ENSG00000163681<br>cdna:known chromosome:NCBI36:3:57850808:57888160:1 gene:ENSG00000163681<br>cdna:Genscan chromosome:NCBI36:3:57873114:57888155:1<br>cdna:Genscan chromosome:NCBI36:3:57818521:57869943:1<br>cdna:Genscan chromosome:NCBI36:3:57780491:57798832:1

	193115	2625846	extended						
	193116	2625847	extended						
	193117	2625848	core						
	193118	2625849	extended						
	193119	2625850	core						
	193120	2625851	extended						
	193121	2625852	extended						
	193122	2625853	extended						
	193123	2625854	extended						
	193124	2625855	extended						
	193125	2625856	extended						
	193126	2625857	full						
	193127	2625858	core						
	193128	2625859	core						
	193129	2625860	full						
	193130	2625861	extended						
	193131	2625862	extended						
	193132	2625863	extended						
	193133	2625864	extended						
	193134	2625865	core						
	193135	2625866	core						
	193136	2625867	extended						
	193137	2625868	extended						
	193138	2625869	extended						
	193139	2625870	extended						
	193140	2625871	full						
	193141	2625872	core						
	193142	2625873	full						
	193143	2625874	extended						
	193144	2625875	full						
	193145	2625876	extended						
		2625877	core						
		2625878	extended						
		2625879	extended						
		2625880	extended						
		2625881	extended						
		2625882	extended						
		2625883	extended						
		2625884	extended						
		2625885	core						
		2625886	extended						
		2625887	extended						
		2625888	extended						
		2625889	core						
		2625890	core						
		2625891	extended						
		2625892	extended						
		2625893	extended						
		2625894	extended						
		2625895	extended						
		2625896	extended						
		2625897	full						
		2625898	extended						
		2625899	extended						

		2625900<br>2625901<br>2625902<br>2625903<br>2625904	extended<br>full<br>full<br>full<br>full						
2633390	197872<br>197873<br>197874<br>197875<br>197876<br>197877<br>197878<br>197879<br>197880<br>197881<br>197882<br>197883<br>197884<br>197885<br>197886<br>197887<br>197888<br>197889<br>197890<br>197891<br>197892<br>197893<br>197894<br>197895<br>197896<br>197897<br>197898<br>197899<br>197900<br>197901<br>197902<br>197903<br>197904<br>197905<br>197906<br>197907<br>197908<br>197909<br>197910<br>197911	2633391<br>2633392<br>2633393<br>2633394<br>2633395<br>2633396<br>2633397<br>2633398<br>2633399<br>2633400<br>2633401<br>2633402<br>2633403<br>2633404<br>2633405<br>2633406<br>2633407<br>2633408<br>2633409<br>2633410<br>2633411<br>2633412<br>2633413<br>2633414<br>2633415<br>2633416<br>2633417<br>2633418<br>2633419<br>2633420<br>2633421<br>2633422<br>2633423<br>2633424<br>2633425<br>2633426<br>2633427<br>2633428<br>2633429<br>2633430<br>2633431<br>2633432<br>2633433<br>2633434<br>2633435<br>2633436<br>2633437<br>2633438<br>2633439	full<br>full<br>full<br>full<br>full<br>full<br>full<br>full<br>full<br>extended<br>extended<br>free<br>extended<br>full<br>full<br>extended<br>extended<br>extended<br>extended<br>core<br>extended<br>core<br>core<br>core<br>core<br>core<br>core<br>core<br>core<br>core<br>extended<br>full<br>extended<br>extended<br>extended<br>extended<br>extended<br>full<br>full<br>full<br>full<br>core<br>full<br>extended<br>full<br>full<br>full<br>full<br>full	3	100750103	101000750	+	XM_936997<br>NM_020351<br>XM_927041<br>NM_001850<br>AL359062<br>ENSESTT00000001517<br>ENSESTT00000001519<br>ENSESTT00000001521<br>ENST00000261037<br>ENST00000273342<br>GENSCAN00000046581	PREDICTED: Homo sapiens similar to Smooth muscle cell-expressed and macrophage conditioned medium-induced protein 64 (Smag-64) (LOC653932), mRNA.<br>Homo sapiens collagen, type VIII, alpha 1 (COL8A1), transcript variant 2, mRNA.<br>PREDICTED: Homo sapiens similar to Smooth muscle cell-expressed and macrophage conditioned medium-induced protein 64 (Smag-64) (LOC653260), mRNA.<br>Homo sapiens collagen, type VIII, alpha 1 (COL8A1), transcript variant 1, mRNA.<br>Homo sapiens mRNA full length insert cDNA clone EUROIMAGE 1913076.<br><br><br><br>cdna:known-ccds chromosome:NCBI36:3:100840144:100997848:1 gene:ENSG00000144810 CCDS2934.1<br>cdna:known chromosome:NCBI36:3:100840145:100881996:1 gene:ENSG00000144810<br>cdna:Genscan chromosome:NCBI36:3:100992217:101030590:1

		2633440<br>2633441<br>2633442<br>2633443<br>2633444<br>2633445<br>2633446<br>2633447<br>2633448	core<br>core<br>core<br>core<br>core<br>core<br>core<br>extended<br>extended						
2650199	208523<br>208524<br>208525<br>208526<br>208527<br>208528<br>208529<br>208530<br>208531<br>208532<br>208533<br>208534<br>208535<br>208536<br>208537<br>208538<br>208539<br>208540<br>208541<br>208542<br>208543<br>208544<br>208545<br>208546<br>208547<br>208548<br>208549<br>208550<br>208551<br>208552<br>208553<br>208554<br>208555<br>208556<br>208557<br>208558<br>208559<br>208560<br>208561	2650200<br>2650201<br>2650202<br>2650203<br>2650204<br>2650205<br>2650206<br>2650207<br>2650208<br>2650209<br>2650210<br>2650211<br>2650212<br>2650213<br>2650214<br>2650215<br>2650216<br>2650217<br>2650218<br>2650219<br>2650220<br>2650221<br>2650222<br>2650223<br>2650224<br>2650225<br>2650226<br>2650227<br>2650228<br>2650229<br>2650230<br>2650231<br>2650232<br>2650233<br>2650234<br>2650235<br>2650236<br>2650237<br>2650238<br>2650239<br>2650240<br>2650241<br>2650242<br>2650243<br>2650244	full<br>full<br>full<br>full<br>full<br>full<br>core<br>core<br>core<br>core<br>core<br>core<br>core<br>core<br>extended<br>core<br>core<br>core<br>extended<br>core<br>core<br>extended<br>extended<br>extended<br>full<br>extended<br>extended<br>core<br>core<br>core<br>extended<br>extended<br>core<br>extended<br>extended<br>core<br>extended<br>core<br>extended<br>extended<br>core<br>extended<br>core<br>extended<br>core	3	161597184	161635431	+	NM_001002799<br>NM_005496<br>NM_001002800<br>AF480552<br>AK002200<br>AK122939<br>AL833949<br>ENSESTT00000013699<br>ENSESTT00000013700<br>ENSESTT00000013701<br>ENSESTT00000013702<br>ENSESTT00000013703<br>ENSESTT00000013704<br>ENSESTT00000013705<br>ENST00000357388<br>ENST00000360111<br>ENST00000344722<br>GENSCAN00000041693	Homo sapiens structural maintenance of chromosomes 4 (SMC4), transcript variant 3, mRNA.<br>Homo sapiens structural maintenance of chromosomes 4 (SMC4), transcript variant 1, mRNA.<br>Homo sapiens structural maintenance of chromosomes 4 (SMC4), transcript variant 2, mRNA.<br>Homo sapiens miR-16-precursor-3 micro RNA, complete sequence.<br>Homo sapiens cDNA FLJ11338 fis, clone PLACE1010720, highly similar to Homo sapiens mRNA for chromosome-associated polypeptide-C.<br>Homo sapiens cDNA FLJ16648 fis, clone TESTI4035508, highly similar to Homo sapiens SMC4 structural maintenance of chromosomes 4-like 1 (yeast) (SMC4L1).<br>Homo sapiens mRNA; cDNA DKFZp434N2050 (from clone DKFZp434N2050).<br>cdna:known-ccds chromosome:NCBI36:3:161600124:161635433:1 gene:ENSG00000113810 CCDS3189.1<br>cdna:known chromosome:NCBI36:3:161600124:161635433:1 gene:ENSG00000113810<br>cdna:known chromosome:NCBI36:3:161600124:161635433:1 gene:ENSG00000113810<br>cdna:Genscan chromosome:NCBI36:3:161597184:161634291:1

		2650245<br>2650246<br>2650247<br>2650248<br>2650249<br>2650250<br>2650251<br>2650252<br>2650253<br>2650254<br>2650255<br>2650256<br>2650257<br>2650258<br>2650259<br>2650260<br>2650261<br>2650262<br>2650263<br>2650264	core<br>core<br>core<br>core<br>core<br>extended<br>extended<br>core<br>extended<br>core<br>core<br>core<br>full<br>core<br>core<br>extended<br>core<br>core<br>core<br>core						
2652675	210065<br>210066<br>210067<br>210068<br>210069<br>210070<br>210071<br>210072<br>210073<br>210074<br>210075<br>210076<br>210077<br>210078<br>210079<br>210080<br>210081<br>210082<br>210083<br>210084<br>210085<br>210086<br>210087<br>210088<br>210089<br>210090<br>210091<br>210092<br>210093<br>210094<br>210095<br>210096<br>210097<br>210098	2652676<br>2652677<br>2652678<br>2652679<br>2652680<br>2652681<br>2652682<br>2652683<br>2652684<br>2652685<br>2652686<br>2652687<br>2652688<br>2652689<br>2652690<br>2652691<br>2652692<br>2652693<br>2652694<br>2652695<br>2652696<br>2652697<br>2652698<br>2652699<br>2652700<br>2652701<br>2652702<br>2652703<br>2652704<br>2652705<br>2652706<br>2652707<br>2652708<br>2652709	full<br>extended<br>extended<br>core<br>core<br>core<br>core<br>extended<br>extended<br>core<br>core<br>core<br>extended<br>core<br>core<br>core<br>full<br>core<br>core<br>core<br>core<br>core<br>core<br>core<br>extended<br>core<br>core<br>core<br>core<br>core<br>extended<br>core<br>core<br>extended	3	173950943	174077327	+	NM_018098<br>DQ847274<br>AL137710<br>ENSESTT00000046493<br>ENSESTT00000046494<br>ENSESTT00000046495<br>ENSESTT00000046496<br>ENSESTT00000046497<br>ENSESTT00000046498<br>ENSESTT00000046499<br>ENSESTT00000046500<br>ENST00000232458<br>GENSCAN00000045148<br>GENSCAN00000044983	Homo sapiens epithelial cell transforming sequence 2 oncogene (ECT2), mRNA.<br>Homo sapiens epithelial cell transforming sequence 2 oncogene protein splice variant b (ECT2) mRNA, complete cds, alternatively spliced.<br>Homo sapiens mRNA; cDNA DKFZp434C0523 (from clone DKFZp434C0523); partial cds.<br>cdna:known-ccds chromosome:NCBI36:3:173951207:174021957:1 gene:ENSG00000114346 CCDS3220.1<br>cdna:Genscan chromosome:NCBI36:3:174043737:174077327:1<br>cdna:Genscan chromosome:NCBI36:3:173955784:174019253:1

	210099<br>210100<br>210101<br>210102<br>210103<br>210104<br>210105<br>210106<br>210107<br>210108<br>210109<br>210110<br>210111	2652710<br>2652711<br>2652712<br>2652713<br>2652714<br>2652715<br>2652716<br>2652717<br>2652718<br>2652719<br>2652720<br>2652721<br>2652722<br>2652723<br>2652724<br>2652725<br>2652726<br>2652727<br>2652728<br>2652729<br>2652730<br>2652731<br>2652732<br>2652733<br>2652734<br>2652735<br>2652736<br>2652737<br>2652738<br>2652739	full<br>extended<br>core<br>extended<br>core<br>core<br>extended<br>extended<br>extended<br>extended<br>extended<br>core<br>core<br>core<br>extended<br>extended<br>extended<br>extended<br>full<br>full<br>full<br>full<br>full<br>full<br>full<br>full<br>full<br>full<br>full<br>full						
2654023	210958<br>210959<br>210960<br>210961<br>210962<br>210963<br>210964<br>210965<br>210966<br>210967<br>210968<br>210969<br>210970<br>210971<br>210972<br>210973<br>210974<br>210975<br>210976<br>210977<br>210978<br>210979<br>210980<br>210981	2654024<br>2654025<br>2654026<br>2654027<br>2654028<br>2654029<br>2654030<br>2654031<br>2654032<br>2654033<br>2654034<br>2654035<br>2654036<br>2654037<br>2654038<br>2654039<br>2654040<br>2654041<br>2654042<br>2654043<br>2654044<br>2654045<br>2654046<br>2654047	full<br>full<br>full<br>full<br>extended<br>core<br>core<br>core<br>core<br>core<br>core<br>extended<br>extended<br>core<br>core<br>extended<br>extended<br>extended<br>extended<br>extended<br>extended<br>core<br>core<br>full	3	180737281	180803275	+	NM_004301<br>NM_177989<br>NM_178042<br>ENSESTT00000054284<br>ENSESTT00000054285<br>ENST00000259034<br>GENSCAN00000024121	Homo sapiens actin-like 6A (ACTL6A), transcript variant 1, mRNA.<br>Homo sapiens actin-like 6A (ACTL6A), transcript variant 2, mRNA.<br>Homo sapiens actin-like 6A (ACTL6A), transcript variant 3, mRNA.<br><br><br>cdna:known-ccds chromosome:NCBI36:3:180763575:180788886:1 gene:ENSG00000136518 CCDS3231.1<br>cdna:Genscan chromosome:NCBI36:3:180763456:180791804:1

	210982<br>210983<br>210984<br>210985<br>210986<br>210987<br>210988<br>210989<br>210990<br>210991<br>210992	2654048<br>2654049<br>2654050<br>2654051<br>2654052<br>2654053<br>2654054<br>2654055<br>2654056<br>2654057<br>2654058<br>2654059<br>2654060<br>2654061<br>2654062<br>2654063<br>2654064<br>2654065<br>2654066<br>2654067<br>2654068	core<br>core<br>core<br>core<br>core<br>extended<br>core<br>extended<br>core<br>extended<br>core<br>core<br>core<br>full<br>full<br>core<br>core<br>core<br>extended<br>core<br>core						
2662020	215906<br>215907<br>215908<br>215909<br>215910<br>215911<br>215912<br>215913<br>215914<br>215915<br>215916<br>215917<br>215918<br>215919<br>215920<br>215921<br>215922<br>215923<br>215924<br>215925<br>215926<br>215927<br>215928<br>215929<br>215930<br>215931<br>215932<br>215933<br>215934<br>215935	2662021<br>2662022<br>2662023<br>2662024<br>2662025<br>2662026<br>2662027<br>2662028<br>2662029<br>2662030<br>2662031<br>2662032<br>2662033<br>2662034<br>2662035<br>2662036<br>2662037<br>2662038<br>2662039<br>2662040<br>2662041<br>2662042<br>2662043<br>2662044<br>2662045<br>2662046<br>2662047<br>2662048<br>2662049<br>2662050<br>2662051<br>2662052<br>2662053	extended<br>extended<br>extended<br>extended<br>full<br>extended<br>extended<br>extended<br>extended<br>core<br>core<br>core<br>core<br>extended<br>core<br>full<br>full<br>extended<br>full<br>full<br>core<br>core<br>extended<br>core<br>core<br>extended<br>core<br>full<br>core<br>full<br>extended<br>extended<br>extended	3	8792108	8980146	-	NM_020165<br>AK123966<br>ENSESTT00000013545<br>ENST00000383837<br>ENST00000264926<br>GENSCAN00000043750	Homo sapiens RAD18 homolog (S. cerevisiae) (RAD18), mRNA.<br>Homo sapiens cDNA FLJ41972 fis, clone SKNMC2003987.<br><br>cdna:known-ccds chromosome:NCBI36:3:8893760:8980186:-1 gene:ENSG00000070950 CCDS2571.1<br>cdna:known chromosome:NCBI36:3:8896561:8980146:-1 gene:ENSG00000070950<br>cdna:Genscan chromosome:NCBI36:3:8886050:8919204:-1

		2662054<br>2662055<br>2662056<br>2662057<br>2662058<br>2662059<br>2662060<br>2662061	core<br>core<br>core<br>core<br>full<br>core<br>core<br>core						
2676009	224521<br>224522<br>224523<br>224524<br>224525<br>224526<br>224527<br>224528<br>224529<br>224530<br>224531<br>224532<br>224533<br>224534<br>224535<br>224536<br>224537	2676010<br>2676011<br>2676012<br>2676013<br>2676014<br>2676015<br>2676016<br>2676017<br>2676018<br>2676019<br>2676020<br>2676021<br>2676022<br>2676023<br>2676024<br>2676025<br>2676026<br>2676027<br>2676028<br>2676029<br>2676030<br>2676031<br>2676032<br>2676033<br>2676034	full<br>core<br>core<br>core<br>extended<br>extended<br>core<br>core<br>core<br>core<br>core<br>core<br>core<br>core<br>core<br>core<br>extended<br>extended<br>extended<br>full<br>core<br>core<br>core<br>full<br>full	3	52237535	52254242	-	NM_007284<br>AF246973<br>AF246974<br>ENSESTT00000004508<br>ENST00000305533<br>ENST00000310209<br>GENSCAN00000007343	Homo sapiens PTK9L protein tyrosine kinase 9-like (A6-related protein) (PTK9L), mRNA.<br>Homo sapiens toll-like receptor 9 (TLR9) mRNA, partial cds, alternatively spliced.<br>Homo sapiens toll-like receptor 9 (TLR9) mRNA, partial cds, alternatively spliced.<br><br>cdna:known-ccds chromosome:NCBI36:3:52237670:52248223:-1 gene:ENSG00000173366 CCDS2849.1<br>cdna:known chromosome:NCBI36:3:52230273:52238999:-1 gene:ENSG00000173366<br>cdna:Genscan chromosome:NCBI36:3:52238090:52241178:-1
2676182	224623<br>224624<br>224625<br>224626<br>224627<br>224628<br>224629<br>224630<br>224631<br>224632<br>224633<br>224634<br>224635<br>224636<br>224637	2676183<br>2676184<br>2676185<br>2676186<br>2676187<br>2676188<br>2676189<br>2676190<br>2676191<br>2676192<br>2676193<br>2676194<br>2676195<br>2676196<br>2676197<br>2676198<br>2676199<br>2676200<br>2676201<br>2676202	core<br>core<br>core<br>core<br>extended<br>core<br>core<br>extended<br>core<br>extended<br>core<br>extended<br>core<br>full<br>core<br>core<br>core<br>core<br>core<br>core	3	52533445	52546209	-	NM_022908<br>AF131781<br>AK022504<br>AK092469<br>CR612874<br>BC047747<br>ENSESTT00000004495<br>ENSESTT00000004496<br>ENSESTT00000004497<br>ENST00000307076<br>ENST00000307092<br>GENSCAN00000040767	Homo sapiens 5'-nucleotidase domain containing 2 (NT5DC2), mRNA.<br>Homo sapiens clone 24901 mRNA sequence, complete cds.<br>Homo sapiens cDNA FLJ12442 fis, clone NT2RM1000153, weakly similar to CYTOSOLIC PURINE 5'-NUCLEOTIDASE (EC 3.1.3.5).<br>Homo sapiens cDNA FLJ35150 fis, clone PLACE6010568, moderately similar to Homo sapiens mRNA for TU12B1-TY.<br>full-length cDNA clone CS0DF029YP13 of Fetal brain of Homo sapiens (human).<br>Homo sapiens 5'-nucleotidase domain containing 2, mRNA (cDNA clone IMAGE:5767684), containing frame-shift errors.<br><br><br><br><br>cdna:known-ccds chromosome:NCBI36:3:52533443:52544114:-1 gene:ENSG00000168268 CCDS2858.1<br>cdna:known chromosome:NCBI36:3:52533443:52544114:-1 gene:ENSG00000168268<br>cdna:Genscan chromosome:NCBI36:3:52533526:52542793:-1

		2676203	core						
		2676204	core						
		2676205	full						
		2676206	extended						
		2676207	core						
		2676208	core						
		2676209	core						
		2676210	core						
		2676211	core						
		2676212	full						
2686458	231322	2686459	extended	3	101950692	102195002	-	NM_015429	Homo sapiens ABI gene family, member 3 (NESH) binding protein (ABI3BP), mRNA.
	231323	2686460	core					AB056106	Homo sapiens mRNA for NeshBP, complete cds.
	231324	2686461	core					AK025204	Homo sapiens cDNA: FLJ21551 fis, clone COL06266.
	231325	2686462	core					AK123737	Homo sapiens cDNA FLJ41743 fis, clone HSYRA2005456.
	231326	2686463	extended					AK123748	Homo sapiens cDNA FLJ41754 fis, clone HSYRA2009075.
	231327	2686464	core					BX648726	Homo sapiens mRNA; cDNA DKFZp686A12242 (from clone DKFZp686A12242).
	231328	2686465	core					AL833204	Homo sapiens mRNA; cDNA DKFZp667H216 (from clone DKFZp667H216).
	231329	2686466	core					ENSESTT00000007182	
	231330	2686467	extended					ENSESTT00000007183	
	231331	2686468	full					ENSESTT00000007184	
	231332	2686469	core					ENSESTT00000007185	
	231333	2686470	full					ENSESTT00000007186	
	231334	2686471	core					ENST00000284322	cdna:known chromosome:NCBI36:3:101950871:102194939:-1 gene:ENSG00000154175
	231335	2686472	core					ENST00000383692	cdna:known chromosome:NCBI36:3:101951990:102038712:-1 gene:ENSG00000154175
	231336	2686473	core					ENST00000383691	cdna:known chromosome:NCBI36:3:101951990:102019008:-1 gene:ENSG00000154175
	231337	2686474	core					ENST00000273339	cdna:known chromosome:NCBI36:3:102017553:102194939:-1 gene:ENSG00000154175
	231338	2686475	core					GENSCAN00000036759	cdna:Genscan chromosome:NCBI36:3:102066135:102068490:-1
	231339	2686476	core					GENSCAN00000034994	cdna:Genscan chromosome:NCBI36:3:101952029:101967675:-1
	231340	2686477	core					GENSCAN00000042526	cdna:Genscan chromosome:NCBI36:3:102100296:102153390:-1
	231341	2686478	extended						
	231342	2686479	core						
	231343	2686480	core						
	231344	2686481	core						
	231345	2686482	extended						
	231346	2686483	core						
	231347	2686484	extended						
	231348	2686485	full						
	231349	2686486	extended						
	231350	2686487	core						
	231351	2686488	full						
	231352	2686489	full						
	231353	2686490	extended						
	231354	2686491	core						
	231355	2686492	extended						
	231356	2686493	extended						
	231357	2686494	extended						
	231358	2686495	extended						
	231359	2686496	extended						
	231360	2686497	extended						
	231361	2686498	extended						
	231362	2686499	extended						
	231363	2686500	extended						
	231364	2686501	extended						
	231365	2686502	extended						

	231366	2686503	extended						
	231367	2686504	full						
	231368	2686505	full						
	231369	2686506	extended						
	231370	2686507	extended						
	231371	2686508	extended						
	231372	2686509	extended						
	231373	2686510	full						
	231374	2686511	extended						
	231375	2686512	extended						
	231376	2686513	extended						
	231377	2686514	extended						
	231378	2686515	extended						
	231379	2686516	full						
	231380	2686517	extended						
	231381	2686518	extended						
	231382	2686519	extended						
	231383	2686520	extended						
	231384	2686521	extended						
	231385	2686522	extended						
	231386	2686523	extended						
	231387	2686524	extended						
	231388	2686525	extended						
	231389	2686526	extended						
	231390	2686527	extended						
	231391	2686528	extended						
	231392	2686529	extended						
	231393	2686530	extended						
	231394	2686531	core						
	231395	2686532	core						
	231396	2686533	core						
	231397	2686534	core						
	231398	2686535	core						
	231399	2686536	extended						
	231400	2686537	core						
	231401	2686538	core						
	231402	2686539	full						
	231403	2686540	full						
	231404	2686541	core						
	231405	2686542	extended						
	231406	2686543	extended						
	231407	2686544	extended						
	231408	2686545	extended						
	231409	2686546	extended						
	231410	2686547	extended						
	231411	2686548	extended						
	231412	2686549	core						
	231413	2686550	extended						
	231414	2686551	core						
	231415	2686552	full						
	231416	2686553	core						
		2686554	core						
		2686555	core						
		2686556	full						

		2686557<br>2686558<br>2686559<br>2686560<br>2686561<br>2686562<br>2686563<br>2686564<br>2686565<br>2686566<br>2686567<br>2686568<br>2686569<br>2686570<br>2686571<br>2686572<br>2686573<br>2686574<br>2686575<br>2686576<br>2686577<br>2686578<br>2686579	core<br>core<br>core<br>core<br>full<br>extended<br>extended<br>extended<br>extended<br>extended<br>extended<br>full<br>full<br>full<br>full<br>full<br>core<br>full<br>full<br>extended<br>full<br>core<br>core						
2690956	234168<br>234169<br>234170<br>234171<br>234172<br>234173<br>234174<br>234175<br>234176<br>234177<br>234178<br>234179	2690957<br>2690958<br>2690959<br>2690960<br>2690961<br>2690962<br>2690963<br>2690964<br>2690965<br>2690966<br>2690967<br>2690968<br>2690969<br>2690970<br>2690971<br>2690972<br>2690973<br>2690974<br>2690975<br>2690976<br>2690977<br>2690978	extended<br>extended<br>core<br>core<br>core<br>extended<br>extended<br>extended<br>core<br>extended<br>extended<br>core<br>core<br>core<br>extended<br>extended<br>extended<br>core<br>core<br>core<br>extended<br>core	3	120838014	120866860	-	NM_022135<br>AK124602<br>BC026911<br>ENSESTT00000018414<br>ENST00000264231<br>ENST00000341124<br>GENSCAN00000017732	Homo sapiens popeye domain containing 2 (POPDC2), mRNA.<br>Homo sapiens cDNA FLJ42611 fis, clone BRACE3013740.<br>Homo sapiens popeye domain containing 2, mRNA (cDNA clone IMAGE:4517469), complete cds.<br><br>cdna:known-ccds chromosome:NCBI36:3:120843596:120862127:-1 gene:ENSG00000121577 CCDS2992.1<br>cdna:known chromosome:NCBI36:3:120843596:120861960:-1 gene:ENSG00000121577<br>cdna:Genscan chromosome:NCBI36:3:120849699:120861960:-1
2692319	234969<br>234970<br>234971<br>234972<br>234973<br>234974<br>234975<br>234976	2692320<br>2692321<br>2692322<br>2692323<br>2692324<br>2692325<br>2692326<br>2692327	extended<br>core<br>core<br>full<br>full<br>full<br>full<br>core	3	124483836	124650490	-	NM_183357<br>AK093840<br>AK098381<br>AK124691<br>ENSESTT00000001512<br>ENSESTT00000001513<br>ENST00000309879<br>GENSCAN00000030501	Homo sapiens adenylate cyclase 5 (ADCY5), mRNA.<br>Homo sapiens cDNA FLJ36521 fis, clone TRACH2002138, highly similar to ADENYLATE CYCLASE, TYPE V (EC 4.6.1.1).<br>Homo sapiens cDNA FLJ25515 fis, clone CBR06479.<br>Homo sapiens cDNA FLJ42701 fis, clone BRAMY3004919, highly similar to Adenylate cyclase, type V (EC 4.6.1.1).<br><br><br>cdna:known-ccds chromosome:NCBI36:3:124486089:124650082:-1 gene:ENSG00000173175 CCDS3022.1<br>cdna:Genscan chromosome:NCBI36:3:124486145:124554362:-1

	234977	2692328	extended					GENSCAN00000005047	cdna:Genscan chromosome:NCBI36:3:124633806:124651441:-1
	234978	2692329	core						
	234979	2692330	full						
	234980	2692331	core						
	234981	2692332	full						
	234982	2692333	full						
	234983	2692334	full						
	234984	2692335	core						
	234985	2692336	extended						
	234986	2692337	extended						
	234987	2692338	extended						
	234988	2692339	core						
	234989	2692340	extended						
	234990	2692341	extended						
	234991	2692342	core						
	234992	2692343	core						
	234993	2692344	full						
	234994	2692345	core						
	234995	2692346	core						
	234996	2692347	core						
	234997	2692348	full						
	234998	2692349	full						
	234999	2692350	full						
	235000	2692351	core						
	235001	2692352	core						
	235002	2692353	core						
	235003	2692354	full						
	235004	2692355	core						
	235005	2692356	full						
	235006	2692357	core						
	235007	2692358	core						
	235008	2692359	core						
	235009	2692360	full						
	235010	2692361	core						
	235011	2692362	full						
	235012	2692363	extended						
	235013	2692364	core						
	235014	2692365	full						
	235015	2692366	core						
	235016	2692367	full						
	235017	2692368	full						
	235018	2692369	full						
	235019	2692370	full						
	235020	2692371	extended						
	235021	2692372	extended						
	235022	2692373	full						
	235023	2692374	full						
		2692375	extended						
		2692376	full						
		2692377	full						
		2692378	extended						
		2692379	extended						
		2692380	extended						
		2692381	extended						

		2692382<br>2692383<br>2692384<br>2692385<br>2692386<br>2692387<br>2692388<br>2692389<br>2692390	extended<br>full<br>full<br>full<br>full<br>core<br>core<br>extended<br>extended						
2692447	235059<br>235060<br>235061<br>235062<br>235063<br>235064<br>235065<br>235066<br>235067<br>235068<br>235069<br>235070<br>235071<br>235072<br>235073<br>235074<br>235075<br>235076<br>235077<br>235078<br>235079<br>235080<br>235081<br>235082<br>235083<br>235084<br>235085<br>235086<br>235087<br>235088<br>235089<br>235090<br>235091<br>235092<br>235093<br>235094<br>235095<br>235096<br>235097<br>235098<br>235099<br>235100<br>235101<br>235102<br>235103	2692448<br>2692449<br>2692450<br>2692451<br>2692452<br>2692453<br>2692454<br>2692455<br>2692456<br>2692457<br>2692458<br>2692459<br>2692460<br>2692461<br>2692462<br>2692463<br>2692464<br>2692465<br>2692466<br>2692467<br>2692468<br>2692469<br>2692470<br>2692471<br>2692472<br>2692473<br>2692474<br>2692475<br>2692476<br>2692477<br>2692478<br>2692479<br>2692480<br>2692481<br>2692482<br>2692483<br>2692484<br>2692485<br>2692486<br>2692487<br>2692488<br>2692489<br>2692490<br>2692491<br>2692492	core<br>core<br>core<br>core<br>core<br>extended<br>extended<br>core<br>full<br>core<br>core<br>core<br>core<br>extended<br>core<br>extended<br>extended<br>core<br>extended<br>core<br>core<br>core<br>full<br>full<br>extended<br>extended<br>core<br>core<br>core<br>extended<br>core<br>extended<br>extended<br>extended<br>full<br>core<br>core<br>core<br>extended<br>core<br>extended<br>core<br>full<br>full<br>extended	3	124813852	125110868	-	NM_005965<br>NM_053026<br>NM_053025<br>NM_053027<br>NM_053028<br>NM_053031<br>NM_053029<br>NM_053032<br>NM_053030<br>AK022278<br>AK127437<br>AL832211<br>BC040115<br>ENSESTT00000001207<br>ENSESTT00000001208<br>ENSESTT00000001209<br>ENSESTT00000001210<br>ENSESTT00000001211<br>ENSESTT00000001212<br>ENSESTT00000001213<br>ENST00000359169<br>ENST00000361290<br>ENST00000360304<br>ENST00000360772<br>ENST00000346322<br>ENST00000360367<br>ENST00000354792<br>GENSCAN00000010069<br>GENSCAN00000042704<br>GENSCAN00000013941	Homo sapiens myosin, light polypeptide kinase (MYLK), transcript variant 6, mRNA.<br>Homo sapiens myosin, light polypeptide kinase (MYLK), transcript variant 2, mRNA.<br>Homo sapiens myosin, light polypeptide kinase (MYLK), transcript variant 1, mRNA.<br>Homo sapiens myosin, light polypeptide kinase (MYLK), transcript variant 3A, mRNA.<br>Homo sapiens myosin, light polypeptide kinase (MYLK), transcript variant 3B, mRNA.<br>Homo sapiens myosin, light polypeptide kinase (MYLK), transcript variant 7, mRNA.<br>Homo sapiens myosin, light polypeptide kinase (MYLK), transcript variant 4, mRNA.<br>Homo sapiens myosin, light polypeptide kinase (MYLK), transcript variant 8, mRNA.<br>Homo sapiens myosin, light polypeptide kinase (MYLK), transcript variant 5, mRNA.<br>Homo sapiens cDNA FLJ12216 fis, clone MAMMA1001038, highly similar to MYOSIN LIGHT CHAIN KINASE, SMOOTH MUSCLE AND NON-MUSCLE ISOZYMES (EC 2.7.1.117).<br>Homo sapiens cDNA FLJ45529 fis, clone BRTHA2027546.<br>Homo sapiens mRNA; cDNA DKFZp686N112 (from clone DKFZp686N112).<br>Homo sapiens, Similar to myosin, light polypeptide kinase, clone IMAGE:5539579, mRNA.<br><br><br><br><br><br><br><br>cdna:known-ccds chromosome:NCBI36:3:124813835:125085839:-1 gene:ENSG00000065534 CCDS3023.1<br>cdna:known-ccds chromosome:NCBI36:3:124813835:125085839:-1 gene:ENSG00000065534 CCDS3024.1<br>cdna:known chromosome:NCBI36:3:124813835:125085839:-1 gene:ENSG00000065534<br>cdna:known chromosome:NCBI36:3:124813835:125085839:-1 gene:ENSG00000065534<br>cdna:known chromosome:NCBI36:3:124813835:125085839:-1 gene:ENSG00000065534<br>cdna:known chromosome:NCBI36:3:124813835:125085839:-1 gene:ENSG00000065534<br>cdna:known chromosome:NCBI36:3:124841989:125085839:-1 gene:ENSG00000065534<br>cdna:Genscan chromosome:NCBI36:3:125079875:125110868:-1<br>cdna:Genscan chromosome:NCBI36:3:124858663:125033207:-1<br>cdna:Genscan chromosome:NCBI36:3:124815642:124850731:-1

	235104	2692493	full						
	235105	2692494	core						
	235106	2692495	core						
	235107	2692496	full						
	235108	2692497	full						
	235109	2692498	core						
	235110	2692499	core						
	235111	2692500	core						
	235112	2692501	core						
	235113	2692502	core						
	235114	2692503	core						
	235115	2692504	full						
	235116	2692505	extended						
	235117	2692506	extended						
	235118	2692507	extended						
	235119	2692508	full						
	235120	2692509	core						
	235121	2692510	core						
	235122	2692511	core						
	235123	2692512	extended						
	235124	2692513	extended						
	235125	2692514	extended						
	235126	2692515	core						
	235127	2692516	core						
	235128	2692517	extended						
	235129	2692518	extended						
	235130	2692519	full						
	235131	2692520	extended						
	235132	2692521	extended						
	235133	2692522	full						
	235134	2692523	full						
	235135	2692524	core						
	235136	2692525	core						
	235137	2692526	core						
	235138	2692527	full						
	235139	2692528	full						
	235140	2692529	core						
	235141	2692530	core						
	235142	2692531	core						
	235143	2692532	core						
	235144	2692533	extended						
	235145	2692534	extended						
	235146	2692535	full						
	235147	2692536	core						
	235148	2692537	core						
	235149	2692538	core						
	235150	2692539	full						
	235151	2692540	core						
	235152	2692541	full						
	235153	2692542	full						
		2692543	full						
		2692544	full						
		2692545	full						
		2692546	full						

		2692547<br>2692548<br>2692549<br>2692550<br>2692551<br>2692552<br>2692553<br>2692554<br>2692555<br>2692556<br>2692557<br>2692558<br>2692559<br>2692560<br>2692561<br>2692562<br>2692563<br>2692564<br>2692565<br>2692566<br>2692567<br>2692568<br>2692569<br>2692570<br>2692571<br>2692572	extended<br>full<br>full<br>full<br>core<br>full<br>full<br>full<br>full<br>extended<br>full<br>extended<br>core<br>core<br>extended<br>full<br>full<br>full<br>full<br>core<br>full<br>full<br>core<br>extended<br>extended<br>full						
2695941	237285<br>237286<br>237287<br>237288<br>237289<br>237290<br>237291<br>237292<br>237293<br>237294<br>237295<br>237296<br>237297<br>237298<br>237299<br>237300<br>237301<br>237302<br>237303<br>237304<br>237305<br>237306<br>237307<br>237308<br>237309<br>237310<br>237311<br>237312	2695942<br>2695943<br>2695944<br>2695945<br>2695946<br>2695947<br>2695948<br>2695949<br>2695950<br>2695951<br>2695952<br>2695953<br>2695954<br>2695955<br>2695956<br>2695957<br>2695958<br>2695959<br>2695960<br>2695961<br>2695962<br>2695963<br>2695964<br>2695965<br>2695966<br>2695967<br>2695968<br>2695969	extended<br>extended<br>extended<br>extended<br>extended<br>extended<br>core<br>core<br>core<br>core<br>extended<br>extended<br>extended<br>extended<br>core<br>core<br>extended<br>core<br>extended<br>core<br>core<br>core<br>core<br>extended<br>extended<br>core<br>extended<br>extended	3	134799729	134863527	-	NM_007027<br>Y18557<br>BC017552<br>BC032633<br>ENSESTT00000054023<br>ENSESTT00000054024<br>ENSESTT00000054025<br>ENST00000260810<br>GENSCAN00000067450	Homo sapiens topoisomerase (DNA) II binding protein 1 (TOPBP1), mRNA.<br>Homo sapiens mRNA for KIAA0259 protein, partial, 3' UTR.<br>Homo sapiens topoisomerase (DNA) II binding protein 1, mRNA (cDNA clone IMAGE:3911342), with apparent retained intron.<br>Homo sapiens topoisomerase (DNA) II binding protein 1, mRNA (cDNA clone IMAGE:5395690), containing frame-shift errors.<br><br><br><br>cdna:known chromosome:NCBI36:3:134802140:134863380:-1 gene:ENSG00000163781<br>cdna:Genscan chromosome:NCBI36:3:134802784:134851477:-1

	237313<br>237314<br>237315<br>237316<br>237317<br>237318<br>237319<br>237320<br>237321<br>237322<br>237323<br>237324<br>237325<br>237326<br>237327	2695970<br>2695971<br>2695972<br>2695973<br>2695974<br>2695975<br>2695976<br>2695977<br>2695978<br>2695979<br>2695980<br>2695981<br>2695982<br>2695983<br>2695984<br>2695985<br>2695986<br>2695987<br>2695988<br>2695989<br>2695990<br>2695991<br>2695992<br>2695993<br>2695994<br>2695995<br>2695996<br>2695997<br>2695998<br>2695999<br>2696000<br>2696001<br>2696002<br>2696003<br>2696004<br>2696005<br>2696006	core<br>core<br>extended<br>core<br>full<br>core<br>core<br>extended<br>core<br>core<br>extended<br>extended<br>core<br>core<br>core<br>core<br>core<br>core<br>core<br>extended<br>extended<br>core<br>extended<br>core<br>extended<br>extended<br>core<br>extended<br>core<br>core<br>core<br>extended<br>core<br>extended<br>core<br>core<br>full						
2709631	245831<br>245832<br>245833<br>245834<br>245835<br>245836<br>245837<br>245838<br>245839<br>245840<br>245841<br>245842<br>245843<br>245844<br>245845<br>245846<br>245847	2709632<br>2709633<br>2709634<br>2709635<br>2709636<br>2709637<br>2709638<br>2709639<br>2709640<br>2709641<br>2709642<br>2709643<br>2709644<br>2709645<br>2709646<br>2709647<br>2709648	full<br>full<br>full<br>full<br>extended<br>extended<br>extended<br>extended<br>extended<br>core<br>core<br>core<br>core<br>core<br>core<br>core<br>core	3	188410796	188519863	-	NM_139125<br>NM_001031849<br>NM_001879<br>D17525<br>BX641029<br>CR749615<br>ENSESTT00000010500<br>ENSESTT00000010501<br>ENST00000337774<br>ENST00000296280<br>ENST00000169293<br>GENSCAN00000046773	Homo sapiens mannan-binding lectin serine peptidase 1 (C4/C2 activating component of Ra-reactive factor) (MASP1), transcript variant 2, mRNA.<br>Homo sapiens mannan-binding lectin serine peptidase 1 (C4/C2 activating component of Ra-reactive factor) (MASP1), transcript variant 3, mRNA.<br>Homo sapiens mannan-binding lectin serine peptidase 1 (C4/C2 activating component of Ra-reactive factor) (MASP1), transcript variant 1, mRNA.<br>Human mRNA for precursor of P100 serine protease of Ra-reactive factor, complete cds.<br>Homo sapiens mRNA; cDNA DKFZp686M0562 (from clone DKFZp686M0562).<br>Homo sapiens mRNA; cDNA DKFZp686I01199 (from clone DKFZp686I01199).<br><br><br>cdna:known chromosome:NCBI36:3:188418632:188492446:-1 gene:ENSG00000127241<br>cdna:known chromosome:NCBI36:3:188434568:188492446:-1 gene:ENSG00000127241<br>cdna:known chromosome:NCBI36:3:188447304:188492446:-1 gene:ENSG00000127241<br>cdna:Genscan chromosome:NCBI36:3:188420553:188532138:-1

	245848<br>245849<br>245850<br>245851<br>245852<br>245853<br>245854<br>245855<br>245856<br>245857<br>245858<br>245859<br>245860<br>245861<br>245862<br>245863<br>245864<br>245865<br>245866<br>245867<br>245868<br>245869<br>245870<br>245871<br>245872<br>245873<br>245874<br>245875<br>245876	2709649<br>2709650<br>2709651<br>2709652<br>2709653<br>2709654<br>2709655<br>2709656<br>2709657<br>2709658<br>2709659<br>2709660<br>2709661<br>2709662<br>2709663<br>2709664<br>2709665<br>2709666<br>2709667<br>2709668<br>2709669<br>2709670<br>2709671<br>2709672<br>2709673<br>2709674<br>2709675<br>2709676<br>2709677<br>2709678<br>2709679<br>2709680<br>2709681<br>2709682<br>2709683<br>2709684<br>2709685<br>2709686<br>2709687<br>2709688<br>2709689<br>2709690<br>2709691<br>2709692<br>2709693<br>2709694<br>2709695<br>2709696<br>2709697<br>2709698<br>2709699	full<br>full<br>full<br>core<br>extended<br>core<br>full<br>core<br>full<br>core<br>core<br>core<br>core<br>full<br>extended<br>core<br>extended<br>core<br>extended<br>extended<br>extended<br>extended<br>extended<br>extended<br>core<br>extended<br>core<br>full<br>core<br>extended<br>extended<br>core<br>core<br>extended<br>core<br>full<br>full<br>full<br>full<br>full<br>full<br>full<br>full<br>full<br>full<br>full<br>core<br>core<br>core<br>extended<br>full						
2710474	246357<br>246358<br>246359	2710475<br>2710476<br>2710477	extended<br>core<br>core	3	191157213	191329336	-	NM_018192<br>ENSESTT00000046375<br>ENSESTT00000046376	Homo sapiens leprecan-like 1 (LEPREL1), mRNA.

	246360	2710478	extended					ENSESTT00000046377	
	246361	2710479	full					ENSESTT00000046378	
	246362	2710480	full					ENSESTT00000046379	
	246363	2710481	extended					ENSESTT00000046380	
	246364	2710482	extended					ENSESTT00000046381	
	246365	2710483	core					ENSESTT00000046382	
	246366	2710484	core					ENST00000319332	cdna:known-ccds chromosome:NCBI36:3:191157213:191321407:-1 gene:ENSG00000090530 CCDS3294.1
	246367	2710485	extended					GENSCAN00000003188	cdna:Genscan chromosome:NCBI36:3:191260068:191321214:-1
	246368	2710486	extended					GENSCAN00000031374	cdna:Genscan chromosome:NCBI36:3:191162190:191201552:-1
	246369	2710487	extended					GENSCAN00000045486	cdna:Genscan chromosome:NCBI36:3:191104112:191157788:-1
	246370	2710488	extended						
	246371	2710489	extended						
	246372	2710490	full						
	246373	2710491	full						
	246374	2710492	core						
	246375	2710493	extended						
	246376	2710494	core						
	246377	2710495	core						
	246378	2710496	core						
	246379	2710497	extended						
	246380	2710498	core						
	246381	2710499	full						
	246382	2710500	full						
	246383	2710501	full						
	246384	2710502	core						
	246385	2710503	core						
	246386	2710504	core						
	246387	2710505	core						
	246388	2710506	core						
	246389	2710507	full						
	246390	2710508	extended						
	246391	2710509	core						
	246392	2710510	core						
	246393	2710511	core						
	246394	2710512	extended						
	246395	2710513	extended						
	246396	2710514	full						
	246397	2710515	extended						
	246398	2710516	full						
	246399	2710517	full						
	246400	2710518	full						
	246401	2710519	extended						
	246402	2710520	full						
	246403	2710521	extended						
	246404	2710522	free						
	246405	2710523	full						
	246406	2710524	full						
	246407	2710525	full						
	246408	2710526	extended						
		2710527	extended						
		2710528	extended						
		2710529	extended						
		2710530	extended						
		2710531	extended						

		2710532<br>2710533<br>2710534<br>2710535<br>2710536<br>2710537<br>2710538<br>2710539<br>2710540<br>2710541<br>2710542<br>2710543<br>2710544<br>2710545<br>2710546<br>2710547<br>2710548<br>2710549<br>2710550<br>2710551	extended<br>full<br>full<br>extended<br>extended<br>full<br>full<br>core<br>core<br>core<br>core<br>core<br>core<br>core<br>core<br>extended<br>full<br>full<br>full<br>full						
2710599	246434<br>246435<br>246436<br>246437<br>246438<br>246439<br>246440<br>246441<br>246442<br>246443<br>246444<br>246445	2710600<br>2710601<br>2710602<br>2710603<br>2710604<br>2710605<br>2710606<br>2710607<br>2710608<br>2710609<br>2710610<br>2710611<br>2710612<br>2710613<br>2710614<br>2710615<br>2710616<br>2710617<br>2710618<br>2710619	full<br>full<br>extended<br>core<br>core<br>core<br>core<br>core<br>core<br>core<br>extended<br>core<br>core<br>extended<br>extended<br>extended<br>full<br>full<br>core<br>extended	3	191483506	191522932	-	NM_021101<br>AF134160<br>AK225963<br>ENSESTT00000014978<br>ENSESTT00000014979<br>ENSESTT00000014980<br>ENST00000295522<br>GENSCAN00000062238<br>GENSCAN00000018618	Homo sapiens claudin 1 (CLDN1), mRNA.<br>Homo sapiens claudin-1 (CLDN1) mRNA, complete cds.<br>Homo sapiens mRNA for claudin 1 variant, clone: FCC114C08.<br><br><br><br>cdna:known-ccds chromosome:NCBI36:3:191506197:191522909:-1 gene:ENSG00000163347 CCDS3295.1<br>cdna:Genscan chromosome:NCBI36:3:191508760:191522689:-1<br>cdna:Genscan chromosome:NCBI36:3:191483506:191491064:-1
2712236	247509<br>247510<br>247511<br>247512<br>247513<br>247514<br>247515<br>247516<br>247517<br>247518<br>247519<br>247520<br>247521	2712237<br>2712238<br>2712239<br>2712240<br>2712241<br>2712242<br>2712243<br>2712244<br>2712245<br>2712246<br>2712247<br>2712248<br>2712249	core<br>core<br>core<br>core<br>core<br>core<br>extended<br>core<br>core<br>core<br>extended<br>core<br>core	3	196959311	197036867	-	NM_138297<br>NM_004532<br>NM_018406<br>AF058804<br>AF177925<br>AK074437<br>AJ242541<br>AJ000281<br>M64594<br>ENSESTT00000006041<br>ENST00000346145<br>ENST00000349607<br>ENST00000339251	Homo sapiens mucin 4, cell surface associated (MUC4), transcript variant 5, mRNA.<br>Homo sapiens mucin 4, cell surface associated (MUC4), transcript variant 4, mRNA.<br>Homo sapiens mucin 4, cell surface associated (MUC4), transcript variant 1, mRNA.<br>Homo sapiens clone G4-10-3 mucin 4 (MUC4) mRNA, partial cds.<br>Homo sapiens mucin 4 (MUC4) mRNA, partial cds.<br>Homo sapiens cDNA FLJ23857 fis, clone LNG07164.<br>Homo sapiens partial mRNA for sv1-MUC4 apomucin.<br>Homo sapiens mRNA for mucin protein, MUC4.<br>Human tracheo-bronchial mucin (MUC4) mRNA, partial cds.<br><br>cdna:known-ccds chromosome:NCBI36:3:196959311:197023545:-1 gene:ENSG00000145113 CCDS3310.1<br>cdna:known-ccds chromosome:NCBI36:3:196959311:197023545:-1 gene:ENSG00000145113 CCDS3311.1<br>cdna:known chromosome:NCBI36:3:196959311:197023545:-1 gene:ENSG00000145113

	247522	2712250	extended					ENST00000308466	cdna:known chromosome:NCBI36:3:196959311:197002767:-1 gene:ENSG00000145113
	247523	2712251	core					ENST00000333177	cdna:known chromosome:NCBI36:3:196959311:197002767:-1 gene:ENSG00000145113
	247524	2712252	core					ENST00000381931	cdna:novel chromosome:NCBI36:3:196991089:196993062:-1 gene:ENSG00000205811
	247525	2712253	core					ENST00000381929	cdna:novel chromosome:NCBI36:3:196991833:196999518:-1 gene:ENSG00000205811
	247526	2712254	extended					ENST00000381928	cdna:novel chromosome:NCBI36:3:196992865:196993734:-1 gene:ENSG00000205811
	247527	2712255	core					ENST00000381926	cdna:novel chromosome:NCBI36:3:196993105:196993734:-1 gene:ENSG00000205811
	247528	2712256	extended					ENST00000381925	cdna:novel chromosome:NCBI36:3:196995841:196997718:-1 gene:ENSG00000205811
	247529	2712257	core					ENST00000381924	cdna:novel chromosome:NCBI36:3:196995841:196996707:-1 gene:ENSG00000205811
	247530	2712258	full					GENSCAN00000034839	cdna:Genscan chromosome:NCBI36:3:196981259:197036867:-1
	247531	2712259	full					GENSCAN00000034842	cdna:Genscan chromosome:NCBI36:3:196959718:196976984:-1
	247532	2712260	full						
	247533	2712261	core						
	247534	2712262	core						
	247535	2712263	full						
	247536	2712264	core						
	247537	2712265	core						
	247538	2712266	core						
	247539	2712267	core						
	247540	2712268	core						
	247541	2712269	core						
	247542	2712270	core						
	247543	2712271	core						
	247544	2712272	full						
	247545	2712273	core						
	247546	2712274	core						
	247547	2712275	full						
	247548	2712276	core						
	247549	2712277	core						
	247550	2712278	core						
	247551	2712279	core						
	247552	2712280	extended						
	247553	2712281	core						
	247554	2712282	core						
	247555	2712283	full						
	247556	2712284	core						
	247557	2712285	extended						
	247558	2712286	full						
	247559	2712287	full						
	247560	2712288	full						
	247561	2712289	core						
	247562	2712290	core						
	247563	2712291	extended						
	247564	2712292	full						
	247565	2712293	full						
	247566	2712294	extended						
	247567	2712295	extended						
	247568	2712296	extended						
	247569	2712297	extended						
	247570	2712298	full						
	247571	2712299	full						
	247572	2712300	full						
	247573	2712301	extended						
	247574	2712302	extended						
	247575	2712303	full						

	247576	2712304	full						
	247577	2712305	full						
	247578	2712306	full						
	247579	2712307	full						
	247580	2712308	full						
	247581	2712309	extended						
	247582	2712310	extended						
	247583	2712311	full						
	247584	2712312	full						
		2712313	full						
		2712314	full						
		2712315	full						
		2712316	extended						
		2712317	full						
		2712318	extended						
		2712319	extended						
		2712320	full						
		2712321	extended						
		2712322	extended						
		2712323	extended						
		2712324	full						
		2712325	full						
		2712326	full						
		2712327	extended						
		2712328	extended						
		2712329	extended						
		2712330	full						
		2712331	full						
		2712332	full						
		2712333	full						
		2712334	extended						
		2712335	extended						
		2712336	extended						
		2712337	extended						
		2712338	extended						
		2712339	extended						
		2712340	full						
		2712341	extended						
		2712342	extended						
		2712343	extended						
		2712344	extended						
		2712345	extended						
		2712346	extended						
		2712347	extended						
		2712348	extended						
		2712349	full						
		2712350	extended						
		2712351	extended						
		2712352	full						
		2712353	full						
		2712354	full						
		2712355	extended						
		2712356	extended						
		2712357	extended						

		2712358<br>2712359<br>2712360<br>2712361<br>2712362<br>2712363<br>2712364<br>2712365<br>2712366<br>2712367<br>2712368<br>2712369<br>2712370<br>2712371<br>2712372<br>2712373<br>2712374<br>2712375<br>2712376<br>2712377<br>2712378<br>2712379<br>2712380<br>2712381<br>2712382<br>2712383<br>2712384<br>2712385<br>2712386<br>2712387<br>2712388<br>2712389<br>2712390<br>2712391<br>2712392<br>2712393<br>2712394<br>2712395<br>2712396<br>2712397<br>2712398<br>2712399<br>2712400<br>2712401<br>2712402	extended<br>extended<br>full<br>extended<br>full<br>full<br>extended<br>full<br>full<br>extended<br>extended<br>core<br>core<br>core<br>full<br>full<br>full<br>full<br>full<br>extended<br>full<br>full<br>full<br>full<br>extended<br>core<br>core<br>core<br>core<br>extended<br>extended<br>extended<br>full<br>extended<br>full<br>full<br>full<br>full<br>full<br>full<br>full<br>full<br>full<br>full<br>full						
2714465	248801<br>248802<br>248803<br>248804<br>248805<br>248806<br>248807<br>248808<br>248809	2714466<br>2714467<br>2714468<br>2714469<br>2714470<br>2714471<br>2714472<br>2714473<br>2714474	full<br>core<br>core<br>extended<br>extended<br>core<br>full<br>full<br>full	4	994884	1063508	+	NM_001004358<br>NM_021923<br>NM_001004356<br>AK172829<br>BC036769<br>ENSESTT00000034385<br>ENSESTT00000034386<br>ENST00000264748<br>GENSCAN00000021123	Homo sapiens fibroblast growth factor receptor-like 1 (FGFRL1), transcript variant 2, mRNA.<br>Homo sapiens fibroblast growth factor receptor-like 1 (FGFRL1), transcript variant 3, mRNA.<br>Homo sapiens fibroblast growth factor receptor-like 1 (FGFRL1), transcript variant 1, mRNA.<br>Homo sapiens cDNA FLJ23990 fis, clone HRC08053, highly similar to Homo sapiens fibroblast growth factor receptor-like 1 (FGFRL1).<br>Homo sapiens fibroblast growth factor receptor-like 1, mRNA (cDNA clone MGC:46034 IMAGE:5744684), complete cds.<br><br><br>cdna:known-ccds chromosome:NCBI36:4:996239:1010685:1 gene:ENSG00000127418 CCDS3344.1

	248810<br>248811<br>248812<br>248813<br>248814<br>248815<br>248816<br>248817<br>248818<br>248819<br>248820<br>248821<br>248822<br>248823<br>248824<br>248825<br>248826<br>248827<br>248828	2714475<br>2714476<br>2714477<br>2714478<br>2714479<br>2714480<br>2714481<br>2714482<br>2714483<br>2714484<br>2714485<br>2714486<br>2714487<br>2714488<br>2714489<br>2714490<br>2714491<br>2714492<br>2714493<br>2714494<br>2714495<br>2714496<br>2714497<br>2714498<br>2714499<br>2714500<br>2714501<br>2714502<br>2714503<br>2714504<br>2714505<br>2714506<br>2714507	full<br>full<br>full<br>full<br>full<br>full<br>full<br>full<br>core<br>core<br>core<br>core<br>core<br>extended<br>extended<br>core<br>core<br>core<br>core<br>core<br>core<br>core<br>core<br>full<br>full<br>full<br>full<br>full<br>full<br>full<br>full<br>extended<br>full						cdna:Genscan chromosome:NCBI36:4:981642:1009135:1
2717857	250895<br>250896<br>250897<br>250898<br>250899<br>250900<br>250901<br>250902<br>250903<br>250904<br>250905<br>250906<br>250907<br>250908<br>250909<br>250910<br>250911<br>250912<br>250913<br>250914<br>250915	2717858<br>2717859<br>2717860<br>2717861<br>2717862<br>2717863<br>2717864<br>2717865<br>2717866<br>2717867<br>2717868<br>2717869<br>2717870<br>2717871<br>2717872<br>2717873<br>2717874<br>2717875<br>2717876<br>2717877<br>2717878	extended<br>core<br>full<br>full<br>full<br>full<br>full<br>extended<br>extended<br>extended<br>core<br>extended<br>full<br>extended<br>core<br>extended<br>core<br>core<br>extended<br>core<br>core	4	8645287	8677776	+	NM_001014447<br>NM_001014448<br>NM_003652<br>AK127844<br>AK226133<br>BC080539<br>ENSESTT00000016106<br>ENSESTT00000016107<br>ENSESTT00000016108<br>ENSESTT00000016109<br>ENSESTT00000016110<br>ENST00000315782<br>ENST00000360986<br>ENST00000382480<br>GENSCAN00000035542<br>GENSCAN00000035530	Homo sapiens carboxypeptidase Z (CPZ), transcript variant 1, mRNA.<br>Homo sapiens carboxypeptidase Z (CPZ), transcript variant 3, mRNA.<br>Homo sapiens carboxypeptidase Z (CPZ), transcript variant 2, mRNA.<br>Homo sapiens cDNA FLJ45947 fis, clone PLACE7007379, highly similar to Homo sapiens carboxypeptidase Z (CPZ).<br>Homo sapiens mRNA for carboxypeptidase Z isoform 3 variant, clone: fh09362.<br>Homo sapiens carboxypeptidase Z, mRNA (cDNA clone MGC:99682 IMAGE:5020194), complete cds.<br><br><br><br><br><br>cdna:known-ccds chromosome:NCBI36:4:8645287:8672379:1 gene:ENSG00000109625 CCDS3404.1<br>cdna:known chromosome:NCBI36:4:8645287:8672379:1 gene:ENSG00000109625<br>cdna:known chromosome:NCBI36:4:8645287:8672378:1 gene:ENSG00000109625<br>cdna:Genscan chromosome:NCBI36:4:8645461:8677776:1<br>cdna:Genscan chromosome:NCBI36:4:8682999:8683412:1

	250916<br>250917<br>250918	2717879<br>2717880<br>2717881<br>2717882<br>2717883<br>2717884<br>2717885<br>2717886<br>2717887<br>2717888<br>2717889<br>2717890<br>2717891<br>2717892<br>2717893<br>2717894<br>2717895<br>2717896<br>2717897	core<br>full<br>extended<br>core<br>extended<br>core<br>extended<br>extended<br>core<br>full<br>full<br>core<br>core<br>core<br>core<br>full<br>full<br>full<br>full						
2726542	256338<br>256339<br>256340<br>256341<br>256342<br>256343<br>256344<br>256345<br>256346<br>256347<br>256348<br>256349<br>256350<br>256351<br>256352<br>256353<br>256354<br>256355<br>256356<br>256357<br>256358<br>256359<br>256360<br>256361<br>256362<br>256363<br>256364	2726543<br>2726544<br>2726545<br>2726546<br>2726547<br>2726548<br>2726549<br>2726550<br>2726551<br>2726552<br>2726553<br>2726554<br>2726555<br>2726556<br>2726557<br>2726558<br>2726559<br>2726560<br>2726561<br>2726562<br>2726563<br>2726564<br>2726565<br>2726566<br>2726567<br>2726568<br>2726569<br>2726570<br>2726571<br>2726572<br>2726573<br>2726574<br>2726575<br>2726576<br>2726577	extended<br>extended<br>core<br>core<br>full<br>full<br>core<br>full<br>core<br>core<br>core<br>core<br>core<br>core<br>full<br>full<br>core<br>core<br>core<br>full<br>core<br>full<br>core<br>core<br>core<br>core<br>extended<br>core<br>core<br>core<br>full<br>core<br>core<br>core<br>core	4	48682896	48778572	+	NM_025087<br>ENSESTT00000005655<br>ENSESTT00000005656<br>ENSESTT00000005657<br>ENSESTT00000005658<br>ENST00000381454<br>ENST00000226432<br>GENSCAN00000062449<br>GENSCAN00000062447	Homo sapiens hypothetical protein FLJ21511 (FLJ21511), mRNA.<br><br><br><br><br>cdna:known-ccds chromosome:NCBI36:4:48683022:48758853:1 gene:ENSG00000109182 CCDS3486.1<br>cdna:known chromosome:NCBI36:4:48683022:48758850:1 gene:ENSG00000109182<br>cdna:Genscan chromosome:NCBI36:4:48683204:48700655:1<br>cdna:Genscan chromosome:NCBI36:4:48715584:48747623:1

		2726578	full						
2727226	256762	2727227	extended	4	53938569	54922990	+	NM_030917	Homo sapiens FIP1 like 1 (S. cerevisiae) (FIP1L1), mRNA.
	256763	2727228	core					XM_936464	PREDICTED: Homo sapiens similar to hypertension-related calcium-regulated gene (LOC441016), mRNA.
	256764	2727229	core					XM_496693	PREDICTED: Homo sapiens similar to hypertension-related calcium-regulated gene (LOC441016), mRNA.
	256765	2727230	extended					NM_006206	Homo sapiens platelet-derived growth factor receptor, alpha polypeptide (PDGFRA), mRNA.
	256766	2727231	extended					AF090902	Homo sapiens clone HQ0202 PRO0202 mRNA, partial cds.
	256767	2727232	core					AK090938	Homo sapiens cDNA FLJ33619 fis, clone BRAMY2020427.
	256768	2727233	extended					AK091371	Homo sapiens cDNA FLJ34052 fis, clone FCBBF3000175.
	256769	2727234	core					AK123992	Homo sapiens cDNA FLJ41998 fis, clone SPLEN2029522.
	256770	2727235	core					AY229892	Homo sapiens FIP1L1/PDGFRA fusion protein (FIP1L1/PDGFRA fusion) mRNA, complete cds; alternatively spliced.
	256771	2727236	core					BC015186	Homo sapiens platelet-derived growth factor receptor, alpha polypeptide, mRNA (cDNA clone IMAGE:4043984), complete cds.
	256772	2727237	extended					BC017724	Homo sapiens FIP1 like 1 (S. cerevisiae), mRNA (cDNA clone MGC:21370 IMAGE:3452247), complete cds.
	256773	2727238	core					BC024016	Homo sapiens FIP1 like 1 (S. cerevisiae), mRNA (cDNA clone MGC:26986 IMAGE:4826276), complete cds.
	256774	2727239	core					BC063414	Homo sapiens platelet-derived growth factor receptor, alpha polypeptide, mRNA (cDNA clone MGC:74795 IMAGE:5205969),
	256775	2727240	core					X76079	complete cds.
	256776	2727241	full					X95095	H.sapiens mRNA for platelet derived growth factor alpha receptor.
	256777	2727242	core					CR595922	H.sapiens mRNA for PDGFRalpha protein.
	256778	2727243	extended					ENSESTT00000016270	full-length cDNA clone CS0DD005YK11 of Neuroblastoma Cot 50-normalized of Homo sapiens (human).
	256779	2727244	core					ENSESTT00000016271	
	256780	2727245	core					ENSESTT00000016272	
	256781	2727246	core					ENSESTT00000016273	
	256782	2727247	extended					ENSESTT00000016274	
	256783	2727248	extended					ENSESTT00000016275	
	256784	2727249	core					ENSESTT00000016276	
	256785	2727250	full					ENSESTT00000016280	
	256786	2727251	extended					ENSESTT00000054339	
	256787	2727252	core					ENSESTT00000054340	
	256788	2727253	core					ENSESTT00000054341	
	256789	2727254	extended					ENSESTT00000054342	
	256790	2727255	extended					ENSESTT00000054343	
	256791	2727256	extended					ENSESTT00000054344	
	256792	2727257	extended					ENSESTT00000054345	
	256793	2727258	extended					ENSESTT00000054346	
	256794	2727259	extended					ENSESTT00000054347	
	256795	2727260	extended					ENSESTT00000054348	
	256796	2727261	free					ENSESTT00000054349	
	256797	2727262	free					ENST00000337488	
	256798	2727263	core					ENST00000257290	cdna:known-ccds chromosome:NCBI36:4:53938620:54020599:1 gene:ENSG00000145216 CCDS3491.1
	256799	2727264	free					ENST00000306932	cdna:known-ccds chromosome:NCBI36:4:54790204:54859168:1 gene:ENSG00000134853 CCDS3495.1
	256800	2727265	core					ENST00000358575	cdna:known chromosome:NCBI36:4:53938620:54020599:1 gene:ENSG00000145216
	256801	2727266	extended					ENST00000381354	cdna:known chromosome:NCBI36:4:53938620:54020545:1 gene:ENSG00000145216
	256802	2727267	extended					ENST00000312008	cdna:known chromosome:NCBI36:4:54790214:54859171:1 gene:ENSG00000134853
	256803	2727268	core					GENSCAN00000034232	cdna:novel chromosome:NCBI36:4:54136637:54137311:1 gene:ENSG00000173966
	256804	2727269	core					GENSCAN00000005148	cdna:Genscan chromosome:NCBI36:4:54133508:54137311:1
	256805	2727270	extended					GENSCAN00000023418	cdna:Genscan chromosome:NCBI36:4:54822150:54856196:1
	256806	2727271	extended					GENSCAN00000013867	cdna:Genscan chromosome:NCBI36:4:53938763:53944800:1
	256807	2727272	extended					GENSCAN00000038257	cdna:Genscan chromosome:NCBI36:4:54648016:54648288:1
	256808	2727273	full					GENSCAN00000010665	cdna:Genscan chromosome:NCBI36:4:54773914:54795393:1
	256809	2727274	core					GENSCAN00000010666	cdna:Genscan chromosome:NCBI36:4:53950733:54020373:1
	256810	2727275	core						cdna:Genscan chromosome:NCBI36:4:54090496:54111479:1
	256811	2727276	core						
	256812	2727277	core						
	256813	2727278	extended						
	256814	2727279	extended						

	256815	2727280	extended						
	256816	2727281	extended						
	256817	2727282	extended						
	256818	2727283	extended						
	256819	2727284	extended						
	256820	2727285	full						
	256821	2727286	full						
	256822	2727287	full						
	256823	2727288	full						
	256824	2727289	full						
	256825	2727290	extended						
	256826	2727291	extended						
	256827	2727292	extended						
	256828	2727293	full						
	256829	2727294	full						
	256830	2727295	full						
	256831	2727296	extended						
	256832	2727297	extended						
	256833	2727298	extended						
	256834	2727299	full						
	256835	2727300	full						
	256836	2727301	full						
	256837	2727302	full						
	256838	2727303	extended						
	256839	2727304	full						
	256840	2727305	full						
	256841	2727306	full						
	256842	2727307	full						
	256843	2727308	full						
	256844	2727309	full						
	256845	2727310	full						
	256846	2727311	full						
	256847	2727312	full						
	256848	2727313	extended						
	256849	2727314	extended						
	256850	2727315	extended						
	256851	2727316	extended						
	256852	2727317	extended						
	256853	2727318	extended						
	256854	2727319	extended						
	256855	2727320	extended						
	256856	2727321	extended						
	256857	2727322	extended						
	256858	2727323	extended						
	256859	2727324	extended						
	256860	2727325	full						
	256861	2727326	full						
	256862	2727327	full						
	256863	2727328	extended						
	256864	2727329	full						
	256865	2727330	full						
	256866	2727331	full						
	256867	2727332	extended						
	256868	2727333	full						

	256869	2727334	full						
	256870	2727335	full						
	256871	2727336	full						
	256872	2727337	full						
	256873	2727338	full						
	256874	2727339	full						
	256875	2727340	full						
	256876	2727341	full						
	256877	2727342	extended						
	256878	2727343	full						
	256879	2727344	full						
	256880	2727345	full						
	256881	2727346	full						
	256882	2727347	full						
	256883	2727348	extended						
	256884	2727349	core						
	256885	2727350	core						
	256886	2727351	full						
	256887	2727352	full						
	256888	2727353	extended						
	256889	2727354	extended						
	256890	2727355	full						
	256891	2727356	full						
	256892	2727357	full						
	256893	2727358	full						
	256894	2727359	full						
	256895	2727360	full						
	256896	2727361	full						
	256897	2727362	full						
	256898	2727363	full						
	256899	2727364	full						
	256900	2727365	full						
	256901	2727366	full						
		2727367	extended						
		2727368	extended						
		2727369	extended						
		2727370	core						
		2727371	full						
		2727372	core						
		2727373	core						
		2727374	core						
		2727375	core						
		2727376	core						
		2727377	core						
		2727378	core						
		2727379	full						
		2727380	core						
		2727381	core						
		2727382	core						
		2727383	core						
		2727384	extended						
		2727385	full						
		2727386	core						
		2727387	core						

		2727388<br>2727389<br>2727390<br>2727391<br>2727392<br>2727393<br>2727394<br>2727395<br>2727396<br>2727397<br>2727398<br>2727399<br>2727400<br>2727401<br>2727402<br>2727403<br>2727404<br>2727405<br>2727406<br>2727407<br>2727408<br>2727409<br>2727410<br>2727411<br>2727412<br>2727413<br>2727414<br>2727415<br>2727416<br>2727417<br>2727418<br>2727419<br>2727420	core<br>core<br>extended<br>core<br>extended<br>core<br>extended<br>core<br>core<br>core<br>core<br>extended<br>extended<br>core<br>extended<br>core<br>core<br>full<br>core<br>core<br>core<br>full<br>full<br>core<br>extended<br>core<br>core<br>core<br>full<br>full<br>full<br>full<br>full						
2727762	257119<br>257120<br>257121<br>257122<br>257123<br>257124<br>257125<br>257126<br>257127<br>257128<br>257129<br>257130<br>257131<br>257132<br>257133	2727763<br>2727764<br>2727765<br>2727766<br>2727767<br>2727768<br>2727769<br>2727770<br>2727771<br>2727772<br>2727773<br>2727774<br>2727775<br>2727776<br>2727777<br>2727778<br>2727779<br>2727780<br>2727781<br>2727782<br>2727783	core<br>core<br>full<br>full<br>full<br>extended<br>extended<br>full<br>full<br>extended<br>core<br>extended<br>core<br>core<br>full<br>full<br>core<br>core<br>core<br>core<br>extended	4	55907166	55933138	+	NM_024592<br>ENSESTT00000025687<br>ENSESTT00000025688<br>ENST00000324691<br>ENST00000264228<br>GENSCAN00000010011	Homo sapiens steroid 5 alpha-reductase 2-like (SRD5A2L), mRNA.<br><br><br>cdna:known-ccds chromosome:NCBI36:4:55907145:55934020:1 gene:ENSG00000128039 CCDS3498.1<br>cdna:known chromosome:NCBI36:4:55907166:55932235:1 gene:ENSG00000128039<br>cdna:Genscan chromosome:NCBI36:4:55907261:55926493:1

		2727784	extended						
2728938	257838<br>257839<br>257840<br>257841<br>257842<br>257843<br>257844<br>257845<br>257846<br>257847<br>257848<br>257849<br>257850<br>257851<br>257852<br>257853<br>257854<br>257855<br>257856<br>257857<br>257858<br>257859<br>257860<br>257861<br>257862<br>257863<br>257864<br>257865<br>257866<br>257867<br>257868<br>257869<br>257870<br>257871<br>257872<br>257873<br>257874<br>257875<br>257876<br>257877<br>257878<br>257879<br>257880<br>257881<br>257882<br>257883<br>257884<br>257885<br>257886<br>257887<br>257888<br>257889<br>257890	2728939<br>2728940<br>2728941<br>2728942<br>2728943<br>2728944<br>2728945<br>2728946<br>2728947<br>2728948<br>2728949<br>2728950<br>2728951<br>2728952<br>2728953<br>2728954<br>2728955<br>2728956<br>2728957<br>2728958<br>2728959<br>2728960<br>2728961<br>2728962<br>2728963<br>2728964<br>2728965<br>2728966<br>2728967<br>2728968<br>2728969<br>2728970<br>2728971<br>2728972<br>2728973<br>2728974<br>2728975<br>2728976<br>2728977<br>2728978<br>2728979<br>2728980<br>2728981<br>2728982<br>2728983<br>2728984<br>2728985<br>2728986<br>2728987<br>2728988<br>2728989<br>2728990<br>2728991	core<br>extended<br>extended<br>extended<br>extended<br>full<br>full<br>extended<br>extended<br>extended<br>extended<br>core<br>full<br>extended<br>extended<br>extended<br>full<br>full<br>core<br>core<br>full<br>full<br>full<br>extended<br>full<br>full<br>full<br>full<br>extended<br>extended<br>extended<br>full<br>full<br>extended<br>full<br>core<br>full<br>extended<br>extended<br>full<br>full<br>full<br>extended<br>full<br>full<br>full<br>full<br>full<br>extended<br>core<br>extended<br>full<br>extended	4	61749976	62622166	+	NM_015236<br>XM_937110<br>ENSESTT00000013498<br>ENSESTT00000022382<br>ENSESTT00000022383<br>ENSESTT00000022384<br>ENSESTT00000022385<br>ENSESTT00000022386<br>ENSESTT00000022387<br>ENSESTT00000022388<br>ENST00000280009<br>ENST00000295349<br>ENST00000355061<br>GENSCAN00000045466<br>GENSCAN00000048856<br>GENSCAN00000001109<br>GENSCAN00000040521<br>GENSCAN00000023734<br>GENSCAN00000012498	Homo sapiens latrophilin 3 (LPHN3), mRNA.<br>PREDICTED: Homo sapiens similar to Latrophilin-3 precursor (Calcium-independent alpha-latrotoxin receptor 3) (Lectomedin-3) (LOC653950), mRNA.<br>cdna:known chromosome:NCBI36:4:62045434:62620762:1 gene:ENSG00000150471<br>cdna:known chromosome:NCBI36:4:62045434:62620762:1 gene:ENSG00000150471<br>cdna:known chromosome:NCBI36:4:62045434:62620110:1 gene:ENSG00000150471<br>cdna:Genscan chromosome:NCBI36:4:62457873:62544670:1<br>cdna:Genscan chromosome:NCBI36:4:62119236:62232946:1<br>cdna:Genscan chromosome:NCBI36:4:62434820:62445414:1<br>cdna:Genscan chromosome:NCBI36:4:62281067:62289968:1<br>cdna:Genscan chromosome:NCBI36:4:62567041:62619221:1<br>cdna:Genscan chromosome:NCBI36:4:62060305:62068317:1

	257891	2728992	extended							
	257892	2728993	full							
	257893	2728994	full							
	257894	2728995	core							
	257895	2728996	core							
	257896	2728997	extended							
	257897	2728998	full							
	257898	2728999	full							
	257899	2729000	full							
	257900	2729001	full							
	257901	2729002	extended							
	257902	2729003	extended							
	257903	2729004	full							
	257904	2729005	extended							
	257905	2729006	full							
	257906	2729007	full							
	257907	2729008	full							
	257908	2729009	core							
	257909	2729010	extended							
	257910	2729011	extended							
	257911	2729012	extended							
	257912	2729013	extended							
	257913	2729014	extended							
	257914	2729015	full							
	257915	2729016	core							
	257916	2729017	core							
	257917	2729018	extended							
	257918	2729019	extended							
	257919	2729020	full							
	257920	2729021	full							
	257921	2729022	core							
	257922	2729023	extended							
	257923	2729024	full							
	257924	2729025	extended							
	257925	2729026	core							
	257926	2729027	core							
	257927	2729028	core							
	257928	2729029	core							
	257929	2729030	extended							
	257930	2729031	core							
	257931	2729032	extended							
	257932	2729033	extended							
	257933	2729034	extended							
	257934	2729035	core							
	257935	2729036	core							
	257936	2729037	full							
	257937	2729038	full							
	257938	2729039	full							
	257939	2729040	core							
	257940	2729041	core							
	257941	2729042	extended							
	257942	2729043	full							
	257943	2729044	core							
	257944	2729045	core							

	257945<br>257946<br>257947<br>257948<br>257949	2729046<br>2729047<br>2729048<br>2729049<br>2729050<br>2729051<br>2729052<br>2729053<br>2729054<br>2729055<br>2729056<br>2729057<br>2729058<br>2729059<br>2729060<br>2729061<br>2729062<br>2729063<br>2729064<br>2729065<br>2729066	full<br>full<br>full<br>core<br>core<br>core<br>core<br>extended<br>extended<br>extended<br>full<br>extended<br>core<br>extended<br>extended<br>extended<br>core<br>core<br>core<br>core<br>free						
2730746	258940<br>258941<br>258942<br>258943<br>258944<br>258945<br>258946<br>258947<br>258948<br>258949<br>258950<br>258951<br>258952<br>258953<br>258954<br>258955<br>258956<br>258957<br>258958<br>258959<br>258960<br>258961<br>258962<br>258963<br>258964<br>258965<br>258966<br>258967<br>258968<br>258969<br>258970<br>258971<br>258972	2730747<br>2730748<br>2730749<br>2730750<br>2730751<br>2730752<br>2730753<br>2730754<br>2730755<br>2730756<br>2730757<br>2730758<br>2730759<br>2730760<br>2730761<br>2730762<br>2730763<br>2730764<br>2730765<br>2730766<br>2730767<br>2730768<br>2730769<br>2730770<br>2730771<br>2730772<br>2730773<br>2730774<br>2730775<br>2730776<br>2730777<br>2730778<br>2730779	full<br>full<br>full<br>full<br>full<br>core<br>extended<br>extended<br>extended<br>extended<br>full<br>full<br>full<br>core<br>full<br>full<br>full<br>full<br>full<br>full<br>full<br>core<br>core<br>full<br>full<br>full<br>full<br>extended<br>extended<br>extended<br>extended<br>full<br>extended	4	72240703	72797108	+	NM_003759<br>AF004813<br>AF011390<br>BC030977<br>ENSESTT00000014873<br>ENSESTT00000014874<br>ENSESTT00000014875<br>ENSESTT00000014876<br>ENSESTT00000014877<br>ENSESTT00000014878<br>ENSESTT00000014879<br>ENST00000340595<br>ENST00000264485<br>ENST00000351898<br>GENSCAN00000042650<br>GENSCAN00000046032<br>GENSCAN00000043402<br>GENSCAN00000050788	Homo sapiens solute carrier family 4, sodium bicarbonate cotransporter, member 4 (SLC4A4), mRNA.<br>Homo sapiens electrogenic Na+ bicarbonate cotransporter (NBC) mRNA, partial cds.<br>Homo sapiens pancreas sodium bicarbonate cotransporter mRNA, complete cds.<br>Homo sapiens solute carrier family 4, sodium bicarbonate cotransporter, member 4, mRNA (cDNA clone MGC:32627 IMAGE:4610968), complete cds.<br>cdna:known-ccds chromosome:NCBI36:4:72423681:72656663:1 gene:ENSG00000080493 CCDS3549.1<br>cdna:known chromosome:NCBI36:4:72271867:72654298:1 gene:ENSG00000080493<br>cdna:known chromosome:NCBI36:4:72271867:72654298:1 gene:ENSG00000080493<br>cdna:Genscan chromosome:NCBI36:4:72271857:72344483:1<br>cdna:Genscan chromosome:NCBI36:4:72423830:72424086:1<br>cdna:Genscan chromosome:NCBI36:4:72614104:72692244:1<br>cdna:Genscan chromosome:NCBI36:4:72434493:72566441:1

	258973<br>258974<br>258975<br>258976<br>258977<br>258978<br>258979<br>258980<br>258981<br>258982<br>258983<br>258984<br>258985<br>258986<br>258987<br>258988<br>258989<br>258990<br>258991<br>258992<br>258993<br>258994<br>258995<br>258996<br>258997<br>258998<br>258999<br>259000<br>259001<br>259002<br>259003<br>259004	2730780<br>2730781<br>2730782<br>2730783<br>2730784<br>2730785<br>2730786<br>2730787<br>2730788<br>2730789<br>2730790<br>2730791<br>2730792<br>2730793<br>2730794<br>2730795<br>2730796<br>2730797<br>2730798<br>2730799<br>2730800<br>2730801<br>2730802<br>2730803<br>2730804<br>2730805<br>2730806<br>2730807<br>2730808<br>2730809<br>2730810<br>2730811<br>2730812<br>2730813<br>2730814<br>2730815<br>2730816<br>2730817<br>2730818<br>2730819<br>2730820<br>2730821<br>2730822<br>2730823<br>2730824<br>2730825<br>2730826	core<br>core<br>core<br>core<br>core<br>full<br>full<br>full<br>full<br>full<br>core<br>full<br>core<br>core<br>core<br>core<br>core<br>full<br>full<br>core<br>core<br>core<br>extended<br>core<br>core<br>core<br>core<br>core<br>core<br>full<br>core<br>core<br>full<br>core<br>core<br>extended<br>extended<br>core<br>core<br>core<br>core<br>core<br>full<br>full<br>full<br>full<br>full						
2734047	260998<br>260999<br>261000<br>261001<br>261002<br>261003<br>261004	2734048<br>2734049<br>2734050<br>2734051<br>2734052<br>2734053<br>2734054	full<br>full<br>extended<br>extended<br>core<br>core<br>full	4	84675910	84746050	+	NM_032717<br>AK055749<br>ENSESTT00000000138<br>ENSESTT00000000139<br>ENSESTT00000000140<br>ENSESTT00000000141<br>ENST00000264409	Homo sapiens lysophosphatidic acid acyltransferase theta (LPAAT-THETA), mRNA.<br>Homo sapiens cDNA FLJ31187 fis, clone KIDNE2000349, moderately similar to Mus musculus putative lysophosphatidic acid acyltransferase mRNA.

	261005<br>261006<br>261007<br>261008<br>261009<br>261010<br>261011<br>261012<br>261013<br>261014<br>261015<br>261016<br>261017<br>261018<br>261019<br>261020<br>261021<br>261022<br>261023<br>261024<br>261025<br>261026	2734055<br>2734056<br>2734057<br>2734058<br>2734059<br>2734060<br>2734061<br>2734062<br>2734063<br>2734064<br>2734065<br>2734066<br>2734067<br>2734068<br>2734069<br>2734070<br>2734071<br>2734072<br>2734073<br>2734074<br>2734075<br>2734076<br>2734077<br>2734078<br>2734079<br>2734080<br>2734081<br>2734082	full<br>extended<br>extended<br>extended<br>full<br>core<br>extended<br>full<br>full<br>full<br>extended<br>full<br>core<br>extended<br>core<br>core<br>full<br>full<br>core<br>core<br>extended<br>extended<br>core<br>core<br>core<br>core<br>core<br>core					GENSCAN00000067582<br>GENSCAN00000021363	cdna:known-ccds chromosome:NCBI36:4:84676588:84746049:1 gene:ENSG00000138678 CCDS3606.1<br>cdna:Genscan chromosome:NCBI36:4:84675910:84693775:1<br>cdna:Genscan chromosome:NCBI36:4:84702894:84744944:1
2735027	261647<br>261648<br>261649<br>261650<br>261651<br>261652<br>261653<br>261654<br>261655<br>261656<br>261657<br>261658<br>261659<br>261660<br>261661<br>261662<br>261663<br>261664<br>261665<br>261666<br>261667<br>261668<br>261669<br>261670<br>261671<br>261672	2735028<br>2735029<br>2735030<br>2735031<br>2735032<br>2735033<br>2735034<br>2735035<br>2735036<br>2735037<br>2735038<br>2735039<br>2735040<br>2735041<br>2735042<br>2735043<br>2735044<br>2735045<br>2735046<br>2735047<br>2735048<br>2735049<br>2735050<br>2735051<br>2735052<br>2735053	extended<br>extended<br>extended<br>full<br>extended<br>extended<br>full<br>full<br>full<br>full<br>full<br>extended<br>extended<br>full<br>full<br>full<br>full<br>full<br>full<br>full<br>extended<br>extended<br>extended<br>full<br>full<br>full	4	88627267	89123585	+	NM_001040058<br>NM_001040060<br>NM_000582<br>AY956318<br>BC007016<br>BX648003<br>ENSESTT00000042535<br>ENSESTT00000042536<br>ENST00000237623<br>ENST00000360804<br>ENST00000359072<br>GENSCAN00000030225	Homo sapiens secreted phosphoprotein 1 (osteopontin, bone sialoprotein I, early T-lymphocyte activation 1) (SPP1), transcript variant 1, mRNA.<br>Homo sapiens secreted phosphoprotein 1 (osteopontin, bone sialoprotein I, early T-lymphocyte activation 1) (SPP1), transcript variant 3, mRNA.<br>Homo sapiens secreted phosphoprotein 1 (osteopontin, bone sialoprotein I, early T-lymphocyte activation 1) (SPP1), transcript variant 2, mRNA.<br>Homo sapiens osteopontin/immunoglobulin alpha 1 heavy chain constant region fusion protein (SPP1/CALPHA1 fusion) mRNA, partial cds.<br>Homo sapiens secreted phosphoprotein 1 (osteopontin, bone sialoprotein I, early T-lymphocyte activation 1), mRNA (cDNA clone MGC:12351 IMAGE:4052438), complete cds.<br>Homo sapiens mRNA; cDNA DKFZp686G0159 (from clone DKFZp686G0159).<br><br><br>cdna:known-ccds chromosome:NCBI36:4:89115890:89123502:1 gene:ENSG00000118785 CCDS3626.1<br>cdna:known chromosome:NCBI36:4:89115890:89123502:1 gene:ENSG00000118785<br>cdna:known chromosome:NCBI36:4:89115905:89123592:1 gene:ENSG00000118785<br>cdna:Genscan chromosome:NCBI36:4:89115845:89123072:1

	261673<br>261674<br>261675<br>261676<br>261677<br>261678<br>261679<br>261680<br>261681<br>261682<br>261683<br>261684<br>261685<br>261686	2735054<br>2735055<br>2735056<br>2735057<br>2735058<br>2735059<br>2735060<br>2735061<br>2735062<br>2735063<br>2735064<br>2735065<br>2735066<br>2735067<br>2735068<br>2735069<br>2735070<br>2735071<br>2735072	extended<br>core<br>extended<br>core<br>core<br>extended<br>extended<br>extended<br>core<br>extended<br>core<br>extended<br>core<br>core<br>core<br>extended<br>core<br>core<br>core						
2740067	264929<br>264930<br>264931<br>264932<br>264933<br>264934<br>264935<br>264936<br>264937<br>264938<br>264939<br>264940<br>264941<br>264942<br>264943<br>264944<br>264945<br>264946<br>264947<br>264948<br>264949<br>264950<br>264951<br>264952<br>264953<br>264954<br>264955<br>264956<br>264957<br>264958<br>264959<br>264960<br>264961<br>264962<br>264963	2740068<br>2740069<br>2740070<br>2740071<br>2740072<br>2740073<br>2740074<br>2740075<br>2740076<br>2740077<br>2740078<br>2740079<br>2740080<br>2740081<br>2740082<br>2740083<br>2740084<br>2740085<br>2740086<br>2740087<br>2740088<br>2740089<br>2740090<br>2740091<br>2740092<br>2740093<br>2740094<br>2740095<br>2740096<br>2740097<br>2740098<br>2740099<br>2740100<br>2740101<br>2740102	extended<br>extended<br>extended<br>full<br>full<br>full<br>extended<br>extended<br>full<br>full<br>full<br>full<br>extended<br>full<br>full<br>full<br>full<br>extended<br>extended<br>extended<br>full<br>extended<br>full<br>full<br>full<br>core<br>full<br>full<br>full<br>full<br>extended<br>extended<br>full<br>extended<br>extended	4	113846291	114577680	+	NM_020977<br>NM_001148<br>AF131823<br>AK021894<br>AK095596<br>BC030740<br>BX537758<br>BX538132<br>ENSESTT00000010240<br>ENSESTT00000010242<br>ENSESTT00000010244<br>ENSESTT00000010245<br>ENSESTT00000010246<br>ENSESTT00000010249<br>ENSESTT00000010250<br>ENSESTT00000010253<br>ENSESTT00000010254<br>ENSESTT00000010255<br>ENSESTT00000010258<br>ENSESTT00000010259<br>ENST00000357077<br>ENST00000361149<br>ENST00000264366<br>ENST00000343056<br>GENSCAN00000066751<br>GENSCAN00000066753<br>GENSCAN00000066752<br>GENSCAN00000038387<br>GENSCAN00000001761<br>GENSCAN00000001760<br>GENSCAN00000013660<br>GENSCAN00000020745<br>GENSCAN00000058961<br>GENSCAN00000058963	Homo sapiens ankyrin 2, neuronal (ANK2), transcript variant 2, mRNA.<br>Homo sapiens ankyrin 2, neuronal (ANK2), transcript variant 1, mRNA.<br>Homo sapiens clone 24997 mRNA sequence.<br>Homo sapiens cDNA FLJ11832 fis, clone HEMBA1006566.<br>Homo sapiens cDNA FLJ38277 fis, clone FCBBF3004955, highly similar to ANKYRIN 2.<br>Homo sapiens cDNA clone IMAGE:4791521.<br>Homo sapiens mRNA; cDNA DKFZp686H0688 (from clone DKFZp686H0688); complete cds.<br>Homo sapiens mRNA; cDNA DKFZp686M09125 (from clone DKFZp686M09125).<br><br><br><br><br><br><br><br><br><br><br><br><br>cdna:known-ccds chromosome:NCBI36:4:114190319:114524334:1 gene:ENSG00000145362 CCDS3702.1<br>cdna:known chromosome:NCBI36:4:114190319:114524334:1 gene:ENSG00000145362<br>cdna:known chromosome:NCBI36:4:114190319:114524334:1 gene:ENSG00000145362<br>cdna:known chromosome:NCBI36:4:114459190:114524333:1 gene:ENSG00000145362<br>cdna:Genscan chromosome:NCBI36:4:114372787:114443444:1<br>cdna:Genscan chromosome:NCBI36:4:114333556:114340486:1<br>cdna:Genscan chromosome:NCBI36:4:114315021:114328201:1<br>cdna:Genscan chromosome:NCBI36:4:114286474:114291756:1<br>cdna:Genscan chromosome:NCBI36:4:114190334:114214445:1<br>cdna:Genscan chromosome:NCBI36:4:114257201:114260384:1<br>cdna:Genscan chromosome:NCBI36:4:114451860:114577680:1<br>cdna:Genscan chromosome:NCBI36:4:113846751:113847162:1<br>cdna:Genscan chromosome:NCBI36:4:113877427:113899559:1<br>cdna:Genscan chromosome:NCBI36:4:113958730:113968287:1

	264964<br>264965<br>264966<br>264967<br>264968<br>264969<br>264970<br>264971<br>264972<br>264973<br>264974<br>264975<br>264976<br>264977<br>264978<br>264979<br>264980<br>264981<br>264982<br>264983<br>264984<br>264985<br>264986<br>264987<br>264988<br>264989<br>264990<br>264991<br>264992<br>264993<br>264994<br>264995<br>264996<br>264997<br>264998<br>264999<br>265000<br>265001<br>265002<br>265003<br>265004<br>265005<br>265006<br>265007<br>265008<br>265009<br>265010<br>265011<br>265012<br>265013<br>265014<br>265015<br>265016<br>265017	2740103<br>2740104<br>2740105<br>2740106<br>2740107<br>2740108<br>2740109<br>2740110<br>2740111<br>2740112<br>2740113<br>2740114<br>2740115<br>2740116<br>2740117<br>2740118<br>2740119<br>2740120<br>2740121<br>2740122<br>2740123<br>2740124<br>2740125<br>2740126<br>2740127<br>2740128<br>2740129<br>2740130<br>2740131<br>2740132<br>2740133<br>2740134<br>2740135<br>2740136<br>2740137<br>2740138<br>2740139<br>2740140<br>2740141<br>2740142<br>2740143<br>2740144<br>2740145<br>2740146<br>2740147<br>2740148<br>2740149<br>2740150<br>2740151<br>2740152<br>2740153<br>2740154<br>2740155<br>2740156	extended<br>extended<br>extended<br>extended<br>extended<br>full<br>full<br>full<br>full<br>extended<br>extended<br>extended<br>extended<br>extended<br>core<br>full<br>full<br>extended<br>full<br>full<br>full<br>extended<br>full<br>extended<br>extended<br>extended<br>extended<br>extended<br>full<br>full<br>extended<br>extended<br>extended<br>extended<br>extended<br>full<br>full<br>full<br>full<br>extended<br>full<br>full<br>extended<br>extended<br>extended<br>extended<br>extended<br>full<br>extended<br>core<br>full<br>full<br>core<br>core						

	265018	2740157	full						
	265019	2740158	extended						
	265020	2740159	extended						
	265021	2740160	full						
	265022	2740161	extended						
	265023	2740162	extended						
	265024	2740163	extended						
	265025	2740164	extended						
	265026	2740165	extended						
	265027	2740166	extended						
	265028	2740167	extended						
	265029	2740168	core						
	265030	2740169	full						
	265031	2740170	core						
	265032	2740171	full						
	265033	2740172	core						
	265034	2740173	full						
	265035	2740174	core						
	265036	2740175	core						
	265037	2740176	extended						
	265038	2740177	extended						
	265039	2740178	core						
	265040	2740179	extended						
	265041	2740180	full						
	265042	2740181	core						
	265043	2740182	extended						
	265044	2740183	core						
	265045	2740184	core						
	265046	2740185	full						
	265047	2740186	extended						
	265048	2740187	extended						
	265049	2740188	core						
	265050	2740189	extended						
	265051	2740190	extended						
	265052	2740191	core						
	265053	2740192	core						
	265054	2740193	extended						
	265055	2740194	core						
	265056	2740195	core						
	265057	2740196	full						
	265058	2740197	core						
	265059	2740198	core						
	265060	2740199	core						
	265061	2740200	extended						
	265062	2740201	extended						
	265063	2740202	core						
	265064	2740203	extended						
	265065	2740204	core						
	265066	2740205	full						
	265067	2740206	full						
	265068	2740207	extended						
	265069	2740208	extended						
	265070	2740209	core						
	265071	2740210	full						

	265072	2740211	full						
	265073	2740212	extended						
	265074	2740213	core						
	265075	2740214	full						
	265076	2740215	full						
	265077	2740216	extended						
	265078	2740217	core						
	265079	2740218	core						
	265080	2740219	core						
	265081	2740220	core						
	265082	2740221	full						
	265083	2740222	core						
	265084	2740223	core						
	265085	2740224	core						
	265086	2740225	core						
	265087	2740226	extended						
	265088	2740227	core						
	265089	2740228	core						
	265090	2740229	core						
	265091	2740230	core						
	265092	2740231	full						
	265093	2740232	core						
	265094	2740233	extended						
	265095	2740234	extended						
	265096	2740235	extended						
	265097	2740236	core						
	265098	2740237	extended						
	265099	2740238	extended						
	265100	2740239	core						
	265101	2740240	core						
	265102	2740241	core						
	265103	2740242	core						
	265104	2740243	core						
	265105	2740244	core						
	265106	2740245	core						
	265107	2740246	core						
	265108	2740247	core						
		2740248	core						
		2740249	core						
		2740250	core						
		2740251	core						
		2740252	core						
		2740253	core						
		2740254	core						
		2740255	core						
		2740256	full						
		2740257	full						
		2740258	core						
		2740259	core						
		2740260	extended						
		2740261	core						
		2740262	core						
		2740263	core						
		2740264	extended						

		2740265<br>2740266<br>2740267<br>2740268<br>2740269<br>2740270<br>2740271<br>2740272<br>2740273<br>2740274<br>2740275<br>2740276<br>2740277<br>2740278<br>2740279<br>2740280<br>2740281<br>2740282<br>2740283<br>2740284<br>2740285<br>2740286<br>2740287<br>2740288<br>2740289<br>2740290<br>2740291<br>2740292<br>2740293<br>2740294<br>2740295<br>2740296<br>2740297<br>2740298<br>2740299<br>2740300<br>2740301<br>2740302<br>2740303<br>2740304	extended<br>core<br>core<br>extended<br>extended<br>extended<br>extended<br>extended<br>extended<br>extended<br>extended<br>extended<br>extended<br>extended<br>full<br>core<br>core<br>core<br>core<br>core<br>core<br>full<br>extended<br>full<br>full<br>full<br>full<br>full<br>full<br>full<br>full<br>full<br>full<br>full<br>full<br>extended<br>full<br>full<br>full<br>full						
2746591	269003<br>269004<br>269005<br>269006<br>269007<br>269008<br>269009<br>269010<br>269011<br>269012<br>269013<br>269014<br>269015<br>269016	2746592<br>2746593<br>2746594<br>2746595<br>2746596<br>2746597<br>2746598<br>2746599<br>2746600<br>2746601<br>2746602<br>2746603<br>2746604<br>2746605	full<br>full<br>full<br>full<br>extended<br>extended<br>core<br>full<br>full<br>core<br>core<br>core<br>full<br>extended	4	148599594	148685542	+	NM_001957<br>AK123169<br>BX537573<br>S67127<br>ENSESTT00000044921<br>ENSESTT00000044922<br>ENSESTT00000044923<br>ENSESTT00000044924<br>ENST00000324300<br>ENST00000358556<br>ENST00000339690<br>GENSCAN00000005966	Homo sapiens endothelin receptor type A (EDNRA), mRNA.<br>Homo sapiens cDNA FLJ41174 fis, clone BRACE2042398.<br>Homo sapiens mRNA; cDNA DKFZp686O049 (from clone DKFZp686O049).<br>endothelin ETA receptor [human, placenta, mRNA, 1661 nt].<br><br><br><br><br>cdna:known-ccds chromosome:NCBI36:4:148621575:148685555:1 gene:ENSG00000151617 CCDS3769.1<br>cdna:known chromosome:NCBI36:4:148621575:148685555:1 gene:ENSG00000151617<br>cdna:known chromosome:NCBI36:4:148621575:148685555:1 gene:ENSG00000151617<br>cdna:Genscan chromosome:NCBI36:4:148599594:148683204:1

	269017<br>269018<br>269019<br>269020<br>269021<br>269022<br>269023<br>269024<br>269025<br>269026<br>269027<br>269028<br>269029<br>269030	2746606<br>2746607<br>2746608<br>2746609<br>2746610<br>2746611<br>2746612<br>2746613<br>2746614<br>2746615<br>2746616<br>2746617<br>2746618<br>2746619<br>2746620<br>2746621<br>2746622<br>2746623<br>2746624<br>2746625<br>2746626<br>2746627<br>2746628<br>2746629<br>2746630<br>2746631<br>2746632<br>2746633	extended<br>extended<br>extended<br>extended<br>extended<br>extended<br>extended<br>extended<br>extended<br>extended<br>extended<br>full<br>core<br>extended<br>extended<br>extended<br>full<br>core<br>core<br>core<br>extended<br>core<br>core<br>extended<br>full<br>full<br>core<br>core						
2758043	276028<br>276029<br>276030<br>276031<br>276032<br>276033<br>276034<br>276035<br>276036	2758044<br>2758045<br>2758046<br>2758047<br>2758048<br>2758049<br>2758050<br>2758051<br>2758052<br>2758053<br>2758054<br>2758055<br>2758056<br>2758057<br>2758058<br>2758059<br>2758060<br>2758061<br>2758062<br>2758063<br>2758064<br>2758065<br>2758066<br>2758067<br>2758068<br>2758069	core<br>core<br>core<br>extended<br>core<br>extended<br>core<br>core<br>extended<br>core<br>extended<br>core<br>full<br>core<br>core<br>core<br>core<br>core<br>core<br>extended<br>core<br>core<br>core<br>core<br>core<br>extended	4	2902092	2906785	-	NM_001120<br>BC023537<br>CR607301<br>CR607704<br>CR624236<br>ENSESTT00000003413<br>ENSESTT00000003415<br>ENSESTT00000003416<br>ENST00000329687<br>ENST00000355443<br>GENSCAN00000068065	Homo sapiens tetracycline transporter-like protein (TETRAN), mRNA.<br>Homo sapiens tetracycline transporter-like protein, mRNA (cDNA clone IMAGE:4125951), partial cds.<br>full-length cDNA clone CS0DI054YC06 of Placenta Cot 25-normalized of Homo sapiens (human).<br>full-length cDNA clone CS0DC021YI14 of Neuroblastoma Cot 25-normalized of Homo sapiens (human).<br>full-length cDNA clone CS0DF011YB07 of Fetal brain of Homo sapiens (human).<br><br><br><br>cdna:known-ccds chromosome:NCBI36:4:2902090:2906384:-1 gene:ENSG00000109736 CCDS3365.1<br>cdna:known chromosome:NCBI36:4:2902128:2906384:-1 gene:ENSG00000109736<br>cdna:Genscan chromosome:NCBI36:4:2902363:2934844:-1

		2758070<br>2758071<br>2758072	extended<br>full<br>full						
2779199	289308<br>289309<br>289310<br>289311<br>289312<br>289313<br>289314<br>289315<br>289316<br>289317<br>289318<br>289319<br>289320<br>289321<br>289322<br>289323	2779200<br>2779201<br>2779202<br>2779203<br>2779204<br>2779205<br>2779206<br>2779207<br>2779208<br>2779209<br>2779210<br>2779211<br>2779212<br>2779213<br>2779214<br>2779215<br>2779216<br>2779217<br>2779218<br>2779219<br>2779220<br>2779221<br>2779222<br>2779223<br>2779224<br>2779225	core<br>core<br>extended<br>extended<br>core<br>extended<br>full<br>core<br>extended<br>full<br>extended<br>core<br>extended<br>core<br>core<br>core<br>full<br>core<br>extended<br>core<br>core<br>core<br>full<br>core<br>core<br>extended	4	100416547	100431188	-	NM_000667<br>ENSESTT00000025743<br>ENST00000209668<br>GENSCAN00000002246	Homo sapiens alcohol dehydrogenase 1A (class I), alpha polypeptide (ADH1A), mRNA.<br><br>cdna:known-ccds chromosome:NCBI36:4:100416547:100431165:-1 gene:ENSG00000187758 CCDS3648.1<br>cdna:Genscan chromosome:NCBI36:4:100419606:100483175:-1
2786322	293934<br>293935<br>293936<br>293937<br>293938<br>293939<br>293940<br>293941<br>293942<br>293943<br>293944<br>293945<br>293946<br>293947<br>293948<br>293949<br>293950<br>293951<br>293952<br>293953<br>293954<br>293955<br>293956<br>293957	2786323<br>2786324<br>2786325<br>2786326<br>2786327<br>2786328<br>2786329<br>2786330<br>2786331<br>2786332<br>2786333<br>2786334<br>2786335<br>2786336<br>2786337<br>2786338<br>2786339<br>2786340<br>2786341<br>2786342<br>2786343<br>2786344<br>2786345<br>2786346	full<br>full<br>core<br>core<br>extended<br>extended<br>extended<br>core<br>core<br>extended<br>extended<br>full<br>core<br>full<br>core<br>full<br>core<br>core<br>core<br>full<br>full<br>extended<br>free<br>extended	4	139279856	139461590	-	NM_014331<br>ENSESTT00000005135<br>ENSESTT00000005136<br>ENSESTT00000005137<br>ENSESTT00000005138<br>ENST00000280612<br>GENSCAN00000041927	Homo sapiens solute carrier family 7, (cationic amino acid transporter, y+ system) member 11 (SLC7A11), mRNA.<br><br><br><br><br>cdna:known-ccds chromosome:NCBI36:4:139304698:139382953:-1 gene:ENSG00000151012 CCDS3742.1<br>cdna:Genscan chromosome:NCBI36:4:139428794:139468806:-1

	293958<br>293959<br>293960<br>293961<br>293962<br>293963<br>293964<br>293965<br>293966<br>293967<br>293968<br>293969<br>293970<br>293971<br>293972<br>293973	2786347<br>2786348<br>2786349<br>2786350<br>2786351<br>2786352<br>2786353<br>2786354<br>2786355<br>2786356<br>2786357<br>2786358<br>2786359<br>2786360<br>2786361<br>2786362<br>2786363<br>2786364<br>2786365<br>2786366<br>2786367<br>2786368<br>2786369<br>2786370	extended<br>core<br>full<br>full<br>core<br>core<br>full<br>core<br>full<br>core<br>extended<br>extended<br>full<br>core<br>core<br>extended<br>full<br>extended<br>full<br>full<br>full<br>extended<br>extended<br>full						
2788926	295645<br>295646<br>295647<br>295648<br>295649<br>295650<br>295651<br>295652<br>295653<br>295654<br>295655<br>295656<br>295657<br>295658<br>295659<br>295660<br>295661<br>295662<br>295663<br>295664<br>295665<br>295666<br>295667<br>295668<br>295669<br>295670<br>295671<br>295672<br>295673<br>295674	2788927<br>2788928<br>2788929<br>2788930<br>2788931<br>2788932<br>2788933<br>2788934<br>2788935<br>2788936<br>2788937<br>2788938<br>2788939<br>2788940<br>2788941<br>2788942<br>2788943<br>2788944<br>2788945<br>2788946<br>2788947<br>2788948<br>2788949<br>2788950<br>2788951<br>2788952<br>2788953<br>2788954<br>2788955<br>2788956	core<br>core<br>extended<br>full<br>extended<br>full<br>full<br>extended<br>full<br>full<br>full<br>full<br>extended<br>full<br>full<br>core<br>core<br>full<br>full<br>full<br>full<br>full<br>full<br>core<br>full<br>extended<br>extended<br>core<br>core<br>core	4	149219371	149585813	-	NM_000901<br>AK000778<br>AK123047<br>ENSESTT00000044933<br>ENSESTT00000044934<br>ENSESTT00000053748<br>ENSESTT00000053749<br>ENST00000358102<br>ENST00000355292<br>ENST00000344721<br>ENST00000342437<br>GENSCAN00000016308<br>GENSCAN00000004189<br>GENSCAN00000050729<br>GENSCAN00000050728	Homo sapiens nuclear receptor subfamily 3, group C, member 2 (NR3C2), mRNA.<br>Homo sapiens cDNA FLJ20771 fis, clone COL06394.<br>Homo sapiens cDNA FLJ41052 fis, clone SMINT2006648.<br><br><br><br><br>cdna:known-ccds chromosome:NCBI36:4:149219370:149582973:-1 gene:ENSG00000151623 CCDS3772.1<br>cdna:known chromosome:NCBI36:4:149219370:149582973:-1 gene:ENSG00000151623<br>cdna:known chromosome:NCBI36:4:149219370:149582973:-1 gene:ENSG00000151623<br>cdna:known chromosome:NCBI36:4:149219370:149582973:-1 gene:ENSG00000151623<br>cdna:Genscan chromosome:NCBI36:4:149221945:149231441:-1<br>cdna:Genscan chromosome:NCBI36:4:149556085:149593977:-1<br>cdna:Genscan chromosome:NCBI36:4:149241979:149307815:-1<br>cdna:Genscan chromosome:NCBI36:4:149321537:149323280:-1

	295675	2788957	core						
	295676	2788958	core						
	295677	2788959	full						
	295678	2788960	full						
	295679	2788961	extended						
	295680	2788962	full						
	295681	2788963	full						
	295682	2788964	full						
	295683	2788965	full						
	295684	2788966	full						
	295685	2788967	full						
	295686	2788968	full						
	295687	2788969	full						
	295688	2788970	extended						
	295689	2788971	extended						
	295690	2788972	extended						
	295691	2788973	core						
	295692	2788974	core						
	295693	2788975	full						
	295694	2788976	full						
	295695	2788977	full						
	295696	2788978	full						
	295697	2788979	full						
	295698	2788980	extended						
	295699	2788981	extended						
	295700	2788982	extended						
	295701	2788983	full						
	295702	2788984	full						
	295703	2788985	extended						
	295704	2788986	full						
	295705	2788987	full						
	295706	2788988	extended						
	295707	2788989	full						
	295708	2788990	extended						
	295709	2788991	extended						
	295710	2788992	core						
	295711	2788993	extended						
	295712	2788994	extended						
	295713	2788995	extended						
	295714	2788996	extended						
	295715	2788997	extended						
	295716	2788998	extended						
		2788999	extended						
		2789000	extended						
		2789001	full						
		2789002	extended						
		2789003	extended						
		2789004	full						
		2789005	full						
		2789006	full						
		2789007	extended						
		2789008	full						
		2789009	extended						
		2789010	core						

		2789011<br>2789012<br>2789013<br>2789014<br>2789015	full<br>full<br>core<br>core<br>extended						
2796995	300730<br>300731<br>300732<br>300733<br>300734<br>300735<br>300736<br>300737<br>300738<br>300739<br>300740<br>300741<br>300742<br>300743<br>300744<br>300745<br>300746<br>300747<br>300748<br>300749<br>300750<br>300751<br>300752<br>300753<br>300754<br>300755<br>300756<br>300757<br>300758<br>300759<br>300760<br>300761<br>300762<br>300763<br>300764<br>300765<br>300766<br>300767<br>300768<br>300769<br>300770<br>300771<br>300772<br>300773<br>300774<br>300775<br>300776<br>300777<br>300778	2796996<br>2796997<br>2796998<br>2796999<br>2797000<br>2797001<br>2797002<br>2797003<br>2797004<br>2797005<br>2797006<br>2797007<br>2797008<br>2797009<br>2797010<br>2797011<br>2797012<br>2797013<br>2797014<br>2797015<br>2797016<br>2797017<br>2797018<br>2797019<br>2797020<br>2797021<br>2797022<br>2797023<br>2797024<br>2797025<br>2797026<br>2797027<br>2797028<br>2797029<br>2797030<br>2797031<br>2797032<br>2797033<br>2797034<br>2797035<br>2797036<br>2797037<br>2797038<br>2797039<br>2797040<br>2797041<br>2797042<br>2797043<br>2797044	full<br>full<br>full<br>full<br>full<br>full<br>full<br>full<br>full<br>full<br>full<br>full<br>full<br>extended<br>extended<br>core<br>core<br>core<br>core<br>core<br>extended<br>extended<br>core<br>extended<br>extended<br>extended<br>extended<br>extended<br>extended<br>core<br>extended<br>full<br>extended<br>extended<br>extended<br>extended<br>extended<br>full<br>extended<br>extended<br>full<br>extended<br>extended<br>extended<br>extended<br>extended<br>core<br>extended<br>full	4	186728441	187114516	-	NM_021069<br>NM_003603<br>AK000782<br>AK022383<br>AK056628<br>AK056758<br>AK225327<br>AK225812<br>BC011883<br>BC035329<br>CR625998<br>ENSESTT00000031828<br>ENSESTT00000031829<br>ENSESTT00000031830<br>ENSESTT00000031831<br>ENSESTT00000031832<br>ENSESTT00000031833<br>ENSESTT00000031834<br>ENSESTT00000031835<br>ENSESTT00000031836<br>ENST00000284776<br>ENST00000319454<br>ENST00000319471<br>ENST00000355634<br>GENSCAN00000035491<br>GENSCAN00000035494<br>GENSCAN00000035489<br>GENSCAN00000060021<br>GENSCAN00000009098<br>GENSCAN00000009099	Homo sapiens sorbin and SH3 domain containing 2 (SORBS2), transcript variant 2, mRNA.<br>Homo sapiens sorbin and SH3 domain containing 2 (SORBS2), transcript variant 1, mRNA.<br>Homo sapiens cDNA FLJ20775 fis, clone COL06005.<br>Homo sapiens cDNA FLJ12321 fis, clone MAMMA1002084.<br>Homo sapiens cDNA FLJ32066 fis, clone OCBBF1000091, highly similar to SORBIN.<br>Homo sapiens cDNA FLJ32196 fis, clone PLACE6002668, highly similar to Homo sapiens Arg/Abl-interacting protein ArgBP2b mRNA.<br>Homo sapiens mRNA for Arg/Abl-interacting protein 2 isoform 1 variant, clone: HEP12372.<br>Homo sapiens mRNA for Arg/Abl-interacting protein 2 isoform 1 variant, clone: FCC129E07.<br>Homo sapiens sorbin and SH3 domain containing 2, mRNA (cDNA clone MGC:20120 IMAGE:3677070), complete cds.<br>Homo sapiens sorbin and SH3 domain containing 2, mRNA (cDNA clone IMAGE:5194874).<br>full-length cDNA clone CS0DC027YF23 of Neuroblastoma Cot 25-normalized of Homo sapiens (human).<br><br><br><br><br><br><br><br><br><br>cdna:known-ccds chromosome:NCBI36:4:186743592:187114516:-1 gene:ENSG00000154556 CCDS3845.1<br>cdna:known chromosome:NCBI36:4:186745470:186969219:-1 gene:ENSG00000154556<br>cdna:known chromosome:NCBI36:4:186745471:186970369:-1 gene:ENSG00000154556<br>cdna:novel chromosome:NCBI36:4:186743592:187114516:-1 gene:ENSG00000154556<br>cdna:Genscan chromosome:NCBI36:4:186728441:186777503:-1<br>cdna:Genscan chromosome:NCBI36:4:186781054:186782793:-1<br>cdna:Genscan chromosome:NCBI36:4:186790704:186820280:-1<br>cdna:Genscan chromosome:NCBI36:4:186958617:186958802:-1<br>cdna:Genscan chromosome:NCBI36:4:186823803:186828968:-1<br>cdna:Genscan chromosome:NCBI36:4:186835136:186848846:-1

	300779	2797045	extended						
	300780	2797046	extended						
	300781	2797047	core						
	300782	2797048	core						
	300783	2797049	extended						
	300784	2797050	extended						
	300785	2797051	extended						
	300786	2797052	core						
	300787	2797053	full						
	300788	2797054	full						
	300789	2797055	core						
	300790	2797056	core						
	300791	2797057	core						
	300792	2797058	core						
	300793	2797059	core						
	300794	2797060	core						
	300795	2797061	core						
	300796	2797062	core						
	300797	2797063	full						
	300798	2797064	core						
	300799	2797065	core						
	300800	2797066	full						
	300801	2797067	core						
	300802	2797068	extended						
	300803	2797069	extended						
	300804	2797070	core						
	300805	2797071	extended						
	300806	2797072	core						
	300807	2797073	core						
	300808	2797074	extended						
	300809	2797075	extended						
	300810	2797076	extended						
	300811	2797077	extended						
	300812	2797078	core						
	300813	2797079	extended						
	300814	2797080	extended						
	300815	2797081	core						
	300816	2797082	extended						
	300817	2797083	core						
	300818	2797084	extended						
	300819	2797085	core						
	300820	2797086	full						
	300821	2797087	extended						
	300822	2797088	extended						
	300823	2797089	extended						
	300824	2797090	extended						
	300825	2797091	full						
	300826	2797092	full						
	300827	2797093	core						
	300828	2797094	extended						
	300829	2797095	extended						
	300830	2797096	extended						
	300831	2797097	extended						
	300832	2797098	extended						

	300833	2797099	extended						
	300834	2797100	core						
	300835	2797101	core						
	300836	2797102	core						
	300837	2797103	full						
	300838	2797104	full						
	300839	2797105	full						
	300840	2797106	full						
	300841	2797107	full						
	300842	2797108	extended						
	300843	2797109	full						
	300844	2797110	full						
	300845	2797111	full						
	300846	2797112	full						
	300847	2797113	core						
	300848	2797114	core						
	300849	2797115	core						
	300850	2797116	extended						
	300851	2797117	extended						
	300852	2797118	extended						
	300853	2797119	extended						
	300854	2797120	core						
	300855	2797121	core						
	300856	2797122	full						
	300857	2797123	core						
	300858	2797124	full						
	300859	2797125	full						
	300860	2797126	extended						
	300861	2797127	extended						
	300862	2797128	extended						
	300863	2797129	extended						
	300864	2797130	full						
	300865	2797131	extended						
	300866	2797132	extended						
	300867	2797133	full						
	300868	2797134	full						
	300869	2797135	extended						
	300870	2797136	extended						
	300871	2797137	extended						
	300872	2797138	extended						
	300873	2797139	extended						
	300874	2797140	extended						
	300875	2797141	extended						
	300876	2797142	extended						
	300877	2797143	extended						
	300878	2797144	extended						
	300879	2797145	extended						
	300880	2797146	extended						
	300881	2797147	extended						
	300882	2797148	full						
		2797149	full						
		2797150	extended						
		2797151	full						
		2797152	extended						

		2797153<br>2797154<br>2797155<br>2797156<br>2797157<br>2797158<br>2797159<br>2797160<br>2797161<br>2797162<br>2797163<br>2797164<br>2797165<br>2797166<br>2797167<br>2797168<br>2797169<br>2797170<br>2797171<br>2797172<br>2797173<br>2797174<br>2797175<br>2797176<br>2797177<br>2797178<br>2797179<br>2797180<br>2797181<br>2797182<br>2797183<br>2797184<br>2797185<br>2797186<br>2797187<br>2797188<br>2797189<br>2797190<br>2797191<br>2797192<br>2797193<br>2797194<br>2797195<br>2797196<br>2797197<br>2797198<br>2797199<br>2797200<br>2797201	extended<br>extended<br>extended<br>extended<br>extended<br>extended<br>extended<br>extended<br>extended<br>full<br>extended<br>core<br>extended<br>extended<br>extended<br>full<br>full<br>extended<br>extended<br>extended<br>extended<br>full<br>full<br>extended<br>extended<br>extended<br>core<br>core<br>full<br>full<br>full<br>full<br>full<br>full<br>full<br>full<br>full<br>full<br>full<br>extended<br>full<br>full<br>extended<br>extended<br>full<br>extended<br>full<br>extended<br>core						
2805786	306201<br>306202<br>306203<br>306204<br>306205	2805787<br>2805788<br>2805789<br>2805790<br>2805791	full<br>full<br>full<br>full<br>full	5	33443285	33505381	+	NM_152295<br>BC003699<br>ENSESTT00000022073<br>ENSESTT00000022074<br>ENSESTT00000022075	Homo sapiens threonyl-tRNA synthetase (TARS), mRNA.<br>Homo sapiens threonyl-tRNA synthetase, mRNA (cDNA clone IMAGE:3461963).

	306206<br>306207<br>306208<br>306209<br>306210<br>306211<br>306212<br>306213<br>306214<br>306215<br>306216<br>306217<br>306218<br>306219<br>306220<br>306221<br>306222<br>306223<br>306224<br>306225<br>306226<br>306227<br>306228<br>306229<br>306230<br>306231<br>306232<br>306233	2805792<br>2805793<br>2805794<br>2805795<br>2805796<br>2805797<br>2805798<br>2805799<br>2805800<br>2805801<br>2805802<br>2805803<br>2805804<br>2805805<br>2805806<br>2805807<br>2805808<br>2805809<br>2805810<br>2805811<br>2805812<br>2805813<br>2805814<br>2805815<br>2805816<br>2805817<br>2805818<br>2805819<br>2805820<br>2805821<br>2805822<br>2805823<br>2805824<br>2805825<br>2805826<br>2805827<br>2805828	full<br>core<br>core<br>core<br>full<br>extended<br>core<br>extended<br>full<br>core<br>core<br>core<br>core<br>core<br>core<br>core<br>core<br>core<br>extended<br>extended<br>core<br>core<br>core<br>core<br>core<br>core<br>extended<br>extended<br>extended<br>extended<br>core<br>full<br>core<br>core<br>core<br>extended<br>extended					ENSESTT00000022076<br>ENST00000382115<br>ENST00000265112<br>GENSCAN00000033387	 cdna:known-ccds chromosome:NCBI36:5:33476639:33505401:1 gene:ENSG00000113407 CCDS3899.1<br>cdna:known chromosome:NCBI36:5:33476655:33503953:1 gene:ENSG00000113407<br>cdna:Genscan chromosome:NCBI36:5:33454348:33481267:1
2812359	310433<br>310434<br>310435<br>310436<br>310437<br>310438<br>310439<br>310440<br>310441<br>310442<br>310443<br>310444<br>310445<br>310446<br>310447<br>310448<br>310449	2812360<br>2812361<br>2812362<br>2812363<br>2812364<br>2812365<br>2812366<br>2812367<br>2812368<br>2812369<br>2812370<br>2812371<br>2812372<br>2812373<br>2812374<br>2812375<br>2812376	full<br>full<br>full<br>full<br>full<br>core<br>core<br>full<br>full<br>full<br>full<br>extended<br>full<br>full<br>core<br>core<br>core	5	65052933	65315724	+	NM_020726<br>AB033052<br>AK026655<br>BC001644<br>ENSESTT00000045063<br>ENSESTT00000045064<br>ENSESTT00000045065<br>ENST00000380985<br>ENST00000340159<br>GENSCAN00000056849<br>GENSCAN00000007676	Homo sapiens neurolysin (metallopeptidase M3 family) (NLN), mRNA.<br>Homo sapiens mRNA for KIAA1226 protein, partial cds.<br>Homo sapiens cDNA: FLJ23002 fis, clone LNG00310.<br>Homo sapiens neurolysin (metallopeptidase M3 family), mRNA (cDNA clone MGC:2361 IMAGE:2958628), complete cds.<br><br><br><br>cdna:known-ccds chromosome:NCBI36:5:65053779:65160867:1 gene:ENSG00000123213 CCDS3989.1<br>cdna:known chromosome:NCBI36:5:65053957:65158495:1 gene:ENSG00000123213<br>cdna:Genscan chromosome:NCBI36:5:65090178:65111864:1<br>cdna:Genscan chromosome:NCBI36:5:65256496:65315724:1

	310450<br>310451<br>310452<br>310453<br>310454<br>310455<br>310456<br>310457<br>310458<br>310459<br>310460<br>310461<br>310462<br>310463<br>310464<br>310465<br>310466<br>310467<br>310468<br>310469<br>310470<br>310471<br>310472<br>310473<br>310474<br>310475<br>310476<br>310477<br>310478<br>310479	2812377<br>2812378<br>2812379<br>2812380<br>2812381<br>2812382<br>2812383<br>2812384<br>2812385<br>2812386<br>2812387<br>2812388<br>2812389<br>2812390<br>2812391<br>2812392<br>2812393<br>2812394<br>2812395<br>2812396<br>2812397<br>2812398<br>2812399<br>2812400<br>2812401<br>2812402<br>2812403<br>2812404<br>2812405<br>2812406<br>2812407<br>2812408<br>2812409<br>2812410<br>2812411<br>2812412<br>2812413<br>2812414<br>2812415<br>2812416<br>2812417<br>2812418<br>2812419	extended<br>extended<br>extended<br>core<br>core<br>core<br>extended<br>extended<br>core<br>core<br>extended<br>core<br>core<br>core<br>core<br>core<br>core<br>full<br>extended<br>full<br>core<br>core<br>core<br>full<br>full<br>full<br>full<br>core<br>extended<br>extended<br>full<br>full<br>full<br>extended<br>full<br>full<br>extended<br>full<br>full<br>full<br>full<br>full<br>full						
2816459	313095<br>313096<br>313097<br>313098<br>313099<br>313100	2816460<br>2816461<br>2816462<br>2816463<br>2816464<br>2816465<br>2816466<br>2816467<br>2816468<br>2816469<br>2816470	core<br>core<br>core<br>extended<br>full<br>core<br>core<br>core<br>core<br>core<br>core	5	76047542	76074271	+	NM_001992<br>AY771596<br>BC016059<br>ENST00000319211<br>GENSCAN00000064107	Homo sapiens coagulation factor II (thrombin) receptor (F2R), mRNA.<br>Homo sapiens cell proliferation-inducing protein 18 mRNA, complete cds.<br>Homo sapiens coagulation factor II (thrombin) receptor, mRNA (cDNA clone IMAGE:4849569), with apparent retained intron.<br>cdna:known-ccds chromosome:NCBI36:5:76047542:76067054:1 gene:ENSG00000181104 CCDS4032.1<br>cdna:Genscan chromosome:NCBI36:5:76047889:76074271:1

		2816471<br>2816472<br>2816473<br>2816474	core<br>extended<br>extended<br>full						
2818517	314370<br>314371<br>314372<br>314373<br>314374<br>314375<br>314376<br>314377<br>314378<br>314379<br>314380<br>314381<br>314382<br>314383<br>314384<br>314385<br>314386<br>314387<br>314388<br>314389<br>314390<br>314391<br>314392<br>314393<br>314394<br>314395<br>314396<br>314397<br>314398<br>314399<br>314400<br>314401<br>314402<br>314403<br>314404<br>314405<br>314406<br>314407<br>314408<br>314409<br>314410<br>314411<br>314412<br>314413<br>314414	2818518<br>2818519<br>2818520<br>2818521<br>2818522<br>2818523<br>2818524<br>2818525<br>2818526<br>2818527<br>2818528<br>2818529<br>2818530<br>2818531<br>2818532<br>2818533<br>2818534<br>2818535<br>2818536<br>2818537<br>2818538<br>2818539<br>2818540<br>2818541<br>2818542<br>2818543<br>2818544<br>2818545<br>2818546<br>2818547<br>2818548<br>2818549<br>2818550<br>2818551<br>2818552<br>2818553<br>2818554<br>2818555<br>2818556<br>2818557<br>2818558<br>2818559<br>2818560<br>2818561<br>2818562<br>2818563<br>2818564<br>2818565<br>2818566<br>2818567	full<br>full<br>full<br>full<br>full<br>extended<br>core<br>core<br>extended<br>extended<br>extended<br>core<br>extended<br>extended<br>core<br>core<br>core<br>core<br>core<br>extended<br>full<br>extended<br>full<br>extended<br>extended<br>core<br>core<br>extended<br>extended<br>free<br>extended<br>core<br>core<br>extended<br>extended<br>extended<br>extended<br>extended<br>extended<br>extended<br>extended<br>core<br>core<br>core<br>core<br>core<br>core<br>core<br>extended<br>extended	5	82778142	82940816	+	NM_004385<br>AF084545<br>BC050524<br>BX641036<br>ENSESTT00000044242<br>ENSESTT00000044243<br>ENSESTT00000044244<br>ENST00000265077<br>ENST00000343200<br>ENST00000342785<br>ENST00000348134<br>GENSCAN00000005837	Homo sapiens chondroitin sulfate proteoglycan 2 (versican) (CSPG2), mRNA.<br>Homo sapiens versican Vint isoform, mRNA, partial cds.<br>Homo sapiens chondroitin sulfate proteoglycan 2 (versican), mRNA (cDNA clone IMAGE:5218077), complete cds.<br>Homo sapiens mRNA; cDNA DKFZp686K06110 (from clone DKFZp686K06110).<br><br><br><br>cdna:known-ccds chromosome:NCBI36:5:82803339:82912737:1 gene:ENSG00000038427 CCDS4060.1<br>cdna:known chromosome:NCBI36:5:82803339:82912737:1 gene:ENSG00000038427<br>cdna:known chromosome:NCBI36:5:82803339:82912737:1 gene:ENSG00000038427<br>cdna:known chromosome:NCBI36:5:82803339:82912737:1 gene:ENSG00000038427<br>cdna:Genscan chromosome:NCBI36:5:82783461:82805959:1

		2818568<br>2818569<br>2818570<br>2818571<br>2818572<br>2818573<br>2818574<br>2818575<br>2818576<br>2818577<br>2818578<br>2818579<br>2818580<br>2818581<br>2818582<br>2818583<br>2818584<br>2818585<br>2818586<br>2818587<br>2818588<br>2818589<br>2818590<br>2818591	core<br>extended<br>extended<br>core<br>core<br>core<br>extended<br>full<br>full<br>core<br>core<br>extended<br>extended<br>core<br>core<br>extended<br>extended<br>extended<br>extended<br>extended<br>full<br>full<br>full<br>full						
2830638	321998<br>321999<br>322000<br>322001<br>322002<br>322003<br>322004<br>322005<br>322006<br>322007<br>322008<br>322009<br>322010<br>322011<br>322012<br>322013<br>322014<br>322015<br>322016<br>322017<br>322018<br>322019<br>322020	2830639<br>2830640<br>2830641<br>2830642<br>2830643<br>2830644<br>2830645<br>2830646<br>2830647<br>2830648<br>2830649<br>2830650<br>2830651<br>2830652<br>2830653<br>2830654<br>2830655<br>2830656<br>2830657<br>2830658<br>2830659<br>2830660<br>2830661<br>2830662<br>2830663<br>2830664<br>2830665<br>2830666<br>2830667<br>2830668	extended<br>extended<br>extended<br>extended<br>extended<br>extended<br>core<br>extended<br>full<br>core<br>core<br>core<br>core<br>core<br>full<br>core<br>core<br>core<br>core<br>core<br>core<br>core<br>core<br>core<br>core<br>core<br>core<br>core<br>extended<br>extended	5	137542289	137552275	+	NM_005733<br>ENSESTT00000045971<br>ENSESTT00000045972<br>ENSESTT00000045973<br>ENSESTT00000045974<br>ENST00000230902<br>GENSCAN00000012413	Homo sapiens kinesin family member 20A (KIF20A), mRNA.<br><br><br><br><br>cdna:known-ccds chromosome:NCBI36:5:137542757:137551304:1 gene:ENSG00000112984 CCDS4199.1<br>cdna:Genscan chromosome:NCBI36:5:137543254:137551001:1

2841964	328904<br>328905<br>328906<br>328907<br>328908<br>328909	2841965<br>2841966<br>2841967<br>2841968<br>2841969<br>2841970<br>2841971<br>2841972<br>2841973<br>2841974<br>2841975	extended<br>extended<br>core<br>extended<br>extended<br>core<br>core<br>core<br>core<br>core<br>core	5	174083895	174090482	+	NR_002307<br>NM_002449<br>ENSESTT00000020818<br>ENST00000239243<br>GENSCAN00000023107	Homo sapiens msh homeobox homolog 2 (Drosophila) pseudogene (MSX2P) on chromosome 17.<br>Homo sapiens msh homeobox homolog 2 (Drosophila) (MSX2), mRNA.<br><br>cdna:known-ccds chromosome:NCBI36:5:174084208:174090507:1 gene:ENSG00000120149 CCDS4392.1<br>cdna:Genscan chromosome:NCBI36:5:174084269:174089192:1
2842624	329299<br>329300<br>329301<br>329302<br>329303<br>329304<br>329305<br>329306<br>329307<br>329308<br>329309<br>329310<br>329311<br>329312<br>329313<br>329314<br>329315<br>329316<br>329317<br>329318<br>329319<br>329320<br>329321<br>329322<br>329323<br>329324<br>329325<br>329326<br>329327<br>329328<br>329329<br>329330<br>329331<br>329332<br>329333<br>329334<br>329335<br>329336<br>329337<br>329338<br>329339<br>329340	2842625<br>2842626<br>2842627<br>2842628<br>2842629<br>2842630<br>2842631<br>2842632<br>2842633<br>2842634<br>2842635<br>2842636<br>2842637<br>2842638<br>2842639<br>2842640<br>2842641<br>2842642<br>2842643<br>2842644<br>2842645<br>2842646<br>2842647<br>2842648<br>2842649<br>2842650<br>2842651<br>2842652<br>2842653<br>2842654<br>2842655<br>2842656<br>2842657<br>2842658<br>2842659<br>2842660<br>2842661<br>2842662<br>2842663<br>2842664<br>2842665<br>2842666<br>2842667	full<br>full<br>full<br>extended<br>extended<br>extended<br>full<br>full<br>extended<br>full<br>full<br>full<br>core<br>core<br>core<br>core<br>core<br>core<br>extended<br>core<br>core<br>core<br>core<br>core<br>core<br>full<br>full<br>core<br>core<br>core<br>core<br>core<br>full<br>core<br>core<br>core<br>core<br>core<br>extended<br>core<br>core<br>core<br>full	5	175902503	175956759	+	NM_017675<br>AK128446<br>ENSESTT00000006503<br>ENSESTT00000006504<br>ENSESTT00000006505<br>ENSESTT00000006506<br>ENSESTT00000006507<br>ENST00000389947<br>ENST00000389946<br>ENST00000261944<br>GENSCAN00000044009	Homo sapiens protocadherin LKC (PCLKC), mRNA.<br>Homo sapiens cDNA FLJ46589 fis, clone THYMU3044188.<br><br><br><br><br><br>cdna:known chromosome:NCBI36:5:175924960:175955279:1 gene:ENSG00000074276<br>cdna:known chromosome:NCBI36:5:175924960:175955279:1 gene:ENSG00000074276<br>cdna:novel chromosome:NCBI36:5:175908971:175955279:1 gene:ENSG00000074276<br>cdna:Genscan chromosome:NCBI36:5:175902503:175950934:1

		2842668<br>2842669<br>2842670<br>2842671<br>2842672<br>2842673<br>2842674<br>2842675<br>2842676<br>2842677<br>2842678<br>2842679<br>2842680<br>2842681<br>2842682	core<br>core<br>core<br>core<br>full<br>core<br>core<br>core<br>core<br>extended<br>core<br>core<br>extended<br>extended<br>full						
2852591	335493<br>335494<br>335495<br>335496<br>335497<br>335498<br>335499<br>335500<br>335501<br>335502<br>335503<br>335504<br>335505<br>335506<br>335507<br>335508<br>335509<br>335510<br>335511<br>335512<br>335513<br>335514<br>335515<br>335516<br>335517<br>335518<br>335519<br>335520<br>335521<br>335522<br>335523<br>335524<br>335525<br>335526<br>335527<br>335528<br>335529<br>335530<br>335531	2852592<br>2852593<br>2852594<br>2852595<br>2852596<br>2852597<br>2852598<br>2852599<br>2852600<br>2852601<br>2852602<br>2852603<br>2852604<br>2852605<br>2852606<br>2852607<br>2852608<br>2852609<br>2852610<br>2852611<br>2852612<br>2852613<br>2852614<br>2852615<br>2852616<br>2852617<br>2852618<br>2852619<br>2852620<br>2852621<br>2852622<br>2852623<br>2852624<br>2852625<br>2852626<br>2852627<br>2852628<br>2852629<br>2852630	full<br>full<br>full<br>core<br>core<br>extended<br>full<br>full<br>core<br>core<br>full<br>core<br>core<br>extended<br>core<br>full<br>full<br>full<br>full<br>core<br>extended<br>core<br>core<br>extended<br>full<br>extended<br>full<br>core<br>core<br>core<br>full<br>full<br>full<br>core<br>core<br>core<br>core<br>core<br>core	5	33553662	33969872	-	NM_030955<br>AY358745<br>BC058841<br>ENSESTT00000022077<br>ENSESTT00000022078<br>ENSESTT00000022079<br>ENSESTT00000022080<br>ENSESTT00000022081<br>ENSESTT00000022082<br>ENST00000352040<br>GENSCAN00000020009<br>GENSCAN00000040911<br>GENSCAN00000017939	Homo sapiens ADAM metallopeptidase with thrombospondin type 1 motif, 12 (ADAMTS12), mRNA.<br>Homo sapiens clone DNA94830 ADAMTS12 (UNQ1918) mRNA, complete cds.<br>Homo sapiens ADAM metallopeptidase with thrombospondin type 1 motif, 12, mRNA (cDNA clone IMAGE:6701691), complete cds.<br><br><br><br><br><br><br>cdna:known chromosome:NCBI36:5:33563043:33928054:-1 gene:ENSG00000151388<br>cdna:Genscan chromosome:NCBI36:5:33621501:33726048:-1<br>cdna:Genscan chromosome:NCBI36:5:33792045:33832114:-1<br>cdna:Genscan chromosome:NCBI36:5:33553647:33613022:-1

	335532	2852631	core						
	335533	2852632	core						
	335534	2852633	core						
	335535	2852634	full						
	335536	2852635	core						
	335537	2852636	full						
	335538	2852637	extended						
	335539	2852638	full						
	335540	2852639	core						
	335541	2852640	core						
	335542	2852641	full						
	335543	2852642	full						
	335544	2852643	full						
	335545	2852644	full						
	335546	2852645	full						
	335547	2852646	core						
	335548	2852647	core						
	335549	2852648	core						
	335550	2852649	extended						
	335551	2852650	full						
	335552	2852651	full						
	335553	2852652	extended						
	335554	2852653	full						
	335555	2852654	full						
	335556	2852655	extended						
	335557	2852656	full						
	335558	2852657	full						
	335559	2852658	full						
	335560	2852659	extended						
	335561	2852660	extended						
	335562	2852661	extended						
	335563	2852662	full						
	335564	2852663	full						
	335565	2852664	full						
	335566	2852665	full						
	335567	2852666	full						
	335568	2852667	full						
	335569	2852668	full						
		2852669	extended						
		2852670	extended						
		2852671	core						
		2852672	extended						
		2852673	extended						
		2852674	extended						
		2852675	full						
		2852676	extended						
		2852677	core						
		2852678	core						
		2852679	core						
		2852680	full						
		2852681	extended						
		2852682	full						
		2852683	extended						
		2852684	full						

		2852685	full						
2881860	353752<br>353753<br>353754<br>353755<br>353756<br>353757<br>353758<br>353759<br>353760<br>353761<br>353762<br>353763<br>353764<br>353765<br>353766<br>353767<br>353768<br>353769<br>353770<br>353771<br>353772<br>353773<br>353774<br>353775<br>353776	2881861<br>2881862<br>2881863<br>2881864<br>2881865<br>2881866<br>2881867<br>2881868<br>2881869<br>2881870<br>2881871<br>2881872<br>2881873<br>2881874<br>2881875<br>2881876<br>2881877<br>2881878<br>2881879<br>2881880<br>2881881<br>2881882<br>2881883<br>2881884<br>2881885<br>2881886<br>2881887<br>2881888<br>2881889<br>2881890<br>2881891<br>2881892<br>2881893<br>2881894<br>2881895<br>2881896<br>2881897<br>2881898<br>2881899<br>2881900<br>2881901<br>2881902<br>2881903<br>2881904<br>2881905<br>2881906<br>2881907	core<br>core<br>core<br>core<br>core<br>core<br>core<br>core<br>extended<br>extended<br>extended<br>core<br>core<br>extended<br>full<br>full<br>core<br>core<br>core<br>core<br>full<br>core<br>extended<br>extended<br>core<br>extended<br>core<br>core<br>full<br>extended<br>extended<br>full<br>extended<br>extended<br>full<br>full<br>full<br>full<br>full<br>extended<br>full<br>full<br>full<br>full<br>core<br>core<br>full	5	150540816	150583974	-	NM_015621<br>AK094658<br>BC016647<br>BC013053<br>ENSESTT00000041912<br>ENSESTT00000041913<br>ENST00000355417<br>ENST00000356749<br>GENSCAN00000053883	Homo sapiens coiled-coil domain containing 69 (CCDC69), mRNA.<br>Homo sapiens cDNA FLJ37339 fis, clone BRAMY2020520.<br>Homo sapiens coiled-coil domain containing 69, mRNA (cDNA clone MGC:16844 IMAGE:4342371), complete cds.<br>Homo sapiens cDNA clone IMAGE:3881233, **** WARNING: chimeric clone ****.<br><br><br>cdna:known-ccds chromosome:NCBI36:5:150540807:150583847:-1 gene:ENSG00000198624 CCDS4312.1<br>cdna:known chromosome:NCBI36:5:150542805:150583847:-1 gene:ENSG00000198624<br>cdna:Genscan chromosome:NCBI36:5:150580245:150604337:-1
2882834	354346<br>354347<br>354348<br>354349<br>354350	2882835<br>2882836<br>2882837<br>2882838<br>2882839	extended<br>extended<br>extended<br>extended<br>core	5	154177388	154220005	-	NM_032385<br>NM_016348<br>AK127944<br>ENSESTT00000026798<br>ENSESTT00000026799	Homo sapiens chromosome 5 open reading frame 4 (C5orf4), transcript variant 2, mRNA.<br>Homo sapiens chromosome 5 open reading frame 4 (C5orf4), transcript variant 1, mRNA.<br>Homo sapiens cDNA FLJ46054 fis, clone SYNOV4005570.

	354351<br>354352<br>354353<br>354354<br>354355<br>354356<br>354357<br>354358<br>354359<br>354360<br>354361<br>354362<br>354363<br>354364<br>354365<br>354366<br>354367<br>354368<br>354369<br>354370<br>354371	2882840<br>2882841<br>2882842<br>2882843<br>2882844<br>2882845<br>2882846<br>2882847<br>2882848<br>2882849<br>2882850<br>2882851<br>2882852<br>2882853<br>2882854<br>2882855<br>2882856<br>2882857<br>2882858<br>2882859<br>2882860<br>2882861<br>2882862<br>2882863<br>2882864<br>2882865<br>2882866<br>2882867<br>2882868<br>2882869<br>2882870<br>2882871<br>2882872<br>2882873<br>2882874<br>2882875<br>2882876<br>2882877<br>2882878<br>2882879	core<br>core<br>core<br>core<br>core<br>core<br>core<br>core<br>core<br>core<br>core<br>core<br>core<br>core<br>core<br>core<br>extended<br>core<br>core<br>extended<br>extended<br>extended<br>core<br>core<br>full<br>extended<br>extended<br>full<br>core<br>full<br>extended<br>extended<br>full<br>extended<br>full<br>core<br>full<br>full<br>full<br>full					ENSESTT00000026800<br>ENSESTT00000026801<br>ENST00000326080<br>GENSCAN00000027955	 <br>cdna:known chromosome:NCBI36:5:154178247:154210406:-1 gene:ENSG00000170271<br>cdna:Genscan chromosome:NCBI36:5:154180069:154204787:-1
2886174	356456<br>356457<br>356458<br>356459<br>356460<br>356461<br>356462<br>356463<br>356464<br>356465<br>356466<br>356467<br>356468<br>356469	2886175<br>2886176<br>2886177<br>2886178<br>2886179<br>2886180<br>2886181<br>2886182<br>2886183<br>2886184<br>2886185<br>2886186<br>2886187<br>2886188	full<br>full<br>extended<br>extended<br>extended<br>extended<br>extended<br>extended<br>extended<br>extended<br>extended<br>core<br>core<br>core	5	168012858	168660580	-	NM_003062<br>AK098553<br>BC032027<br>BC062365<br>AL122074<br>ENSESTT00000034871<br>ENSESTT00000034872<br>ENSESTT00000034873<br>ENST00000332966<br>ENST00000355963<br>GENSCAN00000029949<br>GENSCAN00000029948<br>GENSCAN00000012807<br>GENSCAN00000004101	Homo sapiens slit homolog 3 (Drosophila) (SLIT3), mRNA.<br>Homo sapiens cDNA FLJ25687 fis, clone TST04292.<br>Homo sapiens cDNA clone IMAGE:4825733.<br>Homo sapiens cDNA clone IMAGE:5922621, partial cds.<br>Homo sapiens mRNA; cDNA DKFZp434N0435 (from clone DKFZp434N0435); partial cds.<br><br><br><br>cdna:known-ccds chromosome:NCBI36:5:168026037:168660291:-1 gene:ENSG00000184347 CCDS4369.1<br>cdna:known chromosome:NCBI36:5:168026291:168660291:-1 gene:ENSG00000184347<br>cdna:Genscan chromosome:NCBI36:5:168202378:168245913:-1<br>cdna:Genscan chromosome:NCBI36:5:168266927:168321825:-1<br>cdna:Genscan chromosome:NCBI36:5:168583391:168611041:-1<br>cdna:Genscan chromosome:NCBI36:5:168649123:168660291:-1

	356470	2886189	core					GENSCAN00000028369	cdna:Genscan chromosome:NCBI36:5:168012858:168149494:-1
	356471	2886190	core					GENSCAN00000067587	cdna:Genscan chromosome:NCBI36:5:168410476:168493065:-1
	356472	2886191	core						
	356473	2886192	core						
	356474	2886193	extended						
	356475	2886194	extended						
	356476	2886195	extended						
	356477	2886196	core						
	356478	2886197	core						
	356479	2886198	core						
	356480	2886199	core						
	356481	2886200	core						
	356482	2886201	core						
	356483	2886202	core						
	356484	2886203	full						
	356485	2886204	full						
	356486	2886205	full						
	356487	2886206	full						
	356488	2886207	full						
	356489	2886208	full						
	356490	2886209	extended						
	356491	2886210	extended						
	356492	2886211	extended						
	356493	2886212	extended						
	356494	2886213	core						
	356495	2886214	core						
	356496	2886215	full						
	356497	2886216	full						
	356498	2886217	full						
	356499	2886218	core						
	356500	2886219	full						
	356501	2886220	full						
	356502	2886221	full						
	356503	2886222	full						
	356504	2886223	full						
	356505	2886224	full						
	356506	2886225	core						
	356507	2886226	full						
	356508	2886227	core						
	356509	2886228	core						
	356510	2886229	full						
	356511	2886230	full						
	356512	2886231	core						
	356513	2886232	core						
	356514	2886233	full						
	356515	2886234	core						
	356516	2886235	full						
	356517	2886236	full						
	356518	2886237	extended						
	356519	2886238	full						
	356520	2886239	core						
	356521	2886240	full						
	356522	2886241	full						
	356523	2886242	core						

	356524	2886243	core						
	356525	2886244	extended						
	356526	2886245	core						
	356527	2886246	core						
	356528	2886247	extended						
	356529	2886248	core						
	356530	2886249	full						
	356531	2886250	core						
	356532	2886251	full						
	356533	2886252	full						
	356534	2886253	full						
	356535	2886254	extended						
	356536	2886255	extended						
	356537	2886256	full						
	356538	2886257	full						
	356539	2886258	extended						
	356540	2886259	full						
	356541	2886260	full						
	356542	2886261	full						
	356543	2886262	full						
	356544	2886263	free						
	356545	2886264	core						
	356546	2886265	core						
	356547	2886266	full						
	356548	2886267	full						
	356549	2886268	extended						
	356550	2886269	extended						
	356551	2886270	full						
	356552	2886271	full						
	356553	2886272	full						
	356554	2886273	core						
	356555	2886274	full						
	356556	2886275	full						
	356557	2886276	core						
	356558	2886277	extended						
	356559	2886278	extended						
	356560	2886279	extended						
	356561	2886280	extended						
	356562	2886281	full						
	356563	2886282	full						
	356564	2886283	full						
	356565	2886284	core						
	356566	2886285	full						
	356567	2886286	full						
	356568	2886287	full						
	356569	2886288	full						
	356570	2886289	full						
	356571	2886290	core						
	356572	2886291	full						
	356573	2886292	full						
	356574	2886293	full						
	356575	2886294	full						
	356576	2886295	full						
	356577	2886296	full						

	356578	2886297	core						
	356579	2886298	full						
	356580	2886299	core						
	356581	2886300	full						
	356582	2886301	full						
	356583	2886302	full						
	356584	2886303	full						
	356585	2886304	full						
	356586	2886305	core						
	356587	2886306	extended						
	356588	2886307	extended						
	356589	2886308	full						
	356590	2886309	full						
	356591	2886310	full						
	356592	2886311	full						
	356593	2886312	full						
	356594	2886313	full						
	356595	2886314	full						
	356596	2886315	full						
	356597	2886316	full						
	356598	2886317	extended						
	356599	2886318	full						
	356600	2886319	full						
	356601	2886320	full						
	356602	2886321	full						
	356603	2886322	full						
	356604	2886323	full						
	356605	2886324	core						
	356606	2886325	full						
	356607	2886326	full						
	356608	2886327	full						
	356609	2886328	full						
	356610	2886329	full						
	356611	2886330	full						
	356612	2886331	full						
	356613	2886332	full						
	356614	2886333	extended						
	356615	2886334	extended						
	356616	2886335	full						
	356617	2886336	full						
	356618	2886337	extended						
	356619	2886338	extended						
	356620	2886339	full						
	356621	2886340	extended						
	356622	2886341	full						
	356623	2886342	extended						
	356624	2886343	extended						
	356625	2886344	full						
	356626	2886345	full						
	356627	2886346	full						
	356628	2886347	full						
	356629	2886348	extended						
	356630	2886349	full						
	356631	2886350	extended						

	356632<br>356633	2886351<br>2886352<br>2886353<br>2886354<br>2886355<br>2886356<br>2886357<br>2886358<br>2886359<br>2886360<br>2886361<br>2886362<br>2886363<br>2886364<br>2886365<br>2886366<br>2886367<br>2886368<br>2886369<br>2886370<br>2886371<br>2886372<br>2886373<br>2886374<br>2886375<br>2886376<br>2886377<br>2886378<br>2886379<br>2886380<br>2886381<br>2886382<br>2886383<br>2886384<br>2886385<br>2886386<br>2886387<br>2886388<br>2886389<br>2886390<br>2886391<br>2886392<br>2886393<br>2886394<br>2886395<br>2886396<br>2886397	extended<br>extended<br>extended<br>full<br>extended<br>full<br>extended<br>extended<br>extended<br>full<br>full<br>extended<br>extended<br>extended<br>extended<br>extended<br>extended<br>full<br>extended<br>extended<br>core<br>full<br>full<br>full<br>full<br>full<br>core<br>core<br>core<br>full<br>full<br>extended<br>full<br>full<br>full<br>full<br>full<br>full<br>extended<br>extended<br>extended<br>full<br>full<br>full<br>core<br>core<br>core						
2886679	356791<br>356792<br>356793<br>356794	2886680<br>2886681<br>2886682<br>2886683<br>2886684<br>2886685<br>2886686	core<br>core<br>core<br>core<br>core<br>core<br>core	5	169737753	169749216	-	NM_004137<br>BC025707<br>ENSESTT00000021376<br>ENST00000274629<br>GENSCAN00000006994	Homo sapiens potassium large conductance calcium-activated channel, subfamily M, beta member 1 (KCNMB1), mRNA.<br>Homo sapiens potassium large conductance calcium-activated channel, subfamily M, beta member 1, mRNA (cDNA clone MGC:34483 IMAGE:5224514), complete cds.<br><br>cdna:known-ccds chromosome:NCBI36:5:169737745:169749216:-1 gene:ENSG00000145936 CCDS4373.1<br>cdna:Genscan chromosome:NCBI36:5:169738286:169745011:-1

		2886687	core						
2889916	358697	2889917	core	5	178473474	178717474	-	NM_021599	Homo sapiens ADAM metallopeptidase with thrombospondin type 1 motif, 2 (ADAMTS2), transcript variant 2, mRNA.
	358698	2889918	extended					NM_014244	Homo sapiens ADAM metallopeptidase with thrombospondin type 1 motif, 2 (ADAMTS2), transcript variant 1, mRNA.
	358699	2889919	extended					ENSESTT00000017818	
	358700	2889920	extended					ENSESTT00000017819	
	358701	2889921	extended					ENSESTT00000017820	
	358702	2889922	extended					ENST00000251582	cdna:known-ccds chromosome:NCBI36:5:178473474:178704935:-1 gene:ENSG00000087116 CCDS4444.1
	358703	2889923	extended					ENST00000274609	cdna:known chromosome:NCBI36:5:178510736:178704935:-1 gene:ENSG00000087116
	358704	2889924	extended					GENSCAN00000030552	cdna:Genscan chromosome:NCBI36:5:178702420:178717474:-1
	358705	2889925	core					GENSCAN00000030553	cdna:Genscan chromosome:NCBI36:5:178631866:178639504:-1
	358706	2889926	extended					GENSCAN00000016400	cdna:Genscan chromosome:NCBI36:5:178579985:178580810:-1
	358707	2889927	full					GENSCAN00000016398	cdna:Genscan chromosome:NCBI36:5:178564270:178577858:-1
	358708	2889928	core					GENSCAN00000011848	cdna:Genscan chromosome:NCBI36:5:178473474:178541885:-1
	358709	2889929	extended						
	358710	2889930	extended						
	358711	2889931	full						
	358712	2889932	core						
	358713	2889933	full						
	358714	2889934	core						
	358715	2889935	core						
	358716	2889936	core						
	358717	2889937	full						
	358718	2889938	full						
	358719	2889939	core						
	358720	2889940	full						
	358721	2889941	core						
	358722	2889942	core						
	358723	2889943	core						
	358724	2889944	full						
	358725	2889945	full						
	358726	2889946	core						
	358727	2889947	extended						
	358728	2889948	extended						
	358729	2889949	full						
	358730	2889950	extended						
	358731	2889951	core						
	358732	2889952	core						
	358733	2889953	core						
	358734	2889954	full						
	358735	2889955	core						
	358736	2889956	core						
	358737	2889957	core						
	358738	2889958	full						
	358739	2889959	extended						
	358740	2889960	full						
	358741	2889961	full						
	358742	2889962	full						
	358743	2889963	full						
	358744	2889964	full						
	358745	2889965	full						
	358746	2889966	full						
	358747	2889967	core						
	358748	2889968	full						
	358749	2889969	full						

	358750	2889970	full						
	358751	2889971	full						
	358752	2889972	extended						
	358753	2889973	extended						
	358754	2889974	full						
	358755	2889975	full						
	358756	2889976	extended						
	358757	2889977	full						
	358758	2889978	full						
	358759	2889979	full						
	358760	2889980	core						
	358761	2889981	full						
	358762	2889982	full						
	358763	2889983	full						
	358764	2889984	full						
	358765	2889985	full						
	358766	2889986	full						
	358767	2889987	full						
	358768	2889988	full						
	358769	2889989	full						
	358770	2889990	extended						
	358771	2889991	full						
	358772	2889992	full						
	358773	2889993	full						
	358774	2889994	extended						
	358775	2889995	full						
	358776	2889996	extended						
	358777	2889997	extended						
	358778	2889998	full						
	358779	2889999	full						
	358780	2890000	core						
	358781	2890001	full						
	358782	2890002	full						
	358783	2890003	full						
	358784	2890004	full						
	358785	2890005	full						
		2890006	full						
		2890007	full						
		2890008	full						
		2890009	core						
		2890010	full						
		2890011	full						
		2890012	core						
		2890013	full						
		2890014	full						
		2890015	full						
		2890016	full						
		2890017	full						
		2890018	full						
		2890019	full						
		2890020	full						
		2890021	full						
		2890022	full						
		2890023	full						

2891556	359652<br>359653<br>359654<br>359655<br>359656<br>359657<br>359658<br>359659<br>359660<br>359661<br>359662<br>359663<br>359664<br>359665<br>359666<br>359667<br>359668<br>359669<br>359670<br>359671<br>359672<br>359673<br>359674<br>359675<br>359676<br>359677<br>359678<br>359679<br>359680<br>359681<br>359682<br>359683<br>359684<br>359685<br>359686<br>359687<br>359688<br>359689<br>359690<br>359691<br>359692<br>359693<br>359694<br>359695<br>359696<br>359697<br>359698<br>359699<br>359700<br>359701	2891557<br>2891558<br>2891559<br>2891560<br>2891561<br>2891562<br>2891563<br>2891564<br>2891565<br>2891566<br>2891567<br>2891568<br>2891569<br>2891570<br>2891571<br>2891572<br>2891573<br>2891574<br>2891575<br>2891576<br>2891577<br>2891578<br>2891579<br>2891580<br>2891581<br>2891582<br>2891583<br>2891584<br>2891585<br>2891586<br>2891587<br>2891588<br>2891589<br>2891590<br>2891591<br>2891592<br>2891593<br>2891594<br>2891595<br>2891596<br>2891597<br>2891598<br>2891599<br>2891600<br>2891601<br>2891602<br>2891603<br>2891604<br>2891605<br>2891606<br>2891607<br>2891608<br>2891609<br>2891610	full<br>full<br>full<br>full<br>full<br>full<br>full<br>full<br>full<br>full<br>full<br>full<br>full<br>extended<br>full<br>full<br>full<br>full<br>full<br>full<br>full<br>full<br>full<br>full<br>full<br>full<br>full<br>full<br>full<br>full<br>full<br>full<br>full<br>full<br>full<br>full<br>full<br>full<br>full<br>full<br>full<br>full<br>full<br>full<br>full<br>full<br>full<br>full<br>extended<br>core<br>core<br>core<br>core<br>core	6	1051225	1285512	+	NM_033260<br>ENST00000296839<br>ENST00000380899<br>GENSCAN00000058908<br>GENSCAN00000027934	Homo sapiens forkhead box Q1 (FOXQ1), mRNA.<br>cdna:known-ccds chromosome:NCBI36:6:1257675:1259981:1 gene:ENSG00000164379 CCDS4471.1<br>cdna:known chromosome:NCBI36:6:1257708:1259422:1 gene:ENSG00000164379<br>cdna:Genscan chromosome:NCBI36:6:1257940:1259151:1<br>cdna:Genscan chromosome:NCBI36:6:1211220:1229657:1

		2891611<br>2891612<br>2891613<br>2891614<br>2891615<br>2891616<br>2891617<br>2891618<br>2891619<br>2891620<br>2891621<br>2891622	core<br>core<br>core<br>core<br>core<br>core<br>core<br>full<br>full<br>full<br>full<br>full						
2897899	363651<br>363652<br>363653	2897900<br>2897901<br>2897902<br>2897903<br>2897904<br>2897905<br>2897906<br>2897907<br>2897908<br>2897909<br>2897910<br>2897911<br>2897912<br>2897913<br>2897914<br>2897915<br>2897916<br>2897917<br>2897918	extended<br>core<br>core<br>core<br>core<br>core<br>core<br>core<br>core<br>core<br>core<br>core<br>core<br>core<br>core<br>core<br>core<br>core<br>core	6	21700979	21706814	+	NM_003107<br>X65661<br>ENST00000378570<br>ENST00000244745<br>GENSCAN00000002043	Homo sapiens SRY (sex determining region Y)-box 4 (SOX4), mRNA.<br>H.sapiens Sox-4 mRNA.<br>cdna:known-ccds chromosome:NCBI36:6:21700979:21706829:1 gene:ENSG00000124766 CCDS4547.1<br>cdna:known chromosome:NCBI36:6:21701951:21706826:1 gene:ENSG00000124766<br>cdna:Genscan chromosome:NCBI36:6:21702745:21704169:1
2907671	369267<br>369268<br>369269<br>369270<br>369271<br>369272<br>369273<br>369274<br>369275<br>369276<br>369277<br>369278<br>369279<br>369280<br>369281<br>369282<br>369283<br>369284<br>369285<br>369286<br>369287<br>369288	2907672<br>2907673<br>2907674<br>2907675<br>2907676<br>2907677<br>2907678<br>2907679<br>2907680<br>2907681<br>2907682<br>2907683<br>2907684<br>2907685<br>2907686<br>2907687<br>2907688<br>2907689<br>2907690<br>2907691<br>2907692<br>2907693	core<br>core<br>full<br>full<br>full<br>full<br>full<br>full<br>extended<br>full<br>extended<br>full<br>full<br>full<br>full<br>core<br>extended<br>core<br>core<br>core<br>full<br>core	6	43152004	43237427	+	NM_152880<br>NM_152881<br>NM_152882<br>NM_002821<br>AK093428<br>AK131487<br>BC046109<br>ENSESTT00000056719<br>ENSESTT00000056720<br>ENSESTT00000056721<br>ENSESTT00000056722<br>ENST00000230419<br>ENST00000345201<br>ENST00000352931<br>ENST00000230418<br>ENST00000349241<br>ENST00000325774<br>ENST00000359792<br>GENSCAN00000029527	Homo sapiens PTK7 protein tyrosine kinase 7 (PTK7), transcript variant PTK7-2, mRNA.<br>Homo sapiens PTK7 protein tyrosine kinase 7 (PTK7), transcript variant PTK7-3, mRNA.<br>Homo sapiens PTK7 protein tyrosine kinase 7 (PTK7), transcript variant PTK7-4, mRNA.<br>Homo sapiens PTK7 protein tyrosine kinase 7 (PTK7), transcript variant PTK7-1, mRNA.<br>Homo sapiens cDNA FLJ36109 fis, clone TESTI2021911, weakly similar to TYROSINE-PROTEIN KINASE-LIKE 7 PRECURSOR.<br>Homo sapiens cDNA FLJ16667 fis, clone THYMU2035710, highly similar to TYROSINE-PROTEIN KINASE-LIKE 7 PRECURSOR.<br>Homo sapiens PTK7 protein tyrosine kinase 7, mRNA (cDNA clone IMAGE:5551146), complete cds.<br><br><br><br><br>cdna:known-ccds chromosome:NCBI36:6:43152007:43237435:1 gene:ENSG00000112655 CCDS4884.1<br>cdna:known-ccds chromosome:NCBI36:6:43152007:43237435:1 gene:ENSG00000112655 CCDS4885.1<br>cdna:known-ccds chromosome:NCBI36:6:43152007:43237435:1 gene:ENSG00000112655 CCDS4887.1<br>cdna:known-ccds chromosome:NCBI36:6:43152007:43237435:1 gene:ENSG00000112655 CCDS4888.1<br>cdna:known-ccds chromosome:NCBI36:6:43152007:43237435:1 gene:ENSG00000112655 CCDS4886.1<br>cdna:known chromosome:NCBI36:6:43152007:43237435:1 gene:ENSG00000112655<br>cdna:novel chromosome:NCBI36:6:43152007:43237435:1 gene:ENSG00000112655<br>cdna:Genscan chromosome:NCBI36:6:43152205:43236597:1

	369289<br>369290<br>369291<br>369292<br>369293<br>369294<br>369295<br>369296<br>369297<br>369298<br>369299<br>369300<br>369301<br>369302<br>369303<br>369304<br>369305<br>369306<br>369307	2907694<br>2907695<br>2907696<br>2907697<br>2907698<br>2907699<br>2907700<br>2907701<br>2907702<br>2907703<br>2907704<br>2907705<br>2907706<br>2907707<br>2907708<br>2907709<br>2907710<br>2907711<br>2907712<br>2907713<br>2907714<br>2907715<br>2907716<br>2907717<br>2907718<br>2907719<br>2907720<br>2907721	core<br>core<br>core<br>core<br>core<br>full<br>core<br>core<br>core<br>core<br>core<br>core<br>extended<br>core<br>extended<br>core<br>extended<br>core<br>extended<br>extended<br>core<br>core<br>extended<br>core<br>core<br>core<br>core<br>core						
2909263	370231<br>370232<br>370233<br>370234<br>370235<br>370236<br>370237<br>370238<br>370239<br>370240<br>370241<br>370242<br>370243<br>370244<br>370245<br>370246	2909264<br>2909265<br>2909266<br>2909267<br>2909268<br>2909269<br>2909270<br>2909271<br>2909272<br>2909273<br>2909274<br>2909275<br>2909276<br>2909277<br>2909278<br>2909279<br>2909280<br>2909281<br>2909282<br>2909283<br>2909284<br>2909285<br>2909286	extended<br>core<br>core<br>core<br>core<br>core<br>full<br>core<br>core<br>full<br>core<br>core<br>core<br>core<br>core<br>core<br>core<br>core<br>core<br>core<br>core<br>core<br>core	6	46869051	46915473	+	NM_005588<br>ENSESTT00000019331<br>ENSESTT00000022973<br>ENSESTT00000022974<br>ENST00000230588<br>ENST00000371259<br>ENST00000342681<br>GENSCAN00000006135<br>GENSCAN00000028233<br>GENSCAN00000040891	Homo sapiens meprin A, alpha (PABA peptide hydrolase) (MEP1A), mRNA.<br><br><br><br>cdna:known-ccds chromosome:NCBI36:6:46869085:46915478:1 gene:ENSG00000112818 CCDS4918.1<br>cdna:known chromosome:NCBI36:6:46869086:46915474:1 gene:ENSG00000112818<br>cdna:novel chromosome:NCBI36:9:42328587:42328865:-1 gene:ENSG00000189422<br>cdna:Genscan chromosome:NCBI36:6:46869095:46914832:1<br>cdna:Genscan chromosome:NCBI36:9:43168253:43170505:1<br>cdna:Genscan chromosome:NCBI36:9:44087905:44105500:1
2914777	373674<br>373675	2914778<br>2914779	extended<br>core	6	80770591	80823909	+	NM_003318<br>AK095599	Homo sapiens TTK protein kinase (TTK), mRNA.<br>Homo sapiens cDNA FLJ38280 fis, clone FCBBF3005497, highly similar to DUAL SPECIFICITY PROTEIN KINASE TTK

	373676<br>373677<br>373678<br>373679<br>373680<br>373681<br>373682<br>373683<br>373684<br>373685<br>373686<br>373687<br>373688<br>373689<br>373690<br>373691<br>373692<br>373693<br>373694<br>373695<br>373696<br>373697<br>373698<br>373699<br>373700<br>373701<br>373702<br>373703<br>373704	2914780<br>2914781<br>2914782<br>2914783<br>2914784<br>2914785<br>2914786<br>2914787<br>2914788<br>2914789<br>2914790<br>2914791<br>2914792<br>2914793<br>2914794<br>2914795<br>2914796<br>2914797<br>2914798<br>2914799<br>2914800<br>2914801<br>2914802<br>2914803<br>2914804<br>2914805<br>2914806<br>2914807<br>2914808<br>2914809<br>2914810<br>2914811<br>2914812<br>2914813	core<br>core<br>core<br>core<br>extended<br>core<br>core<br>core<br>core<br>core<br>core<br>core<br>core<br>core<br>extended<br>full<br>core<br>core<br>core<br>full<br>core<br>extended<br>core<br>core<br>core<br>core<br>extended<br>extended<br>core<br>core<br>full<br>extended<br>full<br>full					X70500<br>ENSESTT00000028650<br>ENSESTT00000028651<br>ENSESTT00000028652<br>ENST00000230510<br>ENST00000369798<br>GENSCAN00000051705	(EC 2.7.1.-).<br>H.sapiens mRNA for phosphotyrosine picked threonine kinase (PYT).<br><br><br><br>cdna:known-ccds chromosome:NCBI36:6:80771078:80808956:1 gene:ENSG00000112742 CCDS4993.1<br>cdna:known chromosome:NCBI36:6:80771076:80808958:1 gene:ENSG00000112742<br>cdna:Genscan chromosome:NCBI36:6:80772280:80807139:1
2923868	379326<br>379327<br>379328<br>379329<br>379330<br>379331<br>379332<br>379333<br>379334<br>379335<br>379336<br>379337<br>379338<br>379339<br>379340<br>379341<br>379342<br>379343<br>379344<br>379345	2923869<br>2923870<br>2923871<br>2923872<br>2923873<br>2923874<br>2923875<br>2923876<br>2923877<br>2923878<br>2923879<br>2923880<br>2923881<br>2923882<br>2923883<br>2923884<br>2923885<br>2923886<br>2923887<br>2923888	core<br>core<br>extended<br>extended<br>extended<br>extended<br>full<br>extended<br>core<br>extended<br>extended<br>extended<br>full<br>core<br>full<br>full<br>extended<br>extended<br>full<br>core	6	122834771	123120381	+	NM_181795<br>NM_181794<br>NM_032471<br>AF225513<br>AK026221<br>AK074397<br>AJ420562<br>CR749456<br>ENSESTT00000010766<br>ENSESTT00000010767<br>ENST00000368452<br>ENST00000368448<br>ENST00000368451<br>ENST00000258014<br>ENST00000354275<br>ENST00000368446<br>GENSCAN00000056024<br>GENSCAN00000056025	Homo sapiens protein kinase (cAMP-dependent, catalytic) inhibitor beta (PKIB), transcript variant 1, mRNA.<br>Homo sapiens protein kinase (cAMP-dependent, catalytic) inhibitor beta (PKIB), transcript variant 2, mRNA.<br>Homo sapiens protein kinase (cAMP-dependent, catalytic) inhibitor beta (PKIB), transcript variant 3, mRNA.<br>Homo sapiens cAMP-dependent protein kinase inhibitor beta mRNA, complete cds.<br>Homo sapiens cDNA: FLJ22568 fis, clone HSI02138.<br>Homo sapiens cDNA FLJ23817 fis, clone HSI07950.<br>Homo sapiens mRNA full length insert cDNA clone EUROIMAGE 1525273.<br>Homo sapiens mRNA; cDNA DKFZp781K1114 (from clone DKFZp781K1114).<br><br><br>cdna:known-ccds chromosome:NCBI36:6:122973076:123089216:1 gene:ENSG00000135549 CCDS5126.1<br>cdna:known chromosome:NCBI36:6:122973076:123088736:1 gene:ENSG00000135549<br>cdna:known chromosome:NCBI36:6:122973076:123088736:1 gene:ENSG00000135549<br>cdna:known chromosome:NCBI36:6:123015576:123089217:1 gene:ENSG00000135549<br>cdna:known chromosome:NCBI36:6:123080405:123088736:1 gene:ENSG00000135549<br>cdna:known chromosome:NCBI36:6:123080406:123088125:1 gene:ENSG00000135549<br>cdna:Genscan chromosome:NCBI36:6:122972795:123005538:1<br>cdna:Genscan chromosome:NCBI36:6:123063926:123088039:1

	379346<br>379347<br>379348<br>379349<br>379350<br>379351<br>379352<br>379353<br>379354<br>379355<br>379356<br>379357<br>379358<br>379359<br>379360<br>379361<br>379362<br>379363<br>379364<br>379365<br>379366<br>379367<br>379368<br>379369	2923889<br>2923890<br>2923891<br>2923892<br>2923893<br>2923894<br>2923895<br>2923896<br>2923897<br>2923898<br>2923899<br>2923900<br>2923901<br>2923902<br>2923903<br>2923904<br>2923905<br>2923906<br>2923907<br>2923908<br>2923909<br>2923910<br>2923911<br>2923912<br>2923913<br>2923914<br>2923915<br>2923916<br>2923917<br>2923918<br>2923919<br>2923920<br>2923921<br>2923922<br>2923923<br>2923924	extended<br>extended<br>extended<br>full<br>full<br>full<br>extended<br>extended<br>extended<br>extended<br>extended<br>extended<br>extended<br>extended<br>core<br>extended<br>extended<br>extended<br>extended<br>full<br>full<br>full<br>extended<br>full<br>extended<br>extended<br>core<br>full<br>core<br>core<br>core<br>extended<br>extended<br>full<br>full<br>full						
2924514	379763<br>379764<br>379765<br>379766<br>379767<br>379768<br>379769<br>379770<br>379771<br>379772<br>379773<br>379774<br>379775<br>379776<br>379777<br>379778<br>379779<br>379780	2924515<br>2924516<br>2924517<br>2924518<br>2924519<br>2924520<br>2924521<br>2924522<br>2924523<br>2924524<br>2924525<br>2924526<br>2924527<br>2924528<br>2924529<br>2924530<br>2924531<br>2924532	core<br>extended<br>extended<br>core<br>extended<br>core<br>full<br>extended<br>extended<br>extended<br>extended<br>extended<br>core<br>core<br>full<br>extended<br>extended<br>extended	6	126144000	126294859	+	NM_181782<br>AB074157<br>AK094706<br>AK127512<br>BC071782<br>AL834442<br>ENSESTT00000024648<br>ENSESTT00000024649<br>ENSESTT00000024650<br>ENSESTT00000024651<br>ENSESTT00000024652<br>ENSESTT00000024653<br>ENSESTT00000024654<br>ENSESTT00000024656<br>ENST00000368357<br>ENST00000355470<br>ENST00000368353<br>ENST00000368351	Homo sapiens nuclear receptor coactivator 7 (NCOA7), mRNA.<br>Homo sapiens primary neuroblastoma cDNA, clone:Nbla10993, full insert sequence.<br>Homo sapiens cDNA FLJ37387 fis, clone BRAMY2026685, moderately similar to Homo sapiens oxidation protection protein (OXR1) mRNA.<br>Homo sapiens cDNA FLJ45605 fis, clone BRTHA3021971, moderately similar to Homo sapiens oxidation resistance 1 (OXR1).<br>Homo sapiens nuclear receptor coactivator 7, mRNA (cDNA clone IMAGE:4609474), complete cds.<br>Homo sapiens mRNA; cDNA DKFZp761B2210 (from clone DKFZp761B2210).<br>cdna:known-ccds chromosome:NCBI36:6:126144000:126293950:1 gene:ENSG00000111912 CCDS5132.1<br>cdna:known chromosome:NCBI36:6:126144000:126293949:1 gene:ENSG00000111912<br>cdna:known chromosome:NCBI36:6:126262835:126293949:1 gene:ENSG00000111912

	379781	2924533	extended					ENST00000229634	cdna:known chromosome:NCBI36:6:126282145:126293947:1 gene:ENSG00000111912
	379782	2924534	extended					GENSCAN00000001622	cdna:known chromosome:NCBI36:6:126282171:126293947:1 gene:ENSG00000111912
	379783	2924535	extended					GENSCAN00000062579	cdna:Genscan chromosome:NCBI36:6:126290502:126291610:1
	379784	2924536	extended						cdna:Genscan chromosome:NCBI36:6:126217859:126285673:1
	379785	2924537	extended						
	379786	2924538	full						
	379787	2924539	extended						
	379788	2924540	full						
	379789	2924541	core						
	379790	2924542	core						
	379791	2924543	core						
	379792	2924544	full						
	379793	2924545	full						
	379794	2924546	extended						
	379795	2924547	extended						
	379796	2924548	full						
	379797	2924549	extended						
	379798	2924550	full						
	379799	2924551	core						
	379800	2924552	full						
	379801	2924553	core						
	379802	2924554	core						
	379803	2924555	core						
	379804	2924556	extended						
	379805	2924557	core						
	379806	2924558	extended						
	379807	2924559	extended						
	379808	2924560	extended						
	379809	2924561	core						
	379810	2924562	core						
	379811	2924563	core						
	379812	2924564	core						
	379813	2924565	core						
	379814	2924566	core						
	379815	2924567	core						
	379816	2924568	full						
	379817	2924569	extended						
	379818	2924570	extended						
		2924571	extended						
		2924572	full						
		2924573	full						
		2924574	extended						
		2924575	full						
		2924576	extended						
		2924577	full						
		2924578	full						
		2924579	core						
		2924580	extended						
		2924581	extended						
		2924582	extended						
		2924583	extended						
		2924584	core						
		2924585	core						
		2924586	extended						

		2924587<br>2924588<br>2924589<br>2924590<br>2924591<br>2924592<br>2924593<br>2924594<br>2924595<br>2924596<br>2924597	extended<br>core<br>extended<br>core<br>extended<br>core<br>core<br>core<br>core<br>core<br>extended						
2931391	384141<br>384142<br>384143<br>384144<br>384145<br>384146<br>384147<br>384148<br>384149<br>384150<br>384151<br>384152<br>384153<br>384154<br>384155<br>384156<br>384157<br>384158<br>384159<br>384160<br>384161<br>384162<br>384163<br>384164<br>384165<br>384166<br>384167<br>384168<br>384169<br>384170<br>384171<br>384172<br>384173<br>384174<br>384175<br>384176<br>384177<br>384178<br>384179<br>384180<br>384181<br>384182<br>384183	2931392<br>2931393<br>2931394<br>2931395<br>2931396<br>2931397<br>2931398<br>2931399<br>2931400<br>2931401<br>2931402<br>2931403<br>2931404<br>2931405<br>2931406<br>2931407<br>2931408<br>2931409<br>2931410<br>2931411<br>2931412<br>2931413<br>2931414<br>2931415<br>2931416<br>2931417<br>2931418<br>2931419<br>2931420<br>2931421<br>2931422<br>2931423<br>2931424<br>2931425<br>2931426<br>2931427<br>2931428<br>2931429<br>2931430<br>2931431<br>2931432<br>2931433<br>2931434	core<br>core<br>core<br>core<br>full<br>full<br>full<br>extended<br>extended<br>extended<br>extended<br>extended<br>extended<br>core<br>core<br>core<br>extended<br>core<br>full<br>core<br>core<br>core<br>core<br>full<br>core<br>full<br>extended<br>core<br>full<br>full<br>full<br>extended<br>extended<br>extended<br>core<br>extended<br>core<br>full<br>full<br>core<br>extended<br>extended<br>extended	6	151228378	151579807	+	XM_945232<br>XM_941339<br>XM_945148<br>XM_927886<br>XM_932383<br>NM_015440<br>XM_933400<br>AK127089<br>AY374131<br>AF086425<br>ENSESTT00000035467<br>ENSESTT00000036221<br>ENSESTT00000036222<br>ENSESTT00000036223<br>ENSESTT00000036224<br>ENSESTT00000036225<br>ENSESTT00000036226<br>ENSESTT00000036227<br>ENSESTT00000036228<br>ENST00000367321<br>ENST00000265365<br>ENST00000367310<br>ENST00000367308<br>ENST00000367307<br>GENSCAN00000010791<br>GENSCAN00000056713<br>GENSCAN00000068915<br>GENSCAN00000068917<br>GENSCAN00000068919<br>GENSCAN00000066827	PREDICTED: Homo sapiens hypothetical protein LOC652160 (LOC652160), mRNA.<br>PREDICTED: Homo sapiens similar to ADP-ribosylation factor-like protein 4A, transcript variant 1 (LOC649604), mRNA.<br>PREDICTED: Homo sapiens similar to ADP-ribosylation factor-like protein 4A, transcript variant 2 (LOC649604), mRNA.<br>PREDICTED: Homo sapiens similar to ADP-ribosylation factor-like protein 4A, transcript variant 1 (LOC643366), mRNA.<br>PREDICTED: Homo sapiens similar to ADP-ribosylation factor-like protein 4A, transcript variant 2 (LOC643366), mRNA.<br>Homo sapiens methylenetetrahydrofolate dehydrogenase (NADP+ dependent) 1-like (MTHFD1L), mRNA.<br>PREDICTED: Homo sapiens hypothetical protein LOC646099 (LOC646099), mRNA.<br>Homo sapiens cDNA FLJ45146 fis, clone BRAWH3041556, moderately similar to C-1-tetrahydrofolate synthase, cytoplasmic.<br>Homo sapiens truncated C1-tetrahydrofolate synthase mRNA, complete cds; nuclear gene for mitochondrial product; alternatively spliced.<br>Homo sapiens full length insert cDNA clone ZD78H09.<br>cdna:known-ccds chromosome:NCBI36:6:151228378:151464716:1 gene:ENSG00000120254 CCDS5228.1<br>cdna:known chromosome:NCBI36:6:151228384:151464714:1 gene:ENSG00000120254<br>cdna:known chromosome:NCBI36:6:151228384:151404669:1 gene:ENSG00000120254<br>cdna:known chromosome:NCBI36:6:151228384:151275534:1 gene:ENSG00000120254<br>cdna:known chromosome:NCBI36:6:151228545:151261850:1 gene:ENSG00000120254<br>cdna:Genscan chromosome:NCBI36:10:62114269:62114973:1<br>cdna:Genscan chromosome:NCBI36:6:151240481:151281497:1<br>cdna:Genscan chromosome:NCBI36:6:151464699:151483691:1<br>cdna:Genscan chromosome:NCBI36:6:151447087:151447224:1<br>cdna:Genscan chromosome:NCBI36:6:151397322:151417459:1<br>cdna:Genscan chromosome:NCBI36:6:151514685:151548268:1

	384184	2931435	extended							
	384185	2931436	extended							
	384186	2931437	extended							
	384187	2931438	extended							
	384188	2931439	full							
	384189	2931440	core							
	384190	2931441	core							
	384191	2931442	full							
	384192	2931443	full							
	384193	2931444	full							
	384194	2931445	extended							
	384195	2931446	core							
	384196	2931447	core							
	384197	2931448	core							
	384198	2931449	core							
	384199	2931450	core							
	384200	2931451	core							
	384201	2931452	full							
	384202	2931453	extended							
	384203	2931454	full							
	384204	2931455	full							
	384205	2931456	core							
	384206	2931457	full							
	384207	2931458	full							
	384208	2931459	core							
	384209	2931460	full							
	384210	2931461	extended							
	384211	2931462	full							
	384212	2931463	full							
	384213	2931464	extended							
	384214	2931465	extended							
	384215	2931466	extended							
	384216	2931467	extended							
	384217	2931468	extended							
	384218	2931469	extended							
	384219	2931470	extended							
	384220	2931471	extended							
	384221	2931472	full							
	384222	2931473	extended							
	384223	2931474	extended							
	384224	2931475	extended							
	384225	2931476	full							
	384226	2931477	full							
	384227	2931478	core							
	384228	2931479	extended							
	384229	2931480	extended							
	384230	2931481	core							
	384231	2931482	full							
	384232	2931483	core							
	384233	2931484	core							
	384234	2931485	full							
	384235	2931486	full							
	384236	2931487	extended							
	384237	2931488	full							

	384238<br>384239<br>384240<br>384241<br>384242<br>384243<br>384244<br>384245<br>384246<br>384247<br>384248<br>384249<br>384250<br>384251<br>384252<br>384253<br>384254<br>384255<br>384256<br>384257<br>384258	2931489<br>2931490<br>2931491<br>2931492<br>2931493<br>2931494<br>2931495<br>2931496<br>2931497<br>2931498<br>2931499<br>2931500<br>2931501<br>2931502<br>2931503<br>2931504<br>2931505<br>2931506<br>2931507<br>2931508<br>2931509<br>2931510<br>2931511<br>2931512<br>2931513<br>2931514<br>2931515<br>2931516<br>2931517<br>2931518<br>2931519<br>2931520<br>2931521<br>2931522<br>2931523<br>2931524<br>2931525<br>2931526<br>2931527<br>2931528<br>2931529<br>2931530<br>2931531<br>2931532<br>2931533<br>2931534<br>2931535<br>2931536<br>2931537<br>2931538<br>2931539<br>2931540	core<br>full<br>core<br>full<br>full<br>full<br>extended<br>extended<br>full<br>full<br>full<br>extended<br>extended<br>extended<br>extended<br>extended<br>extended<br>extended<br>full<br>extended<br>extended<br>core<br>core<br>extended<br>extended<br>core<br>extended<br>extended<br>full<br>full<br>full<br>full<br>full<br>full<br>full<br>full<br>full<br>full<br>full<br>full<br>full<br>full<br>full<br>full<br>full<br>full<br>full<br>full<br>full<br>full<br>full<br>full						
2946106	393528<br>393529	2946107<br>2946108	extended<br>extended	6	25941273	25990602	-	NM_005074<br>NM_006632	Homo sapiens solute carrier family 17 (sodium phosphate), member 1 (SLC17A1), mRNA.<br>Homo sapiens solute carrier family 17 (sodium phosphate), member 3 (SLC17A3), mRNA.

	393530<br>393531<br>393532<br>393533<br>393534<br>393535<br>393536<br>393537<br>393538<br>393539<br>393540<br>393541<br>393542<br>393543<br>393544<br>393545<br>393546<br>393547<br>393548<br>393549<br>393550<br>393551<br>393552	2946109<br>2946110<br>2946111<br>2946112<br>2946113<br>2946114<br>2946115<br>2946116<br>2946117<br>2946118<br>2946119<br>2946120<br>2946121<br>2946122<br>2946123<br>2946124<br>2946125<br>2946126<br>2946127<br>2946128<br>2946129<br>2946130<br>2946131<br>2946132<br>2946133<br>2946134<br>2946135<br>2946136<br>2946137<br>2946138<br>2946139<br>2946140<br>2946141	full<br>full<br>extended<br>extended<br>core<br>core<br>core<br>full<br>core<br>core<br>extended<br>core<br>core<br>extended<br>extended<br>full<br>core<br>full<br>full<br>core<br>extended<br>core<br>core<br>core<br>core<br>full<br>extended<br>extended<br>extended<br>core<br>core<br>extended<br>extended					BC017952<br>BC101745<br>Z83953<br>ENSESTT00000049090<br>ENST00000244527<br>ENST00000362070<br>ENST00000377886<br>ENST00000360657<br>ENST00000361703<br>ENST00000308453<br>GENSCAN00000040440	Homo sapiens solute carrier family 17 (sodium phosphate), member 3, mRNA (cDNA clone MGC:24061 IMAGE:4557795), complete cds.<br>Homo sapiens solute carrier family 17 (sodium phosphate), member 1, mRNA (cDNA clone MGC:126794 IMAGE:8069251), complete cds.<br>H.sapiens mRNA; clone CD 274.<br><br>cdna:known-ccds chromosome:NCBI36:6:25891296:25938776:-1 gene:ENSG00000124568 CCDS4565.1<br>cdna:known-ccds chromosome:NCBI36:6:25953312:25990501:-1 gene:ENSG00000124564 CCDS4566.1<br>cdna:known chromosome:NCBI36:6:25891104:25938776:-1 gene:ENSG00000124568<br>cdna:known chromosome:NCBI36:6:25953307:25990493:-1 gene:ENSG00000124564<br>cdna:known chromosome:NCBI36:6:25953312:25990501:-1 gene:ENSG00000124564<br>cdna:known chromosome:NCBI36:6:25953312:25970212:-1 gene:ENSG00000124564<br>cdna:Genscan chromosome:NCBI36:6:25906992:25976594:-1
2949622	395413<br>395414<br>395415<br>395416<br>395417<br>395418<br>395419<br>395420<br>395421<br>395422<br>395423<br>395424<br>395425<br>395426<br>395427<br>395428<br>395429<br>395430<br>395431<br>395432<br>395433	2949623<br>2949624<br>2949625<br>2949626<br>2949627<br>2949628<br>2949629<br>2949630<br>2949631<br>2949632<br>2949633<br>2949634<br>2949635<br>2949636<br>2949637<br>2949638<br>2949639<br>2949640<br>2949641<br>2949642<br>2949643	core<br>core<br>core<br>full<br>core<br>core<br>core<br>full<br>core<br>core<br>core<br>core<br>core<br>core<br>core<br>full<br>core<br>core<br>core<br>core<br>core	6	32116919	32204045	-	NM_019105<br>NM_032470<br>NM_004381<br>NR_001284<br>BC008394<br>BC071883<br>Y17865<br>Y17867<br>Y17868<br>X71923<br>U52696<br>U52700<br>ENSESTT00000021515<br>ENSESTT00000021516<br>ENSESTT00000021517<br>ENSESTT00000021518<br>ENSESTT00000021519<br>ENSESTT00000021520<br>ENSESTT00000021521<br>ENSESTT00000021522<br>ENSESTT00000021523	Homo sapiens tenascin XB (TNXB), transcript variant XB, mRNA.<br>Homo sapiens tenascin XB (TNXB), transcript variant XB-S, mRNA.<br>Homo sapiens cAMP responsive element binding protein-like 1 (CREBL1), mRNA.<br>Homo sapiens tenascin XA pseudogene (TNXA) on chromosome 6.<br>Homo sapiens cAMP responsive element binding protein-like 1, mRNA (cDNA clone IMAGE:4296455), complete cds.<br>Homo sapiens tenascin XB, mRNA (cDNA clone IMAGE:6571962), partial cds.<br>Homo sapiens mRNA for tenascin-X (partial), fibronectin type III repeats ho-h4.<br>Homo sapiens mRNA for tenascin-X (partial), fibronectin type III repeats h13-h16 and h13a.<br>Homo sapiens mRNA for tenascin-X (partial), fibronectin type III repeats ho-h4.<br>H.sapiens XB gene for tenascin-X, exons 1 & 2.<br>Human adrenal Creb-rp homolog (Creb-rp), complete cds, and tenascin-X (XB), partial cds, mRNA.<br>Human tenascin-X (XB) mRNA, RACE clone N1, partial cds.

	395434	2949644	core					ENSESTT00000021524	
	395435	2949645	core					ENSESTT00000021525	
	395436	2949646	core					ENSESTT00000021526	
	395437	2949647	core					ENSESTT00000021527	
	395438	2949648	extended					ENSESTT00000021528	
	395439	2949649	extended					ENSESTT00000021529	
	395440	2949650	full					ENSESTT00000021530	
	395441	2949651	core					ENST00000342991	cdna:known-ccds chromosome:NCBI36:6:32116909:32121883:-1 gene:ENSG00000168477 CCDS4736.1
	395442	2949652	core					ENST00000375203	cdna:known-ccds chromosome:NCBI36:6:32191023:32204008:-1 gene:ENSG00000168477 CCDS4737.1
	395443	2949653	core					ENST00000383160	cdna:known chromosome:NCBI36:c6_COX:32113049:32137454:-1 gene:ENSG00000206258
	395444	2949654	core					ENST00000383159	cdna:known chromosome:NCBI36:c6_COX:32121537:32152869:-1 gene:ENSG00000206258
	395445	2949655	full					ENST00000383158	cdna:known chromosome:NCBI36:c6_COX:32121537:32152842:-1 gene:ENSG00000206258
	395446	2949656	core					ENST00000383157	cdna:known chromosome:NCBI36:c6_COX:32161665:32178823:-1 gene:ENSG00000168468
	395447	2949657	core					ENST00000293709	cdna:known chromosome:NCBI36:c6_COX:32178759:32191727:-1 gene:ENSG00000168468
	395448	2949658	core					ENST00000383156	cdna:known chromosome:NCBI36:c6_COX:32178759:32191727:-1 gene:ENSG00000168468
	395449	2949659	full					ENST00000383317	cdna:known chromosome:NCBI36:c6_QBL:32109441:32116266:-1 gene:ENSG00000206336
	395450	2949660	core					ENST00000383316	cdna:known chromosome:NCBI36:c6_QBL:32125144:32133346:-1 gene:ENSG00000206333
	395451	2949661	core					ENST00000383313	cdna:known chromosome:NCBI36:c6_QBL:32133367:32149681:-1 gene:ENSG00000206332
	395452	2949662	extended					ENST00000375247	cdna:known chromosome:NCBI36:6:32084175:32185131:-1 gene:ENSG00000168477
	395453	2949663	core					ENST00000375244	cdna:known chromosome:NCBI36:6:32116912:32185129:-1 gene:ENSG00000168477
	395454	2949664	core					ENST00000299669	cdna:known chromosome:NCBI36:6:32117105:32185131:-1 gene:ENSG00000168477
	395455	2949665	core					ENST00000308994	cdna:known chromosome:NCBI36:6:32133814:32165140:-1 gene:ENSG00000168477
	395456	2949666	full					ENST00000375207	cdna:known chromosome:NCBI36:6:32173931:32191089:-1 gene:ENSG00000168477
	395457	2949667	core					ENST00000375201	cdna:known chromosome:NCBI36:6:32191023:32204008:-1 gene:ENSG00000168477
	395458	2949668	core					ENST00000375195	cdna:known chromosome:NCBI36:6:32191025:32203995:-1 gene:ENSG00000168477
	395459	2949669	core					ENST00000375192	cdna:known chromosome:NCBI36:6:32191025:32203995:-1 gene:ENSG00000168477
	395460	2949670	core					ENST00000375190	cdna:known chromosome:NCBI36:6:32196197:32204008:-1 gene:ENSG00000168477
	395461	2949671	core					ENST00000359300	cdna:known chromosome:NCBI36:6:32086545:32088297:-1 gene:ENSG00000198493
	395462	2949672	core					ENST00000383161	cdna:novel chromosome:NCBI36:c6_COX:32107318:32108874:-1 gene:ENSG00000206259
	395463	2949673	extended					GENSCAN00000045300	cdna:Genscan chromosome:NCBI36:c6_QBL:32109636:32155653:-1
	395464	2949674	core					GENSCAN00000024658	cdna:Genscan chromosome:NCBI36:6:32084369:32088120:-1
	395465	2949675	core					GENSCAN00000024674	cdna:Genscan chromosome:NCBI36:6:32117105:32158081:-1
	395466	2949676	full					GENSCAN00000042608	cdna:Genscan chromosome:NCBI36:6:32160168:32164803:-1
	395467	2949677	core						
	395468	2949678	core						
	395469	2949679	full						
	395470	2949680	core						
	395471	2949681	core						
	395472	2949682	core						
	395473	2949683	core						
	395474	2949684	core						
	395475	2949685	extended						
	395476	2949686	core						
	395477	2949687	extended						
	395478	2949688	core						
	395479	2949689	full						
	395480	2949690	core						
	395481	2949691	core						
	395482	2949692	full						
	395483	2949693	core						
	395484	2949694	full						
	395485	2949695	core						
	395486	2949696	core						
	395487	2949697	core						

	395488	2949698	extended						
	395489	2949699	core						
		2949700	core						
		2949701	full						
		2949702	full						
		2949703	core						
		2949704	core						
		2949705	core						
		2949706	full						
		2949707	full						
		2949708	core						
		2949709	core						
		2949710	extended						
		2949711	extended						
		2949712	extended						
		2949713	extended						
		2949714	core						
		2949715	core						
		2949716	core						
		2949717	core						
		2949718	core						
		2949719	core						
		2949720	extended						
		2949721	core						
		2949722	core						
		2949723	core						
		2949724	core						
		2949725	full						
		2949726	full						
		2949727	extended						
		2949728	core						
		2949729	core						
		2949730	core						
		2949731	extended						
		2949732	core						
		2949733	core						
		2949734	core						
		2949735	core						
		2949736	core						
		2949737	core						
		2949738	extended						
		2949739	core						
		2949740	extended						
		2949741	core						
		2949742	core						
		2949743	core						
		2949744	extended						
		2949745	core						
		2949746	core						
		2949747	core						
		2949748	core						
		2949749	extended						
		2949750	core						
		2949751	extended						

		2949752<br>2949753<br>2949754<br>2949755<br>2949756<br>2949757<br>2949758<br>2949759	core<br>core<br>core<br>core<br>core<br>full<br>core<br>extended						
2951674	396593<br>396594<br>396595<br>396596<br>396597<br>396598<br>396599<br>396600<br>396601<br>396602<br>396603<br>396604<br>396605<br>396606<br>396607<br>396608<br>396609<br>396610<br>396611<br>396612<br>396613<br>396614<br>396615<br>396616<br>396617<br>396618<br>396619<br>396620<br>396621<br>396622<br>396623<br>396624<br>396625<br>396626<br>396627<br>396628<br>396629<br>396630	2951675<br>2951676<br>2951677<br>2951678<br>2951679<br>2951680<br>2951681<br>2951682<br>2951683<br>2951684<br>2951685<br>2951686<br>2951687<br>2951688<br>2951689<br>2951690<br>2951691<br>2951692<br>2951693<br>2951694<br>2951695<br>2951696<br>2951697<br>2951698<br>2951699<br>2951700<br>2951701<br>2951702<br>2951703<br>2951704<br>2951705<br>2951706<br>2951707<br>2951708<br>2951709<br>2951710<br>2951711<br>2951712<br>2951713<br>2951714<br>2951715<br>2951716<br>2951717<br>2951718<br>2951719<br>2951720	extended<br>core<br>core<br>extended<br>extended<br>full<br>extended<br>extended<br>full<br>core<br>extended<br>extended<br>core<br>extended<br>extended<br>extended<br>full<br>extended<br>extended<br>extended<br>core<br>extended<br>core<br>core<br>extended<br>core<br>core<br>core<br>core<br>core<br>core<br>extended<br>core<br>full<br>core<br>extended<br>extended<br>core<br>extended<br>core<br>core<br>core<br>full<br>extended<br>extended<br>extended	6	35908741	35996942	-	NM_003137<br>AJ318054<br>AL117648<br>ENSESTT00000038413<br>ENSESTT00000038414<br>ENSESTT00000038415<br>ENST00000361690<br>ENST00000373825<br>ENST00000373822<br>ENST00000373821<br>ENST00000346162<br>GENSCAN00000000063	Homo sapiens SFRS protein kinase 1 (SRPK1), mRNA.<br>Homo sapiens mRNA for SRPK1a protein kinase (SRPK1 gene).<br>Homo sapiens mRNA; cDNA DKFZp434P155 (from clone DKFZp434P155).<br><br><br><br>cdna:known chromosome:NCBI36:6:35908789:35996942:-1 gene:ENSG00000096063<br>cdna:known chromosome:NCBI36:6:35908789:35996942:-1 gene:ENSG00000096063<br>cdna:known chromosome:NCBI36:6:35908789:35996934:-1 gene:ENSG00000096063<br>cdna:known chromosome:NCBI36:6:35908789:35996820:-1 gene:ENSG00000096063<br>cdna:known chromosome:NCBI36:6:35908790:35996811:-1 gene:ENSG00000096063<br>cdna:Genscan chromosome:NCBI36:6:35911059:35996811:-1

		2951721<br>2951722<br>2951723<br>2951724<br>2951725<br>2951726	extended<br>full<br>core<br>extended<br>extended<br>core						
2954678	398395<br>398396<br>398397<br>398398<br>398399<br>398400<br>398401<br>398402<br>398403<br>398404<br>398405<br>398406<br>398407<br>398408<br>398409<br>398410<br>398411<br>398412<br>398413<br>398414<br>398415<br>398416<br>398417<br>398418<br>398419<br>398420<br>398421<br>398422<br>398423<br>398424<br>398425<br>398426<br>398427<br>398428<br>398429<br>398430<br>398431<br>398432<br>398433<br>398434<br>398435<br>398436<br>398437<br>398438<br>398439<br>398440<br>398441<br>398442	2954679<br>2954680<br>2954681<br>2954682<br>2954683<br>2954684<br>2954685<br>2954686<br>2954687<br>2954688<br>2954689<br>2954690<br>2954691<br>2954692<br>2954693<br>2954694<br>2954695<br>2954696<br>2954697<br>2954698<br>2954699<br>2954700<br>2954701<br>2954702<br>2954703<br>2954704<br>2954705<br>2954706<br>2954707<br>2954708<br>2954709<br>2954710<br>2954711<br>2954712<br>2954713<br>2954714<br>2954715<br>2954716<br>2954717<br>2954718<br>2954719<br>2954720<br>2954721<br>2954722<br>2954723<br>2954724<br>2954725<br>2954726	extended<br>core<br>core<br>core<br>core<br>core<br>core<br>core<br>core<br>core<br>core<br>extended<br>core<br>extended<br>core<br>extended<br>extended<br>core<br>core<br>core<br>extended<br>core<br>core<br>extended<br>extended<br>core<br>extended<br>full<br>core<br>extended<br>core<br>core<br>full<br>extended<br>extended<br>extended<br>extended<br>extended<br>extended<br>core<br>extended<br>extended<br>core<br>core<br>core<br>core<br>core<br>core	6	43596295	43651746	-	NM_020750<br>AK127513<br>ENSESTT00000056778<br>ENSESTT00000056779<br>ENSESTT00000056780<br>ENSESTT00000056781<br>ENSESTT00000056782<br>ENSESTT00000056783<br>ENST00000372286<br>ENST00000265351<br>ENST00000372258<br>ENST00000372252<br>ENST00000372250<br>GENSCAN00000063258<br>GENSCAN00000063265<br>GENSCAN00000048655	Homo sapiens exportin 5 (XPO5), mRNA.<br>Homo sapiens cDNA FLJ45606 fis, clone BRTHA3022641.<br><br><br><br><br><br><br>cdna:known chromosome:NCBI36:6:43598050:43651654:-1 gene:ENSG00000124571<br>cdna:known chromosome:NCBI36:6:43598053:43651729:-1 gene:ENSG00000124571<br>cdna:known chromosome:NCBI36:6:43598400:43651729:-1 gene:ENSG00000124571<br>cdna:known chromosome:NCBI36:6:43599376:43651729:-1 gene:ENSG00000124571<br>cdna:known chromosome:NCBI36:6:43599514:43651729:-1 gene:ENSG00000124571<br>cdna:Genscan chromosome:NCBI36:6:43599584:43609682:-1<br>cdna:Genscan chromosome:NCBI36:6:43614537:43651579:-1<br>cdna:Genscan chromosome:NCBI36:8:98705607:98706271:-1

	398443<br>398444	2954727<br>2954728<br>2954729<br>2954730<br>2954731<br>2954732<br>2954733<br>2954734<br>2954735<br>2954736<br>2954737<br>2954738<br>2954739<br>2954740<br>2954741<br>2954742<br>2954743<br>2954744<br>2954745<br>2954746<br>2954747<br>2954748<br>2954749<br>2954750<br>2954751	core<br>extended<br>extended<br>core<br>extended<br>extended<br>core<br>extended<br>full<br>core<br>core<br>core<br>core<br>core<br>extended<br>core<br>core<br>core<br>core<br>core<br>core<br>core<br>core<br>core<br>core						
2958325	400704<br>400705<br>400706<br>400707<br>400708<br>400709<br>400710<br>400711<br>400712<br>400713<br>400714<br>400715<br>400716<br>400717<br>400718<br>400719<br>400720<br>400721<br>400722<br>400723<br>400724<br>400725<br>400726<br>400727<br>400728<br>400729<br>400730<br>400731<br>400732	2958326<br>2958327<br>2958328<br>2958329<br>2958330<br>2958331<br>2958332<br>2958333<br>2958334<br>2958335<br>2958336<br>2958337<br>2958338<br>2958339<br>2958340<br>2958341<br>2958342<br>2958343<br>2958344<br>2958345<br>2958346<br>2958347<br>2958348<br>2958349<br>2958350<br>2958351<br>2958352<br>2958353<br>2958354	core<br>core<br>core<br>core<br>core<br>full<br>core<br>extended<br>extended<br>core<br>core<br>core<br>extended<br>extended<br>extended<br>extended<br>extended<br>extended<br>full<br>core<br>extended<br>extended<br>extended<br>extended<br>core<br>full<br>core<br>core<br>extended	6	56430755	56928906	-	NM_183380<br>NM_015548<br>NM_001723<br>NM_020388<br>AF165191<br>AK023487<br>AK025142<br>AK056797<br>AK074310<br>AK095166<br>AK128632<br>BC038763<br>BC065536<br>U31851<br>ENSESTT00000016300<br>ENSESTT00000016301<br>ENSESTT00000016302<br>ENSESTT00000016303<br>ENSESTT00000016304<br>ENSESTT00000016305<br>ENSESTT00000016306<br>ENSESTT00000016307<br>ENSESTT00000016308<br>ENSESTT00000016309<br>ENSESTT00000016310<br>ENSESTT00000016311<br>ENSESTT00000016312<br>ENSESTT00000016313<br>ENSESTT00000016314	Homo sapiens dystonin (DST), transcript variant 1, mRNA.<br>Homo sapiens dystonin (DST), transcript variant 1eA, mRNA.<br>Homo sapiens dystonin (DST), transcript variant 1e, mRNA.<br>Homo sapiens dystonin (DST), transcript variant 1eB, mRNA.<br>Homo sapiens BPAG1n3 (BPAG1) mRNA, partial cds.<br>Homo sapiens cDNA FLJ13425 fis, clone PLACE1002342, highly similar to Homo sapiens mRNA for KIAA0728 protein.<br>Homo sapiens cDNA: FLJ21489 fis, clone COL05450.<br>Homo sapiens cDNA FLJ32235 fis, clone PLACE6004738.<br>Homo sapiens cDNA FLJ23730 fis, clone HEP14530.<br>Homo sapiens cDNA FLJ37847 fis, clone BRSSN2013095.<br>Homo sapiens cDNA FLJ46791 fis, clone TRACH3029462, moderately similar to Actin cross-linking family protein 7.<br>Homo sapiens cDNA clone IMAGE:5270106.<br>Homo sapiens dystonin, mRNA (cDNA clone IMAGE:6008053), complete cds.<br>Human dystonin isoform 2 mRNA, partial cds.

	400733	2958355	core					ENSESTT00000016315	
	400734	2958356	core					ENSESTT00000016316	
	400735	2958357	extended					ENSESTT00000016317	
	400736	2958358	core					ENSESTT00000016318	
	400737	2958359	extended					ENST00000370765	cdna:known-ccds chromosome:NCBI36:6:56587342:56615644:-1 gene:ENSG00000151914 CCDS4959.1
	400738	2958360	core					ENST00000370788	cdna:known chromosome:NCBI36:6:56430744:56816422:-1 gene:ENSG00000151914
	400739	2958361	core					ENST00000361203	cdna:known chromosome:NCBI36:6:56430744:56815902:-1 gene:ENSG00000151914
	400740	2958362	extended					ENST00000340834	cdna:known chromosome:NCBI36:6:56430744:56615545:-1 gene:ENSG00000151914
	400741	2958363	core					ENST00000370769	cdna:known chromosome:NCBI36:6:56430755:56816422:-1 gene:ENSG00000151914
	400742	2958364	extended					ENST00000312431	cdna:known chromosome:NCBI36:6:56430755:56816422:-1 gene:ENSG00000151914
	400743	2958365	core					ENST00000244364	cdna:known chromosome:NCBI36:6:56430996:56615647:-1 gene:ENSG00000151914
	400744	2958366	extended					ENST00000281662	cdna:known chromosome:NCBI36:6:56587354:56927363:-1 gene:ENSG00000151914
	400745	2958367	extended					ENST00000343759	cdna:known chromosome:NCBI36:6:56587354:56615647:-1 gene:ENSG00000151914
	400746	2958368	core					ENST00000370754	cdna:known chromosome:NCBI36:6:56588274:56816422:-1 gene:ENSG00000151914
	400747	2958369	extended					GENSCAN00000059429	cdna:Genscan chromosome:NCBI36:6:56528102:56584746:-1
	400748	2958370	core					GENSCAN00000059433	cdna:Genscan chromosome:NCBI36:6:56588274:56643564:-1
	400749	2958371	core					GENSCAN00000065235	cdna:Genscan chromosome:NCBI36:6:56431762:56470264:-1
	400750	2958372	core					GENSCAN00000052889	cdna:Genscan chromosome:NCBI36:6:56821126:56890544:-1
	400751	2958373	core					GENSCAN00000050222	cdna:Genscan chromosome:NCBI36:6:56512983:56514329:-1
	400752	2958374	core					GENSCAN00000050221	cdna:Genscan chromosome:NCBI36:6:56524812:56526212:-1
	400753	2958375	core					GENSCAN00000017869	cdna:Genscan chromosome:NCBI36:6:56655273:56665906:-1
	400754	2958376	extended					GENSCAN00000017870	cdna:Genscan chromosome:NCBI36:6:56674639:56709389:-1
	400755	2958377	core					GENSCAN00000015269	cdna:Genscan chromosome:NCBI36:6:56925759:56928906:-1
	400756	2958378	core						
	400757	2958379	core						
	400758	2958380	core						
	400759	2958381	extended						
	400760	2958382	core						
	400761	2958383	core						
	400762	2958384	extended						
	400763	2958385	core						
	400764	2958386	core						
	400765	2958387	core						
	400766	2958388	core						
	400767	2958389	core						
	400768	2958390	core						
	400769	2958391	full						
	400770	2958392	core						
	400771	2958393	full						
	400772	2958394	full						
	400773	2958395	core						
	400774	2958396	core						
	400775	2958397	core						
	400776	2958398	core						
	400777	2958399	core						
	400778	2958400	core						
	400779	2958401	core						
	400780	2958402	core						
	400781	2958403	core						
	400782	2958404	core						
	400783	2958405	core						
	400784	2958406	extended						
	400785	2958407	core						
	400786	2958408	extended						

	400787	2958409	full						
	400788	2958410	full						
	400789	2958411	extended						
	400790	2958412	extended						
	400791	2958413	full						
	400792	2958414	extended						
	400793	2958415	full						
	400794	2958416	full						
	400795	2958417	free						
	400796	2958418	extended						
	400797	2958419	full						
	400798	2958420	full						
	400799	2958421	full						
	400800	2958422	full						
	400801	2958423	core						
	400802	2958424	core						
	400803	2958425	core						
	400804	2958426	core						
	400805	2958427	core						
	400806	2958428	core						
	400807	2958429	extended						
	400808	2958430	core						
	400809	2958431	extended						
	400810	2958432	core						
	400811	2958433	extended						
	400812	2958434	core						
	400813	2958435	core						
	400814	2958436	core						
	400815	2958437	core						
	400816	2958438	full						
	400817	2958439	full						
	400818	2958440	core						
	400819	2958441	extended						
	400820	2958442	extended						
	400821	2958443	extended						
	400822	2958444	extended						
	400823	2958445	core						
	400824	2958446	core						
	400825	2958447	core						
	400826	2958448	core						
	400827	2958449	core						
	400828	2958450	extended						
	400829	2958451	extended						
	400830	2958452	extended						
	400831	2958453	extended						
	400832	2958454	core						
	400833	2958455	core						
	400834	2958456	core						
	400835	2958457	core						
	400836	2958458	core						
	400837	2958459	core						
	400838	2958460	core						
	400839	2958461	core						
	400840	2958462	core						

	400841	2958463	core						
	400842	2958464	core						
	400843	2958465	core						
	400844	2958466	core						
	400845	2958467	core						
	400846	2958468	core						
	400847	2958469	core						
	400848	2958470	core						
	400849	2958471	core						
	400850	2958472	full						
	400851	2958473	core						
	400852	2958474	core						
	400853	2958475	core						
	400854	2958476	core						
	400855	2958477	core						
	400856	2958478	core						
	400857	2958479	core						
	400858	2958480	core						
	400859	2958481	free						
	400860	2958482	core						
	400861	2958483	core						
	400862	2958484	core						
	400863	2958485	core						
	400864	2958486	core						
	400865	2958487	core						
	400866	2958488	core						
	400867	2958489	core						
	400868	2958490	core						
	400869	2958491	core						
	400870	2958492	extended						
	400871	2958493	core						
	400872	2958494	core						
	400873	2958495	extended						
	400874	2958496	core						
	400875	2958497	core						
	400876	2958498	core						
	400877	2958499	full						
	400878	2958500	core						
	400879	2958501	core						
	400880	2958502	core						
	400881	2958503	extended						
	400882	2958504	extended						
	400883	2958505	core						
	400884	2958506	core						
	400885	2958507	core						
		2958508	core						
		2958509	core						
		2958510	core						
		2958511	core						
		2958512	core						
		2958513	core						
		2958514	core						
		2958515	core						
		2958516	core						

		2958517	core						
		2958518	extended						
		2958519	core						
		2958520	core						
		2958521	full						
		2958522	full						
		2958523	extended						
		2958524	extended						
		2958525	extended						
		2958526	extended						
		2958527	core						
		2958528	extended						
		2958529	extended						
		2958530	extended						
		2958531	full						
		2958532	extended						
		2958533	extended						
		2958534	extended						
		2958535	full						
		2958536	extended						
		2958537	full						
		2958538	core						
		2958539	extended						
		2958540	extended						
		2958541	core						
		2958542	core						
		2958543	full						
		2958544	full						
		2958545	extended						
		2958546	extended						
		2958547	core						
		2958548	full						
		2958549	full						
		2958550	extended						
		2958551	extended						
		2958552	extended						
		2958553	full						
		2958554	extended						
		2958555	extended						
		2958556	extended						
		2958557	full						
		2958558	extended						
		2958559	full						
		2958560	extended						
		2958561	extended						
		2958562	extended						
		2958563	core						
		2958564	core						
		2958565	full						
		2958566	full						
		2958567	full						
		2958568	full						
		2958569	full						
		2958570	extended						

		2958571<br>2958572<br>2958573<br>2958574<br>2958575<br>2958576<br>2958577<br>2958578<br>2958579<br>2958580<br>2958581<br>2958582<br>2958583<br>2958584<br>2958585<br>2958586<br>2958587<br>2958588<br>2958589<br>2958590<br>2958591<br>2958592<br>2958593<br>2958594<br>2958595<br>2958596<br>2958597<br>2958598	extended<br>full<br>full<br>full<br>full<br>full<br>full<br>full<br>extended<br>full<br>full<br>extended<br>extended<br>extended<br>extended<br>extended<br>full<br>extended<br>extended<br>extended<br>full<br>extended<br>full<br>extended<br>full<br>extended<br>full<br>full						
2961177	402449<br>402450<br>402451<br>402452<br>402453<br>402454<br>402455<br>402456<br>402457<br>402458<br>402459<br>402460<br>402461<br>402462<br>402463<br>402464<br>402465<br>402466<br>402467<br>402468<br>402469<br>402470<br>402471<br>402472<br>402473<br>402474	2961178<br>2961179<br>2961180<br>2961181<br>2961182<br>2961183<br>2961184<br>2961185<br>2961186<br>2961187<br>2961188<br>2961189<br>2961190<br>2961191<br>2961192<br>2961193<br>2961194<br>2961195<br>2961196<br>2961197<br>2961198<br>2961199<br>2961200<br>2961201<br>2961202<br>2961203	full<br>core<br>core<br>core<br>core<br>core<br>core<br>core<br>core<br>extended<br>core<br>core<br>core<br>core<br>full<br>core<br>full<br>core<br>full<br>core<br>core<br>core<br>core<br>core<br>core<br>extended	6	75850643	75972357	-	NM_080645<br>NM_001865<br>NM_004370<br>NM_018247<br>AK022029<br>BC050587<br>AL832490<br>ENSESTT00000012125<br>ENSESTT00000012126<br>ENSESTT00000012127<br>ENSESTT00000016589<br>ENSESTT00000016590<br>ENSESTT00000016591<br>ENSESTT00000016592<br>ENSESTT00000016593<br>ENSESTT00000016594<br>ENSESTT00000016595<br>ENSESTT00000016596<br>ENSESTT00000016597<br>ENSESTT00000016598<br>ENSESTT00000016599<br>ENSESTT00000016600<br>ENSESTT00000016601<br>ENST00000370050<br>ENST00000370134<br>ENST00000322507	Homo sapiens collagen, type XII, alpha 1 (COL12A1), transcript variant short, mRNA.<br>Homo sapiens cytochrome c oxidase subunit VIIa polypeptide 2 (liver) (COX7A2), mRNA.<br>Homo sapiens collagen, type XII, alpha 1 (COL12A1), transcript variant long, mRNA.<br>Homo sapiens transmembrane protein 30A (TMEM30A), mRNA.<br>Homo sapiens cDNA FLJ11967 fis, clone HEMBB1001119.<br>Homo sapiens collagen, type XII, alpha 1, mRNA (cDNA clone IMAGE:6189295), complete cds.<br>Homo sapiens mRNA; cDNA DKFZp686K1722 (from clone DKFZp686K1722).<br><br><br><br><br><br><br><br><br><br><br><br><br><br><br><br><br>cdna:known-ccds chromosome:NCBI36:6:76019360:76051221:-1 gene:ENSG00000112697 CCDS4983.1<br>cdna:known chromosome:NCBI36:6:75850762:75972290:-1 gene:ENSG00000111799<br>cdna:known chromosome:NCBI36:6:75850763:75972290:-1 gene:ENSG00000111799

	402475	2961204	core					ENST00000345356	cdna:known chromosome:NCBI36:6:75850763:75972290:-1 gene:ENSG00000111799	
	402476	2961205	extended					ENST00000370099	cdna:known chromosome:NCBI36:6:75850763:75972290:-1 gene:ENSG00000111799	
	402477	2961206	extended					ENST00000265379	cdna:known chromosome:NCBI36:6:75853714:75972258:-1 gene:ENSG00000111799	
	402478	2961207	core					ENST00000370089	cdna:known chromosome:NCBI36:6:76004111:76010442:-1 gene:ENSG00000112695	
	402479	2961208	core					ENST00000230459	cdna:known chromosome:NCBI36:6:76004223:76010245:-1 gene:ENSG00000112695	
	402480	2961209	core					ENST00000370081	cdna:known chromosome:NCBI36:6:76004247:76016759:-1 gene:ENSG00000112695	
	402481	2961210	core					ENST00000316076	cdna:known chromosome:NCBI36:6:76019369:76051212:-1 gene:ENSG00000112697	
	402482	2961211	core					ENST00000230461	cdna:known chromosome:NCBI36:6:76019369:76051212:-1 gene:ENSG00000112697	
	402483	2961212	full					GENSCAN00000047938	cdna:Genscan chromosome:NCBI36:6:75949486:76072331:-1	
	402484	2961213	core					GENSCAN00000020472	cdna:Genscan chromosome:NCBI36:6:75851626:75932215:-1	
	402485	2961214	core					GENSCAN00000060548	cdna:Genscan chromosome:NCBI36:6:75941474:75947647:-1	
	402486	2961215	full							
	402487	2961216	full							
	402488	2961217	core							
	402489	2961218	core							
	402490	2961219	extended							
	402491	2961220	core							
	402492	2961221	extended							
	402493	2961222	core							
	402494	2961223	extended							
	402495	2961224	core							
	402496	2961225	core							
	402497	2961226	full							
	402498	2961227	core							
	402499	2961228	core							
	402500	2961229	core							
	402501	2961230	core							
	402502	2961231	core							
	402503	2961232	core							
	402504	2961233	core							
	402505	2961234	core							
	402506	2961235	full							
	402507	2961236	core							
	402508	2961237	core							
	402509	2961238	extended							
	402510	2961239	core							
	402511	2961240	core							
	402512	2961241	extended							
	402513	2961242	core							
	402514	2961243	extended							
	402515	2961244	core							
	402516	2961245	core							
	402517	2961246	extended							
	402518	2961247	core							
	402519	2961248	core							
	402520	2961249	extended							
	402521	2961250	extended							
	402522	2961251	core							
	402523	2961252	core							
	402524	2961253	core							
	402525	2961254	core							
	402526	2961255	full							
	402527	2961256	core							
	402528	2961257	core							

	402529<br>402530<br>402531<br>402532<br>402533	2961258<br>2961259<br>2961260<br>2961261<br>2961262<br>2961263<br>2961264<br>2961265<br>2961266<br>2961267<br>2961268<br>2961269<br>2961270<br>2961271<br>2961272<br>2961273<br>2961274<br>2961275<br>2961276<br>2961277<br>2961278<br>2961279<br>2961280<br>2961281<br>2961282<br>2961283<br>2961284<br>2961285<br>2961286<br>2961287	core<br>core<br>core<br>core<br>full<br>core<br>core<br>core<br>core<br>core<br>core<br>full<br>core<br>core<br>core<br>core<br>core<br>extended<br>extended<br>extended<br>extended<br>full<br>core<br>core<br>full<br>full<br>full<br>full<br>full<br>full						
2965206	405036<br>405037<br>405038<br>405039<br>405040<br>405041<br>405042<br>405043<br>405044<br>405045<br>405046<br>405047<br>405048<br>405049<br>405050<br>405051<br>405052<br>405053<br>405054<br>405055<br>405056<br>405057<br>405058<br>405059	2965207<br>2965208<br>2965209<br>2965210<br>2965211<br>2965212<br>2965213<br>2965214<br>2965215<br>2965216<br>2965217<br>2965218<br>2965219<br>2965220<br>2965221<br>2965222<br>2965223<br>2965224<br>2965225<br>2965226<br>2965227<br>2965228<br>2965229<br>2965230	full<br>extended<br>core<br>core<br>core<br>full<br>core<br>core<br>full<br>core<br>full<br>core<br>core<br>extended<br>core<br>core<br>core<br>core<br>full<br>extended<br>full<br>extended<br>extended<br>full	6	93989210	94186101	-	NM_004440<br>BC027940<br>ENSESTT00000049159<br>ENSESTT00000049160<br>ENSESTT00000049161<br>ENST00000369303<br>ENST00000257785<br>ENST00000369297<br>GENSCAN00000024790<br>GENSCAN00000024794<br>GENSCAN00000039600<br>GENSCAN00000050722	Homo sapiens EPH receptor A7 (EPHA7), mRNA.<br>Homo sapiens EPH receptor A7, mRNA (cDNA clone IMAGE:5204580), complete cds.<br><br><br><br>cdna:known-ccds chromosome:NCBI36:6:94006459:94185965:-1 gene:ENSG00000135333 CCDS5031.1<br>cdna:known chromosome:NCBI36:6:94007864:94185993:-1 gene:ENSG00000135333<br>cdna:known chromosome:NCBI36:6:94176093:94185986:-1 gene:ENSG00000135333<br>cdna:Genscan chromosome:NCBI36:6:94144590:94183377:-1<br>cdna:Genscan chromosome:NCBI36:6:94184802:94185754:-1<br>cdna:Genscan chromosome:NCBI36:6:94009865:94038861:-1<br>cdna:Genscan chromosome:NCBI36:6:94122090:94136367:-1

	405060<br>405061<br>405062<br>405063<br>405064<br>405065<br>405066<br>405067<br>405068<br>405069<br>405070<br>405071<br>405072<br>405073<br>405074<br>405075<br>405076<br>405077<br>405078<br>405079<br>405080	2965231<br>2965232<br>2965233<br>2965234<br>2965235<br>2965236<br>2965237<br>2965238<br>2965239<br>2965240<br>2965241<br>2965242<br>2965243<br>2965244<br>2965245<br>2965246<br>2965247<br>2965248<br>2965249<br>2965250<br>2965251<br>2965252<br>2965253<br>2965254<br>2965255<br>2965256<br>2965257<br>2965258<br>2965259<br>2965260	extended<br>extended<br>full<br>full<br>core<br>core<br>core<br>full<br>full<br>full<br>full<br>extended<br>full<br>core<br>core<br>core<br>core<br>full<br>full<br>full<br>full<br>full<br>full<br>full<br>full<br>full<br>full<br>core<br>core<br>extended						
2970942	408619<br>408620<br>408621<br>408622<br>408623<br>408624<br>408625<br>408626<br>408627<br>408628<br>408629<br>408630<br>408631<br>408632<br>408633<br>408634<br>408635<br>408636<br>408637<br>408638	2970943<br>2970944<br>2970945<br>2970946<br>2970947<br>2970948<br>2970949<br>2970950<br>2970951<br>2970952<br>2970953<br>2970954<br>2970955<br>2970956<br>2970957<br>2970958<br>2970959<br>2970960<br>2970961<br>2970962<br>2970963<br>2970964<br>2970965<br>2970966	core<br>core<br>core<br>core<br>core<br>core<br>core<br>core<br>extended<br>core<br>full<br>extended<br>extended<br>extended<br>extended<br>extended<br>extended<br>full<br>full<br>full<br>extended<br>full<br>full<br>full	6	116546785	116625242	-	NM_000493<br>ENSESTT00000052463<br>ENSESTT00000052464<br>ENST00000243222<br>ENST00000327673<br>GENSCAN00000008319<br>GENSCAN00000005484<br>GENSCAN00000050521	Homo sapiens collagen, type X, alpha 1(Schmid metaphyseal chondrodysplasia) (COL10A1), mRNA.<br><br><br>cdna:known-ccds chromosome:NCBI36:6:116546791:116553989:-1 gene:ENSG00000123500 CCDS5105.1<br>cdna:known chromosome:NCBI36:6:116546882:116553756:-1 gene:ENSG00000123500<br>cdna:Genscan chromosome:NCBI36:6:116604443:116625242:-1<br>cdna:Genscan chromosome:NCBI36:6:116596882:116597089:-1<br>cdna:Genscan chromosome:NCBI36:6:116547929:116573933:-1

		2970967<br>2970968<br>2970969<br>2970970<br>2970971	full<br>full<br>full<br>full<br>full						
2976041	411859<br>411860<br>411861<br>411862<br>411863<br>411864<br>411865<br>411866<br>411867<br>411868<br>411869<br>411870<br>411871<br>411872<br>411873<br>411874<br>411875<br>411876<br>411877	2976042<br>2976043<br>2976044<br>2976045<br>2976046<br>2976047<br>2976048<br>2976049<br>2976050<br>2976051<br>2976052<br>2976053<br>2976054<br>2976055<br>2976056<br>2976057<br>2976058<br>2976059<br>2976060<br>2976061<br>2976062<br>2976063<br>2976064<br>2976065<br>2976066<br>2976067<br>2976068<br>2976069<br>2976070<br>2976071<br>2976072<br>2976073<br>2976074	core<br>core<br>core<br>core<br>core<br>extended<br>full<br>core<br>full<br>core<br>extended<br>extended<br>core<br>core<br>core<br>core<br>extended<br>extended<br>full<br>core<br>core<br>extended<br>full<br>full<br>full<br>extended<br>full<br>extended<br>full<br>extended<br>full<br>core<br>extended	6	137362818	137408020	-	NM_014432<br>AK098312<br>ENSESTT00000026617<br>ENST00000316649<br>ENST00000367748<br>ENST00000367747<br>ENST00000367746<br>GENSCAN00000040915	Homo sapiens interleukin 20 receptor, alpha (IL20RA), mRNA.<br>Homo sapiens cDNA FLJ40993 fis, clone UTERU2015405, highly similar to Homo sapiens class II cytokine receptor ZCYTOR7 (ZCYTOR7) mRNA.<br><br>cdna:known-ccds chromosome:NCBI36:6:137362801:137407991:-1 gene:ENSG00000016402 CCDS5181.1<br>cdna:known chromosome:NCBI36:6:137362801:137407991:-1 gene:ENSG00000016402<br>cdna:known chromosome:NCBI36:6:137362801:137407794:-1 gene:ENSG00000016402<br>cdna:known chromosome:NCBI36:6:137371718:137407794:-1 gene:ENSG00000016402<br>cdna:Genscan chromosome:NCBI36:6:137364388:137407558:-1
2985781	417771<br>417772<br>417773<br>417774<br>417775<br>417776<br>417777<br>417778<br>417779<br>417780<br>417781<br>417782<br>417783<br>417784<br>417785	2985782<br>2985783<br>2985784<br>2985785<br>2985786<br>2985787<br>2985788<br>2985789<br>2985790<br>2985791<br>2985792<br>2985793<br>2985794<br>2985795<br>2985796	full<br>full<br>full<br>full<br>full<br>full<br>full<br>full<br>full<br>full<br>full<br>full<br>full<br>full<br>full	6	169204353	169438982	-	NM_003247<br>ENSESTT00000047989<br>ENSESTT00000047990<br>ENST00000366787<br>ENST00000335061<br>GENSCAN00000059173<br>GENSCAN00000014517<br>GENSCAN00000014514<br>GENSCAN00000058308<br>GENSCAN00000058306	Homo sapiens thrombospondin 2 (THBS2), mRNA.<br><br><br>cdna:known chromosome:NCBI36:6:169357800:169396064:-1 gene:ENSG00000186340<br>cdna:known chromosome:NCBI36:6:169357801:169396062:-1 gene:ENSG00000186340<br>cdna:Genscan chromosome:NCBI36:6:169204353:169204885:-1<br>cdna:Genscan chromosome:NCBI36:6:169283411:169284750:-1<br>cdna:Genscan chromosome:NCBI36:6:169327396:169328805:-1<br>cdna:Genscan chromosome:NCBI36:6:169359842:169392804:-1<br>cdna:Genscan chromosome:NCBI36:6:169394400:169403332:-1

	417786	2985797	full						
	417787	2985798	full						
	417788	2985799	full						
	417789	2985800	full						
	417790	2985801	extended						
	417791	2985802	full						
	417792	2985803	full						
	417793	2985804	full						
	417794	2985805	full						
	417795	2985806	extended						
	417796	2985807	extended						
	417797	2985808	extended						
	417798	2985809	full						
	417799	2985810	core						
	417800	2985811	core						
	417801	2985812	core						
	417802	2985813	core						
	417803	2985814	core						
	417804	2985815	core						
	417805	2985816	core						
	417806	2985817	core						
	417807	2985818	core						
	417808	2985819	core						
	417809	2985820	extended						
	417810	2985821	core						
	417811	2985822	full						
	417812	2985823	full						
	417813	2985824	core						
	417814	2985825	core						
	417815	2985826	full						
	417816	2985827	core						
	417817	2985828	core						
	417818	2985829	extended						
	417819	2985830	core						
	417820	2985831	full						
	417821	2985832	full						
	417822	2985833	core						
	417823	2985834	core						
	417824	2985835	full						
	417825	2985836	extended						
	417826	2985837	core						
	417827	2985838	core						
	417828	2985839	core						
	417829	2985840	extended						
	417830	2985841	core						
	417831	2985842	extended						
	417832	2985843	full						
	417833	2985844	core						
	417834	2985845	full						
	417835	2985846	core						
	417836	2985847	extended						
	417837	2985848	extended						
	417838	2985849	core						
	417839	2985850	full						

	417840	2985851<br>2985852<br>2985853<br>2985854<br>2985855<br>2985856<br>2985857<br>2985858<br>2985859<br>2985860<br>2985861<br>2985862<br>2985863<br>2985864<br>2985865<br>2985866<br>2985867<br>2985868<br>2985869<br>2985870<br>2985871<br>2985872<br>2985873	full<br>extended<br>extended<br>extended<br>core<br>core<br>core<br>extended<br>full<br>extended<br>full<br>extended<br>core<br>core<br>full<br>full<br>full<br>full<br>full<br>full<br>extended<br>extended<br>extended						
2987632	418933<br>418934<br>418935<br>418936<br>418937<br>418938<br>418939<br>418940<br>418941<br>418942<br>418943<br>418944<br>418945<br>418946<br>418947<br>418948<br>418949<br>418950<br>418951<br>418952<br>418953<br>418954<br>418955<br>418956<br>418957<br>418958<br>418959<br>418960<br>418961<br>418962<br>418963	2987633<br>2987634<br>2987635<br>2987636<br>2987637<br>2987638<br>2987639<br>2987640<br>2987641<br>2987642<br>2987643<br>2987644<br>2987645<br>2987646<br>2987647<br>2987648<br>2987649<br>2987650<br>2987651<br>2987652<br>2987653<br>2987654<br>2987655<br>2987656<br>2987657<br>2987658<br>2987659<br>2987660<br>2987661<br>2987662<br>2987663	full<br>full<br>core<br>core<br>core<br>full<br>full<br>full<br>full<br>full<br>full<br>extended<br>extended<br>full<br>extended<br>core<br>core<br>core<br>core<br>extended<br>core<br>extended<br>core<br>core<br>full<br>extended<br>core<br>full<br>extended<br>core<br>core	7	2632695	2670954	+	NM_025250<br>AK124608<br>ENSESTT00000034700<br>ENSESTT00000034701<br>ENSESTT00000034702<br>ENST00000258796<br>ENST00000382459<br>GENSCAN00000057072<br>GENSCAN00000043529	Homo sapiens tweety homolog 3 (Drosophila) (TTYH3), mRNA.<br>Homo sapiens cDNA FLJ42617 fis, clone BRACE3014807.<br><br><br><br>cdna:known chromosome:NCBI36:7:2638129:2670953:1 gene:ENSG00000136295<br>cdna:known chromosome:NCBI36:7:2638129:2670953:1 gene:ENSG00000136295<br>cdna:Genscan chromosome:NCBI36:7:2657673:2668344:1<br>cdna:Genscan chromosome:NCBI36:7:2629082:2656148:1

	418964<br>418965<br>418966<br>418967	2987664<br>2987665<br>2987666<br>2987667<br>2987668<br>2987669<br>2987670<br>2987671<br>2987672<br>2987673<br>2987674<br>2987675<br>2987676<br>2987677<br>2987678	core<br>full<br>extended<br>core<br>core<br>extended<br>core<br>core<br>core<br>core<br>core<br>core<br>core<br>core<br>core						
2997376	425188<br>425189<br>425190<br>425191<br>425192<br>425193<br>425194<br>425195<br>425196<br>425197<br>425198<br>425199<br>425200<br>425201<br>425202<br>425203<br>425204<br>425205<br>425206<br>425207<br>425208<br>425209<br>425210<br>425211<br>425212<br>425213<br>425214<br>425215<br>425216<br>425217<br>425218<br>425219<br>425220<br>425221<br>425222<br>425223	2997377<br>2997378<br>2997379<br>2997380<br>2997381<br>2997382<br>2997383<br>2997384<br>2997385<br>2997386<br>2997387<br>2997388<br>2997389<br>2997390<br>2997391<br>2997392<br>2997393<br>2997394<br>2997395<br>2997396<br>2997397<br>2997398<br>2997399<br>2997400<br>2997401<br>2997402<br>2997403<br>2997404<br>2997405<br>2997406<br>2997407<br>2997408<br>2997409<br>2997410<br>2997411<br>2997412<br>2997413<br>2997414<br>2997415	core<br>core<br>extended<br>extended<br>core<br>core<br>extended<br>core<br>core<br>extended<br>core<br>core<br>core<br>full<br>core<br>extended<br>core<br>core<br>core<br>core<br>core<br>core<br>core<br>core<br>full<br>core<br>core<br>extended<br>core<br>core<br>core<br>core<br>full<br>full<br>core<br>extended<br>extended<br>core<br>extended	7	36395957	36462017	+	NM_018685<br>AK094574<br>BC034692<br>ENSESTT00000016725<br>ENSESTT00000016726<br>ENSESTT00000016727<br>ENSESTT00000016728<br>ENSESTT00000016729<br>ENSESTT00000016730<br>ENST00000265748<br>GENSCAN00000003929	Homo sapiens anillin, actin binding protein (scraps homolog, Drosophila) (ANLN), mRNA.<br>Homo sapiens cDNA FLJ37255 fis, clone BRAMY2009023.<br>Homo sapiens anillin, actin binding protein (scraps homolog, Drosophila), mRNA (cDNA clone IMAGE:4753061), complete cds.<br><br><br><br><br><br><br>cdna:known-ccds chromosome:NCBI36:7:36395957:36459923:1 gene:ENSG00000011426 CCDS5447.1<br>cdna:Genscan chromosome:NCBI36:7:36396161:36458734:1

		2997416<br>2997417<br>2997418<br>2997419<br>2997420<br>2997421<br>2997422<br>2997423<br>2997424<br>2997425<br>2997426<br>2997427	extended<br>extended<br>core<br>core<br>extended<br>extended<br>core<br>core<br>core<br>extended<br>extended<br>extended						
2999755	426685<br>426686<br>426687<br>426688<br>426689<br>426690<br>426691<br>426692<br>426693<br>426694<br>426695<br>426696<br>426697<br>426698<br>426699<br>426700<br>426701<br>426702<br>426703<br>426704<br>426705<br>426706<br>426707<br>426708<br>426709<br>426710<br>426711<br>426712	2999756<br>2999757<br>2999758<br>2999759<br>2999760<br>2999761<br>2999762<br>2999763<br>2999764<br>2999765<br>2999766<br>2999767<br>2999768<br>2999769<br>2999770<br>2999771<br>2999772<br>2999773<br>2999774<br>2999775<br>2999776<br>2999777<br>2999778<br>2999779<br>2999780<br>2999781<br>2999782<br>2999783<br>2999784<br>2999785<br>2999786<br>2999787<br>2999788<br>2999789<br>2999790<br>2999791<br>2999792<br>2999793<br>2999794<br>2999795<br>2999796<br>2999797	full<br>full<br>full<br>full<br>full<br>full<br>full<br>full<br>full<br>extended<br>core<br>core<br>core<br>core<br>core<br>core<br>core<br>core<br>core<br>core<br>core<br>core<br>extended<br>core<br>core<br>core<br>extended<br>core<br>extended<br>extended<br>core<br>core<br>extended<br>extended<br>core<br>core<br>core<br>core<br>core<br>core<br>core<br>core	7	44100729	44120683	+	NM_001129<br>AK090931<br>AK127541<br>ENSESTT00000036536<br>ENSESTT00000036537<br>ENSESTT00000036538<br>ENST00000223357<br>GENSCAN00000066406<br>GENSCAN00000066404	Homo sapiens AE binding protein 1 (AEBP1), mRNA.<br>Homo sapiens cDNA FLJ33612 fis, clone BRAMY2016953, highly similar to Adipocyte transcription factor, AEBP1.<br>Homo sapiens cDNA FLJ45634 fis, clone CHONS2002829, moderately similar to Homo sapiens adipocyte enhancer binding protein 1 (AEBP1).<br><br><br><br>cdna:known-ccds chromosome:NCBI36:7:44110485:44120683:1 gene:ENSG00000106624 CCDS5476.1<br>cdna:Genscan chromosome:NCBI36:7:44110790:44120385:1<br>cdna:Genscan chromosome:NCBI36:7:44107538:44109632:1

		2999798<br>2999799<br>2999800<br>2999801<br>2999802<br>2999803	core<br>core<br>core<br>core<br>core<br>core						
3011492	433832<br>433833<br>433834<br>433835<br>433836<br>433837<br>433838<br>433839<br>433840<br>433841<br>433842<br>433843<br>433844<br>433845<br>433846<br>433847<br>433848<br>433849<br>433850<br>433851<br>433852<br>433853<br>433854<br>433855<br>433856<br>433857<br>433858<br>433859<br>433860<br>433861<br>433862<br>433863<br>433864<br>433865<br>433866<br>433867<br>433868<br>433869<br>433870<br>433871<br>433872<br>433873<br>433874<br>433875<br>433876<br>433877<br>433878	3011493<br>3011494<br>3011495<br>3011496<br>3011497<br>3011498<br>3011499<br>3011500<br>3011501<br>3011502<br>3011503<br>3011504<br>3011505<br>3011506<br>3011507<br>3011508<br>3011509<br>3011510<br>3011511<br>3011512<br>3011513<br>3011514<br>3011515<br>3011516<br>3011517<br>3011518<br>3011519<br>3011520<br>3011521<br>3011522<br>3011523<br>3011524<br>3011525<br>3011526<br>3011527<br>3011528<br>3011529<br>3011530<br>3011531<br>3011532<br>3011533<br>3011534<br>3011535<br>3011536<br>3011537<br>3011538<br>3011539<br>3011540	core<br>core<br>extended<br>extended<br>core<br>full<br>full<br>full<br>full<br>core<br>core<br>core<br>extended<br>full<br>core<br>core<br>extended<br>extended<br>core<br>full<br>core<br>core<br>core<br>core<br>core<br>core<br>core<br>full<br>core<br>core<br>core<br>core<br>core<br>core<br>core<br>full<br>core<br>full<br>core<br>core<br>core<br>core<br>full<br>full<br>core<br>core<br>core<br>full	7	87401512	87670138	+	NM_004194<br>NM_021721<br>NM_021722<br>NM_021723<br>NM_016351<br>AF155381<br>AK125432<br>BC036029<br>BC062433<br>AL133090<br>ENSESTT00000013418<br>ENSESTT00000013419<br>ENSESTT00000013420<br>ENSESTT00000013421<br>ENST00000265727<br>ENST00000315984<br>GENSCAN00000064252<br>GENSCAN00000068670	Homo sapiens ADAM metallopeptidase domain 22 (ADAM22), transcript variant 4, mRNA.<br>Homo sapiens ADAM metallopeptidase domain 22 (ADAM22), transcript variant 5, mRNA.<br>Homo sapiens ADAM metallopeptidase domain 22 (ADAM22), transcript variant 2, mRNA.<br>Homo sapiens ADAM metallopeptidase domain 22 (ADAM22), transcript variant 1, mRNA.<br>Homo sapiens ADAM metallopeptidase domain 22 (ADAM22), transcript variant 3, mRNA.<br>Homo sapiens metalloprotease-like, disintegrin-like, cysteine-rich protein 2 delta (ADAM22) mRNA, alternative splice product, complete cds.<br>Homo sapiens cDNA FLJ43443 fis, clone OCBBF2031167, highly similar to Homo sapiens mRNA for MDC2 alpha, MDC2 beta.<br>Homo sapiens ADAM metallopeptidase domain 22, mRNA (cDNA clone IMAGE:5278621), complete cds.<br>Homo sapiens ADAM metallopeptidase domain 22, mRNA (cDNA clone IMAGE:6458074), partial cds.<br>Homo sapiens mRNA; cDNA DKFZp434E0528 (from clone DKFZp434E0528).<br><br><br><br><br>cdna:known chromosome:NCBI36:7:87401638:87664383:1 gene:ENSG00000008277<br>cdna:known chromosome:NCBI36:7:87401638:87664383:1 gene:ENSG00000008277<br>cdna:Genscan chromosome:NCBI36:7:87575427:87664803:1<br>cdna:Genscan chromosome:NCBI36:7:87401717:87453857:1

		3011541<br>3011542<br>3011543<br>3011544<br>3011545<br>3011546<br>3011547<br>3011548<br>3011549<br>3011550<br>3011551<br>3011552	core<br>extended<br>extended<br>extended<br>core<br>extended<br>core<br>core<br>core<br>extended<br>extended<br>extended						
3013054	434885<br>434886<br>434887<br>434888<br>434889<br>434890<br>434891<br>434892<br>434893<br>434894<br>434895<br>434896<br>434897<br>434898<br>434899<br>434900<br>434901<br>434902<br>434903<br>434904<br>434905<br>434906<br>434907<br>434908<br>434909<br>434910<br>434911<br>434912<br>434913<br>434914<br>434915<br>434916<br>434917<br>434918<br>434919<br>434920<br>434921<br>434922<br>434923<br>434924<br>434925<br>434926	3013055<br>3013056<br>3013057<br>3013058<br>3013059<br>3013060<br>3013061<br>3013062<br>3013063<br>3013064<br>3013065<br>3013066<br>3013067<br>3013068<br>3013069<br>3013070<br>3013071<br>3013072<br>3013073<br>3013074<br>3013075<br>3013076<br>3013077<br>3013078<br>3013079<br>3013080<br>3013081<br>3013082<br>3013083<br>3013084<br>3013085<br>3013086<br>3013087<br>3013088<br>3013089<br>3013090<br>3013091<br>3013092<br>3013093<br>3013094<br>3013095<br>3013096	full<br>full<br>full<br>full<br>full<br>extended<br>full<br>full<br>full<br>full<br>full<br>full<br>full<br>full<br>full<br>full<br>full<br>full<br>full<br>full<br>full<br>full<br>core<br>core<br>core<br>extended<br>full<br>extended<br>core<br>core<br>extended<br>core<br>extended<br>extended<br>extended<br>extended<br>core<br>core<br>core<br>core<br>core<br>core	7	93740705	93944039	+	NM_000089<br>X02488<br>V00503<br>AL833478<br>K02046<br>S62614<br>ENSESTT00000027710<br>ENSESTT00000027711<br>ENSESTT00000027712<br>ENSESTT00000027713<br>ENSESTT00000027714<br>ENSESTT00000027715<br>ENSESTT00000027716<br>ENSESTT00000027717<br>ENSESTT00000037982<br>ENSESTT00000037983<br>ENST00000297268<br>GENSCAN00000014258<br>GENSCAN00000014259<br>GENSCAN00000022533<br>GENSCAN00000006923	Homo sapiens collagen, type I, alpha 2 (COL1A2), mRNA.<br>Human pro-alpha-2(I) mRNA for collagen N-prepropeptide.<br>Human mRNA encoding Pro-alpha-2 chain of type I procollagen. (major part).<br>Homo sapiens mRNA; cDNA DKFZp686B1029 (from clone DKFZp686B1029).<br>Human procollagen type I alpha-2 chain, partial exon 1 mutation C-propeptide region.<br>COL1A2=alpha 2 type I procollagen [human, mRNA Partial Mutant, 48 nt].<br><br><br><br><br><br><br><br><br><br><br>cdna:known chromosome:NCBI36:7:93861809:93898480:1 gene:ENSG00000164692<br>cdna:Genscan chromosome:NCBI36:7:93779660:93865639:1<br>cdna:Genscan chromosome:NCBI36:7:93867469:93872500:1<br>cdna:Genscan chromosome:NCBI36:7:93873462:93902745:1<br>cdna:Genscan chromosome:NCBI36:7:93740789:93750686:1

	434927	3013097	extended						
	434928	3013098	core						
	434929	3013099	extended						
	434930	3013100	extended						
	434931	3013101	core						
	434932	3013102	core						
	434933	3013103	core						
	434934	3013104	core						
	434935	3013105	core						
	434936	3013106	core						
	434937	3013107	core						
	434938	3013108	core						
	434939	3013109	core						
	434940	3013110	core						
	434941	3013111	core						
	434942	3013112	extended						
	434943	3013113	core						
	434944	3013114	core						
	434945	3013115	core						
	434946	3013116	core						
	434947	3013117	core						
	434948	3013118	core						
	434949	3013119	core						
	434950	3013120	core						
	434951	3013121	extended						
	434952	3013122	extended						
	434953	3013123	extended						
	434954	3013124	core						
	434955	3013125	core						
	434956	3013126	extended						
	434957	3013127	core						
	434958	3013128	core						
	434959	3013129	core						
	434960	3013130	core						
	434961	3013131	extended						
	434962	3013132	core						
	434963	3013133	extended						
	434964	3013134	extended						
		3013135	core						
		3013136	extended						
		3013137	core						
		3013138	extended						
		3013139	core						
		3013140	extended						
		3013141	core						
		3013142	core						
		3013143	core						
		3013144	core						
		3013145	extended						
		3013146	core						
		3013147	extended						
		3013148	core						
		3013149	full						
		3013150	extended						

		3013151<br>3013152<br>3013153<br>3013154<br>3013155<br>3013156<br>3013157<br>3013158<br>3013159<br>3013160<br>3013161<br>3013162<br>3013163<br>3013164<br>3013165<br>3013166<br>3013167	core<br>extended<br>core<br>full<br>core<br>core<br>core<br>core<br>extended<br>core<br>core<br>core<br>core<br>full<br>full<br>extended<br>full						
3015911	436594<br>436595<br>436596<br>436597<br>436598<br>436599<br>436600<br>436601<br>436602<br>436603<br>436604	3015912<br>3015913<br>3015914<br>3015915<br>3015916<br>3015917<br>3015918<br>3015919<br>3015920<br>3015921<br>3015922<br>3015923<br>3015924<br>3015925<br>3015926<br>3015927<br>3015928<br>3015929<br>3015930<br>3015931<br>3015932<br>3015933<br>3015934<br>3015935<br>3015936<br>3015937<br>3015938	extended<br>core<br>extended<br>core<br>core<br>extended<br>core<br>extended<br>core<br>core<br>core<br>core<br>full<br>full<br>core<br>core<br>extended<br>core<br>extended<br>extended<br>core<br>core<br>core<br>extended<br>extended<br>core<br>extended	7	100302721	100309074	+	NM_003302<br>AK056773<br>CR749505<br>ENSESTT00000028475<br>ENSESTT00000028476<br>ENSESTT00000028477<br>ENST00000200457<br>GENSCAN00000020812	Homo sapiens thyroid hormone receptor interactor 6 (TRIP6), mRNA.<br>Homo sapiens cDNA FLJ32211 fis, clone PLACE6003393, highly similar to THYROID RECEPTOR INTERACTING PROTEIN 6.<br>Homo sapiens mRNA; cDNA DKFZp686J22257 (from clone DKFZp686J22257).<br><br><br><br>cdna:known-ccds chromosome:NCBI36:7:100302910:100309004:1 gene:ENSG00000087077 CCDS5708.1<br>cdna:Genscan chromosome:NCBI36:7:100289756:100324106:1
3020343	439340<br>439341<br>439342<br>439343<br>439344<br>439345<br>439346<br>439347<br>439348	3020344<br>3020345<br>3020346<br>3020347<br>3020348<br>3020349<br>3020350<br>3020351<br>3020352	core<br>full<br>extended<br>full<br>core<br>core<br>core<br>extended<br>full	7	116099694	116230307	+	NM_000245<br>U08818<br>U11813<br>ENSESTT00000028318<br>ENSESTT00000028319<br>ENSESTT00000028320<br>ENSESTT00000028321<br>ENST00000318493<br>GENSCAN00000040592	Homo sapiens met proto-oncogene (hepatocyte growth factor receptor) (MET), mRNA.<br>Human activated met oncogene mRNA, partial cds.<br>Homo sapiens hepatocyte growth factor receptor precursor, mRNA, partial cds; alternatively spliced.<br><br><br><br><br>cdna:known chromosome:NCBI36:7:116099695:116223632:1 gene:ENSG00000105976<br>cdna:Genscan chromosome:NCBI36:7:116222945:116230307:1

	439349	3020353	full					GENSCAN00000065557	cdna:Genscan chromosome:NCBI36:7:116185784:116210759:1
	439350	3020354	extended						
	439351	3020355	extended						
	439352	3020356	full						
	439353	3020357	full						
	439354	3020358	full						
	439355	3020359	full						
	439356	3020360	extended						
	439357	3020361	extended						
	439358	3020362	extended						
	439359	3020363	core						
	439360	3020364	full						
	439361	3020365	core						
	439362	3020366	core						
	439363	3020367	core						
	439364	3020368	full						
	439365	3020369	full						
	439366	3020370	full						
	439367	3020371	extended						
	439368	3020372	extended						
	439369	3020373	extended						
	439370	3020374	core						
	439371	3020375	extended						
	439372	3020376	extended						
	439373	3020377	core						
	439374	3020378	core						
	439375	3020379	core						
	439376	3020380	core						
	439377	3020381	core						
	439378	3020382	core						
	439379	3020383	extended						
	439380	3020384	core						
	439381	3020385	core						
		3020386	extended						
		3020387	core						
		3020388	extended						
		3020389	extended						
		3020390	core						
		3020391	core						
		3020392	core						
		3020393	core						
		3020394	extended						
		3020395	core						
		3020396	core						
		3020397	core						
		3020398	extended						
		3020399	core						
		3020400	extended						
		3020401	core						
		3020402	core						
		3020403	core						
		3020404	core						
		3020405	core						
		3020406	core						

		3020407<br>3020408<br>3020409<br>3020410<br>3020411<br>3020412<br>3020413	core<br>core<br>core<br>core<br>core<br>core<br>full						
3023149	441149<br>441150<br>441151<br>441152<br>441153<br>441154<br>441155<br>441156<br>441157<br>441158<br>441159<br>441160<br>441161<br>441162<br>441163<br>441164<br>441165<br>441166<br>441167<br>441168<br>441169<br>441170<br>441171<br>441172<br>441173<br>441174<br>441175<br>441176<br>441177<br>441178<br>441179<br>441180<br>441181<br>441182<br>441183<br>441184<br>441185<br>441186<br>441187<br>441188<br>441189<br>441190<br>441191<br>441192<br>441193<br>441194<br>441195	3023150<br>3023151<br>3023152<br>3023153<br>3023154<br>3023155<br>3023156<br>3023157<br>3023158<br>3023159<br>3023160<br>3023161<br>3023162<br>3023163<br>3023164<br>3023165<br>3023166<br>3023167<br>3023168<br>3023169<br>3023170<br>3023171<br>3023172<br>3023173<br>3023174<br>3023175<br>3023176<br>3023177<br>3023178<br>3023179<br>3023180<br>3023181<br>3023182<br>3023183<br>3023184<br>3023185<br>3023186<br>3023187<br>3023188<br>3023189<br>3023190<br>3023191<br>3023192<br>3023193<br>3023194<br>3023195<br>3023196	core<br>core<br>core<br>full<br>core<br>extended<br>extended<br>core<br>core<br>core<br>core<br>core<br>core<br>core<br>core<br>core<br>core<br>extended<br>core<br>core<br>core<br>core<br>core<br>core<br>core<br>full<br>core<br>core<br>core<br>core<br>full<br>core<br>core<br>core<br>core<br>core<br>core<br>core<br>extended<br>core<br>core<br>core<br>core<br>core<br>core<br>core<br>core	7	128257706	128286553	+	NM_001458<br>AK126540<br>ENSESTT00000055784<br>ENSESTT00000055785<br>ENSESTT00000055786<br>ENSESTT00000055787<br>ENSESTT00000055788<br>ENSESTT00000055789<br>ENSESTT00000055790<br>ENSESTT00000055791<br>ENSESTT00000055792<br>ENSESTT00000055793<br>ENSESTT00000055794<br>ENSESTT00000055795<br>ENST00000325888<br>ENST00000346177<br>ENST00000388853<br>GENSCAN00000023357	Homo sapiens filamin C, gamma (actin binding protein 280) (FLNC), mRNA.<br>Homo sapiens cDNA FLJ44576 fis, clone UTERU3018616.<br><br><br><br><br><br><br><br><br><br><br><br><br>cdna:known chromosome:NCBI36:7:128257719:128286568:1 gene:ENSG00000128591<br>cdna:known chromosome:NCBI36:7:128257719:128286568:1 gene:ENSG00000128591<br>cdna:known chromosome:NCBI36:7:128257988:128285813:1 gene:ENSG00000128591<br>cdna:Genscan chromosome:NCBI36:7:128257928:128292868:1

	441196<br>441197	3023197<br>3023198<br>3023199<br>3023200<br>3023201<br>3023202<br>3023203<br>3023204<br>3023205<br>3023206<br>3023207<br>3023208<br>3023209<br>3023210	core<br>core<br>core<br>core<br>full<br>core<br>core<br>core<br>core<br>core<br>core<br>core<br>core<br>core						
3023384	441287<br>441288<br>441289<br>441290<br>441291<br>441292<br>441293<br>441294<br>441295<br>441296<br>441297<br>441298<br>441299<br>441300<br>441301<br>441302<br>441303<br>441304<br>441305<br>441306<br>441307<br>441308<br>441309<br>441310<br>441311<br>441312<br>441313<br>441314<br>441315<br>441316<br>441317<br>441318<br>441319<br>441320<br>441321<br>441322<br>441323<br>441324<br>441325<br>441326	3023385<br>3023386<br>3023387<br>3023388<br>3023389<br>3023390<br>3023391<br>3023392<br>3023393<br>3023394<br>3023395<br>3023396<br>3023397<br>3023398<br>3023399<br>3023400<br>3023401<br>3023402<br>3023403<br>3023404<br>3023405<br>3023406<br>3023407<br>3023408<br>3023409<br>3023410<br>3023411<br>3023412<br>3023413<br>3023414<br>3023415<br>3023416<br>3023417<br>3023418<br>3023419<br>3023420<br>3023421<br>3023422<br>3023423<br>3023424	extended<br>core<br>core<br>core<br>extended<br>extended<br>extended<br>extended<br>extended<br>full<br>extended<br>full<br>extended<br>extended<br>extended<br>extended<br>extended<br>extended<br>extended<br>extended<br>extended<br>extended<br>extended<br>extended<br>full<br>extended<br>extended<br>extended<br>extended<br>extended<br>extended<br>extended<br>extended<br>extended<br>extended<br>extended<br>extended<br>extended<br>extended<br>extended	7	128652043	128857283	+	NM_015328<br>AK025372<br>AK129958<br>AK172759<br>ENSESTT00000055809<br>ENSESTT00000055810<br>ENSESTT00000055811<br>ENSESTT00000055812<br>ENSESTT00000055813<br>ENSESTT00000055814<br>ENSESTT00000055815<br>ENSESTT00000055816<br>ENST00000325006<br>ENST00000378622<br>GENSCAN00000062913<br>GENSCAN00000062796	Homo sapiens KIAA0828 protein (KIAA0828), mRNA.<br>Homo sapiens cDNA: FLJ21719 fis, clone COLF0094.<br>Homo sapiens cDNA FLJ26448 fis, clone KDN02955.<br>Homo sapiens cDNA FLJ23920 fis, clone COL02027.<br><br><br><br><br><br><br><br><br>cdna:known-ccds chromosome:NCBI36:7:128652100:128857287:1 gene:ENSG00000158467 CCDS5812.1<br>cdna:novel chromosome:NCBI36:7:128804617:128857287:1 gene:ENSG00000158467<br>cdna:Genscan chromosome:NCBI36:7:128802947:128853752:1<br>cdna:Genscan chromosome:NCBI36:7:128652064:128659769:1

	441327	3023425	extended						
	441328	3023426	extended						
	441329	3023427	extended						
	441330	3023428	extended						
	441331	3023429	extended						
	441332	3023430	extended						
	441333	3023431	extended						
	441334	3023432	extended						
	441335	3023433	extended						
	441336	3023434	extended						
	441337	3023435	extended						
	441338	3023436	extended						
	441339	3023437	extended						
	441340	3023438	core						
	441341	3023439	extended						
	441342	3023440	extended						
	441343	3023441	extended						
	441344	3023442	extended						
	441345	3023443	core						
	441346	3023444	extended						
	441347	3023445	core						
	441348	3023446	extended						
	441349	3023447	extended						
	441350	3023448	core						
	441351	3023449	extended						
	441352	3023450	extended						
	441353	3023451	core						
	441354	3023452	extended						
	441355	3023453	core						
	441356	3023454	extended						
	441357	3023455	core						
	441358	3023456	extended						
	441359	3023457	core						
	441360	3023458	core						
	441361	3023459	extended						
	441362	3023460	core						
	441363	3023461	full						
		3023462	extended						
		3023463	core						
		3023464	extended						
		3023465	extended						
		3023466	extended						
		3023467	extended						
		3023468	full						
		3023469	core						
		3023470	extended						
		3023471	core						
		3023472	core						
		3023473	core						
		3023474	full						
		3023475	core						
		3023476	core						
		3023477	core						
		3023478	core						

3025545	442695	3025546	extended	7	134080806	134306010	+	NM_004342	Homo sapiens caldesmon 1 (CALD1), transcript variant 2, mRNA.
	442696	3025547	full					NM_033157	Homo sapiens caldesmon 1 (CALD1), transcript variant 3, mRNA.
	442697	3025548	full					NM_033139	Homo sapiens caldesmon 1 (CALD1), transcript variant 4, mRNA.
	442698	3025549	full					NM_033140	Homo sapiens caldesmon 1 (CALD1), transcript variant 5, mRNA.
	442699	3025550	full					NM_033138	Homo sapiens caldesmon 1 (CALD1), transcript variant 1, mRNA.
	442700	3025551	full					AF247820	Homo sapiens NAG22 protein mRNA, complete cds.
	442701	3025552	full					AK021691	Homo sapiens cDNA FLJ11629 fis, clone HEMBA1004241.
	442702	3025553	core					AK022222	Homo sapiens cDNA FLJ12160 fis, clone MAMMA1000567.
	442703	3025554	core					AK022456	Homo sapiens cDNA FLJ12394 fis, clone MAMMA1002727.
	442704	3025555	extended					BX649088	Homo sapiens mRNA; cDNA DKFZp779D0769 (from clone DKFZp779D0769).
	442705	3025556	extended					BC005006	Homo sapiens caldesmon 1, mRNA (cDNA clone IMAGE:3640699), containing frame-shift errors.
	442706	3025557	extended					ENSESTT00000042745	
	442707	3025558	extended					ENSESTT00000042746	
	442708	3025559	extended					ENSESTT00000042747	
	442709	3025560	extended					ENSESTT00000042748	
	442710	3025561	extended					ENSESTT00000042749	
	442711	3025562	extended					ENSESTT00000042750	
	442712	3025563	extended					ENSESTT00000042751	
	442713	3025564	full					ENSESTT00000042752	
	442714	3025565	extended					ENSESTT00000042753	
	442715	3025566	extended					ENSESTT00000042754	
	442716	3025567	extended					ENSESTT00000042755	
	442717	3025568	extended					ENSESTT00000042757	
	442718	3025569	extended					ENSESTT00000042758	
	442719	3025570	extended					ENSESTT00000042759	
	442720	3025571	extended					ENST00000361675	cdna:known-ccds chromosome:NCBI36:7:134114711:134306010:1 gene:ENSG00000122786 CCDS5835.1
	442721	3025572	extended					ENST00000361388	cdna:known-ccds chromosome:NCBI36:7:134114711:134306010:1 gene:ENSG00000122786 CCDS5836.1
	442722	3025573	extended					ENST00000361901	cdna:known-ccds chromosome:NCBI36:7:134114711:134306010:1 gene:ENSG00000122786 CCDS5834.1
	442723	3025574	extended					GENSCAN00000002897	cdna:Genscan chromosome:NCBI36:7:134079354:134128361:1
	442724	3025575	extended					GENSCAN00000031244	cdna:Genscan chromosome:NCBI36:7:134171705:134228689:1
	442725	3025576	extended					GENSCAN00000031243	cdna:Genscan chromosome:NCBI36:7:134255820:134323287:1
	442726	3025577	extended						
	442727	3025578	extended						
	442728	3025579	full						
	442729	3025580	full						
	442730	3025581	core						
	442731	3025582	extended						
	442732	3025583	full						
	442733	3025584	full						
	442734	3025585	extended						
	442735	3025586	full						
	442736	3025587	core						
	442737	3025588	core						
	442738	3025589	extended						
	442739	3025590	extended						
	442740	3025591	extended						
	442741	3025592	extended						
	442742	3025593	extended						
	442743	3025594	extended						
	442744	3025595	extended						
	442745	3025596	full						
	442746	3025597	extended						
	442747	3025598	extended						
	442748	3025599	full						

	442749	3025600	full						
	442750	3025601	full						
	442751	3025602	extended						
	442752	3025603	extended						
	442753	3025604	extended						
	442754	3025605	extended						
	442755	3025606	full						
	442756	3025607	full						
	442757	3025608	full						
	442758	3025609	extended						
	442759	3025610	extended						
	442760	3025611	extended						
	442761	3025612	core						
	442762	3025613	full						
	442763	3025614	extended						
	442764	3025615	extended						
	442765	3025616	extended						
	442766	3025617	extended						
	442767	3025618	full						
	442768	3025619	extended						
	442769	3025620	full						
	442770	3025621	full						
	442771	3025622	full						
	442772	3025623	extended						
	442773	3025624	extended						
	442774	3025625	extended						
	442775	3025626	core						
	442776	3025627	extended						
	442777	3025628	core						
	442778	3025629	core						
	442779	3025630	core						
	442780	3025631	core						
		3025632	core						
		3025633	core						
		3025634	core						
		3025635	core						
		3025636	extended						
		3025637	extended						
		3025638	core						
		3025639	extended						
		3025640	extended						
		3025641	extended						
		3025642	extended						
		3025643	extended						
		3025644	core						
		3025645	extended						
		3025646	extended						
		3025647	extended						
		3025648	full						
		3025649	core						
		3025650	core						
		3025651	extended						
		3025652	extended						
		3025653	core						

		3025654<br>3025655<br>3025656<br>3025657<br>3025658<br>3025659<br>3025660<br>3025661<br>3025662<br>3025663<br>3025664<br>3025665<br>3025666<br>3025667<br>3025668<br>3025669<br>3025670<br>3025671<br>3025672<br>3025673<br>3025674<br>3025675<br>3025676<br>3025677	core<br>core<br>extended<br>extended<br>core<br>core<br>core<br>core<br>core<br>core<br>extended<br>extended<br>extended<br>extended<br>core<br>extended<br>full<br>extended<br>extended<br>core<br>core<br>core<br>core<br>core						
3026599	443342<br>443343<br>443344<br>443345<br>443346<br>443347<br>443348<br>443349<br>443350<br>443351<br>443352<br>443353<br>443354<br>443355<br>443356<br>443357<br>443358<br>443359<br>443360<br>443361<br>443362<br>443363<br>443364<br>443365<br>443366<br>443367<br>443368<br>443369<br>443370<br>443371	3026600<br>3026601<br>3026602<br>3026603<br>3026604<br>3026605<br>3026606<br>3026607<br>3026608<br>3026609<br>3026610<br>3026611<br>3026612<br>3026613<br>3026614<br>3026615<br>3026616<br>3026617<br>3026618<br>3026619<br>3026620<br>3026621<br>3026622<br>3026623<br>3026624<br>3026625<br>3026626<br>3026627<br>3026628<br>3026629	full<br>full<br>full<br>extended<br>core<br>core<br>core<br>core<br>extended<br>extended<br>full<br>extended<br>extended<br>extended<br>extended<br>extended<br>extended<br>extended<br>full<br>full<br>full<br>extended<br>core<br>extended<br>extended<br>full<br>core<br>extended<br>core<br>extended	7	137793986	137922306	+	NM_003852<br>NM_015905<br>CR617986<br>ENSESTT00000027381<br>ENSESTT00000038860<br>ENST00000343526<br>ENST00000378381<br>GENSCAN00000024892	Homo sapiens tripartite motif-containing 24 (TRIM24), transcript variant 2, mRNA.<br>Homo sapiens tripartite motif-containing 24 (TRIM24), transcript variant 1, mRNA.<br>full-length cDNA clone CS0DI034YA13 of Placenta Cot 25-normalized of Homo sapiens (human).<br><br><br>cdna:known-ccds chromosome:NCBI36:7:137795619:137920851:1 gene:ENSG00000122779 CCDS5847.1<br>cdna:known chromosome:NCBI36:7:137796350:137921233:1 gene:ENSG00000122779<br>cdna:Genscan chromosome:NCBI36:7:137826030:137920236:1

	443372<br>443373<br>443374<br>443375<br>443376<br>443377<br>443378<br>443379<br>443380<br>443381<br>443382<br>443383<br>443384<br>443385<br>443386<br>443387<br>443388<br>443389<br>443390	3026630<br>3026631<br>3026632<br>3026633<br>3026634<br>3026635<br>3026636<br>3026637<br>3026638<br>3026639<br>3026640<br>3026641<br>3026642<br>3026643<br>3026644<br>3026645<br>3026646<br>3026647<br>3026648<br>3026649<br>3026650<br>3026651<br>3026652<br>3026653<br>3026654<br>3026655<br>3026656<br>3026657<br>3026658<br>3026659<br>3026660<br>3026661<br>3026662<br>3026663<br>3026664<br>3026665<br>3026666<br>3026667<br>3026668	extended<br>extended<br>extended<br>core<br>extended<br>extended<br>core<br>core<br>extended<br>extended<br>extended<br>extended<br>core<br>core<br>core<br>extended<br>extended<br>core<br>core<br>core<br>core<br>core<br>extended<br>core<br>core<br>full<br>core<br>core<br>core<br>extended<br>core<br>core<br>core<br>core<br>core<br>core<br>extended<br>extended<br>extended						
3038065	450356<br>450357<br>450358<br>450359<br>450360<br>450361<br>450362<br>450363<br>450364<br>450365<br>450366<br>450367<br>450368<br>450369<br>450370	3038066<br>3038067<br>3038068<br>3038069<br>3038070<br>3038071<br>3038072<br>3038073<br>3038074<br>3038075<br>3038076<br>3038077<br>3038078<br>3038079<br>3038080	extended<br>extended<br>full<br>full<br>full<br>full<br>full<br>full<br>full<br>full<br>full<br>extended<br>core<br>core<br>core	7	8094327	8291312	-	NM_004968<br>NM_022307<br>BC005922<br>U26592<br>CR605198<br>CR624499<br>ENSESTT00000050117<br>ENSESTT00000050122<br>ENSESTT00000050123<br>ENST00000265577<br>ENST00000339809<br>ENST00000317367<br>GENSCAN00000040231<br>GENSCAN00000038359<br>GENSCAN00000038358	Homo sapiens islet cell autoantigen 1, 69kDa (ICA1), transcript variant 2, mRNA.<br>Homo sapiens islet cell autoantigen 1, 69kDa (ICA1), transcript variant 1, mRNA.<br>Homo sapiens islet cell autoantigen 1, 69kDa, mRNA (cDNA clone MGC:14523 IMAGE:4133583), complete cds.<br>Human clone IS4 diabetes mellitus type I autoantigen (ICAp69) mRNA, complete cds.<br>full-length cDNA clone CS0DJ008YI18 of T cells (Jurkat cell line) Cot 10-normalized of Homo sapiens (human).<br>full-length cDNA clone CL0BB019ZA07 of Neuroblastoma of Homo sapiens (human).<br><br><br><br>cdna:known chromosome:NCBI36:7:8119940:8268693:-1 gene:ENSG00000003147<br>cdna:known chromosome:NCBI36:7:8119940:8268693:-1 gene:ENSG00000003147<br>cdna:known chromosome:NCBI36:7:8163029:8268710:-1 gene:ENSG00000003147<br>cdna:Genscan chromosome:NCBI36:7:8094277:8123846:-1<br>cdna:Genscan chromosome:NCBI36:7:8131945:8150112:-1<br>cdna:Genscan chromosome:NCBI36:7:8158236:8218876:-1

	450371	3038081	core					GENSCAN00000038356	cdna:Genscan chromosome:NCBI36:7:8222049:8268671:-1	
	450372	3038082	extended							
	450373	3038083	extended							
	450374	3038084	extended							
	450375	3038085	extended							
	450376	3038086	extended							
	450377	3038087	extended							
	450378	3038088	extended							
	450379	3038089	extended							
	450380	3038090	extended							
	450381	3038091	extended							
	450382	3038092	extended							
	450383	3038093	extended							
	450384	3038094	extended							
	450385	3038095	extended							
	450386	3038096	full							
	450387	3038097	full							
	450388	3038098	full							
	450389	3038099	core							
	450390	3038100	core							
	450391	3038101	full							
	450392	3038102	full							
	450393	3038103	extended							
	450394	3038104	full							
	450395	3038105	extended							
	450396	3038106	core							
	450397	3038107	core							
	450398	3038108	core							
	450399	3038109	extended							
	450400	3038110	core							
	450401	3038111	extended							
	450402	3038112	full							
	450403	3038113	full							
	450404	3038114	full							
	450405	3038115	core							
	450406	3038116	core							
	450407	3038117	core							
	450408	3038118	core							
	450409	3038119	core							
	450410	3038120	extended							
	450411	3038121	extended							
	450412	3038122	extended							
	450413	3038123	extended							
	450414	3038124	core							
	450415	3038125	core							
	450416	3038126	extended							
	450417	3038127	full							
	450418	3038128	extended							
	450419	3038129	extended							
	450420	3038130	extended							
	450421	3038131	full							
	450422	3038132	full							
	450423	3038133	full							
	450424	3038134	full							

	450425<br>450426<br>450427<br>450428<br>450429<br>450430<br>450431<br>450432<br>450433<br>450434<br>450435<br>450436<br>450437<br>450438<br>450439<br>450440<br>450441	3038135<br>3038136<br>3038137<br>3038138<br>3038139<br>3038140<br>3038141<br>3038142<br>3038143<br>3038144<br>3038145<br>3038146<br>3038147<br>3038148<br>3038149<br>3038150<br>3038151<br>3038152<br>3038153<br>3038154<br>3038155<br>3038156<br>3038157<br>3038158<br>3038159<br>3038160<br>3038161<br>3038162<br>3038163<br>3038164<br>3038165<br>3038166<br>3038167<br>3038168<br>3038169<br>3038170<br>3038171<br>3038172<br>3038173<br>3038174<br>3038175<br>3038176<br>3038177<br>3038178<br>3038179<br>3038180	full<br>full<br>extended<br>extended<br>full<br>full<br>full<br>full<br>full<br>extended<br>full<br>full<br>full<br>full<br>full<br>core<br>extended<br>core<br>full<br>full<br>extended<br>core<br>full<br>core<br>core<br>core<br>core<br>extended<br>extended<br>extended<br>full<br>full<br>full<br>full<br>extended<br>extended<br>extended<br>full<br>core<br>extended<br>extended<br>core<br>extended<br>full<br>full<br>full						
3046444	455638<br>455639<br>455640<br>455641<br>455642<br>455643<br>455644<br>455645	3046445<br>3046446<br>3046447<br>3046448<br>3046449<br>3046450<br>3046451<br>3046452	full<br>full<br>core<br>core<br>core<br>core<br>core<br>core	7	37907848	38031802	-	NM_003014<br>BC032828<br>BC058911<br>ENSESTT00000014891<br>ENST00000223214<br>GENSCAN00000040951	Homo sapiens secreted frizzled-related protein 4 (SFRP4), mRNA.<br>Homo sapiens secreted frizzled-related protein 4, mRNA (cDNA clone MGC:26498 IMAGE:4828181), complete cds.<br>Homo sapiens secreted frizzled-related protein 4, mRNA (cDNA clone MGC:65015 IMAGE:5228231), complete cds.<br><br>cdna:known-ccds chromosome:NCBI36:7:37912247:37922903:-1 gene:ENSG00000106483 CCDS5453.1<br>cdna:Genscan chromosome:NCBI36:7:37907848:37922664:-1

	455646<br>455647<br>455648<br>455649<br>455650<br>455651<br>455652<br>455653	3046453<br>3046454<br>3046455<br>3046456<br>3046457<br>3046458<br>3046459<br>3046460<br>3046461<br>3046462<br>3046463<br>3046464<br>3046465<br>3046466<br>3046467<br>3046468<br>3046469<br>3046470	core<br>extended<br>extended<br>full<br>core<br>extended<br>core<br>core<br>core<br>core<br>full<br>full<br>core<br>core<br>core<br>full<br>extended<br>extended						
3047581	456330<br>456331<br>456332<br>456333<br>456334<br>456335<br>456336	3047582<br>3047583<br>3047584<br>3047585<br>3047586<br>3047587<br>3047588<br>3047589<br>3047590<br>3047591<br>3047592<br>3047593<br>3047594<br>3047595<br>3047596<br>3047597<br>3047598<br>3047599<br>3047600	core<br>core<br>core<br>core<br>core<br>core<br>extended<br>extended<br>extended<br>extended<br>extended<br>extended<br>extended<br>extended<br>core<br>core<br>core<br>extended<br>extended	7	41694877	41709211	-	NM_002192<br>M13436<br>BX648811<br>ENSESTT00000021044<br>ENSESTT00000021045<br>ENSESTT00000021046<br>ENST00000242208<br>GENSCAN00000013491	Homo sapiens inhibin, beta A (activin A, activin AB alpha polypeptide) (INHBA), mRNA.<br>Human ovarian beta-A inhibin mRNA, complete cds.<br>Homo sapiens mRNA; cDNA DKFZp686A06204 (from clone DKFZp686A06204).<br><br><br><br>cdna:known-ccds chromosome:NCBI36:7:41695126:41709231:-1 gene:ENSG00000122641 CCDS5464.1<br>cdna:Genscan chromosome:NCBI36:7:41663877:41729204:-1
3049522	457503<br>457504<br>457505<br>457506<br>457507<br>457508<br>457509<br>457510<br>457511<br>457512<br>457513<br>457514<br>457515<br>457516<br>457517<br>457518	3049523<br>3049524<br>3049525<br>3049526<br>3049527<br>3049528<br>3049529<br>3049530<br>3049531<br>3049532<br>3049533<br>3049534<br>3049535<br>3049536<br>3049537<br>3049538	full<br>full<br>extended<br>full<br>full<br>full<br>full<br>extended<br>full<br>full<br>full<br>full<br>extended<br>full<br>extended<br>core	7	47216313	47599851	-	NM_022748<br>AF147341<br>AK023967<br>AK092864<br>BC071791<br>BX648770<br>BX649002<br>ENSESTT00000030472<br>ENSESTT00000030473<br>ENSESTT00000030474<br>ENSESTT00000030475<br>ENSESTT00000030476<br>ENSESTT00000030477<br>ENSESTT00000030478<br>ENSESTT00000030479<br>ENSESTT00000030480	Homo sapiens tensin 3 (TNS3), mRNA.<br>Homo sapiens full length insert cDNA clone YB29B09.<br>Homo sapiens cDNA FLJ13905 fis, clone THYRO1001907.<br>Homo sapiens cDNA FLJ35545 fis, clone SPLEN2003918, moderately similar to TENSIN.<br>Homo sapiens tensin 3, mRNA (cDNA clone IMAGE:4611842), complete cds.<br>Homo sapiens mRNA; cDNA DKFZp686K12123 (from clone DKFZp686K12123).<br>Homo sapiens mRNA; cDNA DKFZp686G2290 (from clone DKFZp686G2290).

	457519	3049539	core					ENSESTT00000030481	
	457520	3049540	core					ENSESTT00000030482	
	457521	3049541	core					ENST00000355730	cdna:known-ccds chromosome:NCBI36:7:47281279:47588216:-1 gene:ENSG00000136205 CCDS5506.1
	457522	3049542	core					ENST00000311160	cdna:known chromosome:NCBI36:7:47281279:47588216:-1 gene:ENSG00000136205
	457523	3049543	core					GENSCAN00000033803	cdna:Genscan chromosome:NCBI36:7:47542155:47543230:-1
	457524	3049544	core					GENSCAN00000002584	cdna:Genscan chromosome:NCBI36:7:47218738:47219181:-1
	457525	3049545	core					GENSCAN00000000239	cdna:Genscan chromosome:NCBI36:7:47284199:47353643:-1
	457526	3049546	core					GENSCAN00000000241	cdna:Genscan chromosome:NCBI36:7:47372814:47380168:-1
	457527	3049547	core						
	457528	3049548	extended						
	457529	3049549	core						
	457530	3049550	core						
	457531	3049551	extended						
	457532	3049552	extended						
	457533	3049553	core						
	457534	3049554	full						
	457535	3049555	extended						
	457536	3049556	extended						
	457537	3049557	extended						
	457538	3049558	core						
	457539	3049559	core						
	457540	3049560	full						
	457541	3049561	core						
	457542	3049562	extended						
	457543	3049563	core						
	457544	3049564	extended						
	457545	3049565	extended						
	457546	3049566	extended						
	457547	3049567	extended						
	457548	3049568	extended						
	457549	3049569	core						
	457550	3049570	core						
	457551	3049571	full						
	457552	3049572	core						
	457553	3049573	full						
	457554	3049574	extended						
	457555	3049575	extended						
	457556	3049576	full						
	457557	3049577	full						
	457558	3049578	extended						
	457559	3049579	extended						
	457560	3049580	full						
	457561	3049581	full						
	457562	3049582	full						
	457563	3049583	full						
	457564	3049584	extended						
	457565	3049585	extended						
	457566	3049586	full						
	457567	3049587	extended						
	457568	3049588	extended						
	457569	3049589	extended						
	457570	3049590	extended						
	457571	3049591	extended						
	457572	3049592	extended						

	457573	3049593	extended							
	457574	3049594	full							
	457575	3049595	core							
	457576	3049596	core							
	457577	3049597	core							
	457578	3049598	core							
	457579	3049599	full							
	457580	3049600	extended							
	457581	3049601	full							
	457582	3049602	full							
	457583	3049603	full							
	457584	3049604	full							
	457585	3049605	extended							
	457586	3049606	full							
	457587	3049607	full							
	457588	3049608	core							
	457589	3049609	core							
	457590	3049610	core							
	457591	3049611	core							
	457592	3049612	core							
	457593	3049613	full							
	457594	3049614	extended							
	457595	3049615	full							
	457596	3049616	full							
	457597	3049617	full							
	457598	3049618	full							
	457599	3049619	full							
	457600	3049620	extended							
	457601	3049621	core							
	457602	3049622	core							
	457603	3049623	core							
	457604	3049624	extended							
	457605	3049625	core							
	457606	3049626	full							
	457607	3049627	full							
	457608	3049628	full							
	457609	3049629	full							
	457610	3049630	full							
	457611	3049631	extended							
	457612	3049632	core							
	457613	3049633	full							
	457614	3049634	core							
	457615	3049635	full							
	457616	3049636	full							
	457617	3049637	core							
	457618	3049638	extended							
	457619	3049639	extended							
	457620	3049640	extended							
	457621	3049641	core							
	457622	3049642	extended							
	457623	3049643	core							
	457624	3049644	extended							
	457625	3049645	core							
	457626	3049646	core							

	457627<br>457628<br>457629<br>457630<br>457631	3049647<br>3049648<br>3049649<br>3049650<br>3049651<br>3049652<br>3049653<br>3049654<br>3049655<br>3049656<br>3049657<br>3049658<br>3049659<br>3049660<br>3049661<br>3049662<br>3049663<br>3049664<br>3049665<br>3049666<br>3049667<br>3049668<br>3049669<br>3049670<br>3049671<br>3049672<br>3049673<br>3049674<br>3049675<br>3049676<br>3049677<br>3049678<br>3049679<br>3049680<br>3049681<br>3049682	core<br>full<br>extended<br>full<br>extended<br>extended<br>full<br>extended<br>extended<br>extended<br>extended<br>extended<br>core<br>extended<br>extended<br>full<br>extended<br>full<br>core<br>full<br>extended<br>extended<br>extended<br>extended<br>full<br>extended<br>full<br>core<br>extended<br>extended<br>full<br>core<br>extended<br>full<br>full<br>full						
3064293	466664<br>466665<br>466666<br>466667<br>466668<br>466669<br>466670<br>466671<br>466672<br>466673<br>466674<br>466675<br>466676<br>466677<br>466678<br>466679<br>466680<br>466681	3064294<br>3064295<br>3064296<br>3064297<br>3064298<br>3064299<br>3064300<br>3064301<br>3064302<br>3064303<br>3064304<br>3064305<br>3064306<br>3064307<br>3064308<br>3064309<br>3064310<br>3064311	core<br>core<br>core<br>full<br>core<br>core<br>core<br>full<br>full<br>core<br>full<br>core<br>full<br>core<br>core<br>core<br>core<br>extended	7	100238146	100263025	-	NM_004444<br>AB209644<br>ENSESTT00000028523<br>ENSESTT00000028524<br>ENST00000358173<br>ENST00000360620<br>GENSCAN00000020813	Homo sapiens EPH receptor B4 (EPHB4), mRNA.<br>Homo sapiens mRNA for ephrin receptor EphB4 precursor variant protein.<br><br><br>cdna:known-ccds chromosome:NCBI36:7:100238123:100263079:-1 gene:ENSG00000196411 CCDS5706.1<br>cdna:known chromosome:NCBI36:7:100238123:100263079:-1 gene:ENSG00000196411<br>cdna:Genscan chromosome:NCBI36:7:100239019:100262588:-1

	466682<br>466683<br>466684<br>466685<br>466686	3064312<br>3064313<br>3064314<br>3064315<br>3064316<br>3064317<br>3064318<br>3064319<br>3064320<br>3064321<br>3064322<br>3064323<br>3064324<br>3064325<br>3064326<br>3064327<br>3064328<br>3064329<br>3064330<br>3064331<br>3064332<br>3064333	core<br>full<br>core<br>extended<br>core<br>core<br>extended<br>core<br>core<br>extended<br>core<br>core<br>full<br>core<br>core<br>core<br>full<br>core<br>full<br>full<br>core<br>core						
3066436	467959<br>467960<br>467961<br>467962<br>467963<br>467964<br>467965<br>467966<br>467967<br>467968<br>467969<br>467970<br>467971<br>467972<br>467973<br>467974<br>467975<br>467976<br>467977<br>467978<br>467979<br>467980<br>467981<br>467982<br>467983<br>467984<br>467985<br>467986<br>467987	3066437<br>3066438<br>3066439<br>3066440<br>3066441<br>3066442<br>3066443<br>3066444<br>3066445<br>3066446<br>3066447<br>3066448<br>3066449<br>3066450<br>3066451<br>3066452<br>3066453<br>3066454<br>3066455<br>3066456<br>3066457<br>3066458<br>3066459<br>3066460<br>3066461<br>3066462<br>3066463<br>3066464<br>3066465<br>3066466<br>3066467<br>3066468	extended<br>core<br>core<br>core<br>core<br>core<br>core<br>core<br>extended<br>core<br>core<br>core<br>core<br>core<br>core<br>full<br>full<br>core<br>core<br>extended<br>extended<br>core<br>core<br>core<br>extended<br>extended<br>extended<br>extended<br>extended<br>full<br>core<br>extended	7	104884010	104949979	-	NM_019042<br>ENSESTT00000025989<br>ENSESTT00000025991<br>ENST00000356362<br>GENSCAN00000025553<br>GENSCAN00000019342	Homo sapiens pseudouridylate synthase 7 homolog (S. cerevisiae) (PUS7), mRNA.<br><br><br>cdna:known chromosome:NCBI36:7:104884194:104949921:-1 gene:ENSG00000091127<br>cdna:Genscan chromosome:NCBI36:7:104885473:104896146:-1<br>cdna:Genscan chromosome:NCBI36:7:104898371:104936195:-1

		3066469<br>3066470<br>3066471<br>3066472<br>3066473<br>3066474<br>3066475<br>3066476<br>3066477	full<br>core<br>extended<br>core<br>extended<br>core<br>full<br>core<br>full						
3069366	469893<br>469894<br>469895<br>469896<br>469897<br>469898<br>469899<br>469900<br>469901<br>469902<br>469903<br>469904<br>469905<br>469906<br>469907<br>469908<br>469909	3069367<br>3069368<br>3069369<br>3069370<br>3069371<br>3069372<br>3069373<br>3069374<br>3069375<br>3069376<br>3069377<br>3069378<br>3069379<br>3069380<br>3069381<br>3069382<br>3069383<br>3069384<br>3069385<br>3069386<br>3069387<br>3069388<br>3069389<br>3069390<br>3069391<br>3069392	extended<br>core<br>core<br>core<br>core<br>core<br>core<br>full<br>full<br>full<br>full<br>core<br>full<br>full<br>extended<br>core<br>core<br>core<br>full<br>core<br>full<br>core<br>core<br>full<br>full<br>full	7	116703941	116752152	-	NM_003391<br>CR610337<br>ENSESTT00000028337<br>ENSESTT00000028338<br>ENST00000265441<br>GENSCAN00000053585<br>GENSCAN00000009241	Homo sapiens wingless-type MMTV integration site family member 2 (WNT2), mRNA.<br>full-length cDNA clone CS0DI057YJ18 of Placenta Cot 25-normalized of Homo sapiens (human).<br><br><br>cdna:known-ccds chromosome:NCBI36:7:116704518:116750579:-1 gene:ENSG00000105989 CCDS5771.1<br>cdna:Genscan chromosome:NCBI36:7:116723946:116854750:-1<br>cdna:Genscan chromosome:NCBI36:7:116690237:116705674:-1
3079803	476495<br>476496<br>476497<br>476498<br>476499<br>476500<br>476501<br>476502<br>476503<br>476504<br>476505<br>476506<br>476507<br>476508<br>476509<br>476510<br>476511<br>476512	3079804<br>3079805<br>3079806<br>3079807<br>3079808<br>3079809<br>3079810<br>3079811<br>3079812<br>3079813<br>3079814<br>3079815<br>3079816<br>3079817<br>3079818<br>3079819<br>3079820<br>3079821	full<br>core<br>core<br>core<br>core<br>core<br>extended<br>full<br>extended<br>extended<br>full<br>full<br>core<br>core<br>extended<br>core<br>extended<br>extended	7	150865031	151205160	-	XM_932013<br>NM_024429<br>NM_016203<br>NM_001040633<br>AK074675<br>AK130536<br>BC020540<br>CR933725<br>ENSESTT00000042310<br>ENSESTT00000042311<br>ENSESTT00000042312<br>ENSESTT00000042313<br>ENST00000287878<br>GENSCAN00000068126<br>GENSCAN00000061088<br>GENSCAN00000061091<br>GENSCAN00000030887<br>GENSCAN00000006331	PREDICTED: Homo sapiens hypothetical protein LOC644134 (LOC644134), mRNA.<br>Homo sapiens protein kinase, AMP-activated, gamma 2 non-catalytic subunit (PRKAG2), transcript variant b, mRNA.<br>Homo sapiens protein kinase, AMP-activated, gamma 2 non-catalytic subunit (PRKAG2), transcript variant a, mRNA.<br>Homo sapiens protein kinase, AMP-activated, gamma 2 non-catalytic subunit (PRKAG2), transcript variant c, mRNA.<br>Homo sapiens cDNA FLJ90194 fis, clone MAMMA1001284.<br>Homo sapiens cDNA FLJ27026 fis, clone SLV07378.<br>Homo sapiens protein kinase, AMP-activated, gamma 2 non-catalytic subunit, mRNA (cDNA clone IMAGE:4413055), partial cds.<br>Homo sapiens mRNA; cDNA DKFZp781C1522 (from clone DKFZp781C1522).<br><br><br><br><br>cdna:known-ccds chromosome:NCBI36:7:150884960:151204728:-1 gene:ENSG00000106617 CCDS5928.1<br>cdna:Genscan chromosome:NCBI36:7:151107627:151123417:-1<br>cdna:Genscan chromosome:NCBI36:7:151076970:151088035:-1<br>cdna:Genscan chromosome:NCBI36:7:150953970:151072932:-1<br>cdna:Genscan chromosome:NCBI36:7:150884746:150928831:-1

	476513	3079822	core						cdna:Genscan chromosome:NCBI36:7:151190838:151204623:-1
	476514	3079823	core						
	476515	3079824	core						
	476516	3079825	core						
	476517	3079826	extended						
	476518	3079827	extended						
	476519	3079828	core						
	476520	3079829	extended						
	476521	3079830	extended						
	476522	3079831	core						
	476523	3079832	core						
	476524	3079833	extended						
	476525	3079834	extended						
	476526	3079835	extended						
	476527	3079836	core						
	476528	3079837	core						
	476529	3079838	full						
	476530	3079839	full						
	476531	3079840	extended						
	476532	3079841	extended						
	476533	3079842	full						
	476534	3079843	full						
	476535	3079844	extended						
	476536	3079845	full						
	476537	3079846	extended						
	476538	3079847	full						
	476539	3079848	full						
	476540	3079849	full						
	476541	3079850	full						
	476542	3079851	extended						
	476543	3079852	extended						
	476544	3079853	extended						
	476545	3079854	core						
	476546	3079855	core						
	476547	3079856	core						
	476548	3079857	extended						
	476549	3079858	extended						
	476550	3079859	extended						
	476551	3079860	extended						
	476552	3079861	full						
	476553	3079862	extended						
	476554	3079863	extended						
	476555	3079864	extended						
	476556	3079865	extended						
	476557	3079866	full						
	476558	3079867	full						
	476559	3079868	full						
	476560	3079869	extended						
	476561	3079870	full						
	476562	3079871	extended						
	476563	3079872	core						
	476564	3079873	full						
	476565	3079874	full						
	476566	3079875	full						

	476567	3079876	full						
	476568	3079877	extended						
	476569	3079878	full						
	476570	3079879	full						
	476571	3079880	full						
	476572	3079881	full						
	476573	3079882	full						
	476574	3079883	full						
	476575	3079884	full						
	476576	3079885	full						
	476577	3079886	full						
	476578	3079887	full						
	476579	3079888	full						
	476580	3079889	full						
	476581	3079890	full						
	476582	3079891	full						
	476583	3079892	extended						
	476584	3079893	extended						
	476585	3079894	extended						
	476586	3079895	extended						
	476587	3079896	extended						
	476588	3079897	extended						
	476589	3079898	extended						
	476590	3079899	extended						
	476591	3079900	extended						
	476592	3079901	extended						
	476593	3079902	extended						
	476594	3079903	extended						
	476595	3079904	extended						
	476596	3079905	full						
	476597	3079906	full						
	476598	3079907	full						
	476599	3079908	full						
	476600	3079909	full						
	476601	3079910	extended						
	476602	3079911	full						
	476603	3079912	extended						
	476604	3079913	extended						
	476605	3079914	extended						
	476606	3079915	extended						
	476607	3079916	extended						
	476608	3079917	extended						
	476609	3079918	full						
	476610	3079919	full						
	476611	3079920	extended						
	476612	3079921	extended						
	476613	3079922	extended						
	476614	3079923	full						
	476615	3079924	extended						
	476616	3079925	core						
	476617	3079926	core						
		3079927	core						
		3079928	extended						
		3079929	extended						

		3079930<br>3079931<br>3079932<br>3079933<br>3079934<br>3079935<br>3079936<br>3079937<br>3079938<br>3079939<br>3079940<br>3079941<br>3079942<br>3079943<br>3079944<br>3079945<br>3079946<br>3079947<br>3079948<br>3079949<br>3079950<br>3079951<br>3079952<br>3079953<br>3079954<br>3079955<br>3079956<br>3079957<br>3079958<br>3079959<br>3079960<br>3079961<br>3079962<br>3079963<br>3079964<br>3079965<br>3079966<br>3079967<br>3079968<br>3079969<br>3079970	core<br>extended<br>extended<br>full<br>full<br>extended<br>extended<br>core<br>extended<br>full<br>full<br>full<br>full<br>extended<br>extended<br>full<br>full<br>full<br>full<br>full<br>extended<br>extended<br>extended<br>extended<br>extended<br>full<br>extended<br>full<br>extended<br>full<br>full<br>full<br>full<br>full<br>full<br>full<br>full<br>extended<br>extended<br>core<br>extended						
3080283	476819<br>476820<br>476821<br>476822<br>476823<br>476824<br>476825<br>476826<br>476827	3080284<br>3080285<br>3080286<br>3080287<br>3080288<br>3080289<br>3080290<br>3080291<br>3080292<br>3080293<br>3080294<br>3080295<br>3080296	extended<br>core<br>core<br>core<br>core<br>core<br>full<br>full<br>full<br>extended<br>core<br>full<br>core	7	151974018	152004178	-	ENSESTT00000033077<br>GENSCAN00000002298<br>NM_005431	 cdna:Genscan chromosome:NCBI36:7:151976821:152003913:-1<br>Homo sapiens X-ray repair complementing defective repair in Chinese hamster cells 2 (XRCC2), mRNA.

		3080297	core						
3082181	478035	3082182	full	7	158069413	158190349	-	BC028618	Homo sapiens leucine zipper protein 5, mRNA (cDNA clone IMAGE:4821510), with apparent retained intron.
	478036	3082183	full					ENSESTT00000023186	
	478037	3082184	extended					ENSESTT00000023187	
	478038	3082185	full					ENSESTT00000023188	
	478039	3082186	full					ENSESTT00000023189	
	478040	3082187	extended					ENSESTT00000023190	
	478041	3082188	extended					ENST00000275830	cdna:known chromosome:NCBI36:7:158116772:158190224:-1 gene:ENSG00000146918
	478042	3082189	core					ENST00000356309	cdna:known chromosome:NCBI36:7:158126917:158190224:-1 gene:ENSG00000146918
	478043	3082190	core					GENSCAN00000057318	cdna:Genscan chromosome:NCBI36:7:158136219:158187351:-1
	478044	3082191	extended					GENSCAN00000057325	cdna:Genscan chromosome:NCBI36:7:158189519:158208984:-1
	478045	3082192	extended						
	478046	3082193	core						
	478047	3082194	core						
	478048	3082195	core						
	478049	3082196	core						
	478050	3082197	core						
	478051	3082198	core						
	478052	3082199	extended						
	478053	3082200	full						
	478054	3082201	core						
	478055	3082202	core						
	478056	3082203	core						
	478057	3082204	extended						
	478058	3082205	core						
	478059	3082206	extended						
	478060	3082207	core						
	478061	3082208	core						
	478062	3082209	core						
	478063	3082210	core						
	478064	3082211	extended						
	478065	3082212	core						
	478066	3082213	core						
	478067	3082214	core						
	478068	3082215	extended						
	478069	3082216	core						
	478070	3082217	core						
	478071	3082218	core						
	478072	3082219	extended						
	478073	3082220	core						
	478074	3082221	core						
	478075	3082222	core						
	478076	3082223	extended						
	478077	3082224	core						
	478078	3082225	core						
	478079	3082226	core						
	478080	3082227	core						
		3082228	core						
		3082229	core						
		3082230	core						
		3082231	core						
		3082232	core						
		3082233	extended						
		3082234	core						

		3082235<br>3082236<br>3082237<br>3082238<br>3082239	core<br>extended<br>extended<br>extended<br>full						
3092808	484701<br>484702<br>484703<br>484704<br>484705<br>484706<br>484707<br>484708<br>484709<br>484710<br>484711<br>484712<br>484713<br>484714<br>484715<br>484716<br>484717<br>484718<br>484719<br>484720<br>484721<br>484722<br>484723<br>484724<br>484725<br>484726<br>484727<br>484728<br>484729<br>484730<br>484731<br>484732<br>484733<br>484734<br>484735<br>484736<br>484737<br>484738<br>484739<br>484740<br>484741<br>484742<br>484743<br>484744<br>484745<br>484746<br>484747<br>484748<br>484749	3092809<br>3092810<br>3092811<br>3092812<br>3092813<br>3092814<br>3092815<br>3092816<br>3092817<br>3092818<br>3092819<br>3092820<br>3092821<br>3092822<br>3092823<br>3092824<br>3092825<br>3092826<br>3092827<br>3092828<br>3092829<br>3092830<br>3092831<br>3092832<br>3092833<br>3092834<br>3092835<br>3092836<br>3092837<br>3092838<br>3092839<br>3092840<br>3092841<br>3092842<br>3092843<br>3092844<br>3092845<br>3092846<br>3092847<br>3092848<br>3092849<br>3092850<br>3092851<br>3092852<br>3092853<br>3092854<br>3092855<br>3092856<br>3092857	full<br>full<br>full<br>full<br>full<br>full<br>full<br>core<br>core<br>core<br>core<br>core<br>core<br>core<br>full<br>full<br>full<br>full<br>extended<br>full<br>full<br>extended<br>extended<br>extended<br>full<br>full<br>extended<br>extended<br>extended<br>extended<br>extended<br>full<br>full<br>full<br>full<br>full<br>full<br>extended<br>extended<br>extended<br>full<br>extended<br>extended<br>full<br>full<br>full<br>full<br>extended<br>full	8	31605732	32744997	+	XM_926044<br>NM_004495<br>NM_013962<br>NM_013961<br>NM_013964<br>NM_013959<br>NM_013960<br>NM_013957<br>NM_013958<br>NM_013956<br>AY207002<br>U02327<br>U02330<br>CR594949<br>AF176921<br>ENSESTT00000038014<br>ENSESTT00000038015<br>ENSESTT00000038016<br>ENSESTT00000038017<br>ENSESTT00000038018<br>ENST00000287842<br>ENST00000287840<br>ENST00000287845<br>ENST00000356819<br>ENST00000287838<br>ENST00000338921<br>ENST00000355277<br>ENST00000341377<br>ENST00000339085<br>GENSCAN00000062782<br>GENSCAN00000004363<br>GENSCAN00000045879<br>GENSCAN00000008919<br>GENSCAN00000008918	PREDICTED: Homo sapiens similar to neuregulin 1 isoform GGF2 (LOC653104), mRNA.<br>Homo sapiens neuregulin 1 (NRG1), transcript variant HRG-gamma, mRNA.<br>Homo sapiens neuregulin 1 (NRG1), transcript variant GGF2, mRNA.<br>Homo sapiens neuregulin 1 (NRG1), transcript variant GGF, mRNA.<br>Homo sapiens neuregulin 1 (NRG1), transcript variant HRG-alpha, mRNA.<br>Homo sapiens neuregulin 1 (NRG1), transcript variant SMDF, mRNA.<br>Homo sapiens neuregulin 1 (NRG1), transcript variant ndf43, mRNA.<br>Homo sapiens neuregulin 1 (NRG1), transcript variant HRG-beta2, mRNA.<br>Homo sapiens neuregulin 1 (NRG1), transcript variant HRG-beta3, mRNA.<br>Homo sapiens neuregulin 1 (NRG1), transcript variant HRG-beta1, mRNA.<br>Homo sapiens neuregulin 1 isoform 4 (NRG1) mRNA, partial cds, alternatively spliced.<br>Human clone ndf19 neu differentiation factor mRNA, partial cds.<br>Human clone ndf33 neu differentiation factor mRNA, partial cds.<br>full-length cDNA clone CS0DF002YN04 of Fetal brain of Homo sapiens (human).<br>Homo sapiens MSTP131 (MST131) mRNA, complete cds.<br><br><br><br><br><br>cdna:known-ccds chromosome:NCBI36:8:32525327:32741561:1 gene:ENSG00000157168 CCDS6084.1<br>cdna:known-ccds chromosome:NCBI36:8:32525371:32741531:1 gene:ENSG00000157168 CCDS6085.1<br>cdna:known-ccds chromosome:NCBI36:8:32525679:32741058:1 gene:ENSG00000157168 CCDS6086.1<br>cdna:known-ccds chromosome:NCBI36:8:32525695:32741608:1 gene:ENSG00000157168 CCDS6083.1<br>cdna:known-ccds chromosome:NCBI36:8:32624136:32720284:1 gene:ENSG00000157168 CCDS6087.1<br>cdna:known chromosome:NCBI36:8:31617043:32719828:1 gene:ENSG00000157168<br>cdna:known chromosome:NCBI36:8:32525371:32741531:1 gene:ENSG00000157168<br>cdna:known chromosome:NCBI36:8:32525371:32719778:1 gene:ENSG00000157168<br>cdna:known chromosome:NCBI36:8:32743185:32744926:1 gene:ENSG00000187508<br>cdna:Genscan chromosome:NCBI36:8:32699063:32719828:1<br>cdna:Genscan chromosome:NCBI36:8:31617043:31643279:1<br>cdna:Genscan chromosome:NCBI36:8:32397043:32418396:1<br>cdna:Genscan chromosome:NCBI36:8:32511598:32624363:1<br>cdna:Genscan chromosome:NCBI36:8:32624800:32643431:1

	484750	3092858	extended						
	484751	3092859	extended						
	484752	3092860	full						
	484753	3092861	full						
	484754	3092862	extended						
	484755	3092863	full						
	484756	3092864	core						
	484757	3092865	core						
	484758	3092866	core						
	484759	3092867	core						
	484760	3092868	core						
	484761	3092869	core						
	484762	3092870	extended						
	484763	3092871	extended						
	484764	3092872	extended						
	484765	3092873	full						
	484766	3092874	full						
	484767	3092875	full						
	484768	3092876	core						
	484769	3092877	extended						
	484770	3092878	full						
	484771	3092879	core						
	484772	3092880	full						
	484773	3092881	full						
	484774	3092882	extended						
	484775	3092883	core						
	484776	3092884	core						
	484777	3092885	full						
	484778	3092886	extended						
	484779	3092887	extended						
	484780	3092888	core						
	484781	3092889	core						
	484782	3092890	core						
	484783	3092891	full						
	484784	3092892	full						
	484785	3092893	extended						
	484786	3092894	extended						
	484787	3092895	full						
		3092896	core						
		3092897	core						
		3092898	full						
		3092899	core						
		3092900	full						
		3092901	full						
		3092902	core						
		3092903	core						
		3092904	core						
		3092905	core						
		3092906	extended						
		3092907	full						
		3092908	core						
		3092909	core						
		3092910	core						
		3092911	core						

		3092912<br>3092913<br>3092914<br>3092915<br>3092916<br>3092917<br>3092918<br>3092919<br>3092920<br>3092921<br>3092922<br>3092923	core<br>extended<br>extended<br>full<br>core<br>core<br>core<br>core<br>core<br>core<br>core<br>free						
3102372	490692<br>490693<br>490694<br>490695<br>490696<br>490697<br>490698<br>490699<br>490700<br>490701<br>490702<br>490703<br>490704<br>490705<br>490706<br>490707<br>490708<br>490709<br>490710<br>490711<br>490712<br>490713<br>490714<br>490715<br>490716<br>490717<br>490718<br>490719<br>490720<br>490721<br>490722<br>490723<br>490724<br>490725<br>490726<br>490727<br>490728<br>490729<br>490730<br>490731<br>490732<br>490733	3102373<br>3102374<br>3102375<br>3102376<br>3102377<br>3102378<br>3102379<br>3102380<br>3102381<br>3102382<br>3102383<br>3102384<br>3102385<br>3102386<br>3102387<br>3102388<br>3102389<br>3102390<br>3102391<br>3102392<br>3102393<br>3102394<br>3102395<br>3102396<br>3102397<br>3102398<br>3102399<br>3102400<br>3102401<br>3102402<br>3102403<br>3102404<br>3102405<br>3102406<br>3102407<br>3102408<br>3102409<br>3102410<br>3102411<br>3102412<br>3102413<br>3102414	core<br>extended<br>full<br>extended<br>full<br>full<br>full<br>core<br>core<br>full<br>extended<br>extended<br>extended<br>core<br>full<br>full<br>full<br>full<br>core<br>core<br>core<br>full<br>extended<br>full<br>full<br>core<br>extended<br>full<br>extended<br>extended<br>full<br>core<br>core<br>full<br>core<br>full<br>full<br>core<br>core<br>core<br>extended<br>core	8	70541423	70735701	+	NM_015170<br>AK055467<br>BC068565<br>BX571746<br>ENSESTT00000023351<br>ENSESTT00000023352<br>ENSESTT00000023353<br>ENSESTT00000023354<br>ENSESTT00000052664<br>ENSESTT00000052665<br>ENSESTT00000052666<br>ENSESTT00000052667<br>ENSESTT00000052668<br>ENST00000260128<br>GENSCAN00000056157<br>GENSCAN00000056160	Homo sapiens sulfatase 1 (SULF1), mRNA.<br>Homo sapiens cDNA FLJ30905 fis, clone FEBRA2005998.<br>Homo sapiens sulfatase 1, mRNA (cDNA clone MGC:87514 IMAGE:4831138), complete cds.<br>Homo sapiens mRNA; cDNA DKFZp686F13142 (from clone DKFZp686F13142).<br><br><br><br><br><br><br><br><br><br>cdna:known-ccds chromosome:NCBI36:8:70541427:70735701:1 gene:ENSG00000137573 CCDS6204.1<br>cdna:Genscan chromosome:NCBI36:8:70650737:70682774:1<br>cdna:Genscan chromosome:NCBI36:8:70567359:70571876:1

	490734<br>490735<br>490736<br>490737<br>490738<br>490739<br>490740<br>490741<br>490742<br>490743<br>490744<br>490745<br>490746<br>490747<br>490748<br>490749<br>490750<br>490751<br>490752<br>490753<br>490754<br>490755<br>490756<br>490757<br>490758<br>490759<br>490760<br>490761<br>490762	3102415<br>3102416<br>3102417<br>3102418<br>3102419<br>3102420<br>3102421<br>3102422<br>3102423<br>3102424<br>3102425<br>3102426<br>3102427<br>3102428<br>3102429<br>3102430<br>3102431<br>3102432<br>3102433<br>3102434<br>3102435<br>3102436<br>3102437<br>3102438<br>3102439<br>3102440<br>3102441<br>3102442<br>3102443<br>3102444<br>3102445<br>3102446<br>3102447<br>3102448<br>3102449<br>3102450<br>3102451<br>3102452<br>3102453<br>3102454<br>3102455<br>3102456<br>3102457<br>3102458<br>3102459<br>3102460<br>3102461<br>3102462<br>3102463<br>3102464<br>3102465	full<br>full<br>core<br>extended<br>core<br>core<br>extended<br>full<br>extended<br>extended<br>full<br>extended<br>extended<br>extended<br>extended<br>core<br>core<br>extended<br>extended<br>core<br>core<br>full<br>core<br>core<br>core<br>extended<br>extended<br>extended<br>core<br>extended<br>core<br>extended<br>core<br>extended<br>extended<br>core<br>core<br>extended<br>extended<br>extended<br>extended<br>full<br>full<br>full<br>full<br>core<br>core<br>core<br>core<br>core<br>core						
3105600	492790<br>492791<br>492792	3105601<br>3105602<br>3105603	full<br>extended<br>full	8	86555021	86580971	+	NM_000067<br>AK123309<br>BC011949	Homo sapiens carbonic anhydrase II (CA2), mRNA.<br>Homo sapiens cDNA FLJ41315 fis, clone BRAMY2043069.<br>Homo sapiens carbonic anhydrase II, mRNA (cDNA clone MGC:9006 IMAGE:3863603), complete cds.

	492793<br>492794<br>492795<br>492796<br>492797<br>492798<br>492799<br>492800<br>492801<br>492802<br>492803<br>492804<br>492805<br>492806<br>492807<br>492808<br>492809<br>492810	3105604<br>3105605<br>3105606<br>3105607<br>3105608<br>3105609<br>3105610<br>3105611<br>3105612<br>3105613<br>3105614<br>3105615<br>3105616<br>3105617<br>3105618<br>3105619<br>3105620<br>3105621<br>3105622<br>3105623<br>3105624<br>3105625<br>3105626<br>3105627<br>3105628<br>3105629<br>3105630	extended<br>full<br>extended<br>extended<br>core<br>extended<br>full<br>core<br>extended<br>extended<br>extended<br>extended<br>core<br>core<br>extended<br>extended<br>core<br>extended<br>extended<br>extended<br>core<br>extended<br>extended<br>core<br>core<br>core<br>core					S69527<br>ENSESTT00000014430<br>ENSESTT00000035288<br>ENSESTT00000035289<br>ENST00000285379<br>GENSCAN00000049625<br>GENSCAN00000049623	carbonic anhydrase II {clone pHispa, exon 7} [human, Caribbean Hispanic patient, leukocytes, mRNA Partial Mutant, 53 nt].<br><br><br><br>cdna:known-ccds chromosome:NCBI36:8:86563433:86580945:1 gene:ENSG00000104267 CCDS6239.1<br>cdna:Genscan chromosome:NCBI36:8:86563019:86580270:1<br>cdna:Genscan chromosome:NCBI36:8:86555021:86561652:1
3106243	493205<br>493206<br>493207<br>493208<br>493209<br>493210<br>493211<br>493212<br>493213<br>493214<br>493215<br>493216<br>493217<br>493218<br>493219<br>493220<br>493221<br>493222<br>493223<br>493224	3106244<br>3106245<br>3106246<br>3106247<br>3106248<br>3106249<br>3106250<br>3106251<br>3106252<br>3106253<br>3106254<br>3106255<br>3106256<br>3106257<br>3106258<br>3106259<br>3106260<br>3106261<br>3106262<br>3106263<br>3106264<br>3106265<br>3106266<br>3106267<br>3106268<br>3106269<br>3106270	full<br>core<br>core<br>core<br>core<br>core<br>core<br>core<br>core<br>extended<br>core<br>extended<br>extended<br>core<br>core<br>extended<br>core<br>core<br>core<br>core<br>core<br>extended<br>full<br>extended<br>extended<br>full<br>full	8	90838676	90905190	+	NM_003821<br>ENSESTT00000051561<br>ENSESTT00000051564<br>ENST00000220751<br>GENSCAN00000041765<br>GENSCAN00000041764<br>GENSCAN00000041763	Homo sapiens receptor-interacting serine-threonine kinase 2 (RIPK2), mRNA.<br><br><br>cdna:known-ccds chromosome:NCBI36:8:90839173:90872432:1 gene:ENSG00000104312 CCDS6247.1<br>cdna:Genscan chromosome:NCBI36:8:90839424:90851298:1<br>cdna:Genscan chromosome:NCBI36:8:90853083:90896893:1<br>cdna:Genscan chromosome:NCBI36:8:90904834:90905190:1

		3106271	full						
3109687	495423<br>495424<br>495425<br>495426<br>495427<br>495428<br>495429<br>495430<br>495431<br>495432<br>495433<br>495434<br>495435<br>495436<br>495437<br>495438<br>495439<br>495440<br>495441<br>495442<br>495443<br>495444<br>495445<br>495446<br>495447<br>495448<br>495449<br>495450<br>495451<br>495452<br>495453<br>495454<br>495455<br>495456<br>495457<br>495458<br>495459<br>495460<br>495461<br>495462<br>495463<br>495464<br>495465<br>495466<br>495467<br>495468<br>495469<br>495470<br>495471<br>495472<br>495473<br>495474<br>495475	3109688<br>3109689<br>3109690<br>3109691<br>3109692<br>3109693<br>3109694<br>3109695<br>3109696<br>3109697<br>3109698<br>3109699<br>3109700<br>3109701<br>3109702<br>3109703<br>3109704<br>3109705<br>3109706<br>3109707<br>3109708<br>3109709<br>3109710<br>3109711<br>3109712<br>3109713<br>3109714<br>3109715<br>3109716<br>3109717<br>3109718<br>3109719<br>3109720<br>3109721<br>3109722<br>3109723<br>3109724<br>3109725<br>3109726<br>3109727<br>3109728<br>3109729<br>3109730<br>3109731<br>3109732<br>3109733<br>3109734<br>3109735<br>3109736<br>3109737<br>3109738<br>3109739<br>3109740	full<br>full<br>full<br>full<br>full<br>full<br>full<br>core<br>extended<br>extended<br>full<br>full<br>full<br>full<br>full<br>extended<br>extended<br>extended<br>full<br>extended<br>extended<br>full<br>full<br>core<br>core<br>extended<br>extended<br>extended<br>core<br>core<br>full<br>extended<br>full<br>extended<br>core<br>extended<br>core<br>full<br>core<br>full<br>full<br>full<br>full<br>full<br>extended<br>full<br>full<br>core<br>full<br>extended<br>full<br>full<br>full	8	102573566	102751110	+	NM_024915<br>BC069633<br>ENSESTT00000019210<br>ENSESTT00000019211<br>ENSESTT00000019212<br>ENST00000251808<br>GENSCAN00000048548	Homo sapiens grainyhead-like 2 (Drosophila) (GRHL2), mRNA.<br>Homo sapiens grainyhead-like 2 (Drosophila), mRNA (cDNA clone MGC:97159 IMAGE:7262401), complete cds.<br><br><br><br>cdna:known chromosome:NCBI36:8:102574162:102750995:1 gene:ENSG00000083307<br>cdna:Genscan chromosome:NCBI36:8:102624673:102658923:1

	495476<br>495477<br>495478<br>495479<br>495480<br>495481<br>495482<br>495483<br>495484	3109741<br>3109742<br>3109743<br>3109744<br>3109745<br>3109746<br>3109747<br>3109748<br>3109749<br>3109750<br>3109751<br>3109752<br>3109753<br>3109754<br>3109755<br>3109756<br>3109757<br>3109758<br>3109759<br>3109760<br>3109761<br>3109762<br>3109763<br>3109764<br>3109765<br>3109766<br>3109767<br>3109768	full<br>full<br>core<br>core<br>core<br>full<br>extended<br>core<br>core<br>extended<br>full<br>core<br>extended<br>extended<br>full<br>full<br>full<br>full<br>full<br>full<br>full<br>full<br>full<br>core<br>extended<br>core<br>core<br>core						
3110317	495836<br>495837<br>495838<br>495839<br>495840<br>495841<br>495842<br>495843<br>495844<br>495845	3110318<br>3110319<br>3110320<br>3110321<br>3110322<br>3110323<br>3110324<br>3110325<br>3110326<br>3110327<br>3110328<br>3110329<br>3110330<br>3110331<br>3110332<br>3110333<br>3110334<br>3110335<br>3110336	core<br>core<br>core<br>core<br>full<br>full<br>full<br>full<br>core<br>core<br>core<br>core<br>extended<br>core<br>extended<br>extended<br>full<br>core<br>core	8	104452919	104464388	+	NM_138455<br>AF395488<br>ENSESTT00000053027<br>ENSESTT00000053028<br>ENST00000330295<br>ENST00000297577<br>GENSCAN00000004992	Homo sapiens collagen triple helix repeat containing 1 (CTHRC1), mRNA.<br>Homo sapiens NTMC1 mRNA, complete cds.<br><br><br>cdna:known-ccds chromosome:NCBI36:8:104453061:104464397:1 gene:ENSG00000164932 CCDS6299.1<br>cdna:known chromosome:NCBI36:8:104453837:104464371:1 gene:ENSG00000164932<br>cdna:Genscan chromosome:NCBI36:8:104453061:104459647:1
3125571	505373<br>505374<br>505375<br>505376<br>505377<br>505378	3125572<br>3125573<br>3125574<br>3125575<br>3125576<br>3125577	full<br>full<br>full<br>full<br>full<br>full	8	15944161	16116934	-	NM_138715<br>NM_002445<br>NM_138716<br>BC063878<br>ENSESTT00000010844<br>ENSESTT00000010845	Homo sapiens macrophage scavenger receptor 1 (MSR1), transcript variant SR-AI, mRNA.<br>Homo sapiens macrophage scavenger receptor 1 (MSR1), transcript variant SR-AII, mRNA.<br>Homo sapiens macrophage scavenger receptor 1 (MSR1), transcript variant SR-AIII, mRNA.<br>Homo sapiens macrophage scavenger receptor 1, mRNA (cDNA clone MGC:75441 IMAGE:30378951), complete cds.

	505379<br>505380<br>505381<br>505382<br>505383<br>505384<br>505385<br>505386<br>505387<br>505388<br>505389<br>505390<br>505391<br>505392<br>505393<br>505394<br>505395<br>505396<br>505397<br>505398<br>505399<br>505400<br>505401<br>505402<br>505403<br>505404<br>505405<br>505406<br>505407<br>505408<br>505409<br>505410<br>505411<br>505412	3125578<br>3125579<br>3125580<br>3125581<br>3125582<br>3125583<br>3125584<br>3125585<br>3125586<br>3125587<br>3125588<br>3125589<br>3125590<br>3125591<br>3125592<br>3125593<br>3125594<br>3125595<br>3125596<br>3125597<br>3125598<br>3125599<br>3125600<br>3125601<br>3125602<br>3125603<br>3125604<br>3125605<br>3125606<br>3125607<br>3125608<br>3125609<br>3125610<br>3125611<br>3125612<br>3125613<br>3125614<br>3125615<br>3125616<br>3125617<br>3125618<br>3125619<br>3125620	full<br>full<br>full<br>extended<br>full<br>full<br>extended<br>core<br>full<br>core<br>extended<br>full<br>full<br>extended<br>full<br>extended<br>extended<br>core<br>core<br>core<br>core<br>full<br>core<br>core<br>full<br>core<br>extended<br>core<br>core<br>core<br>full<br>core<br>core<br>core<br>core<br>full<br>core<br>extended<br>core<br>core<br>full<br>extended<br>extended					ENSESTT00000010846<br>ENSESTT00000010847<br>ENST00000262101<br>ENST00000350896<br>ENST00000381998<br>ENST00000382001<br>ENST00000355282<br>ENST00000308443<br>GENSCAN00000000576	 <br>cdna:known-ccds chromosome:NCBI36:8:16009761:16094595:-1 gene:ENSG00000038945 CCDS5995.1<br>cdna:known-ccds chromosome:NCBI36:8:16009761:16094595:-1 gene:ENSG00000038945 CCDS5996.1<br>cdna:known-ccds chromosome:NCBI36:8:16041197:16094539:-1 gene:ENSG00000038945 CCDS5997.1<br>cdna:known chromosome:NCBI36:8:16009761:16094595:-1 gene:ENSG00000038945<br>cdna:known chromosome:NCBI36:8:16011965:16079868:-1 gene:ENSG00000038945<br>cdna:known chromosome:NCBI36:8:16041199:16094595:-1 gene:ENSG00000038945<br>cdna:Genscan chromosome:NCBI36:8:15950325:15950592:-1
3129149	507640<br>507641<br>507642<br>507643<br>507644<br>507645<br>507646<br>507647<br>507648<br>507649<br>507650	3129150<br>3129151<br>3129152<br>3129153<br>3129154<br>3129155<br>3129156<br>3129157<br>3129158<br>3129159<br>3129160	core<br>core<br>core<br>core<br>extended<br>core<br>core<br>extended<br>extended<br>extended<br>extended	8	27723084	27751415	-	NM_018492<br>ENSESTT00000045218<br>ENST00000380402<br>ENST00000301905<br>GENSCAN00000013099	Homo sapiens PDZ binding kinase (PBK), mRNA.<br><br>cdna:known-ccds chromosome:NCBI36:8:27723056:27751435:-1 gene:ENSG00000168078 CCDS6063.1<br>cdna:known chromosome:NCBI36:8:27723061:27751268:-1 gene:ENSG00000168078<br>cdna:Genscan chromosome:NCBI36:8:27723741:27736547:-1

	507651<br>507652	3129161<br>3129162<br>3129163<br>3129164<br>3129165<br>3129166<br>3129167<br>3129168<br>3129169<br>3129170<br>3129171<br>3129172	core<br>core<br>core<br>core<br>extended<br>extended<br>core<br>core<br>core<br>full<br>core<br>extended						
3134034	510536<br>510537<br>510538<br>510539<br>510540<br>510541<br>510542<br>510543<br>510544<br>510545<br>510546<br>510547<br>510548<br>510549<br>510550<br>510551<br>510552<br>510553<br>510554<br>510555<br>510556<br>510557<br>510558<br>510559<br>510560<br>510561<br>510562<br>510563<br>510564<br>510565<br>510566<br>510567<br>510568<br>510569<br>510570<br>510571<br>510572<br>510573<br>510574<br>510575<br>510576<br>510577	3134035<br>3134036<br>3134037<br>3134038<br>3134039<br>3134040<br>3134041<br>3134042<br>3134043<br>3134044<br>3134045<br>3134046<br>3134047<br>3134048<br>3134049<br>3134050<br>3134051<br>3134052<br>3134053<br>3134054<br>3134055<br>3134056<br>3134057<br>3134058<br>3134059<br>3134060<br>3134061<br>3134062<br>3134063<br>3134064<br>3134065<br>3134066<br>3134067<br>3134068<br>3134069<br>3134070<br>3134071<br>3134072<br>3134073<br>3134074<br>3134075<br>3134076	full<br>full<br>full<br>full<br>full<br>full<br>full<br>full<br>full<br>full<br>core<br>core<br>core<br>core<br>core<br>core<br>extended<br>core<br>core<br>extended<br>extended<br>extended<br>extended<br>core<br>core<br>core<br>core<br>extended<br>extended<br>core<br>extended<br>extended<br>core<br>core<br>extended<br>core<br>core<br>core<br>extended<br>core<br>core<br>full	8	48806794	49035296	-	NM_006904<br>XM_940235<br>AK022387<br>BC037968<br>ENSESTT00000048296<br>ENSESTT00000048297<br>ENSESTT00000048298<br>ENSESTT00000048299<br>ENSESTT00000048300<br>ENSESTT00000048301<br>ENSESTT00000048302<br>ENSESTT00000048303<br>ENSESTT00000048304<br>ENSESTT00000048305<br>ENSESTT00000048306<br>ENST00000314191<br>ENST00000338368<br>GENSCAN00000030144	Homo sapiens protein kinase, DNA-activated, catalytic polypeptide (PRKDC), mRNA.<br>PREDICTED: Homo sapiens similar to protein kinase, DNA-activated, catalytic polypeptide (LOC651106), mRNA.<br>Homo sapiens cDNA FLJ12325 fis, clone MAMMA1002125.<br>Homo sapiens, Similar to protein kinase, DNA-activated, catalytic polypeptide, clone IMAGE:4508982, mRNA.<br><br><br><br><br><br><br><br><br><br><br><br>cdna:known chromosome:NCBI36:8:48848222:49035296:-1 gene:ENSG00000121031<br>cdna:known chromosome:NCBI36:8:48848222:49035296:-1 gene:ENSG00000121031<br>cdna:Genscan chromosome:NCBI36:8:48849287:49004291:-1

	510578	3134077	core						
	510579	3134078	core						
	510580	3134079	core						
	510581	3134080	full						
	510582	3134081	core						
	510583	3134082	core						
	510584	3134083	core						
	510585	3134084	core						
	510586	3134085	core						
	510587	3134086	core						
	510588	3134087	core						
	510589	3134088	core						
	510590	3134089	core						
	510591	3134090	core						
	510592	3134091	extended						
	510593	3134092	core						
	510594	3134093	extended						
	510595	3134094	core						
	510596	3134095	core						
	510597	3134096	full						
	510598	3134097	full						
	510599	3134098	core						
	510600	3134099	full						
	510601	3134100	core						
	510602	3134101	core						
	510603	3134102	core						
	510604	3134103	core						
	510605	3134104	core						
	510606	3134105	core						
	510607	3134106	full						
	510608	3134107	core						
	510609	3134108	core						
	510610	3134109	full						
	510611	3134110	full						
	510612	3134111	core						
	510613	3134112	core						
	510614	3134113	core						
	510615	3134114	extended						
	510616	3134115	core						
	510617	3134116	core						
	510618	3134117	core						
	510619	3134118	core						
	510620	3134119	core						
	510621	3134120	core						
	510622	3134121	core						
	510623	3134122	core						
	510624	3134123	extended						
	510625	3134124	core						
	510626	3134125	core						
	510627	3134126	core						
	510628	3134127	core						
	510629	3134128	extended						
	510630	3134129	core						
	510631	3134130	core						

	510632	3134131	extended						
	510633	3134132	core						
	510634	3134133	core						
	510635	3134134	full						
	510636	3134135	extended						
	510637	3134136	extended						
	510638	3134137	core						
	510639	3134138	core						
	510640	3134139	core						
	510641	3134140	core						
	510642	3134141	core						
	510643	3134142	core						
	510644	3134143	full						
	510645	3134144	full						
	510646	3134145	core						
	510647	3134146	core						
	510648	3134147	core						
	510649	3134148	core						
	510650	3134149	extended						
	510651	3134150	full						
	510652	3134151	core						
	510653	3134152	full						
	510654	3134153	full						
	510655	3134154	core						
	510656	3134155	core						
	510657	3134156	core						
		3134157	extended						
		3134158	extended						
		3134159	core						
		3134160	core						
		3134161	core						
		3134162	extended						
		3134163	extended						
		3134164	extended						
		3134165	core						
		3134166	extended						
		3134167	extended						
		3134168	extended						
		3134169	core						
		3134170	extended						
		3134171	core						
		3134172	core						
		3134173	core						
		3134174	core						
		3134175	extended						
		3134176	core						
		3134177	core						
		3134178	full						
		3134179	core						
		3134180	core						
		3134181	core						
		3134182	core						
		3134183	extended						
		3134184	core						

		3134185<br>3134186<br>3134187<br>3134188<br>3134189<br>3134190<br>3134191<br>3134192<br>3134193<br>3134194<br>3134195<br>3134196<br>3134197<br>3134198	extended<br>core<br>core<br>core<br>core<br>core<br>core<br>core<br>core<br>core<br>core<br>extended<br>core<br>core						
3142967	516144<br>516145<br>516146<br>516147<br>516148<br>516149<br>516150<br>516151<br>516152<br>516153<br>516154<br>516155<br>516156<br>516157<br>516158<br>516159<br>516160<br>516161<br>516162<br>516163<br>516164<br>516165<br>516166<br>516167<br>516168<br>516169<br>516170<br>516171<br>516172<br>516173<br>516174<br>516175<br>516176<br>516177<br>516178<br>516179	3142968<br>3142969<br>3142970<br>3142971<br>3142972<br>3142973<br>3142974<br>3142975<br>3142976<br>3142977<br>3142978<br>3142979<br>3142980<br>3142981<br>3142982<br>3142983<br>3142984<br>3142985<br>3142986<br>3142987<br>3142988<br>3142989<br>3142990<br>3142991<br>3142992<br>3142993<br>3142994<br>3142995<br>3142996<br>3142997<br>3142998<br>3142999<br>3143000<br>3143001<br>3143002<br>3143003<br>3143004<br>3143005<br>3143006<br>3143007	full<br>full<br>full<br>full<br>full<br>full<br>full<br>full<br>core<br>core<br>core<br>core<br>core<br>core<br>extended<br>extended<br>core<br>core<br>extended<br>core<br>extended<br>extended<br>extended<br>extended<br>extended<br>core<br>extended<br>extended<br>core<br>extended<br>extended<br>extended<br>extended<br>full<br>extended<br>extended<br>full<br>full<br>core<br>full	8	86396770	86478514	-	NM_001738<br>BC027890<br>ENSESTT00000035290<br>ENST00000256119<br>GENSCAN00000015077<br>GENSCAN00000015079	Homo sapiens carbonic anhydrase I (CA1), mRNA.<br>Homo sapiens carbonic anhydrase I, mRNA (cDNA clone MGC:34628 IMAGE:5222602), complete cds.<br><br>cdna:known-ccds chromosome:NCBI36:8:86427089:86478495:-1 gene:ENSG00000133742 CCDS6237.1<br>cdna:Genscan chromosome:NCBI36:8:86468236:86489616:-1<br>cdna:Genscan chromosome:NCBI36:8:86396770:86463161:-1

		3143008<br>3143009<br>3143010<br>3143011<br>3143012<br>3143013<br>3143014<br>3143015<br>3143016<br>3143017<br>3143018	extended<br>full<br>full<br>extended<br>extended<br>extended<br>extended<br>extended<br>extended<br>core<br>full						
3144973	517400<br>517401<br>517402<br>517403<br>517404<br>517405<br>517406<br>517407<br>517408<br>517409<br>517410<br>517411<br>517412<br>517413<br>517414<br>517415<br>517416<br>517417<br>517418<br>517419<br>517420<br>517421<br>517422<br>517423<br>517424<br>517425<br>517426<br>517427<br>517428<br>517429<br>517430<br>517431	3144970<br>3144972<br>3144974<br>3144975<br>3144976<br>3144977<br>3144978<br>3144979<br>3144980<br>3144981<br>3144982<br>3144983<br>3144984<br>3144985<br>3144986<br>3144987<br>3144988<br>3144989<br>3144990<br>3144991<br>3144992<br>3144993<br>3144994<br>3144995<br>3144996<br>3144997<br>3144998<br>3144999<br>3145000<br>3145001<br>3145002<br>3145003<br>3145004<br>3145005<br>3145006<br>3145007<br>3145008<br>3145009<br>3145010<br>3145011<br>3145012<br>3145013<br>3145014	full<br>free<br>core<br>core<br>extended<br>core<br>core<br>core<br>core<br>core<br>core<br>extended<br>extended<br>core<br>core<br>core<br>extended<br>core<br>core<br>core<br>full<br>core<br>extended<br>core<br>core<br>core<br>core<br>extended<br>extended<br>extended<br>extended<br>extended<br>extended<br>core<br>core<br>core<br>core<br>core<br>extended<br>extended<br>extended<br>core<br>extended	8	95434896	95556486	-	NM_012415<br>AF007866<br>AK074272<br>BC020668<br>CR602319<br>ENSESTT00000009462<br>ENSESTT00000009463<br>ENSESTT00000009464<br>ENSESTT00000009465<br>ENSESTT00000022600<br>ENST00000336148<br>ENST00000297592<br>GENSCAN00000021320<br>GENSCAN00000021321	Homo sapiens RAD54 homolog B (S. cerevisiae) (RAD54B), mRNA.<br>Homo sapiens fibrinogen silencer binding protein mRNA, complete cds.<br>Homo sapiens cDNA FLJ23692 fis, clone HEP10227.<br>Homo sapiens cDNA clone IMAGE:4773245.<br>full-length cDNA clone CS0DC004YN13 of Neuroblastoma Cot 25-normalized of Homo sapiens (human).<br><br><br><br><br><br>cdna:known-ccds chromosome:NCBI36:8:95453365:95556486:-1 gene:ENSG00000197275 CCDS6262.1<br>cdna:known-ccds chromosome:NCBI36:8:95513351:95518308:-1 gene:ENSG00000197065 CCDS6263.1<br>cdna:Genscan chromosome:NCBI36:8:95513535:95518272:-1<br>cdna:Genscan chromosome:NCBI36:8:95531512:95634851:-1

		3145015<br>3145016<br>3145017	core<br>extended<br>core						
3148796	519865<br>519866<br>519867<br>519868<br>519869<br>519870<br>519871<br>519872<br>519873<br>519874<br>519875<br>519876<br>519877<br>519878<br>519879<br>519880<br>519881<br>519882<br>519883<br>519884<br>519885<br>519886<br>519887<br>519888	3148797<br>3148798<br>3148799<br>3148800<br>3148801<br>3148802<br>3148803<br>3148804<br>3148805<br>3148806<br>3148807<br>3148808<br>3148809<br>3148810<br>3148811<br>3148812<br>3148813<br>3148814<br>3148815<br>3148816<br>3148817<br>3148818<br>3148819<br>3148820<br>3148821<br>3148822<br>3148823<br>3148824<br>3148825<br>3148826<br>3148827<br>3148828<br>3148829<br>3148830	core<br>core<br>core<br>core<br>core<br>extended<br>extended<br>core<br>extended<br>extended<br>extended<br>extended<br>extended<br>extended<br>core<br>extended<br>core<br>core<br>core<br>core<br>core<br>extended<br>extended<br>full<br>extended<br>core<br>core<br>core<br>extended<br>core<br>core<br>extended<br>extended<br>extended	8	110322324	110415934	-	NM_032869<br>AF283301<br>AF521133<br>BC031258<br>AF086207<br>ENSESTT00000054073<br>ENST00000239690<br>GENSCAN00000047712<br>GENSCAN00000047715	Homo sapiens NudC domain containing 1 (NUDCD1), mRNA.<br>Homo sapiens chronic myelogenous leukemia tumor antigen 66 mRNA, complete cds, alternatively spliced.<br>Homo sapiens tumor antigen CML66 short form mRNA, complete cds, alternatively spliced.<br>Homo sapiens NudC domain containing 1, mRNA (cDNA clone MGC:39710 IMAGE:5272186), complete cds.<br>Homo sapiens full length insert cDNA clone ZC48C05.<br><br>cdna:known-ccds chromosome:NCBI36:8:110322325:110415491:-1 gene:ENSG00000120526 CCDS6312.1<br>cdna:Genscan chromosome:NCBI36:8:110414830:110415415:-1<br>cdna:Genscan chromosome:NCBI36:8:110324414:110411216:-1
3150844	521258<br>521259<br>521260<br>521261<br>521262<br>521263<br>521264<br>521265<br>521266<br>521267<br>521268<br>521269<br>521270<br>521271<br>521272<br>521273	3150845<br>3150846<br>3150847<br>3150848<br>3150849<br>3150850<br>3150851<br>3150852<br>3150853<br>3150854<br>3150855<br>3150856<br>3150857<br>3150858<br>3150859<br>3150860	extended<br>extended<br>core<br>core<br>full<br>full<br>core<br>core<br>full<br>extended<br>full<br>extended<br>extended<br>extended<br>full<br>full	8	121617178	122014368	-	NM_021021<br>AF028828<br>AK025100<br>AK026095<br>ENSESTT00000031965<br>ENSESTT00000031966<br>ENSESTT00000031967<br>ENSESTT00000031968<br>ENST00000346808<br>GENSCAN00000000454<br>GENSCAN00000068873<br>GENSCAN00000039166	Homo sapiens syntrophin, beta 1 (dystrophin-associated protein A1, 59kDa, basic component 1) (SNTB1), mRNA.<br>Homo sapiens Tax interaction protein 43 mRNA, partial cds.<br>Homo sapiens cDNA: FLJ21447 fis, clone COL04468.<br>Homo sapiens cDNA: FLJ22442 fis, clone HRC09342, highly similar to AF028828 Homo sapiens Tax interaction protein 43 mRNA.<br><br><br><br><br>cdna:known-ccds chromosome:NCBI36:8:121619297:121893264:-1 gene:ENSG00000172164 CCDS6334.1<br>cdna:Genscan chromosome:NCBI36:8:121840793:121893264:-1<br>cdna:Genscan chromosome:NCBI36:8:121622131:121674434:-1<br>cdna:Genscan chromosome:NCBI36:8:121719152:121719559:-1

	521274	3150861	extended						
	521275	3150862	extended						
	521276	3150863	extended						
	521277	3150864	core						
	521278	3150865	extended						
	521279	3150866	full						
	521280	3150867	extended						
	521281	3150868	full						
	521282	3150869	full						
	521283	3150870	full						
	521284	3150871	full						
	521285	3150872	full						
	521286	3150873	full						
	521287	3150874	extended						
	521288	3150875	extended						
	521289	3150876	extended						
	521290	3150877	full						
	521291	3150878	full						
	521292	3150879	full						
	521293	3150880	full						
	521294	3150881	full						
	521295	3150882	core						
	521296	3150883	full						
	521297	3150884	full						
	521298	3150885	full						
	521299	3150886	full						
	521300	3150887	full						
	521301	3150888	full						
	521302	3150889	full						
	521303	3150890	full						
	521304	3150891	full						
	521305	3150892	extended						
	521306	3150893	extended						
	521307	3150894	full						
	521308	3150895	extended						
	521309	3150896	full						
	521310	3150897	full						
	521311	3150898	full						
	521312	3150899	extended						
	521313	3150900	full						
	521314	3150901	extended						
	521315	3150902	extended						
	521316	3150903	extended						
	521317	3150904	extended						
	521318	3150905	full						
	521319	3150906	extended						
	521320	3150907	full						
	521321	3150908	full						
	521322	3150909	full						
	521323	3150910	full						
	521324	3150911	core						
	521325	3150912	extended						
	521326	3150913	full						
	521327	3150914	full						

	521328	3150915	full						
	521329	3150916	full						
	521330	3150917	full						
	521331	3150918	full						
	521332	3150919	extended						
	521333	3150920	extended						
	521334	3150921	extended						
	521335	3150922	full						
	521336	3150923	extended						
	521337	3150924	full						
	521338	3150925	extended						
	521339	3150926	extended						
	521340	3150927	full						
	521341	3150928	full						
	521342	3150929	full						
	521343	3150930	full						
	521344	3150931	full						
	521345	3150932	full						
	521346	3150933	full						
	521347	3150934	extended						
	521348	3150935	extended						
	521349	3150936	extended						
	521350	3150937	extended						
	521351	3150938	extended						
	521352	3150939	full						
	521353	3150940	full						
	521354	3150941	core						
	521355	3150942	core						
	521356	3150943	core						
	521357	3150944	core						
	521358	3150945	core						
	521359	3150946	core						
	521360	3150947	core						
	521361	3150948	extended						
	521362	3150949	core						
	521363	3150950	full						
		3150951	extended						
		3150952	full						
		3150953	full						
		3150954	full						
		3150955	full						
		3150956	full						
		3150957	full						
		3150958	full						
		3150959	extended						
		3150960	full						
		3150961	full						
		3150962	full						
		3150963	full						
		3150964	extended						
		3150965	full						
		3150966	full						
		3150967	full						
		3150968	full						

		3150969	full						
3151534	521705<br>521706<br>521707<br>521708<br>521709<br>521710<br>521711<br>521712<br>521713<br>521714<br>521715<br>521716<br>521717<br>521718<br>521719<br>521720<br>521721<br>521722<br>521723<br>521724<br>521725<br>521726<br>521727<br>521728<br>521729<br>521730<br>521731<br>521732<br>521733<br>521734<br>521735<br>521736<br>521737<br>521738<br>521739<br>521740<br>521741<br>521742<br>521743<br>521744<br>521745<br>521746<br>521747<br>521748<br>521749<br>521750<br>521751<br>521752	3151535<br>3151536<br>3151537<br>3151538<br>3151539<br>3151540<br>3151541<br>3151542<br>3151543<br>3151544<br>3151545<br>3151546<br>3151547<br>3151548<br>3151549<br>3151550<br>3151551<br>3151552<br>3151553<br>3151554<br>3151555<br>3151556<br>3151557<br>3151558<br>3151559<br>3151560<br>3151561<br>3151562<br>3151563<br>3151564<br>3151565<br>3151566<br>3151567<br>3151568<br>3151569<br>3151570<br>3151571<br>3151572<br>3151573<br>3151574<br>3151575<br>3151576<br>3151577<br>3151578<br>3151579<br>3151580<br>3151581<br>3151582<br>3151583<br>3151584<br>3151585<br>3151586<br>3151587	full<br>extended<br>extended<br>core<br>core<br>core<br>core<br>extended<br>extended<br>core<br>extended<br>core<br>extended<br>core<br>core<br>extended<br>core<br>core<br>core<br>core<br>extended<br>extended<br>extended<br>core<br>core<br>core<br>core<br>core<br>core<br>core<br>full<br>core<br>core<br>extended<br>extended<br>extended<br>core<br>core<br>core<br>core<br>core<br>core<br>extended<br>core<br>core<br>core<br>core<br>extended<br>core<br>core<br>core<br>full<br>core	8	124393174	124477871	-	NM_014109<br>BC007123<br>CR749832<br>ENSESTT00000036353<br>ENSESTT00000036354<br>ENSESTT00000036355<br>ENST00000287394<br>GENSCAN00000061539	Homo sapiens ATPase family, AAA domain containing 2 (ATAD2), mRNA.<br>Homo sapiens ATPase family, AAA domain containing 2, mRNA (cDNA clone IMAGE:3454033), complete cds.<br>Homo sapiens mRNA; cDNA DKFZp781K1122 (from clone DKFZp781K1122).<br><br><br><br>cdna:known-ccds chromosome:NCBI36:8:124401922:124477868:-1 gene:ENSG00000156802 CCDS6343.1<br>cdna:Genscan chromosome:NCBI36:8:124404359:124477778:-1

		3151588<br>3151589<br>3151590<br>3151591<br>3151592<br>3151593<br>3151594<br>3151595<br>3151596<br>3151597	extended<br>extended<br>extended<br>full<br>extended<br>extended<br>full<br>full<br>core<br>core						
3152220	522147<br>522148<br>522149<br>522150<br>522151<br>522152<br>522153<br>522154<br>522155<br>522156<br>522157<br>522158<br>522159<br>522160<br>522161<br>522162<br>522163<br>522164<br>522165<br>522166<br>522167<br>522168<br>522169<br>522170<br>522171<br>522172<br>522173<br>522174<br>522175<br>522176<br>522177<br>522178<br>522179<br>522180<br>522181<br>522182<br>522183<br>522184<br>522185<br>522186<br>522187	3152221<br>3152222<br>3152223<br>3152224<br>3152225<br>3152226<br>3152227<br>3152228<br>3152229<br>3152230<br>3152231<br>3152232<br>3152233<br>3152234<br>3152235<br>3152236<br>3152237<br>3152238<br>3152239<br>3152240<br>3152241<br>3152242<br>3152243<br>3152244<br>3152245<br>3152246<br>3152247<br>3152248<br>3152249<br>3152250<br>3152251<br>3152252<br>3152253<br>3152254<br>3152255<br>3152256<br>3152257<br>3152258<br>3152259<br>3152260<br>3152261<br>3152262<br>3152263<br>3152264	core<br>core<br>core<br>extended<br>extended<br>extended<br>core<br>full<br>full<br>extended<br>core<br>extended<br>extended<br>core<br>core<br>core<br>full<br>extended<br>core<br>core<br>extended<br>core<br>core<br>core<br>core<br>extended<br>core<br>full<br>core<br>core<br>core<br>core<br>core<br>core<br>extended<br>core<br>extended<br>extended<br>core<br>full<br>core<br>core<br>full<br>core	8	126105691	126173311	-	NM_014846<br>CR620759<br>ENSESTT00000048886<br>ENST00000318410<br>ENST00000377985<br>GENSCAN00000041797	Homo sapiens KIAA0196 (KIAA0196), mRNA.<br>full-length cDNA clone CS0DE003YC24 of Placenta of Homo sapiens (human).<br><br>cdna:known-ccds chromosome:NCBI36:8:126105691:126173191:-1 gene:ENSG00000164961 CCDS6355.1<br>cdna:novel chromosome:NCBI36:8:126105691:126173191:-1 gene:ENSG00000164961<br>cdna:Genscan chromosome:NCBI36:8:126105901:126163185:-1

		3152265<br>3152266<br>3152267<br>3152268<br>3152269<br>3152270<br>3152271<br>3152272<br>3152273<br>3152274<br>3152275<br>3152276<br>3152277	core<br>core<br>core<br>full<br>core<br>core<br>extended<br>core<br>core<br>core<br>extended<br>core<br>full						
3157385	525557<br>525558<br>525559<br>525560<br>525561<br>525562<br>525563<br>525564<br>525565<br>525566<br>525567<br>525568<br>525569<br>525570<br>525571<br>525572<br>525573<br>525574<br>525575<br>525576<br>525577<br>525578<br>525579<br>525580<br>525581<br>525582<br>525583<br>525584<br>525585<br>525586	3157386<br>3157387<br>3157388<br>3157389<br>3157390<br>3157391<br>3157392<br>3157393<br>3157394<br>3157395<br>3157396<br>3157397<br>3157398<br>3157399<br>3157400<br>3157401<br>3157402<br>3157403<br>3157404<br>3157405<br>3157406<br>3157407<br>3157408<br>3157409<br>3157410<br>3157411<br>3157412<br>3157413<br>3157414<br>3157415<br>3157416<br>3157417<br>3157418<br>3157419<br>3157420<br>3157421<br>3157422<br>3157423	full<br>full<br>core<br>core<br>core<br>extended<br>core<br>extended<br>extended<br>core<br>core<br>full<br>core<br>core<br>core<br>core<br>core<br>core<br>core<br>core<br>core<br>extended<br>core<br>core<br>full<br>extended<br>extended<br>extended<br>extended<br>extended<br>extended<br>full<br>core<br>full<br>full<br>full<br>full<br>full	8	144460532	144498845	-	NM_052963<br>CR602435<br>ENSESTT00000009162<br>ENSESTT00000009163<br>ENST00000329245<br>GENSCAN00000060305	Homo sapiens topoisomerase (DNA) I, mitochondrial (TOP1MT), nuclear gene encoding mitochondrial protein, mRNA.<br>full-length cDNA clone CS0DC018YN15 of Neuroblastoma Cot 25-normalized of Homo sapiens (human).<br><br><br>cdna:known-ccds chromosome:NCBI36:8:144462910:144488425:-1 gene:ENSG00000184428 CCDS6400.1<br>cdna:Genscan chromosome:NCBI36:8:144459023:144498845:-1
3168508	532323<br>532324	3168509<br>3168510	core<br>extended	9	36562870	36887829	+	NM_014791<br>ENSESTT00000032490	Homo sapiens maternal embryonic leucine zipper kinase (MELK), mRNA.

	532325	3168511	core					ENSESTT00000032491	
	532326	3168512	core					ENST00000298048	cdna:known-ccds chromosome:NCBI36:9:36562873:36667678:1 gene:ENSG00000165304 CCDS6606.1
	532327	3168513	core					GENSCAN00000059764	cdna:Genscan chromosome:NCBI36:9:36571679:36633080:1
	532328	3168514	core					GENSCAN00000024223	cdna:Genscan chromosome:NCBI36:9:36788005:36891078:1
	532329	3168515	core						
	532330	3168516	core						
	532331	3168517	core						
	532332	3168518	core						
	532333	3168519	core						
	532334	3168520	extended						
	532335	3168521	full						
	532336	3168522	core						
	532337	3168523	core						
	532338	3168524	core						
	532339	3168525	core						
	532340	3168526	core						
	532341	3168527	full						
	532342	3168528	core						
	532343	3168529	core						
	532344	3168530	core						
	532345	3168531	core						
	532346	3168532	extended						
	532347	3168533	core						
	532348	3168534	extended						
	532349	3168535	core						
	532350	3168536	core						
	532351	3168537	core						
	532352	3168538	extended						
	532353	3168539	extended						
	532354	3168540	full						
	532355	3168541	extended						
	532356	3168542	full						
	532357	3168543	full						
	532358	3168544	full						
	532359	3168545	full						
	532360	3168546	full						
	532361	3168547	full						
	532362	3168548	full						
	532363	3168549	full						
	532364	3168550	full						
	532365	3168551	full						
	532366	3168552	full						
	532367	3168553	full						
	532368	3168554	full						
	532369	3168555	full						
	532370	3168556	full						
	532371	3168557	full						
	532372	3168558	full						
	532373	3168559	full						
	532374	3168560	full						
	532375	3168561	full						
	532376	3168562	full						
	532377	3168563	full						
	532378	3168564	full						

	532379<br>532380<br>532381<br>532382<br>532383<br>532384<br>532385<br>532386<br>532387<br>532388<br>532389<br>532390<br>532391<br>532392<br>532393<br>532394<br>532395<br>532396<br>532397<br>532398	3168565<br>3168566<br>3168567<br>3168568<br>3168569<br>3168570<br>3168571<br>3168572<br>3168573<br>3168574<br>3168575<br>3168576<br>3168577<br>3168578<br>3168579<br>3168580<br>3168581<br>3168582<br>3168583<br>3168584<br>3168585<br>3168586<br>3168587<br>3168588<br>3168589<br>3168590<br>3168591<br>3168592<br>3168593<br>3168594<br>3168595<br>3168596<br>3168597<br>3168598<br>3168599	extended<br>full<br>full<br>full<br>extended<br>extended<br>extended<br>full<br>full<br>full<br>full<br>full<br>full<br>full<br>full<br>full<br>full<br>full<br>full<br>full<br>full<br>full<br>full<br>full<br>full<br>full<br>full<br>full<br>full<br>full<br>full<br>full<br>full<br>full<br>full						
3173880	535834<br>535835<br>535836<br>535837<br>535838<br>535839<br>535840<br>535841<br>535842<br>535843<br>535844<br>535845<br>535846<br>535847<br>535848<br>535849<br>535850<br>535851<br>535852	3173881<br>3173882<br>3173883<br>3173884<br>3173885<br>3173886<br>3173887<br>3173888<br>3173889<br>3173890<br>3173891<br>3173892<br>3173893<br>3173894<br>3173895<br>3173896<br>3173897<br>3173898<br>3173899	extended<br>extended<br>extended<br>extended<br>extended<br>core<br>core<br>core<br>core<br>core<br>extended<br>core<br>core<br>full<br>core<br>core<br>core<br>core<br>full	9	70925894	71059931	+	NM_201629<br>NM_004817<br>XM_932429<br>XM_942979<br>AF083893<br>AF489824<br>AK025185<br>U84581<br>ENSESTT00000027841<br>ENSESTT00000027842<br>ENSESTT00000027843<br>ENST00000377245<br>ENST00000348208<br>ENST00000377259<br>ENST00000326029<br>ENST00000265384<br>GENSCAN00000022994	Homo sapiens tight junction protein 2 (zona occludens 2) (TJP2), transcript variant 2, mRNA.<br>Homo sapiens tight junction protein 2 (zona occludens 2) (TJP2), transcript variant 1, mRNA.<br>PREDICTED: Homo sapiens hypothetical protein LOC644829 (LOC644829), mRNA.<br>PREDICTED: Homo sapiens hypothetical protein LOC647728 (LOC647728), mRNA.<br>Homo sapiens tight junction protein ZO-2 isoform C mRNA, partial cds.<br>Homo sapiens tight junction protein 2 (TJP2) mRNA, complete cds; alternatively spliced.<br>Homo sapiens cDNA: FLJ21532 fis, clone COL06049.<br>Human zonula occludens 2 protein mRNA, partial cds.<br><br><br><br>cdna:known-ccds chromosome:NCBI36:9:70978901:71059940:1 gene:ENSG00000119139 CCDS6627.1<br>cdna:known-ccds chromosome:NCBI36:9:70978909:71059938:1 gene:ENSG00000119139 CCDS6628.1<br>cdna:known chromosome:NCBI36:9:70956382:71023275:1 gene:ENSG00000119139<br>cdna:known chromosome:NCBI36:9:70978909:71059938:1 gene:ENSG00000119139<br>cdna:known chromosome:NCBI36:9:70979030:71053726:1 gene:ENSG00000119139<br>cdna:Genscan chromosome:NCBI36:9:71017293:71059110:1

	535853<br>535854<br>535855<br>535856<br>535857<br>535858<br>535859<br>535860<br>535861	3173900<br>3173901<br>3173902<br>3173903<br>3173904<br>3173905<br>3173906<br>3173907<br>3173908<br>3173909<br>3173910<br>3173911<br>3173912<br>3173913<br>3173914<br>3173915<br>3173916<br>3173917<br>3173918<br>3173919<br>3173920<br>3173921<br>3173922<br>3173923<br>3173924<br>3173925<br>3173926<br>3173927<br>3173928<br>3173929	core<br>core<br>core<br>core<br>core<br>core<br>core<br>extended<br>core<br>extended<br>extended<br>core<br>core<br>core<br>core<br>extended<br>core<br>extended<br>extended<br>core<br>core<br>core<br>core<br>extended<br>extended<br>extended<br>extended<br>core<br>core<br>core						
3173974	535884<br>535885<br>535886<br>535887<br>535888<br>535889<br>535890<br>535891<br>535892<br>535893<br>535894<br>535895<br>535896<br>535897<br>535898<br>535899	3173975<br>3173976<br>3173977<br>3173978<br>3173979<br>3173980<br>3173981<br>3173982<br>3173983<br>3173984<br>3173985<br>3173986<br>3173987<br>3173988<br>3173989<br>3173990<br>3173991<br>3173992<br>3173993<br>3173994<br>3173995<br>3173996<br>3173997<br>3173998	full<br>full<br>extended<br>extended<br>extended<br>extended<br>full<br>core<br>core<br>full<br>extended<br>extended<br>core<br>core<br>extended<br>core<br>extended<br>extended<br>core<br>core<br>full<br>full<br>core<br>core	9	71129196	71197188	+	NM_004816<br>BX641153<br>ENSESTT00000027844<br>ENSESTT00000027845<br>ENSESTT00000027846<br>ENSESTT00000027847<br>ENSESTT00000027848<br>ENST00000257515<br>ENST00000377225<br>ENST00000377218<br>ENST00000377216<br>ENST00000303068<br>GENSCAN00000000819	Homo sapiens chromosome 9 open reading frame 61 (C9orf61), mRNA.<br>Homo sapiens mRNA; cDNA DKFZp686J24109 (from clone DKFZp686J24109).<br><br><br><br><br><br>cdna:known-ccds chromosome:NCBI36:9:71134061:71197188:1 gene:ENSG00000135063 CCDS6629.1<br>cdna:known chromosome:NCBI36:9:71130168:71197190:1 gene:ENSG00000135063<br>cdna:known chromosome:NCBI36:9:71134061:71197188:1 gene:ENSG00000135063<br>cdna:known chromosome:NCBI36:9:71192657:71197191:1 gene:ENSG00000135063<br>cdna:novel chromosome:NCBI36:9:71176002:71197191:1 gene:ENSG00000135063<br>cdna:Genscan chromosome:NCBI36:9:71130046:71196540:1

		3173999<br>3174000<br>3174001<br>3174002<br>3174003<br>3174004<br>3174005<br>3174006<br>3174007<br>3174008<br>3174009<br>3174010<br>3174011	core<br>core<br>extended<br>extended<br>extended<br>extended<br>extended<br>extended<br>extended<br>core<br>core<br>core<br>core						
3174121	535962<br>535963<br>535964<br>535965<br>535966<br>535967<br>535968<br>535969<br>535970<br>535971<br>535972<br>535973<br>535974<br>535975<br>535976<br>535977<br>535978<br>535979<br>535980<br>535981<br>535982<br>535983<br>535984<br>535985<br>535986<br>535987<br>535988<br>535989<br>535990<br>535991<br>535992<br>535993<br>535994	3174122<br>3174123<br>3174124<br>3174125<br>3174126<br>3174127<br>3174128<br>3174129<br>3174130<br>3174131<br>3174132<br>3174133<br>3174134<br>3174135<br>3174136<br>3174137<br>3174138<br>3174139<br>3174140<br>3174141<br>3174142<br>3174143<br>3174144<br>3174145<br>3174146<br>3174147<br>3174148<br>3174149<br>3174150<br>3174151<br>3174152<br>3174153<br>3174154<br>3174155<br>3174156<br>3174157<br>3174158<br>3174159<br>3174160<br>3174161<br>3174162	core<br>core<br>full<br>extended<br>full<br>core<br>full<br>full<br>full<br>full<br>full<br>full<br>full<br>full<br>core<br>core<br>core<br>full<br>core<br>full<br>core<br>core<br>core<br>core<br>full<br>full<br>full<br>full<br>extended<br>core<br>core<br>extended<br>full<br>full<br>full<br>full<br>full<br>extended<br>extended<br>extended<br>extended	9	71848327	72033249	+	NM_000986<br>NM_153267<br>BX538309<br>ENSESTT00000015668<br>ENSESTT00000015669<br>ENSESTT00000054563<br>ENSESTT00000054564<br>ENST00000377182<br>ENST00000326586<br>ENST00000324072<br>ENST00000265264<br>GENSCAN00000035642<br>GENSCAN00000035643<br>GENSCAN00000030326<br>GENSCAN00000030325<br>GENSCAN00000043175	Homo sapiens ribosomal protein L24 (RPL24), mRNA.<br>Homo sapiens MAM domain containing 2 (MAMDC2), mRNA.<br>Homo sapiens mRNA; cDNA DKFZp686C09130 (from clone DKFZp686C09130).<br><br><br><br><br>cdna:known-ccds chromosome:NCBI36:9:71848317:72031706:1 gene:ENSG00000165072 CCDS6631.1<br>cdna:known chromosome:NCBI36:6:43032061:43032444:-1 gene:ENSG00000181524<br>cdna:known chromosome:NCBI36:9:71848340:72031708:1 gene:ENSG00000165072<br>cdna:known chromosome:NCBI36:3:102882626:102888253:-1 gene:ENSG00000114391<br>cdna:Genscan chromosome:NCBI36:9:72021889:72022227:1<br>cdna:Genscan chromosome:NCBI36:9:72023186:72030768:1<br>cdna:Genscan chromosome:NCBI36:9:71880693:71976979:1<br>cdna:Genscan chromosome:NCBI36:9:71983618:71983671:1<br>cdna:Genscan chromosome:NCBI36:3:102852700:102888210:-1

		3174163<br>3174164<br>3174165<br>3174166<br>3174167<br>3174168<br>3174169	extended<br>core<br>core<br>core<br>core<br>extended<br>full						
3175274	536614<br>536615<br>536616<br>536617<br>536618<br>536619<br>536620<br>536621<br>536622<br>536623<br>536624<br>536625<br>536626<br>536627<br>536628<br>536629<br>536630<br>536631<br>536632<br>536633<br>536634<br>536635<br>536636<br>536637<br>536638<br>536639<br>536640<br>536641<br>536642<br>536643<br>536644<br>536645<br>536646<br>536647<br>536648<br>536649<br>536650<br>536651<br>536652<br>536653<br>536654<br>536655<br>536656<br>536657<br>536658<br>536659<br>536660	3175275<br>3175276<br>3175277<br>3175278<br>3175279<br>3175280<br>3175281<br>3175282<br>3175283<br>3175284<br>3175285<br>3175286<br>3175287<br>3175288<br>3175289<br>3175290<br>3175291<br>3175292<br>3175293<br>3175294<br>3175295<br>3175296<br>3175297<br>3175298<br>3175299<br>3175300<br>3175301<br>3175302<br>3175303<br>3175304<br>3175305<br>3175306<br>3175307<br>3175308<br>3175309<br>3175310<br>3175311<br>3175312<br>3175313<br>3175314<br>3175315<br>3175316<br>3175317<br>3175318<br>3175319<br>3175320<br>3175321	core<br>core<br>core<br>full<br>full<br>full<br>full<br>full<br>full<br>core<br>core<br>full<br>full<br>full<br>full<br>full<br>full<br>full<br>core<br>core<br>core<br>core<br>full<br>full<br>full<br>core<br>full<br>core<br>extended<br>extended<br>extended<br>extended<br>full<br>full<br>core<br>core<br>full<br>core<br>full<br>full<br>core<br>full<br>core<br>full<br>full<br>core<br>core	9	77695416	78164623	+	NM_006200<br>AK023096<br>AK056202<br>AK122718<br>ENSESTT00000029064<br>ENSESTT00000029065<br>ENSESTT00000029066<br>ENSESTT00000029067<br>ENSESTT00000029068<br>ENSESTT00000029069<br>ENSESTT00000029070<br>ENSESTT00000029071<br>ENST00000376752<br>ENST00000376767<br>ENST00000376754<br>ENST00000265283<br>GENSCAN00000037283<br>GENSCAN00000037286<br>GENSCAN00000037288<br>GENSCAN00000052562<br>GENSCAN00000045937<br>GENSCAN00000034731	Homo sapiens proprotein convertase subtilisin/kexin type 5 (PCSK5), mRNA.<br>Homo sapiens cDNA FLJ13034 fis, clone NT2RP3001232.<br>Homo sapiens cDNA FLJ31640 fis, clone NT2RI2003678, highly similar to SERINE PROTEASE PC6 PRECURSOR (EC 3.4.21.-).<br>Homo sapiens cDNA FLJ16215 fis, clone CTONG2025610, moderately similar to PC6B.<br>cdna:known-ccds chromosome:NCBI36:9:77695441:78000625:1 gene:ENSG00000099139 CCDS6652.1<br>cdna:known chromosome:NCBI36:9:77695406:77980298:1 gene:ENSG00000099139<br>cdna:known chromosome:NCBI36:9:77695441:78163658:1 gene:ENSG00000099139<br>cdna:known chromosome:NCBI36:9:77695441:77998143:1 gene:ENSG00000099139<br>cdna:Genscan chromosome:NCBI36:9:77872693:77877109:1<br>cdna:Genscan chromosome:NCBI36:9:77889798:77912091:1<br>cdna:Genscan chromosome:NCBI36:9:77959148:78002915:1<br>cdna:Genscan chromosome:NCBI36:9:77695918:77831777:1<br>cdna:Genscan chromosome:NCBI36:9:78126158:78163658:1<br>cdna:Genscan chromosome:NCBI36:9:78097261:78102915:1

	536661<br>536662<br>536663<br>536664<br>536665<br>536666<br>536667<br>536668<br>536669<br>536670<br>536671<br>536672<br>536673<br>536674<br>536675<br>536676<br>536677<br>536678<br>536679<br>536680<br>536681<br>536682<br>536683<br>536684<br>536685<br>536686<br>536687<br>536688<br>536689	3175322<br>3175323<br>3175324<br>3175325<br>3175326<br>3175327<br>3175328<br>3175329<br>3175330<br>3175331<br>3175332<br>3175333<br>3175334<br>3175335<br>3175336<br>3175337<br>3175338<br>3175339<br>3175340<br>3175341<br>3175342<br>3175343<br>3175344<br>3175345<br>3175346<br>3175347<br>3175348<br>3175349<br>3175350<br>3175351<br>3175352<br>3175353<br>3175354<br>3175355<br>3175356<br>3175357<br>3175358<br>3175359<br>3175360<br>3175361<br>3175362<br>3175363<br>3175364<br>3175365<br>3175366<br>3175367<br>3175368<br>3175369<br>3175370<br>3175371<br>3175372<br>3175373<br>3175374<br>3175375	core<br>extended<br>extended<br>core<br>extended<br>full<br>full<br>core<br>core<br>extended<br>core<br>extended<br>core<br>core<br>extended<br>core<br>core<br>extended<br>extended<br>extended<br>extended<br>full<br>full<br>extended<br>extended<br>extended<br>full<br>extended<br>full<br>extended<br>extended<br>full<br>full<br>full<br>extended<br>full<br>full<br>extended<br>extended<br>extended<br>full<br>full<br>full<br>extended<br>extended<br>extended<br>full<br>extended<br>extended<br>extended<br>extended<br>extended<br>full<br>full						

		3175376<br>3175377<br>3175378<br>3175379<br>3175380<br>3175381<br>3175382<br>3175383<br>3175384	extended<br>extended<br>extended<br>extended<br>full<br>extended<br>extended<br>extended<br>extended						
3193482	547610<br>547611<br>547612<br>547613<br>547614<br>547615<br>547616<br>547617<br>547618<br>547619<br>547620<br>547621<br>547622<br>547623<br>547624<br>547625<br>547626<br>547627<br>547628<br>547629<br>547630<br>547631<br>547632<br>547633<br>547634<br>547635<br>547636<br>547637<br>547638<br>547639<br>547640<br>547641<br>547642<br>547643<br>547644<br>547645<br>547646<br>547647<br>547648<br>547649<br>547650<br>547651<br>547652<br>547653<br>547654	3193483<br>3193484<br>3193485<br>3193486<br>3193487<br>3193488<br>3193489<br>3193490<br>3193491<br>3193492<br>3193493<br>3193494<br>3193495<br>3193496<br>3193497<br>3193498<br>3193499<br>3193500<br>3193501<br>3193502<br>3193503<br>3193504<br>3193505<br>3193506<br>3193507<br>3193508<br>3193509<br>3193510<br>3193511<br>3193512<br>3193513<br>3193514<br>3193515<br>3193516<br>3193517<br>3193518<br>3193519<br>3193520<br>3193521<br>3193522<br>3193523<br>3193524<br>3193525<br>3193526<br>3193527	full<br>core<br>core<br>extended<br>full<br>extended<br>extended<br>full<br>extended<br>extended<br>full<br>full<br>full<br>full<br>core<br>core<br>extended<br>extended<br>full<br>full<br>full<br>full<br>core<br>core<br>full<br>full<br>full<br>extended<br>full<br>full<br>full<br>full<br>full<br>full<br>full<br>extended<br>core<br>core<br>full<br>extended<br>core<br>core<br>core<br>extended<br>core	9	136672245	136876507	+	NM_000093<br>AK021598<br>AK057231<br>AK127662<br>M76729<br>AF086191<br>ENSESTT00000052419<br>ENSESTT00000052420<br>ENSESTT00000052421<br>ENSESTT00000052422<br>ENSESTT00000052423<br>ENSESTT00000052424<br>ENSESTT00000052425<br>ENSESTT00000052426<br>ENSESTT00000052427<br>ENSESTT00000052428<br>ENSESTT00000052429<br>ENSESTT00000052430<br>ENSESTT00000052431<br>ENSESTT00000052432<br>ENSESTT00000052433<br>ENSESTT00000052434<br>ENSESTT00000052435<br>ENSESTT00000052436<br>ENST00000328009<br>ENST00000355306<br>ENST00000371820<br>ENST00000371817<br>ENST00000371815<br>ENST00000371814<br>GENSCAN00000000521<br>GENSCAN00000000519<br>GENSCAN00000066416	Homo sapiens collagen, type V, alpha 1 (COL5A1), mRNA.<br>Homo sapiens cDNA FLJ11536 fis, clone HEMBA1002712.<br>Homo sapiens cDNA FLJ32669 fis, clone TESTI1000125.<br>Homo sapiens cDNA FLJ45760 fis, clone MESTC1000147, highly similar to Collagen alpha 1(V) chain precursor.<br>Human pro-alpha-1 (V) collagen mRNA, complete cds.<br>Homo sapiens full length insert cDNA clone ZC33A09.<br><br><br><br><br><br><br><br><br><br><br><br><br><br><br><br><br><br><br>cdna:known-ccds chromosome:NCBI36:9:136673473:136874560:1 gene:ENSG00000130635 CCDS6982.1<br>cdna:known chromosome:NCBI36:9:136673473:136874560:1 gene:ENSG00000130635<br>cdna:known chromosome:NCBI36:9:136673473:136874560:1 gene:ENSG00000130635<br>cdna:known chromosome:NCBI36:9:136673626:136876507:1 gene:ENSG00000130635<br>cdna:known chromosome:NCBI36:9:136684852:136740894:1 gene:ENSG00000130635<br>cdna:known chromosome:NCBI36:9:136684852:136740894:1 gene:ENSG00000130635<br>cdna:Genscan chromosome:NCBI36:9:136673855:136676321:1<br>cdna:Genscan chromosome:NCBI36:9:136712147:136763794:1<br>cdna:Genscan chromosome:NCBI36:9:136810414:136873970:1

	547655	3193528	core						
	547656	3193529	full						
	547657	3193530	full						
	547658	3193531	full						
	547659	3193532	full						
	547660	3193533	core						
	547661	3193534	core						
	547662	3193535	core						
	547663	3193536	core						
	547664	3193537	full						
	547665	3193538	core						
	547666	3193539	core						
	547667	3193540	full						
	547668	3193541	core						
	547669	3193542	core						
	547670	3193543	core						
	547671	3193544	extended						
	547672	3193545	extended						
	547673	3193546	core						
	547674	3193547	core						
	547675	3193548	core						
	547676	3193549	core						
	547677	3193550	core						
	547678	3193551	extended						
	547679	3193552	core						
	547680	3193553	core						
	547681	3193554	extended						
	547682	3193555	extended						
	547683	3193556	full						
	547684	3193557	extended						
	547685	3193558	core						
	547686	3193559	core						
	547687	3193560	extended						
	547688	3193561	core						
	547689	3193562	full						
	547690	3193563	core						
	547691	3193564	core						
	547692	3193565	extended						
	547693	3193566	full						
	547694	3193567	core						
	547695	3193568	core						
	547696	3193569	core						
	547697	3193570	core						
	547698	3193571	extended						
	547699	3193572	core						
	547700	3193573	full						
	547701	3193574	extended						
	547702	3193575	extended						
	547703	3193576	extended						
	547704	3193577	extended						
	547705	3193578	extended						
	547706	3193579	extended						
	547707	3193580	extended						
	547708	3193581	core						

	547709<br>547710<br>547711<br>547712<br>547713<br>547714<br>547715<br>547716<br>547717<br>547718<br>547719<br>547720<br>547721<br>547722<br>547723<br>547724<br>547725<br>547726<br>547727	3193582<br>3193583<br>3193584<br>3193585<br>3193586<br>3193587<br>3193588<br>3193589<br>3193590<br>3193591<br>3193592<br>3193593<br>3193594<br>3193595<br>3193596<br>3193597<br>3193598<br>3193599<br>3193600<br>3193601<br>3193602<br>3193603<br>3193604<br>3193605<br>3193606<br>3193607<br>3193608<br>3193609<br>3193610<br>3193611<br>3193612<br>3193613<br>3193614<br>3193615<br>3193616<br>3193617<br>3193618<br>3193619<br>3193620<br>3193621<br>3193622<br>3193623<br>3193624<br>3193625<br>3193626<br>3193627<br>3193628<br>3193629<br>3193630	core<br>extended<br>core<br>core<br>core<br>core<br>extended<br>core<br>core<br>core<br>core<br>core<br>core<br>full<br>extended<br>core<br>extended<br>core<br>core<br>core<br>core<br>full<br>extended<br>core<br>core<br>core<br>core<br>full<br>core<br>core<br>full<br>core<br>extended<br>core<br>core<br>full<br>full<br>core<br>extended<br>extended<br>extended<br>extended<br>core<br>core<br>core<br>core<br>extended<br>extended<br>extended						
3194635	548347<br>548348<br>548349<br>548350<br>548351	3194636<br>3194637<br>3194638<br>3194639<br>3194640	extended<br>core<br>extended<br>core<br>extended	9	138810077	138855460	+	NM_024718<br>NM_001039374<br>AB075864<br>AK023107<br>AK090683	Homo sapiens chromosome 9 open reading frame 86 (C9orf86), mRNA.<br>Homo sapiens KIAA1984 (KIAA1984), mRNA.<br>Homo sapiens mRNA for KIAA1984 protein.<br>Homo sapiens cDNA FLJ13045 fis, clone NT2RP3001356.<br>Homo sapiens cDNA FLJ33364 fis, clone BRACE2005448.

	548352	3194641	core					AK092639	Homo sapiens cDNA FLJ35320 fis, clone PROST2012007.
	548353	3194642	core					AK094220	Homo sapiens cDNA FLJ36901 fis, clone BRACE2002206.
	548354	3194643	core					AK094382	Homo sapiens cDNA FLJ37063 fis, clone BRACE2014824, weakly similar to Gsp1p.
	548355	3194644	core					AK098086	Homo sapiens cDNA FLJ40767 fis, clone TRACH2003484.
	548356	3194645	core					AK128154	Homo sapiens cDNA FLJ46277 fis, clone TESTI4029731.
	548357	3194646	core					BC007542	Homo sapiens KIAA1984, mRNA (cDNA clone IMAGE:2958849), complete cds.
	548358	3194647	core					CR602099	full-length cDNA clone CS0DB004YA18 of Neuroblastoma Cot 10-normalized of Homo sapiens (human).
	548359	3194648	extended					AF318367	Homo sapiens pp8875 mRNA, complete cds.
	548360	3194649	core					ENSESTT00000056105	
	548361	3194650	extended					ENSESTT00000056106	
	548362	3194651	extended					ENSESTT00000056107	
	548363	3194652	extended					ENSESTT00000056108	
	548364	3194653	core					ENSESTT00000056109	
	548365	3194654	full					ENSESTT00000056110	
	548366	3194655	full					ENST00000322866	cdna:known-ccds chromosome:NCBI36:9:138813688:138822013:1 gene:ENSG00000196642 CCDS7008.1
	548367	3194656	full					ENST00000371684	cdna:known chromosome:NCBI36:9:138805628:138814832:1 gene:ENSG00000196642
	548368	3194657	full					ENST00000371682	cdna:known chromosome:NCBI36:9:138810623:138820677:1 gene:ENSG00000196642
	548369	3194658	full					ENST00000338005	cdna:known chromosome:NCBI36:9:138813496:138824308:1 gene:ENSG00000196642
	548370	3194659	full					ENST00000371680	cdna:known chromosome:NCBI36:9:138814128:138820677:1 gene:ENSG00000196642
	548371	3194660	full					ENST00000371679	cdna:known chromosome:NCBI36:9:138814698:138820669:1 gene:ENSG00000196642
	548372	3194661	extended					ENST00000360483	cdna:known chromosome:NCBI36:9:138821120:138822185:1 gene:ENSG00000196642
	548373	3194662	core					ENST00000311502	cdna:known chromosome:NCBI36:9:138822195:138855460:1 gene:ENSG00000196642
	548374	3194663	core					ENST00000371675	cdna:known chromosome:NCBI36:9:138822195:138855460:1 gene:ENSG00000196642
	548375	3194664	core					ENST00000371671	cdna:known chromosome:NCBI36:9:138822216:138848589:1 gene:ENSG00000196642
	548376	3194665	full					ENST00000371669	cdna:known chromosome:NCBI36:9:138822234:138855460:1 gene:ENSG00000196642
	548377	3194666	extended					ENST00000371670	cdna:known chromosome:NCBI36:9:138822234:138855460:1 gene:ENSG00000196642
	548378	3194667	core					ENST00000357466	cdna:known chromosome:NCBI36:9:138822432:138855460:1 gene:ENSG00000196642
	548379	3194668	core					ENST00000371663	cdna:known chromosome:NCBI36:9:138835602:138855460:1 gene:ENSG00000196642
	548380	3194669	core					ENST00000339398	cdna:known chromosome:NCBI36:9:138835602:138854486:1 gene:ENSG00000196642
	548381	3194670	core					ENST00000371673	cdna:novel chromosome:NCBI36:9:138822195:138855241:1 gene:ENSG00000196642
	548382	3194671	core					ENST00000339128	cdna:novel chromosome:NCBI36:9:138858898:138861333:1 gene:ENSG00000187963
	548383	3194672	core					GENSCAN00000048615	cdna:Genscan chromosome:NCBI36:18:14987553:14996438:-1
	548384	3194673	full					GENSCAN00000039581	cdna:Genscan chromosome:NCBI36:2:132269688:132310026:-1
	548385	3194674	core					GENSCAN00000019392	cdna:Genscan chromosome:NCBI36:9:138789639:138821963:1
	548386	3194675	extended					GENSCAN00000019400	cdna:Genscan chromosome:NCBI36:9:138822470:138824150:1
	548387	3194676	extended					GENSCAN00000019401	cdna:Genscan chromosome:NCBI36:9:138835505:138854795:1
	548388	3194677	extended						
	548389	3194678	core						
	548390	3194679	core						
	548391	3194680	core						
	548392	3194681	core						
	548393	3194682	core						
	548394	3194683	core						
	548395	3194684	core						
	548396	3194685	extended						
	548397	3194686	extended						
	548398	3194687	extended						
		3194688	extended						
		3194689	core						
		3194690	core						
		3194691	extended						
		3194692	extended						
		3194693	core						
		3194694	core						

		3194695	core							
		3194696	core							
		3194697	extended							
		3194698	full							
		3194699	extended							
		3194700	extended							
		3194701	extended							
		3194702	extended							
		3194703	full							
		3194704	extended							
		3194705	extended							
		3194706	extended							
		3194707	extended							
		3194708	extended							
		3194709	core							
		3194710	extended							
		3194711	extended							
		3194712	core							
		3194713	extended							
		3194714	extended							
		3194715	extended							
		3194716	extended							
		3194717	extended							
		3194718	core							
		3194719	core							
		3194720	full							
		3194721	full							
		3194722	full							
		3194723	extended							
		3194724	extended							
		3194725	extended							
		3194726	extended							
		3194727	full							
		3194728	core							
		3194729	extended							
		3194730	core							
		3194731	extended							
		3194732	extended							
		3194733	extended							
		3194734	extended							
		3194735	extended							
		3194736	extended							
		3194737	extended							
		3194738	extended							
		3194739	extended							
		3194740	core							
		3194741	extended							
		3194742	extended							
		3194743	extended							
		3194744	extended							
		3194745	extended							
		3194746	extended							
		3194747	extended							
		3194748	extended							

		3194749<br>3194750<br>3194751<br>3194752<br>3194753<br>3194754<br>3194755<br>3194756<br>3194757<br>3194758<br>3194759<br>3194760<br>3194761<br>3194762<br>3194763<br>3194764<br>3194765<br>3194766<br>3194767<br>3194768<br>3194769<br>3194770<br>3194771<br>3194772<br>3194773<br>3194774<br>3194775	core<br>core<br>core<br>core<br>full<br>full<br>core<br>extended<br>core<br>core<br>core<br>core<br>core<br>core<br>core<br>core<br>extended<br>extended<br>extended<br>core<br>core<br>core<br>core<br>core<br>core<br>core<br>core						
3201319	552448<br>552449	3201320<br>3201321<br>3201322<br>3201323<br>3201324<br>3201325	core<br>core<br>core<br>core<br>core<br>core	9	21374254	21375396	-	NM_000605<br>AY255838<br>ENST00000380206<br>ENST00000315719<br>GENSCAN00000028810	Homo sapiens interferon, alpha 2 (IFNA2), mRNA.<br>Homo sapiens interferon alpha 2b mRNA, complete cds.<br>cdna:known-ccds chromosome:NCBI36:9:21374253:21375387:-1 gene:ENSG00000188379 CCDS6506.1<br>cdna:known chromosome:NCBI36:9:21374762:21375328:-1 gene:ENSG00000188379<br>cdna:Genscan chromosome:NCBI36:9:21374762:21375328:-1
3210013	557679<br>557680<br>557681<br>557682<br>557683<br>557684<br>557685<br>557686<br>557687<br>557688<br>557689<br>557690<br>557691<br>557692<br>557693<br>557694<br>557695<br>557696<br>557697<br>557698	3210014<br>3210015<br>3210016<br>3210017<br>3210018<br>3210019<br>3210020<br>3210021<br>3210022<br>3210023<br>3210024<br>3210025<br>3210026<br>3210027<br>3210028<br>3210029<br>3210030<br>3210031<br>3210032<br>3210033	core<br>core<br>core<br>core<br>full<br>core<br>core<br>core<br>full<br>core<br>core<br>core<br>core<br>full<br>full<br>core<br>extended<br>core<br>full<br>core	9	76527231	76742759	-	NM_017662<br>AK026281<br>AK057748<br>AK126029<br>AK225207<br>CR618713<br>ENSESTT00000025172<br>ENSESTT00000025173<br>ENSESTT00000025174<br>ENSESTT00000038457<br>ENSESTT00000038458<br>ENSESTT00000038459<br>ENST00000360774<br>ENST00000376872<br>ENST00000376871<br>ENST00000376870<br>ENST00000376864<br>ENST00000312449<br>ENST00000359047<br>ENST00000361255	Homo sapiens transient receptor potential cation channel, subfamily M, member 6 (TRPM6), mRNA.<br>Homo sapiens cDNA: FLJ22628 fis, clone HSI06177.<br>Homo sapiens cDNA FLJ25019 fis, clone CBL01642.<br>Homo sapiens cDNA FLJ44041 fis, clone TESTI4029370.<br>Homo sapiens mRNA for transient receptor potential cation channel, subfamily M, member 6 variant, clone: COL03793.<br>full-length cDNA clone CS0DF002YD08 of Fetal brain of Homo sapiens (human).<br><br><br><br><br><br><br>cdna:known-ccds chromosome:NCBI36:9:76527231:76692830:-1 gene:ENSG00000119121 CCDS6647.1<br>cdna:known chromosome:NCBI36:9:76527231:76692830:-1 gene:ENSG00000119121<br>cdna:known chromosome:NCBI36:9:76527231:76692830:-1 gene:ENSG00000119121<br>cdna:known chromosome:NCBI36:9:76527231:76692830:-1 gene:ENSG00000119121<br>cdna:known chromosome:NCBI36:9:76528958:76692655:-1 gene:ENSG00000119121<br>cdna:known chromosome:NCBI36:9:76566702:76627223:-1 gene:ENSG00000119121<br>cdna:known chromosome:NCBI36:9:76631493:76692830:-1 gene:ENSG00000119121<br>cdna:novel chromosome:NCBI36:9:76527231:76692830:-1 gene:ENSG00000119121

	557699	3210034	extended					GENSCAN00000025761	cdna:Genscan chromosome:NCBI36:9:76540582:76692698:-1
	557700	3210035	core						
	557701	3210036	core						
	557702	3210037	core						
	557703	3210038	core						
	557704	3210039	core						
	557705	3210040	core						
	557706	3210041	core						
	557707	3210042	core						
	557708	3210043	core						
	557709	3210044	core						
	557710	3210045	extended						
	557711	3210046	full						
	557712	3210047	full						
	557713	3210048	full						
	557714	3210049	full						
	557715	3210050	core						
	557716	3210051	extended						
	557717	3210052	core						
	557718	3210053	core						
	557719	3210054	full						
	557720	3210055	core						
	557721	3210056	full						
	557722	3210057	full						
	557723	3210058	core						
	557724	3210059	core						
	557725	3210060	core						
	557726	3210061	full						
	557727	3210062	core						
	557728	3210063	full						
	557729	3210064	core						
	557730	3210065	core						
	557731	3210066	core						
	557732	3210067	extended						
	557733	3210068	full						
	557734	3210069	core						
	557735	3210070	core						
	557736	3210071	extended						
	557737	3210072	core						
	557738	3210073	core						
	557739	3210074	core						
	557740	3210075	core						
	557741	3210076	core						
	557742	3210077	extended						
	557743	3210078	core						
	557744	3210079	core						
	557745	3210080	extended						
	557746	3210081	full						
	557747	3210082	extended						
	557748	3210083	extended						
	557749	3210084	core						
	557750	3210085	core						
	557751	3210086	core						
		3210087	core						

		3210088<br>3210089<br>3210090<br>3210091<br>3210092<br>3210093<br>3210094<br>3210095<br>3210096<br>3210097<br>3210098<br>3210099<br>3210100<br>3210101<br>3210102<br>3210103<br>3210104<br>3210105<br>3210106<br>3210107	core<br>core<br>core<br>full<br>full<br>full<br>full<br>core<br>core<br>full<br>core<br>core<br>core<br>full<br>extended<br>full<br>extended<br>full<br>full<br>full						
3217194	562145<br>562146<br>562147<br>562148<br>562149<br>562150<br>562151<br>562152<br>562153<br>562154<br>562155<br>562156<br>562157<br>562158<br>562159<br>562160<br>562161<br>562162<br>562163	3217195<br>3217196<br>3217197<br>3217198<br>3217199<br>3217200<br>3217201<br>3217202<br>3217203<br>3217204<br>3217205<br>3217206<br>3217207<br>3217208<br>3217209<br>3217210<br>3217211<br>3217212<br>3217213<br>3217214<br>3217215<br>3217216<br>3217217<br>3217218<br>3217219<br>3217220<br>3217221<br>3217222<br>3217223<br>3217224<br>3217225<br>3217226<br>3217227<br>3217228	core<br>core<br>core<br>core<br>core<br>core<br>full<br>core<br>full<br>extended<br>core<br>full<br>core<br>core<br>core<br>core<br>extended<br>core<br>core<br>core<br>core<br>core<br>core<br>core<br>core<br>core<br>full<br>full<br>extended<br>core<br>full<br>full<br>full<br>core	9	100001121	100058505	-	NM_018421<br>AB209721<br>AK124772<br>AL353935<br>ENSESTT00000027174<br>ENST00000375066<br>ENST00000342112<br>ENST00000375064<br>ENST00000375063<br>ENST00000336475<br>GENSCAN00000020928	Homo sapiens TBC1 domain family, member 2 (TBC1D2), mRNA.<br>Homo sapiens mRNA for Hypothetical protein pp8997 variant protein.<br>Homo sapiens cDNA FLJ42782 fis, clone BRAWH3005912.<br>Homo sapiens mRNA; cDNA DKFZp761D1823 (from clone DKFZp761D1823).<br><br>cdna:known chromosome:NCBI36:9:100001132:100057736:-1 gene:ENSG00000095383<br>cdna:known chromosome:NCBI36:9:100001132:100057693:-1 gene:ENSG00000095383<br>cdna:known chromosome:NCBI36:9:100001132:100057683:-1 gene:ENSG00000095383<br>cdna:known chromosome:NCBI36:9:100001132:100015653:-1 gene:ENSG00000095383<br>cdna:known chromosome:NCBI36:9:100001142:100057736:-1 gene:ENSG00000095383<br>cdna:Genscan chromosome:NCBI36:9:99926877:100023331:-1

		3217229<br>3217230<br>3217231<br>3217232<br>3217233<br>3217234<br>3217235	core<br>core<br>core<br>core<br>extended<br>extended<br>extended						
3219215	563383<br>563384<br>563385<br>563386<br>563387<br>563388<br>563389<br>563390<br>563391<br>563392<br>563393<br>563394<br>563395<br>563396<br>563397<br>563398<br>563399<br>563400<br>563401<br>563402<br>563403<br>563404<br>563405	3219216<br>3219217<br>3219218<br>3219219<br>3219220<br>3219221<br>3219222<br>3219223<br>3219224<br>3219225<br>3219226<br>3219227<br>3219228<br>3219229<br>3219230<br>3219231<br>3219232<br>3219233<br>3219234<br>3219235<br>3219236<br>3219237<br>3219238<br>3219239<br>3219240<br>3219241<br>3219242<br>3219243<br>3219244<br>3219245<br>3219246<br>3219247<br>3219248<br>3219249<br>3219250<br>3219251<br>3219252<br>3219253	full<br>full<br>full<br>full<br>full<br>full<br>full<br>extended<br>extended<br>core<br>core<br>core<br>core<br>core<br>core<br>extended<br>extended<br>core<br>core<br>core<br>extended<br>extended<br>full<br>full<br>core<br>full<br>core<br>full<br>full<br>full<br>full<br>full<br>full<br>full<br>full<br>full<br>full<br>full	9	109245450	109344165	-	NM_004235<br>AK095134<br>AK095894<br>BC030811<br>U70663<br>ENSESTT00000055005<br>ENSESTT00000055006<br>ENST00000374672<br>ENST00000374671<br>ENST00000374670<br>ENST00000316479<br>ENST00000259349<br>GENSCAN00000038643<br>GENSCAN00000063409	Homo sapiens Kruppel-like factor 4 (gut) (KLF4), mRNA.<br>Homo sapiens cDNA FLJ37815 fis, clone BRSSN2003012, highly similar to KRUPPEL-LIKE FACTOR 4.<br>Homo sapiens cDNA FLJ38575 fis, clone HCHON2007046.<br>Homo sapiens Kruppel-like factor 4 (gut), mRNA (cDNA clone MGC:22411 IMAGE:4703002), complete cds.<br>Human zinc finger transcription factor hEZF (EZF) mRNA, complete cds.<br><br><br>cdna:known-ccds chromosome:NCBI36:9:109286954:109291748:-1 gene:ENSG00000136826 CCDS6770.1<br>cdna:known chromosome:NCBI36:9:109286954:109291539:-1 gene:ENSG00000136826<br>cdna:known chromosome:NCBI36:9:109286954:109290549:-1 gene:ENSG00000136826<br>cdna:known chromosome:NCBI36:9:109286956:109291576:-1 gene:ENSG00000136826<br>cdna:known chromosome:NCBI36:9:109286956:109291576:-1 gene:ENSG00000136826<br>cdna:Genscan chromosome:NCBI36:9:109245450:109287433:-1<br>cdna:Genscan chromosome:NCBI36:9:109287853:109294341:-1
3222170	565212<br>565213<br>565214<br>565215<br>565216<br>565217<br>565218<br>565219	3222171<br>3222172<br>3222173<br>3222174<br>3222175<br>3222176<br>3222177<br>3222178	extended<br>extended<br>core<br>core<br>core<br>core<br>core<br>extended	9	116821678	117053473	-	NM_002160<br>AK024586<br>BX641111<br>U88892<br>M96686<br>ENSESTT00000038161<br>ENSESTT00000038162<br>ENSESTT00000038163	Homo sapiens tenascin C (hexabrachion) (TNC), mRNA.<br>Homo sapiens cDNA: FLJ20933 fis, clone ADSE01388.<br>Homo sapiens mRNA; cDNA DKFZp686B15117 (from clone DKFZp686B15117).<br>Human tenascin-C mRNA, splice variant TNCfn-ad2, partial cds.<br>Human tenascin mRNA, partial CDS, AD1 type III repeat region.

	565220	3222179	extended					ENSESTT00000038167		
	565221	3222180	extended					ENSESTT00000038174		
	565222	3222181	core					ENSESTT00000038178		
	565223	3222182	extended					ENSESTT00000038184		
	565224	3222183	core					ENSESTT00000038186		
	565225	3222184	core					ENST00000350763	cdna:known-ccds chromosome:NCBI36:9:116822634:116920260:-1 gene:ENSG00000041982 CCDS6811.1	
	565226	3222185	extended					ENST00000341037	cdna:known chromosome:NCBI36:9:116822634:116920260:-1 gene:ENSG00000041982	
	565227	3222186	core					ENST00000340094	cdna:known chromosome:NCBI36:9:116822634:116920260:-1 gene:ENSG00000041982	
	565228	3222187	core					ENST00000346706	cdna:known chromosome:NCBI36:9:116822634:116920260:-1 gene:ENSG00000041982	
	565229	3222188	core					ENST00000345230	cdna:known chromosome:NCBI36:9:116822634:116920260:-1 gene:ENSG00000041982	
	565230	3222189	extended					GENSCAN00000048533	cdna:Genscan chromosome:NCBI36:9:116823257:116893118:-1	
	565231	3222190	core					GENSCAN00000048529	cdna:Genscan chromosome:NCBI36:9:116914754:116928461:-1	
	565232	3222191	full					GENSCAN00000067967	cdna:Genscan chromosome:NCBI36:9:117048467:117053473:-1	
	565233	3222192	core							
	565234	3222193	core							
	565235	3222194	core							
	565236	3222195	core							
	565237	3222196	extended							
	565238	3222197	extended							
	565239	3222198	core							
	565240	3222199	extended							
	565241	3222200	extended							
	565242	3222201	extended							
	565243	3222202	extended							
	565244	3222203	extended							
	565245	3222204	extended							
	565246	3222205	core							
	565247	3222206	extended							
	565248	3222207	full							
	565249	3222208	full							
	565250	3222209	full							
	565251	3222210	core							
	565252	3222211	extended							
	565253	3222212	core							
	565254	3222213	extended							
	565255	3222214	core							
	565256	3222215	core							
	565257	3222216	core							
	565258	3222217	extended							
	565259	3222218	extended							
	565260	3222219	core							
	565261	3222220	core							
	565262	3222221	core							
	565263	3222222	core							
	565264	3222223	extended							
	565265	3222224	extended							
	565266	3222225	core							
	565267	3222226	core							
	565268	3222227	core							
	565269	3222228	core							
	565270	3222229	core							
	565271	3222230	core							
	565272	3222231	core							
	565273	3222232	extended							

	565274<br>565275<br>565276<br>565277<br>565278<br>565279<br>565280<br>565281<br>565282<br>565283<br>565284<br>565285<br>565286<br>565287	3222233<br>3222234<br>3222235<br>3222236<br>3222237<br>3222238<br>3222239<br>3222240<br>3222241<br>3222242<br>3222243<br>3222244<br>3222245<br>3222246<br>3222247<br>3222248<br>3222249<br>3222250<br>3222251<br>3222252<br>3222253<br>3222254<br>3222255<br>3222256<br>3222257<br>3222258<br>3222259<br>3222260<br>3222261<br>3222262<br>3222263<br>3222264<br>3222265<br>3222266<br>3222267<br>3222268<br>3222269<br>3222270<br>3222271<br>3222272<br>3222273<br>3222274	extended<br>core<br>core<br>extended<br>extended<br>extended<br>full<br>extended<br>extended<br>full<br>full<br>full<br>full<br>full<br>full<br>full<br>full<br>full<br>full<br>full<br>core<br>core<br>full<br>full<br>full<br>full<br>full<br>full<br>full<br>full<br>full<br>full<br>full<br>full<br>full<br>full<br>full<br>full<br>full<br>full<br>extended<br>full						
3226883	568219<br>568220<br>568221<br>568222<br>568223<br>568224<br>568225<br>568226<br>568227<br>568228<br>568229	3226884<br>3226885<br>3226886<br>3226887<br>3226888<br>3226889<br>3226890<br>3226891<br>3226892<br>3226893<br>3226894<br>3226895	extended<br>core<br>core<br>core<br>core<br>extended<br>extended<br>extended<br>extended<br>extended<br>core<br>core	9	130977655	130980638	-	NM_203434<br>CR620204<br>BC013070<br>ENST00000372491<br>ENST00000339671<br>GENSCAN00000021404	Homo sapiens immediate early response 5-like (IER5L), mRNA.<br>full-length cDNA clone CS0DK008YH23 of HeLa cells Cot 25-normalized of Homo sapiens (human).<br>Homo sapiens immediate early response 5-like, mRNA (cDNA clone IMAGE:4149891), with apparent retained intron.<br>cdna:known chromosome:NCBI36:9:130977652:130980362:-1 gene:ENSG00000188483<br>cdna:known chromosome:NCBI36:9:130978938:130980152:-1 gene:ENSG00000188483<br>cdna:Genscan chromosome:NCBI36:9:130978938:130980152:-1

		3226896<br>3226897<br>3226898<br>3226899<br>3226900<br>3226901<br>3226902	core<br>core<br>core<br>core<br>core<br>core<br>full						
3230760	570545<br>570546<br>570547<br>570548<br>570549<br>570550<br>570551<br>570552<br>570553<br>570554	3230761<br>3230762<br>3230763<br>3230764<br>3230765<br>3230766<br>3230767<br>3230768<br>3230769<br>3230770<br>3230771<br>3230772<br>3230773<br>3230774<br>3230775<br>3230776<br>3230777<br>3230778<br>3230779<br>3230780	core<br>core<br>core<br>core<br>extended<br>core<br>core<br>core<br>core<br>core<br>core<br>full<br>full<br>full<br>full<br>core<br>core<br>core<br>core<br>core	9	139076406	139084849	-	NM_178448<br>ENSESTT00000056169<br>ENST00000359018<br>ENST00000334240<br>GENSCAN00000017028	Homo sapiens chromosome 9 open reading frame 140 (C9orf140), mRNA.<br><br>cdna:known-ccds chromosome:NCBI36:9:139076402:139084805:-1 gene:ENSG00000186193 CCDS7027.1<br>cdna:known chromosome:NCBI36:9:139076599:139084849:-1 gene:ENSG00000186193<br>cdna:Genscan chromosome:NCBI36:9:139078932:139084718:-1
3238962	575654<br>575655<br>575656<br>575657<br>575658<br>575659<br>575660<br>575661<br>575662<br>575663<br>575664<br>575665<br>575666<br>575667<br>575668<br>575669<br>575670<br>575671<br>575672<br>575673<br>575674<br>575675<br>575676<br>575677<br>575678<br>575679	3238963<br>3238964<br>3238965<br>3238966<br>3238967<br>3238968<br>3238969<br>3238970<br>3238971<br>3238972<br>3238973<br>3238974<br>3238975<br>3238976<br>3238977<br>3238978<br>3238979<br>3238980<br>3238981<br>3238982<br>3238983<br>3238984<br>3238985<br>3238986<br>3238987<br>3238988	extended<br>full<br>extended<br>full<br>full<br>full<br>full<br>extended<br>extended<br>extended<br>full<br>full<br>full<br>extended<br>extended<br>full<br>extended<br>full<br>full<br>extended<br>extended<br>extended<br>full<br>extended<br>extended<br>full	10	24023672	24901210	+	NM_019590<br>AB033043<br>AK021984<br>AK022045<br>AK024199<br>AK091142<br>AK096446<br>BC017424<br>BC018764<br>BX640796<br>AF119913<br>ENSESTT00000016146<br>ENSESTT00000016147<br>ENSESTT00000016148<br>ENSESTT00000016149<br>ENSESTT00000016150<br>ENSESTT00000016152<br>ENSESTT00000016154<br>ENSESTT00000016156<br>ENSESTT00000016158<br>ENSESTT00000016159<br>ENSESTT00000016161<br>ENSESTT00000016163<br>ENST00000376462<br>ENST00000376456<br>ENST00000376454	Homo sapiens KIAA1217 (KIAA1217), mRNA.<br>Homo sapiens mRNA for KIAA1217 protein, partial cds.<br>Homo sapiens cDNA FLJ11922 fis, clone HEMBB1000336.<br>Homo sapiens cDNA FLJ11983 fis, clone HEMBB1001337.<br>Homo sapiens cDNA FLJ14137 fis, clone MAMMA1002764.<br>Homo sapiens cDNA FLJ33823 fis, clone CTONG2003361, weakly similar to Rattus norvegicus SNIP-a mRNA.<br>Homo sapiens cDNA FLJ39127 fis, clone NTONG2007522, weakly similar to Rattus norvegicus SNIP-a mRNA.<br>Homo sapiens KIAA1217, mRNA (cDNA clone IMAGE:4693518), partial cds.<br>Homo sapiens KIAA1217, mRNA (cDNA clone IMAGE:3604428), complete cds.<br>Homo sapiens mRNA; cDNA DKFZp686E0469 (from clone DKFZp686E0469); complete cds.<br>Homo sapiens PRO3077 mRNA, complete cds.<br><br><br><br><br><br><br><br><br><br><br><br><br>cdna:known chromosome:NCBI36:10:24023681:24876778:1 gene:ENSG00000120549<br>cdna:known chromosome:NCBI36:10:24537726:24853705:1 gene:ENSG00000120549<br>cdna:known chromosome:NCBI36:10:24538099:24876777:1 gene:ENSG00000120549

	575680	3238989	full					ENST00000323059	cdna:known chromosome:NCBI36:10:24538099:24876777:1 gene:ENSG00000120549
	575681	3238990	full					ENST00000376451	cdna:known chromosome:NCBI36:10:24538099:24876609:1 gene:ENSG00000120549
	575682	3238991	full					ENST00000307544	cdna:known chromosome:NCBI36:10:24778372:24874203:1 gene:ENSG00000120549
	575683	3238992	extended					ENST00000376452	cdna:novel chromosome:NCBI36:10:24538099:24876777:1 gene:ENSG00000120549
	575684	3238993	extended					GENSCAN00000007300	cdna:Genscan chromosome:NCBI36:10:24371246:24434036:1
	575685	3238994	core					GENSCAN00000015083	cdna:Genscan chromosome:NCBI36:10:24799354:24819015:1
	575686	3238995	extended					GENSCAN00000015086	cdna:Genscan chromosome:NCBI36:10:24823451:24873411:1
	575687	3238996	extended					GENSCAN00000030209	cdna:Genscan chromosome:NCBI36:10:24028088:24041378:1
	575688	3238997	core					GENSCAN00000047691	cdna:Genscan chromosome:NCBI36:10:24678876:24718948:1
	575689	3238998	full					GENSCAN00000030329	cdna:Genscan chromosome:NCBI36:10:24202361:24248972:1
	575690	3238999	full					GENSCAN00000042748	cdna:Genscan chromosome:NCBI36:10:24584232:24588014:1
	575691	3239000	extended					GENSCAN00000042753	cdna:Genscan chromosome:NCBI36:10:24538129:24552590:1
	575692	3239001	extended						
	575693	3239002	extended						
	575694	3239003	extended						
	575695	3239004	extended						
	575696	3239005	extended						
	575697	3239006	full						
	575698	3239007	extended						
	575699	3239008	extended						
	575700	3239009	extended						
	575701	3239010	full						
	575702	3239011	extended						
	575703	3239012	extended						
	575704	3239013	extended						
	575705	3239014	extended						
	575706	3239015	extended						
	575707	3239016	extended						
	575708	3239017	extended						
	575709	3239018	full						
	575710	3239019	extended						
	575711	3239020	extended						
	575712	3239021	extended						
	575713	3239022	extended						
	575714	3239023	extended						
	575715	3239024	extended						
	575716	3239025	full						
	575717	3239026	full						
	575718	3239027	extended						
	575719	3239028	extended						
	575720	3239029	extended						
	575721	3239030	extended						
	575722	3239031	extended						
	575723	3239032	extended						
	575724	3239033	extended						
	575725	3239034	extended						
	575726	3239035	extended						
	575727	3239036	extended						
	575728	3239037	extended						
	575729	3239038	extended						
	575730	3239039	full						
	575731	3239040	full						
	575732	3239041	extended						
	575733	3239042	extended						

	575734	3239043	full						
	575735	3239044	full						
	575736	3239045	full						
	575737	3239046	full						
	575738	3239047	full						
	575739	3239048	full						
	575740	3239049	full						
	575741	3239050	extended						
	575742	3239051	extended						
	575743	3239052	full						
	575744	3239053	full						
	575745	3239054	core						
	575746	3239055	core						
	575747	3239056	full						
	575748	3239057	full						
	575749	3239058	extended						
	575750	3239059	extended						
	575751	3239060	extended						
	575752	3239061	full						
	575753	3239062	full						
	575754	3239063	extended						
	575755	3239064	full						
	575756	3239065	extended						
	575757	3239066	full						
	575758	3239067	full						
	575759	3239068	full						
	575760	3239069	extended						
	575761	3239070	extended						
	575762	3239071	extended						
	575763	3239072	extended						
	575764	3239073	full						
	575765	3239074	full						
	575766	3239075	core						
	575767	3239076	core						
	575768	3239077	full						
	575769	3239078	full						
	575770	3239079	extended						
	575771	3239080	extended						
	575772	3239081	extended						
	575773	3239082	extended						
	575774	3239083	extended						
	575775	3239084	extended						
	575776	3239085	extended						
	575777	3239086	extended						
	575778	3239087	extended						
	575779	3239088	extended						
	575780	3239089	core						
	575781	3239090	extended						
	575782	3239091	full						
	575783	3239092	full						
	575784	3239093	core						
	575785	3239094	core						
	575786	3239095	extended						
	575787	3239096	extended						

	575788<br>575789<br>575790<br>575791<br>575792<br>575793<br>575794<br>575795<br>575796<br>575797<br>575798<br>575799<br>575800<br>575801<br>575802<br>575803<br>575804<br>575805<br>575806<br>575807<br>575808<br>575809<br>575810<br>575811<br>575812<br>575813<br>575814<br>575815<br>575816<br>575817<br>575818<br>575819<br>575820<br>575821<br>575822<br>575823<br>575824<br>575825<br>575826<br>575827<br>575828<br>575829<br>575830<br>575831<br>575832<br>575833<br>575834<br>575835<br>575836<br>575837<br>575838<br>575839<br>575840<br>575841	3239097<br>3239098<br>3239099<br>3239100<br>3239101<br>3239102<br>3239103<br>3239104<br>3239105<br>3239106<br>3239107<br>3239108<br>3239109<br>3239110<br>3239111<br>3239112<br>3239113<br>3239114<br>3239115<br>3239116<br>3239117<br>3239118<br>3239119<br>3239120<br>3239121<br>3239122<br>3239123<br>3239124<br>3239125<br>3239126<br>3239127<br>3239128<br>3239129<br>3239130<br>3239131<br>3239132<br>3239133<br>3239134<br>3239135<br>3239136<br>3239137<br>3239138<br>3239139<br>3239140<br>3239141<br>3239142<br>3239143<br>3239144<br>3239145<br>3239146<br>3239147<br>3239148<br>3239149<br>3239150	extended<br>extended<br>extended<br>full<br>full<br>full<br>extended<br>extended<br>full<br>full<br>full<br>extended<br>core<br>core<br>extended<br>core<br>extended<br>extended<br>extended<br>core<br>full<br>full<br>extended<br>extended<br>extended<br>extended<br>extended<br>extended<br>extended<br>extended<br>extended<br>extended<br>core<br>core<br>extended<br>full<br>extended<br>extended<br>extended<br>core<br>extended<br>extended<br>extended<br>core<br>extended<br>core<br>core<br>extended<br>core<br>core<br>full<br>extended<br>core<br>core						

	575842<br>575843<br>575844<br>575845	3239151<br>3239152<br>3239153<br>3239154<br>3239155<br>3239156<br>3239157<br>3239158<br>3239159<br>3239160<br>3239161<br>3239162<br>3239163<br>3239164<br>3239165<br>3239166<br>3239167<br>3239168<br>3239169<br>3239170<br>3239171<br>3239172<br>3239173<br>3239174<br>3239175<br>3239176<br>3239177<br>3239178<br>3239179<br>3239180<br>3239181<br>3239182<br>3239183<br>3239184<br>3239185<br>3239186<br>3239187<br>3239188<br>3239189<br>3239190<br>3239191<br>3239192<br>3239193<br>3239194<br>3239195<br>3239196<br>3239197	extended<br>extended<br>full<br>extended<br>core<br>extended<br>extended<br>extended<br>extended<br>extended<br>extended<br>extended<br>extended<br>extended<br>extended<br>full<br>extended<br>core<br>core<br>core<br>core<br>core<br>core<br>core<br>core<br>extended<br>core<br>core<br>core<br>core<br>core<br>core<br>core<br>core<br>core<br>core<br>full<br>full<br>full<br>full<br>full<br>full<br>full<br>full<br>full<br>full<br>full						
3250237	582637<br>582638<br>582639<br>582640<br>582641<br>582642<br>582643	3250238<br>3250239<br>3250240<br>3250241<br>3250242<br>3250243<br>3250244	core<br>core<br>full<br>full<br>core<br>full<br>core	10	70650065	70697308	+	NM_025130<br>BX538078<br>ENSESTT00000015259<br>ENSESTT00000015260<br>ENSESTT00000015261<br>ENSESTT00000040961<br>ENSESTT00000040962	Homo sapiens hexokinase domain containing 1 (HKDC1), mRNA.<br>Homo sapiens mRNA; cDNA DKFZp686I14218 (from clone DKFZp686I14218).

	582644<br>582645<br>582646<br>582647<br>582648<br>582649<br>582650<br>582651<br>582652<br>582653<br>582654<br>582655<br>582656<br>582657<br>582658<br>582659<br>582660<br>582661<br>582662<br>582663<br>582664<br>582665<br>582666<br>582667	3250245<br>3250246<br>3250247<br>3250248<br>3250249<br>3250250<br>3250251<br>3250252<br>3250253<br>3250254<br>3250255<br>3250256<br>3250257<br>3250258<br>3250259<br>3250260<br>3250261<br>3250262<br>3250263<br>3250264<br>3250265<br>3250266<br>3250267<br>3250268<br>3250269<br>3250270<br>3250271<br>3250272<br>3250273<br>3250274<br>3250275<br>3250276<br>3250277	core<br>core<br>core<br>core<br>extended<br>core<br>core<br>core<br>core<br>core<br>core<br>extended<br>core<br>core<br>extended<br>full<br>extended<br>core<br>core<br>core<br>core<br>core<br>full<br>full<br>core<br>extended<br>extended<br>extended<br>core<br>core<br>core<br>core<br>core					ENST00000354624<br>ENST00000298648<br>GENSCAN00000017357	cdna:known-ccds chromosome:NCBI36:10:70650065:70697321:1 gene:ENSG00000156510 CCDS7288.1<br>cdna:known chromosome:NCBI36:10:70650065:70697308:1 gene:ENSG00000156510<br>cdna:Genscan chromosome:NCBI36:10:70650198:70775959:1
3252036	583810<br>583811<br>583812<br>583813<br>583814<br>583815<br>583816<br>583817<br>583818<br>583819<br>583820<br>583821<br>583822<br>583823<br>583824<br>583825<br>583826<br>583827<br>583828	3252037<br>3252038<br>3252039<br>3252040<br>3252041<br>3252042<br>3252043<br>3252044<br>3252045<br>3252046<br>3252047<br>3252048<br>3252049<br>3252050<br>3252051<br>3252052<br>3252053<br>3252054<br>3252055<br>3252056<br>3252057	full<br>full<br>extended<br>extended<br>full<br>core<br>core<br>full<br>core<br>full<br>core<br>extended<br>core<br>core<br>core<br>core<br>core<br>core<br>full<br>core<br>extended	10	75315930	75372084	+	NM_002658<br>ENSESTT00000058209<br>ENSESTT00000058210<br>ENSESTT00000058211<br>ENSESTT00000058212<br>ENST00000372764<br>ENST00000242464<br>ENST00000372762<br>ENST00000372761<br>GENSCAN00000010635	Homo sapiens plasminogen activator, urokinase (PLAU), mRNA.<br><br><br><br><br>cdna:known-ccds chromosome:NCBI36:10:75340896:75347260:1 gene:ENSG00000122861 CCDS7339.1<br>cdna:known chromosome:NCBI36:10:75340921:75347261:1 gene:ENSG00000122861<br>cdna:known chromosome:NCBI36:10:75340921:75347258:1 gene:ENSG00000122861<br>cdna:known chromosome:NCBI36:10:75341320:75346329:1 gene:ENSG00000122861<br>cdna:Genscan chromosome:NCBI36:10:75341320:75346329:1

		3252058<br>3252059<br>3252060<br>3252061<br>3252062<br>3252063<br>3252064<br>3252065<br>3252066	core<br>extended<br>extended<br>core<br>core<br>core<br>core<br>extended<br>full						
3252071	583831<br>583832<br>583833<br>583834<br>583835<br>583836<br>583837<br>583838<br>583839<br>583840<br>583841<br>583842<br>583843<br>583844<br>583845<br>583846<br>583847<br>583848<br>583849<br>583850<br>583851<br>583852<br>583853<br>583854<br>583855<br>583856<br>583857<br>583858<br>583859<br>583860<br>583861<br>583862<br>583863<br>583864<br>583865<br>583866<br>583867<br>583868<br>583869<br>583870<br>583871<br>583872<br>583873<br>583874<br>583875	3252072<br>3252073<br>3252074<br>3252075<br>3252076<br>3252077<br>3252078<br>3252079<br>3252080<br>3252081<br>3252082<br>3252083<br>3252084<br>3252085<br>3252086<br>3252087<br>3252088<br>3252089<br>3252090<br>3252091<br>3252092<br>3252093<br>3252094<br>3252095<br>3252096<br>3252097<br>3252098<br>3252099<br>3252100<br>3252101<br>3252102<br>3252103<br>3252104<br>3252105<br>3252106<br>3252107<br>3252108<br>3252109<br>3252110<br>3252111<br>3252112<br>3252113<br>3252114<br>3252115<br>3252116	core<br>core<br>extended<br>core<br>full<br>extended<br>extended<br>extended<br>full<br>core<br>extended<br>extended<br>extended<br>full<br>core<br>extended<br>extended<br>extended<br>extended<br>extended<br>extended<br>full<br>core<br>core<br>core<br>core<br>core<br>core<br>core<br>core<br>core<br>core<br>core<br>core<br>full<br>core<br>core<br>core<br>core<br>extended<br>core<br>core<br>core<br>core<br>core	10	75424967	75549911	+	NM_014000<br>NM_003373<br>AK126978<br>AL832130<br>BX537994<br>ENSESTT00000058213<br>ENSESTT00000058214<br>ENSESTT00000058215<br>ENSESTT00000058216<br>ENST00000372755<br>ENST00000211998<br>ENST00000277829<br>GENSCAN00000010634<br>GENSCAN00000010633<br>GENSCAN00000018228	Homo sapiens vinculin (VCL), transcript variant 1, mRNA.<br>Homo sapiens vinculin (VCL), transcript variant 2, mRNA.<br>Homo sapiens cDNA FLJ45031 fis, clone BRAWH3018548, highly similar to Vinculin.<br>Homo sapiens mRNA; cDNA DKFZp686H1210 (from clone DKFZp686H1210).<br>Homo sapiens mRNA; cDNA DKFZp686C1586 (from clone DKFZp686C1586); complete cds.<br><br><br><br><br>cdna:known-ccds chromosome:NCBI36:10:75427878:75549924:1 gene:ENSG00000035403 CCDS7340.1<br>cdna:known-ccds chromosome:NCBI36:10:75427878:75549916:1 gene:ENSG00000035403 CCDS7341.1<br>cdna:known chromosome:NCBI36:10:75427878:75549916:1 gene:ENSG00000035403<br>cdna:Genscan chromosome:NCBI36:10:75427972:75429124:1<br>cdna:Genscan chromosome:NCBI36:10:75434390:75504667:1<br>cdna:Genscan chromosome:NCBI36:10:75512230:75547933:1

	583876	3252117<br>3252118<br>3252119<br>3252120<br>3252121<br>3252122<br>3252123<br>3252124<br>3252125<br>3252126<br>3252127<br>3252128<br>3252129<br>3252130<br>3252131<br>3252132<br>3252133<br>3252134<br>3252135<br>3252136<br>3252137<br>3252138<br>3252139<br>3252140<br>3252141	core<br>extended<br>core<br>full<br>core<br>core<br>core<br>extended<br>extended<br>extended<br>extended<br>core<br>core<br>extended<br>extended<br>extended<br>extended<br>core<br>core<br>extended<br>core<br>core<br>core<br>core<br>core						
3257338	587153<br>587154<br>587155<br>587156<br>587157<br>587158<br>587159<br>587160<br>587161<br>587162<br>587163<br>587164<br>587165<br>587166<br>587167<br>587168<br>587169<br>587170<br>587171<br>587172<br>587173<br>587174<br>587175<br>587176<br>587177<br>587178<br>587179<br>587180<br>587181	3257339<br>3257340<br>3257341<br>3257342<br>3257343<br>3257344<br>3257345<br>3257346<br>3257347<br>3257348<br>3257349<br>3257350<br>3257351<br>3257352<br>3257353<br>3257354<br>3257355<br>3257356<br>3257357<br>3257358<br>3257359<br>3257360<br>3257361<br>3257362<br>3257363<br>3257364<br>3257365<br>3257366<br>3257367	full<br>full<br>full<br>full<br>full<br>full<br>full<br>full<br>full<br>full<br>core<br>core<br>extended<br>core<br>core<br>core<br>core<br>core<br>core<br>core<br>core<br>core<br>core<br>core<br>extended<br>core<br>core<br>core<br>extended	10	91435740	91524670	+	NM_016195<br>AB033338<br>AY739715<br>AL137392<br>ENSESTT00000057898<br>ENSESTT00000057899<br>ENSESTT00000057900<br>ENSESTT00000057901<br>ENSESTT00000057902<br>ENST00000260753<br>ENST00000371737<br>ENST00000371736<br>ENST00000371728<br>ENST00000360148<br>GENSCAN00000057653<br>GENSCAN00000001469	Homo sapiens M-phase phosphoprotein 1 (MPHOSPH1), mRNA.<br>Homo sapiens KRMP1 mRNA for mitotic kinesin-related protein, partial cds, alternative exon sequence.<br>Homo sapiens M-phase phosphoprotein 1 (MPHOSPH1) mRNA, partial cds.<br>Homo sapiens mRNA; cDNA DKFZp434I152 (from clone DKFZp434I152).<br><br><br><br><br><br>cdna:known-ccds chromosome:NCBI36:10:91451347:91524680:1 gene:ENSG00000138182 CCDS7407.1<br>cdna:known chromosome:NCBI36:10:91451347:91524680:1 gene:ENSG00000138182<br>cdna:known chromosome:NCBI36:10:91451347:91524676:1 gene:ENSG00000138182<br>cdna:known chromosome:NCBI36:10:91451354:91524676:1 gene:ENSG00000138182<br>cdna:known chromosome:NCBI36:10:91451374:91524680:1 gene:ENSG00000138182<br>cdna:Genscan chromosome:NCBI36:10:91464692:91523785:1<br>cdna:Genscan chromosome:NCBI36:10:91434461:91459198:1

	587182<br>587183<br>587184<br>587185<br>587186<br>587187<br>587188<br>587189<br>587190<br>587191<br>587192	3257368<br>3257369<br>3257370<br>3257371<br>3257372<br>3257373<br>3257374<br>3257375<br>3257376<br>3257377<br>3257378<br>3257379<br>3257380<br>3257381<br>3257382<br>3257383<br>3257384<br>3257385<br>3257386<br>3257387<br>3257388<br>3257389<br>3257390<br>3257391<br>3257392<br>3257393<br>3257394<br>3257395<br>3257396<br>3257397<br>3257398<br>3257399<br>3257400<br>3257401<br>3257402<br>3257403<br>3257404	core<br>core<br>core<br>extended<br>extended<br>extended<br>core<br>core<br>core<br>core<br>core<br>core<br>extended<br>extended<br>extended<br>full<br>core<br>core<br>core<br>extended<br>core<br>extended<br>core<br>core<br>core<br>core<br>full<br>core<br>core<br>core<br>core<br>core<br>core<br>core<br>core<br>core<br>core						
3258477	587876<br>587877<br>587878<br>587879<br>587880<br>587881<br>587882<br>587883<br>587884<br>587885<br>587886<br>587887<br>587888<br>587889<br>587890<br>587891<br>587892	3258478<br>3258479<br>3258480<br>3258481<br>3258482<br>3258483<br>3258484<br>3258485<br>3258486<br>3258487<br>3258488<br>3258489<br>3258490<br>3258491<br>3258492<br>3258493<br>3258494	extended<br>full<br>extended<br>extended<br>extended<br>extended<br>extended<br>extended<br>extended<br>full<br>full<br>extended<br>full<br>extended<br>extended<br>extended<br>extended	10	95293987	96078702	+	NM_016341<br>AF170071<br>AK025366<br>AK074239<br>AL050031<br>BX641131<br>ENSESTT00000044114<br>ENSESTT00000044116<br>ENSESTT00000044117<br>ENSESTT00000044118<br>ENSESTT00000044119<br>ENSESTT00000044120<br>ENST00000260766<br>ENST00000371380<br>ENST00000371375<br>ENST00000285974<br>ENST00000371385	Homo sapiens phospholipase C, epsilon 1 (PLCE1), mRNA.<br>Homo sapiens phospholipase C epsilon mRNA, partial cds.<br>Homo sapiens cDNA: FLJ21713 fis, clone COL10239.<br>Homo sapiens cDNA FLJ23659 fis, clone COLF6818, highly similar to Homo sapiens phospholipase C epsilon mRNA.<br>Homo sapiens mRNA; cDNA DKFZp566E0224 (from clone DKFZp566E0224).<br>Homo sapiens mRNA; cDNA DKFZp686H2494 (from clone DKFZp686H2494).<br><br><br><br><br><br><br>cdna:known chromosome:NCBI36:10:95743736:96078136:1 gene:ENSG00000138193<br>cdna:known chromosome:NCBI36:10:95780559:96078136:1 gene:ENSG00000138193<br>cdna:known chromosome:NCBI36:10:95838842:96075356:1 gene:ENSG00000138193<br>cdna:known chromosome:NCBI36:10:95838842:96074827:1 gene:ENSG00000138193<br>cdna:novel chromosome:NCBI36:10:95743736:96078136:1 gene:ENSG00000138193

	587893	3258495	extended					GENSCAN00000007470	cdna:Genscan chromosome:NCBI36:10:95994054:96074827:1
	587894	3258496	core					GENSCAN00000009402	cdna:Genscan chromosome:NCBI36:10:95405507:95452547:1
	587895	3258497	core					GENSCAN00000062283	cdna:Genscan chromosome:NCBI36:10:95780794:95782011:1
	587896	3258498	full					GENSCAN00000049130	cdna:Genscan chromosome:NCBI36:10:95887632:95887910:1
	587897	3258499	extended					GENSCAN00000049131	cdna:Genscan chromosome:NCBI36:10:95893812:95985867:1
	587898	3258500	extended						
	587899	3258501	full						
	587900	3258502	full						
	587901	3258503	extended						
	587902	3258504	extended						
	587903	3258505	extended						
	587904	3258506	extended						
	587905	3258507	full						
	587906	3258508	full						
	587907	3258509	full						
	587908	3258510	full						
	587909	3258511	full						
	587910	3258512	extended						
	587911	3258513	full						
	587912	3258514	full						
	587913	3258515	core						
	587914	3258516	core						
	587915	3258517	extended						
	587916	3258518	full						
	587917	3258519	full						
	587918	3258520	full						
	587919	3258521	full						
	587920	3258522	full						
	587921	3258523	extended						
	587922	3258524	full						
	587923	3258525	full						
	587924	3258526	extended						
	587925	3258527	full						
	587926	3258528	full						
	587927	3258529	extended						
	587928	3258530	extended						
	587929	3258531	extended						
	587930	3258532	full						
	587931	3258533	core						
	587932	3258534	full						
	587933	3258535	full						
	587934	3258536	extended						
	587935	3258537	full						
	587936	3258538	full						
	587937	3258539	full						
	587938	3258540	full						
	587939	3258541	extended						
	587940	3258542	extended						
	587941	3258543	extended						
	587942	3258544	full						
	587943	3258545	full						
	587944	3258546	full						
	587945	3258547	full						
	587946	3258548	full						

	587947	3258549	extended						
	587948	3258550	full						
	587949	3258551	core						
	587950	3258552	full						
	587951	3258553	core						
	587952	3258554	core						
	587953	3258555	core						
	587954	3258556	full						
	587955	3258557	core						
	587956	3258558	extended						
	587957	3258559	full						
	587958	3258560	extended						
	587959	3258561	extended						
	587960	3258562	extended						
	587961	3258563	core						
	587962	3258564	extended						
	587963	3258565	core						
	587964	3258566	core						
	587965	3258567	full						
	587966	3258568	extended						
	587967	3258569	extended						
	587968	3258570	extended						
	587969	3258571	extended						
	587970	3258572	extended						
	587971	3258573	core						
	587972	3258574	core						
	587973	3258575	core						
	587974	3258576	core						
	587975	3258577	core						
	587976	3258578	core						
	587977	3258579	full						
	587978	3258580	core						
	587979	3258581	core						
	587980	3258582	core						
	587981	3258583	extended						
	587982	3258584	core						
	587983	3258585	full						
	587984	3258586	extended						
	587985	3258587	extended						
	587986	3258588	full						
		3258589	core						
		3258590	full						
		3258591	full						
		3258592	full						
		3258593	core						
		3258594	core						
		3258595	core						
		3258596	core						
		3258597	core						
		3258598	core						
		3258599	core						
		3258600	full						
		3258601	core						
		3258602	full						

		3258603<br>3258604<br>3258605<br>3258606<br>3258607<br>3258608<br>3258609<br>3258610<br>3258611<br>3258612<br>3258613<br>3258614<br>3258615<br>3258616<br>3258617	extended<br>core<br>extended<br>core<br>core<br>extended<br>extended<br>core<br>core<br>core<br>extended<br>extended<br>core<br>extended<br>extended						
3262535	590362<br>590363<br>590364<br>590365<br>590366<br>590367<br>590368<br>590369<br>590370<br>590371<br>590372<br>590373<br>590374<br>590375<br>590376<br>590377<br>590378<br>590379<br>590380<br>590381<br>590382<br>590383<br>590384<br>590385<br>590386<br>590387<br>590388	3262536<br>3262537<br>3262538<br>3262539<br>3262540<br>3262541<br>3262542<br>3262543<br>3262544<br>3262545<br>3262546<br>3262547<br>3262548<br>3262549<br>3262550<br>3262551<br>3262552<br>3262553<br>3262554<br>3262555<br>3262556<br>3262557<br>3262558<br>3262559<br>3262560<br>3262561<br>3262562<br>3262563<br>3262564<br>3262565<br>3262566<br>3262567<br>3262568<br>3262569<br>3262570<br>3262571<br>3262572<br>3262573<br>3262574	core<br>core<br>extended<br>extended<br>extended<br>extended<br>extended<br>extended<br>core<br>core<br>core<br>extended<br>core<br>core<br>core<br>core<br>extended<br>core<br>full<br>extended<br>extended<br>extended<br>full<br>extended<br>extended<br>extended<br>extended<br>extended<br>extended<br>extended<br>core<br>extended<br>extended<br>extended<br>full<br>full<br>extended<br>extended<br>core	10	106018568	106049159	+	NM_183239<br>AK094412<br>BC023583<br>BC046194<br>ENSESTT00000032767<br>ENSESTT00000032768<br>ENSESTT00000032769<br>ENSESTT00000032770<br>ENSESTT00000032771<br>ENST00000369708<br>ENST00000338595<br>ENST00000369707<br>GENSCAN00000027606	Homo sapiens glutathione S-transferase omega 2 (GSTO2), mRNA.<br>Homo sapiens cDNA FLJ37093 fis, clone BRACE2017985, weakly similar to GLUTATHIONE-S-TRANSFERASE.<br>Homo sapiens glutathione S-transferase omega 2, mRNA (cDNA clone IMAGE:4844422).<br>Homo sapiens glutathione S-transferase omega 2, mRNA (cDNA clone IMAGE:6018729), partial cds.<br><br><br><br><br><br>cdna:known-ccds chromosome:NCBI36:10:106018621:106049166:1 gene:ENSG00000065621 CCDS7556.1<br>cdna:known chromosome:NCBI36:10:106018621:106049165:1 gene:ENSG00000065621<br>cdna:known chromosome:NCBI36:10:106024910:106049166:1 gene:ENSG00000065621<br>cdna:Genscan chromosome:NCBI36:10:106024503:106041516:1

		3262575	core						
3265224	592137<br>592138<br>592139<br>592140<br>592141<br>592142<br>592143<br>592144<br>592145<br>592146<br>592147<br>592148<br>592149<br>592150<br>592151<br>592152<br>592153<br>592154<br>592155<br>592156<br>592157<br>592158<br>592159<br>592160<br>592161<br>592162<br>592163<br>592164<br>592165<br>592166<br>592167<br>592168	3265225<br>3265226<br>3265227<br>3265228<br>3265229<br>3265230<br>3265231<br>3265232<br>3265233<br>3265234<br>3265235<br>3265236<br>3265237<br>3265238<br>3265239<br>3265240<br>3265241<br>3265242<br>3265243<br>3265244<br>3265245<br>3265246<br>3265247<br>3265248<br>3265249<br>3265250<br>3265251<br>3265252<br>3265253<br>3265254<br>3265255<br>3265256<br>3265257<br>3265258<br>3265259<br>3265260<br>3265261<br>3265262<br>3265263<br>3265264<br>3265265<br>3265266<br>3265267<br>3265268<br>3265269<br>3265270<br>3265271<br>3265272<br>3265273<br>3265274<br>3265275<br>3265276	core<br>extended<br>full<br>full<br>full<br>full<br>full<br>core<br>full<br>full<br>full<br>full<br>core<br>core<br>full<br>full<br>full<br>core<br>full<br>full<br>full<br>full<br>core<br>core<br>core<br>full<br>full<br>core<br>full<br>core<br>full<br>full<br>full<br>full<br>core<br>full<br>core<br>full<br>full<br>core<br>full<br>core<br>core<br>core<br>extended<br>full<br>extended<br>extended<br>extended<br>extended<br>extended<br>extended	10	115989008	116043062	+	NM_198496<br>AK127756<br>AY572972<br>AJ536328<br>ENSESTT00000023062<br>ENST00000298715<br>ENST00000369277<br>ENST00000369276<br>ENST00000369275	Homo sapiens von Willebrand factor A domain containing 2 (VWA2), mRNA.<br>Homo sapiens cDNA FLJ45857 fis, clone OCBBF2030927, weakly similar to Homo sapiens matrilin 4 (MATN4).<br>Homo sapiens colon cancer secreted protein-2 (CCSP-2) mRNA, complete cds.<br>Homo sapiens mRNA for AMACO.<br><br>cdna:known-ccds chromosome:NCBI36:10:115989008:116039741:1 gene:ENSG00000165816 CCDS7589.1<br>cdna:known chromosome:NCBI36:10:115989079:116041262:1 gene:ENSG00000165816<br>cdna:known chromosome:NCBI36:10:115989079:116039741:1 gene:ENSG00000165816<br>cdna:known chromosome:NCBI36:10:115989241:116041727:1 gene:ENSG00000165816

3265565	592340	3265564	full	10	116842178	117698486	+	NM_207303	Homo sapiens attractin-like 1 (ATRNL1), mRNA.
	592341	3265566	core					BC035157	Homo sapiens attractin-like 1, mRNA (cDNA clone IMAGE:5265311), complete cds.
	592342	3265567	core					BC046219	Homo sapiens attractin-like 1, mRNA (cDNA clone IMAGE:6196446), partial cds.
	592343	3265568	core					BC029592	Homo sapiens attractin-like 1, mRNA (cDNA clone IMAGE:5272894), with apparent retained intron.
	592344	3265569	core					ENSESTT00000011049	
	592345	3265570	core					ENSESTT00000011051	
	592346	3265571	core					ENSESTT00000011053	
	592347	3265572	full					ENSESTT00000011054	
	592348	3265573	extended					ENSESTT00000011055	
	592349	3265574	extended					ENSESTT00000011056	
	592350	3265575	full					ENSESTT00000011059	
	592351	3265576	full					ENST00000355044	cdna:known-ccds chromosome:NCBI36:10:116843114:117698484:1 gene:ENSG00000107518 CCDS7592.1
	592352	3265577	full					ENST00000303745	cdna:known chromosome:NCBI36:10:116843114:117347296:1 gene:ENSG00000107518
	592353	3265578	core					GENSCAN00000059896	cdna:Genscan chromosome:NCBI36:10:117576891:117604127:1
	592354	3265579	core					GENSCAN00000066833	cdna:Genscan chromosome:NCBI36:10:116843294:116965628:1
	592355	3265580	core					GENSCAN00000026905	cdna:Genscan chromosome:NCBI36:10:117049534:117083919:1
	592356	3265581	extended					GENSCAN00000008267	cdna:Genscan chromosome:NCBI36:10:117468773:117476936:1
	592357	3265582	core					GENSCAN00000025858	cdna:Genscan chromosome:NCBI36:10:116991350:117035897:1
	592358	3265583	core					GENSCAN00000028006	cdna:Genscan chromosome:NCBI36:10:117682909:117683157:1
	592359	3265584	core					GENSCAN00000028005	cdna:Genscan chromosome:NCBI36:10:117688735:117694280:1
	592360	3265585	full						
	592361	3265586	core						
	592362	3265587	core						
	592363	3265588	extended						
	592364	3265589	core						
	592365	3265590	core						
	592366	3265591	core						
	592367	3265592	extended						
	592368	3265593	extended						
	592369	3265594	extended						
	592370	3265595	extended						
	592371	3265596	full						
	592372	3265597	core						
	592373	3265598	core						
	592374	3265599	extended						
	592375	3265600	core						
	592376	3265601	extended						
	592377	3265602	core						
	592378	3265603	core						
	592379	3265604	core						
	592380	3265605	core						
	592381	3265606	core						
	592382	3265607	core						
	592383	3265608	full						
	592384	3265609	core						
	592385	3265610	core						
	592386	3265611	full						
	592387	3265612	extended						
	592388	3265613	extended						
	592389	3265614	extended						
	592390	3265615	extended						
	592391	3265616	core						
	592392	3265617	extended						
	592393	3265618	core						

	592394<br>592395<br>592396<br>592397<br>592398<br>592399<br>592400<br>592401<br>592402<br>592403<br>592404<br>592405<br>592406<br>592407<br>592408<br>592409<br>592410<br>592411<br>592412<br>592413<br>592414<br>592415<br>592416<br>592417<br>592418<br>592419<br>592420<br>592421	3265619<br>3265620<br>3265621<br>3265622<br>3265623<br>3265624<br>3265625<br>3265626<br>3265627<br>3265628<br>3265629<br>3265630<br>3265631<br>3265632<br>3265633<br>3265634<br>3265635<br>3265636<br>3265637<br>3265638<br>3265639<br>3265640<br>3265641<br>3265642<br>3265643<br>3265644<br>3265645<br>3265646<br>3265647<br>3265648<br>3265649<br>3265650<br>3265651<br>3265652<br>3265653<br>3265654<br>3265655<br>3265656<br>3265657<br>3265658<br>3265659<br>3265660<br>3265661<br>3265662<br>3265663<br>3265664<br>3265665<br>3265666<br>3265667<br>3265668<br>3265669<br>3265670<br>3265671	core<br>core<br>core<br>extended<br>core<br>full<br>full<br>extended<br>full<br>core<br>full<br>extended<br>full<br>full<br>full<br>core<br>full<br>extended<br>extended<br>full<br>full<br>extended<br>full<br>full<br>full<br>full<br>full<br>full<br>core<br>extended<br>extended<br>extended<br>full<br>extended<br>extended<br>extended<br>extended<br>full<br>full<br>full<br>full<br>full<br>full<br>full<br>core<br>extended<br>extended<br>core<br>extended<br>extended<br>core<br>extended<br>core						
3292946	609665	3292947	full	10	70832726	70846621	-	NM_001057	Homo sapiens tachykinin receptor 2 (TACR2), mRNA.

	609666<br>609667<br>609668<br>609669<br>609670<br>609671<br>609672<br>609673<br>609674<br>609675<br>609676<br>609677	3292948<br>3292949<br>3292950<br>3292951<br>3292952<br>3292953<br>3292954<br>3292955<br>3292956<br>3292957<br>3292958<br>3292959<br>3292960<br>3292961<br>3292962<br>3292963<br>3292964<br>3292965<br>3292966<br>3292967<br>3292968	full<br>extended<br>extended<br>extended<br>core<br>core<br>core<br>core<br>full<br>core<br>core<br>core<br>extended<br>extended<br>core<br>core<br>core<br>core<br>core<br>core<br>extended					AK096906<br>ENSESTT00000015285<br>ENST00000373306<br>ENST00000352020<br>ENST00000373307<br>GENSCAN00000003211	Homo sapiens cDNA FLJ39587 fis, clone SKMUS2008407, highly similar to SUBSTANCE-K RECEPTOR.<br><br>cdna:known-ccds chromosome:NCBI36:10:70834246:70846629:-1 gene:ENSG00000075073 CCDS7293.1<br>cdna:known chromosome:NCBI36:10:70834429:70846607:-1 gene:ENSG00000075073<br>cdna:novel chromosome:NCBI36:10:70833665:70839044:-1 gene:ENSG00000075073<br>cdna:Genscan chromosome:NCBI36:10:70832754:70869113:-1
3296046	611544<br>611545<br>611546<br>611547<br>611548<br>611549<br>611550<br>611551<br>611552<br>611553<br>611554<br>611555<br>611556<br>611557<br>611558<br>611559<br>611560<br>611561<br>611562<br>611563<br>611564<br>611565<br>611566<br>611567<br>611568<br>611569<br>611570<br>611571<br>611572<br>611573<br>611574<br>611575<br>611576	3296047<br>3296048<br>3296049<br>3296050<br>3296051<br>3296052<br>3296053<br>3296054<br>3296055<br>3296056<br>3296057<br>3296058<br>3296059<br>3296060<br>3296061<br>3296062<br>3296063<br>3296064<br>3296065<br>3296066<br>3296067<br>3296068<br>3296069<br>3296070<br>3296071<br>3296072<br>3296073<br>3296074<br>3296075<br>3296076<br>3296077<br>3296078<br>3296079	extended<br>extended<br>extended<br>full<br>full<br>full<br>full<br>full<br>full<br>full<br>full<br>extended<br>core<br>core<br>core<br>core<br>core<br>extended<br>core<br>full<br>core<br>extended<br>extended<br>extended<br>full<br>full<br>full<br>core<br>extended<br>core<br>full<br>full<br>full	10	78306427	79068243	-	NM_001014797<br>NM_002247<br>AB113382<br>AB113575<br>AK098326<br>AK124355<br>AK128392<br>AY445624<br>BC062659<br>BX648925<br>U02632<br>U09384<br>CR627384<br>BC009695<br>ENSESTT00000014112<br>ENSESTT00000014113<br>ENSESTT00000014114<br>ENST00000286628<br>ENST00000372443<br>ENST00000372440<br>ENST00000372437<br>ENST00000331566<br>ENST00000358063<br>ENST00000354876<br>ENST00000359475<br>ENST00000286627<br>ENST00000356701<br>ENST00000372421<br>ENST00000372408<br>ENST00000372403<br>ENST00000354353<br>GENSCAN00000054253<br>GENSCAN00000059964	Homo sapiens potassium large conductance calcium-activated channel, subfamily M, alpha member 1 (KCNMA1), transcript variant 1, mRNA.<br>Homo sapiens potassium large conductance calcium-activated channel, subfamily M, alpha member 1 (KCNMA1), transcript variant 2, mRNA.<br>Homo sapiens SAKCA mRNA for stretch-activated Kca channel, complete cds.<br>Homo sapiens BKTM mRNA for BK variant stretch-activated Kca channel, complete cds.<br>Homo sapiens cDNA FLJ41007 fis, clone UTERU2017810.<br>Homo sapiens cDNA FLJ42364 fis, clone UTERU2027591, moderately similar to Human calcium-activated potassium channel.<br>Homo sapiens cDNA FLJ46535 fis, clone THYMU3037192, highly similar to Mus musculus potassium large conductance calcium-activated channel, subfamily M, alpha member 1 (Kcnma1).<br>Homo sapiens potassium large conductance calcium-activated channel subfamily M alpha member 1 mRNA, partial cds, alternatively spliced.<br>Homo sapiens potassium large conductance calcium-activated channel, subfamily M, alpha member 1, mRNA (cDNA clone IMAGE:4277048), complete cds.<br>Homo sapiens mRNA; cDNA DKFZp686K1437 (from clone DKFZp686K1437).<br>Human calcium-activated potassium channel mRNA, partial cds.<br>Human calcium-activated potassium channel mRNA, partial cds.<br>Homo sapiens mRNA; cDNA DKFZp781N1049 (from clone DKFZp781N1049).<br>Homo sapiens potassium large conductance calcium-activated channel, subfamily M, alpha member 1, mRNA (cDNA clone IMAGE:3900695), with apparent retained intron.<br><br><br><br>cdna:known-ccds chromosome:NCBI36:10:78314645:79068359:-1 gene:ENSG00000156113 CCDS7352.1<br>cdna:known chromosome:NCBI36:10:78307361:79068133:-1 gene:ENSG00000156113<br>cdna:known chromosome:NCBI36:10:78307361:79067757:-1 gene:ENSG00000156113<br>cdna:known chromosome:NCBI36:10:78307361:79067211:-1 gene:ENSG00000156113<br>cdna:known chromosome:NCBI36:10:78307503:79067217:-1 gene:ENSG00000156113<br>cdna:known chromosome:NCBI36:10:78317030:79067406:-1 gene:ENSG00000156113<br>cdna:known chromosome:NCBI36:10:78317030:79067406:-1 gene:ENSG00000156113<br>cdna:known chromosome:NCBI36:10:78317030:79067406:-1 gene:ENSG00000156113<br>cdna:known chromosome:NCBI36:10:78317030:79067406:-1 gene:ENSG00000156113<br>cdna:known chromosome:NCBI36:10:78317030:79067406:-1 gene:ENSG00000156113

	611577	3296080	full					GENSCAN00000068002	cdna:known chromosome:NCBI36:10:78317057:79067406:-1 gene:ENSG00000156113
	611578	3296081	extended					GENSCAN00000049436	cdna:known chromosome:NCBI36:10:78425249:78833789:-1 gene:ENSG00000156113
	611579	3296082	full					GENSCAN00000049435	cdna:known chromosome:NCBI36:10:78441966:78521934:-1 gene:ENSG00000156113
	611580	3296083	full					GENSCAN00000012754	cdna:known chromosome:NCBI36:10:79065969:79067489:-1 gene:ENSG00000156113
	611581	3296084	full					GENSCAN00000012752	cdna:Genscan chromosome:NCBI36:10:78796288:78833787:-1
	611582	3296085	core						cdna:Genscan chromosome:NCBI36:10:78442268:78509314:-1
	611583	3296086	full						cdna:Genscan chromosome:NCBI36:10:78716456:78781263:-1
	611584	3296087	core						cdna:Genscan chromosome:NCBI36:10:78307503:78312879:-1
	611585	3296088	core						cdna:Genscan chromosome:NCBI36:10:78317030:78405361:-1
	611586	3296089	core						cdna:Genscan chromosome:NCBI36:10:78608628:78681020:-1
	611587	3296090	full						cdna:Genscan chromosome:NCBI36:10:78567065:78570493:-1
	611588	3296091	extended						
	611589	3296092	full						
	611590	3296093	full						
	611591	3296094	full						
	611592	3296095	full						
	611593	3296096	core						
	611594	3296097	extended						
	611595	3296098	extended						
	611596	3296099	extended						
	611597	3296100	extended						
	611598	3296101	full						
	611599	3296102	extended						
	611600	3296103	full						
	611601	3296104	full						
	611602	3296105	full						
	611603	3296106	extended						
	611604	3296107	extended						
	611605	3296108	extended						
	611606	3296109	extended						
	611607	3296110	extended						
	611608	3296111	full						
	611609	3296112	full						
	611610	3296113	core						
	611611	3296114	full						
	611612	3296115	free						
	611613	3296116	full						
	611614	3296117	full						
	611615	3296118	core						
	611616	3296119	extended						
	611617	3296120	extended						
	611618	3296121	full						
	611619	3296122	full						
	611620	3296123	full						
	611621	3296124	extended						
	611622	3296125	core						
	611623	3296126	extended						
	611624	3296127	extended						
	611625	3296128	full						
	611626	3296129	full						
	611627	3296130	full						
	611628	3296131	extended						
	611629	3296132	full						
	611630	3296133	core						

	611631	3296134	full						
	611632	3296135	full						
	611633	3296136	full						
	611634	3296137	extended						
	611635	3296138	extended						
	611636	3296139	extended						
	611637	3296140	full						
	611638	3296141	core						
	611639	3296142	extended						
	611640	3296143	full						
	611641	3296144	extended						
	611642	3296145	full						
	611643	3296146	extended						
	611644	3296147	extended						
	611645	3296148	extended						
	611646	3296149	core						
	611647	3296150	core						
	611648	3296151	full						
	611649	3296152	full						
	611650	3296153	full						
	611651	3296154	full						
	611652	3296155	full						
	611653	3296156	core						
	611654	3296157	core						
	611655	3296158	full						
	611656	3296159	core						
	611657	3296160	extended						
	611658	3296161	core						
	611659	3296162	extended						
	611660	3296163	extended						
	611661	3296164	extended						
	611662	3296165	extended						
	611663	3296166	extended						
	611664	3296167	extended						
	611665	3296168	extended						
	611666	3296169	extended						
	611667	3296170	full						
	611668	3296171	full						
	611669	3296172	full						
	611670	3296173	full						
	611671	3296174	core						
	611672	3296175	full						
	611673	3296176	core						
	611674	3296177	core						
	611675	3296178	core						
	611676	3296179	extended						
	611677	3296180	core						
	611678	3296181	extended						
	611679	3296182	full						
	611680	3296183	full						
	611681	3296184	full						
	611682	3296185	full						
	611683	3296186	full						
	611684	3296187	full						

	611685	3296188	full						
	611686	3296189	extended						
	611687	3296190	full						
	611688	3296191	full						
	611689	3296192	full						
	611690	3296193	core						
	611691	3296194	core						
	611692	3296195	core						
	611693	3296196	full						
	611694	3296197	full						
	611695	3296198	full						
	611696	3296199	full						
	611697	3296200	extended						
	611698	3296201	full						
	611699	3296202	full						
	611700	3296203	full						
	611701	3296204	full						
	611702	3296205	full						
	611703	3296206	extended						
	611704	3296207	extended						
	611705	3296208	full						
	611706	3296209	full						
	611707	3296210	full						
	611708	3296211	extended						
	611709	3296212	core						
	611710	3296213	full						
	611711	3296214	full						
	611712	3296215	full						
	611713	3296216	full						
	611714	3296217	full						
	611715	3296218	full						
	611716	3296219	full						
	611717	3296220	full						
	611718	3296221	full						
	611719	3296222	full						
	611720	3296223	full						
	611721	3296224	full						
	611722	3296225	full						
	611723	3296226	extended						
	611724	3296227	full						
	611725	3296228	full						
	611726	3296229	full						
	611727	3296230	full						
	611728	3296231	full						
	611729	3296232	full						
	611730	3296233	full						
		3296234	extended						
		3296235	full						
		3296236	full						
		3296237	full						
		3296238	full						
		3296239	full						
		3296240	extended						
		3296241	extended						

		3296242<br>3296243<br>3296244<br>3296245<br>3296246<br>3296247<br>3296248<br>3296249<br>3296250<br>3296251<br>3296252<br>3296253<br>3296254<br>3296255<br>3296256<br>3296257<br>3296258<br>3296259<br>3296260<br>3296261<br>3296262<br>3296263<br>3296264<br>3296265<br>3296266<br>3296267<br>3296268<br>3296269<br>3296270<br>3296271<br>3296272<br>3296273<br>3296274<br>3296275<br>3296276<br>3296277<br>3296278<br>3296279<br>3296280<br>3296281<br>3296282<br>3296283<br>3296284<br>3296285<br>3296286<br>3296287	core<br>extended<br>extended<br>extended<br>full<br>extended<br>full<br>full<br>extended<br>extended<br>extended<br>extended<br>full<br>full<br>full<br>full<br>extended<br>full<br>full<br>extended<br>extended<br>extended<br>extended<br>extended<br>extended<br>extended<br>full<br>extended<br>extended<br>extended<br>full<br>full<br>full<br>extended<br>extended<br>core<br>core<br>core<br>extended<br>extended<br>full<br>core<br>core<br>core<br>extended<br>full						
3300597	614415<br>614416<br>614417<br>614418<br>614419<br>614420<br>614421<br>614422	3300598<br>3300599<br>3300600<br>3300601<br>3300602<br>3300603<br>3300604<br>3300605	full<br>full<br>extended<br>full<br>extended<br>extended<br>core<br>core	10	95037451	95232136	-	NM_013451<br>NM_133337<br>AK093890<br>BC040110<br>AJ431614<br>BC033616<br>ENSESTT00000025848<br>ENSESTT00000025849	Homo sapiens fer-1-like 3, myoferlin (C. elegans) (FER1L3), transcript variant 1, mRNA.<br>Homo sapiens fer-1-like 3, myoferlin (C. elegans) (FER1L3), transcript variant 2, mRNA.<br>Homo sapiens cDNA FLJ36571 fis, clone TRACH2012242, highly similar to Fer-1 (C.elegans)-like 3 (myoferlin).<br>Homo sapiens, Similar to fer-1-like 3, myoferlin (C. elegans), clone IMAGE:3882757, mRNA, partial cds.<br>Homo sapiens mRNA adjacent to 3' end of integrated HPV16 (INT505).<br>Homo sapiens fer-1-like 3, myoferlin (C. elegans), mRNA (cDNA clone IMAGE:5405291), with apparent retained intron.

	614423	3300606	core					ENSESTT00000025850	
	614424	3300607	full					ENSESTT00000025851	
	614425	3300608	core					ENSESTT00000025852	
	614426	3300609	extended					ENST00000359263	cdna:known chromosome:NCBI36:10:95056177:95232032:-1 gene:ENSG00000138119
	614427	3300610	core					ENST00000358334	cdna:known chromosome:NCBI36:10:95056177:95232032:-1 gene:ENSG00000138119
	614428	3300611	core					ENST00000371502	cdna:known chromosome:NCBI36:10:95056177:95232032:-1 gene:ENSG00000138119
	614429	3300612	core					ENST00000347778	cdna:known chromosome:NCBI36:10:95056177:95231941:-1 gene:ENSG00000138119
	614430	3300613	core					ENST00000371501	cdna:known chromosome:NCBI36:10:95056177:95231941:-1 gene:ENSG00000138119
	614431	3300614	full					ENST00000371490	cdna:known chromosome:NCBI36:10:95056177:95130980:-1 gene:ENSG00000138119
	614432	3300615	core					ENST00000371489	cdna:known chromosome:NCBI36:10:95146932:95232064:-1 gene:ENSG00000138119
	614433	3300616	extended					ENST00000371488	cdna:known chromosome:NCBI36:10:95175488:95231941:-1 gene:ENSG00000138119
	614434	3300617	extended					GENSCAN00000011513	cdna:Genscan chromosome:NCBI36:10:95175818:95231941:-1
	614435	3300618	core					GENSCAN00000038278	cdna:Genscan chromosome:NCBI36:10:95050113:95167060:-1
	614436	3300619	core						
	614437	3300620	extended						
	614438	3300621	core						
	614439	3300622	full						
	614440	3300623	core						
	614441	3300624	core						
	614442	3300625	core						
	614443	3300626	core						
	614444	3300627	extended						
	614445	3300628	core						
	614446	3300629	extended						
	614447	3300630	extended						
	614448	3300631	core						
	614449	3300632	extended						
	614450	3300633	extended						
	614451	3300634	full						
	614452	3300635	extended						
	614453	3300636	extended						
	614454	3300637	core						
	614455	3300638	core						
	614456	3300639	full						
	614457	3300640	full						
	614458	3300641	full						
	614459	3300642	core						
	614460	3300643	core						
	614461	3300644	core						
	614462	3300645	core						
	614463	3300646	core						
	614464	3300647	core						
	614465	3300648	core						
	614466	3300649	core						
	614467	3300650	core						
	614468	3300651	core						
	614469	3300652	core						
	614470	3300653	extended						
	614471	3300654	core						
	614472	3300655	extended						
	614473	3300656	core						
	614474	3300657	core						
	614475	3300658	core						
	614476	3300659	full						

	614477	3300660	core						
	614478	3300661	core						
	614479	3300662	core						
	614480	3300663	full						
	614481	3300664	core						
	614482	3300665	extended						
	614483	3300666	extended						
	614484	3300667	extended						
	614485	3300668	extended						
	614486	3300669	full						
	614487	3300670	full						
	614488	3300671	core						
	614489	3300672	core						
	614490	3300673	full						
	614491	3300674	full						
	614492	3300675	core						
	614493	3300676	full						
	614494	3300677	extended						
	614495	3300678	core						
	614496	3300679	extended						
	614497	3300680	core						
	614498	3300681	core						
	614499	3300682	extended						
	614500	3300683	core						
	614501	3300684	extended						
	614502	3300685	extended						
	614503	3300686	core						
	614504	3300687	core						
	614505	3300688	core						
	614506	3300689	core						
	614507	3300690	core						
	614508	3300691	core						
	614509	3300692	full						
	614510	3300693	full						
	614511	3300694	core						
	614512	3300695	full						
	614513	3300696	full						
	614514	3300697	core						
	614515	3300698	full						
	614516	3300699	full						
	614517	3300700	full						
	614518	3300701	full						
		3300702	extended						
		3300703	full						
		3300704	full						
		3300705	full						
		3300706	extended						
		3300707	core						
		3300708	core						
		3300709	full						
		3300710	full						
		3300711	full						
		3300712	full						
		3300713	full						

		3300714<br>3300715<br>3300716<br>3300717<br>3300718<br>3300719<br>3300720<br>3300721<br>3300722<br>3300723<br>3300724<br>3300725<br>3300726<br>3300727<br>3300728<br>3300729<br>3300730<br>3300731<br>3300732<br>3300733<br>3300734	full<br>full<br>full<br>full<br>full<br>core<br>extended<br>core<br>full<br>full<br>full<br>full<br>full<br>full<br>extended<br>full<br>extended<br>extended<br>full<br>core<br>extended						
3301263	614823<br>614824<br>614825<br>614826<br>614827<br>614828<br>614829<br>614830<br>614831<br>614832<br>614833<br>614834<br>614835<br>614836<br>614837<br>614838<br>614839<br>614840<br>614841<br>614842<br>614843<br>614844<br>614845<br>614846<br>614847<br>614848<br>614849<br>614850<br>614851<br>614852<br>614853<br>614854<br>614855	3301264<br>3301265<br>3301266<br>3301267<br>3301268<br>3301269<br>3301270<br>3301271<br>3301272<br>3301273<br>3301274<br>3301275<br>3301276<br>3301277<br>3301278<br>3301279<br>3301280<br>3301281<br>3301282<br>3301283<br>3301284<br>3301285<br>3301286<br>3301287<br>3301288<br>3301289<br>3301290<br>3301291<br>3301292<br>3301293<br>3301294<br>3301295<br>3301296	full<br>full<br>full<br>full<br>full<br>full<br>full<br>full<br>full<br>extended<br>core<br>core<br>core<br>core<br>core<br>core<br>core<br>core<br>full<br>full<br>core<br>core<br>core<br>extended<br>core<br>full<br>full<br>extended<br>extended<br>extended<br>extended<br>extended<br>extended	10	97043249	97311141	-	NM_006434<br>NM_015385<br>NM_001034954<br>NM_001034955<br>NM_001034956<br>NM_024991<br>NM_001034957<br>AF143880<br>AF356526<br>AK022468<br>BC042612<br>AL832353<br>BX640814<br>ENSESTT00000035127<br>ENSESTT00000035128<br>ENSESTT00000035129<br>ENSESTT00000035130<br>ENSESTT00000035131<br>ENSESTT00000035132<br>ENSESTT00000035133<br>ENSESTT00000035134<br>ENST00000354607<br>ENST00000371254<br>ENST00000306402<br>ENST00000371249<br>ENST00000371247<br>ENST00000371246<br>ENST00000371245<br>ENST00000347291<br>ENST00000265987<br>ENST00000277983<br>ENST00000354106<br>ENST00000371239	Homo sapiens sorbin and SH3 domain containing 1 (SORBS1), transcript variant 1, mRNA.<br>Homo sapiens sorbin and SH3 domain containing 1 (SORBS1), transcript variant 2, mRNA.<br>Homo sapiens sorbin and SH3 domain containing 1 (SORBS1), transcript variant 3, mRNA.<br>Homo sapiens sorbin and SH3 domain containing 1 (SORBS1), transcript variant 4, mRNA.<br>Homo sapiens sorbin and SH3 domain containing 1 (SORBS1), transcript variant 5, mRNA.<br>Homo sapiens sorbin and SH3 domain containing 1 (SORBS1), transcript variant 6, mRNA.<br>Homo sapiens sorbin and SH3 domain containing 1 (SORBS1), transcript variant 7, mRNA.<br>Homo sapiens clone IMAGE:121018 mRNA sequence.<br>Homo sapiens sorbin and SH3 domain containing 1 mRNA, complete cds, alternatively spliced.<br>Homo sapiens cDNA FLJ12406 fis, clone MAMMA1002842, weakly similar to Mus musculus c-Cbl associated protein CAP mRNA.<br>Homo sapiens sorbin and SH3 domain containing 1, mRNA (cDNA clone IMAGE:4821975), complete cds.<br>Homo sapiens mRNA; cDNA DKFZp451C066 (from clone DKFZp451C066).<br>Homo sapiens mRNA; cDNA DKFZp686N08224 (from clone DKFZp686N08224).<br><br><br><br><br><br><br><br><br><br>cdna:known-ccds chromosome:NCBI36:10:97061520:97311125:-1 gene:ENSG00000095637 CCDS7442.1<br>cdna:known chromosome:NCBI36:10:97061518:97190909:-1 gene:ENSG00000095637<br>cdna:known chromosome:NCBI36:10:97061520:97311161:-1 gene:ENSG00000095637<br>cdna:known chromosome:NCBI36:10:97061521:97311161:-1 gene:ENSG00000095637<br>cdna:known chromosome:NCBI36:10:97061521:97190927:-1 gene:ENSG00000095637<br>cdna:known chromosome:NCBI36:10:97061521:97190927:-1 gene:ENSG00000095637<br>cdna:known chromosome:NCBI36:10:97061521:97190927:-1 gene:ENSG00000095637<br>cdna:known chromosome:NCBI36:10:97064574:97187312:-1 gene:ENSG00000095637<br>cdna:known chromosome:NCBI36:10:97064772:97190909:-1 gene:ENSG00000095637<br>cdna:known chromosome:NCBI36:10:97064772:97190909:-1 gene:ENSG00000095637<br>cdna:known chromosome:NCBI36:10:97064806:97190882:-1 gene:ENSG00000095637

	614856	3301297	extended					ENST00000371241	cdna:known chromosome:NCBI36:10:97064806:97190882:-1 gene:ENSG00000095637
	614857	3301298	extended					ENST00000353505	cdna:known chromosome:NCBI36:10:97064806:97190882:-1 gene:ENSG00000095637
	614858	3301299	full					ENST00000361941	cdna:known chromosome:NCBI36:10:97091038:97311161:-1 gene:ENSG00000095637
	614859	3301300	core					ENST00000371228	cdna:known chromosome:NCBI36:10:97099878:97190927:-1 gene:ENSG00000095637
	614860	3301301	core					ENST00000371227	cdna:known chromosome:NCBI36:10:97104693:97106993:-1 gene:ENSG00000095637
	614861	3301302	core					ENST00000277982	cdna:known chromosome:NCBI36:10:97140404:97311120:-1 gene:ENSG00000095637
	614862	3301303	core					ENST00000351042	cdna:novel chromosome:NCBI36:10:97064806:97187312:-1 gene:ENSG00000095637
	614863	3301304	full					GENSCAN00000058507	cdna:novel chromosome:NCBI36:10:97064806:97187312:-1 gene:ENSG00000095637
	614864	3301305	full					GENSCAN00000023778	cdna:Genscan chromosome:NCBI36:10:97229030:97251630:-1
	614865	3301306	extended					GENSCAN00000019317	cdna:Genscan chromosome:NCBI36:10:97064806:97196542:-1
	614866	3301307	extended						cdna:Genscan chromosome:NCBI36:10:97013611:97047602:-1
	614867	3301308	extended						
	614868	3301309	core						
	614869	3301310	core						
	614870	3301311	core						
	614871	3301312	core						
	614872	3301313	core						
	614873	3301314	extended						
	614874	3301315	extended						
	614875	3301316	core						
	614876	3301317	extended						
	614877	3301318	extended						
	614878	3301319	extended						
	614879	3301320	core						
	614880	3301321	extended						
	614881	3301322	full						
	614882	3301323	core						
	614883	3301324	full						
	614884	3301325	extended						
	614885	3301326	extended						
	614886	3301327	core						
	614887	3301328	extended						
	614888	3301329	full						
	614889	3301330	extended						
	614890	3301331	extended						
	614891	3301332	extended						
	614892	3301333	extended						
	614893	3301334	core						
	614894	3301335	core						
	614895	3301336	full						
	614896	3301337	full						
	614897	3301338	extended						
	614898	3301339	core						
	614899	3301340	full						
	614900	3301341	extended						
	614901	3301342	extended						
	614902	3301343	extended						
	614903	3301344	core						
	614904	3301345	extended						
	614905	3301346	core						
	614906	3301347	extended						
	614907	3301348	core						
	614908	3301349	extended						
	614909	3301350	extended						

	614910	3301351	extended						
	614911	3301352	extended						
	614912	3301353	extended						
	614913	3301354	extended						
	614914	3301355	extended						
	614915	3301356	extended						
	614916	3301357	extended						
	614917	3301358	extended						
	614918	3301359	extended						
	614919	3301360	extended						
	614920	3301361	core						
	614921	3301362	full						
	614922	3301363	core						
	614923	3301364	core						
	614924	3301365	extended						
	614925	3301366	extended						
	614926	3301367	extended						
	614927	3301368	core						
	614928	3301369	extended						
	614929	3301370	extended						
	614930	3301371	extended						
	614931	3301372	extended						
	614932	3301373	extended						
	614933	3301374	extended						
	614934	3301375	extended						
	614935	3301376	extended						
	614936	3301377	extended						
	614937	3301378	extended						
	614938	3301379	extended						
	614939	3301380	extended						
	614940	3301381	full						
	614941	3301382	core						
	614942	3301383	extended						
	614943	3301384	core						
	614944	3301385	extended						
	614945	3301386	extended						
	614946	3301387	full						
	614947	3301388	full						
	614948	3301389	core						
	614949	3301390	core						
	614950	3301391	full						
	614951	3301392	extended						
	614952	3301393	core						
	614953	3301394	extended						
	614954	3301395	extended						
	614955	3301396	extended						
		3301397	core						
		3301398	extended						
		3301399	extended						
		3301400	extended						
		3301401	extended						
		3301402	core						
		3301403	core						
		3301404	core						

		3301405	full						
		3301406	extended						
		3301407	extended						
		3301408	extended						
		3301409	extended						
		3301410	extended						
		3301411	core						
		3301412	full						
		3301413	core						
		3301414	full						
		3301415	extended						
		3301416	full						
		3301417	core						
		3301418	full						
		3301419	full						
		3301420	full						
		3301421	full						
		3301422	full						
		3301423	core						
		3301424	full						
		3301425	full						
		3301426	full						
		3301427	full						
		3301428	full						
		3301429	full						
		3301430	full						
		3301431	full						
		3301432	core						
		3301433	full						
		3301434	full						
		3301435	full						
		3301436	full						
		3301437	core						
		3301438	extended						
		3301439	extended						
		3301440	extended						
		3301441	extended						
		3301442	extended						
		3301443	extended						
		3301444	extended						
		3301445	full						
		3301446	extended						
		3301447	core						
		3301448	full						
		3301449	core						
		3301450	core						
		3301451	extended						
		3301452	full						
		3301453	full						
		3301454	full						
		3301455	full						
		3301456	full						
		3301457	full						
		3301458	full						

		3301459<br>3301460<br>3301461<br>3301462<br>3301463<br>3301464<br>3301465<br>3301466<br>3301467<br>3301468<br>3301469<br>3301470<br>3301471<br>3301472<br>3301473<br>3301474<br>3301475<br>3301476<br>3301477<br>3301478	extended<br>extended<br>full<br>extended<br>extended<br>extended<br>full<br>extended<br>full<br>full<br>full<br>extended<br>extended<br>extended<br>extended<br>full<br>full<br>full<br>extended<br>extended						
3304301	616721<br>616722<br>616723<br>616724<br>616725<br>616726<br>616727<br>616728<br>616729<br>616730<br>616731<br>616732<br>616733<br>616734<br>616735<br>616736<br>616737<br>616738<br>616739<br>616740<br>616741<br>616742<br>616743<br>616744<br>616745<br>616746<br>616747<br>616748	3304302<br>3304303<br>3304304<br>3304305<br>3304306<br>3304307<br>3304308<br>3304309<br>3304310<br>3304311<br>3304312<br>3304313<br>3304314<br>3304315<br>3304316<br>3304317<br>3304318<br>3304319<br>3304320<br>3304321<br>3304322<br>3304323<br>3304324<br>3304325<br>3304326<br>3304327<br>3304328<br>3304329<br>3304330<br>3304331<br>3304332<br>3304333<br>3304334<br>3304335	core<br>core<br>core<br>core<br>core<br>extended<br>extended<br>core<br>full<br>core<br>core<br>core<br>extended<br>extended<br>extended<br>core<br>extended<br>extended<br>extended<br>extended<br>core<br>extended<br>core<br>core<br>core<br>core<br>extended<br>extended<br>core<br>core<br>core<br>extended<br>extended<br>extended	10	104152386	104169466	-	NM_002779<br>AF452641<br>CR616163<br>ENSESTT00000007152<br>ENSESTT00000007153<br>ENSESTT00000007154<br>ENST00000020673<br>GENSCAN00000036199	Homo sapiens pleckstrin and Sec7 domain containing (PSD), mRNA.<br>Homo sapiens tissue-type brain hypothetical protein mRNA, complete cds.<br>full-length cDNA clone CS0DF031YD08 of Fetal brain of Homo sapiens (human).<br><br><br><br>cdna:known chromosome:NCBI36:10:104152366:104168891:-1 gene:ENSG00000059915<br>cdna:Genscan chromosome:NCBI36:10:104152947:104166785:-1

		3304336<br>3304337<br>3304338<br>3304339<br>3304340<br>3304341<br>3304342<br>3304343<br>3304344<br>3304345<br>3304346<br>3304347<br>3304348<br>3304349<br>3304350<br>3304351<br>3304352	core<br>core<br>extended<br>core<br>core<br>core<br>core<br>core<br>extended<br>extended<br>extended<br>extended<br>extended<br>core<br>extended<br>extended<br>extended						
3311832	621460<br>621461<br>621462<br>621463<br>621464<br>621465<br>621466<br>621467<br>621468<br>621469<br>621470<br>621471<br>621472<br>621473<br>621474<br>621475<br>621476<br>621477<br>621478<br>621479<br>621480<br>621481<br>621482<br>621483<br>621484<br>621485<br>621486<br>621487<br>621488<br>621489<br>621490<br>621491<br>621492<br>621493<br>621494<br>621495<br>621496	3311833<br>3311834<br>3311835<br>3311836<br>3311837<br>3311838<br>3311839<br>3311840<br>3311841<br>3311842<br>3311843<br>3311844<br>3311845<br>3311846<br>3311847<br>3311848<br>3311849<br>3311850<br>3311851<br>3311852<br>3311853<br>3311854<br>3311855<br>3311856<br>3311857<br>3311858<br>3311859<br>3311860<br>3311861<br>3311862<br>3311863<br>3311864<br>3311865<br>3311866<br>3311867<br>3311868<br>3311869	extended<br>extended<br>extended<br>core<br>core<br>core<br>core<br>full<br>full<br>full<br>core<br>core<br>extended<br>extended<br>extended<br>core<br>extended<br>core<br>extended<br>extended<br>core<br>core<br>extended<br>core<br>full<br>full<br>core<br>core<br>core<br>full<br>full<br>full<br>full<br>full<br>full<br>extended<br>extended	10	127690956	128067045	-	NM_021641<br>NM_003474<br>AK022196<br>AK024385<br>AK055628<br>CR617061<br>ENSESTT00000005567<br>ENSESTT00000005568<br>ENSESTT00000005569<br>ENSESTT00000005570<br>ENSESTT00000005571<br>ENST00000368683<br>ENST00000368676<br>ENST00000278082<br>ENST00000368679<br>ENST00000308483<br>GENSCAN00000056396<br>GENSCAN00000031509<br>GENSCAN00000031508	Homo sapiens ADAM metallopeptidase domain 12 (meltrin alpha) (ADAM12), transcript variant 2, mRNA.<br>Homo sapiens ADAM metallopeptidase domain 12 (meltrin alpha) (ADAM12), transcript variant 1, mRNA.<br>Homo sapiens cDNA FLJ12134 fis, clone MAMMA1000287.<br>Homo sapiens cDNA FLJ14323 fis, clone PLACE4000093.<br>Homo sapiens cDNA FLJ31066 fis, clone HSYRA2001153.<br>full-length cDNA clone CS0DI032YO21 of Placenta Cot 25-normalized of Homo sapiens (human).<br><br><br><br><br><br>cdna:known-ccds chromosome:NCBI36:10:127690940:128067014:-1 gene:ENSG00000148848 CCDS7653.1<br>cdna:known-ccds chromosome:NCBI36:10:127719904:128067055:-1 gene:ENSG00000148848 CCDS7654.1<br>cdna:known chromosome:NCBI36:10:127693415:128067055:-1 gene:ENSG00000148848<br>cdna:known chromosome:NCBI36:10:127693415:128067055:-1 gene:ENSG00000148848<br>cdna:known chromosome:NCBI36:10:127719905:128067014:-1 gene:ENSG00000148848<br>cdna:Genscan chromosome:NCBI36:10:128065709:128066704:-1<br>cdna:Genscan chromosome:NCBI36:10:127688610:127753674:-1<br>cdna:Genscan chromosome:NCBI36:10:127759748:127762855:-1

	621497	3311870	core						
	621498	3311871	full						
	621499	3311872	full						
	621500	3311873	core						
	621501	3311874	extended						
	621502	3311875	extended						
	621503	3311876	extended						
	621504	3311877	extended						
	621505	3311878	core						
	621506	3311879	extended						
	621507	3311880	full						
	621508	3311881	extended						
	621509	3311882	full						
	621510	3311883	full						
	621511	3311884	extended						
	621512	3311885	extended						
	621513	3311886	full						
	621514	3311887	extended						
	621515	3311888	extended						
	621516	3311889	extended						
	621517	3311890	extended						
	621518	3311891	extended						
	621519	3311892	full						
	621520	3311893	extended						
	621521	3311894	core						
	621522	3311895	extended						
	621523	3311896	extended						
	621524	3311897	extended						
	621525	3311898	extended						
	621526	3311899	extended						
	621527	3311900	extended						
	621528	3311901	core						
	621529	3311902	extended						
	621530	3311903	core						
	621531	3311904	core						
	621532	3311905	extended						
	621533	3311906	full						
	621534	3311907	full						
	621535	3311908	full						
	621536	3311909	full						
	621537	3311910	full						
	621538	3311911	full						
	621539	3311912	full						
	621540	3311913	core						
	621541	3311914	core						
	621542	3311915	extended						
	621543	3311916	extended						
	621544	3311917	extended						
	621545	3311918	core						
	621546	3311919	extended						
	621547	3311920	full						
	621548	3311921	extended						
	621549	3311922	extended						
	621550	3311923	extended						

	621551	3311924	full						
	621552	3311925	full						
	621553	3311926	extended						
	621554	3311927	extended						
	621555	3311928	extended						
	621556	3311929	extended						
	621557	3311930	extended						
	621558	3311931	core						
	621559	3311932	extended						
	621560	3311933	full						
	621561	3311934	full						
	621562	3311935	full						
	621563	3311936	full						
	621564	3311937	extended						
	621565	3311938	extended						
	621566	3311939	core						
	621567	3311940	full						
	621568	3311941	extended						
	621569	3311942	extended						
	621570	3311943	extended						
	621571	3311944	extended						
	621572	3311945	full						
	621573	3311946	extended						
	621574	3311947	extended						
	621575	3311948	extended						
	621576	3311949	full						
	621577	3311950	extended						
	621578	3311951	extended						
	621579	3311952	extended						
	621580	3311953	extended						
	621581	3311954	extended						
	621582	3311955	core						
	621583	3311956	full						
		3311957	full						
		3311958	extended						
		3311959	extended						
		3311960	extended						
		3311961	extended						
		3311962	full						
		3311963	extended						
		3311964	full						
		3311965	extended						
		3311966	extended						
		3311967	extended						
		3311968	full						
		3311969	extended						
		3311970	extended						
		3311971	full						
		3311972	extended						
		3311973	core						
		3311974	extended						
		3311975	extended						
		3311976	extended						
		3311977	extended						

		3311978<br>3311979<br>3311980<br>3311981<br>3311982<br>3311983<br>3311984<br>3311985<br>3311986<br>3311987<br>3311988	extended<br>full<br>extended<br>extended<br>full<br>extended<br>extended<br>full<br>full<br>core<br>core						
3315675	623856<br>623857<br>623858<br>623859<br>623860<br>623861	3315676<br>3315677<br>3315678<br>3315679<br>3315680<br>3315681<br>3315682<br>3315683<br>3315684<br>3315685<br>3315686<br>3315687	full<br>full<br>extended<br>core<br>core<br>core<br>extended<br>extended<br>core<br>core<br>core<br>core	11	300891	305260	+	NM_003641<br>ENSESTT00000022153<br>ENST00000328221<br>ENST00000382607<br>GENSCAN00000022433	Homo sapiens interferon induced transmembrane protein 1 (9-27) (IFITM1), mRNA.<br><br>cdna:known chromosome:NCBI36:11:303858:305272:1 gene:ENSG00000185885<br>cdna:novel chromosome:NCBI36:11:303858:305272:1 gene:ENSG00000185885<br>cdna:Genscan chromosome:NCBI36:11:300891:305113:1
3317352	624818<br>624819<br>624820<br>624821<br>624822<br>624823<br>624824<br>624825<br>624826<br>624827<br>624828<br>624829<br>624830<br>624831<br>624832<br>624833<br>624834<br>624835<br>624836<br>624837<br>624838<br>624839<br>624840<br>624841<br>624842<br>624843<br>624844<br>624845<br>624846<br>624847	3317353<br>3317354<br>3317355<br>3317356<br>3317357<br>3317358<br>3317359<br>3317360<br>3317361<br>3317362<br>3317363<br>3317364<br>3317365<br>3317366<br>3317367<br>3317368<br>3317369<br>3317370<br>3317371<br>3317372<br>3317373<br>3317374<br>3317375<br>3317376<br>3317377<br>3317378<br>3317379<br>3317380<br>3317381<br>3317382	full<br>full<br>full<br>core<br>core<br>core<br>core<br>full<br>full<br>full<br>core<br>full<br>extended<br>extended<br>extended<br>extended<br>extended<br>extended<br>full<br>core<br>full<br>core<br>full<br>full<br>full<br>full<br>full<br>full<br>full<br>full	11	2414521	2826915	+	NM_181797<br>NM_181798<br>NM_000218<br>AK054562<br>AK226077<br>AY114213<br>AJ006344<br>U89364<br>CR608266<br>ENSESTT00000027941<br>ENSESTT00000027942<br>ENSESTT00000027943<br>ENSESTT00000027944<br>ENST00000155840<br>ENST00000380776<br>ENST00000335475<br>ENST00000345015<br>GENSCAN00000003960<br>GENSCAN00000003965<br>GENSCAN00000045769<br>GENSCAN00000045770<br>GENSCAN00000021386<br>GENSCAN00000021384	Homo sapiens potassium voltage-gated channel, KQT-like subfamily, member 1 (KCNQ1), transcript variant 3, mRNA.<br>Homo sapiens potassium voltage-gated channel, KQT-like subfamily, member 1 (KCNQ1), transcript variant 2, mRNA.<br>Homo sapiens potassium voltage-gated channel, KQT-like subfamily, member 1 (KCNQ1), transcript variant 1, mRNA.<br>Homo sapiens mRNA for FLJ00054 protein, partial cds.<br>Homo sapiens mRNA for uncoupling protein 2 variant, clone: sh02214.<br>Homo sapiens potassium channel protein KCNQ1 variant mRNA, complete cds.<br>Homo sapiens KvLQT1 gene, isoform 2.<br>Homo sapiens voltage gated potassium channel (KVLQT1) mRNA, complete cds.<br>full-length cDNA clone CS0DK007YA04 of HeLa cells Cot 25-normalized of Homo sapiens (human).<br><br><br><br><br>cdna:known-ccds chromosome:NCBI36:11:2422797:2826915:1 gene:ENSG00000053918 CCDS7736.1<br>cdna:known chromosome:NCBI36:11:2422797:2826797:1 gene:ENSG00000053918<br>cdna:known chromosome:NCBI36:11:2423128:2826915:1 gene:ENSG00000053918<br>cdna:known chromosome:NCBI36:11:2439260:2826044:1 gene:ENSG00000053918<br>cdna:Genscan chromosome:NCBI36:11:2494798:2513468:1<br>cdna:Genscan chromosome:NCBI36:11:2422349:2442095:1<br>cdna:Genscan chromosome:NCBI36:11:2785205:2825809:1<br>cdna:Genscan chromosome:NCBI36:11:2746650:2770724:1<br>cdna:Genscan chromosome:NCBI36:11:2677371:2677625:1<br>cdna:Genscan chromosome:NCBI36:11:2633227:2641485:1

	624848	3317383	full						
	624849	3317384	full						
	624850	3317385	full						
	624851	3317386	full						
	624852	3317387	full						
	624853	3317388	core						
	624854	3317389	core						
	624855	3317390	core						
	624856	3317391	core						
	624857	3317392	full						
	624858	3317393	full						
	624859	3317394	full						
	624860	3317395	core						
	624861	3317396	core						
	624862	3317397	core						
	624863	3317398	core						
	624864	3317399	full						
	624865	3317400	full						
	624866	3317401	full						
	624867	3317402	full						
	624868	3317403	extended						
	624869	3317404	extended						
	624870	3317405	full						
	624871	3317406	full						
	624872	3317407	full						
	624873	3317408	full						
	624874	3317409	full						
	624875	3317410	full						
	624876	3317411	core						
	624877	3317412	full						
	624878	3317413	extended						
	624879	3317414	extended						
	624880	3317415	extended						
	624881	3317416	extended						
	624882	3317417	full						
	624883	3317418	extended						
	624884	3317419	extended						
	624885	3317420	full						
	624886	3317421	extended						
	624887	3317422	extended						
	624888	3317423	full						
	624889	3317424	full						
	624890	3317425	full						
	624891	3317426	full						
	624892	3317427	full						
	624893	3317428	full						
	624894	3317429	full						
	624895	3317430	full						
	624896	3317431	full						
	624897	3317432	extended						
	624898	3317433	full						
	624899	3317434	full						
	624900	3317435	extended						
	624901	3317436	extended						

	624902<br>624903<br>624904<br>624905<br>624906<br>624907<br>624908<br>624909<br>624910<br>624911<br>624912<br>624913<br>624914<br>624915<br>624916<br>624917<br>624918<br>624919<br>624920<br>624921	3317437<br>3317438<br>3317439<br>3317440<br>3317441<br>3317442<br>3317443<br>3317444<br>3317445<br>3317446<br>3317447<br>3317448<br>3317449<br>3317450<br>3317451<br>3317452<br>3317453<br>3317454<br>3317455<br>3317456<br>3317457<br>3317458<br>3317459<br>3317460<br>3317461<br>3317462<br>3317463<br>3317464<br>3317465<br>3317466<br>3317467<br>3317468<br>3317469<br>3317470<br>3317471<br>3317472<br>3317473<br>3317474	extended<br>full<br>core<br>full<br>core<br>core<br>extended<br>full<br>core<br>full<br>full<br>full<br>full<br>full<br>full<br>full<br>full<br>full<br>full<br>full<br>extended<br>extended<br>extended<br>extended<br>extended<br>full<br>full<br>full<br>full<br>full<br>full<br>full<br>full<br>core<br>core<br>core<br>core<br>core						
3326183	630372<br>630373<br>630374<br>630375<br>630376<br>630377<br>630378<br>630379<br>630380<br>630381<br>630382<br>630383<br>630384<br>630385<br>630386<br>630387	3326184<br>3326185<br>3326186<br>3326187<br>3326188<br>3326189<br>3326190<br>3326191<br>3326192<br>3326193<br>3326194<br>3326195<br>3326196<br>3326197<br>3326198<br>3326199	full<br>full<br>full<br>full<br>extended<br>extended<br>extended<br>core<br>core<br>full<br>extended<br>core<br>core<br>core<br>full<br>extended	11	34009711	34081946	+	NM_203364<br>NM_005898<br>AK074309<br>AK127972<br>ENSESTT00000023546<br>ENSESTT00000023547<br>ENSESTT00000023548<br>ENSESTT00000023549<br>ENST00000257845<br>ENST00000389647<br>ENST00000389646<br>ENST00000389645<br>ENST00000389644<br>ENST00000341394<br>GENSCAN00000011465	Homo sapiens GPI-anchored membrane protein 1 (GPIAP1), transcript variant 2, mRNA.<br>Homo sapiens GPI-anchored membrane protein 1 (GPIAP1), transcript variant 1, mRNA.<br>Homo sapiens cDNA FLJ23729 fis, clone HEP14490.<br>Homo sapiens cDNA FLJ46087 fis, clone TESTI2009497, moderately similar to GPI-anchored protein p137.<br><br><br><br><br>cdna:known chromosome:NCBI36:11:34029806:34080733:1 gene:ENSG00000135387<br>cdna:known chromosome:NCBI36:11:34030544:34077490:1 gene:ENSG00000135387<br>cdna:known chromosome:NCBI36:11:34030544:34077490:1 gene:ENSG00000135387<br>cdna:known chromosome:NCBI36:11:34030544:34075904:1 gene:ENSG00000135387<br>cdna:known chromosome:NCBI36:11:34030544:34075904:1 gene:ENSG00000135387<br>cdna:novel chromosome:NCBI36:11:34029806:34080733:1 gene:ENSG00000135387<br>cdna:Genscan chromosome:NCBI36:11:34029860:34077990:1

	630388<br>630389<br>630390<br>630391<br>630392<br>630393<br>630394<br>630395<br>630396<br>630397<br>630398<br>630399<br>630400<br>630401<br>630402<br>630403<br>630404<br>630405<br>630406<br>630407<br>630408<br>630409<br>630410<br>630411<br>630412<br>630413<br>630414	3326200<br>3326201<br>3326202<br>3326203<br>3326204<br>3326205<br>3326206<br>3326207<br>3326208<br>3326209<br>3326210<br>3326211<br>3326212<br>3326213<br>3326214<br>3326215<br>3326216<br>3326217<br>3326218<br>3326219<br>3326220<br>3326221<br>3326222<br>3326223<br>3326224<br>3326225<br>3326226<br>3326227<br>3326228<br>3326229<br>3326230<br>3326231<br>3326232<br>3326233<br>3326234<br>3326235<br>3326236<br>3326237<br>3326238<br>3326239<br>3326240<br>3326241<br>3326242<br>3326243<br>3326244<br>3326245<br>3326246<br>3326247<br>3326248<br>3326249<br>3326250<br>3326251	extended<br>extended<br>extended<br>extended<br>extended<br>extended<br>core<br>core<br>core<br>core<br>core<br>extended<br>extended<br>extended<br>core<br>extended<br>core<br>extended<br>core<br>extended<br>extended<br>core<br>core<br>core<br>core<br>full<br>core<br>core<br>core<br>extended<br>extended<br>extended<br>extended<br>core<br>core<br>full<br>extended<br>core<br>core<br>core<br>extended<br>extended<br>core<br>core<br>core<br>core<br>extended<br>extended<br>extended<br>extended<br>extended<br>extended						
3329099	632205<br>632206	3329100<br>3329101	extended<br>extended	11	45899758	45907222	+	NM_152312<br>AK092526	Homo sapiens glycosyltransferase-like 1B (GYLTL1B), mRNA.<br>Homo sapiens cDNA FLJ35207 fis, clone PLACE6018863.

	632207<br>632208<br>632209<br>632210<br>632211<br>632212<br>632213<br>632214<br>632215<br>632216<br>632217<br>632218<br>632219<br>632220<br>632221<br>632222	3329102<br>3329103<br>3329104<br>3329105<br>3329106<br>3329107<br>3329108<br>3329109<br>3329110<br>3329111<br>3329112<br>3329113<br>3329114<br>3329115<br>3329116<br>3329117<br>3329118<br>3329119<br>3329120<br>3329121<br>3329122<br>3329123<br>3329124<br>3329125<br>3329126<br>3329127<br>3329128<br>3329129<br>3329130<br>3329131<br>3329132<br>3329133<br>3329134<br>3329135	core<br>extended<br>core<br>core<br>core<br>core<br>core<br>extended<br>extended<br>core<br>extended<br>extended<br>extended<br>core<br>core<br>extended<br>core<br>extended<br>core<br>full<br>core<br>extended<br>core<br>extended<br>core<br>core<br>core<br>core<br>extended<br>core<br>core<br>core<br>core<br>core					CR597410<br>ENSESTT00000036692<br>ENSESTT00000036693<br>ENSESTT00000036694<br>ENSESTT00000036695<br>ENSESTT00000036696<br>ENST00000389969<br>ENST00000389968<br>ENST00000298826<br>ENST00000325468<br>GENSCAN00000017011	full-length cDNA clone CS0DI083YB20 of Placenta Cot 25-normalized of Homo sapiens (human).<br><br><br><br><br><br>cdna:known chromosome:NCBI36:11:45901010:45906972:1 gene:ENSG00000165905<br>cdna:known chromosome:NCBI36:11:45901010:45906972:1 gene:ENSG00000165905<br>cdna:novel chromosome:NCBI36:11:45899748:45907223:1 gene:ENSG00000165905<br>cdna:novel chromosome:NCBI36:11:45901010:45907223:1 gene:ENSG00000165905<br>cdna:Genscan chromosome:NCBI36:11:45901010:45906972:1
3332424	634036<br>634037<br>634038<br>634039<br>634040<br>634041<br>634042	3332425<br>3332426<br>3332427<br>3332428<br>3332429<br>3332430<br>3332431<br>3332432<br>3332433	core<br>core<br>core<br>core<br>core<br>core<br>core<br>core<br>core	11	60016827	60031479	+	NM_017716<br>ENSESTT00000011390<br>ENSESTT00000011391<br>ENSESTT00000011392<br>ENST00000016913<br>GENSCAN00000065452	Homo sapiens membrane-spanning 4-domains, subfamily A, member 12 (MS4A12), mRNA.<br><br><br><br>cdna:known-ccds chromosome:NCBI36:11:60016827:60031469:1 gene:ENSG00000071203 CCDS7988.1<br>cdna:Genscan chromosome:NCBI36:11:60021368:60083891:1
3332626	634169<br>634170<br>634171<br>634172<br>634173<br>634174<br>634175<br>634176<br>634177<br>634178	3332627<br>3332628<br>3332629<br>3332630<br>3332631<br>3332632<br>3332633<br>3332634<br>3332635<br>3332636	extended<br>core<br>core<br>extended<br>extended<br>extended<br>extended<br>extended<br>extended<br>extended	11	60448300	60461205	+	NM_017870<br>NM_178031<br>AB046803<br>BC028106<br>CR613429<br>ENSESTT00000011405<br>ENSESTT00000011406<br>ENSESTT00000011407<br>ENSESTT00000011408<br>ENSESTT00000011409	Homo sapiens transmembrane protein 132A (TMEM132A), transcript variant 1, mRNA.<br>Homo sapiens transmembrane protein 132A (TMEM132A), transcript variant 2, mRNA.<br>Homo sapiens mRNA for KIAA1583 protein, partial cds.<br>Homo sapiens transmembrane protein 132A, mRNA (cDNA clone IMAGE:5247458), partial cds.<br>full-length cDNA clone CS0DB001YG10 of Neuroblastoma Cot 10-normalized of Homo sapiens (human).

	634179<br>634180	3332637<br>3332638<br>3332639<br>3332640<br>3332641<br>3332642<br>3332643<br>3332644<br>3332645<br>3332646<br>3332647<br>3332648<br>3332649<br>3332650<br>3332651<br>3332652<br>3332653<br>3332654<br>3332655<br>3332656<br>3332657<br>3332658<br>3332659<br>3332660	core<br>core<br>core<br>core<br>core<br>core<br>core<br>core<br>extended<br>core<br>extended<br>core<br>extended<br>core<br>core<br>core<br>core<br>extended<br>core<br>core<br>core<br>core<br>core<br>core					ENSESTT00000011410<br>ENST00000005286<br>ENST00000345586<br>GENSCAN00000004748	 cdna:known-ccds chromosome:NCBI36:11:60448522:60461206:1 gene:ENSG00000006118 CCDS7997.1<br>cdna:known chromosome:NCBI36:11:60448522:60461206:1 gene:ENSG00000006118<br>cdna:Genscan chromosome:NCBI36:11:60448694:60460955:1
3338293	637389<br>637390<br>637391<br>637392<br>637393<br>637394<br>637395<br>637396<br>637397<br>637398<br>637399<br>637400<br>637401<br>637402<br>637403<br>637404<br>637405<br>637406<br>637407<br>637408<br>637409<br>637410<br>637411<br>637412<br>637413<br>637414<br>637415<br>637416<br>637417<br>637418	3338294<br>3338295<br>3338296<br>3338297<br>3338298<br>3338299<br>3338300<br>3338301<br>3338302<br>3338303<br>3338304<br>3338305<br>3338306<br>3338307<br>3338308<br>3338309<br>3338310<br>3338311<br>3338312<br>3338313<br>3338314<br>3338315<br>3338316<br>3338317<br>3338318<br>3338319<br>3338320<br>3338321<br>3338322<br>3338323	full<br>extended<br>full<br>full<br>full<br>full<br>full<br>extended<br>full<br>extended<br>extended<br>extended<br>full<br>full<br>extended<br>full<br>full<br>full<br>full<br>full<br>full<br>full<br>full<br>full<br>full<br>full<br>full<br>core<br>core<br>core	11	69490351	69713281	+	NM_018043<br>AK097619<br>BC033036<br>ENSESTT00000001686<br>ENSESTT00000001687<br>ENSESTT00000001688<br>ENST00000355303<br>ENST00000356134<br>ENST00000316296<br>GENSCAN00000004033<br>GENSCAN00000033857<br>GENSCAN00000033858	Homo sapiens transmembrane protein 16A (TMEM16A), mRNA.<br>Homo sapiens cDNA FLJ40300 fis, clone TESTI2029131.<br>Homo sapiens transmembrane protein 16A, mRNA (cDNA clone MGC:33580 IMAGE:4823645), complete cds.<br><br><br><br>cdna:known chromosome:NCBI36:11:69602056:69713281:1 gene:ENSG00000131620<br>cdna:known chromosome:NCBI36:11:69602056:69713281:1 gene:ENSG00000131620<br>cdna:known chromosome:NCBI36:11:69609168:69687331:1 gene:ENSG00000131620<br>cdna:Genscan chromosome:NCBI36:11:69602296:69711758:1<br>cdna:Genscan chromosome:NCBI36:11:69490318:69511483:1<br>cdna:Genscan chromosome:NCBI36:11:69553933:69596152:1

	637419	3338324	extended						
	637420	3338325	core						
	637421	3338326	core						
	637422	3338327	core						
	637423	3338328	full						
	637424	3338329	full						
	637425	3338330	full						
	637426	3338331	full						
	637427	3338332	full						
	637428	3338333	extended						
	637429	3338334	full						
	637430	3338335	full						
	637431	3338336	core						
	637432	3338337	core						
	637433	3338338	full						
	637434	3338339	core						
	637435	3338340	extended						
	637436	3338341	core						
	637437	3338342	full						
	637438	3338343	extended						
	637439	3338344	core						
	637440	3338345	full						
	637441	3338346	extended						
	637442	3338347	core						
	637443	3338348	full						
	637444	3338349	core						
	637445	3338350	core						
	637446	3338351	full						
	637447	3338352	core						
	637448	3338353	full						
	637449	3338354	full						
	637450	3338355	core						
	637451	3338356	full						
	637452	3338357	full						
	637453	3338358	extended						
	637454	3338359	extended						
	637455	3338360	core						
	637456	3338361	core						
	637457	3338362	full						
	637458	3338363	core						
	637459	3338364	extended						
		3338365	full						
		3338366	core						
		3338367	core						
		3338368	full						
		3338369	core						
		3338370	extended						
		3338371	extended						
		3338372	core						
		3338373	full						
		3338374	core						
		3338375	core						
		3338376	full						
		3338377	extended						

		3338378<br>3338379<br>3338380<br>3338381<br>3338382<br>3338383<br>3338384<br>3338385<br>3338386<br>3338387<br>3338388<br>3338389<br>3338390<br>3338391	extended<br>core<br>full<br>full<br>extended<br>core<br>core<br>full<br>core<br>core<br>core<br>core<br>core<br>core						
3358201	649631<br>649632<br>649633<br>649634<br>649635<br>649636<br>649637<br>649638<br>649639<br>649640<br>649641<br>649642<br>649643<br>649644<br>649645<br>649646<br>649647<br>649648<br>649649	3358202<br>3358203<br>3358204<br>3358205<br>3358206<br>3358207<br>3358208<br>3358209<br>3358210<br>3358211<br>3358212<br>3358213<br>3358214<br>3358215<br>3358216<br>3358217<br>3358218<br>3358219<br>3358220<br>3358221<br>3358222<br>3358223<br>3358224<br>3358225<br>3358226<br>3358227<br>3358228<br>3358229<br>3358230<br>3358231<br>3358232<br>3358233<br>3358234<br>3358235<br>3358236<br>3358237<br>3358238<br>3358239<br>3358240	core<br>core<br>core<br>core<br>core<br>core<br>core<br>core<br>core<br>core<br>core<br>core<br>core<br>core<br>core<br>core<br>full<br>core<br>core<br>core<br>full<br>core<br>full<br>core<br>core<br>full<br>core<br>core<br>full<br>core<br>core<br>core<br>core<br>core<br>core<br>core<br>core<br>core<br>core	11	606587	616078	-	NM_017717<br>NM_031265<br>NM_031264<br>NM_021924<br>AY358368<br>BC038405<br>BC107063<br>ENSESTT00000022246<br>ENST00000358353<br>ENST00000349570<br>ENST00000326366<br>GENSCAN00000068250	Homo sapiens mucin and cadherin-like (MUCDHL), transcript variant 2, mRNA.<br>Homo sapiens mucin and cadherin-like (MUCDHL), transcript variant 4, mRNA.<br>Homo sapiens mucin and cadherin-like (MUCDHL), transcript variant 3, mRNA.<br>Homo sapiens mucin and cadherin-like (MUCDHL), transcript variant 1, mRNA.<br>Homo sapiens clone DNA102846 MUCDHL (UNQ2781) mRNA, complete cds.<br>Homo sapiens mucin and cadherin-like, mRNA (cDNA clone MGC:34114 IMAGE:5185584), complete cds.<br>Homo sapiens cDNA clone IMAGE:40006352, partial cds.<br><br>cdna:known-ccds chromosome:NCBI36:11:607253:616078:-1 gene:ENSG00000099834 CCDS7707.1<br>cdna:known-ccds chromosome:NCBI36:11:607253:616078:-1 gene:ENSG00000099834 CCDS7708.1<br>cdna:known chromosome:NCBI36:11:606838:614924:-1 gene:ENSG00000099834<br>cdna:Genscan chromosome:NCBI36:11:602645:614902:-1

3385951	666332	3385952	extended	11	88697172	88962417	-	NM_016931	Homo sapiens NADPH oxidase 4 (NOX4), mRNA.
	666333	3385953	extended					BC051371	Homo sapiens NADPH oxidase 4, mRNA (cDNA clone IMAGE:6580820), complete cds.
	666334	3385954	full					BC034780	Homo sapiens mRNA similar to NADPH oxidase 4 (cDNA clone IMAGE:5179443).
	666335	3385955	extended					ENSESTT00000040078	
	666336	3385956	core					ENSESTT00000040079	
	666337	3385957	core					ENST00000263317	cdna:known-ccds chromosome:NCBI36:11:88699163:88864301:-1 gene:ENSG00000086991 CCDS8285.1
	666338	3385958	core					ENST00000343727	cdna:known chromosome:NCBI36:11:88699163:88864301:-1 gene:ENSG00000086991
	666339	3385959	extended					ENST00000375979	cdna:known chromosome:NCBI36:11:88699163:88864301:-1 gene:ENSG00000086991
	666340	3385960	extended					GENSCAN00000050756	cdna:Genscan chromosome:NCBI36:11:88699572:88824658:-1
	666341	3385961	extended						
	666342	3385962	core						
	666343	3385963	extended						
	666344	3385964	extended						
	666345	3385965	full						
	666346	3385966	core						
	666347	3385967	core						
	666348	3385968	full						
	666349	3385969	core						
	666350	3385970	core						
	666351	3385971	core						
	666352	3385972	full						
	666353	3385973	full						
	666354	3385974	full						
	666355	3385975	core						
	666356	3385976	extended						
	666357	3385977	extended						
	666358	3385978	extended						
	666359	3385979	full						
	666360	3385980	extended						
	666361	3385981	extended						
	666362	3385982	core						
	666363	3385983	core						
	666364	3385984	core						
	666365	3385985	core						
	666366	3385986	core						
	666367	3385987	full						
	666368	3385988	core						
	666369	3385989	full						
	666370	3385990	core						
	666371	3385991	core						
	666372	3385992	full						
	666373	3385993	core						
	666374	3385994	full						
	666375	3385995	full						
	666376	3385996	core						
	666377	3385997	full						
	666378	3385998	core						
	666379	3385999	core						
	666380	3386000	extended						
	666381	3386001	full						
	666382	3386002	full						
	666383	3386003	core						
	666384	3386004	core						
	666385	3386005	core						

		3386006<br>3386007<br>3386008<br>3386009<br>3386010<br>3386011<br>3386012<br>3386013<br>3386014<br>3386015<br>3386016	core<br>extended<br>extended<br>full<br>extended<br>full<br>extended<br>extended<br>extended<br>full<br>extended						
3388673	667944<br>667945<br>667946<br>667947<br>667948<br>667949<br>667950	3388674<br>3388675<br>3388676<br>3388677<br>3388678<br>3388679<br>3388680<br>3388681<br>3388682<br>3388683<br>3388684<br>3388685<br>3388686	core<br>core<br>core<br>core<br>core<br>extended<br>full<br>core<br>core<br>core<br>core<br>core<br>core	11	101896483	101906688	-	NM_002423<br>Z11887<br>ENSESTT00000038173<br>ENST00000260227<br>GENSCAN00000041572	Homo sapiens matrix metallopeptidase 7 (matrilysin, uterine) (MMP7), mRNA.<br>H.sapiens PUMP-1 gene encoding PUMP.<br><br>cdna:known-ccds chromosome:NCBI36:11:101896450:101906688:-1 gene:ENSG00000137673 CCDS8317.1<br>cdna:Genscan chromosome:NCBI36:11:101899894:101906641:-1
3388807	668028<br>668029<br>668030<br>668031<br>668032<br>668033<br>668034<br>668035<br>668036<br>668037<br>668038	3388808<br>3388809<br>3388810<br>3388811<br>3388812<br>3388813<br>3388814<br>3388815<br>3388816<br>3388817<br>3388818<br>3388819<br>3388820<br>3388821<br>3388822<br>3388823<br>3388824<br>3388825<br>3388826<br>3388827	core<br>core<br>core<br>full<br>core<br>core<br>core<br>core<br>full<br>core<br>core<br>core<br>core<br>core<br>core<br>full<br>full<br>core<br>core<br>core	11	102165868	102174104	-	NM_002421<br>M15996<br>ENSESTT00000038148<br>ENST00000315274<br>GENSCAN00000001506	Homo sapiens matrix metallopeptidase 1 (interstitial collagenase) (MMP1), mRNA.<br>Human synovial cell collagenase mRNA.<br><br>cdna:known-ccds chromosome:NCBI36:11:102165861:102174099:-1 gene:ENSG00000196611 CCDS8322.1<br>cdna:Genscan chromosome:NCBI36:11:102166353:102174033:-1
3388830	668039<br>668040<br>668041<br>668042<br>668043<br>668044<br>668045<br>668046<br>668047	3388829<br>3388831<br>3388832<br>3388833<br>3388834<br>3388835<br>3388836<br>3388837<br>3388838	full<br>full<br>full<br>full<br>full<br>full<br>full<br>full<br>full	11	102182979	102219563	-	NM_002422<br>ENSESTT00000038141<br>ENSESTT00000038143<br>ENSESTT00000038145<br>ENST00000299855<br>GENSCAN00000016483	Homo sapiens matrix metallopeptidase 3 (stromelysin 1, progelatinase) (MMP3), mRNA.<br><br><br><br>cdna:known-ccds chromosome:NCBI36:11:102211738:102219552:-1 gene:ENSG00000149968 CCDS8323.1<br>cdna:Genscan chromosome:NCBI36:11:102212067:102219487:-1

	668048<br>668049<br>668050<br>668051<br>668052<br>668053<br>668054<br>668055<br>668056<br>668057<br>668058	3388839<br>3388840<br>3388841<br>3388842<br>3388843<br>3388844<br>3388845<br>3388846<br>3388847<br>3388848<br>3388849<br>3388850<br>3388851<br>3388852<br>3388853<br>3388854<br>3388855<br>3388856<br>3388857<br>3388858	core<br>core<br>extended<br>core<br>core<br>full<br>core<br>core<br>core<br>core<br>core<br>core<br>extended<br>core<br>core<br>core<br>core<br>core<br>core<br>extended						
3394315	671451<br>671452<br>671453	3394316<br>3394317<br>3394318<br>3394319<br>3394320<br>3394321<br>3394322<br>3394323	core<br>core<br>core<br>core<br>full<br>extended<br>core<br>core	11	118714862	118716360	-	NM_015645<br>ENST00000360167<br>ENST00000310588<br>ENST00000357470<br>GENSCAN00000016293	Homo sapiens C1q and tumor necrosis factor related protein 5 (C1QTNF5), mRNA.<br>cdna:known-ccds chromosome:NCBI36:11:118714862:118722593:-1 gene:ENSG00000184824 CCDS8421.1<br>cdna:known-ccds chromosome:NCBI36:11:118714862:118716803:-1 gene:ENSG00000184824 CCDS8420.1<br>cdna:novel chromosome:NCBI36:11:118714862:118722593:-1 gene:ENSG00000184824<br>cdna:Genscan chromosome:NCBI36:11:118715251:118729769:-1
3394660	671649<br>671650<br>671651<br>671652<br>671653<br>671654<br>671655<br>671656<br>671657<br>671658<br>671659<br>671660<br>671661<br>671662<br>671663<br>671664<br>671665<br>671666<br>671667<br>671668<br>671669<br>671670<br>671671<br>671672<br>671673	3394661<br>3394662<br>3394663<br>3394664<br>3394665<br>3394666<br>3394667<br>3394668<br>3394669<br>3394670<br>3394671<br>3394672<br>3394673<br>3394674<br>3394675<br>3394676<br>3394677<br>3394678<br>3394679<br>3394680<br>3394681<br>3394682<br>3394683<br>3394684<br>3394685	core<br>core<br>core<br>core<br>core<br>core<br>extended<br>core<br>extended<br>core<br>extended<br>extended<br>extended<br>extended<br>core<br>core<br>extended<br>extended<br>full<br>core<br>extended<br>extended<br>extended<br>full<br>core	11	119487221	119514073	-	NM_012101<br>AF230389<br>AK093404<br>AL832678<br>CR591524<br>CR612298<br>BX648072<br>ENSESTT00000026735<br>ENSESTT00000026736<br>ENST00000350242<br>ENST00000341846<br>GENSCAN00000045646	Homo sapiens tripartite motif-containing 29 (TRIM29), mRNA.<br>Homo sapiens tripartite motif protein TRIM29 beta mRNA, complete cds.<br>Homo sapiens cDNA FLJ36085 fis, clone TESTI2020084.<br>Homo sapiens mRNA; cDNA DKFZp313K2117 (from clone DKFZp313K2117).<br>full-length cDNA clone CS0DI004YC15 of Placenta Cot 25-normalized of Homo sapiens (human).<br>full-length cDNA clone CS0DK004YE16 of HeLa cells Cot 25-normalized of Homo sapiens (human).<br>Homo sapiens mRNA; cDNA DKFZp686I03210 (from clone DKFZp686I03210).<br><br><br>cdna:known-ccds chromosome:NCBI36:11:119487205:119514073:-1 gene:ENSG00000137699 CCDS8429.1<br>cdna:known-ccds chromosome:NCBI36:11:119487205:119513949:-1 gene:ENSG00000137699 CCDS8428.1<br>cdna:Genscan chromosome:NCBI36:11:119488332:119513949:-1

		3394686<br>3394687<br>3394688<br>3394689<br>3394690<br>3394691<br>3394692<br>3394693<br>3394694<br>3394695<br>3394696<br>3394697<br>3394698	core<br>core<br>full<br>core<br>extended<br>extended<br>full<br>full<br>full<br>core<br>core<br>core<br>core						
3399545	674628<br>674629<br>674630<br>674631<br>674632<br>674633<br>674634<br>674635<br>674636<br>674637<br>674638<br>674639<br>674640<br>674641<br>674642<br>674643<br>674644<br>674645<br>674646<br>674647<br>674648<br>674649<br>674650<br>674651<br>674652<br>674653<br>674654<br>674655<br>674656<br>674657<br>674658<br>674659<br>674660<br>674661<br>674662<br>674663<br>674664<br>674665<br>674666<br>674667<br>674668	3399546<br>3399547<br>3399548<br>3399549<br>3399550<br>3399551<br>3399552<br>3399553<br>3399554<br>3399555<br>3399556<br>3399557<br>3399558<br>3399559<br>3399560<br>3399561<br>3399562<br>3399563<br>3399564<br>3399565<br>3399566<br>3399567<br>3399568<br>3399569<br>3399570<br>3399571<br>3399572<br>3399573<br>3399574<br>3399575<br>3399576<br>3399577<br>3399578<br>3399579<br>3399580<br>3399581<br>3399582<br>3399583<br>3399584<br>3399585<br>3399586	full<br>extended<br>extended<br>extended<br>core<br>core<br>extended<br>core<br>full<br>core<br>extended<br>full<br>extended<br>extended<br>extended<br>extended<br>core<br>core<br>core<br>core<br>core<br>core<br>extended<br>core<br>core<br>core<br>core<br>core<br>core<br>extended<br>core<br>core<br>extended<br>core<br>core<br>core<br>extended<br>extended<br>core<br>core<br>full	11	133523648	133600390	-	XM_944757<br>XM_933720<br>NM_015261<br>AK095267<br>CR601575<br>ENSESTT00000013332<br>ENSESTT00000013333<br>ENSESTT00000013334<br>ENSESTT00000013335<br>ENSESTT00000013336<br>ENST00000389503<br>ENST00000389502<br>ENST00000281189<br>GENSCAN00000053600	PREDICTED: Homo sapiens hypothetical protein LOC651550 (LOC651550), mRNA.<br>PREDICTED: Homo sapiens hypothetical protein LOC646578 (LOC646578), mRNA.<br>Homo sapiens KIAA0056 protein (hCAP-D3), mRNA.<br>Homo sapiens cDNA FLJ37948 fis, clone CTONG2009132.<br>full-length cDNA clone CS0CAP004YD09 of Thymus of Homo sapiens (human).<br><br><br><br><br><br>cdna:known chromosome:NCBI36:11:133528049:133599030:-1 gene:ENSG00000151503<br>cdna:known chromosome:NCBI36:11:133528049:133599030:-1 gene:ENSG00000151503<br>cdna:novel chromosome:NCBI36:11:133527547:133599636:-1 gene:ENSG00000151503<br>cdna:Genscan chromosome:NCBI36:11:133528317:133599030:-1

	674669<br>674670<br>674671<br>674672<br>674673<br>674674<br>674675<br>674676	3399587<br>3399588<br>3399589<br>3399590<br>3399591<br>3399592<br>3399593<br>3399594<br>3399595<br>3399596<br>3399597<br>3399598<br>3399599<br>3399600<br>3399601<br>3399602<br>3399603<br>3399604<br>3399605<br>3399606<br>3399607<br>3399608<br>3399609<br>3399610<br>3399611<br>3399612<br>3399613	core<br>core<br>core<br>core<br>core<br>core<br>core<br>core<br>core<br>extended<br>core<br>core<br>core<br>core<br>core<br>core<br>core<br>extended<br>core<br>extended<br>core<br>extended<br>extended<br>extended<br>extended<br>full<br>extended						
3402571	676569<br>676570<br>676571<br>676572<br>676573<br>676574<br>676575<br>676576<br>676577<br>676578<br>676579<br>676580<br>676581<br>676582<br>676583<br>676584<br>676585<br>676586<br>676587<br>676588<br>676589<br>676590<br>676591<br>676592<br>676593<br>676594<br>676595	3402572<br>3402573<br>3402574<br>3402575<br>3402576<br>3402577<br>3402578<br>3402579<br>3402580<br>3402581<br>3402582<br>3402583<br>3402584<br>3402585<br>3402586<br>3402587<br>3402588<br>3402589<br>3402590<br>3402591<br>3402592<br>3402593<br>3402594<br>3402595<br>3402596<br>3402597<br>3402598	core<br>core<br>core<br>core<br>core<br>full<br>extended<br>extended<br>core<br>core<br>core<br>full<br>core<br>core<br>core<br>core<br>core<br>core<br>core<br>core<br>core<br>core<br>core<br>extended<br>extended<br>core<br>core	12	6472816	6511381	+	NM_014865<br>AF086264<br>BC016913<br>ENSESTT00000023951<br>ENSESTT00000023952<br>ENSESTT00000023953<br>ENSESTT00000023954<br>ENSESTT00000023955<br>ENSESTT00000023956<br>ENSESTT00000023957<br>ENSESTT00000023958<br>ENSESTT00000023959<br>ENSESTT00000023960<br>ENST00000315579<br>ENST00000382457<br>GENSCAN00000012788	Homo sapiens chromosome condensation-related SMC-associated protein 1 (CNAP1), mRNA.<br>Homo sapiens full length insert cDNA clone ZD43A10.<br>Homo sapiens chromosome condensation-related SMC-associated protein 1, mRNA (cDNA clone IMAGE:4419375), containing frame-shift errors.<br>cdna:known-ccds chromosome:NCBI36:12:6472806:6511381:1 gene:ENSG00000010292 CCDS8548.1<br>cdna:known chromosome:NCBI36:12:6472806:6511023:1 gene:ENSG00000010292<br>cdna:Genscan chromosome:NCBI36:12:6474538:6517597:1

	676596<br>676597<br>676598<br>676599<br>676600<br>676601<br>676602<br>676603<br>676604<br>676605<br>676606	3402599<br>3402600<br>3402601<br>3402602<br>3402603<br>3402604<br>3402605<br>3402606<br>3402607<br>3402608<br>3402609<br>3402610<br>3402611<br>3402612<br>3402613<br>3402614<br>3402615<br>3402616<br>3402617<br>3402618<br>3402619<br>3402620<br>3402621<br>3402622<br>3402623<br>3402624	core<br>core<br>core<br>full<br>core<br>core<br>core<br>core<br>core<br>core<br>core<br>core<br>core<br>core<br>core<br>core<br>core<br>extended<br>core<br>core<br>core<br>full<br>core<br>core<br>core<br>core						
3406329	678832<br>678833<br>678834<br>678835<br>678836<br>678837<br>678838<br>678839<br>678840<br>678841<br>678842<br>678843<br>678844<br>678845<br>678846<br>678847<br>678848<br>678849<br>678850<br>678851<br>678852<br>678853<br>678854<br>678855<br>678856<br>678857<br>678858<br>678859	3406330<br>3406331<br>3406332<br>3406333<br>3406334<br>3406335<br>3406336<br>3406337<br>3406338<br>3406339<br>3406340<br>3406341<br>3406342<br>3406343<br>3406344<br>3406345<br>3406346<br>3406347<br>3406348<br>3406349<br>3406350<br>3406351<br>3406352<br>3406353<br>3406354<br>3406355<br>3406356<br>3406357	extended<br>core<br>full<br>full<br>extended<br>extended<br>extended<br>extended<br>extended<br>extended<br>extended<br>extended<br>extended<br>extended<br>extended<br>extended<br>extended<br>extended<br>full<br>full<br>full<br>full<br>core<br>extended<br>extended<br>extended<br>core<br>core	12	15366576	15646376	+	NM_030670<br>NM_030671<br>NM_030668<br>NM_030669<br>NM_030667<br>NM_002848<br>AB209729<br>BC035960<br>ENSESTT00000005506<br>ENSESTT00000005507<br>ENSESTT00000005508<br>ENSESTT00000005509<br>ENSESTT00000005510<br>ENSESTT00000005511<br>ENSESTT00000005512<br>ENSESTT00000005513<br>ENSESTT00000005514<br>ENSESTT00000005515<br>ENST00000281171<br>ENST00000348962<br>GENSCAN00000036877<br>GENSCAN00000036882	Homo sapiens protein tyrosine phosphatase, receptor type, O (PTPRO), transcript variant 6, mRNA.<br>Homo sapiens protein tyrosine phosphatase, receptor type, O (PTPRO), transcript variant 5, mRNA.<br>Homo sapiens protein tyrosine phosphatase, receptor type, O (PTPRO), transcript variant 4, mRNA.<br>Homo sapiens protein tyrosine phosphatase, receptor type, O (PTPRO), transcript variant 3, mRNA.<br>Homo sapiens protein tyrosine phosphatase, receptor type, O (PTPRO), transcript variant 1, mRNA.<br>Homo sapiens protein tyrosine phosphatase, receptor type, O (PTPRO), transcript variant 2, mRNA.<br>Homo sapiens mRNA for receptor-type protein tyrosine phosphatase O isoform a precursor variant protein.<br>Homo sapiens protein tyrosine phosphatase, receptor type, O, mRNA (cDNA clone IMAGE:4610767), complete cds.<br><br><br><br><br><br><br><br><br><br><br>cdna:known-ccds chromosome:NCBI36:12:15366754:15641602:1 gene:ENSG00000151490 CCDS8675.1<br>cdna:known-ccds chromosome:NCBI36:12:15366754:15641602:1 gene:ENSG00000151490 CCDS8674.1<br>cdna:Genscan chromosome:NCBI36:12:15516848:15528568:1<br>cdna:Genscan chromosome:NCBI36:12:15483904:15484605:1

	678860<br>678861<br>678862<br>678863<br>678864<br>678865<br>678866<br>678867<br>678868<br>678869<br>678870<br>678871<br>678872<br>678873<br>678874<br>678875<br>678876<br>678877<br>678878<br>678879<br>678880<br>678881<br>678882<br>678883<br>678884<br>678885<br>678886<br>678887<br>678888	3406358<br>3406359<br>3406360<br>3406361<br>3406362<br>3406363<br>3406364<br>3406365<br>3406366<br>3406367<br>3406368<br>3406369<br>3406370<br>3406371<br>3406372<br>3406373<br>3406374<br>3406375<br>3406376<br>3406377<br>3406378<br>3406379<br>3406380<br>3406381<br>3406382<br>3406383<br>3406384<br>3406385<br>3406386<br>3406387<br>3406388<br>3406389<br>3406390<br>3406391<br>3406392<br>3406393<br>3406394<br>3406395<br>3406396<br>3406397<br>3406398<br>3406399<br>3406400<br>3406401<br>3406402<br>3406403<br>3406404<br>3406405<br>3406406	core<br>core<br>extended<br>core<br>extended<br>core<br>core<br>core<br>core<br>core<br>core<br>core<br>full<br>core<br>core<br>core<br>full<br>core<br>full<br>core<br>core<br>core<br>full<br>core<br>core<br>full<br>full<br>core<br>core<br>core<br>core<br>extended<br>full<br>core<br>extended<br>core<br>full<br>full<br>full<br>core<br>core<br>core<br>core<br>core<br>core<br>core<br>extended<br>full<br>extended						
3409127	680642<br>680643<br>680644<br>680645<br>680646	3409128<br>3409129<br>3409130<br>3409131<br>3409132	core<br>core<br>core<br>core<br>full	12	27377064	27469489	+	NM_020183<br>AF231338<br>AF256215<br>ENSESTT00000014715<br>ENSESTT00000014716	Homo sapiens aryl hydrocarbon receptor nuclear translocator-like 2 (ARNTL2), mRNA.<br>Homo sapiens bHLH-PAS transcription factor MOP9 (MOP9) mRNA, long form, complete cds, alternatively spliced.<br>Homo sapiens cycle-like factor CLIF mRNA, complete cds.

	680647<br>680648<br>680649<br>680650<br>680651<br>680652<br>680653<br>680654<br>680655<br>680656<br>680657<br>680658<br>680659<br>680660<br>680661<br>680662<br>680663<br>680664<br>680665<br>680666<br>680667<br>680668<br>680669<br>680670<br>680671<br>680672	3409133<br>3409134<br>3409135<br>3409136<br>3409137<br>3409138<br>3409139<br>3409140<br>3409141<br>3409142<br>3409143<br>3409144<br>3409145<br>3409146<br>3409147<br>3409148<br>3409149<br>3409150<br>3409151<br>3409152<br>3409153<br>3409154<br>3409155<br>3409156<br>3409157<br>3409158<br>3409159<br>3409160<br>3409161<br>3409162<br>3409163<br>3409164<br>3409165<br>3409166<br>3409167<br>3409168<br>3409169<br>3409170<br>3409171<br>3409172	full<br>full<br>full<br>extended<br>core<br>core<br>core<br>core<br>core<br>extended<br>full<br>extended<br>core<br>extended<br>extended<br>full<br>full<br>full<br>core<br>core<br>core<br>core<br>core<br>full<br>full<br>core<br>core<br>core<br>core<br>core<br>core<br>core<br>extended<br>core<br>core<br>full<br>core<br>core<br>extended<br>extended					ENSESTT00000014717<br>ENST00000266503<br>ENST00000311001<br>ENST00000261178<br>GENSCAN00000017633<br>GENSCAN00000068194	 cdna:known-ccds chromosome:NCBI36:12:27377255:27464733:1 gene:ENSG00000029153 CCDS8712.1<br>cdna:known chromosome:NCBI36:12:27377255:27464733:1 gene:ENSG00000029153<br>cdna:known chromosome:NCBI36:12:27377255:27464733:1 gene:ENSG00000029153<br>cdna:Genscan chromosome:NCBI36:12:27377065:27447741:1<br>cdna:Genscan chromosome:NCBI36:12:27462288:27464732:1
3414739	684170<br>684171<br>684172<br>684173<br>684174<br>684175<br>684176	3414740<br>3414741<br>3414742<br>3414743<br>3414744<br>3414745<br>3414746<br>3414747<br>3414748<br>3414749<br>3414750<br>3414751<br>3414752<br>3414753	extended<br>core<br>core<br>extended<br>extended<br>core<br>core<br>core<br>core<br>core<br>core<br>core<br>core<br>core	12	49604734	49612550	+	NM_014033<br>AK225689<br>AF113007<br>ENSESTT00000015926<br>ENST00000332160<br>GENSCAN00000027365	Homo sapiens methyltransferase like 7A (METTL7A), mRNA.<br>Homo sapiens mRNA for hypothetical protein LOC25840 variant, clone: SYN03575.<br>Homo sapiens PRO0066 mRNA, complete cds.<br><br>cdna:known-ccds chromosome:NCBI36:12:49605033:49612557:1 gene:ENSG00000185432 CCDS8804.1<br>cdna:Genscan chromosome:NCBI36:12:49605089:49641152:1

3421177	687907<br>687908<br>687909<br>687910<br>687911<br>687912<br>687913<br>687914<br>687915<br>687916<br>687917<br>687918<br>687919<br>687920<br>687921<br>687922<br>687923<br>687924<br>687925<br>687926<br>687927<br>687928<br>687929<br>687930<br>687931<br>687932<br>687933<br>687934<br>687935<br>687936<br>687937<br>687938<br>687939<br>687940<br>687941<br>687942	3421178<br>3421179<br>3421180<br>3421181<br>3421182<br>3421183<br>3421184<br>3421185<br>3421186<br>3421187<br>3421188<br>3421189<br>3421190<br>3421191<br>3421192<br>3421193<br>3421194<br>3421195<br>3421196<br>3421197<br>3421198<br>3421199<br>3421200<br>3421201<br>3421202<br>3421203<br>3421204<br>3421205<br>3421206<br>3421207<br>3421208<br>3421209<br>3421210<br>3421211<br>3421212<br>3421213<br>3421214<br>3421215<br>3421216<br>3421217<br>3421218<br>3421219<br>3421220<br>3421221<br>3421222<br>3421223<br>3421224	core<br>extended<br>core<br>full<br>core<br>extended<br>extended<br>core<br>core<br>extended<br>extended<br>extended<br>core<br>core<br>core<br>full<br>core<br>extended<br>core<br>extended<br>core<br>extended<br>extended<br>core<br>core<br>full<br>core<br>extended<br>core<br>core<br>core<br>core<br>core<br>core<br>core<br>core<br>core<br>extended<br>core<br>core<br>core<br>extended<br>core<br>extended<br>core<br>core<br>extended	12	67366998	67423025	+	NM_020401<br>CR616821<br>ENSESTT00000039967<br>ENSESTT00000039968<br>ENSESTT00000039969<br>ENSESTT00000039970<br>ENSESTT00000039971<br>ENSESTT00000039972<br>ENSESTT00000039973<br>ENSESTT00000039974<br>ENST00000229179<br>ENST00000378905<br>GENSCAN00000054844<br>GENSCAN00000054848	Homo sapiens nucleoporin 107kDa (NUP107), mRNA.<br>full-length cDNA clone CS0DI015YD13 of Placenta Cot 25-normalized of Homo sapiens (human).<br><br><br><br><br><br><br><br><br>cdna:known-ccds chromosome:NCBI36:12:67366998:67422739:1 gene:ENSG00000111581 CCDS8985.1<br>cdna:known chromosome:NCBI36:12:67366998:67422739:1 gene:ENSG00000111581<br>cdna:Genscan chromosome:NCBI36:12:67370678:67399531:1<br>cdna:Genscan chromosome:NCBI36:12:67401876:67422509:1
3422144	688482<br>688483<br>688484<br>688485<br>688486<br>688487<br>688488	3422145<br>3422146<br>3422147<br>3422148<br>3422149<br>3422150<br>3422151	extended<br>core<br>full<br>extended<br>extended<br>full<br>extended	12	70119948	70266356	+	NM_003667<br>AK075399<br>ENSESTT00000044682<br>ENSESTT00000044683<br>ENST00000266674<br>GENSCAN00000063089<br>GENSCAN00000042728	Homo sapiens leucine-rich repeat-containing G protein-coupled receptor 5 (LGR5), mRNA.<br>Homo sapiens cDNA PSEC0089 fis, clone PLACE1001148, highly similar to Homo sapiens orphan G protein-coupled receptor HG38 mRNA.<br><br><br>cdna:known-ccds chromosome:NCBI36:12:70120031:70264781:1 gene:ENSG00000139292 CCDS9000.1<br>cdna:Genscan chromosome:NCBI36:12:70204453:70264781:1

	688489<br>688490<br>688491<br>688492<br>688493<br>688494<br>688495<br>688496<br>688497<br>688498<br>688499<br>688500<br>688501<br>688502<br>688503<br>688504<br>688505<br>688506<br>688507<br>688508<br>688509<br>688510<br>688511<br>688512<br>688513<br>688514<br>688515<br>688516<br>688517<br>688518<br>688519<br>688520<br>688521<br>688522<br>688523<br>688524	3422152<br>3422153<br>3422154<br>3422155<br>3422156<br>3422157<br>3422158<br>3422159<br>3422160<br>3422161<br>3422162<br>3422163<br>3422164<br>3422165<br>3422166<br>3422167<br>3422168<br>3422169<br>3422170<br>3422171<br>3422172<br>3422173<br>3422174<br>3422175<br>3422176<br>3422177<br>3422178<br>3422179<br>3422180<br>3422181<br>3422182<br>3422183<br>3422184<br>3422185<br>3422186<br>3422187<br>3422188<br>3422189<br>3422190<br>3422191<br>3422192<br>3422193<br>3422194<br>3422195<br>3422196<br>3422197<br>3422198<br>3422199<br>3422200<br>3422201<br>3422202	extended<br>full<br>full<br>full<br>full<br>full<br>extended<br>extended<br>full<br>full<br>core<br>full<br>extended<br>extended<br>core<br>core<br>full<br>full<br>extended<br>extended<br>extended<br>full<br>full<br>core<br>core<br>core<br>extended<br>core<br>core<br>core<br>core<br>extended<br>extended<br>extended<br>extended<br>extended<br>extended<br>core<br>extended<br>core<br>extended<br>extended<br>core<br>core<br>core<br>core<br>core<br>core<br>core<br>core<br>extended					GENSCAN00000042733	cdna:Genscan chromosome:NCBI36:12:70184403:70184864:1<br>cdna:Genscan chromosome:NCBI36:12:70149458:70149712:1
3428447	692447<br>692448<br>692449	3428448<br>3428449<br>3428450	full<br>full<br>full	12	100164755	100304515	+	NM_014503<br>XM_936222<br>AK000333	Homo sapiens UTP20, small subunit (SSU) processome component, homolog (yeast) (UTP20), mRNA.<br>PREDICTED: Homo sapiens similar to Down-regulated in metastasis protein (Key-1A6 protein) (Novel nucleolar protein 73) (NNP73) (LOC653877), mRNA.

	692450	3428451	full					ENSESTT00000044734	Homo sapiens cDNA FLJ20326 fis, clone HEP09907, highly similar to AJ006778 Homo sapiens mRNA for DRIM protein.
	692451	3428452	full					ENSESTT00000044735	
	692452	3428453	core					ENSESTT00000044736	
	692453	3428454	core					ENSESTT00000044737	
	692454	3428455	core					ENSESTT00000044738	
	692455	3428456	core					ENSESTT00000044739	
	692456	3428457	core					ENSESTT00000044740	
	692457	3428458	core					ENSESTT00000044741	
	692458	3428459	extended					ENSESTT00000044742	
	692459	3428460	core					ENST00000261637	
	692460	3428461	extended						cdna:known-ccds chromosome:NCBI36:12:100198048:100304524:1 gene:ENSG00000120800 CCDS9081.1
	692461	3428462	core						
	692462	3428463	extended						
	692463	3428464	core						
	692464	3428465	core						
	692465	3428466	extended						
	692466	3428467	core						
	692467	3428468	core						
	692468	3428469	core						
	692469	3428470	core						
	692470	3428471	extended						
	692471	3428472	full						
	692472	3428473	core						
	692473	3428474	core						
	692474	3428475	core						
	692475	3428476	core						
	692476	3428477	core						
	692477	3428478	full						
	692478	3428479	core						
	692479	3428480	full						
	692480	3428481	full						
	692481	3428482	core						
	692482	3428483	core						
	692483	3428484	full						
	692484	3428485	core						
	692485	3428486	extended						
	692486	3428487	extended						
	692487	3428488	core						
	692488	3428489	core						
	692489	3428490	core						
	692490	3428491	extended						
	692491	3428492	core						
	692492	3428493	extended						
	692493	3428494	core						
	692494	3428495	extended						
	692495	3428496	core						
	692496	3428497	extended						
	692497	3428498	core						
	692498	3428499	core						
	692499	3428500	core						
	692500	3428501	core						
	692501	3428502	core						
	692502	3428503	core						
	692503	3428504	core						

	692504<br>692505<br>692506<br>692507<br>692508<br>692509<br>692510<br>692511<br>692512<br>692513<br>692514<br>692515<br>692516<br>692517<br>692518<br>692519<br>692520<br>692521<br>692522<br>692523<br>692524	3428505<br>3428506<br>3428507<br>3428508<br>3428509<br>3428510<br>3428511<br>3428512<br>3428513<br>3428514<br>3428515<br>3428516<br>3428517<br>3428518<br>3428519<br>3428520<br>3428521<br>3428522<br>3428523<br>3428524<br>3428525<br>3428526<br>3428527<br>3428528<br>3428529<br>3428530<br>3428531<br>3428532<br>3428533<br>3428534<br>3428535<br>3428536<br>3428537<br>3428538<br>3428539<br>3428540<br>3428541<br>3428542<br>3428543<br>3428544<br>3428545<br>3428546<br>3428547	core<br>core<br>core<br>core<br>core<br>core<br>full<br>core<br>core<br>full<br>core<br>core<br>extended<br>core<br>core<br>core<br>core<br>core<br>core<br>full<br>core<br>core<br>core<br>core<br>core<br>core<br>core<br>core<br>core<br>core<br>core<br>core<br>extended<br>core<br>core<br>core<br>extended<br>extended<br>core<br>extended<br>core<br>core<br>core						
3428845	692709<br>692710<br>692711<br>692712<br>692713<br>692714<br>692715<br>692716<br>692717<br>692718<br>692719	3428846<br>3428847<br>3428848<br>3428849<br>3428850<br>3428851<br>3428852<br>3428853<br>3428854<br>3428855<br>3428856	extended<br>extended<br>extended<br>core<br>core<br>core<br>core<br>core<br>core<br>core<br>extended	12	101038106	101115421	+	NM_017915<br>AF274940<br>BC018903<br>BC014313<br>ENSESTT00000002234<br>ENSESTT00000002235<br>ENSESTT00000002237<br>ENSESTT00000002239<br>ENSESTT00000002241<br>ENST00000327680<br>ENST00000358383	Homo sapiens chromosome 12 open reading frame 48 (C12orf48), mRNA.<br>Homo sapiens PNAS-15 mRNA, complete cds.<br>Homo sapiens chromosome 12 open reading frame 48, mRNA (cDNA clone IMAGE:3951688), complete cds.<br>Homo sapiens chromosome 12 open reading frame 48, mRNA (cDNA clone IMAGE:3681336), with apparent retained intron.<br><br><br><br><br><br>cdna:known-ccds chromosome:NCBI36:12:101038155:101115421:1 gene:ENSG00000185480 CCDS9090.1<br>cdna:known chromosome:NCBI36:12:101038155:101115421:1 gene:ENSG00000185480

	692720<br>692721<br>692722<br>692723<br>692724<br>692725<br>692726<br>692727	3428857<br>3428858<br>3428859<br>3428860<br>3428861<br>3428862<br>3428863<br>3428864<br>3428865<br>3428866<br>3428867<br>3428868<br>3428869<br>3428870<br>3428871<br>3428872<br>3428873<br>3428874<br>3428875<br>3428876<br>3428877<br>3428878	extended<br>extended<br>extended<br>core<br>core<br>extended<br>extended<br>core<br>core<br>core<br>core<br>core<br>core<br>core<br>core<br>full<br>core<br>core<br>core<br>core<br>extended<br>core					ENST00000378128	cdna:known chromosome:NCBI36:12:101038155:101115421:1 gene:ENSG00000185480
3434012	696025<br>696026<br>696027<br>696028<br>696029<br>696030<br>696031<br>696032<br>696033<br>696034<br>696035<br>696036<br>696037<br>696038<br>696039<br>696040<br>696041<br>696042<br>696043	3434013<br>3434014<br>3434015<br>3434016<br>3434017<br>3434018<br>3434019<br>3434020<br>3434021<br>3434022<br>3434023<br>3434024<br>3434025<br>3434026<br>3434027<br>3434028<br>3434029<br>3434030<br>3434031<br>3434032<br>3434033<br>3434034<br>3434035<br>3434036<br>3434037<br>3434038<br>3434039<br>3434040<br>3434041<br>3434042<br>3434043<br>3434044	extended<br>core<br>core<br>extended<br>extended<br>extended<br>core<br>extended<br>extended<br>core<br>core<br>extended<br>extended<br>extended<br>extended<br>extended<br>extended<br>extended<br>extended<br>full<br>full<br>full<br>full<br>full<br>extended<br>full<br>full<br>extended<br>full<br>full<br>full<br>full	12	118096134	118180587	+	NM_014365<br>AF133207<br>AK001825<br>ENSESTT00000013225<br>ENSESTT00000025107<br>ENST00000281938<br>ENST00000333189<br>GENSCAN00000062642<br>GENSCAN00000022980<br>GENSCAN00000034243<br>GENSCAN00000053023<br>GENSCAN00000053026<br>GENSCAN00000053025	Homo sapiens heat shock 22kDa protein 8 (HSPB8), mRNA.<br>Homo sapiens protein kinase (H11) mRNA, complete cds.<br>Homo sapiens cDNA FLJ10963 fis, clone PLACE1000716.<br><br><br>cdna:known-ccds chromosome:NCBI36:12:118100978:118116933:1 gene:ENSG00000152137 CCDS9189.1<br>cdna:novel chromosome:NCBI36:5:84881159:84881524:1 gene:ENSG00000184184<br>cdna:Genscan chromosome:NCBI36:6:111351462:111351863:1<br>cdna:Genscan chromosome:NCBI36:5:84881165:84881530:1<br>cdna:Genscan chromosome:NCBI36:7:98832241:98840662:-1<br>cdna:Genscan chromosome:NCBI36:12:118101501:118116046:1<br>cdna:Genscan chromosome:NCBI36:12:118117038:118147043:1<br>cdna:Genscan chromosome:NCBI36:12:118174793:118180587:1

3442641	701303	3442642	full	12	7385475	7488015	-	XM_926050	PREDICTED: Homo sapiens similar to M-phase phosphoprotein, mpp8, transcript variant 1 (LOC642333), mRNA.
	701304	3442643	extended					XM_930918	PREDICTED: Homo sapiens similar to M-phase phosphoprotein, mpp8, transcript variant 2 (LOC642333), mRNA.
	701305	3442644	extended					XM_939884	PREDICTED: Homo sapiens similar to M-phase phosphoprotein, mpp8, transcript variant 1 (LOC648113), mRNA.
	701306	3442645	extended					XM_944367	PREDICTED: Homo sapiens similar to M-phase phosphoprotein, mpp8, transcript variant 2 (LOC648113), mRNA.
	701307	3442646	extended					NM_174941	Homo sapiens CD163 molecule-like 1 (CD163L1), mRNA.
	701308	3442647	core					AY358856	Homo sapiens clone DNA176911 scavenger receptor hlg (UNQ6434) mRNA, complete cds.
	701309	3442648	extended					AL832864	Homo sapiens mRNA; cDNA DKFZp667L062 (from clone DKFZp667L062).
	701310	3442649	core					ENSESTT00000002593	
	701311	3442650	extended					ENSESTT00000006077	
	701312	3442651	core					ENSESTT00000006078	
	701313	3442652	extended					ENSESTT00000006079	
	701314	3442653	extended					ENST00000313599	cdna:known-ccds chromosome:NCBI36:12:7398826:7488015:-1 gene:ENSG00000177675 CCDS8577.1
	701315	3442654	core					GENSCAN00000000489	cdna:Genscan chromosome:NCBI36:12:7385475:7417300:-1
	701316	3442655	full					GENSCAN00000063546	cdna:Genscan chromosome:NCBI36:12:7476521:7483851:-1
	701317	3442656	extended						
	701318	3442657	core						
	701319	3442658	full						
	701320	3442659	core						
	701321	3442660	extended						
	701322	3442661	extended						
	701323	3442662	core						
	701324	3442663	core						
	701325	3442664	full						
	701326	3442665	core						
	701327	3442666	core						
	701328	3442667	core						
	701329	3442668	core						
	701330	3442669	core						
	701331	3442670	core						
	701332	3442671	core						
	701333	3442672	core						
		3442673	core						
		3442674	core						
		3442675	core						
		3442676	core						
		3442677	full						
		3442678	full						
		3442679	core						
		3442680	full						
		3442681	core						
		3442682	core						
		3442683	core						
		3442684	full						
		3442685	full						
		3442686	core						
		3442687	core						
		3442688	core						
		3442689	full						
		3442690	full						
		3442691	extended						
		3442692	extended						
		3442693	extended						
		3442694	extended						
		3442695	extended						

		3442696<br>3442697<br>3442698<br>3442699<br>3442700<br>3442701	extended<br>extended<br>full<br>core<br>core<br>core						
3454892	708725<br>708726<br>708727<br>708728<br>708729<br>708730<br>708731<br>708732<br>708733<br>708734<br>708735<br>708736<br>708737<br>708738<br>708739<br>708740<br>708741<br>708742<br>708743<br>708744<br>708745	3454893<br>3454894<br>3454895<br>3454896<br>3454897<br>3454898<br>3454899<br>3454900<br>3454901<br>3454902<br>3454903<br>3454904<br>3454905<br>3454906<br>3454907<br>3454908<br>3454909<br>3454910<br>3454911<br>3454912<br>3454913<br>3454914<br>3454915<br>3454916<br>3454917<br>3454918<br>3454919<br>3454920<br>3454921<br>3454922<br>3454923<br>3454924<br>3454925<br>3454926	extended<br>extended<br>extended<br>core<br>core<br>core<br>core<br>core<br>core<br>extended<br>extended<br>core<br>core<br>full<br>core<br>full<br>full<br>core<br>core<br>core<br>extended<br>extended<br>extended<br>extended<br>extended<br>full<br>full<br>full<br>core<br>extended<br>extended<br>core<br>full<br>extended	12	50031929	50071677	-	NM_007210<br>NM_001971<br>AK074658<br>AK092496<br>BC035822<br>ENSESTT00000007645<br>ENSESTT00000007646<br>ENST00000293636<br>ENST00000356317<br>GENSCAN00000050356<br>GENSCAN00000050354	Homo sapiens UDP-N-acetyl-alpha-D-galactosamine:polypeptide N-acetylgalactosaminyltransferase 6 (GalNAc-T6) (GALNT6), mRNA.<br>Homo sapiens elastase 1, pancreatic (ELA1), mRNA.<br>Homo sapiens cDNA FLJ90177 fis, clone MAMMA1000591, highly similar to UDP-N-acetyl-alpha-D-galactosamine:polypeptide N-acetylgalactosaminyltransferase 6.<br>Homo sapiens cDNA FLJ35177 fis, clone PLACE6013885, highly similar to Homo sapiens mRNA for UDP-GalNAc:polypeptide N-acetylgalactosaminyltransferase.<br>Homo sapiens UDP-N-acetyl-alpha-D-galactosamine:polypeptide N-acetylgalactosaminyltransferase 6 (GalNAc-T6), mRNA (cDNA clone MGC:46032 IMAGE:5552059), complete cds.<br><br><br>cdna:known-ccds chromosome:NCBI36:12:50008494:50026730:-1 gene:ENSG00000139610 CCDS8812.1<br>cdna:known-ccds chromosome:NCBI36:12:50033954:50071464:-1 gene:ENSG00000139629 CCDS8813.1<br>cdna:Genscan chromosome:NCBI36:12:50008628:50059808:-1<br>cdna:Genscan chromosome:NCBI36:12:50068284:50071481:-1
3457101	709977<br>709978<br>709979<br>709980<br>709981<br>709982<br>709983<br>709984<br>709985<br>709986<br>709987<br>709988<br>709989	3457102<br>3457103<br>3457104<br>3457105<br>3457106<br>3457107<br>3457108<br>3457109<br>3457110<br>3457111<br>3457112<br>3457113<br>3457114	core<br>core<br>extended<br>full<br>extended<br>full<br>core<br>core<br>core<br>extended<br>core<br>core<br>core	12	54364623	54392442	-	NM_002206<br>AF034833<br>AY358882<br>ENSESTT00000043163<br>ENSESTT00000043164<br>ENSESTT00000043165<br>ENSESTT00000043166<br>ENSESTT00000043167<br>ENSESTT00000043168<br>ENST00000257879<br>ENST00000257880<br>ENST00000347027<br>ENST00000353687	Homo sapiens integrin, alpha 7 (ITGA7), mRNA.<br>Homo sapiens integrin alpha 7A subunit mRNA, partial cds.<br>Homo sapiens clone DNA55737 ITGA7 (UNQ406) mRNA, complete cds.<br><br><br><br><br><br><br>cdna:known-ccds chromosome:NCBI36:12:54364640:54387949:-1 gene:ENSG00000135424 CCDS8888.1<br>cdna:known chromosome:NCBI36:12:54364640:54387752:-1 gene:ENSG00000135424<br>cdna:known chromosome:NCBI36:12:54364640:54387752:-1 gene:ENSG00000135424<br>cdna:known chromosome:NCBI36:12:54364640:54387752:-1 gene:ENSG00000135424

	709990<br>709991<br>709992<br>709993<br>709994<br>709995<br>709996<br>709997<br>709998<br>709999<br>710000<br>710001<br>710002<br>710003<br>710004<br>710005<br>710006<br>710007<br>710008<br>710009<br>710010	3457115<br>3457116<br>3457117<br>3457118<br>3457119<br>3457120<br>3457121<br>3457122<br>3457123<br>3457124<br>3457125<br>3457126<br>3457127<br>3457128<br>3457129<br>3457130<br>3457131<br>3457132<br>3457133<br>3457134<br>3457135<br>3457136<br>3457137<br>3457138<br>3457139<br>3457140<br>3457141<br>3457142<br>3457143<br>3457144<br>3457145<br>3457146<br>3457147<br>3457148<br>3457149<br>3457150<br>3457151<br>3457152<br>3457153<br>3457154<br>3457155<br>3457156	extended<br>core<br>core<br>core<br>core<br>extended<br>extended<br>core<br>extended<br>extended<br>core<br>full<br>core<br>extended<br>core<br>core<br>full<br>core<br>core<br>core<br>core<br>extended<br>core<br>core<br>core<br>extended<br>core<br>extended<br>core<br>extended<br>extended<br>core<br>extended<br>core<br>full<br>core<br>full<br>core<br>core<br>core<br>extended<br>extended					GENSCAN00000028867	cdna:Genscan chromosome:NCBI36:12:54365109:54387733:-1
3458248	710611<br>710612<br>710613<br>710614<br>710615<br>710616<br>710617<br>710618<br>710619<br>710620<br>710621<br>710622	3458249<br>3458250<br>3458251<br>3458252<br>3458253<br>3458254<br>3458255<br>3458256<br>3458257<br>3458258<br>3458259<br>3458260	full<br>core<br>core<br>core<br>core<br>extended<br>core<br>extended<br>extended<br>core<br>core<br>extended	12	55707002	55730157	-	NM_005379<br>ENSESTT00000030344<br>ENSESTT00000030345<br>ENSESTT00000030346<br>ENSESTT00000030347<br>ENST00000300119<br>ENST00000379401<br>GENSCAN00000003677	Homo sapiens myosin IA (MYO1A), mRNA.<br><br><br><br><br>cdna:known-ccds chromosome:NCBI36:12:55708569:55730160:-1 gene:ENSG00000166866 CCDS8929.1<br>cdna:known chromosome:NCBI36:12:55708569:55730160:-1 gene:ENSG00000166866<br>cdna:Genscan chromosome:NCBI36:12:55692430:55728374:-1

	710623<br>710624<br>710625<br>710626<br>710627<br>710628<br>710629<br>710630<br>710631<br>710632<br>710633<br>710634<br>710635<br>710636<br>710637<br>710638<br>710639	3458261<br>3458262<br>3458263<br>3458264<br>3458265<br>3458266<br>3458267<br>3458268<br>3458269<br>3458270<br>3458271<br>3458272<br>3458273<br>3458274<br>3458275<br>3458276<br>3458277<br>3458278<br>3458279<br>3458280<br>3458281<br>3458282<br>3458283<br>3458284<br>3458285<br>3458286<br>3458287<br>3458288<br>3458289<br>3458290<br>3458291	core<br>core<br>core<br>core<br>core<br>core<br>core<br>core<br>core<br>core<br>core<br>extended<br>core<br>core<br>full<br>core<br>core<br>core<br>core<br>core<br>core<br>full<br>core<br>core<br>core<br>core<br>core<br>full<br>core<br>extended<br>extended						
3472225	719376<br>719377<br>719378<br>719379<br>719380<br>719381<br>719382<br>719383<br>719384<br>719385<br>719386<br>719387<br>719388<br>719389<br>719390<br>719391<br>719392<br>719393<br>719394<br>719395<br>719396<br>719397<br>719398	3472226<br>3472227<br>3472228<br>3472229<br>3472230<br>3472231<br>3472232<br>3472233<br>3472234<br>3472235<br>3472236<br>3472237<br>3472238<br>3472239<br>3472240<br>3472241<br>3472242<br>3472243<br>3472244<br>3472245<br>3472246<br>3472247<br>3472248	core<br>core<br>core<br>core<br>full<br>core<br>extended<br>full<br>core<br>extended<br>extended<br>extended<br>core<br>core<br>extended<br>core<br>core<br>core<br>full<br>core<br>core<br>core<br>core	12	112079361	112107698	-	NM_024072<br>ENSESTT00000016189<br>ENST00000314045<br>ENST00000306014<br>GENSCAN00000025313	Homo sapiens DEAD (Asp-Glu-Ala-Asp) box polypeptide 54 (DDX54), mRNA.<br><br>cdna:known chromosome:NCBI36:12:112079369:112107667:-1 gene:ENSG00000123064<br>cdna:known chromosome:NCBI36:12:112081065:112107667:-1 gene:ENSG00000123064<br>cdna:Genscan chromosome:NCBI36:12:112081065:112107639:-1

	719399<br>719400<br>719401<br>719402	3472249<br>3472250<br>3472251<br>3472252<br>3472253<br>3472254<br>3472255<br>3472256<br>3472257<br>3472258<br>3472259<br>3472260<br>3472261<br>3472262<br>3472263<br>3472264<br>3472265<br>3472266<br>3472267<br>3472268	extended<br>core<br>core<br>core<br>full<br>core<br>core<br>core<br>extended<br>full<br>core<br>extended<br>core<br>core<br>core<br>core<br>core<br>core<br>core<br>extended						
3474104	720635<br>720636<br>720637<br>720638<br>720639<br>720640<br>720641<br>720642<br>720643<br>720644<br>720645<br>720646<br>720647<br>720648<br>720649<br>720650<br>720651<br>720652<br>720653<br>720654<br>720655<br>720656<br>720657<br>720658<br>720659<br>720660<br>720661<br>720662<br>720663<br>720664<br>720665<br>720666<br>720667<br>720668	3474105<br>3474106<br>3474107<br>3474108<br>3474109<br>3474110<br>3474111<br>3474112<br>3474113<br>3474114<br>3474115<br>3474116<br>3474117<br>3474118<br>3474119<br>3474120<br>3474121<br>3474122<br>3474123<br>3474124<br>3474125<br>3474126<br>3474127<br>3474128<br>3474129<br>3474130<br>3474131<br>3474132<br>3474133<br>3474134<br>3474135<br>3474136<br>3474137<br>3474138	full<br>extended<br>core<br>core<br>core<br>core<br>core<br>core<br>full<br>extended<br>core<br>core<br>core<br>core<br>core<br>extended<br>core<br>core<br>core<br>extended<br>core<br>core<br>full<br>extended<br>full<br>extended<br>extended<br>extended<br>core<br>core<br>core<br>full<br>core<br>core	12	118603215	118832243	-	NM_007174<br>AB023166<br>AK123136<br>ENSESTT00000028292<br>ENSESTT00000028294<br>ENSESTT00000028296<br>ENSESTT00000028298<br>ENSESTT00000028303<br>ENSESTT00000028306<br>ENSESTT00000028307<br>ENSESTT00000028308<br>ENSESTT00000028311<br>ENSESTT00000028312<br>ENST00000261833<br>GENSCAN00000034248	Homo sapiens citron (rho-interacting, serine/threonine kinase 21) (CIT), mRNA.<br>Homo sapiens mRNA for KIAA0949 protein, partial cds.<br>Homo sapiens cDNA FLJ41141 fis, clone BRACE2035485, moderately similar to Mus musculus Citron-K kinase mRNA.<br><br><br><br><br><br><br><br><br><br><br>cdna:known-ccds chromosome:NCBI36:12:118607981:118799475:-1 gene:ENSG00000122966 CCDS9192.1<br>cdna:Genscan chromosome:NCBI36:12:118603215:118683242:-1

	720669	3474139	core						
	720670	3474140	core						
	720671	3474141	core						
	720672	3474142	core						
	720673	3474143	core						
	720674	3474144	core						
	720675	3474145	core						
	720676	3474146	extended						
	720677	3474147	core						
	720678	3474148	core						
	720679	3474149	core						
	720680	3474150	core						
	720681	3474151	full						
	720682	3474152	core						
	720683	3474153	core						
	720684	3474154	core						
	720685	3474155	core						
	720686	3474156	core						
	720687	3474157	extended						
	720688	3474158	core						
	720689	3474159	extended						
	720690	3474160	extended						
	720691	3474161	core						
	720692	3474162	core						
	720693	3474163	full						
	720694	3474164	core						
	720695	3474165	core						
	720696	3474166	extended						
	720697	3474167	extended						
	720698	3474168	core						
	720699	3474169	extended						
	720700	3474170	core						
	720701	3474171	core						
	720702	3474172	extended						
	720703	3474173	extended						
	720704	3474174	extended						
	720705	3474175	extended						
	720706	3474176	extended						
	720707	3474177	core						
		3474178	full						
		3474179	full						
		3474180	extended						
		3474181	core						
		3474182	extended						
		3474183	extended						
		3474184	extended						
		3474185	extended						
		3474186	extended						
		3474187	core						
		3474188	core						
		3474189	core						
		3474190	core						
		3474191	full						
		3474192	core						

		3474193<br>3474194<br>3474195<br>3474196<br>3474197<br>3474198<br>3474199<br>3474200<br>3474201<br>3474202<br>3474203	core<br>core<br>core<br>extended<br>core<br>extended<br>core<br>extended<br>full<br>full<br>full						
3482977	726106<br>726107<br>726108<br>726109<br>726110<br>726111<br>726112<br>726113<br>726114<br>726115<br>726116<br>726117<br>726118<br>726119<br>726120<br>726121<br>726122<br>726123<br>726124<br>726125<br>726126<br>726127<br>726128<br>726129<br>726130	3482978<br>3482979<br>3482980<br>3482981<br>3482982<br>3482983<br>3482984<br>3482985<br>3482986<br>3482987<br>3482988<br>3482989<br>3482990<br>3482991<br>3482992<br>3482993<br>3482994<br>3482995<br>3482996<br>3482997<br>3482998<br>3482999<br>3483000<br>3483001<br>3483002<br>3483003<br>3483004<br>3483005<br>3483006<br>3483007<br>3483008<br>3483009<br>3483010<br>3483011<br>3483012<br>3483013<br>3483014<br>3483015<br>3483016<br>3483017<br>3483018<br>3483019<br>3483020	extended<br>extended<br>extended<br>extended<br>extended<br>extended<br>extended<br>core<br>core<br>core<br>extended<br>core<br>core<br>core<br>core<br>core<br>extended<br>extended<br>full<br>full<br>extended<br>extended<br>core<br>extended<br>extended<br>extended<br>extended<br>extended<br>full<br>extended<br>full<br>full<br>full<br>extended<br>core<br>core<br>core<br>core<br>core<br>core<br>core<br>core<br>core	13	27092903	27169155	+	NM_015972<br>NM_152705<br>AF131855<br>AK097973<br>AF085925<br>CR607657<br>ENSESTT00000006712<br>ENSESTT00000006713<br>ENSESTT00000006714<br>ENSESTT00000006716<br>ENST00000381076<br>ENST00000381064<br>ENST00000302979<br>ENST00000349426<br>GENSCAN00000030786	Homo sapiens polymerase (RNA) I polypeptide D, 16kDa (POLR1D), mRNA.<br>Homo sapiens hypothetical protein MGC9850 (MGC9850), mRNA.<br>Homo sapiens clone 25056 mRNA sequence.<br>Homo sapiens cDNA FLJ40654 fis, clone THYMU2018772.<br>Homo sapiens full length insert cDNA clone YR48C03.<br>full-length cDNA clone CS0DC003YE19 of Neuroblastoma Cot 25-normalized of Homo sapiens (human).<br><br><br><br><br>cdna:known-ccds chromosome:NCBI36:13:27092895:27095533:1 gene:ENSG00000186184 CCDS9325.1<br>cdna:known-ccds chromosome:NCBI36:13:27094003:27139548:1 gene:ENSG00000186184 CCDS9324.1<br>cdna:known chromosome:NCBI36:13:27092903:27095589:1 gene:ENSG00000186184<br>cdna:known chromosome:NCBI36:13:27094003:27139547:1 gene:ENSG00000186184<br>cdna:Genscan chromosome:NCBI36:13:27092304:27139219:1

		3483021<br>3483022<br>3483023	extended<br>full<br>full						
3484641	727151<br>727152<br>727153<br>727154<br>727155<br>727156<br>727157<br>727158<br>727159<br>727160<br>727161<br>727162<br>727163<br>727164<br>727165<br>727166<br>727167<br>727168<br>727169<br>727170<br>727171<br>727172<br>727173<br>727174<br>727175<br>727176<br>727177<br>727178<br>727179<br>727180<br>727181<br>727182<br>727183<br>727184<br>727185<br>727186<br>727187<br>727188	3484642<br>3484643<br>3484644<br>3484645<br>3484646<br>3484647<br>3484648<br>3484649<br>3484650<br>3484651<br>3484652<br>3484653<br>3484654<br>3484655<br>3484656<br>3484657<br>3484658<br>3484659<br>3484660<br>3484661<br>3484662<br>3484663<br>3484664<br>3484665<br>3484666<br>3484667<br>3484668<br>3484669<br>3484670<br>3484671<br>3484672<br>3484673<br>3484674<br>3484675<br>3484676<br>3484677<br>3484678<br>3484679<br>3484680<br>3484681<br>3484682<br>3484683<br>3484684<br>3484685<br>3484686<br>3484687<br>3484688<br>3484689<br>3484690<br>3484691<br>3484692	full<br>core<br>extended<br>core<br>core<br>core<br>core<br>core<br>core<br>core<br>core<br>core<br>core<br>core<br>full<br>core<br>core<br>core<br>core<br>extended<br>core<br>core<br>core<br>extended<br>core<br>core<br>core<br>core<br>core<br>core<br>core<br>core<br>core<br>core<br>core<br>core<br>core<br>extended<br>extended<br>extended<br>extended<br>extended<br>extended<br>extended<br>extended<br>extended<br>full<br>full<br>core<br>extended<br>core	13	31787492	31871806	+	NM_000059<br>ENSESTT00000037590<br>ENSESTT00000037591<br>ENSESTT00000037592<br>ENSESTT00000037593<br>ENSESTT00000037594<br>ENST00000380152<br>ENST00000267071<br>GENSCAN00000015638<br>GENSCAN00000049939<br>GENSCAN00000049937	Homo sapiens breast cancer 2, early onset (BRCA2), mRNA.<br><br><br><br><br><br>cdna:known-ccds chromosome:NCBI36:13:31787617:31871806:1 gene:ENSG00000139618 CCDS9344.1<br>cdna:known chromosome:NCBI36:13:31787617:31871805:1 gene:ENSG00000139618<br>cdna:Genscan chromosome:NCBI36:13:31870723:31870907:1<br>cdna:Genscan chromosome:NCBI36:13:31787492:31857595:1<br>cdna:Genscan chromosome:NCBI36:13:31858167:31869181:1

		3484693<br>3484694<br>3484695<br>3484696<br>3484697<br>3484698<br>3484699	extended<br>core<br>core<br>core<br>core<br>extended<br>core						
3489020	729905<br>729906<br>729907<br>729908<br>729909<br>729910<br>729911<br>729912<br>729913<br>729914<br>729915<br>729916<br>729917<br>729918<br>729919<br>729920<br>729921<br>729922<br>729923<br>729924<br>729925<br>729926<br>729927<br>729928<br>729929<br>729930<br>729931<br>729932<br>729933<br>729934<br>729935<br>729936<br>729937<br>729938<br>729939<br>729940<br>729941<br>729942<br>729943<br>729944<br>729945<br>729946<br>729947<br>729948<br>729949<br>729950<br>729951	3489021<br>3489022<br>3489023<br>3489024<br>3489025<br>3489026<br>3489027<br>3489028<br>3489029<br>3489030<br>3489031<br>3489032<br>3489033<br>3489034<br>3489035<br>3489036<br>3489037<br>3489038<br>3489039<br>3489040<br>3489041<br>3489042<br>3489043<br>3489044<br>3489045<br>3489046<br>3489047<br>3489048<br>3489049<br>3489050<br>3489051<br>3489052<br>3489053<br>3489054<br>3489055<br>3489056<br>3489057<br>3489058<br>3489059<br>3489060<br>3489061<br>3489062<br>3489063<br>3489064<br>3489065<br>3489066<br>3489067	full<br>full<br>core<br>core<br>extended<br>core<br>extended<br>extended<br>extended<br>extended<br>extended<br>extended<br>extended<br>extended<br>extended<br>core<br>core<br>core<br>core<br>extended<br>extended<br>core<br>core<br>extended<br>core<br>core<br>extended<br>extended<br>extended<br>core<br>extended<br>core<br>extended<br>core<br>extended<br>extended<br>core<br>core<br>core<br>core<br>core<br>extended<br>extended<br>extended<br>extended<br>extended<br>full	13	47742938	48071678	+	NM_000321<br>AK097860<br>ENSESTT00000019851<br>ENSESTT00000019852<br>ENSESTT00000019853<br>ENSESTT00000019854<br>ENSESTT00000019855<br>ENST00000267163<br>ENST00000378437<br>ENST00000378436<br>GENSCAN00000027398<br>GENSCAN00000039488<br>GENSCAN00000039489	Homo sapiens retinoblastoma 1 (including osteosarcoma) (RB1), mRNA.<br>Homo sapiens cDNA FLJ40541 fis, clone THYMU1000576.<br><br><br><br><br><br>cdna:known chromosome:NCBI36:13:47775912:47954123:1 gene:ENSG00000139687<br>cdna:known chromosome:NCBI36:13:47775912:47835045:1 gene:ENSG00000139687<br>cdna:known chromosome:NCBI36:13:47775924:47954123:1 gene:ENSG00000139687<br>cdna:Genscan chromosome:NCBI36:13:47904426:47952208:1<br>cdna:Genscan chromosome:NCBI36:13:47766451:47782480:1<br>cdna:Genscan chromosome:NCBI36:13:47812803:47853580:1

	729952<br>729953<br>729954<br>729955<br>729956<br>729957<br>729958<br>729959<br>729960<br>729961<br>729962<br>729963<br>729964<br>729965<br>729966<br>729967<br>729968<br>729969<br>729970<br>729971<br>729972<br>729973<br>729974<br>729975<br>729976<br>729977<br>729978	3489068<br>3489069<br>3489070<br>3489071<br>3489072<br>3489073<br>3489074<br>3489075<br>3489076<br>3489077<br>3489078<br>3489079<br>3489080<br>3489081<br>3489082<br>3489083<br>3489084<br>3489085<br>3489086<br>3489087<br>3489088<br>3489089<br>3489090<br>3489091<br>3489092<br>3489093<br>3489094<br>3489095<br>3489096<br>3489097<br>3489098<br>3489099<br>3489100<br>3489101<br>3489102<br>3489103<br>3489104<br>3489105<br>3489106<br>3489107<br>3489108	full<br>extended<br>extended<br>extended<br>extended<br>extended<br>extended<br>full<br>full<br>full<br>full<br>extended<br>core<br>core<br>core<br>core<br>extended<br>core<br>core<br>extended<br>extended<br>extended<br>core<br>full<br>full<br>extended<br>core<br>core<br>full<br>extended<br>core<br>core<br>extended<br>extended<br>extended<br>extended<br>full<br>full<br>full<br>full<br>extended						
3490655	730934<br>730935<br>730936<br>730937<br>730938<br>730939<br>730940<br>730941<br>730942<br>730943<br>730944	3490656<br>3490657<br>3490658<br>3490659<br>3490660<br>3490661<br>3490662<br>3490663<br>3490664<br>3490665<br>3490666<br>3490667<br>3490668	extended<br>core<br>core<br>core<br>core<br>core<br>core<br>core<br>core<br>core<br>core<br>core<br>core	13	51927516	51948759	+	NM_018204<br>AK027103<br>AY062262<br>BC010901<br>BC105806<br>ENSESTT00000012264<br>ENSESTT00000012265<br>ENSESTT00000012266<br>ENST00000258607<br>ENST00000378037<br>ENST00000378034<br>GENSCAN00000067186	Homo sapiens cytoskeleton associated protein 2 (CKAP2), mRNA.<br>Homo sapiens cDNA: FLJ23450 fis, clone HSI06321.<br>Homo sapiens tumor-associated microtubule-associated protein splice variant (TMAP) mRNA, complete cds; alternatively spliced.<br>Homo sapiens cytoskeleton associated protein 2, mRNA (cDNA clone IMAGE:4096950), complete cds.<br>Homo sapiens cytoskeleton associated protein 2, mRNA (cDNA clone IMAGE:6622858), partial cds.<br><br><br><br>cdna:known-ccds chromosome:NCBI36:13:51927597:51948758:1 gene:ENSG00000136108 CCDS9435.1<br>cdna:known chromosome:NCBI36:13:51927596:51948759:1 gene:ENSG00000136108<br>cdna:known chromosome:NCBI36:13:51933609:51937946:1 gene:ENSG00000136108<br>cdna:Genscan chromosome:NCBI36:13:51927693:51947277:1

		3490669<br>3490670<br>3490671<br>3490672<br>3490673<br>3490674<br>3490675<br>3490676<br>3490677<br>3490678<br>3490679<br>3490680<br>3490681	core<br>extended<br>extended<br>core<br>core<br>core<br>core<br>core<br>core<br>core<br>core<br>core<br>core						
3494137	733061<br>733062<br>733063<br>733064<br>733065<br>733066<br>733067<br>733068<br>733069<br>733070<br>733071<br>733072<br>733073<br>733074<br>733075<br>733076<br>733077<br>733078<br>733079<br>733080<br>733081<br>733082<br>733083<br>733084<br>733085<br>733086<br>733087<br>733088<br>733089<br>733090<br>733091<br>733092<br>733093<br>733094<br>733095<br>733096<br>733097<br>733098<br>733099<br>733100<br>733101	3494138<br>3494139<br>3494140<br>3494141<br>3494142<br>3494143<br>3494144<br>3494145<br>3494146<br>3494147<br>3494148<br>3494149<br>3494150<br>3494151<br>3494152<br>3494153<br>3494154<br>3494155<br>3494156<br>3494157<br>3494158<br>3494159<br>3494160<br>3494161<br>3494162<br>3494163<br>3494164<br>3494165<br>3494166<br>3494167<br>3494168<br>3494169<br>3494170<br>3494171<br>3494172<br>3494173<br>3494174<br>3494175<br>3494176<br>3494177<br>3494178	core<br>core<br>core<br>extended<br>extended<br>extended<br>extended<br>extended<br>extended<br>extended<br>extended<br>full<br>full<br>full<br>full<br>full<br>full<br>full<br>full<br>full<br>extended<br>full<br>full<br>full<br>full<br>full<br>full<br>full<br>full<br>full<br>extended<br>extended<br>extended<br>core<br>extended<br>full<br>full<br>core<br>extended<br>full<br>extended	13	75092571	75566974	+	NM_005358<br>AB020665<br>AK092052<br>AK123459<br>BC036428<br>ENSESTT00000017851<br>ENSESTT00000017852<br>ENSESTT00000017853<br>ENSESTT00000017854<br>ENSESTT00000017855<br>ENSESTT00000017856<br>ENSESTT00000017857<br>ENSESTT00000017858<br>ENSESTT00000017859<br>ENSESTT00000017860<br>ENST00000341547<br>ENST00000357063<br>ENST00000358860<br>ENST00000377534<br>ENST00000321813<br>ENST00000377499<br>ENST00000321797<br>ENST00000377483<br>GENSCAN00000062249<br>GENSCAN00000016848<br>GENSCAN00000038209<br>GENSCAN00000000609<br>GENSCAN00000050419	Homo sapiens LIM domain 7 (LMO7), mRNA.<br>Homo sapiens mRNA for KIAA0858 protein, partial cds.<br>Homo sapiens cDNA FLJ34733 fis, clone MESAN2006953, highly similar to LIM domain only 7 isoform c.<br>Homo sapiens cDNA FLJ41465 fis, clone BRSTN2017761.<br>Homo sapiens LIM domain 7, mRNA (cDNA clone IMAGE:4800225), with apparent retained intron.<br><br><br><br><br><br><br><br><br><br><br>cdna:known-ccds chromosome:NCBI36:13:75092571:75332003:1 gene:ENSG00000136153 CCDS9454.1<br>cdna:known chromosome:NCBI36:13:75092571:75343063:1 gene:ENSG00000136153<br>cdna:known chromosome:NCBI36:13:75092571:75332002:1 gene:ENSG00000136153<br>cdna:known chromosome:NCBI36:13:75092571:75332002:1 gene:ENSG00000136153<br>cdna:known chromosome:NCBI36:13:75092571:75332002:1 gene:ENSG00000136153<br>cdna:known chromosome:NCBI36:13:75108460:75317505:1 gene:ENSG00000136153<br>cdna:known chromosome:NCBI36:13:75232868:75330812:1 gene:ENSG00000136153<br>cdna:known chromosome:NCBI36:13:75295763:75310589:1 gene:ENSG00000136153<br>cdna:Genscan chromosome:NCBI36:13:75289298:75289415:1<br>cdna:Genscan chromosome:NCBI36:13:75151400:75280388:1<br>cdna:Genscan chromosome:NCBI36:13:75554511:75566974:1<br>cdna:Genscan chromosome:NCBI36:13:75291530:75374535:1<br>cdna:Genscan chromosome:NCBI36:13:75449271:75481294:1

	733102<br>733103<br>733104<br>733105<br>733106<br>733107<br>733108<br>733109<br>733110<br>733111<br>733112<br>733113<br>733114<br>733115<br>733116<br>733117<br>733118<br>733119<br>733120<br>733121<br>733122<br>733123<br>733124<br>733125<br>733126<br>733127<br>733128<br>733129<br>733130<br>733131<br>733132<br>733133<br>733134<br>733135<br>733136<br>733137<br>733138<br>733139<br>733140<br>733141<br>733142<br>733143<br>733144<br>733145<br>733146<br>733147<br>733148<br>733149<br>733150<br>733151<br>733152<br>733153<br>733154<br>733155	3494179<br>3494180<br>3494181<br>3494182<br>3494183<br>3494184<br>3494185<br>3494186<br>3494187<br>3494188<br>3494189<br>3494190<br>3494191<br>3494192<br>3494193<br>3494194<br>3494195<br>3494196<br>3494197<br>3494198<br>3494199<br>3494200<br>3494201<br>3494202<br>3494203<br>3494204<br>3494205<br>3494206<br>3494207<br>3494208<br>3494209<br>3494210<br>3494211<br>3494212<br>3494213<br>3494214<br>3494215<br>3494216<br>3494217<br>3494218<br>3494219<br>3494220<br>3494221<br>3494222<br>3494223<br>3494224<br>3494225<br>3494226<br>3494227<br>3494228<br>3494229<br>3494230<br>3494231<br>3494232	extended<br>extended<br>core<br>full<br>full<br>full<br>extended<br>extended<br>extended<br>full<br>full<br>core<br>core<br>core<br>core<br>core<br>extended<br>full<br>extended<br>extended<br>extended<br>extended<br>extended<br>extended<br>full<br>full<br>extended<br>extended<br>extended<br>core<br>extended<br>full<br>core<br>extended<br>core<br>core<br>core<br>core<br>core<br>full<br>core<br>core<br>full<br>extended<br>core<br>core<br>extended<br>core<br>extended<br>extended<br>core<br>core<br>core<br>full						

	733156	3494233	core						
	733157	3494234	core						
	733158	3494235	core						
	733159	3494236	core						
	733160	3494237	core						
	733161	3494238	core						
	733162	3494239	full						
	733163	3494240	extended						
	733164	3494241	extended						
	733165	3494242	core						
	733166	3494243	core						
	733167	3494244	core						
	733168	3494245	free						
	733169	3494246	extended						
	733170	3494247	core						
	733171	3494248	extended						
	733172	3494249	full						
	733173	3494250	extended						
	733174	3494251	extended						
		3494252	core						
		3494253	extended						
		3494254	core						
		3494255	core						
		3494256	extended						
		3494257	extended						
		3494258	extended						
		3494259	extended						
		3494260	extended						
		3494261	core						
		3494262	core						
		3494263	core						
		3494264	extended						
		3494265	full						
		3494266	full						
		3494267	full						
		3494268	full						
		3494269	full						
		3494270	full						
		3494271	full						
		3494272	full						
		3494273	full						
		3494274	full						
		3494275	full						
		3494276	full						
		3494277	full						
		3494278	full						
		3494279	full						
		3494280	extended						
		3494281	extended						
		3494282	extended						
		3494283	full						
		3494284	full						
		3494285	full						
		3494286	full						

		3494287<br>3494288<br>3494289<br>3494290<br>3494291<br>3494292<br>3494293<br>3494294	full<br>full<br>full<br>extended<br>extended<br>extended<br>full<br>full						
3496366	734437<br>734438<br>734439<br>734440<br>734441<br>734442<br>734443<br>734444<br>734445<br>734446<br>734447<br>734448<br>734449<br>734450<br>734451<br>734452<br>734453<br>734454<br>734455<br>734456<br>734457<br>734458<br>734459<br>734460	3496367<br>3496368<br>3496369<br>3496370<br>3496371<br>3496372<br>3496373<br>3496374<br>3496375<br>3496376<br>3496377<br>3496378<br>3496379<br>3496380<br>3496381<br>3496382<br>3496383<br>3496384<br>3496385<br>3496386<br>3496387<br>3496388<br>3496389<br>3496390<br>3496391<br>3496392<br>3496393<br>3496394<br>3496395<br>3496396<br>3496397<br>3496398<br>3496399<br>3496400<br>3496401<br>3496402<br>3496403<br>3496404<br>3496405<br>3496406<br>3496407<br>3496408	full<br>full<br>full<br>full<br>full<br>core<br>full<br>full<br>full<br>full<br>core<br>core<br>core<br>core<br>core<br>core<br>core<br>core<br>core<br>core<br>core<br>core<br>core<br>core<br>core<br>core<br>core<br>core<br>core<br>core<br>core<br>core<br>core<br>extended<br>extended<br>full<br>full<br>full<br>extended<br>full<br>full<br>full	13	90773407	90847722	+	XM_931068<br>XM_942906<br>AB176708<br>AF339828<br>BC040320<br>AY927632<br>ENST00000356529<br>ENST00000360600<br>ENST00000377076<br>GENSCAN00000028136<br>GENSCAN00000029955<br>GENSCAN00000058926	PREDICTED: Homo sapiens chromosome 13 open reading frame 25 (C13orf25), mRNA.<br>PREDICTED: Homo sapiens chromosome 13 open reading frame 25 (C13orf25), mRNA.<br>Homo sapiens C13orf25 v_2 mRNA, complete cds, miR-91-precursor-13 micro RNA, microRNA miR-91, microRNA miR-17, miR-18-precursor-13 micro RNA, microRNA miR-18, miR-19a-precursor-13 micro RNA, microRNA miR-19a, microRNA miR-20, miR-19b-precursor-13 micro RNA, microRNA miR-19b, miR-92-precursor-13 micro RNA, microRNA miR-92.<br>Homo sapiens clone IMAGE:593405, mRNA sequence.<br>Homo sapiens chromosome 13 open reading frame 25, mRNA (cDNA clone IMAGE:4838522).<br>Homo sapiens mRNA sequence.<br>cdna:known-ccds chromosome:NCBI36:13:90798075:90804870:1 gene:ENSG00000198051 CCDS9467.1<br>cdna:known-ccds chromosome:NCBI36:13:90798075:90804830:1 gene:ENSG00000198051 CCDS9466.1<br>cdna:known chromosome:NCBI36:13:90799948:90804834:1 gene:ENSG00000198051<br>cdna:Genscan chromosome:NCBI36:13:90819032:90844764:1<br>cdna:Genscan chromosome:NCBI36:13:90799032:90799403:1<br>cdna:Genscan chromosome:NCBI36:13:90773407:90773451:1
3497790	735410<br>735411<br>735412	3497791<br>3497792<br>3497793	full<br>full<br>full	13	97370094	97486932	+	NM_002271<br>AK125031<br>ENSESTT00000023700	Homo sapiens RAN binding protein 5 (RANBP5), mRNA.<br>Homo sapiens cDNA FLJ43041 fis, clone BRTHA3003339, highly similar to Importin beta-3 subunit.

	735413	3497794	core					ENSESTT00000023701	
	735414	3497795	extended					ENSESTT00000023702	
	735415	3497796	core					ENSESTT00000023703	
	735416	3497797	core					ENSESTT00000023704	
	735417	3497798	extended					ENST00000357602	cdna:known chromosome:NCBI36:13:97403930:97474552:1 gene:ENSG00000065150
	735418	3497799	extended					ENST00000261574	cdna:known chromosome:NCBI36:13:97403930:97474550:1 gene:ENSG00000065150
	735419	3497800	core					ENST00000376594	cdna:known chromosome:NCBI36:13:97403930:97474550:1 gene:ENSG00000065150
	735420	3497801	core					ENST00000389591	cdna:known chromosome:NCBI36:13:97420036:97472077:1 gene:ENSG00000065150
	735421	3497802	extended					GENSCAN00000011654	cdna:Genscan chromosome:NCBI36:13:97370094:97445034:1
	735422	3497803	extended					GENSCAN00000011652	cdna:Genscan chromosome:NCBI36:13:97447806:97462632:1
	735423	3497804	extended					GENSCAN00000011661	cdna:Genscan chromosome:NCBI36:13:97464290:97467106:1
	735424	3497805	core						
	735425	3497806	extended						
	735426	3497807	extended						
	735427	3497808	core						
	735428	3497809	core						
	735429	3497810	core						
	735430	3497811	core						
	735431	3497812	core						
	735432	3497813	extended						
	735433	3497814	core						
	735434	3497815	core						
	735435	3497816	extended						
	735436	3497817	core						
	735437	3497818	core						
	735438	3497819	extended						
	735439	3497820	core						
	735440	3497821	core						
	735441	3497822	core						
	735442	3497823	core						
	735443	3497824	core						
	735444	3497825	full						
	735445	3497826	core						
	735446	3497827	core						
	735447	3497828	core						
	735448	3497829	core						
	735449	3497830	core						
	735450	3497831	full						
	735451	3497832	extended						
	735452	3497833	core						
	735453	3497834	extended						
	735454	3497835	core						
	735455	3497836	core						
	735456	3497837	extended						
	735457	3497838	core						
	735458	3497839	core						
		3497840	core						
		3497841	core						
		3497842	extended						
		3497843	extended						
		3497844	core						
		3497845	extended						
		3497846	extended						
		3497847	core						

		3497848<br>3497849<br>3497850<br>3497851<br>3497852<br>3497853<br>3497854<br>3497855<br>3497856	core<br>core<br>core<br>core<br>full<br>full<br>full<br>full<br>full						
3497881	735475<br>735476<br>735477<br>735478<br>735479<br>735480<br>735481<br>735482<br>735483<br>735484<br>735485<br>735486<br>735487<br>735488<br>735489<br>735490<br>735491<br>735492<br>735493<br>735494<br>735495<br>735496<br>735497<br>735498<br>735499<br>735500<br>735501<br>735502<br>735503<br>735504<br>735505<br>735506<br>735507<br>735508<br>735509<br>735510<br>735511<br>735512<br>735513<br>735514<br>735515<br>735516<br>735517<br>735518<br>735519	3497882<br>3497883<br>3497884<br>3497885<br>3497886<br>3497887<br>3497888<br>3497889<br>3497890<br>3497891<br>3497892<br>3497893<br>3497894<br>3497895<br>3497896<br>3497897<br>3497898<br>3497899<br>3497900<br>3497901<br>3497902<br>3497903<br>3497904<br>3497905<br>3497906<br>3497907<br>3497908<br>3497909<br>3497910<br>3497911<br>3497912<br>3497913<br>3497914<br>3497915<br>3497916<br>3497917<br>3497918<br>3497919<br>3497920<br>3497921<br>3497922<br>3497923<br>3497924<br>3497925<br>3497926	full<br>core<br>full<br>core<br>full<br>full<br>extended<br>extended<br>extended<br>full<br>extended<br>extended<br>extended<br>full<br>full<br>extended<br>extended<br>extended<br>extended<br>full<br>full<br>extended<br>full<br>core<br>full<br>extended<br>extended<br>extended<br>extended<br>extended<br>extended<br>extended<br>extended<br>extended<br>full<br>extended<br>extended<br>extended<br>full<br>full<br>full<br>extended<br>extended<br>extended<br>extended	13	97592742	97902224	+	XM_943174<br>XM_931381<br>NM_005766<br>NM_001001715<br>AF339772<br>AF339798<br>AF339830<br>AK025341<br>AK095747<br>AK123565<br>BC071592<br>BX537667<br>ENSESTT00000023705<br>ENSESTT00000023706<br>ENSESTT00000023707<br>ENSESTT00000023708<br>ENSESTT00000023709<br>ENSESTT00000023711<br>ENST00000319562<br>ENST00000376586<br>ENST00000376584<br>ENST00000376581<br>ENST00000376579<br>GENSCAN00000020340<br>GENSCAN00000020342<br>GENSCAN00000011024<br>GENSCAN00000066616<br>GENSCAN00000066617<br>GENSCAN00000057543	PREDICTED: Homo sapiens hypothetical protein LOC648003 (LOC648003), mRNA.<br>PREDICTED: Homo sapiens hypothetical protein LOC643176 (LOC643176), mRNA.<br>Homo sapiens FERM, RhoGEF (ARHGEF) and pleckstrin domain protein 1 (chondrocyte-derived) (FARP1), transcript variant 1, mRNA.<br>Homo sapiens FERM, RhoGEF (ARHGEF) and pleckstrin domain protein 1 (chondrocyte-derived) (FARP1), transcript variant 2, mRNA.<br>Homo sapiens clone IMAGE:128781, mRNA sequence.<br>Homo sapiens clone IMAGE:234898, mRNA sequence.<br>Homo sapiens clone IMAGE:609899, mRNA sequence.<br>Homo sapiens cDNA: FLJ21688 fis, clone COL09457.<br>Homo sapiens cDNA FLJ38428 fis, clone FEBRA2013274, weakly similar to Homo sapiens mRNA for CDEP.<br>Homo sapiens cDNA FLJ41571 fis, clone CTONG2009531.<br>Homo sapiens FERM, RhoGEF (ARHGEF) and pleckstrin domain protein 1 (chondrocyte-derived), mRNA (cDNA clone MGC:87400 IMAGE:30342239), complete cds.<br>Homo sapiens mRNA; cDNA DKFZp686D08109 (from clone DKFZp686D08109).<br><br><br><br><br><br><br><br>cdna:known-ccds chromosome:NCBI36:13:97592817:97900017:1 gene:ENSG00000152767 CCDS9487.1<br>cdna:known chromosome:NCBI36:13:97592817:97900017:1 gene:ENSG00000152767<br>cdna:known chromosome:NCBI36:13:97592817:97875754:1 gene:ENSG00000152767<br>cdna:known chromosome:NCBI36:13:97593435:97695559:1 gene:ENSG00000152767<br>cdna:known chromosome:NCBI36:13:97593435:97695557:1 gene:ENSG00000152767<br>cdna:Genscan chromosome:NCBI36:13:97500605:97604838:1<br>cdna:Genscan chromosome:NCBI36:13:97663498:97664674:1<br>cdna:Genscan chromosome:NCBI36:13:97854201:97902228:1<br>cdna:Genscan chromosome:NCBI36:13:97752678:97757810:1<br>cdna:Genscan chromosome:NCBI36:13:97791192:97845944:1<br>cdna:Genscan chromosome:NCBI36:13:97711481:97718059:1

	735520	3497927	full						
	735521	3497928	full						
	735522	3497929	full						
	735523	3497930	full						
	735524	3497931	extended						
	735525	3497932	extended						
	735526	3497933	full						
	735527	3497934	extended						
	735528	3497935	full						
	735529	3497936	full						
	735530	3497937	full						
	735531	3497938	extended						
	735532	3497939	full						
	735533	3497940	full						
	735534	3497941	full						
	735535	3497942	full						
	735536	3497943	core						
	735537	3497944	full						
	735538	3497945	full						
	735539	3497946	full						
	735540	3497947	full						
	735541	3497948	full						
	735542	3497949	full						
	735543	3497950	core						
	735544	3497951	core						
	735545	3497952	full						
	735546	3497953	core						
	735547	3497954	full						
	735548	3497955	extended						
	735549	3497956	core						
	735550	3497957	extended						
	735551	3497958	core						
	735552	3497959	extended						
	735553	3497960	core						
	735554	3497961	extended						
	735555	3497962	extended						
	735556	3497963	core						
	735557	3497964	extended						
	735558	3497965	core						
	735559	3497966	extended						
	735560	3497967	extended						
	735561	3497968	core						
	735562	3497969	full						
	735563	3497970	full						
	735564	3497971	core						
	735565	3497972	core						
	735566	3497973	full						
	735567	3497974	full						
	735568	3497975	extended						
	735569	3497976	extended						
	735570	3497977	extended						
	735571	3497978	extended						
	735572	3497979	extended						
	735573	3497980	extended						

	735574	3497981	extended						
	735575	3497982	extended						
	735576	3497983	extended						
	735577	3497984	extended						
	735578	3497985	full						
	735579	3497986	full						
	735580	3497987	core						
	735581	3497988	core						
		3497989	extended						
		3497990	full						
		3497991	core						
		3497992	extended						
		3497993	extended						
		3497994	extended						
		3497995	extended						
		3497996	full						
		3497997	full						
		3497998	full						
		3497999	extended						
		3498000	full						
		3498001	full						
		3498002	core						
		3498003	full						
		3498004	core						
		3498005	core						
		3498006	extended						
		3498007	extended						
		3498008	extended						
		3498009	core						
		3498010	extended						
		3498011	extended						
		3498012	extended						
		3498013	extended						
		3498014	extended						
		3498015	core						
		3498016	core						
		3498017	full						
		3498018	core						
		3498019	full						
		3498020	full						
		3498021	full						
		3498022	full						
		3498023	core						
		3498024	core						
		3498025	full						
		3498026	core						
		3498027	full						
		3498028	full						
		3498029	full						
		3498030	full						
		3498031	full						
		3498032	full						
		3498033	full						
		3498034	full						

		3498035<br>3498036<br>3498037<br>3498038<br>3498039<br>3498040<br>3498041<br>3498042<br>3498043<br>3498044<br>3498045<br>3498046<br>3498047<br>3498048<br>3498049<br>3498050<br>3498051<br>3498052	core<br>full<br>core<br>core<br>extended<br>core<br>core<br>core<br>core<br>core<br>core<br>core<br>core<br>core<br>full<br>full<br>extended<br>full						
3499132	736297<br>736298<br>736299<br>736300<br>736301<br>736302<br>736303<br>736304<br>736305<br>736306<br>736307<br>736308<br>736309<br>736310<br>736311<br>736312<br>736313<br>736314<br>736315<br>736316<br>736317<br>736318<br>736319<br>736320<br>736321<br>736322<br>736323<br>736324<br>736325<br>736326<br>736327<br>736328<br>736329<br>736330<br>736331<br>736332	3499133<br>3499134<br>3499135<br>3499136<br>3499137<br>3499138<br>3499139<br>3499140<br>3499141<br>3499142<br>3499143<br>3499144<br>3499145<br>3499146<br>3499147<br>3499148<br>3499149<br>3499150<br>3499151<br>3499152<br>3499153<br>3499154<br>3499155<br>3499156<br>3499157<br>3499158<br>3499159<br>3499160<br>3499161<br>3499162<br>3499163<br>3499164<br>3499165<br>3499166<br>3499167<br>3499168	extended<br>extended<br>core<br>core<br>core<br>core<br>core<br>core<br>full<br>full<br>full<br>full<br>full<br>full<br>full<br>full<br>full<br>full<br>extended<br>full<br>full<br>full<br>full<br>full<br>core<br>core<br>full<br>extended<br>full<br>extended<br>core<br>core<br>full<br>core<br>full<br>full	13	100902857	101173457	+	NM_004791<br>AF339779<br>AK026784<br>AK075136<br>ENSESTT00000006231<br>ENSESTT00000006232<br>ENSESTT00000006233<br>ENSESTT00000006234<br>ENST00000376180<br>ENST00000376162<br>ENST00000376161<br>ENST00000355866<br>ENST00000376155<br>GENSCAN00000058060<br>GENSCAN00000048252<br>GENSCAN00000057629	Homo sapiens integrin, beta-like 1 (with EGF-like repeat domains) (ITGBL1), mRNA.<br>Homo sapiens clone IMAGE:1602499, mRNA sequence.<br>Homo sapiens cDNA: FLJ23131 fis, clone LNG08502.<br>Homo sapiens cDNA FLJ90655 fis, clone PLACE1004630.<br><br><br><br><br>cdna:known-ccds chromosome:NCBI36:13:100902857:101166795:1 gene:ENSG00000198542 CCDS9499.1<br>cdna:known chromosome:NCBI36:13:100902967:101166794:1 gene:ENSG00000198542<br>cdna:known chromosome:NCBI36:13:100902967:101166790:1 gene:ENSG00000198542<br>cdna:known chromosome:NCBI36:13:100903014:101166790:1 gene:ENSG00000198542<br>cdna:known chromosome:NCBI36:13:101157120:101169146:1 gene:ENSG00000198542<br>cdna:Genscan chromosome:NCBI36:13:101029642:101055024:1<br>cdna:Genscan chromosome:NCBI36:13:101142936:101169102:1<br>cdna:Genscan chromosome:NCBI36:13:100971958:101018197:1

	736333<br>736334<br>736335<br>736336<br>736337<br>736338<br>736339<br>736340<br>736341<br>736342<br>736343<br>736344<br>736345<br>736346<br>736347<br>736348<br>736349<br>736350<br>736351<br>736352<br>736353<br>736354<br>736355<br>736356<br>736357<br>736358	3499169<br>3499170<br>3499171<br>3499172<br>3499173<br>3499174<br>3499175<br>3499176<br>3499177<br>3499178<br>3499179<br>3499180<br>3499181<br>3499182<br>3499183<br>3499184<br>3499185<br>3499186<br>3499187<br>3499188<br>3499189<br>3499190<br>3499191<br>3499192<br>3499193<br>3499194<br>3499195<br>3499196<br>3499197<br>3499198<br>3499199<br>3499200<br>3499201<br>3499202<br>3499203<br>3499204<br>3499205<br>3499206<br>3499207	full<br>full<br>full<br>full<br>full<br>full<br>full<br>full<br>full<br>extended<br>full<br>full<br>full<br>full<br>core<br>extended<br>full<br>full<br>extended<br>core<br>core<br>full<br>full<br>full<br>full<br>extended<br>core<br>core<br>core<br>extended<br>extended<br>extended<br>extended<br>extended<br>extended<br>extended<br>extended<br>extended<br>extended						
3504434	739612<br>739613<br>739614<br>739615<br>739616<br>739617<br>739618<br>739619<br>739620<br>739621<br>739622<br>739623<br>739624<br>739625<br>739626	3504435<br>3504436<br>3504437<br>3504438<br>3504439<br>3504440<br>3504441<br>3504442<br>3504443<br>3504444<br>3504445<br>3504446<br>3504447<br>3504448<br>3504449	core<br>core<br>core<br>core<br>core<br>core<br>core<br>full<br>extended<br>core<br>core<br>core<br>extended<br>core<br>full	13	20251014	20375187	-	NM_022459<br>AK129848<br>BC050680<br>BC098402<br>ENSESTT00000002840<br>ENSESTT00000002841<br>ENSESTT00000002842<br>ENST00000255305<br>ENST00000382693<br>GENSCAN00000022789<br>GENSCAN00000004302<br>GENSCAN00000004301	Homo sapiens exportin 4 (XPO4), mRNA.<br>Homo sapiens cDNA FLJ26338 fis, clone HRT02874.<br>Homo sapiens exportin 4, mRNA (cDNA clone IMAGE:6143088), partial cds.<br>Homo sapiens cDNA clone IMAGE:30528617, with apparent retained intron.<br><br><br><br>cdna:known chromosome:NCBI36:13:20251014:20374949:-1 gene:ENSG00000132953<br>cdna:novel chromosome:NCBI36:13:20262624:20374850:-1 gene:ENSG00000132953<br>cdna:Genscan chromosome:NCBI36:13:20321338:20374874:-1<br>cdna:Genscan chromosome:NCBI36:13:20255861:20299298:-1<br>cdna:Genscan chromosome:NCBI36:13:20315011:20315187:-1

	739627<br>739628<br>739629<br>739630<br>739631<br>739632<br>739633<br>739634<br>739635<br>739636<br>739637<br>739638<br>739639<br>739640<br>739641<br>739642<br>739643<br>739644<br>739645<br>739646<br>739647<br>739648<br>739649<br>739650<br>739651<br>739652<br>739653	3504450<br>3504451<br>3504452<br>3504453<br>3504454<br>3504455<br>3504456<br>3504457<br>3504458<br>3504459<br>3504460<br>3504461<br>3504462<br>3504463<br>3504464<br>3504465<br>3504466<br>3504467<br>3504468<br>3504469<br>3504470<br>3504471<br>3504472<br>3504473<br>3504474<br>3504475<br>3504476<br>3504477<br>3504478<br>3504479<br>3504480<br>3504481<br>3504482<br>3504483<br>3504484<br>3504485<br>3504486<br>3504487<br>3504488	extended<br>core<br>core<br>core<br>core<br>core<br>extended<br>core<br>core<br>core<br>core<br>core<br>core<br>core<br>full<br>extended<br>core<br>extended<br>extended<br>extended<br>extended<br>core<br>extended<br>core<br>extended<br>full<br>core<br>extended<br>extended<br>extended<br>core<br>extended<br>extended<br>core<br>extended<br>full<br>full<br>extended<br>extended						
3508330	742047<br>742048<br>742049<br>742050<br>742051<br>742052<br>742053<br>742054<br>742055<br>742056<br>742057<br>742058<br>742059<br>742060<br>742061	3508331<br>3508332<br>3508333<br>3508334<br>3508335<br>3508336<br>3508337<br>3508338<br>3508339<br>3508340<br>3508341<br>3508342<br>3508343<br>3508344<br>3508345	full<br>full<br>extended<br>full<br>full<br>full<br>full<br>full<br>full<br>full<br>full<br>full<br>full<br>full<br>full	13	30558258	30651692	-	NM_006644<br>BX648125<br>BX648623<br>CR611122<br>ENSESTT00000029039<br>ENSESTT00000029040<br>ENSESTT00000029041<br>ENST00000320027<br>ENST00000380406<br>ENST00000380405<br>ENST00000380400<br>ENST00000380398<br>ENST00000347897<br>ENST00000380363<br>GENSCAN00000029771	Homo sapiens heat shock 105kDa/110kDa protein 1 (HSPH1), mRNA.<br>Homo sapiens mRNA; cDNA DKFZp686G2440 (from clone DKFZp686G2440).<br>Homo sapiens mRNA; cDNA DKFZp686M05240 (from clone DKFZp686M05240).<br>full-length cDNA clone CS0DF022YM06 of Fetal brain of Homo sapiens (human).<br><br><br><br>cdna:known-ccds chromosome:NCBI36:13:30608765:30634117:-1 gene:ENSG00000120694 CCDS9340.1<br>cdna:known chromosome:NCBI36:13:30608765:30634117:-1 gene:ENSG00000120694<br>cdna:known chromosome:NCBI36:13:30608765:30634117:-1 gene:ENSG00000120694<br>cdna:known chromosome:NCBI36:13:30608765:30634064:-1 gene:ENSG00000120694<br>cdna:known chromosome:NCBI36:13:30608765:30634064:-1 gene:ENSG00000120694<br>cdna:known chromosome:NCBI36:13:30608765:30634064:-1 gene:ENSG00000120694<br>cdna:known chromosome:NCBI36:13:30609226:30634502:-1 gene:ENSG00000120694<br>cdna:Genscan chromosome:NCBI36:13:30609455:30651692:-1

	742062	3508346	full						
	742063	3508347	extended						
	742064	3508348	full						
	742065	3508349	full						
	742066	3508350	full						
	742067	3508351	full						
	742068	3508352	extended						
	742069	3508353	extended						
	742070	3508354	extended						
	742071	3508355	extended						
	742072	3508356	core						
	742073	3508357	core						
	742074	3508358	core						
	742075	3508359	core						
	742076	3508360	core						
	742077	3508361	core						
	742078	3508362	core						
	742079	3508363	extended						
	742080	3508364	extended						
	742081	3508365	core						
	742082	3508366	core						
	742083	3508367	core						
	742084	3508368	core						
	742085	3508369	extended						
	742086	3508370	extended						
	742087	3508371	core						
		3508372	extended						
		3508373	core						
		3508374	extended						
		3508375	core						
		3508376	extended						
		3508377	core						
		3508378	extended						
		3508379	core						
		3508380	core						
		3508381	extended						
		3508382	extended						
		3508383	core						
		3508384	core						
		3508385	extended						
		3508386	core						
		3508387	core						
		3508388	extended						
		3508389	core						
		3508390	extended						
		3508391	core						
		3508392	extended						
		3508393	core						
		3508394	extended						
		3508395	extended						
		3508396	core						
		3508397	extended						
		3508398	extended						
		3508399	core						

		3508400<br>3508401<br>3508402<br>3508403<br>3508404<br>3508405<br>3508406<br>3508407	core<br>core<br>extended<br>extended<br>extended<br>extended<br>full<br>full						
3510066	743150<br>743151<br>743152<br>743153<br>743154<br>743155<br>743156<br>743157<br>743158<br>743159<br>743160<br>743161<br>743162<br>743163<br>743164<br>743165<br>743166<br>743167<br>743168<br>743169<br>743170<br>743171<br>743172<br>743173<br>743174<br>743175<br>743176<br>743177<br>743178<br>743179<br>743180<br>743181	3510067<br>3510068<br>3510069<br>3510070<br>3510071<br>3510072<br>3510073<br>3510074<br>3510075<br>3510076<br>3510077<br>3510078<br>3510079<br>3510080<br>3510081<br>3510082<br>3510083<br>3510084<br>3510085<br>3510086<br>3510087<br>3510088<br>3510089<br>3510090<br>3510091<br>3510092<br>3510093<br>3510094<br>3510095<br>3510096<br>3510097<br>3510098<br>3510099<br>3510100<br>3510101<br>3510102<br>3510103<br>3510104<br>3510105<br>3510106<br>3510107<br>3510108<br>3510109<br>3510110<br>3510111	core<br>core<br>core<br>core<br>extended<br>core<br>extended<br>extended<br>extended<br>full<br>full<br>extended<br>extended<br>core<br>core<br>core<br>extended<br>core<br>extended<br>extended<br>free<br>extended<br>core<br>free<br>core<br>core<br>core<br>core<br>core<br>core<br>core<br>core<br>core<br>core<br>core<br>core<br>core<br>core<br>core<br>extended<br>extended<br>extended<br>core<br>core<br>extended	13	37034726	37070900	-	NM_006475<br>AK021444<br>AK021638<br>AK023481<br>ENSESTT00000017843<br>ENSESTT00000017844<br>ENSESTT00000017845<br>ENSESTT00000017846<br>ENST00000379747<br>ENST00000379749<br>ENST00000379743<br>ENST00000379742<br>ENST00000350926<br>ENST00000255475<br>ENST00000379733<br>ENST00000349737<br>GENSCAN00000025140	Homo sapiens periostin, osteoblast specific factor (POSTN), mRNA.<br>Homo sapiens cDNA FLJ11382 fis, clone HEMBA1000504.<br>Homo sapiens cDNA FLJ11576 fis, clone HEMBA1003548.<br>Homo sapiens cDNA FLJ13419 fis, clone PLACE1002115.<br><br><br><br><br>cdna:known-ccds chromosome:NCBI36:13:37034726:37070893:-1 gene:ENSG00000133110 CCDS9364.1<br>cdna:known chromosome:NCBI36:13:37034722:37070898:-1 gene:ENSG00000133110<br>cdna:known chromosome:NCBI36:13:37034728:37070887:-1 gene:ENSG00000133110<br>cdna:known chromosome:NCBI36:13:37034771:37070895:-1 gene:ENSG00000133110<br>cdna:known chromosome:NCBI36:13:37034776:37070885:-1 gene:ENSG00000133110<br>cdna:known chromosome:NCBI36:13:37034779:37070874:-1 gene:ENSG00000133110<br>cdna:known chromosome:NCBI36:13:37034779:37070874:-1 gene:ENSG00000133110<br>cdna:known chromosome:NCBI36:13:37034779:37070874:-1 gene:ENSG00000133110<br>cdna:Genscan chromosome:NCBI36:13:37038790:37052834:-1

3544678	764536	3544679	extended	14	75197089	75499237	+	NM_015072	Homo sapiens tubulin tyrosine ligase-like family, member 5 (TTLL5), mRNA.
	764537	3544680	extended					AK021879	Homo sapiens cDNA FLJ11817 fis, clone HEMBA1006421.
	764538	3544681	extended					AK024259	Homo sapiens cDNA FLJ14197 fis, clone NT2RP3002484, highly similar to Homo sapiens mRNA for KIAA0998 protein.
	764539	3544682	extended					BC002766	Homo sapiens tubulin tyrosine ligase-like family, member 5, mRNA (cDNA clone IMAGE:3632160), complete cds.
	764540	3544683	extended					AL136808	Homo sapiens mRNA; cDNA DKFZp434N2321 (from clone DKFZp434N2321); complete cds.
	764541	3544684	core					BX647530	Homo sapiens mRNA; cDNA DKFZp686L04267 (from clone DKFZp686L04267).
	764542	3544685	core					BC016625	Homo sapiens tubulin tyrosine ligase-like family, member 5, mRNA (cDNA clone IMAGE:3868578), containing frame-shift
	764543	3544686	core					ENSESTT00000003613	errors.
	764544	3544687	extended					ENSESTT00000003614	
	764545	3544688	core					ENSESTT00000003615	
	764546	3544689	core					ENSESTT00000003616	
	764547	3544690	extended					ENSESTT00000003617	
	764548	3544691	extended					ENSESTT00000003618	
	764549	3544692	core					ENSESTT00000003619	
	764550	3544693	extended					ENSESTT00000003620	
	764551	3544694	core					ENSESTT00000003621	
	764552	3544695	core					ENSESTT00000003622	
	764553	3544696	full					ENSESTT00000003623	
	764554	3544697	full					ENSESTT00000003624	
	764555	3544698	full					ENST00000298832	
	764556	3544699	core					ENST00000286650	cdna:known chromosome:NCBI36:14:75197374:75491174:1 gene:ENSG00000119685
	764557	3544700	core					ENST00000286653	cdna:known chromosome:NCBI36:14:75197374:75245924:1 gene:ENSG00000119685
	764558	3544701	core					GENSCAN00000012810	cdna:known chromosome:NCBI36:14:75281226:75423392:1 gene:ENSG00000119685
	764559	3544702	extended					GENSCAN00000057488	cdna:Genscan chromosome:NCBI36:14:75356103:75465196:1
	764560	3544703	core						cdna:Genscan chromosome:NCBI36:14:75199258:75205618:1
	764561	3544704	full						
	764562	3544705	extended						
	764563	3544706	core						
	764564	3544707	core						
	764565	3544708	extended						
	764566	3544709	full						
	764567	3544710	extended						
	764568	3544711	extended						
	764569	3544712	extended						
	764570	3544713	core						
	764571	3544714	core						
	764572	3544715	core						
	764573	3544716	extended						
	764574	3544717	extended						
	764575	3544718	extended						
	764576	3544719	extended						
	764577	3544720	extended						
	764578	3544721	extended						
	764579	3544722	core						
	764580	3544723	core						
	764581	3544724	extended						
	764582	3544725	core						
	764583	3544726	core						
	764584	3544727	core						
	764585	3544728	extended						
	764586	3544729	full						
	764587	3544730	full						
	764588	3544731	core						
	764589	3544732	extended						

	764590	3544733	core						
	764591	3544734	core						
	764592	3544735	extended						
	764593	3544736	core						
	764594	3544737	full						
	764595	3544738	core						
	764596	3544739	extended						
	764597	3544740	core						
	764598	3544741	core						
	764599	3544742	core						
	764600	3544743	core						
	764601	3544744	core						
	764602	3544745	core						
	764603	3544746	extended						
	764604	3544747	extended						
	764605	3544748	extended						
	764606	3544749	core						
	764607	3544750	core						
	764608	3544751	full						
	764609	3544752	extended						
	764610	3544753	full						
	764611	3544754	full						
	764612	3544755	full						
	764613	3544756	full						
	764614	3544757	extended						
	764615	3544758	core						
	764616	3544759	core						
	764617	3544760	extended						
	764618	3544761	extended						
	764619	3544762	extended						
	764620	3544763	extended						
	764621	3544764	full						
	764622	3544765	extended						
	764623	3544766	full						
	764624	3544767	full						
	764625	3544768	extended						
	764626	3544769	extended						
	764627	3544770	extended						
	764628	3544771	extended						
	764629	3544772	full						
	764630	3544773	extended						
	764631	3544774	extended						
	764632	3544775	extended						
	764633	3544776	extended						
	764634	3544777	extended						
	764635	3544778	extended						
	764636	3544779	extended						
	764637	3544780	extended						
		3544781	core						
		3544782	extended						
		3544783	full						
		3544784	extended						
		3544785	core						
		3544786	core						

		3544787<br>3544788<br>3544789<br>3544790<br>3544791<br>3544792<br>3544793<br>3544794<br>3544795<br>3544796<br>3544797<br>3544798<br>3544799<br>3544800<br>3544801<br>3544802<br>3544803<br>3544804<br>3544805<br>3544806<br>3544807<br>3544808<br>3544809<br>3544810<br>3544811<br>3544812<br>3544813<br>3544814<br>3544815<br>3544816	extended<br>core<br>core<br>extended<br>extended<br>full<br>core<br>extended<br>extended<br>full<br>extended<br>full<br>full<br>full<br>extended<br>full<br>full<br>full<br>full<br>full<br>full<br>extended<br>extended<br>extended<br>core<br>core<br>extended<br>extended<br>extended<br>extended						
3556990	772051<br>772052<br>772053<br>772054<br>772055<br>772056<br>772057<br>772058<br>772059<br>772060<br>772061<br>772062	3556991<br>3556992<br>3556993<br>3556994<br>3556995<br>3556996<br>3556997<br>3556998<br>3556999<br>3557000<br>3557001<br>3557002<br>3557003<br>3557004<br>3557005<br>3557006<br>3557007<br>3557008<br>3557009<br>3557010<br>3557011<br>3557012<br>3557013<br>3557014	extended<br>extended<br>core<br>core<br>core<br>core<br>core<br>core<br>core<br>core<br>core<br>full<br>core<br>core<br>core<br>core<br>extended<br>extended<br>core<br>core<br>core<br>core<br>core<br>extended	14	22506538	22521696	-	NM_032876<br>NM_198086<br>ENSESTT00000058313<br>ENST00000262713<br>ENST00000361265<br>GENSCAN00000022101	Homo sapiens jub, ajuba homolog (Xenopus laevis) (JUB), transcript variant 1, mRNA.<br>Homo sapiens jub, ajuba homolog (Xenopus laevis) (JUB), transcript variant 2, mRNA.<br><br>cdna:known-ccds chromosome:NCBI36:14:22510249:22521691:-1 gene:ENSG00000129474 CCDS9581.1<br>cdna:known-ccds chromosome:NCBI36:14:22510249:22521691:-1 gene:ENSG00000129474 CCDS9582.1<br>cdna:Genscan chromosome:NCBI36:14:22485574:22538250:-1

3557851	772510<br>772511<br>772512<br>772513<br>772514<br>772515<br>772516<br>772517<br>772518<br>772519<br>772520<br>772521<br>772522<br>772523<br>772524<br>772525<br>772526<br>772527<br>772528<br>772529<br>772530<br>772531<br>772532<br>772533<br>772534<br>772535<br>772536<br>772537<br>772538<br>772539<br>772540<br>772541<br>772542	3557852<br>3557853<br>3557854<br>3557855<br>3557856<br>3557857<br>3557858<br>3557859<br>3557860<br>3557861<br>3557862<br>3557863<br>3557864<br>3557865<br>3557866<br>3557867<br>3557868<br>3557869<br>3557870<br>3557871<br>3557872<br>3557873<br>3557874<br>3557875<br>3557876<br>3557877<br>3557878<br>3557879<br>3557880<br>3557881<br>3557882<br>3557883<br>3557884<br>3557885<br>3557886<br>3557887<br>3557888<br>3557889<br>3557890<br>3557891<br>3557892<br>3557893<br>3557894<br>3557895<br>3557896<br>3557897	extended<br>core<br>core<br>core<br>core<br>core<br>full<br>core<br>core<br>core<br>core<br>extended<br>core<br>extended<br>core<br>core<br>core<br>core<br>core<br>core<br>core<br>core<br>core<br>core<br>core<br>full<br>core<br>core<br>core<br>core<br>core<br>core<br>core<br>core<br>core<br>core<br>core<br>full<br>core<br>core<br>extended<br>core<br>core<br>extended<br>core<br>extended	14	23719103	23727944	-	NM_024658<br>AB209476<br>AK094897<br>ENSESTT00000007467<br>ENSESTT00000007468<br>ENSESTT00000007469<br>ENST00000354464<br>GENSCAN00000004003	Homo sapiens importin 4 (IPO4), mRNA.<br>Homo sapiens mRNA for importin 4 variant protein.<br>Homo sapiens cDNA FLJ37578 fis, clone BRCOC2003732.<br><br><br><br>cdna:known chromosome:NCBI36:14:23719488:23727833:-1 gene:ENSG00000196497<br>cdna:Genscan chromosome:NCBI36:14:23719488:23744036:-1
3565571	777200<br>777201<br>777202<br>777203<br>777204<br>777205<br>777206<br>777207	3565572<br>3565573<br>3565574<br>3565575<br>3565576<br>3565577<br>3565578<br>3565579	extended<br>extended<br>extended<br>core<br>core<br>core<br>extended<br>core	14	54475358	54564164	-	NM_001008396<br>NM_007086<br>AK001620<br>AY517556<br>ENSESTT00000031883<br>ENSESTT00000031884<br>ENSESTT00000031885<br>ENSESTT00000031886	Homo sapiens WD repeat and HMG-box DNA binding protein 1 (WDHD1), transcript variant 2, mRNA.<br>Homo sapiens WD repeat and HMG-box DNA binding protein 1 (WDHD1), transcript variant 1, mRNA.<br>Homo sapiens cDNA FLJ10758 fis, clone NT2RP3004594, highly similar to Homo sapiens mRNA for AND-1 protein.<br>Homo sapiens hypothetical rhabdomyosarcoma antigen MU-RMS-40.1 mRNA, partial cds.

	777208<br>777209<br>777210<br>777211<br>777212<br>777213<br>777214<br>777215<br>777216<br>777217<br>777218<br>777219<br>777220<br>777221<br>777222<br>777223<br>777224<br>777225<br>777226<br>777227<br>777228<br>777229<br>777230<br>777231<br>777232<br>777233	3565580<br>3565581<br>3565582<br>3565583<br>3565584<br>3565585<br>3565586<br>3565587<br>3565588<br>3565589<br>3565590<br>3565591<br>3565592<br>3565593<br>3565594<br>3565595<br>3565596<br>3565597<br>3565598<br>3565599<br>3565600<br>3565601<br>3565602<br>3565603<br>3565604<br>3565605<br>3565606<br>3565607<br>3565608<br>3565609<br>3565610<br>3565611<br>3565612<br>3565613<br>3565614<br>3565615<br>3565616<br>3565617<br>3565618<br>3565619<br>3565620<br>3565621<br>3565622<br>3565623	core<br>core<br>core<br>core<br>core<br>core<br>core<br>core<br>extended<br>full<br>core<br>extended<br>extended<br>core<br>core<br>core<br>core<br>core<br>core<br>core<br>core<br>core<br>core<br>extended<br>extended<br>extended<br>extended<br>extended<br>extended<br>extended<br>extended<br>extended<br>extended<br>core<br>core<br>full<br>core<br>core<br>core<br>core<br>extended<br>extended<br>extended<br>full					ENST00000360586<br>ENST00000359167	cdna:known-ccds chromosome:NCBI36:14:54476692:54563557:-1 gene:ENSG00000198554 CCDS9721.1<br>cdna:novel chromosome:NCBI36:14:54476692:54563557:-1 gene:ENSG00000198554
3568616	779089<br>779090<br>779091<br>779092<br>779093<br>779094<br>779095<br>779096<br>779097<br>779098	3568617<br>3568618<br>3568619<br>3568620<br>3568621<br>3568622<br>3568623<br>3568624<br>3568625<br>3568626	full<br>full<br>core<br>core<br>core<br>core<br>core<br>core<br>core<br>core	14	64479707	64509231	-	NM_198686<br>BX640825<br>BC014511<br>ENSESTT00000003672<br>ENSESTT00000003673<br>ENSESTT00000003674<br>ENST00000267512<br>GENSCAN00000006565	Homo sapiens RAB15, member RAS onocogene family (RAB15), mRNA.<br>Homo sapiens mRNA; cDNA DKFZp686J06205 (from clone DKFZp686J06205).<br>Homo sapiens RAB15, member RAS onocogene family, mRNA (cDNA clone IMAGE:4866926), with apparent retained intron.<br><br><br><br>cdna:known-ccds chromosome:NCBI36:14:64482288:64508551:-1 gene:ENSG00000139998 CCDS9768.1<br>cdna:Genscan chromosome:NCBI36:14:64484825:64509231:-1

	779099<br>779100<br>779101<br>779102<br>779103<br>779104<br>779105<br>779106<br>779107<br>779108<br>779109<br>779110<br>779111<br>779112<br>779113<br>779114	3568627<br>3568628<br>3568629<br>3568630<br>3568631<br>3568632<br>3568633<br>3568634<br>3568635<br>3568636<br>3568637<br>3568638<br>3568639<br>3568640<br>3568641<br>3568642<br>3568643<br>3568644<br>3568645<br>3568646<br>3568647<br>3568648<br>3568649<br>3568650<br>3568651<br>3568652<br>3568653<br>3568654<br>3568655<br>3568656<br>3568657	core<br>core<br>core<br>core<br>full<br>core<br>extended<br>core<br>core<br>core<br>full<br>full<br>core<br>full<br>full<br>full<br>extended<br>extended<br>full<br>full<br>full<br>full<br>extended<br>full<br>full<br>extended<br>core<br>core<br>extended<br>extended<br>full						
3569814	779802<br>779803<br>779804<br>779805<br>779806<br>779807<br>779808<br>779809<br>779810<br>779811<br>779812<br>779813<br>779814<br>779815<br>779816<br>779817<br>779818<br>779819<br>779820<br>779821<br>779822<br>779823<br>779824	3569815<br>3569816<br>3569817<br>3569818<br>3569819<br>3569820<br>3569821<br>3569822<br>3569823<br>3569824<br>3569825<br>3569826<br>3569827<br>3569828<br>3569829<br>3569830<br>3569831<br>3569832<br>3569833<br>3569834<br>3569835<br>3569836<br>3569837	extended<br>extended<br>extended<br>core<br>core<br>core<br>core<br>core<br>extended<br>extended<br>core<br>extended<br>extended<br>extended<br>extended<br>core<br>extended<br>core<br>full<br>core<br>core<br>core<br>full	14	68409817	68515815	-	XM_927071<br>XM_938941<br>NM_001102<br>AB209245<br>AK022224<br>AK098203<br>AK124378<br>BC070154<br>BC107741<br>BX641076<br>M95178<br>BX248020<br>CR620987<br>ENSESTT00000029087<br>ENSESTT00000029088<br>ENSESTT00000029089<br>ENST00000193403<br>ENST00000378969<br>ENST00000380748<br>GENSCAN00000045145	PREDICTED: Homo sapiens similar to Nonhistone chromosomal protein HMG-14 (High-mobility group nucleosome binding domain 1) (LOC643790), mRNA.<br>PREDICTED: Homo sapiens similar to Nonhistone chromosomal protein HMG-14 (High-mobility group nucleosome binding domain 1) (LOC649866), mRNA.<br>Homo sapiens actinin, alpha 1 (ACTN1), mRNA.<br>Homo sapiens mRNA for Hypothetical protein FLJ31471 variant protein.<br>Homo sapiens cDNA FLJ12162 fis, clone MAMMA1000583.<br>Homo sapiens cDNA FLJ40884 fis, clone UTERU2000607, highly similar to ALPHA-ACTININ, SMOOTH MUSCLE ISOFORM.<br>Homo sapiens cDNA FLJ42387 fis, clone UTERU2037791, highly similar to Human mRNA for alpha-actinin.<br>Homo sapiens high-mobility group nucleosome binding domain 1, mRNA (cDNA clone MGC:88134 IMAGE:6495327), complete cds.<br>Homo sapiens high-mobility group nucleosome binding domain 1, mRNA (cDNA clone MGC:104230 IMAGE:6495117), complete cds.<br>Homo sapiens mRNA; cDNA DKFZp686J22226 (from clone DKFZp686J22226).<br>Human non-muscle alpha-actinin mRNA, complete cds.<br>human full-length cDNA clone CS0DB009YA03 of Neuroblastoma of Homo sapiens (human).<br>full-length cDNA clone CS0DM010YM22 of Fetal liver of Homo sapiens (human).<br>cdna:known-ccds chromosome:NCBI36:14:68410793:68515747:-1 gene:ENSG00000072110 CCDS9792.1<br>cdna:novel chromosome:NCBI36:15:91055981:91057109:1 gene:ENSG00000157576

	779825	3569838	extended						cdna:novel chromosome:NCBI36:21:39636488:39642849:-1 gene:ENSG00000205581
	779826	3569839	extended						cdna:Genscan chromosome:NCBI36:14:68411329:68542331:-1
	779827	3569840	core						
	779828	3569841	core						
	779829	3569842	core						
	779830	3569843	extended						
	779831	3569844	extended						
	779832	3569845	extended						
	779833	3569846	core						
	779834	3569847	core						
	779835	3569848	full						
	779836	3569849	core						
	779837	3569850	core						
	779838	3569851	full						
	779839	3569852	full						
	779840	3569853	extended						
	779841	3569854	full						
	779842	3569855	extended						
	779843	3569856	extended						
	779844	3569857	extended						
	779845	3569858	full						
	779846	3569859	core						
	779847	3569860	core						
	779848	3569861	full						
	779849	3569862	core						
	779850	3569863	extended						
	779851	3569864	core						
	779852	3569865	extended						
	779853	3569866	core						
	779854	3569867	extended						
	779855	3569868	extended						
	779856	3569869	core						
	779857	3569870	extended						
	779858	3569871	core						
	779859	3569872	extended						
	779860	3569873	core						
	779861	3569874	full						
	779862	3569875	full						
	779863	3569876	full						
	779864	3569877	full						
	779865	3569878	full						
		3569879	extended						
		3569880	full						
		3569881	full						
		3569882	full						
		3569883	full						
		3569884	full						
		3569885	full						
		3569886	full						
		3569887	full						
		3569888	full						
		3569889	full						
		3569890	extended						
		3569891	extended						

		3569892<br>3569893<br>3569894<br>3569895<br>3569896<br>3569897<br>3569898<br>3569899<br>3569900<br>3569901<br>3569902<br>3569903<br>3569904<br>3569905<br>3569906<br>3569907<br>3569908<br>3569909<br>3569910	full<br>full<br>full<br>full<br>full<br>full<br>full<br>extended<br>full<br>full<br>extended<br>extended<br>extended<br>extended<br>extended<br>full<br>core<br>core<br>extended						
3571667	780962<br>780963<br>780964<br>780965<br>780966<br>780967<br>780968<br>780969<br>780970<br>780971<br>780972<br>780973<br>780974<br>780975<br>780976<br>780977<br>780978<br>780979<br>780980<br>780981<br>780982<br>780983<br>780984<br>780985<br>780986<br>780987<br>780988<br>780989<br>780990<br>780991<br>780992<br>780993<br>780994<br>780995<br>780996	3571668<br>3571669<br>3571670<br>3571671<br>3571672<br>3571673<br>3571674<br>3571675<br>3571676<br>3571677<br>3571678<br>3571679<br>3571680<br>3571681<br>3571682<br>3571683<br>3571684<br>3571685<br>3571686<br>3571687<br>3571688<br>3571689<br>3571690<br>3571691<br>3571692<br>3571693<br>3571694<br>3571695<br>3571696<br>3571697<br>3571698<br>3571699<br>3571700<br>3571701<br>3571702	extended<br>core<br>extended<br>extended<br>extended<br>extended<br>extended<br>extended<br>extended<br>extended<br>extended<br>core<br>full<br>full<br>core<br>core<br>core<br>core<br>core<br>full<br>core<br>full<br>core<br>core<br>extended<br>core<br>extended<br>core<br>full<br>extended<br>core<br>extended<br>extended<br>core<br>core	14	73494392	73555748	-	NM_001249<br>AK025209<br>BC020966<br>ENSESTT00000025801<br>ENSESTT00000025802<br>ENSESTT00000025803<br>ENSESTT00000025804<br>ENSESTT00000025805<br>ENST00000334696<br>ENST00000381104<br>GENSCAN00000035247	Homo sapiens ectonucleoside triphosphate diphosphohydrolase 5 (ENTPD5), mRNA.<br>Homo sapiens cDNA: FLJ21556 fis, clone COL06353.<br>Homo sapiens ectonucleoside triphosphate diphosphohydrolase 5, mRNA (cDNA clone IMAGE:3847878), with apparent retained intron.<br><br><br><br><br><br>cdna:known-ccds chromosome:NCBI36:14:73502946:73552387:-1 gene:ENSG00000187097 CCDS9825.1<br>cdna:novel chromosome:NCBI36:14:73502946:73552387:-1 gene:ENSG00000187097<br>cdna:Genscan chromosome:NCBI36:14:73499913:73532434:-1

	780997	3571703<br>3571704<br>3571705<br>3571706<br>3571707<br>3571708<br>3571709<br>3571710<br>3571711<br>3571712<br>3571713<br>3571714	core<br>full<br>extended<br>extended<br>full<br>full<br>extended<br>core<br>extended<br>core<br>core<br>extended						
3571944	781128<br>781129<br>781130<br>781131<br>781132<br>781133<br>781134<br>781135<br>781136<br>781137<br>781138<br>781139<br>781140<br>781141<br>781142<br>781143<br>781144<br>781145<br>781146<br>781147<br>781148<br>781149<br>781150<br>781151<br>781152<br>781153<br>781154<br>781155<br>781156<br>781157<br>781158<br>781159<br>781160<br>781161<br>781162<br>781163<br>781164<br>781165<br>781166<br>781167<br>781168<br>781169	3571945<br>3571946<br>3571947<br>3571948<br>3571949<br>3571950<br>3571951<br>3571952<br>3571953<br>3571954<br>3571955<br>3571956<br>3571957<br>3571958<br>3571959<br>3571960<br>3571961<br>3571962<br>3571963<br>3571964<br>3571965<br>3571966<br>3571967<br>3571968<br>3571969<br>3571970<br>3571971<br>3571972<br>3571973<br>3571974<br>3571975<br>3571976<br>3571977<br>3571978<br>3571979<br>3571980<br>3571981<br>3571982<br>3571983<br>3571984<br>3571985<br>3571986	core<br>core<br>core<br>core<br>core<br>extended<br>core<br>extended<br>core<br>core<br>core<br>core<br>core<br>core<br>core<br>core<br>full<br>core<br>core<br>core<br>core<br>core<br>full<br>core<br>core<br>core<br>core<br>core<br>full<br>core<br>full<br>core<br>core<br>full<br>core<br>core<br>full<br>extended<br>extended<br>extended<br>extended<br>extended	14	74034646	74156510	-	NM_000428<br>BC032434<br>ENSESTT00000050195<br>ENSESTT00000050196<br>ENSESTT00000050197<br>ENSESTT00000050198<br>ENST00000261978<br>GENSCAN00000030992<br>GENSCAN00000022517	Homo sapiens latent transforming growth factor beta binding protein 2 (LTBP2), mRNA.<br>Homo sapiens latent transforming growth factor beta binding protein 2, mRNA (cDNA clone IMAGE:4471812), partial cds.<br><br><br><br><br>cdna:known-ccds chromosome:NCBI36:14:74034642:74148787:-1 gene:ENSG00000119681 CCDS9831.1<br>cdna:Genscan chromosome:NCBI36:14:74037340:74069004:-1<br>cdna:Genscan chromosome:NCBI36:14:74141434:74141905:-1

	781170<br>781171<br>781172<br>781173<br>781174<br>781175<br>781176<br>781177<br>781178<br>781179<br>781180<br>781181<br>781182<br>781183<br>781184<br>781185<br>781186<br>781187<br>781188<br>781189<br>781190	3571987<br>3571988<br>3571989<br>3571990<br>3571991<br>3571992<br>3571993<br>3571994<br>3571995<br>3571996<br>3571997<br>3571998<br>3571999<br>3572000<br>3572001<br>3572002<br>3572003<br>3572004<br>3572005<br>3572006<br>3572007<br>3572008<br>3572009<br>3572010<br>3572011<br>3572012<br>3572013<br>3572014<br>3572015<br>3572016<br>3572017<br>3572018<br>3572019<br>3572020<br>3572021<br>3572022<br>3572023<br>3572024<br>3572025<br>3572026<br>3572027<br>3572028<br>3572029<br>3572030	extended<br>extended<br>core<br>core<br>core<br>full<br>core<br>full<br>full<br>extended<br>full<br>full<br>core<br>core<br>core<br>full<br>core<br>core<br>core<br>full<br>extended<br>full<br>full<br>extended<br>full<br>core<br>full<br>full<br>core<br>extended<br>full<br>full<br>full<br>full<br>full<br>full<br>full<br>core<br>core<br>core<br>full<br>full<br>full<br>full						
3573870	782342<br>782343<br>782344<br>782345<br>782346<br>782347<br>782348<br>782349<br>782350<br>782351	3573871<br>3573872<br>3573873<br>3573874<br>3573875<br>3573876<br>3573877<br>3573878<br>3573879<br>3573880	full<br>full<br>full<br>full<br>full<br>full<br>core<br>core<br>core<br>core	14	79703503	79748573	-	NM_013989<br>NM_000793<br>NM_001007023<br>AB041843<br>AB041844<br>BC063118<br>ENSESTT00000052738<br>ENSESTT00000052739<br>ENSESTT00000052740<br>ENSESTT00000052741	Homo sapiens deiodinase, iodothyronine, type II (DIO2), transcript variant 1, mRNA.<br>Homo sapiens deiodinase, iodothyronine, type II (DIO2), transcript variant 2, mRNA.<br>Homo sapiens deiodinase, iodothyronine, type II (DIO2), transcript variant 3, mRNA.<br>Homo sapiens mRNA for type II iodothyronine deiodinase, complete cds.<br>Homo sapiens mRNA for type II iodothyronine deiodinase, complete cds.<br>Homo sapiens deiodinase, iodothyronine, type II, mRNA (cDNA clone IMAGE:30351753), complete cds.

	782352<br>782353<br>782354<br>782355<br>782356<br>782357<br>782358<br>782359	3573881<br>3573882<br>3573883<br>3573884<br>3573885<br>3573886<br>3573887<br>3573888<br>3573889<br>3573890<br>3573891<br>3573892<br>3573893<br>3573894<br>3573895<br>3573896	core<br>core<br>core<br>core<br>core<br>extended<br>core<br>core<br>full<br>extended<br>core<br>core<br>core<br>core<br>core<br>extended					ENSESTT00000052742<br>ENST00000388841<br>ENST00000388840<br>ENST00000388838<br>ENST00000388839<br>GENSCAN00000064920<br>GENSCAN00000064922	 cdna:known chromosome:NCBI36:14:79736595:79747604:-1 gene:ENSG00000211448<br>cdna:known chromosome:NCBI36:14:79738694:79747596:-1 gene:ENSG00000211448<br>cdna:known chromosome:NCBI36:14:79738809:79747568:-1 gene:ENSG00000211448<br>cdna:known chromosome:NCBI36:14:79738915:79747409:-1 gene:ENSG00000211448<br>cdna:Genscan chromosome:NCBI36:14:79703503:79736354:-1<br>cdna:Genscan chromosome:NCBI36:14:79738809:79747568:-1
3577443	784685<br>784686<br>784687<br>784688<br>784689<br>784690<br>784691<br>784692<br>784693<br>784694<br>784695<br>784696<br>784697<br>784698<br>784699<br>784700<br>784701<br>784702<br>784703	3577444<br>3577445<br>3577446<br>3577447<br>3577448<br>3577449<br>3577450<br>3577451<br>3577452<br>3577453<br>3577454<br>3577455<br>3577456<br>3577457<br>3577458<br>3577459<br>3577460<br>3577461<br>3577462<br>3577463<br>3577464<br>3577465<br>3577466<br>3577467<br>3577468<br>3577469<br>3577470<br>3577471	core<br>core<br>core<br>core<br>extended<br>core<br>extended<br>extended<br>core<br>extended<br>core<br>full<br>core<br>full<br>core<br>full<br>core<br>extended<br>core<br>core<br>core<br>core<br>full<br>extended<br>extended<br>extended<br>extended<br>extended	14	93470270	93512829	-	NM_016150<br>AK002049<br>AK057078<br>AK093271<br>ENSESTT00000021177<br>ENSESTT00000021178<br>ENSESTT00000021179<br>ENSESTT00000021180<br>ENSESTT00000021181<br>ENST00000315988	Homo sapiens ankyrin repeat and SOCS box-containing 2 (ASB2), mRNA.<br>Homo sapiens cDNA FLJ11187 fis, clone PLACE1007537, highly similar to Homo sapiens ankyrin repeat-containing protein ASB-2 mRNA.<br>Homo sapiens cDNA FLJ32516 fis, clone SMINT1000103, highly similar to Homo sapiens ankyrin repeat-containing protein ASB-2 mRNA.<br>Homo sapiens cDNA FLJ35952 fis, clone TESTI2012152, highly similar to Homo sapiens ankyrin repeat-containing protein ASB-2 mRNA.<br><br><br><br><br><br>cdna:known-ccds chromosome:NCBI36:14:93470266:93493520:-1 gene:ENSG00000100628 CCDS9915.1
3581485	787095<br>787096<br>787097<br>787098<br>787099<br>787100<br>787101<br>787102<br>787103	3581486<br>3581487<br>3581488<br>3581489<br>3581490<br>3581491<br>3581492<br>3581493<br>3581494	core<br>core<br>core<br>extended<br>extended<br>extended<br>core<br>extended<br>core	14	104710321	104720367	-	NM_177533<br>BC041584<br>ENSESTT00000049520<br>ENSESTT00000049523<br>ENST00000339418<br>GENSCAN00000030098	Homo sapiens nudix (nucleoside diphosphate linked moiety X)-type motif 14 (NUDT14), mRNA.<br>Homo sapiens nudix (nucleoside diphosphate linked moiety X)-type motif 14, mRNA (cDNA clone MGC:52411 IMAGE:4810207), complete cds.<br><br><br>cdna:known-ccds chromosome:NCBI36:14:104710322:104718685:-1 gene:ENSG00000183828 CCDS10000.1<br>cdna:Genscan chromosome:NCBI36:14:104710403:104779116:-1

	787104	3581495	core						
		3581496	extended						
		3581497	core						
		3581498	core						
		3581499	core						
		3581500	extended						
		3581501	extended						
3581637	787180	3581638	extended	14	105120712	106352439	-	XM_370833	PREDICTED: Homo sapiens similar to ribosomal protein S8, transcript variant 1 (LOC388076), mRNA.
	787181	3581639	extended					XM_931016	PREDICTED: Homo sapiens similar to ribosomal protein S8, transcript variant 2 (LOC388076), mRNA.
	787182	3581640	extended					XM_929297	PREDICTED: Homo sapiens similar to Ig heavy chain V-II region ARH-77 precursor (LOC646372), mRNA.
	787183	3581641	extended					XM_942073	PREDICTED: Homo sapiens hypothetical protein LOC652566 (LOC652566), mRNA.
	787184	3581642	core					XM_930154	PREDICTED: Homo sapiens similar to Ig heavy chain V-I region V35 precursor (LOC647133), mRNA.
	787185	3581643	core					XM_942618	PREDICTED: Homo sapiens similar to a disintegrin and metallopeptidase domain 6 (LOC652892), mRNA.
	787186	3581644	core					XM_930155	PREDICTED: Homo sapiens similar to Ig heavy chain V-III region VH26 precursor (LOC647134), mRNA.
	787187	3581645	core					XM_377426	PREDICTED: Homo sapiens similar to Ig heavy chain V-II region SESS precursor (LOC401845), mRNA.
	787188	3581646	core					XM_370973	PREDICTED: Homo sapiens similar to Ig heavy chain V-III region VH26 precursor (LOC388255), mRNA.
	787189	3581647	core					XM_941249	PREDICTED: Homo sapiens similar to Ig heavy chain V-I region HG3 precursor (LOC651963), mRNA.
	787190	3581648	core					NM_001040070	Homo sapiens similar to Ig gamma-2 chain C region (LOC649897), mRNA.
	787191	3581649	core					XM_372543	PREDICTED: Homo sapiens similar to Ig heavy chain V-I region HG3 precursor (LOC390530), mRNA.
	787192	3581650	core					NR_002224	Homo sapiens ADAM metallopeptidase domain 6 (ADAM6) on chromosome 14.
	787193	3581651	extended					NM_001012	Homo sapiens ribosomal protein S8 (RPS8), mRNA.
	787194	3581652	extended					XM_941956	PREDICTED: Homo sapiens similar to Ig heavy chain V-III region VH26 precursor (LOC652494), mRNA.
	787195	3581653	full					XM_941959	PREDICTED: Homo sapiens similar to Ig gamma-3 chain C region, membrane-bound form (LOC652495), mRNA.
	787196	3581654	full					XM_938317	PREDICTED: Homo sapiens similar to Ig heavy chain V-II region SESS precursor (LOC401845), mRNA.
	787197	3581655	full					NM_001040077	Homo sapiens anti-rabies SO57 immunoglobulin heavy chain (IGHG1), mRNA.
	787198	3581656	full					XM_942215	PREDICTED: Homo sapiens similar to Ig heavy chain V-III region VH26 precursor (LOC652651), mRNA.
	787199	3581657	full					XM_940414	PREDICTED: Homo sapiens similar to Ig gamma-2B chain C region, membrane-bound form (LOC651286), mRNA.
	787200	3581658	full					XM_936258	PREDICTED: Homo sapiens similar to ribosomal protein S8 (LOC642115), mRNA.
	787201	3581659	full					XM_936278	PREDICTED: Homo sapiens similar to Ig heavy chain V-II region ARH-77 precursor (LOC642131), mRNA.
	787202	3581660	extended					XM_940985	PREDICTED: Homo sapiens similar to Ig gamma-4 chain C region (LOC651766), mRNA.
	787203	3581661	extended					XM_941366	PREDICTED: Homo sapiens similar to Ig gamma-1 chain C region (LOC652050), mRNA.
	787204	3581662	extended					XM_941385	PREDICTED: Homo sapiens similar to Ig heavy chain V-II region ARH-77 precursor (LOC652065), mRNA.
	787205	3581663	extended					XM_938874	PREDICTED: Homo sapiens similar to Ig heavy chain V-II region SESS precursor (LOC649805), mRNA.
	787206	3581664	extended					XM_941391	PREDICTED: Homo sapiens similar to Ig heavy chain V-II region ARH-77 precursor (LOC652068), mRNA.
	787207	3581665	extended					XM_941404	PREDICTED: Homo sapiens similar to Ig heavy chain V-III region VH26 precursor (LOC652080), mRNA.
	787208	3581666	extended					XM_941407	PREDICTED: Homo sapiens similar to Ig heavy chain V-II region ARH-77 precursor (LOC652082), mRNA.
	787209	3581667	extended					XM_941434	PREDICTED: Homo sapiens similar to Ig heavy chain V-I region HG3 precursor (LOC652102), mRNA.
	787210	3581668	extended					XM_941436	PREDICTED: Homo sapiens similar to Ig heavy chain V-III region VH26 precursor (LOC652106), mRNA.
	787211	3581669	full					XM_941444	PREDICTED: Homo sapiens similar to Ig heavy chain V-III region VH26 precursor (LOC652113), mRNA.
	787212	3581670	extended					XM_941448	PREDICTED: Homo sapiens similar to Ig heavy chain V region 102 precursor (LOC652114), mRNA.
	787213	3581671	extended					XM_941463	PREDICTED: Homo sapiens similar to Ig heavy chain V-I region V35 precursor (LOC652126), mRNA.
	787214	3581672	extended					XM_941465	PREDICTED: Homo sapiens similar to Ig heavy chain V-II region ARH-77 precursor (LOC652128), mRNA.
	787215	3581673	extended					XM_941478	PREDICTED: Homo sapiens similar to Ig heavy chain V-III region VH26 precursor (LOC652141), mRNA.
	787216	3581674	extended					XM_941494	PREDICTED: Homo sapiens similar to Ig heavy chain V-I region V35 precursor (LOC652152), mRNA.
	787217	3581675	extended					XM_941499	PREDICTED: Homo sapiens similar to Ig heavy chain V-I region HG3 precursor (LOC652155), mRNA.
	787218	3581676	extended					XM_946339	PREDICTED: Homo sapiens hypothetical protein MGC27165, transcript variant 2 (MGC27165), mRNA.
	787219	3581677	extended					XM_941503	PREDICTED: Homo sapiens similar to Ig heavy chain V-III region VH26 precursor (LOC652159), mRNA.
	787220	3581678	extended					XM_942451	PREDICTED: Homo sapiens similar to Ig heavy chain V-III region VH26 precursor (LOC652791), mRNA.
	787221	3581679	full					XM_942723	PREDICTED: Homo sapiens hypothetical protein MGC27165, transcript variant 1 (MGC27165), mRNA.
	787222	3581680	extended					XM_941506	PREDICTED: Homo sapiens similar to Ig heavy chain V-III region VH26 precursor (LOC652161), mRNA.
	787223	3581681	full					XM_946340	PREDICTED: Homo sapiens hypothetical protein MGC27165, transcript variant 3 (MGC27165), mRNA.
	787224	3581682	full					XM_939003	PREDICTED: Homo sapiens similar to Ig gamma-2 chain C region (LOC649923), mRNA.
	787225	3581683	full					XM_496025	PREDICTED: Homo sapiens similar to Ig heavy chain V-I region HG3 precursor (LOC390531), mRNA.
	787226	3581684	extended					XM_929032	PREDICTED: Homo sapiens similar to a disintegrin and metallopeptidase domain 6 (LOC646071), mRNA.

	787227	3581685	full					AB004303	Homo sapiens mRNA for IgG heavy chain, partial cds.
	787228	3581686	extended					AB021508	Homo sapiens mRNA for immunoglobulin heavy chain variable region (IgM), partial cds, clone kgpbmc15.
	787229	3581687	extended					AB021509	Homo sapiens mRNA for immunoglobulin heavy chain variable region (IgM), partial cds, clone kgpbmc17.
	787230	3581688	extended					AB021511	Homo sapiens mRNA for immunoglobulin heavy chain variable region (IgM), partial cds, clone kgpbmc26.
	787231	3581689	extended					AB021512	Homo sapiens mRNA for immunoglobulin heavy chain variable region (IgM), partial cds, clone kgpbmc27.
	787232	3581690	extended					AB021517	Homo sapiens mRNA for immunoglobulin heavy chain variable region (IgM), partial cds, clone kgpbmc6.
	787233	3581691	core					AB021518	Homo sapiens mRNA for immunoglobulin heavy chain variable region (IgM), partial cds, clone kgpbmc61.
	787234	3581692	core					AB021519	Homo sapiens mRNA for immunoglobulin heavy chain variable region (IgM), partial cds, clone kgpbmc75.
	787235	3581693	core					AB021520	Homo sapiens mRNA for immunoglobulin heavy chain variable region (IgM), partial cds, clone kgpbmc45.
	787236	3581694	core					AB021522	Homo sapiens mRNA for immunoglobulin heavy chain variable region (IgM), partial cds, clone kgpbmc79.
	787237	3581695	core					AB021523	Homo sapiens mRNA for immunoglobulin heavy chain variable region (IgM), partial cds, clone kgpbmc84.
	787238	3581696	core					AB021525	Homo sapiens mRNA for immunoglobulin heavy chain variable region (IgM), partial cds, clone krpbmc1.
	787239	3581697	core					AB021527	Homo sapiens mRNA for immunoglobulin heavy chain variable region (IgM), partial cds, clone krpbmc23.
	787240	3581698	full					AB021528	Homo sapiens mRNA for immunoglobulin heavy chain variable region (IgM), partial cds, clone krpbmc26.
	787241	3581699	full					AB021530	Homo sapiens mRNA for immunoglobulin heavy chain variable region (IgM), partial cds, clone krpbmc32.
	787242	3581700	extended					AB021532	Homo sapiens mRNA for immunoglobulin heavy chain variable region (IgM), partial cds, clone krpbmc39.
	787243	3581701	extended					AB021533	Homo sapiens mRNA for immunoglobulin heavy chain variable region (IgM), partial cds, clone krpbmc3.
	787244	3581702	full					AB021534	Homo sapiens mRNA for immunoglobulin heavy chain variable region (IgM), partial cds, clone krpbmc41.
	787245	3581703	full					AB021535	Homo sapiens mRNA for immunoglobulin heavy chain variable region (IgM), partial cds, clone krpbmc46.
	787246	3581704	full					AB021536	Homo sapiens mRNA for immunoglobulin heavy chain variable region (IgM), partial cds, clone krpbmc54.
	787247	3581705	core					AB021538	Homo sapiens mRNA for immunoglobulin heavy chain variable region (IgM), partial cds, clone krpbmc61.
	787248	3581706	core					AB021539	Homo sapiens mRNA for immunoglobulin heavy chain variable region (IgM), clone krpbmc63.
	787249	3581707	core					AB021541	Homo sapiens mRNA for immunoglobulin heavy chain variable region (IgM), partial cds, clone krpbmc72.
	787250	3581708	core					AB022652	Homo sapiens mRNA for anti-Entamoeba histolytica immunoglobulin gamma heavy chain (V-CH1 region), partial cds,
	787251	3581709	extended					AB022655	clone:B220-H2.
	787252	3581710	core					AB027435	Homo sapiens mRNA for anti-Entamoeba histolytica immunoglobulin gamma heavy chain (V-CH1 region), partial cds,
	787253	3581711	core					AB027437	clone:E244-H1.
	787254	3581712	core					AB027439	Homo sapiens mRNA for anti TNF-alpha antibody heavy-chain Fab fragment, partial cds, clone:1F8-gk10H.
	787255	3581713	core					AB027441	Homo sapiens mRNA for anti TNF-alpha antibody heavy-chain Fab fragment, partial cds, clone:1F8-gk20H.
	787256	3581714	core					AB027443	Homo sapiens mRNA for anti TNF-alpha antibody heavy-chain Fab fragment, partial cds, clone:1F8-mk26H.
	787257	3581715	free					AB027445	Homo sapiens mRNA for anti HBs antibody heavy-chain Fab fragment, partial cds, clone:303-15H.
	787258	3581716	core					AB027447	Homo sapiens mRNA for anti HBs antibody heavy-chain Fab fragment, partial cds, clone:305-51H.
	787259	3581717	core					AB027766	Homo sapiens mRNA for anti HBs antibody heavy-chain Fab fragment, partial cds, clone:308-09H.
	787260	3581718	core					AB030639	Homo sapiens mRNA for anti HBs antibody heavy-chain Fab fragment, partial cds, clone:308-19H.
	787261	3581719	core					AB062401	Homo sapiens mRNA for antibody heavy-chain Fab, partial cds.
	787262	3581720	core					AB063654	Homo sapiens mRNA for immunoglobulin gamma heavy chain, partial cds, V and CH1 region, specific for Entamoeba histolytica.
	787263	3581721	extended					AB063656	Homo sapiens OK/SW-cl.83 mRNA for ribosomal protein S8, complete cds.
	787264	3581722	extended					AB063658	Homo sapiens IGH mRNA for immunoglobulin heavy chain VHDJ region, partial cds, clone:H4.
	787265	3581723	extended					AB063660	Homo sapiens IGH mRNA for immunoglobulin heavy chain VHDJ region, partial cds, clone:H6.
	787266	3581724	extended					AB063662	Homo sapiens IGH mRNA for immunoglobulin heavy chain VHDJ region, partial cds, clone:H8.
	787267	3581725	full					AB063663	Homo sapiens IGH mRNA for immunoglobulin heavy chain VHDJ region, partial cds, clone:H10.
	787268	3581726	extended					AB063664	Homo sapiens IGH mRNA for immunoglobulin heavy chain VHDJ region, partial cds, clone:H12.
	787269	3581727	extended					AB063665	Homo sapiens IGH mRNA for immunoglobulin heavy chain VHDJ region, partial cds, clone:H13.
	787270	3581728	full					AB063668	Homo sapiens IGH mRNA for immunoglobulin heavy chain VHDJ region, partial cds, clone:H14.
	787271	3581729	extended					AB063671	Homo sapiens IGH mRNA for immunoglobulin heavy chain VHDJ region, partial cds, clone:H15.
	787272	3581730	extended					AB063672	Homo sapiens IGH mRNA for immunoglobulin heavy chain VHDJ region, partial cds, clone:H18.
	787273	3581731	extended					AB063673	Homo sapiens IGH mRNA for immunoglobulin heavy chain VHDJ region, partial cds, clone:H21.
	787274	3581732	extended					AB063674	Homo sapiens IGH mRNA for immunoglobulin heavy chain VHDJ region, partial cds, clone:H22.
	787275	3581733	extended					AB063675	Homo sapiens IGH mRNA for immunoglobulin heavy chain VHDJ region, partial cds, clone:H23.
	787276	3581734	extended					AB063676	Homo sapiens IGH mRNA for immunoglobulin heavy chain VHDJ region, partial cds, clone:H24.
	787277	3581735	extended					AB063678	Homo sapiens IGH mRNA for immunoglobulin heavy chain VHDJ region, partial cds, clone:H25.
	787278	3581736	extended					AB063679	Homo sapiens IGH mRNA for immunoglobulin heavy chain VHDJ region, partial cds, clone:H26.
	787279	3581737	extended					AB063680	Homo sapiens IGH mRNA for immunoglobulin heavy chain VHDJ region, partial cds, clone:H28.
	787280	3581738	core					AB063684	Homo sapiens IGH mRNA for immunoglobulin heavy chain VHDJ region, partial cds, clone:H29.

	787281	3581739	extended					AB063686	Homo sapiens IGH mRNA for immunoglobulin heavy chain VHDJ region, partial cds, clone:H30.
	787282	3581740	extended					AB063694	Homo sapiens IGH mRNA for immunoglobulin heavy chain VHDJ region, partial cds, clone:H34.
	787283	3581741	extended					AB063695	Homo sapiens IGH mRNA for immunoglobulin heavy chain VHDJ region, partial cds, clone:H36.
	787284	3581742	extended					AB063696	Homo sapiens IGH mRNA for immunoglobulin heavy chain VHDJ region, partial cds, clone:H44.
	787285	3581743	extended					AB063697	Homo sapiens IGH mRNA for immunoglobulin heavy chain VHDJ region, partial cds, clone:H45.
	787286	3581744	extended					AB063699	Homo sapiens IGH mRNA for immunoglobulin heavy chain VHDJ region, partial cds, clone:H46.
	787287	3581745	extended					AB063703	Homo sapiens IGH mRNA for immunoglobulin heavy chain VHDJ region, partial cds, clone:H47.
	787288	3581746	extended					AB063704	Homo sapiens IGH mRNA for immunoglobulin heavy chain VHDJ region, partial cds, clone:H49.
	787289	3581747	extended					AB063708	Homo sapiens IGH mRNA for immunoglobulin heavy chain VHDJ region, partial cds, clone:H53.
	787290	3581748	extended					AB063713	Homo sapiens IGH mRNA for immunoglobulin heavy chain VHDJ region, partial cds, clone:H54.
	787291	3581749	extended					AB063717	Homo sapiens IGH mRNA for immunoglobulin heavy chain VHDJ region, partial cds, clone:H58.
	787292	3581750	extended					AB063718	Homo sapiens IGH mRNA for immunoglobulin heavy chain VHDJ region, partial cds, clone:H63.
	787293	3581751	extended					AB063719	Homo sapiens IGH mRNA for immunoglobulin heavy chain VHDJ region, partial cds, clone:H67.
	787294	3581752	extended					AB063720	Homo sapiens IGH mRNA for immunoglobulin heavy chain VHDJ region, partial cds, clone:H68.
	787295	3581753	extended					AB063750	Homo sapiens IGH mRNA for immunoglobulin heavy chain VHDJ region, partial cds, clone:H69.
	787296	3581754	extended					AB063784	Homo sapiens IGH mRNA for immunoglobulin heavy chain VHDJ region, partial cds, clone:H70.
	787297	3581755	extended					AB063786	Homo sapiens IGH mRNA for immunoglobulin heavy chain VHDJ region, partial cds, clone:H100.
	787298	3581756	extended					AB063790	Homo sapiens IGH mRNA for immunoglobulin heavy chain VHDJ region, partial cds, clone:H134.
	787299	3581757	extended					AB063791	Homo sapiens IGH mRNA for immunoglobulin heavy chain VHDJ region, partial cds, clone:H136.
	787300	3581758	extended					AB063792	Homo sapiens IGH mRNA for immunoglobulin heavy chain VHDJ region, partial cds, clone:H140.
	787301	3581759	extended					AB063795	Homo sapiens IGH mRNA for immunoglobulin heavy chain VHDJ region, partial cds, clone:H141.
	787302	3581760	extended					AB063796	Homo sapiens IGH mRNA for immunoglobulin heavy chain VHDJ region, partial cds, clone:H142.
	787303	3581761	extended					AB063797	Homo sapiens IGH mRNA for immunoglobulin heavy chain VHDJ region, partial cds, clone:H145.
	787304	3581762	extended					AB063798	Homo sapiens IGH mRNA for immunoglobulin heavy chain VHDJ region, partial cds, clone:H146.
	787305	3581763	extended					AB063799	Homo sapiens IGH mRNA for immunoglobulin heavy chain VHDJ region, partial cds, clone:H147.
	787306	3581764	extended					AB063800	Homo sapiens IGH mRNA for immunoglobulin heavy chain VHDJ region, partial cds, clone:H148.
	787307	3581765	extended					AB063801	Homo sapiens IGH mRNA for immunoglobulin heavy chain VHDJ region, partial cds, clone:H149.
	787308	3581766	extended					AB063803	Homo sapiens IGH mRNA for immunoglobulin heavy chain VHDJ region, partial cds, clone:H150.
	787309	3581767	extended					AB063804	Homo sapiens IGH mRNA for immunoglobulin heavy chain VHDJ region, partial cds, clone:H151.
	787310	3581768	full					AB063807	Homo sapiens IGH mRNA for immunoglobulin heavy chain VHDJ region, partial cds, clone:H153.
	787311	3581769	full					AB063810	Homo sapiens IGH mRNA for immunoglobulin heavy chain VHDJ region, partial cds, clone:H154.
	787312	3581770	full					AB063811	Homo sapiens IGH mRNA for immunoglobulin heavy chain VHDJ region, partial cds, clone:H157.
	787313	3581771	full					AB063812	Homo sapiens IGH mRNA for immunoglobulin heavy chain VHDJ region, partial cds, clone:H160.
	787314	3581772	full					AB063817	Homo sapiens IGH mRNA for immunoglobulin heavy chain VHDJ region, partial cds, clone:H161.
	787315	3581773	full					AB063824	Homo sapiens IGH mRNA for immunoglobulin heavy chain VHDJ region, partial cds, clone:H162.
	787316	3581774	extended					AB063827	Homo sapiens IGH mRNA for immunoglobulin heavy chain VHDJ region, partial cds, clone:H167.
	787317	3581775	extended					AB063828	Homo sapiens IGH mRNA for immunoglobulin heavy chain VHDJ region, partial cds, clone:H174.
	787318	3581776	extended					AB063833	Homo sapiens IGH mRNA for immunoglobulin heavy chain VHDJ region, partial cds, clone:H177.
	787319	3581777	extended					AB063834	Homo sapiens IGH mRNA for immunoglobulin heavy chain VHDJ region, partial cds, clone:H178.
	787320	3581778	core					AB063838	Homo sapiens IGH mRNA for immunoglobulin heavy chain VHDJ region, partial cds, clone:H183.
	787321	3581779	core					AB063842	Homo sapiens IGH mRNA for immunoglobulin heavy chain VHDJ region, partial cds, clone:H184.
	787322	3581780	core					AB063845	Homo sapiens IGH mRNA for immunoglobulin heavy chain VHDJ region, partial cds, clone:H188.
	787323	3581781	core					AB063849	Homo sapiens IGH mRNA for immunoglobulin heavy chain VHDJ region, partial cds, clone:H192.
	787324	3581782	core					AB063872	Homo sapiens IGH mRNA for immunoglobulin heavy chain VHDJ region, partial cds, clone:H195.
	787325	3581783	core					AB063882	Homo sapiens IGH mRNA for immunoglobulin heavy chain VHDJ region, partial cds, clone:H199.
	787326	3581784	core					AB063889	Homo sapiens IGH mRNA for immunoglobulin heavy chain VHDJ region, partial cds, clone:H222.
	787327	3581785	core					AB063892	Homo sapiens IGH mRNA for immunoglobulin heavy chain VHDJ region, partial cds, clone:H232.
	787328	3581786	extended					AB063894	Homo sapiens IGH mRNA for immunoglobulin heavy chain VHDJ region, partial cds, clone:H239.
	787329	3581787	extended					AB063896	Homo sapiens IGH mRNA for immunoglobulin heavy chain VHDJ region, partial cds, clone:H242.
	787330	3581788	extended					AB063897	Homo sapiens IGH mRNA for immunoglobulin heavy chain VHDJ region, partial cds, clone:H244.
	787331	3581789	core					AB063899	Homo sapiens IGH mRNA for immunoglobulin heavy chain VHDJ region, partial cds, clone:H246.
	787332	3581790	core					AB063900	Homo sapiens IGH mRNA for immunoglobulin heavy chain VHDJ region, partial cds, clone:H247.
	787333	3581791	core					AB063904	Homo sapiens IGH mRNA for immunoglobulin heavy chain VHDJ region, partial cds, clone:H249.
	787334	3581792	extended					AB063905	Homo sapiens IGH mRNA for immunoglobulin heavy chain VHDJ region, partial cds, clone:H250.

	787335	3581793	extended					AB063907	Homo sapiens IGH mRNA for immunoglobulin heavy chain VHDJ region, partial cds, clone:H254.
	787336	3581794	extended					AB063909	Homo sapiens IGH mRNA for immunoglobulin heavy chain VHDJ region, partial cds, clone:H255.
	787337	3581795	extended					AB063910	Homo sapiens IGH mRNA for immunoglobulin heavy chain VHDJ region, partial cds, clone:H257.
	787338	3581796	extended					AB063911	Homo sapiens IGH mRNA for immunoglobulin heavy chain VHDJ region, partial cds, clone:H259.
	787339	3581797	full					AB063912	Homo sapiens IGH mRNA for immunoglobulin heavy chain VHDJ region, partial cds, clone:H260.
	787340	3581798	full					AB063913	Homo sapiens IGH mRNA for immunoglobulin heavy chain VHDJ region, partial cds, clone:H261.
	787341	3581799	extended					AB063915	Homo sapiens IGH mRNA for immunoglobulin heavy chain VHDJ region, partial cds, clone:H262.
	787342	3581800	extended					AB063916	Homo sapiens IGH mRNA for immunoglobulin heavy chain VHDJ region, partial cds, clone:H263.
	787343	3581801	extended					AB063919	Homo sapiens IGH mRNA for immunoglobulin heavy chain VHDJ region, partial cds, clone:H265.
	787344	3581802	extended					AB063920	Homo sapiens IGH mRNA for immunoglobulin heavy chain VHDJ region, partial cds, clone:H266.
	787345	3581803	full					AB063922	Homo sapiens IGH mRNA for immunoglobulin heavy chain VHDJ region, partial cds, clone:H269.
	787346	3581804	extended					AB063923	Homo sapiens IGH mRNA for immunoglobulin heavy chain VHDJ region, partial cds, clone:H270.
	787347	3581805	extended					AB063927	Homo sapiens IGH mRNA for immunoglobulin heavy chain VHDJ region, partial cds, clone:H272.
	787348	3581806	extended					AB066827	Homo sapiens IGH mRNA for immunoglobulin heavy chain VHDJ region, partial cds, clone:H273.
	787349	3581807	full					AB066831	Homo sapiens IGH mRNA for immunoglobulin heavy chain VHDJ region, partial cds, clone:H277.
	787350	3581808	extended					AB066838	Homo sapiens IGH mRNA for immunoglobulin heavy chain VHDJ region, partial cds, clone:TRH1-10.
	787351	3581809	extended					AB066852	Homo sapiens IGH mRNA for immunoglobulin heavy chain VHDJ region, partial cds, clone:TRH1-17.
	787352	3581810	extended					AB066853	Homo sapiens IGH mRNA for immunoglobulin heavy chain VHDJ region, partial cds, clone:TRH1-24.
	787353	3581811	extended					AB066856	Homo sapiens IGH mRNA for immunoglobulin heavy chain VHDJ region, partial cds, clone:TRH2-17.
	787354	3581812	extended					AB066858	Homo sapiens IGH mRNA for immunoglobulin heavy chain VHDJ region, partial cds, clone:TRH2-18.
	787355	3581813	extended					AB066859	Homo sapiens IGH mRNA for immunoglobulin heavy chain VHDJ region, partial cds, clone:TRH2-24.
	787356	3581814	extended					AB066862	Homo sapiens IGH mRNA for immunoglobulin heavy chain VHDJ region, partial cds, clone:TRH2-5.
	787357	3581815	extended					AB066864	Homo sapiens IGH mRNA for immunoglobulin heavy chain VHDJ region, partial cds, clone:TRH2-7.
	787358	3581816	extended					AB066865	Homo sapiens IGH mRNA for immunoglobulin heavy chain VHDJ region, partial cds, clone:TRH22-10.
	787359	3581817	extended					AB066867	Homo sapiens IGH mRNA for immunoglobulin heavy chain VHDJ region, partial cds, clone:TRH22-12.
	787360	3581818	extended					AB066868	Homo sapiens IGH mRNA for immunoglobulin heavy chain VHDJ region, partial cds, clone:TRH22-15.
	787361	3581819	full					AB066869	Homo sapiens IGH mRNA for immunoglobulin heavy chain VHDJ region, partial cds, clone:TRH22-17.
	787362	3581820	full					AB066870	Homo sapiens IGH mRNA for immunoglobulin heavy chain VHDJ region, partial cds, clone:TRH22-18.
	787363	3581821	core					AB066871	Homo sapiens IGH mRNA for immunoglobulin heavy chain VHDJ region, partial cds, clone:TRH22-2.
	787364	3581822	core					AB066872	Homo sapiens IGH mRNA for immunoglobulin heavy chain VHDJ region, partial cds, clone:TRH22-20.
	787365	3581823	core					AB066873	Homo sapiens IGH mRNA for immunoglobulin heavy chain VHDJ region, partial cds, clone:TRH22-21.
	787366	3581824	core					AB066877	Homo sapiens IGH mRNA for immunoglobulin heavy chain VHDJ region, partial cds, clone:TRH22-22.
	787367	3581825	extended					AB066878	Homo sapiens IGH mRNA for immunoglobulin heavy chain VHDJ region, partial cds, clone:TRH22-23.
	787368	3581826	extended					AB066881	Homo sapiens IGH mRNA for immunoglobulin heavy chain VHDJ region, partial cds, clone:TRH22-5.
	787369	3581827	core					AB066882	Homo sapiens IGH mRNA for immunoglobulin heavy chain VHDJ region, partial cds, clone:TRH22-8.
	787370	3581828	core					AB066884	Homo sapiens IGH mRNA for immunoglobulin heavy chain VHDJ region, partial cds, clone:TRH3-10.
	787371	3581829	core					AB066885	Homo sapiens IGH mRNA for immunoglobulin heavy chain VHDJ region, partial cds, clone:TRH3-11.
	787372	3581830	core					AB066886	Homo sapiens IGH mRNA for immunoglobulin heavy chain VHDJ region, partial cds, clone:TRH3-14.
	787373	3581831	core					AB066887	Homo sapiens IGH mRNA for immunoglobulin heavy chain VHDJ region, partial cds, clone:TRH3-15.
	787374	3581832	core					AB066889	Homo sapiens IGH mRNA for immunoglobulin heavy chain VHDJ region, partial cds, clone:TRH3-16.
	787375	3581833	extended					AB066890	Homo sapiens IGH mRNA for immunoglobulin heavy chain VHDJ region, partial cds, clone:TRH3-17.
	787376	3581834	core					AB066892	Homo sapiens IGH mRNA for immunoglobulin heavy chain VHDJ region, partial cds, clone:TRH3-2.
	787377	3581835	core					AB066894	Homo sapiens IGH mRNA for immunoglobulin heavy chain VHDJ region, partial cds, clone:TRH3-23.
	787378	3581836	core					AB066895	Homo sapiens IGH mRNA for immunoglobulin heavy chain VHDJ region, partial cds, clone:TRH3-3.
	787379	3581837	core					AB066900	Homo sapiens IGH mRNA for immunoglobulin heavy chain VHDJ region, partial cds, clone:TRH3-7.
	787380	3581838	core					AB066903	Homo sapiens IGH mRNA for immunoglobulin heavy chain VHDJ region, partial cds, clone:TRH3-8.
	787381	3581839	core					AB066905	Homo sapiens IGH mRNA for immunoglobulin heavy chain VHDJ region, partial cds, clone:TRH4-15.
	787382	3581840	extended					AB066909	Homo sapiens IGH mRNA for immunoglobulin heavy chain VHDJ region, partial cds, clone:TRH4-19.
	787383	3581841	core					AB066910	Homo sapiens IGH mRNA for immunoglobulin heavy chain VHDJ region, partial cds, clone:TRH4-20.
	787384	3581842	core					AB066912	Homo sapiens IGH mRNA for immunoglobulin heavy chain VHDJ region, partial cds, clone:TRH4-24.
	787385	3581843	core					AB066923	Homo sapiens IGH mRNA for immunoglobulin heavy chain VHDJ region, partial cds, clone:TRH4-4.
	787386	3581844	extended					AB066925	Homo sapiens IGH mRNA for immunoglobulin heavy chain VHDJ region, partial cds, clone:TRH4-7.
	787387	3581845	extended					AB066926	Homo sapiens IGH mRNA for immunoglobulin heavy chain VHDJ region, partial cds, clone:TRH42-5.
	787388	3581846	full					AB066943	Homo sapiens IGH mRNA for immunoglobulin heavy chain VHDJ region, partial cds, clone:TRH5-1.

	787389	3581847	extended					AB066946	Homo sapiens IGH mRNA for immunoglobulin heavy chain VHDJ region, partial cds, clone:TRH5-10.
	787390	3581848	full					AB066964	Homo sapiens IGH mRNA for immunoglobulin heavy chain VHDJ region, partial cds, clone:TRH6-10.
	787391	3581849	full					AB066966	Homo sapiens IGH mRNA for immunoglobulin heavy chain VHDJ region, partial cds, clone:TRH6-15.
	787392	3581850	extended					AB066970	Homo sapiens IGH mRNA for immunoglobulin heavy chain VHDJ region, partial cds, clone:TRH7-23.
	787393	3581851	extended					AB066972	Homo sapiens IGH mRNA for immunoglobulin heavy chain VHDJ region, partial cds, clone:TRH7-3.
	787394	3581852	extended					AB066973	Homo sapiens IGH mRNA for immunoglobulin heavy chain VHDJ region, partial cds, clone:aims0002h.
	787395	3581853	extended					AB066974	Homo sapiens IGH mRNA for immunoglobulin heavy chain VHDJ region, partial cds, clone:aims0006h.
	787396	3581854	extended					AB066975	Homo sapiens IGH mRNA for immunoglobulin heavy chain VHDJ region, partial cds, clone:aims0010h.
	787397	3581855	extended					AB066976	Homo sapiens IGH mRNA for immunoglobulin heavy chain VHDJ region, partial cds, clone:aims0012h.
	787398	3581856	core					AB066977	Homo sapiens IGH mRNA for immunoglobulin heavy chain VHDJ region, partial cds, clone:aims0013h.
	787399	3581857	core					AB066980	Homo sapiens IGH mRNA for immunoglobulin heavy chain VHDJ region, partial cds, clone:aims0014h.
	787400	3581858	core					AB066981	Homo sapiens IGH mRNA for immunoglobulin heavy chain VHDJ region, partial cds, clone:aims0015h.
	787401	3581859	core					AB066982	Homo sapiens IGH mRNA for immunoglobulin heavy chain VHDJ region, partial cds, clone:aims0019h.
	787402	3581860	core					AB066983	Homo sapiens IGH mRNA for immunoglobulin heavy chain VHDJ region, partial cds, clone:aims0020h.
	787403	3581861	core					AB066984	Homo sapiens IGH mRNA for immunoglobulin heavy chain VHDJ region, partial cds, clone:aims0023h.
	787404	3581862	core					AB066999	Homo sapiens IGH mRNA for immunoglobulin heavy chain VHDJ region, partial cds, clone:aims0024h.
	787405	3581863	extended					AB067002	Homo sapiens IGH mRNA for immunoglobulin heavy chain VHDJ region, partial cds, clone:aims0027h.
	787406	3581864	core					AB067004	Homo sapiens IGH mRNA for immunoglobulin heavy chain VHDJ region, partial cds, clone:aims0049h.
	787407	3581865	extended					AB067006	Homo sapiens IGH mRNA for immunoglobulin heavy chain VHDJ region, partial cds, clone:aims0053h.
	787408	3581866	core					AB067010	Homo sapiens IGH mRNA for immunoglobulin heavy chain VHDJ region, partial cds, clone:aims0055h.
	787409	3581867	extended					AB067014	Homo sapiens IGH mRNA for immunoglobulin heavy chain VHDJ region, partial cds, clone:aims0057h.
	787410	3581868	core					AB067015	Homo sapiens IGH mRNA for immunoglobulin heavy chain VHDJ region, partial cds, clone:aims0062h.
	787411	3581869	core					AB067016	Homo sapiens IGH mRNA for immunoglobulin heavy chain VHDJ region, partial cds, clone:aims0071h.
	787412	3581870	core					AB067025	Homo sapiens IGH mRNA for immunoglobulin heavy chain VHDJ region, partial cds, clone:aims0073h.
	787413	3581871	core					AB067026	Homo sapiens IGH mRNA for immunoglobulin heavy chain VHDJ region, partial cds, clone:aims0074h.
	787414	3581872	core					AB067027	Homo sapiens IGH mRNA for immunoglobulin heavy chain VHDJ region, partial cds, clone:aims0088h.
	787415	3581873	extended					AB067028	Homo sapiens IGH mRNA for immunoglobulin heavy chain VHDJ region, partial cds, clone:aims0089h.
	787416	3581874	extended					AB067030	Homo sapiens IGH mRNA for immunoglobulin heavy chain VHDJ region, partial cds, clone:aims0090h.
	787417	3581875	extended					AB067033	Homo sapiens IGH mRNA for immunoglobulin heavy chain VHDJ region, partial cds, clone:aims0091h.
	787418	3581876	extended					AB067034	Homo sapiens IGH mRNA for immunoglobulin heavy chain VHDJ region, partial cds, clone:aims0093h.
	787419	3581877	full					AB067040	Homo sapiens IGH mRNA for immunoglobulin heavy chain VHDJ region, partial cds, clone:aims0096h.
	787420	3581878	extended					AB067041	Homo sapiens IGH mRNA for immunoglobulin heavy chain VHDJ region, partial cds, clone:aims0097h.
	787421	3581879	extended					AB067055	Homo sapiens IGH mRNA for immunoglobulin heavy chain VHDJ region, partial cds, clone:aims0107h.
	787422	3581880	extended					AB067056	Homo sapiens IGH mRNA for immunoglobulin heavy chain VHDJ region, partial cds, clone:aims0108h.
	787423	3581881	extended					AB067057	Homo sapiens IGH mRNA for immunoglobulin heavy chain VHDJ region, partial cds, clone:aims0126h.
	787424	3581882	extended					AB067060	Homo sapiens IGH mRNA for immunoglobulin heavy chain VHDJ region, partial cds, clone:aims0127h.
	787425	3581883	extended					AB067069	Homo sapiens IGH mRNA for immunoglobulin heavy chain VHDJ region, partial cds, clone:aims0130h.
	787426	3581884	extended					AB067071	Homo sapiens IGH mRNA for immunoglobulin heavy chain VHDJ region, partial cds, clone:aims0134h.
	787427	3581885	full					AB067072	Homo sapiens IGH mRNA for immunoglobulin heavy chain VHDJ region, partial cds, clone:aims0145h.
	787428	3581886	extended					AB067076	Homo sapiens IGH mRNA for immunoglobulin heavy chain VHDJ region, partial cds, clone:aims0149h.
	787429	3581887	extended					AB067077	Homo sapiens IGH mRNA for immunoglobulin heavy chain VHDJ region, partial cds, clone:aims0150h.
	787430	3581888	extended					AB067081	Homo sapiens IGH mRNA for immunoglobulin heavy chain VHDJ region, partial cds, clone:aims0156h.
	787431	3581889	full					AB067083	Homo sapiens IGH mRNA for immunoglobulin heavy chain VHDJ region, partial cds, clone:aims0157h.
	787432	3581890	full					AB067084	Homo sapiens IGH mRNA for immunoglobulin heavy chain VHDJ region, partial cds, clone:aims0166h.
	787433	3581891	full					AB067089	Homo sapiens IGH mRNA for immunoglobulin heavy chain VHDJ region, partial cds, clone:aims0168h.
	787434	3581892	extended					AB067097	Homo sapiens IGH mRNA for immunoglobulin heavy chain VHDJ region, partial cds, clone:aims0171h.
	787435	3581893	extended					AB067098	Homo sapiens IGH mRNA for immunoglobulin heavy chain VHDJ region, partial cds, clone:aims0176h.
	787436	3581894	core					AB067102	Homo sapiens IGH mRNA for immunoglobulin heavy chain VHDJ region, partial cds, clone:aims0184h.
	787437	3581895	core					AB067103	Homo sapiens IGH mRNA for immunoglobulin heavy chain VHDJ region, partial cds, clone:aims0185h.
	787438	3581896	full					AB067108	Homo sapiens IGH mRNA for immunoglobulin heavy chain VHDJ region, partial cds, clone:aims0191h.
	787439	3581897	full					AB067111	Homo sapiens IGH mRNA for immunoglobulin heavy chain VHDJ region, partial cds, clone:aims0192h.
	787440	3581898	core					AB067115	Homo sapiens IGH mRNA for immunoglobulin heavy chain VHDJ region, partial cds, clone:aims0198h.
	787441	3581899	core					AB067120	Homo sapiens IGH mRNA for immunoglobulin heavy chain VHDJ region, partial cds, clone:aims0201h.
	787442	3581900	extended					AB067123	Homo sapiens IGH mRNA for immunoglobulin heavy chain VHDJ region, partial cds, clone:aims0205h.

	787443	3581901	extended					AB067124	Homo sapiens IGH mRNA for immunoglobulin heavy chain VHDJ region, partial cds, clone:aims0211h.
	787444	3581902	extended					AB067126	Homo sapiens IGH mRNA for immunoglobulin heavy chain VHDJ region, partial cds, clone:aims0214h.
	787445	3581903	core					AB067128	Homo sapiens IGH mRNA for immunoglobulin heavy chain VHDJ region, partial cds, clone:aims0215h.
	787446	3581904	core					AB067130	Homo sapiens IGH mRNA for immunoglobulin heavy chain VHDJ region, partial cds, clone:aims0217h.
	787447	3581905	core					AB067131	Homo sapiens IGH mRNA for immunoglobulin heavy chain VHDJ region, partial cds, clone:aims0221h.
	787448	3581906	extended					AB067133	Homo sapiens IGH mRNA for immunoglobulin heavy chain VHDJ region, partial cds, clone:aims0225h.
	787449	3581907	core					AB067134	Homo sapiens IGH mRNA for immunoglobulin heavy chain VHDJ region, partial cds, clone:aims0228h.
	787450	3581908	extended					AB067136	Homo sapiens IGH mRNA for immunoglobulin heavy chain VHDJ region, partial cds, clone:aims0230h.
	787451	3581909	extended					AB067146	Homo sapiens IGH mRNA for immunoglobulin heavy chain VHDJ region, partial cds, clone:aims0231h.
	787452	3581910	extended					AB067152	Homo sapiens IGH mRNA for immunoglobulin heavy chain VHDJ region, partial cds, clone:aims0234h.
	787453	3581911	core					AB067155	Homo sapiens IGH mRNA for immunoglobulin heavy chain VHDJ region, partial cds, clone:aims0246h.
	787454	3581912	full					AB067156	Homo sapiens IGH mRNA for immunoglobulin heavy chain VHDJ region, partial cds, clone:ya0031h.
	787455	3581913	full					AB067158	Homo sapiens IGH mRNA for immunoglobulin heavy chain VHDJ region, partial cds, clone:ya0034h.
	787456	3581914	core					AB067160	Homo sapiens IGH mRNA for immunoglobulin heavy chain VHDJ region, partial cds, clone:ya0035h.
	787457	3581915	extended					AB067161	Homo sapiens IGH mRNA for immunoglobulin heavy chain VHDJ region, partial cds, clone:ya0037h.
	787458	3581916	core					AB067170	Homo sapiens IGH mRNA for immunoglobulin heavy chain VHDJ region, partial cds, clone:ya0039h.
	787459	3581917	core					AB067171	Homo sapiens IGH mRNA for immunoglobulin heavy chain VHDJ region, partial cds, clone:ya0040h.
	787460	3581918	full					AB067172	Homo sapiens IGH mRNA for immunoglobulin heavy chain VHDJ region, partial cds, clone:ya0049h.
	787461	3581919	core					AB067173	Homo sapiens IGH mRNA for immunoglobulin heavy chain VHDJ region, partial cds, clone:ya0050h.
	787462	3581920	extended					AB067174	Homo sapiens IGH mRNA for immunoglobulin heavy chain VHDJ region, partial cds, clone:ya0051h.
	787463	3581921	core					AB067175	Homo sapiens IGH mRNA for immunoglobulin heavy chain VHDJ region, partial cds, clone:ya0052h.
	787464	3581922	core					AB067177	Homo sapiens IGH mRNA for immunoglobulin heavy chain VHDJ region, partial cds, clone:ya0053h.
	787465	3581923	full					AB067179	Homo sapiens IGH mRNA for immunoglobulin heavy chain VHDJ region, partial cds, clone:ya0054h.
	787466	3581924	core					AB067182	Homo sapiens IGH mRNA for immunoglobulin heavy chain VHDJ region, partial cds, clone:ya0056h.
	787467	3581925	core					AB067184	Homo sapiens IGH mRNA for immunoglobulin heavy chain VHDJ region, partial cds, clone:ya0058h.
	787468	3581926	core					AB067185	Homo sapiens IGH mRNA for immunoglobulin heavy chain VHDJ region, partial cds, clone:ya0061h.
	787469	3581927	core					AB067186	Homo sapiens IGH mRNA for immunoglobulin heavy chain VHDJ region, partial cds, clone:ya0063h.
	787470	3581928	core					AB067189	Homo sapiens IGH mRNA for immunoglobulin heavy chain VHDJ region, partial cds, clone:ya0064h.
	787471	3581929	core					AB067190	Homo sapiens IGH mRNA for immunoglobulin heavy chain VHDJ region, partial cds, clone:ya0065h.
	787472	3581930	core					AB067192	Homo sapiens IGH mRNA for immunoglobulin heavy chain VHDJ region, partial cds, clone:ya0068h.
	787473	3581931	core					AB067193	Homo sapiens IGH mRNA for immunoglobulin heavy chain VHDJ region, partial cds, clone:ya0069h.
	787474	3581932	core					AB067214	Homo sapiens IGH mRNA for immunoglobulin heavy chain VHDJ region, partial cds, clone:ya0071h.
	787475	3581933	core					AB067221	Homo sapiens IGH mRNA for immunoglobulin heavy chain VHDJ region, partial cds, clone:ya0072h.
	787476	3581934	extended					AB067222	Homo sapiens IGH mRNA for immunoglobulin heavy chain VHDJ region, partial cds, clone:ya0093h.
	787477	3581935	extended					AB067225	Homo sapiens IGH mRNA for immunoglobulin heavy chain VHDJ region, partial cds, clone:ya0100h.
	787478	3581936	extended					AB067226	Homo sapiens IGH mRNA for immunoglobulin heavy chain VHDJ region, partial cds, clone:ya0101h.
	787479	3581937	extended					AB067228	Homo sapiens IGH mRNA for immunoglobulin heavy chain VHDJ region, partial cds, clone:ya0104h.
	787480	3581938	extended					AB067229	Homo sapiens IGH mRNA for immunoglobulin heavy chain VHDJ region, partial cds, clone:ya0105h.
	787481	3581939	extended					AB067230	Homo sapiens IGH mRNA for immunoglobulin heavy chain VHDJ region, partial cds, clone:ya0107h.
	787482	3581940	extended					AB067231	Homo sapiens IGH mRNA for immunoglobulin heavy chain VHDJ region, partial cds, clone:ya0108h.
	787483	3581941	extended					AB067232	Homo sapiens IGH mRNA for immunoglobulin heavy chain VHDJ region, partial cds, clone:ya0109h.
	787484	3581942	extended					AB067233	Homo sapiens IGH mRNA for immunoglobulin heavy chain VHDJ region, partial cds, clone:ya0110h.
	787485	3581943	extended					AB067234	Homo sapiens IGH mRNA for immunoglobulin heavy chain VHDJ region, partial cds, clone:ya0111h.
	787486	3581944	extended					AB067235	Homo sapiens IGH mRNA for immunoglobulin heavy chain VHDJ region, partial cds, clone:ya0112h.
	787487	3581945	core					AB067237	Homo sapiens IGH mRNA for immunoglobulin heavy chain VHDJ region, partial cds, clone:ya0113h.
	787488	3581946	extended					AB067238	Homo sapiens IGH mRNA for immunoglobulin heavy chain VHDJ region, partial cds, clone:ya0114h.
	787489	3581947	extended					AB067240	Homo sapiens IGH mRNA for immunoglobulin heavy chain VHDJ region, partial cds, clone:ya0116h.
	787490	3581948	extended					AB067247	Homo sapiens IGH mRNA for immunoglobulin heavy chain VHDJ region, partial cds, clone:ya0117h.
	787491	3581949	extended					AB067250	Homo sapiens IGH mRNA for immunoglobulin heavy chain VHDJ region, partial cds, clone:ya0119h.
	787492	3581950	extended					AB067252	Homo sapiens IGH mRNA for immunoglobulin heavy chain VHDJ region, partial cds, clone:ya0126h.
	787493	3581951	full					AB067254	Homo sapiens IGH mRNA for immunoglobulin heavy chain VHDJ region, partial cds, clone:ya0129h.
	787494	3581952	extended					AB067255	Homo sapiens IGH mRNA for immunoglobulin heavy chain VHDJ region, partial cds, clone:ya0131h.
	787495	3581953	extended					AB067256	Homo sapiens IGH mRNA for immunoglobulin heavy chain VHDJ region, partial cds, clone:ya0133h.
	787496	3581954	extended					AB067257	Homo sapiens IGH mRNA for immunoglobulin heavy chain VHDJ region, partial cds, clone:ya0134h.

	787497	3581955	extended					AB067258	Homo sapiens IGH mRNA for immunoglobulin heavy chain VHDJ region, partial cds, clone:ya0135h.
	787498	3581956	extended					AB067260	Homo sapiens IGH mRNA for immunoglobulin heavy chain VHDJ region, partial cds, clone:ya0136h.
	787499	3581957	extended					AB067261	Homo sapiens IGH mRNA for immunoglobulin heavy chain VHDJ region, partial cds, clone:ya0137h.
	787500	3581958	extended					AB067262	Homo sapiens IGH mRNA for immunoglobulin heavy chain VHDJ region, partial cds, clone:ya0139h.
	787501	3581959	full					AB067264	Homo sapiens IGH mRNA for immunoglobulin heavy chain VHDJ region, partial cds, clone:ya0140h.
	787502	3581960	extended					AB067265	Homo sapiens IGH mRNA for immunoglobulin heavy chain VHDJ region, partial cds, clone:ya0141h.
	787503	3581961	full					AB067266	Homo sapiens IGH mRNA for immunoglobulin heavy chain VHDJ region, partial cds, clone:ya0143h.
	787504	3581962	full					AB067269	Homo sapiens IGH mRNA for immunoglobulin heavy chain VHDJ region, partial cds, clone:ya0144h.
	787505	3581963	full					AB067270	Homo sapiens IGH mRNA for immunoglobulin heavy chain VHDJ region, partial cds, clone:ya0145h.
	787506	3581964	full					AB067271	Homo sapiens IGH mRNA for immunoglobulin heavy chain VHDJ region, partial cds, clone:ya0148h.
	787507	3581965	full					AB067272	Homo sapiens IGH mRNA for immunoglobulin heavy chain VHDJ region, partial cds, clone:ya0149h.
	787508	3581966	extended					AB067275	Homo sapiens IGH mRNA for immunoglobulin heavy chain VHDJ region, partial cds, clone:ya0150h.
	787509	3581967	full					AB067280	Homo sapiens IGH mRNA for immunoglobulin heavy chain VHDJ region, partial cds, clone:ya0151h.
	787510	3581968	full					AB067283	Homo sapiens IGH mRNA for immunoglobulin heavy chain VHDJ region, partial cds, clone:ya0154h.
	787511	3581969	full					AB067284	Homo sapiens IGH mRNA for immunoglobulin heavy chain VHDJ region, partial cds, clone:ya0159h.
	787512	3581970	full					AB067286	Homo sapiens IGH mRNA for immunoglobulin heavy chain VHDJ region, partial cds, clone:ya0162h.
	787513	3581971	full					AB067287	Homo sapiens IGH mRNA for immunoglobulin heavy chain VHDJ region, partial cds, clone:ya0163h.
	787514	3581972	extended					AB067291	Homo sapiens IGH mRNA for immunoglobulin heavy chain VHDJ region, partial cds, clone:ya0165h.
	787515	3581973	full					AB067294	Homo sapiens IGH mRNA for immunoglobulin heavy chain VHDJ region, partial cds, clone:ya0166h.
	787516	3581974	full					AB067295	Homo sapiens IGH mRNA for immunoglobulin heavy chain VHDJ region, partial cds, clone:ya0170h.
	787517	3581975	full					AB067299	Homo sapiens IGH mRNA for immunoglobulin heavy chain VHDJ region, partial cds, clone:ya0173h.
	787518	3581976	full					AB067300	Homo sapiens IGH mRNA for immunoglobulin heavy chain VHDJ region, partial cds, clone:ya0174h.
	787519	3581977	full					AB067301	Homo sapiens IGH mRNA for immunoglobulin heavy chain VHDJ region, partial cds, clone:ya0178h.
	787520	3581978	full					AB067302	Homo sapiens IGH mRNA for immunoglobulin heavy chain VHDJ region, partial cds, clone:ya0179h.
	787521	3581979	full					AB067303	Homo sapiens IGH mRNA for immunoglobulin heavy chain VHDJ region, partial cds, clone:ya0180h.
	787522	3581980	extended					AB067304	Homo sapiens IGH mRNA for immunoglobulin heavy chain VHDJ region, partial cds, clone:ya0181h.
	787523	3581981	extended					AB067306	Homo sapiens IGH mRNA for immunoglobulin heavy chain VHDJ region, partial cds, clone:ya0182h.
	787524	3581982	extended					AB067308	Homo sapiens IGH mRNA for immunoglobulin heavy chain VHDJ region, partial cds, clone:ya0183h.
	787525	3581983	full					AB067311	Homo sapiens IGH mRNA for immunoglobulin heavy chain VHDJ region, partial cds, clone:ya0185h.
	787526	3581984	extended					AB067312	Homo sapiens IGH mRNA for immunoglobulin heavy chain VHDJ region, partial cds, clone:ya0187h.
	787527	3581985	full					AB067318	Homo sapiens IGH mRNA for immunoglobulin heavy chain VHDJ region, partial cds, clone:ya0190h.
	787528	3581986	full					AB067324	Homo sapiens IGH mRNA for immunoglobulin heavy chain VHDJ region, partial cds, clone:ya0191h.
	787529	3581987	full					AB067329	Homo sapiens IGH mRNA for immunoglobulin heavy chain VHDJ region, partial cds, clone:ya0197h.
	787530	3581988	extended					AB067336	Homo sapiens IGH mRNA for immunoglobulin heavy chain VHDJ region, partial cds, clone:ya0203h.
	787531	3581989	extended					AB067340	Homo sapiens IGH mRNA for immunoglobulin heavy chain VHDJ region, partial cds, clone:ya0208h.
	787532	3581990	extended					AB067341	Homo sapiens IGH mRNA for immunoglobulin heavy chain VHDJ region, partial cds, clone:ya0215h.
	787533	3581991	extended					AB067342	Homo sapiens IGH mRNA for immunoglobulin heavy chain VHDJ region, partial cds, clone:ya0219h.
	787534	3581992	extended					AB067343	Homo sapiens IGH mRNA for immunoglobulin heavy chain VHDJ region, partial cds, clone:ya0220h.
	787535	3581993	extended					AB067344	Homo sapiens IGH mRNA for immunoglobulin heavy chain VHDJ region, partial cds, clone:ya0221h.
	787536	3581994	extended					AB067347	Homo sapiens IGH mRNA for immunoglobulin heavy chain VHDJ region, partial cds, clone:ya0222h.
	787537	3581995	full					AB067349	Homo sapiens IGH mRNA for immunoglobulin heavy chain VHDJ region, partial cds, clone:ya0223h.
	787538	3581996	full					AB067351	Homo sapiens IGH mRNA for immunoglobulin heavy chain VHDJ region, partial cds, clone:ya0226h.
	787539	3581997	full					AB067353	Homo sapiens IGH mRNA for immunoglobulin heavy chain VHDJ region, partial cds, clone:ya0228h.
	787540	3581998	full					AB067357	Homo sapiens IGH mRNA for immunoglobulin heavy chain VHDJ region, partial cds, clone:ya0230h.
	787541	3581999	extended					AB067360	Homo sapiens IGH mRNA for immunoglobulin heavy chain VHDJ region, partial cds, clone:ya0232h.
	787542	3582000	extended					AB067361	Homo sapiens IGH mRNA for immunoglobulin heavy chain VHDJ region, partial cds, clone:ya0236h.
	787543	3582001	extended					AB067363	Homo sapiens IGH mRNA for immunoglobulin heavy chain VHDJ region, partial cds, clone:ya0239h.
	787544	3582002	full					AB067364	Homo sapiens IGH mRNA for immunoglobulin heavy chain VHDJ region, partial cds, clone:ya0240h.
	787545	3582003	core					AB067365	Homo sapiens IGH mRNA for immunoglobulin heavy chain VHDJ region, partial cds, clone:ya0242h.
	787546	3582004	full					AB067366	Homo sapiens IGH mRNA for immunoglobulin heavy chain VHDJ region, partial cds, clone:ya0243h.
	787547	3582005	core					AB067368	Homo sapiens IGH mRNA for immunoglobulin heavy chain VHDJ region, partial cds, clone:ya0244h.
	787548	3582006	extended					AB067369	Homo sapiens IGH mRNA for immunoglobulin heavy chain VHDJ region, partial cds, clone:ya0245h.
	787549	3582007	extended					AB067370	Homo sapiens IGH mRNA for immunoglobulin heavy chain VHDJ region, partial cds, clone:ya0247h.
	787550	3582008	full					AB067373	Homo sapiens IGH mRNA for immunoglobulin heavy chain VHDJ region, partial cds, clone:ya0248h.

	787551	3582009	full						AB067374	Homo sapiens IGH mRNA for immunoglobulin heavy chain VHDJ region, partial cds, clone:ya0249h.
	787552	3582010	full						AB067376	Homo sapiens IGH mRNA for immunoglobulin heavy chain VHDJ region, partial cds, clone:ya0252h.
	787553	3582011	full						AB067378	Homo sapiens IGH mRNA for immunoglobulin heavy chain VHDJ region, partial cds, clone:ya0253h.
	787554	3582012	full						AB067379	Homo sapiens IGH mRNA for immunoglobulin heavy chain VHDJ region, partial cds, clone:ya0255h.
	787555	3582013	extended						AB067380	Homo sapiens IGH mRNA for immunoglobulin heavy chain VHDJ region, partial cds, clone:ya0257h.
	787556	3582014	extended						AB067385	Homo sapiens IGH mRNA for immunoglobulin heavy chain VHDJ region, partial cds, clone:ya0258h.
	787557	3582015	extended						AB067390	Homo sapiens IGH mRNA for immunoglobulin heavy chain VHDJ region, partial cds, clone:ya0259h.
	787558	3582016	extended						AB067393	Homo sapiens IGH mRNA for immunoglobulin heavy chain VHDJ region, partial cds, clone:ya0264h.
	787559	3582017	extended						AB067394	Homo sapiens IGH mRNA for immunoglobulin heavy chain VHDJ region, partial cds, clone:ya0269h.
	787560	3582018	extended						AB067395	Homo sapiens IGH mRNA for immunoglobulin heavy chain VHDJ region, partial cds, clone:ya0272h.
	787561	3582019	extended						AB084083	Homo sapiens IGH mRNA for immunoglobulin heavy chain VHDJ region, partial cds, clone:ya0273h.
	787562	3582020	extended						AB084084	Homo sapiens IGH mRNA for immunoglobulin heavy chain VHDJ region, partial cds, clone:ya0274h.
	787563	3582021	extended						AB087877	Homo sapiens mRNA for IgM myu chain, partial cds, clone:HO702-001H.
	787564	3582022	extended						AB095276	Homo sapiens mRNA for IgM myu chain, partial cds, clone:HO702-016H.
	787565	3582023	extended						AB110646	Homo sapiens IgVH mRNA for Ig H-chain V-region, partial cds.
	787566	3582024	extended						AB159728	Homo sapiens VH mRNA for immunoglobulin gamma heavy chain, partial cds, V region, clone:H-CP5.
	787567	3582025	full						AB245095	Homo sapiens IgH mRNA for Immunoglobulin H chain V region, partial cds.
	787568	3582026	full						AB266513	Homo sapiens IgH mRNA for anti-HBs antibody heavy chain, partial cds.
	787569	3582027	core						AF001424	Homo sapiens mRNA for immunoglobulin heavy chain, partial cds, anti-SARS coronavirus spike protein.
	787570	3582028	core						AF001791	Homo sapiens IgG mRNA for IgG variable heavy chain, complete cds, note: Fab-aVCA21 heavy chain.
	787571	3582029	core						AF004319	Homo sapiens anti-CD34 immunoglobulin heavy chain V region mRNA, partial cds.
	787572	3582030	core						AF004322	Homo sapiens anti-CD34 Ig heavy chain variable region mRNA, partial cds.
	787573	3582031	core						AF004323	Homo sapiens anti-melanoma Ig heavy chain variable region mRNA, partial cds.
	787574	3582032	core						AF004324	Homo sapiens anti-melanoma Ig heavy chain variable region mRNA, partial cds.
	787575	3582033	core						AF004937	Homo sapiens anti-melanoma Ig heavy chain variable region mRNA, partial cds.
	787576	3582034	core						AF004938	Homo sapiens anti-melanoma Ig heavy chain variable region mRNA, partial cds.
	787577	3582035	core						AF004939	Homo sapiens rearranged immunoglobulin heavy chain variable region (ML9-VH4) mRNA, partial cds.
	787578	3582036	extended						AF004940	Homo sapiens rearranged immunoglobulin heavy chain variable region (ML11-VH3) mRNA, partial cds.
	787579	3582037	full						AF006523	Homo sapiens rearranged immunoglobulin heavy chain variable region (ML12-VH4) mRNA, partial cds.
	787580	3582038	full						AF006526	Homo sapiens rearranged immunoglobulin heavy chain variable region (ML13-VH4) mRNA, partial cds.
	787581	3582039	full						AF006527	Homo sapiens immunoglobulin heavy chain variable region mRNA, partial cds.
	787582	3582040	full						AF013615	Homo sapiens immunoglobulin heavy chain variable region mRNA, partial cds.
	787583	3582041	extended						AF013622	Homo sapiens immunoglobulin heavy chain variable region mRNA, partial cds.
	787584	3582042	extended						AF015128	Homo sapiens immunoglobulin heavy chain variable region (VH6) mRNA, partial cds.
	787585	3582043	core						AF015129	Homo sapiens immunoglobulin heavy chain variable region (VH4) mRNA, VH4-39 allele, partial cds.
	787586	3582044	core						AF015130	Homo sapiens IgG heavy chain variable region (Vh26) mRNA, partial cds.
	787587	3582045	core						AF015135	Homo sapiens IgG heavy chain variable region (Vh26) mRNA, partial cds.
	787588	3582046	core						AF015137	Homo sapiens IgG heavy chain variable region (Vh26) mRNA, partial cds.
	787589	3582047	extended						AF015138	Homo sapiens IgG heavy chain variable region (Vh26) mRNA, partial cds.
	787590	3582048	core						AF015139	Homo sapiens IgG heavy chain variable region (Vh6) mRNA, partial cds.
	787591	3582049	extended						AF015140	Homo sapiens IgG heavy chain variable region (Vh6) mRNA, partial cds.
	787592	3582050	full						AF015141	Homo sapiens IgG heavy chain variable region (Vh6) mRNA, partial cds.
	787593	3582051	full						AF015146	Homo sapiens IgG heavy chain variable region (Vh6) mRNA, partial cds.
	787594	3582052	core						AF017459	Homo sapiens IgG heavy chain variable region (Vh6) mRNA, partial cds.
	787595	3582053	full						AF019642	Homo sapiens IgG heavy chain variable region (Vh6) mRNA, partial cds.
	787596	3582054	core						AF021940	Homo sapiens isolate case 9 rearranged immunoglobulin heavy chain variable region (IgH) mRNA, partial cds.
	787597	3582055	extended						AF021941	Homo sapiens CD20-binding IgG1, Ig heavy chain VH6 region (IgH) mRNA, partial cds.
	787598	3582056	full						AF021942	Homo sapiens ID:CLL097 IgA heavy chain variable region mRNA, partial cds.
	787599	3582057	full						AF021949	Homo sapiens ID:CLL158 IgA heavy chain variable region mRNA, partial cds.
	787600	3582058	core						AF021952	Homo sapiens ID:CLL078 IgG heavy chain variable region mRNA, partial cds.
	787601	3582059	core						AF021953	Homo sapiens ID:CLL003 IgM heavy chain variable region mRNA, partial cds.
	787602	3582060	core						AF021958	Homo sapiens ID:CLL017 IgM heavy chain variable region mRNA, partial cds.
	787603	3582061	core						AF021961	Homo sapiens ID:CLL018 IgM heavy chain variable region mRNA, partial cds.
	787604	3582062	full						AF021963	Homo sapiens ID:CLL025 IgM heavy chain variable region mRNA, partial cds.

	787605	3582063	extended					AF021965	Homo sapiens ID:CLL035 IgM heavy chain variable region mRNA, partial cds.
	787606	3582064	full					AF021967	Homo sapiens ID:CLL041 IgM heavy chain variable region mRNA, partial cds.
	787607	3582065	extended					AF021968	Homo sapiens ID:CLL040 IgG heavy chain variable region mRNA, partial cds.
	787608	3582066	full					AF021969	Homo sapiens ID:CLL042 IgM heavy chain variable region mRNA, partial cds.
	787609	3582067	extended					AF021972	Homo sapiens ID:CLL047 IgM heavy chain variable region mRNA, partial cds.
	787610	3582068	extended					AF021973	Homo sapiens ID:CLL048 IgM heavy chain variable region mRNA, partial cds.
	787611	3582069	extended					AF021975	Homo sapiens ID:CLL058 IgM heavy chain variable region mRNA, partial cds.
	787612	3582070	extended					AF021978	Homo sapiens ID:CLL059 IgM heavy chain variable region mRNA, partial cds.
	787613	3582071	full					AF021979	Homo sapiens ID:CLL064 IgM heavy chain variable region mRNA, partial cds.
	787614	3582072	core					AF021980	Homo sapiens ID:CLL079 IgM heavy chain variable region mRNA, partial cds.
	787615	3582073	core					AF021986	Homo sapiens ID:CLL081 IgM heavy chain variable region mRNA, partial cds.
	787616	3582074	core					AF021988	Homo sapiens ID:CLL083 IgM heavy chain variable region mRNA, partial cds.
	787617	3582075	full					AF021993	Homo sapiens ID:CLL105 Ig heavy chain variable region mRNA, partial cds.
	787618	3582076	full					AF021995	Homo sapiens ID:CLL110 Ig heavy chain variable region mRNA, partial cds.
	787619	3582077	core					AF021996	Homo sapiens ID:CLL119 Ig heavy chain variable region mRNA, partial cds.
	787620	3582078	extended					AF021997	Homo sapiens ID:CLL122 Ig heavy chain variable region mRNA, partial cds.
	787621	3582079	core					AF021998	Homo sapiens ID:CLL123 Ig heavy chain variable region mRNA, partial cds.
	787622	3582080	core					AF021999	Homo sapiens ID:CLL125 Ig heavy chain variable region mRNA, partial cds.
	787623	3582081	core					AF022000	Homo sapiens ID:CLL126 Ig heavy chain variable region mRNA, partial cds.
	787624	3582082	full					AF022001	Homo sapiens ID:CLL127 Ig heavy chain variable region mRNA, partial cds.
	787625	3582083	core					AF022006	Homo sapiens ID:CLL130 Ig heavy chain variable region mRNA, partial cds.
	787626	3582084	core					AF022007	Homo sapiens ID:CLL135 Ig heavy chain variable region mRNA, partial cds.
	787627	3582085	core					AF022008	Homo sapiens ID:CLL147 Ig heavy chain variable region mRNA, partial cds.
	787628	3582086	core					AF022009	Homo sapiens ID:CLL152 Ig heavy chain variable region mRNA, partial cds.
	787629	3582087	core					AF022011	Homo sapiens ID:CLL153 Ig heavy chain variable region mRNA, partial cds.
	787630	3582088	core					AF026930	Homo sapiens ID:CLL154 Ig heavy chain variable region mRNA, partial cds.
	787631	3582089	full					AF028103	Homo sapiens ID:CLL201 Ig heavy chain variable region mRNA, partial cds.
	787632	3582090	full					AF028108	Homo sapiens alpha heavy chain variable region mRNA from amyloid patient, partial cds.
	787633	3582091	core					AF029052	Homo sapiens immunoglobulin heavy chain (IGH) mRNA, CDR3 region, partial cds.
	787634	3582092	core					AF029328	Homo sapiens immunoglobulin heavy chain (IGH) mRNA, CDR3 region, partial cds.
	787635	3582093	extended					AF030493	Homo sapiens rearranged immunoglobulin heavy chain variable region mRNA, partial cds.
	787636	3582094	extended					AF032350	Homo sapiens puL77 immunoglobulin heavy chain V region mRNA, partial cds.
	787637	3582095	extended					AF032352	Homo sapiens rearranged immunoglobulin heavy chain variable region mRNA, partial cds.
	787638	3582096	full					AF032354	Homo sapiens variable immunoglobulin anti-HLA heavy chain (IgH) mRNA, partial cds.
	787639	3582097	core					AF032360	Homo sapiens variable immunoglobulin anti-HLA heavy chain (IgH) mRNA, partial cds.
	787640	3582098	core					AF035018	Homo sapiens variable immunoglobulin anti-HLA heavy chain (IgH) mRNA, partial cds.
	787641	3582099	core					AF035019	Homo sapiens variable immunoglobulin anti-HLA heavy chain (IgH) mRNA, partial cds.
	787642	3582100	core					AF035020	Homo sapiens clone B1F8B5H myosin-reactive immunoglobulin heavy chain variable region mRNA, partial cds.
	787643	3582101	full					AF035021	Homo sapiens clone B2C2G4H myosin-reactive immunoglobulin heavy chain variable region mRNA, partial cds.
	787644	3582102	extended					AF035023	Homo sapiens clone B2E9D5H myosin-reactive immunoglobulin heavy chain variable region mRNA, partial cds.
	787645	3582103	extended					AF035025	Homo sapiens clone B4E9F5H myosin-reactive immunoglobulin heavy chain variable region mRNA, partial cds.
	787646	3582104	full					AF035027	Homo sapiens clone MCA1H myosin-reactive immunoglobulin heavy chain variable region mRNA, partial cds.
	787647	3582105	extended					AF035030	Homo sapiens clone S1G7G8H myosin-reactive immunoglobulin heavy chain variable region mRNA, partial cds.
	787648	3582106	extended					AF035039	Homo sapiens clone S12G10H myosin-reactive immunoglobulin heavy chain variable region mRNA, partial cds.
	787649	3582107	extended					AF035040	Homo sapiens clone MC57H myosin-reactive immunoglobulin heavy chain variable region mRNA, partial cds.
	787650	3582108	extended					AF035041	Homo sapiens clone B5G5G4H myosin-reactive immunoglobulin heavy chain variable region mRNA, partial cds.
	787651	3582109	extended					AF035042	Homo sapiens clone S3F5H myosin-reactive immunoglobulin heavy chain variable region mRNA, partial cds.
	787652	3582110	extended					AF035043	Homo sapiens clone S2E4H myosin-reactive immunoglobulin heavy chain variable region mRNA, partial cds.
	787653	3582111	extended					AF035789	Homo sapiens clone F22H myosin-reactive immunoglobulin heavy chain variable region mRNA, partial cds.
	787654	3582112	extended					AF035791	Homo sapiens clone FD2H myosin-reactive immunoglobulin heavy chain variable region mRNA, partial cds.
	787655	3582113	core					AF035792	Homo sapiens rheumatoid factor RF-IP10 mRNA, partial cds.
	787656	3582114	core					AF035796	Homo sapiens rheumatoid factor RF-IP13 mRNA, partial cds.
	787657	3582115	core					AF035797	Homo sapiens rheumatoid factor RF-IP14 mRNA, partial cds.
	787658	3582116	core					AF035799	Homo sapiens rheumatoid factor RF-IP24 mRNA, partial cds.

	787659	3582117	core					AF035800	Homo sapiens rheumatoid factor RF-ET6 mRNA, partial cds.
	787660	3582118	core					AF035801	Homo sapiens rheumatoid factor RF-ET10 mRNA, partial cds.
	787661	3582119	extended					AF039297	Homo sapiens rheumatoid factor RF-ET11 mRNA, partial cds.
	787662	3582120	extended					AF052520	Homo sapiens rheumatoid factor RF-ET12 mRNA, partial cds.
	787663	3582121	extended					AF052526	Homo sapiens anti-dsDNA IgM heavy chain variable region mRNA, partial cds.
	787664	3582122	extended					AF052527	Homo sapiens monoclonal antibody MM1.4 immunoglobulin heavy chain variable region mRNA, partial cds.
	787665	3582123	extended					AF062092	Homo sapiens monoclonal antibody RL1 immunoglobulin heavy chain variable region mRNA, partial cds.
	787666	3582124	extended					AF062095	Homo sapiens monoclonal antibody EP16 immunoglobulin heavy chain variable region mRNA, partial cds.
	787667	3582125	extended					AF062097	Homo sapiens clone 21u-2 immunoglobulin heavy chain variable region (IGH) mRNA, partial cds.
	787668	3582126	extended					AF062099	Homo sapiens clone 21u-5 immunoglobulin heavy chain variable region (IGH) mRNA, partial cds.
	787669	3582127	extended					AF062102	Homo sapiens clone 21u-7 IGH pseudogene, partial sequence.
	787670	3582128	full					AF062103	Homo sapiens clone 21u-4 immunoglobulin heavy chain variable region (IGH) mRNA, partial cds.
	787671	3582129	full					AF062105	Homo sapiens clone 21u-13 immunoglobulin heavy chain variable region (IGH) mRNA, partial cds.
	787672	3582130	extended					AF062106	Homo sapiens clone 21u-16 immunoglobulin heavy chain variable region (IGH) mRNA, partial cds.
	787673	3582131	core					AF062107	Homo sapiens clone 21u-19 immunoglobulin heavy chain variable region (IGH) mRNA, partial cds.
	787674	3582132	core					AF062108	Homo sapiens clone 21u-20 immunoglobulin heavy chain variable region (IGH) mRNA, partial cds.
	787675	3582133	full					AF062110	Homo sapiens clone 21u-21 immunoglobulin heavy chain variable region (IGH) mRNA, partial cds.
	787676	3582134	full					AF062111	Homo sapiens clone 21u-22 immunoglobulin heavy chain variable region (IGH) mRNA, partial cds.
	787677	3582135	core					AF062112	Homo sapiens clone 21u-24 immunoglobulin heavy chain variable region (IGH) mRNA, partial cds.
	787678	3582136	core					AF062113	Homo sapiens clone 21u-25 immunoglobulin heavy chain variable region (IGH) mRNA, partial cds.
	787679	3582137	full					AF062114	Homo sapiens clone 21u-26 immunoglobulin heavy chain variable region (IGH) mRNA, partial cds.
	787680	3582138	full					AF062115	Homo sapiens clone 21u-28 immunoglobulin heavy chain variable region (IGH) mRNA, partial cds.
	787681	3582139	full					AF062116	Homo sapiens clone 21u-29 immunoglobulin heavy chain variable region (IGH) mRNA, partial cds.
	787682	3582140	extended					AF062118	Homo sapiens clone 21u-30 immunoglobulin heavy chain variable region (IGH) mRNA, partial cds.
	787683	3582141	extended					AF062119	Homo sapiens clone 21u-31 immunoglobulin heavy chain variable region (IGH) mRNA, partial cds.
	787684	3582142	full					AF062120	Homo sapiens clone 21u-33 immunoglobulin heavy chain variable region (IGH) mRNA, partial cds.
	787685	3582143	full					AF062121	Homo sapiens clone 21u-34 immunoglobulin heavy chain variable region (IGH) mRNA, partial cds.
	787686	3582144	full					AF062122	Homo sapiens clone 21u-39 immunoglobulin heavy chain variable region (IGH) mRNA, partial cds.
	787687	3582145	extended					AF062123	Homo sapiens clone 21u-40 immunoglobulin heavy chain variable region (IGH) mRNA, partial cds.
	787688	3582146	extended					AF062126	Homo sapiens clone 21u-43 immunoglobulin heavy chain variable region (IGH) mRNA, partial cds.
	787689	3582147	full					AF062128	Homo sapiens clone 21u-44 immunoglobulin heavy chain variable region (IGH) mRNA, partial cds.
	787690	3582148	extended					AF062129	Homo sapiens clone 21u-48 immunoglobulin heavy chain variable region (IGH) mRNA, partial cds.
	787691	3582149	full					AF062130	Homo sapiens clone 21u-50 immunoglobulin heavy chain variable region (IGH) mRNA, partial cds.
	787692	3582150	full					AF062132	Homo sapiens clone 21u-51 immunoglobulin heavy chain variable region (IGH) mRNA, partial cds.
	787693	3582151	extended					AF062133	Homo sapiens clone 45u-1 immunoglobulin heavy chain variable region (IGH) mRNA, partial cds.
	787694	3582152	full					AF062134	Homo sapiens clone 45u-3 immunoglobulin heavy chain variable region (IGH) mRNA, partial cds.
	787695	3582153	extended					AF062135	Homo sapiens clone 45u-4 immunoglobulin heavy chain variable region (IGH) mRNA, partial cds.
	787696	3582154	extended					AF062136	Homo sapiens clone 45u-5 immunoglobulin heavy chain variable region (IGH) mRNA, partial cds.
	787697	3582155	extended					AF062137	Homo sapiens clone 45u-6 immunoglobulin heavy chain variable region (IGH) mRNA, partial cds.
	787698	3582156	extended					AF062138	Homo sapiens clone 45u-7 immunoglobulin heavy chain variable region (IGH) mRNA, partial cds.
	787699	3582157	extended					AF062140	Homo sapiens clone 45u-8 immunoglobulin heavy chain variable region (IGH) mRNA, partial cds.
	787700	3582158	extended					AF062141	Homo sapiens clone 45u-11 immunoglobulin heavy chain variable region (IGH) mRNA, partial cds.
	787701	3582159	full					AF062142	Homo sapiens clone 45u-13 immunoglobulin heavy chain variable region (IGH) mRNA, partial cds.
	787702	3582160	full					AF062143	Homo sapiens clone 45u-14 immunoglobulin heavy chain variable region (IGH) mRNA, partial cds.
	787703	3582161	extended					AF062145	Homo sapiens clone 45u-15 immunoglobulin heavy chain variable region (IGH) mRNA, partial cds.
	787704	3582162	extended					AF062146	Homo sapiens clone 45u-17 immunoglobulin heavy chain variable region (IGH) mRNA, partial cds.
	787705	3582163	core					AF062147	Homo sapiens clone 45u-19 immunoglobulin heavy chain variable region (IGH) mRNA, partial cds.
	787706	3582164	core					AF062148	Homo sapiens clone 45u-21 immunoglobulin heavy chain variable region (IGH) mRNA, partial cds.
	787707	3582165	core					AF062149	Homo sapiens clone 45u-22 immunoglobulin heavy chain variable region (IGH) mRNA, partial cds.
	787708	3582166	core					AF062150	Homo sapiens clone 45u-23 immunoglobulin heavy chain variable region (IGH) mRNA, partial cds.
	787709	3582167	core					AF062151	Homo sapiens clone 45u-24 immunoglobulin heavy chain variable region (IGH) mRNA, partial cds.
	787710	3582168	full					AF062152	Homo sapiens clone 45u-25 immunoglobulin heavy chain variable region (IGH) mRNA, partial cds.
	787711	3582169	extended					AF062153	Homo sapiens clone 45u-26 immunoglobulin heavy chain variable region (IGH) mRNA, partial cds.
	787712	3582170	extended					AF062155	Homo sapiens clone 45u-27 immunoglobulin heavy chain variable region (IGH) mRNA, partial cds.

	787713	3582171	core					AF062158	Homo sapiens clone 45u-28 immunoglobulin heavy chain variable region (IGH) mRNA, partial cds.
	787714	3582172	core					AF062159	Homo sapiens clone 45u-30 immunoglobulin heavy chain variable region (IGH) mRNA, partial cds.
	787715	3582173	core					AF062161	Homo sapiens clone 45u-33 immunoglobulin heavy chain variable region (IGH) mRNA, partial cds.
	787716	3582174	full					AF062162	Homo sapiens clone 45u-34 immunoglobulin heavy chain variable region (IGH) mRNA, partial cds.
	787717	3582175	core					AF062163	Homo sapiens clone 45u-36 immunoglobulin heavy chain variable region (IGH) mRNA, partial cds.
	787718	3582176	core					AF062165	Homo sapiens clone 45u-37 immunoglobulin heavy chain variable region (IGH) mRNA, partial cds.
	787719	3582177	core					AF062166	Homo sapiens clone 45u-38 immunoglobulin heavy chain variable region (IGH) mRNA, partial cds.
	787720	3582178	core					AF062168	Homo sapiens clone 45u-40 immunoglobulin heavy chain variable region (IGH) mRNA, partial cds.
	787721	3582179	full					AF062169	Homo sapiens clone 45u-41 immunoglobulin heavy chain variable region (IGH) mRNA, partial cds.
	787722	3582180	core					AF062170	Homo sapiens clone 45u-43 immunoglobulin heavy chain variable region (IGH) mRNA, partial cds.
	787723	3582181	full					AF062171	Homo sapiens clone 45u-44 immunoglobulin heavy chain variable region (IGH) mRNA, partial cds.
	787724	3582182	extended					AF062177	Homo sapiens clone 45u-45 immunoglobulin heavy chain variable region (IGH) mRNA, partial cds.
	787725	3582183	extended					AF062178	Homo sapiens clone 45u-46 immunoglobulin heavy chain variable region (IGH) mRNA, partial cds.
	787726	3582184	extended					AF062179	Homo sapiens clone 48u-6 immunoglobulin heavy chain variable region (IGH) mRNA, partial cds.
	787727	3582185	full					AF062182	Homo sapiens clone 48u-7 immunoglobulin heavy chain variable region (IGH) mRNA, partial cds.
	787728	3582186	core					AF062183	Homo sapiens clone 48u-8 immunoglobulin heavy chain variable region (IGH) mRNA, partial cds.
	787729	3582187	core					AF062184	Homo sapiens clone 48u-11 immunoglobulin heavy chain variable region (IGH) mRNA, partial cds.
	787730	3582188	core					AF062185	Homo sapiens clone 48u-12 immunoglobulin heavy chain variable region (IGH) mRNA, partial cds.
	787731	3582189	core					AF062186	Homo sapiens clone 48u-13 immunoglobulin heavy chain variable region (IGH) mRNA, partial cds.
	787732	3582190	core					AF062188	Homo sapiens clone 48u-15 immunoglobulin heavy chain variable region (IGH) mRNA, partial cds.
	787733	3582191	full					AF062190	Homo sapiens clone 48u-17 immunoglobulin heavy chain variable region (IGH) mRNA, partial cds.
	787734	3582192	full					AF062191	Homo sapiens clone 48u-19 immunoglobulin heavy chain variable region (IGH) mRNA, partial cds.
	787735	3582193	extended					AF062192	Homo sapiens clone 48u-25 immunoglobulin heavy chain variable region (IGH) mRNA, partial cds.
	787736	3582194	extended					AF062194	Homo sapiens clone 48u-26 immunoglobulin heavy chain variable region (IGH) mRNA, partial cds.
	787737	3582195	extended					AF062195	Homo sapiens clone 48u-27 immunoglobulin heavy chain variable region (IGH) mRNA, partial cds.
	787738	3582196	extended					AF062196	Homo sapiens clone 48u-30 immunoglobulin heavy chain variable region (IGH) mRNA, partial cds.
	787739	3582197	extended					AF062199	Homo sapiens clone 48u-31 immunoglobulin heavy chain variable region (IGH) mRNA, partial cds.
	787740	3582198	full					AF062200	Homo sapiens clone 48u-35 immunoglobulin heavy chain variable region (IGH) mRNA, partial cds.
	787741	3582199	extended					AF062201	Homo sapiens clone 48u-38 immunoglobulin heavy chain variable region (IGH) mRNA, partial cds.
	787742	3582200	extended					AF062202	Homo sapiens clone 48u-40 immunoglobulin heavy chain variable region (IGH) mRNA, partial cds.
	787743	3582201	full					AF062203	Homo sapiens clone 48u-41 immunoglobulin heavy chain variable region (IGH) mRNA, partial cds.
	787744	3582202	full					AF062204	Homo sapiens clone 48u-42 immunoglobulin heavy chain variable region (IGH) mRNA, partial cds.
	787745	3582203	core					AF062205	Homo sapiens clone 48u-43 immunoglobulin heavy chain variable region (IGH) mRNA, partial cds.
	787746	3582204	core					AF062206	Homo sapiens clone 48u-44 immunoglobulin heavy chain variable region (IGH) mRNA, partial cds.
	787747	3582205	core					AF062208	Homo sapiens clone 48u-46 immunoglobulin heavy chain variable region (IGH) mRNA, partial cds.
	787748	3582206	core					AF062209	Homo sapiens clone 48u-47 immunoglobulin heavy chain variable region (IGH) mRNA, partial cds.
	787749	3582207	full					AF062210	Homo sapiens clone 48u-49 immunoglobulin heavy chain variable region (IGH) mRNA, partial cds.
	787750	3582208	full					AF062211	Homo sapiens clone 48u-50 immunoglobulin heavy chain variable region (IGH) mRNA, partial cds.
	787751	3582209	full					AF062212	Homo sapiens clone 48u-52 immunoglobulin heavy chain variable region (IGH) mRNA, partial cds.
	787752	3582210	extended					AF062213	Homo sapiens clone 48u-53 immunoglobulin heavy chain variable region (IGH) mRNA, partial cds.
	787753	3582211	extended					AF062214	Homo sapiens clone 23u-1 immunoglobulin heavy chain variable region (IGH) mRNA, partial cds.
	787754	3582212	extended					AF062217	Homo sapiens clone 23u-2 immunoglobulin heavy chain variable region (IGH) mRNA, partial cds.
	787755	3582213	extended					AF062218	Homo sapiens clone 23u-3 immunoglobulin heavy chain variable region (IGH) mRNA, partial cds.
	787756	3582214	extended					AF062219	Homo sapiens clone 23u-9 immunoglobulin heavy chain variable region (IGH) mRNA, partial cds.
	787757	3582215	core					AF062220	Homo sapiens clone 23u-10 immunoglobulin heavy chain variable region (IGH) mRNA, partial cds.
	787758	3582216	core					AF062221	Homo sapiens clone 23u-16 immunoglobulin heavy chain variable region (IGH) mRNA, partial cds.
	787759	3582217	core					AF062222	Homo sapiens clone 23u-17 immunoglobulin heavy chain variable region (IGH) mRNA, partial cds.
	787760	3582218	core					AF062223	Homo sapiens clone 23u-20 immunoglobulin heavy chain variable region (IGH) mRNA, partial cds.
	787761	3582219	full					AF062226	Homo sapiens clone 23u-22 immunoglobulin heavy chain variable region (IGH) mRNA, partial cds.
	787762	3582220	extended					AF062228	Homo sapiens clone 23u-23 immunoglobulin heavy chain variable region (IGH) mRNA, partial cds.
		3582221	extended					AF062230	Homo sapiens clone 23u-26 immunoglobulin heavy chain variable region (IGH) mRNA, partial cds.
		3582222	extended					AF062232	Homo sapiens clone 23u-29 immunoglobulin heavy chain variable region (IGH) mRNA, partial cds.
		3582223	extended					AF062233	Homo sapiens clone 23u-31 immunoglobulin heavy chain variable region (IGH) mRNA, partial cds.
		3582224	extended					AF062236	Homo sapiens clone 23u-34 immunoglobulin heavy chain variable region (IGH) mRNA, partial cds.

		3582225	extended					AF062237	Homo sapiens clone 23u-35 immunoglobulin heavy chain variable region (IGH) mRNA, partial cds.
		3582226	extended					AF062239	Homo sapiens clone 23u-40 immunoglobulin heavy chain variable region (IGH) mRNA, partial cds.
		3582227	extended					AF062240	Homo sapiens clone 23u-41 immunoglobulin heavy chain variable region (IGH) mRNA, partial cds.
		3582228	extended					AF062241	Homo sapiens clone 23u-43 immunoglobulin heavy chain variable region (IGH) mRNA, partial cds.
		3582229	extended					AF062242	Homo sapiens clone 23u-44 immunoglobulin heavy chain variable region (IGH) mRNA, partial cds.
		3582230	extended					AF062243	Homo sapiens clone 23u-45 immunoglobulin heavy chain variable region (IGH) mRNA, partial cds.
		3582231	extended					AF062245	Homo sapiens clone 23u-46 immunoglobulin heavy chain variable region (IGH) mRNA, partial cds.
		3582232	full					AF062246	Homo sapiens clone 23u-47 immunoglobulin heavy chain variable region (IGH) mRNA, partial cds.
		3582233	extended					AF062247	Homo sapiens clone 23u-50 immunoglobulin heavy chain variable region (IGH) mRNA, partial cds.
		3582234	extended					AF062249	Homo sapiens clone 23u-51 immunoglobulin heavy chain variable region (IGH) mRNA, partial cds.
		3582235	full					AF062250	Homo sapiens clone 23u-52 immunoglobulin heavy chain variable region (IGH) mRNA, partial cds.
		3582236	full					AF062251	Homo sapiens clone Xu-2 immunoglobulin heavy chain variable region (IGH) mRNA, partial cds.
		3582237	full					AF062252	Homo sapiens clone Xu-3 immunoglobulin heavy chain variable region (IGH) mRNA, partial cds.
		3582238	extended					AF062255	Homo sapiens clone Xu-6 immunoglobulin heavy chain variable region (IGH) mRNA, partial cds.
		3582239	extended					AF062256	Homo sapiens clone Xu-7 immunoglobulin heavy chain variable region (IGH) mRNA, partial cds.
		3582240	extended					AF062257	Homo sapiens clone Xu-10 immunoglobulin heavy chain variable region (IGH) mRNA, partial cds.
		3582241	full					AF062258	Homo sapiens clone Xu-11 immunoglobulin heavy chain variable region (IGH) mRNA, partial cds.
		3582242	full					AF062261	Homo sapiens clone Xu-12 immunoglobulin heavy chain variable region (IGH) mRNA, partial cds.
		3582243	full					AF062262	Homo sapiens clone Xu-13 immunoglobulin heavy chain variable region (IGH) mRNA, partial cds.
		3582244	full					AF062263	Homo sapiens clone Xu-16 immunoglobulin heavy chain variable region (IGH) mRNA, partial cds.
		3582245	extended					AF062265	Homo sapiens clone Xu-17 immunoglobulin heavy chain variable region (IGH) mRNA, partial cds.
		3582246	extended					AF062266	Homo sapiens clone Xu-18 immunoglobulin heavy chain variable region (IGH) mRNA, partial cds.
		3582247	full					AF062267	Homo sapiens clone Xu-20 immunoglobulin heavy chain variable region (IGH) mRNA, partial cds.
		3582248	extended					AF062268	Homo sapiens clone Xu-21 immunoglobulin heavy chain variable region (IGH) mRNA, partial cds.
		3582249	extended					AF062269	Homo sapiens clone Xu-25 immunoglobulin heavy chain variable region (IGH) mRNA, partial cds.
		3582250	full					AF062270	Homo sapiens clone Xu-28 immunoglobulin heavy chain variable region (IGH) mRNA, partial cds.
		3582251	extended					AF062271	Homo sapiens clone Xu-31 immunoglobulin heavy chain variable region (IGH) mRNA, partial cds.
		3582252	extended					AF062272	Homo sapiens clone Xu-42 immunoglobulin heavy chain variable region (IGH) mRNA, partial cds.
		3582253	full					AF062273	Homo sapiens clone Xu-44 immunoglobulin heavy chain variable region (IGH) mRNA, partial cds.
		3582254	full					AF062275	Homo sapiens clone Xu-45 immunoglobulin heavy chain variable region (IGH) mRNA, partial cds.
		3582255	full					AF062276	Homo sapiens clone Xu-46 immunoglobulin heavy chain variable region (IGH) mRNA, partial cds.
		3582256	core					AF062277	Homo sapiens clone Xu-47 immunoglobulin heavy chain variable region (IGH) mRNA, partial cds.
		3582257	core					AF062278	Homo sapiens clone Xu-48 immunoglobulin heavy chain variable region (IGH) mRNA, partial cds.
		3582258	core					AF062280	Homo sapiens clone Xu-49 immunoglobulin heavy chain variable region (IGH) mRNA, partial cds.
		3582259	core					AF062281	Homo sapiens clone Xu-50 immunoglobulin heavy chain variable region (IGH) mRNA, partial cds.
		3582260	full					AF062283	Homo sapiens clone Xu-52 immunoglobulin heavy chain variable region (IGH) mRNA, partial cds.
		3582261	extended					AF062284	Homo sapiens clone Xu-53 immunoglobulin heavy chain variable region (IGH) mRNA, partial cds.
		3582262	extended					AF062285	Homo sapiens clone Xu-55 immunoglobulin heavy chain variable region (IGH) mRNA, partial cds.
		3582263	extended					AF062286	Homo sapiens clone Xu-56 immunoglobulin heavy chain variable region (IGH) mRNA, partial cds.
		3582264	extended					AF062289	Homo sapiens clone Xu-57 immunoglobulin heavy chain variable region (IGH) mRNA, partial cds.
		3582265	extended					AF062291	Homo sapiens clone Xu-58 immunoglobulin heavy chain variable region (IGH) mRNA, partial cds.
		3582266	extended					AF062292	Homo sapiens clone 48sc-4 immunoglobulin heavy chain variable region (IGH) mRNA, partial cds.
		3582267	extended					AF062293	Homo sapiens clone 48sc-13 immunoglobulin heavy chain variable region (IGH) mRNA, partial cds.
		3582268	extended					AF062294	Homo sapiens clone 48sc-15 immunoglobulin heavy chain variable region (IGH) mRNA, partial cds.
		3582269	extended					AF062295	Homo sapiens clone 48sc-16 immunoglobulin heavy chain variable region (IGH) mRNA, partial cds.
		3582270	extended					AF062297	Homo sapiens clone 48sc-27 immunoglobulin heavy chain variable region (IGH) mRNA, partial cds.
		3582271	extended					AF062298	Homo sapiens clone 48sc-28 immunoglobulin heavy chain variable region (IGH) mRNA, partial cds.
		3582272	extended					AF062301	Homo sapiens clone 48sc-31 immunoglobulin heavy chain variable region (IGH) mRNA, partial cds.
		3582273	extended					AF062302	Homo sapiens clone 48sc-33 immunoglobulin heavy chain variable region (IGH) mRNA, partial cds.
		3582274	extended					AF062303	Homo sapiens clone 48sc-76 immunoglobulin heavy chain variable region (IGH) mRNA, partial cds.
		3582275	full					AF062304	Homo sapiens clone 48sc-88 immunoglobulin heavy chain variable region (IGH) mRNA, partial cds.
		3582276	core					AF062306	Homo sapiens clone 48sc-91 immunoglobulin heavy chain variable region (IGH) mRNA, partial cds.
		3582277	core					AF062307	Homo sapiens clone 48sc-93 immunoglobulin heavy chain variable region (IGH) mRNA, partial cds.
		3582278	core					AF067420	Homo sapiens clone 48sc-97 immunoglobulin heavy chain variable region (IGH) mRNA, partial cds.

		3582279	extended					AF069905	Homo sapiens clone 48sc-100 immunoglobulin heavy chain variable region (IGH) mRNA, partial cds.
		3582280	core					AF082193	Homo sapiens SNC73 protein (SNC73) mRNA, complete cds.
		3582281	core					AF082194	Homo sapiens anti-HLA-A2 immunoglobulin mu heavy chain variable region (IGHM) mRNA, partial cds.
		3582282	extended					AF082195	Homo sapiens clone 2.64 anti-pneumococcal Ig H-chain Fab fragment (VH1-N-D3-3-N-JH4) mRNA, partial cds.
		3582283	extended					AF082196	Homo sapiens clone 4.14 anti-pneumococcal/anti-dsDNA Ig H-chain Fab fragment (VH3-D6-13-JH6) mRNA, partial cds.
		3582284	extended					AF082197	Homo sapiens clone 5.5 anti-pneumococcal/anti-dsDNA Ig H-chain Fab fragment (VH3-D-JH4) mRNA, partial cds.
		3582285	full					AF082199	Homo sapiens clone 1.6 anti-pneumococcal/anti-dsDNA Ig H-chain Fab fragment (VH3-D-JH6) mRNA, partial cds.
		3582286	extended					AF082200	Homo sapiens clone 10.82 anti-pneumococcal/anti-dsDNA Ig H-chain Fab fragment (VH3-N-D2-2-JH6) mRNA, partial cds.
		3582287	extended					AF089001	Homo sapiens clone 8.55 anti-pneumococcal/anti-dsDNA Ig H-chain Fab fragment (VH3-D-JH4) mRNA, partial cds.
		3582288	extended					AF090138	Homo sapiens clone 6.29 anti-pneumococcal/anti-dsDNA Ig H-chain Fab fragment (VH6-D-JH4) mRNA, partial cds.
		3582289	extended					AF090139	Homo sapiens IgA type multiple myeloma clone PEF-IgD4 immunoglobulin heavy chain variable region (VH4) mRNA,
		3582290	extended					AF092502	VH4-DP63 allele, partial cds.
		3582291	extended					AF098360	Homo sapiens IgA type multiple myeloma clone PEF-IgD6 immunoglobulin heavy chain variable region (VH4) mRNA,
		3582292	core					AF099198	VH4-DP63 allele, partial cds.
		3582293	core					AF099199	Homo sapiens IgA type multiple myeloma clone PEF-IgM1 immunoglobulin heavy chain variable region (VH4) mRNA,
		3582294	core					AF099201	VH4-DP63 allele, partial cds.
		3582295	core					AF103027	Homo sapiens immunoglobulin gamma subclass 3 heavy chain variable region mRNA, partial cds.
		3582296	full					AF103028	Homo sapiens clone HuF15-VH immunoglobulin heavy chain variable region mRNA, partial cds.
		3582297	core					AF103030	Homo sapiens TreVH immunoglobulin IgM heavy chain variable region mRNA, partial cds.
		3582298	core					AF103031	Homo sapiens YarVH immunoglobulin IgM heavy chain variable region mRNA, partial cds.
		3582299	extended					AF103060	Homo sapiens JakVH immunoglobulin IgM heavy chain variable region mRNA, partial cds.
		3582300	extended					AF103061	Homo sapiens isolate donor D clone D10 immunoglobulin heavy chain variable region mRNA, partial cds.
		3582301	extended					AF103063	Homo sapiens isolate donor D clone D101 immunoglobulin heavy chain variable region mRNA, partial cds.
		3582302	extended					AF103067	Homo sapiens isolate donor D clone D103 immunoglobulin heavy chain variable region mRNA, partial cds.
		3582303	extended					AF103075	Homo sapiens isolate donor D clone D104 immunoglobulin heavy chain variable region mRNA, partial cds.
		3582304	extended					AF103080	Homo sapiens isolate donor D clone D3 immunoglobulin heavy chain variable region mRNA, partial cds.
		3582305	extended					AF103084	Homo sapiens isolate donor D clone D30 immunoglobulin heavy chain variable region mRNA, partial cds.
		3582306	extended					AF103090	Homo sapiens isolate donor D clone D32 immunoglobulin heavy chain variable region mRNA, partial cds.
		3582307	extended					AF103094	Homo sapiens isolate donor D clone D37 immunoglobulin heavy chain variable region mRNA, partial cds.
		3582308	extended					AF103095	Homo sapiens isolate donor D clone D55 immunoglobulin heavy chain variable region mRNA, partial cds.
		3582309	extended					AF103096	Homo sapiens isolate donor D clone D60 immunoglobulin heavy chain variable region mRNA, partial cds.
		3582310	extended					AF103104	Homo sapiens isolate donor D clone D67 immunoglobulin heavy chain variable region mRNA, partial cds.
		3582311	extended					AF103114	Homo sapiens isolate donor D clone D79 immunoglobulin heavy chain variable region mRNA, partial cds.
		3582312	extended					AF103118	Homo sapiens isolate donor D clone D83 immunoglobulin heavy chain variable region mRNA, partial cds.
		3582313	extended					AF103121	Homo sapiens isolate donor D clone D85 immunoglobulin heavy chain variable region mRNA, partial cds.
		3582314	full					AF103124	Homo sapiens isolate donor D clone D87 immunoglobulin heavy chain variable region mRNA, partial cds.
		3582315	extended					AF103140	Homo sapiens clone N10 immunoglobulin heavy chain variable region mRNA, partial cds.
		3582316	extended					AF103142	Homo sapiens clone N116 immunoglobulin heavy chain variable region mRNA, partial cds.
		3582317	full					AF103148	Homo sapiens clone N12 immunoglobulin heavy chain variable region mRNA, partial cds.
		3582318	full					AF103153	Homo sapiens clone N123 immunoglobulin heavy chain variable region mRNA, partial cds.
		3582319	full					AF103159	Homo sapiens clone N126 immunoglobulin heavy chain variable region mRNA, partial cds.
		3582320	extended					AF103164	Homo sapiens clone N145 immunoglobulin heavy chain variable region mRNA, partial cds.
		3582321	full					AF103169	Homo sapiens clone N147 immunoglobulin heavy chain variable region mRNA, partial cds.
		3582322	extended					AF103172	Homo sapiens clone N154 immunoglobulin heavy chain variable region mRNA, partial cds.
		3582323	extended					AF103182	Homo sapiens clone N16 immunoglobulin heavy chain variable region mRNA, partial cds.
		3582324	core					AF103184	Homo sapiens clone N17 immunoglobulin heavy chain variable region mRNA, partial cds.
		3582325	core					AF103185	Homo sapiens clone N177 immunoglobulin heavy chain variable region mRNA, partial cds.
		3582326	core					AF103191	Homo sapiens clone N181 immunoglobulin heavy chain variable region mRNA, partial cds.
		3582327	extended					AF103197	Homo sapiens clone N184 immunoglobulin heavy chain variable region mRNA, partial cds.
		3582328	extended					AF103205	Homo sapiens clone N195 immunoglobulin heavy chain variable region mRNA, partial cds.
		3582329	core					AF103210	Homo sapiens clone N197 immunoglobulin heavy chain variable region mRNA, partial cds.
		3582330	extended					AF103214	Homo sapiens clone N198 immunoglobulin heavy chain variable region mRNA, partial cds.
		3582331	extended					AF103218	Homo sapiens clone N203 immunoglobulin heavy chain variable region mRNA, partial cds.
		3582332	extended					AF103221	Homo sapiens clone N21 immunoglobulin heavy chain variable region mRNA, partial cds.

		3582333	extended					AF103230	Homo sapiens clone N217 immunoglobulin heavy chain variable region mRNA, partial cds.
		3582334	extended					AF103232	Homo sapiens clone N222 immunoglobulin heavy chain variable region mRNA, partial cds.
		3582335	extended					AF103242	Homo sapiens clone N227 immunoglobulin heavy chain variable region mRNA, partial cds.
		3582336	extended					AF103252	Homo sapiens clone N230 immunoglobulin heavy chain variable region mRNA, partial cds.
		3582337	extended					AF103255	Homo sapiens clone N237 immunoglobulin heavy chain variable region mRNA, partial cds.
		3582338	full					AF103271	Homo sapiens clone N27 immunoglobulin heavy chain variable region mRNA, partial cds.
		3582339	extended					AF103272	Homo sapiens clone N29 immunoglobulin heavy chain variable region mRNA, partial cds.
		3582340	full					AF103273	Homo sapiens clone N40 immunoglobulin heavy chain variable region mRNA, partial cds.
		3582341	extended					AF103286	Homo sapiens clone N5 immunoglobulin heavy chain variable region mRNA, partial cds.
		3582342	full					AF103290	Homo sapiens clone N53 immunoglobulin heavy chain variable region mRNA, partial cds.
		3582343	full					AF103298	Homo sapiens clone N71 immunoglobulin heavy chain variable region mRNA, partial cds.
		3582344	full					AF103300	Homo sapiens clone N72 immunoglobulin heavy chain variable region mRNA, partial cds.
		3582345	extended					AF103301	Homo sapiens clone N73 immunoglobulin heavy chain variable region mRNA, partial cds.
		3582346	extended					AF103309	Homo sapiens clone N86 immunoglobulin heavy chain variable region mRNA, partial cds.
		3582347	extended					AF103313	Homo sapiens clone N9 immunoglobulin heavy chain variable region mRNA, partial cds.
		3582348	full					AF103317	Homo sapiens clone Z100 immunoglobulin heavy chain variable region mRNA, partial cds.
		3582349	extended					AF103318	Homo sapiens clone Z103 immunoglobulin heavy chain variable region mRNA, partial cds.
		3582350	extended					AF103321	Homo sapiens clone Z104 immunoglobulin heavy chain variable region mRNA, partial cds.
		3582351	extended					AF103322	Homo sapiens clone Z112 immunoglobulin heavy chain variable region mRNA, partial cds.
		3582352	extended					AF103325	Homo sapiens clone Z118 immunoglobulin heavy chain variable region mRNA, partial cds.
		3582353	extended					AF103326	Homo sapiens clone Z123 immunoglobulin heavy chain variable region mRNA, partial cds.
		3582354	extended					AF103328	Homo sapiens clone Z125 immunoglobulin heavy chain variable region mRNA, partial cds.
		3582355	full					AF103329	Homo sapiens clone Z129 immunoglobulin heavy chain variable region mRNA, partial cds.
		3582356	extended					AF103330	Homo sapiens clone Z19 immunoglobulin heavy chain variable region mRNA, partial cds.
		3582357	extended					AF103331	Homo sapiens clone Z21 immunoglobulin heavy chain variable region mRNA, partial cds.
		3582358	extended					AF103335	Homo sapiens clone Z22 immunoglobulin heavy chain variable region mRNA, partial cds.
		3582359	extended					AF103338	Homo sapiens clone Z26 immunoglobulin heavy chain variable region mRNA, partial cds.
		3582360	extended					AF103343	Homo sapiens clone Z27 immunoglobulin heavy chain variable region mRNA, partial cds.
		3582361	core					AF103352	Homo sapiens clone Z28 immunoglobulin heavy chain variable region mRNA, partial cds.
		3582362	core					AF103354	Homo sapiens clone Z29 immunoglobulin heavy chain variable region mRNA, partial cds.
		3582363	core					AF103357	Homo sapiens clone Z33 immunoglobulin heavy chain variable region mRNA, partial cds.
		3582364	full					AF103360	Homo sapiens clone Z36 immunoglobulin heavy chain variable region mRNA, partial cds.
		3582365	core					AF103363	Homo sapiens clone Z41 immunoglobulin heavy chain variable region mRNA, partial cds.
		3582366	full					AF103365	Homo sapiens clone Z57 immunoglobulin heavy chain variable region mRNA, partial cds.
		3582367	full					AF103369	Homo sapiens clone Z59 immunoglobulin heavy chain variable region mRNA, partial cds.
		3582368	full					AF103371	Homo sapiens clone Z64 immunoglobulin heavy chain variable region mRNA, partial cds.
		3582369	extended					AF103768	Homo sapiens clone Z69 immunoglobulin heavy chain variable region mRNA, partial cds.
		3582370	extended					AF103769	Homo sapiens clone Z75 immunoglobulin heavy chain variable region mRNA, partial cds.
		3582371	extended					AF107235	Homo sapiens clone Z81 immunoglobulin heavy chain variable region mRNA, partial cds.
		3582372	extended					AF107236	Homo sapiens clone Z91 immunoglobulin heavy chain variable region mRNA, partial cds.
		3582373	extended					AF107237	Homo sapiens clone Z97 immunoglobulin heavy chain variable region mRNA, partial cds.
		3582374	extended					AF107239	Homo sapiens clone B14 anti-phospholipid immunoglobulin heavy chain VH-D-J region mRNA, partial cds.
		3582375	core					AF107240	Homo sapiens clone B22 anti-phospholipid immunoglobulin heavy chain VH-D-J region mRNA, partial cds.
		3582376	core					AF109406	Homo sapiens clone MD05 immunoglobulin heavy chain variable region mRNA, partial cds.
		3582377	full					AF110149	Homo sapiens clone MD06 immunoglobulin heavy chain variable region mRNA, partial cds.
		3582378	full					AF115108	Homo sapiens clone MD07 immunoglobulin heavy chain variable region mRNA, partial cds.
		3582379	extended					AF115109	Homo sapiens clone MD09 immunoglobulin heavy chain variable region mRNA, partial cds.
		3582380	extended					AF115110	Homo sapiens clone MD10 immunoglobulin heavy chain variable region mRNA, partial cds.
		3582381	extended					AF115111	Homo sapiens immunoglobin heavy chain mRNA, partial cds.
		3582382	extended					AF115112	Homo sapiens immunoglobulin heavy chain mRNA, partial cds.
		3582383	extended					AF115113	Homo sapiens clone dn2 immunoglobulin heavy chain variable region mRNA, partial cds.
		3582384	extended					AF115116	Homo sapiens clone dn3 immunoglobulin heavy chain variable region mRNA, partial cds.
		3582385	extended					AF115117	Homo sapiens clone dn4 immunoglobulin heavy chain variable region mRNA, partial cds.
		3582386	core					AF115120	Homo sapiens clone dn5 immunoglobulin heavy chain variable region mRNA, partial cds.

		3582387	extended					AF115121	Homo sapiens clone dn6 immunoglobulin heavy chain variable region mRNA, partial cds.
		3582388	extended					AF115122	Homo sapiens clone dn7 immunoglobulin heavy chain variable region mRNA, partial cds.
		3582389	full					AF115125	Homo sapiens clone dn10 immunoglobulin heavy chain variable region mRNA, partial cds.
		3582390	extended					AF115128	Homo sapiens clone dn11 immunoglobulin heavy chain variable region mRNA, partial cds.
		3582391	extended					AF115129	Homo sapiens clone sc1 immunoglobulin heavy chain variable region mRNA, partial cds.
		3582392	extended					AF115130	Homo sapiens clone sc2 immunoglobulin heavy chain variable region mRNA, partial cds.
		3582393	extended					AF115132	Homo sapiens clone sc3 immunoglobulin heavy chain variable region mRNA, partial cds.
		3582394	extended					AF115134	Homo sapiens clone sc6 immunoglobulin heavy chain variable region mRNA, partial cds.
		3582395	extended					AF119797	Homo sapiens clone sc9 immunoglobulin heavy chain variable region mRNA, partial cds.
		3582396	core					AF123351	Homo sapiens clone sc10 immunoglobulin heavy chain variable region mRNA, partial cds.
		3582397	core					AF123354	Homo sapiens clone sc11 immunoglobulin heavy chain variable region mRNA, partial cds.
		3582398	full					AF123355	Homo sapiens clone sc13 immunoglobulin heavy chain variable region mRNA, partial cds.
		3582399	core					AF123356	Homo sapiens clone sc15 immunoglobulin heavy chain variable region mRNA, partial cds.
		3582400	full					AF123357	Homo sapiens clone 1362 immunoglobulin IgM heavy chain variable region mRNA, partial cds.
		3582401	core					AF123358	Homo sapiens clone T12 immunoglobulin heavy chain variable region mRNA, partial cds.
		3582402	core					AF123359	Homo sapiens clone T3 nonfunctional immunoglobulin heavy chain variable region mRNA, partial sequence.
		3582403	extended					AF123360	Homo sapiens clone T5 immunoglobulin heavy chain variable region mRNA, partial cds.
		3582404	extended					AF123361	Homo sapiens clone T4 immunoglobulin heavy chain variable region mRNA, partial cds.
		3582405	core					AF123362	Homo sapiens clone T6 immunoglobulin heavy chain variable region mRNA, partial cds.
		3582406	core					AF123364	Homo sapiens clone T7 immunoglobulin heavy chain variable region mRNA, partial cds.
		3582407	extended					AF123365	Homo sapiens clone T9 immunoglobulin heavy chain variable region mRNA, partial cds.
		3582408	full					AF123367	Homo sapiens clone T10 nonfunctional immunoglobulin heavy chain variable region mRNA, partial sequence.
		3582409	full					AF123369	Homo sapiens clone T11 immunoglobulin heavy chain variable region mRNA, partial cds.
		3582410	full					AF123371	Homo sapiens clone T8 immunoglobulin heavy chain variable region mRNA, partial cds.
		3582411	extended					AF123372	Homo sapiens clone T14 immunoglobulin heavy chain variable region mRNA, partial cds.
		3582412	extended					AF123373	Homo sapiens clone C1 immunoglobulin heavy chain variable region mRNA, partial cds.
		3582413	full					AF123374	Homo sapiens clone C3 immunoglobulin heavy chain variable region mRNA, partial cds.
		3582414	full					AF123375	Homo sapiens clone C5 immunoglobulin heavy chain variable region mRNA, partial cds.
		3582415	full					AF123376	Homo sapiens clone C7 immunoglobulin heavy chain variable region mRNA, partial cds.
		3582416	extended					AF123377	Homo sapiens clone C8 immunoglobulin heavy chain variable region mRNA, partial cds.
		3582417	extended					AF123379	Homo sapiens clone C9 immunoglobulin heavy chain variable region mRNA, partial cds.
		3582418	full					AF135164	Homo sapiens clone C10 immunoglobulin heavy chain variable region mRNA, partial cds.
		3582419	core					AF151702	Homo sapiens clone C11 immunoglobulin heavy chain variable region mRNA, partial cds.
		3582420	full					AF151703	Homo sapiens clone C12 immunoglobulin heavy chain variable region mRNA, partial cds.
		3582421	full					AF151704	Homo sapiens clone C13 nonfunctional immunoglobulin heavy chain variable region mRNA, partial sequence.
		3582422	full					AF158380	Homo sapiens clone C15 immunoglobulin heavy chain variable region mRNA, partial cds.
		3582423	full					AF165100	Homo sapiens immunoglobulin heavy chain precursor (IGH@) mRNA, partial cds.
		3582424	full					AF165102	Homo sapiens clone 19 immunoglobulin IgG heavy chain variable region mRNA, partial cds.
		3582425	extended					AF165105	Homo sapiens clone 33 immunoglobulin IgG heavy chain variable region mRNA, partial cds.
		3582426	extended					AF165109	Homo sapiens clone 22 immunoglobulin IgG heavy chain variable region mRNA, partial cds.
		3582427	extended					AF166264	Homo sapiens clone 1-224 immunoglobulin heavy chain variable region mRNA, partial cds.
		3582428	extended					AF174006	Homo sapiens clone 6B.1 immunoglobulin heavy chain variable region DP-54 mRNA, partial cds.
		3582429	extended					AF174007	Homo sapiens clone 23F.2 immunoglobulin heavy chain variable region DP-35 mRNA, partial cds.
		3582430	extended					AF174010	Homo sapiens clone 14.3 immunoglobulin heavy chain variable region DP-47 mRNA, partial cds.
		3582431	full					AF174012	Homo sapiens clone 14.2 immunoglobulin heavy chain variable region LSG6.1 mRNA, partial cds.
		3582432	extended					AF174013	Homo sapiens immunoglobulin gamma1 heavy chain variable region mRNA, partial cds.
		3582433	extended					AF174015	Homo sapiens clone 77u-a02 immunoglobulin heavy chain variable region precursor (IgH) mRNA, partial cds.
		3582434	core					AF174016	Homo sapiens clone 77u-a03 IgH pseudogene, partial sequence.
		3582435	core					AF174018	Homo sapiens clone 77u-a06 immunoglobulin heavy chain variable region precursor (IgH) mRNA, partial cds.
		3582436	core					AF174019	Homo sapiens clone 77u-a09 immunoglobulin heavy chain variable region precursor (IgH) mRNA, partial cds.
		3582437	extended					AF174020	Homo sapiens clone 77u-a10 immunoglobulin heavy chain variable region precursor (IgH) mRNA, partial cds.
		3582438	extended					AF174021	Homo sapiens clone 77u-a12 immunoglobulin heavy chain variable region precursor (IgH) mRNA, partial cds.
		3582439	extended					AF174022	Homo sapiens clone 77u-a14 immunoglobulin heavy chain variable region precursor (IgH) mRNA, partial cds.
		3582440	extended					AF174024	Homo sapiens clone 77u-a19 immunoglobulin heavy chain variable region precursor (IgH) mRNA, partial cds.

		3582441	extended					AF174025	Homo sapiens clone 77u-b02 immunoglobulin heavy chain variable region precursor (IgH) mRNA, partial cds.
		3582442	extended					AF174028	Homo sapiens clone 77u-b04 IgH pseudogene, partial sequence.
		3582443	extended					AF174029	Homo sapiens clone 77u-c01 immunoglobulin heavy chain variable region precursor (IgH) mRNA, partial cds.
		3582444	extended					AF174030	Homo sapiens clone 77u-c04 immunoglobulin heavy chain variable region precursor (IgH) mRNA, partial cds.
		3582445	full					AF174033	Homo sapiens clone 77u-c06 immunoglobulin heavy chain variable region precursor (IgH) mRNA, partial cds.
		3582446	full					AF174034	Homo sapiens clone 77u-c07 immunoglobulin heavy chain variable region precursor (IgH) mRNA, partial cds.
		3582447	extended					AF174036	Homo sapiens clone 77u-c10 immunoglobulin heavy chain variable region precursor (IgH) mRNA, partial cds.
		3582448	extended					AF174037	Homo sapiens clone 77u-c14 immunoglobulin heavy chain variable region precursor (IgH) mRNA, partial cds.
		3582449	extended					AF174046	Homo sapiens clone 77u-c17 immunoglobulin heavy chain variable region precursor (IgH) mRNA, partial cds.
		3582450	extended					AF174048	Homo sapiens clone 77u-c20 immunoglobulin heavy chain variable region precursor (IgH) mRNA, partial cds.
		3582451	extended					AF174056	Homo sapiens clone 77u-c26 immunoglobulin heavy chain variable region precursor (IgH) mRNA, partial cds.
		3582452	core					AF174057	Homo sapiens clone 77u-c29 immunoglobulin heavy chain variable region precursor (IgH) mRNA, partial cds.
		3582453	extended					AF174059	Homo sapiens clone sc48u-01 immunoglobulin heavy chain variable region (IgH) mRNA, partial cds.
		3582454	extended					AF174060	Homo sapiens clone sc48u-19 immunoglobulin heavy chain variable region (IgH) mRNA, partial cds.
		3582455	extended					AF174063	Homo sapiens clone sc48u-21 immunoglobulin heavy chain variable region (IgH) mRNA, partial cds.
		3582456	extended					AF174066	Homo sapiens clone sc48u-37 immunoglobulin heavy chain variable region (IgH) mRNA, partial cds.
		3582457	extended					AF174068	Homo sapiens clone sc48u-38 immunoglobulin heavy chain variable region (IgH) mRNA, partial cds.
		3582458	extended					AF174069	Homo sapiens clone sc48u-43 immunoglobulin heavy chain variable region (IgH) mRNA, partial cds.
		3582459	extended					AF174070	Homo sapiens clone sc48u-46 immunoglobulin heavy chain variable region (IgH) mRNA, partial cds.
		3582460	full					AF174074	Homo sapiens clone sc48u-51 immunoglobulin heavy chain variable region (IgH) mRNA, partial cds.
		3582461	extended					AF174077	Homo sapiens clone sc48u-55 immunoglobulin heavy chain variable region (IgH) mRNA, partial cds.
		3582462	extended					AF174080	Homo sapiens clone sc48u-74 immunoglobulin heavy chain variable region (IgH) mRNA, partial cds.
		3582463	full					AF174082	Homo sapiens clone sc48u-75 immunoglobulin heavy chain variable region (IgH) mRNA, partial cds.
		3582464	full					AF174083	Homo sapiens clone sc48u-79 immunoglobulin heavy chain variable region (IgH) mRNA, partial cds.
		3582465	extended					AF174084	Homo sapiens clone sc48u-86 immunoglobulin heavy chain variable region (IgH) mRNA, partial cds.
		3582466	extended					AF174085	Homo sapiens clone sc48u-91 immunoglobulin heavy chain variable region (IgH) mRNA, partial cds.
		3582467	extended					AF174086	Homo sapiens clone sc48u-97 immunoglobulin heavy chain variable region (IgH) mRNA, partial cds.
		3582468	extended					AF174088	Homo sapiens clone sc48u-101 immunoglobulin heavy chain variable region (IgH) mRNA, partial cds.
		3582469	extended					AF174090	Homo sapiens clone sc48u-104 immunoglobulin heavy chain variable region (IgH) mRNA, partial cds.
		3582470	core					AF174092	Homo sapiens clone sc48u-106 immunoglobulin heavy chain variable region (IgH) mRNA, partial cds.
		3582471	extended					AF174094	Homo sapiens clone sc48u-108 immunoglobulin heavy chain variable region (IgH) mRNA, partial cds.
		3582472	full					AF174095	Homo sapiens clone sc48u-110 immunoglobulin heavy chain variable region (IgH) mRNA, partial cds.
		3582473	full					AF174097	Homo sapiens clone sc48u-112 immunoglobulin heavy chain variable region (IgH) mRNA, partial cds.
		3582474	full					AF174098	Homo sapiens clone sc77u-03 immunoglobulin heavy chain variable region (IgH) mRNA, partial cds.
		3582475	extended					AF174101	Homo sapiens clone sc77u-05 immunoglobulin heavy chain variable region (IgH) mRNA, partial cds.
		3582476	full					AF174102	Homo sapiens clone sc77u-07 immunoglobulin heavy chain variable region (IgH) mRNA, partial cds.
		3582477	extended					AF174103	Homo sapiens clone sc77u-09 immunoglobulin heavy chain variable region (IgH) mRNA, partial cds.
		3582478	extended					AF174105	Homo sapiens clone sc77u-13 immunoglobulin heavy chain variable region (IgH) mRNA, partial cds.
		3582479	extended					AF174107	Homo sapiens clone sc77u-14 immunoglobulin heavy chain variable region (IgH) mRNA, partial cds.
		3582480	full					AF174108	Homo sapiens clone sc77u-17 immunoglobulin heavy chain variable region (IgH) mRNA, partial cds.
		3582481	extended					AF174110	Homo sapiens clone sc77u-19 immunoglobulin heavy chain variable region (IgH) mRNA, partial cds.
		3582482	extended					AF174112	Homo sapiens clone sc77u-21 immunoglobulin heavy chain variable region (IgH) mRNA, partial cds.
		3582483	extended					AF174113	Homo sapiens clone sc77u-23 immunoglobulin heavy chain variable region (IgH) mRNA, partial cds.
		3582484	full					AF174114	Homo sapiens clone sc77u-29 immunoglobulin heavy chain variable region (IgH) mRNA, partial cds.
		3582485	extended					AF174115	Homo sapiens clone sc77u-30 immunoglobulin heavy chain variable region (IgH) mRNA, partial cds.
		3582486	extended					AF174116	Homo sapiens clone sc77u-32 immunoglobulin heavy chain variable region (IgH) mRNA, partial cds.
		3582487	extended					AF174117	Homo sapiens clone sc77u-35 immunoglobulin heavy chain variable region (IgH) mRNA, partial cds.
		3582488	full					AF174118	Homo sapiens clone sc77u-36 immunoglobulin heavy chain variable region (IgH) mRNA, partial cds.
		3582489	extended					AF174123	Homo sapiens clone sc77u-38 immunoglobulin heavy chain variable region (IgH) mRNA, partial cds.
		3582490	full					AF174125	Homo sapiens clone sc77u-39 immunoglobulin heavy chain variable region (IgH) mRNA, partial cds.
		3582491	extended					AF174126	Homo sapiens clone sc77u-40 immunoglobulin heavy chain variable region (IgH) mRNA, partial cds.
		3582492	extended					AF174127	Homo sapiens clone sc77u-41 immunoglobulin heavy chain variable region (IgH) mRNA, partial cds.
		3582493	extended					AF184762	Homo sapiens clone sc77u-44 immunoglobulin heavy chain variable region (IgH) mRNA, partial cds.
		3582494	extended					AF184764	Homo sapiens clone sc77u-49 immunoglobulin heavy chain variable region (IgH) mRNA, partial cds.

		3582495	extended					AF191092	Homo sapiens clone sc77u-52 immunoglobulin heavy chain variable region (IgH) mRNA, partial cds.
		3582496	full					AF193847	Homo sapiens clone sc77u-54 immunoglobulin heavy chain variable region (IgH) mRNA, partial cds.
		3582497	full					AF194118	Homo sapiens clone sc77u-55 immunoglobulin heavy chain variable region (IgH) mRNA, partial cds.
		3582498	full					AF209733	Homo sapiens IgA1 heavy chain mRNA, partial cds.
		3582499	core					AF209735	Homo sapiens IgG1 heavy chain mRNA, partial cds.
		3582500	core					AF209737	Homo sapiens immunoglobulin heavy chain Fd region mRNA, partial cds.
		3582501	core					AF209739	Homo sapiens clone 3HC immunoglobulin heavy chain variable region mRNA, partial cds.
		3582502	full					AF209741	Homo sapiens clone N246 immunoglobulin heavy chain variable region mRNA, partial cds.
		3582503	core					AF209900	Homo sapiens anti-pneumococcal antibody 1.10 heavy chain variable region mRNA, partial cds.
		3582504	full					AF209902	Homo sapiens anti-pneumococcal antibody 1.2 heavy chain variable region mRNA, partial cds.
		3582505	full					AF211930	Homo sapiens anti-pneumococcal antibody 1.4 heavy chain variable region mRNA, partial cds.
		3582506	extended					AF212993	Homo sapiens anti-pneumococcal antibody 2.1 heavy chain variable region mRNA, partial cds.
		3582507	extended					AF231395	Homo sapiens anti-pneumococcal antibody 1.1 heavy chain variable region mRNA, partial cds.
		3582508	extended					AF231397	Homo sapiens immunoglobulin heavy chain variable region 194-122 mRNA, partial cds.
		3582509	extended					AF231398	Homo sapiens nonfunctional immunoglobulin heavy chain variable region 194-135 mRNA, partial sequence.
		3582510	extended					AF231400	Homo sapiens immunoglobulin heavy chain variable region 194-4 mRNA, partial cds.
		3582511	extended					AF231401	Homo sapiens immunoglobulin heavy chain variable region mRNA, partial cds.
		3582512	full					AF231402	Homo sapiens clone 1-2 immunoglobulin heavy chain variable region mRNA, partial cds.
		3582513	extended					AF231403	Homo sapiens clone 1-4 immunoglobulin heavy chain variable region mRNA, partial cds.
		3582514	extended					AF231404	Homo sapiens clone 1-5 immunoglobulin heavy chain variable region mRNA, partial cds.
		3582515	core					AF245307	Homo sapiens clone 4-2 immunoglobulin heavy chain variable region mRNA, partial cds.
		3582516	full					AF245308	Homo sapiens clone 4-3 nonfunctional immunoglobulin heavy chain variable region mRNA, partial cds.
		3582517	extended					AF245309	Homo sapiens clone 4-4 immunoglobulin heavy chain variable region mRNA, partial cds.
		3582518	core					AF245310	Homo sapiens clone 4-5 immunoglobulin heavy chain variable region mRNA, partial cds.
		3582519	extended					AF245311	Homo sapiens clone 4-6 immunoglobulin heavy chain variable region mRNA, partial cds.
		3582520	extended					AF247737	Homo sapiens IgM immunoglobulin heavy chain variable region mRNA, partial cds.
		3582521	extended					AF262072	Homo sapiens IgD heavy chain variable region mRNA, partial cds.
		3582522	extended					AF262079	Homo sapiens IgG1 immunoglobulin heavy chain variable region mRNA, partial cds.
		3582523	full					AF262081	Homo sapiens IgG3 heavy chain variable region mRNA, partial cds.
		3582524	extended					AF262086	Homo sapiens IgA heavy chain variable region mRNA, partial cds.
		3582525	full					AF262096	Homo sapiens clone 28PCR immunoglobulin alpha chain mRNA, partial cds.
		3582526	extended					AF262097	Homo sapiens clone cp2-004 immunoglobulin heavy chain variable region mRNA, partial cds.
		3582527	core					AF262147	Homo sapiens clone cp2-011 immunoglobulin heavy chain variable region mRNA, partial cds.
		3582528	core					AF262150	Homo sapiens clone cp2-013 immunoglobulin heavy chain variable region mRNA, partial cds.
		3582529	core					AF262154	Homo sapiens clone cp2-018 immunoglobulin heavy chain variable region mRNA, partial cds.
		3582530	core					AF262157	Homo sapiens clone cp2-028 immunoglobulin heavy chain variable region mRNA, partial cds.
		3582531	core					AF262171	Homo sapiens clone cp2-029 immunoglobulin heavy chain variable region mRNA, partial cds.
		3582532	core					AF262173	Homo sapiens clone cp1-003 immunoglobulin heavy chain variable region mRNA, partial cds.
		3582533	core					AF262174	Homo sapiens clone cp1-006 immunoglobulin heavy chain variable region mRNA, partial cds.
		3582534	extended					AF262178	Homo sapiens clone cp1-010 immunoglobulin heavy chain variable region mRNA, partial cds.
		3582535	full					AF262179	Homo sapiens clone cp1-013 immunoglobulin heavy chain variable region mRNA, partial cds.
		3582536	full					AF262189	Homo sapiens clone cp1-027 immunoglobulin heavy chain variable region mRNA, partial cds.
		3582537	full					AF262195	Homo sapiens clone cp1-029 immunoglobulin heavy chain variable region mRNA, partial cds.
		3582538	extended					AF262202	Homo sapiens clone cp1-030 immunoglobulin heavy chain variable region mRNA, partial cds.
		3582539	full					AF262203	Homo sapiens clone cp1-034 immunoglobulin heavy chain variable region mRNA, partial cds.
		3582540	extended					AF262207	Homo sapiens clone cp1-035 immunoglobulin heavy chain variable region mRNA, partial cds.
		3582541	full					AF262208	Homo sapiens clone cp1-045 immunoglobulin heavy chain variable region mRNA, partial cds.
		3582542	extended					AF283666	Homo sapiens clone cp1-051 immunoglobulin heavy chain variable region mRNA, partial cds.
		3582543	extended					AF283781	Homo sapiens clone cp1-hb1-1 immunoglobulin heavy chain variable region mRNA, partial cds.
		3582544	extended					AF283805	Homo sapiens clone cp1-hb1-2 immunoglobulin heavy chain variable region mRNA, partial cds.
		3582545	full					AF284470	Homo sapiens clone cp1-hb1-6 immunoglobulin heavy chain variable region mRNA, partial cds.
		3582546	core					AF301483	Homo sapiens clone cp1-hb1-7 immunoglobulin heavy chain variable region mRNA, partial cds.
		3582547	core					AF302819	Homo sapiens SNC66 protein mRNA, complete cds.
		3582548	full					AF302820	Homo sapiens isolate B-DLCL0041 clone 3 immunoglobulin heavy chain variable region mRNA, partial cds.

		3582549<br>3582550<br>3582551<br>3582552<br>3582553<br>3582554<br>3582555<br>3582556<br>3582557<br>3582558<br>3582559<br>3582560<br>3582561<br>3582562<br>3582563<br>3582564<br>3582565<br>3582566<br>3582567<br>3582568<br>3582569<br>3582570<br>3582571<br>3582572<br>3582573<br>3582574<br>3582575<br>3582576<br>3582577<br>3582578<br>3582579	full<br>extended<br>extended<br>extended<br>full<br>core<br>core<br>full<br>full<br>extended<br>extended<br>extended<br>extended<br>full<br>extended<br>extended<br>extended<br>extended<br>extended<br>extended<br>extended<br>extended<br>extended<br>full<br>core<br>core<br>extended<br>full<br>extended<br>extended<br>extended					AF302822<br>AF306366<br>AF306370<br>AF306371<br>AF306372<br>AF306376<br>AF306377<br>AF308544<br>AF308548<br>AF308550<br>AF308554<br>AF308555<br>AF308557<br>AF308558<br>AF308560<br>AF332144<br>AF336987<br>AF343363<br>AF343422<br>AF348831<br>AF359365<br>AF361063<br>AF361069<br>AF381601<br>AF394201<br>AF396472<br>AF396474<br>AF398955<br>AF398959<br>AF398961<br>AF398963<br>AF398965<br>AF400158<br>AF408719<br>AF408724<br>AF408726<br>AF408728<br>AF408730<br>AF408731<br>AF408735<br>AF408736<br>AF408738<br>AF408740<br>AF417843<br>AF417845<br>AF431049<br>AF431051<br>AF431053<br>AF431055<br>AF442756<br>AF442758<br>AF442765<br>AF442766<br>AF453266	Homo sapiens isolate B-DLCL0013 clone 2 immunoglobulin heavy chain variable region mRNA, partial cds.<br>Homo sapiens immunoglobulin heavy chain variable region mRNA, partial cds.<br>Homo sapiens clone CIC 11 anticardiolipin immunoglobulin heavy chain mRNA, partial cds.<br>Homo sapiens isolate 103 immunoglobulin heavy chain variable region mRNA, partial cds.<br>Homo sapiens isolate 163 immunoglobulin heavy chain variable region mRNA, partial cds.<br>Homo sapiens isolate 83 immunoglobulin heavy chain variable region mRNA, partial cds.<br>Homo sapiens clone T2.11 immunoglobulin heavy chain variable region mRNA, partial cds.<br>Homo sapiens clone T3.4 immunoglobulin heavy chain variable region mRNA, partial cds.<br>Homo sapiens clone T3.5 immunoglobulin heavy chain variable region mRNA, partial cds.<br>Homo sapiens clone T2.2 immunoglobulin heavy chain variable region mRNA, partial cds.<br>Homo sapiens clone T3.13 immunoglobulin heavy chain variable region mRNA, partial cds.<br>Homo sapiens clone T3.15 immunoglobulin heavy chain variable region mRNA, partial cds.<br>Homo sapiens clone ST1L_G19 immunoglobulin heavy chain mRNA, partial cds.<br>Homo sapiens clone ST1L_G29 immunoglobulin heavy chain mRNA, partial cds.<br>Homo sapiens clone ST1L_G8 immunoglobulin heavy chain mRNA, partial cds.<br>Homo sapiens clone ST1R_M10 immunoglobulin heavy chain mRNA, partial cds.<br>Homo sapiens clone ST1R_M17 immunoglobulin heavy chain mRNA, partial cds.<br>Homo sapiens clone ST1R_M31 immunoglobulin heavy chain mRNA, partial cds.<br>Homo sapiens clone ST1R_M5 immunoglobulin heavy chain mRNA, partial cds.<br>Homo sapiens clone ST1R_M8 immunoglobulin heavy chain mRNA, partial cds.<br>Homo sapiens anti-pneumococcal antibody NAD heavy chain variable region mRNA, partial cds.<br>Homo sapiens IgG1/L immunoglobulin heavy chain VH3 variable region mRNA, partial cds.<br>Homo sapiens clone k149-1 psoriasis vulgaris immunoglobulin heavy chain mRNA, partial cds.<br>Homo sapiens clone k177-17 psoriasis vulgaris immunoglobulin heavy chain mRNA, partial cds.<br>Homo sapiens clone 26P-16 immunoglobulin heavy chain variable region mRNA, partial cds.<br>Homo sapiens clone CH205 immunoglobulin heavy chain variable region mRNA, partial cds.<br>Homo sapiens clone 4-29 IgE heavy chain variable region mRNA, partial cds.<br>Homo sapiens clone 5-66 IgE heavy chain variable region mRNA, partial cds.<br>Homo sapiens clone BL2H.1A immunoglobulin heavy chain variable region mRNA, partial cds.<br>Homo sapiens clone B12 anti-GPIIb/IIIa immunoglobulin heavy chain variable region mRNA, partial cds.<br>Homo sapiens clone C6 anti-thrombospondin immunoglobulin heavy chain variable region mRNA, partial cds.<br>Homo sapiens clone D2 anti-thrombospondin immunoglobulin heavy chain variable region mRNA, partial cds.<br>Homo sapiens isolate F7 immunoglobulin heavy chain variable region mRNA, partial cds.<br>Homo sapiens isolate F11 immunoglobulin heavy chain variable region mRNA, partial cds.<br>Homo sapiens isolate F14 immunoglobulin heavy chain variable region mRNA, partial cds.<br>Homo sapiens isolate F16 immunoglobulin heavy chain variable region mRNA, partial cds.<br>Homo sapiens isolate F72 immunoglobulin heavy chain variable region mRNA, partial cds.<br>Homo sapiens anti-hepatitis B surface antigen immunoglobulin heavy chain mRNA, partial cds.<br>Homo sapiens clone 6b5B12 anti-pneumococcal capsular polysaccharide immunoglobulin heavy chain variable region mRNA, partial cds.<br>Homo sapiens clone 6bC3 anti-pneumococcal capsular polysaccharide immunoglobulin heavy chain variable region (IgA) mRNA, partial cds.<br>Homo sapiens clone AbA5h anti-pneumococcal capsular polysaccharide immunoglobulin heavy chain variable region (IgG1) mRNA, partial cds.<br>Homo sapiens clone CbB2 anti-pneumococcal capsular polysaccharide immunoglobulin heavy chain variable region (IgG2) mRNA, partial cds.<br>Homo sapiens clone CbE2 anti-pneumococcal capsular polysaccharide immunoglobulin heavy chain variable region (IgG2) mRNA, partial cds.<br>Homo sapiens clone CbG8h anti-pneumococcal capsular polysaccharide immunoglobulin heavy chain variable region (IgA) mRNA, partial cds.<br>Homo sapiens clone Db7D4 anti-pneumococcal capsular polysaccharide immunoglobulin variable region (IgG2) mRNA, partial cds.<br>Homo sapiens clone Db8C11 anti-pneumococcal capsular polysaccharide immunoglobulin heavy chain variable region (IgA) mRNA, partial cds.

								AF455545<br>AF455546<br>AF455547<br>AF455548<br>AF455553<br>AF455922<br>AF455923<br>AF455924<br>AF459629<br>AF459631<br>AF460662<br>AF460673<br>AF460675<br>AF460678<br>AF460679<br>AF460684<br>AF460700<br>AF460706<br>AF460737<br>AF460804<br>AF460807<br>AF460810<br>AF460966<br>AF460967<br>AF460968<br>AF471167<br>AF471168<br>AF471170<br>AF471171<br>AF471172<br>AF471173<br>AF471174<br>AF471175<br>AF471177<br>AF471179<br>AF471180<br>AF471181<br>AF471183<br>AF471184<br>AF471185<br>AF471186<br>AF471187<br>AF471188<br>AF471189<br>AF471190<br>AF471193<br>AF471194<br>AF471195<br>AF471198<br>AF471199<br>AF471200<br>AF471201<br>AF471202<br>AF471203	Homo sapiens clone DMF4 anti-pneumococcal capsular polysaccharide immunoglobulin heavy chain variable region (IgA) mRNA, partial cds.<br>Homo sapiens clone DM5 anti-pneumococcal capsular polysaccharide immunoglobulin heavy chain variable region (IgG2) mRNA, partial cds.<br>Homo sapiens clone IS1HC immunoglobulin heavy chain mRNA, partial cds.<br>Homo sapiens clone IS4H immunoglobulin heavy chain mRNA, partial cds.<br>Homo sapiens anti-pneumococcal antibody 7C5 heavy chain variable region mRNA, partial cds.<br>Homo sapiens anti-pneumococcal antibody 3H1 heavy chain variable region mRNA, partial cds.<br>Homo sapiens anti-pneumococcal antibody 1A2 heavy chain variable region mRNA, partial cds.<br>Homo sapiens anti-pneumococcal antibody A7 heavy chain variable region mRNA, partial cds.<br>Homo sapiens isolate JC-4 immunoglobulin heavy chain variable region mRNA, partial cds.<br>Homo sapiens isolate JW-5 immunoglobulin heavy chain variable region mRNA, partial cds.<br>Homo sapiens isolate PR-8 immunoglobulin heavy chain variable region mRNA, partial cds.<br>Homo sapiens isolate WD-9 immunoglobulin heavy chain variable region mRNA, partial cds.<br>Homo sapiens clone 11 immunoglobulin heavy chain variable region mRNA, partial cds.<br>Homo sapiens clone RIZMVH1 anti-cardiolipin immunoglobulin heavy chain mRNA, partial cds.<br>Homo sapiens clone RIZMVH2 anti-cardiolipin immunoglobulin heavy chain mRNA, partial cds.<br>Homo sapiens clone RIZMVH3 anti-cardiolipin immunoglobulin heavy chain mRNA, partial cds.<br>Homo sapiens clone RIZMVH4 anti-cardiolipin immunoglobulin heavy chain mRNA, partial cds.<br>Homo sapiens clone RIZMVH9 anti-cardiolipin immunoglobulin heavy chain mRNA, partial cds.<br>Homo sapiens clone 12 anti-hepatitis B surface antigen immunoglobulin heavy chain variable region mRNA, partial cds.<br>Homo sapiens clone 14 anti-hepatitis B surface antigen immunoglobulin heavy chain variable region mRNA, partial cds.<br>Homo sapiens clone 15 anti-hepatitis B surface antigen immunoglobulin heavy chain variable region mRNA, partial cds.<br>Homo sapiens BS immunoglobulin heavy chain variable region (IGVH) mRNA, partial cds.<br>Homo sapiens ED immunoglobulin heavy chain variable region (IGVH) mRNA, partial cds.<br>Homo sapiens sequence ga35a01 immunoglobulin heavy chain variable region mRNA, partial cds.<br>Homo sapiens sequence ga35a16 immunoglobulin heavy chain variable region mRNA, partial cds.<br>Homo sapiens sequence ga35a21 immunoglobulin heavy chain variable region mRNA, partial cds.<br>Homo sapiens sequence ga35a26 immunoglobulin heavy chain variable region mRNA, partial cds.<br>Homo sapiens sequence ga35a27 immunoglobulin heavy chain variable region mRNA, partial cds.<br>Homo sapiens sequence ga34a-3M6 immunoglobulin heavy chain variable region mRNA, partial cds.<br>Homo sapiens sequence ga36a19 immunoglobulin heavy chain variable region mRNA, partial cds.<br>Homo sapiens sequence ga36a28 immunoglobulin heavy chain variable region mRNA, partial cds.<br>Homo sapiens sequence rva42d09 immunoglobulin heavy chain variable region mRNA, partial cds.<br>Homo sapiens sequence rc43a19 immunoglobulin heavy chain variable region mRNA, partial cds.<br>Homo sapiens sequence rc43a34 immunoglobulin heavy chain variable region mRNA, partial cds.<br>Homo sapiens sequence rc43a43 immunoglobulin heavy chain variable region mRNA, partial cds.<br>Homo sapiens isolate RIZ GVH3 anticardiolipin immunoglobulin heavy chain mRNA, partial cds.<br>Homo sapiens isolate RIZ GVH4 anticardiolipin immunoglobulin heavy chain mRNA, partial cds.<br>Homo sapiens isolate RIZ GVH5 anticardiolipin immunoglobulin heavy chain mRNA, partial cds.<br>Homo sapiens clone 68-7a Ig heavy chain variable region, VH3 family mRNA, partial cds.<br>Homo sapiens clone 68-6a Ig heavy chain variable region, VH3 family mRNA, partial cds.<br>Homo sapiens clone 68-48m Ig heavy chain variable region, VH3 family mRNA, partial cds.<br>Homo sapiens clone 68-32a Ig heavy chain variable region, VH3 family mRNA, partial cds.<br>Homo sapiens clone 68-29m Ig heavy chain variable region, VH3 family mRNA, partial cds.<br>Homo sapiens clone 68-19m Ig heavy chain variable region, VH3 family mRNA, partial cds.<br>Homo sapiens clone 68-14m Ig heavy chain variable region, VH3 family mRNA, partial cds.<br>Homo sapiens clone 68-13m Ig heavy chain variable region, VH3 family mRNA, partial cds.<br>Homo sapiens clone 56-84m Ig heavy chain variable region, VH3 family mRNA, partial cds.<br>Homo sapiens clone 56-68m Ig heavy chain variable region, VH3 family mRNA, partial cds.<br>Homo sapiens clone 56-65a Ig heavy chain variable region, VH3 family mRNA, partial cds.<br>Homo sapiens clone 56-63m Ig heavy chain variable region, VH3 family mRNA, partial cds.<br>Homo sapiens clone 56-55m Ig heavy chain variable region, VH3 family mRNA, partial cds.<br>Homo sapiens clone 56-51m Ig heavy chain variable region, VH3 family mRNA, partial cds.

								AF471204	Homo sapiens clone 52-8a Ig heavy chain variable region, VH3 family mRNA, partial cds.
								AF471205	Homo sapiens clone 52-4m Ig heavy chain variable region, VH3 family mRNA, partial cds.
								AF471206	Homo sapiens clone 52-44m Ig heavy chain variable region, VH3 family mRNA, partial cds.
								AF471208	Homo sapiens clone 52-42m Ig heavy chain variable region, VH3 family mRNA, partial cds.
								AF471212	Homo sapiens clone 52-41m Ig heavy chain variable region, VH3 family mRNA, partial cds.
								AF471213	Homo sapiens clone 52-30m Ig heavy chain variable region, VH3 family mRNA, partial cds.
								AF471214	Homo sapiens clone 46-96m Ig heavy chain variable region, VH3 family mRNA, partial cds.
								AF471216	Homo sapiens clone 46-95a Ig heavy chain variable region, VH3 family mRNA, partial cds.
								AF471219	Homo sapiens clone 46-90m Ig heavy chain variable region, VH3 family mRNA, partial cds.
								AF471222	Homo sapiens clone 46-68m Ig heavy chain variable region, VH3 family mRNA, partial cds.
								AF471223	Homo sapiens clone 46-64m Ig heavy chain variable region, VH3 family mRNA, partial cds.
								AF471224	Homo sapiens clone 46-63a Ig heavy chain variable region, VH3 family mRNA, partial cds.
								AF471226	Homo sapiens clone 46-62m Ig heavy chain variable region, VH3 family mRNA, partial cds.
								AF471227	Homo sapiens clone 46-61a Ig heavy chain variable region, VH3 family mRNA, partial cds.
								AF471229	Homo sapiens clone 46-58a Ig heavy chain variable region, VH3 family mRNA, partial cds.
								AF471232	Homo sapiens clone 46-53m Ig heavy chain variable region, VH3 family mRNA, partial cds.
								AF471235	Homo sapiens clone 46-50a Ig heavy chain variable region, VH3 family mRNA, partial cds.
								AF471237	Homo sapiens clone 18-95a Ig heavy chain variable region, VH3 family mRNA, partial cds.
								AF471238	Homo sapiens clone 18-77a Ig heavy chain variable region, VH3 family mRNA, partial cds.
								AF471239	Homo sapiens clone 10-7m Ig heavy chain variable region, VH3 family mRNA, partial cds.
								AF471241	Homo sapiens clone 10-6m Ig heavy chain variable region, VH3 family mRNA, partial cds.
								AF471243	Homo sapiens clone 10-4m Ig heavy chain variable region, VH3 family mRNA, partial cds.
								AF471244	Homo sapiens clone 10-3m Ig heavy chain variable region, VH3 family mRNA, partial cds.
								AF471245	Homo sapiens clone 10-2m Ig heavy chain variable region, VH3 family mRNA, partial cds.
								AF471246	Homo sapiens clone 10-12m Ig heavy chain variable region, VH3 family mRNA, partial cds.
								AF471250	Homo sapiens clone 68-38a Ig heavy chain variable region, VH3 family mRNA, partial cds.
								AF471251	Homo sapiens clone 68-45a Ig heavy chain variable region, VH3 family mRNA, partial cds.
								AF471252	Homo sapiens clone 46-71a Ig heavy chain variable region, VH3 family mRNA, partial cds.
								AF471253	Homo sapiens clone 46-93a Ig heavy chain variable region, VH3 family mRNA, partial cds.
								AF471257	Homo sapiens clone 68-10m Ig heavy chain variable region, VH3 family mRNA, partial cds.
								AF471258	Homo sapiens clone 56-53a Ig heavy chain variable region, VH3 family mRNA, partial cds.
								AF471259	Homo sapiens clone 56-64a Ig heavy chain variable region, VH3 family mRNA, partial cds.
								AF471260	Homo sapiens clone 56-79a Ig heavy chain variable region, VH3 family mRNA, partial cds.
								AF471261	Homo sapiens clone 56-84a Ig heavy chain variable region, VH3 family mRNA, partial cds.
								AF471262	Homo sapiens clone 68-36a Ig heavy chain variable region, VH3 family mRNA, partial cds.
								AF471264	Homo sapiens clone 10-46m Ig heavy chain variable region, VH3 family mRNA, partial cds.
								AF471265	Homo sapiens clone 56-80m Ig heavy chain variable region, VH3 family mRNA, partial cds.
								AF471266	Homo sapiens clone 68-43m Ig heavy chain variable region, VH3 family mRNA, partial cds.
								AF471267	Homo sapiens clone 68-51m Ig heavy chain variable region, VH3 family mRNA, partial cds.
								AF471268	Homo sapiens clone 46-52m Ig heavy chain variable region, VH3 family mRNA, partial cds.
								AF471269	Homo sapiens clone 56-56a Ig heavy chain variable region, VH3 family mRNA, partial cds.
								AF471270	Homo sapiens clone 56-58a Ig heavy chain variable region, VH3 family mRNA, partial cds.
								AF471271	Homo sapiens clone 68-15a Ig heavy chain variable region, VH3 family mRNA, partial cds.
								AF471272	Homo sapiens clone 46-76a Ig heavy chain variable region, VH3 family mRNA, partial cds.
								AF471273	Homo sapiens clone 52-42a Ig heavy chain variable region, VH3 family mRNA, partial cds.
								AF471274	Homo sapiens clone 18-72a Ig heavy chain variable region, VH3 family mRNA, partial cds.
								AF471276	Homo sapiens clone 10-31m Ig heavy chain variable region, VH3 family mRNA, partial cds.
								AF471277	Homo sapiens clone 52-39m Ig heavy chain variable region, VH3 family mRNA, partial cds.
								AF471278	Homo sapiens clone 56-58m Ig heavy chain variable region, VH3 family mRNA, partial cds.
								AF471279	Homo sapiens clone 56-87m Ig heavy chain variable region, VH3 family mRNA, partial cds.
								AF471282	Homo sapiens clone 68-57m Ig heavy chain variable region, VH3 family mRNA, partial cds.
								AF471288	Homo sapiens clone 10-34m Ig heavy chain variable region, VH3 family mRNA, partial cds.
								AF471289	Homo sapiens clone 56-49m Ig heavy chain variable region, VH3 family mRNA, partial cds.
								AF471291	Homo sapiens clone 56-66m Ig heavy chain variable region, VH3 family mRNA, partial cds.

								AF471294	Homo sapiens clone 56-67m Ig heavy chain variable region, VH3 family mRNA, partial cds.
								AF471297	Homo sapiens clone 56-76m Ig heavy chain variable region, VH3 family mRNA, partial cds.
								AF471300	Homo sapiens clone 68-41m Ig heavy chain variable region, VH3 family mRNA, partial cds.
								AF471306	Homo sapiens clone 68-55m Ig heavy chain variable region, VH3 family mRNA, partial cds.
								AF471314	Homo sapiens clone 46-59m Ig heavy chain variable region, VH3 family mRNA, partial cds.
								AF471316	Homo sapiens clone 46-71m Ig heavy chain variable region, VH3 family mRNA, partial cds.
								AF471318	Homo sapiens clone 46-74m Ig heavy chain variable region, VH3 family mRNA, partial cds.
								AF471319	Homo sapiens clone 56-60a Ig heavy chain variable region, VH3 family mRNA, partial cds.
								AF471322	Homo sapiens clone 56-68a Ig heavy chain variable region, VH3 family mRNA, partial cds.
								AF471325	Homo sapiens clone 56-81a Ig heavy chain variable region, VH3 family mRNA, partial cds.
								AF471326	Homo sapiens clone 68-25a Ig heavy chain variable region, VH3 family mRNA, partial cds.
								AF471330	Homo sapiens clone 68-44a Ig heavy chain variable region, VH3 family mRNA, partial cds.
								AF471331	Homo sapiens clone 46-91m Ig heavy chain variable region, VH3 family mRNA, partial cds.
								AF471338	Homo sapiens clone 46-92m Ig heavy chain variable region, VH3 family mRNA, partial cds.
								AF471339	Homo sapiens clone 46-94m Ig heavy chain variable region, VH3 family mRNA, partial cds.
								AF471343	Homo sapiens clone 10-6a Ig heavy chain variable region, VH3 family mRNA, partial cds.
								AF471349	Homo sapiens clone 52-26a Ig heavy chain variable region, VH3 family mRNA, partial cds.
								AF471350	Homo sapiens clone 18-73a Ig heavy chain variable region, VH3 family mRNA, partial cds.
								AF471355	Homo sapiens clone 56-50a Ig heavy chain variable region, VH3 family mRNA, partial cds.
								AF471357	Homo sapiens clone 56-93a Ig heavy chain variable region, VH3 family mRNA, partial cds.
								AF471359	Homo sapiens clone 68-13a Ig heavy chain variable region, VH3 family mRNA, partial cds.
								AF471362	Homo sapiens clone 68-17a Ig heavy chain variable region, VH3 family mRNA, partial cds.
								AF471369	Homo sapiens clone 68-18a Ig heavy chain variable region, VH3 family mRNA, partial cds.
								AF471370	Homo sapiens clone 68-24a Ig heavy chain variable region, VH3 family mRNA, partial cds.
								AF471371	Homo sapiens clone 68-31a Ig heavy chain variable region, VH3 family mRNA, partial cds.
								AF471372	Homo sapiens clone 68-39a Ig heavy chain variable region, VH3 family mRNA, partial cds.
								AF471373	Homo sapiens clone 68-47a Ig heavy chain variable region, VH3 family mRNA, partial cds.
								AF471375	Homo sapiens clone 68-8a Ig heavy chain variable region, VH3 family mRNA, partial cds.
								AF471376	Homo sapiens clone 46-75a Ig heavy chain variable region, VH3 family mRNA, partial cds.
								AF471377	Homo sapiens clone 46-88a Ig heavy chain variable region, VH3 family mRNA, partial cds.
								AF471378	Homo sapiens clone 10-22m Ig heavy chain variable region, VH3 family mRNA, partial cds.
								AF471382	Homo sapiens clone 10-47m Ig heavy chain variable region, VH3 family mRNA, partial cds.
								AF471386	Homo sapiens clone 10-8m Ig heavy chain variable region, VH3 family mRNA, partial cds.
								AF471391	Homo sapiens clone 52-18m Ig heavy chain variable region, VH3 family mRNA, partial cds.
								AF471392	Homo sapiens clone 10-36a Ig heavy chain variable region, VH3 family mRNA, partial cds.
								AF471393	Homo sapiens clone 52-46m Ig heavy chain variable region, VH3 family mRNA, partial cds.
								AF471394	Homo sapiens clone 56-75m Ig heavy chain variable region, VH3 family mRNA, partial cds.
								AF471395	Homo sapiens clone 68-3m Ig heavy chain variable region, VH3 family mRNA, partial cds.
								AF471398	Homo sapiens clone 68-30m Ig heavy chain variable region, VH3 family mRNA, partial cds.
								AF471403	Homo sapiens clone 68-31m Ig heavy chain variable region, VH3 family mRNA, partial cds.
								AF471405	Homo sapiens clone 68-32m Ig heavy chain variable region, VH3 family mRNA, partial cds.
								AF471407	Homo sapiens clone 68-39m Ig heavy chain variable region, VH3 family mRNA, partial cds.
								AF471408	Homo sapiens clone 68-47m Ig heavy chain variable region, VH3 family mRNA, partial cds.
								AF471410	Homo sapiens clone 68-49m Ig heavy chain variable region, VH3 family mRNA, partial cds.
								AF471412	Homo sapiens clone 68-5m Ig heavy chain variable region, VH3 family mRNA, partial cds.
								AF471414	Homo sapiens clone 68-63m Ig heavy chain variable region, VH3 family mRNA, partial cds.
								AF471425	Homo sapiens clone 46-56m Ig heavy chain variable region, VH3 family mRNA, partial cds.
								AF471427	Homo sapiens clone 46-75m Ig heavy chain variable region, VH3 family mRNA, partial cds.
								AF471428	Homo sapiens clone 56-70a Ig heavy chain variable region, VH3 family mRNA, partial cds.
								AF471430	Homo sapiens clone 56-96a Ig heavy chain variable region, VH3 family mRNA, partial cds.
								AF471432	Homo sapiens clone 46-66a Ig heavy chain variable region, VH3 family mRNA, partial cds.
								AF471433	Homo sapiens clone 46-79a Ig heavy chain variable region, VH3 family mRNA, partial cds.
								AF471434	Homo sapiens clone 46-82a Ig heavy chain variable region, VH3 family mRNA, partial cds.
								AF471435	Homo sapiens clone 52-19m Ig heavy chain variable region, VH3 family mRNA, partial cds.

								AF471437	Homo sapiens clone 52-7m Ig heavy chain variable region, VH3 family mRNA, partial cds.
								AF471438	Homo sapiens clone 46-49m Ig heavy chain variable region, VH3 family mRNA, partial cds.
								AF471439	Homo sapiens clone 46-55m Ig heavy chain variable region, VH3 family mRNA, partial cds.
								AF471440	Homo sapiens clone 46-65m Ig heavy chain variable region, VH3 family mRNA, partial cds.
								AF471441	Homo sapiens clone 46-80m Ig heavy chain variable region, VH3 family mRNA, partial cds.
								AF471443	Homo sapiens clone 46-86m Ig heavy chain variable region, VH3 family mRNA, partial cds.
								AF471445	Homo sapiens clone 52-28a Ig heavy chain variable region, VH3 family mRNA, partial cds.
								AF471446	Homo sapiens clone 56-57m Ig heavy chain variable region, VH3 family mRNA, partial cds.
								AF471448	Homo sapiens clone 56-71a Ig heavy chain variable region, VH3 family mRNA, partial cds.
								AF471449	Homo sapiens clone 56-73a Ig heavy chain variable region, VH3 family mRNA, partial cds.
								AF471452	Homo sapiens clone 56-91a Ig heavy chain variable region, VH3 family mRNA, partial cds.
								AF471454	Homo sapiens clone 68-43a Ig heavy chain variable region, VH3 family mRNA, partial cds.
								AF471457	Homo sapiens clone 10-17m Ig heavy chain variable region, VH3 family mRNA, partial cds.
								AF471460	Homo sapiens clone 10-27m Ig heavy chain variable region, VH3 family mRNA, partial cds.
								AF471462	Homo sapiens clone 52-23m Ig heavy chain variable region, VH3 family mRNA, partial cds.
								AF471476	Homo sapiens clone 52-34m Ig heavy chain variable region, VH3 family mRNA, partial cds.
								AF471477	Homo sapiens clone 52-9m Ig heavy chain variable region, VH3 family mRNA, partial cds.
								AF471486	Homo sapiens clone 68-22m Ig heavy chain variable region, VH3 family mRNA, partial cds.
								AF471487	Homo sapiens clone 68-37m Ig heavy chain variable region, VH3 family mRNA, partial cds.
								AF471490	Homo sapiens clone 68-46m Ig heavy chain variable region, VH3 family mRNA, partial cds.
								AF471491	Homo sapiens clone 10-18a Ig heavy chain variable region, VH3 family mRNA, partial cds.
								AF471492	Homo sapiens clone 52-27a Ig heavy chain variable region, VH3 family mRNA, partial cds.
								AF471504	Homo sapiens clone 52-3a Ig heavy chain variable region, VH3 family mRNA, partial cds.
								AF471510	Homo sapiens clone 18-52a Ig heavy chain variable region, VH3 family mRNA, partial cds.
								AF471518	Homo sapiens clone 18-54a Ig heavy chain variable region, VH3 family mRNA, partial cds.
								AF471519	Homo sapiens clone 68-1a Ig heavy chain variable region, VH3 family mRNA, partial cds.
								AF471530	Homo sapiens clone 68-46a Ig heavy chain variable region, VH3 family mRNA, partial cds.
								AF484410	Homo sapiens clone 10-9m Ig heavy chain variable region, VH3 family mRNA, partial cds.
								AF484411	Homo sapiens clone 68-56m Ig heavy chain variable region, VH3 family mRNA, partial cds.
								AF485421	Homo sapiens clone 68-8m Ig heavy chain variable region, VH3 family mRNA, partial cds.
								AF485435	Homo sapiens clone 52-13m Ig heavy chain variable region, VH3 family mRNA, partial cds.
								AF485447	Homo sapiens clone 52-16m Ig heavy chain variable region, VH3 family mRNA, partial cds.
								AF485457	Homo sapiens clone 68-42m Ig heavy chain variable region, VH3 family mRNA, partial cds.
								AF485461	Homo sapiens clone 68-62m Ig heavy chain variable region, VH3 family mRNA, partial cds.
								AF485465	Homo sapiens clone 56-66a Ig heavy chain variable region, VH3 family mRNA, partial cds.
								AJ276097	Homo sapiens clone 68-29a Ig heavy chain variable region, VH3 family mRNA, partial cds.
								AJ512649	Homo sapiens clone 68-5a Ig heavy chain variable region, VH3 family mRNA, partial cds.
								AJ512650	Homo sapiens clone 52-36m Ig heavy chain variable region, VH3 family mRNA, partial cds.
								AJ617568	Homo sapiens clone 46-78a Ig heavy chain variable region, VH3 family mRNA, partial cds.
								AJ617570	Homo sapiens clone 56-54m Ig heavy chain variable region, VH3 family mRNA, partial cds.
								AJ617572	Homo sapiens clone 56-56m Ig heavy chain variable region, VH3 family mRNA, partial cds.
								AJ617574	Homo sapiens clone 68-52m Ig heavy chain variable region, VH3 family mRNA, partial cds.
								AJ627229	Homo sapiens isolate CLL-01 immunoglobulin heavy chain variable region mRNA, partial cds.
								AJ627230	Homo sapiens isolate CLL-07 immunoglobulin heavy chain variable region mRNA, partial cds.
								AJ627232	Homo sapiens clone 023.102H immunoglobulin heavy chain variable region mRNA, partial cds.
								AJ627233	Homo sapiens clone 018.6G5H immunoglobulin heavy chain variable region mRNA, partial cds.
								AJ627236	Homo sapiens clone 025.7F5H immunoglobulin heavy chain variable region mRNA, partial cds.
								AJ627237	Homo sapiens clone 008.1F9H immunoglobulin heavy chain variable region mRNA, partial cds.
								AJ627238	Homo sapiens clone 008.3C12H immunoglobulin heavy chain variable region mRNA, partial cds.
								AJ627240	Homo sapiens clone 008.4A3H immunoglobulin heavy chain variable region mRNA, partial cds.
								AJ627241	Homo sapiens partial mRNA for immunoglobulin heavy chain variable region (IGHV gene), clone LTMalpha-15 VH.
								AJ627243	Homo sapiens partial mRNA for immunoglobulin heavy chain variable region (IGHV gene), clone 10.
								AJ627244	Homo sapiens partial mRNA for immunoglobulin heavy chain variable region (IGHV gene), clone 43.
								AJ627245	Homo sapiens partial mRNA for immunoglobulin heavy chain variable region (IGHV1 gene), clone case 1 (MCL-MUT).

								AJ627246	Homo sapiens partial mRNA for immunoglobulin heavy chain variable region (IGHV1 gene), clone case 3 (MCL-MUT).
								AJ627249	Homo sapiens partial mRNA for immunoglobulin heavy chain variable region (IGHV1 gene), clone case 8 (MCL-UM).
								AJ627250	Homo sapiens partial mRNA for immunoglobulin heavy chain variable region (IGHV1 gene), clone case 10 (MCL-UM).
								AJ633622	Homo sapiens partial mRNA for IgD immunoglobulin heavy chain variable region (IGHV3-30 gene), clone T3.3.1.
								AJ633623	Homo sapiens partial mRNA for IgD immunoglobulin heavy chain variable region (IGHV3-30 gene), clone T3.3.2.
								AK027379	Homo sapiens partial mRNA for IgD immunoglobulin heavy chain variable region (IGHV3-30 gene), clone T3.3.5.
								AK056731	Homo sapiens partial mRNA for IgD immunoglobulin heavy chain variable region (IGHV3-23 gene), clone T3.3.6.
								AK057614	Homo sapiens partial mRNA for IgD immunoglobulin heavy chain variable region (IGHV3-07 gene), clone T3.3.10.
								AK057617	Homo sapiens partial mRNA for IgD immunoglobulin heavy chain variable region (IGHV3-07 gene), clone T3.3.11.
								AK057754	Homo sapiens partial mRNA for IgD immunoglobulin heavy chain variable region (IGHV3-15 gene), clone T3.3.12.
								AK057775	Homo sapiens partial mRNA for IgD immunoglobulin heavy chain variable region (IGHV3-23 gene), clone T3.3.14.
								AK057979	Homo sapiens partial mRNA for IgD immunoglobulin heavy chain variable region (IGHV3-73 gene), clone T3.3.15.
								AK058027	Homo sapiens partial mRNA for IgD immunoglobulin heavy chain variable region (IGHV3-30 gene), clone T3.3.20.
								AK074651	Homo sapiens partial mRNA for truncated IgD immunoglobulin heavy chain variable region (IGHV3-30 gene), clone T3.3.21.
								AK090461	Homo sapiens partial mRNA for IgD immunoglobulin heavy chain variable region (IGHV3-30 gene), clone T3.3.22.
								AK090464	Homo sapiens partial mRNA for IgD immunoglobulin heavy chain variable region (IGHV3-11 gene), clone T3.3.23.
								AK092384	Homo sapiens partial mRNA for IgD immunoglobulin heavy chain variable region (IGHV3-48 gene), clone T3.3.26.
								AK092785	Homo sapiens partial mRNA for IgD immunoglobulin heavy chain variable region (IGHV3-23 gene), clone T3.3.29.
								AK092803	Homo sapiens partial mRNA for immunoglobulin heavy chain variable region (IGHV gene), clone Hu19-VH.
								AK092847	Homo sapiens partial mRNA for immunoglobulin heavy chain variable region (IGHV gene), clone Hu22-VH.
								AK092976	Homo sapiens cDNA FLJ14473 fis, clone MAMMA1001080, highly similar to Homo sapiens SNC73 protein (SNC73) mRNA.
								AK093636	Homo sapiens cDNA FLJ32169 fis, clone PLACE6000523.
								AK093721	Homo sapiens cDNA FLJ33052 fis, clone TRACH1000074, highly similar to Ig delta chain.
								AK093806	Homo sapiens cDNA FLJ33055 fis, clone TRACH1000125, highly similar to Ig delta chain.
								AK093943	Homo sapiens cDNA FLJ25025 fis, clone CBL01928, highly similar to Ig gamma immunoglobulin heavy chain.
								AK095222	Homo sapiens cDNA FLJ25046 fis, clone CBL03624, highly similar to Ig gamma immunoglobulin heavy chain.
								AK096985	Homo sapiens cDNA FLJ25250 fis, clone STM03501, highly similar to IG ALPHA-2 CHAIN C REGION.
								AK097010	Homo sapiens cDNA FLJ25298 fis, clone STM07683, highly similar to Protein Tro alpha1 H,myeloma.
								AK097017	Homo sapiens cDNA FLJ90170 fis, clone MAMMA1000370, highly similar to Ig alpha-1 chain C region.
								AK097028	Homo sapiens mRNA for FLJ00382 protein.
								AK097071	Homo sapiens mRNA for FLJ00385 protein.
								AK097076	Homo sapiens cDNA FLJ35065 fis, clone PEBLM2000180, highly similar to IG ALPHA-1 CHAIN C REGION.
								AK097206	Homo sapiens cDNA FLJ35466 fis, clone SMINT2005623, moderately similar to Ig mu chain precursor, membrane-bound.
								AK097303	Homo sapiens cDNA FLJ35484 fis, clone SMINT2008329, highly similar to IG ALPHA-1 CHAIN C REGION.
								AK097305	Homo sapiens cDNA FLJ35528 fis, clone SPLEN2001881, highly similar to IG GAMMA-1 CHAIN C REGION.
								AK097306	Homo sapiens cDNA FLJ35657 fis, clone SPLEN2013910, highly similar to Human (hybridoma H210) anti-hepatitis A IgG.
								AK097307	Homo sapiens cDNA FLJ36317 fis, clone THYMU2005283, highly similar to Homo sapiens mRNA for immunoglobulin lambda
								AK097320	heavy chain.
								AK097329	Homo sapiens cDNA FLJ36402 fis, clone THYMU2009906, highly similar to IG ALPHA-1 CHAIN C REGION.
								AK097345	Homo sapiens cDNA FLJ36487 fis, clone THYMU2017844, highly similar to Homo sapiens mRNA for immunoglobulin lambda
								AK097348	heavy chain.
								AK097350	Homo sapiens cDNA FLJ36624 fis, clone TRACH2017086, highly similar to IG DELTA CHAIN C REGION.
								AK097354	Homo sapiens cDNA FLJ37903 fis, clone COLON1000135, highly similar to IG ALPHA-1 CHAIN C REGION.
								AK097355	Homo sapiens cDNA FLJ39666 fis, clone SMINT2007365, highly similar to IG ALPHA-1 CHAIN C REGION.
								AK097356	Homo sapiens cDNA FLJ39691 fis, clone SMINT2010672, highly similar to Homo sapiens mRNA for immunoglobulin lambda
								AK097357	heavy chain.
								AK097359	Homo sapiens cDNA FLJ39698 fis, clone SMINT2011273, highly similar to IG ALPHA-1 CHAIN C REGION.
								AK097360	Homo sapiens cDNA FLJ39709 fis, clone SMINT2012793, highly similar to IG ALPHA-1 CHAIN C REGION.
								AK097361	Homo sapiens cDNA FLJ39752 fis, clone SMINT2017974, highly similar to IG MU CHAIN C REGION.
								AK097362	Homo sapiens cDNA FLJ39757 fis, clone SMINT2019017, highly similar to IG ALPHA-1 CHAIN C REGION.
								AK097363	Homo sapiens cDNA FLJ39887 fis, clone SPLEN2016531, highly similar to Homo sapiens mRNA for immunoglobulin lambda
								AK097364	heavy chain.
								AK097365	Homo sapiens cDNA FLJ39984 fis, clone STOMA2000183, highly similar to IG ALPHA-1 CHAIN C REGION.
								AK097366	Homo sapiens cDNA FLJ39986 fis, clone STOMA2000539, highly similar to IG ALPHA-1 CHAIN C REGION.

								AK097367<br>AK097572<br>AK097859<br>AK097906<br>AK097950<br>AK097955<br>AK098108<br>AK098153<br>AK098516<br>AK123070<br>AK123546<br>AK123783<br>AK123800<br>AK123975<br>AK123981<br>AK125079<br>AK125238<br>AK125582<br>AK125632<br>AK125703<br>AK126132<br>AK126133<br>AK126279<br>AK126280<br>AK126281<br>AK126320<br>AK126352<br>AK126358<br>AK126543<br>AK127409<br>AK127415<br>AK127921<br>AK128301<br>AK128313<br>AK128394<br>AK128421<br>AK128476<br>AK128477<br>AK128533<br>AK128565<br>AK128568<br>AK128579<br>AK128580<br>AK128614<br>AK128625<br>AK128635<br>AK128649<br>AK128652<br>AK128658<br>AK128664<br>AK129508<br>AK129510<br>AK129512<br>AK129514	Homo sapiens cDNA FLJ39987 fis, clone STOMA2000567, highly similar to Homo sapiens mRNA for immunoglobulin lambda heavy chain.<br>Homo sapiens cDNA FLJ39988 fis, clone STOMA2000640, highly similar to Homo sapiens IGHG3 gene for immunoglobulin heavy chain gamma 3 constant region.<br>Homo sapiens cDNA FLJ40001 fis, clone STOMA2003477, highly similar to IG ALPHA-1 CHAIN C REGION.<br>Homo sapiens cDNA FLJ40010 fis, clone STOMA2005667, highly similar to IG ALPHA-1 CHAIN C REGION.<br>Homo sapiens cDNA FLJ40026 fis, clone STOMA2008361, highly similar to IG ALPHA-1 CHAIN C REGION.<br>Homo sapiens cDNA FLJ40029 fis, clone STOMA2008838, highly similar to IG ALPHA-1 CHAIN C REGION.<br>Homo sapiens cDNA FLJ40031 fis, clone STOMA2009253, highly similar to Homo sapiens mRNA for immunoglobulin lambda heavy chain.<br>Homo sapiens cDNA FLJ40035 fis, clone SYNOV2000173, highly similar to Human (hybridoma H210) anti-hepatitis A IgG variable region, constant region, complementarity-determining regions mRNA.<br>Homo sapiens cDNA FLJ40036 fis, clone SYNOV2000251, highly similar to Homo sapiens IGHG3 gene for immunoglobulin heavy chain gamma 3 constant region.<br>Homo sapiens cDNA FLJ40037 fis, clone SYNOV2000279, highly similar to Human (hybridoma H210) anti-hepatitis A IgG variable region, constant region, complementarity-determining regions mRNA.<br>Homo sapiens cDNA FLJ40038 fis, clone SYNOV2000297, highly similar to Human (hybridoma H210) anti-hepatitis A IgG variable region, constant region, complementarity-determining regions mRNA.<br>Homo sapiens cDNA FLJ40040 fis, clone SYNOV2000426, highly similar to Homo sapiens mRNA for immunoglobulin lambda heavy chain.<br>Homo sapiens cDNA FLJ40041 fis, clone SYNOV2000700, highly similar to Human (hybridoma H210) anti-hepatitis A IgG variable region, constant region, complementarity-determining regions mRNA.<br>Homo sapiens cDNA FLJ40042 fis, clone SYNOV2000824, highly similar to Human (hybridoma H210) anti-hepatitis A IgG variable region, constant region, complementarity-determining regions mRNA.<br>Homo sapiens cDNA FLJ40043 fis, clone SYNOV2000923, highly similar to IG ALPHA-1 CHAIN C REGION.<br>Homo sapiens cDNA FLJ40044 fis, clone SYNOV2001088, highly similar to Human (hybridoma H210) anti-hepatitis A IgG variable region, constant region, complementarity-determining regions mRNA.<br>Homo sapiens cDNA FLJ40045 fis, clone SYNOV2001111, highly similar to Human (hybridoma H210) anti-hepatitis A IgG variable region, constant region, complementarity-determining regions mRNA.<br>Homo sapiens cDNA FLJ40046 fis, clone SYNOV2001300, highly similar to Ig gamma =immunoglobulin heavy chain.<br>Homo sapiens cDNA FLJ40047 fis, clone SYNOV2001356, highly similar to Homo sapiens mRNA for immunoglobulin lambda heavy chain.<br>Homo sapiens cDNA FLJ40048 fis, clone SYNOV2001390, highly similar to Homo sapiens mRNA for immunoglobulin kappa heavy chain.<br>Homo sapiens cDNA FLJ40253 fis, clone TESTI2024419, highly similar to Homo sapiens IGHG3 gene for immunoglobulin heavy chain gamma 3 constant region.<br>Homo sapiens cDNA FLJ40540 fis, clone THYMU1000554, highly similar to Homo sapiens mRNA for immunoglobulin lambda heavy chain.<br>Homo sapiens cDNA FLJ40587 fis, clone THYMU2008928, highly similar to IG GAMMA-2 CHAIN C REGION.<br>Homo sapiens cDNA FLJ40631 fis, clone THYMU2014777, highly similar to Homo sapiens mRNA for immunoglobulin lambda heavy chain.<br>Homo sapiens cDNA FLJ40636 fis, clone THYMU2015943, highly similar to IG ALPHA-1 CHAIN C REGION.<br>Homo sapiens cDNA FLJ40789 fis, clone TRACH2006918, highly similar to Homo sapiens IGHG3 gene for immunoglobulin heavy chain gamma 3 constant region.<br>Homo sapiens cDNA FLJ40834 fis, clone TRACH2012823, highly similar to Homo sapiens IGHG3 gene for immunoglobulin heavy chain gamma 3 constant region.<br>Homo sapiens cDNA FLJ25650 fis, clone SYN01104, highly similar to Ig gamma =immunoglobulin heavy chain.<br>Homo sapiens cDNA FLJ41075 fis, clone ADIPS2000088, highly similar to Human (hybridoma H210) anti-hepatitis A IgG.<br>Homo sapiens cDNA FLJ41552 fis, clone COLON2004478, highly similar to Protein Tro alpha1 H,myeloma.<br>Homo sapiens cDNA FLJ41789 fis, clone NESOP2001433, highly similar to ALC1_HUMAN Ig alpha-1 chain C region.<br>Homo sapiens cDNA FLJ41806 fis, clone NOVAR2001108, highly similar to Human (hybridoma H210) anti-hepatitis A IgG variable region, constant region, complementarity-determining regions mRNA, complete cds.<br>Homo sapiens cDNA FLJ41981 fis, clone SMINT2011888, highly similar to Protein Tro alpha1 H,myeloma.<br>Homo sapiens cDNA FLJ41987 fis, clone SPLEN2015267, highly similar to Homo sapiens IGHG3 gene for immunoglobulin

								AK129517	heavy chain gamma 3 constant region.
								AK129592	Homo sapiens cDNA FLJ43089 fis, clone COLON2000568, highly similar to Ig alpha-2 chain C region.
								AK129620	Homo sapiens cDNA FLJ43248 fis, clone HEART2001680, highly similar to Ig alpha-1 chain C region.
								AK129622	Homo sapiens cDNA FLJ43594 fis, clone SMINT2010076, highly similar to Ig alpha-1 chain C region.
								AK129637	Homo sapiens cDNA FLJ43644 fis, clone SYNOV3000231, highly similar to Ig gamma-1 chain C region.
								AK129776	Homo sapiens cDNA FLJ43715 fis, clone TESOP2005485, highly similar to Ig delta chain C region.
								AK129777	Homo sapiens cDNA FLJ44144 fis, clone THYMU2023711, highly similar to Homo sapiens mRNA for immunoglobulin lambda
								AK129787	heavy chain.
								AK129809	Homo sapiens cDNA FLJ44145 fis, clone THYMU2027695, moderately similar to Ig gamma-1 chain C region.
								AK129812	Homo sapiens cDNA FLJ44293 fis, clone TRACH2022425, highly similar to Ig alpha-1 chain C region.
								AK130433	Homo sapiens cDNA FLJ44294 fis, clone TRACH2022553, highly similar to Human germline IgD-chain gene, C-region, second
								AK130434	domain of membrane terminus.
								AK130453	Homo sapiens cDNA FLJ44295 fis, clone TRACH2022649, highly similar to Ig gamma-1 chain C region.
								AK130465	Homo sapiens cDNA FLJ44344 fis, clone TRACH3005549, moderately similar to Ig heavy chain V region IR2 precursor.
								AK130468	Homo sapiens cDNA FLJ44381 fis, clone TRACH3035526, highly similar to Ig alpha-2 chain C region.
								AK130476	Homo sapiens cDNA FLJ44394 fis, clone TSTOM2000442, moderately similar to Ig gamma-1 chain C region.
								AK130501	Homo sapiens cDNA FLJ44579 fis, clone ADIPS2000069, highly similar to Ig alpha-1 chain C region.
								AK130529	Homo sapiens cDNA FLJ45501 fis, clone BRTHA2019726, highly similar to Ig gamma-1 chain C region.
								AK130539	Homo sapiens cDNA FLJ45507 fis, clone BRTHA2020721, highly similar to Ig gamma-1 chain C region.
								AK130577	Homo sapiens cDNA FLJ46028 fis, clone SPLEN2025012, highly similar to Ig alpha-1 chain C region.
								AK130585	Homo sapiens cDNA FLJ46441 fis, clone THYMU3016518, highly similar to Ig gamma-1 chain C region.
								AK130586	Homo sapiens cDNA FLJ46454 fis, clone THYMU3020221, highly similar to Ig gamma-1 chain C region.
								AK130589	Homo sapiens cDNA FLJ46537 fis, clone THYMU3037772, highly similar to Ig gamma-1 chain C region.
								AK130609	Homo sapiens cDNA FLJ46564 fis, clone THYMU3040746, highly similar to Ig gamma-2 chain C region.
								AK130611	Homo sapiens cDNA FLJ46621 fis, clone TLUNG2001445, highly similar to Ig alpha-1 chain C region.
								AK130614	Homo sapiens cDNA FLJ46622 fis, clone TLUNG2001600, highly similar to Ig gamma-4 chain C region.
								AK130642	Homo sapiens cDNA FLJ46691 fis, clone TRACH3012659, highly similar to Ig heavy chain V-III region VH26 precursor.
								AK130786	Homo sapiens cDNA FLJ46724 fis, clone TRACH3018907, highly similar to Ig alpha-1 chain C region.
								AK130796	Homo sapiens cDNA FLJ46727 fis, clone TRACH3019058, highly similar to Ig delta chain C region.
								AK130813	Homo sapiens cDNA FLJ46738 fis, clone TRACH3020930, highly similar to Ig gamma-1 chain C region.
								AK130817	Homo sapiens cDNA FLJ46739 fis, clone TRACH3021023, highly similar to Ig delta chain C region.
								AK130825	Homo sapiens cDNA FLJ46773 fis, clone TRACH3026299, highly similar to Ig delta chain C region.
								AK130844	Homo sapiens cDNA FLJ46784 fis, clone TRACH3028441, highly similar to Ig delta chain C region.
								AK131045	Homo sapiens cDNA FLJ46794 fis, clone TRACH3029670, highly similar to Ig delta chain C region.
								AK131065	Homo sapiens cDNA FLJ46808 fis, clone TRACH3034680, moderately similar to Ig delta chain C region.
								AK225676	Homo sapiens cDNA FLJ46811 fis, clone TRACH3036103, moderately similar to Ig heavy chain V-III region VH26 precursor.
								AM040520	Homo sapiens cDNA FLJ46817 fis, clone TRACH3037505, highly similar to Ig delta chain C region.
								AM040588	Homo sapiens cDNA FLJ46824 fis, clone TUTER2001433, highly similar to Ig alpha-1 chain C region.
								AM183975	Homo sapiens cDNA FLJ25997 fis, clone DMC06923, highly similar to Ig gamma-1 chain C region.
								AM183976	Homo sapiens cDNA FLJ25999 fis, clone DMC07355, highly similar to Ig gamma-1 chain C region.
								AM183977	Homo sapiens cDNA FLJ26001 fis, clone DMC07585, highly similar to Ig gamma-1 chain C region.
								AM183978	Homo sapiens cDNA FLJ26003 fis, clone DMC07928, highly similar to Ig gamma-1 chain C region.
								AM183979	Homo sapiens cDNA FLJ26006 fis, clone DMC08725, highly similar to Ig gamma-1 chain C region.
								AM183980	Homo sapiens cDNA FLJ26081 fis, clone RCT03819, highly similar to Ig alpha-1 chain C region.
								AM183981	Homo sapiens cDNA FLJ26109 fis, clone SPL05286, highly similar to Ig alpha-1 chain C region.
								AM183982	Homo sapiens cDNA FLJ26111 fis, clone SPL06037, highly similar to Ig gamma-1 chain C region.
								AM183983	Homo sapiens cDNA FLJ26126 fis, clone TMS02464, highly similar to Ig gamma-1 chain C region.
								AM183985	Homo sapiens cDNA FLJ26265 fis, clone DMC05516, highly similar to Ig gamma-1 chain C region.
								AM183986	Homo sapiens cDNA FLJ26266 fis, clone DMC05613.
								AM183987	Homo sapiens cDNA FLJ26276 fis, clone DMC06522, highly similar to Ig gamma-1 chain C region.
								AM183988	Homo sapiens cDNA FLJ26298 fis, clone DMC07404, highly similar to Ig gamma-1 chain C region.
								AM183989	Homo sapiens cDNA FLJ26301 fis, clone DMC07540.
								AM183990	Homo sapiens cDNA FLJ26923 fis, clone RCT04885, highly similar to Ig alpha-1 chain C region.
								AM183991	Homo sapiens cDNA FLJ26924 fis, clone RCT05096, highly similar to Ig gamma-1 chain C region.

								AM183993	Homo sapiens cDNA FLJ26943 fis, clone RCT07570, highly similar to Ig alpha-1 chain C region.
								AM183994	Homo sapiens cDNA FLJ26955 fis, clone SLV00309, highly similar to Ig alpha-1 chain C region.
								AM183995	Homo sapiens cDNA FLJ26958 fis, clone SLV00548, highly similar to Ig alpha-1 chain C region.
								AM183996	Homo sapiens cDNA FLJ26966 fis, clone SLV01429, highly similar to Ig alpha-1 chain C region.
								AM183997	Homo sapiens cDNA FLJ26991 fis, clone SLV03638, highly similar to Ig alpha-1 chain C region.
								AM233752	Homo sapiens cDNA FLJ27019 fis, clone SLV06008, highly similar to Ig alpha-1 chain C region.
								AM233753	Homo sapiens cDNA FLJ27029 fis, clone SLV07518, highly similar to Ig gamma-1 chain C region.
								AM233754	Homo sapiens cDNA FLJ27067 fis, clone SPL01356, highly similar to Ig gamma-1 chain C region.
								AM233755	Homo sapiens cDNA FLJ27075 fis, clone SPL02093, highly similar to Ig gamma-1 chain C region.
								AM233756	Homo sapiens cDNA FLJ27076 fis, clone SPL02374, highly similar to Ig gamma-1 chain C region.
								AM233757	Homo sapiens cDNA FLJ27079 fis, clone SPL02746, highly similar to Ig gamma-1 chain C region.
								AM233758	Homo sapiens cDNA FLJ27099 fis, clone SPL04664, highly similar to Ig gamma-2 chain C region.
								AM233759	Homo sapiens cDNA FLJ27101 fis, clone SPL04668, highly similar to Ig gamma-1 chain C region.
								AM233760	Homo sapiens cDNA FLJ27104 fis, clone SPL04981, highly similar to Ig gamma-2 chain C region.
								AM233761	Homo sapiens cDNA FLJ27132 fis, clone SPL08253, highly similar to Ig gamma-1 chain C region.
								AM233762	Homo sapiens cDNA FLJ27276 fis, clone TMS01151, highly similar to Ig gamma-1 chain C region.
								AM233763	Homo sapiens cDNA FLJ27286 fis, clone TMS01681, highly similar to Ig alpha-1 chain C region.
								AM233764	Homo sapiens cDNA FLJ27303 fis, clone TMS04901, highly similar to Ig gamma-1 chain C region.
								AM233767	Homo sapiens cDNA FLJ27307 fis, clone TMS05849, highly similar to Ig gamma-1 chain C region.
								AM233768	Homo sapiens cDNA FLJ27315 fis, clone TMS06851, highly similar to Ig epsilon chain C region.
								AM233769	Homo sapiens cDNA FLJ27334 fis, clone TMS09201, highly similar to Ig gamma-1 chain C region.
								AM233770	Homo sapiens cDNA FLJ29011 fis, clone DMC03382, highly similar to Ig gamma-2 chain C region.
								AM233772	Homo sapiens cDNA FLJ29031 fis, clone TMS03168, highly similar to Ig alpha-1 chain C region.
								AM233773	Homo sapiens mRNA for Ig alpha-1 chain C region variant, clone: STM08018.
								AM233776	Homo sapiens partial mRNA for immunoglobulin heavy chain variable region (IGHV gene).
								AM233782	Homo sapiens partial mRNA for immunoglobulin heavy chain variable region (IGHV gene).
								AM233783	Homo sapiens partial mRNA for immunoglobulin heavy chain (IGHV gene), cell line SP53.
								AM233786	Homo sapiens partial mRNA for immunoglobulin heavy chain (IGHV gene), cell line SP53, clone 2.
								AM233787	Homo sapiens partial mRNA for immunoglobulin heavy chain (IGHV gene), cell line SP53, clone 3.
								AM233788	Homo sapiens partial mRNA for immunoglobulin heavy chain (IGHV gene), cell line SP53, clone 4.
								AM233790	Homo sapiens partial mRNA for immunoglobulin heavy chain (IGHV gene), cell line Granta-519, clone 1.
								AM233791	Homo sapiens partial mRNA for immunoglobulin heavy chain (IGHV gene), cell line Granta-519, clone 2.
								AM233792	Homo sapiens partial mRNA for immunoglobulin heavy chain (IGHV gene), cell line JVM-2, clone 1.
								AM233793	Homo sapiens partial mRNA for immunoglobulin heavy chain (IGHV gene), cell line JVM-2, clone 2.
								AM233794	Homo sapiens partial mRNA for immunoglobulin heavy chain (IGHV gene), cell line JVM-2, clone 3.
								AM233795	Homo sapiens partial mRNA for immunoglobulin heavy chain (IGHV gene), cell line HBL-2, clone 1.
								AM233796	Homo sapiens partial mRNA for immunoglobulin heavy chain (IGHV gene), cell line HBL-2, clone 2.
								AM233797	Homo sapiens partial mRNA for immunoglobulin heavy chain (IGHV gene), cell line Jeko-1, clone 1.
								AM233798	Homo sapiens partial mRNA for immunoglobulin heavy chain (IGHV gene), isolate MCL001, clone 1.
								AM233799	Homo sapiens partial mRNA for immunoglobulin heavy chain (IGHV gene), isolate MCL001, clone 2.
								AM233800	Homo sapiens partial mRNA for immunoglobulin heavy chain (IGHV gene), isolate MCL002, clone 1.
								AM233801	Homo sapiens partial mRNA for immunoglobulin heavy chain (IGHV gene), isolate MCL003, clone 1.
								AM233810	Homo sapiens partial mRNA for immunoglobulin heavy chain (IGHV gene), isolate MCL239, clone 1.
								AM233811	Homo sapiens partial mRNA for immunoglobulin heavy chain (IGHV gene), isolate MCL478, clone 1.
								AM233812	Homo sapiens partial mRNA for immunoglobulin heavy chain (IGHV gene), isolate MCL601, clone 1.
								AM233813	Homo sapiens partial mRNA for immunoglobulin heavy chain (IGHV gene), isolate MCL898, clone 1.
								AM233815	Homo sapiens partial mRNA for immunoglobulin heavy chain (IGHV gene), isolate MCL942, clone 1.
								AM233817	Homo sapiens partial mRNA for immunoglobulin heavy chain variable region (IGHV4-31 gene), clone HL 10.
								AM233818	Homo sapiens partial mRNA for immunoglobulin heavy chain variable region (IGHV4-31 gene), clone HL 23.
								AM233819	Homo sapiens partial mRNA for immunoglobulin heavy chain variable region (IGHV4-31 gene), clone HN 1.
								AM233820	Homo sapiens partial mRNA for immunoglobulin heavy chain variable region (IGHV4-b gene), clone HN 6.
								AM233821	Homo sapiens partial mRNA for immunoglobulin heavy chain variable region (IGHV4-31 gene), clone HN 16.
								AM233825	Homo sapiens partial mRNA for immunoglobulin heavy chain variable region (IGHV4-28 gene), clone HN 17.
								AM233826	Homo sapiens partial mRNA for immunoglobulin heavy chain variable region (IGHV4-31 gene), clone HN 19.

								AM233827	Homo sapiens partial mRNA for immunoglobulin heavy chain variable region (IGHV4-31 gene), clone HN 22.
								AM233828	Homo sapiens partial mRNA for immunoglobulin heavy chain variable region (IGHV4-4 gene), clone HN 41.
								AM233829	Homo sapiens partial mRNA for immunoglobulin heavy chain variable region (IGHV4-31 gene), clone HN 52.
								AM233830	Homo sapiens partial mRNA for immunoglobulin heavy chain variable region (IGHV4-34 gene), clone HN 54.
								AM233831	Homo sapiens partial mRNA for immunoglobulin heavy chain variable region (IGHV4-31 gene), clone HN 93.
								AM233832	Homo sapiens partial mRNA for immunoglobulin heavy chain variable region (IGHV4-34 gene), clone HO 65.
								AM233833	Homo sapiens partial mRNA for immunoglobulin heavy chain variable region (IGHV4-4 gene), clone HO 71.
								AM233834	Homo sapiens partial mRNA for immunoglobulin heavy chain variable region (IGHV4-39 gene), clone HO 74.
								AM233835	Homo sapiens partial mRNA for immunoglobulin heavy chain variable region (IGHV4-4 gene), clone HO 75.
								AM233836	Homo sapiens partial mRNA for immunoglobulin heavy chain variable region (IGHV4-31 gene), clone HO 77.
								AM233837	Homo sapiens partial mRNA for immunoglobulin heavy chain variable region (IGHV4-31 gene), clone HO 82.
								AM233839	Homo sapiens partial mRNA for immunoglobulin heavy chain variable region (IGHV4-59 gene), clone HO 83.
								AM233840	Homo sapiens partial mRNA for immunoglobulin heavy chain variable region (IGHV4-28 gene), clone HO 88.
								AM259131	Homo sapiens partial mRNA for immunoglobulin heavy chain variable region (IGHV5-51 gene), clone HL 56.
								AM259132	Homo sapiens partial mRNA for immunoglobulin heavy chain variable region (IGHV5-51 gene), clone HL 61.
								AM259136	Homo sapiens partial mRNA for immunoglobulin heavy chain variable region (IGHV4-31 gene), clone HL 84.
								AM259137	Homo sapiens partial mRNA for immunoglobulin heavy chain variable region (IGHV4-31 gene), clone HL 87.
								AM259139	Homo sapiens partial mRNA for immunoglobulin heavy chain variable region (IGHV4-31 gene), clone HL 88.
								AM259140	Homo sapiens partial mRNA for immunoglobulin heavy chain variable region (IGHV4-31 gene), clone HL 101.
								AY003712	Homo sapiens partial mRNA for immunoglobulin heavy chain variable region (IGHV4-31 gene), clone HL 103.
								AY003713	Homo sapiens partial mRNA for immunoglobulin heavy chain variable region (IGHV4-34 gene), clone HL 111.
								AY003715	Homo sapiens partial mRNA for immunoglobulin heavy chain variable region (IGHV4-34 gene), clone HO 1.
								AY003728	Homo sapiens partial mRNA for immunoglobulin heavy chain variable region (IGHV4-31 gene), clone HO 6.
								AY003731	Homo sapiens partial mRNA for immunoglobulin heavy chain variable region (IGHV4-34 gene), clone HO 12.
								AY003735	Homo sapiens partial mRNA for immunoglobulin heavy chain variable region (IGHV4-31 gene), clone HO 14.
								AY003737	Homo sapiens partial mRNA for immunoglobulin heavy chain variable region (IGHV4-4 gene), clone HO 18.
								AY003738	Homo sapiens partial mRNA for immunoglobulin heavy chain variable region (IGHV4-4 gene), clone HO 22.
								AY003764	Homo sapiens partial mRNA for immunoglobulin heavy chain variable region (IGHV4-31 gene), clone HO 24.
								AY003765	Homo sapiens partial mRNA for immunoglobulin heavy chain variable region (IGHV4-34 gene), clone HO 28.
								AY003766	Homo sapiens partial mRNA for immunoglobulin heavy chain variable region (IGHV4-34 gene), clone HO 31.
								AY003767	Homo sapiens partial mRNA for immunoglobulin heavy chain variable region (IGHV4-34 gene), clone HP 7.
								AY003770	Homo sapiens partial mRNA for immunoglobulin heavy chain variable region (IGHV4-4 gene), clone HP 10.
								AY003772	Homo sapiens partial mRNA for immunoglobulin heavy chain variable region (IGHV4-31 gene), clone HP 49.
								AY003773	Homo sapiens partial mRNA for immunoglobulin heavy chain variable region (IGHV4-31 gene), clone HP 50.
								AY003774	Homo sapiens partial mRNA for immunoglobulin heavy chain variable region (IGHV4-34 gene), clone HP 66.
								AY003776	Homo sapiens partial mRNA for immunoglobulin heavy chain variable region (IGHV4-31 gene), clone HP 16.
								AY003777	Homo sapiens partial mRNA for immunoglobulin heavy chain variable region (IGHV4-4 gene), clone HP 19.
								AY003778	Homo sapiens partial mRNA for immunoglobulin heavy chain variable region (IGHV4-31 gene), clone HP 21.
								AY003780	Homo sapiens partial mRNA for immunoglobulin heavy chain variable region (IGHV4-31 gene), clone HP 26.
								AY003782	Homo sapiens partial mRNA for immunoglobulin heavy chain variable region (IGHV4-31 gene), clone HP 33.
								AY003784	Homo sapiens partial mRNA for immunoglobulin heavy chain variable region (IGHV4-4 gene), clone HP 41.
								AY003786	Homo sapiens partial mRNA for immunoglobulin heavy chain variable region (IGHV5-a gene), clone HP 72.
								AY003787	Homo sapiens partial mRNA for immunoglobulin heavy chain variable region (IGHV5-51 gene), clone HP 74.
								AY003789	Homo sapiens partial mRNA for immunoglobulin heavy chain variable region (IGHV5-51 gene), clone HP 75.
								AY003790	Homo sapiens partial mRNA for immunoglobulin heavy chain variable region (IGHV5-51 gene), clone HP 111.
								AY003796	Homo sapiens partial mRNA for immunoglobulin heavy chain variable region (IGHV5-51 gene), clone HP 114.
								AY003797	Homo sapiens partial mRNA for immunoglobulin heavy chain variable region (IGHV5-51 gene), clone HP 116.
								AY003798	Homo sapiens partial mRNA for immunoglobulin heavy chain variable region (IGHV5-51 gene), clone HP 119.
								AY003800	Homo sapiens partial mRNA for immunoglobulin heavy chain variable region (IGHV5-51 gene), clone HP 121.
								AY003801	Homo sapiens partial mRNA for immunoglobulin heavy chain variable region (IGHV5-51 gene), clone HP 80.
								AY003802	Homo sapiens partial mRNA for immunoglobulin heavy chain variable region (IGHV5-51 gene), clone HP 82.
								AY003803	Homo sapiens partial mRNA for immunoglobulin heavy chain variable region (IGHV5-51 gene), clone HP 84.
								AY003808	Homo sapiens partial mRNA for immunoglobulin heavy chain variable region (IGHV5-51 gene), clone HP 85.
								AY003809	Homo sapiens partial mRNA for immunoglobulin heavy chain variable region (IGHV5-51 gene), clone HO 115.

								AY003815	Homo sapiens partial mRNA for immunoglobulin heavy chain variable region (IGHV4-30-2 gene), clone HL 104.
								AY003818	Homo sapiens partial mRNA for immunoglobulin heavy chain variable region (GK2-HC gene), clone GK-2.
								AY003819	Homo sapiens partial mRNA for immunoglobulin heavy chain variable region (GK8-HC gene), clone GK-8.
								AY003822	Homo sapiens partial mRNA for immunoglobulin heavy chain variable region (T14-9-HC gene), clone T14-9.
								AY003823	Homo sapiens partial mRNA for immunoglobulin heavy chain variable region (TCH-3-HC gene), clone TCH-3.
								AY003825	Homo sapiens partial mRNA for immunoglobulin heavy chain variable region (TCH-6-HC gene), clone TCH-6.
								AY003826	Homo sapiens partial mRNA for immunoglobulin heavy chain variable region (TCH-8-HC gene), clone TCH-8.
								AY003827	Homo sapiens isolate sy-3A/12-F6 immunoglobulin alpha heavy chain variable region mRNA, partial cds.
								AY003828	Homo sapiens isolate sy-3A/11-D3 immunoglobulin alpha heavy chain variable region mRNA, partial cds.
								AY003829	Homo sapiens isolate sy-3A/12-H12 immunoglobulin alpha heavy chain variable region mRNA, partial cds.
								AY003831	Homo sapiens isolate sy-3A/11-A2 immunoglobulin alpha heavy chain variable region mRNA, partial cds.
								AY003837	Homo sapiens isolate sy-3A/11-C2 immunoglobulin alpha heavy chain variable region mRNA, partial cds.
								AY003839	Homo sapiens isolate sy-3A/12-G1 immunoglobulin alpha heavy chain variable region mRNA, partial cds.
								AY003842	Homo sapiens isolate sy-3A/12-G5' immunoglobulin alpha heavy chain variable region mRNA, partial cds.
								AY003843	Homo sapiens isolate sy-3A/12-C6 immunoglobulin alpha heavy chain variable region mRNA, partial cds.
								AY003844	Homo sapiens isolate sy-3A/17-F10 immunoglobulin alpha heavy chain variable region mRNA, partial cds.
								AY003845	Homo sapiens isolate sy-3A/17-H12 immunoglobulin alpha heavy chain variable region mRNA, partial cds.
								AY003847	Homo sapiens isolate sy-3M/7.2-A1 immunoglobulin mu heavy chain variable region mRNA, partial cds.
								AY003848	Homo sapiens isolate sy-1M/7-A10 immunoglobulin mu heavy chain variable region mRNA, partial cds.
								AY003849	Homo sapiens isolate sy-4M/7-F1 immunoglobulin mu heavy chain variable region mRNA, partial cds.
								AY003853	Homo sapiens isolate sy-4M/9-D5 immunoglobulin mu heavy chain variable region mRNA, partial cds.
								AY003854	Homo sapiens isolate sy-1M/11-B1 immunoglobulin mu heavy chain variable region mRNA, partial cds.
								AY003855	Homo sapiens isolate sy-3M/16-F1 immunoglobulin mu heavy chain variable region mRNA, partial cds.
								AY003860	Homo sapiens isolate sy-3M/5-A11 immunoglobulin mu heavy chain variable region mRNA, partial cds.
								AY003861	Homo sapiens isolate sy-3M/7-G9 immunoglobulin mu heavy chain variable region mRNA, partial cds.
								AY003864	Homo sapiens isolate sy-3M/16-E4 immunoglobulin mu heavy chain variable region mRNA, partial cds.
								AY003866	Homo sapiens isolate sy-3M/5-G10 immunoglobulin mu heavy chain variable region mRNA, partial cds.
								AY028777	Homo sapiens isolate sy-4M/9-F7 immunoglobulin mu heavy chain variable region mRNA, partial cds.
								AY033238	Homo sapiens isolate sy-3M/5-F10 immunoglobulin mu heavy chain variable region mRNA, partial cds.
								AY033239	Homo sapiens isolate sy-3M/16-B5 immunoglobulin mu heavy chain variable region mRNA, partial cds.
								AY033240	Homo sapiens isolate sy-4M/7-A6 immunoglobulin mu heavy chain variable region mRNA, partial cds.
								AY033241	Homo sapiens isolate sy-3M/5-H3 immunoglobulin mu heavy chain variable region mRNA, partial cds.
								AY033243	Homo sapiens isolate sy-3M/12-E12 immunoglobulin mu heavy chain variable region mRNA, partial cds.
								AY033245	Homo sapiens isolate sy-4M/7-E7 immunoglobulin mu heavy chain variable region mRNA, partial cds.
								AY033248	Homo sapiens isolate sy-1M/9-H1 immunoglobulin mu heavy chain variable region mRNA, partial cds.
								AY033251	Homo sapiens isolate sy-3M/12-E3 immunoglobulin mu heavy chain variable region mRNA, partial cds.
								AY033252	Homo sapiens isolate sy-3M/5-F1 immunoglobulin mu heavy chain variable region mRNA, partial cds.
								AY033255	Homo sapiens isolate sy-3M/9.1-C8 immunoglobulin mu heavy chain variable region mRNA, partial cds.
								AY033256	Homo sapiens isolate sy-1M/12-D1 immunoglobulin mu heavy chain variable region mRNA, partial cds.
								AY033257	Homo sapiens isolate sy-3M/16-G11 immunoglobulin mu heavy chain variable region mRNA, partial cds.
								AY033262	Homo sapiens isolate sy-1M/12-G9 nonfunctional immunoglobulin heavy chain mRNA, partial sequence.
								AY033263	Homo sapiens isolate sy-3M/12-E7 immunoglobulin mu heavy chain variable region mRNA, partial cds.
								AY033264	Homo sapiens isolate sy-3M/12-E9 immunoglobulin heavy chain variable region mRNA, partial cds.
								AY033267	Homo sapiens isolate sy-3M/9-H4 immunoglobulin heavy chain variable region mRNA, partial cds.
								AY033270	Homo sapiens isolate sy-2M/11-G3 immunoglobulin heavy chain variable region mRNA, partial cds.
								AY033272	Homo sapiens isolate sy-3M/9-B1 immunoglobulin heavy chain variable region mRNA, partial cds.
								AY033273	Homo sapiens isolate sy-3M/12-H11 immunoglobulin heavy chain variable region mRNA, partial cds.
								AY033277	Homo sapiens isolate sy-4M/7.3-F4 immunoglobulin heavy chain variable region mRNA, partial cds.
								AY033279	Homo sapiens isolate sy-4M/5-G7 immunoglobulin heavy chain variable region mRNA, partial cds.
								AY033285	Homo sapiens isolate sy-3M/9-B12 immunoglobulin heavy chain variable region mRNA, partial cds.
								AY033288	Homo sapiens isolate sy-3M/11-H3 immunoglobulin heavy chain variable region mRNA, partial cds.
								AY039025	Homo sapiens isolate sy-1M/16-H1 immunoglobulin heavy chain variable region mRNA, partial cds.
								AY050698	Homo sapiens isolate sy-3M/11-H4 immunoglobulin heavy chain variable region mRNA, partial cds.
								AY055477	Homo sapiens isolate sy-1M/16-G11 immunoglobulin heavy chain variable region mRNA, partial cds.

								AY055479	Homo sapiens isolate sy-3M/7-F2 immunoglobulin heavy chain variable region mRNA, partial cds.
								AY055482	Homo sapiens isolate sy-3M/11-H11 immunoglobulin heavy chain variable region mRNA, partial cds.
								AY055484	Homo sapiens isolate sy-3M/7-D6 immunoglobulin heavy chain variable region mRNA, partial cds.
								AY055485	Homo sapiens isolate sy-3M/9.2-F1 immunoglobulin heavy chain variable region mRNA, partial cds.
								AY058013	Homo sapiens isolate sy-3M/11-F12 immunoglobulin heavy chain variable region mRNA, partial cds.
								AY062296	Homo sapiens isolate sy-3M/7-F5 immunoglobulin heavy chain variable region mRNA, partial cds.
								AY062297	Homo sapiens isolate sy-1M/5-H11 immunoglobulin heavy chain variable region mRNA, partial cds.
								AY062298	Homo sapiens isolate sy-3M/9.2-H1 immunoglobulin heavy chain variable region mRNA, partial cds.
								AY062299	Homo sapiens isolate sy-3M/9.2-D1 immunoglobulin heavy chain variable region mRNA, partial cds.
								AY062300	Homo sapiens isolate sy-4M/12-H1 immunoglobulin heavy chain variable region mRNA, partial cds.
								AY062304	Homo sapiens isolate sy-4M/12-F2 nonfunctional immunoglobulin heavy chain variable region mRNA, partial sequence.
								AY062305	Homo sapiens isolate sy-4M/12-C6 nonfunctional immunoglobulin heavy chain variable region mRNA, partial sequence.
								AY062307	Homo sapiens isolate sy-4M/7-A6 immunoglobulin heavy chain variable region mRNA, partial cds.
								AY062314	Homo sapiens isolate sy-3M/4-H7 immunoglobulin mu heavy chain variable region mRNA, partial cds.
								AY062317	Homo sapiens isolate sy-3M/7-G11 immunoglobulin mu heavy chain variable region mRNA, partial cds.
								AY062319	Homo sapiens immunoglobulin heavy chain variable region mRNA, partial cds.
								AY062321	Homo sapiens clone case1-01 immunoglobulin heavy chain variable region mRNA, partial cds.
								AY062323	Homo sapiens clone case1-02 immunoglobulin heavy chain variable region mRNA, partial cds.
								AY062324	Homo sapiens clone case1-03 immunoglobulin heavy chain variable region mRNA, partial cds.
								AY062325	Homo sapiens clone case1-04 immunoglobulin heavy chain variable region mRNA, partial cds.
								AY062327	Homo sapiens clone case1-06 immunoglobulin heavy chain variable region mRNA, partial cds.
								AY099259	Homo sapiens clone case1-08 immunoglobulin heavy chain variable region mRNA, partial cds.
								AY099261	Homo sapiens clone case1-11 immunoglobulin heavy chain variable region mRNA, partial cds.
								AY148344	Homo sapiens clone case2-01 immunoglobulin heavy chain variable region mRNA, partial cds.
								AY155554	Homo sapiens clone case2-02 immunoglobulin heavy chain variable region mRNA, partial cds.
								AY155558	Homo sapiens clone case2-05 immunoglobulin heavy chain variable region mRNA, partial cds.
								AY155560	Homo sapiens clone case2-06 immunoglobulin heavy chain variable region mRNA, partial cds.
								AY165854	Homo sapiens clone case2-07 immunoglobulin heavy chain variable region mRNA, partial cds.
								AY165855	Homo sapiens clone case2-12 immunoglobulin heavy chain variable region mRNA, partial cds.
								AY165857	Homo sapiens clone case2-13 immunoglobulin heavy chain variable region mRNA, partial cds.
								AY172957	Homo sapiens clone case2-14 immunoglobulin heavy chain variable region mRNA, partial cds.
								AY172958	Homo sapiens clone case2-17 immunoglobulin heavy chain variable region mRNA, partial cds.
								AY172959	Homo sapiens clone case2-20 immunoglobulin heavy chain variable region mRNA, partial cds.
								AY179845	Homo sapiens clone case3-02 immunoglobulin heavy chain variable region mRNA, partial cds.
								AY180387	Homo sapiens clone case3-03 immunoglobulin heavy chain variable region mRNA, partial cds.
								AY190818	Homo sapiens clone case3-07 immunoglobulin heavy chain variable region mRNA, partial cds.
								AY190820	Homo sapiens clone case3-09 immunoglobulin heavy chain variable region mRNA, partial cds.
								AY190822	Homo sapiens clone case3-15 immunoglobulin heavy chain variable region mRNA, partial cds.
								AY190824	Homo sapiens clone case3-18 immunoglobulin heavy chain variable region mRNA, partial cds.
								AY190826	Homo sapiens putative matrix cell adhesion molecule-3 mRNA, complete cds.
								AY193806	Homo sapiens clone WR16 immunoglobulin heavy chain variable region mRNA, partial cds.
								AY193810	Homo sapiens isolate CLL007 immunoglobulin heavy chain variable region mRNA, partial cds.
								AY193812	Homo sapiens isolate CLL165 immunoglobulin heavy chain variable region mRNA, partial cds.
								AY193816	Homo sapiens isolate CLL178 immunoglobulin heavy chain variable region mRNA, partial cds.
								AY204755	Homo sapiens isolate CLL249 immunoglobulin heavy chain variable region mRNA, partial cds.
								AY204761	Homo sapiens isolate CLL258 immunoglobulin heavy chain variable region mRNA, partial cds.
								AY206987	Homo sapiens isolate D20RT1 7F immunoglobulin heavy chain variable region (VH4-34) mRNA, partial cds.
								AY240160	Homo sapiens clone 118/707 immunoglobulin heavy chain variable region mRNA, partial cds.
								AY268370	Homo sapiens clone 105/707 immunoglobulin heavy chain variable region mRNA, partial cds.
								AY268372	Homo sapiens clone 114/707 immunoglobulin heavy chain variable region mRNA, partial cds.
								AY268373	Homo sapiens clone K93/707 immunoglobulin heavy chain variable region mRNA, partial cds.
								AY270188	Homo sapiens clone K116/707 immunoglobulin heavy chain variable region mRNA, partial cds.
								AY270189	Homo sapiens clone 7/707 immunoglobulin heavy chain variable region mRNA, partial cds.
								AY270190	Homo sapiens clone 3/707 immunoglobulin heavy chain variable region mRNA, partial cds.

								AY270191	Homo sapiens clone 32/707 immunoglobulin heavy chain variable region mRNA, partial cds.
								AY281326	Homo sapiens clone 10/707 immunoglobulin heavy chain variable region mRNA, partial cds.
								AY312398	Homo sapiens clone 48/707 immunoglobulin heavy chain variable region mRNA, partial cds.
								AY331056	Homo sapiens clone K69/707 immunoglobulin heavy chain variable region mRNA, partial cds.
								AY345917	Homo sapiens clone K41/707 immunoglobulin heavy chain variable region mRNA, partial cds.
								AY345918	Homo sapiens clone 77/707 immunoglobulin heavy chain variable region mRNA, partial cds.
								AY345920	Homo sapiens clone K81/707 immunoglobulin heavy chain variable region mRNA, partial cds.
								AY345921	Homo sapiens clone 11/707 immunoglobulin heavy chain variable region mRNA, partial cds.
								AY345922	Homo sapiens clone 59/707 immunoglobulin heavy chain variable region mRNA, partial cds.
								AY345923	Homo sapiens isolate ELZ510 immunoglobulin heavy chain variable region mRNA, partial cds.
								AY345924	Homo sapiens isolate KZ51 immunoglobulin heavy chain variable region mRNA, partial cds.
								AY376074	Homo sapiens anti-oxidized LDL immunoglobulin heavy chain variable region mRNA, partial cds.
								AY376077	Homo sapiens clone X6 immunoglobulin heavy chain mRNA, partial cds.
								AY376080	Homo sapiens clone X10 immunoglobulin heavy chain mRNA, partial cds.
								AY376081	Homo sapiens clone X12 immunoglobulin heavy chain mRNA, partial cds.
								AY376082	Homo sapiens isolate Black93 immunoglobulin heavy chain variable region (IGVH) mRNA, partial cds.
								AY376083	Homo sapiens isolate BW-1 immunoglobulin heavy chain variable region (IGVH) mRNA, partial cds.
								AY376084	Homo sapiens isolate Rice94 immunoglobulin heavy chain variable region (IGVH) mRNA, partial cds.
								AY376085	Homo sapiens anti-rabies SO57 immunoglobulin heavy chain mRNA, complete cds.
								AY376090	Homo sapiens anti-rabies SOJB immunoglobulin heavy chain mRNA, complete cds.
								AY376101	Homo sapiens anti-rabies SOJA immunoglobulin heavy chain mRNA, complete cds.
								AY376111	Homo sapiens clone cm-u immunoglobulin heavy chain variable region (IGHV1-69) mRNA, partial cds.
								AY376120	Homo sapiens immunoglobulin heavy chain variable and constant region mRNA, partial cds.
								AY392846	Homo sapiens clone BPG48 immunoglobulin heavy chain VDJ region (IGH@) mRNA, partial cds.
								AY392847	Homo sapiens clone BPG49 immunoglobulin heavy chain VDJ region (IGH@) mRNA, partial cds.
								AY392849	Homo sapiens clone BPG79 immunoglobulin heavy chain VDJ region (IGH@) mRNA, partial cds.
								AY392850	Homo sapiens clone BPG169 immunoglobulin heavy chain VDJ region (IGH@) mRNA, partial cds.
								AY392852	Homo sapiens clone BPG256 immunoglobulin heavy chain VDJ region (IGH@) mRNA, partial cds.
								AY392854	Homo sapiens anti-HPA-5b immunoglobulin heavy chain variable region mRNA, partial cds.
								AY392856	Homo sapiens anti-HPA-5b immunoglobulin heavy chain variable region mRNA, partial cds.
								AY392863	Homo sapiens anti-HPA-5b immunoglobulin heavy chain variable region mRNA, partial cds.
								AY392865	Homo sapiens anti-HPA-5b immunoglobulin heavy chain variable region mRNA, partial cds.
								AY392866	Homo sapiens immunoglobulin heavy chain VDJ region mRNA, partial cds.
								AY392867	Homo sapiens immunoglobulin heavy chain VDJ region mRNA, partial cds.
								AY392869	Homo sapiens clone P2-114 anti-oxidized LDL immunoglobulin heavy chain Fab mRNA, partial cds.
								AY392871	Homo sapiens clone HA6 anti-HAV capsid immunoglobulin G heavy chain variable region mRNA, partial cds.
								AY392872	Homo sapiens clone CLL185 immunoglobulin heavy chain variable region mRNA, partial cds.
								AY392875	Homo sapiens clone CLL114 immunoglobulin heavy chain variable region mRNA, partial cds.
								AY392876	Homo sapiens clone CLL202 immunoglobulin heavy chain variable region mRNA, partial cds.
								AY392881	Homo sapiens immunoglobulin mu heavy chain mRNA, partial cds.
								AY392883	Homo sapiens immunoglobulin mu heavy chain mRNA, partial cds.
								AY392887	Homo sapiens immunoglobulin gamma heavy chain mRNA, partial cds.
								AY392891	Homo sapiens immunoglobulin gamma heavy chain mRNA, partial cds.
								AY392893	Homo sapiens isolate M6 immunoglobulin heavy chain variable region mRNA, partial cds.
								AY392896	Homo sapiens clone I54 immunoglobulin heavy chain variable region mRNA, partial cds.
								AY392897	Homo sapiens clone CB1B8 immunoglobulin heavy chain variable region (VH4) mRNA, partial cds.
								AY392898	Homo sapiens isolate case E70 immunoglobulin heavy chain variable region mRNA, partial cds.
								AY392900	Homo sapiens isolate case G13 immunoglobulin heavy chain variable region mRNA, partial cds.
								AY392905	Homo sapiens isolate case 216 immunoglobulin heavy chain variable region mRNA, partial cds.
								AY392907	Homo sapiens isolate case 266 immunoglobulin heavy chain variable region mRNA, partial cds.
								AY392908	Homo sapiens isolate case 283 immunoglobulin heavy chain variable region mRNA, partial cds.
								AY392909	Homo sapiens isolate case 330 immunoglobulin heavy chain variable region mRNA, partial cds.
								AY392910	Homo sapiens isolate case 343 immunoglobulin heavy chain variable region mRNA, partial cds.
								AY392912	Homo sapiens isolate VH-CE-A2 immunoglobulin E heavy chain variable region mRNA, partial cds.

								AY392914	Homo sapiens isolate VH-CE-A40 immunoglobulin E heavy chain variable region mRNA, partial cds.
								AY392915	Homo sapiens isolate VH-CE-A49 immunoglobulin E heavy chain variable region mRNA, partial cds.
								AY392917	Homo sapiens isolate VH-CE-A53 immunoglobulin E heavy chain variable region mRNA, partial cds.
								AY392919	Homo sapiens isolate VH-CE-A54 immunoglobulin E heavy chain variable region mRNA, partial cds.
								AY392920	Homo sapiens isolate VH-CE-A57 immunoglobulin E heavy chain variable region mRNA, partial cds.
								AY392921	Homo sapiens isolate VH-CE-A59 immunoglobulin E heavy chain variable region mRNA, partial cds.
								AY392922	Homo sapiens isolate VH-CE-A60 immunoglobulin E heavy chain variable region mRNA, partial cds.
								AY392925	Homo sapiens isolate VH-CE-A7 immunoglobulin E heavy chain variable region mRNA, partial cds.
								AY392926	Homo sapiens isolate VH-CE-B33 immunoglobulin E heavy chain variable region mRNA, partial cds.
								AY392928	Homo sapiens isolate VH-CE-C17 immunoglobulin E heavy chain variable region mRNA, partial cds.
								AY392930	Homo sapiens isolate VH-CE-C7 immunoglobulin E heavy chain variable region mRNA, partial cds.
								AY392931	Homo sapiens clone RA702-A1-14.fa immunoglobulin heavy chain mRNA, partial cds.
								AY392934	Homo sapiens clone RA702-A1-15.fa immunoglobulin heavy chain mRNA, partial cds.
								AY392937	Homo sapiens clone RA702-A1-17/18.fa immunoglobulin heavy chain mRNA, partial cds.
								AY392939	Homo sapiens clone RA702-A1-19.fa immunoglobulin heavy chain mRNA, partial cds.
								AY392940	Homo sapiens clone RA702-A1-20.fa immunoglobulin heavy chain mRNA, partial cds.
								AY392942	Homo sapiens clone RA702-A1-23.fa immunoglobulin heavy chain mRNA, partial cds.
								AY392944	Homo sapiens clone RA702-A1-26.fa immunoglobulin heavy chain mRNA, partial cds.
								AY392946	Homo sapiens clone RA702-A1-32.fa immunoglobulin heavy chain mRNA, partial cds.
								AY392947	Homo sapiens clone RA702-A1-34.fa immunoglobulin heavy chain mRNA, partial cds.
								AY392948	Homo sapiens clone RA702-A1-35.fa immunoglobulin heavy chain mRNA, partial cds.
								AY392951	Homo sapiens clone RA702-A1-36.fa immunoglobulin heavy chain mRNA, partial cds.
								AY392956	Homo sapiens clone RA702-A1-38.fa immunoglobulin heavy chain mRNA, partial cds.
								AY392964	Homo sapiens clone RA702-A1-4.fa immunoglobulin heavy chain mRNA, partial cds.
								AY392993	Homo sapiens clone RA702-A1-40.fa immunoglobulin heavy chain mRNA, partial cds.
								AY392995	Homo sapiens clone RA702-A1-43.fa immunoglobulin heavy chain mRNA, partial cds.
								AY392996	Homo sapiens clone RA702-A1-44.fa immunoglobulin heavy chain mRNA, partial cds.
								AY392997	Homo sapiens clone RA702-A1-8.fa immunoglobulin heavy chain mRNA, partial cds.
								AY393000	Homo sapiens clone RA702-A2-1.fa immunoglobulin heavy chain mRNA, partial cds.
								AY393001	Homo sapiens clone RA702-A2-15.fa immunoglobulin heavy chain mRNA, partial cds.
								AY393002	Homo sapiens clone RA702-A2-20/26.fa immunoglobulin heavy chain mRNA, partial cds.
								AY393003	Homo sapiens clone RA702-A2-21.fa immunoglobulin heavy chain mRNA, partial cds.
								AY393007	Homo sapiens clone RA702-A2-25/30.fa immunoglobulin heavy chain mRNA, partial cds.
								AY393008	Homo sapiens clone RA702-A2-27.fa immunoglobulin heavy chain mRNA, partial cds.
								AY393009	Homo sapiens clone RA702-A2-28.fa immunoglobulin heavy chain mRNA, partial cds.
								AY393010	Homo sapiens clone RA702-A2-3.fa immunoglobulin heavy chain mRNA, partial cds.
								AY393011	Homo sapiens clone RA702-A2-8.fa immunoglobulin heavy chain mRNA, partial cds.
								AY393012	Homo sapiens clone RA702-A3-1/23.fa immunoglobulin heavy chain mRNA, partial cds.
								AY393013	Homo sapiens clone RA702-A3-10.fa immunoglobulin heavy chain mRNA, partial cds.
								AY393014	Homo sapiens clone RA702-A3-11.fa immunoglobulin heavy chain mRNA, partial cds.
								AY393018	Homo sapiens clone RA702-A3-13.fa immunoglobulin heavy chain mRNA, partial cds.
								AY393020	Homo sapiens clone RA702-A3-15.fa immunoglobulin heavy chain mRNA, partial cds.
								AY393021	Homo sapiens clone RA702-A3-17.fa immunoglobulin heavy chain mRNA, partial cds.
								AY393023	Homo sapiens clone RA702-A3-18.fa immunoglobulin heavy chain mRNA, partial cds.
								AY393024	Homo sapiens clone RA702-A3-2.fa immunoglobulin heavy chain mRNA, partial cds.
								AY393025	Homo sapiens clone RA702-A3-21.fa immunoglobulin heavy chain mRNA, partial cds.
								AY393026	Homo sapiens clone RA702-A3-22.fa immunoglobulin heavy chain mRNA, partial cds.
								AY393027	Homo sapiens clone RA702-A3-25.fa immunoglobulin heavy chain mRNA, partial cds.
								AY393028	Homo sapiens clone RA702-A3-26.fa immunoglobulin heavy chain mRNA, partial cds.
								AY393029	Homo sapiens clone RA702-A3-3.fa immunoglobulin heavy chain mRNA, partial cds.
								AY393031	Homo sapiens clone RA702-A3-30.fa immunoglobulin heavy chain mRNA, partial cds.
								AY393032	Homo sapiens clone RA702-A3-5.fa immunoglobulin heavy chain mRNA, partial cds.
								AY393033	Homo sapiens clone RA702-A3-7.fa immunoglobulin heavy chain mRNA, partial cds.
								AY393034	Homo sapiens clone RA702-A3-8.fa immunoglobulin heavy chain mRNA, partial cds.

								AY393036	Homo sapiens clone RA702-A4-10.fa immunoglobulin heavy chain mRNA, partial cds.
								AY393037	Homo sapiens clone RA702-A4-13.fa immunoglobulin heavy chain mRNA, partial cds.
								AY393038	Homo sapiens clone RA702-A4-15.fa immunoglobulin heavy chain mRNA, partial cds.
								AY393039	Homo sapiens clone RA702-A4-16.fa immunoglobulin heavy chain mRNA, partial cds.
								AY393040	Homo sapiens clone RA702-A4-19.fa immunoglobulin heavy chain mRNA, partial cds.
								AY393041	Homo sapiens clone RA702-A4-21.fa immunoglobulin heavy chain mRNA, partial cds.
								AY393042	Homo sapiens clone RA702-A4-23.fa immunoglobulin heavy chain mRNA, partial cds.
								AY393043	Homo sapiens clone RA702-A4-24.fa immunoglobulin heavy chain mRNA, partial cds.
								AY393045	Homo sapiens clone RA702-A4-25.fa immunoglobulin heavy chain mRNA, partial cds.
								AY393046	Homo sapiens clone RA702-A4-3.fa immunoglobulin heavy chain mRNA, partial cds.
								AY393047	Homo sapiens clone RA702-A4-9.fa immunoglobulin heavy chain mRNA, partial cds.
								AY393048	Homo sapiens clone RA702-A5-16.fa immunoglobulin heavy chain mRNA, partial cds.
								AY393049	Homo sapiens clone RA702-A6-27.fa immunoglobulin heavy chain mRNA, partial cds.
								AY393050	Homo sapiens clone RA702-A6-4.fa immunoglobulin heavy chain mRNA, partial cds.
								AY393051	Homo sapiens clone RA702-A6-6.fa immunoglobulin heavy chain mRNA, partial cds.
								AY393052	Homo sapiens clone RA702-A6-7.fa immunoglobulin heavy chain mRNA, partial cds.
								AY393053	Homo sapiens clone RA702-G1-11.fa immunoglobulin heavy chain mRNA, partial cds.
								AY393055	Homo sapiens clone RA702-G1-12.fa nonfunctional immunoglobulin heavy chain mRNA, partial sequence.
								AY393056	Homo sapiens clone RA702-G1-13.fa immunoglobulin heavy chain mRNA, partial cds.
								AY393057	Homo sapiens clone RA702-G1-14.fa immunoglobulin heavy chain mRNA, partial cds.
								AY393060	Homo sapiens clone RA702-G1-19.fa immunoglobulin heavy chain mRNA, partial cds.
								AY393061	Homo sapiens clone RA702-G1-20.fa immunoglobulin heavy chain mRNA, partial cds.
								AY393063	Homo sapiens clone RA702-G1-21.fa immunoglobulin heavy chain mRNA, partial cds.
								AY393064	Homo sapiens clone RA702-G1-26.fa immunoglobulin heavy chain mRNA, partial cds.
								AY393065	Homo sapiens clone RA702-G1-27.fa immunoglobulin heavy chain mRNA, partial cds.
								AY393066	Homo sapiens clone RA702-G1-28.fa immunoglobulin heavy chain mRNA, partial cds.
								AY393068	Homo sapiens clone RA702-G1-29.fa immunoglobulin heavy chain mRNA, partial cds.
								AY393070	Homo sapiens clone RA702-G1-30.fa immunoglobulin heavy chain mRNA, partial cds.
								AY393073	Homo sapiens clone RA702-G1-34.fa immunoglobulin heavy chain mRNA, partial cds.
								AY393075	Homo sapiens clone RA702-G1-5.fa immunoglobulin heavy chain mRNA, partial cds.
								AY393076	Homo sapiens clone RA702-G1-6.fa immunoglobulin heavy chain mRNA, partial cds.
								AY393077	Homo sapiens clone RA702-G2-1.fa immunoglobulin heavy chain mRNA, partial cds.
								AY393079	Homo sapiens clone RA702-G2-10.fa immunoglobulin heavy chain mRNA, partial cds.
								AY393081	Homo sapiens clone RA702-G2-11.fa immunoglobulin heavy chain mRNA, partial cds.
								AY393082	Homo sapiens clone RA702-G2-12.fa immunoglobulin heavy chain mRNA, partial cds.
								AY393085	Homo sapiens clone RA702-G2-13.fa immunoglobulin heavy chain mRNA, partial cds.
								AY393087	Homo sapiens clone RA702-G2-14.fa immunoglobulin heavy chain mRNA, partial cds.
								AY393088	Homo sapiens clone RA702-G2-15.fa immunoglobulin heavy chain mRNA, partial cds.
								AY393090	Homo sapiens clone RA702-G2-17.fa immunoglobulin heavy chain mRNA, partial cds.
								AY393091	Homo sapiens clone RA702-G2-18.fa immunoglobulin heavy chain mRNA, partial cds.
								AY393092	Homo sapiens clone RA702-G2-19.fa immunoglobulin heavy chain mRNA, partial cds.
								AY393094	Homo sapiens clone RA702-G2-2.fa immunoglobulin heavy chain mRNA, partial cds.
								AY393095	Homo sapiens clone RA702-G2-21.fa immunoglobulin heavy chain mRNA, partial cds.
								AY393096	Homo sapiens clone RA702-G2-22.fa immunoglobulin heavy chain mRNA, partial cds.
								AY393097	Homo sapiens clone RA702-G2-23.fa immunoglobulin heavy chain mRNA, partial cds.
								AY393098	Homo sapiens clone RA702-G2-24.fa immunoglobulin heavy chain mRNA, partial cds.
								AY393101	Homo sapiens clone RA702-G2-25.fa immunoglobulin heavy chain mRNA, partial cds.
								AY393102	Homo sapiens clone RA702-G2-26.fa immunoglobulin heavy chain mRNA, partial cds.
								AY393103	Homo sapiens clone RA702-G2-27.fa immunoglobulin heavy chain mRNA, partial cds.
								AY393105	Homo sapiens clone RA702-G2-28.fa nonfunctional immunoglobulin heavy chain mRNA, partial sequence.
								AY393106	Homo sapiens clone RA702-G2-3.fa immunoglobulin heavy chain mRNA, partial cds.
								AY393107	Homo sapiens clone RA702-G2-30.fa immunoglobulin heavy chain mRNA, partial cds.
								AY393108	Homo sapiens clone RA702-G2-4.fa immunoglobulin heavy chain mRNA, partial cds.
								AY393110	Homo sapiens clone RA702-G2-5.fa immunoglobulin heavy chain mRNA, partial cds.

								AY393111<br>AY393112<br>AY393113<br>AY393114<br>AY393115<br>AY393116<br>AY393117<br>AY393118<br>AY393120<br>AY393121<br>AY393122<br>AY393123<br>AY393124<br>AY393126<br>AY393127<br>AY393128<br>AY393129<br>AY393130<br>AY393131<br>AY393133<br>AY393134<br>AY393136<br>AY393137<br>AY393138<br>AY393139<br>AY393140<br>AY393141<br>AY393142<br>AY393143<br>AY393144<br>AY393145<br>AY393146<br>AY393147<br>AY393148<br>AY393149<br>AY393150<br>AY393151<br>AY393153<br>AY393154<br>AY393155<br>AY393156<br>AY393157<br>AY393158<br>AY393159<br>AY393160<br>AY393161<br>AY393162<br>AY393164<br>AY393165<br>AY393166<br>AY393167<br>AY393169<br>AY393170<br>AY393172	Homo sapiens clone RA702-G2-6.fa immunoglobulin heavy chain mRNA, partial cds.<br>Homo sapiens clone RA702-G2-7.fa immunoglobulin heavy chain mRNA, partial cds.<br>Homo sapiens clone RA702-G2-8.fa immunoglobulin heavy chain mRNA, partial cds.<br>Homo sapiens clone RA702-G2-9.fa immunoglobulin heavy chain mRNA, partial cds.<br>Homo sapiens clone RA702-G3-1.fa immunoglobulin heavy chain mRNA, partial cds.<br>Homo sapiens clone RA702-G3-13.fa immunoglobulin heavy chain mRNA, partial cds.<br>Homo sapiens clone RA702-G3-14.fa immunoglobulin heavy chain mRNA, partial cds.<br>Homo sapiens clone RA702-G3-16.fa immunoglobulin heavy chain mRNA, partial cds.<br>Homo sapiens clone RA702-G3-19.fa immunoglobulin heavy chain mRNA, partial cds.<br>Homo sapiens clone RA702-G3-21.fa immunoglobulin heavy chain mRNA, partial cds.<br>Homo sapiens clone RA702-G3-24.fa immunoglobulin heavy chain mRNA, partial cds.<br>Homo sapiens clone RA702-G3-25.fa immunoglobulin heavy chain mRNA, partial cds.<br>Homo sapiens clone RA702-G3-27.fa immunoglobulin heavy chain mRNA, partial cds.<br>Homo sapiens clone RA702-G3-28.fa immunoglobulin heavy chain mRNA, partial cds.<br>Homo sapiens clone RA702-G3-30.fa immunoglobulin heavy chain mRNA, partial cds.<br>Homo sapiens clone RA702-G3-6.fa immunoglobulin heavy chain mRNA, partial cds.<br>Homo sapiens clone RA702-G3-9.fa immunoglobulin heavy chain mRNA, partial cds.<br>Homo sapiens clone RA702-G4-13.fa nonfunctional immunoglobulin heavy chain mRNA, partial sequence.<br>Homo sapiens clone RA702-G4-2.fa immunoglobulin heavy chain mRNA, partial cds.<br>Homo sapiens clone RA702-G4-21.fa immunoglobulin heavy chain mRNA, partial cds.<br>Homo sapiens clone RA702-G4-4.fa immunoglobulin heavy chain mRNA, partial cds.<br>Homo sapiens clone RA702-G4-7.fa immunoglobulin heavy chain mRNA, partial cds.<br>Homo sapiens clone RA702-G4-8.fa immunoglobulin heavy chain mRNA, partial cds.<br>Homo sapiens clone RA702-G5-14.fa immunoglobulin heavy chain mRNA, partial cds.<br>Homo sapiens clone RA702-G5-17.fa immunoglobulin heavy chain mRNA, partial cds.<br>Homo sapiens clone RA702-G5-19.fa immunoglobulin heavy chain mRNA, partial cds.<br>Homo sapiens clone RA702-G5-20.fa immunoglobulin heavy chain mRNA, partial cds.<br>Homo sapiens clone RA702-G5-23.fa immunoglobulin heavy chain mRNA, partial cds.<br>Homo sapiens clone RA702-G5-25.fa immunoglobulin heavy chain mRNA, partial cds.<br>Homo sapiens clone RA702-G5-30.fa immunoglobulin heavy chain mRNA, partial cds.<br>Homo sapiens clone RA702-G5-5.fa immunoglobulin heavy chain mRNA, partial cds.<br>Homo sapiens clone RA702-G5-7.fa immunoglobulin heavy chain mRNA, partial cds.<br>Homo sapiens clone RA702-G5-8.fa immunoglobulin heavy chain mRNA, partial cds.<br>Homo sapiens clone RA702-G6-12.fa immunoglobulin heavy chain mRNA, partial cds.<br>Homo sapiens clone RA702-G6-22.fa immunoglobulin heavy chain mRNA, partial cds.<br>Homo sapiens clone RA702-G6-29.fa immunoglobulin heavy chain mRNA, partial cds.<br>Homo sapiens clone RA702-G6-3.fa immunoglobulin heavy chain mRNA, partial cds.<br>Homo sapiens clone RA702-M1-1.fa immunoglobulin heavy chain mRNA, partial cds.<br>Homo sapiens clone RA702-M1-10.fa immunoglobulin heavy chain mRNA, partial cds.<br>Homo sapiens clone RA702-M1-11.fa immunoglobulin heavy chain mRNA, partial cds.<br>Homo sapiens clone RA702-M1-12.fa immunoglobulin heavy chain mRNA, partial cds.<br>Homo sapiens clone RA702-M1-14.fa immunoglobulin heavy chain mRNA, partial cds.<br>Homo sapiens clone RA702-M1-15.fa immunoglobulin heavy chain mRNA, partial cds.<br>Homo sapiens clone RA702-M1-16.fa nonfunctional immunoglobulin heavy chain mRNA, partial sequence.<br>Homo sapiens clone RA702-M1-17.fa immunoglobulin heavy chain mRNA, partial cds.<br>Homo sapiens clone RA702-M1-18.fa immunoglobulin heavy chain mRNA, partial cds.<br>Homo sapiens clone RA702-M1-20.fa immunoglobulin heavy chain mRNA, partial cds.<br>Homo sapiens clone RA702-M1-21.fa immunoglobulin heavy chain mRNA, partial cds.<br>Homo sapiens clone RA702-M1-22.fa immunoglobulin heavy chain mRNA, partial cds.<br>Homo sapiens clone RA702-M1-23.fa immunoglobulin heavy chain mRNA, partial cds.<br>Homo sapiens clone RA702-M1-25.fa immunoglobulin heavy chain mRNA, partial cds.<br>Homo sapiens clone RA702-M1-26.fa immunoglobulin heavy chain mRNA, partial cds.<br>Homo sapiens clone RA702-M1-27.fa immunoglobulin heavy chain mRNA, partial cds.<br>Homo sapiens clone RA702-M1-28.fa immunoglobulin heavy chain mRNA, partial cds.

								AY393173	Homo sapiens clone RA702-M1-31.fa immunoglobulin heavy chain mRNA, partial cds.
								AY393175	Homo sapiens clone RA702-M1-32.fa immunoglobulin heavy chain mRNA, partial cds.
								AY393176	Homo sapiens clone RA702-M1-34.fa immunoglobulin heavy chain mRNA, partial cds.
								AY393177	Homo sapiens clone RA702-M1-35.fa immunoglobulin heavy chain mRNA, partial cds.
								AY393178	Homo sapiens clone RA702-M1-36.fa immunoglobulin heavy chain mRNA, partial cds.
								AY393179	Homo sapiens clone RA702-M1-37.fa immunoglobulin heavy chain mRNA, partial cds.
								AY393180	Homo sapiens clone RA702-M1-38.fa immunoglobulin heavy chain mRNA, partial cds.
								AY393181	Homo sapiens clone RA702-M1-4.fa immunoglobulin heavy chain mRNA, partial cds.
								AY393182	Homo sapiens clone RA702-M1-40.fa immunoglobulin heavy chain mRNA, partial cds.
								AY393183	Homo sapiens clone RA702-M1-42.fa immunoglobulin heavy chain mRNA, partial cds.
								AY393184	Homo sapiens clone RA702-M1-5.fa immunoglobulin heavy chain mRNA, partial cds.
								AY393185	Homo sapiens clone RA702-M1-6.fa immunoglobulin heavy chain mRNA, partial cds.
								AY393186	Homo sapiens clone RA702-M1-8.fa immunoglobulin heavy chain mRNA, partial cds.
								AY393187	Homo sapiens clone RA702-M1-9.fa immunoglobulin heavy chain mRNA, partial cds.
								AY393188	Homo sapiens clone RA702-M2-1.fa immunoglobulin heavy chain mRNA, partial cds.
								AY393190	Homo sapiens clone RA702-M2-13.fa immunoglobulin heavy chain mRNA, partial cds.
								AY393191	Homo sapiens clone RA702-M2-16.fa immunoglobulin heavy chain mRNA, partial cds.
								AY393192	Homo sapiens clone RA702-M2-18.fa immunoglobulin heavy chain mRNA, partial cds.
								AY393193	Homo sapiens clone RA702-M2-2.fa immunoglobulin heavy chain mRNA, partial cds.
								AY393195	Homo sapiens clone RA702-M2-24.fa immunoglobulin heavy chain mRNA, partial cds.
								AY393196	Homo sapiens clone RA702-M2-27.fa nonfunctional immunoglobulin heavy chain mRNA, partial sequence.
								AY393197	Homo sapiens clone RA702-M2-4.fa immunoglobulin heavy chain mRNA, partial cds.
								AY393199	Homo sapiens clone RA702-M2-6/9.fa immunoglobulin heavy chain mRNA, partial cds.
								AY393200	Homo sapiens clone RA702-M2-8/10/12/29.fa immunoglobulin heavy chain mRNA, partial cds.
								AY393201	Homo sapiens clone RA702-M3-1.fa immunoglobulin heavy chain mRNA, partial cds.
								AY393202	Homo sapiens clone RA702-M3-12.fa immunoglobulin heavy chain mRNA, partial cds.
								AY393203	Homo sapiens clone RA702-M3-14.fa immunoglobulin heavy chain mRNA, partial cds.
								AY393208	Homo sapiens clone RA702-M3-16.fa immunoglobulin heavy chain mRNA, partial cds.
								AY393222	Homo sapiens clone RA702-M3-2.fa immunoglobulin heavy chain mRNA, partial cds.
								AY393223	Homo sapiens clone RA702-M3-24.fa immunoglobulin heavy chain mRNA, partial cds.
								AY393225	Homo sapiens clone RA702-M3-26.fa immunoglobulin heavy chain mRNA, partial cds.
								AY393226	Homo sapiens clone RA702-M3-29.fa immunoglobulin heavy chain mRNA, partial cds.
								AY393227	Homo sapiens clone RA702-M3-30.fa immunoglobulin heavy chain mRNA, partial cds.
								AY393228	Homo sapiens clone RA702-M3-6.fa immunoglobulin heavy chain mRNA, partial cds.
								AY393230	Homo sapiens clone RA702-M3-8.fa immunoglobulin heavy chain mRNA, partial cds.
								AY393233	Homo sapiens clone RA702-M4-11.fa immunoglobulin heavy chain mRNA, partial cds.
								AY393237	Homo sapiens clone RA702-M4-12.fa immunoglobulin heavy chain mRNA, partial cds.
								AY393241	Homo sapiens clone RA702-M4-13.fa immunoglobulin heavy chain mRNA, partial cds.
								AY393244	Homo sapiens clone RA702-M4-14.fa nonfunctional immunoglobulin heavy chain mRNA, partial sequence.
								AY393246	Homo sapiens clone RA702-M4-16.fa immunoglobulin heavy chain mRNA, partial cds.
								AY393248	Homo sapiens clone RA702-M4-17.fa immunoglobulin heavy chain mRNA, partial cds.
								AY393250	Homo sapiens clone RA702-M4-2.fa immunoglobulin heavy chain mRNA, partial cds.
								AY393251	Homo sapiens clone RA702-M4-20.fa immunoglobulin heavy chain mRNA, partial cds.
								AY393253	Homo sapiens clone RA702-M4-23.fa immunoglobulin heavy chain mRNA, partial cds.
								AY393254	Homo sapiens clone RA702-M4-26.fa immunoglobulin heavy chain mRNA, partial cds.
								AY393255	Homo sapiens clone RA702-M4-28.fa immunoglobulin heavy chain mRNA, partial cds.
								AY393257	Homo sapiens clone RA702-M4-29.fa immunoglobulin heavy chain mRNA, partial cds.
								AY393258	Homo sapiens clone RA702-M4-3.fa immunoglobulin heavy chain mRNA, partial cds.
								AY393259	Homo sapiens clone RA702-M4-30.fa immunoglobulin heavy chain mRNA, partial cds.
								AY393260	Homo sapiens clone RA702-M4-4.fa immunoglobulin heavy chain mRNA, partial cds.
								AY393270	Homo sapiens clone RA702-M4-6.fa immunoglobulin heavy chain mRNA, partial cds.
								AY393271	Homo sapiens clone RA702-M4-7.fa immunoglobulin heavy chain mRNA, partial cds.
								AY393272	Homo sapiens clone RA702-M4-8.fa immunoglobulin heavy chain mRNA, partial cds.
								AY393274	Homo sapiens clone RA702-M5-11.fa immunoglobulin heavy chain mRNA, partial cds.

								AY393279	Homo sapiens clone RA702-M5-12.fa immunoglobulin heavy chain mRNA, partial cds.
								AY393282	Homo sapiens clone RA702-M5-13.fa immunoglobulin heavy chain mRNA, partial cds.
								AY393283	Homo sapiens clone RA702-M5-16.fa immunoglobulin heavy chain mRNA, partial cds.
								AY393294	Homo sapiens clone RA702-M5-18.fa immunoglobulin heavy chain mRNA, partial cds.
								AY393295	Homo sapiens clone RA702-M5-19.fa immunoglobulin heavy chain mRNA, partial cds.
								AY393300	Homo sapiens clone RA702-M5-2.fa immunoglobulin heavy chain mRNA, partial cds.
								AY393301	Homo sapiens clone RA702-M5-20.fa immunoglobulin heavy chain mRNA, partial cds.
								AY393302	Homo sapiens clone RA702-M5-22.fa immunoglobulin heavy chain mRNA, partial cds.
								AY393303	Homo sapiens clone RA702-M5-23.fa immunoglobulin heavy chain mRNA, partial cds.
								AY393305	Homo sapiens clone RA702-M5-24.fa immunoglobulin heavy chain mRNA, partial cds.
								AY393307	Homo sapiens clone RA702-M5-29.fa immunoglobulin heavy chain mRNA, partial cds.
								AY393310	Homo sapiens clone RA702-M5-30.fa immunoglobulin heavy chain mRNA, partial cds.
								AY393325	Homo sapiens clone RA702-M5-5.fa immunoglobulin heavy chain mRNA, partial cds.
								AY393326	Homo sapiens clone RA702-M5-6.fa immunoglobulin heavy chain mRNA, partial cds.
								AY393327	Homo sapiens clone RA702-M5-7.fa immunoglobulin heavy chain mRNA, partial cds.
								AY393329	Homo sapiens clone RA702-M6-27.fa immunoglobulin heavy chain mRNA, partial cds.
								AY393332	Homo sapiens isolate RA129-A1-17.fa immunoglobulin heavy chain variable region mRNA, partial cds.
								AY393334	Homo sapiens isolate RA129-A1-18.fa immunoglobulin heavy chain variable region mRNA, partial cds.
								AY393339	Homo sapiens isolate RA129-A1-2.fa immunoglobulin heavy chain variable region mRNA, partial cds.
								AY393340	Homo sapiens isolate RA129-A1-20.fa immunoglobulin heavy chain variable region mRNA, partial cds.
								AY393342	Homo sapiens isolate RA129-A1-21.fa immunoglobulin heavy chain variable region mRNA, partial cds.
								AY393343	Homo sapiens isolate RA129-A1-22.fa immunoglobulin heavy chain variable region mRNA, partial cds.
								AY393344	Homo sapiens isolate RA129-A1-24/40/46.fa immunoglobulin heavy chain variable region mRNA, partial cds.
								AY393348	Homo sapiens isolate RA129-A1-29.fa immunoglobulin heavy chain variable region mRNA, partial cds.
								AY393349	Homo sapiens isolate RA129-A1-33.fa immunoglobulin heavy chain variable region mRNA, partial cds.
								AY393353	Homo sapiens isolate RA129-A1-4.fa immunoglobulin heavy chain variable region mRNA, partial cds.
								AY393354	Homo sapiens isolate RA129-A1-45.fa immunoglobulin heavy chain variable region mRNA, partial cds.
								AY393355	Homo sapiens isolate RA129-A1-48.fa immunoglobulin heavy chain variable region mRNA, partial cds.
								AY393358	Homo sapiens isolate RA129-A1-52.fa immunoglobulin heavy chain variable region mRNA, partial cds.
								AY393361	Homo sapiens isolate RA129-A1-58/88.fa immunoglobulin heavy chain variable region mRNA, partial cds.
								AY393362	Homo sapiens isolate RA129-A1-59.fa immunoglobulin heavy chain variable region mRNA, partial cds.
								AY393363	Homo sapiens isolate RA129-A1-60.fa immunoglobulin heavy chain variable region mRNA, partial cds.
								AY393364	Homo sapiens isolate RA129-A1-61.fa immunoglobulin heavy chain variable region mRNA, partial cds.
								AY393372	Homo sapiens isolate RA129-A1-62.fa immunoglobulin heavy chain variable region mRNA, partial cds.
								AY393375	Homo sapiens isolate RA129-A1-67.fa immunoglobulin heavy chain variable region mRNA, partial cds.
								AY393377	Homo sapiens isolate RA129-A1-68.fa immunoglobulin heavy chain variable region mRNA, partial cds.
								AY393380	Homo sapiens isolate RA129-A1-69.fa immunoglobulin heavy chain variable region mRNA, partial cds.
								AY393382	Homo sapiens isolate RA129-A1-71.fa immunoglobulin heavy chain variable region mRNA, partial cds.
								AY393386	Homo sapiens isolate RA129-A1-86.fa immunoglobulin heavy chain variable region mRNA, partial cds.
								AY393388	Homo sapiens isolate RA129-A1-9/66.fa immunoglobulin heavy chain variable region mRNA, partial cds.
								AY393389	Homo sapiens isolate RA129-A1-90.fa immunoglobulin heavy chain variable region mRNA, partial cds.
								AY393390	Homo sapiens isolate RA129-A2-10 et al4.fa' immunoglobulin heavy chain variable region mRNA, partial cds.
								AY393391	Homo sapiens isolate RA129-A2-2.fa immunoglobulin heavy chain variable region mRNA, partial cds.
								AY393392	Homo sapiens isolate RA129-A2-23.fa immunoglobulin heavy chain variable region mRNA, partial cds.
								AY393393	Homo sapiens isolate RA129-A2-25.fa immunoglobulin heavy chain variable region mRNA, partial cds.
								AY393395	Homo sapiens isolate RA129-A2-50.fa immunoglobulin heavy chain variable region mRNA, partial cds.
								AY393405	Homo sapiens isolate RA129-A2-51.fa immunoglobulin heavy chain variable region mRNA, partial cds.
								AY393409	Homo sapiens isolate RA129-A2-61.fa immunoglobulin heavy chain variable region mRNA, partial cds.
								AY393413	Homo sapiens isolate RA129-A2-64.fa immunoglobulin heavy chain variable region mRNA, partial cds.
								AY393414	Homo sapiens isolate RA129-A2-65.fa immunoglobulin heavy chain variable region mRNA, partial cds.
								AY393416	Homo sapiens isolate RA129-A2-66.fa immunoglobulin heavy chain variable region mRNA, partial cds.
								AY393417	Homo sapiens isolate RA129-A2-70/90.fa immunoglobulin heavy chain variable region mRNA, partial cds.
								AY393418	Homo sapiens isolate RA129-A2-77.fa immunoglobulin heavy chain variable region mRNA, partial cds.
								AY393419	Homo sapiens isolate RA129-A2-84.fa immunoglobulin heavy chain variable region mRNA, partial cds.

								AY393427	Homo sapiens isolate RA129-A6-19.fa immunoglobulin heavy chain variable region mRNA, partial cds.
								AY393428	Homo sapiens isolate RA129-A6-2.fa immunoglobulin heavy chain variable region mRNA, partial cds.
								AY393434	Homo sapiens isolate RA129-A6-21.fa immunoglobulin heavy chain variable region mRNA, partial cds.
								AY393435	Homo sapiens isolate RA129-A6-23.fa immunoglobulin heavy chain variable region mRNA, partial cds.
								AY393436	Homo sapiens isolate RA129-A6-3.fa immunoglobulin heavy chain variable region mRNA, partial cds.
								AY393438	Homo sapiens isolate RA129-A6-6.fa immunoglobulin heavy chain variable region mRNA, partial cds.
								AY393440	Homo sapiens isolate RA129-G1-14.fa immunoglobulin heavy chain variable region mRNA, partial cds.
								AY393446	Homo sapiens isolate RA129-G1-17.fa nonfunctional immunoglobulin heavy chain mRNA, partial sequence.
								AY393449	Homo sapiens isolate RA129-G1-21.fa immunoglobulin heavy chain variable region mRNA, partial cds.
								AY393452	Homo sapiens isolate RA129-G1-22.fa immunoglobulin heavy chain variable region mRNA, partial cds.
								AY393455	Homo sapiens isolate RA129-G1-26.fa immunoglobulin heavy chain variable region mRNA, partial cds.
								AY393458	Homo sapiens isolate RA129-G1-38.fa immunoglobulin heavy chain variable region mRNA, partial cds.
								AY393459	Homo sapiens isolate RA129-G1-4.fa immunoglobulin heavy chain variable region mRNA, partial cds.
								AY393462	Homo sapiens isolate RA129-G1-44.fa immunoglobulin heavy chain variable region mRNA, partial cds.
								AY393466	Homo sapiens isolate RA129-G1-45.fa immunoglobulin heavy chain variable region mRNA, partial cds.
								AY393468	Homo sapiens isolate RA129-G1-48.fa immunoglobulin heavy chain variable region mRNA, partial cds.
								AY393469	Homo sapiens isolate RA129-G1-54.fa immunoglobulin heavy chain variable region mRNA, partial cds.
								AY393476	Homo sapiens isolate RA129-G1-6.fa immunoglobulin heavy chain variable region mRNA, partial cds.
								AY393478	Homo sapiens isolate RA129-G1-63.fa immunoglobulin heavy chain variable region mRNA, partial cds.
								AY393479	Homo sapiens isolate RA129-G1-64.fa immunoglobulin heavy chain variable region mRNA, partial cds.
								AY393481	Homo sapiens isolate RA129-G1-67.fa immunoglobulin heavy chain variable region mRNA, partial cds.
								AY393483	Homo sapiens isolate RA129-G1-78.fa immunoglobulin heavy chain variable region mRNA, partial cds.
								AY393484	Homo sapiens isolate RA129-G1-83.fa immunoglobulin heavy chain variable region mRNA, partial cds.
								AY393486	Homo sapiens isolate RA129-G1-86.fa immunoglobulin heavy chain variable region mRNA, partial cds.
								AY393487	Homo sapiens isolate RA129-G1-9.fa immunoglobulin heavy chain variable region mRNA, partial cds.
								AY393489	Homo sapiens isolate RA129-G2-1.fa immunoglobulin heavy chain variable region mRNA, partial cds.
								AY393491	Homo sapiens isolate RA129-G2-15.fa immunoglobulin heavy chain variable region mRNA, partial cds.
								AY393492	Homo sapiens isolate RA129-G2-17.fa immunoglobulin heavy chain variable region mRNA, partial cds.
								AY393494	Homo sapiens isolate RA129-G2-18/61.fa immunoglobulin heavy chain variable region mRNA, partial cds.
								AY393497	Homo sapiens isolate RA129-G2-19.fa immunoglobulin heavy chain variable region mRNA, partial cds.
								AY393500	Homo sapiens isolate RA129-G2-2.fa immunoglobulin heavy chain variable region mRNA, partial cds.
								AY393506	Homo sapiens isolate RA129-G2-21.fa immunoglobulin heavy chain variable region mRNA, partial cds.
								AY393507	Homo sapiens isolate RA129-G2-23.fa immunoglobulin heavy chain variable region mRNA, partial cds.
								AY393509	Homo sapiens isolate RA129-G2-25.fa immunoglobulin heavy chain variable region mRNA, partial cds.
								AY393510	Homo sapiens isolate RA129-G2-35.fa immunoglobulin heavy chain variable region mRNA, partial cds.
								AY393514	Homo sapiens isolate RA129-G2-4.fa immunoglobulin heavy chain variable region mRNA, partial cds.
								AY393519	Homo sapiens isolate RA129-G2-45.fa immunoglobulin heavy chain variable region mRNA, partial cds.
								AY393521	Homo sapiens isolate RA129-G2-46.fa immunoglobulin heavy chain variable region mRNA, partial cds.
								AY393524	Homo sapiens isolate RA129-G2-48.fa immunoglobulin heavy chain variable region mRNA, partial cds.
								AY393527	Homo sapiens isolate RA129-G2-49.fa immunoglobulin heavy chain variable region mRNA, partial cds.
								AY393528	Homo sapiens isolate RA129-G2-5.fa immunoglobulin heavy chain variable region mRNA, partial cds.
								AY393529	Homo sapiens isolate RA129-G2-51.fa immunoglobulin heavy chain variable region mRNA, partial cds.
								AY393531	Homo sapiens isolate RA129-G2-59.fa immunoglobulin heavy chain variable region mRNA, partial cds.
								AY393533	Homo sapiens isolate RA129-G2-6.fa nonfunctional immunoglobulin heavy chain mRNA, partial sequence.
								AY393534	Homo sapiens isolate RA129-G2-68.fa immunoglobulin heavy chain variable region mRNA, partial cds.
								AY393537	Homo sapiens isolate RA129-G2-69.fa immunoglobulin heavy chain variable region mRNA, partial cds.
								AY393538	Homo sapiens isolate RA129-G2-7.fa immunoglobulin heavy chain variable region mRNA, partial cds.
								AY393539	Homo sapiens isolate RA129-G2-71.fa immunoglobulin heavy chain variable region mRNA, partial cds.
								AY393542	Homo sapiens isolate RA129-G2-73.fa immunoglobulin heavy chain variable region mRNA, partial cds.
								AY393547	Homo sapiens isolate RA129-G2-78.fa immunoglobulin heavy chain variable region mRNA, partial cds.
								AY393548	Homo sapiens isolate RA129-G2-85.fa immunoglobulin heavy chain variable region mRNA, partial cds.
								AY393549	Homo sapiens isolate RA129-G2-89.fa immunoglobulin heavy chain variable region mRNA, partial cds.
								AY393551	Homo sapiens isolate RA129-G5-1.fa immunoglobulin heavy chain variable region mRNA, partial cds.
								AY393553	Homo sapiens isolate RA129-G5-12.fa immunoglobulin heavy chain variable region mRNA, partial cds.

								AY393554	Homo sapiens isolate RA129-G5-13.fa immunoglobulin heavy chain variable region mRNA, partial cds.
								AY393559	Homo sapiens isolate RA129-G5-17.fa immunoglobulin heavy chain variable region mRNA, partial cds.
								AY393560	Homo sapiens isolate RA129-G5-21.fa immunoglobulin heavy chain variable region mRNA, partial cds.
								AY393562	Homo sapiens isolate RA129-G5-4.fa immunoglobulin heavy chain variable region mRNA, partial cds.
								AY393563	Homo sapiens isolate RA129-G5-5.fa immunoglobulin heavy chain variable region mRNA, partial cds.
								AY393564	Homo sapiens isolate RA129-G6-13.fa immunoglobulin heavy chain variable region mRNA, partial cds.
								AY393565	Homo sapiens isolate RA129-G6-15.fa immunoglobulin heavy chain variable region mRNA, partial cds.
								AY393566	Homo sapiens isolate RA129-G6-16.fa immunoglobulin heavy chain variable region mRNA, partial cds.
								AY393567	Homo sapiens isolate RA129-G6-19.fa immunoglobulin heavy chain variable region mRNA, partial cds.
								AY393568	Homo sapiens isolate RA129-G6-22.fa immunoglobulin heavy chain variable region mRNA, partial cds.
								AY393569	Homo sapiens isolate RA129-G6-23.fa immunoglobulin heavy chain variable region mRNA, partial cds.
								AY393570	Homo sapiens isolate RA129-G6-29.fa immunoglobulin heavy chain variable region mRNA, partial cds.
								AY393571	Homo sapiens isolate RA129-G6-3.fa immunoglobulin heavy chain variable region mRNA, partial cds.
								AY393573	Homo sapiens isolate RA129-G6-4.fa immunoglobulin heavy chain variable region mRNA, partial cds.
								AY393580	Homo sapiens isolate RA129-G6-6.fa immunoglobulin heavy chain variable region mRNA, partial cds.
								AY393588	Homo sapiens isolate RA129-G6-7.fa immunoglobulin heavy chain variable region mRNA, partial cds.
								AY393590	Homo sapiens isolate RA129-M1-11.fa immunoglobulin heavy chain variable region mRNA, partial cds.
								AY393592	Homo sapiens isolate RA129-M1-14/65.fa immunoglobulin heavy chain variable region mRNA, partial cds.
								AY393605	Homo sapiens isolate RA129-M1-19/76.fa immunoglobulin heavy chain variable region mRNA, partial cds.
								AY393607	Homo sapiens isolate RA129-M1-3.fa immunoglobulin heavy chain variable region mRNA, partial cds.
								AY393608	Homo sapiens isolate RA129-M1-30.fa nonfunctional immunoglobulin heavy chain mRNA, partial sequence.
								AY393609	Homo sapiens isolate RA129-M1-32/81/88/90.fa immunoglobulin heavy chain variable region mRNA, partial cds.
								AY393610	Homo sapiens isolate RA129-M1-35.fa immunoglobulin heavy chain variable region mRNA, partial cds.
								AY393613	Homo sapiens isolate RA129-M1-39.fa immunoglobulin heavy chain variable region mRNA, partial cds.
								AY393617	Homo sapiens isolate RA129-M1-47.fa immunoglobulin heavy chain variable region mRNA, partial cds.
								AY393625	Homo sapiens isolate RA129-M1-53.fa immunoglobulin heavy chain variable region mRNA, partial cds.
								AY393626	Homo sapiens isolate RA129-M1-56.fa immunoglobulin heavy chain variable region mRNA, partial cds.
								AY393631	Homo sapiens isolate RA129-M1-59.fa immunoglobulin heavy chain variable region mRNA, partial cds.
								AY393633	Homo sapiens isolate RA129-M1-63.fa immunoglobulin heavy chain variable region mRNA, partial cds.
								AY393634	Homo sapiens isolate RA129-M1-64.fa immunoglobulin heavy chain variable region mRNA, partial cds.
								AY393637	Homo sapiens isolate RA129-M1-67.fa immunoglobulin heavy chain variable region mRNA, partial cds.
								AY393640	Homo sapiens isolate RA129-M1-69.fa immunoglobulin heavy chain variable region mRNA, partial cds.
								AY393641	Homo sapiens isolate RA129-M1-7.fa immunoglobulin heavy chain variable region mRNA, partial cds.
								AY393642	Homo sapiens isolate RA129-M1-73.fa immunoglobulin heavy chain variable region mRNA, partial cds.
								AY393644	Homo sapiens isolate RA129-M1-74.fa immunoglobulin heavy chain variable region mRNA, partial cds.
								AY393645	Homo sapiens isolate RA129-M1-75.fa immunoglobulin heavy chain variable region mRNA, partial cds.
								AY393648	Homo sapiens isolate RA129-M1-84.fa immunoglobulin heavy chain variable region mRNA, partial cds.
								AY393651	Homo sapiens isolate RA129-M2-11.fa immunoglobulin heavy chain variable region mRNA, partial cds.
								AY393652	Homo sapiens isolate RA129-M2-12.fa immunoglobulin heavy chain variable region mRNA, partial cds.
								AY393654	Homo sapiens isolate RA129-M2-18.fa immunoglobulin heavy chain variable region mRNA, partial cds.
								AY393655	Homo sapiens isolate RA129-M2-20.fa immunoglobulin heavy chain variable region mRNA, partial cds.
								AY393658	Homo sapiens isolate RA129-M2-28.fa immunoglobulin heavy chain variable region mRNA, partial cds.
								AY393659	Homo sapiens isolate RA129-M2-30.fa immunoglobulin heavy chain variable region mRNA, partial cds.
								AY393662	Homo sapiens isolate RA129-M2-53.fa nonfunctional immunoglobulin heavy chain mRNA, partial sequence.
								AY393663	Homo sapiens isolate RA129-M2-67/68.fa immunoglobulin heavy chain variable region mRNA, partial cds.
								AY393664	Homo sapiens isolate RA129-M2-74.fa immunoglobulin heavy chain variable region mRNA, partial cds.
								AY393665	Homo sapiens isolate RA129-M2-84.fa immunoglobulin heavy chain variable region mRNA, partial cds.
								AY393666	Homo sapiens isolate RA129-M2-87.fa nonfunctional immunoglobulin heavy chain mRNA, partial sequence.
								AY393668	Homo sapiens isolate RA129-M2-9/55/72/75.fa immunoglobulin heavy chain variable region mRNA, partial cds.
								AY393671	Homo sapiens isolate RA129-M5-1.fa immunoglobulin heavy chain variable region mRNA, partial cds.
								AY393675	Homo sapiens isolate RA129-M5-2.fa immunoglobulin heavy chain variable region mRNA, partial cds.
								AY393676	Homo sapiens isolate RA129-M5-3.fa immunoglobulin heavy chain variable region mRNA, partial cds.
								AY393679	Homo sapiens isolate RA129-M5-4.fa immunoglobulin heavy chain variable region mRNA, partial cds.
								AY393680	Homo sapiens isolate RA129-M5-5.fa immunoglobulin heavy chain variable region mRNA, partial cds.

								AY393681<br>AY393684<br>AY393686<br>AY393690<br>AY393695<br>AY422530<br>AY422534<br>AY422540<br>AY423163<br>AY423164<br>AY423166<br>AY423167<br>AY423168<br>AY423180<br>AY423182<br>AY423188<br>AY423189<br>AY423192<br>AY423196<br>AY452137<br>AY452138<br>AY466506<br>AY466507<br>AY466508<br>AY466509<br>AY466513<br>AY485363<br>AY505567<br>AY505569<br>AY506559<br>AY510104<br>AY510105<br>AY515000<br>AY515002<br>AY515004<br>AY515006<br>AY515008<br>AY515010<br>AY530312<br>AY530313<br>AY530314<br>AY530315<br>AY530316<br>AY530317<br>AY530318<br>AY530319<br>AY532650<br>AY553641<br>AY553642<br>AY553643<br>AY553644<br>AY553645<br>AY553646<br>AY553648	Homo sapiens isolate RA129-M5-6.fa immunoglobulin heavy chain variable region mRNA, partial cds.<br>Homo sapiens isolate RA129-M5-8.fa immunoglobulin heavy chain variable region mRNA, partial cds.<br>Homo sapiens isolate RA129-M6-17.fa immunoglobulin heavy chain variable region mRNA, partial cds.<br>Homo sapiens isolate RA129-M6-26.fa immunoglobulin heavy chain variable region mRNA, partial cds.<br>Homo sapiens isolate RA129-M6-29.fa immunoglobulin heavy chain variable region mRNA, partial cds.<br>Homo sapiens isolate RA129-M6-30.fa immunoglobulin heavy chain variable region mRNA, partial cds.<br>Homo sapiens clone RA130BM-G1-106 immunoglobulin heavy chain variable region mRNA, partial cds.<br>Homo sapiens clone RA130BM-G1-108 immunoglobulin heavy chain variable region mRNA, partial cds.<br>Homo sapiens clone RA130BM-G1-109 immunoglobulin heavy chain variable region mRNA, partial cds.<br>Homo sapiens clone RA130BM-G1-11 immunoglobulin heavy chain variable region mRNA, partial cds.<br>Homo sapiens clone RA130BM-G1-110 immunoglobulin heavy chain variable region mRNA, partial cds.<br>Homo sapiens clone RA130BM-G1-113 immunoglobulin heavy chain variable region mRNA, partial cds.<br>Homo sapiens clone RA130BM-G1-117 immunoglobulin heavy chain variable region mRNA, partial cds.<br>Homo sapiens clone RA130BM-G1-131 immunoglobulin heavy chain variable region mRNA, partial cds.<br>Homo sapiens clone RA130BM-G1-132 immunoglobulin heavy chain variable region mRNA, partial cds.<br>Homo sapiens clone RA130BM-G1-137 immunoglobulin heavy chain variable region mRNA, partial cds.<br>Homo sapiens clone RA130BM-G1-139/187 immunoglobulin heavy chain variable region mRNA, partial cds.<br>Homo sapiens clone RA130BM-G1-140.<br>Homo sapiens clone RA130BM-G1-145 immunoglobulin heavy chain variable region mRNA, partial cds.<br>Homo sapiens clone RA130BM-G1-148 immunoglobulin heavy chain variable region mRNA, partial cds.<br>Homo sapiens clone RA130BM-G1-150 immunoglobulin heavy chain variable region mRNA, partial cds.<br>Homo sapiens clone RA130BM-G1-151 immunoglobulin heavy chain variable region mRNA, partial cds.<br>Homo sapiens clone RA130BM-G1-153 immunoglobulin heavy chain variable region mRNA, partial cds.<br>Homo sapiens clone RA130BM-G1-154 immunoglobulin heavy chain variable region mRNA, partial cds.<br>Homo sapiens clone RA130BM-G1-158 immunoglobulin heavy chain variable region mRNA, partial cds.<br>Homo sapiens clone RA130BM-G1-161 immunoglobulin heavy chain variable region mRNA, partial cds.<br>Homo sapiens clone RA130BM-G1-162 immunoglobulin heavy chain variable region mRNA, partial cds.<br>Homo sapiens clone RA130BM-G1-164 immunoglobulin heavy chain variable region mRNA, partial cds.<br>Homo sapiens clone RA130BM-G1-165 immunoglobulin heavy chain variable region mRNA, partial cds.<br>Homo sapiens clone RA130BM-G1-168 immunoglobulin heavy chain variable region mRNA, partial cds.<br>Homo sapiens clone RA130BM-G1-17 immunoglobulin heavy chain variable region mRNA, partial cds.<br>Homo sapiens clone RA130BM-G1-173 immunoglobulin heavy chain variable region mRNA, partial cds.<br>Homo sapiens clone RA130BM-G1-174 immunoglobulin heavy chain variable region mRNA, partial cds.<br>Homo sapiens clone RA130BM-G1-175 immunoglobulin heavy chain variable region mRNA, partial cds.<br>Homo sapiens clone RA130BM-G1-176 immunoglobulin heavy chain variable region mRNA, partial cds.<br>Homo sapiens clone RA130BM-G1-177 immunoglobulin heavy chain variable region mRNA, partial cds.<br>Homo sapiens clone RA130BM-G1-179 immunoglobulin heavy chain variable region mRNA, partial cds.<br>Homo sapiens clone RA130BM-G1-181 immunoglobulin heavy chain variable region mRNA, partial cds.<br>Homo sapiens clone RA130BM-G1-186 immunoglobulin heavy chain variable region mRNA, partial cds.<br>Homo sapiens clone RA130BM-G1-188 immunoglobulin heavy chain variable region mRNA, partial cds.<br>Homo sapiens clone RA130BM-G1-34 immunoglobulin heavy chain variable region mRNA, partial cds.<br>Homo sapiens clone RA130BM-G1-37 immunoglobulin heavy chain variable region mRNA, partial cds.<br>Homo sapiens clone RA130BM-G1-38 immunoglobulin heavy chain variable region mRNA, partial cds.<br>Homo sapiens clone RA130BM-G1-41 immunoglobulin heavy chain variable region mRNA, partial cds.<br>Homo sapiens clone RA130BM-G1-44 immunoglobulin heavy chain variable region mRNA, partial cds.<br>Homo sapiens clone RA130BM-G1-60 immunoglobulin heavy chain variable region mRNA, partial cds.<br>Homo sapiens clone RA130BM-G1-8 immunoglobulin heavy chain variable region mRNA, partial cds.<br>Homo sapiens clone 2-5 immunoglobulin heavy chain mRNA, partial cds.<br>Homo sapiens clone 2-12 nonfunctional immunoglobulin heavy chain gene, partial sequence.<br>Homo sapiens clone 32 immunoglobulin heavy chain mRNA, partial cds.<br>Homo sapiens clone 001.5A1H immunoglobulin heavy chain variable region mRNA, partial cds.<br>Homo sapiens clone 002.1F2H immunoglobulin heavy chain variable region mRNA, partial cds.<br>Homo sapiens clone 002.4B4H immunoglobulin heavy chain variable region mRNA, partial cds.<br>Homo sapiens clone 002.4E6H immunoglobulin heavy chain variable region mRNA, partial cds.

								AY569446	Homo sapiens clone 002.7B11H immunoglobulin heavy chain variable region mRNA, partial cds.
								AY573190	Homo sapiens clone 010.2C3H immunoglobulin heavy chain variable region mRNA, partial cds.
								AY621628	Homo sapiens clone 010.3H10H immunoglobulin heavy chain variable region mRNA, partial cds.
								AY623427	Homo sapiens clone 010.8D9H immunoglobulin heavy chain variable region mRNA, partial cds.
								AY626664	Homo sapiens clone 011.10E1H immunoglobulin heavy chain variable region mRNA, partial cds.
								AY640472	Homo sapiens clone 011.14A5H immunoglobulin heavy chain variable region mRNA, partial cds.
								AY640473	Homo sapiens clone 011.16A4H immunoglobulin heavy chain variable region mRNA, partial cds.
								AY640474	Homo sapiens clone G14F7E5 immunoglobulin heavy chain mRNA, partial cds.
								AY640476	Homo sapiens clone G15B4G5 immunoglobulin heavy chain mRNA, partial cds.
								AY640477	Homo sapiens isolate B10 immunoglobulin heavy chain variable region mRNA, partial cds.
								AY640480	Homo sapiens isolate B46 immunoglobulin heavy chain variable region mRNA, partial cds.
								AY640484	Homo sapiens isolate B3814 immunoglobulin heavy chain variable region mRNA, partial cds.
								AY640485	Homo sapiens isolate B4847 immunoglobulin heavy chain variable region mRNA, partial cds.
								AY640486	Homo sapiens isolate DLBCL21 immunoglobulin heavy chain variable region mRNA, partial cds.
								AY640497	Homo sapiens CSF mAb MS2 immunoglobulin G heavy chain variable region mRNA, partial cds.
								AY640506	Homo sapiens immunoglobulin heavy chain mRNA, partial cds.
								AY640509	Homo sapiens immunoglobulin heavy chain mRNA, partial cds.
								AY640510	Homo sapiens anti-Toxoplasma gondii SAG1 immunoglobulin heavy chain variable region mRNA, partial cds.
								AY640511	Homo sapiens 2G9 monoclonal IgM antibody heavy chain mRNA, complete cds.
								AY640512	Homo sapiens 9F11 monoclonal IgM antibody heavy chain mRNA, complete cds.
								AY640513	Homo sapiens anti-HIV-1 gp120 immunoglobulin 48d heavy chain mRNA, partial cds.
								AY640514	Homo sapiens anti-HIV-1 gp120 immunoglobulin 411g heavy chain mRNA, partial cds.
								AY640528	Homo sapiens anti-HIV-1 gp120 immunoglobulin 16c heavy chain mRNA, partial cds.
								AY640529	Homo sapiens anti-HIV-1 gp120 immunoglobulin 23e heavy chain mRNA, partial cds.
								AY640531	Homo sapiens anti-HIV-1 gp120 immunoglobulin 47e heavy chain mRNA, partial cds.
								AY640539	Homo sapiens anti-HIV-1 gp120 immunoglobulin 412d heavy chain mRNA, partial cds.
								AY640543	Homo sapiens clone 6A anti-SARS S protein immunoglobulin heavy chain variable region mRNA, partial cds.
								AY640547	Homo sapiens clone 8C anti-SARS S protein immunoglobulin heavy chain variable region mRNA, partial cds.
								AY640548	Homo sapiens clone 12E anti-SARS S protein immunoglobulin heavy chain variable region mRNA, partial cds.
								AY640549	Homo sapiens clone 26H anti-SARS S protein immunoglobulin heavy chain variable region mRNA, partial cds.
								AY640550	Homo sapiens clone 27D anti-SARS S protein immunoglobulin heavy chain variable region mRNA, partial cds.
								AY640552	Homo sapiens clone 80R anti-SARS S protein immunoglobulin heavy chain variable region mRNA, partial cds.
								AY640557	Homo sapiens clone 91M anti-SARS S protein immunoglobulin heavy chain variable region mRNA, partial cds.
								AY640558	Homo sapiens clone 92N anti-SARS S protein immunoglobulin heavy chain variable region mRNA, partial cds.
								AY640561	Homo sapiens immunoglobulin heavy chain variable region mRNA, partial cds.
								AY640567	Homo sapiens clone CLL240 immunoglobulin gamma heavy chain variable region mRNA, partial cds.
								AY640568	Homo sapiens clone CLL266 immunoglobulin mu heavy chain variable region mRNA, partial cds.
								AY640570	Homo sapiens clone CLL282 immunoglobulin mu heavy chain variable region mRNA, partial cds.
								AY640574	Homo sapiens clone CLL336 immunoglobulin mu heavy chain variable region mRNA, partial cds.
								AY640579	Homo sapiens clone CLL340 immunoglobulin mu heavy chain variable region mRNA, partial cds.
								AY640580	Homo sapiens clone CLL342 immunoglobulin gamma heavy chain variable region mRNA, partial cds.
								AY646422	Homo sapiens clone CLL412 immunoglobulin mu heavy chain variable region mRNA, partial cds.
								AY685252	Homo sapiens anti-histone monoclonal antibody Fab SLEhis19 immunoglobulin heavy chain variable region mRNA, partial cds.
								AY685258	Homo sapiens N2A immunoglobulin heavy chain variable region mRNA, partial cds.
								AY685266	Homo sapiens immunoglobulin heavy chain variable region mRNA, partial cds.
								AY685268	Homo sapiens immunoglobulin heavy constant gamma 1-like protein (IGHG1) mRNA, complete cds.
								AY685298	Homo sapiens hybridoma F428 immunoglobulin heavy chain variable region mRNA, partial cds.
								AY685306	Homo sapiens clone AA immunoglobulin E variable region mRNA, partial cds.
								AY685317	Homo sapiens clone AB immunoglobulin E variable region mRNA, partial cds.
								AY685318	Homo sapiens clone AC immunoglobulin E variable region mRNA, partial cds.
								AY686868	Homo sapiens clone AE immunoglobulin E variable region mRNA, partial cds.
								AY686869	Homo sapiens clone AF immunoglobulin E variable region mRNA, partial cds.
								AY686870	Homo sapiens clone AI nonfunctional immunoglobulin E variable region mRNA, partial sequence.
								AY686872	Homo sapiens clone AM immunoglobulin E variable region mRNA, partial cds.

								AY686884	Homo sapiens clone AN immunoglobulin E variable region mRNA, partial cds.
								AY686891	Homo sapiens clone AO immunoglobulin E variable region mRNA, partial cds.
								AY686900	Homo sapiens clone AZ immunoglobulin E variable region mRNA, partial cds.
								AY686906	Homo sapiens clone BJ immunoglobulin E variable region mRNA, partial cds.
								AY686907	Homo sapiens clone BM immunoglobulin E variable region mRNA, partial cds.
								AY686908	Homo sapiens clone BN immunoglobulin E variable region mRNA, partial cds.
								AY686909	Homo sapiens clone BO immunoglobulin E variable region mRNA, partial cds.
								AY686918	Homo sapiens clone BP immunoglobulin E variable region mRNA, partial cds.
								AY686923	Homo sapiens clone BQ immunoglobulin E variable region mRNA, partial cds.
								AY686925	Homo sapiens clone BR immunoglobulin E variable region mRNA, partial cds.
								AY686930	Homo sapiens clone CF immunoglobulin E variable region mRNA, partial cds.
								AY686931	Homo sapiens clone CG nonfunctional immunoglobulin E variable region mRNA, partial sequence.
								AY686937	Homo sapiens clone CI immunoglobulin E variable region mRNA, partial cds.
								AY686938	Homo sapiens clone PF immunoglobulin E variable region mRNA, partial cds.
								AY686939	Homo sapiens clone PJ immunoglobulin E variable region mRNA, partial cds.
								AY749161	Homo sapiens clone PN immunoglobulin E variable region mRNA, partial cds.
								AY781807	Homo sapiens clone PO immunoglobulin E variable region mRNA, partial cds.
								AY781808	Homo sapiens clone PP immunoglobulin E variable region mRNA, partial cds.
								AY781817	Homo sapiens clone PQ immunoglobulin E variable region mRNA, partial cds.
								AY781820	Homo sapiens clone PS immunoglobulin E variable region mRNA, partial cds.
								AY781821	Homo sapiens clone PX immunoglobulin E variable region mRNA, partial cds.
								AY781823	Homo sapiens clone PY immunoglobulin E variable region mRNA, partial cds.
								AY781824	Homo sapiens clone RB immunoglobulin E variable region mRNA, partial cds.
								AY781826	Homo sapiens clone RH immunoglobulin E variable region mRNA, partial cds.
								AY781827	Homo sapiens clone RI immunoglobulin E variable region mRNA, partial cds.
								AY781828	Homo sapiens clone RK immunoglobulin E variable region mRNA, partial cds.
								AY781831	Homo sapiens clone RO nonfunctional immunoglobulin E variable region mRNA, partial sequence.
								AY781832	Homo sapiens clone RT immunoglobulin E variable region mRNA, partial cds.
								AY781837	Homo sapiens clone RU immunoglobulin E variable region mRNA, partial cds.
								AY781840	Homo sapiens immunoglobulin heavy chain mRNA, partial cds.
								AY781845	Homo sapiens clone ASYN02 immunoglobulin variable region VH gamma domain mRNA, partial cds.
								AY781846	Homo sapiens clone ASYN08 immunoglobulin variable region VH gamma domain mRNA, partial cds.
								AY781847	Homo sapiens clone ASYN16 immunoglobulin variable region VH gamma domain mRNA, partial cds.
								AY781848	Homo sapiens clone ASYN18 immunoglobulin variable region VH gamma domain mRNA, partial cds.
								AY781849	Homo sapiens clone BSYN04 immunoglobulin variable region VH gamma domain mRNA, partial cds.
								AY781852	Homo sapiens clone BSYN12 immunoglobulin variable region VH gamma domain mRNA, partial cds.
								AY781854	Homo sapiens clone BSYN23 immunoglobulin variable region VH gamma domain mRNA, partial cds.
								AY781855	Homo sapiens clone BSYN24 immunoglobulin variable region VH gamma domain mRNA, partial cds.
								AY781856	Homo sapiens clone RBCI-1 random intestinal-homing antibody heavy chain variable region mRNA, partial cds.
								AY781858	Homo sapiens clone RBCI-2 random intestinal-homing antibody heavy chain variable region mRNA, partial cds.
								AY781859	Homo sapiens clone RBCI-3 random intestinal-homing antibody heavy chain variable region mRNA, partial cds.
								AY781862	Homo sapiens clone RBCI-7 random intestinal-homing antibody heavy chain variable region mRNA, partial cds.
								AY781863	Homo sapiens clone RBCI-23 random intestinal-homing antibody heavy chain variable region mRNA, partial cds.
								AY781865	Homo sapiens clone RVI-5 rotavirus-specific intestinal-homing antibody heavy chain variable region mRNA, partial cds.
								AY781867	Homo sapiens clone RVI-14 rotavirus-specific intestinal-homing antibody heavy chain variable region mRNA, partial cds.
								AY781877	Homo sapiens clone RVI-20 rotavirus-specific intestinal-homing antibody heavy chain variable region mRNA, partial cds.
								AY781879	Homo sapiens clone RVI-21 rotavirus-specific intestinal-homing antibody heavy chain variable region mRNA, partial cds.
								AY781882	Homo sapiens clone RVI-22 rotavirus-specific intestinal-homing antibody heavy chain variable region mRNA, partial cds.
								AY781883	Homo sapiens clone RVI-23 rotavirus-specific intestinal-homing antibody heavy chain variable region mRNA, partial cds.
								AY789123	Homo sapiens clone RVI-30 rotavirus-specific intestinal-homing antibody heavy chain variable region mRNA, partial cds.
								AY846266	Homo sapiens clone RVI-33 rotavirus-specific intestinal-homing antibody heavy chain variable region mRNA, partial cds.
								AY846269	Homo sapiens clone RVI-34 rotavirus-specific intestinal-homing antibody heavy chain variable region mRNA, partial cds.
								AY846272	Homo sapiens clone RVI-37 rotavirus-specific intestinal-homing antibody heavy chain variable region mRNA, partial cds.
								AY866546	Homo sapiens clone RVI-40 rotavirus-specific intestinal-homing antibody heavy chain variable region mRNA, partial cds.

								AY866551<br>AY866567<br>AY866625<br>AY866641<br>AY866678<br>AY866728<br>AY866751<br>AY866766<br>AY866771<br>AY866786<br>AY866788<br>AY866792<br>AY866812<br>AY880990<br>AY885180<br>AY885215<br>AY885219<br>AY894992<br>AY921575<br>AY921576<br>AY927661<br>AY941845<br>AY941848<br>AY941850<br>AY941855<br>AY941856<br>AY941867<br>AY941873<br>AY941885<br>AY941886<br>AY941891<br>AY941892<br>AY941895<br>AY941897<br>AY941899<br>AY941901<br>AY941902<br>AY941903<br>AY941905<br>AY941906<br>AY941912<br>AY941917<br>AY941918<br>AY941925<br>AY941926<br>AY941927<br>AY941928<br>AY941930<br>AY941931<br>AY941934<br>AY941935<br>AY941937<br>AY941938<br>AY941939	Homo sapiens clone RVI-58 rotavirus-specific intestinal-homing antibody heavy chain variable region mRNA, partial cds.<br>Homo sapiens clone RVI-60 rotavirus-specific intestinal-homing antibody heavy chain variable region mRNA, partial cds.<br>Homo sapiens clone RVI-61 rotavirus-specific intestinal-homing antibody heavy chain variable region mRNA, partial cds.<br>Homo sapiens clone 011.11A11 immunoglobulin heavy chain variable region mRNA, partial cds.<br>Homo sapiens isolate C10 MK2 non allergic IgE Heavy chain IGHV3-21*01 mRNA, partial cds.<br>Homo sapiens isolate C15 MK2 non-allergic IgE heavy chain IGHV3-11 mRNA, partial cds.<br>Homo sapiens isolate A1 AF2 non-allergic IgE heavy chain IGHV3-33*05 mRNA, partial cds.<br>Homo sapiens isolate A16 AF2 non-allergic IgE heavy chain IGHV2-70 mRNA, partial cds.<br>Homo sapiens isolate A20 AF2 non-allergic IgE heavy chain IGHV3-31 mRNA, partial cds.<br>Homo sapiens isolate A22 AF2 non-allergic IgE heavy chain IGHV4-31 mRNA, partial cds.<br>Homo sapiens isolate A30 AF2 non-allergic IgE heavy chain IGHV3-23*01 mRNA, partial cds.<br>Homo sapiens isolate A37 AF2 non-allergic IgE heavy chain IGHV3-23*01 mRNA, partial cds.<br>Homo sapiens isolate A44 AF2 non-allergic IgE heavy chain IGHV3-23 mRNA, partial cds.<br>Homo sapiens isolate A49 AF2 non-allergic IgE heavy chain IGHV3-23 mRNA, partial cds.<br>Homo sapiens isolate L12 AF1 allergic IgE heavy chain IGHV4-4 mRNA, partial cds.<br>Homo sapiens isolate L14 AF1 allergic IgE heavy chain IGHV4-4 mRNA, partial cds.<br>Homo sapiens isolate E21 AF1 non-allergic IgE heavy chain IGHV4-30-4 mRNA, partial cds.<br>Homo sapiens isolate L17 AF1 non-allergic IgE heavy chain IGHV4-30-4 mRNA, partial cds.<br>Homo sapiens isolate L3 AF1 non-allergic IgE heavy chain IGHV4-31 mRNA, partial cds.<br>Homo sapiens isolate L4 AF1 non-allergic IgE heavy chain IGHV4 mRNA, partial cds.<br>Homo sapiens isolate L6 AF1 non-allergic IgE heavy chain IGHV4-31 mRNA, partial cds.<br>Homo sapiens isolate C2 AF1 non-allergic IgE heavy chain IGHV4-39 mRNA, partial cds.<br>Homo sapiens isolate E14 AF1 non-allergic IgE heavy chain IGHV4-39 mRNA, partial cds.<br>Homo sapiens isolate E2 AF1 non-allergic IgE heavy chain IGHV4-59 mRNA, partial cds.<br>Homo sapiens isolate A5 AF1 non-allergic IgE heavy chain IGHV4-61*02 mRNA, partial cds.<br>Homo sapiens isolate A29 AF1 non-allergic IgE heavy chain IGHV4-61 mRNA, partial cds.<br>Homo sapiens isolate C4 AF1 non-allergic IgE heavy chain IGHV4-61 mRNA, partial cds.<br>Homo sapiens isolate A10 AF1 non-allergic IgE heavy chain IGHV1-8 mRNA, partial cds.<br>Homo sapiens isolate E12 AF1 non-allergic IgE heavy chain VH3-30 mRNA, partial cds.<br>Homo sapiens isolate E24 AF1 non-allergic IgE heavy chain VH4-61 mRNA, partial cds.<br>Homo sapiens isolate L15 AF1 non-allergic IgE heavy chain IGHV4-59 mRNA, partial cds.<br>Homo sapiens isolate L25 AF1 non-allergic IgE heavy chain IGHV4-61 mRNA, partial cds.<br>Homo sapiens isolate P5 AF1 non-allergic IgE heavy chain IGHV6-1 mRNA, partial cds.<br>Homo sapiens isolate A7 AF1 non-allergic IgE heavy chain VH3-30 mRNA, partial cds.<br>Homo sapiens isolate A32 AF1 non-allergic IgE heavy chain IGHV3-30 mRNA, partial cds.<br>Homo sapiens isolate E7 AF1 non-allergic IgE heavy chain IGHV3-30-3 mRNA, partial cds.<br>Homo sapiens isolate E16 AF1 non-allergic IgE heavy chain IGHV3-30-3 mRNA, partial cds.<br>Homo sapiens anti-Toxoplasma gondii SAG1 immunoglobulin s2 heavy chain variable region mRNA, partial cds.<br>Homo sapiens clone NA-1a immunoglobulin epsilon heavy chain variable region mRNA, partial cds.<br>Homo sapiens clone NA-2b immunoglobulin epsilon heavy chain variable region mRNA, partial cds.<br>Homo sapiens clone NA-5 immunoglobulin epsilon heavy chain variable region mRNA, partial cds.<br>Homo sapiens clone 60a02 anti-tetanus toxoid immunoglobulin heavy chain variable region (IGH@) mRNA, partial cds.<br>Homo sapiens clone 60a11 anti-tetanus toxoid immunoglobulin heavy chain variable region (IGH@) mRNA, partial cds.<br>Homo sapiens clone 60f03 anti-tetanus toxoid immunoglobulin heavy chain variable region-like (IGH@) mRNA, partial<br>sequence.<br>Homo sapiens clone 62c06 anti-tetanus toxoid immunoglobulin heavy chain variable region (IGH@) mRNA, partial cds.<br>Homo sapiens clone 62e05 anti-tetanus toxoid immunoglobulin heavy chain variable region (IGH@) mRNA, partial cds.<br>Homo sapiens clone 63b07 anti-tetanus toxoid immunoglobulin heavy chain variable region (IGH@) mRNA, partial cds.<br>Homo sapiens clone 135b10 anti-tetanus toxoid immunoglobulin heavy chain variable region (IGH@) mRNA, partial cds.<br>Homo sapiens clone 135e03 anti-tetanus toxoid immunoglobulin heavy chain variable region (IGH@) mRNA, partial cds.<br>Homo sapiens clone 135f11 anti-tetanus toxoid immunoglobulin heavy chain variable region (IGH@) mRNA, partial cds.<br>Homo sapiens clone 135g05 anti-tetanus toxoid immunoglobulin heavy chain variable region (IGH@) mRNA, partial cds.<br>Homo sapiens clone 152a11 anti-tetanus toxoid immunoglobulin heavy chain variable region (IGH@) mRNA, partial cds.<br>Homo sapiens clone 152b06 anti-tetanus toxoid immunoglobulin heavy chain variable region (IGH@) mRNA, partial cds.

								AY941941	Homo sapiens clone 152b10 anti-tetanus toxoid immunoglobulin heavy chain variable region (IGH@) mRNA, partial cds.
								AY941943	Homo sapiens clone 152e03 anti-tetanus toxoid immunoglobulin heavy chain variable region (IGH@) mRNA, partial cds.
								AY941946	Homo sapiens anti-asialoglycoprotein receptor immunoglobulin C2 heavy chain variable region mRNA, partial cds.
								AY941947	Homo sapiens isolate HBL-5-VH1 immunoglobulin heavy chain variable region mRNA, partial cds.
								AY941949	Homo sapiens isolate A-Rice94-MT-IgA1 immunoglobulin alpha 1 heavy chain constant region mRNA, partial cds.
								AY941954	Homo sapiens isolate BW1-MT-IgG1 immunoglobulin gamma 1 heavy chain constant region mRNA, partial cds.
								AY941960	Homo sapiens anti-RhD monoclonal T125 gamma1 heavy chain precursor, mRNA, complete cds.
								AY941962	Homo sapiens isolate BL case 4 immunoglobulin heavy chain variable region mRNA, partial cds.
								AY941963	Homo sapiens Igamma3-Cmu fusion RNA transcript gene, complete sequence.
								AY941964	Homo sapiens isolate M46 immunoglobulin heavy chain variable region mRNA, partial cds.
								AY941966	Homo sapiens clone SC4008 anti-rabies virus immunoglobulin heavy chain variable region mRNA, partial cds.
								AY941979	Homo sapiens clone SC4011 anti-rabies virus immunoglobulin heavy chain variable region mRNA, partial cds.
								AY941981	Homo sapiens clone SC4013 anti-rabies virus immunoglobulin heavy chain variable region mRNA, partial cds.
								AY941983	Homo sapiens clone SC4019 anti-rabies virus immunoglobulin heavy chain variable region mRNA, partial cds.
								AY941988	Homo sapiens clone SC4020 anti-rabies virus immunoglobulin heavy chain variable region mRNA, partial cds.
								AY944687	Homo sapiens clone SC4032 anti-rabies virus immunoglobulin heavy chain variable region mRNA, partial cds.
								AY944688	Homo sapiens clone SC4039 anti-rabies virus immunoglobulin heavy chain variable region mRNA, partial cds.
								AY944692	Homo sapiens clone SC4052 anti-rabies virus immunoglobulin heavy chain variable region mRNA, partial cds.
								AY944693	Homo sapiens clone SC4053 anti-rabies virus immunoglobulin heavy chain variable region mRNA, partial cds.
								AY944694	Homo sapiens clone SC4058 anti-rabies virus immunoglobulin heavy chain variable region mRNA, partial cds.
								AY944695	Homo sapiens clone SC4059 anti-rabies virus immunoglobulin heavy chain variable region mRNA, partial cds.
								AY944696	Homo sapiens clone SC4062 anti-rabies virus immunoglobulin heavy chain variable region mRNA, partial cds.
								AY944698	Homo sapiens clone SC4064 anti-rabies virus immunoglobulin heavy chain variable region mRNA, partial cds.
								AY944703	Homo sapiens clone SC4066 anti-rabies virus immunoglobulin heavy chain variable region mRNA, partial cds.
								AY944704	Homo sapiens clone SC4068 anti-rabies virus immunoglobulin heavy chain variable region mRNA, partial cds.
								AY944705	Homo sapiens clone SC4069 anti-rabies virus immunoglobulin heavy chain variable region mRNA, partial cds.
								AY944707	Homo sapiens clone SC4070 anti-rabies virus immunoglobulin heavy chain variable region mRNA, partial cds.
								AY944708	Homo sapiens clone SC4073 anti-rabies virus immunoglobulin heavy chain variable region mRNA, partial cds.
								AY944709	Homo sapiens clone SC4074 anti-rabies virus immunoglobulin heavy chain variable region mRNA, partial cds.
								AY944710	Homo sapiens clone SC4082 anti-rabies virus immunoglobulin heavy chain variable region mRNA, partial cds.
								AY944711	Homo sapiens clone SC4087 anti-rabies virus immunoglobulin heavy chain variable region mRNA, partial cds.
								AY944714	Homo sapiens clone SC4088 anti-rabies virus immunoglobulin heavy chain variable region mRNA, partial cds.
								AY944715	Homo sapiens clone SC4095 anti-rabies virus immunoglobulin heavy chain variable region mRNA, partial cds.
								AY944716	Homo sapiens clone SC4096 anti-rabies virus immunoglobulin heavy chain variable region mRNA, partial cds.
								AY944717	Homo sapiens clone SC4097 anti-rabies virus immunoglobulin heavy chain variable region mRNA, partial cds.
								AY944719	Homo sapiens clone SC4098 anti-rabies virus immunoglobulin heavy chain variable region mRNA, partial cds.
								AY944720	Homo sapiens clone SC4100 anti-rabies virus immunoglobulin heavy chain variable region mRNA, partial cds.
								AY944721	Homo sapiens clone SC4101 anti-rabies virus immunoglobulin heavy chain variable region mRNA, partial cds.
								AY944722	Homo sapiens clone SC4104 anti-rabies virus immunoglobulin heavy chain variable region mRNA, partial cds.
								AY952380	Homo sapiens clone SC4105 anti-rabies virus immunoglobulin heavy chain variable region mRNA, partial cds.
								AY952642	Homo sapiens clone SC4107 anti-rabies virus immunoglobulin heavy chain variable region mRNA, partial cds.
								AY955070	Homo sapiens clone SC4108 anti-rabies virus immunoglobulin heavy chain variable region mRNA, partial cds.
								AY957510	Homo sapiens clone SC4109 anti-rabies virus immunoglobulin heavy chain variable region mRNA, partial cds.
								AY957534	Homo sapiens clone SC4111 anti-rabies virus immunoglobulin heavy chain variable region mRNA, partial cds.
								AY960198	Homo sapiens clone SC4113 anti-rabies virus immunoglobulin heavy chain variable region mRNA, partial cds.
								AY960200	Homo sapiens clone SC4116 anti-rabies virus immunoglobulin heavy chain variable region mRNA, partial cds.
								AY960861	Homo sapiens clone SC4117 anti-rabies virus immunoglobulin heavy chain variable region mRNA, partial cds.
								AY960870	Homo sapiens clone SC4119 anti-rabies virus immunoglobulin heavy chain variable region mRNA, partial cds.
								AY962565	Homo sapiens clone SC4125 anti-rabies virus immunoglobulin heavy chain variable region mRNA, partial cds.
								AY962566	Homo sapiens clone SC4133 anti-rabies virus immunoglobulin heavy chain variable region mRNA, partial cds.
								AY971013	Homo sapiens clone SC4135 anti-rabies virus immunoglobulin heavy chain variable region mRNA, partial cds.
								AY971016	Homo sapiens clone SC4136 anti-rabies virus immunoglobulin heavy chain variable region mRNA sequence.
								AY971018	Homo sapiens clone SC4137 anti-rabies virus immunoglobulin heavy chain variable region mRNA, partial cds.
								AY971023	Homo sapiens clone SC4140 anti-rabies virus immunoglobulin heavy chain variable region mRNA, partial cds.

								AY971027<br>AY971029<br>AY971032<br>AY971033<br>AY971034<br>AY971036<br>AY971037<br>AY971039<br>AY971043<br>AY971044<br>AY971045<br>AY971046<br>AY971048<br>AY971054<br>AY971055<br>AY971056<br>AY971057<br>AY971058<br>AY971059<br>AY971061<br>AY971062<br>AY971064<br>AY971072<br>AY971075<br>AY971076<br>AY971079<br>AY971080<br>AY971081<br>AY971083<br>AY971084<br>AY971085<br>AY971091<br>AY971093<br>AY971094<br>AY971097<br>AY971098<br>AY971100<br>AY971101<br>AY971106<br>AY971108<br>AY971109<br>AY971111<br>AY971112<br>AY971114<br>AY971117<br>AY971118<br>AY971119<br>AY971124<br>AY971125<br>AY971126<br>AY971127<br>AY971128<br>AY971129<br>AY971130	Homo sapiens clone SC4154 anti-rabies virus immunoglobulin heavy chain variable region mRNA, partial cds.<br>Homo sapiens clone SC4156 anti-rabies virus immunoglobulin heavy chain variable region mRNA, partial cds.<br>Homo sapiens clone SC4158 anti-rabies virus immunoglobulin heavy chain variable region mRNA, partial cds.<br>Homo sapiens clone SC4163 anti-rabies virus immunoglobulin heavy chain variable region mRNA, partial cds.<br>Homo sapiens clone CRV-CD5-1-VH immunoglobulin heavy chain variable region mRNA, partial cds.<br>Homo sapiens clone CRV-CD5-2-VH immunoglobulin heavy chain variable region mRNA, partial cds.<br>Homo sapiens clone CRV-CD5-7-VH immunoglobulin heavy chain variable region mRNA, partial cds.<br>Homo sapiens clone CRV-CD5-8-VH immunoglobulin heavy chain variable region mRNA, partial cds.<br>Homo sapiens clone CRV-CD5-9-VH immunoglobulin heavy chain variable region mRNA, partial cds.<br>Homo sapiens clone CRV-CD5-10-VH immunoglobulin heavy chain variable region mRNA, partial cds.<br>Homo sapiens clone CRV-CD5-11-VH immunoglobulin heavy chain variable region mRNA, partial cds.<br>Homo sapiens clone CRV-CD5-13-VH immunoglobulin heavy chain variable region mRNA, partial cds.<br>Homo sapiens clone CD-19-VH immunoglobulin heavy chain variable region mRNA, partial cds.<br>Homo sapiens clone CRV-CD5-20-VH immunoglobulin heavy chain variable region mRNA, partial cds.<br>Homo sapiens clone CRV-CD5-21-VH immunoglobulin heavy chain variable region mRNA, partial cds.<br>Homo sapiens clone CRV-CD5-23-VH immunoglobulin heavy chain variable region mRNA, partial cds.<br>Homo sapiens clone CRV-CD5-24-VH immunoglobulin heavy chain variable region mRNA, partial cds.<br>Homo sapiens clone CRV-CD5-25-VH immunoglobulin heavy chain variable region mRNA, partial cds.<br>Homo sapiens clone CRV-CD5-26-VH immunoglobulin heavy chain variable region mRNA, partial cds.<br>Homo sapiens clone CD-27-VH immunoglobulin heavy chain variable region mRNA, partial cds.<br>Homo sapiens clone CRV-CD5-30-VH immunoglobulin heavy chain variable region mRNA, partial cds.<br>Homo sapiens clone CRV-CD5-31-VH immunoglobulin heavy chain variable region mRNA, partial cds.<br>Homo sapiens clone CRV-CD5-32-VH immunoglobulin heavy chain variable region mRNA, partial cds.<br>Homo sapiens clone CRV-CD5-33-VH immunoglobulin heavy chain variable region mRNA, partial cds.<br>Homo sapiens clone CRV-CD5-35-VH immunoglobulin heavy chain variable region mRNA, partial cds.<br>Homo sapiens clone CRV-CD5-36-VH immunoglobulin heavy chain variable region mRNA, partial cds.<br>Homo sapiens clone CRV-CD5-37-VH immunoglobulin heavy chain variable region mRNA, partial cds.<br>Homo sapiens clone CRV-CD5-38-VH immunoglobulin heavy chain variable region mRNA, partial cds.<br>Homo sapiens clone plaque 3.4-15 anti-oxLDL immunoglobulin heavy chain variable region mRNA, partial cds.<br>Homo sapiens isolate FLpat5 clone 2 immunoglobuliln heavy chain variable region mRNA, partial cds.<br>Homo sapiens clone plaque 3.4-46 anti-oxidized LDL immunoglobulin heavy chain variable region mRNA, partial cds.<br>Homo sapiens isolate VCVHb anti-platelet immunoglobulin heavy chain mRNA, partial cds.<br>Homo sapiens clone plaque 15.16-146 anti-oxLDL immunoglobulin heavy chain variable region mRNA, partial cds.<br>Homo sapiens isolate FLpat2 clone 2 immunoglobulin heavy chain variable region mRNA, partial cds.<br>Homo sapiens isolate FLpat2 clone 1.1.2 immunoglobulin heavy chain variable region mRNA, partial cds.<br>Homo sapiens clone H-oxLDL-3 anti-oxLDL immunoglobulin heavy chain variable region mRNA, partial cds.<br>Homo sapiens clone H-oxLDL-124 anti-oxLDL immunoglobulin heavy chain variable region mRNA, partial cds.<br>Homo sapiens isolate FLpat4 clone 1.1.2 immunoglobulin heavy chain variable region mRNA, partial cds.<br>Homo sapiens isolate FLpat4 clone 1.1.1 immunoglobulin heavy chain variable region mRNA, partial cds.<br>Homo sapiens clone 2 immunoglobulin alpha heavy chain variable region mRNA, partial cds.<br>Homo sapiens clone 5 immunoglobulin alpha heavy chain variable region mRNA, partial cds.<br>Homo sapiens clone 7 immunoglobulin alpha heavy chain variable region mRNA, partial cds.<br>Homo sapiens clone 12 immunoglobulin alpha heavy chain variable region mRNA, partial cds.<br>Homo sapiens clone 16 immunoglobulin alpha heavy chain variable region mRNA, partial cds.<br>Homo sapiens clone 18 immunoglobulin alpha heavy chain variable region mRNA, partial cds.<br>Homo sapiens clone 21 immunoglobulin alpha heavy chain variable region mRNA, partial cds.<br>Homo sapiens clone 22 immunoglobulin alpha heavy chain variable region mRNA, partial cds.<br>Homo sapiens clone 23 immunoglobulin alpha heavy chain variable region mRNA, partial cds.<br>Homo sapiens clone 25 immunoglobulin alpha heavy chain variable region mRNA, partial cds.<br>Homo sapiens clone 26 immunoglobulin alpha heavy chain variable region mRNA, partial cds.<br>Homo sapiens clone 28 immunoglobulin alpha heavy chain variable region mRNA, partial cds.<br>Homo sapiens clone 32 immunoglobulin alpha heavy chain variable region mRNA, partial cds.<br>Homo sapiens clone 33 immunoglobulin alpha heavy chain variable region mRNA, partial cds.<br>Homo sapiens clone 34 immunoglobulin alpha heavy chain variable region mRNA, partial cds.

								AY971132<br>AY971133<br>AY971134<br>AY971135<br>AY971138<br>AY971140<br>AY971141<br>AY971142<br>AY986390<br>AY986395<br>AY986400<br>AY986402<br>AY994080<br>AY998716<br>AY998723<br>AY998724<br>AY998731<br>AY998750<br>BC003657<br>BC005951<br>BC006180<br>BC006402<br>BC009851<br>BC011857<br>BC014258<br>BC014667<br>BC015760<br>BC016369<br>BC016381<br>BC017356<br>BC018747<br>BC019337<br>BC020240<br>BC021276<br>BC024289<br>BC025314<br>BC025985<br>BC026038<br>BC032249<br>BC033178<br>BC037361<br>BC041037<br>BC042994<br>BC051328<br>BC053984<br>BC062335<br>BC062336<br>BC062358<br>BC063384<br>BC064496<br>BC065733<br>BC065820<br>BC066594<br>BC066642	Homo sapiens clone 35 immunoglobulin alpha heavy chain variable region mRNA, partial cds.<br>Homo sapiens clone 37 immunoglobulin alpha heavy chain variable region mRNA, partial cds.<br>Homo sapiens clone 43 immunoglobulin alpha heavy chain variable region mRNA, partial cds.<br>Homo sapiens clone 44 immunoglobulin alpha heavy chain variable region mRNA, partial cds.<br>Homo sapiens clone 45 immunoglobulin alpha heavy chain variable region mRNA, partial cds.<br>Homo sapiens clone 46 immunoglobulin alpha heavy chain variable region mRNA, partial cds.<br>Homo sapiens clone 47 immunoglobulin alpha heavy chain variable region mRNA, partial cds.<br>Homo sapiens clone 48 immunoglobulin alpha heavy chain variable region mRNA, partial cds.<br>Homo sapiens clone 50 immunoglobulin alpha heavy chain variable region mRNA, partial cds.<br>Homo sapiens clone 51 immunoglobulin alpha heavy chain variable region mRNA, partial cds.<br>Homo sapiens clone 53 immunoglobulin alpha heavy chain variable region mRNA, partial cds.<br>Homo sapiens clone 61 immunoglobulin alpha heavy chain variable region mRNA, partial cds.<br>Homo sapiens clone 64 immunoglobulin alpha heavy chain variable region mRNA, partial cds.<br>Homo sapiens clone 65 immunoglobulin alpha heavy chain variable region mRNA, partial cds.<br>Homo sapiens clone 68 immunoglobulin alpha heavy chain variable region mRNA, partial cds.<br>Homo sapiens clone 69 immunoglobulin alpha heavy chain variable region mRNA, partial cds.<br>Homo sapiens clone 70 immunoglobulin alpha heavy chain variable region mRNA, partial cds.<br>Homo sapiens clone 72 immunoglobulin alpha heavy chain variable region mRNA, partial cds.<br>Homo sapiens clone 73 immunoglobulin alpha heavy chain variable region mRNA, partial cds.<br>Homo sapiens clone 74 immunoglobulin alpha heavy chain variable region mRNA, partial cds.<br>Homo sapiens clone 80 immunoglobulin alpha heavy chain variable region mRNA, partial cds.<br>Homo sapiens clone 82 immunoglobulin alpha heavy chain variable region mRNA, partial cds.<br>Homo sapiens clone 83 immunoglobulin alpha heavy chain variable region mRNA, partial cds.<br>Homo sapiens clone 86 immunoglobulin alpha heavy chain variable region mRNA, partial cds.<br>Homo sapiens clone 87 immunoglobulin alpha heavy chain variable region mRNA, partial cds.<br>Homo sapiens clone 89 immunoglobulin alpha heavy chain variable region mRNA, partial cds.<br>Homo sapiens clone 90 immunoglobulin alpha heavy chain variable region mRNA, partial cds.<br>Homo sapiens clone 95 immunoglobulin alpha heavy chain variable region mRNA, partial cds.<br>Homo sapiens clone 97 immunoglobulin alpha heavy chain variable region mRNA, partial cds.<br>Homo sapiens clone 98 immunoglobulin alpha heavy chain variable region mRNA, partial cds.<br>Homo sapiens clone 100 immunoglobulin alpha heavy chain variable region mRNA, partial cds.<br>Homo sapiens clone 101 immunoglobulin alpha heavy chain variable region mRNA, partial cds.<br>Homo sapiens clone 103 immunoglobulin alpha heavy chain variable region mRNA, partial cds.<br>Homo sapiens clone 106 immunoglobulin alpha heavy chain variable region mRNA, partial cds.<br>Homo sapiens clone 107 immunoglobulin alpha heavy chain variable region mRNA, partial cds.<br>Homo sapiens clone 108 immunoglobulin alpha heavy chain variable region mRNA, partial cds.<br>Homo sapiens clone 113 immunoglobulin alpha heavy chain variable region mRNA, partial cds.<br>Homo sapiens clone 114 immunoglobulin alpha heavy chain variable region mRNA, partial cds.<br>Homo sapiens clone 115 immunoglobulin alpha heavy chain variable region mRNA, partial cds.<br>Homo sapiens clone 116 immunoglobulin alpha heavy chain variable region mRNA, partial cds.<br>Homo sapiens clone 117 immunoglobulin alpha heavy chain variable region mRNA, partial cds.<br>Homo sapiens clone 118 immunoglobulin alpha heavy chain variable region mRNA, partial cds.<br>Homo sapiens clone 119 immunoglobulin alpha heavy chain variable region mRNA, partial cds.<br>Homo sapiens clone 121 immunoglobulin alpha heavy chain variable region mRNA, partial cds.<br>Homo sapiens clone 122 immunoglobulin alpha heavy chain variable region mRNA, partial cds.<br>Homo sapiens clone 123 immunoglobulin alpha heavy chain variable region mRNA, partial cds.<br>Homo sapiens clone 124 immunoglobulin alpha heavy chain variable region mRNA, partial cds.<br>Homo sapiens clone 127 immunoglobulin alpha heavy chain variable region mRNA, partial cds.<br>Homo sapiens clone 129 immunoglobulin alpha heavy chain variable region mRNA, partial cds.<br>Homo sapiens clone 130 immunoglobulin alpha heavy chain variable region mRNA, partial cds.<br>Homo sapiens clone 131 immunoglobulin alpha heavy chain variable region mRNA, partial cds.<br>Homo sapiens clone D1(11) immunoglobulin heavy chain mRNA, partial cds.<br>Homo sapiens clone G2S1 immunoglobulin heavy chain mRNA, partial cds.<br>Homo sapiens clone G20 immunoglobulin heavy chain mRNA, partial cds.

								BC069016<br>BC069020<br>BC070333<br>BC072419<br>BC073758<br>BC073765<br>BC073766<br>BC073771<br>BC073782<br>BC073789<br>BC075840<br>BC075842<br>BC075846<br>BC078670<br>BC078671<br>BC080557<br>BC087841<br>BC089412<br>BC089417<br>BC089421<br>BC090938<br>BC090939<br>BC090940<br>BC092449<br>BC092518<br>BC111019<br>BC113815<br>DQ001065<br>DQ001066<br>DQ003033<br>DQ005254<br>DQ005255<br>DQ005257<br>DQ005259<br>DQ005260<br>DQ005261<br>DQ005264<br>DQ005265<br>DQ005267<br>DQ005273<br>DQ005274<br>DQ005275<br>DQ005276<br>DQ005282<br>DQ005284<br>DQ005285<br>DQ005287<br>DQ005288<br>DQ005289<br>DQ005293<br>DQ005294<br>DQ005298<br>DQ005305<br>DQ021409	Homo sapiens clone G35 immunoglobulin heavy chain mRNA, partial cds.<br>Homo sapiens MM4 immunoglobulin heavy chain variable region (IgH) mRNA, partial cds.<br>Homo sapiens isolate 12H immunoglobulin heavy chain variable region (IGHV4) mRNA, IGHV4-59*01 allele, partial cds.<br>Homo sapiens isolate 16H immunoglobulin heavy chain variable region (IGHV4) mRNA, IGHV4-39*01 allele, partial cds.<br>Homo sapiens isolate 3H immunoglobulin heavy chain variable region (IGHV1) mRNA, IGHV1-18*01 allele, partial cds.<br>Homo sapiens isolate 1H immunoglobulin heavy chain variable region (IGHV4) mRNA, IGHV4-39*01 allele, partial cds.<br>Homo sapiens isolate 4H immunoglobulin heavy chain variable region (IGHV1) mRNA, IGHV1-2*04 allele, partial cds.<br>Homo sapiens cDNA clone IMAGE:3543357, partial cds.<br>Homo sapiens immunoglobulin alpha 1 heavy chain constant region, mRNA (cDNA clone MGC:14588 IMAGE:4249174), complete cds.<br>Homo sapiens immunoglobulin heavy constant mu, mRNA (cDNA clone MGC:1905 IMAGE:3528484), complete cds.<br>Homo sapiens immunoglobulin heavy constant gamma 1 (G1m marker), mRNA (cDNA clone MGC:12848 IMAGE:4308411), complete cds.<br>Homo sapiens immunoglobulin heavy constant mu, mRNA (cDNA clone MGC:15420 IMAGE:4054579), complete cds.<br>Homo sapiens immunoglobulin heavy constant mu, mRNA (cDNA clone MGC:20337 IMAGE:4309235), complete cds.<br>Homo sapiens immunoglobulin heavy constant gamma 1 (G1m marker), mRNA (cDNA clone MGC:20688 IMAGE:4764579), complete cds.<br>Homo sapiens immunoglobulin heavy constant gamma 1 (G1m marker), mRNA (cDNA clone MGC:23153 IMAGE:4850078), complete cds.<br>Homo sapiens immunoglobulin heavy constant mu, mRNA (cDNA clone MGC:23134 IMAGE:4878806), complete cds.<br>Homo sapiens immunoglobulin alpha 1 heavy chain constant region, mRNA (cDNA clone MGC:27165 IMAGE:4701069), complete cds.<br>Homo sapiens cDNA clone MGC:27378 IMAGE:4688865, complete cds.<br>Homo sapiens immunoglobulin heavy constant mu, mRNA (cDNA clone MGC:29722 IMAGE:5095131), complete cds.<br>Homo sapiens immunoglobulin heavy constant gamma 1 (G1m marker), mRNA (cDNA clone MGC:31937 IMAGE:4851063), complete cds.<br>Homo sapiens immunoglobulin heavy constant gamma 1 (G1m marker), mRNA (cDNA clone MGC:12853 IMAGE:4054679), complete cds.<br>Homo sapiens immunoglobulin heavy constant mu, mRNA (cDNA clone MGC:31973 IMAGE:4309253), complete cds.<br>Homo sapiens immunoglobulin heavy constant delta, mRNA (cDNA clone MGC:29633 IMAGE:4855067), complete cds.<br>Homo sapiens cDNA clone MGC:39273 IMAGE:5440834, complete cds.<br>Homo sapiens immunoglobulin heavy constant gamma 1 (G1m marker), mRNA (cDNA clone MGC:39308 IMAGE:5450493), complete cds.<br>Homo sapiens immunoglobulin heavy constant gamma 4 (G4m marker), mRNA (cDNA clone MGC:22866 IMAGE:4042623), complete cds.<br>Homo sapiens immunoglobulin heavy constant gamma 1 (G1m marker), mRNA (cDNA clone MGC:39303 IMAGE:5440392), complete cds.<br>Homo sapiens immunoglobulin alpha 1 heavy chain constant region, mRNA (cDNA clone MGC:40072 IMAGE:5217556), complete cds.<br>Homo sapiens immunoglobulin heavy constant gamma 3 (G3m marker), mRNA (cDNA clone MGC:45809 IMAGE:4566267), complete cds.<br>Homo sapiens immunoglobulin heavy constant gamma 1 (G1m marker), mRNA (cDNA clone MGC:45938 IMAGE:5440797), complete cds.<br>Homo sapiens immunoglobulin heavy constant mu, mRNA (cDNA clone MGC:52291 IMAGE:4765763), complete cds.<br>Homo sapiens cDNA clone IMAGE:5296752.<br>Homo sapiens immunoglobulin heavy constant gamma 1 (G1m marker), mRNA (cDNA clone MGC:59934 IMAGE:6278990), complete cds.<br>Homo sapiens anti-rabies SO57 immunoglobulin heavy chain, mRNA (cDNA clone MGC:59926 IMAGE:5480266), complete cds.<br>Homo sapiens cDNA clone MGC:71314 IMAGE:6281452, complete cds.<br>Homo sapiens immunoglobulin heavy constant gamma 1 (G1m marker), mRNA (cDNA clone MGC:71315 IMAGE:6300554), complete cds.<br>Homo sapiens cDNA clone IMAGE:5087972, partial cds.<br>Homo sapiens immunoglobulin heavy constant delta, mRNA (cDNA clone MGC:71262 IMAGE:4764956), complete cds.

								DQ065616<br>DQ065617<br>DQ065626<br>DQ065629<br>DQ065630<br>DQ065631<br>DQ065632<br>DQ065633<br>DQ065634<br>DQ065639<br>DQ065641<br>DQ065646<br>DQ065648<br>DQ065649<br>DQ065651<br>DQ065653<br>DQ065657<br>DQ065662<br>DQ065666<br>DQ065674<br>DQ065679<br>DQ065680<br>DQ065689<br>DQ065691<br>DQ065692<br>DQ065693<br>DQ065695<br>DQ065696<br>DQ065697<br>DQ065700<br>DQ065704<br>DQ065707<br>DQ065708<br>DQ065711<br>DQ065714<br>DQ065716<br>DQ065721<br>DQ065722<br>DQ065723<br>DQ065725<br>DQ065728<br>DQ083946<br>DQ097894<br>DQ097896<br>DQ097897<br>DQ097898<br>DQ097899<br>DQ097900<br>DQ100660<br>DQ100662<br>DQ100669<br>DQ100677<br>DQ100679<br>DQ100687	Homo sapiens immunoglobulin heavy constant gamma 1 (G1m marker), mRNA (cDNA clone MGC:71313 IMAGE:6280923), complete cds.<br>Homo sapiens cDNA clone MGC:72071 IMAGE:30349896, complete cds.<br>Homo sapiens cDNA clone MGC:75197 IMAGE:6178926, complete cds.<br>Homo sapiens cDNA clone MGC:70893 IMAGE:5756125, complete cds.<br>Homo sapiens immunoglobulin heavy constant gamma 1 (G1m marker), mRNA (cDNA clone MGC:71306 IMAGE:5451018), complete cds.<br>Homo sapiens immunoglobulin heavy constant gamma 1 (G1m marker), mRNA (cDNA clone MGC:78595 IMAGE:6302910), complete cds.<br>Homo sapiens immunoglobulin heavy constant gamma 1 (G1m marker), mRNA (cDNA clone MGC:78608 IMAGE:6214622), complete cds.<br>Homo sapiens immunoglobulin heavy variable 1-69, mRNA (cDNA clone MGC:88338 IMAGE:6557369), complete cds.<br>Homo sapiens immunoglobulin heavy constant gamma 1 (G1m marker), mRNA (cDNA clone MGC:88064 IMAGE:6182140), complete cds.<br>Homo sapiens immunoglobulin heavy constant mu, mRNA (cDNA clone MGC:88764 IMAGE:4878851), complete cds.<br>Homo sapiens immunoglobulin heavy constant alpha 2 (A2m marker), mRNA (cDNA clone MGC:88772 IMAGE:4765168), complete cds.<br>Homo sapiens anti-rabies SO57 immunoglobulin heavy chain, mRNA (cDNA clone MGC:88774 IMAGE:4855124), complete cds.<br>Homo sapiens immunoglobulin heavy constant alpha 1, mRNA (cDNA clone MGC:88780 IMAGE:6279126), complete cds.<br>Homo sapiens cDNA clone MGC:88796 IMAGE:6295732, complete cds.<br>Homo sapiens immunoglobulin heavy constant gamma 1 (G1m marker), mRNA (cDNA clone MGC:88807 IMAGE:5480220), complete cds.<br>Homo sapiens immunoglobulin heavy constant gamma 1 (G1m marker), mRNA (cDNA clone MGC:88762 IMAGE:4575935), complete cds.<br>Homo sapiens immunoglobulin heavy constant gamma 1 (G1m marker), mRNA (cDNA clone MGC:88778 IMAGE:6215815), complete cds.<br>Homo sapiens immunoglobulin heavy constant gamma 1 (G1m marker), mRNA (cDNA clone MGC:88812 IMAGE:6281279), complete cds.<br>Homo sapiens immunoglobulin heavy constant gamma 1 (G1m marker), mRNA (cDNA clone MGC:88797 IMAGE:6295788), complete cds.<br>Homo sapiens immunoglobulin heavy constant gamma 1 (G1m marker), mRNA (cDNA clone MGC:88811 IMAGE:6281248), complete cds.<br>Homo sapiens immunoglobulin heavy constant gamma 1 (G1m marker), mRNA (cDNA clone MGC:99684 IMAGE:6302293), complete cds.<br>Homo sapiens immunoglobulin alpha 1 heavy chain constant region, mRNA (cDNA clone MGC:102857 IMAGE:6183642), complete cds.<br>Homo sapiens cDNA clone MGC:104996 IMAGE:3054299, complete cds.<br>Homo sapiens immunoglobulin heavy constant gamma 1 (G1m marker), mRNA (cDNA clone MGC:105003 IMAGE:3054816), complete cds.<br>Homo sapiens cDNA clone MGC:105008 IMAGE:3055541, complete cds.<br>Homo sapiens immunoglobulin heavy constant gamma 1 (G1m marker), mRNA (cDNA clone MGC:104997 IMAGE:3057210), complete cds.<br>Homo sapiens immunoglobulin heavy constant gamma 1 (G1m marker), mRNA (cDNA clone IMAGE:3056020), partial cds.<br>Homo sapiens immunoglobulin heavy constant gamma 1 (G1m marker), mRNA (cDNA clone MGC:105011 IMAGE:3059913), complete cds.<br>Homo sapiens immunoglobulin heavy constant alpha 1, mRNA (cDNA clone MGC:104433 IMAGE:30330298), complete cds.<br>Homo sapiens immunoglobulin heavy constant gamma 1 (G1m marker), mRNA (cDNA clone MGC:105004 IMAGE:3056327), complete cds.<br>Homo sapiens immunoglobulin heavy constant gamma 4 (G4m marker), mRNA (cDNA clone MGC:117419 IMAGE:5211387), complete cds.<br>Homo sapiens cDNA clone IMAGE:40022581.<br>Homo sapiens isolate MM37 immunoglobulin heavy chain variable region mRNA, partial cds.<br>Homo sapiens isolate MM38 immunoglobulin heavy chain variable region mRNA, partial cds.

								DQ100692	Homo sapiens desmoglein 3-specific antibody heavy chain variable region mRNA, partial cds.
								DQ100693	Homo sapiens isolate C3 HF1 allergic immunoglobulin epsilon heavy chain variable region mRNA, partial cds.
								DQ100695	Homo sapiens isolate C16 HF1 allergic immunoglobulin epsilon heavy chain variable region mRNA, partial cds.
								DQ100699	Homo sapiens isolate E9 HF1 allergic immunoglobulin epsilon heavy chain variable region mRNA, partial cds.
								DQ100700	Homo sapiens isolate L7 HF1 allergic immunoglobulin epsilon heavy chain variable region mRNA, partial cds.
								DQ100701	Homo sapiens isolate A15 HF1 allergic immunoglobulin epsilon heavy chain variable region mRNA, partial cds.
								DQ100708	Homo sapiens isolate L24 HF1 allergic immunoglobulin epsilon heavy chain variable region mRNA, partial cds.
								DQ100710	Homo sapiens isolate C10 HF1 allergic immunoglobulin epsilon heavy chain variable region mRNA, partial cds.
								DQ100712	Homo sapiens isolate A2 HF1 allergic immunoglobulin epsilon heavy chain variable region mRNA, partial cds.
								DQ100713	Homo sapiens isolate L13 HF1 allergic immunoglobulin epsilon heavy chain variable region mRNA, partial cds.
								DQ100714	Homo sapiens isolate K14 AF3 non-allergic immunoglobulin epsilon heavy chain variable region mRNA, partial cds.
								DQ100717	Homo sapiens isolate K15 AF3 non-allergic immunoglobulin epsilon heavy chain variable region mRNA, partial cds.
								DQ100722	Homo sapiens isolate K22 AF3 non-allergic immunoglobulin epsilon heavy chain variable region mRNA, partial cds.
								DQ100725	Homo sapiens isolate K23 AF3 non-allergic immunoglobulin epsilon heavy chain variable region mRNA, partial cds.
								DQ100728	Homo sapiens isolate K42 AF3 non-allergic immunoglobulin epsilon heavy chain variable region mRNA, partial cds.
								DQ100733	Homo sapiens isolate K65 AF3 non-allergic immunoglobulin epsilon heavy chain variable region mRNA, partial cds.
								DQ100743	Homo sapiens isolate K66 AF3 non-allergic immunoglobulin epsilon heavy chain variable region mRNA, partial cds.
								DQ100747	Homo sapiens isolate K84 AF3 non-allergic immunoglobulin epsilon heavy chain variable region mRNA, partial cds.
								DQ100748	Homo sapiens isolate K88 AF3 non-allergic immunoglobulin epsilon heavy chain variable region mRNA, partial cds.
								DQ100752	Homo sapiens isolate K89 AF3 non-allergic immunoglobulin epsilon heavy chain variable region mRNA, partial cds.
								DQ100756	Homo sapiens isolate C1 MK2 non-allergic immunoglobulin epsilon heavy chain variable region mRNA, partial cds.
								DQ100760	Homo sapiens isolate L4 AF4 non-allergic immunoglobulin epsilon heavy chain variable region mRNA, partial cds.
								DQ100762	Homo sapiens isolate L30 AF4 non-allergic immunoglobulin epsilon heavy chain variable region mRNA, partial cds.
								DQ100772	Homo sapiens isolate L76 AF4 non-allergic immunoglobulin epsilon heavy chain variable region mRNA, partial cds.
								DQ100773	Homo sapiens clone TTP-A16 immunoglobulin heavy chain variable region mRNA, partial cds.
								DQ100775	Homo sapiens clone A4-97 immunoglobulin epsilon heavy chain variable region mRNA, partial cds.
								DQ100776	Homo sapiens clone A4-96 immunoglobulin epsilon heavy chain variable region mRNA, partial cds.
								DQ100778	Homo sapiens clone A4-64 immunoglobulin epsilon heavy chain variable region mRNA, partial cds.
								DQ100785	Homo sapiens clone A4-58 immunoglobulin epsilon heavy chain variable region mRNA, partial cds.
								DQ100786	Homo sapiens clone A4-57 immunoglobulin epsilon heavy chain variable region mRNA, partial cds.
								DQ100790	Homo sapiens clone A4-56 immunoglobulin epsilon heavy chain variable region mRNA, partial cds.
								DQ100791	Homo sapiens clone A4-55 immunoglobulin epsilon heavy chain variable region mRNA, partial cds.
								DQ100794	Homo sapiens clone A4-54 immunoglobulin epsilon heavy chain variable region mRNA, partial cds.
								DQ100795	Homo sapiens clone A4-53 immunoglobulin epsilon heavy chain variable region mRNA, partial cds.
								DQ100796	Homo sapiens clone A4-44 immunoglobulin epsilon heavy chain variable region mRNA, partial cds.
								DQ100799	Homo sapiens clone A4-42 immunoglobulin epsilon heavy chain variable region mRNA, partial cds.
								DQ100802	Homo sapiens clone A4-158 immunoglobulin epsilon heavy chain variable region mRNA, partial cds.
								DQ100803	Homo sapiens clone A4-155 immunoglobulin epsilon heavy chain variable region mRNA, partial cds.
								DQ100810	Homo sapiens clone A4-154 immunoglobulin epsilon heavy chain variable region-like mRNA, partial sequence.
								DQ100815	Homo sapiens clone A4-149 immunoglobulin epsilon heavy chain variable region mRNA, partial cds.
								DQ100816	Homo sapiens clone A4-147 immunoglobulin epsilon heavy chain variable region mRNA, partial cds.
								DQ100817	Homo sapiens clone A4-141 immunoglobulin epsilon heavy chain variable region mRNA, partial cds.
								DQ100819	Homo sapiens clone A4-EQEA16 immunoglobulin epsilon heavy chain variable region mRNA, partial cds.
								DQ100823	Homo sapiens clone A3-EQEA2 immunoglobulin epsilon heavy chain variable region mRNA, partial cds.
								DQ100824	Homo sapiens clone A3-EQCA21 immunoglobulin epsilon heavy chain variable region mRNA, partial cds.
								DQ100826	Homo sapiens clone A3-EQCA14 immunoglobulin epsilon heavy chain variable region mRNA, partial cds.
								DQ100827	Homo sapiens clone A3-EQCA12 immunoglobulin epsilon heavy chain variable region mRNA, partial cds.
								DQ100828	Homo sapiens clone A3-EE73 immunoglobulin epsilon heavy chain variable region mRNA, partial cds.
								DQ100831	Homo sapiens clone A3-EE71 immunoglobulin epsilon heavy chain variable region mRNA, partial cds.
								DQ100833	Homo sapiens clone A3-EE70 immunoglobulin epsilon heavy chain variable region mRNA, partial cds.
								DQ100834	Homo sapiens clone A3-EE7 immunoglobulin epsilon heavy chain variable region mRNA, partial cds.
								DQ100838	Homo sapiens clone A3-EE65 immunoglobulin epsilon heavy chain variable region mRNA, partial cds.
								DQ100841	Homo sapiens clone A3-EE64 immunoglobulin epsilon heavy chain variable region mRNA, partial cds.
								DQ100843	Homo sapiens clone A3-EE6 immunoglobulin epsilon heavy chain variable region mRNA, partial cds.

								DQ100848<br>DQ100851<br>DQ100854<br>DQ100859<br>DQ100860<br>DQ100867<br>DQ100870<br>DQ100871<br>DQ100876<br>DQ100883<br>DQ100886<br>DQ100887<br>DQ100888<br>DQ100889<br>DQ100891<br>DQ100892<br>DQ100893<br>DQ100894<br>DQ100895<br>DQ100896<br>DQ100897<br>DQ100898<br>DQ100899<br>DQ100901<br>DQ100902<br>DQ100903<br>DQ100905<br>DQ100907<br>DQ100909<br>DQ100911<br>DQ100912<br>DQ100914<br>DQ100915<br>DQ100917<br>DQ100918<br>DQ100919<br>DQ100921<br>DQ100922<br>DQ100923<br>DQ100926<br>DQ100928<br>DQ100930<br>DQ100948<br>DQ100949<br>DQ100953<br>DQ100963<br>DQ100965<br>DQ100968<br>DQ100970<br>DQ100974<br>DQ100975<br>DQ100982<br>DQ100983<br>DQ100985	Homo sapiens clone A3-EE52 immunoglobulin epsilon heavy chain variable region mRNA, partial cds.<br>Homo sapiens clone A3-EE49 immunoglobulin epsilon heavy chain variable region mRNA, partial cds.<br>Homo sapiens clone A3-EE44B immunoglobulin epsilon heavy chain variable region mRNA, partial cds.<br>Homo sapiens clone A3-EE43 immunoglobulin epsilon heavy chain variable region mRNA, partial cds.<br>Homo sapiens clone A3-EE37 immunoglobulin epsilon heavy chain variable region mRNA, partial cds.<br>Homo sapiens clone A3-EE30 immunoglobulin epsilon heavy chain variable region mRNA, partial cds.<br>Homo sapiens clone A3-EE28 immunoglobulin epsilon heavy chain variable region mRNA, partial cds.<br>Homo sapiens clone A3-EE19 immunoglobulin epsilon heavy chain variable region mRNA, partial cds.<br>Homo sapiens clone A3-EE18 immunoglobulin epsilon heavy chain variable region mRNA, partial cds.<br>Homo sapiens clone A3-EE17 immunoglobulin epsilon heavy chain variable region mRNA, partial cds.<br>Homo sapiens clone A3-EE13 immunoglobulin epsilon heavy chain variable region mRNA, partial cds.<br>Homo sapiens clone A3-EE1 immunoglobulin epsilon heavy chain variable region mRNA, partial cds.<br>Homo sapiens immunoglobulin heavy chain Ialpha/Cgamma fusion switch-circle transcript mRNA, partial sequence.<br>Homo sapiens clone F1P3C3 immunoglobulin heavy chain variable region mRNA, partial cds.<br>Homo sapiens clone F6P2F3 immunoglobulin heavy chain variable region mRNA, partial cds.<br>Homo sapiens clone F5P1G11 immunoglobulin heavy chain variable region mRNA, partial cds.<br>Homo sapiens clone F6P1F11 immunoglobulin heavy chain variable region mRNA, partial cds.<br>Homo sapiens clone F6P5D11 immunoglobulin heavy chain variable region mRNA, partial cds.<br>Homo sapiens clone F7P1G3 immunoglobulin heavy chain variable region mRNA, partial cds.<br>Homo sapiens isolate N1200H immunoglobulin heavy chain variable region (IGHV4-34) mRNA, IGHV4-34*01 allele, partial cds.<br>Homo sapiens isolate N1214H immunoglobulin heavy chain variable region (IGHV1-2) mRNA, IGHV1-2*02 allele, partial cds.<br>Homo sapiens isolate N1457H immunoglobulin heavy chain variable region (IGHV1-18) mRNA, IGHV1-18*01 allele, partial cds.<br>Homo sapiens isolate N1508H immunoglobulin heavy chain variable region (IGHV3-9) mRNA, IGHV3-9*01 allele, partial cds.<br>Homo sapiens isolate N1542H immunoglobulin heavy chain variable region (IGHV3-30) mRNA, IGHV3-30*01 allele, partial cds.<br>Homo sapiens isolate N1622H immunoglobulin heavy chain variable region (IGHV4-39) mRNA, IGHV4-39*01 allele, partial cds.<br>Homo sapiens isolate N1701H immunoglobulin heavy chain variable region (IGHV3-15) mRNA, IGHV3-15*02 allele, partial cds.<br>Homo sapiens isolate N1704H immunoglobulin heavy chain variable region (IGHV3-21) mRNA, IGHV3-21*01 allele, partial cds.<br>Homo sapiens isolate N1713H immunoglobulin heavy chain variable region (IGHV1-18) mRNA, IGHV1-18*01 allele, partial cds.<br>Homo sapiens isolate N1777H immunoglobulin heavy chain variable region (IGHV3-30) mRNA, IGHV3-30*18 allele, partial cds.<br>Homo sapiens isolate N1809H immunoglobulin heavy chain variable region (IGHV3-23) mRNA, IGHV3-23*01 allele, partial cds.<br>Homo sapiens isolate N1810H immunoglobulin heavy chain variable region (IGHV4-39) mRNA, IGHV4-39*01 allele, partial cds.<br>Homo sapiens isolate N2123H immunoglobulin heavy chain variable region (IGHV1-2) mRNA, IGHV1-2*04 allele, partial cds.<br>Homo sapiens isolate N2213H immunoglobulin heavy chain variable region (IGHV3-23) mRNA, IGHV3-23*01 allele, partial cds.<br>Homo sapiens isolate N2238H immunoglobulin heavy chain variable region (IGHV4-39) mRNA, IGHV4-39*01 allele, partial cds.<br>Homo sapiens isolate N2272H immunoglobulin heavy chain variable region (IGHV3-23) mRNA, IGHV3-23*01 allele, partial cds.<br>Homo sapiens isolate N23H immunoglobulin heavy chain variable region (IGHV3-21) mRNA, IGHV3-21*02 allele, partial cds.<br>Homo sapiens isolate N2325H immunoglobulin heavy chain variable region (IGHV3-23) mRNA, IGHV3-23*01 allele, partial cds.<br>Homo sapiens isolate N240H immunoglobulin heavy chain variable region (IGHV1-2) mRNA, IGHV1-2*04 allele, partial cds.<br>Homo sapiens isolate N2527H immunoglobulin heavy chain variable region (IGHV4-34) mRNA, IGHV4-34*01 allele, partial cds.

								DQ100997<br>DQ118259<br>DQ187519<br>DQ187520<br>DQ187521<br>DQ187554<br>DQ187555<br>DQ187556<br>DQ187557<br>DQ187558<br>DQ187599<br>DQ187600<br>DQ187601<br>DQ187602<br>DQ187603<br>DQ187604<br>DQ187605<br>DQ187606<br>DQ187608<br>DQ187628<br>DQ187629<br>DQ187648<br>DQ187649<br>DQ187667<br>DQ187668<br>DQ187669<br>DQ187670<br>DQ187671<br>DQ187672<br>DQ187673<br>DQ187674<br>DQ187677<br>DQ187706<br>DQ187708<br>DQ187709<br>DQ187711<br>DQ187712<br>DQ187732<br>DQ187733<br>DQ187735<br>DQ187739<br>DQ205138<br>DQ205143<br>DQ205163<br>DQ205169<br>DQ205179<br>DQ205182<br>DQ212498<br>DQ212499<br>DQ212500<br>DQ212501<br>DQ212505<br>DQ212506<br>DQ212509	Homo sapiens isolate N29H immunoglobulin heavy chain variable region (IGHV3-23) mRNA, IGHV3-23*01 allele, partial cds.<br>Homo sapiens isolate N950H immunoglobulin heavy chain variable region (IGHV1-69) mRNA, IGHV1-69*01 allele, partial cds.<br>Homo sapiens isolate P2143H immunoglobulin heavy chain variable region (IGHV3-33) mRNA, IGHV3-33*01 allele, partial cds.<br>Homo sapiens isolate P427H immunoglobulin heavy chain variable region (IGHV3-30) mRNA, IGHV3-30*18 allele, partial cds.<br>Homo sapiens isolate P1823H immunoglobulin heavy chain variable region (IGHV3-23) mRNA, IGHV3-23*01 allele, partial cds.<br>Homo sapiens isolate P1191H immunoglobulin heavy chain variable region (IGHV3-66) mRNA, IGHV3-66*04 allele, partial cds.<br>Homo sapiens isolate P1165H immunoglobulin heavy chain variable region (IGHV3-15) mRNA, IGHV3-15*01 allele, partial cds.<br>Homo sapiens isolate P409H immunoglobulin heavy chain variable region (IGHV3-23) mRNA, IGHV3-23*01 allele, partial cds.<br>Homo sapiens isolate P1157H immunoglobulin heavy chain variable region (IGHV3-30) mRNA, IGHV3-30*01 allele, partial cds.<br>Homo sapiens isolate P341H immunoglobulin heavy chain variable region (IGHV1-8) mRNA, IGHV1-8*01 allele, partial cds.<br>Homo sapiens isolate P573H immunoglobulin heavy chain variable region (IGHV3-7) mRNA, IGHV3-7*01 allele, partial cds.<br>Homo sapiens isolate P1306H immunoglobulin heavy chain variable region (IGHV3-30) mRNA, IGHV3-30*18 allele, partial cds.<br>Homo sapiens isolate P1540H immunoglobulin heavy chain variable region (IGHV4-59) mRNA, IGHV4-59*01 allele, partial cds.<br>Homo sapiens isolate P2184H immunoglobulin heavy chain variable region (IGHV3-7) mRNA, IGHV3-7*01 allele, partial cds.<br>Homo sapiens isolate P283H immunoglobulin heavy chain variable region (IGHV2-5) mRNA, IGHV2-5*10 allele, partial cds.<br>Homo sapiens isolate P1182H immunoglobulin heavy chain variable region (IGHV1-2) mRNA, IGHV1-2*02 allele, partial cds.<br>Homo sapiens isolate P1627H immunoglobulin heavy chain variable region (IGHV3-7) mRNA, IGHV3-7*01 allele, partial cds.<br>Homo sapiens isolate P1060H immunoglobulin heavy chain variable region (IGHV4-b) mRNA, IGHV4-b*02 allele, partial cds.<br>Homo sapiens isolate P1178H immunoglobulin heavy chain variable region (IGHV3-23) mRNA, IGHV3-23*01 allele, partial cds.<br>Homo sapiens isolate P323H immunoglobulin heavy chain variable region (IGHV4-59) mRNA, IGHV4-59*01 allele, partial cds.<br>Homo sapiens isolate P711H immunoglobulin heavy chain variable region (IGHV4-34) mRNA, IGHV4-34*02 allele, partial cds.<br>Homo sapiens isolate P441H immunoglobulin heavy chain variable region (IGHV3-33) mRNA, IGHV3-33*01 allele, partial cds.<br>Homo sapiens isolate P14H immunoglobulin heavy chain variable region (IGHV3-64) mRNA, IGHV3-64*05 allele, partial cds.<br>Homo sapiens isolate P1173H immunoglobulin heavy chain variable region (IGHV5-a) mRNA, IGHV5-a*01 allele, partial cds.<br>Homo sapiens isolate P324H immunoglobulin heavy chain variable region (IGHV3-23) mRNA, IGHV3-23*01 allele, partial cds.<br>Homo sapiens isolate P1215H immunoglobulin heavy chain variable region (IGHV3-21) mRNA, IGHV3-21*01 allele, partial cds.<br>Homo sapiens isolate P2033H immunoglobulin heavy chain variable region (IGHV1-2) mRNA, IGHV1-2*04 allele, partial cds.<br>Homo sapiens isolate P333H immunoglobulin heavy chain variable region (IGHV3-23) mRNA, IGHV3-23*01 allele, partial cds.<br>Homo sapiens isolate P835H immunoglobulin heavy chain variable region (IGHV3-30) mRNA, IGHV3-30*18 allele, partial cds.<br>Homo sapiens isolate P1177H immunoglobulin heavy chain variable region (IGHV1-18) mRNA, IGHV1-18*01 allele, partial cds.<br>Homo sapiens isolate P1403H immunoglobulin heavy chain variable region (IGHV4-39) mRNA, IGHV4-39*01 allele, partial cds.<br>Homo sapiens isolate P1707H immunoglobulin heavy chain variable region (IGHV1-8) mRNA, IGHV1-8*01 allele, partial cds.<br>Homo sapiens isolate P1971H immunoglobulin heavy chain variable region (IGHV3-7) mRNA, IGHV3-7*01 allele, partial cds.<br>Homo sapiens isolate P1986H immunoglobulin heavy chain variable region (IGHV1-8) mRNA, IGHV1-8*01 allele, partial cds.<br>Homo sapiens isolate P452H immunoglobulin heavy chain variable region (IGHV3-13) mRNA, IGHV3-13*01 allele, partial cds.<br>Homo sapiens isolate N1319H immunoglobulin heavy chain variable region (IGHV3-33) mRNA, IGHV3-33*01 allele, partial cds.<br>Homo sapiens isolate N1404H immunoglobulin heavy chain variable region (IGHV1-3) mRNA, IGHV1-3*01 allele, partial cds.<br>Homo sapiens isolate N1526H immunoglobulin heavy chain variable region (IGHV3-23) mRNA, IGHV3-23*01 allele, partial cds.<br>Homo sapiens isolate N1568H immunoglobulin heavy chain variable region (IGHV1-8) mRNA, IGHV1-8*01 allele, partial cds.<br>Homo sapiens isolate N1599H immunoglobulin heavy chain variable region (IGHV3-23) mRNA, IGHV3-23*01 allele, partial cds.<br>Homo sapiens isolate N1674H immunoglobulin heavy chain variable region (IGHV3-30) mRNA, IGHV3-30*18 allele, partial cds.<br>Homo sapiens isolate N1707H immunoglobulin heavy chain variable region (IGHV3-23) mRNA, IGHV3-23*01 allele, partial cds.<br>Homo sapiens isolate N1816H immunoglobulin heavy chain variable region (IGHV1-8) mRNA, IGHV1-8*01 allele, partial cds.<br>Homo sapiens isolate N2122H immunoglobulin heavy chain variable region (IGHV3-20) mRNA, IGHV3-20*01 allele, partial cds.<br>Homo sapiens isolate N2205H nonfunctional immunoglobulin heavy chain (IGHV3-23) mRNA, IGHV3-23*01 allele, partial sequence.<br>Homo sapiens isolate N248H immunoglobulin heavy chain variable region (IGHV4-61) mRNA, IGHV4-61*02 allele, partial cds.<br>Homo sapiens isolate N563H immunoglobulin heavy chain variable region (IGHV3-13) mRNA, IGHV3-13*01 allele, partial cds.

								DQ212511<br>DQ212512<br>DQ212514<br>DQ212521<br>DQ212525<br>DQ212526<br>DQ225095<br>DQ322735<br>DQ322736<br>DQ322737<br>DQ322738<br>DQ322746<br>DQ322747<br>DQ322748<br>DQ322750<br>DQ322751<br>DQ322752<br>DQ322765<br>DQ322766<br>DQ322768<br>DQ322777<br>DQ322779<br>DQ322780<br>DQ322781<br>DQ322782<br>DQ322783<br>DQ322790<br>DQ322796<br>DQ322798<br>DQ322802<br>DQ322804<br>DQ322805<br>DQ322813<br>DQ322853<br>DQ322866<br>DQ322875<br>DQ322877<br>DQ322882<br>DQ322889<br>DQ322892<br>DQ322893<br>DQ322894<br>DQ322897<br>DQ322910<br>DQ322923<br>DQ322946<br>DQ322951<br>DQ322954<br>DQ322958<br>DQ322960<br>DQ322973<br>DQ322981<br>DQ322987<br>DQ323006	Homo sapiens isolate N893H immunoglobulin heavy chain variable region (IGHV3-7) mRNA, IGHV3-7*01 allele, partial cds.<br>Homo sapiens isolate P534H immunoglobulin heavy chain variable region (IGHV4-34) mRNA, IGHV4-34*03 allele, partial cds.<br>Homo sapiens isolate P280H immunoglobulin heavy chain variable region (IGHV4-31) mRNA, IGHV4-31*01 allele, partial cds.<br>Homo sapiens isolate P450H immunoglobulin heavy chain variable region (IGHV6-1) mRNA, IGHV6-1*01 allele, partial cds.<br>Homo sapiens isolate P906H immunoglobulin heavy chain variable region (IGHV3-74) mRNA, IGHV3-74*02 allele, partial cds.<br>Homo sapiens isolate P922H immunoglobulin heavy chain variable region (IGHV3-7) mRNA, IGHV3-7*01 allele, partial cds.<br>Homo sapiens isolate P1188H immunoglobulin heavy chain variable region (IGHV3-9) mRNA, IGHV3-9*01 allele, partial cds.<br>Homo sapiens isolate P1597H immunoglobulin heavy chain variable region (IGHV6-1) mRNA, IGHV6-1*01 allele, partial cds.<br>Homo sapiens isolate P460H immunoglobulin heavy chain variable region (IGHV4-b) mRNA, IGHV4-b*01 allele, partial cds.<br>Homo sapiens isolate P1531H immunoglobulin heavy chain variable region (IGHV3-21) mRNA, IGHV3-21*01 allele, partial cds.<br>Homo sapiens isolate P458H immunoglobulin heavy chain variable region (IGHV3-11) mRNA, IGHV3-11*01 allele, partial cds.<br>Homo sapiens isolate P202H immunoglobulin heavy chain variable region (IGHV3-33) mRNA, IGHV3-33*01 allele, partial cds.<br>Homo sapiens isolate P1522H immunoglobulin heavy chain variable region (IGHV4-61) mRNA, IGHV4-61*02 allele, partial cds.<br>Homo sapiens isolate P1132H immunoglobulin heavy chain variable region (IGHV4-39) mRNA, IGHV4-39*01 allele, partial cds.<br>Homo sapiens isolate P1321H immunoglobulin heavy chain variable region (IGHV3-21) mRNA, IGHV3-21*01 allele, partial cds.<br>Homo sapiens isolate P326H immunoglobulin heavy chain variable region (IGHV3-21) mRNA, IGHV3-21*02 allele, partial cds.<br>Homo sapiens isolate P173H immunoglobulin heavy chain variable region (IGHV3-21) mRNA, IGHV3-21*01 allele, partial cds.<br>Homo sapiens isolate P1532H immunoglobulin heavy chain variable region (IGHV1-69) mRNA, IGHV1-69*01 allele, partial cds.<br>Homo sapiens isolate P104H immunoglobulin heavy chain variable region (IGHV4-39) mRNA, IGHV4-39*06 allele, partial cds.<br>Homo sapiens isolate P1346H immunoglobulin heavy chain variable region (IGHV3-48) mRNA, IGHV3-48*01 allele, partial cds.<br>Homo sapiens isolate P1529H immunoglobulin heavy chain variable region (IGHV3-30) mRNA, IGHV3-30*18 allele, partial cds.<br>Homo sapiens isolate P373H immunoglobulin heavy chain variable region (IGHV3-48) mRNA, IGHV3-48*01 allele, partial cds.<br>Homo sapiens isolate P611H immunoglobulin heavy chain variable region (IGHV3-11) mRNA, IGHV3-11*01 allele, partial cds.<br>Homo sapiens isolate P650H immunoglobulin heavy chain variable region (IGHV1-69) mRNA, IGHV1-69*01 allele, partial cds.<br>Homo sapiens isolate P788H immunoglobulin heavy chain variable region (IGHV1-2) mRNA, IGHV1-2*04 allele, partial cds.<br>Homo sapiens isolate P131H immunoglobulin heavy chain variable region (IGHV3-23) mRNA, IGHV3-23*01 allele, partial cds.<br>Homo sapiens isolate P1659H immunoglobulin heavy chain variable region (IGHV3-21) mRNA, IGHV3-21*01 allele, partial cds.<br>Homo sapiens isolate P1812H immunoglobulin heavy chain variable region (IGHV3-48) mRNA, IGHV3-48*01 allele, partial cds.<br>Homo sapiens isolate P1837H immunoglobulin heavy chain variable region (IGHV1-2) mRNA, IGHV1-2*02 allele, partial cds.<br>Homo sapiens isolate P437H immunoglobulin heavy chain variable region (IGHV4-39) mRNA, IGHV4-39*01 allele, partial cds.<br>Homo sapiens isolate P511H immunoglobulin heavy chain variable region (IGHV3-20) mRNA, IGHV3-20*01 allele, partial cds.<br>Homo sapiens isolate P630H immunoglobulin heavy chain variable region (IGHV3-15) mRNA, IGHV3-15*01 allele, partial cds.<br>Homo sapiens isolate P451H immunoglobulin heavy chain variable region (IGHV3-30-3) mRNA, IGHV3-30-3*01 allele, partial<br>cds.<br>Homo sapiens isolate P1678H immunoglobulin heavy chain variable region (IGHV4-59) mRNA, IGHV4-59*01 allele, partial cds.<br>Homo sapiens isolate P790H immunoglobulin heavy chain variable region (IGHV3-30) mRNA, IGHV3-30*18 allele, partial cds.<br>Homo sapiens isolate P1337H immunoglobulin heavy chain variable region (IGHV1-24) mRNA, IGHV1-24*01 allele, partial cds.<br>Homo sapiens isolate P1484H immunoglobulin heavy chain variable region (IGHV3-21) mRNA, IGHV3-21*01 allele, partial cds.<br>Homo sapiens isolate P196H immunoglobulin heavy chain variable region (IGHV4-39) mRNA, IGHV4-39*01 allele, partial cds.<br>Homo sapiens isolate P225H immunoglobulin heavy chain variable region (IGHV2-5) mRNA, IGHV2-5*10 allele, partial cds.<br>Homo sapiens isolate P247H immunoglobulin heavy chain variable region (IGHV3-30-3) mRNA, IGHV3-30-3*01 allele, partial<br>cds.<br>Homo sapiens isolate P86H immunoglobulin heavy chain variable region (IGHV3-7) mRNA, IGHV3-7*01 allele, partial cds.<br>Homo sapiens isolate PMM34H immunoglobulin heavy chain variable region (IGHV4-4) mRNA, IGHV4-4*07 allele, partial cds.<br>Homo sapiens isolate N1784H immunoglobulin heavy chain variable region (IGHV1-24) mRNA, IGHV1-24*01 allele, partial<br>cds.<br>Homo sapiens isolate N228H immunoglobulin heavy chain variable region (IGHV3-15) mRNA, IGHV3-15*01 allele, partial cds.<br>Homo sapiens isolate N314H immunoglobulin heavy chain variable region (IGHV1-2) mRNA, IGHV1-2*02 allele, partial cds.<br>Homo sapiens isolate N328H immunoglobulin heavy chain variable region (IGHV3-21) mRNA, IGHV3-21*01 allele, partial cds.<br>Homo sapiens isolate N469H immunoglobulin heavy chain variable region (IGHV2-26) mRNA, IGHV2-26*01 allele, partial cds.<br>Homo sapiens isolate P250H immunoglobulin heavy chain variable region (IGHV6-1) mRNA, IGHV6-1*01 allele, partial cds.<br>Homo sapiens isolate BL49 immunoglobulin heavy chain VDJ region mRNA, partial cds.<br>Homo sapiens donor 1 serotype PPS14 clone 19 immunoglobulin heavy chain variable region mRNA, partial cds.<br>Homo sapiens donor 1 serotype PPS14 clone 2 immunoglobulin heavy chain variable region mRNA, partial cds.

								DQ323028	Homo sapiens donor 1 serotype PPS4 clone 3 immunoglobulin light chain variable region mRNA, partial cds.
								DQ323029	Homo sapiens donor 2 serotype PPS14 clone 1 immunoglobulin heavy chain variable region mRNA, partial cds.
								DQ328866	Homo sapiens donor 2 serotype PPS14 clone 14 immunoglobulin heavy chain variable region mRNA, partial cds.
								DQ328872	Homo sapiens donor 2 serotype PPS14 clone 16 immunoglobulin heavy chain variable region mRNA, partial cds.
								DQ379471	Homo sapiens donor 2 serotype PPS14 clone 5 immunoglobulin heavy chain variable region mRNA, partial cds.
								DQ379473	Homo sapiens donor 2 serotype PPS4 clone 19 immunoglobulin heavy chain variable region mRNA, partial cds.
								DQ379475	Homo sapiens donor 3 serotype PPS14 clone 16 immunoglobulin heavy chain variable region mRNA, partial cds.
								DQ431482	Homo sapiens donor 3 serotype PPS14 clone 8 immunoglobulin heavy chain variable region mRNA, partial cds.
								DQ431484	Homo sapiens donor 3 serotype PPS4 clone 33 immunoglobulin heavy chain variable region mRNA, partial cds.
								DQ431490	Homo sapiens donor 3 serotype PPS4 clone 9 immunoglobulin heavy chain variable region mRNA, partial cds.
								DQ431497	Homo sapiens donor 3 serotype PPS14 clone 30 immunoglobulin heavy chain variable region mRNA, partial cds.
								DQ431505	Homo sapiens donor 3 serotype PPS14 clone 36 immunoglobulin heavy chain variable region mRNA, partial cds.
								DQ431506	Homo sapiens donor 3 serotype PPS14 clone 79 immunoglobulin heavy chain variable region mRNA, partial cds.
								DQ431510	Homo sapiens donor 3 serotype PPS4 clone 12 immunoglobulin heavy chain variable region mRNA, partial cds.
								DQ431548	Homo sapiens donor 3 serotype PPS4 clone 69 immunoglobulin heavy chain variable region mRNA, partial cds.
								DQ431563	Homo sapiens donor 4 serotype PPS14 clone 15 immunoglobulin heavy chain variable region mRNA, partial cds.
								DQ431572	Homo sapiens donor 4 serotype PPS4 clone 13 immunoglobulin heavy chain variable region mRNA, partial cds.
								DQ431573	Homo sapiens donor 5 serotype PPS14 clone 5 immunoglobulin heavy chain variable region mRNA, partial cds.
								DQ431575	Homo sapiens donor 5 serotype PPS14 clone 6 immunoglobulin heavy chain variable region mRNA, partial cds.
								DQ431578	Homo sapiens donor 6 serotype PPS14 clone 54 immunoglobulin heavy chain variable region mRNA, partial cds.
								DQ431582	Homo sapiens donor 6 serotype PPS14 clone 58 immunoglobulin heavy chain variable region mRNA, partial cds.
								DQ431583	Homo sapiens donor 6 serotype PPS4 clone 15 immunoglobulin heavy chain variable region mRNA, partial cds.
								DQ431584	Homo sapiens donor 6 serotype PPS4 clone 20 immunoglobulin heavy chain variable region mRNA, partial cds.
								DQ431587	Homo sapiens donor 6 serotype PPS4 clone 3 immunoglobulin heavy chain variable region mRNA, partial cds.
								DQ431591	Homo sapiens donor 6 serotype PPS14 clone 79 immunoglobulin heavy chain variable region mRNA, partial cds.
								DQ431596	Homo sapiens donor 6 serotype PPS14 clone 81 immunoglobulin heavy chain variable region mRNA, partial cds.
								DQ431598	Homo sapiens donor 6 serotype PPS14 clone 83 immunoglobulin heavy chain variable region mRNA, partial cds.
								DQ431602	Homo sapiens donor 6 serotype PPS4 clone 3 immunoglobulin heavy chain variable region mRNA, partial cds.
								DQ431603	Homo sapiens donor 7 serotype PPS4 clone 10 immunoglobulin heavy chain variable region mRNA, partial cds.
								DQ431605	Homo sapiens donor 7 serotype PPS4 clone 9 immunoglobulin heavy chain variable region mRNA, partial cds.
								DQ431606	Homo sapiens donor 7 serotype PPS14 clone 64 immunoglobulin heavy chain variable region mRNA, partial cds.
								DQ431607	Homo sapiens donor 7 serotype PPS14 clone 81 immunoglobulin heavy chain variable region mRNA, partial cds.
								DQ431608	Homo sapiens donor 7 serotype PPS4 clone 4 immunoglobulin heavy chain variable region mRNA, partial cds.
								DQ431610	Homo sapiens donor 8 serotype PPS14 clone 3 immunoglobulin heavy chain variable region mRNA, partial cds.
								DQ431617	Homo sapiens donor 8 serotype PPS14 clone 48 immunoglobulin heavy chain variable region mRNA, partial cds.
								DQ431618	Homo sapiens donor 8 serotype PPS4 clone 57 immunoglobulin heavy chain variable region mRNA, partial cds.
								DQ431619	Homo sapiens donor 8 serotype PPS14 clone 48 immunoglobulin heavy chain variable region mRNA, partial cds.
								DQ431624	Homo sapiens clone IM1-3 immunoglobulin heavy chain variable region VH mRNA, partial cds.
								DQ431625	Homo sapiens clone IM2-3 immunoglobulin heavy chain variable region VH mRNA, partial cds.
								DQ431626	Homo sapiens clone IM4-5 immunoglobulin heavy chain variable region VH mRNA, partial cds.
								DQ431636	Homo sapiens clone IM5-3 immunoglobulin heavy chain variable region VH mRNA, partial cds.
								DQ431645	Homo sapiens clone IM6-1 immunoglobulin heavy chain variable region VH mRNA, partial cds.
								DQ431652	Homo sapiens clone IM6-4 immunoglobulin heavy chain variable region VH mRNA, partial cds.
								DQ431654	Homo sapiens clone 43 immunoglobulin epsilon heavy chain variable region mRNA, partial cds.
								DQ431655	Homo sapiens clone 31 immunoglobulin epsilon heavy chain variable region mRNA, partial cds.
								DQ490728	Homo sapiens clone 34R immunoglobulin epsilon heavy chain variable region mRNA, partial cds.
								DQ490736	Homo sapiens clone 14 immunoglobulin epsilon heavy chain variable region mRNA, partial cds.
								DQ490738	Homo sapiens clone 3 immunoglobulin epsilon heavy chain variable region mRNA, partial cds.
								DQ497189	Homo sapiens clone 60 immunoglobulin epsilon heavy chain variable region mRNA, partial cds.
								DQ535261	Homo sapiens clone 2 immunoglobulin epsilon heavy chain variable region mRNA, partial cds.
								DQ535263	Homo sapiens clone 25 immunoglobulin epsilon heavy chain variable region mRNA, partial cds.
								DQ535264	Homo sapiens clone 39 immunoglobulin epsilon heavy chain variable region mRNA, partial cds.
								DQ535265	Homo sapiens clone 24 immunoglobulin epsilon heavy chain variable region mRNA, partial cds.
								DQ535267	Homo sapiens clone 22 immunoglobulin epsilon heavy chain variable region mRNA, partial cds.

								DQ535272<br>DQ535274<br>DQ535276<br>DQ535277<br>DQ535278<br>DQ535279<br>DQ535280<br>DQ535281<br>DQ535282<br>DQ535283<br>DQ535285<br>DQ535287<br>DQ535289<br>DQ535290<br>DQ535292<br>DQ535293<br>DQ535295<br>DQ535297<br>DQ535298<br>DQ535299<br>DQ535300<br>DQ535302<br>DQ535303<br>DQ535305<br>DQ535306<br>DQ535307<br>DQ535308<br>DQ535313<br>DQ535316<br>DQ535317<br>DQ535319<br>DQ535320<br>DQ535321<br>DQ535323<br>DQ535328<br>DQ535334<br>DQ535340<br>DQ535341<br>DQ535348<br>DQ535355<br>DQ535356<br>DQ535363<br>DQ535369<br>DQ535373<br>DQ535374<br>DQ535375<br>DQ535376<br>DQ535390<br>DQ535394<br>DQ535396<br>DQ535397<br>DQ535399<br>DQ535404<br>DQ535406	Homo sapiens clone 4 immunoglobulin epsilon heavy chain variable region mRNA, partial cds.<br>Homo sapiens clone 51 immunoglobulin epsilon heavy chain variable region mRNA, partial cds.<br>Homo sapiens clone TTP-A41 immunoglobulin heavy chain variable region mRNA, partial cds.<br>Homo sapiens immunoglobulin heavy chain variable region EM1-PPS-14-VH3-11 mRNA, partial cds.<br>Homo sapiens immunoglobulin heavy chain variable region EM1-PPS-14-VH3-13 mRNA, partial cds.<br>Homo sapiens immunoglobulin heavy chain variable region EM1-PPS-14-VH3-15 mRNA, partial cds.<br>Homo sapiens immunoglobulin heavy chain variable region EM1-PPS-14-VH3-20 mRNA, partial cds.<br>Homo sapiens immunoglobulin heavy chain variable region EM1-PPS-4-VH3-10 mRNA, partial cds.<br>Homo sapiens immunoglobulin heavy chain variable region EM1-PPS-4-VH3-2 mRNA, partial cds.<br>Homo sapiens immunoglobulin heavy chain variable region EM1-PPS-4-VH3-3 mRNA, partial cds.<br>Homo sapiens immunoglobulin heavy chain variable region EM1-PPS-4-VH3-5 mRNA, partial cds.<br>Homo sapiens immunoglobulin heavy chain variable region EM1-PPS-4-VH3-6 mRNA, partial cds.<br>Homo sapiens immunoglobulin heavy chain variable region EM1-PPS-4-VH3-7 mRNA, partial cds.<br>Homo sapiens immunoglobulin heavy chain variable region EM2-PPS-14-VH3-16 mRNA, partial cds.<br>Homo sapiens immunoglobulin heavy chain variable region EM2-PPS-14-VH3-17 mRNA, partial cds.<br>Homo sapiens immunoglobulin heavy chain variable region EM2-PPS-14-VH3-20 mRNA, partial cds.<br>Homo sapiens immunoglobulin heavy chain variable region EM2-PPS-VH3-10 mRNA, partial cds.<br>Homo sapiens immunoglobulin heavy chain variable region EM2-PPS-4-VH3-3 mRNA, partial cds.<br>Homo sapiens immunoglobulin heavy chain variable region EM2-PPS-4-VH3-4 mRNA, partial cds.<br>Homo sapiens immunoglobulin heavy chain variable region EM2-PPS-4-VH3-5 mRNA, partial cds.<br>Homo sapiens immunoglobulin heavy chain variable region EM2-PPS-4-VH3-6 mRNA, partial cds.<br>Homo sapiens immunoglobulin heavy chain variable region EM2-PPS-4-VH3-7 mRNA, partial cds.<br>Homo sapiens immunoglobulin heavy chain variable region EM3-PPS-14-VH3-7 mRNA, partial cds.<br>Homo sapiens immunoglobulin heavy chain variable region EM3-PPS-4-VH3-10 mRNA, partial cds.<br>Homo sapiens immunoglobulin heavy chain variable region EM3-PPS-4-VH3-3 mRNA, partial cds.<br>Homo sapiens immunoglobulin heavy chain variable region EM4-PPS-14-VH3-30 mRNA, partial cds.<br>Homo sapiens immunoglobulin heavy chain variable region EM4-14-PPS-VH3-6 mRNA, partial cds.<br>Homo sapiens immunoglobulin heavy chain variable region EM4-14-PPS-VH3-7 mRNA, partial cds.<br>Homo sapiens immunoglobulin heavy chain variable region EM4-4-PPS-VH3-3 mRNA, partial cds.<br>Homo sapiens immunoglobulin heavy chain variable region EV2-14-VH3-7 mRNA, partial cds.<br>Homo sapiens immunoglobulin heavy chain variable region EV2-4-VH3-7 mRNA, partial cds.<br>Homo sapiens immunoglobulin heavy chain variable region EV3-4-VH3-1 mRNA, partial cds.<br>Homo sapiens immunoglobulin heavy chain variable region EV3-4-VH3-4 mRNA, partial cds.<br>Homo sapiens immunoglobulin heavy chain variable region EV4-14-VH3-14 mRNA, partial cds.<br>Homo sapiens immunoglobulin heavy chain variable region EV4-4-VH3-65 mRNA, partial cds.<br>Homo sapiens immunoglobulin heavy chain variable region EV5-14-VH3-8 mRNA, partial cds.<br>Homo sapiens immunoglobulin heavy chain variable region EV5-14-VH3-95 mRNA, partial cds.<br>Homo sapiens immunoglobulin heavy chain variable region EV5-4-VH3-10 mRNA, partial cds.<br>Homo sapiens immunoglobulin heavy chain variable region EV5-4-VH3-4 mRNA, partial cds.<br>Homo sapiens immunoglobulin heavy chain variable region YM2-PPS-14-VH3-29 mRNA, partial cds.<br>Homo sapiens immunoglobulin heavy chain variable region YM2-PPS-4-VH3-3 mRNA, partial cds.<br>Homo sapiens immunoglobulin heavy chain variable region YM3-PPS-4-VH3-7 mRNA, partial cds.<br>Homo sapiens immunoglobulin heavy chain variable region YM4-PPS-14-VH3-9 mRNA, partial cds.<br>Homo sapiens immunoglobulin heavy chain variable region YM4-PPS-4-VH3-7 mRNA, partial cds.<br>Homo sapiens immunoglobulin heavy chain variable region YM5-PPS-14-VH3-40 mRNA, partial cds.<br>Homo sapiens immunoglobulin heavy chain variable region YM5-PPS-4-VH3-14 mRNA, partial cds.<br>Homo sapiens immunoglobulin heavy chain variable region YV1-14-VH3-59 mRNA, partial cds.<br>Homo sapiens immunoglobulin heavy chain variable region YV1-4-VH3-A4 mRNA, partial cds.<br>Homo sapiens immunoglobulin heavy chain variable region YV2-14-VH3-H20 mRNA, partial cds.<br>Homo sapiens immunoglobulin heavy chain variable region YV3-14-VH3-H9 mRNA, partial cds.<br>Homo sapiens immunoglobulin heavy chain variable region YV4-14-VH3-4 mRNA, partial cds.<br>Homo sapiens immunoglobulin heavy chain variable region YV4-4-VH3-2 mRNA, partial cds.<br>Homo sapiens patient 7 immunoglobulin heavy chain variable region mRNA, partial cds.<br>Homo sapiens patient 13 immunoglobulin heavy chain variable region mRNA, partial cds.

								DQ535409	Homo sapiens MDA-MB-231 immunoglobulin heavy chain mRNA, partial cds.
								DQ535411	Homo sapiens SKBR3 immunoglobulin heavy chain mRNA, partial cds.
								DQ535412	Homo sapiens ZR75-1 immunoglobulin heavy chain mRNA, partial cds.
								DQ535413	Homo sapiens isolate D01-119F10 anti-Rh(D) antibody immunoglobulin heavy chain variable region mRNA, partial cds.
								DQ535415	Homo sapiens isolate D01-119A02 anti-Rh(D) antibody immunoglobulin heavy chain variable region mRNA, partial cds.
								DQ535418	Homo sapiens isolate D01-119C06 anti-Rh(D) antibody immunoglobulin heavy chain variable region mRNA, partial cds.
								DQ535419	Homo sapiens isolate D01-119B09 anti-Rh(D) antibody immunoglobulin heavy chain variable region mRNA, partial cds.
								DQ535420	Homo sapiens isolate D02-109A09 anti-Rh(D) antibody immunoglobulin heavy chain variable region mRNA, partial cds.
								DQ535422	Homo sapiens isolate D02-109A05 anti-Rh(D) antibody immunoglobulin heavy chain variable region mRNA, partial cds.
								DQ535425	Homo sapiens isolate D02-186A07 anti-Rh(D) antibody immunoglobulin heavy chain variable region mRNA, partial cds.
								DQ535427	Homo sapiens isolate D04-126B05 anti-Rh(D) antibody immunoglobulin heavy chain variable region mRNA, partial cds.
								DQ535428	Homo sapiens isolate D04-126B12 anti-Rh(D) antibody immunoglobulin heavy chain variable region mRNA, partial cds.
								DQ535429	Homo sapiens isolate D04-126G10 anti-Rh(D) antibody immunoglobulin heavy chain variable region mRNA, partial cds.
								DQ535432	Homo sapiens isolate D04-229B07 anti-Rh(D) antibody immunoglobulin heavy chain variable region mRNA, partial cds.
								DQ535433	Homo sapiens isolate D05-230D05 anti-Rh(D) antibody immunoglobulin heavy chain variable region mRNA, partial cds.
								DQ535434	Homo sapiens isolate D05-230F06 anti-Rh(D) antibody immunoglobulin heavy chain variable region mRNA, partial cds.
								DQ535435	Homo sapiens isolate D05-158E03 anti-Rh(D) antibody immunoglobulin heavy chain variable region mRNA, partial cds.
								DQ535436	Homo sapiens isolate D05-158B10 anti-Rh(D) antibody immunoglobulin heavy chain variable region mRNA, partial cds.
								DQ535437	Homo sapiens isolate D05-158A03 anti-Rh(D) antibody immunoglobulin heavy chain variable region mRNA, partial cds.
								DQ535438	Homo sapiens isolate D05-158E02 anti-Rh(D) antibody immunoglobulin heavy chain variable region mRNA, partial cds.
								DQ535441	Homo sapiens isolate D05-127A07 anti-Rh(D) antibody immunoglobulin heavy chain variable region mRNA, partial cds.
								DQ535442	Homo sapiens isolate D05-127G11 anti-Rh(D) antibody immunoglobulin heavy chain variable region mRNA, partial cds.
								DQ535444	Homo sapiens isolate D05-134C02 anti-Rh(D) antibody immunoglobulin heavy chain variable region mRNA, partial cds.
								DQ535452	Homo sapiens isolate D05-134E03 anti-Rh(D) antibody immunoglobulin heavy chain variable region mRNA, partial cds.
								DQ535453	Homo sapiens isolate D06-160A02 anti-Rh(D) antibody immunoglobulin heavy chain variable region mRNA, partial cds.
								DQ535456	Homo sapiens isolate D06-160A09 anti-Rh(D) antibody immunoglobulin heavy chain variable region mRNA, partial cds.
								DQ535457	Homo sapiens isolate D06-160B10 anti-Rh(D) antibody immunoglobulin heavy chain variable region mRNA, partial cds.
								DQ535461	Homo sapiens isolate D06-160B11 anti-Rh(D) antibody immunoglobulin heavy chain variable region mRNA, partial cds.
								DQ535463	Homo sapiens isolate D06-160E03 anti-Rh(D) antibody immunoglobulin heavy chain variable region mRNA, partial cds.
								DQ535465	Homo sapiens isolate D06-187C09 anti-Rh(D) antibody immunoglobulin heavy chain variable region mRNA, partial cds.
								DQ535466	Homo sapiens isolate D07-164C08 anti-Rh(D) antibody immunoglobulin heavy chain variable region mRNA, partial cds.
								DQ535468	Homo sapiens isolate D07-164E06 anti-Rh(D) antibody immunoglobulin heavy chain variable region mRNA, partial cds.
								DQ535469	Homo sapiens isolate D07-164E03 anti-Rh(D) antibody immunoglobulin heavy chain variable region mRNA, partial cds.
								DQ535470	Homo sapiens isolate D07-184E08 anti-Rh(D) antibody immunoglobulin heavy chain variable region mRNA, partial cds.
								DQ535473	Homo sapiens isolate D07-184C11 anti-Rh(D) antibody immunoglobulin heavy chain variable region mRNA, partial cds.
								DQ535474	Homo sapiens isolate D07-184H06 anti-Rh(D) antibody immunoglobulin heavy chain variable region mRNA, partial cds.
								DQ535475	Homo sapiens isolate D08-179B11 anti-Rh(D) antibody immunoglobulin heavy chain variable region mRNA, partial cds.
								DQ535476	Homo sapiens isolate D08-181F05 anti-Rh(D) antibody immunoglobulin heavy chain variable region mRNA, partial cds.
								DQ535477	Homo sapiens isolate D08-181D12 anti-Rh(D) antibody immunoglobulin heavy chain variable region mRNA, partial cds.
								DQ535480	Homo sapiens isolate D08-181E07 anti-Rh(D) antibody immunoglobulin heavy chain variable region mRNA, partial cds.
								DQ535481	Homo sapiens isolate D08-224A03 anti-Rh(D) antibody immunoglobulin heavy chain variable region mRNA, partial cds.
								DQ535483	Homo sapiens clone SC4299 anti-West Nile virus immunoglobulin heavy chain variable region mRNA, partial cds.
								DQ535484	Homo sapiens clone SC4368 anti-West Nile virus immunoglobulin heavy chain variable region mRNA, partial cds.
								DQ768422	Homo sapiens clone SC4353 anti-West Nile virus immunoglobulin heavy chain variable region mRNA, partial cds.
								Z47249	Homo sapiens nonfunctional anti-SARS-CoV S protein immunoglobulin heavy chain Fd fragment mRNA, partial sequence.
								Z47252	Homo sapiens clone HA1-9 circulating B cell antibody heavy chain variable region mRNA, partial cds.
								Z47257	Homo sapiens clone HA2-12 circulating B cell antibody heavy chain variable region mRNA, partial cds.
								Z47260	Homo sapiens clone HA2-21 circulating B cell antibody heavy chain variable region mRNA, partial cds.
								Z47267	Homo sapiens clone HA2-24 circulating B cell antibody heavy chain variable region mRNA, partial cds.
								Z47271	Homo sapiens clone HA3-2 circulating B cell antibody heavy chain variable region mRNA, partial cds.
								Z47277	Homo sapiens clone HA3-16 circulating B cell antibody heavy chain variable region mRNA, partial cds.
								X53613	Homo sapiens clone HA3-25 circulating B cell antibody heavy chain variable region mRNA, partial cds.
								Z70612	Homo sapiens clone HB1-4 circulating B cell antibody heavy chain variable region mRNA, partial cds.
								Z70615	Homo sapiens clone HB1-5 circulating B cell antibody heavy chain variable region mRNA, partial cds.

								Z70617	Homo sapiens clone HB1-6 circulating B cell antibody heavy chain variable region mRNA, partial cds.
								Z70618	Homo sapiens clone HB1-10 circulating B cell antibody heavy chain variable region mRNA, partial cds.
								Z70616	Homo sapiens clone HB1-14 circulating B cell antibody heavy chain variable region mRNA, partial cds.
								Z70621	Homo sapiens clone HB1-15 circulating B cell antibody heavy chain variable region mRNA, partial cds.
								Z70613	Homo sapiens clone HB1-18 circulating B cell antibody heavy chain variable region mRNA, partial cds.
								Z70638	Homo sapiens clone HB1-21 circulating B cell antibody heavy chain variable region mRNA, partial cds.
								Z70655	Homo sapiens clone HB1-30 circulating B cell antibody heavy chain variable region mRNA, partial cds.
								Z70637	Homo sapiens clone HB1-33 circulating B cell antibody heavy chain variable region mRNA, partial cds.
								Z70654	Homo sapiens clone HB1-41 circulating B cell antibody heavy chain variable region mRNA, partial cds.
								Z70656	Homo sapiens clone HB1-44 circulating B cell antibody heavy chain variable region mRNA, partial cds.
								Z70645	Homo sapiens clone HB1-49 circulating B cell antibody heavy chain variable region mRNA, partial cds.
								Z70646	Homo sapiens clone HB1-50 circulating B cell antibody heavy chain variable region mRNA, partial cds.
								Z70658	Homo sapiens clone HB1-66 circulating B cell antibody heavy chain variable region mRNA, partial cds.
								Z70652	Homo sapiens clone HB1-68 circulating B cell antibody heavy chain variable region mRNA, partial cds.
								Z70643	Homo sapiens clone HB1-70 circulating B cell antibody heavy chain variable region mRNA, partial cds.
								Z70641	Homo sapiens clone HB1-72 circulating B cell antibody heavy chain variable region mRNA, partial cds.
								Z70657	Homo sapiens clone HB1-76 circulating B cell antibody heavy chain variable region mRNA, partial cds.
								Z70653	Homo sapiens clone HB1-80 circulating B cell antibody heavy chain variable region mRNA, partial cds.
								Z70632	Homo sapiens clone HB2-1 circulating B cell antibody heavy chain variable region mRNA, partial cds.
								Z70620	Homo sapiens clone HB2-8 circulating B cell antibody heavy chain variable region mRNA, partial cds.
								Z70635	Homo sapiens clone HB2-9 circulating B cell antibody heavy chain variable region mRNA, partial cds.
								AJ004954	Homo sapiens clone HB2-12 circulating B cell antibody heavy chain variable region mRNA, partial cds.
								AJ006161	Homo sapiens clone HB2-13 circulating B cell antibody heavy chain variable region mRNA, partial cds.
								AJ010443	Homo sapiens clone HB2-46 circulating B cell antibody heavy chain variable region mRNA, partial cds.
								AJ010445	Homo sapiens clone HB2-51 circulating B cell antibody heavy chain variable region mRNA, partial cds.
								AJ010447	Homo sapiens clone HB2-64 circulating B cell antibody heavy chain variable region mRNA, partial cds.
								AJ131062	Homo sapiens clone HB2-66 circulating B cell antibody heavy chain variable region mRNA, partial cds.
								AJ132565	Homo sapiens clone HB2-70 circulating B cell antibody heavy chain variable region mRNA, partial cds.
								AJ132847	Homo sapiens clone HB2-73 circulating B cell antibody heavy chain variable region mRNA, partial cds.
								AJ132850	Homo sapiens clone HB2-89 circulating B cell antibody heavy chain variable region mRNA, partial cds.
								AJ132851	Homo sapiens clone HB3-9 circulating B cell antibody heavy chain variable region mRNA, partial cds.
								Y17934	Homo sapiens clone HB3-22 circulating B cell antibody heavy chain variable region mRNA, partial cds.
								Y17945	Homo sapiens clone HB3-31 circulating B cell antibody heavy chain variable region mRNA, partial cds.
								AJ233694	Homo sapiens clone HB3-32 circulating B cell antibody heavy chain variable region mRNA, partial cds.
								AJ233695	Homo sapiens clone HB3-42 circulating B cell antibody heavy chain variable region mRNA, partial cds.
								AJ233696	Homo sapiens clone HB3-58 circulating B cell antibody heavy chain variable region mRNA, partial cds.
								AJ233698	Homo sapiens clone HB3-61 circulating B cell antibody heavy chain variable region mRNA, partial cds.
								AJ233701	Homo sapiens clone HB3-71 circulating B cell antibody heavy chain variable region mRNA, partial cds.
								AJ233703	Homo sapiens clone HC1-9 circulating B cell antibody heavy chain variable region mRNA, partial cds.
								AJ233707	Homo sapiens clone HC1-18 circulating B cell antibody heavy chain variable region mRNA, partial cds.
								AJ233708	Homo sapiens clone HC1-24 circulating B cell antibody heavy chain variable region mRNA, partial cds.
								AJ238038	Homo sapiens clone HC1-26 circulating B cell antibody heavy chain variable region mRNA, partial cds.
								AJ238039	Homo sapiens clone HC2-7 circulating B cell antibody heavy chain variable region mRNA, partial cds.
								AJ239331	Homo sapiens clone HC3-12 circulating B cell antibody heavy chain variable region mRNA, partial cds.
								AJ239332	Homo sapiens clone HC3-18 circulating B cell antibody heavy chain variable region mRNA, partial cds.
								AJ239334	Homo sapiens clone HC3-20 circulating B cell antibody heavy chain variable region mRNA, partial cds.
								AJ239335	Homo sapiens clone HC3-21 circulating B cell antibody heavy chain variable region mRNA, partial cds.
								AJ239336	Homo sapiens clone HC3-23 circulating B cell antibody heavy chain variable region mRNA, partial cds.
								AJ239338	Homo sapiens clone HC3-33 circulating B cell antibody heavy chain variable region mRNA, partial cds.
								AJ239339	Homo sapiens clone HC3-36 circulating B cell antibody heavy chain variable region mRNA, partial cds.
								AJ239341	Homo sapiens clone HE1-28 circulating B cell antibody heavy chain variable region mRNA, partial cds.
								AJ239345	Homo sapiens clone HE1-33 circulating B cell antibody heavy chain variable region mRNA, partial cds.
								AJ239349	Homo sapiens clone HE1-34 circulating B cell antibody heavy chain variable region mRNA, partial cds.
								AJ239350	Homo sapiens clone HE2-1 circulating B cell antibody heavy chain variable region mRNA, partial cds.

								AJ239353<br>AJ239354<br>AJ239356<br>AJ239357<br>AJ239363<br>AJ239367<br>AJ239370<br>AJ239371<br>AJ239375<br>AJ239376<br>AJ239379<br>AJ239383<br>AJ239387<br>AJ239390<br>AJ240562<br>AJ240564<br>AJ240569<br>AJ240580<br>AJ240581<br>AJ240589<br>AJ240590<br>AJ240595<br>AJ240604<br>AJ241349<br>AJ241351<br>AJ241421<br>AJ243108<br>AJ243110<br>AJ244928<br>AJ244930<br>AJ244932<br>AJ244933<br>AJ244934<br>AJ244936<br>AJ244937<br>AJ244939<br>AJ244943<br>AJ244945<br>AJ244946<br>AJ244948<br>AJ244949<br>AJ244952<br>AJ244953<br>AJ244954<br>AJ244955<br>AJ244956<br>AJ244959<br>AJ244962<br>AJ244963<br>AJ244964<br>AJ244965<br>AJ244966<br>AJ244967<br>AJ244970	Homo sapiens clone HE2-23 circulating B cell antibody heavy chain variable region mRNA, partial cds.<br>Homo sapiens clone HE3-10 circulating B cell antibody heavy chain variable region mRNA, partial cds.<br>Homo sapiens clone HF1-2 circulating B cell antibody heavy chain variable region mRNA, partial cds.<br>Homo sapiens clone HF1-3 circulating B cell antibody heavy chain variable region mRNA, partial cds.<br>Homo sapiens clone HF1-27 circulating B cell antibody heavy chain variable region mRNA, partial cds.<br>Homo sapiens clone HF1-39 circulating B cell antibody heavy chain variable region mRNA, partial cds.<br>Homo sapiens clone HF1-51 circulating B cell antibody heavy chain variable region mRNA, partial cds.<br>Homo sapiens clone HF1-52 circulating B cell antibody heavy chain variable region mRNA, partial cds.<br>Homo sapiens clone HF1-53 circulating B cell antibody heavy chain variable region mRNA, partial cds.<br>Homo sapiens clone HF1-68 circulating B cell antibody heavy chain variable region mRNA, partial cds.<br>Homo sapiens clone HF1-72 circulating B cell antibody heavy chain variable region mRNA, partial cds.<br>Homo sapiens clone HF1-76 circulating B cell antibody heavy chain variable region mRNA, partial cds.<br>Homo sapiens clone HF1-80 circulating B cell antibody heavy chain variable region mRNA, partial cds.<br>Homo sapiens clone HF1-81 circulating B cell antibody heavy chain variable region mRNA, partial cds.<br>Homo sapiens clone HF1-97 circulating B cell antibody heavy chain variable region mRNA, partial cds.<br>Homo sapiens clone HF1-99 circulating B cell antibody heavy chain variable region mRNA, partial cds.<br>Homo sapiens clone HF1-102 circulating B cell antibody heavy chain variable region mRNA, partial cds.<br>Homo sapiens clone HF1-103 circulating B cell antibody heavy chain variable region mRNA, partial cds.<br>Homo sapiens clone HF1-108 circulating B cell antibody heavy chain variable region mRNA, partial cds.<br>Homo sapiens clone HF2-23 circulating B cell antibody heavy chain variable region mRNA, partial cds.<br>Homo sapiens clone HF2-26 circulating B cell antibody heavy chain variable region mRNA, partial cds.<br>Homo sapiens clone HF2-40 circulating B cell antibody heavy chain variable region mRNA, partial cds.<br>Homo sapiens clone HF2-42 circulating B cell antibody heavy chain variable region mRNA, partial cds.<br>Homo sapiens clone HF2-61 circulating B cell antibody heavy chain variable region mRNA, partial cds.<br>Homo sapiens clone HF2-79 circulating B cell antibody heavy chain variable region mRNA, partial cds.<br>Homo sapiens clone HF2-95 circulating B cell antibody heavy chain variable region mRNA, partial cds.<br>Homo sapiens clone HF2-98 circulating B cell antibody heavy chain variable region mRNA, partial cds.<br>Homo sapiens clone HG1-1 circulating B cell antibody heavy chain variable region mRNA, partial cds.<br>Homo sapiens clone HG1-2 circulating B cell antibody heavy chain variable region mRNA, partial cds.<br>Homo sapiens clone HG1-3 circulating B cell antibody heavy chain variable region mRNA, partial cds.<br>Homo sapiens clone HG1-12 circulating B cell antibody heavy chain variable region mRNA, partial cds.<br>Homo sapiens clone HG1-13 circulating B cell antibody heavy chain variable region mRNA, partial cds.<br>Homo sapiens clone HG1-16 circulating B cell antibody heavy chain variable region mRNA, partial cds.<br>Homo sapiens clone HG1-21 circulating B cell antibody heavy chain variable region mRNA, partial cds.<br>Homo sapiens clone HG2-1 circulating B cell antibody heavy chain variable region mRNA, partial cds.<br>Homo sapiens clone HG2-6 circulating B cell antibody heavy chain variable region mRNA, partial cds.<br>Homo sapiens clone HG2-8 circulating B cell antibody heavy chain variable region mRNA, partial cds.<br>Homo sapiens clone HJ2-1 circulating B cell antibody heavy chain variable region mRNA, partial cds.<br>Homo sapiens clone HJ2-5 circulating B cell antibody heavy chain variable region mRNA, partial cds.<br>Homo sapiens anti-streptococcal/anti-lysoganglioside immunoglobulin heavy chain variable region mRNA, partial cds.<br>H.sapiens mRNA for immunoglobulin variable region (clone 201-E9).<br>H.sapiens mRNA for immunoglobulin variable region (clone 201-G6).<br>H.sapiens mRNA for immunoglobulin variable region (clone 202-E4).<br>H.sapiens mRNA for immunoglobulin variable region (clone 202-G8).<br>H.sapiens mRNA for immunoglobulin variable region (clone 203-E9).<br>H.sapiens mRNA for immunoglobulin variable region (clone 203-G4).<br>H.sapiens mRNA for immunoglobulin variable region (clone 204-G2).<br>Human mRNA for 3D6 heavy chain variable region.<br>H.sapiens mRNA for immunoglobulin heavy chain variable region (6D4-8, VH4, 4-59/DP-71).<br>H.sapiens mRNA for immunoglobulin heavy chain variable region (6D4-A4, VH4, 4-59/DP-71).<br>H.sapiens mRNA for immunoglobulin heavy chain variable region (6D4-A7, VH4, 4-34/DP-63).<br>H.sapiens mRNA for immunoglobulin heavy chain variable region (6D4-D1, VH4, 4-31/DP-65).<br>H.sapiens mRNA for immunoglobulin heavy chain variable region (6D4-F1, VH4, 4-34/DP-63).<br>H.sapiens mRNA for immunoglobulin heavy chain variable region (6D4-F2, VH4, 4-39/DP-79).

								AJ244971	H.sapiens mRNA for immunoglobulin heavy chain variable region (6D4-G2, VH4, 4-59/DP-71).
								AJ244973	H.sapiens mRNA for immunoglobulin heavy chain variable region (83-6B2, VH4, 4-34/DP-63).
								AJ244976	H.sapiens mRNA for immunoglobulin heavy chain variable region (83-6B5, VH4, 4-39/DP-79).
								AJ244977	H.sapiens mRNA for immunoglobulin heavy chain variable region (83-6C3, VH4, 4-34/DP-63).
								AJ244978	H.sapiens mRNA for immunoglobulin heavy chain variable region (83-6C4, VH4, 4-39/DP-79).
								AJ244980	H.sapiens mRNA for immunoglobulin heavy chain variable region (83-6C5, VH4, 4-39/DP-79).
								AJ244981	H.sapiens mRNA for immunoglobulin heavy chain variable region (83-6F2, VH4, 4-31/DP-65).
								AJ244983	H.sapiens mRNA for immunoglobulin heavy chain variable region (83-6F3, VH4, 4-31/DP-65).
								AJ244986	H.sapiens mRNA for immunoglobulin heavy chain variable region (83-6H3, VH4, 4-61/DP-66).
								AJ244990	H.sapiens mRNA for immunoglobulin heavy chain variable region (83-9A6, VH4, 4-59/DP-71).
								AJ244992	H.sapiens mRNA for immunoglobulin heavy chain variable region (83-9B6, VH4, 4-34/DP-63).
								AJ244993	H.sapiens mRNA for immunoglobulin heavy chain variable region (83-9E8, VH4, 4-34/DP-63).
								AJ244994	H.sapiens mRNA for immunoglobulin heavy chain variable region (83-9E9, VH4, 4-39/DP-79).
								AJ244995	H.sapiens mRNA for immunoglobulin heavy chain variable region (83-9G6, VH4, 4-59/DP-71).
								AJ244998	H.sapiens mRNA for immunoglobulin heavy chain variable region (9D4-9, VH4, 4-39/DP-79).
								AJ244999	H.sapiens mRNA for immunoglobulin heavy chain variable region (9D4-B6, VH4, 4-31/DP-65).
								AJ245000	H.sapiens mRNA for immunoglobulin heavy chain variable region (9D4-F3, VH4, 4-4).
								AJ245002	Homo sapiens Immunoglobulin variable region germ-line transcripts (RF.BM, YF.PB2 cell line).
								AJ245003	Homo sapiens mRNA for variable region of Ig heavy chain, rearrangement of BC2 cell line).
								AJ245004	Homo sapiens mRNA for immunoglobulin IgG3 heavy chain, anti-RhD, therad 7.
								AJ245006	Homo sapiens mRNA for immunoglobulin IgG1 heavy chain, anti-RhD, therad 10.
								AJ245008	Homo sapiens mRNA for immunoglobulin IgG3 heavy chain, anti RhD, therad 24.
								AJ245009	Homo sapiens mRNA for birch pollen allergen specific immunoglobulin gamma chain (BAB1-H), complete V-D-J region.
								AJ245010	Homo sapiens mRNA for immunoglobulin heavy chain, partial.
								AJ245012	Homo sapiens mRNA for immunoglobulin heavy chain variable region, clone MGgisb14, partial.
								AJ245013	Homo sapiens mRNA for immunoglobulin heavy chain variable region, clone MGgisa24, partial.
								AJ245014	Homo sapiens mRNA for immunoglobulin heavy chain variable region, clone MGgisa29, partial.
								AJ245016	Homo sapiens mRNA for immunoglobulin heavy chain, JK31H.
								AJ245018	Homo sapiens mRNA for immunoglobulin heavy chain, KC13H.
								AJ245022	Homo sapiens partial mRNA for immunoglobulin G heavy chain variable region (IGHV3 gene), clone pComb3-LO-ie02.
								AJ245023	Homo sapiens partial mRNA for immunoglobulin G heavy chain variable region (IGHV3 gene), clone pComb3-LO-ie06.
								AJ245027	Homo sapiens partial mRNA for immunoglobulin G heavy chain variable region (IGHV4 gene), clone pComb3-LO-ie10.
								AJ245029	Homo sapiens partial mRNA for immunoglobulin G heavy Chain variable region (IGHV4 gene), clone pComb3-LO-ie21.
								AJ245031	Homo sapiens partial mRNA for immunoglobulin G heavy chain variable region (IGHV3 gene), clone pComb3-LO-ie39.
								AJ245032	Homo sapiens partial mRNA for immunoglobulin G heavy chain variable region (IGHV3 gene), clone pComb3-GG-ib02.
								AJ245033	Homo sapiens partial mRNA for immunoglobulin G heavy chain variable region (IGHV3 gene), clone pComb3-NK-ib02.
								AJ245034	Homo sapiens partial mRNA for immunoglobulin G heavy chain variable region (IGHV3 gene), clone pComb3-NK-ib07.
								AJ245039	Homo sapiens mRNA for IGHV gene for immunoglobulin heavy chain variable region, IgM multiple myeloma patient DEZ.
								AJ245040	Homo sapiens mRNA for IGHV gene for immunoglobulin heavy chain variable region, IgM multiple myeloma patient JON.
								AJ245041	Homo sapiens mRNA for immunoglobulin heavy chain variable region, ID 14.
								AJ245044	Homo sapiens mRNA for immunoglobulin heavy chain variable region, ID 33.
								AJ245045	Homo sapiens mRNA for immunoglobulin heavy chain variable region, ID 78.
								AJ245047	Homo sapiens mRNA for immunoglobulin heavy chain variable region, ID 42.
								AJ245048	Homo sapiens mRNA for immunoglobulin heavy chain variable region, ID 20.
								AJ245049	Homo sapiens mRNA for immunoglobulin heavy chain variable region, ID 29.
								AJ245053	Homo sapiens mRNA for immunoglobulin heavy chain variable region, ID 61.
								AJ245055	Homo sapiens mRNA for immunoglobulin heavy chain variable region, ID 27.
								AJ245056	Homo sapiens mRNA for immunoglobulin heavy chain variable region, ID 11.
								AJ245060	Homo sapiens mRNA for immunoglobulin heavy chain variable region, ID 44.
								AJ245062	Homo sapiens mRNA for immunoglobulin heavy chain variable region, ID 30.
								AJ245064	Homo sapiens mRNA for immunoglobulin heavy chain variable region, ID 66.
								AJ245065	Homo sapiens mRNA for immunoglobulin heavy chain variable region, ID 10.
								AJ245066	Homo sapiens mRNA for immunoglobulin heavy chain variable region, ID 76.
								AJ245071	Homo sapiens mRNA for immunoglobulin heavy chain variable region, ID 12.

								AJ245214	Homo sapiens mRNA for immunoglobulin heavy chain variable region, ID 41.
								AJ245229	Homo sapiens mRNA for immunoglobulin heavy chain variable region, ID 70.
								AJ245230	Homo sapiens mRNA for immunoglobulin heavy chain variable region, ID 63.
								AJ245233	Homo sapiens mRNA for immunoglobulin heavy chain variable region, ID 3.
								AJ245234	Homo sapiens mRNA for immunoglobulin heavy chain variable region, ID 5.
								AJ245237	Homo sapiens mRNA for immunoglobulin heavy chain variable region, ID 32.
								AJ245238	Homo sapiens mRNA for immunoglobulin heavy chain variable region, ID 28.
								AJ245240	Homo sapiens mRNA for immunoglobulin heavy chain variable region, ID 31.
								AJ245242	Homo sapiens mRNA for immunoglobulin heavy chain variable region, ID 81.
								AJ245261	Homo sapiens mRNA for immunoglobulin heavy chain variable region, ID 68.
								AJ245274	Homo sapiens mRNA for rearranged immunoglobulin A1 heavy chain, IgVH4-34-D-JH3-Calpha1, clone EW 70.
								AJ245276	Homo sapiens mRNA for rearranged immunoglobulin A1 heavy chain, IgVH4-34-D-JH4-Calpha1, clone EW 72.
								AJ245277	Homo sapiens mRNA for rearranged immunoglobulin A1 heavy chain, IgVH4-34-D-JH3-Calpha1, clone EW 80.
								AJ245279	Homo sapiens mRNA for rearranged immunoglobulin G1 heavy chain, IgVH4-34-D-JH4B-Cgamma1, clone EU 83.
								AJ245280	Homo sapiens mRNA for rearranged immunoglobulin G1 heavy chain, IgVH4-34-D-JH4B-Cgamma1, clone EU 89.
								AJ245281	Homo sapiens mRNA for rearranged immunoglobulin G1 heavy chain, IgVH4-34-D-JH4B-Cgamma1, clone EV 6.
								AJ245282	Homo sapiens mRNA for rearranged immunoglobulin G1 heavy chain, IgVH4-34-D-JH4B-Cgamma1, clone EV 9.
								AJ245284	Homo sapiens mRNA for rearranged immunoglobulin G1 heavy chain, IgVH4-34-D-JH5B-Cgamma1, clone EV 35.
								AJ245285	Homo sapiens mRNA for rearranged immunoglobulin G3 heavy chain, IgVH4-34-D-JH4B-Cgamma3, clone EU 40.
								AJ245286	Homo sapiens partial mRNA for vaccinia virus specific immunoglobulin heavy chain variable region (IGHV gene), clone 27H.
								AJ245289	Homo sapiens partial mRNA for vaccinia virus specific immunoglobulin heavy chain variable region (IGHV gene), clone 40H.
								AJ245290	Homo sapiens mRNA for partial immunoglobulin heavy chain variable region, clone Z5scFv7VH.
								AJ245291	Homo sapiens partial mRNA for immunoglobulin heavy chain variable region (IGHV gene).
								AJ245292	Homo sapiens partial mRNA for immunoglobulin heavy chain variable region (dp-47 gene).
								AJ245293	Homo sapiens mRNA for immunoglobulin mu heavy chain variable region, partial, clone 1-A31.
								AJ245296	Homo sapiens mRNA for immunoglobulin mu heavy chain variable region, partial, clone 1-A34.
								AJ245299	Homo sapiens mRNA for immunoglobulin mu heavy chain variable region, partial, clone 1-A39.
								AJ245304	Homo sapiens mRNA for immunoglobulin mu heavy chain variable region, partial, clone 1-A42.
								AJ245305	Homo sapiens mRNA for immunoglobulin mu heavy chain variable region, partial, clone 1-A45.
								AJ245306	Homo sapiens mRNA for immunoglobulin mu heavy chain variable region, partial, clone 1-A62.
								AJ245307	Homo sapiens mRNA for immunoglobulin mu heavy chain variable region, partial, clone 1-A66.
								AJ245308	Homo sapiens mRNA for immunoglobulin mu heavy chain variable region, partial, clone 2-A2.
								AJ245309	Homo sapiens mRNA for immunoglobulin mu heavy chain variable region, partial, clone 2-A8.
								AJ245322	Homo sapiens mRNA for immunoglobulin mu heavy chain variable region, partial, clone 2-A12.
								AJ245324	Homo sapiens mRNA for immunoglobulin mu heavy chain variable region, partial, clone 3-A1.
								AJ245334	Homo sapiens mRNA for immunoglobulin mu heavy chain variable region, partial, clone 3-A3.
								AJ245337	Homo sapiens mRNA for immunoglobulin mu heavy chain variable region, partial, clone 3-A5.
								AJ245341	Homo sapiens mRNA for immunoglobulin mu heavy chain variable region, partial, clone 3-A9.
								AJ245342	Homo sapiens mRNA for immunoglobulin mu heavy chain variable region, partial, clone 3-A15.
								AJ245345	Homo sapiens mRNA for immunoglobulin mu heavy chain variable region, partial, clone 1-B47.
								AJ245346	Homo sapiens mRNA for immunoglobulin mu heavy chain variable region, partial, clone 1-B49.
								AJ245347	Homo sapiens mRNA for immunoglobulin mu heavy chain variable region, partial, clone 1-B50.
								AJ245350	Homo sapiens mRNA for immunoglobulin mu heavy chain variable region, partial, clone 1-B72.
								AJ245354	Homo sapiens mRNA for immunoglobulin mu heavy chain variable region, partial, clone 1-B75.
								AJ245356	Homo sapiens mRNA for immunoglobulin mu heavy chain variable region, partial, clone 1-B76.
								AJ245357	Homo sapiens mRNA for immunoglobulin mu heavy chain variable region, partial, clone 1-B77.
								AJ245359	Homo sapiens mRNA for immunoglobulin mu heavy chain variable region, partial, clone 2-B17.
								AJ249633	Homo sapiens mRNA for immunoglobulin mu heavy chain variable region, partial, clone 2-B18.
								AJ253044	Homo sapiens mRNA for immunoglobulin mu heavy chain variable region, partial, clone 2-B19.
								AJ253050	Homo sapiens mRNA for immunoglobulin mu heavy chain variable region, partial, clone 2-B22.
								AJ253054	Homo sapiens mRNA for immunoglobulin mu heavy chain variable region, partial, clone 2-B23.
								AJ253059	Homo sapiens mRNA for immunoglobulin mu heavy chain variable region, partial, clone 2-B27.
								AJ253074	Homo sapiens mRNA for immunoglobulin mu heavy chain variable region, partial, clone 3-B24.
								AJ253079	Homo sapiens mRNA for immunoglobulin mu heavy chain variable region, partial, clone 3-B25.

								AJ253081	Homo sapiens mRNA for immunoglobulin mu heavy chain variable region, partial, clone 3-B27.
								AJ253088	Homo sapiens mRNA for immunoglobulin mu heavy chain variable region, partial, clone 3-B29.
								AJ253098	Homo sapiens mRNA for immunoglobulin mu heavy chain variable region, partial, clone 3-B30.
								AJ253104	Homo sapiens mRNA for immunoglobulin mu heavy chain variable region, partial, clone 1-C1.
								AJ253109	Homo sapiens mRNA for immunoglobulin mu heavy chain variable region, partial, clone 1-C4.
								AJ253111	Homo sapiens mRNA for immunoglobulin mu heavy chain variable region, partial, clone 1-C8.
								AJ271547	Homo sapiens mRNA for immunoglobulin mu heavy chain variable region, partial, clone 1-C10.
								AJ271548	Homo sapiens mRNA for immunoglobulin mu heavy chain variable region, partial, clone 1-C12.
								AJ271550	Homo sapiens mRNA for immunoglobulin mu heavy chain variable region, partial, clone 2-C81.
								AJ271551	Homo sapiens mRNA for immunoglobulin mu heavy chain variable region, partial, clone 2-C82.
								AJ271553	Homo sapiens mRNA for immunoglobulin mu heavy chain variable region, partial, clone 2-C85.
								AJ271554	Homo sapiens mRNA for immunoglobulin mu heavy chain variable region, partial, clone 2-C86.
								AJ271555	Homo sapiens mRNA for immunoglobulin mu heavy chain variable region, partial, clone 2-C87.
								AJ271556	Homo sapiens mRNA for immunoglobulin mu heavy chain variable region, partial, clone 2-C89.
								AJ271557	Homo sapiens mRNA for immunoglobulin mu heavy chain variable region, partial, clone 2-C90.
								AJ271602	Homo sapiens mRNA for immunoglobulin mu heavy chain variable region, partial, clone 2-C91.
								AJ271604	Homo sapiens mRNA for immunoglobulin mu heavy chain variable region, partial, clone 2-C94.
								AJ272334	Homo sapiens mRNA for immunoglobulin mu heavy chain variable region, partial, clone 1-E5.
								AJ279515	Homo sapiens mRNA for immunoglobulin mu heavy chain variable region, partial, clone 1-E7.
								AJ279516	Homo sapiens mRNA for immunoglobulin mu heavy chain variable region, partial, clone 1-E9.
								AJ279517	Homo sapiens mRNA for immunoglobulin mu heavy chain variable region, partial, clone 1-E13.
								AJ279518	Homo sapiens mRNA for immunoglobulin mu heavy chain variable region, partial, clone 1-E15.
								AJ279520	Homo sapiens mRNA for immunoglobulin mu heavy chain variable region, partial, clone 1-E85.
								AJ279522	Homo sapiens mRNA for immunoglobulin mu heavy chain variable region, partial, clone 1-E88.
								AJ279523	Homo sapiens mRNA for immunoglobulin mu heavy chain variable region, partial, clone 2-E62.
								AJ279525	Homo sapiens mRNA for immunoglobulin mu heavy chain variable region, partial, clone 2-E68.
								AJ279526	Homo sapiens mRNA for immunoglobulin mu heavy chain variable region, partial, clone 2-E69.
								AJ279527	Homo sapiens mRNA for immunoglobulin mu heavy chain variable region, partial, clone 2-E75.
								AJ279529	Homo sapiens mRNA for immunoglobulin mu heavy chain variable region, partial, clone 1-F12.
								AJ279530	Homo sapiens mRNA for immunoglobulin mu heavy chain variable region, partial, clone 1-F21.
								AJ279531	Homo sapiens mRNA for immunoglobulin mu heavy chain variable region, partial, clone 1-F24.
								AJ279532	Homo sapiens mRNA for immunoglobulin mu heavy chain variable region, partial, clone 1-F26.
								AJ279534	Homo sapiens mRNA for immunoglobulin mu heavy chain variable region, partial, clone 1-F27.
								AJ279535	Homo sapiens mRNA for immunoglobulin mu heavy chain variable region, partial, clone 2-F39.
								AJ279536	Homo sapiens mRNA for immunoglobulin mu heavy chain variable region, partial, clone 2-F41.
								AJ279537	Homo sapiens mRNA for immunoglobulin mu heavy chain variable region, partial, clone 3-F41.
								AJ293739	Homo sapiens mRNA for immunoglobulin mu heavy chain variable region, partial, clone 3-F45.
								AJ298606	Homo sapiens mRNA for immunoglobulin mu heavy chain variable region, partial, clone 3-F46.
								AJ298607	Homo sapiens mRNA for immunoglobulin mu heavy chain variable region, partial, clone 3-F48.
								AJ298608	Homo sapiens mRNA for immunoglobulin mu heavy chain variable region, partial, clone 3-F49.
								AJ298609	Homo sapiens mRNA for immunoglobulin mu heavy chain variable region, partial, clone 3-F50.
								AJ298610	Homo sapiens mRNA for immunoglobulin gamma heavy chain variable region, partial, clone 1-D15.
								AJ298611	Homo sapiens mRNA for immunoglobulin gamma heavy chain variable region, partial, clone 1-D17.
								AJ300780	Homo sapiens mRNA for immunoglobulin gamma heavy chain variable region, partial, clone 1-D21.
								AJ300782	Homo sapiens mRNA for immunoglobulin gamma heavy chain variable region, partial, clone 1-D26.
								AJ300783	Homo sapiens mRNA for immunoglobulin gamma heavy chain variable region, partial, clone 1-D29.
								AJ300784	Homo sapiens mRNA for immunoglobulin gamma heavy chain variable region, partial, clone 2-D123.
								AJ300787	Homo sapiens mRNA for immunoglobulin gamma heavy chain variable region, partial, clone 2-D124.
								AJ300791	Homo sapiens mRNA for immunoglobulin gamma heavy chain variable region, partial, clone 2-D125.
								AJ300794	Homo sapiens mRNA for immunoglobulin gamma heavy chain variable region, partial, clone 2-D130.
								AJ300797	Homo sapiens mRNA for immunoglobulin gamma heavy chain variable region, partial, clone 1A-4G23.
								AJ300798	Homo sapiens mRNA for immunoglobulin gamma heavy chain variable region, partial, clone 2A-3G36.
								AJ300799	Homo sapiens mRNA for immunoglobulin gamma heavy chain variable region, partial, clone 2A-3G37.
								AJ300803	Homo sapiens mRNA for immunoglobulin gamma heavy chain variable region, partial, clone 2A-4G18.

								AJ300806	Homo sapiens mRNA for immunoglobulin gamma heavy chain variable region, partial, clone 2A-4G19.
								AJ300807	Homo sapiens mRNA for immunoglobulin gamma heavy chain variable region, partial, clone 2A-4G25.
								AJ300809	Homo sapiens mRNA for immunoglobulin gamma heavy chain variable region, partial, clone 2A-4G32.
								AJ305232	Homo sapiens mRNA for immunoglobulin gamma heavy chain variable region, partial, clone 2A-4G42.
								AJ306670	Homo sapiens mRNA for immunoglobulin gamma heavy chain variable region, partial, clone 2A-4G46.
								AJ306671	Homo sapiens mRNA for immunoglobulin gamma heavy chain variable region, partial, clone 2A-3G1.17.
								AJ306673	Homo sapiens mRNA for immunoglobulin mu heavy chain variable region, partial, clone 1A-3M2.
								AJ306675	Homo sapiens mRNA for immunoglobulin mu heavy chain variable region, partial, clone 1A-3M5.
								AJ306678	Homo sapiens mRNA for immunoglobulin mu heavy chain variable region, partial, clone 1A-3M17.
								AJ306838	Homo sapiens mRNA for immunoglobulin mu heavy chain variable region, partial, clone 1A-3M8.
								AJ308447	Homo sapiens mRNA for immunoglobulin mu heavy chain variable region, partial, clone 1A-3M11.
								AJ308463	Homo sapiens mRNA for immunoglobulin mu heavy chain variable region, partial, clone 1A-3M13.
								AJ309318	Homo sapiens mRNA for immunoglobulin mu heavy chain variable region, partial, clone 1A-3M20.
								AJ309825	Homo sapiens mRNA for immunoglobulin mu heavy chain variable region, partial, clone 1A-3M3.
								AJ309826	Homo sapiens mRNA for immunoglobulin mu heavy chain variable region, partial, clone 1A-3M14.
								AJ389171	Homo sapiens mRNA for immunoglobulin mu heavy chain variable region, partial, clone 1A-3M6.
								AJ389176	Homo sapiens mRNA for immunoglobulin mu heavy chain variable region, partial, clone 1A-3M35.
								AJ389178	Homo sapiens mRNA for immunoglobulin mu heavy chain variable region, partial, clone 1A-3M38.
								AJ389180	Homo sapiens mRNA for immunoglobulin mu heavy chain variable region, partial, clone 1A-3M39.
								AJ389182	Homo sapiens mRNA for immunoglobulin mu heavy chain variable region, partial, clone 1A-3M40.
								AJ389184	Homo sapiens mRNA for immunoglobulin mu heavy chain variable region, partial, clone 2A-3M19.
								AJ389188	Homo sapiens mRNA for immunoglobulin mu heavy chain variable region, partial, clone 2A-3M17.
								AJ389193	Homo sapiens mRNA for immunoglobulin mu heavy chain variable region, partial, clone 2A-3M33.
								AJ389194	Homo sapiens mRNA for immunoglobulin mu heavy chain variable region, partial, clone 2A-3M14.
								AJ389195	Homo sapiens mRNA for immunoglobulin mu heavy chain variable region, partial, clone 2A-3M16.
								AJ399801	Homo sapiens mRNA for immunoglobulin mu heavy chain variable region, partial, clone 2A-3M37.
								AJ399804	Homo sapiens mRNA for immunoglobulin mu heavy chain variable region, partial, clone 2A-3M30.
								AJ399831	Homo sapiens mRNA for immunoglobulin mu heavy chain variable region, partial, clone 2A-3M20.
								AJ413991	Homo sapiens mRNA for immunoglobulin mu heavy chain variable region, partial, clone 2A-3M35.
								AJ413992	Homo sapiens mRNA for immunoglobulin gamma heavy chain variable region, partial, clone 10B-3G13.
								AJ413993	Homo sapiens mRNA for immunoglobulin gamma heavy chain variable region, partial, clone 10B-3G18.
								AJ413994	Homo sapiens mRNA for immunoglobulin gamma heavy chain variable region, partial, clone 10B-3G1.12.
								AJ414005	Homo sapiens mRNA for immunoglobulin gamma heavy chain variable region, partial, clone 10B-3G1.6.
								AJ458383	Homo sapiens mRNA for immunoglobulin gamma heavy chain variable region, partial, clone 10B-3G1.15.
								AJ487489	Homo sapiens mRNA for immunoglobulin gamma heavy chain variable region, partial, clone 1A-3GP1.
								AJ487491	Homo sapiens mRNA for immunoglobulin gamma heavy chain variable region, partial, clone 1A-3GP4.
								AJ487492	Homo sapiens mRNA for immunoglobulin gamma heavy chain variable region, partial, clone 1A-3GP5.
								AJ488540	Homo sapiens mRNA for immunoglobulin gamma heavy chain variable region, partial, clone 1A-3GP6.
								AJ491904	Homo sapiens mRNA for immunoglobulin gamma heavy chain variable region, partial, clone 1A-3GP10.
								AJ491912	Homo sapiens mRNA for immunoglobulin gamma heavy chain variable region, partial, clone 1A-3GP20.
								AJ519222	Homo sapiens mRNA for immunoglobulin gamma heavy chain variable region, partial, clone 1A-3GP15.
								AJ519223	Homo sapiens mRNA for immunoglobulin gamma heavy chain variable region, partial, clone 1A-3GP16.
								AJ519227	Homo sapiens mRNA for immunoglobulin gamma heavy chain variable region, partial, clone 1A-3GP28.
								AJ519228	Homa sapiens mRNA for immunoglobulin heavy chain variable region (IGHV gene), ET6 anti-F(ab')2.
								AJ519236	Homo sapiens partial mRNA for immunoglobulin heavy chain (IGH2aM37 gene).
								AJ519238	Homo sapiens partial mRNA for immunoglobulin heavy chain (IGH1dM43a gene).
								AJ519239	Homo sapiens partial mRNA for immunoglobulin heavy chain (IGH2aM45 gene).
								AJ519240	Homo sapiens partial mRNA for immunoglobulin heavy chain (IGH4aA5 gene).
								AJ519241	Homo sapiens partial mRNA for immunoglobulin heavy chain (IGH4bM17 gene).
								AJ519242	Homo sapiens partial mRNA for immunoglobulin heavy chain (IGH5bA49a gene).
								AJ519243	Homo sapiens partial mRNA for immunoglobulin heavy chain (IGH5bA50 gene).
								AJ519244	Homo sapiens partial mRNA for immunoglobulin heavy chain (IGH5bM55 gene).
								AJ519247	Homo sapiens partial mRNA for immunoglobulin heavy chain (IGH6aM65 gene).
								AJ519248	Homo sapiens partial mRNA for immunoglobulin heavy chain (IGH5cM71 gene).

								AJ519250	Homo sapiens partial mRNA for immunoglobulin heavy chain (IGH5dM74 gene).
								AJ519252	Homo sapiens partial mRNA for immunoglobulin heavy chain (IGH5dA76 gene).
								AJ519257	Homo sapiens mRNA for immunoglobulin gamma heavy chain variable region, IGHV3-30 gene, clone MGgis13b.
								AJ519258	Homo sapiens mRNA for immunoglobulin gamma heavy chain variable region, IGHV1-02 gene, clone MGgis16b.
								AJ519260	Homo sapiens mRNA for immunoglobulin gamma heavy chain variable region, IGHV4-39 gene, clone MGgis26a.
								AJ519261	Homo sapiens mRNA for immunoglobulin mu heavy chain variable region IGHV3-23 gene, clone MMmis3.
								AJ519263	Homo sapiens mRNA for immunoglobulin mu heavy chain variable region IGHV3-15 gene, clone MMmis20.
								AJ519264	Homo sapiens mRNA for immunoglobulin mu heavy chain variable region IGHV3-21 gene, clone MMmis29.
								AJ519265	Homo sapiens mRNA for immunoglobulin mu heavy chain variable region IGHV3-23 gene, clone MMmis53.
								AJ519266	Homo sapiens mRNA for immunoglobulin mu heavy chain variable region IGHV4-39 gene, clone MMmis66.
								AJ519270	Homo sapiens mRNA for immunoglobulin mu heavy chain variable region IGHV3-23 gene, clone MMmis74.
								AJ519276	Homo sapiens partial mRNA for immunoglobulin gamma heavy chain variable region (IGHVDJ gene), patient BRE.
								AJ519277	Homo sapiens partial mRNA for immunoglobulin gamma heavy chain variable region (IGHVDJ gene), patient VES.
								AJ519279	Homo sapiens mRNA for immunoglobulin mu heavy chain variable region IGHV3-23 gene, clone MMmis117.
								AJ519281	Homo sapiens mRNA for immunoglobulin mu heavy chain variable region, partial, clone CD27low-3.
								AJ519288	Homo sapiens mRNA for immunoglobulin mu heavy chain variable region, partial, clone CD27low-4.
								AJ519290	Homo sapiens mRNA for immunoglobulin mu heavy chain variable region, partial, clone CD27low-5.
								AJ519292	Homo sapiens mRNA for immunoglobulin mu heavy chain variable region, partial, clone CD27low-6.
								AJ519295	Homo sapiens mRNA for immunoglobulin mu heavy chain variable region, partial, clone CD27low-8.
								AJ519296	Homo sapiens mRNA for immunoglobulin mu heavy chain variable region, partial, clone CD27low-12.
								AJ519302	Homo sapiens mRNA for immunoglobulin mu heavy chain variable region, partial, clone CD27low-13.
								AJ519303	Homo sapiens mRNA for immunoglobulin mu heavy chain variable region, partial, clone CD27high-2.
								AJ519304	Homo sapiens mRNA for immunoglobulin mu heavy chain variable region, partial, clone CD27high-5.
								AJ519307	Homo sapiens mRNA for immunoglobulin mu heavy chain variable region, partial, clone CD27high-7.
								AJ519308	Homo sapiens mRNA for immunoglobulin mu heavy chain variable region, partial, clone CD27high-10.
								AJ519311	Homo sapiens mRNA for immunoglobulin mu heavy chain variable region, partial, clone CD27high-11.
								AJ519313	Homo sapiens mRNA for immunoglobulin mu heavy chain variable region, partial, clone CD27high-13.
								AJ519316	Homo sapiens mRNA for immunoglobulin mu heavy chain variable region, partial, clone CD27high-15.
								AJ519317	Homo sapiens mRNA for immunoglobulin mu heavy chain variable region, partial, clone CD27high-18.
								AJ519318	Homo sapiens mRNA for immunoglobulin mu heavy chain variable region, partial, clone CD27high-19.
								AJ519319	Homo sapiens mRNA for immunoglobulin mu heavy chain variable region, partial, clone CD27high-20.
								AJ519320	Homo sapiens mRNA for immunoglobulin mu heavy chain variable region, partial, clone CD27high-22.
								AJ519321	Homo sapiens IGHV gene for immunoglobulin anti-GrB heavy chain variable region, clone GP91.
								AJ519322	Homo sapiens mRNA for immunoglobulin gamma heavy chain variable region (IGHV3-23 gene), clone shgi08, SLE patient.
								AJ519323	Homo sapiens mRNA for immunoglobulin gamma heavy chain variable region (IGHV3-23 gene), clone shgi21, SLE patient.
								AJ519326	Homo sapiens mRNA for immunoglobulin gamma heavy chain variable region (IGHV3-23 gene), clone shgi44, SLE patient.
								AJ519328	Homo sapiens mRNA for immunoglobulin gamma heavy chain variable region (IGHV3-23 gene), clone shgi51, SLE patient.
								AJ519329	Homo sapiens mRNA for immunoglobulin gamma heavy chain variable region (IGHV3-23 gene), clone shgi55, SLE patient.
								AJ519330	Homo sapiens mRNA for immunoglobulin gamma heavy chain variable region (IGHV3-23 gene), clone shgi58, SLE patient.
								AJ519331	Homo sapiens partial mRNA for immunoglobulin gamma heavy chain variable region (IGHV3-23), clone M1-11 (m1hie11),
								AJ519333	Kawasaki disease patient.
								AJ536051	Homo sapiens partial mRNA for immunoglobulin gamma heavy chain variable region (DP58/hv3d1EG), clone M1-22 (m1hie22),
								AJ548507	Kawasaki disease patient.
								AJ555251	Homo sapiens partial mRNA for immunoglobulin gamma heavy chain variable region (IGHV3-7), clone M1-28 (m1hie28),
								AJ555253	Kawasaki disease patient.
								AJ555256	Homo sapiens partial mRNA for immunoglobulin mu heavy chain variable region (IGHV3-23), clone M1-36(IgM) (m1hie36),
								AJ555257	Kawasaki disease patient.
								AJ555259	Homo sapiens partial mRNA for immunoglobulin gamma heavy chain variable region (IGHV3-30), clone M1-57 (m1hie57),
								AJ555260	Kawasaki disease patient.
								AJ555261	Homo sapiens partial mRNA for immunoglobulin gamma heavy chain variable region (IGHV3-20), clone M2-10 (m2hie10),
								AJ555262	Kawasaki disease patient.
								AJ555263	Homo sapiens partial mRNA for immunoglobulin gamma heavy chain variable region (IGHV3-23), clone M2-48 (m2hie48),
								AJ555264	Kawasaki disease patient.
								AJ555266	Homo sapiens partial mRNA for immunoglobulin gamma heavy chain variable region (IGHV3-30), clone M2-134 (m2hie134),

								AJ555270<br>AJ555272<br>AJ555273<br>AJ555274<br>AJ556638<br>AJ556646<br>AJ556648<br>AJ556650<br>AJ556658<br>AJ556664<br>AJ556669<br>AJ556676<br>AJ556677<br>AJ556684<br>AJ556685<br>AJ556691<br>AJ556696<br>AJ556711<br>AJ556714<br>AJ556715<br>AJ556745<br>AJ567669<br>AJ574777<br>AJ574779<br>AJ574781<br>AJ574786<br>AJ579095<br>AJ579097<br>AJ579098<br>AJ579099<br>AJ579102<br>AJ579107<br>AJ579110<br>AJ579111<br>AJ579112<br>AJ579113<br>AJ579115<br>AJ579116<br>AJ579120<br>AJ579121<br>AJ579123<br>AJ579126<br>AJ579128<br>AJ579132<br>AJ579134<br>AJ579135<br>AJ579136<br>AJ579137<br>AJ579140<br>AJ579141<br>AJ579142<br>AJ579144<br>AJ579145<br>AJ579146	Kawasaki disease patient.<br>Homo sapiens partial mRNA for immunoglobulin gamma heavy chain variable region (IGHV3-30), clone M2-142 (m2hie142), Kawasaki disease patient.<br>Homo sapiens partial mRNA for immunoglobulin gamma heavy chain variable region (IGHV4-59), clone M1u-04 (m1hoe04), Kawasaki disease patient.<br>Homo sapiens partial mRNA for immunoglobulin gamma heavy chain variable region (IGHV3-30), clone M1u-10 (m1hoe10), Kawasaki disease patient.<br>Homo sapiens partial mRNA for immunoglobulin gamma heavy chain variable region (IGHV3-15), clone M2u-05 (m2hoe05), Kawasaki disease patient.<br>Homo sapiens partial mRNA for immunoglobulin gamma heavy chain variable region (DP58/hv3d1EG), clone M2u-06 (m2hoe06), Kawasaki disease patient.<br>Homo sapiens partial mRNA for immunoglobulin gamma heavy chain variable region (IGHV4-39), clone M2u-10 (m2hoe10), Kawasaki disease patient.<br>Homo sapiens mRNA for immunoglobulin heavy chain variable region V(D)J-rearrangement (IGHV gene), patient 11.<br>Homo sapiens partial mRNA for anti-DNA immunoglobulin heavy chain variable region (IGHV-R4C15 gene).<br>Homo sapiens partial mRNA for anti-DNA immunoglobulin heavy chain variable region (IGHV-R4C17 gene).<br>Homo sapiens partial mRNA for anti-DNA immunoglobulin heavy chain variable region (IGHV-R4C4 gene).<br>Homo sapiens partial mRNA for anti-DNA immunoglobulin heavy chain variable region (IGHV-R4C9 gene).<br>Homo sapiens partial mRNA for anti-DNA immunoglobulin heavy chain variable region (IGHV-R5C6 gene).<br>Homo sapiens partial mRNA for anti-MVP monoclonal antibody heavy chain variable region, clone CES1.<br>Homo sapiens partial mRNA for immunoglobulin heavy chain variable region (IGHV1 gene), clone EBB10.<br>Homo sapiens partial mRNA for immunoglobulin heavy chain variable region (IGHV3 gene), clone TEG4.<br>Homo sapiens partial mRNA for anti-peptide/MHC complex HLA-A1/MAGE-A1 monoclonal antibody heavy chain.<br>Homo sapiens partial IGHV3 gene for immunoglobulin G heavy chain variable region.<br>Homo sapiens partial mRNA for variable part of immunoglobulin A heavy chain (IGVH3 gene).<br>Homo sapiens partial mRNA for immunoglobulin heavy chain variable region (IGVH gene), sample GN03.<br>Homo sapiens partial mRNA for immunoglobulin heavy chain variable region (IGVH gene), sample GN08.<br>Homo sapiens partial mRNA for immunoglobulin heavy chain variable region (IGVH gene), sample GN11.<br>Homo sapiens partial mRNA for immunoglobulin heavy chain variable region (IGVH gene), sample GN13.<br>Homo sapiens partial mRNA for immunoglobulin heavy chain variable region (IGVH gene), sample GN15.<br>Homo sapiens partial mRNA for immunoglobulin heavy chain variable region (IGVH gene), sample GN18.<br>Homo sapiens partial mRNA for immunoglobulin heavy chain variable region (IGVH gene), sample GN22.<br>Homo sapiens partial mRNA for immunoglobulin heavy chain variable region (IGVH gene), sample GN28.<br>Homo sapiens partial mRNA for immunoglobulin heavy chain variable region (IGVH gene), sample GN29.<br>Homo sapiens partial mRNA for immunoglobulin heavy chain variable region (IGVH gene), sample GN30.<br>Homo sapiens partial mRNA for thyroid peroxidase specific immunoglobulin heavy chain variable region (IGHV gene), clone A1.<br>Homo sapiens partial mRNA for thyroid peroxidase-specific immunoglobulin heavy chain variable region (IGHV gene), clone A4.<br>Homo sapiens partial mRNA for thyroid peroxidase-specific immunoglobulin heavy chain variable region (IGHV gene), clone T5.<br>Homo sapiens partial mRNA for immunoglobulin heavy chain variable region (IGVH gene), isolate CLL005.<br>Homo sapiens partial mRNA for immunoglobulin heavy chain variable region (IGVH gene), isolate CLL006.<br>Homo sapiens partial mRNA for immunoglobulin heavy chain variable region (IGVH gene), isolate CLL007.<br>Homo sapiens partial mRNA for immunoglobulin heavy chain variable region (IGVH gene), isolate CLL008.<br>Homo sapiens partial mRNA for immunoglobulin heavy chain variable region (IGVH gene), isolate CLL019.<br>Homo sapiens partial mRNA for immunoglobulin heavy chain variable region (IGHV gene), clone 60.<br>Homo sapiens partial mRNA for immunoglobulin heavy chain variable region (IGHV gene), clone slvl5.<br>Homo sapiens partial mRNA for immunoglobulin heavy chain variable region (IGHV gene), clone slvl7.<br>Homo sapiens partial mRNA for immunoglobulin heavy chain variable region (IGHV gene), clone slvl8.<br>Homo sapiens partial mRNA for immunoglobulin heavy chain variable region (IGHV gene), patient WM3(WS).<br>Homo sapiens mRNA for immunoglobulin heavy chain V-D-J-Ce region (clone SO16 C2).<br>Homo sapiens mRNA for immunoglobulin heavy chain V-D-J-Ce region (clone HD17 C37).<br>Homo sapiens partial mRNA for IgA1 immunoglobulin heavy chain variable region (IGHV gene), clone AN2PA448.<br>Homo sapiens partial mRNA for IgA1 immunoglobulin heavy chain variable region (IGHV gene), clone AN2PA506.<br>Homo sapiens partial mRNA for IgA1 immunoglobulin heavy chain variable region (IGHV gene), clone AN2PA521.

								AJ579149	Homo sapiens partial mRNA for IgA1 immunoglobulin heavy chain variable region (IGHV gene), clone AN2PA526.
								AJ579150	Homo sapiens partial mRNA for IgG2 immunoglobulin heavy chain variable region (IGHV gene), clone AN2PG481.
								AJ579151	Homo sapiens partial mRNA for IgG2 immunoglobulin heavy chain variable region (IGHV gene), clone AN2PG497.
								AJ579154	Homo sapiens partial mRNA for IgG2 immunoglobulin heavy chain variable region (IGHV gene), clone AN2PG549.
								AJ579155	Homo sapiens partial mRNA for IgG2 immunoglobulin heavy chain variable region (IGHV gene), clone AN2PG553.
								AJ579156	Homo sapiens partial mRNA for IgG2 immunoglobulin heavy chain variable region (IGHV gene), clone AN2PG557.
								AJ579157	Homo sapiens partial mRNA for IgG1 immunoglobulin heavy chain variable region (IGHV gene), clone AN2PG558.
								AJ579158	Homo sapiens partial mRNA for IgG1 immunoglobulin heavy chain variable region (IGHV gene), clone AN2PG566.
								AJ579160	Homo sapiens partial mRNA for IgG1 immunoglobulin heavy chain variable region (IGHV gene), clone AN2PG568.
								AJ579162	Homo sapiens partial mRNA for IgM immunoglobulin heavy chain variable region (IGHV gene), clone AN2PM454.
								AJ579169	Homo sapiens partial mRNA for IgM immunoglobulin heavy chain variable region (IGHV gene), clone AN2PM456.
								AJ579170	Homo sapiens partial mRNA for IgM immunoglobulin heavy chain variable region (IGHV gene), clone AN2PM488.
								AJ579171	Homo sapiens partial mRNA for IgM immunoglobulin heavy chain variable region (IGHV gene), clone AN2PM510.
								AJ579172	Homo sapiens partial mRNA for IgA1 immunoglobulin heavy chain variable region (IGHV gene), clone ANBPA226.
								AJ579173	Homo sapiens partial mRNA for IgA2 immunoglobulin heavy chain variable region (IGHV gene), clone ANBPA228.
								AJ579174	Homo sapiens partial mRNA for IgA1 immunoglobulin heavy chain variable region (IGHV gene), clone ANBPA234.
								AJ579176	Homo sapiens partial mRNA for IgA1 immunoglobulin heavy chain variable region (IGHV gene), clone ANBPA310.
								AJ579177	Homo sapiens partial mRNA for IgA1 immunoglobulin heavy chain variable region (IGHV gene), clone ANBPA371.
								AJ579180	Homo sapiens partial mRNA for IgA1 immunoglobulin heavy chain variable region (IGHV gene), clone ANBPA374.
								AJ579183	Homo sapiens partial mRNA for IgG1 immunoglobulin heavy chain variable region (IGHV gene), clone ANBPG257.
								AJ579184	Homo sapiens partial mRNA for IgG1 immunoglobulin heavy chain variable region (IGHV gene), clone ANBPG258.
								AJ579186	Homo sapiens partial mRNA for IgG2 immunoglobulin heavy chain variable region (IGHV gene), clone ANBPG270.
								AJ579188	Homo sapiens partial mRNA for IgG1 immunoglobulin heavy chain variable region (IGHV gene), clone ANBPG285.
								AJ579190	Homo sapiens partial mRNA for IgG1 immunoglobulin heavy chain variable region (IGHV gene), clone ANBPG286.
								AJ579192	Homo sapiens partial mRNA for IgG2 immunoglobulin heavy chain variable region (IGHV gene), clone ANBPG289.
								AJ579193	Homo sapiens partial mRNA for IgG1 immunoglobulin heavy chain variable region (IGHV gene), clone ANBPG296.
								AJ579195	Homo sapiens partial mRNA for IgM immunoglobulin heavy chain variable region (IGHV gene), clone ANBPM208.
								AJ579196	Homo sapiens partial mRNA for IgM immunoglobulin heavy chain variable region (IGHV gene), clone ANBPM220.
								AJ579198	Homo sapiens partial mRNA for IgM immunoglobulin heavy chain variable region (IGHV gene), clone ANBPM223.
								AJ579200	Homo sapiens partial mRNA for IgM immunoglobulin heavy chain variable region (IGHV gene), clone ANBPM229.
								AJ579201	Homo sapiens partial mRNA for IgM immunoglobulin heavy chain variable region (IGHV gene), clone ANBPM230.
								AJ579202	Homo sapiens partial mRNA for IgA2 immunoglobulin heavy chain variable region (IGHV gene), clone LIBPA217.
								AJ579204	Homo sapiens partial mRNA for IgA1 immunoglobulin heavy chain variable region (IGHV gene), clone LIBPA219.
								AJ579205	Homo sapiens partial mRNA for IgA1 immunoglobulin heavy chain variable region (IGHV gene), clone LIBPA225.
								AJ579206	Homo sapiens partial mRNA for IgA1 immunoglobulin heavy chain variable region (IGHV gene), clone LIBPA232.
								AJ579207	Homo sapiens partial mRNA for IgA2 immunoglobulin heavy chain variable region (IGHV gene), clone LIBPA234.
								AJ579210	Homo sapiens partial mRNA for IgA1 immunoglobulin heavy chain variable region (IGHV gene), clone LIBPA352.
								AJ579211	Homo sapiens partial mRNA for IgG2 immunoglobulin heavy chain variable region (IGHV gene), clone LIBPG316.
								AJ579214	Homo sapiens partial mRNA for IgG2 immunoglobulin heavy chain variable region (IGHV gene), clone LIBPG372.
								AJ579215	Homo sapiens partial mRNA for IgM immunoglobulin heavy chain variable region (IGHV gene), clone LIBPM202.
								AJ579216	Homo sapiens partial mRNA for IgM immunoglobulin heavy chain variable region (IGHV gene), clone LIBPM204.
								AJ579217	Homo sapiens partial mRNA for IgM immunoglobulin heavy chain variable region (IGHV gene), clone LIBPM205.
								AJ579218	Homo sapiens partial mRNA for IgM immunoglobulin heavy chain variable region (IGHV gene), clone LIBPM212.
								AJ579219	Homo sapiens partial mRNA for IgM immunoglobulin heavy chain variable region (IGHV gene), clone LIBPM213.
								AJ579220	Homo sapiens partial mRNA for IgM immunoglobulin heavy chain variable region (IGHV gene), clone LIBPM214.
								AJ579221	Homo sapiens partial mRNA for IgM immunoglobulin heavy chain variable region (IGHV gene), clone LIBPM253.
								AJ579222	Homo sapiens partial mRNA for IgM immunoglobulin heavy chain variable region (IGHV gene), clone LIBPM261.
								AJ579223	Homo sapiens partial mRNA for IgM immunoglobulin heavy chain variable region (IGHV gene), clone LIBPM265.
								AJ579225	Homo sapiens partial mRNA for IgM immunoglobulin heavy chain variable region (IGHV gene), clone LIBPM267.
								AJ579226	Homo sapiens partial mRNA for IgM immunoglobulin heavy chain variable region (IGHV gene), clone LIBPM272.
								AJ579227	Homo sapiens partial mRNA for IgM immunoglobulin heavy chain variable region (IGHV gene), clone LIBPM317.
								AJ579228	Homo sapiens partial mRNA for IgM immunoglobulin heavy chain variable region (IGHV gene), clone LIBPM322.
								AJ579229	Homo sapiens partial mRNA for immunoglobulin heavy chain variable region (IGHV gene), case 2.
								AJ579230	Homo sapiens partial mRNA for immunoglobulin heavy chain variable region (IGHV gene), clone RN86.

								AJ579233	Homo sapiens partial mRNA for immunoglobulin heavy chain variable region (IGHV gene), isolate B-CLL G002.
								AJ579234	Homo sapiens partial mRNA for immunoglobulin heavy chain variable region (IGHV gene), isolate B-CLL G004.
								AJ579235	Homo sapiens partial mRNA for immunoglobulin heavy chain variable region (IGHV gene), isolate B-CLL G007.
								AJ579236	Homo sapiens partial mRNA for immunoglobulin heavy chain variable region (IGHV gene), isolate B-CLL G008.
								AJ579237	Homo sapiens partial mRNA for immunoglobulin heavy chain variable region (IGHV gene), isolate B-CLL G010.
								AJ579238	Homo sapiens partial mRNA for immunoglobulin heavy chain variable region (IGHV gene), isolate B-CLL G011.
								AJ579239	Homo sapiens partial mRNA for immunoglobulin heavy chain variable region (IGHV gene), isolate B-CLL G012.
								AJ579245	Homo sapiens partial mRNA for immunoglobulin heavy chain variable region (IGHV gene), isolate B-CLL G013.
								AJ579249	Homo sapiens partial mRNA for immunoglobulin heavy chain variable region (IGHV gene), isolate B-CLL G014.
								AJ579252	Homo sapiens partial mRNA for immunoglobulin heavy chain variable region (IGHV gene), isolate B-CLL G015.
								AJ579254	Homo sapiens partial mRNA for immunoglobulin heavy chain variable region (IGHV gene), isolate B-CLL G017.
								AJ579256	Homo sapiens partial mRNA for immunoglobulin heavy chain variable region (IGHV gene), isolate B-CLL G021.
								AJ579259	Homo sapiens partial mRNA for immunoglobulin heavy chain variable region (IGHV gene), isolate B-CLL G023.
								AJ579263	Homo sapiens partial mRNA for immunoglobulin heavy chain variable region (IGHV gene), isolate B-CLL G024.
								AJ579268	Homo sapiens partial mRNA for immunoglobulin heavy chain variable region (IGHV gene), isolate B-CLL G025.
								AJ579269	Homo sapiens partial mRNA for immunoglobulin gamma heavy chain variable region (IGHV3-07 gene), clone 1A-3G 04.
								AJ579270	Homo sapiens partial mRNA for immunoglobulin gamma heavy chain variable region (IGHV 3-07 gene), clone 1A-3G 015.
								AJ579271	Homo sapiens partial mRNA for immunoglobulin gamma heavy chain variable region (IGHV3-33 gene), clone 1A- 3G 017.
								AJ579273	Homo sapiens partial mRNA for immunoglobulin gamma heavy chain variable region (IGHV3-07 gene), clone 1A-3G 019.
								AJ579275	Homo sapiens partial mRNA for immunoglobulin gamma heavy chain variable region (IGHV3-07 gene), clone 1A-3G 26.
								AJ579277	Homo sapiens partial mRNA for immunoglobulin gamma heavy chain variable region (IGHV3-07 gene), clone 1A 3G 30.
								AJ579278	Homo sapiens partial mRNA for immunoglobulin gamma heavy chain variable region (IGHV3-07 gene), clone 1A-3G 37.
								AJ579279	Homo sapiens partial mRNA for immunoglobulin gamma heavy chain variable region (IGHV2-1+ gene), clone 1A-4G 09.
								AJ579280	Homo sapiens partial mRNA for immunoglobulin gamma heavy chain variable region (IGHV4-30.1 gene), clone 1A-4G 010.
								AJ579283	Homo sapiens partial mRNA for immunoglobulin gamma heavy chain variable region (IGHV4-04 gene), clone 1A-4G 019.
								AJ579284	Homo sapiens partial mRNA for immunoglobulin gamma heavy chain variable region (IGHV4-30.1 gene), clone 1A-4G 020.
								AJ579285	Homo sapiens partial mRNA for immunoglobulin gamma heavy chain variable region (IGHV4-39 gene), clone 1A-4G 7.
								AJ579292	Homo sapiens partial mRNA for immunoglobulin gamma heavy chain variable region (IGHV4-30.2 gene), clone 1A-4G 20.
								AJ579294	Homo sapiens partial mRNA for immunoglobulin gamma heavy chain variable region (IGHV3-30.3 gene), clone 2B-3G 035.
								AJ579300	Homo sapiens partial mRNA for immunoglobulin gamma heavy chain variable region (IGHV3-30.3 gene), clone 2B 3G 014.
								AJ579301	Homo sapiens partial mRNA for immunoglobulin gamma heavy chain variable region (IGHV3-30.3 gene), clone 2B-3G 030.
								AJ579302	Homo sapiens partial mRNA for immunoglobulin gamma heavy chain variable region (IGHV4-28 gene), clone 2B-4G 09.
								AJ579303	Homo sapiens mRNA for switch circle transcript arising from IgG to IgE class switch recombination (clone AA01).
								AJ006648	Homo sapiens partial mRNA for Immunoglobulin Heavy Chain Variable region (IGHV gene), isolate G030.
								X77017	Homo sapiens partial mRNA for Immunoglobulin Heavy Chain Variable region (IGHV gene), isolate G032.
								Y12699	Homo sapiens partial mRNA for Immunoglobulin Heavy Chain Variable region (IGHV gene), isolate G034.
								Z37152	Homo sapiens partial mRNA for Immunoglobulin Heavy Chain Variable region (IGHV gene), isolate G029.
								X99992	Homo sapiens partial mRNA for IgM immunoglobulin heavy chain variable region (IGHV3-23 gene), clone T1.3.2.
								Z35120	Homo sapiens partial mRNA for IgM immunoglobulin heavy chain variable region (IGHV3-30 gene), clone T1.3.4.
								Z35126	Homo sapiens partial mRNA for IgM immunoglobulin heavy chain variable region (IGHV3-30 gene), clone T1.3.5.
								AJ222559	Homo sapiens partial mRNA for IgM immunoglobulin heavy chain variable region (IGHV3-30 gene), clone T1.3.6.
								AJ222562	Homo sapiens partial mRNA for IgM immunoglobulin heavy chain variable region (IGHV3-23 gene), clone T1.3.9.
								AJ222551	Homo sapiens partial mRNA for IgM immunoglobulin heavy chain variable region (IGHV3-33 gene), clone T1.3.14.
								AJ222552	Homo sapiens partial mRNA for IgM immunoglobulin heavy chain variable region (IGHV3-33 gene), clone T1.3.19.
								AJ222554	Homo sapiens partial mRNA for IgM immunoglobulin heavy chain variable region (IGHV3-74 gene), clone T1.3.20.
								AJ222557	Homo sapiens partial mRNA for IgM immunoglobulin heavy chain variable region (IGHV3-23 gene), clone T1.3.21.
								AJ222558	Homo sapiens partial mRNA for IgM immunoglobulin heavy chain variable region (IGHV3-23 gene), clone T1.3.22.
								AJ222563	Homo sapiens partial mRNA for IgM immunoglobulin heavy chain variable region (IGHV3-15 gene), clone T1.3.24.
								AJ222564	Homo sapiens partial mRNA for IgM immunoglobulin heavy chain variable region (IGHV4-30 gene), clone T1.4.25.
								AJ222565	Homo sapiens partial mRNA for IgM immunoglobulin heavy chain variable region (IGHV4-61 gene), clone T1.4.29.
								AJ222566	Homo sapiens partial mRNA for IgM immunoglobulin heavy chain variable region (IGHV4-34 gene), clone T1.4.30.
								AJ222575	Homo sapiens partial mRNA for IgM immunoglobulin heavy chain variable region (IGHV4-4 gene), clone T1.4.32.
								AJ224083	Homo sapiens partial mRNA for IgM immunoglobulin heavy chain variable region (IGHV4-39 gene), clone T1.4.35.
								AJ224899	Homo sapiens partial mRNA for IgM immunoglobulin heavy chain variable region (IGHV4-4 gene), clone T1.4.37.

								AJ008181	Homo sapiens partial mRNA for IgM immunoglobulin heavy chain variable region (IGHV4-61 gene), clone T1.4.42.
								AJ008185	Homo sapiens partial mRNA for IgM immunoglobulin heavy chain variable region (IGHV4-39 gene), clone T1.4.44.
								AJ008257	Homo sapiens partial mRNA for IgM immunoglobulin heavy chain variable region (IGHV4-34 gene), clone T1.4.46.
								Y09390	Homo sapiens partial mRNA for IgM immunoglobulin heavy chain variable region (IGHV4-39 gene), clone T1.4.47.
								Y18126	Homo sapiens partial mRNA for IgM immunoglobulin heavy chain variable region (IGHV4-4 gene), clone T1.4.48.
								X81723	Homo sapiens partial mRNA for IgM immunoglobulin heavy chain variable region (IGHV3-15 gene), clone T2.3.2.
								X81724	Homo sapiens partial mRNA for IgM immunoglobulin heavy chain variable region (IGHV3-7 gene), clone T2.3.3.
								X81726	Homo sapiens partial mRNA for IgM immunoglobulin heavy chain variable region (IGHV3-23 gene), clone T2.3.4.
								X81728	Homo sapiens partial mRNA for IgM immunoglobulin heavy chain variable region (IGHV3-30.3 gene), clone T2.3.6.
								X81729	Homo sapiens partial mRNA for IgM immunoglobulin heavy chain variable region (IGHV3-30.3 gene), clone T2.3.7.
								X81730	Homo sapiens partial mRNA for IgM immunoglobulin heavy chain variable region (IGHV3-30.3 gene), clone T2.3.8.
								X81731	Homo sapiens partial mRNA for IgM immunoglobulin heavy chain variable region (IGHV3-9 gene), clone T2.3.11.
								X81732	Homo sapiens partial mRNA for IgM immunoglobulin heavy chain variable region (IGHV3-74 gene), clone T2.3.12.
								X81733	Homo sapiens partial mRNA for IgM immunoglobulin heavy chain variable region (IGHV3-30.3 gene), clone T2.3.13.
								X81734	Homo sapiens partial mRNA for IgM immunoglobulin heavy chain variable region (IGHV3-30 gene), clone T2.3.16.
								X81735	Homo sapiens partial mRNA for IgM immunoglobulin heavy chain variable region (IGHV3-11 gene), clone T2.3.17.
								X87442	Homo sapiens partial mRNA for IgM immunoglobulin heavy chain variable region (IGHV3-9 gene), clone T2.3.18.
								X87444	Homo sapiens partial mRNA for IgM immunoglobulin heavy chain variable region (IGHV3-23 gene), clone T2.3.19.
								X87887	Homo sapiens partial mRNA for IgM immunoglobulin heavy chain variable region (IGHV3-30 gene), clone T2.3.20.
								X64149	Homo sapiens partial mRNA for IgM immunoglobulin heavy chain variable region (IGHV3-15 gene), clone T2.3.22.
								Z46348	Homo sapiens partial mRNA for IgM immunoglobulin heavy chain variable region (IGHV3-7 gene), clone T2.3.24.
								Z46379	Homo sapiens partial mRNA for IgM immunoglobulin heavy chain variable region (IGHV4-39 gene), clone T2.4.34.
								X87447	Homo sapiens partial mRNA for IgM immunoglobulin heavy chain variable region (IGHV4-39 gene), clone T2.4.35.
								Z70609	Homo sapiens partial mRNA for IgM immunoglobulin heavy chain variable region (IGHV4-30.4 gene), clone T2.4.36.
								Z70607	Homo sapiens partial mRNA for IgM immunoglobulin heavy chain variable region (IGHV4-30.1 gene), clone T2.4.37.
								Z70606	Homo sapiens partial mRNA for IgM immunoglobulin heavy chain variable region (IGHV4-30.4 gene), clone T2.4.38.
								Z70604	Homo sapiens partial mRNA for IgM immunoglobulin heavy chain variable region (IGHV4-30.4 gene), clone T2.4.40.
								Z70605	Homo sapiens partial mRNA for IgM immunoglobulin heavy chain variable region (IGHV4-34 gene), clone T2.4.42.
								X57415	Homo sapiens partial mRNA for IgM immunoglobulin heavy chain variable region (IGHV4-30.2 gene), clone T2.4.45.
								X87891	Homo sapiens partial mRNA for IgM immunoglobulin heavy chain variable region (IGHV3-15 gene), clone T3.3.31.
								Z14200	Homo sapiens partial mRNA for IgM immunoglobulin heavy chain variable region (IGHV3-43 gene), clone T3.3.35.
								Z14212	Homo sapiens partial mRNA for IgM immunoglobulin heavy chain variable region (IGHV3-23 gene), clone T3.3.37.
								Z14201	Homo sapiens partial mRNA for IgM immunoglobulin heavy chain variable region (IGHV3-73 gene), clone T3.3.39.
								Z14202	Homo sapiens partial mRNA for IgM immunoglobulin heavy chain variable region (IGHV3-23 gene), clone T3.3.42.
								Z14204	Homo sapiens partial mRNA for IgM immunoglobulin heavy chain variable region (IGHV3-11 gene), clone T3.3.44.
								Z14205	Homo sapiens partial mRNA for IgM immunoglobulin heavy chain variable region (IGHV3-73 gene), clone T3.3.47.
								Z14206	Homo sapiens partial mRNA for IgM immunoglobulin heavy chain variable region (IGHV3-30 gene), clone T3.3.48.
								Z14194	Homo sapiens partial mRNA for IgM immunoglobulin heavy chain variable region (IGHV3-23 gene), clone T3.3.51.
								Z14195	Homo sapiens partial mRNA for IgM immunoglobulin heavy chain variable region (IGHV3-23 gene), clone T3.3.53.
								Z14196	Homo sapiens partial mRNA for IgM immunoglobulin heavy chain variable region (IGHV3-7 gene), clone T3.3.55.
								Z14198	Homo sapiens partial mRNA for IgM immunoglobulin heavy chain variable region (IGHV4-30 gene), clone T3.4.61.
								Z14199	Homo sapiens partial mRNA for IgM immunoglobulin heavy chain variable region (IGHV4-34 gene), clone T3.4.62.
								Z14211	Homo sapiens partial mRNA for IgM immunoglobulin heavy chain variable region (IGHV4-30 gene), clone T3.4.63.
								Z14165	Homo sapiens partial mRNA for IgM immunoglobulin heavy chain variable region (IGHV4-4 gene), clone T3.4.65.
								Z14166	Homo sapiens partial mRNA for IgM immunoglobulin heavy chain variable region (IGHV4-30 gene), clone T3.4.66.
								Z14167	Homo sapiens partial mRNA for IgM immunoglobulin heavy chain variable region (IGHV4-34 gene), clone T3.4.68.
								Z14170	Homo sapiens partial mRNA for IgM immunoglobulin heavy chain variable region (IGHV4-b gene), clone T3.4.70.
								Z14169	Homo sapiens partial mRNA for IgM immunoglobulin heavy chain variable region (IGHV4-30 gene), clone T3.4.73.
								Z14189	Homo sapiens partial mRNA for IgM immunoglobulin heavy chain variable region (IGHV4-34 gene), clone T3.4.74.
								Z14171	Homo sapiens partial mRNA for IgM immunoglobulin heavy chain variable region (IGHV4-30 gene), clone T3.4.81.
								Z14172	Homo sapiens partial mRNA for IgM immunoglobulin heavy chain variable region (IGHV3-9 gene), clone T4.3.1(27).
								Z14173	Homo sapiens partial mRNA for IgM immunoglobulin heavy chain variable region (IGHV3-7 gene), clone T4.3.2(27).
								Z14174	Homo sapiens partial mRNA for IgM immunoglobulin heavy chain variable region (IGHV3-7 gene), clone T4.3.3(27).
								Z14175	Homo sapiens partial mRNA for IgM immunoglobulin heavy chain variable region (IGHV3-33 gene), clone T4.3.4(27).

								Z14176<br>Z14192<br>Z14177<br>Z14178<br>Z14190<br>Z14179<br>Z14180<br>Z14181<br>Z14182<br>Z14184<br>Z14185<br>Z14186<br>Z14188<br>Z14158<br>Z14161<br>Z14163<br>Z14164<br>Z46277<br>Y08143<br>Y15773<br>X64153<br>X94432<br>X64158<br>X98899<br>X98902<br>X94058<br>Z33898<br>Z33899<br>Z33900<br>Y11323<br>X59702<br>X59703<br>X64154<br>X77472<br>X63080<br>X63081<br>X84336<br>X84338<br>X84339<br>X59134<br>X61021<br>X61011<br>X61013<br>X61014<br>X61023<br>Y09388<br>Y09386<br>X83965<br>Z49973<br>Y14735<br>Y14737<br>X97483<br>X81695<br>X81696	Homo sapiens partial mRNA for IgM immunoglobulin heavy chain variable region (IGHV3-15 gene), clone T4.3.5(27).<br>Homo sapiens partial mRNA for IgM immunoglobulin heavy chain variable region (IGHV3-30 gene), clone T4.3.6(27).<br>Homo sapiens partial mRNA for IgM immunoglobulin heavy chain variable region (IGHV3-23 gene), clone T4.3.7(27).<br>Homo sapiens partial mRNA for IgM immunoglobulin heavy chain variable region (IGHV3-74 gene), clone T4.3.9(27).<br>Homo sapiens partial mRNA for IgM immunoglobulin heavy chain variable region (IGHV3-11 gene), clone T4.3.11(27).<br>Homo sapiens partial mRNA for IgM immunoglobulin heavy chain variable region (IGHV3-23 gene), clone T4.3.13(27).<br>Homo sapiens partial mRNA for IgM immunoglobulin heavy chain variable region (IGHV3-23 gene), clone T4.3.14(27).<br>Homo sapiens partial mRNA for IgM immunoglobulin heavy chain variable region (IGHV3-15 gene), clone T4.3.15(27).<br>Homo sapiens partial mRNA for IgM immunoglobulin heavy chain variable region (IGHV3-23 gene), clone T4.3.17(27).<br>Homo sapiens partial mRNA for IgM immunoglobulin heavy chain variable region (IGHV3-9 gene), clone T4.3.19(27).<br>Homo sapiens partial mRNA for IgM immunoglobulin heavy chain variable region (IGHV3-30 gene), clone T4.3.20(27).<br>Homo sapiens partial mRNA for IgM immunoglobulin heavy chain variable region (IGHV3-7 gene), clone T4.3.23(27).<br>Homo sapiens partial mRNA for IgM immunoglobulin heavy chain variable region (IGHV3-7 gene), clone T4.3.24(27).<br>Homo sapiens partial mRNA for IgM immunoglobulin heavy chain variable region (IGHV3-15 gene), clone T4.3.26(27).<br>Homo sapiens partial mRNA for IgM immunoglobulin heavy chain variable region (IGHV3-23 gene), clone T4.3.27(27).<br>Homo sapiens partial mRNA for IgM immunoglobulin heavy chain variable region (IGHV3-11 gene), clone T4.3.28(27).<br>Homo sapiens partial mRNA for IgM immunoglobulin heavy chain variable region (IGHV3-23 gene), clone T4.3.29(27).<br>Homo sapiens partial mRNA for IgM immunoglobulin heavy chain variable region (IGHV3-7 gene), clone T4.3.30(27).<br>Homo sapiens partial mRNA for IgM immunoglobulin heavy chain variable region (IGHV4-39 gene), clone T4.4.36(27).<br>Homo sapiens partial mRNA for IgM immunoglobulin heavy chain variable region (IGHV4-34 gene), clone T4.4.40(27).<br>Homo sapiens partial mRNA for IgM immunoglobulin heavy chain variable region (IGHV4-59 gene), clone T4.4.43(27).<br>Homo sapiens partial mRNA for IgM immunoglobulin heavy chain variable region (IGHV4-61 gene), clone T4.4.45(27).<br>Homo sapiens partial mRNA for IgM immunoglobulin heavy chain variable region (IGHV4-34 gene), clone T4.4.50(27).<br>Homo sapiens partial mRNA for IgM immunoglobulin heavy chain variable region (IGHV4-34 gene), clone T4.4.54(27).<br>Homo sapiens partial mRNA for IgM immunoglobulin heavy chain variable region (IGHV3-15 gene), clone T5.3.3(27).<br>Homo sapiens partial mRNA for IgM immunoglobulin heavy chain variable region (IGHV3-7 gene), clone T5.3.10(27).<br>Homo sapiens partial mRNA for IgM immunoglobulin heavy chain variable region (IGHV3-11 gene), clone T5.3.11(27).<br>Homo sapiens partial mRNA for IgM immunoglobulin heavy chain variable region (IGHV3-23 gene), clone T5.3.14(27).<br>Homo sapiens partial mRNA for IgM immunoglobulin heavy chain variable region (IGHV3-23 gene), clone T5.3.15(27).<br>Homo sapiens partial mRNA for IgM immunoglobulin heavy chain variable region (IGHV3-13 gene), clone T5.3.17(27).<br>Homo sapiens partial mRNA for IgM immunoglobulin heavy chain variable region (IGHV3-15 gene), clone T5.3.19(27).<br>Homo sapiens partial mRNA for IgM immunoglobulin heavy chain variable region (IGHV3-23 gene), clone T5.3.21(27).<br>Homo sapiens partial mRNA for IgM immunoglobulin heavy chain variable region (IGHV3-23 gene), clone T5.3.22(27).<br>Homo sapiens partial mRNA for IgM immunoglobulin heavy chain variable region (IGHV3-30 gene), clone T5.3.23(27).<br>Homo sapiens partial mRNA for IgM immunoglobulin heavy chain variable region (IGHV3-23 gene), clone T5.3.24(27).<br>Homo sapiens partial mRNA for IgM immunoglobulin heavy chain variable region (IGHV3-23 gene), clone T5.3.27(27).<br>Homo sapiens partial mRNA for IgM immunoglobulin heavy chain variable region (IGHV3-30 gene), clone T5.3.29(27).<br>Homo sapiens partial mRNA for IgM immunoglobulin heavy chain variable region (IGHV3-15 gene), clone T5.3.30(27).<br>Homo sapiens partial mRNA for IgM immunoglobulin heavy chain variable region (IGHV4-39 gene), clone T5.4.44(27).<br>Homo sapiens partial mRNA for IgM immunoglobulin heavy chain variable region (IGHV4-61 gene), clone T5.4.47(27).<br>Homo sapiens partial mRNA for IgM immunoglobulin heavy chain variable region (IGHV4-30.1 gene), clone T5.4.54(27).<br>Homo sapiens partial mRNA for IgM immunoglobulin heavy chain variable region (IGHV4-34 gene), clone T5.4.55(27).<br>Homo sapiens partial mRNA for IgM immunoglobulin heavy chain variable region (IGHV4-34 gene), clone T5.4.57(27).<br>Homo sapiens partial mRNA for IgM immunoglobulin heavy chain variable region (IGHV4-39 gene), clone T5.4.59(27).<br>Homo sapiens mRNA for IgG heavy chain variable region, clone ac.r.4, partial.<br>H.sapiens mRNA for anti-acetylcholine receptor monoclonal autoantibody Vh region.<br>H.sapiens mRNA for anti-idiotypic antibody, variable heavy chain.<br>H.sapiens rearranged anti-Desmosome antibody immunoglobulin heavy-chain variable region.<br>H.sapiens mRNA for anti folate binding protein, HuVH8B VH.<br>H.sapiens mRNA for monoclonal antibody to gp41 HIV-1, heavy chain V region.<br>H.sapiens mRNA for monoclonal antibody to gp120 HIV-1, heavy chain V region.<br>Homo sapiens mRNA for IgG, clone GD2.<br>Homo sapiens mRNA for IgG, clone GD6.<br>Homo sapiens mRNA for IgA, clone AF6.

								X95661	Homo sapiens mRNA for IgA, clone AG6.
								X80305	Homo sapiens mRNA for IgG, clone GA4.
								X95740	Homo sapiens mRNA for IgG, clone GC2.
								X69861	Homo sapiens mRNA for IgG, clone GC5.
								X69862	Homo sapiens mRNA for IgG, clone GE1.
								X69863	Homo sapiens mRNA for IgG, clone GG3.
								X69866	Homo sapiens mRNA for IgG, clone GG5.
								X17676	Homo sapiens mRNA for IgG, clone GG6.
								Z18822	Homo sapiens mRNA for IgM, clone MF3.
								Z18832	Homo Sapiens heavy chain variable region cDNA.
								Z18834	Homo sapiens mRNA for rearranged IgM variable region, heavy chain (B7 VH).
								Z18843	Homo sapiens mRNA for immunoglobulin heavy chain, VHDJH rearrangement : VHLI22.
								Z18846	Homo sapiens mRNA for immunoglobulin heavy chain, VHDJH rearrangement : VHLI27.
								Z18851	Homo sapiens mRNA for immunoglobulin heavy chain, VHDJH rearrangement : VHUW13.
								X65883	H.sapiens mRNA for variable immunoglobulin anti-steroid, heavy chain, gene family VH4.
								X65893	Homo sapiens mRNA for immunoglobulin B3 variable chain heavy region.
								X65895	H.sapiens rearranged VDJ region (BEL1).
								X65896	H.sapiens rearranged VDJ region (BEL14).
								X65897	H.sapiens rearranged VDJ region (BEL28).
								X65898	H.sapiens rearranged VDJ region (BEL3).
								X65900	H.sapiens rearranged VDJ region (BEL34).
								X65901	H.sapiens rearranged VDJ region (BEL43).
								X65884	H.sapiens rearranged VDJ region (BEL45).
								X65902	H.sapiens rearranged VDJ region (BEL5).
								X65903	H.sapiens rearranged VDJ region (BEL54).
								X65905	H.sapiens rearranged VDJ region (BEL56).
								X65907	H.sapiens rearranged VDJ region (BEL69).
								X65908	H.sapiens mRNA for immunoglobulin V and J regions (clone BL36H3).
								X65909	H.sapiens mRNA for immunoglobulin V and J regions (clone BL41H3).
								X65910	H.sapiens mRNA for heavy chain variable region, BO1-VH.
								X65911	H.sapiens mRNA for BradH3 heavy chain variable Ig domain.
								X65885	Homo sapiens mRNA for anti-Sm antibody VH chain (VH1/DK1 or DM1/JH4b).
								X65917	Homo sapiens mRNA for anti-Sm antibody VH chain (VH3/Dxp4/JH6).
								X65918	H.sapiens mRNA for immunoglobulin V and J regions (clone CA46H5).
								X65920	H.sapiens mRNA for immunoglobulin heavy chain variable region (D4-D10, VH4, 4-61/DP-66).
								X65887	H.sapiens mRNA for immunoglobulin heavy chain variable region (D4-F21, VH4, 4-31/DP-65).
								X65888	H.sapiens mRNA for immunoglobulin heavy chain variable region (D4-I10, VH4, 4-31/DP-65).
								X65889	H.sapiens mRNA for immunoglobulin heavy chain variable region (D4-I4, VH4, 4-34/DP-63).
								X65891	H.sapiens mRNA for immunoglobulin heavy chain variable region (D4-L3, VH4, 4-34/DP-63).
								X56525	Homo sapiens mRNA for immunoglobulin heavy chain variable region, clone DIG4.
								X17115	H.sapiens mRNA for heavy chain variable region, DO1-VH.
								X91804	H.sapiens rearranged Ig heavy chain variable region (VDJ).
								X58529	H.sapiens rearranged Ig heavy chain variable region (VDJ).
								X14584	H.sapiens rearranged Ig heavy chain variable region (VDJ).
								Z18316	H.sapiens rearranged Ig heavy chain variable region (VDJ).
								Z18317	H.sapiens rearranged Ig heavy chain variable region (VDJ).
								Z18318	H.sapiens rearranged Ig heavy chain variable region (VDJ).
								Z18319	H.sapiens rearranged Ig heavy chain variable region (VDJ).
								Z18320	H.sapiens rearranged Ig heavy chain variable region (VDJ).
								Z18322	H.sapiens rearranged Ig heavy chain variable region (VDJ).
								Z18324	H.sapiens rearranged Ig heavy chain variable region (VDJ).
								X56524	H.sapiens rearranged Ig heavy chain variable region (VD).
								X60505	H.sapiens rearranged Ig heavy chain variable region (VDJ).
								X73605	H.sapiens rearranged Ig heavy chain variable region (VDJ).

								Z34893	H.sapiens rearranged Ig heavy chain variable region (VDJ).
								X56523	H.sapiens rearranged Ig heavy chain variable region (VDJ).
								X67943	H.sapiens rearranged Ig heavy chain variable region (VDJ).
								X67908	H.sapiens rearranged Ig heavy chain variable region (VDJ).
								X67906	H.sapiens rearranged Ig heavy chain variable region (VDJ).
								X67907	H.sapiens rearranged Ig heavy chain variable region (VDJ).
								X93001	H.sapiens rearranged Ig heavy chain variable region (VDJ).
								Z33400	H.sapiens rearranged Ig heavy chain variable region (VDJ).
								Y08406	H.sapiens rearranged Ig heavy chain variable region (VDJ).
								Y08428	H.sapiens rearranged Ig heavy chain variable region (VDJ).
								X67879	H.sapiens rearranged Ig heavy chain variable region (VDJ).
								X17675	H.sapiens rearranged Ig heavy chain variable region (VDJ).
								X17677	H.sapiens rearranged Ig heavy chain variable region (VDJ).
								X15611	H.sapiens rearranged Ig heavy chain variable region (VDJ).
								X17678	H.sapiens rearranged Ig heavy chain variable region (VDJ).
								Y08593	H.sapiens rearranged Ig heavy chain variable region (VDJ).
								X99361	H.sapiens rearranged Ig heavy chain variable region (VDJ).
								X99362	H.sapiens rearranged Ig heavy chain variable region (VDJ).
								X99364	H.sapiens rearranged Ig heavy chain variable region (VDJ).
								X99365	H.sapiens rearranged Ig heavy chain variable region (VDJ).
								X99366	H.sapiens rearranged Ig heavy chain variable region (VDJ).
								X99353	H.sapiens rearranged Ig heavy chain variable region (VDJ).
								X99354	H.sapiens rearranged Ig heavy chain variable region (VDJ).
								X99355	H.sapiens rearranged Ig heavy chain variable region (VDJ).
								X99357	H.sapiens rearranged Ig heavy chain variable region (VDJ).
								X99358	H.sapiens rearranged Ig heavy chain variable region (VDJ).
								Z70257	H.sapiens rearranged Ig heavy chain variable region (VDJ).
								Z36887	H.sapiens rearranged Ig heavy chain variable region (VDJ).
								Z36889	H.sapiens rearranged immunoglobulin heavy chain V region (VDJ).
								Z11951	H.sapiens mRNA for heavy chain Fab fragment, clone LBalpha-94 h.
								Z11959	Homo sapiens mRNA for immunoglobulin heavy chain, Fab fragment, isolate AI-X40.
								Z11950	H.sapiens mRNA for FomAH heavy chain variable Ig domain.
								Z11958	H.sapiens rearranged mRNA for polyreactive natural autoantibody (IgM VDJC region).
								Z11945	H.sapiens mRNA for Gad2H heavy chain variable Ig domain.
								Z11946	H.sapiens mRNA for immunoglobulin gamma heavy chain, isolate VL1.
								Z11956	H.sapiens mRNA for immunoglobulin gamma heavy chain, isolate VL5.
								Z33901	H.sapiens mRNA for immunoglobulin heavy chain V-D-J region (clone ST IgG VH5 15).
								Z33903	H.sapiens rearranged immunoglobulin heavy chain variable region (VDJ).
								AJ001171	H.sapiens rearranged immunoglobulin heavy chain variable region (VDJ).
								AJ001410	H.sapiens rearranged immunoglobulin heavy chain variable region (VDJ).
								L38560	H.sapiens mRNA for immunoglobulin heavy chain, partial, clone V16-10.
								L38566	Homo sapiens rearranged IGHV-1L1 gene encoding IgG heavy chain.
								BX538066	Homo sapiens rearranged Humigha3d1 gene encoding IgG heavy chain variable region.
								BX538077	H.sapiens mRNA for HamBH heavy chain variable Ig domain.
								BX538126	H.sapiens HB4C5 antibody heavy chain variable region mRNA.
								BX640623	H.sapiens mRNA for fetal IG heavy chain (FL13-29).
								BX640624	H.sapiens mRNA for fetal IG heavy chain (FL13-45).
								BX640625	H.sapiens mRNA for Ig heavy chain, variable region (ID:CLL039VH).
								BX640626	H.sapiens mRNA for Ig heavy chain, variable region (ID:CLL055VH).
								BX640710	H.sapiens mRNA for Ig heavy chain, variable region (ID:CLL057VH).
								BX640824	H.sapiens mRNA for immunoglobulin 0-81VH.
								BX640847	Human Immunoglobulin VH mRNA (106 bp).
								BX640854	Human Immunoglobulin VH mRNA (486 bp).
								BX647160	Human Immunoglobulin VH mRNA (487 bp).

								BX648082	Human Immunoglobulin VH mRNA (488 bp).
								BX648616	Human Immunoglobulin VH mRNA (498 bp).
								BX648874	H.sapiens mRNA for variable immunoglobulin anti-steroid, heavy chain, gene family VH3.
								X64159	H.sapiens mRNA for variable immunoglobulin anti-testosterone, heavy chain, gene family VH3.
								Z46847	Homo sapiens mRNA for IgE Heavy chain.
								Z46855	H.sapiens mRNA for immunoglobulin heavy chain (clone F1cr).
								X75021	Homo sapiens mRNA for immunoglobulin kappa heavy chain.
								X75024	Homo sapiens mRNA for immunoglobulin lambda heavy chain.
								Z46761	H.sapiens mRNA for immunoglobulin gamma1 heavy chain, G1HC-rFab-H85.
								Z46762	H.sapiens rearranged IgG VH-D-JH-Hinge-CH2-CH3 region.
								Z46763	H.sapiens rearranged IgG VHIII-D-JH-CH3 region.
								Z46764	H.sapiens mRNA for variable region of IgG (VH3 family).
								Z46767	H.sapiens mRNA for IG heavy chain V-region, VH3 gene.
								Z46769	H.sapiens mRNA for immunoglobulin heavy chain variable region.
								X94004	H.sapiens Ig rearranged heavy chain mRNA VDJ region.
								X94048	H.sapiens Ig rearranged heavy chain mRNA VDJ region.
								X94059	H.sapiens Ig rearranged heavy chain mRNA VDJ region.
								X94038	H.sapiens Ig rearranged heavy chain mRNA VDJ region.
								Z46285	H.sapiens Ig H region switch/C gamma-1 sterile transcript.
								Z33904	Homo sapiens rearranged Ig H-chain V-domain cDNA.
								X64155	Homo sapiens rearranged Ig H-chain V-domain cDNA.
								X64156	Homo sapiens rearranged Ig H-chain V-domain cDNA.
								X52111	Homo sapiens rearranged Ig H-chain V-domain cDNA.
								X75388	Homo sapiens rearranged Ig H-chain V-domain cDNA.
								X72745	Homo sapiens rearranged Ig H-chain V-domain cDNA.
								X81738	H.sapiens mRNA for XLA IG heavy chain VDJ region (LE 1-10).
								X81739	H.sapiens mRNA for XLA IG heavy chain VDJ region (LE 1-9).
								X81740	H.sapiens mRNA for XLA IG heavy chain VDJ region (LE 3-12).
								X81741	H.sapiens mRNA for XLA IG heavy chain VDJ region (LE 3-13).
								X81742	H.sapiens mRNA for XLA IG heavy chain VDJ region (LE 3-15).
								X81743	H.sapiens mRNA for XLA IG heavy chain VDJ region (LE 3-16).
								X81744	H.sapiens mRNA for XLA IG heavy chain VDJ region (LE 3-20).
								X81745	H.sapiens mRNA for XLA IG heavy chain VDJ region (LE 3-21).
								X81746	H.sapiens mRNA for XLA IG heavy chain VDJ region (LE 1-100).
								X81747	H.sapiens mRNA for XLA IG heavy chain VDJ region (LE 4-104).
								X81748	H.sapiens mRNA for XLA IG heavy chain VDJ region (LE 4-105).
								X81751	H.sapiens mRNA for XLA IG heavy chain VDJ region (LE 4-30).
								X81752	H.sapiens mRNA for XLA IG heavy chain VDJ region (LE 4-5).
								X81753	H.sapiens mRNA for XLA IG heavy chain VDJ region (LE 4-56).
								X81755	H.sapiens mRNA for XLA IG heavy chain VDJ region (LE 4-63).
								Z49143	H.sapiens mRNA for XLA IG heavy chain VDJ region (LE 4-8).
								Z46304	H.sapiens mRNA for XLA IG heavy chain VDJ region (LE 4-89).
								Z46306	H.sapiens mRNA for XLA IG heavy chain VDJ region (LE 1-17).
								Z46308	H.sapiens mRNA for XLA IG heavy chain VDJ region (LE 6-14).
								Z46315	H.sapiens mRNA for XLA IG heavy chain VDJ region (LE 6-16).
								Z46316	H.sapiens mRNA for XLA IG heavy chain VDJ region (RS 6-21).
								Z46319	H.sapiens mRNA for XLA IG heavy chain VDJ region (LE 1-29).
								Z46322	H.sapiens mRNA for XLA IG heavy chain VDJ region (LE 1-42).
								Z46327	H.sapiens mRNA for XLA IG heavy chain VDJ region (LE 1-54).
								Z46332	H.sapiens mRNA for XLA IG heavy chain VDJ region (LE 1-76).
								X56592	Human mRNA for Ig heavy chain variable region (hybridoma LSF2).
								Z75362	Human mRNA for IgM heavy chain complete sequence.
								Z75363	H.sapiens mRNA for Ig heavy chain variable and joining regions.
								Z75404	Human rearranged immunoglobulin mRNA for mu heavy chain enhancer and constant region.

								Z75364	Human mRNA for Ig mu-chain.
								Z75365	H.sapiens immunoglobulin V-region mu chain.
								Z75366	H.sapiens mRNA for immunoglobulin V-region of mu chain.
								Z75405	H.sapiens mRNA for immunoglobulin V-region of mu chain.
								Z75370	H.sapiens mRNA for immunoglobulin V-region of mu chain.
								Z75406	H.sapiens mRNA for immunoglobulin V-region of mu chain.
								Z75371	H.sapiens mRNA for immunoglobulin V-region of mu chain.
								Z75373	H.sapiens mRNA for immunoglobulin V-region of mu chain.
								Z75374	Human mRNA for Ig heavy chain variable region (hybridoma Ray 4).
								Z75375	Human immunoglobulin heavy chain variable VH1 family gene (second relapse N4).
								Z75377	H.sapiens (RFKL5H) mRNA for immunoglobulin gamma heavy chain variable region, rheumatoid factor (301bp).
								Z75379	H.sapiens (RFTS7H) mRNA for immunoglobulin gamma chain variable region, rheumatoid factor (313bp).
								Z75386	Human mRNA for Ig heavy chain variable region (hybridoma SB5/D6).
								Z75387	H.sapiens rearranged immunoglobulin heavy chain variable region (VDJ).
								Z75389	H.sapiens rearranged immunoglobulin heavy chain variable region (VDJ).
								Z75392	H.sapiens rearranged immunoglobulin heavy chain variable region (VDJ).
								Z75394	H.sapiens rearranged immunoglobulin heavy chain variable region (VDJ).
								Z75395	H.sapiens mRNA for IgG heavy chain V-D-J-C region (clone Tgl9).
								Z75396	H.sapiens (KR) mRNA for immunoglobulin variable region V-D-J.
								Z75397	H.sapiens mRNA for immunoglobulin, VH3 family, patient Del.G.
								Z75399	H.sapiens mRNA for immunoglobulin, VH4 family, patient Vanh.G.
								Z75402	H.sapiens VH6 mRNA for IG heavy chain V region (N1).
								U00506	H.sapiens Ig V-H region (clone gamma-F1).
								U00507	H.sapiens Ig V-H region (clone gamma-F9).
								U00508	Human mRNA for LS2/EVB Ig V(H), autoantibody with RBC specifity.
								U00509	H.sapiens Ig V-H region (clone mu-F7).
								U00510	H.sapiens mRNA for Ig heavy chain anti-TTd, variable region.
								U00515	H.sapiens mRNA for rearranged Ig heavy chain variable domain, cell-type RA-2-L21.
								U00521	H.sapiens mRNA for rearranged Ig heavy chain variable domain, cell-type RA-1-DT7.
								U03400	H.sapiens mRNA for rearranged Ig heavy chain variable domain, cell-type RA-4-Pd2.
								U04370	H.sapiens mRNA for rearranged Ig heavy chain variable domain, cell-type RA-4-Pd18.
								U04371	H.sapiens mRNA for rearranged Ig heavy chain variable domain, cell-type RA-2-L5.
								U05891	H.sapiens mRNA for rearranged Ig heavy chain variable domain, 330bp.
								U05893	H.sapiens mRNA for rearranged Ig heavy chain variable domain, cell-type TB-1-M2.
								U06787	H.sapiens mRNA for rearranged Ig heavy chain variable domain, cell-type TB-1-P7.
								U07985	H.sapiens mRNA for rearranged Ig heavy chain variable domain, cell-type TB-2-H68.
								U07986	H.sapiens mRNA for rearranged Ig heavy chain variable domain, cell-type TB-3-D13.
								U07987	H.sapiens mRNA for immunoglobulin heavy chain V-D-J region (patient 7).
								U07988	H.sapiens (JLH) mRNA for immunoglobulin heavy chain V-D-J region.
								U09058	H.sapiens (PBAH) mRNA for immunoglobulin heavy chain V-D-J region.
								U09062	H.sapiens rearranged VH3 gene encoding immunoglobulin variable region.
								U09063	H.sapiens rearranged VH gene encoding immunoglobulin variable region.
								U09064	H.sapiens rearranged VH3 gene encoding immunoglobulin variable region.
								U09065	H.sapiens rearranged VH4 gene encoding immunoglobulin variable region.
								U09066	H.sapiens rearranged VH3 gene encoding immunoglobulin variable region.
								U13195	H.sapiens rearranged immunoglobulin IgA1 variable heavy chain gene encoding immunoglobulin variable region.
								U13196	H.sapiens rearranged VH3 gene encoding immunoglobulin variable region.
								U13197	H.sapiens rearranged immunoglobulin heavy chain variable region (VDJ).
								U13200	H.sapiens rearranged immunoglobulin heavy chain variable region (VDJ).
								U13201	Homo sapiens rearranged immunoglobulin VH gene.
								U13202	Homo sapiens mRNA for immunoglobulin heavy chain variable region to JH segment, EJ-3F.
								U19885	Human clone rev9Fd immunoglobulin heavy chain, V3-J-C1 region, mRNA, partial cds.
								U21250	Human clone tat16/31Fab, immunoglobulin heavy chain, V3-J-C1 region, partial cds.
								U21251	Homo sapiens mRNA; cDNA DKFZp686K04218 (from clone DKFZp686K04218).

								U21253	Homo sapiens mRNA; cDNA DKFZp686C02218 (from clone DKFZp686C02218); complete cds.
								U21262	Homo sapiens mRNA; cDNA DKFZp686H11213 (from clone DKFZp686H11213).
								U21264	Homo sapiens mRNA; cDNA DKFZp686I04196 (from clone DKFZp686I04196); complete cds.
								U22391	Homo sapiens mRNA; cDNA DKFZp686K18196 (from clone DKFZp686K18196).
								U24080	Homo sapiens mRNA; cDNA DKFZp686C02220 (from clone DKFZp686C02220).
								U24081	Homo sapiens mRNA; cDNA DKFZp686G21220 (from clone DKFZp686G21220).
								U27189	Homo sapiens mRNA; cDNA DKFZp686O16217 (from clone DKFZp686O16217).
								U32154	Homo sapiens mRNA; cDNA DKFZp686M24218 (from clone DKFZp686M24218).
								U32200	Homo sapiens mRNA; cDNA DKFZp686J11235 (from clone DKFZp686J11235).
								U32268	Homo sapiens mRNA; cDNA DKFZp686L19235 (from clone DKFZp686L19235).
								U32488	Homo sapiens mRNA; cDNA DKFZp686M17196 (from clone DKFZp686M17196).
								U32490	Homo sapiens mRNA; cDNA DKFZp686L15237 (from clone DKFZp686L15237).
								U32491	Homo sapiens mRNA; cDNA DKFZp686A15235 (from clone DKFZp686A15235).
								U32492	Homo sapiens mRNA; cDNA DKFZp686N22238 (from clone DKFZp686N22238).
								U32598	H.sapiens mRNA for Mad2H heavy chain variable Ig domain.
								U32610	H.sapiens mRNA for immunoglobulin heavy chain (MICA 1).
								U32949	H.sapiens mRNA for immunoglobulin heavy chain (MICA 5).
								U32950	H.sapiens (F1.1) mRNA for Immunoglobulin M.
								U32956	H.sapiens (J2.2) mRNA for Immunoglobulin M.
								U32960	H.sapiens mRNA for immunoglobulin heavy chain VDJ region (MT1.4).
								U32961	H.sapiens mRNA for immunoglobulin heavy chain VDJ region (MT1.5).
								U38588	H.sapiens mRNA for immunoglobulin heavy chain VDJ region (MT1.6).
								U41571	H.sapiens mRNA for immunoglobulin heavy chain VDJ region (MT1.7).
								U42468	H.sapiens mRNA for immunoglobulin heavy chain VDJ region (MT2.3).
								U43754	H.sapiens mRNA for immunoglobulin heavy chain VDJ region (MT2.5).
								U43756	H.sapiens mRNA for immunoglobulin heavy chain V-D-J region (clone ST IgM VH5 1).
								U43757	H.sapiens mRNA for immunoglobulin heavy chain V-D-J region (clone ST IgM VH5 10).
								U43758	H.sapiens mRNA for immunoglobulin heavy chain V-D-J region (clone ST IgM VH5 15).
								U43759	H.sapiens mRNA for immunoglobulin heavy chain V-D-J region (clone ST IgM VH5 3).
								U43760	H.sapiens rearranged immunoglobulin heavy chain V region (VDJ).
								U43763	H.sapiens rearranged immunoglobulin heavy chain variable region (VDJ).
								U43927	H.sapiens mRNA for Oak3H heavy chain variable Ig domain.
								U44483	H.sapiens mRNA for Og31H heavy chain variable Ig domain.
								U44489	Human mRNA for immunoglobulin heavy chain VDJ region, Vh-IV family (cell line Pag-1).
								U44491	H.sapiens rearranged mRNA for immunoglobulin heavy chain.
								U44494	H.sapiens mRNA for rearranged IG heavy chain V-region (VDJ).
								U44496	H.sapiens rearranged VDJ region (ROU111).
								U44502	H.sapiens rearranged VDJ region (ROU112).
								U44506	H.sapiens rearranged VDJ region (ROU119).
								U50344	H.sapiens rearranged VDJ region (ROU12).
								U51549	H.sapiens rearranged VDJ region (ROU122).
								U63211	H.sapiens rearranged VDJ region (ROU125).
								U64467	H.sapiens rearranged VDJ region (ROU126).
								U64470	H.sapiens rearranged VDJ region (ROU127).
								U64478	H.sapiens rearranged VDJ region (ROU129).
								U64480	H.sapiens rearranged VDJ region (ROU13).
								U64486	H.sapiens rearranged VDJ region (ROU131).
								U64487	H.sapiens rearranged VDJ region (ROU221).
								U64497	H.sapiens rearranged VDJ region (ROU222).
								U64499	H.sapiens rearranged VDJ region (ROU228).
								U66189	H.sapiens rearranged VDJ region (ROU233).
								U70238	H.sapiens mRNA for immunoglobulin heavy chain VDJ region (RT79/2).
								U71103	H.sapiens immunoglobulin heavy chain VDJ region (S17B).
								U71105	H.sapiens immunoglobulin heavy chain VDJ region (S20A).

								U71106	H.sapiens immunoglobulin heavy chain VDJ region (S8A).
								U76679	H.sapiens immunoglobulin heavy chain VDJ region (SAg28 IgG).
								U76681	H.sapiens immunoglobulin heavy chain VDJ region (SAg5 IgG).
								U76685	H.sapiens immunoglobulin heavy chain VDJ region (SEBV A18).
								U77371	H.sapiens immunoglobulin heavy chain VDJ region (SEBV B13).
								U77372	H.sapiens immunoglobulin heavy chain VDJ region (SEBV B53).
								U77373	H.sapiens immunoglobulin heavy chain VDJ region (SEBV C8).
								U77374	Human mRNA for immunoglobulin heavy chain (T14:Vh4-T14).
								U77375	H.sapiens mRNA for Ig heavy chain variable region (VH4DJ) (clone T14.1).
								U77539	H.sapiens mRNA for Ig heavy chain variable region (VH4DJ) (clone T14.10).
								U77544	H.sapiens mRNA for Ig heavy chain variable region (VH4DJ) (clone T14.11).
								U77549	H.sapiens mRNA for Ig heavy chain variable region (VH4DJ) (clone T14.12).
								U77551	H.sapiens mRNA for Ig heavy chain variable region (VH4DJ) (clone T14.13).
								U77552	H.sapiens mRNA for Ig heavy chain variable region (VH4DJ) (clone T14.14).
								U79585	H.sapiens mRNA for Ig heavy chain variable region (nonfunctional; VH4DJ) (clone T14.16).
								U82257	H.sapiens mRNA for Ig heavy chain variable region (VH4DJ) (clone T14.19).
								U85240	H.sapiens mRNA for partial immunoglobulin variable region (VH4DJ) (clone T14.2).
								U85243	H.sapiens mRNA for Ig heavy chain variable region (VH4DJ) (clone T14.20).
								U86524	H.sapiens mRNA for Ig heavy chain variable region (nonfunctional; VH4DJ) (clone T14.22).
								U86525	H.sapiens mRNA for Ig heavy chain variable region (VH4DJ) (clone T14.23).
								U86668	H.sapiens mRNA for Ig heavy chain variable region (VH4DJ) (clone T14.24).
								U86670	H.sapiens mRNA for Ig heavy chain variable region (VH4DJ) (clone T14.3).
								U86785	H.sapiens mRNA for Ig heavy chain variable region (VH4DJ) (clone T14.5).
								U92452	H.sapiens mRNA for Ig heavy chain variable region (VH4DJ) (clone T22.11).
								U93176	H.sapiens mRNA for Ig heavy chain variable region (VH4DJ) (clone T22.12).
								U95238	H.sapiens mRNA for Ig heavy chain variable region (VH4DJ) (clone T22.14).
								U95240	H.sapiens mRNA for Ig heavy chain variable region (VH4DJ) (clone T22.18).
								U95241	H.sapiens mRNA for Ig heavy chain variable region (VH4DJ) (clone T22.20).
								U96392	H.sapiens mRNA for Ig heavy chain variable region (VH4DJ) (clone T22.21).
								U97242	H.sapiens mRNA for Ig heavy chain variable region (VH4DJ) (clone T22.22).
								U97245	H.sapiens mRNA for Ig heavy chain variable region (VH4DJ) (clone T22.23).
								U97246	H.sapiens mRNA for Ig heavy chain variable region (VH4DJ) (clone T22.26).
								X94019	H.sapiens mRNA for Ig heavy chain variable region (VH4DJ) (clone T22.30).
								Z30671	Human immunoglobulin heavy chain variable region (clone 1f6-3) mRNA, partial cds.
								Z30672	Human immunoglobulin heavy chain variable region (clone 1f7-3) mRNA, partial cds.
								Z30594	Human immunoglobulin heavy chain variable region (clone 1g11-3) mRNA, partial cds.
								Z30675	Human immunoglobulin heavy chain variable region (clone 1g4-3) mRNA, partial cds.
								Z30676	Human immunoglobulin heavy chain variable region (clone 1g5-3) mRNA, partial cds.
								Z30548	Human immunoglobulin heavy chain variable region (clone Agamma14-3) mRNA, partial cds.
								Z30549	Human immunoglobulin heavy chain variable region (clone Agamma21-3) mRNA, partial cds.
								Z30551	Human Ig heavy chain V-D-J region mRNA, partial cds.
								Z30552	Human clone OF7H1.19 Ig heavy chain V-region mRNA, partial cds.
								Z30557	Human clone OF7H1.2 Ig heavy chain V-region mRNA, partial cds.
								Z30562	Human clone 3542 Ig heavy chain rearranged sterile mRNA, partial sequence.
								Z30563	Human clone 4429 Ig heavy chain germline sterile mRNA, partial sequence.
								Y11325	Human monoclonal antibody CA4 Ig heavy chain variable region mRNA, partial cds.
								Y11326	Human myeloma immunoglobulin heavy chain Fd region V-D-J-CH1 mRNA, partial cds.
								X61647	Human immunoglobulin IgH heavy chain Fd fragment mRNA, partial cds.
								X62955	Human Burkitt's lymphoma immunoglobulin IgM heavy chain Fd fragment mRNA, partial cds.
								X62956	Human lymphoma immunoglobulin IgM heavy chain Fd fragment mRNA, partial cds.
								X62957	Human immunoglobulin heavy chain, V-region (SPH1.10) mRNA, partial cds.
								X62958	Human immunoglobulin heavy chain, V-region (SPH1.2) mRNA, partial cds.
								X62959	Human immunoglobulin heavy chain, V-region (SPH1.20) mRNA, partial cds.
								X62964	Human immunoglobulin heavy chain, V-region (SPH1.3) mRNA, partial cds.

								X62961	Human immunoglobulin heavy chain, V-region (SPH1.4) mRNA, partial cds.
								X62962	Human immunoglobulin heavy chain, V-region (SPH1.5) mRNA, partial cds.
								X62965	Human clone 2-17M IgM heavy chain V region (VH6DJ) and C region (C Mu) mRNA, partial cds.
								X62966	Human clone 1-5M IgM heavy chain V region (VH6DJ) and C region (C Mu) mRNA, partial cds.
								X62967	Human clone 2-15M IgM heavy chain V region (VH6DJ) and C region (C Mu) mRNA, partial cds.
								X62968	Human clone 2-3G IgG heavy chain V region (VH6DJ) and C region (C Gamma) mRNA, partial cds.
								X62969	Human clone 2-26M IgM heavy chain V region (VH6DJ) and C region (C Mu) mRNA, partial cds.
								X62970	Human clone 2-18M IgM heavy chain V region (VH6DJ) and C region (C Mu) mRNA, partial cds.
								Y11328	Human rearranged immunoglobulin heavy chain mRNA, partial cds.
								X62971	Human rearranged Ig gamma heavy chain V region (VH6-JH1) mRNA, partial cds, clone JGpFv3-01 VH.
								X61646	Human rearranged Ig gamma heavy chain V region (VH6-JH4) mRNA, partial cds, clone JGpFv3-02 VH.
								X56158	Human rearranged Ig gamma heavy chain V region (VH3-JH1) mRNA, partial cds, clone JGpFv3-07 VH.
								Y11324	Human rearranged Ig mu heavy chain V region (VH3) mRNA, partial cds, clone JMpFv2-51 VH.
								X67888	Human rearranged Ig mu heavy chain V region (VH6) mRNA, partial cds, clone JMpFv2-56 VH.
								X67889	Human B cell chronic lymphocytic leukemia immunoglobulin heavy chain mRNA, VH4, JH6, Cgamma3 regions, partial cds.
								X67880	Human immunoglobulin heavy chain VH3 (H11) mRNA, partial cds.
								X67881	Human immunoglobulin heavy chain VH3 (HV3019B9) mRNA, partial cds.
								X67882	Human anti-cryptococcal antibody 3B6 Ig heavy chain mRNA, V region, partial cds.
								X67883	Human clone PBUF-BC2 non-productive immunoglobulin heavy chain third complementarity-determining region rearrangement,
								X67884	partial sequence.
								X67886	Human pre-B cell Ig heavy chain mRNA, third complementarity-determining region, clone PBT-137, partial cds.
								X67887	Human pre-B cell Ig heavy chain mRNA, third complementarity-determining region, clone PBT-124, partial cds.
								X67900	Human pre-B cell Ig heavy chain mRNA, third complementarity-determining region, clone PBT-116, partial cds.
								X67890	Human pre-B cell Ig heavy chain mRNA, third complementarity-determining region, clone PBT-55, partial cds.
								X67891	Human pre-B cell Ig heavy chain mRNA, third complementarity-determining region, clone PBT-127, partial cds.
								X67893	Human pre-B cell Ig heavy chain mRNA, third complementarity-determining region, clone PBT-121, partial cds.
								X67895	Human pre-B cell Ig heavy chain mRNA, third complementarity-determining region, clone PBT-C3, partial cds.
								X67896	Human pre-B cell Ig heavy chain mRNA, third complementarity-determining region, clone PBT-178, partial cds.
								X67897	Human immature B cell Ig heavy chain mRNA, third complementarity-determining region, clone MBT-36, partial cds.
								X67898	Human immature B cell Ig heavy chain mRNA, third complementarity-determining region, clone MBT-171, partial cds.
								Z47229	Human immature B cell Ig heavy chain mRNA, third complementarity-determining region, clone MBT-17, partial cds.
								Z47349	Human immature B cell Ig heavy chain mRNA, third complementarity-determining region, clone MBT-159, partial cds.
								Z47231	Human immature B cell Ig heavy chain mRNA, third complementarity-determining region, clone MBT-196, partial cds.
								Z47214	Human anti-ds DNA IgG heavy chain variable region mRNA, partial cds.
								Z47213	Human clone STMAG H.10 immunoglobulin heavy chain variable region (VH26) mRNA, partial cds.
								Z47234	Human immunoglobulin rearranged heavy chain variable region mRNA, partial cds.
								Z47215	Human immunoglobulin heavy chain variable region mRNA, cell line 46e7, anti-RhD, partial cds.
								Z47236	Human immunoglobulin heavy chain variable region mRNA, cell line 28e4, anti-RhD, partial cds.
								Z14213	Human immunoglobulin heavy chain variable region mRNA, cell line 26c1, anti-RhD, partial cds.
								Z14215	Human immunoglobulin heavy chain variable region mRNA, cell line 25f5, anti-RhD, partial cds.
								Z14216	Human immunoglobulin heavy chain variable region mRNA, cell line 24a5, anti-RhD, partial cds.
								Z14217	Human immunoglobulin heavy chain variable region mRNA, cell line 21h9, anti-RhD, partial cds.
								Z14218	Human immunoglobulin heavy chain variable region mRNA, cell line 13d2, anti-RhD, partial cds.
								Z14219	Human anti-dsDNA IgM heavy chain variable region mRNA, partial cds.
								Z14220	Human immunoglobulin E heavy chain VH251 (VH5) mRNA, partial cds.
								Z14221	Human immunoglobulin E heavy chain VH251 (VH5) mRNA, partial cds.
								Z14223	Human immunoglobulin E heavy chain VH251 (VH5) mRNA, partial cds.
								Z47237	Human immunoglobulin E heavy chain VH32 (VH5) mRNA, partial cds.
								Z47218	Human immunoglobulin E heavy chain VH32 (VH5) mRNA, partial cds.
								Z47217	Human immunoglobulin E heavy chain VH251 (VH5) mRNA, partial cds.
								Z47222	Human immunoglobulin E heavy chain VH32 (VH5) mRNA, partial cds.
								Z47242	Human NANUC-1 heavy chain VDJ and part of IgG1 constant region mRNA, partial cds.
								Z47226	Human immunoglobulin heavy chain variable region (VH-D-JH) mRNA, partial cds.
								Z47243	Human Ig heavy chain variable region (humha311) mRNA, partial cds.

								Z47223	Human rheumatoid arthritis synovium immunoglobulin heavy chain variable region mRNA, partial cds.
								Z47241	Human rheumatoid arthritis synovium immunoglobulin heavy chain variable region mRNA, partial cds.
								Z47220	Human rheumatoid arthritis synovium immunoglobulin heavy chain variable region mRNA, partial cds.
								Z47238	Human rheumatoid arthritis synovium immunoglobulin heavy chain variable region mRNA, partial cds.
								Z47219	Human rheumatoid arthritis synovium immunoglobulin heavy chain variable region mRNA, partial cds.
								Z47227	Human rheumatoid arthritis synovium immunoglobulin heavy chain variable region mRNA, partial cds.
								Z47235	Human rheumatoid arthritis synovium immunoglobulin heavy chain variable region mRNA, partial cds.
								Z47232	Human rheumatoid arthritis synovium immunoglobulin heavy chain variable region mRNA, partial cds.
								X54437	Human monoclonal antibody heavy chain variable region mRNA, partial cds.
								X54441	Human clone AD4-37VH anti-DNA immunoglobulin heavy chain mRNA, partial cds.
								X54445	Human immunoglobulin IgM heavy chain VH4 region mRNA, partial cds.
								X54447	Human immunoglobulin IgM heavy chain VH1 region mRNA, partial cds.
								X64241	Human immunoglobulin IgM heavy chain VH4 region mRNA, partial cds.
								X64238	Homo sapiens clone CDC-1hc IgM heavy chain variable region mRNA, partial cds.
								X64240	Homo sapiens clone IRM-10hc IgM heavy chain variable region mRNA, partial cds.
								X64234	Homo sapiens clone IRM-8hc IgM heavy chain variable region mRNA, partial cds.
								X64235	Homo sapiens clone BY-4HC IgM heavy chain variable region mRNA, partial cds.
								X64239	Homo sapiens clone BY-7HC IgM heavy chain variable region mRNA, partial cds.
								X64147	Homo sapiens clone BY-12HC IgM heavy chain variable region mRNA, partial cds.
								Z30573	Homo sapiens clone IRM-3HC IgM heavy chain variable region mRNA, partial cds.
								Z30574	Homo sapiens clone IRM-7HC IgM heavy chain variable region mRNA, partial cds.
								Z30580	Human clone 2A2 immunoglobulin variable region (VH5-D-JH4) gene, partial cds.
								Z30581	Human clone 3C1 immunoglobulin variable region (VH5-D-JH4) gene, partial cds.
								X79169	Human clone 54A13 immunoglobulin variable region (VH5-D-JH4) gene, partial cds.
								X79171	Human clone 54A2 immunoglobulin variable region (VH5-D-JH5) gene, partial cds.
								X79172	Human clone 54B3 immunoglobulin variable region (VH5-D-JH6) gene, partial cds.
								X79165	Human clone SC immunoglobulin V-region heavy chain mRNA, partial cds.
								X79166	Homo sapiens anti-ssDNA antibody heavy chain variable region (VH26) mRNA, partial cds.
								X62126	Human IgM rheumatoid factor RF-TT1, variable heavy chain, mRNA, partial cds.
								Y12720	Human IgM rheumatoid factor RF-TT9, variable heavy chain, mRNA, partial cds.
								Y14935	Human immunoglobulin heavy chain variable region precursor (V4-39) mRNA, partial cds.
								Y14936	Human immunoglobulin heavy chain variable region precursor (V4-59) mRNA, partial cds.
								Y14944	Human immunoglobulin heavy chain V region (Humha403) mRNA, partial cds.
								Y18120	Human immunoglobulin heavy chain V region (Humha448) mRNA, partial cds.
								Z74668	Homo sapiens cell-type B cells Ig heavy chain variable domain (CLL-H1B) mRNA, partial cds.
								Z74671	Human anti-HBsAg immunoglobulin Fd chain mRNA, partial cds.
								Z74689	Human anti-GM2 ganglioside IgM monoclonal heavy chain variable region mRNA, partial cds.
								Z80836	Human clone B12-2 immunoglobulin rearranged heavy chain mRNA, partial cds.
								Z80839	Human clone MC90 immunoglobulin rearranged heavy chain mRNA, partial cds.
								Z80840	Human clone O50 immunoglobulin rearranged heavy chain mRNA, partial cds.
								Z80842	Homo sapiens anti-streptococcal/anti-myosin immunoglobulin heavy chain variable region mRNA, partial cds.
								Z80843	Homo sapiens IgM heavy chain variable region (humha316) mRNA, partial cds.
								Z80845	Homo sapiens IgM heavy chain variable region (humha327) mRNA, partial cds.
								Z80846	Homo sapiens IgM heavy chain variable region (humha4n3) mRNA, partial cds.
								Z80847	H.sapiens mRNA for immunoglobulin heavy chain V-D-J region (clone ST IgE VH5 2.A5,12).
								Z80848	H.sapiens mRNA for rearranged Ig heavy chain variable region (VH4) (VDJ) HSVBG7.
								Z80849	H.sapiens mRNA for rearranged Ig heavy chain variable region (VH4) (VDJ) HSVCBG3.
								Z80852	H.sapiens mRNA for rearranged Ig heavy chain variable region (VH4) (VDJ) HVC3M6.
								Z92888	H.sapiens mRNA for rearranged Ig heavy chain variable region (VH4) (VDJ) HSVCD55.
								Z92889	H.sapiens mRNA for rearranged Ig heavy chain variable region (VH4) (VDJ) HSVCD56.
								Z92890	H.sapiens mRNA for rearranged Ig heavy chain variable region (VH4) (VDJ) HSVGCG1.
								Z92893	H.sapiens mRNA for rearranged Ig heavy chain variable region (VH4) (VDJ) HSVGCG2.
								Z92895	H.sapiens mRNA for rearranged Ig heavy chain variable region (VH4) (VDJ) HSVGCG4.
								Z92896	H.sapiens mRNA for rearranged Ig heavy chain variable region (VH4) (VDJ) HSVGCG5.

								Z92898	H.sapiens mRNA for rearranged Ig heavy chain variable region (VH4) (VDJ) HSVGCM2.
								Z96955	H.sapiens mRNA for rearranged Ig heavy chain variable region (VH4) (VDJ) HSVGCM7.
								Z96960	H.sapiens mRNA for rearranged Ig heavy chain variable region (VH4) (VDJ) HSVGCM8.
								Z96961	H.sapiens mRNA for immunoglobulin heavy chain, partial, clone VH1-1.
								Z96962	H.sapiens mRNA for immunoglobulin heavy chain, partial, clone VH1-2.
								Z96964	Human Ig rearranged heavy chain mRNA V-region (VH1DJH6)VHantiPhOX15 (anti-2-phenyloxazal-5-one).
								Z97032	H.sapiens rearranged mRNA for fetal IG heavy chain Vh3, an inverted DIR2, a possible DXP1' and Jh4 (clone FL13-10).
								Z97036	H.sapiens rearranged mRNA for fetal IG heavy chain Vh3, DM1 or Dq52 and Jh4 (clone FL13-15).
								Z98694	H.sapiens rearranged mRNA for fetal IG heavy chain Vh3, a possible DQ52, an inverted DLR5, DXP'1 and Jh3 (clone FL13-16).
								Z98711	H.sapiens rearranged mRNA for fetal IG heavy chain Vh3, an possible inverted DIR2 and Jh4 (clone FL13-18).
								Z98713	H.sapiens rearranged mRNA for fetal IG heavy chain Vh3, DQ52, a possible inverted Dm2 (overlapping with DQ52), and Jh3
								Z98714	(clone FL13-2).
								Z98716	H.sapiens rearranged mRNA for fetal IG heavy chain Vh3, DN4 and Jh4 (clone FL13-21).
								Z98717	H.sapiens rearranged mRNA for fetal IG heavy chain Vh3, Dlr2 and Jh4 (clone FL13-23).
								Z98719	H.sapiens rearranged mRNA for fetal IG heavy chain Vh3, DQ52, DK5 and Jh3 (clone FL13-25).
								Z98720	H.sapiens rearranged mRNA for fetal IG heavy chain Vh3, an unidentified D (or N) region and Jh4 (clone FL13-28).
								Z98721	H.sapiens rearranged mRNA for fetal IG heavy chain Vh3, an unknown D element or possible inverted dlr5 and Jh3 (clone
								Z98722	FL13-42).
								Z98723	H.sapiens rearranged mRNA for fetal IG heavy chain Vh3, DQ52 and Jh4 (clone FL13-48).
								Z98724	H.sapiens rearranged mRNA for fetal IG heavy chain Vh3, DXP'1 and Jh4 (clone FL13-57).
								Z98730	H.sapiens rearranged mRNA for fetal IG heavy chain Vh3, an inverted DN4, DM1, a possible inverted DXP4 overlapping both
								Z98731	other D elements and Jh3 (clone FL13-6).
								Z98732	H.sapiens rearranged mRNA for fetal IG heavy chain Vh3, DK1 and Jh6 (clone FL18-2).
								Z98733	H.sapiens mRNA for immunoglobulin heavy chain, partial, clone VH3-7.
								Z98737	H.sapiens rearranged mRNA for fetal IG heavy chain Vh3, DN4 and Jh4 (clone FL7-4).
								Z98738	Human Ig rearranged heavy chain mRNA V-region (VH3DJH4) VHantiBSA3 (anti-bovine serum albumin).
								Z98739	Human mRNA for a VH4 immunoglobulin.
								Z98740	H.sapiens mRNA for immunoglobulin heavy chain, partial, clone V37-7.
								M88500	H.sapiens VH6 mRNA for IG heavy chain V region (N11).
								M84507	H.sapiens VH6 mRNA for IG heavy chain V region (N12).
								L39956	H.sapiens VH6 mRNA for IG heavy chain V region (N2).
								L39958	H.sapiens VH6 mRNA for IG heavy chain V region (N4).
								M82813	H.sapiens VH6 mRNA for IG heavy chain V region (N5).
								L38425	H.sapiens VH6 mRNA for IG heavy chain V region (N6).
								L38429	H.sapiens VH6 mRNA for IG heavy chain V region (N7).
								L38431	H.sapiens VH6 mRNA for IG heavy chain V region (N9).
								L38433	H.sapiens VH6 mRNA for IG heavy chain V region (N10).
								L38435	H.sapiens VH6 mRNA for IG heavy chain V region (A11).
								L28049	H.sapiens VH6 mRNA for IG heavy chain V region (A1).
								L28051	H.sapiens VH6 mRNA for IG heavy chain V region (A2).
								L28052	H.sapiens VH6 mRNA for IG heavy chain V region (A4).
								L28053	H.sapiens VH6 mRNA for IG heavy chain V region (A6).
								L01276	H.sapiens VH6 mRNA for IG heavy chain V region (A7).
								L04768	H.sapiens VH6 mRNA for IG heavy chain V region (A8).
								L04770	H.sapiens VH6 mRNA for IG heavy chain V region (A9).
								L13487	H.sapiens mRNA for immunoglobulin heavy chain V-region (clone CDN3FA2).
								L26904	H.sapiens mRNA for immunoglobulin heavy chain V-region (clone CDN3FA7).
								L08082	H.sapiens mRNA for immunoglobulin heavy chain V-region (clone CDN3FB5).
								L08087	H.sapiens mRNA for immunoglobulin heavy chain V-region (clone CDN3FC2).
								L08088	H.sapiens mRNA for immunoglobulin heavy chain V-region (clone CDN3FD7).
								L03681	H.sapiens mRNA for immunoglobulin heavy chain V-region (clone CDN3FE5).
								L03683	H.sapiens mRNA for immunoglobulin heavy chain V-region (clone CDN3GD9).
								L06912	H.sapiens mRNA for immunoglobulin heavy chain V-region (clone CDN3GH7).
								L06913	H.sapiens germline transcript of Ig heavy chain variable region (V).

								L06916	H.sapiens germline transcript of Ig heavy chain variable region (V).
								L06922	H.sapiens germline transcript of Ig heavy chain variable region (V).
								M97919	H.sapiens germline transcript of Ig heavy chain variable region (V).
								M97920	H.sapiens germline transcript of Ig heavy chain variable region (V).
								J00231	H.sapiens germline transcript of Ig heavy chain variable region (V).
								L07310	H.sapiens germline transcript of Ig heavy chain variable region (V).
								L07311	H.sapiens germline transcript of Ig heavy chain variable region (VDJ).
								L07312	H.sapiens germline transcript of Ig heavy chain variable region (V).
								J00257	H.sapiens mRNA for immunoglobulin heavy chain V-region (clone CDN3HA10).
								M17748	H.sapiens mRNA for immunoglobulin heavy chain V-region (clone CDN3IA2).
								M17751	H.sapiens mRNA for immunoglobulin heavy chain V-region (clone CDN3IA5).
								L26399	H.sapiens mRNA for immunoglobulin heavy chain V-region (clone CDN3IB6).
								M21385	H.sapiens mRNA for immunoglobulin heavy chain V-region (clone CDN3IC1).
								M21388	H.sapiens mRNA for immunoglobulin heavy chain V-region (clone CDN3IC10).
								M24668	H.sapiens mRNA for immunoglobulin heavy chain V-region (clone CDN3IC11).
								M24669	H.sapiens mRNA for immunoglobulin heavy chain V-region (clone CDN3IC2).
								M28136	H.sapiens mRNA for immunoglobulin heavy chain V-region (clone CDN3IC3).
								L11699	H.sapiens mRNA for immunoglobulin heavy chain V-region (clone CDN3ID1).
								M29470	H.sapiens mRNA for immunoglobulin heavy chain V-region (clone CDN3ID5).
								L39128	H.sapiens mRNA for immunoglobulin heavy chain V-region (clone CDN3ID7).
								M34021	H.sapiens mRNA for immunoglobulin heavy chain V-region (clone CDN3IE4).
								M34023	H.sapiens mRNA for immunoglobulin heavy chain V-region (clone CDN3IF3).
								M34024	H.sapiens mRNA for immunoglobulin heavy chain V-region (clone CDN3IG2).
								M34025	H.sapiens IG VH mRNA (clone mAB 61VH).
								M34026	H.sapiens IG VH mRNA (clone mAB 63VH).
								M34027	H.sapiens IG VH mRNA (clone mAB 67VH).
								M34029	H.sapiens IG VH gene (clone MLH4-1).
								M34030	H.sapiens mRNA P2 for IG heavy chain variable region.
								M34031	H.sapiens rearranged mRNA for IG heavy chain variable region.
								M34032	H.sapiens mRNA P29 for IG heavy chain variable region.
								L23556	H.sapiens mRNA P30 for IG heavy chain variable region.
								L23557	H.sapiens mRNA P32 for IG heavy chain variable region.
								L23558	H.sapiens mRNA P4 for IG heavy chain variable region.
								L23559	H.sapiens mRNA P46 for IG heavy chain variable region.
								L23560	H.sapiens mRNA for rearranged Ig heavy chain variable region (VH4) (VDJ) HSVIGD1.
								L23561	H.sapiens mRNA for rearranged Ig heavy chain variable region (VH4) (VDJ) HSVIGD10.
								L23562	H.sapiens mRNA for rearranged Ig heavy chain variable region (VH4) (VDJ) HSVIGD8.
								L23563	H.sapiens mRNA for rearranged Ig heavy chain variable region (VH4) (VDJ) HSVIGD9.
								L23564	H.sapiens heavy chain variable region.
								L23565	H.sapiens heavy chain variable region.
								L23566	H.sapiens heavy chain variable region.
								L23567	H.sapiens heavy chain variable region.
								L23568	H.sapiens heavy chain variable region.
								L23569	H.sapiens rearranged WHR gene for immunoglobulin heavy-chain variable region.
								L23570	H.sapiens mRNA for immunoglobulin heavy chain, Fab fragment, clones PDG13/PDG17/PDG31/PDG37.
								M57944	Homo sapiens mRNA for IgG1 rheumatoid factor immunoglobulin heavy chain, clone 1012.
								M57945	Homo sapiens mRNA for IgM rheumatoid factor immunoglobulin heavy chain, clones 1042, 785, 786, 854, 856, 881.
								L22587	Homo sapiens mRNA for IgA rheumatoid factor immunoglobulin heavy chain, clones 1341, 1354, 1346, 1349, 1352.
								M87789	Homo sapiens mRNA for immunoglobulin heavy chain variable region.
								M81847	H.sapiens mRNA for immunoglobulin, heavy chain, VDJ region.
								M81848	H.sapiens mRNA for immunoglobulin, heavy chain, VDJ region.
								L23516	H.sapiens mRNA for immunoglobulin, heavy chain, VDJ region.
								L23517	H.sapiens rearranged immunoglobulin heavy chain variable region gene, Framework1 to J (#31).
								L23518	H.sapiens rearranged immunoglobulin heavy chain variable region gene, framework1 to J (#34).

								M28074	H.sapiens rearranged immunoglobulin heavy chain variable region gene, leader to J (#35).
								L03385	H.sapiens rearranged immunoglobulin heavy chain variable region gene, framework1 to J (#101).
								L33025	H.sapiens rearranged immunoglobulin heavy chain variable region gene, leader to J (#102).
								L33035	H.sapiens rearranged immunoglobulin heavy chain variable region gene, leader to J (#104).
								L33037	H.sapiens rearranged immunoglobulin heavy chain variable region gene, leader to J (#105).
								L33039	H.sapiens rearranged immunoglobulin heavy chain variable region gene, leader to J (#106).
								L03815	H.sapiens rearranged immunoglobulin heavy chain variable region gene, leader to J (#107).
								L03816	H.sapiens rearranged immunoglobulin heavy chain variable region gene, leader to J (#108).
								L03818	H.sapiens rearranged immunoglobulin heavy chain variable region gene, leader to J (#111).
								L03819	H.sapiens mRNA for rearranged immunoglobulin VH-CE in peanut allergy (CO 9.4).
								L03820	H.sapiens mRNA for rearranged immunoglobulin VH-CE in peanut allergy (CO 15.4).
								L25291	H.sapiens mRNA for rearranged immunoglobulin VH-CE in peanut allergy (CO 4.4).
								L25292	H.sapiens mRNA for rearranged immunoglobulin VH-CE in peanut allergy (CO 10.4).
								L25294	H.sapiens mRNA for rearranged immunoglobulin VH-CE in peanut allergy (KH 1.4).
								L03823	H.sapiens mRNA for rearranged immunoglobulin VH-CE in peanut allergy (KH 7.4).
								L03826	H.sapiens mRNA for rearranged immunoglobulin VH-CE in peanut allergy (KH 15.4).
								L03829	H.sapiens mRNA for immunoglobulin heavy chain variable region (A3-M2; VH3, 3-23/DP-47).
								L03830	H.sapiens mRNA for immunoglobulin heavy chain variable region (A3-C9; VH3, 3-07/DP-54).
								M99601	H.sapiens mRNA for immunoglobulin heavy chain variable region (A3-J2; VH3, 3-07/DP-54).
								M62726	H.sapiens mRNA for immunoglobulin heavy chain variable region (A3-D3; VH3, 3-07/DP-54).
								M62728	H.sapiens mRNA for immunoglobulin heavy chain variable region (A3-E4; VH3, 3-53/DP-42).
								M62731	Homo sapiens rearanged immunoglobulin VH-CE in peanut allergy (CO 4.1).
								M99607	Homo sapiens rearanged immunoglobulin VH-CE in peanut allergy (CO 6.1).
								M99609	Homo sapiens mRNA for immunoglobulin heavy chain variable region, clone ToPA101.
								L14821	Homo sapiens mRNA for immunoglobulin heavy chain variable region, clone ToPA230.
								L19913	Homo sapiens mRNA for immunoglobulin heavy chain variable region, clone ToPA138.
								L19918	Homo sapiens mRNA for immunoglobulin heavy chain variable region, clone ToPA114.
								M90808	Homo sapiens mRNA for immunoglobulin heavy chain variable region, clone ToPG435.
								L06101	Homo sapiens mRNA for immunoglobulin heavy chain variable region, clone ToPG331.
								M18508	Homo sapiens mRNA for immunoglobulin heavy chain variable region, clone ToPG344.
								M18510	Homo sapiens mRNA for immunoglobulin heavy chain variable region, clone ToPG410.
								M18512	Homo sapiens mRNA for immunoglobulin heavy chain variable region, clone ToPG426.
								M18513	Homo sapiens mRNA for immunoglobulin heavy chain variable region, clone ToPG437.
								M18515	Homo sapiens mRNA for immunoglobulin heavy chain variable region, clone ToPG438.
								M18516	Homo sapiens mRNA for immunoglobulin heavy chain variable region, clone ToPG440.
								M18517	Homo sapiens mRNA for immunoglobulin heavy chain variable region, clone ToPG342.
								M18518	Homo sapiens mRNA for immunoglobulin heavy chain variable region, clone ToPA208.
								M18519	Homo sapiens mRNA for immunoglobulin heavy chain variable region, clone ToPA113.
								M18520	Homo sapiens mRNA for immunoglobulin heavy chain variable region, clone ToPA214.
								M18521	Homo sapiens mRNA for immunoglobulin heavy chain variable region, clone ToPA110.
								L26533	Homo sapiens mRNA for immunoglobulin heavy chain variable region, clone ToPA137.
								L26535	Homo sapiens mRNA for immunoglobulin heavy chain variable region, clone ToPG341.
								L43083	Homo sapiens mRNA for immunoglobulin heavy chain variable region, clone ToPG405.
								L43085	Human anti-glycoprotein 120 monoclonal antibody variable region, diversity region, and joining region mRNA.
								L43087	Human mRNA sequence.
								L43091	Homo sapiens monospecific anti-ssDNA antibody heavy chain variable region mRNA, complementarity determining regions 1-3
								L23931	and framework regions 1-4.
								L23932	Human anti-ssDNA antibody heavy chain variable region mRNA, complementarity determining regions 1-3 and framework
								L23933	regions 1-4.
								L38894	Human Ig rearranged heavy chain, V region.
								L26898	Homo sapiens Ig rearranged H-chain mRNA V region.
								M16951	Homo sapiens Ig rearranged H-chain mRNA V region.
								L12186	Homo sapiens Ig rearranged H-chain mRNA V region.
								L12188	Homo sapiens Ig rearranged H-chain mRNA V region.

								L12189	Homo sapiens Ig rearranged H-chain mRNA V region.
								L12191	Human Ig rearranged alpha-chain mRNA VH1-DN1-JH4 region, monoreactive, partial cds.
								L12194	Human Ig rearranged alpha-chain mRNA VH3-DM2-JH4 region, monoreactive, partial cds.
								L12196	Human Ig rearranged alpha-chain mRNA VH3-Dxp'4-JH4 region, polyreactive, partial cds.
								L12197	Human Ig rearranged alpha-chain mRNA VH4-DLR2-JH4 region, polyreactive, partial cds.
								L06021	Homo sapiens Ig rearranged alpha-chain mRNA V-C-region, 5' end.
								L06023	Homo sapiens (clone A2618) rearranged Ig alpha-1 chain VH6-CH1 region (IGHA1) mRNA, partial cds.
								M74018	Homo sapiens (clone A5219) rearranged Ig alpha-1 chain VH6-CH1 region (IGHA1) mRNA, partial cds.
								M71217	Human IgG4 chain V-region CH1 domain mRNA.
								M71219	Human Ig gamma-chain V-D-J region (HCV-63), partial cds.
								M71220	Human (clone MAb105) Ig H-chain (IGH@) mRNA, VH-III subgroup mature peptide.
								M71225	Human (clone MAb58) Ig H-chain (IGH@) mRNA, partial cds, VH-IV subgroup.
								M71226	Human (clone MAb59) Ig H-chain (IGH@) mRNA, partial cds, VH-III subgroup.
								M71227	Homo sapiens Ig rearranged gamma-chain mRNA; variable, diversity, joining region hybridoma.
								M71228	Homo sapiens Ig rearranged gamma-chain mRNA; variable, diversity, joining region hybridoma.
								M71229	Human Ig rearranged gamma-chain mRNA V-region, 5' end of cds (from clone S1P15).
								M71230	Human Ig rearranged gamma-chain mRNA V-region, 5' end of cds (from clone S1P9).
								M71231	Human Ig rearranged gamma-chain mRNA V-region, partial cds (from clone S2P10).
								M71234	Homo sapiens Ig gamma-chain V-region (IGHV@) mRNA, partial cds.
								M86599	Homo sapiens Ig rearranged H-chain V-region mRNA, partial cds.
								L06610	Human Ig rearranged H-chain mRNA V-region sequence.
								L29121	Human Ig gamma3 heavy chain disease OMM protein mRNA.
								L29122	Human Ig rearranged H-chain mRNA V3-D-J6-region, partial cds from clone 8.
								L29153	Human Ig rearranged H-chain mRNA V3-D-J6-region, partial cds from clone 19.
								L29155	Homo sapiens Ig rearranged H-chain mRNA V3-D-J1-region, partial cds from clone 7.
								L01410	Human ig mu H-chain constant region, partial CH3 domain, mRNA.
								L04324	Human Ig rearranged mu heavy chain mRNA V-region (VH26) from B-cell hybridoma cell line 18/9.
								L04325	Human Ig rearranged mu heavy chain mRNA V-region (V-D-J) from B-cell hybridoma cell line 8E10.
								L04327	Homo sapiens immunoglobulin heavy chain VDJ region mRNA, partial cds.
								L04328	Human active Ig mu-chain mRNA V-region (VDJ), 5' end, clone mu-11-8.
								L04329	Human unproductively rearranged Ig mu-chain mRNA V-region (VD), 5' end, clone mu-3A1A.
								L04330	Human Ig rearranged H-chain V-region mRNA (C-D-JH4), complete cds.
								L04332	Human Ig rearranged H-chain V-region mRNA (C-D-JH6), complete cds.
								L04323	Human Ig H chain mRNA V-region (V-D-J) heterohybridoma.
								L14453	Human immunoglobulin heavy chain variable region complementarity region 3 (CDR3) mRNA.
								L14456	Human Ig rearranged anti-myelin H-chain mRNA V-J4-region, hybridoma AE6-5, 5' end.
								M74300	Homo sapiens Ig heavy chain V-region mRNA, partial cds.
								L34164	Human fetal Ig heavy chain variable region (clone 74P1) mRNA, partial cds.
								L21726	Human fetal Ig heavy chain variable region (clone M26) mRNA, partial cds.
								L21727	Human fetal Ig heavy chain variable region (clone M43) mRNA, partial cds.
								L21729	Human fetal Ig heavy chain variable region (clone M44) mRNA, partial cds.
								L21730	Human fetal Ig heavy chain variable region (clone M49) mRNA, partial cds.
								L21731	Human fetal Ig heavy chain variable region (clone M60) mRNA, partial cds.
								L21732	Human fetal Ig heavy chain variable region (clone M71) processed pseudogene mRNA, partial cds.
								L21733	Human fetal Ig heavy chain variable region (clone M72) mRNA, partial cds.
								L21735	Human fetal Ig heavy chain variable region (clone M74) mRNA, partial cds.
								L21737	Human fetal Ig heavy chain variable region (clone M85) mRNA, 5' end.
								L21738	Human IgH chain VDJC region mRNA, partial cds.
								L21739	Human IgH chain VDJC region mRNA, partial cds.
								L21740	Human IgH chain VDJ region mRNA, partial cds.
								L21741	Human IgH chain VDJC region mRNA, partial cds.
								L21742	Human IgH chain VDJH region mRNA, partial cds.
								L21743	Human IgH chain VDJC region mRNA, partial cds.
								L21745	Human IgH chain VDJC region mRNA, partial cds.

								L21747	Human IgH chain VDJ region mRNA, partial cds.
								L21748	Human IgH chain VDJC region mRNA, partial cds.
								M85255	Human IgH chain VDJC region mRNA, partial cds.
								L01278	Human IgH chain VDJRCregion mRNA, partial cds.
								M87268	Human IgH chain VDJC region mRNA, partial cds.
								L32897	Human IgH chain VDJC region mRNA, partial cds.
								L14819	Human IgH chain VDJC region mRNA, partial cds.
								L14822	Human IgH chain VDJC region mRNA, partial cds.
								L14818	Human Ig germline H-chain V-A95 region.
								L48237	Human Ig germline H-chain V-A57 region.
								L48241	Human immunoglobulin heavy chain (IGH@) mRNA, V region, partial cds.
								L26907	Human (hybridoma H210) anti-hepatitis A IgG variable region, constant region, complementarity-determining regions mRNA, complete cds.
								L04334	
								L04339	Human (clone Fb9) immunoglobulin F heavy chain LS-Cf mRNA, exon 1 and 5'cds.
								L04340	Human (clone 73 2-33) immunoglobulin F heavy chain LS-Cf mRNA, exon 1 and 5'cds.
								L04342	Human Ig rearranged gamma-chain, V-DXP'4-JH6c, complete cds.
								L04343	Human Ig rearranged gamma-chain, V-DK4-JH3, complete cds.
								L04344	Human Ig rearranged gamma-chain, V-DXP'1-JH4b, complete cds.
								L04345	Homo sapiens immunoglobulin mu chain VDJC region (IgM) mRNA, partial cds.
								M26435	Human Ig rearranged mu-chain mRNA variable region (V3), diversity region, joining region (J4), partial cds.
								M27127	Human immunoglobulin mu heavy chain (IGHM) mRNA variable region, joining region, and diversity region.
								M26997	Human immunoglobulin mu heavy chain (IGHM) mRNA variable region, diversity region, joining region, and constant region.
								M27125	Human immunoglobulin mu heavy chain (IGHM) mRNA variable region, diversity region, and joining region.
								M99372	Human immunoglobulin mu heavy chain (IGHM) mRNA variable region, diversity region, and joining region.
								L23571	Human IG rearranged H-chain mRNA V-region, partial cds.
								L21960	Human IG rearranged H-chain mRNA V-region, partial cds.
								L21958	Human IG rearranged H-chain mRNA V-region, partial cds.
								L21957	Human IG rearranged H-chain mRNA V-region, partial cds.
								M24236	Human IG rearranged H-chain mRNA V-region, partial cds.
								L43116	Human polyreactive Ig rearranged mu-chain, V-D21/10-JH4, partial cds.
								M35478	Homo sapiens polyreactive immunoglobulin mu-chain variable region (V-DN2-JH4) mRNA, partial cds.
								D83675	Homo sapiens monoreactive immunoglobulin mu-chain variable region (V-Dk4-JH2) mRNA, partial cds.
								D83676	Human IG rearranged H-chain mRNA V-region, partial cds.
								D83677	Human IG rearranged H-chain mRNA V-region, partial cds.
								D83678	Human IG rearranged H-chain mRNA V-region, partial cds.
								D83682	Human IG rearranged H-chain mRNA V-region, partial cds.
								D83684	Human (clone R6B8H) Ig rearranged H-chain mRNA V-region, 5' end.
								D83686	Human IgH chain (monospecific rheumatoid factor) mRNA VDJ-region.
								D83688	Human IgH chain (monospecific rheumatoid factor) mRNA VDJ-region.
								D83689	Human IgH chain (monospecific rheumatoid factor) mRNA VDJ-region.
								D83693	Human (clone R5A3H) Ig rearranged H-chain mRNA V-region, 5' end.
								D83694	Human (clone B5G10H) Ig rearranged H-chain mRNA V-region, 5' end.
								S42403	Homo sapiens productively rearranged IgH mRNA, V3b region.
								S42567	Human clone SpA1-17VH immunoglobulin heavy-chain subgroup VIII mRNA V-D-J region, partial cds.
								S50735	Human clone SpA3-33VH immunoglobulin heavy-chain subgroup VIII mRNA V-D-J region, partial cds.
								S55271	Homo sapiens immunoglobulin heavy chain variable region VH2 (IGH@) mRNA, partial cds.
								S55277	Homo sapiens immunoglobulin heavy chain V-region (IGH@) mRNA, partial cds.
								S55735	Human (fetal) IgM chain V-region (VDJ) mRNA, clone 2P1.
								S56197	Human (fetal) rearranged IgM chain VDJ region mRNA, clone 15P1.
								S59161	Human (fetal) rearranged IgM chain VDJ-region mRNA, clone 20P3.
								S64473	Human (fetal) rearranged IgM chain VDJ-region mRNA, clone 30P1.
								S65196	Human (fetal) Ig rearranged H-chain VDJ-region mRNA, clone 38P1.
								S66098	Human (fetal) Ig rearranged H-chain VDJ-region mRNA, clone 51P1.
								S67984	Human (fetal) Ig rearranged H-chain VDJ-region mRNA, clone 56P1.

								S70739	Human Ig rearranged H-chain VDJ-region mRNA, clone 58P2.
								S71250	Human (fetal) Ig rearranged H-chain VDJ-region mRNA, clone 60P1.
								S71435	Human (fetal) Ig rearranged H-chain VDJ-region mRNA, clone 60P2.
								S71439	Human (fetal) Ig rearranged H-chain VDJ-region mRNA, clone 63P1.
								S72729	Homo sapiens immunoglobulin variable region mRNA, partial cds.
								S73132	Homo sapiens immunoglobulin variable region mRNA, partial cds.
								S73138	Homo sapiens (clone a5vl) immunogloublin light chain mRNA, V-region.
								S73912	Homo sapiens (clone bouvh) immunogloublin heavy chain mRNA, V-region.
								S73961	Homo sapiens (clone budvh) immunoglobulin heavy chain mRNA, V-region.
								S74640	Homo sapiens (clone h5vh) immunoglobulin heavy chain mRNA, variable region.
								S74642	Homo sapiens (clone mAb 18) IgH chain mRNA, VDJ region, heterohybridoma F3B6.
								S75886	Homo sapiens (clone mAb 21) IgH chain mRNA, VDJ region, heterohybridoma F3B6.
								S75888	Homo sapiens (clone mAb 26) process-pseudogene IgH chain mRNA, VDJ region, heterohybridoma F3B6.
								S75891	Homo sapiens (clone MEA11/65VH) Ig heavy chain V-region (VH6) mRNA, partial cds.
								S76144	Homo sapiens immunoglobulin heavy chain (IGH) mRNA, VJ region, partial cds.
								S76904	Human Ig mu-chain mRNA VDJ4-region, 5' end, from heterohybridoma K5G5.
								S77600	Human (clone: EBV R16) immunoglobulin rearranged mu-chain mRNA Vh3-D-J4 region, partial cds.
								S85240	Human (clone: EBV R16) immunoglobulin rearranged mu-chain mRNA V3-D-J region, partial cds.
								U00486	Human (clone: R16) immunoglobulin rearranged mu-chain mRNA Vh4-D-J6 region, partial cds.
								U00487	Human (clone:EBV R16) immunoglobulin rearranged mu-chain mRNA Vh4-D-J5 region, partial cds.
								U00488	Human (clone: EBV R16) immunoglobulin rearranged alpha-chain mRNA Vh4-D-J6 region, partial cds.
								U00492	Homo sapiens immunoglobulin mu chain Vh4-D-J2 region (IGHM) mRNA, partial cds.
								U00493	Human (clone: EBV R16) immunoglobulin rearranged mu-chain mRNA Vh4-D-J4 region, partial cds.
								U00494	Human antiplatelet immunoglobulin heavy chain (IGH@) variable region mRNA, partial cds.
								U00495	Human antiplatelet immunoglobulin heavy chain (IGH@) variable region mRNA, partial cds.
								U00497	Homo sapiens immunoglobulin heavy chain V region mRNA, partial cds.
								U00498	Human Ig active gamma-chain V(H), D(J), J(H) mRNA partial cds, clone EB10.
								U00499	Human Ig active gamma-chain V(H), D(J), J(H) mRNA partial cds, clone EX8.
								U00500	Homo sapiens clone EB2 immunoglobulin heavy chain V-region mRNA, partial cds.
								U00501	Human Ig active gamma-chain V(H), D(J), J(H) mRNA partial cds, clone BB11.
								U00503	Human Ig active gamma-chain V(H), D(J), J(H) mRNA partial cds, clone BB9.
								U00504	Human Ig active gamma-chain V(H), D(J), J(H) mRNA partial cds, clone E5.
								U00525	Human Ig active gamma-chain V(H), D(J), J(H) mRNA partial cds, clone EE6.
								U00526	Human Ig active gamma-chain V(H), D(J), J(H) mRNA partial cds, clone FD10.
								U00529	Human Ig active gamma-chain V(H), D(J), J(H) mRNA partial cds, clone FF3.
								U00534	Human Ig active gamma-chain V(H), D(J), J(H) mRNA partial cds, clone FD2.
								U00535	Human Ig active gamma-chain V(H), D(J), J(H) mRNA partial cds, clone FE2.
								U00536	Human Ig rearranged H-chain mRNA V-D-J region, subgroup VH-III, partial cds.
								U00537	Human rearranged immunoglobulin heavy chain (IGH@) gene, 5' end.
								U00538	Human immunoglobulin heavy chain variable region (VH I family) from IgM rheumatoid factor.
								U00539	Human immunoglobulin heavy chain variable region (VH IV family) from IgM rheumatoid factor.
								U00540	Human immunoglobulin heavy chain variable region (VH III family) from IgM rheumatoid factor.
								U00541	Human immunoglobulin heavy chain variable region (VH III family) from IgM rheumatoid factor.
								U00542	Homo sapiens Ig rearranged gamma-1 chain (IGHG1) mRNA subgroup VH-III hybridoma, partial cds.
								U00545	Human (cell line: L2B10) Ig rearranged H-chain mRNA V-region, partial cds.
								U00546	Human (cell line: L2E5) Ig rearranged H-chain mRNA V-region, partial cds.
								U00547	Human (cell line: L2F10) Ig rearranged H-chain mRNA V-region, partial cds.
								U00548	Human (cell line: L2H7) Ig rearranged H-chain mRNA V-region, partial cds.
								U00549	Human (cell line: L3A10) Ig rearranged H-chain mRNA V-region, partial cds.
								U00551	Human (cell line: L3B2) Ig rearranged H-chain mRNA V-region, partial cds.
								U00552	Human (cell line: L3G5) Ig rearranged H-chain mRNA V-region, partial cds.
								U00554	Human (cell line: L2A12) Ig rearranged H-chain mRNA V-region, partial cds.
								U00555	Human Ig rearranged mu-chain gene V-N-D-N-J-region, complete cds.
								U00556	Human Ig rearranged mu-chain gene V-N-D-N-J-region, complete cds.

								U00557	Human immunoglobulin non-truncated mu-chain mRNA, 5' end.
								U00558	Human Ig rearranged mu-chain gene VH3-D21/10-JH2, complete cds., polyreactive.
								U00560	Human (patient GRE) rearranged IgM heavy chain variable region, diversity region, and joining region (VH6DJ), partial cds.
								U00561	Human (patient GRE) rearranged IgM heavy chain variable region, diversity region, and joining region (VH6DJ), partial cds.
								U00562	Human (patient GRE) rearranged IgM heavy chain variable region, diversity region, and joining region (VH6DJ), partial cds.
								U00563	Human (patient GRE) rearranged IgM heavy chain variable region, diversity region, and joining region (VH6DJ), partial cds.
								U00567	Human (patient GRE) rearranged IgM heavy chain variable region, diversity region, and joining region (VH6DJ), partial cds.
								U00568	Human (patient GRE) rearranged IgM heavy chain variable region, diversity region, and joining region (VH6DJ), partial cds.
								U00570	Human (patient GRE) rearranged IgM heavy chain variable region, diversity region, and joining region (VH6DJ), partial cds.
								U00571	Human (patient GRE) rearranged IgM heavy chain variable region, diversity region, and joining region (VH6DJ), partial cds.
								U00572	Human (patient MAR) rearranged IgM heavy chain variable region, diversity region, and joining region (VH6DJ), partial cds.
								U00575	Human (patient GRE) rearranged IgM heavy chain variable region, diversity region, and joining region (VH6DJ), partial cds.
								U00577	Human (patient MAR) rearranged IgM heavy chain variable region, diversity region, and joining region (VH6DJ), partial cds.
								U00578	Human (patient PAU) rearranged IgM heavy chain variable region, diversity region, and joining region (VH6DJ), partial cds.
								U00579	Human (patient PAU) rearranged IgM heavy chain variable region, diversity region, and joining region (VH6DJ), partial cds.
								U00580	Human (patient PAU) rearranged IgM heavy chain variable region, diversity region, and joining region (VH6DJ), partial cds.
								U00581	Human (patient PAU) rearranged IgM heavy chain variable region, diversity region, and joining region (VH6DJ), partial cds.
								U00582	Human (patient PAU) rearranged IgM heavy chain variable region, diversity region, and joining region (VH6DJ), partial cds.
								U00583	Human (patient MAR) rearranged IgM heavy chain variable region, diversity region, and joining region (VH6DJ), partial cds.
								U00586	Human (patient MAR) rearranged IgM heavy chain variable region, diversity region, and joining region (VH6DJ), partial cds.
								U00589	Homo sapiens immunoglobulin mu-chain VDJ-region (IGHM) mRNA, partial cds.
								U00590	Homo sapiens Ig rearranged mu-chain mRNA V-C-region, 5' end.
								U96277	Human IgM VDJ-region mRNA, complete cds.
								U96282	Human Ig rearranged mu-chain mRNA V-D-J region.
								U96283	Homo sapiens (ED6) rearranged processed pseudo-IgH mRNA, V3b region.
								U96284	Homo sapiens immunoglobulin heavy chain V-region (IGH@) mRNA, partial cds.
								U96286	Homo sapiens immunoglobulin heavy chain VH3b region (IGH) mRNA, partial cds.
								U96287	Homo sapiens (cell line B20) IgM mRNA.
								CR590925	Homo sapiens (cell line MF8) IgM mRNA, partial cds.
								CR592363	Human immunoglobulin heavy chain (IGH) mRNA, VDJ region, partial cds.
								CR594000	Human (cell line: s5B7) Ig rearranged H-chain mRNA V-region, partial cds.
								CR595242	Human Ig rearranged H-chain mRNA V-region, partial cds.
								CR603811	Human Ig rearranged H-chain mRNA V-region, partial cds.
								CR616485	Human Ig rearranged H-chain mRNA V-region, partial cds.
								CR617188	Human Ig rearranged H-chain mRNA V-region, partial cds.
								CR623477	Human Ig rearranged H-chain mRNA V-region, partial cds.
								CR933690	Human Ig rearranged H-chain mRNA V-region, partial cds.
								BX538118	Homo sapiens Ig leprosy-derived antibody TH9 heavy chain V region mRNA.
								BX640619	Homo sapiens antibody 47 immunoglobulin heavy chain variable region (IGHV@) mRNA, partial cds.
								BX640620	Human (antibody 26) IgH chain (IGHV@) mRNA, V4 region, 5'end.
								BX640621	Human (antibody 2022) unproductively rearranged IgH chain mRNA, V5 region, partial cds.
								BX640622	Homo sapiens immunoglobulin heavy chain (IGH@) mRNA, partial cds.
								BX640627	Homo sapiens IgH chain mRNA, VDJC region.
								BX640724	Human Ig rearranged gamma-chain mRNA, subgroup VH2, V-D-J region, partial cds.
								BX640822	Human Ig rearranged gamma-chain mRNA, subgroup VH3, V-D-J region, 5' end of cds.
								BX640853	Human Ig rearranged mu-chain mRNA, subgroup VH6, V-D-J region, 5' end of cds.
								BX640874	Human Ig mu-chain mRNA V3b-D-J4 region, 5' end.
								BX640947	Homo sapiens immunoglobulin heavy chain (LH92 VH) mRNA, V-region.
								AY670707	Human Ig mu-chain transgenic mRNA, partial cds.
								AY670708	Human mRNA for immunoglobulin M (IgM), partial cds (VH3-N-D-N-JH6), clone H2-8E.
								AY670711	Homo sapiens mRNA for Immunoglobulin M, partial cds, clone: UB1-24E.
								AY670712	Human mRNA for immunoglobulin M (IgM), partial cds (VH3-N-D-N-JH3), clone AD-181E.
								AY670713	Human mRNA for immunoglobulin M (IgM), partial cds (VH3-N-D-N-JH4), clone IW2-101E.
								AY670714	Human mRNA for immunoglobulin M (IgM), partial cds (VH3-N-D-N-JH6), clone H2-47E.

								AY670717<br>AY670719<br>AY670720<br>AY670721<br>AY670722<br>AY670725<br>AY670726<br>AY670728<br>AY670729<br>AY670731<br>AY670734<br>AY670736<br>AY670738<br>AY670739<br>AY670740<br>AY670742<br>AY670744<br>AY670746<br>AY670747<br>AY670750<br>AY670751<br>AY670753<br>AY670756<br>AY670758<br>AY670759<br>AY670760<br>AY670761<br>AY670762<br>AY670763<br>AY670764<br>AY670765<br>AY670766<br>AY670769<br>AY670771<br>AY670773<br>AY670774<br>AY670775<br>AY670776<br>AY670778<br>AY670779<br>AY670781<br>AY670783<br>AY670784<br>AY670785<br>AY670786<br>AY670790<br>AY670793<br>AY670796<br>AY670797<br>AY670799<br>AY670802<br>AY670803<br>AY670804<br>AY670805	Human mRNA for immunoglobulin M (IgM), partial cds (VH3-N-D-N-JH4), clone H2-27E.<br>Human mRNA for immunoglobulin M (IgM), partial cds (VH3-N-D-N-JH4), clone H2-21E.<br>Human mRNA for immunoglobulin M (IgM), partial cds (VH3-N-D-N-JH4), clone UB1-17E.<br>Human mRNA for immunoglobulin M (IgM), partial cds (VH3-N-D-N-JH4), clone IW2-105E.<br>Human mRNA for immunoglobulin M (IgM), partial cds (VH3-N-D-N-JH6), clone H2-35E.<br>Human mRNA for immunoglobulin M (IgM), partial cds (VH3-N-D-N-JH4), clone UB1-53E.<br>IgVH=anti-Rh(D) antibody [human, mRNA Partial, 773 nt].<br>Ig I epsilon {germline region exon} [human, peripheral blood mononuclear cells, IL-4 stimulated, mRNA Partial, 82 nt].<br>immunoglobulin M heavy chain V region=anti-lipid A antibody [human, hybridoma cell line HR78, mRNA Partial, 528 nt].<br>immunoglobulin epsilon chain constant region=membrane-bound form {M:A, alternatively spliced, exon C4, membrane domain<br>exon 1} [human, B cell myeloma U-266, mRNA Partial, 230 nt].<br>immunoglobulin epsilon chain membrane-bound form {3' region, C4 non-coding region, M:D, alternatively spliced, membrane<br>domain exon 1} [human, B cell myeloma U-266, mRNA Partial, 357 nt].<br>Homo sapiens immunoglobulin A1-A2 lambda hybrid GAU heavy chain mRNA, partial cds.<br>VH4=rheumatoid factor G9 heavy chain [human, rheumatoid synovial cells, mRNA Partial, 357 nt].<br>Ig VH=anti-single/double-stranded DNA antibody NE-1 heavy chain variable region [human, B-cells, mRNA Partial, 459 nt].<br>immunoglobulin heavy chain V region [human, X-linked agammaglobulinemia patients, B lymphoblastoid cell lines, mRNA<br>Partial, 429 nt].<br>Ig gamma VH3 [human, chronic lymphocytic leukemia case P103, mRNA Partial Mutant, 294 nt].<br>Ig VH [human, mRNA Partial, 366 nt].<br>Ig VHII JH5=anti-HIV gp120 antibody heavy chain variable region [human, hybridoma HBW4, mRNA Partial, 441 nt].<br>Homo sapiens anti-DNA monoclonal antibody WRI176 beta heavy chain variable region mRNA, partial cds.<br>Ig VH6=IgG heavy chain variable region {clone 3G13} [human, tonsil B cells, mRNA Partial, 372 nt].<br>epsilon , IgE=membrane-bound IgE, epsilon m/s isoform {alternative splicing} [human, mRNA Partial, 216 nt].<br>epsilon , IgE=membrane-bound IgE, epsilon m/1 isoform {alternative splicing} [human, mRNA Partial, 166 nt].<br>IgH=T-cell receptor V beta-specific IgM kappa autoantibody heavy chain variable region [human, IARC307 cell line, mRNA<br>Partial, 459 nt].<br>IgM VH4=IgM rheumatoid factor D53 heavy chain variable region [human, rheumatoid arthritis patient, mRNA Partial, 366 nt].<br>Homo sapiens IgM rheumatoid factor H6 mRNA, partial cds.<br>Homo sapiens IgM autoantibody heavy chain mRNA, partial cds.<br>Ig VH=immunoglobulin heavy chain VDJ region [human, chronic lymphocytic leukemia patient DIE, mRNA Partial, 336 nt].<br>Ig VH4=Ig heavy chain variable region {clone E28} [human, systemic lupus erythematosus, mRNA Partial, 378 nt].<br>Ig VH4=Ig heavy chain variable region {clone H44} [human, systemic lupus erythematosus, mRNA Partial, 262 nt].<br>IgH=immunoglobulin heavy chain variable region {clone M5} [human, multiple myeloma patient, mRNA Partial, 246 nt].<br>IgH=immunoglobulin heavy chain variable region {clone M3} [human, multiple myeloma patient, mRNA Partial, 225 nt].<br>IgH=immunoglobulin heavy chain variable region {clone M11} [human, multiple myeloma patient, mRNA Partial, 225 nt].<br>Ig gamma 1=Ig heavy chain {heavy chain hinge region} [human, mRNA Partial, 48 nt].<br>Ig VH=immunoglobulin heavy chain variable region {clone DDM-1} [human, thymus, mRNA Partial, 249 nt].<br>anti-carcinoma surface antigen monoclonal antibody CR4E8=IgM mu chain variable region {CDR region} [human, CR4E8<br>hybridoma cells, mRNA Partial, 456 nt].<br>Homo sapiens immunoglobulin mu heavy chain variable (Ig VHB2-mu) mRNA, partial cds.<br>Human immunoglobulin heavy chain variable region (clone Amu1a3-3) mRNA, partial cds.<br>Human immunoglobulin heavy chain variable region (clone Amu1a4-3) mRNA, partial cds.<br>Human immunoglobulin heavy chain variable region (clone Amu1b6-3) mRNA, partial cds.<br>Human immunoglobulin heavy chain variable region (clone Amu1c9-3) mRNA, partial cds.<br>Human immunoglobulin heavy chain variable region (clone Amu1d1-3) mRNA, partial cds.<br>Human immunoglobulin heavy chain variable region (clone Amu1d10-3) mRNA, partial cds.<br>Human immunoglobulin heavy chain variable region (clone Amu1d11-3) mRNA, partial cds.<br>Human immunoglobulin heavy chain variable region (clone Amu1d3-3) mRNA, partial cds.<br>Human immunoglobulin heavy chain variable region (clone Amu1d4-3) mRNA, partial cds.<br>Human immunoglobulin heavy chain variable region (clone Amu1d8-3) mRNA, partial sequence.<br>Human immunoglobulin heavy chain variable region (clone Amu1e10-3) mRNA, partial cds.<br>Human immunoglobulin heavy chain variable region (clone Amu1e11-3) mRNA, partial cds.<br>Human immunoglobulin heavy chain variable region (clone 1e8-3) mRNA, partial cds.

								AY670807<br>AY670811<br>AY670812<br>AY670816<br>AY670817<br>AY670822<br>AY670823<br>AY670825<br>AY670833<br>AY670837<br>AY670839<br>AY670840<br>AY670842<br>AY670847<br>AY670848<br>AY670849<br>AY670850<br>AY670851<br>AY670852<br>AY670853<br>AY670854<br>AY670856<br>AY670857<br>AY670858<br>AY670859<br>AY670860<br>AY670861<br>AY670863<br>AY670865<br>AY670866<br>AY670868<br>AY670869<br>AY670870<br>AY670871<br>AY670872<br>AY670873<br>AY670874<br>AY670876<br>AY670877<br>AY670878<br>AY670879<br>AY670880<br>AY670882<br>AY670884<br>AY670885<br>AY670886<br>AY670887<br>AY670889<br>AY670890<br>AY670891<br>AY670893<br>AY670895<br>AY670896<br>AY670897	Human immunoglobulin heavy chain variable region (clone 1f2-3) mRNA, partial cds.<br>Human immunoglobulin heavy chain variable region (clone Agamma34-1) mRNA, partial cds.<br>Human immunoglobulin heavy chain variable region (clone Agamma35-1) mRNA, partial cds.<br>Human immunoglobulin heavy chain variable region (clone Agamma41-3) mRNA, partial cds.<br>Human immunoglobulin heavy chain variable region (clone Agamma6-1) mRNA, partial cds.<br>Human immunoglobulin heavy chain variable region (clone Agamma6-2) mRNA, partial cds.<br>Human immunoglobulin heavy chain variable region (clone Agamma6-3) mRNA, partial cds.<br>Human immunoglobulin heavy chain variable region (clone Agamma62-3) mRNA, partial cds.<br>Human immunoglobulin heavy chain variable region (clone Agamma8-3) mRNA, partial cds.<br>Human immunoglobulin heavy chain variable region (clone Amu1-1) mRNA, partial cds.<br>Human immunoglobulin heavy chain variable region (clone Amu1-3) mRNA, partial cds.<br>Human immunoglobulin heavy chain variable region (clone Amu10-2) mRNA, partial cds.<br>Human immunoglobulin heavy chain variable region (clone Amu10-3) mRNA, partial cds.<br>Human clone Amu3-2 Ig heavy chain variable region mRNA, partial cds.<br>Human clone Amu31-2 Ig heavy chain variable region mRNA, partial cds.<br>Human clone Amu32-2 Ig heavy chain variable region mRNA, partial cds.<br>Human clone Amu34-2 Ig heavy chain variable region mRNA, partial cds.<br>Human clone Amu35-2 Ig heavy chain variable region mRNA, partial cds.<br>Human clone Amu4-1 Ig heavy chain variable region mRNA, partial cds.<br>Human clone Amu4-3 Ig heavy chain variable region mRNA, partial cds.<br>Human clone Amu41-1 Ig heavy chain variable region mRNA, partial cds.<br>Human clone Amu43-2 Ig heavy chain variable region mRNA, partial cds.<br>Human clone Amu44-2 Ig heavy chain variable region mRNA, partial cds.<br>Human clone Amu45-2 Ig heavy chain variable region mRNA, partial cds.<br>Human clone Amu46-2 Ig heavy chain variable region mRNA, partial cds.<br>Human clone Amu5-1 Ig heavy chain variable region mRNA, partial sequence.<br>Human clone Amu5-2 Ig heavy chain variable region mRNA, partial cds.<br>Human clone Amu5-3 Ig heavy chain variable region mRNA, partial cds.<br>Human clone Amu52-1 Ig heavy chain variable region mRNA, partial cds.<br>Human immunoglobulin heavy chain variable region (clone Amu61-1) mRNA, partial cds.<br>Human immunoglobulin heavy chain variable region (clone Amu70-1) mRNA, partial cds.<br>Human immunoglobulin heavy chain variable region (clone Amu8-2) mRNA, partial cds.<br>Human immunoglobulin heavy chain variable region (clone Amu9-3) mRNA, partial cds.<br>Human immunoglobulin heavy chain variable region (clone Tmu10) mRNA, partial cds.<br>Human immunoglobulin heavy chain variable region (clone Tmu17) mRNA, partial cds.<br>Human immunoglobulin heavy chain variable region (clone Tmu46) mRNA, partial cds.<br>Human immunoglobulin heavy chain variable region (clone Tmu47) mRNA, partial cds.<br>Human immunoglobulin heavy chain variable region (clone Tmu49) mRNA, partial cds.<br>Human immunoglobulin heavy chain variable region (clone Tmu50) mRNA, partial sequence.<br>Human immunoglobulin heavy chain variable region (clone Tmu56) mRNA, partial cds.<br>Human immunoglobulin heavy chain variable region (clone Tmu58) mRNA, partial cds.<br>Human immunoglobulin heavy chain variable region (clone Tmu60) mRNA, partial cds.<br>Human immunoglobulin heavy chain variable region (clone Tmu75) mRNA, partial cds.<br>Human immunoglobulin heavy chain variable region (clone Tmu84) mRNA, partial cds.<br>Homo sapiens immunoglobulin heavy chain variable region mRNA, partial cds.<br>Homo sapiens Ig heavy chain VH3 region (VH3-33) mRNA, partial cds.<br>Homo sapiens Ig heavy chain VH3 region (VH3-7) mRNA, partial cds.<br>Homo sapiens Ig heavy chain VH4 region (VH4-59) mRNA, partial cds.<br>Homo sapiens Ig heavy chain VH3 region (VH3-30) mRNA, partial cds.<br>Homo sapiens Ig heavy chain VH3 region (VH3-23) mRNA, partial cds.<br>Homo sapiens Ig heavy chain VH4 region (VH4-61) mRNA, partial cds.<br>full-length cDNA clone CS0DB001YL04 of Neuroblastoma Cot 10-normalized of Homo sapiens (human).<br>full-length cDNA clone CS0DE006YK04 of Placenta of Homo sapiens (human).<br>full-length cDNA clone CS0DI017YH15 of Placenta Cot 25-normalized of Homo sapiens (human).

								AY670898	full-length cDNA clone CS0DG004YB13 of B cells (Ramos cell line) of Homo sapiens (human).
								AY670901	full-length cDNA clone CS0DI054YM15 of Placenta Cot 25-normalized of Homo sapiens (human).
								AY670902	full-length cDNA clone CS0DM007YB20 of Fetal liver of Homo sapiens (human).
								AY670906	full-length cDNA clone CS0DL006YI09 of B cells (Ramos cell line) Cot 25-normalized of Homo sapiens (human).
								AY670908	full-length cDNA clone CS0DB006YA08 of Neuroblastoma Cot 10-normalized of Homo sapiens (human).
								AY670909	Homo sapiens mRNA; cDNA DKFZp686F17185 (from clone DKFZp686F17185).
								AY670910	Homo sapiens mRNA; cDNA DKFZp686N02209 (from clone DKFZp686N02209).
								AY670911	Homo sapiens mRNA; cDNA DKFZp686H20196 (from clone DKFZp686H20196).
								AY670912	Homo sapiens mRNA; cDNA DKFZp686I15196 (from clone DKFZp686I15196).
								AY670913	Homo sapiens mRNA; cDNA DKFZp686K03196 (from clone DKFZp686K03196).
								AY670917	Homo sapiens mRNA; cDNA DKFZp686O01196 (from clone DKFZp686O01196).
								AY670920	Homo sapiens mRNA; cDNA DKFZp686P15220 (from clone DKFZp686P15220).
								AY670921	Homo sapiens mRNA; cDNA DKFZp686I15212 (from clone DKFZp686I15212).
								AY670923	Homo sapiens mRNA; cDNA DKFZp686M08189 (from clone DKFZp686M08189).
								AY670924	Homo sapiens mRNA; cDNA DKFZp686C11235 (from clone DKFZp686C11235).
								AY670925	Homo sapiens mRNA; cDNA DKFZp686C15213 (from clone DKFZp686C15213).
								AY670927	Homo sapiens mRNA; cDNA DKFZp686G11190 (from clone DKFZp686G11190).
								AY670928	Homo sapiens clone cbc3bm-A1 immunoglobulin mu heavy chain mRNA, partial cds.
								AY670929	Homo sapiens clone cbc3bm-A2 immunoglobulin mu heavy chain-like mRNA, partial sequence.
								AY670930	Homo sapiens clone cbc3bm-A5 immunoglobulin mu heavy chain-like mRNA, partial sequence.
								AY670932	Homo sapiens clone cbc3bm-A6 immunoglobulin mu heavy chain mRNA, partial cds.
								AY670933	Homo sapiens clone cbc3bm-A7 immunoglobulin mu heavy chain mRNA, partial cds.
								AY670935	Homo sapiens clone cbc3bm-A8 immunoglobulin mu heavy chain mRNA, partial cds.
								AY670936	Homo sapiens clone cbc3bm-B3 immunoglobulin mu heavy chain mRNA, partial cds.
								AY670937	Homo sapiens clone cbc3bm-B5 immunoglobulin mu heavy chain mRNA, partial cds.
								AY670938	Homo sapiens clone cbc3bm-B6 immunoglobulin mu heavy chain mRNA, partial cds.
								AY670941	Homo sapiens clone cbc3bm-B7 immunoglobulin mu heavy chain mRNA, partial cds.
								AY670943	Homo sapiens clone cbc3bm-B8 immunoglobulin mu heavy chain mRNA, partial cds.
								AY670944	Homo sapiens clone cbc3bm-C3 immunoglobulin mu heavy chain mRNA, partial cds.
								AY670945	Homo sapiens clone cbc3bm-C4 immunoglobulin mu heavy chain mRNA, partial cds.
								AY670947	Homo sapiens clone cbc3bm-C6 immunoglobulin mu heavy chain-like mRNA, partial sequence.
								AY670948	Homo sapiens clone cbc3bm-C7 immunoglobulin mu heavy chain mRNA, partial cds.
								AY670949	Homo sapiens clone cbc3bm-D1 immunoglobulin mu heavy chain-like mRNA, partial sequence.
								AY670951	Homo sapiens clone cbc3bm-D5 immunoglobulin mu heavy chain mRNA, partial cds.
								AY670952	Homo sapiens clone cbc3bm-D7 immunoglobulin mu heavy chain mRNA, partial cds.
								AY670953	Homo sapiens clone cbc3bm-E2 immunoglobulin mu heavy chain mRNA, partial cds.
								AY670956	Homo sapiens clone cbc3bm-E3 immunoglobulin mu heavy chain mRNA, partial cds.
								AY670957	Homo sapiens clone cbc3bm-E4 immunoglobulin mu heavy chain mRNA, partial cds.
								AY670958	Homo sapiens clone cbc3bm-E6 immunoglobulin mu heavy chain mRNA, partial cds.
								AY670959	Homo sapiens clone cbc3bm-F1 immunoglobulin mu heavy chain mRNA, partial cds.
								AY670962	Homo sapiens clone cbc3bm-F3 immunoglobulin mu heavy chain mRNA, partial cds.
								AY670965	Homo sapiens clone cbc3bm-F4 immunoglobulin mu heavy chain mRNA, partial cds.
								AY670967	Homo sapiens clone cbc3bm-F7 immunoglobulin mu heavy chain mRNA, partial cds.
								AY670969	Homo sapiens clone cbc3bm-F8 immunoglobulin mu heavy chain mRNA, partial cds.
								AY670971	Homo sapiens clone CBc3bmMK-d4 immunoglobulin mu heavy chain mRNA, partial cds.
								AY670972	Homo sapiens clone CBc3bmMK-e1 immunoglobulin mu heavy chain mRNA, partial cds.
								AY670976	Homo sapiens clone CBc3bmMK-e4 immunoglobulin mu heavy chain mRNA, partial cds.
								AY670978	Homo sapiens clone CBc3bmMK-e5 immunoglobulin mu heavy chain mRNA, partial cds.
								AY670979	Homo sapiens clone CBc3bmMK-e6 immunoglobulin mu heavy chain mRNA, partial cds.
								AY670980	Homo sapiens clone CBc3bmMK-e7 immunoglobulin mu heavy chain mRNA, partial cds.
								AY670981	Homo sapiens clone CBc3bmMK-e8 immunoglobulin mu heavy chain mRNA, partial cds.
								AY670983	Homo sapiens clone CBc3bmMK-f1 immunoglobulin mu heavy chain mRNA, partial cds.
								AY670984	Homo sapiens clone CBc3bmMK-f2 immunoglobulin mu heavy chain mRNA, partial cds.
								AY670985	Homo sapiens clone CBc3bmMK-f3 immunoglobulin mu heavy chain mRNA, partial cds.

								AY670986	Homo sapiens clone CBc3bmMK-f4 immunoglobulin mu heavy chain mRNA, partial cds.
								AY670987	Homo sapiens clone CBc3bmMK-f8 immunoglobulin mu heavy chain mRNA, partial cds.
								AY670988	Homo sapiens clone cbc3mk(2)-B6 immunoglobulin mu heavy chain mRNA, partial cds.
								AY670989	Homo sapiens clone cbc3mk(2)-C6 immunoglobulin mu heavy chain mRNA, partial cds.
								AY670990	Homo sapiens clone cbc3mk(2)-C7 immunoglobulin mu heavy chain mRNA, partial cds.
								AY670991	Homo sapiens clone cbc3mk(2)-D7 immunoglobulin mu heavy chain mRNA, partial cds.
								AY670993	Homo sapiens clone cbc3mk(2)-E6 immunoglobulin mu heavy chain mRNA, partial cds.
								AY670994	Homo sapiens clone cbc3mk(2)-F6 immunoglobulin mu heavy chain mRNA, partial cds.
								AY670996	Homo sapiens clone cbc3mk(2)-F7 immunoglobulin mu heavy chain mRNA, partial cds.
								AY670997	Homo sapiens clone cbc3mk(2)-G7 immunoglobulin mu heavy chain mRNA, partial cds.
								AY670998	Homo sapiens clone cbc3mk(2)-H7 immunoglobulin mu heavy chain mRNA, partial cds.
								AY670999	Homo sapiens clone cbc3mk(2)-i6 immunoglobulin mu heavy chain mRNA, partial cds.
								AY671000	Homo sapiens clone cbc3mk(2)-i7 immunoglobulin mu heavy chain mRNA, partial cds.
								AY671001	Homo sapiens clone cbc3mk(2)-J6 immunoglobulin mu heavy chain mRNA, partial cds.
								AY671002	Homo sapiens clone cbc3bmL(2)-B1 immunoglobulin mu heavy chain mRNA, partial cds.
								AY671003	Homo sapiens clone cbc3bmL(2)-G2 immunoglobulin mu heavy chain mRNA, partial cds.
								AY671004	Homo sapiens clone cbc3bmL(2)-i3 immunoglobulin mu heavy chain mRNA, partial cds.
								AY671006	Homo sapiens clone cbc3bmL(2)-K1 immunoglobulin mu heavy chain-like mRNA, partial sequence.
								AY671007	Homo sapiens clone cbc3bmL(2)-L3 immunoglobulin mu heavy chain mRNA, partial cds.
								AY671009	Homo sapiens clone CBc3bmML-a1b immunoglobulin mu heavy chain mRNA, partial cds.
								AY671010	Homo sapiens clone CBc3bmML-a2 immunoglobulin mu heavy chain mRNA, partial cds.
								AY671011	Homo sapiens clone CBc3bmML-a2b immunoglobulin mu heavy chain mRNA, partial cds.
								AY671012	Homo sapiens clone CBc3bmML-a3 immunoglobulin mu heavy chain-like mRNA, partial sequence.
								AY671013	Homo sapiens clone CBc3bmML-a4b immunoglobulin mu heavy chain mRNA, partial cds.
								AY671014	Homo sapiens clone CBc3bmML-b1b immunoglobulin mu heavy chain mRNA, partial cds.
								AY671015	Homo sapiens clone CBc3bmML-b3 immunoglobulin mu heavy chain mRNA, partial cds.
								AY671017	Homo sapiens clone CBc3bmML-c1 immunoglobulin mu heavy chain mRNA, partial cds.
								AY671019	Homo sapiens clone CBc3bmML-c1b immunoglobulin mu heavy chain mRNA, partial cds.
								AY671020	Homo sapiens clone CBc3bmML-c4b immunoglobulin mu heavy chain mRNA, partial cds.
								AY671021	Homo sapiens clone CBc3bmML-c5 immunoglobulin mu heavy chain mRNA, partial cds.
								AY671022	Homo sapiens clone CBc3bmML-c8 immunoglobulin mu heavy chain mRNA, partial cds.
								AY671023	Homo sapiens clone CBc3bmML-h1 immunoglobulin mu heavy chain mRNA, partial cds.
								AY671024	Homo sapiens clone CBc3bmML-i4 immunoglobulin mu heavy chain mRNA, partial cds.
								AY671025	Homo sapiens clone CBc3bmML-j2 immunoglobulin mu heavy chain mRNA, partial cds.
								AY671026	Homo sapiens clone CBc3bmML-j4 immunoglobulin mu heavy chain mRNA, partial cds.
								AY671027	Homo sapiens clone CBc3bmML-k2 immunoglobulin mu heavy chain mRNA, partial cds.
								AY671028	Homo sapiens clone 3ap11 immunoglobulin mu heavy chain mRNA, partial cds.
								AY671029	Homo sapiens clone 3ap12 immunoglobulin mu heavy chain mRNA, partial cds.
								AY671030	Homo sapiens clone 3ap13 immunoglobulin mu heavy chain mRNA, partial cds.
								AY671032	Homo sapiens clone 3ap14 immunoglobulin mu heavy chain mRNA, partial cds.
								AY671033	Homo sapiens clone 3ap16 immunoglobulin mu heavy chain mRNA, partial cds.
								AY671034	Homo sapiens clone 3ap17 immunoglobulin mu heavy chain mRNA, partial cds.
								AY671036	Homo sapiens clone 3ap18 immunoglobulin mu heavy chain mRNA, partial cds.
								AY671037	Homo sapiens clone 3ap19 immunoglobulin mu heavy chain mRNA, partial cds.
								AY671038	Homo sapiens clone 3ap21 immunoglobulin mu heavy chain mRNA, partial cds.
								AY671039	Homo sapiens clone 3ap22 immunoglobulin mu heavy chain mRNA, partial cds.
								AY671040	Homo sapiens clone 3ap23 immunoglobulin mu heavy chain mRNA, partial cds.
								AY671041	Homo sapiens clone 3ap24 immunoglobulin mu heavy chain mRNA, partial cds.
								AY671042	Homo sapiens clone 3ap25 immunoglobulin mu heavy chain mRNA, partial cds.
								AY671043	Homo sapiens clone 3ap26 immunoglobulin mu heavy chain mRNA, partial cds.
								AY671044	Homo sapiens clone 3ap28 immunoglobulin mu heavy chain mRNA, partial cds.
								AY671045	Homo sapiens clone 3ap3 immunoglobulin mu heavy chain mRNA, partial cds.
								AY671046	Homo sapiens clone 3ap30 immunoglobulin mu heavy chain mRNA, partial cds.
								AY671047	Homo sapiens clone 3ap32 immunoglobulin mu heavy chain mRNA, partial cds.

								AY671048	Homo sapiens clone 3ap33 immunoglobulin mu heavy chain mRNA, partial cds.
								AY671049	Homo sapiens clone 3ap34 immunoglobulin mu heavy chain mRNA, partial cds.
								AY671050	Homo sapiens clone 3ap35 immunoglobulin mu heavy chain mRNA, partial cds.
								AY671051	Homo sapiens clone 3ap36 immunoglobulin mu heavy chain mRNA, partial cds.
								AY671052	Homo sapiens clone 3ap37 immunoglobulin mu heavy chain mRNA, partial cds.
								AY671054	Homo sapiens clone 3ap38 immunoglobulin mu heavy chain mRNA, partial cds.
								AY671055	Homo sapiens clone 3ap4 immunoglobulin mu heavy chain mRNA, partial cds.
								AY671056	Homo sapiens clone 3ap40 immunoglobulin mu heavy chain mRNA, partial sequence.
								AY671057	Homo sapiens clone 3ap41 immunoglobulin mu heavy chain mRNA, partial cds.
								AY671058	Homo sapiens clone 3ap43 immunoglobulin mu heavy chain mRNA, partial cds.
								AY671059	Homo sapiens clone 3ap44 immunoglobulin mu heavy chain mRNA, partial cds.
								AY671060	Homo sapiens clone 3ap46 immunoglobulin mu heavy chain mRNA, partial cds.
								AY671061	Homo sapiens clone 3ap49 immunoglobulin mu heavy chain mRNA, partial cds.
								AY671063	Homo sapiens clone 3ap5 immunoglobulin mu heavy chain mRNA, partial cds.
								AY671064	Homo sapiens clone 3ap50 immunoglobulin mu heavy chain mRNA, partial cds.
								AY671066	Homo sapiens clone 3ap54 immunoglobulin mu heavy chain mRNA, partial cds.
								AY671067	Homo sapiens clone 3ap58 immunoglobulin mu heavy chain mRNA, partial cds.
								AY671069	Homo sapiens clone 3ap59 immunoglobulin mu heavy chain mRNA, partial cds.
								AY671070	Homo sapiens clone 3ap6 immunoglobulin mu heavy chain mRNA, partial cds.
								AY671071	Homo sapiens clone 3ap7 immunoglobulin mu heavy chain mRNA, partial cds.
								AY671072	Homo sapiens clone 3ap9 immunoglobulin mu heavy chain mRNA, partial cds.
								AY671074	Homo sapiens clone 1ap1 immunoglobulin mu heavy chain-like mRNA, partial sequence.
								AY671077	Homo sapiens clone 1ap10 immunoglobulin mu heavy chain mRNA, partial cds.
								AY671080	Homo sapiens clone 1ap11 immunoglobulin mu heavy chain mRNA, partial cds.
								AY671081	Homo sapiens clone 1ap14 immunoglobulin mu heavy chain mRNA, partial cds.
								AY671083	Homo sapiens clone 1ap15 immunoglobulin mu heavy chain-like mRNA, partial sequence.
								AY671084	Homo sapiens clone 1ap19 immunoglobulin mu heavy chain mRNA, partial cds.
								AY671085	Homo sapiens clone 1ap21 immunoglobulin mu heavy chain-like mRNA, partial sequence.
								AY671086	Homo sapiens clone 1ap22 immunoglobulin mu heavy chain mRNA, partial cds.
								AY671087	Homo sapiens clone 1ap24 immunoglobulin mu heavy chain mRNA, partial cds.
								AY671091	Homo sapiens clone 1ap25 immunoglobulin mu heavy chain mRNA, partial cds.
								AY671096	Homo sapiens clone 1ap26 immunoglobulin mu heavy chain mRNA, partial cds.
								AY671097	Homo sapiens clone 1ap27 immunoglobulin mu heavy chain-like mRNA, partial sequence.
								AY671101	Homo sapiens clone 1ap30 immunoglobulin mu heavy chain mRNA, partial cds.
								AY671103	Homo sapiens clone 1ap33 immunoglobulin mu heavy chain-like mRNA, partial sequence.
								AY671108	Homo sapiens clone 1ap34 immunoglobulin mu heavy chain mRNA, partial cds.
								AY671111	Homo sapiens clone 1ap36 immunoglobulin mu heavy chain mRNA, partial cds.
								AY671112	Homo sapiens clone 1ap37 immunoglobulin mu heavy chain mRNA, partial cds.
								AY671114	Homo sapiens clone 1ap4 immunoglobulin mu heavy chain mRNA, partial cds.
								AY671115	Homo sapiens clone 1ap44 immunoglobulin mu heavy chain-like mRNA, partial sequence.
								AY671117	Homo sapiens clone 1ap45 immunoglobulin mu heavy chain-like mRNA, partial sequence.
								AY671118	Homo sapiens clone 1ap47 immunoglobulin mu heavy chain-like mRNA, partial sequence.
								AY671119	Homo sapiens clone 1ap48 immunoglobulin mu heavy chain mRNA, partial cds.
								AY671121	Homo sapiens clone 1ap5 immunoglobulin mu heavy chain mRNA, partial cds.
								AY671123	Homo sapiens clone 1ap50 immunoglobulin mu heavy chain mRNA, partial cds.
								AY671128	Homo sapiens clone 1ap53 immunoglobulin mu heavy chain mRNA, partial cds.
								AY671130	Homo sapiens clone 1ap54 immunoglobulin mu heavy chain mRNA, partial cds.
								AY671133	Homo sapiens clone 1ap55 immunoglobulin mu heavy chain mRNA, partial cds.
								AY671140	Homo sapiens clone 1ap56 immunoglobulin mu heavy chain mRNA, partial cds.
								AY671141	Homo sapiens clone 1ap6 immunoglobulin mu heavy chain mRNA, partial cds.
								AY671142	Homo sapiens clone 1ap7 immunoglobulin mu heavy chain mRNA, partial cds.
								AY671143	Homo sapiens clone 1ap9 immunoglobulin mu heavy chain mRNA, partial cds.
								AY671144	Homo sapiens clone 2ap immunoglobulin mu heavy chain mRNA, partial cds.
								AY671146	Homo sapiens clone 2ap11 immunoglobulin mu heavy chain mRNA, partial cds.

								AY671149	Homo sapiens clone 2ap13 immunoglobulin mu heavy chain mRNA, partial cds.
								AY671151	Homo sapiens clone 2ap14 immunoglobulin mu heavy chain mRNA, partial cds.
								AY671152	Homo sapiens clone 2ap22 immunoglobulin mu heavy chain mRNA, partial cds.
								AY671154	Homo sapiens clone 2ap24 immunoglobulin mu heavy chain mRNA, partial cds.
								AY671156	Homo sapiens clone 2ap26 immunoglobulin mu heavy chain mRNA, partial cds.
								AY671157	Homo sapiens clone 2ap36 immunoglobulin mu heavy chain mRNA, partial cds.
								AY671158	Homo sapiens clone 2ap38 immunoglobulin mu heavy chain mRNA, partial cds.
								AY671159	Homo sapiens clone 2ap39 immunoglobulin mu heavy chain mRNA, partial cds.
								AY671160	Homo sapiens clone 2ap41 immunoglobulin mu heavy chain mRNA, partial cds.
								AY671161	Homo sapiens clone 2ap44 immunoglobulin mu heavy chain mRNA, partial cds.
								AY671162	Homo sapiens clone 2ap49 immunoglobulin mu heavy chain mRNA, partial cds.
								AY671163	Homo sapiens clone 2ap52 immunoglobulin mu heavy chain mRNA, partial cds.
								AY671164	Homo sapiens clone 2ap60 immunoglobulin mu heavy chain mRNA, partial cds.
								AY671165	Homo sapiens clone 2CB5db1 immunoglobulin mu heavy chain mRNA, partial cds.
								AY671166	Homo sapiens clone 2CB5db10 immunoglobulin mu heavy chain mRNA, partial cds.
								AY671167	Homo sapiens clone 2CB5db4 immunoglobulin mu heavy chain mRNA, partial cds.
								AY671168	Homo sapiens clone 2CB5db6 immunoglobulin mu heavy chain mRNA, partial cds.
								AY671169	Homo sapiens clone 2CB5db7 immunoglobulin mu heavy chain-like mRNA, partial sequence.
								AY671170	Homo sapiens clone 2CB5db8 immunoglobulin mu heavy chain mRNA, partial cds.
								AY671174	Homo sapiens clone 2CB5db9 immunoglobulin mu heavy chain mRNA, partial cds.
								AY671175	Homo sapiens clone 303105+t7 immunoglobulin mu heavy chain-like mRNA, partial sequence.
								AY671176	Homo sapiens clone 303108+t7 immunoglobulin mu heavy chain mRNA, partial cds.
								AY671177	Homo sapiens clone 303113+t7 immunoglobulin mu heavy chain mRNA, partial cds.
								AY671178	Homo sapiens clone 303114+t7 immunoglobulin mu heavy chain mRNA, partial cds.
								AY671179	Homo sapiens clone 303116+t7 immunoglobulin mu heavy chain mRNA, partial cds.
								AY671180	Homo sapiens clone 303117+t7 immunoglobulin mu heavy chain mRNA, partial cds.
								AY671181	Homo sapiens clone 303119+t7 immunoglobulin mu heavy chain mRNA, partial cds.
								AY671182	Homo sapiens clone 303120+t7 immunoglobulin mu heavy chain mRNA, partial cds.
								AY671183	Homo sapiens clone 303121+t7 immunoglobulin mu heavy chain mRNA, partial cds.
								AY671184	Homo sapiens clone 303124+t7 immunoglobulin mu heavy chain mRNA, partial cds.
								AY671185	Homo sapiens clone 303125+t7 immunoglobulin mu heavy chain mRNA, partial cds.
								AY671188	Homo sapiens clone 303128+t7 immunoglobulin mu heavy chain mRNA, partial cds.
								AY671190	Homo sapiens clone 303129+t7 immunoglobulin mu heavy chain mRNA, partial cds.
								AY671191	Homo sapiens clone 303130+t7 immunoglobulin mu heavy chain mRNA, partial cds.
								AY671196	Homo sapiens clone 303131+t7 immunoglobulin mu heavy chain mRNA, partial cds.
								AY671197	Homo sapiens clone 303133+t7 immunoglobulin mu heavy chain mRNA, partial cds.
								AY671198	Homo sapiens clone 303135+t7 immunoglobulin mu heavy chain mRNA, partial cds.
								AY671199	Homo sapiens clone 303136+t7 immunoglobulin mu heavy chain mRNA, partial cds.
								AY671200	Homo sapiens clone 303137+t7 immunoglobulin mu heavy chain mRNA, partial cds.
								AY671201	Homo sapiens clone 303138+t7 immunoglobulin mu heavy chain mRNA, partial cds.
								AY671202	Homo sapiens clone 303142+t7 immunoglobulin mu heavy chain mRNA, partial cds.
								AY671203	Homo sapiens clone 303143+t7 immunoglobulin mu heavy chain mRNA, partial cds.
								AY671204	Homo sapiens clone 303146+t7 immunoglobulin mu heavy chain mRNA, partial cds.
								AY671205	Homo sapiens clone 303148+t7 immunoglobulin mu heavy chain mRNA, partial cds.
								AY671207	Homo sapiens clone 303150+t7 immunoglobulin mu heavy chain mRNA, partial cds.
								AY671208	Homo sapiens clone 303151+t7 immunoglobulin mu heavy chain mRNA, partial cds.
								AY671210	Homo sapiens clone 303152+t7 immunoglobulin mu heavy chain mRNA, partial cds.
								AY671211	Homo sapiens clone 303153+t7 immunoglobulin mu heavy chain mRNA, partial cds.
								AY671212	Homo sapiens clone 303154+t7 immunoglobulin mu heavy chain mRNA, partial cds.
								AY671213	Homo sapiens clone 303156+t7 immunoglobulin mu heavy chain mRNA, partial cds.
								AY671214	Homo sapiens clone 303158+t7 immunoglobulin mu heavy chain mRNA, partial cds.
								AY671217	Homo sapiens clone 303159+t7 immunoglobulin mu heavy chain mRNA, partial cds.
								AY671222	Homo sapiens clone 303160+t7 immunoglobulin mu heavy chain mRNA, partial cds.
								AY671223	Homo sapiens clone 303201+t7 immunoglobulin mu heavy chain mRNA, partial cds.

								AY671224	Homo sapiens clone 303202+t7 immunoglobulin mu heavy chain mRNA, partial cds.
								AY671225	Homo sapiens clone 303206+t7 immunoglobulin mu heavy chain mRNA, partial cds.
								AY671227	Homo sapiens clone 303207+t7 immunoglobulin mu heavy chain mRNA, partial cds.
								AY671228	Homo sapiens clone 303208+t7 immunoglobulin mu heavy chain-like mRNA, partial sequence.
								AY671229	Homo sapiens clone 303209+t7 immunoglobulin mu heavy chain mRNA, partial cds.
								AY671230	Homo sapiens clone 303211+t7 immunoglobulin mu heavy chain mRNA, partial cds.
								AY671232	Homo sapiens clone 303212+t7 immunoglobulin mu heavy chain mRNA, partial cds.
								AY671233	Homo sapiens clone 303213+t7 immunoglobulin mu heavy chain-like mRNA, partial sequence.
								AY671235	Homo sapiens clone 303217+t7 immunoglobulin mu heavy chain mRNA, partial cds.
								AY671236	Homo sapiens clone 303219+t7 immunoglobulin mu heavy chain mRNA, partial cds.
								AY671237	Homo sapiens clone 303220+t7 immunoglobulin mu heavy chain mRNA, partial cds.
								AY671238	Homo sapiens clone 303222+t7 immunoglobulin mu heavy chain mRNA, partial cds.
								AY671239	Homo sapiens clone 303223+t7 immunoglobulin mu heavy chain mRNA, partial cds.
								AY671240	Homo sapiens clone 303224+t7 immunoglobulin mu heavy chain mRNA, partial cds.
								AY671241	Homo sapiens clone 303225+t7 immunoglobulin mu heavy chain mRNA, partial cds.
								AY671242	Homo sapiens clone 303226+t7 immunoglobulin mu heavy chain mRNA, partial cds.
								AY671249	Homo sapiens clone 303228+t7 immunoglobulin mu heavy chain mRNA, partial cds.
								AY671252	Homo sapiens clone 303230+t7 immunoglobulin mu heavy chain mRNA, partial cds.
								AY671253	Homo sapiens clone 303231+t7 immunoglobulin mu heavy chain mRNA, partial cds.
								AY671254	Homo sapiens clone 303232+t7 immunoglobulin mu heavy chain mRNA, partial sequence.
								AY671255	Homo sapiens clone 303233+t7 immunoglobulin mu heavy chain mRNA, partial cds.
								AY671256	Homo sapiens clone 303234+t7 immunoglobulin mu heavy chain mRNA, partial cds.
								AY671258	Homo sapiens clone 303236+t7 immunoglobulin mu heavy chain mRNA, partial cds.
								AY671259	Homo sapiens clone 303237+t7 immunoglobulin mu heavy chain mRNA, partial cds.
								AY671260	Homo sapiens clone 303239+t7 immunoglobulin mu heavy chain mRNA, partial sequence.
								AY671262	Homo sapiens clone 303240+t7 immunoglobulin mu heavy chain mRNA, partial cds.
								AY671263	Homo sapiens clone 303243+t7 immunoglobulin mu heavy chain mRNA, partial cds.
								AY671264	Homo sapiens clone 303244+t7 immunoglobulin mu heavy chain mRNA, partial cds.
								AY671265	Homo sapiens clone 303251+t7 immunoglobulin mu heavy chain mRNA, partial cds.
								AY671266	Homo sapiens clone 303252+t7 immunoglobulin mu heavy chain mRNA, partial cds.
								AY671267	Homo sapiens clone 303254+t7 immunoglobulin mu heavy chain mRNA, partial cds.
								AY671268	Homo sapiens clone 303256+t7 immunoglobulin mu heavy chain mRNA, partial cds.
								AY671269	Homo sapiens clone 303258+t7 immunoglobulin mu heavy chain mRNA, partial cds.
								AY671270	Homo sapiens clone 303259+t7 immunoglobulin mu heavy chain mRNA, partial cds.
								AY671271	Homo sapiens clone 303260+t7 immunoglobulin mu heavy chain mRNA, partial cds.
								AY671272	Homo sapiens clone 303273+t7 immunoglobulin mu heavy chain mRNA, partial cds.
								AY671275	Homo sapiens clone 303277+t7 immunoglobulin mu heavy chain mRNA, partial cds.
								AY671276	Homo sapiens clone 303278+t7 immunoglobulin mu heavy chain mRNA, partial cds.
								AY671278	Homo sapiens clone 303282+t7 immunoglobulin mu heavy chain mRNA, partial cds.
								AY671279	Homo sapiens clone 303284+t7 immunoglobulin mu heavy chain mRNA, partial cds.
								AY671280	Homo sapiens clone 5d4113 immunoglobulin mu heavy chain mRNA, partial cds.
								AY671281	Homo sapiens clone 5d4114 immunoglobulin mu heavy chain mRNA, partial cds.
								AY671282	Homo sapiens clone 5d4115 immunoglobulin mu heavy chain mRNA, partial cds.
								AY671284	Homo sapiens clone 5d4117 immunoglobulin mu heavy chain mRNA, partial cds.
								AY671285	Homo sapiens clone 5d4119 immunoglobulin mu heavy chain mRNA, partial cds.
								AY671286	Homo sapiens clone 5d4129 immunoglobulin mu heavy chain mRNA, partial cds.
								AY671288	Homo sapiens clone 5d4134 immunoglobulin mu heavy chain mRNA, partial cds.
								AY671289	Homo sapiens clone 5d4136 immunoglobulin mu heavy chain mRNA, partial cds.
								AY671290	Homo sapiens clone 5d414 immunoglobulin mu heavy chain mRNA, partial cds.
								AY671291	Homo sapiens clone 5d416 immunoglobulin mu heavy chain mRNA, partial cds.
								AY671294	Homo sapiens clone 5d429 immunoglobulin mu heavy chain mRNA, partial cds.
								AY671295	Homo sapiens clone 5d430 immunoglobulin mu heavy chain mRNA, partial cds.
								AY671296	Homo sapiens clone 5d46 immunoglobulin mu heavy chain mRNA, partial cds.
								AY671297	Homo sapiens clone 7fb04 immunoglobulin mu heavy chain mRNA, partial cds.

								AY671298	Homo sapiens clone 7fb10 immunoglobulin mu heavy chain mRNA, partial cds.
								AY671300	Homo sapiens clone 7fb11 immunoglobulin mu heavy chain mRNA, partial cds.
								AY671301	Homo sapiens clone 7fb15 immunoglobulin mu heavy chain mRNA, partial cds.
								AY671302	Homo sapiens clone 7fb17 immunoglobulin mu heavy chain mRNA, partial sequence.
								AY671303	Homo sapiens clone 7fb22 immunoglobulin mu heavy chain mRNA, partial cds.
								AY671304	Homo sapiens clone 7fb26 immunoglobulin mu heavy chain mRNA, partial cds.
								AY671305	Homo sapiens clone 7fb27 immunoglobulin mu heavy chain mRNA, partial cds.
								AY671306	Homo sapiens clone 7fb29 immunoglobulin mu heavy chain mRNA, partial cds.
								AY671308	Homo sapiens clone 7fb30 immunoglobulin mu heavy chain mRNA, partial cds.
								AY671310	Homo sapiens clone 7fb32 immunoglobulin mu heavy chain mRNA, partial cds.
								AY671311	Homo sapiens clone 7fb33 immunoglobulin mu heavy chain mRNA, partial sequence.
								AY671313	Homo sapiens clone 7fb35 immunoglobulin mu heavy chain mRNA, partial cds.
								AY671316	Homo sapiens clone 7fb38 immunoglobulin mu heavy chain mRNA, partial cds.
								AY671317	Homo sapiens clone 7fb41 immunoglobulin mu heavy chain mRNA, partial cds.
								AY671318	Homo sapiens clone 7fb46 immunoglobulin mu heavy chain mRNA, partial cds.
								AY671320	Homo sapiens clone 7fb48 immunoglobulin mu heavy chain mRNA, partial sequence.
								AY671321	Homo sapiens clone 7fb52 immunoglobulin mu heavy chain mRNA, partial cds.
								AY671324	Homo sapiens clone 7fb60 immunoglobulin mu heavy chain mRNA, partial cds.
								AY671325	Homo sapiens clone 40412+T7 immunoglobulin mu heavy chain mRNA, partial cds.
								AY671327	Homo sapiens clone 40413+T7 immunoglobulin mu heavy chain mRNA, partial cds.
								AY671330	Homo sapiens clone 40414+T7 immunoglobulin mu heavy chain mRNA, partial cds.
								AY671331	Homo sapiens clone 40415+T7 immunoglobulin mu heavy chain mRNA, partial cds.
								AY671335	Homo sapiens clone 40420+t7 immunoglobulin mu heavy chain mRNA, partial cds.
								AY671336	Homo sapiens clone 404210+t7 immunoglobulin mu heavy chain mRNA, partial cds.
								AY671337	Homo sapiens clone 404215+t7 immunoglobulin mu heavy chain mRNA, partial cds.
								AY671338	Homo sapiens clone 404219+t7 immunoglobulin mu heavy chain mRNA, partial cds.
								AY671339	Homo sapiens clone 404224+t7 immunoglobulin mu heavy chain mRNA, partial cds.
								AY671340	Homo sapiens clone 404228+t7 immunoglobulin mu heavy chain mRNA, partial cds.
								AY671341	Homo sapiens clone 404232+t7 immunoglobulin mu heavy chain mRNA, partial cds.
								AY671342	Homo sapiens clone 404237+t7 immunoglobulin mu heavy chain mRNA, partial cds.
								AY671344	Homo sapiens clone 404245+t7 immunoglobulin mu heavy chain mRNA, partial cds.
								AY671345	Homo sapiens clone 404247+t7 immunoglobulin mu heavy chain mRNA, partial cds.
								AY671346	Homo sapiens clone 404252+t7 immunoglobulin mu heavy chain mRNA, partial cds.
								AY671349	Homo sapiens clone 404253+t7 immunoglobulin mu heavy chain mRNA, partial cds.
								AY671350	Homo sapiens clone 404257+t7 immunoglobulin mu heavy chain mRNA, partial cds.
								AY671351	Homo sapiens clone 404259+t7 immunoglobulin mu heavy chain mRNA, partial cds.
								AY671352	Homo sapiens clone 40426+t7 immunoglobulin mu heavy chain mRNA, partial cds.
								AY671356	Homo sapiens clone 40427+t7 immunoglobulin mu heavy chain mRNA, partial cds.
								AY671358	Homo sapiens clone 40429+t7 immunoglobulin mu heavy chain mRNA, partial cds.
								AY671359	Homo sapiens clone 4044+T7 immunoglobulin mu heavy chain mRNA, partial cds.
								AY671362	Homo sapiens clone 4045+T7 immunoglobulin mu heavy chain mRNA, partial cds.
								AY671363	Homo sapiens clone 404702+t7 immunoglobulin mu heavy chain mRNA, partial cds.
								AY671365	Homo sapiens clone 404713+t7 immunoglobulin mu heavy chain-like mRNA, partial sequence.
								AY671369	Homo sapiens clone 404717+t7 immunoglobulin mu heavy chain mRNA, partial cds.
								AY671371	Homo sapiens clone 404718+t7 immunoglobulin mu heavy chain mRNA, partial cds.
								AY671373	Homo sapiens clone 404720+t7 immunoglobulin mu heavy chain mRNA, partial cds.
								AY671375	Homo sapiens clone 404723+t7 immunoglobulin mu heavy chain mRNA, partial cds.
								AY671378	Homo sapiens clone 404724+t7 immunoglobulin mu heavy chain mRNA, partial cds.
								AY671379	Homo sapiens clone 404725+t7 immunoglobulin mu heavy chain mRNA, partial cds.
								AY671380	Homo sapiens clone 404727+t7 immunoglobulin mu heavy chain mRNA, partial cds.
								AY671381	Homo sapiens clone 404728+t7 immunoglobulin mu heavy chain-like mRNA, partial sequence.
								AY671382	Homo sapiens clone 4048+T7 immunoglobulin mu heavy chain mRNA, partial cds.
								AY671383	Homo sapiens clone 404802+t7 immunoglobulin mu heavy chain mRNA, partial cds.
								AY671384	Homo sapiens clone 404818+t7 immunoglobulin mu heavy chain-like mRNA, partial sequence.

								AY671385	Homo sapiens clone 101106+t7 immunoglobulin mu heavy chain mRNA, partial cds.
								AY671386	Homo sapiens clone 101109+t7 immunoglobulin mu heavy chain mRNA, partial cds.
								AY671388	Homo sapiens clone 101110+t7 immunoglobulin mu heavy chain mRNA, partial cds.
								AY671389	Homo sapiens clone 101121+t7 immunoglobulin mu heavy chain mRNA, partial cds.
								AY671394	Homo sapiens clone 101122+t7 immunoglobulin mu heavy chain mRNA, partial cds.
								AY671400	Homo sapiens clone 101124+t7 immunoglobulin mu heavy chain-like mRNA, partial sequence.
								AY671406	Homo sapiens clone 101126+t7 immunoglobulin mu heavy chain mRNA, partial cds.
								AY671407	Homo sapiens clone 101127+t7 immunoglobulin mu heavy chain mRNA, partial cds.
								AY671414	Homo sapiens clone 101129+t7 immunoglobulin mu heavy chain mRNA, partial cds.
								AY671416	Homo sapiens clone 101130+t7 immunoglobulin mu heavy chain mRNA, partial cds.
								AY671417	Homo sapiens clone 101131+t7 immunoglobulin mu heavy chain mRNA, partial cds.
								AY671418	Homo sapiens clone 101136+t7 immunoglobulin mu heavy chain-like mRNA, partial sequence.
								AY671420	Homo sapiens clone 101138+t7 immunoglobulin mu heavy chain mRNA, partial cds.
								AY671421	Homo sapiens clone 101142+t7 immunoglobulin mu heavy chain mRNA, partial cds.
								AY671422	Homo sapiens clone 101143+m13rev immunoglobulin mu heavy chain-like mRNA, partial sequence.
								AY671426	Homo sapiens clone 101147+t7 immunoglobulin mu heavy chain mRNA, partial cds.
								AY671428	Homo sapiens clone 101148+t7 immunoglobulin mu heavy chain mRNA, partial cds.
								AY671429	Homo sapiens clone 101149+t7 immunoglobulin mu heavy chain mRNA, partial cds.
								AY671435	Homo sapiens clone 101152+t7 immunoglobulin mu heavy chain mRNA, partial cds.
								AY671436	Homo sapiens clone 101154+t7 immunoglobulin mu heavy chain mRNA, partial cds.
								AY671437	Homo sapiens clone 101172+t7 immunoglobulin mu heavy chain mRNA, partial cds.
								AY671439	Homo sapiens clone 101184+t7 immunoglobulin mu heavy chain mRNA, partial cds.
								AY671440	Homo sapiens clone 101186+t7 immunoglobulin mu heavy chain mRNA, partial cds.
								AY671441	Homo sapiens clone 101187+t7 immunoglobulin mu heavy chain mRNA, partial cds.
								AY671442	Homo sapiens clone 101188+t7 immunoglobulin mu heavy chain-like mRNA, partial sequence.
								AY671443	Homo sapiens clone 101190+t7 immunoglobulin mu heavy chain mRNA, partial cds.
								AY671445	Homo sapiens clone 101191+t7 immunoglobulin mu heavy chain mRNA, partial cds.
								AY671448	Homo sapiens clone 101196+t7 immunoglobulin mu heavy chain mRNA, partial cds.
								AY671450	Homo sapiens clone 101198+t7 immunoglobulin mu heavy chain mRNA, partial cds.
								AY671455	Homo sapiens clone 101200+t7 immunoglobulin mu heavy chain mRNA, partial cds.
								AY671460	Homo sapiens clone 10136+t7 immunoglobulin mu heavy chain mRNA, partial cds.
								AY671463	Homo sapiens clone 10139+t7 immunoglobulin mu heavy chain mRNA, partial sequence.
								AY671464	Homo sapiens clone 10142+t7 immunoglobulin mu heavy chain mRNA, partial cds.
								AY671469	Homo sapiens clone 10148+m13rev immunoglobulin mu heavy chain mRNA, partial cds.
								AY671470	Homo sapiens clone 10151+t7 immunoglobulin mu heavy chain mRNA, partial cds.
								AY671472	Homo sapiens clone 10152+t7 immunoglobulin mu heavy chain mRNA, partial cds.
								AY671475	Homo sapiens clone 10153+t7 immunoglobulin mu heavy chain mRNA, partial cds.
								AY671476	Homo sapiens clone 10154+t7 immunoglobulin mu heavy chain mRNA, partial cds.
								AY671479	Homo sapiens clone 10155+m13rev immunoglobulin mu heavy chain mRNA, partial cds.
								AY671480	Homo sapiens clone 505117+t7 immunoglobulin mu heavy chain mRNA, partial cds.
								AY671482	Homo sapiens clone 50522+T7 immunoglobulin mu heavy chain mRNA, partial cds.
								AY671485	Homo sapiens clone 50523+T7 immunoglobulin mu heavy chain mRNA, partial cds.
								AY671486	Homo sapiens clone 50525+T7 immunoglobulin mu heavy chain mRNA, partial cds.
								AY671487	Homo sapiens clone 50527+T7 immunoglobulin mu heavy chain mRNA, partial cds.
								AY671488	Homo sapiens clone 50528+T7 immunoglobulin mu heavy chain mRNA, partial cds.
								AY671489	Homo sapiens clone 505302+t7 immunoglobulin mu heavy chain mRNA, partial cds.
								AY671491	Homo sapiens clone 505308+t7 immunoglobulin mu heavy chain mRNA, partial cds.
								AY671492	Homo sapiens clone 505309+t7 immunoglobulin mu heavy chain mRNA, partial cds.
								AY671493	Homo sapiens clone 505310+t7 immunoglobulin mu heavy chain mRNA, partial cds.
								AY671494	Homo sapiens clone 505311+t7 immunoglobulin mu heavy chain mRNA, partial cds.
								AY671495	Homo sapiens clone 505312+t7 immunoglobulin mu heavy chain mRNA, partial cds.
								AY671499	Homo sapiens clone 505314+t7 immunoglobulin mu heavy chain mRNA, partial cds.
								AY671502	Homo sapiens clone 505317+t7 immunoglobulin mu heavy chain mRNA, partial cds.
								AY671503	Homo sapiens clone 505320+t7 immunoglobulin mu heavy chain mRNA, partial cds.

								AY671504	Homo sapiens clone 505321+t7 immunoglobulin mu heavy chain mRNA, partial cds.
								AY671506	Homo sapiens clone 505322+t7 immunoglobulin mu heavy chain mRNA, partial cds.
								AY671507	Homo sapiens clone 505323+t7 immunoglobulin mu heavy chain mRNA, partial cds.
								AY671508	Homo sapiens clone 505325+t7 immunoglobulin mu heavy chain mRNA, partial cds.
								AY671511	Homo sapiens clone 505326+t7 immunoglobulin mu heavy chain mRNA, partial cds.
								AY671512	Homo sapiens clone 50534+t7 immunoglobulin mu heavy chain mRNA, partial cds.
								AY671516	Homo sapiens clone 50540+t7 immunoglobulin mu heavy chain mRNA, partial cds.
								AY671518	Homo sapiens clone 50544+t7 immunoglobulin mu heavy chain mRNA, partial cds.
								AY671519	Homo sapiens clone 50547+t7 immunoglobulin mu heavy chain-like mRNA, partial sequence.
								AY671520	Homo sapiens clone 50550+t7 immunoglobulin mu heavy chain mRNA, partial cds.
								AY671521	Homo sapiens clone 50554+t7 immunoglobulin mu heavy chain mRNA, partial cds.
								AY671522	Homo sapiens clone 50555+t7 immunoglobulin mu heavy chain mRNA, partial cds.
								AY671523	Homo sapiens clone 50557+t7 immunoglobulin mu heavy chain mRNA, partial cds.
								AY671524	Homo sapiens clone 50561+t7 immunoglobulin mu heavy chain mRNA, partial cds.
								AY671526	Homo sapiens clone 50588+t7 immunoglobulin mu heavy chain mRNA, partial cds.
								AY671529	Homo sapiens clone 20231+T7 immunoglobulin mu heavy chain-like mRNA, partial sequence.
								AY671531	Homo sapiens clone 202312+t7 immunoglobulin mu heavy chain-like mRNA, partial sequence.
								AY671532	Homo sapiens clone 20234+T7 immunoglobulin mu heavy chain mRNA, partial cds.
								AY671533	Homo sapiens clone 20235+T7 immunoglobulin mu heavy chain mRNA, partial cds.
								AY671535	Homo sapiens clone 20240+T7 immunoglobulin mu heavy chain mRNA, partial cds.
								AY671536	Homo sapiens clone 20242+t7 immunoglobulin mu heavy chain mRNA, partial cds.
								AY671537	Homo sapiens clone 20243+t7 immunoglobulin mu heavy chain mRNA, partial cds.
								AY671538	Homo sapiens clone 20245+t7 immunoglobulin mu heavy chain-like mRNA, partial sequence.
								AY671540	Homo sapiens clone 20246+t7 immunoglobulin mu heavy chain mRNA, partial cds.
								AY671541	Homo sapiens clone 20249+t7 immunoglobulin mu heavy chain mRNA, partial cds.
								AY671545	Homo sapiens clone 20252+t7 immunoglobulin mu heavy chain mRNA, partial cds.
								AY671551	Homo sapiens clone 20254+t7 immunoglobulin mu heavy chain mRNA, partial cds.
								AY671552	Homo sapiens clone 20256+t7 immunoglobulin mu heavy chain mRNA, partial cds.
								AY671553	Homo sapiens clone 20258+t7 immunoglobulin mu heavy chain mRNA, partial cds.
								AY671556	Homo sapiens clone 20260+t7 immunoglobulin mu heavy chain mRNA, partial cds.
								AY671557	Homo sapiens clone 20261+t7 immunoglobulin mu heavy chain mRNA, partial cds.
								AY671559	Homo sapiens clone 20263+t7 immunoglobulin mu heavy chain mRNA, partial cds.
								AY671563	Homo sapiens clone 20265+t7 immunoglobulin mu heavy chain mRNA, partial cds.
								AY671566	Homo sapiens clone 20267+t7 immunoglobulin mu heavy chain mRNA, partial cds.
								AY671567	Homo sapiens clone 2CB4K7 immunoglobulin mu heavy chain mRNA, partial cds.
								AY671569	Homo sapiens clone 2CB4L8 immunoglobulin mu heavy chain mRNA, partial cds.
								AY671570	Homo sapiens clone 2CB4M1 immunoglobulin mu heavy chain mRNA, partial cds.
								AY671575	Homo sapiens clone 2CB4M2 immunoglobulin mu heavy chain mRNA, partial cds.
								AY671578	Homo sapiens clone 2CB4M4 immunoglobulin mu heavy chain mRNA, partial cds.
								AY671579	Homo sapiens clone 2CB4M5 immunoglobulin mu heavy chain mRNA, partial cds.
								AY671580	Homo sapiens clone 2CB4N2 immunoglobulin mu heavy chain mRNA, partial cds.
								AY671582	Homo sapiens clone 2CB4N4 immunoglobulin mu heavy chain mRNA, partial cds.
								AY671583	Homo sapiens clone 4d4107 immunoglobulin mu heavy chain mRNA, partial cds.
								AY671584	Homo sapiens clone 4d4114 immunoglobulin mu heavy chain mRNA, partial cds.
								AY671586	Homo sapiens clone 4d4117 immunoglobulin mu heavy chain mRNA, partial cds.
								AY671589	Homo sapiens clone 4d420 immunoglobulin mu heavy chain mRNA, partial cds.
								AY671591	Homo sapiens clone 4d421 immunoglobulin mu heavy chain mRNA, partial cds.
								AY671592	Homo sapiens clone 4d428 immunoglobulin mu heavy chain mRNA, partial cds.
								AY671593	Homo sapiens clone 4d429 immunoglobulin mu heavy chain mRNA, partial cds.
								AY671595	Homo sapiens clone 4d433 immunoglobulin mu heavy chain mRNA, partial cds.
								AY671597	Homo sapiens clone 4d434 immunoglobulin mu heavy chain mRNA, partial cds.
								AY671598	Homo sapiens clone 4d436 immunoglobulin mu heavy chain mRNA, partial cds.
								AY671599	Homo sapiens clone 4d439 immunoglobulin mu heavy chain mRNA, partial cds.
								AY671602	Homo sapiens clone 4d442 immunoglobulin mu heavy chain mRNA, partial cds.

								AY671608	Homo sapiens clone 4d452 immunoglobulin mu heavy chain mRNA, partial cds.
								AY671610	Homo sapiens clone 4d456 immunoglobulin mu heavy chain mRNA, partial cds.
								AY671611	Homo sapiens clone 4d461 immunoglobulin mu heavy chain mRNA, partial cds.
								AY671613	Homo sapiens clone 4d462 immunoglobulin mu heavy chain mRNA, partial cds.
								AY671615	Homo sapiens clone 4d465 immunoglobulin mu heavy chain mRNA, partial cds.
								AY671616	Homo sapiens clone 4d466 immunoglobulin mu heavy chain mRNA, partial cds.
								AY671618	Homo sapiens clone 4d474 immunoglobulin mu heavy chain mRNA, partial cds.
								AY671620	Homo sapiens clone 4d478 immunoglobulin mu heavy chain mRNA, partial cds.
								AY671621	Homo sapiens clone 4d479 immunoglobulin mu heavy chain mRNA, partial cds.
								AY671622	Homo sapiens clone 4d485 immunoglobulin mu heavy chain mRNA, partial cds.
								AY671624	Homo sapiens clone 4d488 immunoglobulin mu heavy chain mRNA, partial cds.
								AY671625	Homo sapiens clone 4d491 immunoglobulin mu heavy chain mRNA, partial cds.
								AY671627	Homo sapiens clone 4d499 immunoglobulin mu heavy chain mRNA, partial cds.
								AY671629	Homo sapiens clone 3d4107 immunoglobulin mu heavy chain mRNA, partial cds.
								AY671630	Homo sapiens clone 3d4109 immunoglobulin mu heavy chain mRNA, partial cds.
								AY671631	Homo sapiens clone 3d4111 immunoglobulin mu heavy chain mRNA, partial cds.
								AY671635	Homo sapiens clone 3d4117 immunoglobulin mu heavy chain mRNA, partial cds.
								AY671636	Homo sapiens clone 3d4201 immunoglobulin mu heavy chain mRNA, partial cds.
								AY671637	Homo sapiens clone 3d4202 immunoglobulin mu heavy chain mRNA, partial cds.
								AY671638	Homo sapiens clone 3d4203 immunoglobulin mu heavy chain-like mRNA, partial sequence.
								AY671639	Homo sapiens clone 3d4205 immunoglobulin mu heavy chain mRNA, partial cds.
								AY671641	Homo sapiens clone 3d4209 immunoglobulin mu heavy chain mRNA, partial cds.
								AY671642	Homo sapiens clone 3d4211 immunoglobulin mu heavy chain mRNA, partial cds.
								AY671648	Homo sapiens clone 3d4215 immunoglobulin mu heavy chain-like mRNA, partial sequence.
								AY671651	Homo sapiens clone 3d4219 immunoglobulin mu heavy chain-like mRNA, partial sequence.
								AY671652	Homo sapiens clone 3d431 immunoglobulin mu heavy chain mRNA, partial cds.
								AY671653	Homo sapiens clone 3d439 immunoglobulin mu heavy chain mRNA, partial cds.
								AY671654	Homo sapiens clone 3d496 immunoglobulin mu heavy chain mRNA, partial cds.
								AY671655	Homo sapiens clone 6ap17 immunoglobulin mu heavy chain mRNA, partial cds.
								AY671656	Homo sapiens clone 6ap33 immunoglobulin mu heavy chain mRNA, partial cds.
								AY671667	Homo sapiens clone 6ap35 immunoglobulin mu heavy chain mRNA, partial cds.
								AY671668	Homo sapiens clone 6ap49 immunoglobulin mu heavy chain mRNA, partial cds.
								AY671670	Homo sapiens clone 6ap53 immunoglobulin mu heavy chain mRNA, partial cds.
								AY671672	Homo sapiens clone 6ap54 immunoglobulin mu heavy chain-like mRNA, partial sequence.
								AY671673	Homo sapiens clone 6ap57 immunoglobulin mu heavy chain mRNA, partial cds.
								AY671674	Homo sapiens clone 6ap59 immunoglobulin mu heavy chain mRNA, partial cds.
								AY671675	Homo sapiens clone 6ap8 immunoglobulin mu heavy chain mRNA, partial cds.
								AY671678	Homo sapiens clone 6ap9 immunoglobulin mu heavy chain mRNA, partial cds.
								AY671680	Homo sapiens clone 5ap16 immunoglobulin mu heavy chain mRNA, partial cds.
								AY671681	Homo sapiens clone 5ap18 immunoglobulin mu heavy chain mRNA, partial cds.
								AY671683	Homo sapiens clone 5ap19 immunoglobulin mu heavy chain mRNA, partial cds.
								AY671684	Homo sapiens clone 5ap38 immunoglobulin mu heavy chain mRNA, partial cds.
								AY671686	Homo sapiens clone 5ap39 immunoglobulin mu heavy chain mRNA, partial cds.
								AY671687	Homo sapiens clone 5ap40 immunoglobulin mu heavy chain mRNA, partial cds.
								AY671688	Homo sapiens clone 5ap46 immunoglobulin mu heavy chain mRNA, partial cds.
								AY671689	Homo sapiens clone 5ap47 immunoglobulin mu heavy chain mRNA, partial cds.
								AY671690	Homo sapiens clone 5ap49 immunoglobulin mu heavy chain mRNA, partial cds.
								AY671691	Homo sapiens clone 5ap5 immunoglobulin mu heavy chain mRNA, partial cds.
								AY671692	Homo sapiens clone 5ap50 immunoglobulin mu heavy chain mRNA, partial cds.
								AY671693	Homo sapiens clone 5ap52 immunoglobulin mu heavy chain mRNA, partial cds.
								AY671695	Homo sapiens clone 5ap56 immunoglobulin mu heavy chain mRNA, partial cds.
								AY671697	Homo sapiens clone 5ap6 immunoglobulin mu heavy chain-like mRNA, partial sequence.
								AY671699	Homo sapiens clone 1d4110 immunoglobulin mu heavy chain mRNA, partial cds.
								AY671700	Homo sapiens clone 1d422 immunoglobulin mu heavy chain mRNA, partial cds.

								AY671701	Homo sapiens clone 1d431 immunoglobulin mu heavy chain mRNA, partial cds.
								AY671703	Homo sapiens clone 1d436 immunoglobulin mu heavy chain mRNA, partial cds.
								AY671705	Homo sapiens clone 1d462 immunoglobulin mu heavy chain mRNA, partial cds.
								AY671708	Homo sapiens clone 1d47 immunoglobulin mu heavy chain mRNA, partial cds.
								AY671710	Homo sapiens clone 1d480 immunoglobulin mu heavy chain mRNA, partial cds.
								AY671713	Homo sapiens clone 2d41 immunoglobulin mu heavy chain mRNA, partial cds.
								AY671715	Homo sapiens clone 2d414 immunoglobulin mu heavy chain mRNA, partial cds.
								AY671720	Homo sapiens clone 2d422 immunoglobulin mu heavy chain mRNA, partial cds.
								AY671721	Homo sapiens clone 2d426 immunoglobulin mu heavy chain mRNA, partial cds.
								AY671722	Homo sapiens clone 2d44 immunoglobulin mu heavy chain mRNA, partial cds.
								AY671724	Homo sapiens clone 2d453 immunoglobulin mu heavy chain mRNA, partial cds.
								AY671728	Homo sapiens clone 2d461 immunoglobulin mu heavy chain mRNA, partial cds.
								AY671731	Homo sapiens clone 2d464 immunoglobulin mu heavy chain mRNA, partial cds.
								AY671733	Homo sapiens clone 2d465 immunoglobulin mu heavy chain mRNA, partial cds.
								AY671734	Homo sapiens clone 2d466 immunoglobulin mu heavy chain mRNA, partial cds.
								AY671735	Homo sapiens clone 2d469 immunoglobulin mu heavy chain mRNA, partial cds.
								AY671737	Homo sapiens clone 2d47 immunoglobulin mu heavy chain mRNA, partial cds.
								AY671738	Homo sapiens clone 2d473 immunoglobulin mu heavy chain mRNA, partial cds.
								AY671740	Homo sapiens clone 2d474 immunoglobulin mu heavy chain mRNA, partial cds.
								AY671747	Homo sapiens clone 2d475 immunoglobulin mu heavy chain mRNA, partial cds.
								AY671748	Homo sapiens clone 2d480 immunoglobulin mu heavy chain-like mRNA, partial sequence.
								AY671750	Homo sapiens clone 2d497 immunoglobulin mu heavy chain mRNA, partial cds.
								AY671751	Homo sapiens clone 2d498 immunoglobulin mu heavy chain mRNA, partial cds.
								AY671753	Homo sapiens clone 2d499 immunoglobulin mu heavy chain mRNA, partial cds.
								AY671754	Homo sapiens clone 4fb02 immunoglobulin mu heavy chain mRNA, partial cds.
								AY671758	Homo sapiens clone 4fb03 immunoglobulin mu heavy chain mRNA, partial cds.
								AY671759	Homo sapiens clone 4fb04 immunoglobulin mu heavy chain mRNA, partial cds.
								AY671761	Homo sapiens clone 4fb08 immunoglobulin mu heavy chain mRNA, partial cds.
								AY671762	Homo sapiens clone 4fb09 immunoglobulin mu heavy chain-like mRNA, partial sequence.
								AY671763	Homo sapiens clone 4fb13 immunoglobulin mu heavy chain mRNA, partial cds.
								AY671766	Homo sapiens clone 4fb15 immunoglobulin mu heavy chain mRNA, partial cds.
								AY671767	Homo sapiens clone 4fb16 immunoglobulin mu heavy chain mRNA, partial cds.
								AY671770	Homo sapiens clone 4fb17 immunoglobulin mu heavy chain mRNA, partial cds.
								AY671773	Homo sapiens clone 4fb18 immunoglobulin mu heavy chain mRNA, partial cds.
								AY671775	Homo sapiens clone 4fb19 immunoglobulin mu heavy chain mRNA, partial cds.
								AY671778	Homo sapiens clone 4fb21 immunoglobulin mu heavy chain mRNA, partial cds.
								AY671779	Homo sapiens clone 4fb22 immunoglobulin mu heavy chain mRNA, partial cds.
								AY671780	Homo sapiens clone 4fb24 immunoglobulin mu heavy chain mRNA, partial cds.
								AY671784	Homo sapiens clone 4fb27 immunoglobulin mu heavy chain mRNA, partial cds.
								AY671785	Homo sapiens clone 4fb29 immunoglobulin mu heavy chain-like mRNA, partial sequence.
								AY671786	Homo sapiens clone 4fb30 immunoglobulin mu heavy chain mRNA, partial cds.
								BC051319	Homo sapiens clone 4fb31 immunoglobulin mu heavy chain mRNA, partial cds.
								ENSESTT00000014426	Homo sapiens clone 4fb34 immunoglobulin mu heavy chain-like mRNA, partial sequence.
								ENSESTT00000016607	Homo sapiens clone 4fb35 immunoglobulin mu heavy chain mRNA, partial cds.
								ENSESTT00000031044	Homo sapiens clone 4fb36 immunoglobulin mu heavy chain mRNA, partial cds.
								ENSESTT00000031045	Homo sapiens clone 4fb37 immunoglobulin mu heavy chain mRNA, partial cds.
								ENSESTT00000031046	Homo sapiens clone 4fb39 immunoglobulin mu heavy chain mRNA, partial cds.
								ENSESTT00000031047	Homo sapiens clone 4fb40 immunoglobulin mu heavy chain mRNA, partial cds.
								ENSESTT00000031049	Homo sapiens clone 4fb44 immunoglobulin mu heavy chain mRNA, partial cds.
								ENSESTT00000031050	Homo sapiens clone 4fb50 immunoglobulin mu heavy chain mRNA, partial cds.
								ENSESTT00000031051	Homo sapiens clone 4fb51 immunoglobulin mu heavy chain mRNA, partial cds.
								ENSESTT00000031052	Homo sapiens clone 4fb52 immunoglobulin mu heavy chain mRNA, partial cds.
								ENSESTT00000031053	Homo sapiens clone 4fb55 immunoglobulin mu heavy chain mRNA, partial cds.
								ENSESTT00000031054	Homo sapiens clone 4fb56 immunoglobulin mu heavy chain mRNA, partial cds.

								ENSESTT00000031055	Homo sapiens clone 4fb58 immunoglobulin mu heavy chain mRNA, partial cds.
								ENSESTT00000031056	Homo sapiens clone 6fb07 immunoglobulin mu heavy chain mRNA, partial cds.
								ENSESTT00000049475	Homo sapiens clone 6fb31 immunoglobulin mu heavy chain mRNA, partial cds.
								ENSESTT00000049477	Homo sapiens clone 6fb43 immunoglobulin mu heavy chain-like mRNA, partial sequence.
								ENSESTT00000049478	Homo sapiens clone 6fb47 immunoglobulin mu heavy chain mRNA, partial cds.
								ENSESTT00000049480	Homo sapiens clone 6fb01 immunoglobulin mu heavy chain mRNA, partial cds.
								ENSESTT00000049481	Homo sapiens clone 6fb13 immunoglobulin mu heavy chain mRNA, partial cds.
								ENSESTT00000049483	Homo sapiens clone 6fb16 immunoglobulin mu heavy chain mRNA, partial cds.
								ENSESTT00000049486	Homo sapiens clone 6fb17 immunoglobulin mu heavy chain mRNA, partial cds.
								ENSESTT00000049487	Homo sapiens clone 6fb18 immunoglobulin mu heavy chain mRNA, partial cds.
								ENSESTT00000049488	Homo sapiens clone 6fb20 immunoglobulin mu heavy chain mRNA, partial cds.
								ENSESTT00000049490	Homo sapiens clone 6fb32 immunoglobulin mu heavy chain mRNA, partial cds.
								ENSESTT00000049492	Homo sapiens clone 6fb33 immunoglobulin mu heavy chain-like mRNA, partial sequence.
								ENSESTT00000049493	Homo sapiens clone 6fb38 immunoglobulin mu heavy chain mRNA, partial cds.
								ENSESTT00000049494	Homo sapiens clone 6fb44 immunoglobulin mu heavy chain mRNA, partial cds.
								ENSESTT00000049495	Homo sapiens clone 6fb50 immunoglobulin mu heavy chain mRNA, partial cds.
								ENSESTT00000049497	Homo sapiens clone 6fb51 immunoglobulin mu heavy chain mRNA, partial cds.
								ENSESTT00000049499	Homo sapiens clone 6fb53 immunoglobulin mu heavy chain mRNA, partial cds.
								ENSESTT00000049500	Homo sapiens clone 6fb56 immunoglobulin mu heavy chain mRNA, partial cds.
								ENSESTT00000049501	Homo sapiens clone 6fb58 immunoglobulin mu heavy chain mRNA, partial cds.
								ENSESTT00000049502	Homo sapiens clone 6fb59 immunoglobulin mu heavy chain mRNA, partial cds.
								ENSESTT00000049503	Homo sapiens clone 6fb60 immunoglobulin mu heavy chain-like mRNA, partial sequence.
								ENSESTT00000049504	Homo sapiens clone 3fb03 immunoglobulin mu heavy chain mRNA, partial cds.
								ENSESTT00000049506	Homo sapiens clone 3fb12 immunoglobulin mu heavy chain mRNA, partial cds.
								ENSESTT00000053702	Homo sapiens clone 3fb16 immunoglobulin mu heavy chain mRNA, partial cds.
								ENST00000338912	Homo sapiens clone 3fb19 immunoglobulin mu heavy chain mRNA, partial cds.
								ENST00000271105	Homo sapiens clone 3fb23 immunoglobulin mu heavy chain mRNA, partial cds.
								ENST00000372209	Homo sapiens clone 3fb25 immunoglobulin mu heavy chain mRNA, partial cds.
								ENST00000322032	Homo sapiens clone 3fb26 immunoglobulin mu heavy chain mRNA, partial cds.
								ENST00000321715	Homo sapiens clone 3fb28 immunoglobulin mu heavy chain mRNA, partial cds.
								ENST00000379913	Homo sapiens clone 3fb30 immunoglobulin mu heavy chain-like mRNA, partial sequence.
								ENST00000379910	Homo sapiens clone 3fb31 immunoglobulin mu heavy chain mRNA, partial cds.
								ENST00000379909	Homo sapiens clone 3fb32 immunoglobulin mu heavy chain mRNA, partial cds.
								ENST00000327649	Homo sapiens clone 3fb34 immunoglobulin mu heavy chain mRNA, partial cds.
								ENST00000300887	Homo sapiens clone 3fb35 immunoglobulin mu heavy chain mRNA, partial cds.
								ENST00000383048	Homo sapiens clone 3fb37 immunoglobulin mu heavy chain mRNA, partial cds.
								ENST00000337025	Homo sapiens clone 3fb40 immunoglobulin mu heavy chain mRNA, partial cds.
								ENST00000251006	Homo sapiens clone 3fb41 immunoglobulin mu heavy chain mRNA, partial cds.
								ENST00000383047	Homo sapiens clone 3fb42 immunoglobulin mu heavy chain mRNA, partial cds.
								ENST00000361266	Homo sapiens clone 3fb48 immunoglobulin mu heavy chain mRNA, partial cds.
								ENST00000358917	Homo sapiens clone 3fb49 immunoglobulin mu heavy chain mRNA, partial cds.
								ENST00000216649	Homo sapiens clone 3fb50 immunoglobulin mu heavy chain mRNA, partial cds.
								ENST00000312636	Homo sapiens clone 3fb52 immunoglobulin mu heavy chain mRNA, partial cds.
								ENST00000359196	Homo sapiens clone 3fb53 immunoglobulin mu heavy chain mRNA, partial cds.
								ENST00000356012	Homo sapiens clone 3fb55 immunoglobulin mu heavy chain mRNA, partial cds.
								ENST00000379900	Homo sapiens clone 3fb57 immunoglobulin mu heavy chain-like mRNA, partial sequence.
								ENST00000379894	Homo sapiens clone 5fb46 immunoglobulin mu heavy chain mRNA, partial sequence.
								ENST00000379884	Homo sapiens clone 5fb04 immunoglobulin mu heavy chain mRNA, partial sequence.
								ENST00000379877	Homo sapiens clone 5fb05 immunoglobulin mu heavy chain mRNA, partial sequence.
								ENST00000343496	Homo sapiens clone 5fb07 immunoglobulin mu heavy chain mRNA, partial cds.
								ENST00000356161	Homo sapiens clone 5fb09 immunoglobulin mu heavy chain mRNA, partial sequence.
								ENST00000329794	Homo sapiens clone 5fb10 immunoglobulin mu heavy chain mRNA, partial sequence.
								ENST00000389889	Homo sapiens clone 5fb22 immunoglobulin mu heavy chain mRNA, partial sequence.
								ENST00000332874	Homo sapiens clone 5fb37 immunoglobulin mu heavy chain mRNA, partial cds.

								ENST00000283694	Homo sapiens clone 5fb38 immunoglobulin mu heavy chain mRNA, partial sequence.
								ENST00000338567	Homo sapiens clone 5fb42 immunoglobulin mu heavy chain mRNA, partial sequence.
								ENST00000354689	Homo sapiens clone 5fb45 immunoglobulin mu heavy chain mRNA, partial cds.
								ENST00000379905	Homo sapiens clone 5fb47 immunoglobulin mu heavy chain mRNA, partial sequence.
								ENST00000379903	Homo sapiens clone 5fb48 immunoglobulin mu heavy chain mRNA, partial cds.
								ENST00000379901	Homo sapiens clone 5fb49 immunoglobulin mu heavy chain mRNA, partial sequence.
								ENST00000361286	Homo sapiens clone 5fb53 immunoglobulin mu heavy chain mRNA, partial cds.
								ENST00000379897	Homo sapiens clone 5fb56 immunoglobulin mu heavy chain mRNA, partial cds.
								ENST00000379895	Homo sapiens clone 5fb57 immunoglobulin mu heavy chain-like mRNA, partial sequence.
								ENST00000358800	Homo sapiens clone 5fb59 immunoglobulin mu heavy chain mRNA, partial cds.
								ENST00000358811	Homo sapiens clone 5fb60 immunoglobulin mu heavy chain mRNA, partial cds.
								ENST00000379891	Homo sapiens clone 5fb63 immunoglobulin mu heavy chain-like mRNA, partial sequence.
								ENST00000379886	Homo sapiens clone 5fb64 immunoglobulin mu heavy chain mRNA, partial cds.
								ENST00000357815	Homo sapiens clone 5fb65 immunoglobulin mu heavy chain-like mRNA, partial sequence.
								ENST00000315216	Homo sapiens clone 5fb67 immunoglobulin mu heavy chain mRNA, partial cds.
								ENST00000379880	Homo sapiens clone 5fb68 immunoglobulin mu heavy chain mRNA, partial cds.
								ENST00000379879	Homo sapiens clone 5fb69 immunoglobulin mu heavy chain mRNA, partial cds.
								ENST00000379875	Homo sapiens clone 5fb70 immunoglobulin mu heavy chain mRNA, partial cds.
								ENST00000359714	Homo sapiens clone pCmpA1 immunoglobulin mu heavy chain mRNA, partial cds.
								ENST00000360623	Homo sapiens clone PCMP-a2 immunoglobulin mu heavy chain mRNA, partial cds.
								ENST00000379872	Homo sapiens clone PCMP-a3 immunoglobulin mu heavy chain mRNA, partial cds.
								ENST00000360592	Homo sapiens clone PCMP-a4 immunoglobulin mu heavy chain mRNA, partial cds.
								ENST00000355807	Homo sapiens clone PCMP-a6 immunoglobulin mu heavy chain-like mRNA, partial sequence.
								ENST00000302092	Homo sapiens clone PCMP-a7 immunoglobulin mu heavy chain-like mRNA, partial sequence.
								GENSCAN00000042049	Homo sapiens clone pCmpB2 immunoglobulin mu heavy chain mRNA, partial cds.
								GENSCAN00000042050	Homo sapiens clone pCmpB3 immunoglobulin mu heavy chain mRNA, partial cds.
								GENSCAN00000013558	Homo sapiens clone PCMP-b6 immunoglobulin mu heavy chain-like mRNA, partial sequence.
								GENSCAN00000013563	Homo sapiens clone PCMP-c1 immunoglobulin mu heavy chain mRNA, partial cds.
								GENSCAN00000013559	Homo sapiens clone pCmpC3 immunoglobulin mu heavy chain mRNA, partial cds.
								GENSCAN00000030621	Homo sapiens clone PCMP-c4 immunoglobulin mu heavy chain mRNA, partial cds.
								GENSCAN00000048487	Homo sapiens clone pCmpD3 immunoglobulin mu heavy chain mRNA, partial cds.
								GENSCAN00000025465	Homo sapiens clone pCmpD4 immunoglobulin mu heavy chain mRNA, partial cds.
								GENSCAN00000017885	Homo sapiens clone pCmpE1 immunoglobulin mu heavy chain mRNA, partial cds.
								GENSCAN00000034509	Homo sapiens clone pCmpF1 immunoglobulin mu heavy chain mRNA, partial cds.
								GENSCAN00000016664	Homo sapiens clone pCmpH3 immunoglobulin mu heavy chain-like mRNA, partial sequence.
								GENSCAN00000016663	Homo sapiens clone PCMN-a6 immunoglobulin mu heavy chain mRNA, partial cds.
								GENSCAN00000059304	Homo sapiens clone PCMN-b1 immunoglobulin mu heavy chain mRNA, partial cds.
								GENSCAN00000059309	Homo sapiens clone PCMN-b2 immunoglobulin mu heavy chain mRNA, partial cds.
								GENSCAN00000059307	Homo sapiens clone PCMN-b3 immunoglobulin mu heavy chain mRNA, partial cds.
								GENSCAN00000059306	Homo sapiens clone PCMN-b5 immunoglobulin mu heavy chain mRNA, partial cds.
								GENSCAN00000059310	Homo sapiens clone PCMN-b6 immunoglobulin mu heavy chain-like mRNA, partial sequence.
								GENSCAN00000059300	Homo sapiens clone PCMN-c2 immunoglobulin mu heavy chain mRNA, partial cds.
								GENSCAN00000006017	Homo sapiens clone PCMN-d2 immunoglobulin mu heavy chain mRNA, partial cds.
								GENSCAN00000006020	Homo sapiens clone PCMN-d3 immunoglobulin mu heavy chain mRNA, partial cds.
								GENSCAN00000006018	Homo sapiens clone PCMN-d5 immunoglobulin mu heavy chain mRNA, partial cds.
								GENSCAN00000006016	Homo sapiens clone PCMN-d6 immunoglobulin mu heavy chain mRNA, partial cds.
								GENSCAN00000006021	Homo sapiens clone PCMN-d8 immunoglobulin mu heavy chain mRNA, partial cds.
								GENSCAN00000006015	Homo sapiens clone PCMN-e1 immunoglobulin mu heavy chain mRNA, partial cds.
								GENSCAN00000006019	Homo sapiens clone pCmnE5 immunoglobulin mu heavy chain mRNA, partial cds.
								GENSCAN00000006014	Homo sapiens clone PCMN-e6 immunoglobulin mu heavy chain mRNA, partial cds.
								GENSCAN00000040297	Homo sapiens clone PCMN-e7 immunoglobulin mu heavy chain mRNA, partial cds.
								GENSCAN00000040303	Homo sapiens clone pCmnE8 immunoglobulin mu heavy chain mRNA, partial cds.
								GENSCAN00000040301	Homo sapiens clone PCMN-e8 immunoglobulin mu heavy chain mRNA, partial cds.
								GENSCAN00000040299	Homo sapiens clone PCMN-f4 immunoglobulin mu heavy chain mRNA, partial cds.

								GENSCAN00000040304<br>GENSCAN00000051369<br>GENSCAN00000051376<br>GENSCAN00000051371<br>GENSCAN00000051377<br>GENSCAN00000051368<br>GENSCAN00000029220<br>GENSCAN00000029205<br>GENSCAN00000029221<br>GENSCAN00000029200<br>GENSCAN00000029214<br>GENSCAN00000064300<br>GENSCAN00000064310<br>GENSCAN00000064316<br>GENSCAN00000064299<br>GENSCAN00000017144<br>GENSCAN00000066981<br>GENSCAN00000066982<br>GENSCAN00000049235	Homo sapiens clone PCMN-f5 immunoglobulin mu heavy chain mRNA, partial cds.<br>Homo sapiens clone pCmnQTS12 immunoglobulin mu heavy chain mRNA, partial cds.<br>Homo sapiens clone pCmnQTS17 immunoglobulin mu heavy chain mRNA, partial cds.<br>Homo sapiens clone pCmnQTS4 immunoglobulin mu heavy chain mRNA, partial cds.<br>Homo sapiens clone pCmnZ1 immunoglobulin mu heavy chain mRNA, partial cds.<br>Homo sapiens clone pCmnZ10 immunoglobulin mu heavy chain mRNA, partial cds.<br>Homo sapiens clone pCmnZ2 immunoglobulin mu heavy chain mRNA, partial cds.<br>Homo sapiens clone pCmnZ6 immunoglobulin mu heavy chain mRNA, partial cds.<br>Homo sapiens clone pCmnZ8 immunoglobulin mu heavy chain mRNA, partial cds.<br>Homo sapiens clone pCmnZ9 immunoglobulin mu heavy chain mRNA, partial cds.<br>Homo sapiens cDNA clone IMAGE:6215762, **** WARNING: chimeric clone ****.<br>cdna:known chromosome:NCBI36:15:18429923:18430368:-1 gene:ENSG00000188403<br>cdna:known chromosome:NCBI36:1:45013826:45016996:1 gene:ENSG00000142937<br>cdna:known chromosome:NCBI36:1:45013832:45017038:1 gene:ENSG00000142937<br>cdna:known chromosome:NCBI36:14:105124275:105125777:-1 gene:ENSG00000177199<br>cdna:known chromosome:NCBI36:14:105137448:105139110:-1 gene:ENSG00000177154<br>cdna:known chromosome:NCBI36:14:105161725:105163451:-1 gene:ENSG00000205402

									cdna:known chromosome:NCBI36:14:105180585:105182175:-1 gene:ENSG00000205401<br>cdna:known chromosome:NCBI36:14:105191187:105191732:-1 gene:ENSG00000205400<br>cdna:known chromosome:NCBI36:14:105205503:105210208:-1 gene:ENSG00000184981<br>cdna:known chromosome:NCBI36:14:105244506:105589977:-1 gene:ENSG00000130076<br>cdna:known chromosome:NCBI36:14:105273726:105796730:-1 gene:ENSG00000130076<br>cdna:known chromosome:NCBI36:14:105301886:105310425:-1 gene:ENSG00000130076<br>cdna:known chromosome:NCBI36:14:105306486:105589977:-1 gene:ENSG00000130076<br>cdna:known chromosome:NCBI36:14:105306486:105589977:-1 gene:ENSG00000130076<br>cdna:known chromosome:NCBI36:14:105306493:105524151:-1 gene:ENSG00000130076<br>cdna:known chromosome:NCBI36:14:105389082:105589898:-1 gene:ENSG00000130076<br>cdna:known chromosome:NCBI36:14:105389085:105549605:-1 gene:ENSG00000130076<br>cdna:known chromosome:NCBI36:14:105391367:105589898:-1 gene:ENSG00000130076<br>cdna:known chromosome:NCBI36:14:105391369:105589898:-1 gene:ENSG00000130076<br>cdna:known chromosome:NCBI36:14:105828698:105828988:-1 gene:ENSG00000196662<br>cdna:known chromosome:NCBI36:14:105862047:105862343:-1 gene:ENSG00000205394<br>cdna:known chromosome:NCBI36:14:105900621:105901069:-1 gene:ENSG00000205391<br>cdna:known chromosome:NCBI36:14:106038091:106038530:-1 gene:ENSG00000205385<br>cdna:known chromosome:NCBI36:14:106154116:106154770:-1 gene:ENSG00000205381<br>cdna:known chromosome:NCBI36:14:106240720:106241470:-1 gene:ENSG00000205377<br>cdna:novel chromosome:NCBI36:15:18452925:18453224:-1 gene:ENSG00000198465<br>cdna:novel chromosome:NCBI36:15:18512602:18513585:-1 gene:ENSG00000184177<br>cdna:novel chromosome:NCBI36:15:19522870:19524984:-1 gene:ENSG00000185520<br>cdna:novel chromosome:NCBI36:15:19524031:19525014:-1 gene:ENSG00000185520<br>cdna:novel chromosome:NCBI36:15:19949736:19950183:-1 gene:ENSG00000153613<br>cdna:novel chromosome:NCBI36:15:19974101:19974756:-1 gene:ENSG00000189039<br>cdna:novel chromosome:NCBI36:16:33648305:33660388:-1 gene:ENSG00000198229<br>cdna:novel chromosome:NCBI36:14:105712596:105713042:-1 gene:ENSG00000205398<br>cdna:novel chromosome:NCBI36:14:105804192:105804624:-1 gene:ENSG00000205397<br>cdna:novel chromosome:NCBI36:14:105851274:105852029:-1 gene:ENSG00000205395<br>cdna:novel chromosome:NCBI36:14:105875944:105876728:-1 gene:ENSG00000205393<br>cdna:novel chromosome:NCBI36:14:105876124:105876689:-1 gene:ENSG00000205393<br>cdna:novel chromosome:NCBI36:14:105886764:105887060:-1 gene:ENSG00000205392<br>cdna:novel chromosome:NCBI36:14:105916362:105916661:-1 gene:ENSG00000205390<br>cdna:novel chromosome:NCBI36:14:105937454:105937901:-1 gene:ENSG00000205389<br>cdna:novel chromosome:NCBI36:14:105948664:105949137:-1 gene:ENSG00000205388<br>cdna:novel chromosome:NCBI36:14:106033964:106034411:-1 gene:ENSG00000205386<br>cdna:novel chromosome:NCBI36:14:106045553:106065152:-1 gene:ENSG00000198577<br>cdna:novel chromosome:NCBI36:14:106105753:106106085:-1 gene:ENSG00000205384<br>cdna:novel chromosome:NCBI36:14:106113413:106113673:-1 gene:ENSG00000205383<br>cdna:novel chromosome:NCBI36:14:106119714:106120007:-1 gene:ENSG00000205382<br>cdna:novel chromosome:NCBI36:14:106165986:106166645:-1 gene:ENSG00000205380<br>cdna:novel chromosome:NCBI36:14:106184786:106185239:-1 gene:ENSG00000205379<br>cdna:novel chromosome:NCBI36:14:106202078:106202526:-1 gene:ENSG00000205378<br>cdna:novel chromosome:NCBI36:14:106249865:106250307:-1 gene:ENSG00000205376<br>cdna:novel chromosome:NCBI36:14:106269977:106282276:-1 gene:ENSG00000198378<br>cdna:novel chromosome:NCBI36:14:106289721:106290014:-1 gene:ENSG00000197655<br>cdna:novel chromosome:NCBI36:21:9884493:9884938:1 gene:ENSG00000169861<br>cdna:Genscan chromosome:NCBI36:15:18511396:18513585:-1<br>cdna:Genscan chromosome:NCBI36:15:18438039:18438485:-1<br>cdna:Genscan chromosome:NCBI36:15:19962527:19969486:-1<br>cdna:Genscan chromosome:NCBI36:15:19949736:19950183:-1<br>cdna:Genscan chromosome:NCBI36:15:19941659:19942210:-1<br>cdna:Genscan chromosome:NCBI36:15:19522825:19525014:-1<br>cdna:Genscan chromosome:NCBI36:16:33518707:33520855:1

									cdna:Genscan chromosome:NCBI36:16:32766535:32767076:1<br>cdna:Genscan chromosome:NCBI36:16:31870854:31875458:1<br>cdna:Genscan chromosome:NCBI36:1:45013856:45016944:1<br>cdna:Genscan chromosome:NCBI36:14:106190939:106307157:-1<br>cdna:Genscan chromosome:NCBI36:14:106166155:106188232:-1<br>cdna:Genscan chromosome:NCBI36:14:106149406:106159273:-1<br>cdna:Genscan chromosome:NCBI36:14:106136783:106137251:-1<br>cdna:Genscan chromosome:NCBI36:14:106110787:106133627:-1<br>cdna:Genscan chromosome:NCBI36:14:106038082:106106207:-1<br>cdna:Genscan chromosome:NCBI36:14:106033964:106034411:-1<br>cdna:Genscan chromosome:NCBI36:14:105987384:106016822:-1<br>cdna:Genscan chromosome:NCBI36:14:105904233:105949121:-1<br>cdna:Genscan chromosome:NCBI36:14:105899469:105901090:-1<br>cdna:Genscan chromosome:NCBI36:14:105883315:105896433:-1<br>cdna:Genscan chromosome:NCBI36:14:105851217:105876689:-1<br>cdna:Genscan chromosome:NCBI36:14:105845053:105845544:-1<br>cdna:Genscan chromosome:NCBI36:14:105828515:105829137:-1<br>cdna:Genscan chromosome:NCBI36:14:105804177:105815298:-1<br>cdna:Genscan chromosome:NCBI36:14:105762712:105798956:-1<br>cdna:Genscan chromosome:NCBI36:14:105707515:105739210:-1<br>cdna:Genscan chromosome:NCBI36:14:105656280:105703502:-1<br>cdna:Genscan chromosome:NCBI36:14:105623321:105650348:-1<br>cdna:Genscan chromosome:NCBI36:14:105572463:105610978:-1<br>cdna:Genscan chromosome:NCBI36:14:105565081:105565622:-1<br>cdna:Genscan chromosome:NCBI36:14:105544137:105549605:-1<br>cdna:Genscan chromosome:NCBI36:14:105537290:105542726:-1<br>cdna:Genscan chromosome:NCBI36:14:105523704:105524151:-1<br>cdna:Genscan chromosome:NCBI36:14:105507089:105508921:-1<br>cdna:Genscan chromosome:NCBI36:14:105454374:105482324:-1<br>cdna:Genscan chromosome:NCBI36:14:105275144:105284872:-1<br>cdna:Genscan chromosome:NCBI36:14:105303296:105323213:-1<br>cdna:Genscan chromosome:NCBI36:14:105354978:105355526:-1<br>cdna:Genscan chromosome:NCBI36:14:105374358:105390137:-1<br>cdna:Genscan chromosome:NCBI36:14:105391493:105432642:-1<br>cdna:Genscan chromosome:NCBI36:14:105178347:105182529:-1<br>cdna:Genscan chromosome:NCBI36:14:105159294:105175324:-1<br>cdna:Genscan chromosome:NCBI36:14:105124319:105156078:-1<br>cdna:Genscan chromosome:NCBI36:14:105121536:105122443:-1<br>cdna:Genscan chromosome:NCBI36:14:105202008:105206374:-1<br>cdna:Genscan chromosome:NCBI36:14:105244550:105245428:-1<br>cdna:Genscan chromosome:NCBI36:14:105241770:105242669:-1<br>cdna:Genscan chromosome:NCBI36:21:9884493:9884938:1
3590014	792270<br>792271<br>792272<br>792273<br>792274<br>792275<br>792276<br>792277<br>792278<br>792279<br>792280<br>792281	3590015<br>3590016<br>3590017<br>3590018<br>3590019<br>3590020<br>3590021<br>3590022<br>3590023<br>3590024<br>3590025<br>3590026	core<br>core<br>core<br>core<br>extended<br>extended<br>extended<br>core<br>extended<br>core<br>core<br>core	15	38673510	38743829	+	NM_144508<br>NM_170589<br>AF173994<br>AF248041<br>ENSESTT00000034799<br>ENSESTT00000034800<br>ENSESTT00000034801<br>ENST00000346991<br>ENST00000260369<br>GENSCAN00000007069<br>GENSCAN00000007068	Homo sapiens cancer susceptibility candidate 5 (CASC5), transcript variant 2, mRNA.<br>Homo sapiens cancer susceptibility candidate 5 (CASC5), transcript variant 1, mRNA.<br>Homo sapiens AF15q14 mRNA, complete cds.<br>Homo sapiens AF15q14 protein mRNA, complete cds.<br><br><br><br>cdna:known chromosome:NCBI36:15:38673755:38734467:1 gene:ENSG00000137812<br>cdna:known chromosome:NCBI36:15:38673755:38734467:1 gene:ENSG00000137812<br>cdna:Genscan chromosome:NCBI36:15:38682422:38714510:1<br>cdna:Genscan chromosome:NCBI36:15:38725197:38741782:1

	792282<br>792283<br>792284<br>792285<br>792286<br>792287<br>792288<br>792289<br>792290<br>792291<br>792292<br>792293<br>792294<br>792295<br>792296<br>792297<br>792298<br>792299<br>792300<br>792301<br>792302<br>792303<br>792304<br>792305<br>792306<br>792307	3590027<br>3590028<br>3590029<br>3590030<br>3590031<br>3590032<br>3590033<br>3590034<br>3590035<br>3590036<br>3590037<br>3590038<br>3590039<br>3590040<br>3590041<br>3590042<br>3590043<br>3590044<br>3590045<br>3590046<br>3590047<br>3590048<br>3590049<br>3590050<br>3590051<br>3590052<br>3590053<br>3590054<br>3590055<br>3590056<br>3590057<br>3590058<br>3590059<br>3590060<br>3590061<br>3590062<br>3590063<br>3590064<br>3590065<br>3590066<br>3590067<br>3590068<br>3590069<br>3590070<br>3590071<br>3590072<br>3590073<br>3590074<br>3590075<br>3590076<br>3590077	core<br>core<br>core<br>core<br>extended<br>extended<br>core<br>core<br>core<br>core<br>core<br>core<br>core<br>core<br>core<br>core<br>core<br>core<br>core<br>core<br>core<br>core<br>full<br>core<br>core<br>full<br>extended<br>core<br>core<br>core<br>core<br>extended<br>core<br>core<br>extended<br>core<br>core<br>core<br>core<br>extended<br>core<br>core<br>extended<br>core<br>core<br>core<br>core<br>core<br>extended<br>extended<br>extended						
3590388	792495<br>792496<br>792497	3590389<br>3590390<br>3590391	extended<br>full<br>core	15	39392386	39460532	+	NM_016359<br>NM_018454<br>CR591711	Homo sapiens nucleolar and spindle associated protein 1 (NUSAP1), transcript variant 1, mRNA.<br>Homo sapiens nucleolar and spindle associated protein 1 (NUSAP1), transcript variant 2, mRNA.<br>full-length cDNA clone CS0DL011YO18 of B cells (Ramos cell line) Cot 25-normalized of Homo sapiens (human).

	792498<br>792499<br>792500<br>792501<br>792502<br>792503<br>792504<br>792505<br>792506<br>792507<br>792508	3590392<br>3590393<br>3590394<br>3590395<br>3590396<br>3590397<br>3590398<br>3590399<br>3590400<br>3590401<br>3590402<br>3590403<br>3590404<br>3590405<br>3590406<br>3590407<br>3590408	core<br>core<br>core<br>extended<br>core<br>core<br>core<br>core<br>core<br>core<br>core<br>core<br>core<br>core<br>core<br>core<br>core					BC011008<br>ENSESTT00000041086<br>ENSESTT00000041087<br>ENSESTT00000041088<br>ENST00000260359<br>GENSCAN00000014732	Homo sapiens nucleolar and spindle associated protein 1, mRNA (cDNA clone IMAGE:4068708), with apparent retained intron.<br><br><br><br>cdna:known chromosome:NCBI36:15:39412361:39460535:1 gene:ENSG00000137804<br>cdna:Genscan chromosome:NCBI36:15:39411646:39459676:1
3592755	793950<br>793951<br>793952<br>793953<br>793954<br>793955<br>793956<br>793957<br>793958<br>793959<br>793960<br>793961<br>793962<br>793963<br>793964<br>793965<br>793966<br>793967<br>793968<br>793969<br>793970<br>793971<br>793972<br>793973<br>793974<br>793975<br>793976<br>793977<br>793978<br>793979<br>793980<br>793981<br>793982<br>793983<br>793984<br>793985<br>793986	3592750<br>3592752<br>3592754<br>3592756<br>3592757<br>3592758<br>3592759<br>3592760<br>3592761<br>3592762<br>3592763<br>3592764<br>3592765<br>3592766<br>3592767<br>3592768<br>3592769<br>3592770<br>3592771<br>3592772<br>3592773<br>3592774<br>3592775<br>3592776<br>3592777<br>3592778<br>3592779<br>3592780<br>3592781<br>3592782<br>3592783<br>3592784<br>3592785<br>3592786<br>3592787<br>3592788<br>3592789	extended<br>free<br>free<br>full<br>extended<br>full<br>extended<br>extended<br>extended<br>extended<br>extended<br>full<br>full<br>full<br>full<br>extended<br>extended<br>full<br>full<br>full<br>full<br>full<br>full<br>full<br>full<br>full<br>full<br>extended<br>extended<br>extended<br>full<br>full<br>full<br>extended<br>full<br>full<br>full	15	45263585	45853709	+	NM_153616<br>NM_024966<br>NM_153617<br>NM_153618<br>XM_944130<br>NM_153619<br>NM_020858<br>XM_932857<br>AB040912<br>AB073902<br>CR596993<br>ENSESTT00000047054<br>ENSESTT00000047055<br>ENSESTT00000047056<br>ENSESTT00000047057<br>ENSESTT00000047058<br>ENSESTT00000047059<br>ENST00000358066<br>ENST00000389434<br>ENST00000389433<br>ENST00000389432<br>ENST00000389431<br>ENST00000389430<br>ENST00000389429<br>ENST00000389428<br>ENST00000389427<br>ENST00000389426<br>ENST00000389425<br>ENST00000389424<br>ENST00000316364<br>ENST00000354744<br>ENST00000355997<br>GENSCAN00000060984<br>GENSCAN00000052700	Homo sapiens sema domain, transmembrane domain (TM), and cytoplasmic domain, (semaphorin) 6D (SEMA6D), transcript variant 2, mRNA.<br>Homo sapiens sema domain, transmembrane domain (TM), and cytoplasmic domain, (semaphorin) 6D (SEMA6D), transcript variant 6, mRNA.<br>Homo sapiens sema domain, transmembrane domain (TM), and cytoplasmic domain, (semaphorin) 6D (SEMA6D), transcript variant 3, mRNA.<br>Homo sapiens sema domain, transmembrane domain (TM), and cytoplasmic domain, (semaphorin) 6D (SEMA6D), transcript variant 4, mRNA.<br>PREDICTED: Homo sapiens similar to sema domain, transmembrane domain (TM), and cytoplasmic domain, (semaphorin) 6D (LOC654102), mRNA.<br>Homo sapiens sema domain, transmembrane domain (TM), and cytoplasmic domain, (semaphorin) 6D (SEMA6D), transcript variant 5, mRNA.<br>Homo sapiens sema domain, transmembrane domain (TM), and cytoplasmic domain, (semaphorin) 6D (SEMA6D), transcript variant 1, mRNA.<br>PREDICTED: Homo sapiens similar to sema domain, transmembrane domain (TM), and cytoplasmic domain, (semaphorin) 6D (LOC653614), mRNA.<br>Homo sapiens mRNA for KIAA1479 protein, partial cds.<br>Homo sapiens primary neuroblastoma cDNA, clone:Nbla03862, full insert sequence.<br>full-length cDNA clone CS0DI027YO21 of Placenta Cot 25-normalized of Homo sapiens (human).<br><br><br><br><br><br><br><br>cdna:known chromosome:NCBI36:15:45797978:45853709:1 gene:ENSG00000137872<br>cdna:known chromosome:NCBI36:15:45839288:45851274:1 gene:ENSG00000137872<br>cdna:known chromosome:NCBI36:15:45839288:45851274:1 gene:ENSG00000137872<br>cdna:known chromosome:NCBI36:15:45839288:45851274:1 gene:ENSG00000137872<br>cdna:known chromosome:NCBI36:15:45839288:45851274:1 gene:ENSG00000137872<br>cdna:known chromosome:NCBI36:15:45839288:45851274:1 gene:ENSG00000137872<br>cdna:known chromosome:NCBI36:15:45839288:45851274:1 gene:ENSG00000137872<br>cdna:known chromosome:NCBI36:15:45839288:45851274:1 gene:ENSG00000137872<br>cdna:known chromosome:NCBI36:15:45839288:45851274:1 gene:ENSG00000137872<br>cdna:known chromosome:NCBI36:15:45839288:45850036:1 gene:ENSG00000137872<br>cdna:known chromosome:NCBI36:15:45839288:45844549:1 gene:ENSG00000137872<br>cdna:known chromosome:NCBI36:15:45839288:45844549:1 gene:ENSG00000137872

	793987	3592790	full						cdna:novel chromosome:NCBI36:15:45797978:45853709:1 gene:ENSG00000137872
	793988	3592791	full						cdna:novel chromosome:NCBI36:15:45797978:45853709:1 gene:ENSG00000137872
	793989	3592792	full						cdna:novel chromosome:NCBI36:15:45797978:45853709:1 gene:ENSG00000137872
	793990	3592793	full						cdna:Genscan chromosome:NCBI36:15:45839288:45851274:1
	793991	3592794	full						cdna:Genscan chromosome:NCBI36:15:45794634:45798201:1
	793992	3592795	full						
	793993	3592796	full						
	793994	3592797	full						
	793995	3592798	full						
	793996	3592799	full						
	793997	3592800	full						
	793998	3592801	full						
	793999	3592802	full						
	794000	3592803	extended						
	794001	3592804	extended						
	794002	3592805	full						
	794003	3592806	full						
	794004	3592807	full						
	794005	3592808	full						
	794006	3592809	full						
	794007	3592810	full						
	794008	3592811	full						
	794009	3592812	full						
	794010	3592813	full						
	794011	3592814	full						
	794012	3592815	full						
	794013	3592816	full						
	794014	3592817	full						
	794015	3592818	full						
	794016	3592819	extended						
	794017	3592820	full						
	794018	3592821	full						
	794019	3592822	full						
	794020	3592823	full						
	794021	3592824	extended						
	794022	3592825	full						
	794023	3592826	full						
	794024	3592827	full						
	794025	3592828	full						
	794026	3592829	extended						
	794027	3592830	extended						
	794028	3592831	full						
	794029	3592832	full						
	794030	3592833	full						
	794031	3592834	full						
	794032	3592835	full						
	794033	3592836	full						
	794034	3592837	full						
	794035	3592838	full						
	794036	3592839	full						
	794037	3592840	full						
	794038	3592841	core						
	794039	3592842	core						
	794040	3592843	core						

	794041	3592844<br>3592845<br>3592846<br>3592847<br>3592848<br>3592849<br>3592850<br>3592851<br>3592852<br>3592853<br>3592854<br>3592855<br>3592856<br>3592857<br>3592858<br>3592859<br>3592860<br>3592861<br>3592862<br>3592863<br>3592864<br>3592865<br>3592866<br>3592867<br>3592868<br>3592869<br>3592870<br>3592871<br>3592872<br>3592873<br>3592874<br>3592875<br>3592876<br>3592877<br>3592878<br>3592879<br>3592880	full<br>full<br>core<br>core<br>core<br>core<br>core<br>core<br>core<br>core<br>full<br>full<br>core<br>full<br>core<br>core<br>core<br>core<br>core<br>core<br>core<br>full<br>core<br>core<br>core<br>full<br>core<br>full<br>full<br>core<br>core<br>full<br>full<br>full<br>core<br>core<br>core						
3595979	795970<br>795971<br>795972<br>795973<br>795974<br>795975<br>795976<br>795977<br>795978<br>795979<br>795980<br>795981	3595980<br>3595981<br>3595982<br>3595983<br>3595984<br>3595985<br>3595986<br>3595987<br>3595988<br>3595989<br>3595990<br>3595991<br>3595992<br>3595993<br>3595994<br>3595995<br>3595996	full<br>core<br>full<br>core<br>core<br>extended<br>core<br>full<br>core<br>core<br>core<br>core<br>core<br>full<br>core<br>extended<br>core	15	57184508	57204566	+	NM_004701<br>ENSESTT00000027806<br>ENST00000288207<br>GENSCAN00000065620	Homo sapiens cyclin B2 (CCNB2), mRNA.<br><br>cdna:known-ccds chromosome:NCBI36:15:57184612:57204535:1 gene:ENSG00000157456 CCDS10170.1<br>cdna:Genscan chromosome:NCBI36:15:57092573:57204368:1

		3595997<br>3595998	core<br>extended						
3597338	796797<br>796798<br>796799<br>796800<br>796801<br>796802<br>796803<br>796804<br>796805<br>796806<br>796807<br>796808<br>796809<br>796810<br>796811<br>796812<br>796813<br>796814<br>796815<br>796816<br>796817<br>796818<br>796819<br>796820<br>796821<br>796822<br>796823<br>796824<br>796825<br>796826<br>796827<br>796828<br>796829<br>796830<br>796831	3597339<br>3597340<br>3597341<br>3597342<br>3597343<br>3597344<br>3597345<br>3597346<br>3597347<br>3597348<br>3597349<br>3597350<br>3597351<br>3597352<br>3597353<br>3597354<br>3597355<br>3597356<br>3597357<br>3597358<br>3597359<br>3597360<br>3597361<br>3597362<br>3597363<br>3597364<br>3597365<br>3597366<br>3597367<br>3597368<br>3597369<br>3597370<br>3597371<br>3597372<br>3597373<br>3597374<br>3597375<br>3597376<br>3597377<br>3597378<br>3597379<br>3597380<br>3597381<br>3597382<br>3597383<br>3597384<br>3597385<br>3597386<br>3597387<br>3597388<br>3597389<br>3597390	full<br>full<br>full<br>full<br>full<br>full<br>full<br>full<br>extended<br>core<br>core<br>extended<br>extended<br>extended<br>core<br>extended<br>core<br>extended<br>extended<br>extended<br>full<br>extended<br>extended<br>extended<br>core<br>core<br>extended<br>extended<br>extended<br>extended<br>extended<br>extended<br>extended<br>extended<br>extended<br>extended<br>core<br>core<br>extended<br>extended<br>extended<br>core<br>extended<br>core<br>extended<br>core<br>extended<br>extended<br>extended<br>core<br>extended<br>core	15	61105711	61151151	+	NM_001018007<br>NM_001018008<br>NM_001018005<br>NM_001018006<br>NM_000366<br>NM_001018004<br>NM_001018020<br>AF474156<br>AK055197<br>AK092051<br>AK092573<br>AK094175<br>AK131384<br>AY640414<br>BC053545<br>DQ424903<br>X12369<br>M19267<br>M19713<br>M19714<br>ENSESTT00000056490<br>ENSESTT00000056491<br>ENSESTT00000056492<br>ENSESTT00000056493<br>ENSESTT00000056494<br>ENSESTT00000056495<br>ENST00000288398<br>ENST00000358278<br>ENST00000317516<br>ENST00000357980<br>ENST00000267996<br>ENST00000380364<br>ENST00000334895<br>GENSCAN00000013116<br>GENSCAN00000013115	Homo sapiens tropomyosin 1 (alpha) (TPM1), transcript variant 2, mRNA.<br>Homo sapiens tropomyosin 1 (alpha) (TPM1), transcript variant 6, mRNA.<br>Homo sapiens tropomyosin 1 (alpha) (TPM1), transcript variant 1, mRNA.<br>Homo sapiens tropomyosin 1 (alpha) (TPM1), transcript variant 4, mRNA.<br>Homo sapiens tropomyosin 1 (alpha) (TPM1), transcript variant 5, mRNA.<br>Homo sapiens tropomyosin 1 (alpha) (TPM1), transcript variant 3, mRNA.<br>Homo sapiens tropomyosin 1 (alpha) (TPM1), transcript variant 7, mRNA.<br>Homo sapiens TPMsk1 (TPM1) mRNA, partial cds.<br>Homo sapiens cDNA FLJ30635 fis, clone CTONG2002520.<br>Homo sapiens cDNA FLJ34732 fis, clone MESAN2006743, moderately similar to TROPOMYOSIN, FIBROBLAST ISOFORM TM3.<br>Homo sapiens cDNA FLJ35254 fis, clone PROST2003775, highly similar to TROPOMYOSIN, FIBROBLAST ISOFORM TM3.<br>Homo sapiens cDNA FLJ36856 fis, clone ASTRO2014863.<br>Homo sapiens cDNA FLJ16459 fis, clone BRCAN2002473, moderately similar to Tropomyosin, fibroblast isoform 2.<br>Homo sapiens sarcomeric tropomyosin kappa (TPM1) mRNA, complete cds; alternatively spliced.<br>Homo sapiens tropomyosin 1 (alpha), mRNA (cDNA clone MGC:61803 IMAGE:5730451), complete cds.<br>Homo sapiens clone FG060306 tropomyosin 1 alpha variant 6 mRNA, complete cds, alternatively spliced.<br>Human mRNA (exon 6-9 part.) for smooth muscle tropomyosin.<br>Human tropomyosin mRNA, complete cds.<br>Human skeletal muscle alpha-tropomyosin (hTM-alpha) mRNA, complete cds.<br>Human skeletal muscle alpha-tropomyosin gene (hTM-alpha) mRNA, 3' end.<br>cdna:known-ccds chromosome:NCBI36:15:61121891:61145344:1 gene:ENSG00000140416 CCDS10181.1<br>cdna:known chromosome:NCBI36:15:61121891:61151164:1 gene:ENSG00000140416<br>cdna:known chromosome:NCBI36:15:61121891:61150413:1 gene:ENSG00000140416<br>cdna:known chromosome:NCBI36:15:61122003:61151164:1 gene:ENSG00000140416<br>cdna:known chromosome:NCBI36:15:61122043:61151157:1 gene:ENSG00000140416<br>cdna:known chromosome:NCBI36:15:61122082:61143398:1 gene:ENSG00000140416<br>cdna:known chromosome:NCBI36:15:61127652:61151164:1 gene:ENSG00000140416<br>cdna:Genscan chromosome:NCBI36:15:61105711:61111660:1<br>cdna:Genscan chromosome:NCBI36:15:61121422:61143398:1

		3597391<br>3597392<br>3597393<br>3597394<br>3597395<br>3597396<br>3597397<br>3597398<br>3597399<br>3597400<br>3597401<br>3597402<br>3597403<br>3597404<br>3597405<br>3597406<br>3597407<br>3597408	extended<br>extended<br>core<br>extended<br>core<br>core<br>extended<br>core<br>core<br>extended<br>extended<br>core<br>core<br>core<br>extended<br>core<br>core<br>extended						
3598758	797662<br>797663<br>797664<br>797665<br>797666<br>797667<br>797668<br>797669<br>797670<br>797671<br>797672<br>797673<br>797674<br>797675<br>797676<br>797677<br>797678<br>797679<br>797680<br>797681<br>797682<br>797683<br>797684<br>797685<br>797686<br>797687<br>797688<br>797689<br>797690<br>797691<br>797692<br>797693<br>797694<br>797695<br>797696<br>797697	3598759<br>3598760<br>3598761<br>3598762<br>3598763<br>3598764<br>3598765<br>3598766<br>3598767<br>3598768<br>3598769<br>3598770<br>3598771<br>3598772<br>3598773<br>3598774<br>3598775<br>3598776<br>3598777<br>3598778<br>3598779<br>3598780<br>3598781<br>3598782<br>3598783<br>3598784<br>3598785<br>3598786<br>3598787<br>3598788<br>3598789<br>3598790<br>3598791<br>3598792<br>3598793<br>3598794	full<br>full<br>full<br>full<br>full<br>full<br>full<br>full<br>full<br>full<br>full<br>full<br>full<br>full<br>full<br>extended<br>core<br>core<br>core<br>core<br>core<br>core<br>core<br>core<br>core<br>core<br>core<br>core<br>core<br>core<br>extended<br>extended<br>core<br>extended<br>extended<br>full	15	64660815	64861380	+	NM_005585<br>AF035528<br>BC052569<br>U59914<br>ENSESTT00000055702<br>ENSESTT00000055703<br>ENST00000288840<br>ENST00000338426<br>GENSCAN00000031932<br>GENSCAN00000015553	Homo sapiens SMAD, mothers against DPP homolog 6 (Drosophila) (SMAD6), mRNA.<br>Homo sapiens Smad6 mRNA, complete cds.<br>Homo sapiens SMAD, mothers against DPP homolog 6 (Drosophila), mRNA (cDNA clone MGC:59969 IMAGE:5922640), complete cds.<br>Human chromosome 15 Mad homolog Smad6 mRNA, complete cds.<br><br><br>cdna:known-ccds chromosome:NCBI36:15:64781722:64861377:1 gene:ENSG00000137834 CCDS10221.1<br>cdna:known chromosome:NCBI36:15:64788097:64861377:1 gene:ENSG00000137834<br>cdna:Genscan chromosome:NCBI36:15:64860397:64860927:1<br>cdna:Genscan chromosome:NCBI36:15:64683564:64804329:1

	797698<br>797699<br>797700<br>797701<br>797702	3598795<br>3598796<br>3598797<br>3598798<br>3598799<br>3598800<br>3598801<br>3598802<br>3598803<br>3598804<br>3598805<br>3598806<br>3598807<br>3598808<br>3598809<br>3598810<br>3598811<br>3598812<br>3598813<br>3598814<br>3598815<br>3598816<br>3598817<br>3598818<br>3598819	core<br>extended<br>extended<br>core<br>full<br>extended<br>full<br>full<br>full<br>full<br>extended<br>extended<br>extended<br>extended<br>full<br>full<br>full<br>full<br>full<br>full<br>full<br>extended<br>extended<br>core<br>core						
3599811	798357<br>798358<br>798359<br>798360<br>798361<br>798362<br>798363<br>798364<br>798365<br>798366<br>798367<br>798368<br>798369<br>798370<br>798371<br>798372<br>798373<br>798374<br>798375<br>798376<br>798377<br>798378<br>798379<br>798380<br>798381<br>798382<br>798383	3599812<br>3599813<br>3599814<br>3599815<br>3599816<br>3599817<br>3599818<br>3599819<br>3599820<br>3599821<br>3599822<br>3599823<br>3599824<br>3599825<br>3599826<br>3599827<br>3599828<br>3599829<br>3599830<br>3599831<br>3599832<br>3599833<br>3599834<br>3599835<br>3599836<br>3599837<br>3599838<br>3599839<br>3599840	extended<br>core<br>extended<br>core<br>core<br>core<br>core<br>extended<br>core<br>extended<br>extended<br>core<br>extended<br>core<br>core<br>full<br>core<br>core<br>core<br>core<br>core<br>core<br>full<br>extended<br>core<br>core<br>core<br>core<br>extended	15	67493677	67527808	+	NM_138555<br>NM_004856<br>X67155<br>ENSESTT00000003162<br>ENSESTT00000003163<br>ENSESTT00000003164<br>ENSESTT00000003165<br>ENSESTT00000003166<br>ENSESTT00000003167<br>ENST00000352331<br>ENST00000260363<br>GENSCAN00000006631	Homo sapiens kinesin family member 23 (KIF23), transcript variant 1, mRNA.<br>Homo sapiens kinesin family member 23 (KIF23), transcript variant 2, mRNA.<br>Homo sapiens mRNA for mitotic kinesin-like protein-1 (MKLP-1 gene).<br><br><br><br><br><br><br>cdna:known chromosome:NCBI36:15:67493697:67527810:1 gene:ENSG00000137807<br>cdna:known chromosome:NCBI36:15:67493742:67527810:1 gene:ENSG00000137807<br>cdna:Genscan chromosome:NCBI36:15:67437393:67534900:1

		3599841<br>3599842<br>3599843<br>3599844<br>3599845<br>3599846<br>3599847<br>3599848<br>3599849<br>3599850<br>3599851<br>3599852<br>3599853	extended<br>extended<br>core<br>extended<br>core<br>extended<br>extended<br>core<br>extended<br>extended<br>core<br>core<br>core						
3604147	801005<br>801006<br>801007<br>801008<br>801009<br>801010<br>801011<br>801012<br>801013<br>801014<br>801015<br>801016<br>801017<br>801018<br>801019<br>801020<br>801021<br>801022<br>801023<br>801024<br>801025<br>801026<br>801027<br>801028<br>801029<br>801030<br>801031<br>801032<br>801033<br>801034<br>801035<br>801036<br>801037<br>801038<br>801039<br>801040<br>801041<br>801042<br>801043<br>801044<br>801045	3604148<br>3604149<br>3604150<br>3604151<br>3604152<br>3604153<br>3604154<br>3604155<br>3604156<br>3604157<br>3604158<br>3604159<br>3604160<br>3604161<br>3604162<br>3604163<br>3604164<br>3604165<br>3604166<br>3604167<br>3604168<br>3604169<br>3604170<br>3604171<br>3604172<br>3604173<br>3604174<br>3604175<br>3604176<br>3604177<br>3604178<br>3604179<br>3604180<br>3604181<br>3604182<br>3604183<br>3604184<br>3604185<br>3604186<br>3604187<br>3604188	core<br>full<br>full<br>extended<br>extended<br>full<br>extended<br>extended<br>extended<br>full<br>full<br>full<br>extended<br>full<br>full<br>full<br>full<br>full<br>full<br>extended<br>extended<br>extended<br>core<br>full<br>core<br>core<br>core<br>full<br>core<br>full<br>extended<br>core<br>extended<br>extended<br>core<br>extended<br>core<br>extended<br>core<br>core<br>core	15	78858767	79031152	+	NM_018689<br>ENSESTT00000056272<br>ENSESTT00000056273<br>ENSESTT00000056274<br>ENSESTT00000056275<br>ENSESTT00000056276<br>ENSESTT00000056277<br>ENSESTT00000056278<br>ENST00000220244<br>ENST00000356249<br>GENSCAN00000029643	Homo sapiens KIAA1199 (KIAA1199), mRNA.<br><br><br><br><br><br><br><br>cdna:known-ccds chromosome:NCBI36:15:78858767:79031054:1 gene:ENSG00000103888 CCDS10315.1<br>cdna:known chromosome:NCBI36:15:78858767:79031054:1 gene:ENSG00000103888<br>cdna:Genscan chromosome:NCBI36:15:78916631:79028320:1

	801046<br>801047<br>801048<br>801049<br>801050<br>801051<br>801052<br>801053<br>801054<br>801055<br>801056<br>801057<br>801058<br>801059<br>801060<br>801061<br>801062<br>801063<br>801064<br>801065	3604189<br>3604190<br>3604191<br>3604192<br>3604193<br>3604194<br>3604195<br>3604196<br>3604197<br>3604198<br>3604199<br>3604200<br>3604201<br>3604202<br>3604203<br>3604204<br>3604205<br>3604206<br>3604207<br>3604208<br>3604209<br>3604210<br>3604211<br>3604212<br>3604213<br>3604214<br>3604215<br>3604216<br>3604217<br>3604218<br>3604219<br>3604220<br>3604221	full<br>extended<br>full<br>full<br>full<br>core<br>core<br>extended<br>core<br>core<br>core<br>core<br>core<br>core<br>core<br>core<br>core<br>core<br>core<br>core<br>extended<br>extended<br>core<br>core<br>core<br>full<br>full<br>core<br>core<br>core<br>core<br>core<br>extended						
3605395	801769<br>801770<br>801771<br>801772<br>801773<br>801774<br>801775<br>801776<br>801777<br>801778<br>801779<br>801780<br>801781<br>801782<br>801783<br>801784<br>801785<br>801786<br>801787<br>801788<br>801789	3605396<br>3605397<br>3605398<br>3605399<br>3605400<br>3605401<br>3605402<br>3605403<br>3605404<br>3605405<br>3605406<br>3605407<br>3605408<br>3605409<br>3605410<br>3605411<br>3605412<br>3605413<br>3605414<br>3605415<br>3605416	core<br>full<br>core<br>core<br>full<br>extended<br>extended<br>extended<br>extended<br>full<br>full<br>full<br>extended<br>full<br>core<br>core<br>full<br>full<br>full<br>full<br>full	15	82113852	82499577	+	NM_207517<br>ENSESTT00000002568<br>ENSESTT00000002569<br>ENSESTT00000002570<br>ENSESTT00000002669<br>ENST00000286744<br>ENST00000286745<br>GENSCAN00000025265<br>GENSCAN00000025272<br>GENSCAN00000025268<br>GENSCAN00000035975	Homo sapiens ADAMTS-like 3 (ADAMTSL3), mRNA.<br><br><br><br><br>cdna:known-ccds chromosome:NCBI36:15:82113842:82499595:1 gene:ENSG00000156218 CCDS10326.1<br>cdna:known chromosome:NCBI36:15:82113842:82321329:1 gene:ENSG00000156218<br>cdna:Genscan chromosome:NCBI36:15:82352759:82373928:1<br>cdna:Genscan chromosome:NCBI36:15:82297783:82346678:1<br>cdna:Genscan chromosome:NCBI36:15:82199704:82279905:1<br>cdna:Genscan chromosome:NCBI36:15:82442043:82491192:1

	801790	3605417	extended						
	801791	3605418	full						
	801792	3605419	full						
	801793	3605420	full						
	801794	3605421	full						
	801795	3605422	full						
	801796	3605423	extended						
	801797	3605424	extended						
	801798	3605425	full						
	801799	3605426	full						
	801800	3605427	full						
	801801	3605428	full						
	801802	3605429	full						
	801803	3605430	full						
	801804	3605431	core						
	801805	3605432	extended						
	801806	3605433	core						
	801807	3605434	extended						
	801808	3605435	core						
	801809	3605436	extended						
	801810	3605437	core						
	801811	3605438	full						
	801812	3605439	full						
	801813	3605440	core						
	801814	3605441	extended						
	801815	3605442	core						
	801816	3605443	extended						
	801817	3605444	core						
	801818	3605445	core						
	801819	3605446	full						
	801820	3605447	core						
	801821	3605448	core						
	801822	3605449	core						
	801823	3605450	full						
	801824	3605451	full						
	801825	3605452	core						
	801826	3605453	core						
	801827	3605454	extended						
	801828	3605455	extended						
	801829	3605456	extended						
	801830	3605457	core						
	801831	3605458	full						
	801832	3605459	full						
	801833	3605460	core						
	801834	3605461	extended						
	801835	3605462	core						
	801836	3605463	core						
	801837	3605464	full						
	801838	3605465	extended						
	801839	3605466	extended						
	801840	3605467	core						
	801841	3605468	full						
	801842	3605469	full						
	801843	3605470	full						

	801844<br>801845<br>801846<br>801847<br>801848<br>801849<br>801850<br>801851<br>801852<br>801853	3605471<br>3605472<br>3605473<br>3605474<br>3605475<br>3605476<br>3605477<br>3605478<br>3605479<br>3605480<br>3605481<br>3605482<br>3605483<br>3605484<br>3605485<br>3605486<br>3605487<br>3605488<br>3605489<br>3605490<br>3605491<br>3605492<br>3605493<br>3605494<br>3605495<br>3605496<br>3605497	full<br>core<br>extended<br>core<br>core<br>full<br>full<br>extended<br>full<br>full<br>extended<br>full<br>full<br>full<br>core<br>core<br>core<br>core<br>core<br>core<br>core<br>core<br>full<br>core<br>core<br>core<br>core						
3607537	803098<br>803099<br>803100<br>803101<br>803102<br>803103<br>803104<br>803105<br>803106<br>803107<br>803108<br>803109<br>803110<br>803111<br>803112<br>803113<br>803114<br>803115<br>803116<br>803117<br>803118<br>803119<br>803120<br>803121<br>803122<br>803123<br>803124	3607538<br>3607539<br>3607540<br>3607541<br>3607542<br>3607543<br>3607544<br>3607545<br>3607546<br>3607547<br>3607548<br>3607549<br>3607550<br>3607551<br>3607552<br>3607553<br>3607554<br>3607555<br>3607556<br>3607557<br>3607558<br>3607559<br>3607560<br>3607561<br>3607562<br>3607563<br>3607564	extended<br>core<br>core<br>core<br>core<br>full<br>core<br>core<br>core<br>extended<br>core<br>core<br>core<br>extended<br>extended<br>core<br>extended<br>extended<br>core<br>core<br>core<br>core<br>extended<br>extended<br>extended<br>extended<br>extended	15	87618421	87661942	+	NM_018193<br>AB058697<br>BC004277<br>ENSESTT00000019515<br>ENSESTT00000019516<br>ENSESTT00000019517<br>ENSESTT00000019518<br>ENSESTT00000019519<br>ENSESTT00000019520<br>ENSESTT00000019521<br>ENSESTT00000019522<br>ENSESTT00000019523<br>ENSESTT00000019524<br>ENSESTT00000019525<br>ENSESTT00000019526<br>ENSESTT00000019527<br>ENSESTT00000019528<br>ENSESTT00000019529<br>ENSESTT00000019530<br>ENSESTT00000019531<br>ENSESTT00000019532<br>ENST00000310775<br>ENST00000300027<br>ENST00000345047<br>GENSCAN00000000357	Homo sapiens KIAA1794 (KIAA1794), mRNA.<br>Homo sapiens mRNA for KIAA1794 protein, partial cds.<br>Homo sapiens KIAA1794, mRNA (cDNA clone IMAGE:3615489), complete cds.<br>cdna:known-ccds chromosome:NCBI36:15:87588198:87661366:1 gene:ENSG00000140525 CCDS10349.1<br>cdna:known chromosome:NCBI36:15:87588198:87661366:1 gene:ENSG00000140525<br>cdna:known chromosome:NCBI36:15:87622915:87661363:1 gene:ENSG00000140525<br>cdna:Genscan chromosome:NCBI36:15:87591883:87659666:1

	803125<br>803126<br>803127<br>803128<br>803129<br>803130<br>803131<br>803132<br>803133<br>803134	3607565<br>3607566<br>3607567<br>3607568<br>3607569<br>3607570<br>3607571<br>3607572<br>3607573<br>3607574<br>3607575<br>3607576<br>3607577<br>3607578<br>3607579<br>3607580<br>3607581<br>3607582<br>3607583<br>3607584<br>3607585<br>3607586<br>3607587<br>3607588<br>3607589<br>3607590	core<br>core<br>core<br>full<br>core<br>core<br>core<br>core<br>core<br>core<br>core<br>core<br>core<br>extended<br>core<br>extended<br>extended<br>extended<br>core<br>core<br>core<br>extended<br>full<br>core<br>core<br>extended						
3610982	805286<br>805287<br>805288<br>805289<br>805290<br>805291<br>805292<br>805293<br>805294<br>805295<br>805296<br>805297<br>805298<br>805299<br>805300<br>805301<br>805302<br>805303<br>805304<br>805305<br>805306<br>805307<br>805308<br>805309<br>805310<br>805311	3610983<br>3610984<br>3610985<br>3610986<br>3610987<br>3610988<br>3610989<br>3610990<br>3610991<br>3610992<br>3610993<br>3610994<br>3610995<br>3610996<br>3610997<br>3610998<br>3610999<br>3611000<br>3611001<br>3611002<br>3611003<br>3611004<br>3611005<br>3611006<br>3611007<br>3611008<br>3611009<br>3611010	full<br>full<br>full<br>full<br>full<br>extended<br>full<br>full<br>extended<br>full<br>full<br>full<br>extended<br>core<br>core<br>core<br>core<br>core<br>core<br>core<br>full<br>full<br>full<br>core<br>core<br>full<br>extended<br>full	15	97424857	97493311	+	NM_015286<br>NM_145728<br>BC110066<br>AL833262<br>ENSESTT00000032557<br>ENST00000328642<br>ENST00000336292<br>GENSCAN00000002866	Homo sapiens desmuslin (DMN), transcript variant B, mRNA.<br>Homo sapiens desmuslin (DMN), transcript variant A, mRNA.<br>Homo sapiens desmuslin, mRNA (cDNA clone IMAGE:40027592), partial cds.<br>Homo sapiens mRNA; cDNA DKFZp451C2210 (from clone DKFZp451C2210).<br><br>cdna:known chromosome:NCBI36:15:97462771:97490786:1 gene:ENSG00000182253<br>cdna:known chromosome:NCBI36:15:97462809:97490786:1 gene:ENSG00000182253<br>cdna:Genscan chromosome:NCBI36:15:97424857:97490786:1

		3611011<br>3611012<br>3611013<br>3611014<br>3611015<br>3611016<br>3611017<br>3611018<br>3611019<br>3611020<br>3611021<br>3611022<br>3611023<br>3611024<br>3611025	full<br>full<br>full<br>core<br>core<br>core<br>core<br>core<br>core<br>core<br>core<br>core<br>core<br>core<br>core						
3629243	816715<br>816716<br>816717<br>816718<br>816719<br>816720<br>816721<br>816722<br>816723<br>816724<br>816725<br>816726<br>816727<br>816728<br>816729<br>816730<br>816731<br>816732	3629244<br>3629245<br>3629246<br>3629247<br>3629248<br>3629249<br>3629250<br>3629251<br>3629252<br>3629253<br>3629254<br>3629255<br>3629256<br>3629257<br>3629258<br>3629259<br>3629260<br>3629261<br>3629262<br>3629263<br>3629264<br>3629265	core<br>core<br>core<br>core<br>core<br>core<br>full<br>core<br>core<br>core<br>core<br>core<br>full<br>full<br>extended<br>full<br>full<br>full<br>full<br>core<br>extended<br>extended	15	62819156	62855719	-	NM_194272<br>AK124123<br>ENSESTT00000044177<br>ENST00000300069<br>GENSCAN00000039549	Homo sapiens RNA binding protein with multiple splicing 2 (RBPMS2), mRNA.<br>Homo sapiens cDNA FLJ42129 fis, clone TESTI2013889, moderately similar to Homo sapiens RNA binding protein with multiple splicing (RBPMS).<br><br>cdna:known chromosome:NCBI36:15:62819150:62854839:-1 gene:ENSG00000166831<br>cdna:Genscan chromosome:NCBI36:15:62851518:62854571:-1
3630736	817633<br>817634<br>817635<br>817636<br>817637<br>817638<br>817639<br>817640<br>817641<br>817642<br>817643<br>817644<br>817645<br>817646<br>817647<br>817648	3630737<br>3630738<br>3630739<br>3630740<br>3630741<br>3630742<br>3630743<br>3630744<br>3630745<br>3630746<br>3630747<br>3630748<br>3630749<br>3630750<br>3630751<br>3630752	extended<br>extended<br>extended<br>extended<br>extended<br>extended<br>core<br>core<br>core<br>core<br>core<br>core<br>core<br>core<br>full<br>core	15	66375474	66511535	-	NM_012211<br>NM_001004439<br>AL117454<br>AL359064<br>ENSESTT00000019384<br>ENSESTT00000019385<br>ENSESTT00000019386<br>ENSESTT00000019387<br>ENSESTT00000019388<br>ENSESTT00000019389<br>ENST00000315757<br>GENSCAN00000028557	Homo sapiens integrin, alpha 11 (ITGA11), transcript variant 2, mRNA.<br>Homo sapiens integrin, alpha 11 (ITGA11), transcript variant 1, mRNA.<br>Homo sapiens mRNA; cDNA DKFZp586J1717 (from clone DKFZp586J1717).<br>Homo sapiens mRNA full length insert cDNA clone EUROIMAGE 322987.<br><br><br><br><br><br><br>cdna:known chromosome:NCBI36:15:66381469:66511531:-1 gene:ENSG00000137809<br>cdna:Genscan chromosome:NCBI36:15:66381546:66487450:-1

	817649	3630753	core						
	817650	3630754	core						
	817651	3630755	core						
	817652	3630756	core						
	817653	3630757	core						
	817654	3630758	core						
	817655	3630759	core						
	817656	3630760	core						
	817657	3630761	full						
	817658	3630762	core						
	817659	3630763	full						
	817660	3630764	core						
	817661	3630765	extended						
	817662	3630766	core						
	817663	3630767	extended						
	817664	3630768	extended						
	817665	3630769	extended						
	817666	3630770	extended						
	817667	3630771	core						
	817668	3630772	core						
	817669	3630773	core						
	817670	3630774	full						
	817671	3630775	full						
	817672	3630776	core						
	817673	3630777	core						
	817674	3630778	full						
	817675	3630779	core						
	817676	3630780	extended						
	817677	3630781	full						
	817678	3630782	core						
	817679	3630783	core						
	817680	3630784	full						
	817681	3630785	core						
	817682	3630786	full						
	817683	3630787	full						
	817684	3630788	full						
	817685	3630789	full						
	817686	3630790	full						
	817687	3630791	full						
	817688	3630792	core						
	817689	3630793	core						
	817690	3630794	full						
	817691	3630795	core						
	817692	3630796	full						
	817693	3630797	core						
	817694	3630798	extended						
	817695	3630799	extended						
	817696	3630800	extended						
	817697	3630801	core						
	817698	3630802	full						
	817699	3630803	core						
	817700	3630804	core						
		3630805	full						
		3630806	core						

		3630807<br>3630808<br>3630809<br>3630810<br>3630811<br>3630812<br>3630813<br>3630814<br>3630815<br>3630816<br>3630817<br>3630818<br>3630819<br>3630820<br>3630821<br>3630822<br>3630823<br>3630824<br>3630825<br>3630826<br>3630827<br>3630828<br>3630829<br>3630830<br>3630831<br>3630832<br>3630833<br>3630834<br>3630835	extended<br>core<br>full<br>full<br>full<br>full<br>full<br>full<br>full<br>full<br>full<br>full<br>full<br>full<br>core<br>full<br>full<br>full<br>full<br>full<br>full<br>full<br>full<br>full<br>full<br>full<br>full<br>core<br>core						
3632806	818891<br>818892<br>818893<br>818894<br>818895<br>818896<br>818897<br>818898<br>818899<br>818900<br>818901<br>818902<br>818903<br>818904<br>818905<br>818906<br>818907<br>818908<br>818909<br>818910<br>818911<br>818912<br>818913<br>818914<br>818915	3632807<br>3632808<br>3632809<br>3632810<br>3632811<br>3632812<br>3632813<br>3632814<br>3632815<br>3632816<br>3632817<br>3632818<br>3632819<br>3632820<br>3632821<br>3632822<br>3632823<br>3632824<br>3632825<br>3632826<br>3632827<br>3632828<br>3632829<br>3632830<br>3632831	core<br>core<br>extended<br>core<br>core<br>core<br>core<br>core<br>core<br>core<br>full<br>extended<br>extended<br>core<br>extended<br>core<br>core<br>core<br>core<br>core<br>core<br>core<br>extended<br>core<br>core	15	72258874	72364951	-	NM_022369<br>AK056125<br>AK091152<br>AK092227<br>BC015881<br>BX537413<br>ENSESTT00000022349<br>ENSESTT00000022350<br>ENSESTT00000022351<br>ENSESTT00000022352<br>ENSESTT00000022353<br>ENSESTT00000022354<br>ENST00000323940<br>GENSCAN00000056778	Homo sapiens stimulated by retinoic acid gene 6 homolog (mouse) (STRA6), mRNA.<br>Homo sapiens cDNA FLJ31563 fis, clone NT2RI2001449, moderately similar to Mus musculus retinoic acid-responsive protein (Stra6) mRNA.<br>Homo sapiens cDNA FLJ33833 fis, clone CTONG2004126, moderately similar to Mus musculus retinoic acid-responsive protein (Stra6) mRNA.<br>Homo sapiens cDNA FLJ34908 fis, clone NT2RI2006294, moderately similar to Mus musculus retinoic acid-responsive protein (Stra6) mRNA.<br>Homo sapiens stimulated by retinoic acid gene 6 homolog (mouse), mRNA (cDNA clone IMAGE:3530901), complete cds.<br>Homo sapiens mRNA; cDNA DKFZp686P1959 (from clone DKFZp686P1959); complete cds.<br><br><br><br><br><br><br>cdna:known-ccds chromosome:NCBI36:15:72258863:72288419:-1 gene:ENSG00000137868 CCDS10261.1<br>cdna:Genscan chromosome:NCBI36:15:72259474:72281661:-1

	818916<br>818917<br>818918<br>818919	3632832<br>3632833<br>3632834<br>3632835<br>3632836<br>3632837<br>3632838<br>3632839<br>3632840<br>3632841<br>3632842<br>3632843<br>3632844<br>3632845<br>3632846<br>3632847	extended<br>core<br>core<br>core<br>extended<br>core<br>extended<br>core<br>core<br>extended<br>extended<br>extended<br>extended<br>extended<br>extended<br>extended						
3638607	822444<br>822445<br>822446<br>822447<br>822448<br>822449<br>822450<br>822451<br>822452<br>822453<br>822454<br>822455<br>822456<br>822457<br>822458<br>822459<br>822460<br>822461<br>822462<br>822463<br>822464<br>822465<br>822466<br>822467<br>822468<br>822469<br>822470<br>822471<br>822472<br>822473<br>822474<br>822475<br>822476	3638608<br>3638609<br>3638610<br>3638611<br>3638612<br>3638613<br>3638614<br>3638615<br>3638616<br>3638617<br>3638618<br>3638619<br>3638620<br>3638621<br>3638622<br>3638623<br>3638624<br>3638625<br>3638626<br>3638627<br>3638628<br>3638629<br>3638630<br>3638631<br>3638632<br>3638633<br>3638634<br>3638635<br>3638636<br>3638637<br>3638638<br>3638639<br>3638640<br>3638641<br>3638642<br>3638643<br>3638644<br>3638645	core<br>core<br>core<br>core<br>core<br>extended<br>core<br>core<br>core<br>extended<br>extended<br>extended<br>extended<br>extended<br>core<br>core<br>core<br>core<br>extended<br>extended<br>extended<br>core<br>full<br>core<br>extended<br>core<br>full<br>core<br>extended<br>core<br>full<br>core<br>core<br>core<br>full<br>core<br>core<br>core	15	88129142	88159617	-	NM_001150<br>ENSESTT00000019549<br>ENSESTT00000019550<br>ENSESTT00000019551<br>ENST00000300060<br>GENSCAN00000035739	Homo sapiens alanyl (membrane) aminopeptidase (aminopeptidase N, aminopeptidase M, microsomal aminopeptidase, CD13, p150) (ANPEP), mRNA.<br><br><br><br>cdna:known-ccds chromosome:NCBI36:15:88129131:88159072:-1 gene:ENSG00000166825 CCDS10356.1<br>cdna:Genscan chromosome:NCBI36:15:88129584:88143794:-1

		3638646<br>3638647<br>3638648<br>3638649<br>3638650<br>3638651<br>3638652<br>3638653<br>3638654<br>3638655<br>3638656	core<br>core<br>core<br>extended<br>extended<br>full<br>full<br>full<br>extended<br>full<br>extended						
3643143	825261<br>825262<br>825263<br>825264<br>825265<br>825266<br>825267<br>825268<br>825269<br>825270<br>825271<br>825272<br>825273<br>825274<br>825275<br>825276<br>825277<br>825278<br>825279<br>825280<br>825281<br>825282<br>825283<br>825284<br>825285<br>825286<br>825287<br>825288	3643144<br>3643145<br>3643146<br>3643147<br>3643148<br>3643149<br>3643150<br>3643151<br>3643152<br>3643153<br>3643154<br>3643155<br>3643156<br>3643157<br>3643158<br>3643159<br>3643160<br>3643161<br>3643162<br>3643163<br>3643164<br>3643165<br>3643166<br>3643167<br>3643168<br>3643169<br>3643170<br>3643171<br>3643172<br>3643173<br>3643174<br>3643175<br>3643176<br>3643177<br>3643178<br>3643179<br>3643180<br>3643181<br>3643182<br>3643183<br>3643184<br>3643185<br>3643186	core<br>core<br>core<br>core<br>extended<br>core<br>core<br>core<br>extended<br>core<br>core<br>core<br>core<br>core<br>core<br>core<br>core<br>core<br>core<br>core<br>core<br>core<br>core<br>core<br>extended<br>core<br>core<br>core<br>core<br>core<br>core<br>core<br>core<br>core<br>core<br>core<br>core<br>core<br>full<br>core<br>core<br>core<br>extended	16	639312	650702	+	NM_145294<br>AB067511<br>AK091062<br>AK092719<br>AK093609<br>AK126622<br>AK131510<br>BC065836<br>BC013032<br>ENSESTT00000033328<br>ENSESTT00000033329<br>ENSESTT00000033330<br>ENSESTT00000033335<br>ENSESTT00000033336<br>ENSESTT00000033337<br>ENSESTT00000033338<br>ENSESTT00000033339<br>ENSESTT00000033340<br>ENSESTT00000033341<br>ENSESTT00000033342<br>ENSESTT00000033343<br>ENSESTT00000033344<br>ENSESTT00000033345<br>ENSESTT00000033346<br>ENST00000317787<br>ENST00000293879<br>ENST00000389495<br>ENST00000293880<br>ENST00000315764<br>GENSCAN00000033001<br>GENSCAN00000006401	Homo sapiens hypothetical protein KIAA1924 (KIAA1924), mRNA.<br>Homo sapiens mRNA for KIAA1924 protein, partial cds.<br>Homo sapiens cDNA FLJ33743 fis, clone BRAWH2019055.<br>Homo sapiens cDNA FLJ35400 fis, clone SKNSH2005240.<br>Homo sapiens cDNA FLJ36290 fis, clone THYMU2003932.<br>Homo sapiens cDNA FLJ44660 fis, clone BRACE3002344, weakly similar to Vegetatible incompatibility protein HET-E-1.<br>Homo sapiens cDNA FLJ16728 fis, clone UTERU3015412.<br>Homo sapiens WD repeat domain 90, mRNA (cDNA clone IMAGE:30407723), complete cds.<br>Homo sapiens cDNA clone IMAGE:3534806, **** WARNING: chimeric clone ****.<br>cdna:known chromosome:NCBI36:16:639312:651076:1 gene:ENSG00000161996<br>cdna:known chromosome:NCBI36:16:639312:651076:1 gene:ENSG00000161996<br>cdna:known chromosome:NCBI36:16:639418:643846:1 gene:ENSG00000161996<br>cdna:known chromosome:NCBI36:16:645059:653434:1 gene:ENSG00000161996<br>cdna:known chromosome:NCBI36:16:654996:657830:1 gene:ENSG00000161998<br>cdna:Genscan chromosome:NCBI36:16:626784:646538:1<br>cdna:Genscan chromosome:NCBI36:16:646816:668053:1

		3643187<br>3643188<br>3643189<br>3643190<br>3643191	extended<br>core<br>extended<br>core<br>core						
3643396	825383<br>825384<br>825385<br>825386<br>825387<br>825388<br>825389<br>825390<br>825391<br>825392<br>825393<br>825394<br>825395<br>825396<br>825397<br>825398<br>825399<br>825400	3643397<br>3643398<br>3643399<br>3643400<br>3643401<br>3643402<br>3643403<br>3643404<br>3643405<br>3643406<br>3643407<br>3643408<br>3643409<br>3643410<br>3643411<br>3643412<br>3643413<br>3643414<br>3643415<br>3643416<br>3643417<br>3643418<br>3643419<br>3643420<br>3643421<br>3643422<br>3643423<br>3643424<br>3643425<br>3643426<br>3643427<br>3643428	core<br>core<br>core<br>core<br>extended<br>core<br>core<br>core<br>core<br>core<br>core<br>core<br>core<br>full<br>core<br>core<br>full<br>core<br>core<br>core<br>core<br>core<br>core<br>core<br>core<br>core<br>core<br>extended<br>core<br>core<br>core<br>full	16	750777	759156	+	NM_005823<br>NM_013404<br>AF180951<br>U40434<br>ENSESTT00000033373<br>ENSESTT00000033374<br>ENSESTT00000033375<br>ENST00000382862<br>ENST00000318336<br>GENSCAN00000022971	Homo sapiens mesothelin (MSLN), transcript variant 1, mRNA.<br>Homo sapiens mesothelin (MSLN), transcript variant 2, mRNA.<br>Homo sapiens mesothelin/megakaryocyte potentiating factor mRNA, partial cds.<br>Human mesothelin or CAK1 antigen precursor mRNA, complete cds.<br><br><br><br>cdna:known chromosome:NCBI36:16:752638:758866:1 gene:ENSG00000102854<br>cdna:known chromosome:NCBI36:16:752638:758866:1 gene:ENSG00000102854<br>cdna:Genscan chromosome:NCBI36:16:752682:758734:1
3643580	825498<br>825499<br>825500<br>825501<br>825502<br>825503<br>825504<br>825505<br>825506<br>825507<br>825508<br>825509<br>825510<br>825511<br>825512<br>825513	3643581<br>3643582<br>3643583<br>3643584<br>3643585<br>3643586<br>3643587<br>3643588<br>3643589<br>3643590<br>3643591<br>3643592<br>3643593<br>3643594<br>3643595<br>3643596	extended<br>full<br>full<br>full<br>full<br>full<br>full<br>full<br>full<br>extended<br>core<br>core<br>core<br>core<br>extended<br>extended	16	1105774	1211977	+	NM_001005407<br>NM_021098<br>ENSESTT00000030137<br>ENSESTT00000030138<br>ENSESTT00000030139<br>ENSESTT00000030140<br>ENSESTT00000030141<br>ENST00000358590<br>ENST00000356546<br>GENSCAN00000065760<br>GENSCAN00000065756<br>GENSCAN00000065769<br>GENSCAN00000002163	Homo sapiens calcium channel, voltage-dependent, alpha 1H subunit (CACNA1H), transcript variant 2, mRNA.<br>Homo sapiens calcium channel, voltage-dependent, alpha 1H subunit (CACNA1H), transcript variant 1, mRNA.<br><br><br><br><br><br>cdna:known chromosome:NCBI36:16:1143739:1211772:1 gene:ENSG00000196557<br>cdna:known chromosome:NCBI36:16:1143739:1211772:1 gene:ENSG00000196557<br>cdna:Genscan chromosome:NCBI36:16:1130990:1131400:1<br>cdna:Genscan chromosome:NCBI36:16:1135479:1146660:1<br>cdna:Genscan chromosome:NCBI36:16:1183205:1210995:1<br>cdna:Genscan chromosome:NCBI36:16:1103204:1115253:1

	825514	3643597	full						
	825515	3643598	full						
	825516	3643599	extended						
	825517	3643600	extended						
	825518	3643601	full						
	825519	3643602	full						
	825520	3643603	full						
	825521	3643604	full						
	825522	3643605	core						
	825523	3643606	core						
	825524	3643607	core						
	825525	3643608	core						
	825526	3643609	full						
	825527	3643610	core						
	825528	3643611	extended						
	825529	3643612	full						
	825530	3643613	core						
	825531	3643614	full						
	825532	3643615	core						
	825533	3643616	core						
	825534	3643617	core						
	825535	3643618	core						
	825536	3643619	core						
	825537	3643620	core						
	825538	3643621	core						
	825539	3643622	core						
	825540	3643623	core						
	825541	3643624	core						
	825542	3643625	full						
	825543	3643626	core						
	825544	3643627	core						
	825545	3643628	core						
	825546	3643629	core						
	825547	3643630	core						
	825548	3643631	core						
	825549	3643632	core						
	825550	3643633	core						
	825551	3643634	core						
	825552	3643635	full						
	825553	3643636	core						
		3643637	core						
		3643638	core						
		3643639	core						
		3643640	full						
		3643641	core						
		3643642	core						
		3643643	core						
		3643644	core						
		3643645	full						
		3643646	core						
		3643647	core						
		3643648	core						
		3643649	core						
		3643650	core						

		3643651	core						
		3643652	core						
		3643653	core						
		3643654	core						
		3643655	core						
		3643656	core						
		3643657	extended						
		3643658	extended						
3643966	825730	3643967	extended	16	1602398	1692068	+	NM_144570	Homo sapiens chromosome 16 open reading frame 34 (C16orf34), mRNA.
	825731	3643968	extended					NM_020825	Homo sapiens Crm, cramped-like (Drosophila) (CRAMP1L), mRNA.
	825732	3643969	extended					AK095591	Homo sapiens cDNA FLJ38272 fis, clone FCBBF3002818.
	825733	3643970	extended					AK097575	Homo sapiens cDNA FLJ40256 fis, clone TESTI2025229.
	825734	3643971	extended					AL833832	Homo sapiens mRNA; cDNA DKFZp434O1216 (from clone DKFZp434O1216).
	825735	3643972	extended					ENSESTT00000030164	
	825736	3643973	full					ENSESTT00000030165	
	825737	3643974	extended					ENSESTT00000030166	
	825738	3643975	full					ENSESTT00000030167	
	825739	3643976	full					ENSESTT00000030168	
	825740	3643977	extended					ENSESTT00000030169	
	825741	3643978	extended					ENSESTT00000030170	
	825742	3643979	extended					ENSESTT00000030171	
	825743	3643980	extended					ENSESTT00000030172	
	825744	3643981	core					ENST00000262317	cdna:known-ccds chromosome:NCBI36:16:1645914:1667910:1 gene:ENSG00000007545 CCDS10440.1
	825745	3643982	extended					ENST00000248098	cdna:known-ccds chromosome:NCBI36:16:1668279:1692073:1 gene:ENSG00000206053 CCDS10441.1
	825746	3643983	core					ENST00000293925	cdna:known chromosome:NCBI36:16:1603974:1667910:1 gene:ENSG00000007545
	825747	3643984	extended					ENST00000359804	cdna:known chromosome:NCBI36:16:1604902:1667910:1 gene:ENSG00000007545
	825748	3643985	extended					ENST00000382711	cdna:known chromosome:NCBI36:16:1668279:1692073:1 gene:ENSG00000206053
	825749	3643986	extended					ENST00000382710	cdna:known chromosome:NCBI36:16:1668279:1682447:1 gene:ENSG00000206053
	825750	3643987	extended					GENSCAN00000052352	cdna:Genscan chromosome:NCBI36:16:1658835:1664047:1
	825751	3643988	extended					GENSCAN00000052355	cdna:Genscan chromosome:NCBI36:16:1668210:1687862:1
	825752	3643989	extended					GENSCAN00000005653	cdna:Genscan chromosome:NCBI36:16:1627826:1658195:1
	825753	3643990	extended					GENSCAN00000062408	cdna:Genscan chromosome:NCBI36:16:1688813:1689000:1
	825754	3643991	core					GENSCAN00000024476	cdna:Genscan chromosome:NCBI36:16:1603974:1623509:1
	825755	3643992	core						
	825756	3643993	extended						
	825757	3643994	extended						
	825758	3643995	core						
	825759	3643996	extended						
	825760	3643997	core						
	825761	3643998	core						
	825762	3643999	extended						
	825763	3644000	extended						
	825764	3644001	extended						
	825765	3644002	extended						
	825766	3644003	core						
	825767	3644004	core						
	825768	3644005	core						
	825769	3644006	core						
	825770	3644007	extended						
	825771	3644008	core						
	825772	3644009	core						
	825773	3644010	extended						
	825774	3644011	core						
	825775	3644012	extended						

	825776<br>825777<br>825778<br>825779<br>825780<br>825781<br>825782	3644013<br>3644014<br>3644015<br>3644016<br>3644017<br>3644018<br>3644019<br>3644020<br>3644021<br>3644022<br>3644023<br>3644024<br>3644025<br>3644026<br>3644027<br>3644028<br>3644029<br>3644030<br>3644031<br>3644032<br>3644033<br>3644034<br>3644035<br>3644036<br>3644037<br>3644038<br>3644039<br>3644040<br>3644041<br>3644042<br>3644043<br>3644044<br>3644045<br>3644046<br>3644047<br>3644048<br>3644049<br>3644050<br>3644051<br>3644052	extended<br>core<br>extended<br>extended<br>core<br>core<br>full<br>core<br>core<br>core<br>full<br>core<br>core<br>full<br>extended<br>core<br>core<br>extended<br>extended<br>full<br>core<br>core<br>core<br>extended<br>core<br>extended<br>extended<br>core<br>extended<br>extended<br>extended<br>extended<br>extended<br>extended<br>core<br>core<br>core<br>core<br>core<br>core						
3644541	826056<br>826057<br>826058<br>826059<br>826060<br>826061<br>826062<br>826063<br>826064<br>826065<br>826066<br>826067<br>826068<br>826069	3644542<br>3644543<br>3644544<br>3644545<br>3644546<br>3644547<br>3644548<br>3644549<br>3644550<br>3644551<br>3644552<br>3644553<br>3644554<br>3644555	full<br>full<br>full<br>core<br>core<br>core<br>core<br>core<br>extended<br>extended<br>full<br>full<br>core<br>core	16	2148314	2168125	+	NM_206835<br>NM_032271<br>ENSESTT00000027991<br>ENSESTT00000027993<br>ENSESTT00000027995<br>ENSESTT00000027996<br>ENST00000326181<br>ENST00000357892<br>GENSCAN00000008762	Homo sapiens TNF receptor-associated factor 7 (TRAF7), transcript variant 2, mRNA.<br>Homo sapiens TNF receptor-associated factor 7 (TRAF7), transcript variant 1, mRNA.<br><br><br><br><br>cdna:known-ccds chromosome:NCBI36:16:2145800:2168130:1 gene:ENSG00000131653 CCDS10461.1<br>cdna:novel chromosome:NCBI36:16:2145800:2168130:1 gene:ENSG00000131653<br>cdna:Genscan chromosome:NCBI36:16:2138650:2167960:1

	826070<br>826071<br>826072<br>826073<br>826074<br>826075<br>826076<br>826077<br>826078<br>826079<br>826080<br>826081<br>826082<br>826083<br>826084	3644556<br>3644557<br>3644558<br>3644559<br>3644560<br>3644561<br>3644562<br>3644563<br>3644564<br>3644565<br>3644566<br>3644567<br>3644568<br>3644569<br>3644570<br>3644571<br>3644572<br>3644573<br>3644574<br>3644575<br>3644576<br>3644577<br>3644578<br>3644579<br>3644580<br>3644581<br>3644582<br>3644583<br>3644584<br>3644585<br>3644586<br>3644587<br>3644588<br>3644589<br>3644590	core<br>core<br>core<br>core<br>extended<br>core<br>core<br>extended<br>core<br>extended<br>core<br>extended<br>extended<br>extended<br>extended<br>core<br>extended<br>extended<br>core<br>extended<br>core<br>extended<br>core<br>extended<br>core<br>full<br>core<br>full<br>core<br>core<br>core<br>core<br>core<br>core<br>core						
3651152	830107<br>830108<br>830109<br>830110<br>830111<br>830112<br>830113<br>830114<br>830115<br>830116<br>830117<br>830118<br>830119<br>830120<br>830121<br>830122<br>830123<br>830124<br>830125	3651153<br>3651154<br>3651155<br>3651156<br>3651157<br>3651158<br>3651159<br>3651160<br>3651161<br>3651162<br>3651163<br>3651164<br>3651165<br>3651166<br>3651167<br>3651168<br>3651169<br>3651170<br>3651171	core<br>core<br>core<br>extended<br>full<br>full<br>core<br>full<br>core<br>extended<br>core<br>extended<br>extended<br>extended<br>full<br>extended<br>extended<br>extended<br>extended	16	19635279	19776576	+	NM_153208<br>AK000122<br>AK093894<br>BC034823<br>CR605335<br>ENSESTT00000027145<br>ENSESTT00000027146<br>ENSESTT00000027147<br>ENSESTT00000027148<br>ENSESTT00000027149<br>ENST00000320394<br>ENST00000308214<br>GENSCAN00000057934	Homo sapiens IQ motif containing K (IQCK), mRNA.<br>Homo sapiens cDNA FLJ20115 fis, clone COL05594.<br>Homo sapiens cDNA FLJ36575 fis, clone TRACH2012387.<br>Homo sapiens IQ motif containing K, mRNA (cDNA clone MGC:35048 IMAGE:5165639), complete cds.<br>full-length cDNA clone CS0DI075YB15 of Placenta Cot 25-normalized of Homo sapiens (human).<br><br><br><br><br><br>cdna:known-ccds chromosome:NCBI36:16:19635279:19776358:1 gene:ENSG00000174628 CCDS10580.1<br>cdna:known chromosome:NCBI36:16:19635279:19776358:1 gene:ENSG00000174628<br>cdna:Genscan chromosome:NCBI36:16:19710745:19775371:1

	830126<br>830127<br>830128<br>830129<br>830130<br>830131<br>830132<br>830133<br>830134<br>830135<br>830136	3651172<br>3651173<br>3651174<br>3651175<br>3651176<br>3651177<br>3651178<br>3651179<br>3651180<br>3651181<br>3651182<br>3651183<br>3651184<br>3651185<br>3651186<br>3651187<br>3651188<br>3651189<br>3651190<br>3651191<br>3651192<br>3651193	core<br>extended<br>core<br>extended<br>extended<br>core<br>extended<br>extended<br>full<br>full<br>extended<br>extended<br>core<br>full<br>extended<br>extended<br>core<br>core<br>core<br>core<br>extended<br>extended						
3652902	831078<br>831079<br>831080<br>831081<br>831082<br>831083<br>831084<br>831085<br>831086<br>831087<br>831088<br>831089<br>831090<br>831091<br>831092<br>831093<br>831094<br>831095<br>831096<br>831097<br>831098<br>831099<br>831100<br>831101<br>831102<br>831103<br>831104<br>831105<br>831106<br>831107	3652903<br>3652904<br>3652905<br>3652906<br>3652907<br>3652908<br>3652909<br>3652910<br>3652911<br>3652912<br>3652913<br>3652914<br>3652915<br>3652916<br>3652917<br>3652918<br>3652919<br>3652920<br>3652921<br>3652922<br>3652923<br>3652924<br>3652925<br>3652926<br>3652927<br>3652928<br>3652929<br>3652930<br>3652931<br>3652932<br>3652933<br>3652934	full<br>extended<br>full<br>full<br>core<br>extended<br>extended<br>full<br>full<br>full<br>extended<br>full<br>full<br>full<br>full<br>full<br>full<br>full<br>core<br>core<br>extended<br>core<br>extended<br>core<br>core<br>full<br>full<br>full<br>core<br>full<br>core<br>core	16	23173592	23300115	+	NM_000336<br>AF260226<br>ENSESTT00000039379<br>ENSESTT00000039381<br>ENSESTT00000039382<br>ENSESTT00000039383<br>ENSESTT00000039384<br>ENST00000343070<br>ENST00000307331<br>GENSCAN00000045128<br>GENSCAN00000045129	Homo sapiens sodium channel, nonvoltage-gated 1, beta (Liddle syndrome) (SCNN1B), mRNA.<br>Homo sapiens epithelial sodium channel beta subunit (SCNN1B) mRNA, partial cds.<br><br><br><br><br><br>cdna:known-ccds chromosome:NCBI36:16:23221133:23300120:1 gene:ENSG00000168447 CCDS10609.1<br>cdna:known chromosome:NCBI36:16:23222822:23300120:1 gene:ENSG00000168447<br>cdna:Genscan chromosome:NCBI36:16:23173592:23253075:1<br>cdna:Genscan chromosome:NCBI36:16:23267422:23286781:1

		3652935<br>3652936<br>3652937<br>3652938<br>3652939<br>3652940<br>3652941<br>3652942<br>3652943<br>3652944<br>3652945<br>3652946	extended<br>extended<br>extended<br>core<br>extended<br>core<br>core<br>full<br>core<br>core<br>core<br>core						
3653123	831211<br>831212<br>831213<br>831214<br>831215<br>831216<br>831217<br>831218<br>831219<br>831220<br>831221<br>831222<br>831223<br>831224<br>831225<br>831226<br>831227<br>831228<br>831229<br>831230<br>831231<br>831232<br>831233<br>831234<br>831235<br>831236<br>831237<br>831238<br>831239<br>831240<br>831241<br>831242<br>831243<br>831244<br>831245<br>831246<br>831247<br>831248<br>831249<br>831250<br>831251<br>831252	3653124<br>3653125<br>3653126<br>3653127<br>3653128<br>3653129<br>3653130<br>3653131<br>3653132<br>3653133<br>3653134<br>3653135<br>3653136<br>3653137<br>3653138<br>3653139<br>3653140<br>3653141<br>3653142<br>3653143<br>3653144<br>3653145<br>3653146<br>3653147<br>3653148<br>3653149<br>3653150<br>3653151<br>3653152<br>3653153<br>3653154<br>3653155<br>3653156<br>3653157<br>3653158<br>3653159<br>3653160<br>3653161<br>3653162<br>3653163<br>3653164<br>3653165	extended<br>extended<br>core<br>core<br>core<br>full<br>extended<br>extended<br>extended<br>extended<br>extended<br>extended<br>extended<br>extended<br>extended<br>extended<br>extended<br>full<br>extended<br>extended<br>full<br>extended<br>extended<br>full<br>full<br>full<br>extended<br>full<br>full<br>full<br>full<br>extended<br>extended<br>extended<br>extended<br>full<br>full<br>full<br>full<br>full<br>extended<br>full	16	23754493	24139426	+	NM_212535<br>NM_002738<br>AJ002788<br>AL833252<br>ENSESTT00000008904<br>ENSESTT00000008905<br>ENSESTT00000039398<br>ENSESTT00000039399<br>ENSESTT00000039400<br>ENST00000303531<br>ENST00000321728<br>GENSCAN00000040078<br>GENSCAN00000060382	Homo sapiens protein kinase C, beta 1 (PRKCB1), transcript variant 1, mRNA.<br>Homo sapiens protein kinase C, beta 1 (PRKCB1), transcript variant 2, mRNA.<br>Homo sapiens mRNA for protein kinase C beta 1, 3' UTR; fetal brain cDNA ICRFp507K04156.<br>Homo sapiens mRNA; cDNA DKFZp761J0720 (from clone DKFZp761J0720).<br><br><br><br><br><br>cdna:known-ccds chromosome:NCBI36:16:23754823:24134803:1 gene:ENSG00000166501 CCDS10619.1<br>cdna:known-ccds chromosome:NCBI36:16:23754861:24139358:1 gene:ENSG00000166501 CCDS10618.1<br>cdna:Genscan chromosome:NCBI36:16:24011474:24130942:1<br>cdna:Genscan chromosome:NCBI36:16:23754998:23918175:1

	831253	3653166	extended						
	831254	3653167	core						
	831255	3653168	full						
	831256	3653169	extended						
	831257	3653170	full						
	831258	3653171	extended						
	831259	3653172	full						
	831260	3653173	extended						
	831261	3653174	extended						
	831262	3653175	full						
	831263	3653176	full						
	831264	3653177	extended						
	831265	3653178	extended						
	831266	3653179	core						
	831267	3653180	full						
	831268	3653181	core						
	831269	3653182	extended						
	831270	3653183	full						
	831271	3653184	full						
	831272	3653185	extended						
	831273	3653186	full						
	831274	3653187	extended						
	831275	3653188	full						
	831276	3653189	full						
	831277	3653190	core						
	831278	3653191	core						
	831279	3653192	extended						
	831280	3653193	extended						
	831281	3653194	extended						
	831282	3653195	core						
	831283	3653196	extended						
	831284	3653197	core						
	831285	3653198	full						
	831286	3653199	full						
	831287	3653200	full						
	831288	3653201	full						
	831289	3653202	full						
	831290	3653203	core						
	831291	3653204	extended						
	831292	3653205	full						
	831293	3653206	full						
	831294	3653207	full						
	831295	3653208	full						
	831296	3653209	extended						
	831297	3653210	extended						
	831298	3653211	full						
	831299	3653212	core						
	831300	3653213	core						
		3653214	extended						
		3653215	extended						
		3653216	core						
		3653217	core						
		3653218	core						
		3653219	full						

		3653220<br>3653221<br>3653222<br>3653223<br>3653224<br>3653225<br>3653226<br>3653227<br>3653228<br>3653229<br>3653230<br>3653231<br>3653232<br>3653233<br>3653234<br>3653235<br>3653236<br>3653237<br>3653238<br>3653239<br>3653240<br>3653241<br>3653242<br>3653243	extended<br>full<br>core<br>extended<br>extended<br>extended<br>full<br>extended<br>extended<br>extended<br>full<br>extended<br>extended<br>core<br>core<br>core<br>core<br>extended<br>extended<br>extended<br>extended<br>extended<br>core<br>core						
3653677	831560<br>831561<br>831562<br>831563<br>831564<br>831565<br>831566<br>831567<br>831568<br>831569	3653678<br>3653679<br>3653680<br>3653681<br>3653682<br>3653683<br>3653684<br>3653685<br>3653686<br>3653687<br>3653688<br>3653689<br>3653690<br>3653691	full<br>full<br>core<br>extended<br>core<br>core<br>core<br>core<br>core<br>core<br>core<br>core<br>core<br>core	16	25130677	25147754	+	NM_001169<br>BC040630<br>ENSESTT00000042207<br>ENST00000219660	Homo sapiens aquaporin 8 (AQP8), mRNA.<br>Homo sapiens aquaporin 8, mRNA (cDNA clone MGC:50607 IMAGE:5759105), complete cds.<br><br>cdna:known-ccds chromosome:NCBI36:16:25135784:25147752:1 gene:ENSG00000103375 CCDS10626.1
3658980	834574<br>834575<br>834576<br>834577<br>834578<br>834579<br>834580<br>834581<br>834582<br>834583<br>834584<br>834585<br>834586<br>834587<br>834588	3658981<br>3658982<br>3658983<br>3658984<br>3658985<br>3658986<br>3658987<br>3658988<br>3658989<br>3658990<br>3658991<br>3658992<br>3658993<br>3658994<br>3658995	full<br>full<br>full<br>full<br>full<br>full<br>core<br>extended<br>core<br>extended<br>extended<br>extended<br>extended<br>full<br>extended	16	45472454	45522696	+	NM_133443<br>AK024372<br>AK094971<br>AL833351<br>ENSESTT00000019659<br>ENST00000340124	Homo sapiens glutamic pyruvate transaminase (alanine aminotransferase) 2 (GPT2), mRNA.<br>Homo sapiens cDNA FLJ14310 fis, clone PLACE3000271.<br>Homo sapiens cDNA FLJ37652 fis, clone BRHIP2009685, moderately similar to ALANINE AMINOTRANSFERASE (EC 2.6.1.2).<br>Homo sapiens mRNA; cDNA DKFZp686D1635 (from clone DKFZp686D1635).<br><br>cdna:known-ccds chromosome:NCBI36:16:45475809:45522699:1 gene:ENSG00000166123 CCDS10725.1

	834589<br>834590<br>834591<br>834592<br>834593<br>834594<br>834595<br>834596<br>834597<br>834598<br>834599<br>834600<br>834601<br>834602<br>834603<br>834604<br>834605<br>834606<br>834607<br>834608	3658996<br>3658997<br>3658998<br>3658999<br>3659000<br>3659001<br>3659002<br>3659003<br>3659004<br>3659005<br>3659006<br>3659007<br>3659008<br>3659009<br>3659010<br>3659011<br>3659012<br>3659013<br>3659014<br>3659015<br>3659016<br>3659017<br>3659018<br>3659019<br>3659020<br>3659021<br>3659022<br>3659023<br>3659024<br>3659025<br>3659026<br>3659027<br>3659028<br>3659029<br>3659030<br>3659031<br>3659032<br>3659033<br>3659034<br>3659035<br>3659036<br>3659037<br>3659038<br>3659039	core<br>full<br>full<br>core<br>full<br>full<br>full<br>full<br>core<br>full<br>core<br>core<br>core<br>full<br>full<br>full<br>full<br>extended<br>extended<br>core<br>full<br>full<br>full<br>full<br>full<br>full<br>full<br>core<br>full<br>extended<br>core<br>core<br>extended<br>core<br>extended<br>core<br>extended<br>extended<br>core<br>core<br>core<br>core<br>core<br>core						
3662808	837007<br>837008<br>837009<br>837010<br>837011<br>837012<br>837013<br>837014<br>837015<br>837016	3662809<br>3662810<br>3662811<br>3662812<br>3662813<br>3662814<br>3662815<br>3662816<br>3662817<br>3662818	extended<br>extended<br>extended<br>extended<br>core<br>extended<br>full<br>full<br>full<br>full	16	56211168	56256552	+	NM_201525<br>NM_201524<br>NM_005682<br>AK131550<br>CR936747<br>BC013207<br>ENSESTT00000006099<br>ENSESTT00000006100<br>ENSESTT00000006101<br>ENSESTT00000006102	Homo sapiens G protein-coupled receptor 56 (GPR56), transcript variant 3, mRNA.<br>Homo sapiens G protein-coupled receptor 56 (GPR56), transcript variant 2, mRNA.<br>Homo sapiens G protein-coupled receptor 56 (GPR56), transcript variant 1, mRNA.<br>Homo sapiens cDNA FLJ16789 fis, clone PROST2010326, highly similar to Homo sapiens G protein-coupled receptor 56 (GPR56).<br>Homo sapiens mRNA; cDNA DKFZp781L1398 (from clone DKFZp781L1398).<br>Homo sapiens G protein-coupled receptor 56, mRNA (cDNA clone IMAGE:4157209).

	837017<br>837018<br>837019<br>837020<br>837021<br>837022<br>837023<br>837024<br>837025<br>837026<br>837027<br>837028<br>837029<br>837030<br>837031<br>837032<br>837033<br>837034<br>837035<br>837036<br>837037<br>837038	3662819<br>3662820<br>3662821<br>3662822<br>3662823<br>3662824<br>3662825<br>3662826<br>3662827<br>3662828<br>3662829<br>3662830<br>3662831<br>3662832<br>3662833<br>3662834<br>3662835<br>3662836<br>3662837<br>3662838<br>3662839<br>3662840<br>3662841<br>3662842<br>3662843<br>3662844<br>3662845<br>3662846<br>3662847<br>3662848	extended<br>core<br>full<br>full<br>extended<br>extended<br>extended<br>extended<br>extended<br>full<br>extended<br>core<br>core<br>full<br>core<br>core<br>extended<br>core<br>core<br>core<br>core<br>core<br>core<br>core<br>core<br>core<br>core<br>core<br>core<br>extended					ENSESTT00000006103<br>ENSESTT00000006104<br>ENSESTT00000006105<br>ENSESTT00000006106<br>ENST00000379694<br>ENST00000388815<br>ENST00000388814<br>ENST00000388813<br>ENST00000388812<br>ENST00000379696<br>GENSCAN00000035943	 <br><br><br><br>cdna:known chromosome:NCBI36:16:56241701:56256445:1 gene:ENSG00000205336<br>cdna:known chromosome:NCBI36:16:56241701:56254995:1 gene:ENSG00000205336<br>cdna:known chromosome:NCBI36:16:56241701:56254995:1 gene:ENSG00000205336<br>cdna:known chromosome:NCBI36:16:56241701:56254995:1 gene:ENSG00000205336<br>cdna:known chromosome:NCBI36:16:56241701:56254995:1 gene:ENSG00000205336<br>cdna:novel chromosome:NCBI36:16:56241701:56256445:1 gene:ENSG00000205336<br>cdna:Genscan chromosome:NCBI36:16:56241701:56254995:1
3664924	838247<br>838248<br>838249<br>838250<br>838251<br>838252<br>838253<br>838254<br>838255<br>838256<br>838257<br>838258<br>838259<br>838260<br>838261<br>838262<br>838263<br>838264<br>838265<br>838266<br>838267<br>838268<br>838269<br>838270	3664925<br>3664926<br>3664927<br>3664928<br>3664929<br>3664930<br>3664931<br>3664932<br>3664933<br>3664934<br>3664935<br>3664936<br>3664937<br>3664938<br>3664939<br>3664940<br>3664941<br>3664942<br>3664943<br>3664944<br>3664945<br>3664946<br>3664947<br>3664948	extended<br>extended<br>extended<br>extended<br>extended<br>full<br>full<br>full<br>core<br>core<br>core<br>core<br>core<br>full<br>core<br>core<br>core<br>core<br>core<br>core<br>core<br>full<br>full<br>full	16	65422552	65466169	+	NM_005182<br>NM_001014435<br>AY075019<br>ENSESTT00000041232<br>ENSESTT00000041233<br>ENSESTT00000041234<br>ENST00000338437<br>GENSCAN00000004945	Homo sapiens carbonic anhydrase VII (CA7), transcript variant 1, mRNA.<br>Homo sapiens carbonic anhydrase VII (CA7), transcript variant 2, mRNA.<br>Homo sapiens carbonic anhydrase VII mRNA, complete cds.<br><br><br><br>cdna:known-ccds chromosome:NCBI36:16:65435781:65445545:1 gene:ENSG00000168748 CCDS10821.1<br>cdna:Genscan chromosome:NCBI36:16:65434124:65444902:1

	838271	3664949<br>3664950<br>3664951	full<br>full<br>extended						
3664982	838286<br>838287<br>838288<br>838289<br>838290<br>838291<br>838292<br>838293<br>838294<br>838295<br>838296<br>838297<br>838298	3664983<br>3664984<br>3664985<br>3664986<br>3664987<br>3664988<br>3664989<br>3664990<br>3664991<br>3664992<br>3664993<br>3664994<br>3664995<br>3664996<br>3664997<br>3664998<br>3664999<br>3665000<br>3665001<br>3665002<br>3665003<br>3665004<br>3665005<br>3665006<br>3665007<br>3665008<br>3665009<br>3665010<br>3665011<br>3665012<br>3665013<br>3665014<br>3665015<br>3665016	core<br>core<br>core<br>core<br>core<br>core<br>core<br>core<br>extended<br>extended<br>extended<br>core<br>core<br>core<br>core<br>core<br>core<br>core<br>core<br>extended<br>core<br>extended<br>core<br>full<br>extended<br>core<br>core<br>core<br>core<br>extended<br>core<br>core<br>core<br>core	16	65525848	65536496	+	NM_003869<br>NM_198061<br>AK095522<br>Y09616<br>ENSESTT00000041236<br>ENSESTT00000041237<br>ENSESTT00000041238<br>ENSESTT00000041239<br>ENST00000317091<br>GENSCAN00000065159	Homo sapiens carboxylesterase 2 (intestine, liver) (CES2), transcript variant 1, mRNA.<br>Homo sapiens carboxylesterase 2 (intestine, liver) (CES2), transcript variant 2, mRNA.<br>Homo sapiens cDNA FLJ38203 fis, clone FCBBF1000403, highly similar to Human carboxylesterase (hCE-2) mRNA.<br>H.sapiens mRNA for putative carboxylesterase.<br><br><br><br><br>cdna:known-ccds chromosome:NCBI36:16:65525848:65536473:1 gene:ENSG00000172831 CCDS10825.1<br>cdna:Genscan chromosome:NCBI36:16:65527040:65535429:1
3665029	838304<br>838305<br>838306<br>838307<br>838308<br>838309<br>838310<br>838311<br>838312<br>838313<br>838314<br>838315<br>838316	3665030<br>3665031<br>3665032<br>3665033<br>3665034<br>3665035<br>3665036<br>3665037<br>3665038<br>3665039<br>3665040<br>3665041<br>3665042<br>3665043<br>3665044<br>3665045	core<br>core<br>core<br>core<br>core<br>extended<br>core<br>core<br>core<br>core<br>extended<br>core<br>extended<br>core<br>core<br>core	16	65552637	65566553	+	NM_024922<br>AK097538<br>ENSESTT00000041240<br>ENSESTT00000041241<br>ENSESTT00000041242<br>ENSESTT00000041243<br>ENST00000303334<br>GENSCAN00000065163	Homo sapiens esterase 31 (FLJ21736), mRNA.<br>Homo sapiens cDNA FLJ40219 fis, clone TESTI2021637, moderately similar to LIVER CARBOXYLESTERASE PRECURSOR (EC 3.1.1.1).<br><br><br><br><br>cdna:known-ccds chromosome:NCBI36:16:65552639:65566552:1 gene:ENSG00000172828 CCDS10826.1<br>cdna:Genscan chromosome:NCBI36:16:65552712:65564453:1

		3665046<br>3665047<br>3665048	core<br>core<br>core						
3665501	838557<br>838558<br>838559<br>838560<br>838561<br>838562	3665502<br>3665503<br>3665504<br>3665505<br>3665506<br>3665507<br>3665508<br>3665509<br>3665510<br>3665511	full<br>core<br>core<br>core<br>core<br>core<br>core<br>core<br>core<br>core	16	66021931	66028948	+	NM_000196<br>ENSESTT00000035319<br>ENST00000326152<br>GENSCAN00000036844	Homo sapiens hydroxysteroid (11-beta) dehydrogenase 2 (HSD11B2), mRNA.<br><br>cdna:known-ccds chromosome:NCBI36:16:66022518:66028953:1 gene:ENSG00000176387 CCDS10837.1<br>cdna:Genscan chromosome:NCBI36:16:66022653:66028407:1
3666366	839053<br>839054<br>839055<br>839056<br>839057<br>839058<br>839059<br>839060<br>839061<br>839062<br>839063<br>839064<br>839065<br>839066<br>839067<br>839068<br>839069<br>839070<br>839071<br>839072<br>839073<br>839074<br>839075<br>839076	3666367<br>3666368<br>3666369<br>3666370<br>3666371<br>3666372<br>3666373<br>3666374<br>3666375<br>3666376<br>3666377<br>3666378<br>3666379<br>3666380<br>3666381<br>3666382<br>3666383<br>3666384<br>3666385<br>3666386<br>3666387<br>3666388<br>3666389<br>3666390<br>3666391<br>3666392<br>3666393<br>3666394<br>3666395<br>3666396<br>3666397<br>3666398<br>3666399<br>3666400<br>3666401<br>3666402	core<br>core<br>core<br>extended<br>full<br>extended<br>extended<br>full<br>extended<br>core<br>extended<br>extended<br>extended<br>core<br>core<br>core<br>full<br>core<br>core<br>core<br>core<br>core<br>core<br>core<br>extended<br>free<br>full<br>core<br>core<br>core<br>core<br>core<br>extended<br>extended<br>core<br>core	16	67236240	67290441	+	NM_001793<br>ENSESTT00000052546<br>ENSESTT00000052547<br>ENST00000264012<br>ENST00000379136<br>GENSCAN00000026981	Homo sapiens cadherin 3, type 1, P-cadherin (placental) (CDH3), mRNA.<br><br><br>cdna:known-ccds chromosome:NCBI36:16:67236240:67290440:1 gene:ENSG00000062038 CCDS10868.1<br>cdna:novel chromosome:NCBI36:16:67236240:67290440:1 gene:ENSG00000062038<br>cdna:Genscan chromosome:NCBI36:16:67267789:67289804:1
3674199	843975<br>843976<br>843977<br>843978	3674200<br>3674201<br>3674202<br>3674203	core<br>core<br>core<br>core	16	88169677	88191145	+	XM_942425<br>NM_014427<br>NM_153636<br>ENSESTT00000047135	PREDICTED: Homo sapiens similar to Copine-7 (Copine VII) (LOC652777), mRNA.<br>Homo sapiens copine VII (CPNE7), transcript variant 2, mRNA.<br>Homo sapiens copine VII (CPNE7), transcript variant 1, mRNA.

	843979<br>843980<br>843981<br>843982<br>843983<br>843984<br>843985<br>843986<br>843987<br>843988<br>843989<br>843990<br>843991<br>843992<br>843993<br>843994<br>843995<br>843996<br>843997<br>843998<br>843999<br>844000<br>844001	3674204<br>3674205<br>3674206<br>3674207<br>3674208<br>3674209<br>3674210<br>3674211<br>3674212<br>3674213<br>3674214<br>3674215<br>3674216<br>3674217<br>3674218<br>3674219<br>3674220<br>3674221<br>3674222<br>3674223<br>3674224<br>3674225<br>3674226<br>3674227<br>3674228<br>3674229<br>3674230<br>3674231<br>3674232<br>3674233<br>3674234<br>3674235<br>3674236<br>3674237<br>3674238<br>3674239	extended<br>extended<br>core<br>full<br>full<br>core<br>core<br>core<br>core<br>full<br>core<br>full<br>core<br>core<br>full<br>full<br>core<br>extended<br>extended<br>extended<br>core<br>full<br>full<br>core<br>core<br>core<br>extended<br>full<br>core<br>extended<br>extended<br>extended<br>core<br>core<br>core<br>core					ENST00000268720<br>ENST00000319518<br>GENSCAN00000003787<br>GENSCAN00000003788	cdna:known-ccds chromosome:NCBI36:16:88169677:88191155:1 gene:ENSG00000178773 CCDS10980.1<br>cdna:known-ccds chromosome:NCBI36:16:88169677:88191155:1 gene:ENSG00000178773 CCDS10981.1<br>cdna:Genscan chromosome:NCBI36:16:88177154:88179462:1<br>cdna:Genscan chromosome:NCBI36:16:88166838:88173838:1
3677752	845970<br>845971<br>845972<br>845973<br>845974<br>845975<br>845976<br>845977<br>845978<br>845979<br>845980<br>845981<br>845982<br>845983<br>845984<br>845985<br>845986<br>845987	3677753<br>3677754<br>3677755<br>3677756<br>3677757<br>3677758<br>3677759<br>3677760<br>3677761<br>3677762<br>3677763<br>3677764<br>3677765<br>3677766<br>3677767<br>3677768<br>3677769<br>3677770	extended<br>core<br>core<br>core<br>extended<br>core<br>core<br>core<br>core<br>core<br>extended<br>full<br>full<br>core<br>full<br>core<br>core<br>extended	16	3640638	3713275	-	NM_016292<br>AJ890085<br>BC035759<br>ENSESTT00000044828<br>ENSESTT00000044829<br>ENST00000246957<br>GENSCAN00000031977	Homo sapiens TNF receptor-associated protein 1 (TRAP1), mRNA.<br>Homo sapiens mRNA for TNF receptor-associated protein 1 (TRAP1 gene).<br>Homo sapiens cDNA clone IMAGE:5557641, partial cds.<br><br><br>cdna:known-ccds chromosome:NCBI36:16:3648040:3707521:-1 gene:ENSG00000126602 CCDS10508.1<br>cdna:Genscan chromosome:NCBI36:16:3648131:3707510:-1

	845988<br>845989<br>845990<br>845991<br>845992<br>845993<br>845994<br>845995<br>845996<br>845997<br>845998<br>845999<br>846000<br>846001	3677771<br>3677772<br>3677773<br>3677774<br>3677775<br>3677776<br>3677777<br>3677778<br>3677779<br>3677780<br>3677781<br>3677782<br>3677783<br>3677784<br>3677785<br>3677786<br>3677787<br>3677788<br>3677789<br>3677790	core<br>full<br>core<br>extended<br>core<br>extended<br>core<br>extended<br>extended<br>core<br>core<br>core<br>core<br>extended<br>extended<br>extended<br>full<br>core<br>extended<br>extended						
3682028	848602<br>848603<br>848604<br>848605<br>848606<br>848607<br>848608<br>848609<br>848610<br>848611<br>848612<br>848613<br>848614<br>848615<br>848616<br>848617<br>848618<br>848619<br>848620<br>848621<br>848622<br>848623<br>848624<br>848625<br>848626<br>848627<br>848628<br>848629<br>848630<br>848631<br>848632<br>848633<br>848634<br>848635	3682029<br>3682030<br>3682031<br>3682032<br>3682033<br>3682034<br>3682035<br>3682036<br>3682037<br>3682038<br>3682039<br>3682040<br>3682041<br>3682042<br>3682043<br>3682044<br>3682045<br>3682046<br>3682047<br>3682048<br>3682049<br>3682050<br>3682051<br>3682052<br>3682053<br>3682054<br>3682055<br>3682056<br>3682057<br>3682058<br>3682059<br>3682060<br>3682061<br>3682062	core<br>core<br>core<br>core<br>extended<br>core<br>core<br>full<br>core<br>full<br>full<br>full<br>core<br>core<br>core<br>core<br>core<br>core<br>core<br>full<br>core<br>core<br>extended<br>core<br>extended<br>core<br>extended<br>extended<br>core<br>extended<br>extended<br>extended<br>extended<br>core	16	15704506	15858425	-	NM_001040114<br>NM_002474<br>NM_022844<br>NM_001040113<br>AK125033<br>AK130413<br>BC031040<br>CR749516<br>ENSESTT00000003774<br>ENSESTT00000003775<br>ENSESTT00000003776<br>ENSESTT00000003777<br>ENSESTT00000003778<br>ENSESTT00000003779<br>ENSESTT00000003780<br>ENSESTT00000003781<br>ENSESTT00000003782<br>ENST00000338282<br>ENST00000300036<br>GENSCAN00000061168<br>GENSCAN00000058215	Homo sapiens myosin, heavy polypeptide 11, smooth muscle (MYH11), transcript variant SM1B, mRNA.<br>Homo sapiens myosin, heavy polypeptide 11, smooth muscle (MYH11), transcript variant SM1A, mRNA.<br>Homo sapiens myosin, heavy polypeptide 11, smooth muscle (MYH11), transcript variant SM2A, mRNA.<br>Homo sapiens myosin, heavy polypeptide 11, smooth muscle (MYH11), transcript variant SM2B, mRNA.<br>Homo sapiens cDNA FLJ43043 fis, clone BRTHA3003449, highly similar to MYOSIN HEAVY CHAIN, SMOOTH MUSCLE ISOFORM.<br>Homo sapiens cDNA FLJ26903 fis, clone RCT01362.<br>Homo sapiens myosin, heavy polypeptide 11, smooth muscle, mRNA (cDNA clone IMAGE:5272844), complete cds.<br>Homo sapiens mRNA; cDNA DKFZp686D10126 (from clone DKFZp686D10126).<br>cdna:known-ccds chromosome:NCBI36:16:15704495:15858369:-1 gene:ENSG00000133392 CCDS10566.1<br>cdna:known-ccds chromosome:NCBI36:16:15704495:15858369:-1 gene:ENSG00000133392 CCDS10565.1<br>cdna:Genscan chromosome:NCBI36:16:15834988:15889943:-1<br>cdna:Genscan chromosome:NCBI36:16:15705349:15705448:-1

	848636	3682063	extended						
	848637	3682064	extended						
	848638	3682065	extended						
	848639	3682066	core						
	848640	3682067	core						
	848641	3682068	core						
	848642	3682069	core						
	848643	3682070	core						
	848644	3682071	core						
	848645	3682072	core						
	848646	3682073	full						
	848647	3682074	core						
	848648	3682075	full						
	848649	3682076	core						
	848650	3682077	full						
	848651	3682078	core						
	848652	3682079	core						
	848653	3682080	core						
	848654	3682081	extended						
	848655	3682082	core						
	848656	3682083	core						
	848657	3682084	core						
	848658	3682085	full						
	848659	3682086	core						
	848660	3682087	full						
	848661	3682088	full						
	848662	3682089	extended						
	848663	3682090	full						
	848664	3682091	core						
	848665	3682092	core						
	848666	3682093	extended						
	848667	3682094	core						
	848668	3682095	extended						
	848669	3682096	extended						
	848670	3682097	extended						
	848671	3682098	extended						
		3682099	core						
		3682100	extended						
		3682101	extended						
		3682102	extended						
		3682103	core						
		3682104	extended						
		3682105	full						
		3682106	full						
		3682107	core						
		3682108	extended						
		3682109	core						
		3682110	extended						
		3682111	extended						
		3682112	extended						
		3682113	core						
		3682114	full						
		3682115	extended						
		3682116	extended						

		3682117<br>3682118<br>3682119<br>3682120<br>3682121<br>3682122<br>3682123<br>3682124<br>3682125<br>3682126<br>3682127<br>3682128<br>3682129<br>3682130<br>3682131<br>3682132	extended<br>core<br>full<br>full<br>full<br>core<br>full<br>extended<br>extended<br>extended<br>full<br>full<br>core<br>full<br>core<br>full						
3685329	850506<br>850507<br>850508<br>850509<br>850510<br>850511<br>850512<br>850513<br>850514<br>850515<br>850516<br>850517<br>850518<br>850519<br>850520<br>850521<br>850522	3685330<br>3685331<br>3685332<br>3685333<br>3685334<br>3685335<br>3685336<br>3685337<br>3685338<br>3685339<br>3685340<br>3685341<br>3685342<br>3685343<br>3685344<br>3685345<br>3685346<br>3685347<br>3685348<br>3685349<br>3685350<br>3685351<br>3685352<br>3685353<br>3685354<br>3685355<br>3685356<br>3685357<br>3685358<br>3685359	core<br>core<br>core<br>core<br>extended<br>core<br>extended<br>core<br>core<br>core<br>extended<br>core<br>extended<br>extended<br>core<br>core<br>core<br>core<br>core<br>core<br>core<br>core<br>core<br>full<br>core<br>core<br>extended<br>extended<br>core<br>extended	16	23521990	23560146	-	NM_024675<br>ENSESTT00000039401<br>ENSESTT00000039402<br>ENST00000261584<br>ENST00000388994<br>ENST00000388993<br>GENSCAN00000033635	Homo sapiens hypothetical protein FLJ21816 (FLJ21816), mRNA.<br><br><br>cdna:known chromosome:NCBI36:16:23521984:23560128:-1 gene:ENSG00000083093<br>cdna:known chromosome:NCBI36:16:23522281:23559979:-1 gene:ENSG00000083093<br>cdna:known chromosome:NCBI36:16:23522281:23559979:-1 gene:ENSG00000083093<br>cdna:Genscan chromosome:NCBI36:16:23501106:23559127:-1
3694657	856159<br>856160<br>856161<br>856162<br>856163<br>856164<br>856165	3694658<br>3694659<br>3694660<br>3694661<br>3694662<br>3694663<br>3694664	extended<br>extended<br>extended<br>extended<br>extended<br>extended<br>core	16	63535165	63713893	-	NM_001797<br>AK021537<br>ENSESTT00000019933<br>ENSESTT00000019934<br>ENSESTT00000019935<br>ENSESTT00000019936<br>ENST00000268603	Homo sapiens cadherin 11, type 2, OB-cadherin (osteoblast) (CDH11), mRNA.<br>Homo sapiens cDNA FLJ11475 fis, clone HEMBA1001734, moderately similar to CADHERIN-11 PRECURSOR.<br><br><br><br><br>cdna:known-ccds chromosome:NCBI36:16:63538186:63713431:-1 gene:ENSG00000140937 CCDS10803.1

	856166	3694665	core					ENST00000268602	cdna:known chromosome:NCBI36:16:63538186:63713431:-1 gene:ENSG00000140937
	856167	3694666	core					GENSCAN00000012275	cdna:Genscan chromosome:NCBI36:16:63619026:63684171:-1
	856168	3694667	core					GENSCAN00000049842	cdna:Genscan chromosome:NCBI36:16:63539007:63596273:-1
	856169	3694668	core						
	856170	3694669	full						
	856171	3694670	full						
	856172	3694671	full						
	856173	3694672	extended						
	856174	3694673	extended						
	856175	3694674	extended						
	856176	3694675	extended						
	856177	3694676	core						
	856178	3694677	core						
	856179	3694678	full						
	856180	3694679	core						
	856181	3694680	full						
	856182	3694681	extended						
	856183	3694682	extended						
	856184	3694683	full						
	856185	3694684	core						
	856186	3694685	extended						
	856187	3694686	extended						
	856188	3694687	extended						
	856189	3694688	extended						
	856190	3694689	extended						
	856191	3694690	extended						
	856192	3694691	core						
	856193	3694692	extended						
	856194	3694693	core						
	856195	3694694	core						
	856196	3694695	extended						
	856197	3694696	full						
	856198	3694697	core						
	856199	3694698	core						
	856200	3694699	full						
	856201	3694700	full						
	856202	3694701	full						
	856203	3694702	core						
	856204	3694703	core						
	856205	3694704	core						
	856206	3694705	core						
	856207	3694706	core						
	856208	3694707	extended						
	856209	3694708	extended						
	856210	3694709	extended						
	856211	3694710	extended						
	856212	3694711	extended						
	856213	3694712	extended						
	856214	3694713	full						
	856215	3694714	full						
	856216	3694715	full						
	856217	3694716	full						
	856218	3694717	full						
		3694718	full						

		3694719	extended						
		3694720	full						
		3694721	full						
		3694722	full						
		3694723	full						
		3694724	extended						
		3694725	full						
		3694726	full						
		3694727	core						
		3694728	core						
		3694729	full						
		3694730	full						
		3694731	full						
		3694732	full						
		3694733	full						
		3694734	full						
		3694735	full						
		3694736	full						
		3694737	full						
		3694738	full						
		3694739	full						
		3694740	full						
		3694741	extended						
		3694742	full						
		3694743	full						
		3694744	full						
		3694745	extended						
		3694746	extended						
		3694747	extended						
		3694748	extended						
		3694749	extended						
		3694750	extended						
		3694751	extended						
		3694752	full						
		3694753	extended						
		3694754	core						
		3694755	extended						
3699634	859168	3699635	extended	16	74090507	74147659	-	NM_012126	Homo sapiens carbohydrate (N-acetylglucosamine 6-O) sulfotransferase 5 (CHST5), transcript variant 1, mRNA.
	859169	3699636	extended					NM_024533	Homo sapiens carbohydrate (N-acetylglucosamine 6-O) sulfotransferase 5 (CHST5), transcript variant 2, mRNA.
	859170	3699637	extended					XM_928789	PREDICTED: Homo sapiens similar to carbohydrate (N-acetylglucosamine 6-O) sulfotransferase 5 (LOC645799), mRNA.
	859171	3699638	core					XM_937064	PREDICTED: Homo sapiens similar to carbohydrate (N-acetylglucosamine 6-O) sulfotransferase 5 (LOC648009), mRNA.
	859172	3699639	core					AK057689	Homo sapiens cDNA FLJ33127 fis, clone TRACH2001592.
	859173	3699640	core					AK096650	Homo sapiens cDNA FLJ39331 fis, clone OCBBF2016467.
	859174	3699641	core					BC063677	Homo sapiens carbohydrate (N-acetylglucosamine 6-O) sulfotransferase 5, mRNA (cDNA clone IMAGE:4363402), complete cds.
	859175	3699642	core					ENSESTT00000038327	
	859176	3699643	core					ENSESTT00000038328	
	859177	3699644	full					ENSESTT00000038329	
	859178	3699645	full					ENSESTT00000038330	
	859179	3699646	full					ENSESTT00000038331	
	859180	3699647	core					ENSESTT00000038332	
	859181	3699648	extended					ENST00000336257	cdna:known-ccds chromosome:NCBI36:16:74119934:74126569:-1 gene:ENSG00000135702 CCDS10919.1
	859182	3699649	full					ENST00000258173	cdna:novel chromosome:NCBI36:16:74129555:74147667:-1 gene:ENSG00000205084
	859183	3699650	full					GENSCAN00000036002	cdna:Genscan chromosome:NCBI36:16:74087123:74100000:-1
	859184	3699651	extended					GENSCAN00000036004	cdna:Genscan chromosome:NCBI36:16:74120548:74121777:-1

	859185<br>859186<br>859187<br>859188<br>859189<br>859190<br>859191	3699652<br>3699653<br>3699654<br>3699655<br>3699656<br>3699657<br>3699658<br>3699659<br>3699660<br>3699661<br>3699662<br>3699663<br>3699664<br>3699665<br>3699666<br>3699667<br>3699668<br>3699669<br>3699670<br>3699671<br>3699672<br>3699673<br>3699674<br>3699675<br>3699676	extended<br>core<br>core<br>core<br>core<br>extended<br>core<br>extended<br>extended<br>full<br>extended<br>core<br>extended<br>extended<br>core<br>core<br>core<br>core<br>core<br>extended<br>full<br>core<br>extended<br>core<br>core					GENSCAN00000035998	cdna:Genscan chromosome:NCBI36:16:74130720:74134082:-1
3703885	861799<br>861800<br>861801<br>861802<br>861803<br>861804<br>861805<br>861806<br>861807<br>861808<br>861809<br>861810<br>861811<br>861812<br>861813<br>861814<br>861815<br>861816<br>861817<br>861818<br>861819<br>861820<br>861821<br>861822<br>861823<br>861824<br>861825<br>861826<br>861827	3703886<br>3703887<br>3703888<br>3703889<br>3703890<br>3703891<br>3703892<br>3703893<br>3703894<br>3703895<br>3703896<br>3703897<br>3703898<br>3703899<br>3703900<br>3703901<br>3703902<br>3703903<br>3703904<br>3703905<br>3703906<br>3703907<br>3703908<br>3703909<br>3703910<br>3703911<br>3703912<br>3703913<br>3703914	core<br>core<br>core<br>core<br>core<br>core<br>core<br>core<br>core<br>full<br>full<br>core<br>full<br>full<br>core<br>core<br>core<br>core<br>core<br>extended<br>core<br>full<br>extended<br>extended<br>full<br>full<br>full<br>full<br>full	16	86421130	86460615	-	NR_002593<br>NM_003486<br>CR936746<br>ENSESTT00000011243<br>ENSESTT00000011245<br>ENSESTT00000039076<br>ENST00000261622<br>ENST00000357918<br>GENSCAN00000067992<br>GENSCAN00000037705	Homo sapiens SLC7A5 pseudogene (LAT1-3TM) on chromosome 16.<br>Homo sapiens solute carrier family 7 (cationic amino acid transporter, y+ system), member 5 (SLC7A5), mRNA.<br>Homo sapiens mRNA; cDNA DKFZp686N23106 (from clone DKFZp686N23106).<br><br><br><br>cdna:known-ccds chromosome:NCBI36:16:86421131:86460615:-1 gene:ENSG00000103257 CCDS10964.1<br>cdna:known chromosome:NCBI36:16:29531923:29532462:-1 gene:ENSG00000197017<br>cdna:Genscan chromosome:NCBI36:16:86422427:86460529:-1<br>cdna:Genscan chromosome:NCBI36:16:21438522:21439112:-1

	861828<br>861829<br>861830<br>861831	3703915<br>3703916<br>3703917<br>3703918<br>3703919<br>3703920<br>3703921<br>3703922<br>3703923<br>3703924<br>3703925<br>3703926<br>3703927<br>3703928<br>3703929<br>3703930<br>3703931<br>3703932<br>3703933<br>3703934	full<br>core<br>core<br>full<br>full<br>full<br>full<br>full<br>full<br>full<br>full<br>full<br>full<br>full<br>full<br>full<br>core<br>full<br>core<br>core						
3710108	865369<br>865370<br>865371<br>865372<br>865373<br>865374<br>865375<br>865376<br>865377<br>865378<br>865379<br>865380<br>865381<br>865382<br>865383<br>865384<br>865385<br>865386<br>865387<br>865388<br>865389<br>865390<br>865391<br>865392<br>865393<br>865394<br>865395<br>865396<br>865397<br>865398<br>865399	3710109<br>3710110<br>3710111<br>3710112<br>3710113<br>3710114<br>3710115<br>3710116<br>3710117<br>3710118<br>3710119<br>3710120<br>3710121<br>3710122<br>3710123<br>3710124<br>3710125<br>3710126<br>3710127<br>3710128<br>3710129<br>3710130<br>3710131<br>3710132<br>3710133<br>3710134<br>3710135<br>3710136<br>3710137<br>3710138<br>3710139<br>3710140<br>3710141<br>3710142	extended<br>core<br>full<br>core<br>full<br>core<br>full<br>extended<br>core<br>core<br>full<br>full<br>core<br>core<br>core<br>core<br>core<br>full<br>extended<br>extended<br>full<br>core<br>full<br>core<br>core<br>core<br>extended<br>extended<br>extended<br>extended<br>extended<br>extended<br>extended<br>extended	17	9670012	9789064	+	NM_004246<br>BC019818<br>BC043390<br>ENSESTT00000006387<br>ENST00000262441<br>ENST00000304773<br>GENSCAN00000007295	Homo sapiens glucagon-like peptide 2 receptor (GLP2R), mRNA.<br>Homo sapiens, clone IMAGE:5107203, mRNA, partial cds.<br>Homo sapiens glucagon-like peptide 2 receptor, mRNA (cDNA clone IMAGE:6024259), partial cds.<br><br>cdna:known-ccds chromosome:NCBI36:17:9670106:9733747:1 gene:ENSG00000065325 CCDS11150.1<br>cdna:known chromosome:NCBI36:17:9686597:9711009:1 gene:ENSG00000065325<br>cdna:Genscan chromosome:NCBI36:17:9672026:9733747:1

		3710143	extended						
3721452	872266<br>872267<br>872268<br>872269<br>872270<br>872271<br>872272<br>872273<br>872274<br>872275	3721453<br>3721454<br>3721455<br>3721456<br>3721457<br>3721458<br>3721459<br>3721460<br>3721461<br>3721462<br>3721463<br>3721464<br>3721465<br>3721466<br>3721467<br>3721468<br>3721469<br>3721470<br>3721471<br>3721472<br>3721473<br>3721474<br>3721475<br>3721476<br>3721477<br>3721478<br>3721479<br>3721480<br>3721481<br>3721482<br>3721483<br>3721484	extended<br>core<br>core<br>core<br>full<br>extended<br>core<br>core<br>core<br>core<br>extended<br>core<br>core<br>extended<br>core<br>extended<br>extended<br>extended<br>extended<br>core<br>extended<br>extended<br>extended<br>extended<br>core<br>core<br>full<br>core<br>core<br>core<br>core<br>core	17	37222498	37232949	+	NM_021939<br>AK000690<br>AK092042<br>AK092708<br>ENSESTT00000018473<br>ENST00000321562<br>ENST00000269598<br>ENST00000357255<br>GENSCAN00000049809	Homo sapiens FK506 binding protein 10, 65 kDa (FKBP10), mRNA.<br>Homo sapiens cDNA FLJ20683 fis, clone KAIA3541.<br>Homo sapiens cDNA FLJ34723 fis, clone MESAN2005811, highly similar to 65 KDA FK506-BINDING PROTEIN PRECURSOR (EC 5.2.1.8).<br>Homo sapiens cDNA FLJ35389 fis, clone SKNMC2000635, moderately similar to 65 KDA FK506-BINDING PROTEIN PRECURSOR (EC 5.2.1.8).<br><br>cdna:known-ccds chromosome:NCBI36:17:37222727:37232995:1 gene:ENSG00000141756 CCDS11409.1<br>cdna:known chromosome:NCBI36:17:37222727:37232995:1 gene:ENSG00000141756<br>cdna:known chromosome:NCBI36:17:37222767:37232989:1 gene:ENSG00000141756<br>cdna:Genscan chromosome:NCBI36:17:37222813:37232186:1
3725572	874631<br>874632<br>874633<br>874634<br>874635<br>874636<br>874637<br>874638<br>874639<br>874640<br>874641<br>874642<br>874643<br>874644<br>874645<br>874646	3725570<br>3725571<br>3725573<br>3725574<br>3725575<br>3725576<br>3725577<br>3725578<br>3725579<br>3725580<br>3725581<br>3725582<br>3725583<br>3725584<br>3725585<br>3725586<br>3725587<br>3725588<br>3725589<br>3725590	full<br>full<br>core<br>core<br>core<br>core<br>core<br>extended<br>core<br>full<br>core<br>full<br>core<br>full<br>full<br>core<br>core<br>core<br>core<br>core	17	44565084	44602225	+	NM_153446<br>ENSESTT00000006183<br>ENST00000300404<br>GENSCAN00000023720	Homo sapiens beta-1,4-N-acetyl-galactosaminyl transferase 2 (B4GALNT2), mRNA.<br><br>cdna:known-ccds chromosome:NCBI36:17:44565328:44602235:1 gene:ENSG00000167080 CCDS11544.1<br>cdna:Genscan chromosome:NCBI36:17:44565084:44602089:1

		3725591	full						
3726934	875429<br>875430<br>875431<br>875432<br>875433<br>875434<br>875435<br>875436<br>875437<br>875438<br>875439<br>875440<br>875441	3726935<br>3726936<br>3726937<br>3726938<br>3726939<br>3726940<br>3726941<br>3726942<br>3726943<br>3726944<br>3726945<br>3726946<br>3726947<br>3726948<br>3726949<br>3726950<br>3726951<br>3726952<br>3726953<br>3726954<br>3726955<br>3726956<br>3726957<br>3726958<br>3726959	full<br>full<br>extended<br>full<br>full<br>full<br>full<br>core<br>extended<br>core<br>core<br>extended<br>core<br>extended<br>extended<br>core<br>extended<br>core<br>extended<br>extended<br>core<br>core<br>core<br>core<br>extended	17	46564693	46594905	+	NM_000269<br>NM_198175<br>NM_001018136<br>BC107894<br>ENSESTT00000004090<br>ENSESTT00000004091<br>ENST00000311032<br>ENST00000336097<br>ENST00000013034<br>ENST00000376392<br>GENSCAN00000025937	Homo sapiens non-metastatic cells 1, protein (NM23A) expressed in (NME1), transcript variant 2, mRNA.<br>Homo sapiens non-metastatic cells 1, protein (NM23A) expressed in (NME1), transcript variant 1, mRNA.<br>Homo sapiens NM23-LV (NME1-NME2), mRNA.<br>Homo sapiens NM23-LV, mRNA (cDNA clone MGC:111212 IMAGE:6717551), complete cds.<br><br><br>cdna:known-ccds chromosome:NCBI36:17:46585919:46604103:1 gene:ENSG00000011052 CCDS11580.1<br>cdna:known-ccds chromosome:NCBI36:17:46585919:46594449:1 gene:ENSG00000011052 CCDS11578.1<br>cdna:known-ccds chromosome:NCBI36:17:46585919:46594449:1 gene:ENSG00000011052 CCDS11579.1<br>cdna:known chromosome:NCBI36:17:46585919:46604103:1 gene:ENSG00000011052<br>cdna:Genscan chromosome:NCBI36:17:46564693:46594205:1
3726992	875458<br>875459<br>875460<br>875461<br>875462<br>875463<br>875464<br>875465<br>875466<br>875467<br>875468<br>875469<br>875470<br>875471<br>875472<br>875473<br>875474<br>875475<br>875476<br>875477<br>875478<br>875479<br>875480<br>875481	3726993<br>3726994<br>3726995<br>3726996<br>3726997<br>3726998<br>3726999<br>3727000<br>3727001<br>3727002<br>3727003<br>3727004<br>3727005<br>3727006<br>3727007<br>3727008<br>3727009<br>3727010<br>3727011<br>3727012<br>3727013<br>3727014<br>3727015<br>3727016<br>3727017<br>3727018<br>3727019	full<br>full<br>extended<br>core<br>core<br>core<br>core<br>core<br>core<br>core<br>core<br>core<br>extended<br>core<br>core<br>core<br>core<br>core<br>core<br>full<br>extended<br>core<br>full<br>extended<br>extended<br>core<br>core	17	46692081	46730289	+	NM_016001<br>ENSESTT00000004095<br>ENSESTT00000004096<br>ENSESTT00000004097<br>ENST00000225298<br>GENSCAN00000054453	Homo sapiens UTP18, small subunit (SSU) processome component, homolog (yeast) (UTP18), mRNA.<br><br><br><br>cdna:known chromosome:NCBI36:17:46692945:46730289:1 gene:ENSG00000011260<br>cdna:Genscan chromosome:NCBI36:17:46685799:46719119:1

		3727020<br>3727021<br>3727022<br>3727023<br>3727024<br>3727025<br>3727026<br>3727027	extended<br>extended<br>extended<br>core<br>extended<br>core<br>extended<br>core						
3728776	876615<br>876616<br>876617<br>876618<br>876619<br>876620<br>876621<br>876622<br>876623<br>876624<br>876625<br>876626<br>876627<br>876628<br>876629<br>876630<br>876631<br>876632<br>876633<br>876634<br>876635<br>876636<br>876637	3728777<br>3728778<br>3728779<br>3728780<br>3728781<br>3728782<br>3728783<br>3728784<br>3728785<br>3728786<br>3728787<br>3728788<br>3728789<br>3728790<br>3728791<br>3728792<br>3728793<br>3728794<br>3728795<br>3728796<br>3728797<br>3728798<br>3728799<br>3728800<br>3728801<br>3728802<br>3728803<br>3728804<br>3728805<br>3728806<br>3728807<br>3728808<br>3728809<br>3728810<br>3728811<br>3728812<br>3728813<br>3728814<br>3728815	extended<br>core<br>core<br>extended<br>extended<br>extended<br>extended<br>extended<br>extended<br>core<br>core<br>extended<br>core<br>extended<br>extended<br>extended<br>core<br>extended<br>extended<br>extended<br>extended<br>core<br>extended<br>extended<br>extended<br>core<br>extended<br>extended<br>core<br>extended<br>full<br>extended<br>extended<br>core<br>extended<br>extended<br>core<br>core<br>core	17	54124953	54166685	+	NM_058216<br>NM_002876<br>AK129738<br>CR623930<br>BC073161<br>ENSESTT00000033921<br>ENSESTT00000033922<br>ENSESTT00000033923<br>ENSESTT00000033924<br>ENST00000337432<br>ENST00000308321<br>GENSCAN00000067746	Homo sapiens RAD51 homolog C (S. cerevisiae) (RAD51C), transcript variant 1, mRNA.<br>Homo sapiens RAD51 homolog C (S. cerevisiae) (RAD51C), transcript variant 2, mRNA.<br>Homo sapiens cDNA FLJ26227 fis, clone ADG08867.<br>full-length cDNA clone CS0DN003YG15 of Adult brain of Homo sapiens (human).<br>Homo sapiens cDNA clone IMAGE:6156549, containing frame-shift errors.<br><br><br><br><br>cdna:known-ccds chromosome:NCBI36:17:54124962:54166689:1 gene:ENSG00000108384 CCDS11611.1<br>cdna:known-ccds chromosome:NCBI36:17:54125004:54166689:1 gene:ENSG00000108384 CCDS11612.1<br>cdna:Genscan chromosome:NCBI36:17:54125004:54142350:1
3728964	876750<br>876751<br>876752<br>876753<br>876754<br>876755	3728965<br>3728966<br>3728967<br>3728968<br>3728969<br>3728970	full<br>full<br>full<br>extended<br>extended<br>core	17	54565803	54638833	+	NM_018304<br>AK000296<br>AK225380<br>BC008669<br>ENSESTT00000033927<br>ENSESTT00000033928	Homo sapiens proline rich 11 (PRR11), mRNA.<br>Homo sapiens cDNA FLJ20289 fis, clone HEP04492.<br>Homo sapiens mRNA for hypothetical protein LOC55771 variant, clone: HEP19570.<br>Homo sapiens proline rich 11, mRNA (cDNA clone MGC:8798 IMAGE:3867293), complete cds.

	876756<br>876757<br>876758<br>876759<br>876760<br>876761<br>876762<br>876763<br>876764<br>876765<br>876766<br>876767<br>876768<br>876769<br>876770<br>876771<br>876772	3728971<br>3728972<br>3728973<br>3728974<br>3728975<br>3728976<br>3728977<br>3728978<br>3728979<br>3728980<br>3728981<br>3728982<br>3728983<br>3728984<br>3728985<br>3728986<br>3728987<br>3728988<br>3728989<br>3728990<br>3728991<br>3728992<br>3728993	full<br>full<br>full<br>full<br>full<br>full<br>full<br>core<br>core<br>core<br>core<br>core<br>core<br>core<br>core<br>core<br>extended<br>core<br>core<br>extended<br>core<br>extended<br>extended					ENST00000262293<br>GENSCAN00000046107<br>GENSCAN00000046110	cdna:known-ccds chromosome:NCBI36:17:54587900:54635977:1 gene:ENSG00000068489 CCDS11614.1<br>cdna:Genscan chromosome:NCBI36:17:54601896:54633774:1<br>cdna:Genscan chromosome:NCBI36:17:54565803:54587865:1
3733590	879587<br>879588<br>879589<br>879590<br>879591<br>879592<br>879593<br>879594<br>879595<br>879596<br>879597<br>879598	3733591<br>3733592<br>3733593<br>3733594<br>3733595<br>3733596<br>3733597<br>3733598<br>3733599<br>3733600<br>3733601<br>3733602<br>3733603<br>3733604<br>3733605<br>3733606<br>3733607<br>3733608<br>3733609<br>3733610	full<br>full<br>full<br>full<br>full<br>full<br>full<br>full<br>full<br>extended<br>core<br>core<br>core<br>core<br>core<br>core<br>core<br>core<br>core<br>core	17	67611413	67634147	+	NM_000346<br>BC056420<br>ENSESTT00000042914<br>ENST00000245479<br>GENSCAN00000059056	Homo sapiens SRY (sex determining region Y)-box 9 (campomelic dysplasia, autosomal sex-reversal) (SOX9), mRNA.<br>Homo sapiens SRY (sex determining region Y)-box 9 (campomelic dysplasia, autosomal sex-reversal), mRNA (cDNA clone MGC:65106 IMAGE:6200521), complete cds.<br><br>cdna:known-ccds chromosome:NCBI36:17:67628756:67634147:1 gene:ENSG00000125398 CCDS11689.1<br>cdna:Genscan chromosome:NCBI36:17:67611413:67632123:1
3735151	880505<br>880506<br>880507<br>880508<br>880509<br>880510<br>880511<br>880512<br>880513<br>880514	3735150<br>3735152<br>3735153<br>3735154<br>3735155<br>3735156<br>3735157<br>3735158<br>3735159<br>3735160	full<br>extended<br>extended<br>core<br>core<br>extended<br>core<br>core<br>core<br>core	17	71228300	71265487	+	NM_001005619<br>NM_000213<br>NM_001005731<br>ENSESTT00000053252<br>ENSESTT00000053253<br>ENSESTT00000053254<br>ENSESTT00000053255<br>ENSESTT00000053256<br>ENSESTT00000053257<br>ENSESTT00000053258	Homo sapiens integrin, beta 4 (ITGB4), transcript variant 2, mRNA.<br>Homo sapiens integrin, beta 4 (ITGB4), transcript variant 1, mRNA.<br>Homo sapiens integrin, beta 4 (ITGB4), transcript variant 3, mRNA.

	880515	3735161	core					ENSESTT00000053259		
	880516	3735162	core					ENSESTT00000053260		
	880517	3735163	full					ENSESTT00000053261		
	880518	3735164	core					ENSESTT00000053262		
	880519	3735165	core					ENST00000200181	cdna:known-ccds chromosome:NCBI36:17:71229111:71265493:1 gene:ENSG00000132470 CCDS11727.1	
	880520	3735166	core					ENST00000339591	cdna:known chromosome:NCBI36:17:71232371:71265493:1 gene:ENSG00000132470	
	880521	3735167	core					GENSCAN00000004418	cdna:Genscan chromosome:NCBI36:17:71232379:71265231:1	
	880522	3735168	core							
	880523	3735169	core							
	880524	3735170	core							
	880525	3735171	extended							
	880526	3735172	core							
	880527	3735173	extended							
	880528	3735174	core							
	880529	3735175	extended							
	880530	3735176	core							
	880531	3735177	core							
	880532	3735178	extended							
	880533	3735179	extended							
	880534	3735180	core							
	880535	3735181	extended							
	880536	3735182	core							
	880537	3735183	core							
	880538	3735184	core							
	880539	3735185	core							
	880540	3735186	extended							
	880541	3735187	core							
	880542	3735188	extended							
	880543	3735189	core							
	880544	3735190	core							
	880545	3735191	extended							
	880546	3735192	core							
	880547	3735193	extended							
	880548	3735194	extended							
	880549	3735195	extended							
		3735196	core							
		3735197	core							
		3735198	core							
		3735199	core							
		3735200	extended							
		3735201	core							
		3735202	core							
		3735203	extended							
		3735204	core							
		3735205	extended							
		3735206	core							
		3735207	core							
		3735208	extended							
		3735209	extended							
		3735210	core							
		3735211	extended							
		3735212	core							
		3735213	core							
		3735214	core							

		3735215<br>3735216<br>3735217<br>3735218<br>3735219	core<br>extended<br>core<br>core<br>core						
3741800	884518<br>884519<br>884520<br>884521<br>884522<br>884523<br>884524<br>884525<br>884526<br>884527<br>884528<br>884529<br>884530<br>884531<br>884532<br>884533<br>884534<br>884535<br>884536<br>884537<br>884538<br>884539<br>884540<br>884541<br>884542<br>884543<br>884544<br>884545<br>884546<br>884547<br>884548<br>884549<br>884550<br>884551<br>884552<br>884553<br>884554<br>884555<br>884556	3741801<br>3741802<br>3741803<br>3741804<br>3741805<br>3741806<br>3741807<br>3741808<br>3741809<br>3741810<br>3741811<br>3741812<br>3741813<br>3741814<br>3741815<br>3741816<br>3741817<br>3741818<br>3741819<br>3741820<br>3741821<br>3741822<br>3741823<br>3741824<br>3741825<br>3741826<br>3741827<br>3741828<br>3741829<br>3741830<br>3741831<br>3741832<br>3741833<br>3741834<br>3741835<br>3741836<br>3741837<br>3741838<br>3741839<br>3741840<br>3741841<br>3741842<br>3741843<br>3741844<br>3741845<br>3741846<br>3741847<br>3741848<br>3741849	core<br>core<br>core<br>core<br>core<br>core<br>core<br>core<br>extended<br>full<br>extended<br>core<br>extended<br>core<br>extended<br>core<br>extended<br>core<br>core<br>extended<br>extended<br>extended<br>core<br>extended<br>core<br>extended<br>extended<br>core<br>extended<br>full<br>core<br>core<br>core<br>core<br>core<br>core<br>full<br>extended<br>full<br>full<br>core<br>full<br>core<br>core<br>full<br>core<br>core<br>extended<br>core	17	3773944	3814520	-	NM_174953<br>NM_174954<br>NM_174955<br>NM_174956<br>NM_174957<br>NM_174958<br>NM_005173<br>CR610387<br>ENSESTT00000034896<br>ENSESTT00000034897<br>ENST00000359983<br>ENST00000352011<br>ENST00000309890	Homo sapiens ATPase, Ca++ transporting, ubiquitous (ATP2A3), transcript variant 5, mRNA.<br>Homo sapiens ATPase, Ca++ transporting, ubiquitous (ATP2A3), transcript variant 4, mRNA.<br>Homo sapiens ATPase, Ca++ transporting, ubiquitous (ATP2A3), transcript variant 2, mRNA.<br>Homo sapiens ATPase, Ca++ transporting, ubiquitous (ATP2A3), transcript variant 3, mRNA.<br>Homo sapiens ATPase, Ca++ transporting, ubiquitous (ATP2A3), transcript variant 6, mRNA.<br>Homo sapiens ATPase, Ca++ transporting, ubiquitous (ATP2A3), transcript variant 7, mRNA.<br>Homo sapiens ATPase, Ca++ transporting, ubiquitous (ATP2A3), transcript variant 1, mRNA.<br>full-length cDNA clone CS0CAP008YK17 of Thymus of Homo sapiens (human).<br><br><br>cdna:known-ccds chromosome:NCBI36:17:3773918:3814485:-1 gene:ENSG00000074370 CCDS11042.1<br>cdna:known-ccds chromosome:NCBI36:17:3773918:3814485:-1 gene:ENSG00000074370 CCDS11041.1<br>cdna:known chromosome:NCBI36:17:3773918:3814485:-1 gene:ENSG00000074370

		3741850<br>3741851<br>3741852<br>3741853<br>3741854<br>3741855<br>3741856<br>3741857<br>3741858	full<br>full<br>extended<br>full<br>full<br>full<br>core<br>core<br>core						
3741875	884566<br>884567<br>884568<br>884569<br>884570<br>884571<br>884572<br>884573<br>884574<br>884575<br>884576<br>884577<br>884578<br>884579<br>884580<br>884581<br>884582<br>884583<br>884584<br>884585<br>884586<br>884587<br>884588<br>884589<br>884590<br>884591<br>884592<br>884593<br>884594<br>884595<br>884596<br>884597<br>884598<br>884599<br>884600<br>884601<br>884602<br>884603<br>884604<br>884605<br>884606<br>884607<br>884608<br>884609<br>884610	3741876<br>3741877<br>3741878<br>3741879<br>3741880<br>3741881<br>3741882<br>3741883<br>3741884<br>3741885<br>3741886<br>3741887<br>3741888<br>3741889<br>3741890<br>3741891<br>3741892<br>3741893<br>3741894<br>3741895<br>3741896<br>3741897<br>3741898<br>3741899<br>3741900<br>3741901<br>3741902<br>3741903<br>3741904<br>3741905<br>3741906<br>3741907<br>3741908<br>3741909<br>3741910<br>3741911<br>3741912<br>3741913<br>3741914<br>3741915<br>3741916<br>3741917<br>3741918<br>3741919<br>3741920	core<br>core<br>core<br>core<br>core<br>core<br>core<br>core<br>extended<br>extended<br>core<br>extended<br>extended<br>extended<br>core<br>core<br>core<br>core<br>full<br>core<br>core<br>extended<br>extended<br>core<br>core<br>core<br>core<br>extended<br>extended<br>extended<br>core<br>core<br>core<br>core<br>extended<br>core<br>extended<br>extended<br>core<br>core<br>full<br>full<br>core<br>full<br>core	17	3854494	3993741	-	NM_015113<br>AK001683<br>AK122719<br>AK127482<br>AK128386<br>AL831960<br>U33270<br>BC047285<br>ENSESTT00000034892<br>ENSESTT00000034893<br>ENSESTT00000034894<br>ENSESTT00000034895<br>ENST00000263089<br>ENST00000381638<br>GENSCAN00000046753	Homo sapiens zinc finger, ZZ-type with EF-hand domain 1 (ZZEF1), mRNA.<br>Homo sapiens cDNA FLJ10821 fis, clone NT2RP4001057.<br>Homo sapiens cDNA FLJ16216 fis, clone CTONG2028486.<br>Homo sapiens cDNA FLJ45574 fis, clone BRTHA3011187.<br>Homo sapiens cDNA FLJ46529 fis, clone THYMU3034983.<br>Homo sapiens mRNA; cDNA DKFZp761G0818 (from clone DKFZp761G0818).<br>Human normal keratinocyte mRNA, clone A47, partial 3' sequence.<br>Homo sapiens zinc finger, ZZ-type with EF-hand domain 1, mRNA (cDNA clone IMAGE:5103260), containing frame-shift errors.<br><br><br><br><br>cdna:known-ccds chromosome:NCBI36:17:3854488:3993063:-1 gene:ENSG00000074755 CCDS11043.1<br>cdna:known chromosome:NCBI36:17:3854489:3993002:-1 gene:ENSG00000074755<br>cdna:Genscan chromosome:NCBI36:17:3856933:3926802:-1

	884611	3741921	full						
	884612	3741922	core						
	884613	3741923	core						
	884614	3741924	core						
	884615	3741925	core						
	884616	3741926	core						
	884617	3741927	core						
	884618	3741928	extended						
	884619	3741929	core						
	884620	3741930	extended						
	884621	3741931	extended						
	884622	3741932	core						
	884623	3741933	core						
	884624	3741934	core						
	884625	3741935	core						
	884626	3741936	extended						
	884627	3741937	core						
	884628	3741938	core						
	884629	3741939	core						
	884630	3741940	core						
	884631	3741941	core						
	884632	3741942	core						
	884633	3741943	extended						
	884634	3741944	core						
	884635	3741945	core						
	884636	3741946	core						
	884637	3741947	core						
	884638	3741948	core						
	884639	3741949	extended						
		3741950	core						
		3741951	core						
		3741952	core						
		3741953	core						
		3741954	core						
		3741955	core						
		3741956	full						
		3741957	core						
		3741958	core						
		3741959	core						
		3741960	core						
		3741961	core						
		3741962	core						
		3741963	extended						
		3741964	core						
		3741965	core						
		3741966	core						
		3741967	full						
		3741968	core						
		3741969	core						
		3741970	core						
		3741971	core						
		3741972	core						
		3741973	full						
		3741974	extended						

		3741975<br>3741976<br>3741977<br>3741978<br>3741979<br>3741980<br>3741981<br>3741982<br>3741983<br>3741984<br>3741985<br>3741986<br>3741987<br>3741988<br>3741989	core<br>full<br>extended<br>extended<br>full<br>full<br>full<br>full<br>full<br>full<br>core<br>core<br>core<br>full<br>full						
3742285	884821<br>884822<br>884823<br>884824<br>884825<br>884826<br>884827	3742286<br>3742287<br>3742288<br>3742289<br>3742290<br>3742291<br>3742292<br>3742293<br>3742294<br>3742295<br>3742296<br>3742297	extended<br>core<br>core<br>core<br>core<br>core<br>core<br>extended<br>core<br>core<br>core<br>core	17	4582958	4589964	-	NM_022059<br>CR611932<br>CR623711<br>ENSESTT00000058624<br>ENST00000381478<br>ENST00000293778<br>GENSCAN00000052915	Homo sapiens chemokine (C-X-C motif) ligand 16 (CXCL16), mRNA.<br>full-length cDNA clone CS0DI028YA08 of Placenta Cot 25-normalized of Homo sapiens (human).<br>full-length cDNA clone CS0DJ002YJ19 of T cells (Jurkat cell line) Cot 10-normalized of Homo sapiens (human).<br><br>cdna:known-ccds chromosome:NCBI36:17:4583577:4589972:-1 gene:ENSG00000161921 CCDS11052.1<br>cdna:known chromosome:NCBI36:17:4583579:4589890:-1 gene:ENSG00000161921<br>cdna:Genscan chromosome:NCBI36:17:4585119:4589383:-1
3748798	888696<br>888697<br>888698<br>888699<br>888700<br>888701<br>888702<br>888703<br>888704<br>888705	3748799<br>3748800<br>3748801<br>3748802<br>3748803<br>3748804<br>3748805<br>3748806<br>3748807<br>3748808<br>3748809<br>3748810<br>3748811<br>3748812<br>3748813<br>3748814	core<br>core<br>core<br>core<br>core<br>core<br>core<br>core<br>core<br>core<br>core<br>core<br>full<br>extended<br>extended<br>extended	17	19227355	19231126	-	NM_002404<br>CR613174<br>BC047725<br>ENST00000299610<br>GENSCAN00000035631	Homo sapiens microfibrillar-associated protein 4 (MFAP4), mRNA.<br>full-length cDNA clone CS0DE008YE19 of Placenta of Homo sapiens (human).<br>Homo sapiens microfibrillar-associated protein 4, mRNA (cDNA clone IMAGE:5752730), containing frame-shift errors.<br>cdna:known-ccds chromosome:NCBI36:17:19227349:19231086:-1 gene:ENSG00000166482 CCDS11208.1<br>cdna:Genscan chromosome:NCBI36:17:19228368:19231061:-1
3750662	889759<br>889760<br>889761<br>889762<br>889763<br>889764<br>889765<br>889766	3750663<br>3750664<br>3750665<br>3750666<br>3750667<br>3750668<br>3750669<br>3750670<br>3750671<br>3750672	core<br>core<br>core<br>core<br>extended<br>core<br>extended<br>core<br>core<br>extended	17	23718435	23721844	-	NM_000638<br>ENSESTT00000018072<br>ENST00000226218<br>GENSCAN00000050503	Homo sapiens vitronectin (VTN), mRNA.<br><br>cdna:known-ccds chromosome:NCBI36:17:23718425:23721844:-1 gene:ENSG00000109072 CCDS11229.1<br>cdna:Genscan chromosome:NCBI36:17:23718517:23721351:-1

		3750673<br>3750674<br>3750675<br>3750676<br>3750677<br>3750678<br>3750679<br>3750680	core<br>core<br>core<br>core<br>core<br>core<br>core<br>core						
3751859	890455<br>890456<br>890457<br>890458<br>890459<br>890460<br>890461<br>890462<br>890463<br>890464<br>890465	3751860<br>3751861<br>3751862<br>3751863<br>3751864<br>3751865<br>3751866<br>3751867<br>3751868<br>3751869<br>3751870<br>3751871<br>3751872	extended<br>core<br>core<br>full<br>core<br>full<br>full<br>core<br>core<br>core<br>core<br>core<br>core	17	25667001	25685191	-	NM_206832<br>ENSESTT00000002811<br>ENST00000328886	Homo sapiens transmembrane and immunoglobulin domain containing 1 (TMIGD1), mRNA.<br><br>cdna:known chromosome:NCBI36:17:25667492:25685191:-1 gene:ENSG00000182271
3756193	893049<br>893050<br>893051<br>893052<br>893053<br>893054<br>893055<br>893056<br>893066<br>893069<br>893057<br>893058<br>893079<br>893080<br>893081<br>893059<br>893060<br>893082<br>893116<br>893083<br>893084<br>893085<br>893086<br>893087<br>893088<br>893089<br>893090<br>893117<br>893091<br>893092<br>893093<br>893094	3756144<br>3756145<br>3756146<br>3756147<br>3756148<br>3756149<br>3756150<br>3756151<br>3756163<br>3756165<br>3756167<br>3756169<br>3756152<br>3756153<br>3756194<br>3756195<br>3756196<br>3756197<br>3756154<br>3756155<br>3756198<br>3756199<br>3756237<br>3756200<br>3756201<br>3756202<br>3756203<br>3756204<br>3756205<br>3756206<br>3756207<br>3756208	full<br>full<br>full<br>full<br>full<br>extended<br>full<br>full<br>extended<br>extended<br>extended<br>extended<br>extended<br>extended<br>core<br>core<br>core<br>core<br>extended<br>full<br>core<br>core<br>extended<br>core<br>core<br>core<br>core<br>core<br>core<br>core<br>core<br>core	17	35717597	35847034	-	NM_001067<br>AF285157<br>AF285158<br>AF285159<br>AK123052<br>ENSESTT00000026172<br>ENSESTT00000026173<br>ENSESTT00000026174<br>ENST00000269577<br>ENST00000357601<br>ENST00000348049<br>GENSCAN00000049618<br>GENSCAN00000049629	Homo sapiens topoisomerase (DNA) II alpha 170kDa (TOP2A), mRNA.<br>Homo sapiens topoisomerase II alpha-2 (TOP2A) mRNA, partial cds.<br>Homo sapiens topoisomerase II alpha-3 (TOP2A) mRNA, partial cds.<br>Homo sapiens topoisomerase II alpha-4 (TOP2A) mRNA, partial cds.<br>Homo sapiens cDNA FLJ41057 fis, clone TESTI2009812.<br><br><br><br>cdna:known chromosome:NCBI36:17:35798321:35827695:-1 gene:ENSG00000131747<br>cdna:known chromosome:NCBI36:17:35798321:35827695:-1 gene:ENSG00000131747<br>cdna:known chromosome:NCBI36:17:35798321:35827695:-1 gene:ENSG00000131747<br>cdna:Genscan chromosome:NCBI36:17:35799297:35827598:-1<br>cdna:Genscan chromosome:NCBI36:17:35717597:35726753:-1

	893095<br>893096<br>893097<br>893098<br>893099<br>893100<br>893101<br>893102<br>893103<br>893104<br>893106<br>893107<br>893108<br>893109<br>893110<br>893061<br>893111<br>893112<br>893113<br>893114<br>893115<br>893062<br>893063<br>893064<br>893065	3756239<br>3756209<br>3756210<br>3756211<br>3756212<br>3756213<br>3756214<br>3756215<br>3756216<br>3756217<br>3756218<br>3756219<br>3756220<br>3756221<br>3756222<br>3756241<br>3756223<br>3756243<br>3756224<br>3756245<br>3756225<br>3756247<br>3756226<br>3756227<br>3756228<br>3756229<br>3756156<br>3756230<br>3756231<br>3756232<br>3756233<br>3756234<br>3756235<br>3756157<br>3756158<br>3756159<br>3756160<br>3756161	extended<br>core<br>core<br>core<br>core<br>core<br>core<br>core<br>core<br>core<br>core<br>core<br>core<br>core<br>core<br>extended<br>core<br>extended<br>core<br>extended<br>core<br>extended<br>core<br>core<br>core<br>core<br>full<br>core<br>core<br>core<br>core<br>core<br>core<br>full<br>full<br>full<br>full<br>full						
3756566	893300<br>893301<br>893302<br>893303<br>893304<br>893305<br>893306<br>893307<br>893308	3756567<br>3756568<br>3756569<br>3756570<br>3756571<br>3756572<br>3756573<br>3756574<br>3756575<br>3756576<br>3756577<br>3756578<br>3756579<br>3756580	extended<br>core<br>core<br>core<br>core<br>core<br>core<br>core<br>core<br>extended<br>core<br>core<br>core<br>core	17	36285377	36294991	-	NM_019010<br>ENSESTT00000038973<br>ENSESTT00000038974<br>ENSESTT00000038975<br>ENST00000167588<br>GENSCAN00000060622	Homo sapiens keratin 20 (KRT20), mRNA.<br><br><br><br>cdna:known-ccds chromosome:NCBI36:17:36285721:36295005:-1 gene:ENSG00000171431 CCDS11379.1<br>cdna:Genscan chromosome:NCBI36:17:36271439:36346381:-1
3758510	894366	3758511	core	17	38960742	38979228	-	NM_001986	Homo sapiens ets variant gene 4 (E1A enhancer binding protein, E1AF) (ETV4), mRNA.

	894367<br>894368<br>894369<br>894370<br>894371<br>894372<br>894373<br>894374<br>894375<br>894376<br>894377<br>894378<br>894379<br>894380	3758512<br>3758513<br>3758514<br>3758515<br>3758516<br>3758517<br>3758518<br>3758519<br>3758520<br>3758521<br>3758522<br>3758523<br>3758524<br>3758525<br>3758526<br>3758527<br>3758528<br>3758529<br>3758530<br>3758531<br>3758532	core<br>core<br>full<br>full<br>core<br>core<br>full<br>core<br>extended<br>core<br>core<br>core<br>core<br>core<br>core<br>extended<br>core<br>core<br>full<br>extended<br>extended					BC007242<br>DQ396625<br>ENSESTT00000014412<br>ENSESTT00000014413<br>ENST00000319349<br>GENSCAN00000064255	Homo sapiens ets variant gene 4 (E1A enhancer binding protein, E1AF), mRNA (cDNA clone IMAGE:2964546), complete cds.<br>Homo sapiens TMPRSS2/ETV4a fusion transcript.<br><br><br>cdna:known-ccds chromosome:NCBI36:17:38960738:38979230:-1 gene:ENSG00000175832 CCDS11465.1<br>cdna:Genscan chromosome:NCBI36:17:38961413:38980118:-1
3764399	897921<br>897922<br>897923<br>897924<br>897925<br>897926<br>897927<br>897928<br>897929<br>897930<br>897931<br>897932<br>897933<br>897934<br>897935<br>897936<br>897937<br>897938<br>897939<br>897940<br>897941<br>897942<br>897943<br>897944<br>897945<br>897946<br>897947	3764400<br>3764401<br>3764402<br>3764403<br>3764404<br>3764405<br>3764406<br>3764407<br>3764408<br>3764409<br>3764410<br>3764411<br>3764412<br>3764413<br>3764414<br>3764415<br>3764416<br>3764417<br>3764418<br>3764419<br>3764420<br>3764421<br>3764422<br>3764423<br>3764424<br>3764425<br>3764426<br>3764427<br>3764428<br>3764429<br>3764430<br>3764431<br>3764432	core<br>core<br>core<br>full<br>extended<br>extended<br>extended<br>extended<br>extended<br>core<br>core<br>core<br>core<br>full<br>core<br>extended<br>core<br>full<br>core<br>core<br>full<br>full<br>full<br>full<br>extended<br>extended<br>extended<br>full<br>full<br>full<br>full<br>full<br>full	17	53785272	53849935	-	NM_017763<br>AB081837<br>ENSESTT00000033962<br>ENSESTT00000033963<br>ENST00000225504<br>ENST00000376226<br>ENST00000225507<br>GENSCAN00000020233	Homo sapiens ring finger protein 43 (RNF43), mRNA.<br>Homo sapiens urcc mRNA, complete cds.<br><br><br>cdna:known-ccds chromosome:NCBI36:17:53778293:53785690:-1 gene:ENSG00000108375 CCDS11606.1<br>cdna:known-ccds chromosome:NCBI36:17:53784860:53849479:-1 gene:ENSG00000108375 CCDS11607.1<br>cdna:novel chromosome:NCBI36:17:53784860:53847937:-1 gene:ENSG00000108375<br>cdna:Genscan chromosome:NCBI36:17:53765508:53803393:-1

		3764433<br>3764434<br>3764435<br>3764436<br>3764437<br>3764438<br>3764439<br>3764440<br>3764441<br>3764442	extended<br>full<br>core<br>core<br>core<br>core<br>extended<br>extended<br>extended<br>extended						
3765580	898639<br>898640<br>898641<br>898642<br>898643<br>898644<br>898645<br>898646<br>898647<br>898648<br>898649<br>898650<br>898651<br>898652<br>898653<br>898654<br>898655<br>898656<br>898657<br>898658<br>898659<br>898660<br>898661<br>898662<br>898663<br>898664<br>898665<br>898666<br>898667<br>898668<br>898669<br>898670<br>898671<br>898672<br>898673<br>898674<br>898675<br>898676	3765581<br>3765582<br>3765583<br>3765584<br>3765585<br>3765586<br>3765587<br>3765588<br>3765589<br>3765590<br>3765591<br>3765592<br>3765593<br>3765594<br>3765595<br>3765596<br>3765597<br>3765598<br>3765599<br>3765600<br>3765601<br>3765602<br>3765603<br>3765604<br>3765605<br>3765606<br>3765607<br>3765608<br>3765609<br>3765610<br>3765611<br>3765612<br>3765613<br>3765614<br>3765615<br>3765616<br>3765617<br>3765618<br>3765619<br>3765620<br>3765621<br>3765622<br>3765623<br>3765624	full<br>full<br>extended<br>core<br>core<br>core<br>extended<br>core<br>core<br>core<br>core<br>extended<br>extended<br>extended<br>extended<br>full<br>extended<br>core<br>core<br>extended<br>extended<br>extended<br>extended<br>full<br>core<br>core<br>extended<br>core<br>extended<br>core<br>core<br>core<br>extended<br>core<br>extended<br>core<br>core<br>extended<br>core<br>full<br>full<br>full<br>core<br>extended	17	57078794	57295537	-	NM_032043<br>AK074713<br>ENSESTT00000053504<br>ENSESTT00000053505<br>ENSESTT00000053506<br>ENSESTT00000053507<br>ENST00000259008<br>GENSCAN00000024247<br>GENSCAN00000024197	Homo sapiens BRCA1 interacting protein C-terminal helicase 1 (BRIP1), mRNA.<br>Homo sapiens cDNA FLJ90232 fis, clone NT2RM2000497, weakly similar to CHL1 PROTEIN.<br><br><br><br><br>cdna:known-ccds chromosome:NCBI36:17:57114767:57295537:-1 gene:ENSG00000136492 CCDS11631.1<br>cdna:Genscan chromosome:NCBI36:17:57115439:57125655:-1<br>cdna:Genscan chromosome:NCBI36:17:57148094:57281399:-1

		3765625<br>3765626<br>3765627<br>3765628<br>3765629<br>3765630<br>3765631<br>3765632	core<br>extended<br>core<br>core<br>core<br>core<br>core<br>core						
3768627	900498<br>900499<br>900500<br>900501<br>900502<br>900503<br>900504<br>900505<br>900506<br>900507<br>900508<br>900509<br>900510<br>900511<br>900512<br>900513<br>900514<br>900515<br>900516<br>900517<br>900518<br>900519<br>900520<br>900521<br>900522<br>900523<br>900524<br>900525<br>900526<br>900527<br>900528<br>900529<br>900530<br>900531<br>900532<br>900533<br>900534<br>900535<br>900536<br>900537<br>900538<br>900539<br>900540<br>900541<br>900542<br>900543	3768628<br>3768629<br>3768630<br>3768631<br>3768632<br>3768633<br>3768634<br>3768635<br>3768636<br>3768637<br>3768638<br>3768639<br>3768640<br>3768641<br>3768642<br>3768643<br>3768644<br>3768645<br>3768646<br>3768647<br>3768648<br>3768649<br>3768650<br>3768651<br>3768652<br>3768653<br>3768654<br>3768655<br>3768656<br>3768657<br>3768658<br>3768659<br>3768660<br>3768661<br>3768662<br>3768663<br>3768664<br>3768665<br>3768666<br>3768667<br>3768668<br>3768669<br>3768670<br>3768671<br>3768672<br>3768673	core<br>core<br>core<br>core<br>core<br>extended<br>core<br>extended<br>extended<br>full<br>core<br>core<br>extended<br>core<br>extended<br>core<br>full<br>extended<br>core<br>full<br>full<br>core<br>core<br>core<br>extended<br>core<br>core<br>extended<br>full<br>full<br>core<br>full<br>core<br>core<br>core<br>core<br>extended<br>extended<br>core<br>full<br>core<br>full<br>core<br>core<br>core<br>core	17	64375028	64463128	-	NM_007168<br>AK090894<br>BC024003<br>BC037309<br>BC047765<br>ENSESTT00000032152<br>ENSESTT00000032154<br>ENSESTT00000032157<br>ENSESTT00000032158<br>ENST00000269080<br>ENST00000356282<br>ENST00000375538<br>GENSCAN00000039049	Homo sapiens ATP-binding cassette, sub-family A (ABC1), member 8 (ABCA8), mRNA.<br>Homo sapiens cDNA FLJ33575 fis, clone BRAMY2011216.<br>Homo sapiens ATP-binding cassette, sub-family A (ABC1), member 8, mRNA (cDNA clone IMAGE:4821839), complete cds.<br>Homo sapiens ATP-binding cassette, sub-family A (ABC1), member 8, mRNA (cDNA clone IMAGE:5262251), complete cds.<br>Homo sapiens ATP-binding cassette, sub-family A (ABC1), member 8, mRNA (cDNA clone IMAGE:5761384), complete cds.<br><br><br><br><br>cdna:known-ccds chromosome:NCBI36:17:64375028:64463128:-1 gene:ENSG00000141338 CCDS11680.1<br>cdna:known chromosome:NCBI36:17:64375028:64463128:-1 gene:ENSG00000141338<br>cdna:known chromosome:NCBI36:17:64429151:64463128:-1 gene:ENSG00000141338<br>cdna:Genscan chromosome:NCBI36:17:64375821:64448611:-1

	900544<br>900545<br>900546	3768674<br>3768675<br>3768676<br>3768677<br>3768678<br>3768679<br>3768680<br>3768681<br>3768682<br>3768683<br>3768684<br>3768685<br>3768686<br>3768687<br>3768688<br>3768689<br>3768690<br>3768691<br>3768692<br>3768693<br>3768694<br>3768695<br>3768696<br>3768697<br>3768698<br>3768699<br>3768700	core<br>core<br>extended<br>full<br>core<br>extended<br>core<br>core<br>extended<br>core<br>core<br>core<br>core<br>core<br>core<br>core<br>core<br>core<br>extended<br>core<br>core<br>core<br>core<br>core<br>core<br>extended<br>core						
3771602	902364<br>902365<br>902366<br>902367<br>902368<br>902369<br>902370<br>902371<br>902372<br>902373<br>902374<br>902375<br>902376<br>902377<br>902378<br>902379<br>902380<br>902381<br>902382<br>902383<br>902384<br>902385	3771603<br>3771604<br>3771605<br>3771606<br>3771607<br>3771608<br>3771609<br>3771610<br>3771611<br>3771612<br>3771613<br>3771614<br>3771615<br>3771616<br>3771617<br>3771618<br>3771619<br>3771620<br>3771621<br>3771622<br>3771623<br>3771624<br>3771625<br>3771626<br>3771627<br>3771628<br>3771629	core<br>core<br>extended<br>core<br>extended<br>core<br>core<br>core<br>core<br>core<br>full<br>core<br>core<br>core<br>extended<br>core<br>core<br>full<br>core<br>core<br>core<br>core<br>extended<br>core<br>extended<br>core<br>core	17	71978579	72009192	-	NM_024599<br>NM_001005498<br>AK057882<br>AK074034<br>AK097267<br>AL833680<br>BX648448<br>ENSESTT00000056564<br>ENSESTT00000056565<br>ENSESTT00000056566<br>ENSESTT00000056567<br>ENST00000389765<br>ENST00000389764<br>ENST00000389762<br>ENST00000389760<br>ENST00000313080<br>GENSCAN00000019012	Homo sapiens rhomboid 5 homolog 2 (Drosophila) (RHBDF2), transcript variant 1, mRNA.<br>Homo sapiens rhomboid 5 homolog 2 (Drosophila) (RHBDF2), transcript variant 2, mRNA.<br>Homo sapiens cDNA FLJ25153 fis, clone CBR07880.<br>Homo sapiens mRNA for FLJ00080 protein.<br>Homo sapiens cDNA FLJ39948 fis, clone SPLEN2024273.<br>Homo sapiens mRNA; cDNA DKFZp667E1422 (from clone DKFZp667E1422).<br>Homo sapiens mRNA; cDNA DKFZp686O04259 (from clone DKFZp686O04259).<br><br><br><br><br>cdna:known chromosome:NCBI36:17:71979310:71989201:-1 gene:ENSG00000129667<br>cdna:known chromosome:NCBI36:17:71979310:71989201:-1 gene:ENSG00000129667<br>cdna:known chromosome:NCBI36:17:71979310:71989201:-1 gene:ENSG00000129667<br>cdna:known chromosome:NCBI36:17:71979310:71989201:-1 gene:ENSG00000129667<br>cdna:novel chromosome:NCBI36:17:71978574:72009084:-1 gene:ENSG00000129667<br>cdna:Genscan chromosome:NCBI36:17:71979310:72009192:-1

		3771630<br>3771631<br>3771632<br>3771633<br>3771634<br>3771635<br>3771636<br>3771637<br>3771638<br>3771639<br>3771640<br>3771641	core<br>extended<br>full<br>extended<br>core<br>extended<br>extended<br>extended<br>extended<br>extended<br>core<br>full						
3771800	902482<br>902483<br>902484<br>902485<br>902486<br>902487<br>902488<br>902489<br>902490<br>902491	3771801<br>3771802<br>3771803<br>3771804<br>3771805<br>3771806<br>3771807<br>3771808<br>3771809<br>3771810<br>3771811<br>3771812<br>3771813<br>3771814<br>3771815<br>3771816<br>3771817<br>3771818	core<br>core<br>core<br>core<br>core<br>core<br>core<br>core<br>core<br>core<br>core<br>extended<br>core<br>core<br>core<br>core<br>core<br>extended	17	72241812	72245582	-	NM_003016<br>CR603955<br>ENSESTT00000056547<br>ENSESTT00000056548<br>ENST00000293233<br>ENST00000303996<br>ENST00000359995<br>ENST00000358156<br>GENSCAN00000042263	Homo sapiens splicing factor, arginine/serine-rich 2 (SFRS2), mRNA.<br>full-length cDNA clone CS0DI043YD16 of Placenta Cot 25-normalized of Homo sapiens (human).<br><br><br>cdna:known-ccds chromosome:NCBI36:17:72241806:72244837:-1 gene:ENSG00000161547 CCDS11749.1<br>cdna:known chromosome:NCBI36:17:72220514:72234158:-1 gene:ENSG00000070495<br>cdna:known chromosome:NCBI36:17:72241796:72244837:-1 gene:ENSG00000161547<br>cdna:known chromosome:NCBI36:17:72243774:72244837:-1 gene:ENSG00000161547<br>cdna:Genscan chromosome:NCBI36:17:72226406:72244837:-1
3773244	903376<br>903377<br>903378<br>903379<br>903380<br>903381<br>903382<br>903383<br>903384<br>903385<br>903386<br>903387<br>903388<br>903389<br>903390<br>903391<br>903392<br>903393<br>903394<br>903395<br>903396<br>903397<br>903398	3773245<br>3773246<br>3773247<br>3773248<br>3773249<br>3773250<br>3773251<br>3773252<br>3773253<br>3773254<br>3773255<br>3773256<br>3773257<br>3773258<br>3773259<br>3773260<br>3773261<br>3773262<br>3773263<br>3773264<br>3773265<br>3773266<br>3773267	core<br>core<br>core<br>extended<br>extended<br>extended<br>extended<br>core<br>core<br>core<br>core<br>core<br>core<br>core<br>core<br>extended<br>full<br>core<br>full<br>full<br>extended<br>full<br>full	17	75525146	75624279	-	NM_019020<br>BC001525<br>AL157485<br>AL512709<br>CR936670<br>ENSESTT00000044716<br>ENSESTT00000044717<br>ENSESTT00000044718<br>ENSESTT00000044719<br>ENSESTT00000044720<br>ENSESTT00000044721<br>ENSESTT00000044722<br>ENSESTT00000044723<br>ENSESTT00000044724<br>ENST00000310924<br>ENST00000340848<br>GENSCAN00000040687<br>GENSCAN00000029393	Homo sapiens TBC1 domain family, member 16 (TBC1D16), mRNA.<br>Homo sapiens TBC1 domain family, member 16, mRNA (cDNA clone IMAGE:3050107), partial cds.<br>Homo sapiens mRNA; cDNA DKFZp762O207 (from clone DKFZp762O207).<br>Homo sapiens mRNA; cDNA DKFZp762G216 (from clone DKFZp762G216).<br>Homo sapiens mRNA; cDNA DKFZp451F1311 (from clone DKFZp451F1311).<br><br><br><br><br><br><br><br><br><br>cdna:known-ccds chromosome:NCBI36:17:75528416:75624242:-1 gene:ENSG00000167291 CCDS11766.1<br>cdna:known chromosome:NCBI36:17:75526303:75539232:-1 gene:ENSG00000167291<br>cdna:Genscan chromosome:NCBI36:17:75529253:75542981:-1<br>cdna:Genscan chromosome:NCBI36:17:75589304:75601941:-1

	903399<br>903400<br>903401<br>903402<br>903403<br>903404<br>903405<br>903406<br>903407	3773268<br>3773269<br>3773270<br>3773271<br>3773272<br>3773273<br>3773274<br>3773275<br>3773276<br>3773277<br>3773278<br>3773279<br>3773280<br>3773281<br>3773282<br>3773283<br>3773284<br>3773285<br>3773286	extended<br>extended<br>extended<br>extended<br>full<br>full<br>full<br>full<br>extended<br>extended<br>core<br>extended<br>core<br>core<br>core<br>full<br>extended<br>extended<br>extended						
3776504	905249<br>905250<br>905251<br>905252<br>905253<br>905254<br>905255<br>905256<br>905257<br>905258<br>905259<br>905260<br>905261<br>905262<br>905263<br>905264<br>905265<br>905266<br>905267<br>905268<br>905269<br>905270	3776505<br>3776506<br>3776507<br>3776508<br>3776509<br>3776510<br>3776511<br>3776512<br>3776513<br>3776514<br>3776515<br>3776516<br>3776517<br>3776518<br>3776519<br>3776520<br>3776521<br>3776522<br>3776523<br>3776524<br>3776525<br>3776526<br>3776527<br>3776528<br>3776529<br>3776530<br>3776531<br>3776532<br>3776533<br>3776534<br>3776535<br>3776536<br>3776537<br>3776538<br>3776539	extended<br>core<br>extended<br>core<br>extended<br>full<br>core<br>core<br>core<br>extended<br>full<br>full<br>full<br>full<br>extended<br>extended<br>extended<br>core<br>core<br>core<br>core<br>full<br>full<br>core<br>core<br>core<br>core<br>core<br>core<br>core<br>core<br>extended<br>extended<br>extended<br>core	18	3401626	3449278	+	NM_170695<br>NM_174886<br>NM_003244<br>NM_173208<br>NM_173207<br>NM_173211<br>NM_173209<br>NM_173210<br>AK026766<br>ENSESTT00000030542<br>ENSESTT00000030543<br>ENSESTT00000030544<br>ENSESTT00000030545<br>ENSESTT00000030546<br>ENSESTT00000030547<br>ENSESTT00000030548<br>ENSESTT00000030549<br>ENST00000345133<br>ENST00000340165<br>ENST00000343820<br>ENST00000330513<br>GENSCAN00000003867<br>GENSCAN00000003863	Homo sapiens TGFB-induced factor (TALE family homeobox) (TGIF), transcript variant 1, mRNA.<br>Homo sapiens TGFB-induced factor (TALE family homeobox) (TGIF), transcript variant 8, mRNA.<br>Homo sapiens TGFB-induced factor (TALE family homeobox) (TGIF), transcript variant 4, mRNA.<br>Homo sapiens TGFB-induced factor (TALE family homeobox) (TGIF), transcript variant 3, mRNA.<br>Homo sapiens TGFB-induced factor (TALE family homeobox) (TGIF), transcript variant 2, mRNA.<br>Homo sapiens TGFB-induced factor (TALE family homeobox) (TGIF), transcript variant 7, mRNA.<br>Homo sapiens TGFB-induced factor (TALE family homeobox) (TGIF), transcript variant 5, mRNA.<br>Homo sapiens TGFB-induced factor (TALE family homeobox) (TGIF), transcript variant 6, mRNA.<br>Homo sapiens cDNA: FLJ23113 fis, clone LNG07875, highly similar to HSTGIFPRO Homo sapiens mRNA for TGIF protein.<br><br><br><br><br><br><br><br><br>cdna:known-ccds chromosome:NCBI36:18:3437608:3448404:1 gene:ENSG00000177426 CCDS11832.1<br>cdna:known-ccds chromosome:NCBI36:18:3437608:3448404:1 gene:ENSG00000177426 CCDS11835.1<br>cdna:known-ccds chromosome:NCBI36:18:3440031:3448404:1 gene:ENSG00000177426 CCDS11833.1<br>cdna:known-ccds chromosome:NCBI36:18:3441591:3448404:1 gene:ENSG00000177426 CCDS11834.1<br>cdna:Genscan chromosome:NCBI36:18:3396607:3405570:1<br>cdna:Genscan chromosome:NCBI36:18:3439935:3447938:1

		3776540<br>3776541<br>3776542<br>3776543<br>3776544<br>3776545<br>3776546<br>3776547<br>3776548<br>3776549<br>3776550	extended<br>extended<br>extended<br>extended<br>core<br>core<br>core<br>core<br>core<br>extended<br>extended						
3778772	906695<br>906696<br>906697<br>906698<br>906699<br>906700<br>906701<br>906702<br>906703<br>906704<br>906705<br>906706<br>906707<br>906708<br>906709<br>906710<br>906711<br>906712<br>906713	3778773<br>3778774<br>3778775<br>3778776<br>3778777<br>3778778<br>3778779<br>3778780<br>3778781<br>3778782<br>3778783<br>3778784<br>3778785<br>3778786<br>3778787<br>3778788<br>3778789<br>3778790<br>3778791<br>3778792<br>3778793<br>3778794<br>3778795<br>3778796<br>3778797<br>3778798<br>3778799<br>3778800<br>3778801<br>3778802<br>3778803<br>3778804<br>3778805<br>3778806	core<br>core<br>core<br>core<br>core<br>full<br>extended<br>extended<br>full<br>extended<br>core<br>core<br>core<br>core<br>core<br>extended<br>full<br>extended<br>extended<br>extended<br>extended<br>extended<br>extended<br>extended<br>extended<br>extended<br>extended<br>extended<br>core<br>extended<br>full<br>core<br>core<br>core	18	10444553	10478692	+	NM_153000<br>AK126528<br>BC053324<br>ENSESTT00000014907<br>ENSESTT00000014908<br>ENSESTT00000014909<br>ENSESTT00000014910<br>ENST00000355285<br>GENSCAN00000058172	Homo sapiens adenomatosis polyposis coli down-regulated 1 (APCDD1), mRNA.<br>Homo sapiens cDNA FLJ44564 fis, clone UTERU3009259.<br>Homo sapiens adenomatosis polyposis coli down-regulated 1, mRNA (cDNA clone MGC:60008 IMAGE:6644230), complete cds.<br><br><br><br><br>cdna:known-ccds chromosome:NCBI36:18:10444543:10478697:1 gene:ENSG00000154856 CCDS11849.1<br>cdna:Genscan chromosome:NCBI36:18:10444979:10478035:1
3783398	909559<br>909560<br>909561<br>909562<br>909563<br>909564<br>909565<br>909566	3783399<br>3783400<br>3783401<br>3783402<br>3783403<br>3783404<br>3783405<br>3783406	full<br>full<br>full<br>core<br>core<br>core<br>core<br>core	18	27151913	27191594	+	NM_001942<br>ENSESTT00000032400<br>ENSESTT00000032401<br>ENST00000257192	Homo sapiens desmoglein 1 (DSG1), mRNA.<br><br><br>cdna:known-ccds chromosome:NCBI36:18:27152050:27190457:1 gene:ENSG00000134760 CCDS11896.1

	909567<br>909568<br>909569<br>909570<br>909571<br>909572<br>909573<br>909574<br>909575	3783407<br>3783408<br>3783409<br>3783410<br>3783411<br>3783412<br>3783413<br>3783414<br>3783415<br>3783416<br>3783417<br>3783418<br>3783419<br>3783420<br>3783421<br>3783422<br>3783423<br>3783424<br>3783425<br>3783426<br>3783427<br>3783428<br>3783429<br>3783430	core<br>core<br>extended<br>core<br>extended<br>core<br>core<br>core<br>core<br>core<br>core<br>core<br>extended<br>core<br>core<br>core<br>core<br>core<br>core<br>core<br>core<br>core<br>extended<br>extended						
3784208	910024<br>910025<br>910026<br>910027<br>910028<br>910029<br>910030<br>910031<br>910032<br>910033<br>910034<br>910035<br>910036<br>910037<br>910038<br>910039<br>910040<br>910041<br>910042<br>910043<br>910044<br>910045<br>910046<br>910047<br>910048<br>910049<br>910050<br>910051<br>910052<br>910053	3784209<br>3784210<br>3784211<br>3784212<br>3784213<br>3784214<br>3784215<br>3784216<br>3784217<br>3784218<br>3784219<br>3784220<br>3784221<br>3784222<br>3784223<br>3784224<br>3784225<br>3784226<br>3784227<br>3784228<br>3784229<br>3784230<br>3784231<br>3784232<br>3784233<br>3784234<br>3784235<br>3784236<br>3784237<br>3784238	core<br>full<br>extended<br>extended<br>full<br>extended<br>extended<br>extended<br>extended<br>extended<br>extended<br>extended<br>extended<br>full<br>extended<br>full<br>core<br>extended<br>extended<br>extended<br>full<br>full<br>extended<br>extended<br>full<br>extended<br>extended<br>full<br>core<br>full	18	30327279	30828541	+	NM_032980<br>NM_001391<br>NM_001390<br>NM_032975<br>NM_032978<br>NM_032981<br>NM_032979<br>NM_001392<br>BC005300<br>AL833285<br>U46745<br>AF086074<br>ENSESTT00000032151<br>ENSESTT00000032153<br>ENSESTT00000032155<br>ENSESTT00000035519<br>ENSESTT00000035520<br>ENSESTT00000035522<br>ENST00000348997<br>ENST00000269192<br>ENST00000283365<br>ENST00000315456<br>ENST00000269191<br>ENST00000269190<br>GENSCAN00000062615<br>GENSCAN00000001011	Homo sapiens dystrobrevin, alpha (DTNA), transcript variant 6, mRNA.<br>Homo sapiens dystrobrevin, alpha (DTNA), transcript variant 3, mRNA.<br>Homo sapiens dystrobrevin, alpha (DTNA), transcript variant 1, mRNA.<br>Homo sapiens dystrobrevin, alpha (DTNA), transcript variant 2, mRNA.<br>Homo sapiens dystrobrevin, alpha (DTNA), transcript variant 4, mRNA.<br>Homo sapiens dystrobrevin, alpha (DTNA), transcript variant 8, mRNA.<br>Homo sapiens dystrobrevin, alpha (DTNA), transcript variant 5, mRNA.<br>Homo sapiens dystrobrevin, alpha (DTNA), transcript variant 7, mRNA.<br>Homo sapiens dystrobrevin, alpha, mRNA (cDNA clone MGC:12368 IMAGE:3933795), complete cds.<br>Homo sapiens mRNA; cDNA DKFZp451O128 (from clone DKFZp451O128).<br>Human dystrobrevin-beta mRNA, complete cds.<br>Homo sapiens full length insert cDNA clone YZ56G10.<br><br><br><br><br><br><br>cdna:known-ccds chromosome:NCBI36:18:30544234:30701367:1 gene:ENSG00000134769 CCDS11908.1<br>cdna:known-ccds chromosome:NCBI36:18:30652330:30725341:1 gene:ENSG00000134769 CCDS11909.1<br>cdna:known chromosome:NCBI36:18:30327279:30725341:1 gene:ENSG00000134769<br>cdna:known chromosome:NCBI36:18:30327279:30663290:1 gene:ENSG00000134769<br>cdna:known chromosome:NCBI36:18:30544234:30701367:1 gene:ENSG00000134769<br>cdna:known chromosome:NCBI36:18:30544246:30725341:1 gene:ENSG00000134769<br>cdna:Genscan chromosome:NCBI36:18:30799628:30828541:1<br>cdna:Genscan chromosome:NCBI36:18:30552625:30628216:1

	910054	3784239	full						
	910055	3784240	extended						
	910056	3784241	full						
	910057	3784242	full						
	910058	3784243	core						
	910059	3784244	full						
	910060	3784245	extended						
	910061	3784246	full						
	910062	3784247	full						
	910063	3784248	full						
	910064	3784249	core						
	910065	3784250	full						
	910066	3784251	full						
	910067	3784252	core						
	910068	3784253	full						
	910069	3784254	core						
	910070	3784255	extended						
	910071	3784256	extended						
	910072	3784257	extended						
	910073	3784258	extended						
	910074	3784259	core						
	910075	3784260	core						
	910076	3784261	core						
	910077	3784262	core						
	910078	3784263	extended						
	910079	3784264	extended						
	910080	3784265	core						
	910081	3784266	core						
	910082	3784267	core						
	910083	3784268	core						
	910084	3784269	full						
	910085	3784270	core						
	910086	3784271	core						
	910087	3784272	extended						
	910088	3784273	extended						
	910089	3784274	extended						
	910090	3784275	extended						
	910091	3784276	full						
	910092	3784277	extended						
	910093	3784278	core						
	910094	3784279	core						
	910095	3784280	core						
	910096	3784281	full						
	910097	3784282	extended						
	910098	3784283	extended						
	910099	3784284	full						
	910100	3784285	core						
	910101	3784286	core						
	910102	3784287	extended						
	910103	3784288	extended						
	910104	3784289	extended						
	910105	3784290	core						
	910106	3784291	extended						
	910107	3784292	extended						

	910108<br>910109<br>910110<br>910111<br>910112<br>910113<br>910114<br>910115<br>910116<br>910117<br>910118<br>910119<br>910120<br>910121<br>910122<br>910123<br>910124<br>910125<br>910126<br>910127<br>910128	3784293<br>3784294<br>3784295<br>3784296<br>3784297<br>3784298<br>3784299<br>3784300<br>3784301<br>3784302<br>3784303<br>3784304<br>3784305<br>3784306<br>3784307<br>3784308<br>3784309<br>3784310<br>3784311<br>3784312<br>3784313<br>3784314<br>3784315<br>3784316<br>3784317<br>3784318<br>3784319<br>3784320<br>3784321<br>3784322<br>3784323<br>3784324<br>3784325<br>3784326<br>3784327<br>3784328<br>3784329<br>3784330<br>3784331<br>3784332<br>3784333<br>3784334<br>3784335	full<br>core<br>extended<br>extended<br>core<br>full<br>core<br>extended<br>core<br>core<br>core<br>core<br>core<br>core<br>extended<br>extended<br>extended<br>extended<br>full<br>core<br>core<br>core<br>core<br>core<br>full<br>core<br>full<br>core<br>full<br>full<br>core<br>core<br>core<br>core<br>extended<br>full<br>full<br>full<br>full<br>full<br>full<br>full<br>full						
3791782	914938<br>914939<br>914940<br>914941<br>914942<br>914943<br>914944<br>914945<br>914946<br>914947<br>914948	3791783<br>3791784<br>3791785<br>3791786<br>3791787<br>3791788<br>3791789<br>3791790<br>3791791<br>3791792<br>3791793	extended<br>full<br>full<br>full<br>full<br>extended<br>core<br>extended<br>core<br>core<br>core	18	59295141	59323295	+	NM_002639<br>BC020713<br>BX640597<br>ENSESTT00000001363<br>ENSESTT00000057391<br>ENST00000382771<br>ENST00000389255<br>ENST00000389254<br>GENSCAN00000011646<br>GENSCAN00000011645	Homo sapiens serpin peptidase inhibitor, clade B (ovalbumin), member 5 (SERPINB5), mRNA.<br>Homo sapiens serpin peptidase inhibitor, clade B (ovalbumin), member 5, mRNA (cDNA clone MGC:22489 IMAGE:4248249), complete cds.<br>Homo sapiens mRNA; cDNA DKFZp686G03223 (from clone DKFZp686G03223).<br><br><br>cdna:known chromosome:NCBI36:18:59295199:59323297:1 gene:ENSG00000206075<br>cdna:known chromosome:NCBI36:18:59302642:59321935:1 gene:ENSG00000206075<br>cdna:known chromosome:NCBI36:18:59302642:59321935:1 gene:ENSG00000206075<br>cdna:Genscan chromosome:NCBI36:18:59317363:59321935:1<br>cdna:Genscan chromosome:NCBI36:18:59240439:59315613:1

	914949<br>914950<br>914951<br>914952<br>914953<br>914954<br>914955<br>914956<br>914957	3791794<br>3791795<br>3791796<br>3791797<br>3791798<br>3791799<br>3791800<br>3791801<br>3791802<br>3791803<br>3791804<br>3791805<br>3791806<br>3791807<br>3791808<br>3791809<br>3791810<br>3791811<br>3791812<br>3791813<br>3791814	full<br>core<br>core<br>core<br>extended<br>extended<br>extended<br>extended<br>extended<br>extended<br>extended<br>extended<br>extended<br>extended<br>core<br>core<br>extended<br>extended<br>core<br>core<br>core						
3815399	929917<br>929918<br>929919<br>929920<br>929921<br>929922<br>929923<br>929924	3815400<br>3815401<br>3815402<br>3815403<br>3815404<br>3815405<br>3815406<br>3815407<br>3815408<br>3815409<br>3815410<br>3815411<br>3815412<br>3815413<br>3815414<br>3815415	extended<br>core<br>core<br>core<br>full<br>core<br>core<br>core<br>full<br>core<br>core<br>core<br>core<br>core<br>core<br>core	19	977004	990059	+	NM_004368<br>NM_201277<br>AK093630<br>AY927510<br>AY927514<br>ENSESTT00000021874<br>ENST00000263097<br>ENST00000348419<br>GENSCAN00000013300<br>GENSCAN00000009502	Homo sapiens calponin 2 (CNN2), transcript variant 1, mRNA.<br>Homo sapiens calponin 2 (CNN2), transcript variant 2, mRNA.<br>Homo sapiens cDNA FLJ36311 fis, clone THYMU2005046, highly similar to CALPONIN H2, SMOOTH MUSCLE.<br>Homo sapiens mRNA sequence.<br>Homo sapiens mRNA sequence.<br><br>cdna:known-ccds chromosome:NCBI36:19:977298:990060:1 gene:ENSG00000064666 CCDS12053.1<br>cdna:known-ccds chromosome:NCBI36:19:977298:990060:1 gene:ENSG00000064666 CCDS12054.1<br>cdna:Genscan chromosome:NCBI36:19:977661:977723:1<br>cdna:Genscan chromosome:NCBI36:19:986955:988899:1
3820414	932804<br>932805<br>932806<br>932807<br>932808<br>932809	3820415<br>3820416<br>3820417<br>3820418<br>3820419<br>3820420<br>3820421<br>3820422<br>3820423<br>3820424<br>3820425<br>3820426<br>3820427<br>3820428<br>3820429<br>3820430	extended<br>core<br>core<br>core<br>extended<br>extended<br>core<br>extended<br>core<br>extended<br>core<br>extended<br>core<br>core<br>core<br>extended	19	10223597	10231716	+	NM_015956<br>NM_146387<br>NM_146388<br>CR611784<br>CR624166<br>ENSESTT00000043694<br>ENSESTT00000043695<br>ENST00000253099<br>ENST00000307422<br>GENSCAN00000015606	Homo sapiens mitochondrial ribosomal protein L4 (MRPL4), nuclear gene encoding mitochondrial protein, transcript variant 1, mRNA.<br>Homo sapiens mitochondrial ribosomal protein L4 (MRPL4), nuclear gene encoding mitochondrial protein, transcript variant 2, mRNA.<br>Homo sapiens mitochondrial ribosomal protein L4 (MRPL4), nuclear gene encoding mitochondrial protein, transcript variant 3, mRNA.<br>full-length cDNA clone CS0DI043YJ13 of Placenta Cot 25-normalized of Homo sapiens (human).<br>full-length cDNA clone CS0DC007YA18 of Neuroblastoma Cot 25-normalized of Homo sapiens (human).<br><br><br>cdna:known-ccds chromosome:NCBI36:19:10223640:10231489:1 gene:ENSG00000105364 CCDS12230.1<br>cdna:novel chromosome:NCBI36:19:10223640:10231489:1 gene:ENSG00000105364<br>cdna:Genscan chromosome:NCBI36:19:10223930:10231489:1

		3820431<br>3820432<br>3820433<br>3820434<br>3820435<br>3820436<br>3820437<br>3820438<br>3820439<br>3820440	core<br>core<br>core<br>core<br>core<br>core<br>core<br>core<br>core<br>core						
3821263	933284<br>933285<br>933286<br>933287<br>933288<br>933289<br>933290<br>933291<br>933292<br>933293<br>933294<br>933295<br>933296<br>933297<br>933298<br>933299<br>933300<br>933301<br>933302	3821264<br>3821265<br>3821266<br>3821267<br>3821268<br>3821269<br>3821270<br>3821271<br>3821272<br>3821273<br>3821274<br>3821275<br>3821276<br>3821277<br>3821278<br>3821279<br>3821280<br>3821281<br>3821282<br>3821283<br>3821284<br>3821285<br>3821286	full<br>core<br>core<br>extended<br>extended<br>core<br>extended<br>extended<br>core<br>core<br>core<br>core<br>core<br>core<br>core<br>core<br>core<br>core<br>full<br>full<br>extended<br>full<br>full	19	11502173	11538332	+	NM_001299<br>ENSESTT00000055953<br>ENSESTT00000055954<br>ENSESTT00000055955<br>ENST00000252456<br>GENSCAN00000044395<br>GENSCAN00000066495	Homo sapiens calponin 1, basic, smooth muscle (CNN1), mRNA.<br><br><br><br>cdna:known-ccds chromosome:NCBI36:19:11510579:11522138:1 gene:ENSG00000130176 CCDS12263.1<br>cdna:Genscan chromosome:NCBI36:19:11495251:11523578:1<br>cdna:Genscan chromosome:NCBI36:19:11531015:11538332:1
3830216	938551<br>938552<br>938553<br>938554<br>938555<br>938556<br>938557<br>938558<br>938559<br>938560<br>938561<br>938562	3830217<br>3830218<br>3830219<br>3830220<br>3830221<br>3830222<br>3830223<br>3830224<br>3830225<br>3830226<br>3830227<br>3830228<br>3830229<br>3830230<br>3830231<br>3830232<br>3830233<br>3830234<br>3830235<br>3830236	core<br>core<br>core<br>core<br>core<br>extended<br>core<br>extended<br>core<br>extended<br>core<br>core<br>core<br>core<br>core<br>core<br>extended<br>extended<br>core<br>core	19	40337411	40352625	+	NM_014164<br>NM_144779<br>AF177940<br>AY358991<br>BX648809<br>BC018798<br>ENSESTT00000029497<br>ENSESTT00000029498<br>ENST00000342879<br>GENSCAN00000000389	Homo sapiens FXYD domain containing ion transport regulator 5 (FXYD5), transcript variant 2, mRNA.<br>Homo sapiens FXYD domain containing ion transport regulator 5 (FXYD5), transcript variant 1, mRNA.<br>Homo sapiens ion channel homolog (IWU1) mRNA, complete cds.<br>Homo sapiens clone DNA108936 FXYD5 (UNQ2561) mRNA, complete cds.<br>Homo sapiens mRNA; cDNA DKFZp686M18208 (from clone DKFZp686M18208).<br>Homo sapiens FXYD domain containing ion transport regulator 5, mRNA (cDNA clone IMAGE:3504451).<br><br><br>cdna:known-ccds chromosome:NCBI36:19:40337467:40352625:1 gene:ENSG00000089327 CCDS12447.1<br>cdna:Genscan chromosome:NCBI36:19:40322824:40338354:1

3833992	940722<br>940723<br>940724<br>940725<br>940726<br>940727<br>940728<br>940729<br>940730<br>940731<br>940732<br>940733<br>940734<br>940735<br>940736<br>940737<br>940738<br>940739<br>940740<br>940741<br>940742<br>940743<br>940744	3833993<br>3833994<br>3833995<br>3833996<br>3833997<br>3833998<br>3833999<br>3834000<br>3834001<br>3834002<br>3834003<br>3834004<br>3834005<br>3834006<br>3834007<br>3834008<br>3834009<br>3834010<br>3834011<br>3834012<br>3834013<br>3834014<br>3834015<br>3834016<br>3834017<br>3834018<br>3834019<br>3834020<br>3834021<br>3834022<br>3834023<br>3834024<br>3834025<br>3834026<br>3834027<br>3834028<br>3834029<br>3834030<br>3834031<br>3834032<br>3834033<br>3834034<br>3834035	full<br>full<br>full<br>full<br>full<br>full<br>full<br>full<br>full<br>full<br>full<br>full<br>full<br>full<br>full<br>full<br>full<br>full<br>full<br>core<br>core<br>core<br>core<br>core<br>core<br>core<br>core<br>core<br>core<br>full<br>full<br>core<br>full<br>full<br>core<br>core<br>core<br>core<br>core<br>core<br>core<br>core<br>core	19	46332467	46405278	+	NM_030622<br>ENSESTT00000008181<br>ENSESTT00000008182<br>ENST00000310054<br>ENST00000301173<br>GENSCAN00000035095<br>GENSCAN00000037061	Homo sapiens cytochrome P450, family 2, subfamily S, polypeptide 1 (CYP2S1), mRNA.<br><br><br>cdna:known-ccds chromosome:NCBI36:19:46390955:46405283:1 gene:ENSG00000167600 CCDS12573.1<br>cdna:known chromosome:NCBI36:19:46390955:46404515:1 gene:ENSG00000167600<br>cdna:Genscan chromosome:NCBI36:19:46313934:46335098:1<br>cdna:Genscan chromosome:NCBI36:19:46391010:46405167:1
3838425	943247<br>943248<br>943249<br>943250<br>943251<br>943252<br>943253<br>943254<br>943255<br>943256	3838426<br>3838427<br>3838428<br>3838429<br>3838430<br>3838431<br>3838432<br>3838433<br>3838434<br>3838435<br>3838436	extended<br>extended<br>extended<br>extended<br>core<br>core<br>core<br>core<br>core<br>extended<br>full	19	54556731	54570185	+	NM_014419<br>AF177398<br>ENSESTT00000010393<br>ENSESTT00000010394<br>ENSESTT00000010395<br>ENST00000221498<br>GENSCAN00000036904<br>GENSCAN00000036908	Homo sapiens dickkopf-like 1 (soggy) (DKKL1), mRNA.<br>Homo sapiens soggy-1 protein (SGY-1) mRNA, complete cds.<br><br><br><br>cdna:known-ccds chromosome:NCBI36:19:54558854:54570183:1 gene:ENSG00000104901 CCDS12762.1<br>cdna:Genscan chromosome:NCBI36:19:54556945:54565532:1<br>cdna:Genscan chromosome:NCBI36:19:54569795:54570097:1

		3838437<br>3838438<br>3838439<br>3838440	full<br>full<br>core<br>core						
3839346	943764<br>943765<br>943766<br>943767<br>943768<br>943769<br>943770	3839347<br>3839348<br>3839349<br>3839350<br>3839351<br>3839352<br>3839353<br>3839354<br>3839355<br>3839356<br>3839357<br>3839358<br>3839359	extended<br>extended<br>core<br>core<br>core<br>core<br>core<br>extended<br>core<br>core<br>core<br>extended<br>extended	19	55614962	55626382	+	NM_003121<br>AK025419<br>ENSESTT00000004335<br>ENSESTT00000004336<br>ENST00000270632<br>GENSCAN00000031985	Homo sapiens Spi-B transcription factor (Spi-1/PU.1 related) (SPIB), mRNA.<br>Homo sapiens cDNA: FLJ21766 fis, clone COLF7179.<br><br><br>cdna:known chromosome:NCBI36:19:55614028:55624058:1 gene:ENSG00000142539<br>cdna:Genscan chromosome:NCBI36:19:55614032:55623405:1
3845909	947335<br>947336<br>947337<br>947338<br>947339<br>947340<br>947341<br>947342<br>947343<br>947344<br>947345<br>947346<br>947347	3845910<br>3845911<br>3845912<br>3845913<br>3845914<br>3845915<br>3845916<br>3845917<br>3845918<br>3845919<br>3845920<br>3845921<br>3845922<br>3845923<br>3845924<br>3845925<br>3845926<br>3845927<br>3845928<br>3845929<br>3845930<br>3845931<br>3845932	core<br>core<br>core<br>core<br>extended<br>core<br>core<br>core<br>core<br>core<br>core<br>core<br>extended<br>core<br>full<br>core<br>core<br>core<br>core<br>core<br>core<br>core<br>core	19	2379179	2407958	-	NM_032737<br>ENSESTT00000017138<br>ENSESTT00000017139<br>ENSESTT00000017140<br>ENST00000325327<br>GENSCAN00000000132<br>GENSCAN00000006816	Homo sapiens lamin B2 (LMNB2), mRNA.<br><br><br><br>cdna:known-ccds chromosome:NCBI36:19:2379165:2407958:-1 gene:ENSG00000176619 CCDS12090.1<br>cdna:Genscan chromosome:NCBI36:19:2402793:2407871:-1<br>cdna:Genscan chromosome:NCBI36:19:2381909:2395538:-1
3850069	949811<br>949812<br>949813<br>949814<br>949815<br>949816<br>949817<br>949818<br>949819<br>949820<br>949821<br>949822<br>949823	3850070<br>3850071<br>3850072<br>3850073<br>3850074<br>3850075<br>3850076<br>3850077<br>3850078<br>3850079<br>3850080<br>3850081<br>3850082	core<br>core<br>extended<br>core<br>extended<br>extended<br>core<br>core<br>core<br>core<br>extended<br>core<br>extended	19	10105029	10172848	-	NM_001379<br>AF290894<br>AK122759<br>CR598167<br>AY927518<br>ENSESTT00000043764<br>ENSESTT00000043765<br>ENSESTT00000043766<br>ENST00000340748<br>ENST00000359526<br>GENSCAN00000010328	Homo sapiens DNA (cytosine-5-)-methyltransferase 1 (DNMT1), mRNA.<br>Homo sapiens DNA (cytosine-5) methyltransferase isoform 1C (DNMT1) mRNA, partial sequence, alternatively spliced.<br>Homo sapiens cDNA FLJ16293 fis, clone OCBBF2021518, highly similar to DNA (CYTOSINE-5)-METHYLTRANSFERASE (EC 2.1.1.37).<br>full-length cDNA clone CS0DI012YG19 of Placenta Cot 25-normalized of Homo sapiens (human).<br>Homo sapiens mRNA sequence.<br><br><br><br>cdna:known-ccds chromosome:NCBI36:19:10105023:10166811:-1 gene:ENSG00000130816 CCDS12228.1<br>cdna:known chromosome:NCBI36:19:10105023:10166811:-1 gene:ENSG00000130816<br>cdna:Genscan chromosome:NCBI36:19:10087151:10113896:-1

	949824	3850083	core						
	949825	3850084	core						
	949826	3850085	core						
	949827	3850086	extended						
	949828	3850087	extended						
	949829	3850088	core						
	949830	3850089	extended						
	949831	3850090	core						
	949832	3850091	extended						
	949833	3850092	core						
	949834	3850093	core						
	949835	3850094	extended						
	949836	3850095	extended						
	949837	3850096	core						
	949838	3850097	extended						
	949839	3850098	core						
	949840	3850099	extended						
	949841	3850100	extended						
	949842	3850101	core						
	949843	3850102	core						
	949844	3850103	core						
	949845	3850104	full						
	949846	3850105	core						
	949847	3850106	extended						
	949848	3850107	core						
	949849	3850108	core						
	949850	3850109	core						
	949851	3850110	core						
	949852	3850111	core						
	949853	3850112	core						
	949854	3850113	core						
	949855	3850114	core						
	949856	3850115	core						
	949857	3850116	core						
	949858	3850117	core						
	949859	3850118	core						
	949860	3850119	extended						
	949861	3850120	core						
	949862	3850121	extended						
	949863	3850122	core						
	949864	3850123	extended						
	949865	3850124	extended						
	949866	3850125	core						
	949867	3850126	extended						
	949868	3850127	extended						
	949869	3850128	core						
		3850129	core						
		3850130	full						
		3850131	full						
		3850132	full						
		3850133	core						
		3850134	core						
		3850135	core						
		3850136	full						

		3850137<br>3850138<br>3850139<br>3850140<br>3850141<br>3850142<br>3850143<br>3850144<br>3850145<br>3850146<br>3850147<br>3850148<br>3850149<br>3850150<br>3850151<br>3850152<br>3850153<br>3850154<br>3850155<br>3850156	core<br>core<br>extended<br>core<br>core<br>core<br>core<br>extended<br>full<br>extended<br>extended<br>full<br>extended<br>extended<br>core<br>core<br>core<br>extended<br>extended<br>extended						
3851651	950696<br>950697<br>950698<br>950699<br>950700<br>950701<br>950702<br>950703<br>950704<br>950705<br>950706<br>950707<br>950708<br>950709<br>950710<br>950711<br>950712<br>950713<br>950714<br>950715<br>950716<br>950717<br>950718<br>950719<br>950720<br>950721<br>950722<br>950723<br>950724<br>950725<br>950726<br>950727<br>950728<br>950729	3851652<br>3851653<br>3851654<br>3851655<br>3851656<br>3851657<br>3851658<br>3851659<br>3851660<br>3851661<br>3851662<br>3851663<br>3851664<br>3851665<br>3851666<br>3851667<br>3851668<br>3851669<br>3851670<br>3851671<br>3851672<br>3851673<br>3851674<br>3851675<br>3851676<br>3851677<br>3851678<br>3851679<br>3851680<br>3851681<br>3851682<br>3851683<br>3851684<br>3851685	extended<br>core<br>core<br>core<br>core<br>core<br>core<br>core<br>extended<br>core<br>extended<br>core<br>extended<br>core<br>extended<br>core<br>core<br>extended<br>core<br>core<br>core<br>full<br>core<br>extended<br>extended<br>core<br>extended<br>extended<br>core<br>core<br>extended<br>extended<br>core<br>core	19	12670912	12695776	-	NM_013433<br>NR_002751<br>AB210011<br>AF007748<br>AF019039<br>AK022217<br>AK056183<br>CR626762<br>BC063679<br>ENSESTT00000046112<br>ENSESTT00000046113<br>ENSESTT00000046114<br>ENSESTT00000046115<br>ENSESTT00000046116<br>ENSESTT00000046117<br>ENSESTT00000046118<br>ENST00000356861<br>GENSCAN00000043248	Homo sapiens transportin 2 (importin 3, karyopherin beta 2b) (TNPO2), mRNA.<br>Homo sapiens small nucleolar RNA, C/D box 41 (SNORD41) on chromosome 19.<br>Homo sapiens mRNA for TNPO2 variant protein, partial cds, clone: fh13310.<br>Homo sapiens karyopherin beta2b homolog mRNA, complete cds.<br>Homo sapiens transportin2 mRNA, complete cds.<br>Homo sapiens cDNA FLJ12155 fis, clone MAMMA1000472.<br>Homo sapiens cDNA FLJ31621 fis, clone NT2RI2003205, highly similar to Homo sapiens transportin2 mRNA.<br>full-length cDNA clone CS0DC001YB11 of Neuroblastoma Cot 25-normalized of Homo sapiens (human).<br>Homo sapiens cDNA clone IMAGE:4358892, **** WARNING: chimeric clone ****.<br><br><br><br><br><br><br><br>cdna:known chromosome:NCBI36:19:12672967:12694386:-1 gene:ENSG00000105576<br>cdna:Genscan chromosome:NCBI36:19:12672974:12693067:-1

	950730<br>950731<br>950732	3851686<br>3851687<br>3851688<br>3851689<br>3851690<br>3851691<br>3851692<br>3851693<br>3851694<br>3851695<br>3851696<br>3851697<br>3851698<br>3851699<br>3851700<br>3851701<br>3851702	core<br>core<br>core<br>core<br>extended<br>extended<br>core<br>core<br>core<br>full<br>core<br>core<br>core<br>core<br>core<br>extended<br>extended						
3852381	951113<br>951114<br>951115<br>951116<br>951117<br>951118<br>951119<br>951120<br>951121<br>951122<br>951123	3852382<br>3852383<br>3852384<br>3852385<br>3852386<br>3852387<br>3852388<br>3852389<br>3852390<br>3852391<br>3852392<br>3852393<br>3852394<br>3852395<br>3852396<br>3852397<br>3852398<br>3852399<br>3852400<br>3852401<br>3852402	extended<br>extended<br>extended<br>extended<br>extended<br>extended<br>core<br>core<br>core<br>extended<br>core<br>core<br>core<br>core<br>core<br>core<br>extended<br>core<br>extended<br>extended<br>extended	19	13903002	13925183	-	NM_024825<br>AK027100<br>BC052610<br>ENSESTT00000052598<br>ENSESTT00000052599<br>ENSESTT00000052600<br>ENSESTT00000052601<br>ENSESTT00000052602<br>ENST00000254320<br>ENST00000339560<br>GENSCAN00000034926<br>GENSCAN00000034927	Homo sapiens hypothetical protein FLJ23447 (FLJ23447), mRNA.<br>Homo sapiens cDNA: FLJ23447 fis, clone HSI03346.<br>Homo sapiens cDNA clone IMAGE:6255997, partial cds.<br><br><br><br><br><br>cdna:known-ccds chromosome:NCBI36:19:13904167:13910289:-1 gene:ENSG00000132000 CCDS12300.1<br>cdna:known chromosome:NCBI36:19:13904168:13925193:-1 gene:ENSG00000132000<br>cdna:Genscan chromosome:NCBI36:19:13903550:13908379:-1<br>cdna:Genscan chromosome:NCBI36:19:13909559:13910015:-1
3853108	951512<br>951513<br>951514<br>951515<br>951516<br>951517<br>951518<br>951519<br>951520<br>951521<br>951522<br>951523<br>951524<br>951525<br>951526	3853109<br>3853110<br>3853111<br>3853112<br>3853113<br>3853114<br>3853115<br>3853116<br>3853117<br>3853118<br>3853119<br>3853120<br>3853121<br>3853122<br>3853123	full<br>extended<br>extended<br>extended<br>extended<br>core<br>core<br>core<br>core<br>core<br>core<br>core<br>core<br>core<br>core	19	15124493	15172792	-	NM_000435<br>DQ156541<br>DQ156542<br>ENSESTT00000032947<br>ENSESTT00000032948<br>ENSESTT00000032949<br>ENSESTT00000032950<br>ENSESTT00000032951<br>ENSESTT00000032952<br>ENST00000263388<br>GENSCAN00000066104<br>GENSCAN00000015517	Homo sapiens Notch homolog 3 (Drosophila) (NOTCH3), mRNA.<br>Homo sapiens notch3-like (NOTCH3) pseudogene mRNA, partial sequence.<br>Homo sapiens notch3-like (NOTCH3) pseudogene mRNA, partial sequence.<br><br><br><br><br><br><br>cdna:known-ccds chromosome:NCBI36:19:15131445:15172792:-1 gene:ENSG00000074181 CCDS12326.1<br>cdna:Genscan chromosome:NCBI36:19:15172599:15172716:-1<br>cdna:Genscan chromosome:NCBI36:19:15132473:15168156:-1

	951527<br>951528<br>951529<br>951530<br>951531<br>951532<br>951533<br>951534<br>951535<br>951536<br>951537<br>951538<br>951539<br>951540<br>951541<br>951542<br>951543<br>951544<br>951545<br>951546<br>951547<br>951548<br>951549<br>951550<br>951551<br>951552<br>951553	3853124<br>3853125<br>3853126<br>3853127<br>3853128<br>3853129<br>3853130<br>3853131<br>3853132<br>3853133<br>3853134<br>3853135<br>3853136<br>3853137<br>3853138<br>3853139<br>3853140<br>3853141<br>3853142<br>3853143<br>3853144<br>3853145<br>3853146<br>3853147<br>3853148<br>3853149<br>3853150<br>3853151<br>3853152<br>3853153<br>3853154<br>3853155<br>3853156<br>3853157<br>3853158<br>3853159<br>3853160<br>3853161<br>3853162<br>3853163<br>3853164<br>3853165<br>3853166<br>3853167<br>3853168<br>3853169<br>3853170<br>3853171<br>3853172<br>3853173<br>3853174<br>3853175	core<br>extended<br>core<br>core<br>core<br>core<br>core<br>core<br>full<br>core<br>core<br>core<br>core<br>core<br>core<br>core<br>core<br>core<br>core<br>core<br>extended<br>core<br>core<br>core<br>core<br>full<br>core<br>core<br>core<br>core<br>core<br>full<br>full<br>core<br>core<br>core<br>core<br>core<br>core<br>core<br>core<br>full<br>core<br>core<br>core<br>core<br>core<br>extended<br>full<br>core<br>core<br>core						
3855218	952735<br>952736	3855219<br>3855220	core<br>core	19	18754583	18763114	-	NM_000095<br>AK074508	Homo sapiens cartilage oligomeric matrix protein (COMP), mRNA.<br>Homo sapiens cDNA FLJ90027 fis, clone HEMBA1001228, highly similar to Human germline oligomeric matrix protein

	952737<br>952738<br>952739<br>952740<br>952741<br>952742<br>952743<br>952744<br>952745<br>952746<br>952747<br>952748<br>952749<br>952750<br>952751<br>952752<br>952753<br>952754	3855221<br>3855222<br>3855223<br>3855224<br>3855225<br>3855226<br>3855227<br>3855228<br>3855229<br>3855230<br>3855231<br>3855232<br>3855233<br>3855234<br>3855235<br>3855236<br>3855237<br>3855238<br>3855239<br>3855240<br>3855241<br>3855242<br>3855243<br>3855244<br>3855245	core<br>core<br>core<br>full<br>core<br>core<br>core<br>full<br>core<br>core<br>core<br>core<br>core<br>core<br>core<br>core<br>core<br>core<br>core<br>core<br>core<br>core<br>core<br>core<br>core					ENST00000222271<br>GENSCAN00000025753	(COMP) mRNA.<br>cdna:known-ccds chromosome:NCBI36:19:18754584:18763114:-1 gene:ENSG00000105664 CCDS12385.1<br>cdna:Genscan chromosome:NCBI36:19:18754725:18763078:-1
3859761	955436<br>955437<br>955438<br>955439<br>955440<br>955441<br>955442<br>955443<br>955444<br>955445<br>955446<br>955447<br>955448<br>955449<br>955450<br>955451<br>955452<br>955453<br>955454<br>955455<br>955456	3859762<br>3859763<br>3859764<br>3859765<br>3859766<br>3859767<br>3859768<br>3859769<br>3859770<br>3859771<br>3859772<br>3859773<br>3859774<br>3859775<br>3859776<br>3859777<br>3859778<br>3859779<br>3859780<br>3859781<br>3859782<br>3859783<br>3859784<br>3859785<br>3859786<br>3859787<br>3859788<br>3859789<br>3859790	full<br>core<br>extended<br>core<br>extended<br>extended<br>extended<br>core<br>extended<br>extended<br>core<br>extended<br>core<br>extended<br>core<br>full<br>core<br>full<br>core<br>core<br>core<br>core<br>core<br>extended<br>extended<br>core<br>extended<br>core<br>extended	19	40677867	40696394	-	NM_033317<br>NM_001035516<br>AK096215<br>AK125695<br>AY358412<br>AY789699<br>AY789701<br>BC004493<br>ENSESTT00000029532<br>ENSESTT00000029533<br>ENSESTT00000029534<br>ENSESTT00000029535<br>ENSESTT00000029536<br>ENSESTT00000029537<br>ENSESTT00000029538<br>ENSESTT00000029539<br>ENST00000339686<br>ENST00000379075<br>ENST00000379074<br>ENST00000379073<br>GENSCAN00000027868	Homo sapiens dermokine (DMKN), transcript variant 2, mRNA.<br>Homo sapiens dermokine (DMKN), transcript variant 1, mRNA.<br>Homo sapiens cDNA FLJ38896 fis, clone NOVAR2000352.<br>Homo sapiens cDNA FLJ43707 fis, clone TESOP2001865.<br>Homo sapiens clone DNA59212 KFQG729 (UNQ729) mRNA, complete cds.<br>Homo sapiens dermokine-delta 2 mRNA, complete cds, alternatively spliced.<br>Homo sapiens dermokine-delta 4 mRNA, complete cds, alternatively spliced.<br>Homo sapiens dermokine, mRNA (cDNA clone IMAGE:3690018), complete cds.<br>cdna:known-ccds chromosome:NCBI36:19:40679963:40696394:-1 gene:ENSG00000161249 CCDS12463.1<br>cdna:known chromosome:NCBI36:19:40679963:40693212:-1 gene:ENSG00000161249<br>cdna:known chromosome:NCBI36:19:40679963:40684653:-1 gene:ENSG00000161249<br>cdna:known chromosome:NCBI36:19:40686049:40696400:-1 gene:ENSG00000161249<br>cdna:Genscan chromosome:NCBI36:19:40681458:40692994:-1

		3859791<br>3859792<br>3859793<br>3859794<br>3859795<br>3859796<br>3859797<br>3859798<br>3859799<br>3859800<br>3859801<br>3859802<br>3859803<br>3859804<br>3859805<br>3859806<br>3859807<br>3859808	core<br>extended<br>core<br>full<br>core<br>extended<br>extended<br>full<br>full<br>core<br>core<br>full<br>core<br>core<br>core<br>core<br>core<br>core						
3862167	956756<br>956757<br>956758<br>956759<br>956760<br>956761<br>956762<br>956763<br>956764<br>956765<br>956766<br>956767<br>956768	3862168<br>3862169<br>3862170<br>3862171<br>3862172<br>3862173<br>3862174<br>3862175<br>3862176<br>3862177<br>3862178<br>3862179<br>3862180<br>3862181<br>3862182<br>3862183<br>3862184<br>3862185	core<br>extended<br>core<br>core<br>full<br>core<br>extended<br>extended<br>core<br>core<br>extended<br>extended<br>core<br>core<br>core<br>core<br>core<br>extended	19	45016950	45028874	-	NM_001436<br>X56597<br>ENSESTT00000004231<br>ENSESTT00000004232<br>ENST00000221801<br>GENSCAN00000012956	Homo sapiens fibrillarin (FBL), mRNA.<br>Human humFib mRNA for fibrillarin.<br><br><br>cdna:known-ccds chromosome:NCBI36:19:45016938:45028848:-1 gene:ENSG00000105202 CCDS12545.1<br>cdna:Genscan chromosome:NCBI36:19:45008195:45023267:-1
3868828	960520<br>960521<br>960522<br>960523<br>960524<br>960525<br>960526<br>960527<br>960528<br>960529	3868829<br>3868830<br>3868831<br>3868832<br>3868833<br>3868834<br>3868835<br>3868836<br>3868837<br>3868838<br>3868839<br>3868840	extended<br>core<br>core<br>core<br>core<br>extended<br>core<br>core<br>core<br>core<br>core<br>extended	19	56207817	56215223	-	NM_145888<br>NM_002776<br>AK026045<br>ENSESTT00000012576<br>ENSESTT00000012577<br>ENSESTT00000012578<br>ENST00000309958<br>ENST00000358789	Homo sapiens kallikrein 10 (KLK10), transcript variant 2, mRNA.<br>Homo sapiens kallikrein 10 (KLK10), transcript variant 1, mRNA.<br>Homo sapiens cDNA: FLJ22392 fis, clone HRC07868.<br><br><br><br>cdna:known-ccds chromosome:NCBI36:19:56209338:56215094:-1 gene:ENSG00000129451 CCDS12817.1<br>cdna:novel chromosome:NCBI36:19:56209338:56215094:-1 gene:ENSG00000129451
3871192	961787<br>961788<br>961789<br>961790<br>961791	3871193<br>3871194<br>3871195<br>3871196<br>3871197	core<br>core<br>core<br>core<br>core	19	60384427	60427858	-	NM_002842<br>NM_014931<br>AK097257<br>BC113443<br>ENSESTT00000007329	Homo sapiens protein tyrosine phosphatase, receptor type, H (PTPRH), mRNA.<br>Homo sapiens SAPS domain family, member 1 (SAPS1), mRNA.<br>Homo sapiens cDNA FLJ39938 fis, clone SPLEN2022162, highly similar to Homo sapiens transmembrane-type protein tyrosine phosphatase H (PTPRH) gene.<br>Homo sapiens SAPS domain family, member 1, mRNA (cDNA clone MGC:142003 IMAGE:8322495), complete cds.

	961792<br>961793<br>961794<br>961795<br>961796<br>961797<br>961798<br>961799<br>961800<br>961801<br>961802<br>961803<br>961804<br>961805<br>961806<br>961807<br>961808<br>961809<br>961810<br>961811<br>961812<br>961813<br>961814<br>961815<br>961816	3871198<br>3871199<br>3871200<br>3871201<br>3871202<br>3871203<br>3871204<br>3871205<br>3871206<br>3871207<br>3871208<br>3871209<br>3871210<br>3871211<br>3871212<br>3871213<br>3871214<br>3871215<br>3871216<br>3871217<br>3871218<br>3871219<br>3871220<br>3871221<br>3871222<br>3871223<br>3871224<br>3871225<br>3871226<br>3871227<br>3871228<br>3871229<br>3871230<br>3871231<br>3871232<br>3871233<br>3871234<br>3871235<br>3871236<br>3871237<br>3871238	full<br>core<br>core<br>core<br>core<br>core<br>core<br>core<br>extended<br>core<br>core<br>extended<br>core<br>core<br>core<br>core<br>core<br>extended<br>core<br>core<br>extended<br>extended<br>core<br>core<br>core<br>extended<br>full<br>full<br>full<br>full<br>full<br>full<br>full<br>full<br>extended<br>extended<br>extended<br>extended<br>extended<br>extended<br>extended					ENSESTT00000007331<br>ENSESTT00000007332<br>ENST00000376350<br>ENST00000263434<br>ENST00000327021<br>GENSCAN00000017481<br>GENSCAN00000017466	 <br><br>cdna:known chromosome:NCBI36:19:60384428:60412654:-1 gene:ENSG00000080031<br>cdna:known chromosome:NCBI36:19:60384428:60412654:-1 gene:ENSG00000080031<br>cdna:known chromosome:NCBI36:19:60432960:60462175:-1 gene:ENSG00000105063<br>cdna:Genscan chromosome:NCBI36:19:60355014:60412613:-1<br>cdna:Genscan chromosome:NCBI36:19:60419663:60450469:-1
3873160	962838<br>962839<br>962840<br>962841<br>962842<br>962843<br>962844<br>962845<br>962846<br>962847<br>962848<br>962849<br>962850	3873161<br>3873162<br>3873163<br>3873164<br>3873165<br>3873166<br>3873167<br>3873168<br>3873169<br>3873170<br>3873171<br>3873172<br>3873173	extended<br>full<br>extended<br>full<br>core<br>core<br>core<br>core<br>extended<br>extended<br>extended<br>core<br>core	20	291499	326200	+	NM_021158<br>AJ697940<br>BC019363<br>ENSESTT00000010636<br>ENSESTT00000010637<br>ENSESTT00000010638<br>ENSESTT00000010639<br>ENST00000217233<br>ENST00000382266<br>GENSCAN00000064737	Homo sapiens tribbles homolog 3 (Drosophila) (TRIB3), mRNA.<br>Homo sapiens partial mRNA for Neuronal cell death Inducible Putative Kinase (NIPK gene), 5'UTR, isoform 1B4.<br>Homo sapiens tribbles homolog 3 (Drosophila), mRNA (cDNA clone MGC:854 IMAGE:3528490), complete cds.<br><br><br><br><br>cdna:known-ccds chromosome:NCBI36:20:309273:326205:1 gene:ENSG00000101255 CCDS12997.1<br>cdna:known chromosome:NCBI36:20:309487:326203:1 gene:ENSG00000101255<br>cdna:Genscan chromosome:NCBI36:20:310300:325334:1

	962851	3873174<br>3873175<br>3873176<br>3873177<br>3873178<br>3873179<br>3873180<br>3873181<br>3873182	core<br>core<br>core<br>core<br>core<br>core<br>core<br>core<br>core						
3881282	967901<br>967902<br>967903<br>967904<br>967905<br>967906<br>967907<br>967908<br>967909<br>967910<br>967911<br>967912<br>967913<br>967914<br>967915<br>967916<br>967917<br>967918<br>967919<br>967920<br>967921<br>967922<br>967923<br>967924<br>967925<br>967926<br>967927<br>967928<br>967929<br>967930<br>967931<br>967932<br>967933<br>967934<br>967935<br>967936<br>967937<br>967938<br>967939	3881283<br>3881284<br>3881285<br>3881286<br>3881287<br>3881288<br>3881289<br>3881290<br>3881291<br>3881292<br>3881293<br>3881294<br>3881295<br>3881296<br>3881297<br>3881298<br>3881299<br>3881300<br>3881301<br>3881302<br>3881303<br>3881304<br>3881305<br>3881306<br>3881307<br>3881308<br>3881309<br>3881310<br>3881311<br>3881312<br>3881313<br>3881314<br>3881315<br>3881316<br>3881317<br>3881318<br>3881319<br>3881320<br>3881321<br>3881322<br>3881323<br>3881324<br>3881325<br>3881326<br>3881327	full<br>extended<br>full<br>full<br>full<br>extended<br>core<br>core<br>core<br>extended<br>extended<br>extended<br>core<br>extended<br>extended<br>extended<br>core<br>core<br>core<br>core<br>extended<br>extended<br>full<br>extended<br>extended<br>core<br>extended<br>extended<br>extended<br>extended<br>extended<br>extended<br>extended<br>extended<br>extended<br>extended<br>extended<br>extended<br>extended<br>extended<br>extended<br>core<br>extended<br>core<br>core	20	29548493	29623431	+	NM_178581<br>NM_178582<br>NM_178580<br>NM_030789<br>BC062595<br>AF075033<br>BC053868<br>ENSESTT00000040696<br>ENSESTT00000040697<br>ENSESTT00000040698<br>ENSESTT00000040699<br>ENSESTT00000040700<br>ENST00000340852<br>ENST00000335574<br>ENST00000344042<br>ENST00000262657<br>ENST00000376127<br>ENST00000376118<br>GENSCAN00000057053<br>GENSCAN00000062391	Homo sapiens histocompatibility (minor) 13 (HM13), transcript variant 3, mRNA.<br>Homo sapiens histocompatibility (minor) 13 (HM13), transcript variant 4, mRNA.<br>Homo sapiens histocompatibility (minor) 13 (HM13), transcript variant 2, mRNA.<br>Homo sapiens histocompatibility (minor) 13 (HM13), transcript variant 1, mRNA.<br>Homo sapiens histocompatibility (minor) 13, mRNA (cDNA clone MGC:74542 IMAGE:5245982), complete cds.<br>Homo sapiens full length insert cDNA YN52F10.<br>Homo sapiens cDNA clone IMAGE:5194843, **** WARNING: chimeric clone ****.<br><br><br><br><br><br>cdna:known-ccds chromosome:NCBI36:20:29565892:29621031:1 gene:ENSG00000101294 CCDS13182.1<br>cdna:known-ccds chromosome:NCBI36:20:29565902:29621029:1 gene:ENSG00000101294 CCDS13183.1<br>cdna:known-ccds chromosome:NCBI36:20:29565902:29591257:1 gene:ENSG00000101294 CCDS13184.1<br>cdna:known chromosome:NCBI36:20:29565902:29621029:1 gene:ENSG00000101294<br>cdna:known chromosome:NCBI36:20:29565902:29621029:1 gene:ENSG00000101294<br>cdna:known chromosome:NCBI36:20:29565921:29590079:1 gene:ENSG00000101294<br>cdna:Genscan chromosome:NCBI36:20:29578948:29657277:1<br>cdna:Genscan chromosome:NCBI36:20:29548613:29566198:1

		3881328<br>3881329<br>3881330<br>3881331<br>3881332<br>3881333<br>3881334<br>3881335<br>3881336<br>3881337<br>3881338<br>3881339<br>3881340<br>3881341<br>3881342<br>3881343<br>3881344<br>3881345<br>3881346<br>3881347<br>3881348<br>3881349<br>3881350<br>3881351<br>3881352<br>3881353<br>3881354<br>3881355<br>3881356<br>3881357<br>3881358	extended<br>core<br>extended<br>extended<br>core<br>extended<br>extended<br>core<br>core<br>extended<br>extended<br>extended<br>core<br>extended<br>extended<br>extended<br>full<br>extended<br>extended<br>extended<br>core<br>core<br>core<br>extended<br>extended<br>core<br>core<br>core<br>extended<br>extended<br>extended						
3881443	967993<br>967994<br>967995<br>967996<br>967997<br>967998<br>967999<br>968000<br>968001<br>968002<br>968003<br>968004<br>968005<br>968006<br>968007<br>968008<br>968009<br>968010<br>968011<br>968012<br>968013<br>968014<br>968015	3881444<br>3881445<br>3881446<br>3881447<br>3881448<br>3881449<br>3881450<br>3881451<br>3881452<br>3881453<br>3881454<br>3881455<br>3881456<br>3881457<br>3881458<br>3881459<br>3881460<br>3881461<br>3881462<br>3881463<br>3881464<br>3881465<br>3881466	full<br>full<br>free<br>core<br>core<br>full<br>full<br>core<br>extended<br>extended<br>core<br>core<br>extended<br>core<br>core<br>core<br>extended<br>full<br>core<br>core<br>core<br>extended<br>extended	20	29774755	29853260	+	NM_012112<br>AF287265<br>AK093379<br>ENSESTT00000017907<br>ENSESTT00000017908<br>ENST00000300403<br>ENST00000340513<br>ENST00000376017<br>ENST00000376026<br>GENSCAN00000000998<br>GENSCAN00000000996	Homo sapiens TPX2, microtubule-associated, homolog (Xenopus laevis) (TPX2), mRNA.<br>Homo sapiens hepatocellular carcinoma-associated antigen 90 (HCA90) mRNA, complete cds.<br>Homo sapiens cDNA FLJ36060 fis, clone TESTI2018521, moderately similar to Targeting protein for Xklp2; TPX2.<br><br><br>cdna:known-ccds chromosome:NCBI36:20:29790565:29853264:1 gene:ENSG00000088325 CCDS13190.1<br>cdna:known chromosome:NCBI36:20:29790565:29853264:1 gene:ENSG00000088325<br>cdna:known chromosome:NCBI36:20:29790754:29853264:1 gene:ENSG00000088325<br>cdna:known chromosome:NCBI36:20:29790754:29853264:1 gene:ENSG00000088325<br>cdna:Genscan chromosome:NCBI36:20:29774755:29775513:1<br>cdna:Genscan chromosome:NCBI36:20:29808941:29852544:1

	968016<br>968017<br>968018<br>968019<br>968020<br>968021<br>968022<br>968023<br>968024<br>968025<br>968026<br>968027	3881467<br>3881468<br>3881469<br>3881470<br>3881471<br>3881472<br>3881473<br>3881474<br>3881475<br>3881476<br>3881477<br>3881478<br>3881479<br>3881480<br>3881481<br>3881482<br>3881483<br>3881484<br>3881485<br>3881486<br>3881487<br>3881488<br>3881489<br>3881490<br>3881491<br>3881492<br>3881493	core<br>core<br>full<br>core<br>extended<br>core<br>extended<br>extended<br>extended<br>core<br>core<br>core<br>core<br>full<br>extended<br>core<br>core<br>full<br>extended<br>extended<br>core<br>core<br>core<br>core<br>core<br>core<br>core						
3881786	968215<br>968216<br>968217<br>968218<br>968219<br>968220<br>968221<br>968222<br>968223<br>968224<br>968225<br>968226	3881787<br>3881788<br>3881789<br>3881790<br>3881791<br>3881792<br>3881793<br>3881794<br>3881795<br>3881796<br>3881797<br>3881798<br>3881799<br>3881800<br>3881801<br>3881802<br>3881803<br>3881804<br>3881805<br>3881806<br>3881807<br>3881808<br>3881809	core<br>core<br>core<br>core<br>core<br>core<br>core<br>core<br>core<br>extended<br>extended<br>full<br>full<br>full<br>core<br>core<br>extended<br>core<br>core<br>core<br>core<br>core<br>core	20	30259344	30290099	+	NM_015352<br>NM_172236<br>AK124093<br>ENSESTT00000017919<br>ENSESTT00000017920<br>ENSESTT00000017921<br>ENST00000375749<br>ENST00000375730<br>ENST00000217326<br>ENST00000278989<br>GENSCAN00000064538	Homo sapiens protein O-fucosyltransferase 1 (POFUT1), transcript variant 1, mRNA.<br>Homo sapiens protein O-fucosyltransferase 1 (POFUT1), transcript variant 2, mRNA.<br>Homo sapiens cDNA FLJ42099 fis, clone TESOP2006110.<br><br><br><br>cdna:known-ccds chromosome:NCBI36:20:30259344:30290131:1 gene:ENSG00000101346 CCDS13198.1<br>cdna:known-ccds chromosome:NCBI36:20:30259357:30269387:1 gene:ENSG00000101346 CCDS13199.1<br>cdna:known chromosome:NCBI36:20:30259357:30290125:1 gene:ENSG00000101346<br>cdna:known chromosome:NCBI36:20:30259357:30269386:1 gene:ENSG00000101346<br>cdna:Genscan chromosome:NCBI36:20:30259406:30286125:1
3884100	969628<br>969629<br>969630	3884101<br>3884102<br>3884103	extended<br>core<br>core	20	35240227	35303721	+	NM_002951<br>AK123690<br>ENSESTT00000008346	Homo sapiens ribophorin II (RPN2), mRNA.<br>Homo sapiens cDNA FLJ41696 fis, clone HCHON2001853.

	969631<br>969632<br>969633<br>969634<br>969635<br>969636<br>969637<br>969638<br>969639<br>969640<br>969641<br>969642<br>969643<br>969644<br>969645<br>969646<br>969647<br>969648<br>969649<br>969650<br>969651<br>969652<br>969653<br>969654<br>969655<br>969656<br>969657<br>969658<br>969659<br>969660<br>969661<br>969662<br>969663<br>969664	3884104<br>3884105<br>3884106<br>3884107<br>3884108<br>3884109<br>3884110<br>3884111<br>3884112<br>3884113<br>3884114<br>3884115<br>3884116<br>3884117<br>3884118<br>3884119<br>3884120<br>3884121<br>3884122<br>3884123<br>3884124<br>3884125<br>3884126<br>3884127<br>3884128<br>3884129<br>3884130<br>3884131<br>3884132<br>3884133<br>3884134<br>3884135<br>3884136<br>3884137<br>3884138<br>3884139<br>3884140<br>3884141<br>3884142<br>3884143<br>3884144<br>3884145<br>3884146<br>3884147<br>3884148<br>3884149	full<br>core<br>extended<br>extended<br>core<br>extended<br>core<br>extended<br>extended<br>extended<br>extended<br>extended<br>extended<br>core<br>core<br>core<br>core<br>core<br>extended<br>core<br>core<br>extended<br>extended<br>extended<br>extended<br>core<br>core<br>extended<br>extended<br>extended<br>core<br>extended<br>core<br>core<br>core<br>extended<br>full<br>full<br>extended<br>extended<br>full<br>extended<br>core<br>core<br>extended<br>extended					ENSESTT00000008347<br>ENSESTT00000008348<br>ENSESTT00000008349<br>ENSESTT00000008350<br>ENSESTT00000008351<br>ENSESTT00000008352<br>ENSESTT00000008353<br>ENSESTT00000008354<br>ENST00000237530<br>ENST00000338768<br>ENST00000237531<br>ENST00000373632<br>ENST00000373628<br>ENST00000373623<br>ENST00000373622<br>ENST00000279021<br>GENSCAN00000046809	 <br><br><br><br><br><br><br>cdna:known-ccds chromosome:NCBI36:20:35240888:35303440:1 gene:ENSG00000118705 CCDS13291.1<br>cdna:known chromosome:NCBI36:20:35240888:35303434:1 gene:ENSG00000118705<br>cdna:known chromosome:NCBI36:20:35240888:35303434:1 gene:ENSG00000118705<br>cdna:known chromosome:NCBI36:20:35240888:35303434:1 gene:ENSG00000118705<br>cdna:known chromosome:NCBI36:20:35240888:35303434:1 gene:ENSG00000118705<br>cdna:known chromosome:NCBI36:20:35240888:35303434:1 gene:ENSG00000118705<br>cdna:known chromosome:NCBI36:20:35240978:35303440:1 gene:ENSG00000118705<br>cdna:known chromosome:NCBI36:20:35240978:35303202:1 gene:ENSG00000118705<br>cdna:Genscan chromosome:NCBI36:20:35221844:35299586:1
3887017	971428<br>971429<br>971430<br>971431<br>971432<br>971433<br>971434<br>971435	3887018<br>3887019<br>3887020<br>3887021<br>3887022<br>3887023<br>3887024<br>3887025	full<br>core<br>core<br>core<br>extended<br>core<br>core<br>core	20	43840743	43873796	+	NM_052951<br>ENSESTT00000048446<br>ENSESTT00000048447<br>ENST00000372622<br>ENST00000217439<br>ENST00000372614<br>ENST00000372605<br>GENSCAN00000027336	Homo sapiens deoxynucleotidyltransferase, terminal, interacting protein 1 (DNTTIP1), mRNA.<br><br><br>cdna:known-ccds chromosome:NCBI36:20:43853983:43873473:1 gene:ENSG00000101457 CCDS13369.1<br>cdna:known chromosome:NCBI36:20:43853983:43873471:1 gene:ENSG00000101457<br>cdna:known chromosome:NCBI36:20:43853983:43873471:1 gene:ENSG00000101457<br>cdna:known chromosome:NCBI36:20:43853983:43873471:1 gene:ENSG00000101457<br>cdna:Genscan chromosome:NCBI36:20:43854051:43878813:1

	971436<br>971437<br>971438<br>971439<br>971440<br>971441<br>971442<br>971443<br>971444<br>971445<br>971446<br>971447<br>971448	3887026<br>3887027<br>3887028<br>3887029<br>3887030<br>3887031<br>3887032<br>3887033<br>3887034<br>3887035<br>3887036<br>3887037<br>3887038<br>3887039<br>3887040<br>3887041<br>3887042	core<br>extended<br>extended<br>core<br>core<br>core<br>extended<br>core<br>core<br>core<br>core<br>core<br>core<br>core<br>extended<br>extended<br>extended						
3887049	971452<br>971453<br>971454<br>971455<br>971456<br>971457<br>971458	3887050<br>3887051<br>3887052<br>3887053<br>3887054<br>3887055<br>3887056<br>3887057<br>3887058<br>3887059<br>3887060<br>3887061<br>3887062<br>3887063<br>3887064<br>3887065<br>3887066<br>3887067<br>3887068	core<br>core<br>core<br>core<br>core<br>core<br>core<br>extended<br>extended<br>core<br>extended<br>core<br>core<br>core<br>core<br>full<br>core<br>core<br>core	20	43874662	43878994	+	NM_181803<br>NM_181799<br>NM_181802<br>NM_007019<br>NM_181800<br>NM_181801<br>BC032677<br>ENSESTT00000048448<br>ENSESTT00000048449<br>ENST00000243893<br>ENST00000372579<br>ENST00000335046<br>ENST00000356455<br>ENST00000352551<br>ENST00000343198<br>ENST00000372568<br>GENSCAN00000027336	Homo sapiens ubiquitin-conjugating enzyme E2C (UBE2C), transcript variant 6, mRNA.<br>Homo sapiens ubiquitin-conjugating enzyme E2C (UBE2C), transcript variant 2, mRNA.<br>Homo sapiens ubiquitin-conjugating enzyme E2C (UBE2C), transcript variant 5, mRNA.<br>Homo sapiens ubiquitin-conjugating enzyme E2C (UBE2C), transcript variant 1, mRNA.<br>Homo sapiens ubiquitin-conjugating enzyme E2C (UBE2C), transcript variant 3, mRNA.<br>Homo sapiens ubiquitin-conjugating enzyme E2C (UBE2C), transcript variant 4, mRNA.<br>Homo sapiens ubiquitin-conjugating enzyme E2C, mRNA (cDNA clone IMAGE:5574059), partial cds.<br><br><br>cdna:known-ccds chromosome:NCBI36:20:43874662:43879003:1 gene:ENSG00000175063 CCDS13373.1<br>cdna:known-ccds chromosome:NCBI36:20:43874662:43878999:1 gene:ENSG00000175063 CCDS13374.1<br>cdna:known-ccds chromosome:NCBI36:20:43874662:43878999:1 gene:ENSG00000175063 CCDS13371.1<br>cdna:known-ccds chromosome:NCBI36:20:43874710:43879003:1 gene:ENSG00000175063 CCDS13370.1<br>cdna:known-ccds chromosome:NCBI36:20:43874742:43878999:1 gene:ENSG00000175063 CCDS13372.1<br>cdna:known chromosome:NCBI36:20:43874742:43878999:1 gene:ENSG00000175063<br>cdna:known chromosome:NCBI36:20:43875098:43878884:1 gene:ENSG00000175063<br>cdna:Genscan chromosome:NCBI36:20:43854051:43878813:1
3888133	972126<br>972127<br>972128<br>972129<br>972130<br>972131<br>972132<br>972133<br>972134<br>972135<br>972136<br>972137<br>972138<br>972139<br>972140<br>972141<br>972142	3888134<br>3888135<br>3888136<br>3888137<br>3888138<br>3888139<br>3888140<br>3888141<br>3888142<br>3888143<br>3888144<br>3888145<br>3888146<br>3888147<br>3888148<br>3888149<br>3888150	extended<br>core<br>core<br>extended<br>extended<br>core<br>core<br>core<br>core<br>core<br>core<br>core<br>core<br>extended<br>extended<br>extended<br>core	20	47096213	47146993	+	NM_001316<br>AF053640<br>AF053642<br>ENSESTT00000052892<br>ENSESTT00000052893<br>ENSESTT00000052894<br>ENST00000262982<br>ENST00000338534<br>ENST00000371903<br>GENSCAN00000066066<br>GENSCAN00000032974	Homo sapiens CSE1 chromosome segregation 1-like (yeast) (CSE1L), mRNA.<br>Homo sapiens trachea cellular apoptosis susceptibility protein (CSE1) mRNA, complete cds.<br>Homo sapiens liver cellular apoptosis susceptibilty protein (CSE1) mRNA, partial cds.<br><br><br><br>cdna:known-ccds chromosome:NCBI36:20:47096245:47146891:1 gene:ENSG00000124207 CCDS13412.1<br>cdna:known-ccds chromosome:NCBI36:20:47096245:47146891:1 gene:ENSG00000124207 CCDS13413.1<br>cdna:known chromosome:NCBI36:20:47096256:47146896:1 gene:ENSG00000124207<br>cdna:Genscan chromosome:NCBI36:20:47055014:47125464:1<br>cdna:Genscan chromosome:NCBI36:20:47133970:47146382:1

	972143<br>972144<br>972145<br>972146<br>972147<br>972148<br>972149<br>972150<br>972151<br>972152<br>972153<br>972154<br>972155<br>972156<br>972157<br>972158<br>972159<br>972160<br>972161	3888151<br>3888152<br>3888153<br>3888154<br>3888155<br>3888156<br>3888157<br>3888158<br>3888159<br>3888160<br>3888161<br>3888162<br>3888163<br>3888164<br>3888165<br>3888166<br>3888167<br>3888168<br>3888169<br>3888170<br>3888171<br>3888172<br>3888173<br>3888174<br>3888175<br>3888176<br>3888177<br>3888178<br>3888179<br>3888180<br>3888181<br>3888182<br>3888183<br>3888184	core<br>extended<br>core<br>extended<br>core<br>core<br>core<br>core<br>core<br>core<br>core<br>core<br>extended<br>core<br>extended<br>core<br>core<br>core<br>extended<br>extended<br>core<br>extended<br>core<br>core<br>extended<br>core<br>core<br>core<br>core<br>core<br>core<br>core<br>core<br>extended						
3888217	972179<br>972180<br>972181<br>972182<br>972183<br>972184<br>972185<br>972186<br>972187<br>972188<br>972189<br>972190<br>972191<br>972192<br>972193<br>972194<br>972195<br>972196<br>972197<br>972198	3888218<br>3888219<br>3888220<br>3888221<br>3888222<br>3888223<br>3888224<br>3888225<br>3888226<br>3888227<br>3888228<br>3888229<br>3888230<br>3888231<br>3888232<br>3888233<br>3888234<br>3888235<br>3888236<br>3888237	full<br>extended<br>extended<br>core<br>core<br>core<br>core<br>core<br>extended<br>core<br>core<br>core<br>core<br>core<br>core<br>extended<br>core<br>extended<br>core<br>extended	20	47237780	47294013	+	NM_017895<br>AK127128<br>BC104480<br>AL512707<br>AF161377<br>ENSESTT00000052895<br>ENSESTT00000052896<br>ENST00000371764<br>ENST00000338362<br>GENSCAN00000028408<br>GENSCAN00000032980<br>GENSCAN00000015018	Homo sapiens DEAD (Asp-Glu-Ala-Asp) box polypeptide 27 (DDX27), mRNA.<br>Homo sapiens cDNA FLJ45185 fis, clone BRAWH3048317, highly similar to ATP dependent RNA helicase.<br>Homo sapiens DEAD (Asp-Glu-Ala-Asp) box polypeptide 27, mRNA (cDNA clone IMAGE:6387875), partial cds.<br>Homo sapiens mRNA; cDNA DKFZp667N057 (from clone DKFZp667N057).<br>Homo sapiens HSPC259 mRNA, partial cds.<br><br><br>cdna:known-ccds chromosome:NCBI36:20:47269291:47294021:1 gene:ENSG00000124228 CCDS13416.1<br>cdna:known chromosome:NCBI36:20:47269291:47294019:1 gene:ENSG00000124228<br>cdna:Genscan chromosome:NCBI36:20:47272901:47292629:1<br>cdna:Genscan chromosome:NCBI36:20:47237156:47240105:1<br>cdna:Genscan chromosome:NCBI36:20:47269300:47271542:1

	972199<br>972200<br>972201<br>972202<br>972203<br>972204	3888238<br>3888239<br>3888240<br>3888241<br>3888242<br>3888243<br>3888244<br>3888245<br>3888246<br>3888247<br>3888248<br>3888249<br>3888250<br>3888251<br>3888252<br>3888253	core<br>extended<br>extended<br>core<br>extended<br>core<br>core<br>core<br>core<br>core<br>core<br>core<br>extended<br>core<br>extended<br>core						
3891278	974017<br>974018<br>974019<br>974020<br>974021<br>974022<br>974023<br>974024<br>974025<br>974026<br>974027<br>974028<br>974029<br>974030<br>974031<br>974032	3891279<br>3891280<br>3891281<br>3891282<br>3891283<br>3891284<br>3891285<br>3891286<br>3891287<br>3891288<br>3891289<br>3891290<br>3891291<br>3891292<br>3891293<br>3891294<br>3891295<br>3891296<br>3891297<br>3891298<br>3891299<br>3891300<br>3891301<br>3891302<br>3891303<br>3891304<br>3891305<br>3891306<br>3891307<br>3891308<br>3891309<br>3891310<br>3891311<br>3891312<br>3891313<br>3891314<br>3891315<br>3891316	extended<br>extended<br>core<br>core<br>core<br>extended<br>core<br>core<br>extended<br>core<br>core<br>core<br>extended<br>extended<br>core<br>extended<br>core<br>extended<br>extended<br>core<br>core<br>extended<br>core<br>extended<br>core<br>extended<br>core<br>extended<br>core<br>core<br>core<br>core<br>extended<br>extended<br>extended<br>core<br>core<br>extended	20	56989216	57005174	+	NM_198976<br>AK023310<br>AK127431<br>AJ238374<br>AJ238376<br>ENSESTT00000024233<br>ENSESTT00000024234<br>ENSESTT00000024235<br>ENSESTT00000024236<br>ENSESTT00000024237<br>ENSESTT00000024238<br>ENSESTT00000024239<br>ENST00000344018<br>ENST00000371062<br>ENST00000217129<br>ENST00000371056<br>GENSCAN00000057993	Homo sapiens TH1-like (Drosophila) (TH1L), transcript variant 1, mRNA.<br>Homo sapiens cDNA FLJ13248 fis, clone OVARC1000703.<br>Homo sapiens cDNA FLJ45523 fis, clone BRTHA2026071.<br>Homo sapiens mRNA for putative protein TH1, partial, clone IMAGE ID 785447.<br>Homo sapiens mRNA for putative protein TH1, partial, clone IMAGE ID 255931.<br><br><br><br><br><br><br><br>cdna:known-ccds chromosome:NCBI36:20:56989706:57003581:1 gene:ENSG00000101158 CCDS13473.1<br>cdna:known chromosome:NCBI36:20:56989725:57003583:1 gene:ENSG00000101158<br>cdna:known chromosome:NCBI36:20:56989725:57003583:1 gene:ENSG00000101158<br>cdna:known chromosome:NCBI36:20:56989725:56998850:1 gene:ENSG00000101158<br>cdna:Genscan chromosome:NCBI36:20:56989706:57003126:1

		3891317<br>3891318<br>3891319<br>3891320<br>3891321<br>3891322<br>3891323<br>3891324	core<br>core<br>core<br>core<br>core<br>extended<br>extended<br>extended						
3891447	974097<br>974098<br>974099<br>974100<br>974101<br>974102<br>974103<br>974104<br>974105<br>974106<br>974107<br>974108<br>974109<br>974110<br>974111<br>974112<br>974113<br>974114<br>974115<br>974116<br>974117<br>974118<br>974119<br>974120<br>974121<br>974122<br>974123<br>974124<br>974125<br>974126	3891448<br>3891449<br>3891450<br>3891451<br>3891452<br>3891453<br>3891454<br>3891455<br>3891456<br>3891457<br>3891458<br>3891459<br>3891460<br>3891461<br>3891462<br>3891463<br>3891464<br>3891465<br>3891466<br>3891467<br>3891468<br>3891469<br>3891470<br>3891471<br>3891472<br>3891473<br>3891474<br>3891475<br>3891476<br>3891477<br>3891478<br>3891479<br>3891480<br>3891481<br>3891482<br>3891483<br>3891484<br>3891485<br>3891486<br>3891487<br>3891488<br>3891489	full<br>full<br>full<br>full<br>full<br>full<br>core<br>core<br>extended<br>core<br>core<br>core<br>core<br>full<br>extended<br>extended<br>extended<br>extended<br>full<br>full<br>core<br>core<br>core<br>core<br>extended<br>extended<br>core<br>core<br>core<br>core<br>core<br>full<br>full<br>full<br>full<br>full<br>extended<br>full<br>full<br>full<br>full<br>full	20	57298558	57374412	+	NM_000114<br>NM_207034<br>NM_207033<br>NM_207032<br>ENSESTT00000024241<br>ENSESTT00000024242<br>ENST00000337938<br>ENST00000371025<br>ENST00000311585<br>ENST00000371028<br>ENST00000342429<br>GENSCAN00000001669	Homo sapiens endothelin 3 (EDN3), transcript variant 1, mRNA.<br>Homo sapiens endothelin 3 (EDN3), transcript variant 4, mRNA.<br>Homo sapiens endothelin 3 (EDN3), transcript variant 3, mRNA.<br>Homo sapiens endothelin 3 (EDN3), transcript variant 2, mRNA.<br><br><br>cdna:known-ccds chromosome:NCBI36:20:57308877:57334441:1 gene:ENSG00000124205 CCDS13477.1<br>cdna:known-ccds chromosome:NCBI36:20:57308892:57334442:1 gene:ENSG00000124205 CCDS13478.1<br>cdna:known-ccds chromosome:NCBI36:20:57308894:57334441:1 gene:ENSG00000124205 CCDS13479.1<br>cdna:known chromosome:NCBI36:20:57308892:57334442:1 gene:ENSG00000124205<br>cdna:known chromosome:NCBI36:20:57308894:57334441:1 gene:ENSG00000124205<br>cdna:Genscan chromosome:NCBI36:20:57277408:57336086:1
3893287	975267<br>975268<br>975269	3893288<br>3893289<br>3893290	core<br>extended<br>full	20	61374610	61392545	+	NM_018209<br>NM_175609<br>AK027268	Homo sapiens ADP-ribosylation factor GTPase activating protein 1 (ARFGAP1), transcript variant 1, mRNA.<br>Homo sapiens ADP-ribosylation factor GTPase activating protein 1 (ARFGAP1), transcript variant 2, mRNA.<br>Homo sapiens cDNA FLJ14362 fis, clone HEMBA1000657, moderately similar to Rattus norvegicus ADP-ribosylation

	975270<br>975271<br>975272<br>975273<br>975274<br>975275<br>975276<br>975277<br>975278<br>975279<br>975280<br>975281<br>975282<br>975283<br>975284<br>975285<br>975286<br>975287<br>975288<br>975289	3893291<br>3893292<br>3893293<br>3893294<br>3893295<br>3893296<br>3893297<br>3893298<br>3893299<br>3893300<br>3893301<br>3893302<br>3893303<br>3893304<br>3893305<br>3893306<br>3893307<br>3893308<br>3893309<br>3893310<br>3893311<br>3893312<br>3893313<br>3893314<br>3893315<br>3893316<br>3893317<br>3893318<br>3893319<br>3893320<br>3893321<br>3893322<br>3893323<br>3893324<br>3893325<br>3893326<br>3893327<br>3893328<br>3893329<br>3893330<br>3893331<br>3893332<br>3893333<br>3893334<br>3893335<br>3893336<br>3893337	core<br>core<br>core<br>core<br>extended<br>extended<br>core<br>extended<br>core<br>extended<br>core<br>full<br>core<br>extended<br>extended<br>extended<br>extended<br>core<br>extended<br>extended<br>extended<br>extended<br>extended<br>extended<br>core<br>extended<br>extended<br>extended<br>core<br>extended<br>extended<br>core<br>extended<br>core<br>full<br>core<br>core<br>core<br>core<br>core<br>core<br>core<br>core<br>core<br>core<br>full<br>full					AK095048<br>AK123367<br>AK123873<br>ENSESTT00000045423<br>ENSESTT00000045424<br>ENSESTT00000045425<br>ENST00000370283<br>ENST00000353546<br>ENST00000351930<br>ENST00000370275<br>GENSCAN00000029186	factor-directed GTPase activating protein mRNA.<br>Homo sapiens cDNA FLJ37729 fis, clone BRHIP2020509.<br>Homo sapiens cDNA FLJ41373 fis, clone BRCAN2007409.<br>Homo sapiens cDNA FLJ41879 fis, clone OCBBF2020741.<br><br><br><br>cdna:known-ccds chromosome:NCBI36:20:61374610:61391590:1 gene:ENSG00000101199 CCDS13515.1<br>cdna:known-ccds chromosome:NCBI36:20:61374610:61391587:1 gene:ENSG00000101199 CCDS13516.1<br>cdna:known chromosome:NCBI36:20:61374610:61391587:1 gene:ENSG00000101199<br>cdna:known chromosome:NCBI36:20:61374630:61391585:1 gene:ENSG00000101199<br>cdna:Genscan chromosome:NCBI36:20:61377093:61392545:1
3893520	975403<br>975404<br>975405<br>975406<br>975407<br>975408<br>975409	3893521<br>3893522<br>3893523<br>3893524<br>3893525<br>3893526<br>3893527	full<br>extended<br>extended<br>extended<br>extended<br>core<br>core	20	61755129	61800485	+	NM_032945<br>NM_003823<br>NM_032957<br>NM_016434<br>AB029011<br>AK131105<br>BC034349	Homo sapiens tumor necrosis factor receptor superfamily, member 6b, decoy (TNFRSF6B), transcript variant M68C, mRNA.<br>Homo sapiens tumor necrosis factor receptor superfamily, member 6b, decoy (TNFRSF6B), transcript variant M68E, mRNA.<br>Homo sapiens regulator of telomere elongation helicase 1 (RTEL1), transcript variant 2, mRNA.<br>Homo sapiens regulator of telomere elongation helicase 1 (RTEL1), transcript variant 1, mRNA.<br>Homo sapiens mRNA for KIAA1088 protein, partial cds.<br>Homo sapiens mRNA for FLJ00362 protein.<br>Homo sapiens tumor necrosis factor receptor superfamily, member 6b, decoy, mRNA (cDNA clone MGC:21079

	975410<br>975411<br>975412<br>975413<br>975414<br>975415<br>975416<br>975417<br>975418<br>975419<br>975420<br>975421<br>975422<br>975423<br>975424<br>975425<br>975426<br>975427<br>975428<br>975429<br>975430<br>975431<br>975432<br>975433<br>975434<br>975435<br>975436<br>975437<br>975438<br>975439<br>975440<br>975441<br>975442<br>975443<br>975444<br>975445<br>975446<br>975447<br>975448<br>975449<br>975450<br>975451<br>975452	3893528<br>3893529<br>3893530<br>3893531<br>3893532<br>3893533<br>3893534<br>3893535<br>3893536<br>3893537<br>3893538<br>3893539<br>3893540<br>3893541<br>3893542<br>3893543<br>3893544<br>3893545<br>3893546<br>3893547<br>3893548<br>3893549<br>3893550<br>3893551<br>3893552<br>3893553<br>3893554<br>3893555<br>3893556<br>3893557<br>3893558<br>3893559<br>3893560<br>3893561<br>3893562<br>3893563<br>3893564<br>3893565<br>3893566<br>3893567<br>3893568<br>3893569<br>3893570<br>3893571<br>3893572<br>3893573<br>3893574<br>3893575<br>3893576<br>3893577<br>3893578<br>3893579<br>3893580<br>3893581	extended<br>full<br>core<br>extended<br>core<br>extended<br>extended<br>core<br>core<br>extended<br>full<br>full<br>core<br>core<br>extended<br>core<br>extended<br>core<br>core<br>full<br>core<br>full<br>full<br>full<br>core<br>full<br>core<br>extended<br>core<br>core<br>core<br>extended<br>extended<br>core<br>core<br>extended<br>extended<br>core<br>core<br>core<br>core<br>core<br>extended<br>core<br>core<br>core<br>extended<br>core<br>core<br>extended<br>core<br>core<br>core<br>full					AL080127<br>ENSESTT00000057010<br>ENSESTT00000057011<br>ENSESTT00000057012<br>ENSESTT00000057013<br>ENSESTT00000057014<br>ENSESTT00000057015<br>ENSESTT00000057016<br>ENSESTT00000057017<br>ENST00000370018<br>ENST00000318100<br>ENST00000370006<br>ENST00000355223<br>ENST00000342852<br>ENST00000356810<br>ENST00000360203<br>ENST00000369999<br>ENST00000369996<br>ENST00000369993<br>ENST00000370003<br>GENSCAN00000047839<br>GENSCAN00000045781	IMAGE:4752507), complete cds.<br>Homo sapiens mRNA; cDNA DKFZp434C013 (from clone DKFZp434C013).<br>cdna:known-ccds chromosome:NCBI36:20:61759607:61798050:1 gene:ENSG00000026036 CCDS13531.1<br>cdna:known-ccds chromosome:NCBI36:20:61760091:61800495:1 gene:ENSG00000026036 CCDS13530.1<br>cdna:known-ccds chromosome:NCBI36:20:61760091:61800495:1 gene:ENSG00000026036 CCDS13532.1<br>cdna:known chromosome:NCBI36:20:61760091:61800495:1 gene:ENSG00000026036<br>cdna:known chromosome:NCBI36:20:61760091:61800495:1 gene:ENSG00000026036<br>cdna:known chromosome:NCBI36:20:61761200:61800481:1 gene:ENSG00000026036<br>cdna:known chromosome:NCBI36:20:61788168:61800481:1 gene:ENSG00000026036<br>cdna:known chromosome:NCBI36:20:61796732:61800481:1 gene:ENSG00000026036<br>cdna:known chromosome:NCBI36:20:61798465:61800481:1 gene:ENSG00000026036<br>cdna:known chromosome:NCBI36:20:61798565:61800358:1 gene:ENSG00000026036<br>cdna:novel chromosome:NCBI36:20:61791763:61798045:1 gene:ENSG00000026036<br>cdna:Genscan chromosome:NCBI36:20:61798565:61800360:1<br>cdna:Genscan chromosome:NCBI36:20:61755129:61797490:1

		3893582<br>3893583<br>3893584<br>3893585<br>3893586<br>3893587<br>3893588<br>3893589<br>3893590<br>3893591<br>3893592<br>3893593<br>3893594<br>3893595<br>3893596<br>3893597<br>3893598<br>3893599<br>3893600<br>3893601<br>3893602<br>3893603<br>3893604<br>3893605<br>3893606<br>3893607	core<br>extended<br>core<br>core<br>core<br>extended<br>core<br>core<br>core<br>core<br>core<br>core<br>core<br>extended<br>extended<br>core<br>core<br>extended<br>core<br>core<br>core<br>extended<br>extended<br>extended<br>core<br>core						
3895118	976328<br>976329<br>976330<br>976331<br>976332<br>976333<br>976334<br>976335<br>976336<br>976337<br>976338<br>976339<br>976340	3895119<br>3895120<br>3895121<br>3895122<br>3895123<br>3895124<br>3895125<br>3895126<br>3895127<br>3895128<br>3895129<br>3895130<br>3895131<br>3895132<br>3895133<br>3895134<br>3895135<br>3895136<br>3895137<br>3895138<br>3895139<br>3895140<br>3895141<br>3895142<br>3895143<br>3895144<br>3895145	core<br>core<br>core<br>core<br>core<br>core<br>core<br>core<br>core<br>core<br>core<br>core<br>core<br>core<br>core<br>core<br>core<br>core<br>core<br>core<br>core<br>core<br>extended<br>extended<br>core<br>core<br>extended	20	2722721	2729280	-	NM_019609<br>ENST00000380605<br>ENST00000326350<br>GENSCAN00000036136	Homo sapiens carboxypeptidase X (M14 family) (CPXM), mRNA.<br>cdna:known-ccds chromosome:NCBI36:20:2722715:2729282:-1 gene:ENSG00000088882 CCDS13033.1<br>cdna:known chromosome:NCBI36:20:2722715:2729280:-1 gene:ENSG00000088882<br>cdna:Genscan chromosome:NCBI36:20:2722836:2729218:-1

3900833	979807<br>979808<br>979809<br>979810<br>979811<br>979812<br>979813<br>979814<br>979815<br>979816	3900834<br>3900835<br>3900836<br>3900837<br>3900838<br>3900839<br>3900840<br>3900841<br>3900842<br>3900843<br>3900844<br>3900845<br>3900846<br>3900847<br>3900848	full<br>full<br>full<br>full<br>full<br>full<br>extended<br>core<br>core<br>core<br>core<br>core<br>core<br>core<br>core	20	22436145	22514103	-	NM_021784<br>NR_001558<br>NM_153675<br>ENSESTT00000000806<br>ENST00000377115<br>ENST00000319993<br>ENST00000245994<br>GENSCAN00000067129	Homo sapiens forkhead box A2 (FOXA2), transcript variant 1, mRNA.<br>Homo sapiens chromosome 20 open reading frame 56 (C20orf56) on chromosome 20.<br>Homo sapiens forkhead box A2 (FOXA2), transcript variant 2, mRNA.<br><br>cdna:known-ccds chromosome:NCBI36:20:22509643:22514093:-1 gene:ENSG00000125798 CCDS13147.1<br>cdna:known chromosome:NCBI36:20:22509643:22513101:-1 gene:ENSG00000125798<br>cdna:known chromosome:NCBI36:20:22509823:22514102:-1 gene:ENSG00000125798<br>cdna:Genscan chromosome:NCBI36:20:22510488:22515530:-1
3903361	981279<br>981280<br>981281<br>981282<br>981283<br>981284<br>981285<br>981286<br>981287<br>981288<br>981289<br>981290<br>981291<br>981292<br>981293<br>981294<br>981295<br>981296<br>981297<br>981298<br>981299<br>981300<br>981301<br>981302<br>981303<br>981304<br>981305<br>981306<br>981307<br>981308<br>981309	3903362<br>3903363<br>3903364<br>3903365<br>3903366<br>3903367<br>3903368<br>3903369<br>3903370<br>3903371<br>3903372<br>3903373<br>3903374<br>3903375<br>3903376<br>3903377<br>3903378<br>3903379<br>3903380<br>3903381<br>3903382<br>3903383<br>3903384<br>3903385<br>3903386<br>3903387<br>3903388<br>3903389<br>3903390<br>3903391<br>3903392<br>3903393<br>3903394<br>3903395<br>3903396<br>3903397<br>3903398<br>3903399<br>3903400	extended<br>extended<br>full<br>extended<br>extended<br>extended<br>extended<br>extended<br>extended<br>extended<br>extended<br>extended<br>extended<br>extended<br>extended<br>core<br>core<br>core<br>core<br>core<br>core<br>core<br>core<br>core<br>core<br>core<br>core<br>extended<br>core<br>core<br>core<br>core<br>full<br>extended<br>extended<br>extended<br>core<br>extended<br>extended	20	32286310	32363259	-	NM_000687<br>AK097610<br>ENSESTT00000023520<br>ENSESTT00000023521<br>ENST00000217426<br>ENST00000374942<br>ENST00000331585<br>GENSCAN00000032092	Homo sapiens S-adenosylhomocysteine hydrolase (AHCY), mRNA.<br>Homo sapiens cDNA FLJ40291 fis, clone TESTI2028384, highly similar to ADENOSYLHOMOCYSTEINASE (EC 3.3.1.1).<br><br><br>cdna:known-ccds chromosome:NCBI36:20:32331737:32354811:-1 gene:ENSG00000101444 CCDS13233.1<br>cdna:known chromosome:NCBI36:20:32331737:32354784:-1 gene:ENSG00000101444<br>cdna:known chromosome:NCBI36:20:32331737:32354784:-1 gene:ENSG00000101444<br>cdna:Genscan chromosome:NCBI36:20:32332501:32354497:-1

		3903401	extended						
3907111	983589<br>983590<br>983591<br>983592<br>983593<br>983594<br>983595<br>983596<br>983597<br>983598<br>983599	3907112<br>3907113<br>3907114<br>3907115<br>3907116<br>3907117<br>3907118<br>3907119<br>3907120<br>3907121<br>3907122<br>3907123<br>3907124<br>3907125<br>3907126	core<br>core<br>core<br>core<br>core<br>extended<br>core<br>core<br>core<br>core<br>core<br>extended<br>extended<br>core<br>core	20	43004185	43022528	-	NM_006809<br>ENSESTT00000012876<br>ENSESTT00000012877<br>ENST00000372813<br>ENST00000216891<br>ENST00000372810<br>GENSCAN00000052376	Homo sapiens translocase of outer mitochondrial membrane 34 (TOMM34), nuclear gene encoding mitochondrial protein, mRNA.<br><br><br>cdna:known-ccds chromosome:NCBI36:20:43004185:43022541:-1 gene:ENSG00000025772 CCDS13340.1<br>cdna:known chromosome:NCBI36:20:43004186:43022528:-1 gene:ENSG00000025772<br>cdna:known chromosome:NCBI36:20:43004186:43022528:-1 gene:ENSG00000025772<br>cdna:Genscan chromosome:NCBI36:20:43005164:43022604:-1
3910785	985845<br>985846<br>985847<br>985848<br>985849<br>985850<br>985851<br>985852<br>985853<br>985854<br>985855<br>985856<br>985857<br>985858<br>985859<br>985860<br>985861<br>985862<br>985863<br>985864	3910786<br>3910787<br>3910788<br>3910789<br>3910790<br>3910791<br>3910792<br>3910793<br>3910794<br>3910795<br>3910796<br>3910797<br>3910798<br>3910799<br>3910800<br>3910801<br>3910802<br>3910803<br>3910804<br>3910805<br>3910806<br>3910807<br>3910808<br>3910809<br>3910810	core<br>core<br>core<br>core<br>core<br>core<br>full<br>full<br>extended<br>full<br>core<br>core<br>extended<br>extended<br>core<br>full<br>core<br>core<br>core<br>core<br>core<br>core<br>core<br>full<br>full	20	54377855	54402553	-	NM_198436<br>NM_198434<br>NM_198437<br>NM_198435<br>NM_198433<br>NM_003600<br>NR_001587<br>ENSESTT00000006498<br>ENSESTT00000006499<br>ENST00000347343<br>ENST00000371356<br>ENST00000371353<br>ENST00000312783<br>GENSCAN00000011605	Homo sapiens aurora kinase A (AURKA), transcript variant 5, mRNA.<br>Homo sapiens aurora kinase A (AURKA), transcript variant 3, mRNA.<br>Homo sapiens aurora kinase A (AURKA), transcript variant 6, mRNA.<br>Homo sapiens aurora kinase A (AURKA), transcript variant 4, mRNA.<br>Homo sapiens aurora kinase A (AURKA), transcript variant 1, mRNA.<br>Homo sapiens aurora kinase A (AURKA), transcript variant 2, mRNA.<br>Homo sapiens serine/threonine kinase 6 pseudogene (STK6P) on chromosome 1.<br><br><br>cdna:known-ccds chromosome:NCBI36:20:54377852:54400758:-1 gene:ENSG00000087586 CCDS13451.1<br>cdna:known chromosome:NCBI36:20:54377852:54400659:-1 gene:ENSG00000087586<br>cdna:known chromosome:NCBI36:20:54377852:54400656:-1 gene:ENSG00000087586<br>cdna:known chromosome:NCBI36:20:54377853:54400682:-1 gene:ENSG00000087586<br>cdna:Genscan chromosome:NCBI36:20:54378256:54402553:-1
3911217	986113<br>986114<br>986115<br>986116<br>986117<br>986118<br>986119<br>986120<br>986121<br>986122<br>986123<br>986124	3911218<br>3911219<br>3911220<br>3911221<br>3911222<br>3911223<br>3911224<br>3911225<br>3911226<br>3911227<br>3911228<br>3911229	core<br>core<br>core<br>core<br>core<br>core<br>core<br>core<br>core<br>core<br>core<br>core	20	55656877	55781123	-	NM_199169<br>NM_020182<br>NM_199170<br>NM_199171<br>AY128643<br>BC080635<br>ENSESTT00000014631<br>ENST00000265626<br>ENST00000347215<br>ENST00000341744<br>ENST00000371171<br>ENST00000371170	Homo sapiens transmembrane, prostate androgen induced RNA (TMEPAI), transcript variant 2, mRNA.<br>Homo sapiens transmembrane, prostate androgen induced RNA (TMEPAI), transcript variant 1, mRNA.<br>Homo sapiens transmembrane, prostate androgen induced RNA (TMEPAI), transcript variant 3, mRNA.<br>Homo sapiens transmembrane, prostate androgen induced RNA (TMEPAI), transcript variant 4, mRNA.<br>Homo sapiens PMEPA1 variant A protein mRNA, complete cds.<br>Homo sapiens transmembrane, prostate androgen induced RNA, mRNA (cDNA clone MGC:99523 IMAGE:6569922), complete cds.<br><br>cdna:known-ccds chromosome:NCBI36:20:55656860:55719947:-1 gene:ENSG00000124225 CCDS13464.1<br>cdna:known-ccds chromosome:NCBI36:20:55656860:55719031:-1 gene:ENSG00000124225 CCDS13462.1<br>cdna:known-ccds chromosome:NCBI36:20:55656860:55718437:-1 gene:ENSG00000124225 CCDS13463.1<br>cdna:known chromosome:NCBI36:20:55656860:55718364:-1 gene:ENSG00000124225

	986125<br>986126<br>986127<br>986128<br>986129<br>986130<br>986131<br>986132<br>986133<br>986134<br>986135<br>986136<br>986137<br>986138<br>986139<br>986140<br>986141<br>986142<br>986143<br>986144<br>986145<br>986146<br>986147<br>986148<br>986149<br>986150<br>986151<br>986152<br>986153	3911230<br>3911231<br>3911232<br>3911233<br>3911234<br>3911235<br>3911236<br>3911237<br>3911238<br>3911239<br>3911240<br>3911241<br>3911242<br>3911243<br>3911244<br>3911245<br>3911246<br>3911247<br>3911248<br>3911249<br>3911250<br>3911251<br>3911252<br>3911253<br>3911254<br>3911255<br>3911256<br>3911257<br>3911258<br>3911259<br>3911260<br>3911261<br>3911262<br>3911263<br>3911264<br>3911265<br>3911266<br>3911267<br>3911268<br>3911269<br>3911270<br>3911271<br>3911272<br>3911273	core<br>core<br>full<br>core<br>full<br>full<br>full<br>core<br>core<br>extended<br>full<br>full<br>extended<br>full<br>full<br>full<br>full<br>full<br>full<br>full<br>full<br>full<br>extended<br>full<br>full<br>extended<br>extended<br>extended<br>extended<br>core<br>extended<br>core<br>full<br>core<br>extended<br>full<br>full<br>full<br>full<br>full<br>full<br>full<br>full<br>full					GENSCAN00000014968<br>GENSCAN00000014973<br>GENSCAN00000055391<br>GENSCAN00000055394<br>GENSCAN00000055393<br>GENSCAN00000054003	cdna:known chromosome:NCBI36:20:55660241:55718985:-1 gene:ENSG00000124225<br>cdna:Genscan chromosome:NCBI36:20:55598639:55659468:-1<br>cdna:Genscan chromosome:NCBI36:20:55660515:55662299:-1<br>cdna:Genscan chromosome:NCBI36:20:55668005:55677057:-1<br>cdna:Genscan chromosome:NCBI36:20:55695503:55698405:-1<br>cdna:Genscan chromosome:NCBI36:20:55699829:55701167:-1<br>cdna:Genscan chromosome:NCBI36:20:55664637:55665060:-1
3913960	987773<br>987774<br>987775<br>987776<br>987777<br>987778<br>987779<br>987780<br>987781<br>987782	3913961<br>3913962<br>3913963<br>3913964<br>3913965<br>3913966<br>3913967<br>3913968<br>3913969<br>3913970	full<br>core<br>core<br>core<br>core<br>full<br>core<br>core<br>core<br>core	20	61659170	61688269	-	NM_001037335<br>NM_033405<br>AF517673<br>BC035130<br>ENST00000370091<br>ENST00000252889<br>ENST00000358289<br>ENST00000338175<br>ENST00000370082<br>GENSCAN00000021520	Homo sapiens peroxisomal proliferator-activated receptor A interacting complex 285 (PRIC285), transcript variant 1, mRNA.<br>Homo sapiens peroxisomal proliferator-activated receptor A interacting complex 285 (PRIC285), transcript variant 2, mRNA.<br>Homo sapiens peroxisomal proliferator-activated receptor A interacting complex-285 peptide (PRIC285) mRNA, complete cds.<br>Homo sapiens peroxisomal proliferator-activated receptor A interacting complex 285, mRNA (cDNA clone IMAGE:5263749), complete cds.<br>cdna:known-ccds chromosome:NCBI36:20:61659883:61669871:-1 gene:ENSG00000130589 CCDS13527.1<br>cdna:known chromosome:NCBI36:20:61659884:61676033:-1 gene:ENSG00000130589<br>cdna:known chromosome:NCBI36:20:61659884:61676033:-1 gene:ENSG00000130589<br>cdna:known chromosome:NCBI36:20:61659908:61669856:-1 gene:ENSG00000130589<br>cdna:known chromosome:NCBI36:20:61671936:61676036:-1 gene:ENSG00000130589

	987783<br>987784<br>987785<br>987786<br>987787<br>987788<br>987789<br>987790<br>987791<br>987792<br>987793<br>987794<br>987795<br>987796<br>987797<br>987798<br>987799<br>987800<br>987801<br>987802<br>987803	3913971<br>3913972<br>3913973<br>3913974<br>3913975<br>3913976<br>3913977<br>3913978<br>3913979<br>3913980<br>3913981<br>3913982<br>3913983<br>3913984<br>3913985<br>3913986<br>3913987<br>3913988<br>3913989<br>3913990<br>3913991<br>3913992<br>3913993<br>3913994<br>3913995<br>3913996<br>3913997<br>3913998<br>3913999<br>3914000<br>3914001<br>3914002<br>3914003<br>3914004<br>3914005<br>3914006<br>3914007<br>3914008<br>3914009<br>3914010<br>3914011<br>3914012<br>3914013<br>3914014<br>3914015<br>3914016<br>3914017	core<br>core<br>core<br>core<br>core<br>core<br>core<br>full<br>core<br>core<br>core<br>core<br>core<br>core<br>core<br>core<br>core<br>core<br>core<br>core<br>core<br>core<br>core<br>core<br>full<br>extended<br>extended<br>extended<br>extended<br>extended<br>extended<br>extended<br>extended<br>extended<br>extended<br>extended<br>extended<br>extended<br>extended<br>extended<br>extended<br>extended<br>full<br>full<br>full<br>full<br>full						cdna:Genscan chromosome:NCBI36:20:61659170:61680020:-1
3923218	993539<br>993540<br>993541<br>993542<br>993543<br>993544<br>993545	3923219<br>3923220<br>3923221<br>3923222<br>3923223<br>3923224<br>3923225	extended<br>core<br>extended<br>extended<br>extended<br>extended<br>core	21	43903644	43940364	+	NM_015056<br>AK124620<br>ENSESTT00000018442<br>ENSESTT00000018443<br>ENST00000340648<br>GENSCAN00000014033	Homo sapiens KIAA0179 (KIAA0179), mRNA.<br>Homo sapiens cDNA FLJ42629 fis, clone BRACE3019055.<br><br><br>cdna:known chromosome:NCBI36:21:43903860:43940388:1 gene:ENSG00000160208<br>cdna:Genscan chromosome:NCBI36:21:43903974:43937692:1

	993546<br>993547<br>993548<br>993549<br>993550<br>993551<br>993552<br>993553<br>993554<br>993555<br>993556<br>993557<br>993558<br>993559<br>993560<br>993561<br>993562<br>993563<br>993564	3923226<br>3923227<br>3923228<br>3923229<br>3923230<br>3923231<br>3923232<br>3923233<br>3923234<br>3923235<br>3923236<br>3923237<br>3923238<br>3923239<br>3923240<br>3923241<br>3923242<br>3923243<br>3923244<br>3923245<br>3923246<br>3923247<br>3923248<br>3923249<br>3923250<br>3923251<br>3923252<br>3923253<br>3923254	core<br>core<br>core<br>core<br>core<br>core<br>core<br>core<br>extended<br>extended<br>core<br>core<br>extended<br>core<br>full<br>core<br>core<br>core<br>core<br>extended<br>full<br>full<br>core<br>core<br>extended<br>core<br>core<br>core<br>core						
3923257	993566<br>993567<br>993568<br>993569<br>993570<br>993571<br>993572<br>993573<br>993574<br>993575<br>993576<br>993577<br>993578<br>993579<br>993580<br>993581<br>993582<br>993583<br>993584<br>993585<br>993586<br>993587<br>993588<br>993589	3923258<br>3923259<br>3923260<br>3923261<br>3923262<br>3923263<br>3923264<br>3923265<br>3923266<br>3923267<br>3923268<br>3923269<br>3923270<br>3923271<br>3923272<br>3923273<br>3923274<br>3923275<br>3923276<br>3923277<br>3923278<br>3923279<br>3923280<br>3923281<br>3923282	core<br>core<br>extended<br>extended<br>extended<br>extended<br>extended<br>extended<br>extended<br>core<br>core<br>core<br>core<br>core<br>core<br>extended<br>core<br>core<br>full<br>extended<br>core<br>full<br>extended<br>extended<br>core	21	43963416	44006601	+	NM_032920<br>NM_003681<br>AK094630<br>AY303972<br>BC005825<br>AL355713<br>CR613269<br>ENSESTT00000033512<br>ENSESTT00000033513<br>ENSESTT00000033514<br>ENSESTT00000033515<br>ENSESTT00000033516<br>ENST00000291565<br>ENST00000327574<br>ENST00000343528<br>GENSCAN00000014031	Homo sapiens chromosome 21 open reading frame 124 (C21orf124), mRNA.<br>Homo sapiens pyridoxal (pyridoxine, vitamin B6) kinase (PDXK), mRNA.<br>Homo sapiens cDNA FLJ37311 fis, clone BRAMY2016771.<br>Homo sapiens pyridoxal kinase mRNA, complete cds.<br>Homo sapiens pyridoxal (pyridoxine, vitamin B6) kinase, mRNA (cDNA clone MGC:1687 IMAGE:2989843), complete cds.<br>Homo sapiens EST from clone 284549, full insert.<br>full-length cDNA clone CS0DI073YB04 of Placenta Cot 25-normalized of Homo sapiens (human).<br><br><br><br><br><br>cdna:known-ccds chromosome:NCBI36:21:43963406:44006608:1 gene:ENSG00000160209 CCDS13699.1<br>cdna:known-ccds chromosome:NCBI36:21:43973209:43983167:1 gene:ENSG00000160209 CCDS13700.1<br>cdna:known chromosome:NCBI36:21:43963406:44006608:1 gene:ENSG00000160209<br>cdna:Genscan chromosome:NCBI36:21:43961377:43965507:1

		3923283<br>3923284<br>3923285<br>3923286<br>3923287<br>3923288<br>3923289<br>3923290<br>3923291<br>3923292<br>3923293<br>3923294<br>3923295<br>3923296<br>3923297<br>3923298<br>3923299<br>3923300<br>3923301<br>3923302<br>3923303<br>3923304<br>3923305	extended<br>extended<br>extended<br>extended<br>core<br>core<br>core<br>core<br>extended<br>core<br>core<br>core<br>core<br>core<br>core<br>core<br>core<br>core<br>core<br>core<br>core<br>core<br>core						
3923312	993593<br>993594<br>993595<br>993596<br>993597<br>993598<br>993599<br>993600<br>993601<br>993602<br>993603<br>993604<br>993605<br>993606<br>993607<br>993608<br>993609<br>993610<br>993611<br>993612<br>993613<br>993614<br>993615<br>993616<br>993617<br>993618<br>993619<br>993620<br>993621<br>993622	3923313<br>3923314<br>3923315<br>3923316<br>3923317<br>3923318<br>3923319<br>3923320<br>3923321<br>3923322<br>3923323<br>3923324<br>3923325<br>3923326<br>3923327<br>3923328<br>3923329<br>3923330<br>3923331<br>3923332<br>3923333<br>3923334<br>3923335<br>3923336<br>3923337<br>3923338<br>3923339<br>3923340<br>3923341<br>3923342<br>3923343	extended<br>full<br>full<br>core<br>core<br>core<br>extended<br>full<br>core<br>core<br>extended<br>core<br>core<br>core<br>core<br>core<br>core<br>core<br>core<br>full<br>core<br>core<br>full<br>full<br>core<br>extended<br>core<br>core<br>core<br>extended<br>extended	21	44020499	44080008	+	NM_003683<br>AK026354<br>AY033999<br>AL137757<br>ENSESTT00000033517<br>ENSESTT00000033518<br>ENST00000291569<br>GENSCAN00000025060<br>GENSCAN00000025057	Homo sapiens DNA segment on chromosome 21 (unique) 2056 expressed sequence (D21S2056E), mRNA.<br>Homo sapiens cDNA: FLJ22701 fis, clone HSI12115.<br>Homo sapiens NNP3 mRNA, complete cds.<br>Homo sapiens mRNA; cDNA DKFZp434C085 (from clone DKFZp434C085); partial cds.<br><br><br>cdna:known chromosome:NCBI36:21:44033859:44049585:1 gene:ENSG00000160214<br>cdna:Genscan chromosome:NCBI36:21:44033939:44048083:1<br>cdna:Genscan chromosome:NCBI36:21:44054437:44080008:1

		3923344<br>3923345<br>3923346<br>3923347<br>3923348<br>3923349<br>3923350<br>3923351<br>3923352<br>3923353	full<br>full<br>full<br>full<br>full<br>full<br>extended<br>extended<br>full<br>full						
3924144	994118<br>994119<br>994120<br>994121<br>994122<br>994123<br>994124<br>994125<br>994126<br>994127<br>994128<br>994129<br>994130<br>994131<br>994132<br>994133<br>994134<br>994135<br>994136<br>994137<br>994138<br>994139<br>994140<br>994141<br>994142<br>994143<br>994144<br>994145<br>994146<br>994147<br>994148<br>994149<br>994150<br>994151<br>994152<br>994153<br>994154<br>994155<br>994156<br>994157<br>994158<br>994159<br>994160<br>994161	3924145<br>3924146<br>3924147<br>3924148<br>3924149<br>3924150<br>3924151<br>3924152<br>3924153<br>3924154<br>3924155<br>3924156<br>3924157<br>3924158<br>3924159<br>3924160<br>3924161<br>3924162<br>3924163<br>3924164<br>3924165<br>3924166<br>3924167<br>3924168<br>3924169<br>3924170<br>3924171<br>3924172<br>3924173<br>3924174<br>3924175<br>3924176<br>3924177<br>3924178<br>3924179<br>3924180<br>3924181<br>3924182<br>3924183<br>3924184<br>3924185<br>3924186<br>3924187<br>3924188	core<br>core<br>extended<br>extended<br>extended<br>extended<br>core<br>core<br>core<br>core<br>extended<br>extended<br>extended<br>extended<br>full<br>full<br>full<br>extended<br>full<br>core<br>core<br>core<br>extended<br>core<br>core<br>core<br>extended<br>core<br>core<br>core<br>core<br>core<br>core<br>core<br>core<br>full<br>core<br>core<br>extended<br>core<br>core<br>full<br>extended<br>core	21	45649495	45758055	+	NM_130444<br>NM_030582<br>NM_130445<br>AF018082<br>AK098216<br>BC013148<br>ENSESTT00000006909<br>ENSESTT00000006912<br>ENSESTT00000006913<br>ENSESTT00000006914<br>ENSESTT00000006915<br>ENST00000342220<br>ENST00000355480<br>ENST00000359759<br>GENSCAN00000052450<br>GENSCAN00000012167	Homo sapiens collagen, type XVIII, alpha 1 (COL18A1), transcript variant 3, mRNA.<br>Homo sapiens collagen, type XVIII, alpha 1 (COL18A1), transcript variant 1, mRNA.<br>Homo sapiens collagen, type XVIII, alpha 1 (COL18A1), transcript variant 2, mRNA.<br>Homo sapiens type XVIII collagen (COL18A1) mRNA, alternatively spliced, short form, complete cds.<br>Homo sapiens cDNA FLJ40897 fis, clone UTERU2002964, highly similar to Homo sapiens type XVIII collagen (COL18A1) mRNA.<br>Homo sapiens, clone IMAGE:3925108, mRNA.<br><br><br><br><br><br>cdna:known chromosome:NCBI36:21:45649516:45758061:1 gene:ENSG00000182871<br>cdna:known chromosome:NCBI36:21:45699833:45758061:1 gene:ENSG00000182871<br>cdna:known chromosome:NCBI36:21:45699873:45758061:1 gene:ENSG00000182871<br>cdna:Genscan chromosome:NCBI36:21:45685621:45756740:1<br>cdna:Genscan chromosome:NCBI36:21:45648352:45650508:1

	994162<br>994163<br>994164<br>994165<br>994166<br>994167<br>994168<br>994169<br>994170<br>994171<br>994172<br>994173<br>994174<br>994175<br>994176<br>994177<br>994178<br>994179<br>994180<br>994181<br>994182	3924189<br>3924190<br>3924191<br>3924192<br>3924193<br>3924194<br>3924195<br>3924196<br>3924197<br>3924198<br>3924199<br>3924200<br>3924201<br>3924202<br>3924203<br>3924204<br>3924205<br>3924206<br>3924207<br>3924208<br>3924209<br>3924210<br>3924211<br>3924212<br>3924213<br>3924214<br>3924215<br>3924216<br>3924217<br>3924218<br>3924219<br>3924220<br>3924221<br>3924222<br>3924223<br>3924224<br>3924225<br>3924226<br>3924227	core<br>core<br>full<br>core<br>extended<br>extended<br>core<br>core<br>core<br>core<br>core<br>extended<br>extended<br>extended<br>core<br>core<br>core<br>core<br>core<br>core<br>core<br>full<br>core<br>extended<br>extended<br>extended<br>extended<br>core<br>core<br>core<br>core<br>full<br>core<br>core<br>core<br>core<br>core<br>core<br>core						
3930360	997935<br>997936<br>997937<br>997938<br>997939<br>997940<br>997941<br>997942<br>997943<br>997944<br>997945<br>997946<br>997947<br>997948<br>997949	3930361<br>3930362<br>3930363<br>3930364<br>3930365<br>3930366<br>3930367<br>3930368<br>3930369<br>3930370<br>3930371<br>3930372<br>3930373<br>3930374<br>3930375	core<br>core<br>core<br>core<br>core<br>core<br>core<br>extended<br>extended<br>extended<br>extended<br>extended<br>core<br>full<br>extended	21	35081978	35343511	-	NM_001754<br>NM_001001890<br>AK026743<br>AK091167<br>AK130660<br>BC050363<br>X90976<br>X90978<br>X90981<br>D43967<br>S60998<br>S76345<br>S76346<br>ENSESTT00000044875<br>ENSESTT00000044876	Homo sapiens runt-related transcription factor 1 (acute myeloid leukemia 1; aml1 oncogene) (RUNX1), transcript variant 1, mRNA.<br>Homo sapiens runt-related transcription factor 1 (acute myeloid leukemia 1; aml1 oncogene) (RUNX1), transcript variant 2, mRNA.<br>Homo sapiens cDNA: FLJ23090 fis, clone LNG07119.<br>Homo sapiens cDNA FLJ33848 fis, clone CTONG2005567.<br>Homo sapiens cDNA FLJ27150 fis, clone SYN00521.<br>Homo sapiens, clone IMAGE:5725598, mRNA.<br>H.sapiens mRNA for an acute myeloid leukaemia protein (3917bp).<br>H.sapiens mRNA for an acute myeloid leukaemia protein (1793bp).<br>H.sapiens mRNA for an acute myeloid leukaemia protein (538bp).<br>Homo sapiens mRNA for AML1a protein, complete cds.<br>AML1=acute myeloid leukemia {alternatively spliced} [human, mRNA Partial, 894 nt].<br>AML1=AML1 {alternatively spliced, exons 5 and a} [human, mRNA Partial, 265 nt].<br>AML1=AML1 {alternatively spliced, exons 5 and b} [human, mRNA Partial, 284 nt].

	997950<br>997951<br>997952<br>997953<br>997954<br>997955<br>997956<br>997957<br>997958<br>997959<br>997960<br>997961<br>997962<br>997963<br>997964<br>997965<br>997966<br>997967<br>997968<br>997969<br>997970<br>997971<br>997972<br>997973<br>997974<br>997975<br>997976<br>997977<br>997978<br>997979<br>997980<br>997981<br>997982<br>997983<br>997984<br>997985<br>997986<br>997987<br>997988<br>997989<br>997990<br>997991<br>997992<br>997993<br>997994<br>997995<br>997996<br>997997<br>997998<br>997999<br>998000<br>998001<br>998002<br>998003	3930376<br>3930377<br>3930378<br>3930379<br>3930380<br>3930381<br>3930382<br>3930383<br>3930384<br>3930385<br>3930386<br>3930387<br>3930388<br>3930389<br>3930390<br>3930391<br>3930392<br>3930393<br>3930394<br>3930395<br>3930396<br>3930397<br>3930398<br>3930399<br>3930400<br>3930401<br>3930402<br>3930403<br>3930404<br>3930405<br>3930406<br>3930407<br>3930408<br>3930409<br>3930410<br>3930411<br>3930412<br>3930413<br>3930414<br>3930415<br>3930416<br>3930417<br>3930418<br>3930419<br>3930420<br>3930421<br>3930422<br>3930423<br>3930424<br>3930425<br>3930426<br>3930427<br>3930428<br>3930429	extended<br>full<br>full<br>full<br>full<br>full<br>full<br>full<br>full<br>full<br>full<br>full<br>full<br>full<br>full<br>full<br>core<br>core<br>extended<br>extended<br>extended<br>extended<br>full<br>extended<br>extended<br>extended<br>extended<br>extended<br>core<br>core<br>extended<br>extended<br>extended<br>extended<br>extended<br>core<br>full<br>full<br>extended<br>extended<br>extended<br>extended<br>extended<br>full<br>full<br>full<br>full<br>extended<br>extended<br>full<br>full<br>core<br>core<br>free					ENSESTT00000044877<br>ENSESTT00000044878<br>ENSESTT00000044879<br>ENSESTT00000044880<br>ENSESTT00000044881<br>ENST00000300305<br>ENST00000325074<br>ENST00000344691<br>ENST00000358356<br>ENST00000342083<br>GENSCAN00000065028<br>GENSCAN00000019438<br>GENSCAN00000024063	cdna:known-ccds chromosome:NCBI36:21:35081975:35343511:-1 gene:ENSG00000159216 CCDS13639.1<br>cdna:known chromosome:NCBI36:21:35081975:35183904:-1 gene:ENSG00000159216<br>cdna:known chromosome:NCBI36:21:35086146:35182857:-1 gene:ENSG00000159216<br>cdna:known chromosome:NCBI36:21:35115444:35182857:-1 gene:ENSG00000159216<br>cdna:known chromosome:NCBI36:21:35150580:35182857:-1 gene:ENSG00000159216<br>cdna:Genscan chromosome:NCBI36:21:35086302:35113505:-1<br>cdna:Genscan chromosome:NCBI36:21:35128577:35181279:-1<br>cdna:Genscan chromosome:NCBI36:21:35235463:35251998:-1

	998004	3930430	free						
	998005	3930431	extended						
	998006	3930432	extended						
	998007	3930433	core						
	998008	3930434	core						
	998009	3930435	core						
	998010	3930436	core						
	998011	3930437	core						
	998012	3930438	core						
	998013	3930439	core						
	998014	3930440	full						
	998015	3930441	full						
	998016	3930442	extended						
	998017	3930443	extended						
	998018	3930444	extended						
	998019	3930445	extended						
	998020	3930446	core						
	998021	3930447	extended						
	998022	3930448	extended						
	998023	3930449	extended						
	998024	3930450	extended						
	998025	3930451	extended						
	998026	3930452	full						
	998027	3930453	extended						
	998028	3930454	extended						
	998029	3930455	extended						
	998030	3930456	extended						
	998031	3930457	extended						
	998032	3930458	extended						
	998033	3930459	extended						
	998034	3930460	extended						
	998035	3930461	extended						
	998036	3930462	extended						
	998037	3930463	full						
	998038	3930464	full						
		3930465	full						
		3930466	full						
		3930467	full						
		3930468	extended						
		3930469	extended						
		3930470	full						
		3930471	extended						
		3930472	full						
		3930473	extended						
		3930474	extended						
		3930475	extended						
		3930476	extended						
		3930477	full						
		3930478	full						
		3930479	full						
		3930480	extended						
		3930481	extended						
		3930482	full						
		3930483	extended						

		3930484<br>3930485<br>3930486<br>3930487<br>3930488<br>3930489<br>3930490<br>3930491<br>3930492<br>3930493<br>3930494<br>3930495<br>3930496<br>3930497<br>3930498<br>3930499<br>3930500<br>3930501<br>3930502<br>3930503<br>3930504<br>3930505<br>3930506<br>3930507<br>3930508<br>3930509<br>3930510	extended<br>extended<br>extended<br>extended<br>full<br>extended<br>full<br>full<br>full<br>full<br>full<br>full<br>full<br>full<br>extended<br>extended<br>full<br>full<br>extended<br>extended<br>extended<br>extended<br>core<br>core<br>core<br>core<br>core						
3933205	999741<br>999742<br>999743<br>999744<br>999745<br>999746<br>999747<br>999748<br>999749<br>999750<br>999751<br>999752<br>999753<br>999754<br>999755<br>999756<br>999757<br>999758<br>999759<br>999760<br>999761	3933206<br>3933207<br>3933208<br>3933209<br>3933210<br>3933211<br>3933212<br>3933213<br>3933214<br>3933215<br>3933216<br>3933217<br>3933218<br>3933219<br>3933220<br>3933221<br>3933222<br>3933223<br>3933224<br>3933225<br>3933226<br>3933227<br>3933228<br>3933229<br>3933230<br>3933231<br>3933232	core<br>core<br>core<br>core<br>core<br>core<br>extended<br>core<br>core<br>extended<br>extended<br>full<br>full<br>core<br>core<br>core<br>full<br>full<br>full<br>full<br>full<br>full<br>full<br>core<br>full<br>full<br>full	21	42032613	42060325	-	NM_020639<br>ENSESTT00000046202<br>ENSESTT00000046203<br>ENST00000332512<br>ENST00000352483<br>ENST00000330470<br>GENSCAN00000014337	Homo sapiens receptor-interacting serine-threonine kinase 4 (RIPK4), mRNA.<br><br><br>cdna:known-ccds chromosome:NCBI36:21:42032614:42060335:-1 gene:ENSG00000183421 CCDS13675.1<br>cdna:known chromosome:NCBI36:21:42032614:42060335:-1 gene:ENSG00000183421<br>cdna:known chromosome:NCBI36:21:42034423:42060335:-1 gene:ENSG00000183421<br>cdna:Genscan chromosome:NCBI36:21:42060089:42060270:-1

		3933233<br>3933234<br>3933235<br>3933236<br>3933237<br>3933238<br>3933239<br>3933240	full<br>full<br>full<br>extended<br>extended<br>extended<br>core<br>extended						
3933566	999956<br>999957<br>999958<br>999959<br>999960<br>999961<br>999962<br>999963<br>999964<br>999965<br>999966<br>999967<br>999968<br>999969<br>999970<br>999971<br>999972<br>999973<br>999974<br>999975<br>999976<br>999977<br>999978<br>999979<br>999980<br>999981	3933567<br>3933568<br>3933569<br>3933570<br>3933571<br>3933572<br>3933573<br>3933574<br>3933575<br>3933576<br>3933577<br>3933578<br>3933579<br>3933580<br>3933581<br>3933582<br>3933583<br>3933584<br>3933585<br>3933586<br>3933587<br>3933588<br>3933589<br>3933590<br>3933591<br>3933592<br>3933593<br>3933594<br>3933595<br>3933596<br>3933597<br>3933598<br>3933599<br>3933600<br>3933601<br>3933602<br>3933603<br>3933604	full<br>full<br>core<br>full<br>full<br>full<br>full<br>full<br>core<br>core<br>core<br>core<br>core<br>core<br>core<br>full<br>full<br>core<br>core<br>core<br>full<br>full<br>extended<br>extended<br>core<br>core<br>core<br>core<br>core<br>core<br>extended<br>extended<br>core<br>core<br>full<br>full<br>core<br>extended	21	42662437	42699567	-	NM_032401<br>NM_024022<br>NM_032405<br>NM_032404<br>AK131212<br>ENSESTT00000046183<br>ENSESTT00000046184<br>ENSESTT00000046185<br>ENSESTT00000046186<br>ENSESTT00000046187<br>ENST00000291532<br>ENST00000380399<br>GENSCAN00000054753	Homo sapiens transmembrane protease, serine 3 (TMPRSS3), transcript variant B, mRNA.<br>Homo sapiens transmembrane protease, serine 3 (TMPRSS3), transcript variant A, mRNA.<br>Homo sapiens transmembrane protease, serine 3 (TMPRSS3), transcript variant D, mRNA.<br>Homo sapiens transmembrane protease, serine 3 (TMPRSS3), transcript variant C, mRNA.<br>Homo sapiens cDNA FLJ16093 fis, clone PROST2000452, moderately similar to TRANSMEMBRANE PROTEASE, SERINE 2 (EC 3.4.21.-).<br>cdna:known-ccds chromosome:NCBI36:21:42665069:42690024:-1 gene:ENSG00000160183 CCDS13686.1<br>cdna:known chromosome:NCBI36:21:42665069:42690024:-1 gene:ENSG00000160183<br>cdna:Genscan chromosome:NCBI36:21:42665905:42688595:-1
3935016	1000773<br>1000774<br>1000775<br>1000776<br>1000777<br>1000778<br>1000779	3935017<br>3935018<br>3935019<br>3935020<br>3935021<br>3935022<br>3935023	extended<br>extended<br>extended<br>extended<br>extended<br>extended<br>core	21	45746875	45832626	-	NM_194255<br>NM_003056<br>AB209069<br>AK127669<br>BC003068<br>BC023005<br>CR602937	Homo sapiens solute carrier family 19 (folate transporter), member 1 (SLC19A1), transcript variant 2, mRNA.<br>Homo sapiens solute carrier family 19 (folate transporter), member 1 (SLC19A1), transcript variant 1, mRNA.<br>Homo sapiens mRNA for solute carrier family 19 member 1 isoform b variant protein.<br>Homo sapiens cDNA FLJ45767 fis, clone NETRP2000439.<br>Homo sapiens solute carrier family 19 (folate transporter), member 1, mRNA (cDNA clone MGC:2165 IMAGE:3509422), complete cds.<br>Homo sapiens, clone IMAGE:4994464, mRNA.

	1000780<br>1000781<br>1000782<br>1000783<br>1000784<br>1000785<br>1000786<br>1000787<br>1000788<br>1000789<br>1000790<br>1000791<br>1000792<br>1000793<br>1000794<br>1000795<br>1000796<br>1000797<br>1000798<br>1000799<br>1000800<br>1000801<br>1000802<br>1000803<br>1000804<br>1000805<br>1000806<br>1000807	3935024<br>3935025<br>3935026<br>3935027<br>3935028<br>3935029<br>3935030<br>3935031<br>3935032<br>3935033<br>3935034<br>3935035<br>3935036<br>3935037<br>3935038<br>3935039<br>3935040<br>3935041<br>3935042<br>3935043<br>3935044<br>3935045<br>3935046<br>3935047<br>3935048<br>3935049<br>3935050<br>3935051<br>3935052<br>3935053<br>3935054<br>3935055<br>3935056<br>3935057<br>3935058<br>3935059<br>3935060<br>3935061<br>3935062<br>3935063<br>3935064<br>3935065<br>3935066<br>3935067<br>3935068	core<br>core<br>core<br>core<br>core<br>full<br>full<br>full<br>core<br>core<br>core<br>core<br>extended<br>extended<br>extended<br>extended<br>extended<br>full<br>extended<br>full<br>core<br>core<br>core<br>core<br>core<br>core<br>extended<br>full<br>extended<br>extended<br>full<br>extended<br>extended<br>full<br>full<br>full<br>full<br>full<br>full<br>full<br>full<br>extended<br>full<br>full<br>extended					ENSESTT00000006916<br>ENSESTT00000006917<br>ENST00000311124<br>ENST00000380010<br>ENST00000380014<br>ENST00000344609<br>GENSCAN00000036250<br>GENSCAN00000052457	full-length cDNA clone CS0DB008YI05 of Neuroblastoma Cot 10-normalized of Homo sapiens (human).<br><br><br>cdna:known-ccds chromosome:NCBI36:21:45759057:45786779:-1 gene:ENSG00000173638 CCDS13725.1<br>cdna:known-ccds chromosome:NCBI36:21:45759057:45786779:-1 gene:ENSG00000173638 CCDS13726.1<br>cdna:known chromosome:NCBI36:21:45737914:45775883:-1 gene:ENSG00000173638<br>cdna:known chromosome:NCBI36:21:45759057:45786690:-1 gene:ENSG00000173638<br>cdna:Genscan chromosome:NCBI36:21:45818397:45829606:-1<br>cdna:Genscan chromosome:NCBI36:21:45760000:45803767:-1
3939470	1003304<br>1003305<br>1003306<br>1003307<br>1003308<br>1003309<br>1003310<br>1003311	3939471<br>3939472<br>3939473<br>3939474<br>3939475<br>3939476<br>3939477<br>3939478<br>3939479	extended<br>extended<br>full<br>core<br>core<br>core<br>core<br>core<br>extended	22	22440423	22456501	+	NM_005940<br>AK125911<br>CR626443<br>ENSESTT00000003878<br>ENSESTT00000003879<br>GENSCAN00000024043	Homo sapiens matrix metallopeptidase 11 (stromelysin 3) (MMP11), mRNA.<br>Homo sapiens cDNA FLJ43923 fis, clone TESTI4012448, highly similar to Stromelysin-3 precursor (EC 3.4.24.-).<br>full-length cDNA clone CS0DI013YL21 of Placenta Cot 25-normalized of Homo sapiens (human).<br><br><br>cdna:Genscan chromosome:NCBI36:22:22445058:22475609:1

		3939480<br>3939481<br>3939482<br>3939483<br>3939484<br>3939485<br>3939486<br>3939487<br>3939488<br>3939489<br>3939490<br>3939491<br>3939492<br>3939493<br>3939494<br>3939495	core<br>core<br>core<br>core<br>extended<br>core<br>extended<br>extended<br>core<br>core<br>extended<br>core<br>core<br>core<br>core<br>core						
3942681	1005166<br>1005167<br>1005168<br>1005169<br>1005170<br>1005171<br>1005172<br>1005173<br>1005174<br>1005175<br>1005176<br>1005177<br>1005178<br>1005179<br>1005180<br>1005181<br>1005182<br>1005183<br>1005184<br>1005185<br>1005186<br>1005187<br>1005188<br>1005189<br>1005190<br>1005191<br>1005192<br>1005193<br>1005194<br>1005195<br>1005196<br>1005197<br>1005198<br>1005199<br>1005200<br>1005201<br>1005202	3942682<br>3942683<br>3942684<br>3942685<br>3942686<br>3942687<br>3942688<br>3942689<br>3942690<br>3942691<br>3942692<br>3942693<br>3942694<br>3942695<br>3942696<br>3942697<br>3942698<br>3942699<br>3942700<br>3942701<br>3942702<br>3942703<br>3942704<br>3942705<br>3942706<br>3942707<br>3942708<br>3942709<br>3942710<br>3942711<br>3942712<br>3942713<br>3942714<br>3942715<br>3942716<br>3942717<br>3942718<br>3942719	extended<br>full<br>full<br>full<br>full<br>full<br>extended<br>extended<br>full<br>extended<br>full<br>full<br>extended<br>full<br>core<br>core<br>extended<br>extended<br>extended<br>extended<br>extended<br>extended<br>extended<br>core<br>core<br>extended<br>extended<br>core<br>core<br>core<br>core<br>extended<br>full<br>core<br>core<br>core<br>core<br>core	22	29790111	29830726	+	NM_134270<br>NM_134269<br>AB209643<br>AK092684<br>AK092939<br>AK095930<br>AK128627<br>AK128719<br>ENSESTT00000036599<br>ENSESTT00000036601<br>ENSESTT00000036603<br>ENSESTT00000036604<br>ENSESTT00000036606<br>ENSESTT00000036608<br>ENSESTT00000036609<br>ENST00000329852<br>GENSCAN00000056471	Homo sapiens smoothelin (SMTN), transcript variant 1, mRNA.<br>Homo sapiens smoothelin (SMTN), transcript variant 2, mRNA.<br>Homo sapiens mRNA for smoothelin isoform b variant protein.<br>Homo sapiens cDNA FLJ35365 fis, clone SKMUS2000873, highly similar to Smoothelin large isoform L2.<br>Homo sapiens cDNA FLJ35620 fis, clone SPLEN2010846.<br>Homo sapiens cDNA FLJ38611 fis, clone HEART2005610, highly similar to Homo sapiens smoothelin large isoform L2 (SMTN) mRNA.<br>Homo sapiens cDNA FLJ46786 fis, clone TRACH3028837, moderately similar to Smoothelin.<br>Homo sapiens cDNA FLJ46886 fis, clone UTERU3016308, moderately similar to Smoothelin.<br>cdna:known chromosome:NCBI36:22:29807305:29830609:1 gene:ENSG00000183963<br>cdna:Genscan chromosome:NCBI36:22:29790112:29830452:1

		3942720<br>3942721<br>3942722<br>3942723<br>3942724<br>3942725<br>3942726<br>3942727<br>3942728<br>3942729<br>3942730<br>3942731<br>3942732<br>3942733<br>3942734<br>3942735<br>3942736<br>3942737<br>3942738<br>3942739<br>3942740<br>3942741<br>3942742<br>3942743<br>3942744<br>3942745<br>3942746<br>3942747<br>3942748<br>3942749<br>3942750<br>3942751<br>3942752<br>3942753<br>3942754<br>3942755<br>3942756<br>3942757<br>3942758<br>3942759	core<br>core<br>full<br>extended<br>extended<br>extended<br>extended<br>extended<br>extended<br>extended<br>core<br>extended<br>core<br>extended<br>extended<br>core<br>core<br>extended<br>core<br>core<br>core<br>extended<br>extended<br>core<br>full<br>core<br>core<br>extended<br>extended<br>extended<br>extended<br>extended<br>extended<br>extended<br>extended<br>extended<br>extended<br>core<br>core<br>extended						
3950872	1010171<br>1010172<br>1010173<br>1010174<br>1010175<br>1010176<br>1010177<br>1010178<br>1010179<br>1010180<br>1010181<br>1010182<br>1010183<br>1010184	3950873<br>3950874<br>3950875<br>3950876<br>3950877<br>3950878<br>3950879<br>3950880<br>3950881<br>3950882<br>3950883<br>3950884<br>3950885<br>3950886	extended<br>core<br>extended<br>core<br>core<br>core<br>core<br>core<br>core<br>core<br>core<br>core<br>full<br>core	22	49293499	49310068	+	NM_152299<br>NM_014551<br>ENSESTT00000044456<br>ENSESTT00000044457<br>ENSESTT00000044458<br>ENST00000380768<br>ENST00000299821<br>ENST00000361482<br>GENSCAN00000046424	Homo sapiens kleisin beta (hCAP-H2), transcript variant 2, mRNA.<br>Homo sapiens kleisin beta (hCAP-H2), transcript variant 1, mRNA.<br><br><br><br>cdna:known-ccds chromosome:NCBI36:22:49293539:49308670:1 gene:ENSG00000025770 CCDS14094.1<br>cdna:known chromosome:NCBI36:22:49293532:49308753:1 gene:ENSG00000025770<br>cdna:known chromosome:NCBI36:22:49293532:49308753:1 gene:ENSG00000025770<br>cdna:Genscan chromosome:NCBI36:22:49293633:49308670:1

	1010185<br>1010186<br>1010187<br>1010188<br>1010189<br>1010190<br>1010191<br>1010192<br>1010193<br>1010194	3950887<br>3950888<br>3950889<br>3950890<br>3950891<br>3950892<br>3950893<br>3950894<br>3950895<br>3950896<br>3950897<br>3950898<br>3950899<br>3950900<br>3950901<br>3950902<br>3950903<br>3950904<br>3950905<br>3950906<br>3950907<br>3950908<br>3950909<br>3950910<br>3950911<br>3950912<br>3950913<br>3950914<br>3950915<br>3950916	full<br>core<br>core<br>core<br>core<br>core<br>core<br>extended<br>core<br>core<br>core<br>full<br>full<br>core<br>full<br>core<br>core<br>core<br>core<br>extended<br>core<br>core<br>core<br>full<br>core<br>core<br>core<br>core<br>core<br>extended						
3952825	1011309<br>1011310<br>1011311<br>1011312<br>1011313<br>1011314<br>1011315<br>1011316<br>1011317<br>1011318<br>1011319<br>1011320<br>1011321<br>1011322<br>1011323<br>1011324<br>1011325<br>1011326<br>1011327<br>1011328<br>1011329<br>1011330<br>1011331<br>1011332	3952826<br>3952827<br>3952828<br>3952829<br>3952830<br>3952831<br>3952832<br>3952833<br>3952834<br>3952835<br>3952836<br>3952837<br>3952838<br>3952839<br>3952840<br>3952841<br>3952842<br>3952843<br>3952844<br>3952845<br>3952846<br>3952847<br>3952848<br>3952849	extended<br>core<br>core<br>core<br>full<br>full<br>full<br>full<br>extended<br>extended<br>core<br>core<br>full<br>full<br>full<br>full<br>full<br>core<br>full<br>core<br>core<br>core<br>extended<br>full	22	18150747	18222562	-	NM_024627<br>NM_053004<br>AB051432<br>CR604918<br>BC008696<br>ENSESTT00000054227<br>ENSESTT00000054228<br>ENSESTT00000054229<br>ENST00000329517<br>ENST00000328554<br>GENSCAN00000059338	Homo sapiens hypothetical protein FLJ21125 (FLJ21125), mRNA.<br>Homo sapiens guanine nucleotide binding protein (G protein), beta polypeptide 1-like (GNB1L), mRNA.<br>Homo sapiens mRNA for KIAA1645 protein, partial cds.<br>full-length cDNA clone CS0DI021YF08 of Placenta Cot 25-normalized of Homo sapiens (human).<br>Homo sapiens hypothetical protein FLJ21125, mRNA (cDNA clone IMAGE:2820627), with apparent retained intron.<br><br><br><br>cdna:known-ccds chromosome:NCBI36:22:18150747:18222403:-1 gene:ENSG00000185838 CCDS13768.1<br>cdna:known-ccds chromosome:NCBI36:22:18213671:18222339:-1 gene:ENSG00000185838 CCDS13769.1<br>cdna:Genscan chromosome:NCBI36:22:18166274:18188898:-1

	1011333<br>1011334<br>1011335	3952850<br>3952851<br>3952852<br>3952853<br>3952854<br>3952855<br>3952856<br>3952857<br>3952858<br>3952859<br>3952860<br>3952861<br>3952862<br>3952863<br>3952864<br>3952865<br>3952866<br>3952867<br>3952868<br>3952869	full<br>full<br>full<br>full<br>core<br>core<br>core<br>core<br>core<br>core<br>core<br>core<br>core<br>core<br>core<br>core<br>core<br>extended<br>core<br>extended						
3955185	1012658<br>1012659<br>1012660<br>1012661<br>1012662<br>1012663<br>1012664<br>1012665<br>1012666<br>1012667<br>1012668<br>1012669<br>1012670<br>1012671<br>1012672<br>1012673<br>1012674	3955186<br>3955187<br>3955188<br>3955189<br>3955190<br>3955191<br>3955192<br>3955193<br>3955194<br>3955195<br>3955196<br>3955197<br>3955198<br>3955199<br>3955200<br>3955201<br>3955202<br>3955203<br>3955204<br>3955205<br>3955206<br>3955207	core<br>core<br>extended<br>extended<br>extended<br>core<br>core<br>core<br>core<br>extended<br>core<br>core<br>core<br>core<br>core<br>core<br>core<br>extended<br>full<br>full<br>core<br>core	22	22945631	22971090	-	NM_004121<br>AL117414<br>ENSESTT00000008153<br>ENSESTT00000008154<br>ENSESTT00000008155<br>ENST00000327365<br>GENSCAN00000065104	Homo sapiens gamma-glutamyltransferase-like activity 1 (GGTLA1), mRNA.<br>Homo sapiens mRNA; cDNA DKFZp566O011 (from clone DKFZp566O011).<br><br><br><br>cdna:known-ccds chromosome:NCBI36:22:22945628:22971100:-1 gene:ENSG00000099998 CCDS13825.1<br>cdna:Genscan chromosome:NCBI36:22:22945938:22951305:-1
3959388	1015225<br>1015226<br>1015227<br>1015228<br>1015229<br>1015230<br>1015231<br>1015232<br>1015233<br>1015234	3959389<br>3959390<br>3959391<br>3959392<br>3959393<br>3959394<br>3959395<br>3959396<br>3959397<br>3959398<br>3959399	extended<br>core<br>core<br>core<br>core<br>core<br>core<br>core<br>core<br>core<br>core	22	34914873	34930825	-	NM_145660<br>NM_030643<br>BC006276<br>ENSESTT00000045170<br>ENST00000332987<br>GENSCAN00000024540	Homo sapiens apolipoprotein L, 4 (APOL4), transcript variant b, mRNA.<br>Homo sapiens apolipoprotein L, 4 (APOL4), transcript variant a, mRNA.<br>Homo sapiens apolipoprotein L, 4, mRNA (cDNA clone IMAGE:3956995), complete cds.<br><br>cdna:known chromosome:NCBI36:22:34917069:34930825:-1 gene:ENSG00000100336<br>cdna:Genscan chromosome:NCBI36:22:34917069:34928028:-1

		3959400<br>3959401<br>3959402<br>3959403<br>3959404	extended<br>core<br>core<br>core<br>core						
3960478	1015886<br>1015887<br>1015888<br>1015889<br>1015890<br>1015891<br>1015892<br>1015893<br>1015894<br>1015895<br>1015896<br>1015897<br>1015898<br>1015899<br>1015900<br>1015901<br>1015902<br>1015903<br>1015904<br>1015905<br>1015906<br>1015907<br>1015908<br>1015909<br>1015910<br>1015911<br>1015912<br>1015913<br>1015914<br>1015915<br>1015916<br>1015917<br>1015918<br>1015919<br>1015920<br>1015921<br>1015922<br>1015923<br>1015924<br>1015925<br>1015926<br>1015927<br>1015928<br>1015929<br>1015930<br>1015931<br>1015932<br>1015933<br>1015934	3960479<br>3960480<br>3960481<br>3960482<br>3960483<br>3960484<br>3960485<br>3960486<br>3960487<br>3960488<br>3960489<br>3960490<br>3960491<br>3960492<br>3960493<br>3960494<br>3960495<br>3960496<br>3960497<br>3960498<br>3960499<br>3960500<br>3960501<br>3960502<br>3960503<br>3960504<br>3960505<br>3960506<br>3960507<br>3960508<br>3960509<br>3960510<br>3960511<br>3960512<br>3960513<br>3960514<br>3960515<br>3960516<br>3960517<br>3960518<br>3960519<br>3960520<br>3960521<br>3960522<br>3960523<br>3960524<br>3960525<br>3960526<br>3960527	full<br>full<br>full<br>core<br>core<br>core<br>core<br>core<br>extended<br>extended<br>extended<br>core<br>core<br>extended<br>extended<br>core<br>core<br>extended<br>core<br>extended<br>extended<br>extended<br>extended<br>extended<br>extended<br>extended<br>extended<br>extended<br>extended<br>extended<br>extended<br>extended<br>extended<br>core<br>extended<br>core<br>core<br>extended<br>core<br>core<br>extended<br>extended<br>extended<br>extended<br>extended<br>extended<br>extended<br>extended<br>core	22	37010115	37150916	-	NM_001894<br>NM_152221<br>XM_929784<br>AK057067<br>AK074438<br>AK092269<br>CR590991<br>CR618521<br>CR619665<br>ENSESTT00000023405<br>ENSESTT00000023406<br>ENSESTT00000023407<br>ENSESTT00000023408<br>ENSESTT00000023409<br>ENSESTT00000023410<br>ENSESTT00000023411<br>ENSESTT00000023412<br>ENSESTT00000023413<br>ENST00000381646<br>ENST00000381645<br>GENSCAN00000063986<br>GENSCAN00000028336<br>GENSCAN00000058420	Homo sapiens casein kinase 1, epsilon (CSNK1E), transcript variant 2, mRNA.<br>Homo sapiens casein kinase 1, epsilon (CSNK1E), transcript variant 1, mRNA.<br>PREDICTED: Homo sapiens KIAA1660 protein (KIAA1660), mRNA.<br>Homo sapiens cDNA FLJ32505 fis, clone SMINT1000039.<br>Homo sapiens cDNA FLJ23858 fis, clone LNG07565.<br>Homo sapiens cDNA FLJ34950 fis, clone NT2RP7017284, highly similar to CASEIN KINASE I, EPSILON ISOFORM (EC 2.7.1.-).<br>full-length cDNA clone CS0DK002YC23 of HeLa cells Cot 25-normalized of Homo sapiens (human).<br>full-length cDNA clone CS0DK002YK23 of HeLa cells Cot 25-normalized of Homo sapiens (human).<br>full-length cDNA clone CS0DF038YM04 of Fetal brain of Homo sapiens (human).<br>cdna:known chromosome:NCBI36:22:37073488:37124473:-1 gene:ENSG00000100181<br>cdna:known chromosome:NCBI36:22:37087346:37123367:-1 gene:ENSG00000100181<br>cdna:Genscan chromosome:NCBI36:22:37010115:37040108:-1<br>cdna:Genscan chromosome:NCBI36:22:37123259:37123367:-1<br>cdna:Genscan chromosome:NCBI36:22:37060787:37107935:-1

	1015935	3960528	extended						
	1015936	3960529	extended						
	1015937	3960530	core						
	1015938	3960531	extended						
	1015939	3960532	full						
	1015940	3960533	extended						
	1015941	3960534	extended						
	1015942	3960535	extended						
	1015943	3960536	extended						
	1015944	3960537	extended						
	1015945	3960538	extended						
	1015946	3960539	extended						
	1015947	3960540	extended						
	1015948	3960541	extended						
	1015949	3960542	extended						
	1015950	3960543	full						
	1015951	3960544	extended						
	1015952	3960545	extended						
	1015953	3960546	extended						
	1015954	3960547	full						
	1015955	3960548	full						
	1015956	3960549	full						
	1015957	3960550	extended						
	1015958	3960551	extended						
	1015959	3960552	extended						
	1015960	3960553	extended						
	1015961	3960554	extended						
	1015962	3960555	extended						
	1015963	3960556	extended						
	1015964	3960557	extended						
	1015965	3960558	extended						
	1015966	3960559	extended						
	1015967	3960560	extended						
	1015968	3960561	extended						
	1015969	3960562	extended						
	1015970	3960563	extended						
		3960564	extended						
		3960565	extended						
		3960566	extended						
		3960567	extended						
		3960568	extended						
		3960569	extended						
		3960570	extended						
		3960571	extended						
		3960572	extended						
		3960573	full						
		3960574	full						
		3960575	extended						
		3960576	extended						
		3960577	full						
		3960578	full						
		3960579	full						
		3960580	extended						
		3960581	full						

		3960582<br>3960583<br>3960584<br>3960585<br>3960586<br>3960587<br>3960588<br>3960589<br>3960590<br>3960591<br>3960592<br>3960593<br>3960594<br>3960595<br>3960596<br>3960597<br>3960598<br>3960599	full<br>extended<br>extended<br>extended<br>full<br>extended<br>full<br>extended<br>extended<br>full<br>full<br>full<br>full<br>full<br>full<br>full<br>full<br>full						
3964049	1017988<br>1017989<br>1017990<br>1017991<br>1017992<br>1017993<br>1017994<br>1017995<br>1017996<br>1017997<br>1017998<br>1017999<br>1018000<br>1018001<br>1018002<br>1018003<br>1018004<br>1018005<br>1018006<br>1018007<br>1018008<br>1018009<br>1018010<br>1018011<br>1018012<br>1018013<br>1018014<br>1018015<br>1018016<br>1018017<br>1018018<br>1018019<br>1018020<br>1018021<br>1018022<br>1018023	3964050<br>3964051<br>3964052<br>3964053<br>3964054<br>3964055<br>3964056<br>3964057<br>3964058<br>3964059<br>3964060<br>3964061<br>3964062<br>3964063<br>3964064<br>3964065<br>3964066<br>3964067<br>3964068<br>3964069<br>3964070<br>3964071<br>3964072<br>3964073<br>3964074<br>3964075<br>3964076<br>3964077<br>3964078<br>3964079<br>3964080<br>3964081<br>3964082<br>3964083<br>3964084<br>3964085	core<br>core<br>extended<br>core<br>core<br>core<br>core<br>core<br>core<br>core<br>core<br>full<br>core<br>full<br>core<br>core<br>full<br>full<br>core<br>core<br>core<br>core<br>core<br>core<br>extended<br>core<br>core<br>core<br>extended<br>core<br>core<br>full<br>full<br>core<br>core<br>core	22	45135395	45311820	-	XM_937419<br>NM_014246<br>BC000059<br>AL831846<br>ENSESTT00000016721<br>GENSCAN00000010112<br>GENSCAN00000010118<br>GENSCAN00000010114<br>GENSCAN00000046451<br>GENSCAN00000046453<br>GENSCAN00000046449<br>GENSCAN00000059163	PREDICTED: Homo sapiens similar to CG5327-PA (LOC648363), mRNA.<br>Homo sapiens cadherin, EGF LAG seven-pass G-type receptor 1 (flamingo homolog, Drosophila) (CELSR1), mRNA.<br>Homo sapiens cadherin, EGF LAG seven-pass G-type receptor 1 (flamingo homolog, Drosophila), mRNA (cDNA clone IMAGE:3506648), partial cds.<br>Homo sapiens mRNA; cDNA DKFZp434P0729 (from clone DKFZp434P0729).<br><br>cdna:Genscan chromosome:NCBI36:22:45167753:45203023:-1<br>cdna:Genscan chromosome:NCBI36:22:45143858:45166509:-1<br>cdna:Genscan chromosome:NCBI36:22:45138553:45142448:-1<br>cdna:Genscan chromosome:NCBI36:22:45291947:45301557:-1<br>cdna:Genscan chromosome:NCBI36:22:45232530:45239932:-1<br>cdna:Genscan chromosome:NCBI36:22:45207954:45214809:-1<br>cdna:Genscan chromosome:NCBI36:17:59041861:59042278:-1

	1018024<br>1018025<br>1018026<br>1018027<br>1018028<br>1018029<br>1018030<br>1018031<br>1018032<br>1018033<br>1018034<br>1018035<br>1018036<br>1018037<br>1018038<br>1018039<br>1018040<br>1018041<br>1018042<br>1018043<br>1018044<br>1018045<br>1018046<br>1018047<br>1018048<br>1018049	3964086<br>3964087<br>3964088<br>3964089<br>3964090<br>3964091<br>3964092<br>3964093<br>3964094<br>3964095<br>3964096<br>3964097<br>3964098<br>3964099<br>3964100<br>3964101<br>3964102<br>3964103<br>3964104<br>3964105<br>3964106<br>3964107<br>3964108<br>3964109<br>3964110<br>3964111<br>3964112<br>3964113<br>3964114<br>3964115<br>3964116<br>3964117<br>3964118<br>3964119<br>3964120<br>3964121	core<br>core<br>core<br>extended<br>full<br>core<br>core<br>core<br>extended<br>full<br>full<br>core<br>core<br>core<br>full<br>full<br>extended<br>core<br>full<br>full<br>full<br>full<br>extended<br>extended<br>full<br>full<br>full<br>extended<br>extended<br>full<br>extended<br>core<br>core<br>core<br>core<br>full						
3972093	1023031<br>1023032<br>1023033<br>1023034<br>1023035<br>1023036<br>1023037<br>1023038<br>1023039<br>1023040<br>1023041<br>1023042<br>1023043<br>1023044<br>1023045<br>1023046<br>1023047<br>1023048	3972094<br>3972095<br>3972096<br>3972097<br>3972098<br>3972099<br>3972100<br>3972101<br>3972102<br>3972103<br>3972104<br>3972105<br>3972106<br>3972107<br>3972108<br>3972109<br>3972110<br>3972111	extended<br>core<br>full<br>full<br>core<br>core<br>full<br>core<br>core<br>core<br>core<br>core<br>core<br>core<br>core<br>extended<br>core<br>core	X	24621967	24925014	+	NR_003007<br>NM_016937<br>BX648513<br>AF085825<br>CR625340<br>ENSESTT00000043783<br>ENSESTT00000043784<br>ENSESTT00000043785<br>ENSESTT00000043786<br>ENSESTT00000043787<br>ENST00000379059<br>ENST00000325036<br>ENST00000379068<br>GENSCAN00000055156<br>GENSCAN00000024211<br>GENSCAN00000042893<br>GENSCAN00000042890	Homo sapiens small Cajal body-specific RNA 23 (SCARNA23) on chromosome X.<br>Homo sapiens polymerase (DNA directed), alpha (POLA), mRNA.<br>Homo sapiens mRNA; cDNA DKFZp686K1672 (from clone DKFZp686K1672).<br>Homo sapiens full length insert cDNA clone YF46C08.<br>full-length cDNA clone CS0DI005YF08 of Placenta Cot 25-normalized of Homo sapiens (human).<br><br><br><br><br><br>cdna:known-ccds chromosome:NCBI36:X:24621985:24925024:1 gene:ENSG00000101868 CCDS14214.1<br>cdna:known chromosome:NCBI36:X:24621985:24925021:1 gene:ENSG00000101868<br>cdna:novel chromosome:NCBI36:X:24621957:24925024:1 gene:ENSG00000101868<br>cdna:Genscan chromosome:NCBI36:X:24622000:24655980:1<br>cdna:Genscan chromosome:NCBI36:X:24830939:24863774:1<br>cdna:Genscan chromosome:NCBI36:X:24737957:24781243:1<br>cdna:Genscan chromosome:NCBI36:X:24660408:24689972:1

	1023049	3972112	core						
	1023050	3972113	core						
	1023051	3972114	core						
	1023052	3972115	core						
	1023053	3972116	extended						
	1023054	3972117	core						
	1023055	3972118	core						
	1023056	3972119	extended						
	1023057	3972120	core						
	1023058	3972121	core						
	1023059	3972122	core						
	1023060	3972123	core						
	1023061	3972124	core						
	1023062	3972125	extended						
	1023063	3972126	core						
	1023064	3972127	core						
	1023065	3972128	full						
	1023066	3972129	core						
	1023067	3972130	core						
	1023068	3972131	core						
	1023069	3972132	full						
	1023070	3972133	full						
	1023071	3972134	full						
	1023072	3972135	extended						
	1023073	3972136	full						
	1023074	3972137	extended						
	1023075	3972138	extended						
	1023076	3972139	extended						
	1023077	3972140	full						
	1023078	3972141	extended						
	1023079	3972142	extended						
	1023080	3972143	full						
	1023081	3972144	full						
	1023082	3972145	full						
	1023083	3972146	full						
	1023084	3972147	core						
	1023085	3972148	extended						
	1023086	3972149	core						
	1023087	3972150	extended						
	1023088	3972151	extended						
	1023089	3972152	core						
	1023090	3972153	core						
	1023091	3972154	extended						
	1023092	3972155	core						
	1023093	3972156	core						
	1023094	3972157	full						
	1023095	3972158	core						
	1023096	3972159	extended						
	1023097	3972160	core						
	1023098	3972161	full						
	1023099	3972162	extended						
	1023100	3972163	full						
	1023101	3972164	full						
	1023102	3972165	free						

	1023103<br>1023104<br>1023105<br>1023106<br>1023107<br>1023108	3972166<br>3972167<br>3972168<br>3972169<br>3972170<br>3972171<br>3972172<br>3972173<br>3972174<br>3972175<br>3972176<br>3972177<br>3972178<br>3972179<br>3972180<br>3972181<br>3972182<br>3972183<br>3972184<br>3972185<br>3972186<br>3972187<br>3972188<br>3972189<br>3972190	core<br>full<br>full<br>full<br>full<br>extended<br>full<br>full<br>core<br>extended<br>full<br>extended<br>extended<br>extended<br>full<br>extended<br>extended<br>full<br>full<br>full<br>full<br>full<br>full<br>core<br>core						
3985169	1030867<br>1030868<br>1030869<br>1030870<br>1030871<br>1030872<br>1030873<br>1030874<br>1030875<br>1030876<br>1030877<br>1030878<br>1030879<br>1030880<br>1030881<br>1030882<br>1030883<br>1030884<br>1030885<br>1030886<br>1030887<br>1030888<br>1030889<br>1030890<br>1030891	3985170<br>3985171<br>3985172<br>3985173<br>3985174<br>3985175<br>3985176<br>3985177<br>3985178<br>3985179<br>3985180<br>3985181<br>3985182<br>3985183<br>3985184<br>3985185<br>3985186<br>3985187<br>3985188<br>3985189<br>3985190<br>3985191<br>3985192<br>3985193<br>3985194<br>3985195<br>3985196<br>3985197<br>3985198	full<br>full<br>full<br>core<br>core<br>core<br>core<br>full<br>core<br>full<br>core<br>core<br>full<br>core<br>core<br>core<br>core<br>core<br>core<br>core<br>full<br>core<br>full<br>full<br>core<br>full<br>full<br>core<br>core	X	101687459	101735422	+	NR_002216<br>ENST00000360035<br>GENSCAN00000002018	Homo sapiens nuclear RNA export factor 4 pseudogene (NXF4) on chromosome X.<br>cdna:known-ccds chromosome:NCBI36:X:101691549:101713277:1 gene:ENSG00000196970 CCDS14499.1<br>cdna:Genscan chromosome:NCBI36:X:101689785:101745402:1

		3985199<br>3985200<br>3985201<br>3985202<br>3985203<br>3985204<br>3985205<br>3985206<br>3985207<br>3985208<br>3985209<br>3985210<br>3985211<br>3985212<br>3985213<br>3985214<br>3985215<br>3985216<br>3985217	core<br>core<br>full<br>core<br>core<br>core<br>core<br>core<br>core<br>core<br>core<br>core<br>core<br>core<br>core<br>core<br>full<br>full<br>full						
3985717	1031165<br>1031166<br>1031167<br>1031168<br>1031169<br>1031170<br>1031171<br>1031172<br>1031173<br>1031174<br>1031175<br>1031176<br>1031177<br>1031178<br>1031179<br>1031180<br>1031181<br>1031182<br>1031183<br>1031184<br>1031185<br>1031186<br>1031187<br>1031188<br>1031189<br>1031190<br>1031191<br>1031192<br>1031193<br>1031194<br>1031195<br>1031196<br>1031197<br>1031198<br>1031199	3985718<br>3985719<br>3985720<br>3985721<br>3985722<br>3985723<br>3985724<br>3985725<br>3985726<br>3985727<br>3985728<br>3985729<br>3985730<br>3985731<br>3985732<br>3985733<br>3985734<br>3985735<br>3985736<br>3985737<br>3985738<br>3985739<br>3985740<br>3985741<br>3985742<br>3985743<br>3985744<br>3985745<br>3985746<br>3985747<br>3985748<br>3985749<br>3985750<br>3985751<br>3985752	full<br>full<br>full<br>extended<br>full<br>extended<br>full<br>full<br>full<br>full<br>full<br>full<br>full<br>full<br>full<br>extended<br>extended<br>full<br>full<br>full<br>extended<br>core<br>core<br>full<br>full<br>full<br>full<br>full<br>full<br>full<br>full<br>extended<br>full<br>full<br>core	X	102876363	102934160	+	NM_199478<br>NM_000533<br>ENSESTT00000034957<br>ENSESTT00000034958<br>ENSESTT00000034959<br>ENSESTT00000034960<br>ENSESTT00000034961<br>ENSESTT00000034962<br>ENSESTT00000034963<br>ENSESTT00000034964<br>ENSESTT00000034965<br>ENSESTT00000034966<br>ENST00000303958<br>ENST00000361621<br>ENST00000372608<br>ENST00000372606<br>GENSCAN00000068947	Homo sapiens proteolipid protein 1 (Pelizaeus-Merzbacher disease, spastic paraplegia 2, uncomplicated) (PLP1), transcript variant 2, mRNA.<br>Homo sapiens proteolipid protein 1 (Pelizaeus-Merzbacher disease, spastic paraplegia 2, uncomplicated) (PLP1), transcript variant 1, mRNA.<br>cdna:known-ccds chromosome:NCBI36:X:102918410:102934201:1 gene:ENSG00000123560 CCDS14513.1<br>cdna:known-ccds chromosome:NCBI36:X:102918410:102934201:1 gene:ENSG00000123560 CCDS14514.1<br>cdna:known chromosome:NCBI36:X:102918434:102934204:1 gene:ENSG00000123560<br>cdna:known chromosome:NCBI36:X:102918434:102934204:1 gene:ENSG00000123560<br>cdna:Genscan chromosome:NCBI36:X:102918204:102932182:1

	1031200	3985753<br>3985754<br>3985755<br>3985756<br>3985757<br>3985758<br>3985759<br>3985760<br>3985761<br>3985762<br>3985763	core<br>core<br>core<br>extended<br>core<br>core<br>core<br>full<br>full<br>core<br>core						
3996667	1037749<br>1037750<br>1037751<br>1037752<br>1037753<br>1037754<br>1037755<br>1037756<br>1037757<br>1037758<br>1037759<br>1037760<br>1037761<br>1037762<br>1037763<br>1037764<br>1037765<br>1037766<br>1037767<br>1037768<br>1037769	3996668<br>3996669<br>3996670<br>3996671<br>3996672<br>3996673<br>3996674<br>3996675<br>3996676<br>3996677<br>3996678<br>3996679<br>3996680<br>3996681<br>3996682<br>3996683<br>3996684<br>3996685<br>3996686<br>3996687<br>3996688<br>3996689<br>3996690<br>3996691<br>3996692<br>3996693<br>3996694<br>3996695<br>3996696	core<br>core<br>extended<br>core<br>core<br>core<br>core<br>core<br>core<br>core<br>full<br>extended<br>core<br>full<br>full<br>core<br>core<br>core<br>extended<br>full<br>extended<br>core<br>full<br>core<br>extended<br>core<br>core<br>core<br>core	X	153644243	153659150	+	NR_002969<br>NM_001363<br>NR_002984<br>ENSESTT00000045529<br>ENSESTT00000045530<br>ENSESTT00000045531<br>ENSESTT00000045532<br>ENST00000369550<br>ENST00000253127<br>GENSCAN00000045573	Homo sapiens small nucleolar RNA, H/ACA box 36A (SNORA36A) on chromosome X.<br>Homo sapiens dyskeratosis congenita 1, dyskerin (DKC1), mRNA.<br>Homo sapiens small nucleolar RNA, H/ACA box 56 (SNORA56) on chromosome X.<br><br><br><br><br>cdna:known-ccds chromosome:NCBI36:X:153644229:153659158:1 gene:ENSG00000130826 CCDS14761.1<br>cdna:known chromosome:NCBI36:X:153644233:153659150:1 gene:ENSG00000130826<br>cdna:Genscan chromosome:NCBI36:X:153644435:153658336:1
3998766	1039044<br>1039045<br>1039046<br>1039047<br>1039048<br>1039049<br>1039050<br>1039051<br>1039052<br>1039053<br>1039054<br>1039055<br>1039056	3998767<br>3998768<br>3998769<br>3998770<br>3998771<br>3998772<br>3998773<br>3998774<br>3998775<br>3998776<br>3998777<br>3998778<br>3998779	full<br>full<br>full<br>core<br>core<br>core<br>core<br>core<br>core<br>core<br>full<br>core<br>extended	X	8448039	8691489	-	NM_000216<br>AK001102<br>BX647754<br>ENSESTT00000052686<br>ENSESTT00000052687<br>ENSESTT00000052688<br>ENSESTT00000052689<br>ENSESTT00000052690<br>ENST00000262648<br>GENSCAN00000062955<br>GENSCAN00000067774<br>GENSCAN00000067775<br>GENSCAN00000024169	Homo sapiens Kallmann syndrome 1 sequence (KAL1), mRNA.<br>Homo sapiens cDNA FLJ10240 fis, clone HEMBB1000591.<br>Homo sapiens mRNA; cDNA DKFZp686I0431 (from clone DKFZp686I0431).<br><br><br><br><br><br>cdna:known-ccds chromosome:NCBI36:X:8456915:8660227:-1 gene:ENSG00000011201 CCDS14130.1<br>cdna:Genscan chromosome:NCBI36:X:8625709:8660077:-1<br>cdna:Genscan chromosome:NCBI36:X:8513308:8527270:-1<br>cdna:Genscan chromosome:NCBI36:X:8546483:8581331:-1<br>cdna:Genscan chromosome:NCBI36:X:8494053:8498745:-1

	1039057<br>1039058<br>1039059<br>1039060<br>1039061<br>1039062<br>1039063<br>1039064<br>1039065<br>1039066<br>1039067<br>1039068<br>1039069<br>1039070<br>1039071<br>1039072<br>1039073<br>1039074<br>1039075<br>1039076<br>1039077<br>1039078<br>1039079<br>1039080<br>1039081<br>1039082<br>1039083<br>1039084<br>1039085<br>1039086<br>1039087<br>1039088	3998780<br>3998781<br>3998782<br>3998783<br>3998784<br>3998785<br>3998786<br>3998787<br>3998788<br>3998789<br>3998790<br>3998791<br>3998792<br>3998793<br>3998794<br>3998795<br>3998796<br>3998797<br>3998798<br>3998799<br>3998800<br>3998801<br>3998802<br>3998803<br>3998804<br>3998805<br>3998806<br>3998807<br>3998808<br>3998809<br>3998810<br>3998811<br>3998812<br>3998813<br>3998814<br>3998815<br>3998816<br>3998817<br>3998818<br>3998819<br>3998820<br>3998821<br>3998822<br>3998823<br>3998824<br>3998825<br>3998826<br>3998827<br>3998828	core<br>core<br>extended<br>core<br>core<br>full<br>full<br>core<br>extended<br>extended<br>extended<br>extended<br>extended<br>core<br>core<br>core<br>full<br>extended<br>extended<br>extended<br>extended<br>extended<br>extended<br>core<br>core<br>core<br>full<br>full<br>core<br>full<br>full<br>full<br>full<br>full<br>full<br>full<br>full<br>extended<br>full<br>extended<br>core<br>full<br>full<br>extended<br>core<br>core<br>core<br>full<br>full					GENSCAN00000024172	cdna:Genscan chromosome:NCBI36:X:8448039:8475422:-1
4004044	1042334<br>1042335<br>1042336<br>1042337<br>1042338	4004045<br>4004046<br>4004047<br>4004048<br>4004049	extended<br>full<br>core<br>core<br>core	X	31025735	33267637	-	NM_004012<br>NM_004016<br>NM_004015<br>NM_004022<br>NM_004006	Homo sapiens dystrophin (muscular dystrophy, Duchenne and Becker types) (DMD), transcript variant Dp260-2, mRNA.<br>Homo sapiens dystrophin (muscular dystrophy, Duchenne and Becker types) (DMD), transcript variant Dp71b, mRNA.<br>Homo sapiens dystrophin (muscular dystrophy, Duchenne and Becker types) (DMD), transcript variant Dp71, mRNA.<br>Homo sapiens dystrophin (muscular dystrophy, Duchenne and Becker types) (DMD), transcript variant D140ab, mRNA.<br>Homo sapiens dystrophin (muscular dystrophy, Duchenne and Becker types) (DMD), transcript variant Dp427m, mRNA.

	1042339	4004050	core					NM_004018	Homo sapiens dystrophin (muscular dystrophy, Duchenne and Becker types) (DMD), transcript variant Dp71ab, mRNA.
	1042340	4004051	core					NM_004023	Homo sapiens dystrophin (muscular dystrophy, Duchenne and Becker types) (DMD), transcript variant Dp140bc, mRNA.
	1042341	4004052	extended					NM_004019	Homo sapiens dystrophin (muscular dystrophy, Duchenne and Becker types) (DMD), transcript variant Dp40, mRNA.
	1042342	4004053	core					NM_004020	Homo sapiens dystrophin (muscular dystrophy, Duchenne and Becker types) (DMD), transcript variant Dp140c, mRNA.
	1042343	4004054	full					NM_000109	Homo sapiens dystrophin (muscular dystrophy, Duchenne and Becker types) (DMD), transcript variant Dp427c, mRNA.
	1042344	4004055	core					NM_004013	Homo sapiens dystrophin (muscular dystrophy, Duchenne and Becker types) (DMD), transcript variant Dp140, mRNA.
	1042345	4004056	extended					NM_004017	Homo sapiens dystrophin (muscular dystrophy, Duchenne and Becker types) (DMD), transcript variant Dp71a, mRNA.
	1042346	4004057	extended					NM_004011	Homo sapiens dystrophin (muscular dystrophy, Duchenne and Becker types) (DMD), transcript variant Dp260-1, mRNA.
	1042347	4004058	core					NM_004010	Homo sapiens dystrophin (muscular dystrophy, Duchenne and Becker types) (DMD), transcript variant Dp427p2, mRNA.
	1042348	4004059	core					NM_004009	Homo sapiens dystrophin (muscular dystrophy, Duchenne and Becker types) (DMD), transcript variant Dp427p1, mRNA.
	1042349	4004060	extended					NM_004014	Homo sapiens dystrophin (muscular dystrophy, Duchenne and Becker types) (DMD), transcript variant Dp116, mRNA.
	1042350	4004061	core					NM_004007	Homo sapiens dystrophin (muscular dystrophy, Duchenne and Becker types) (DMD), transcript variant Dp427l, mRNA.
	1042351	4004062	full					NM_004021	Homo sapiens dystrophin (muscular dystrophy, Duchenne and Becker types) (DMD), transcript variant Dp140b, mRNA.
	1042352	4004063	extended					AB208836	Homo sapiens mRNA for dystrophin Dp427c isoform variant protein.
	1042353	4004064	full					BC028720	Homo sapiens dystrophin (muscular dystrophy, Duchenne and Becker types), mRNA (cDNA clone IMAGE:4822807), complete
	1042354	4004065	extended					BC036103	cds.
	1042355	4004066	extended					L35854	Homo sapiens dystrophin (muscular dystrophy, Duchenne and Becker types), mRNA (cDNA clone IMAGE:5274415), complete
	1042356	4004067	full					S60972	cds.
	1042357	4004068	core					S62617	Human dystrophin (dp140) mRNA, 5' end.
	1042358	4004069	core					BC010932	dystrophin {out-of-frame deletion of exons 3-7} [human, lymphoblasts, mRNA Mutant, 144 nt].
	1042359	4004070	core					ENSESTT00000016577	dystrophin gene {alternatively spliced} [human, peripheral nerve, mRNA Partial, 106 nt].
	1042360	4004071	extended					ENSESTT00000016578	Homo sapiens dystrophin (muscular dystrophy, Duchenne and Becker types), mRNA (cDNA clone IMAGE:4273908), with
	1042361	4004072	core					ENSESTT00000016579	apparent retained intron.
	1042362	4004073	extended					ENSESTT00000016580	
	1042363	4004074	core					ENSESTT00000016581	
	1042364	4004075	core					ENSESTT00000016582	
	1042365	4004076	core					ENSESTT00000016583	
	1042366	4004077	extended					ENSESTT00000016584	
	1042367	4004078	extended					ENSESTT00000016585	
	1042368	4004079	extended					ENSESTT00000016586	
	1042369	4004080	core					ENSESTT00000016587	
	1042370	4004081	extended					ENSESTT00000034368	
	1042371	4004082	extended					ENSESTT00000034369	
	1042372	4004083	extended					ENSESTT00000034370	
	1042373	4004084	core					ENSESTT00000034371	
	1042374	4004085	core					ENSESTT00000034372	
	1042375	4004086	core					ENST00000357033	
	1042376	4004087	full					ENST00000378723	
	1042377	4004088	extended					ENST00000361593	
	1042378	4004089	extended					ENST00000358062	cdna:known-ccds chromosome:NCBI36:X:31047257:33139632:-1 gene:ENSG00000198947 CCDS14233.1
	1042379	4004090	extended					ENST00000359836	cdna:known-ccds chromosome:NCBI36:X:31047257:31194990:-1 gene:ENSG00000198947 CCDS14231.1
	1042380	4004091	full					ENST00000343523	cdna:known-ccds chromosome:NCBI36:X:31047257:31194895:-1 gene:ENSG00000198947 CCDS14234.1
	1042381	4004092	core					ENST00000361471	cdna:known-ccds chromosome:NCBI36:X:31047266:32083507:-1 gene:ENSG00000198947 CCDS14228.1
	1042382	4004093	full					ENST00000378707	cdna:known-ccds chromosome:NCBI36:X:31047266:32083507:-1 gene:ENSG00000198947 CCDS14229.1
	1042383	4004094	extended					ENST00000378705	cdna:known-ccds chromosome:NCBI36:X:31047266:32083507:-1 gene:ENSG00000198947 CCDS14230.1
	1042384	4004095	extended					ENST00000378702	cdna:known-ccds chromosome:NCBI36:X:31047268:31194989:-1 gene:ENSG00000198947 CCDS14232.1
	1042385	4004096	full					ENST00000361448	cdna:known chromosome:NCBI36:X:31047266:32083507:-1 gene:ENSG00000198947
	1042386	4004097	core					ENST00000378696	cdna:known chromosome:NCBI36:X:31047266:31436336:-1 gene:ENSG00000198947
	1042387	4004098	extended					ENST00000378690	cdna:known chromosome:NCBI36:X:31047266:31194945:-1 gene:ENSG00000198947
	1042388	4004099	extended					ENST00000378687	cdna:known chromosome:NCBI36:X:31047268:31194989:-1 gene:ENSG00000198947
	1042389	4004100	full					ENST00000378684	cdna:known chromosome:NCBI36:X:31049594:31194945:-1 gene:ENSG00000198947
	1042390	4004101	full					ENST00000378682	cdna:known chromosome:NCBI36:X:31049957:33139350:-1 gene:ENSG00000198947
	1042391	4004102	extended					ENST00000378680	cdna:known chromosome:NCBI36:X:31054671:32948245:-1 gene:ENSG00000198947
	1042392	4004103	full					ENST00000288447	cdna:known chromosome:NCBI36:X:31054671:32340247:-1 gene:ENSG00000198947

	1042393	4004104	full					ENST00000378677	cdna:known chromosome:NCBI36:X:31054671:32340095:-1 gene:ENSG00000198947
	1042394	4004105	free					GENSCAN00000066631	cdna:known chromosome:NCBI36:X:31109483:31194973:-1 gene:ENSG00000198947
	1042395	4004106	core					GENSCAN00000068209	cdna:known chromosome:NCBI36:X:32444634:33267479:-1 gene:ENSG00000198947
	1042396	4004107	full					GENSCAN00000037199	cdna:known chromosome:NCBI36:X:32472327:33056468:-1 gene:ENSG00000198947
	1042397	4004108	extended					GENSCAN00000023576	cdna:Genscan chromosome:NCBI36:X:32493740:32573190:-1
	1042398	4004109	full					GENSCAN00000053807	cdna:Genscan chromosome:NCBI36:X:31606287:31657780:-1
	1042399	4004110	full					GENSCAN00000028533	cdna:Genscan chromosome:NCBI36:X:32215563:32215739:-1
	1042400	4004111	full					GENSCAN00000025188	cdna:Genscan chromosome:NCBI36:X:32366273:32474136:-1
	1042401	4004112	full					GENSCAN00000006439	cdna:Genscan chromosome:NCBI36:X:33229047:33247880:-1
	1042402	4004113	core					GENSCAN00000067277	cdna:Genscan chromosome:NCBI36:X:32625908:32777858:-1
	1042403	4004114	extended					GENSCAN00000067278	cdna:Genscan chromosome:NCBI36:X:32254208:32339951:-1
	1042404	4004115	full					GENSCAN00000030020	cdna:Genscan chromosome:NCBI36:X:31049871:31049968:-1
	1042405	4004116	core					GENSCAN00000030024	cdna:Genscan chromosome:NCBI36:X:32915239:32915379:-1
	1042406	4004117	core					GENSCAN00000030021	cdna:Genscan chromosome:NCBI36:X:32973853:32973991:-1
	1042407	4004118	full					GENSCAN00000019292	cdna:Genscan chromosome:NCBI36:X:32083410:32085864:-1
	1042408	4004119	full					GENSCAN00000049410	cdna:Genscan chromosome:NCBI36:X:32015850:32028875:-1
	1042409	4004120	full					GENSCAN00000057883	cdna:Genscan chromosome:NCBI36:X:31927744:31994563:-1
	1042410	4004121	full					GENSCAN00000057884	cdna:Genscan chromosome:NCBI36:X:31060069:31222465:-1
	1042411	4004122	full					GENSCAN00000063408	cdna:Genscan chromosome:NCBI36:X:31435315:31586182:-1
	1042412	4004123	full						cdna:Genscan chromosome:NCBI36:X:31276594:31296032:-1
	1042413	4004124	full						cdna:Genscan chromosome:NCBI36:X:31314901:31424982:-1
	1042414	4004125	full						cdna:Genscan chromosome:NCBI36:X:31734986:31803411:-1
	1042415	4004126	full						
	1042416	4004127	full						
	1042417	4004128	full						
	1042418	4004129	full						
	1042419	4004130	extended						
	1042420	4004131	extended						
	1042421	4004132	extended						
	1042422	4004133	extended						
	1042423	4004134	full						
	1042424	4004135	core						
	1042425	4004136	full						
	1042426	4004137	full						
	1042427	4004138	extended						
	1042428	4004139	extended						
	1042429	4004140	full						
	1042430	4004141	core						
	1042431	4004142	full						
	1042432	4004143	core						
	1042433	4004144	core						
	1042434	4004145	full						
	1042435	4004146	core						
	1042436	4004147	core						
	1042437	4004148	core						
	1042438	4004149	core						
	1042439	4004150	full						
	1042440	4004151	full						
	1042441	4004152	full						
	1042442	4004153	full						
	1042443	4004154	full						
	1042444	4004155	extended						
	1042445	4004156	extended						
	1042446	4004157	full						

	1042447	4004158	extended						
	1042448	4004159	extended						
	1042449	4004160	extended						
	1042450	4004161	extended						
	1042451	4004162	full						
	1042452	4004163	extended						
	1042453	4004164	extended						
	1042454	4004165	full						
	1042455	4004166	extended						
	1042456	4004167	full						
	1042457	4004168	full						
	1042458	4004169	full						
	1042459	4004170	full						
	1042460	4004171	extended						
	1042461	4004172	full						
	1042462	4004173	core						
	1042463	4004174	extended						
	1042464	4004175	full						
	1042465	4004176	core						
	1042466	4004177	full						
	1042467	4004178	full						
	1042468	4004179	full						
	1042469	4004180	full						
	1042470	4004181	core						
	1042471	4004182	full						
	1042472	4004183	full						
	1042473	4004184	full						
	1042474	4004185	extended						
	1042475	4004186	extended						
	1042476	4004187	extended						
	1042477	4004188	full						
	1042478	4004189	full						
	1042479	4004190	extended						
	1042480	4004191	core						
	1042481	4004192	extended						
	1042482	4004193	extended						
	1042483	4004194	full						
	1042484	4004195	full						
	1042485	4004196	full						
	1042486	4004197	core						
	1042487	4004198	core						
	1042488	4004199	core						
	1042489	4004200	full						
	1042490	4004201	full						
	1042491	4004202	full						
	1042492	4004203	full						
	1042493	4004204	extended						
	1042494	4004205	full						
	1042495	4004206	full						
	1042496	4004207	full						
	1042497	4004208	full						
	1042498	4004209	full						
	1042499	4004210	core						
	1042500	4004211	full						

	1042501	4004212	core						
	1042502	4004213	full						
	1042503	4004214	extended						
	1042504	4004215	extended						
	1042505	4004216	extended						
	1042506	4004217	core						
	1042507	4004218	full						
	1042508	4004219	full						
	1042509	4004220	full						
	1042510	4004221	full						
	1042511	4004222	full						
	1042512	4004223	full						
	1042513	4004224	core						
	1042514	4004225	core						
	1042515	4004226	full						
	1042516	4004227	extended						
	1042517	4004228	core						
	1042518	4004229	full						
	1042519	4004230	full						
	1042520	4004231	full						
	1042521	4004232	full						
	1042522	4004233	full						
	1042523	4004234	full						
	1042524	4004235	full						
	1042525	4004236	full						
	1042526	4004237	full						
	1042527	4004238	full						
	1042528	4004239	extended						
	1042529	4004240	full						
	1042530	4004241	full						
	1042531	4004242	full						
	1042532	4004243	full						
	1042533	4004244	full						
	1042534	4004245	full						
	1042535	4004246	full						
	1042536	4004247	full						
	1042537	4004248	full						
	1042538	4004249	full						
	1042539	4004250	extended						
	1042540	4004251	extended						
	1042541	4004252	full						
	1042542	4004253	full						
	1042543	4004254	full						
	1042544	4004255	full						
	1042545	4004256	extended						
	1042546	4004257	extended						
	1042547	4004258	extended						
	1042548	4004259	extended						
	1042549	4004260	full						
	1042550	4004261	full						
	1042551	4004262	core						
	1042552	4004263	extended						
	1042553	4004264	full						
	1042554	4004265	full						

	1042555	4004266	full						
	1042556	4004267	core						
	1042557	4004268	full						
	1042558	4004269	full						
	1042559	4004270	full						
	1042560	4004271	full						
	1042561	4004272	core						
	1042562	4004273	full						
	1042563	4004274	extended						
	1042564	4004275	extended						
	1042565	4004276	extended						
	1042566	4004277	full						
	1042567	4004278	extended						
	1042568	4004279	extended						
	1042569	4004280	extended						
	1042570	4004281	extended						
	1042571	4004282	extended						
	1042572	4004283	core						
	1042573	4004284	extended						
	1042574	4004285	extended						
	1042575	4004286	core						
	1042576	4004287	core						
	1042577	4004288	full						
	1042578	4004289	core						
	1042579	4004290	extended						
	1042580	4004291	extended						
	1042581	4004292	full						
	1042582	4004293	full						
	1042583	4004294	core						
	1042584	4004295	core						
	1042585	4004296	core						
	1042586	4004297	full						
	1042587	4004298	full						
	1042588	4004299	core						
	1042589	4004300	core						
	1042590	4004301	core						
	1042591	4004302	core						
	1042592	4004303	core						
	1042593	4004304	full						
	1042594	4004305	full						
	1042595	4004306	core						
	1042596	4004307	core						
	1042597	4004308	core						
	1042598	4004309	core						
	1042599	4004310	core						
	1042600	4004311	core						
	1042601	4004312	full						
	1042602	4004313	full						
	1042603	4004314	extended						
	1042604	4004315	full						
	1042605	4004316	core						
	1042606	4004317	full						
	1042607	4004318	core						
	1042608	4004319	core						

	1042609	4004320	core						
	1042610	4004321	core						
	1042611	4004322	core						
	1042612	4004323	core						
	1042613	4004324	core						
	1042614	4004325	core						
	1042615	4004326	full						
	1042616	4004327	core						
	1042617	4004328	full						
	1042618	4004329	core						
	1042619	4004330	core						
	1042620	4004331	extended						
	1042621	4004332	core						
	1042622	4004333	full						
	1042623	4004334	extended						
	1042624	4004335	extended						
	1042625	4004336	core						
	1042626	4004337	full						
	1042627	4004338	full						
	1042628	4004339	core						
	1042629	4004340	core						
	1042630	4004341	core						
	1042631	4004342	core						
	1042632	4004343	core						
	1042633	4004344	core						
	1042634	4004345	full						
	1042635	4004346	extended						
	1042636	4004347	core						
	1042637	4004348	core						
	1042638	4004349	core						
	1042639	4004350	full						
	1042640	4004351	full						
	1042641	4004352	full						
	1042642	4004353	core						
	1042643	4004354	full						
	1042644	4004355	core						
	1042645	4004356	core						
		4004357	full						
		4004358	full						
		4004359	extended						
		4004360	extended						
		4004361	full						
		4004362	full						
		4004363	full						
		4004364	extended						
		4004365	extended						
		4004366	full						
		4004367	full						
		4004368	extended						
		4004369	core						
		4004370	core						
		4004371	core						
		4004372	core						
		4004373	core						

		4004374<br>4004375<br>4004376<br>4004377<br>4004378<br>4004379<br>4004380<br>4004381<br>4004382<br>4004383<br>4004384<br>4004385<br>4004386<br>4004387<br>4004388<br>4004389<br>4004390<br>4004391<br>4004392<br>4004393<br>4004394<br>4004395<br>4004396<br>4004397<br>4004398<br>4004399<br>4004400<br>4004401<br>4004402<br>4004403<br>4004404<br>4004405<br>4004406<br>4004407<br>4004408<br>4004409<br>4004410<br>4004411<br>4004412<br>4004413<br>4004414	full<br>core<br>full<br>extended<br>extended<br>extended<br>extended<br>extended<br>full<br>full<br>extended<br>full<br>extended<br>full<br>extended<br>core<br>extended<br>full<br>full<br>extended<br>core<br>core<br>extended<br>extended<br>extended<br>core<br>full<br>full<br>full<br>extended<br>full<br>extended<br>full<br>full<br>extended<br>extended<br>full<br>full<br>core<br>core<br>core						
4018080	1050696<br>1050697<br>1050698<br>1050699<br>1050700<br>1050701<br>1050702<br>1050703<br>1050704<br>1050705<br>1050706<br>1050707<br>1050708	4018081<br>4018082<br>4018083<br>4018084<br>4018085<br>4018086<br>4018087<br>4018088<br>4018089<br>4018090<br>4018091<br>4018092<br>4018093	full<br>full<br>full<br>full<br>full<br>extended<br>core<br>core<br>full<br>core<br>full<br>core<br>core	X	109673850	109993562	-	NM_145234<br>AK092245<br>CR604833<br>ENSESTT00000024315<br>ENSESTT00000024316<br>ENSESTT00000024317<br>ENSESTT00000024318<br>ENSESTT00000024319<br>ENSESTT00000024320<br>ENST00000218054<br>ENST00000372045<br>ENST00000372042<br>GENSCAN00000004555	Homo sapiens chordin-like 1 (CHRDL1), mRNA.<br>Homo sapiens cDNA FLJ34926 fis, clone NT2RP7003319, highly similar to Mus musculus neuralin mRNA.<br>full-length cDNA clone CS0DI073YE23 of Placenta Cot 25-normalized of Homo sapiens (human).<br><br><br><br><br><br><br>cdna:known-ccds chromosome:NCBI36:X:109805133:109925649:-1 gene:ENSG00000101938 CCDS14553.1<br>cdna:known chromosome:NCBI36:X:109803740:109925649:-1 gene:ENSG00000101938<br>cdna:known chromosome:NCBI36:X:109805660:109925695:-1 gene:ENSG00000101938<br>cdna:Genscan chromosome:NCBI36:X:109806115:109892691:-1

	1050709<br>1050710<br>1050711<br>1050712<br>1050713<br>1050714<br>1050715<br>1050716<br>1050717<br>1050718<br>1050719<br>1050720<br>1050721<br>1050722<br>1050723<br>1050724<br>1050725<br>1050726<br>1050727<br>1050728<br>1050729<br>1050730<br>1050731<br>1050732<br>1050733<br>1050734<br>1050735	4018094<br>4018095<br>4018096<br>4018097<br>4018098<br>4018099<br>4018100<br>4018101<br>4018102<br>4018103<br>4018104<br>4018105<br>4018106<br>4018107<br>4018108<br>4018109<br>4018110<br>4018111<br>4018112<br>4018113<br>4018114<br>4018115<br>4018116<br>4018117<br>4018118<br>4018119<br>4018120<br>4018121<br>4018122<br>4018123<br>4018124<br>4018125<br>4018126<br>4018127	full<br>core<br>core<br>core<br>full<br>full<br>full<br>full<br>full<br>full<br>full<br>full<br>full<br>full<br>core<br>full<br>full<br>core<br>full<br>full<br>full<br>core<br>core<br>full<br>full<br>core<br>full<br>full<br>full<br>full<br>full<br>full<br>full<br>full					GENSCAN00000007941<br>GENSCAN00000044078	cdna:Genscan chromosome:NCBI36:X:109673850:109717564:-1<br>cdna:Genscan chromosome:NCBI36:X:109921498:109945203:-1
4021341	1052697<br>1052698<br>1052699<br>1052700<br>1052701<br>1052702<br>1052703<br>1052704<br>1052705<br>1052706<br>1052707<br>1052708<br>1052709<br>1052710<br>1052711<br>1052712<br>1052713<br>1052714<br>1052715	4021342<br>4021343<br>4021344<br>4021345<br>4021346<br>4021347<br>4021348<br>4021349<br>4021350<br>4021351<br>4021352<br>4021353<br>4021354<br>4021355<br>4021356<br>4021357<br>4021358<br>4021359<br>4021360	extended<br>full<br>extended<br>extended<br>core<br>core<br>core<br>core<br>core<br>core<br>core<br>extended<br>extended<br>core<br>core<br>extended<br>extended<br>extended<br>extended	X	128759109	128805645	-	NM_016032<br>NM_001008222<br>AF151847<br>CR596648<br>ENSESTT00000022312<br>ENST00000371071<br>ENST00000357166<br>ENST00000341406<br>ENST00000371064<br>GENSCAN00000005674	Homo sapiens zinc finger, DHHC-type containing 9 (ZDHHC9), transcript variant 1, mRNA.<br>Homo sapiens zinc finger, DHHC-type containing 9 (ZDHHC9), transcript variant 2, mRNA.<br>Homo sapiens CGI-89 protein mRNA, complete cds.<br>full-length cDNA clone CS0DI071YF17 of Placenta Cot 25-normalized of Homo sapiens (human).<br><br>cdna:known chromosome:NCBI36:X:128764945:128805541:-1 gene:ENSG00000188706<br>cdna:known chromosome:NCBI36:X:128766596:128805554:-1 gene:ENSG00000188706<br>cdna:known chromosome:NCBI36:X:128766596:128805554:-1 gene:ENSG00000188706<br>cdna:known chromosome:NCBI36:X:128766986:128805117:-1 gene:ENSG00000188706<br>cdna:Genscan chromosome:NCBI36:X:128768027:128790798:-1

	1052716	4021361	extended						
	1052717	4021362	extended						
	1052718	4021363	extended						
	1052719	4021364	core						
	1052720	4021365	core						
	1052721	4021366	core						
	1052722	4021367	full						
	1052723	4021368	full						
	1052724	4021369	extended						
		4021370	core						
		4021371	core						
		4021372	core						
		4021373	core						
		4021374	extended						
		4021375	extended						
		4021376	extended						
		4021377	extended						
		4021378	extended						
		4021379	extended						
		4021380	extended						
		4021381	extended						
		4021382	core						
		4021383	extended						
		4021384	core						
		4021385	core						
		4021386	extended						
		4021387	full						
