# Supplementary material for: Comparison of Two Methods for Detecting Alternative Splice Variants Using GeneChip® Exon Arrays
Source: Int J Biomed Sci. 2011 Sep;7(3):172–80. (PMC3614835)
Supplement: Supplementary file 4 [file IJBS-7-172_SD8.pdf]

Table OL2. Affymetrix Human Exon 1.0 ST Transcription ID for alternative spice variants detected by MIDAS

2425756	2425756	2515933	3973505	3573870	2692319	2949622	2425756	2425756	3569814	2949622	2605321	3388830	2907671
3422144	3047581	2425756	3394660	3515965	2676009	2584134	3982242	3758510	3157385	3653677	4004044	2398706	2425756
2710599	2425756	2652410	3651509	3939470	3828278	3581637	2425756	3110317	3020343	3428845	3607537	2535830	3904566
3998766	2479433	3504617	3910785	3189422	2425756	3581637	2494484	2949622	3643580	3825609	2891556	3662808	3708399
2891556	3446137	3704376	2425756	2326237	2652675	3581637	3907111	3396084	3305198	3581442	2377094	3733590	2425756
3220180	2693563	2531589	3730601	3859761	4019465	2570616	3358361	2652675	3939470	3304301	3490655	2931391	3577443
3705151	2746591	3023483	3095057	2730746	2425756	2671101	3150844	3988165	3265224	3887049	2425756	3674199	3733590
3855818	3330897	3047581	2425756	2891556	2999303	3604147	3290649	2419046	2425756	3041875	3910785	3758845	3020343
2425756	2949622	3009399	3643752	3873629	3756193	2842624	3589697	2434609	2758076	3846390	2949622	2993029	2949622
3674199	3047581	3204243	2961177	3826041	2574984	3250055	2746591	2949622	2425756	3168508	2570193	2692319	2570616
2740067	3871192	3237396	3089360	2961177	2735027	3985717	3716113	2376168	2353337	2866225	3470597	2779199	3632806
3901387	3432030	3618736	2560076	2425756	3510362	2497301	2635184	3816645	3047581	3358201	3343832	4027176	2584134
3238962	3427282	3292946	2786322	3157385	3694657	2376168	3907111	3011911	2853642	2335922	3930360	3057650	3332626
3878836	3151534	2692319	3025545	3156848	3901387	3595979	2889916	3230760	3252036	3924573	3296046	3032243	3454892
2376168	3555340	2727226	3793760	2377427	2949622	3697090	2532480	3069366	3952825	3630736	3049522	2489140	2949622
3795866	3815399	3063727	2625793	3923257	2923868	3173974	3934245	3381150	3349293	2949622	3654614	3646164	2949622
2854445	2371139	4007865	3457101	3457667	2404999	3388673	2411228	2369325	3632806	3891278	3832760	3581637	3213219
3335907	2574984	2611848	3735478	2411228	3910785	3820501	2929168	3980560	2924514	2459042	3644541	3416290	2728938
3881282	2438892	3881443	3738842	2625793	3127775	2595443	3712675	2402942	2652675	3590086	2949622	3881786	3771259
3441849	3046444	3821263	3110317	3105581	2712236	2371139	3296046	3515965	3236538	2453370	2924514	3320301	2330133
3790982	3105600	3970642	2450345	3839346	3728776	3556990	4009667	3933550	3707258	2574984	3719150	3090697	3886179
2897899	2991395	3605395	3841076	3653677	3952825	3742285	3768627	3859761	2350596	3175971	3950872	2710474	2602653
3331487	3399004	3591459	2886679	3110317	3174121	3110317	2566848	3789442	3125571	2549565	3931112	4004044	2346575
2976041	3258444	3962997	2582979	3158478	3882012	3265565	2604254	2853768	3418249	3728964	3913960	3893520	3025433
2686458	2559637	3436236	3901085	2786322	3505937	2443120	3102372	2413203	2584134	3773244	2985781	3175971	3442641
3388914	3235789	2425756	3685329	3783788	2604998	2328868	3771160	2924514	2690956	3222170	2669488	4026624	3079803
3909777	3733590	2446567	3726375	3984655	2425756	3020343	3843690	3887049	2502842	2949622	3781429	3853108	3638337
3597338	3874438	2946106	2451593	3965833	3790259	2956904	3959388	2801694	3996667	3703885	2693563	3556990	3838425
2734047	3569200	3642765	3201319	3056320	3883064	2746693	3724197	4026956	2331213	2740067	3765167	3839346	3923218
3108526	2585400	2376168	3643580	3673684	2424102	2999334	2566848	3510066	4004853	3728889	3229741	2409104	3610982
2570616	3442427	3751859	3837796	2730746	3645779	2961177	3601051	3147286	3676127	2730746	3581637	3607332	3456081
3121751	2730746	2977949	2955691	3750662	2746591	3630736	2958232	2960146	2820925	2810395	3883309	2992814	3939183
3486883	3710823	3457101	4005644	3604147	3851703	2925237	3547375	3326252	3026599	3557851	3751859	2678298	2500165
