# Supplementary material for: Comparison of Two Methods for Detecting Alternative Splice Variants Using GeneChip® Exon Arrays
Source: Int J Biomed Sci. 2011 Sep;7(3):172–80. (PMC3614835)
Supplement: Supplementary file 5 [file IJBS-7-172_SD3.pdf]

**Additional file 3.** Statistics for alternative splice variants selected by MIDAS		
**ProbeSet ID (For each Exon)**	**P-values for Differentially expressed between normal and tumor (sorted)**	**Transcript ID**
2425810	0.010415	2425756
2425819	0.012973	2425756
2516011	0.015146	2515933
3973512	0.016221	3973505
3573884	0.016586	3573870
2692358	0.016989	2692319
2949695	0.017701	2949622
2425850	0.018236	2425756
2425825	0.02013	2425756
3569830	0.02106	3569814
2949723	0.02178	2949622
2605390	0.022336	2605321
3388842	0.022682	3388830
2907687	0.023041	2907671
3422189	0.023405	3422144
3047584	0.023835	3047581
2425849	0.024679	2425756
3394663	0.024756	3394660
3516014	0.024806	3515965
2676011	0.024905	2676009
2584166	0.025093	2584134
3982251	0.025152	3982242
3758526	0.025442	3758510
3157389	0.025446	3157385
3653680	0.025626	3653677
4004323	0.02599	4004044
2398720	0.02607	2398706
2425821	0.026448	2425756
2710603	0.026784	2710599
2425793	0.027389	2425756
2652528	0.027449	2652410
3651532	0.027492	3651509
3939477	0.027583	3939470
3828304	0.027996	3828278
3581841	0.028182	3581637
2425778	0.028274	2425756
3110331	0.028381	3110317
3020410	0.028855	3020343
3428850	0.028875	3428845
3607589	0.029136	3607537

2535846	0.029167	2535830
3904586	0.029229	3904566
3998804	0.029233	3998766
2479493	0.029345	2479433
3504635	0.029497	3504617
3910796	0.029588	3910785
3189493	0.02959	3189422
2425766	0.029814	2425756
3581693	0.029834	3581637
2494507	0.029857	2494484
2949734	0.029885	2949622
3643592	0.02992	3643580
3825620	0.029942	3825609
2891616	0.030021	2891556
3662813	0.030275	3662808
3708405	0.03033	3708399
2891610	0.030534	2891556
3446145	0.030584	3446137
3704449	0.030824	3704376
2425785	0.031061	2425756
2326249	0.031344	2326237
2652679	0.031372	2652675
3581862	0.031386	3581637
3907112	0.031445	3907111
3396092	0.031518	3396084
3305230	0.031543	3305198
3581443	0.031905	3581442
2377111	0.032122	2377094
3733601	0.032231	3733590
2425802	0.032446	2425756
3220182	0.032447	3220180
2693564	0.032639	2693563
2531590	0.032945	2531589
3730618	0.033021	3730601
3859783	0.033038	3859761
4019469	0.033241	4019465
2570630	0.033251	2570616
3358383	0.033333	3358361
2652721	0.03345	2652675
3939491	0.033468	3939470
3304306	0.033497	3304301
3490674	0.033651	3490655
2931416	0.033689	2931391

3577465	0.033693	3577443
3705153	0.033861	3705151
2746628	0.03394	2746591
3023526	0.034027	3023483
3095087	0.034039	3095057
2730780	0.03409	2730746
2425807	0.034306	2425756
2671165	0.034342	2671101
3150949	0.034361	3150844
3988181	0.034434	3988165
3265254	0.034443	3265224
3887066	0.034446	3887049
2425801	0.034474	2425756
3674216	0.034515	3674199
3733603	0.034584	3733590
3855837	0.034628	3855818
3330900	0.034728	3330897
3047597	0.034795	3047581
2425826	0.034813	2425756
2891617	0.034827	2891556
2999313	0.034968	2999303
3604218	0.035146	3604147
3290654	0.035184	3290649
2419049	0.035196	2419046
2425786	0.035303	2425756
3041902	0.035305	3041875
3910807	0.035378	3910785
3758864	0.035539	3758845
3020408	0.035553	3020343
2425805	0.035613	2425756
2949629	0.035617	2949622
3009402	0.035635	3009399
3643783	0.035672	3643752
3873670	0.035689	3873629
3756205	0.035956	3756193
2842662	0.036437	2842624
3589720	0.036507	3589697
2434627	0.036573	2434609
2758105	0.036575	2758076
3846394	0.036608	3846390
2949627	0.036622	2949622
2993053	0.036658	2993029
2949752	0.036701	2949622

3674229	0.036723	3674199
3047583	0.036725	3047581
3204253	0.036745	3204243
2961268	0.036792	2961177
3826071	0.036808	3826041
2575021	0.036857	2574984
3250065	0.036888	3250055
2746632	0.036896	2746591
2949636	0.036922	2949622
2425775	0.03694	2425756
3168530	0.037197	3168508
2570194	0.037246	2570193
2692329	0.037258	2692319
2570677	0.03739	2570616
2740248	0.03742	2740067
3871205	0.037481	3871192
3237511	0.037483	3237396
3089375	0.037666	3089360
2961190	0.037707	2961177
2735071	0.037762	2735027
3985763	0.03796	3985717
3716126	0.038054	3716113
2376214	0.038065	2376168
2353352	0.038154	2353337
2866232	0.038163	2866225
3470604	0.038168	3470597
2779200	0.038191	2779199
3632816	0.038226	3632806
3901398	0.038259	3901387
3432061	0.0384	3432030
3618775	0.038427	3618736
2560100	0.038462	2560076
2425790	0.038486	2425756
3510363	0.03859	3510362
2497305	0.038605	2497301
2635193	0.038606	2635184
3816655	0.038607	3816645
3047582	0.038616	3047581
3358234	0.038798	3358201
3343835	0.038886	3343832
4027241	0.038907	4027176
2584139	0.038929	2584134
3239178	0.039007	3238962

3427285	0.039086	3427282
3292953	0.039254	3292946
2786337	0.03933	2786322
3157401	0.039375	3157385
3694667	0.039533	3694657
2376193	0.03954	2376168
3907126	0.039651	3907111
3011936	0.039658	3011911
2853654	0.039738	2853642
2335929	0.039748	2335922
3930433	0.039845	3930360
3057662	0.039993	3057650
3332658	0.040025	3332626
3878856	0.040043	3878836
3151559	0.040288	3151534
2692359	0.040314	2692319
3025632	0.040388	3025545
3156918	0.040393	3156848
3901399	0.040434	3901387
3595997	0.040449	3595979
2889917	0.040717	2889916
3230761	0.040775	3230760
3252054	0.040837	3252036
3924659	0.040898	3924573
3296279	0.040905	3296046
3032259	0.041012	3032243
3454924	0.04106	3454892
2376269	0.041074	2376168
3555425	0.041127	3555340
2727236	0.041128	2727226
3793781	0.041162	3793760
2377456	0.041163	2377427
2949688	0.041245	2949622
3697103	0.041398	3697090
2532483	0.041426	2532480
3069370	0.041429	3069366
3952863	0.041436	3952825
3630758	0.041443	3630736
3049538	0.041452	3049522
2489144	0.041461	2489140
2949643	0.041491	2949622
3795922	0.041515	3795866
3815413	0.041713	3815399

3063757	0.041744	3063727
2625821	0.041786	2625793
3923258	0.041796	3923257
2923869	0.041885	2923868
3173982	0.041889	3173974
3934252	0.041904	3934245
3381183	0.041908	3381150
3349368	0.041982	3349293
2949758	0.042027	2949622
3654628	0.042137	3654614
3646194	0.042165	3646164
2949684	0.04223	2949622
2854488	0.042388	2854445
2371189	0.042484	2371139
4007881	0.042487	4007865
3457139	0.042496	3457101
3457678	0.042512	3457667
2405001	0.042533	2404999
3388674	0.04254	3388673
2411240	0.042558	2411228
2369338	0.04258	2369325
3632839	0.042614	3632806
3891321	0.04264	3891278
3832767	0.042744	3832760
3581869	0.042747	3581637
3213228	0.042783	3213219
3335920	0.04281	3335907
2575036	0.042864	2574984
2611945	0.042956	2611848
3735489	0.042979	3735478
2411267	0.042996	2411228
3910808	0.043005	3910785
3820546	0.04303	3820501
2929345	0.043032	2929168
3980607	0.043083	3980560
2924557	0.043121	2924514
2459095	0.043129	2459042
3644590	0.043171	3644541
3416293	0.043182	3416290
2729063	0.043217	2728938
3881289	0.043238	3881282
2438918	0.043252	2438892
3881464	0.043306	3881443

3738889	0.043356	3738842
2625820	0.043409	2625793
3127797	0.04342	3127775
2595450	0.043444	2595443
3712740	0.04351	3712675
2402945	0.043535	2402942
2652714	0.043536	2652675
3590094	0.043645	3590086
2949696	0.043717	2949622
3881787	0.043756	3881786
3771265	0.043779	3771259
3441868	0.043799	3441849
3046447	0.043804	3046444
3821280	0.043816	3821263
3110320	0.043831	3110317
3105591	0.043866	3105581
2712385	0.043876	2712236
2371179	0.0439	2371139
3296059	0.043952	3296046
3516031	0.043962	3515965
3236548	0.044105	3236538
2453461	0.044122	2453370
2924567	0.044149	2924514
3320347	0.044242	3320301
2330176	0.044242	2330133
3791024	0.044358	3790982
3105629	0.044414	3105600
3970711	0.04442	3970642
2450408	0.044465	2450345
3839355	0.044566	3839346
3728778	0.04458	3728776
3557013	0.044674	3556990
4009714	0.044698	4009667
3933551	0.044706	3933550
3707281	0.044708	3707258
2575027	0.044714	2574984
3719158	0.044756	3719150
3090726	0.044822	3090697
3886210	0.044853	3886179
2897904	0.044865	2897899
2991564	0.044865	2991395
3605495	0.044899	3605395
3841091	0.045006	3841076

3653691	0.045031	3653677
3952847	0.045055	3952825
3742296	0.045074	3742285
3768666	0.045108	3768627
3859781	0.045116	3859761
2350615	0.045156	2350596
3175980	0.045156	3175971
3950876	0.045181	3950872
2710544	0.045198	2710474
2602669	0.045242	2602653
3331572	0.045412	3331487
3399071	0.045433	3399004
3591477	0.045445	3591459
2886683	0.04547	2886679
3110328	0.045539	3110317
3174122	0.045568	3174121
3110336	0.045571	3110317
2566934	0.045574	2566848
3789456	0.045588	3789442
3125616	0.045593	3125571
2549569	0.045638	2549565
3931206	0.045671	3931112
4004319	0.045695	4004044
2346615	0.045703	2346575
2976045	0.045734	2976041
3258459	0.045818	3258444
3963026	0.04583	3962997
2583008	0.045848	2582979
3158502	0.045886	3158478
3882057	0.045893	3882012
3265582	0.045905	3265565
2604261	0.045909	2604254
2853812	0.045914	2853768
3418272	0.045929	3418249
3728991	0.045985	3728964
3913983	0.045997	3913960
3893541	0.046053	3893520
3025456	0.046093	3025433
2686559	0.046142	2686458
2559646	0.046169	2559637
3436240	0.046186	3436236
3901094	0.046195	3901085
2786361	0.046212	2786322

3505958	0.046303	3505937
2443143	0.046304	2443120
3102465	0.046352	3102372
2413218	0.046364	2413203
2584187	0.046376	2584134
3773253	0.046386	3773244
2985828	0.046395	2985781
3175990	0.046416	3175971
3442673	0.04643	3442641
3388938	0.046444	3388914
3235798	0.046446	3235789
2425783	0.046467	2425756
3685350	0.04648	3685329
3783804	0.046586	3783788
2605035	0.046597	2604998
2328891	0.046619	2328868
3771188	0.046665	3771160
2924555	0.046685	2924514
2690978	0.046725	2690956
3222198	0.046767	3222170
2669508	0.046863	2669488
4026633	0.046874	4026624
3079969	0.046883	3079803
3909778	0.046918	3909777
3733605	0.046949	3733590
2446620	0.046973	2446567
3726377	0.047118	3726375
3984661	0.047202	3984655
2425796	0.047249	2425756
3020409	0.047268	3020343
3843716	0.047274	3843690
3887052	0.047322	3887049
2502853	0.047331	2502842
2949754	0.047335	2949622
3781483	0.047384	3781429
3853151	0.047394	3853108
3638338	0.047424	3638337
3597384	0.047522	3597338
3874442	0.04756	3874438
2946117	0.047604	2946106
2451615	0.047618	2451593
3965891	0.047673	3965833
3790286	0.047776	3790259

2957058	0.047797	2956904
3959404	0.047801	3959388
2801699	0.047804	2801694
3996693	0.047845	3996667
3703931	0.047848	3703885
2693566	0.047849	2693563
3557012	0.04785	3556990
3838431	0.047872	3838425
2734077	0.047875	2734047
3569228	0.047904	3569200
3642784	0.04791	3642765
3201324	0.047953	3201319
3056323	0.047985	3056320
3883068	0.047993	3883064
2746804	0.048076	2746693
3724236	0.048086	3724197
4026975	0.048104	4026956
2331354	0.048125	2331213
2740243	0.048147	2740067
3765182	0.048148	3765167
3839352	0.04815	3839346
3923244	0.048185	3923218
3108614	0.048208	3108526
2585429	0.048243	2585400
2376237	0.048243	2376168
3643594	0.048297	3643580
3673695	0.048305	3673684
2424108	0.048336	2424102
2999401	0.048338	2999334
2566938	0.048365	2566848
3510089	0.048384	3510066
4004875	0.048408	4004853
3728909	0.04846	3728889
3229770	0.048548	3229741
2409153	0.048584	2409104
3610996	0.048623	3610982
2570676	0.048687	2570616
3442436	0.048687	3442427
3751864	0.048706	3751859
3837815	0.04873	3837796
2730794	0.048735	2730746
3645791	0.048739	3645779
2961188	0.048765	2961177

3601095	0.048794	3601051
3147294	0.048806	3147286
3676133	0.048894	3676127
2730769	0.048907	2730746
3581858	0.048999	3581637
3607351	0.049025	3607332
3456094	0.049044	3456081
3121865	0.049091	3121751
2730781	0.049171	2730746
2978002	0.049183	2977949
2955707	0.049203	2955691
3750673	0.04927	3750662
2746627	0.049319	2746591
3630757	0.049329	3630736
2958257	0.049539	2958232
2960175	0.049585	2960146
2820948	0.049603	2820925
2810409	0.04961	2810395
3883326	0.049619	3883309
2992815	0.04963	2992814
3939212	0.049646	3939183
3486907	0.049712	3486883
3710825	0.049733	3710823
3457141	0.049743	3457101
4005674	0.049758	4005644
3604184	0.049763	3604147
3851708	0.049781	3851703
2925308	0.049784	2925237
3547382	0.049792	3547375
3326260	0.049866	3326252
3026644	0.049877	3026599
3557859	0.049899	3557851
3751869	0.049906	3751859
2678303	0.049914	2678298
2500243	0.049966	2500165
