# Supplementary material for: Comparison of Two Methods for Detecting Alternative Splice Variants Using GeneChip® Exon Arrays
Source: Int J Biomed Sci. 2011 Sep;7(3):172–80. (PMC3614835)
Supplement: Supplementary file 6 [file IJBS-7-172_SD4.pdf]

**Additional file 4.** Annotation for alternative splice variants selected by MIDAS

| Probe Set ID | Exon Cluster ID | Transcript Cluster ID | Probe Set Grade | Current Genome Probe Set Start | Current Genome Probe Set Stop | Current Genome Chromosome | Current Genome Strand | Transcript ID (Annotated) | Representative Transcript Description |
|---|---|---|---|---|---|---|---|---|---|
| 2326249 | 6874 | 2326237 | core | 26222425 | 26222503 | 1 | + | NM_004455<br>ENST00000374280<br>ENST00000288898<br>GENSCAN00000013067<br>ENSESTT00000058403<br>ENSESTT00000058404 | Homo sapiens exostoses (multiple)-like 1 (EXTL1), mRNA.<br>cdna:known-ccds chromosome:NCBI36:1:26220858:26235542:1 gene:ENSG00000158008 CCDS271.1<br>cdna:known chromosome:NCBI36:1:26220863:26235519:1 gene:ENSG00000158008<br>cdna:Genscan chromosome:NCBI36:1:26216890:26234425:1 |
| 2328891 | 8422 | 2328868 | core | 32569665 | 32569689 | 1 | + | ENST00000373548<br>ENST00000373541<br>AK225555<br>GENSCAN00000018653<br>ENST00000271095<br>NM_004964<br>U50079<br>ENSESTT00000029617<br>BX648055 | cdna:known-ccds chromosome:NCBI36:1:32530274:32571823:1 gene:ENSG00000116478 CCDS360.1<br>cdna:known chromosome:NCBI36:1:32530295:32571811:1 gene:ENSG00000116478<br>Homo sapiens mRNA for histone deacetylase 1 variant, clone: KDN05801.<br>cdna:Genscan chromosome:NCBI36:1:32530358:32571232:1<br>cdna:known chromosome:NCBI36:1:32530295:32571811:1 gene:ENSG00000116478<br>Homo sapiens histone deacetylase 1 (HDAC1), mRNA.<br>Human histone deacetylase HD1 mRNA, complete cds.<br><br>Homo sapiens mRNA; cDNA DKFZp686H12203 (from clone DKFZp686H12203). |
| 2330176 | 9212 | 2330133 | core | 36247683 | 36247764 | 1 | + | BC025769<br>ENST00000246314<br>ENSESTT00000058020<br>ENST00000324335<br>ENST00000373185<br>NM_177422<br>NM_024852<br>AK027796<br>ENST00000324350<br>GENSCAN00000028954<br>ENST00000373191<br>ENSESTT00000058022<br>GENSCAN00000006289 | Homo sapiens eukaryotic translation initiation factor 2C, 3, mRNA (cDNA clone IMAGE:5210832).<br>cdna:known chromosome:NCBI36:1:36169446:36294650:1 gene:ENSG00000126070<br><br>cdna:known chromosome:NCBI36:1:36169359:36294649:1 gene:ENSG00000126070<br>cdna:known-ccds chromosome:NCBI36:1:36169359:36294649:1 gene:ENSG00000126070 CCDS400.1<br>Homo sapiens eukaryotic translation initiation factor 2C, 3 (EIF2C3), transcript variant 2, mRNA.<br>Homo sapiens eukaryotic translation initiation factor 2C, 3 (EIF2C3), transcript variant 1, mRNA.<br>Homo sapiens cDNA FLJ14890 fis, clone PLACE1004149, weakly similar to Rattus norvegicus GERp95 mRNA.<br>cdna:known chromosome:NCBI36:1:36168906:36221597:1 gene:ENSG00000126070<br>cdna:Genscan chromosome:NCBI36:1:36169133:36210420:1<br>cdna:known-ccds chromosome:NCBI36:1:36169266:36294650:1 gene:ENSG00000126070 CCDS399.1<br><br>cdna:Genscan chromosome:NCBI36:1:36242529:36308908:1 |
| 2331354 | 9914 | 2331213 | core | 39569734 | 39570118 | 1 | + | ENST00000361888<br>GENSCAN00000052288<br>ENST00000372915<br>ENST00000317713<br>ENST00000356138<br>ENST00000335924<br>ENST00000372925<br>NM_012090<br>ENSESTT00000008297<br>ENST00000361689<br>AF141968<br>ENST00000372900<br>ENST00000289894<br>AB033077<br>NM_033044<br>ENST00000289893<br>ENST00000360115<br>ENSESTT00000008309<br>GENSCAN00000002540<br>ENSESTT00000008314<br>ENSESTT00000008313 | cdna:known chromosome:NCBI36:1:39569397:39725246:1 gene:ENSG00000127603<br>cdna:Genscan chromosome:NCBI36:1:39468631:39585469:1<br>cdna:known chromosome:NCBI36:1:39322426:39725376:1 gene:ENSG00000127603<br>cdna:known chromosome:NCBI36:1:39466940:39725376:1 gene:ENSG00000127603<br>cdna:known chromosome:NCBI36:1:39322426:39725376:1 gene:ENSG00000127603<br>cdna:known chromosome:NCBI36:1:39322426:39725376:1 gene:ENSG00000127603<br>cdna:known chromosome:NCBI36:1:39322426:39725376:1 gene:ENSG00000127603<br>Homo sapiens microtubule-actin crosslinking factor 1 (MACF1), transcript variant 1, mRNA.<br><br>cdna:known-ccds chromosome:NCBI36:1:39322426:39725376:1 gene:ENSG00000127603 CCDS435.1<br>Homo sapiens trabeculin-alpha mRNA, complete cds.<br>cdna:known chromosome:NCBI36:1:39322426:39725376:1 gene:ENSG00000127603<br>cdna:known chromosome:NCBI36:1:39322426:39725246:1 gene:ENSG00000127603<br>Homo sapiens mRNA for KIAA1251 protein, partial cds.<br>Homo sapiens microtubule-actin crosslinking factor 1 (MACF1), transcript variant 2, mRNA.<br>cdna:known chromosome:NCBI36:1:39569397:39725376:1 gene:ENSG00000127603<br>cdna:known-ccds chromosome:NCBI36:1:39569397:39725376:1 gene:ENSG00000127603 CCDS436.1<br><br>cdna:Genscan chromosome:NCBI36:1:39648525:39697445:1 |

| | | | | | | | | | |
|---|---|---|---|---|---|---|---|---|---|
| | | | | | | | | ENSESTT00000008310<br>ENSESTT00000008315<br>ENSESTT00000008311 | |
| 2335929 | 12651 | 2335922 | core | 51207886 | 51208084 | 1 | + | NM_001262<br>ENST00000262662 | Homo sapiens cyclin-dependent kinase inhibitor 2C (p18, inhibits CDK4) (CDKN2C), transcript variant 1, mRNA.<br>cdna:known-ccds chromosome:NCBI36:1:51199005:51212893:1 gene:ENSG00000123080 CCDS555.1 |
| 2346615 | 19524 | 2346575 | core | 92232231 | 92232316 | 1 | + | ENSESTT00000001716<br>GENSCAN00000053897<br>ENST00000361158<br>BX648135<br>ENST00000370389<br>NM_207189<br>NM_001726<br>ENST00000347608<br>ENST00000362005 | <br>cdna:Genscan chromosome:NCBI36:1:92148515:92252419:1<br>cdna:known chromosome:NCBI36:1:92187516:92252563:1 gene:ENSG00000137948<br>Homo sapiens mRNA; cDNA DKFZp686P0940 (from clone DKFZp686P0940).<br>cdna:novel chromosome:NCBI36:1:92187781:92252573:1 gene:ENSG00000137948<br>Homo sapiens bromodomain, testis-specific (BRDT), transcript variant 1, mRNA.<br>Homo sapiens bromodomain, testis-specific (BRDT), transcript variant 2, mRNA.<br>cdna:known-ccds chromosome:NCBI36:1:92187516:92252566:1 gene:ENSG00000137948 CCDS735.1<br>cdna:known chromosome:NCBI36:1:92187516:92252563:1 gene:ENSG00000137948 |
| 2350615 | 21999 | 2350596 | core | 109605673 | 109605753 | 1 | + | NM_001408<br>ENST00000271332<br>GENSCAN00000007705<br>AK091437<br>ENSESTT00000054051<br>ENSESTT00000054052<br>ENSESTT00000054053<br>AK127086 | Homo sapiens cadherin, EGF LAG seven-pass G-type receptor 2 (flamingo homolog, Drosophila) (CELSR2), mRNA.<br>cdna:known-ccds chromosome:NCBI36:1:109594164:109619895:1 gene:ENSG00000143126 CCDS796.1<br>cdna:Genscan chromosome:NCBI36:1:109594225:109617819:1<br>Homo sapiens cDNA FLJ34118 fis, clone FCBBF3009428, highly similar to Homo sapiens FLAMINGO 1 mRNA.<br><br><br><br>Homo sapiens cDNA FLJ45143 fis, clone BRAWH3040900, moderately similar to Homo sapiens cadherin, EGF LAG seven-pass G-type receptor 2 (flamingo homolog, Drosophila) (CELSR2) mRNA. |
| 2353352 | 23671 | 2353337 | core | 116363738 | 116363836 | 1 | + | ENST00000369502<br>AY358258<br>ENST00000295587<br>ENST00000369503<br>NM_018420<br>GENSCAN00000021535<br>ENSESTT00000036795 | cdna:known chromosome:NCBI36:1:116320758:116376034:1 gene:ENSG00000163393<br>Homo sapiens clone DNA175711 trans-like protein (UNQ9429) mRNA, complete cds.<br>cdna:known chromosome:NCBI36:1:116320642:116413548:1 gene:ENSG00000163393<br>cdna:known chromosome:NCBI36:1:116320642:116414198:1 gene:ENSG00000163393<br>Homo sapiens solute carrier family 22 (organic cation transporter), member 15 (SLC22A15), mRNA.<br>cdna:Genscan chromosome:NCBI36:1:116351552:116411252:1 |
| 2369338 | 33166 | 2369325 | core | 176784174 | 176784509 | 1 | + | AK097518<br>ENST00000319387<br>ENST00000258298<br>NM_207467 | Homo sapiens cDNA FLJ40199 fis, clone TESTI2020026.<br>cdna:known-ccds chromosome:NCBI36:1:176778554:176784647:1 gene:ENSG00000135820 CCDS1324.1<br>cdna:known chromosome:NCBI36:1:176748833:176767316:1 gene:ENSG00000184909<br>Homo sapiens FLJ35530 protein (FLJ35530), mRNA. |
| 2371179 | 34369 | 2371139 | core | 181476326 | 181476732 | 1 | + | NM_018891<br>ENST00000264144<br>ENST00000367544<br>GENSCAN00000000951<br>NM_005562<br>ENSESTT00000017773 | Homo sapiens laminin, gamma 2 (LAMC2), transcript variant 2, mRNA.<br>cdna:known-ccds chromosome:NCBI36:1:181422022:181480662:1 gene:ENSG00000058085 CCDS1352.1<br>cdna:known chromosome:NCBI36:1:181422046:181480658:1 gene:ENSG00000058085<br>cdna:Genscan chromosome:NCBI36:1:181400862:181479158:1<br>Homo sapiens laminin, gamma 2 (LAMC2), transcript variant 1, mRNA. |
| 2371189 | 34374 | 2371139 | core | 181480385 | 181480600 | 1 | + | ENST00000264144<br>NM_005562<br>GENSCAN00000000951<br>ENSESTT00000017773<br>NM_018891<br>ENST00000367544 | cdna:known-ccds chromosome:NCBI36:1:181422022:181480662:1 gene:ENSG00000058085 CCDS1352.1<br>Homo sapiens laminin, gamma 2 (LAMC2), transcript variant 1, mRNA.<br>cdna:Genscan chromosome:NCBI36:1:181400862:181479158:1<br><br>Homo sapiens laminin, gamma 2 (LAMC2), transcript variant 2, mRNA.<br>cdna:known chromosome:NCBI36:1:181422046:181480658:1 gene:ENSG00000058085 |
| 2376193 | 37577 | 2376168 | core | 203179982 | 203180043 | 1 | + | ENSESTT00000016404<br>ENST00000367169<br>NM_015090<br>ENSESTT00000016402 | <br>cdna:known chromosome:NCBI36:1:203064405:203212895:1 gene:ENSG00000163531<br>Homo sapiens neurofascin homolog (chicken) (NFASC), mRNA. |

| | | | | | | | | | |
|---|---|---|---|---|---|---|---|---|---|
| | | | | | | | | ENST00000367171<br>ENST00000367172<br>ENST00000367170<br>ENSESTT00000016403<br>AK090639<br>ENST00000339876<br>GENSCAN00000017937<br>BC040674<br>ENST00000295776<br>ENST00000338515<br>ENST00000338586<br>ENST00000360049<br>ENST00000367173 | cdna:known chromosome:NCBI36:1:203064405:203258587:1 gene:ENSG00000163531<br>cdna:known chromosome:NCBI36:1:203064405:203258587:1 gene:ENSG00000163531<br>cdna:known chromosome:NCBI36:1:203064405:203258587:1 gene:ENSG00000163531<br><br>Homo sapiens cDNA FLJ33320 fis, clone BNGH42007798, highly similar to Rattus norvegicus ankyrin binding cell adhesion molecule neurofascin mRNA.<br>cdna:known chromosome:NCBI36:1:203064405:203258587:1 gene:ENSG00000163531<br>cdna:Genscan chromosome:NCBI36:1:203173804:203252290:1<br>Homo sapiens neurofascin, mRNA (cDNA clone IMAGE:4817018).<br>cdna:known chromosome:NCBI36:1:203156423:203258572:1 gene:ENSG00000163531<br>cdna:known chromosome:NCBI36:1:203156423:203258572:1 gene:ENSG00000163531<br>cdna:known chromosome:NCBI36:1:203156423:203258572:1 gene:ENSG00000163531<br>cdna:known chromosome:NCBI36:1:203156423:203258572:1 gene:ENSG00000163531<br>cdna:novel chromosome:NCBI36:1:203064402:203246653:1 gene:ENSG00000163531 |
| 2376214 | 37593 | 2376168 | core | 203197860 | 203197908 | 1 | + | ENST00000295776<br>ENSESTT00000016402<br>ENST00000367171<br>NM_015090<br>ENST00000367172<br>ENST00000367173<br>ENST00000339876<br>AK090639<br>ENST00000360049<br>BC040674<br>GENSCAN00000017937<br>ENST00000367170<br>ENST00000367169<br>AB177861<br>ENST00000338515<br>ENSESTT00000016405<br>ENST00000338586<br>ENSESTT00000016407 | cdna:known chromosome:NCBI36:1:203156423:203258572:1 gene:ENSG00000163531<br><br>cdna:known chromosome:NCBI36:1:203064405:203258587:1 gene:ENSG00000163531<br>Homo sapiens neurofascin homolog (chicken) (NFASC), mRNA.<br>cdna:known chromosome:NCBI36:1:203064405:203258587:1 gene:ENSG00000163531<br>cdna:novel chromosome:NCBI36:1:203064402:203246653:1 gene:ENSG00000163531<br>cdna:known chromosome:NCBI36:1:203064405:203258587:1 gene:ENSG00000163531<br>Homo sapiens cDNA FLJ33320 fis, clone BNGH42007798, highly similar to Rattus norvegicus ankyrin binding cell adhesion molecule neurofascin mRNA.<br>cdna:known chromosome:NCBI36:1:203156423:203258572:1 gene:ENSG00000163531<br>Homo sapiens neurofascin, mRNA (cDNA clone IMAGE:4817018).<br>cdna:Genscan chromosome:NCBI36:1:203173804:203252290:1<br>cdna:known chromosome:NCBI36:1:203064405:203258587:1 gene:ENSG00000163531<br>cdna:known chromosome:NCBI36:1:203064405:203212895:1 gene:ENSG00000163531<br>Homo sapiens mRNA for KIAA0756 splice variant 1.<br>cdna:known chromosome:NCBI36:1:203156423:203258572:1 gene:ENSG00000163531<br><br>cdna:known chromosome:NCBI36:1:203156423:203258572:1 gene:ENSG00000163531 |
| 2376237 | 37612 | 2376168 | core | 203217552 | 203217757 | 1 | + | ENST00000339876<br>ENST00000295776<br>ENSESTT00000016402<br>ENST00000367171<br>NM_015090<br>ENST00000367172<br>ENST00000367173<br>AK090639<br>ENST00000360049<br>BC040674<br>GENSCAN00000017937<br>ENST00000338586<br>ENST00000367169<br>ENST00000367170<br>AB177861<br>ENST00000338515<br>ENSESTT00000016405<br>ENSESTT00000016407<br>BX641048<br>AK128699 | cdna:known chromosome:NCBI36:1:203064405:203258587:1 gene:ENSG00000163531<br>cdna:known chromosome:NCBI36:1:203156423:203258572:1 gene:ENSG00000163531<br><br>cdna:known chromosome:NCBI36:1:203064405:203258587:1 gene:ENSG00000163531<br>Homo sapiens neurofascin homolog (chicken) (NFASC), mRNA.<br>cdna:known chromosome:NCBI36:1:203064405:203258587:1 gene:ENSG00000163531<br>cdna:novel chromosome:NCBI36:1:203064402:203246653:1 gene:ENSG00000163531<br>Homo sapiens cDNA FLJ33320 fis, clone BNGH42007798, highly similar to Rattus norvegicus ankyrin binding cell adhesion molecule neurofascin mRNA.<br>cdna:known chromosome:NCBI36:1:203156423:203258572:1 gene:ENSG00000163531<br>Homo sapiens neurofascin, mRNA (cDNA clone IMAGE:4817018).<br>cdna:Genscan chromosome:NCBI36:1:203173804:203252290:1<br>cdna:known chromosome:NCBI36:1:203156423:203258572:1 gene:ENSG00000163531<br>cdna:known chromosome:NCBI36:1:203064405:203212895:1 gene:ENSG00000163531<br>cdna:known chromosome:NCBI36:1:203064405:203258587:1 gene:ENSG00000163531<br>Homo sapiens mRNA for KIAA0756 splice variant 1.<br>cdna:known chromosome:NCBI36:1:203156423:203258572:1 gene:ENSG00000163531<br><br><br><br>Homo sapiens mRNA; cDNA DKFZp686J0597 (from clone DKFZp686J0597). |

| | | | | | | | | | |
|---|---|---|---|---|---|---|---|---|---|
| | | | | | | | | AK127424 | Homo sapiens cDNA FLJ46866 fis, clone UTERU3011837, moderately similar to NG-CAM related cell adhesion molecule precursor.<br>Homo sapiens cDNA FLJ45516 fis, clone BRTHA2022968, moderately similar to NG-CAM related cell adhesion molecule precursor. |
| 2376269 | 37627 | 2376168 | core | 203238363 | 203238406 | 1 | + | ENST00000339876<br>ENST00000338586<br>GENSCAN00000017937<br>AK090639<br>NM_015090<br>ENST00000295776<br>ENST00000367169<br>ENST00000367172<br>ENST00000367170<br>ENST00000367171<br>ENST00000360049<br>ENST00000367173<br>BC040674<br>ENST00000338515<br>ENSESTT00000016407<br>AB177861<br>BX641048<br>AK128699<br>AK127424 | cdna:known chromosome:NCBI36:1:203064405:203258587:1 gene:ENSG00000163531<br>cdna:known chromosome:NCBI36:1:203156423:203258572:1 gene:ENSG00000163531<br>cdna:Genscan chromosome:NCBI36:1:203173804:203252290:1<br>Homo sapiens cDNA FLJ33320 fis, clone BNGH42007798, highly similar to Rattus norvegicus ankyrin binding cell adhesion molecule neurofascin mRNA.<br>Homo sapiens neurofascin homolog (chicken) (NFASC), mRNA.<br>cdna:known chromosome:NCBI36:1:203156423:203258572:1 gene:ENSG00000163531<br>cdna:known chromosome:NCBI36:1:203064405:203212895:1 gene:ENSG00000163531<br>cdna:known chromosome:NCBI36:1:203064405:203258587:1 gene:ENSG00000163531<br>cdna:known chromosome:NCBI36:1:203064405:203258587:1 gene:ENSG00000163531<br>cdna:known chromosome:NCBI36:1:203064405:203258587:1 gene:ENSG00000163531<br>cdna:known chromosome:NCBI36:1:203156423:203258572:1 gene:ENSG00000163531<br>cdna:novel chromosome:NCBI36:1:203064402:203246653:1 gene:ENSG00000163531<br>Homo sapiens neurofascin, mRNA (cDNA clone IMAGE:4817018).<br>cdna:known chromosome:NCBI36:1:203156423:203258572:1 gene:ENSG00000163531<br><br>Homo sapiens mRNA for KIAA0756 splice variant 1.<br>Homo sapiens mRNA; cDNA DKFZp686J0597 (from clone DKFZp686J0597).<br>Homo sapiens cDNA FLJ46866 fis, clone UTERU3011837, moderately similar to NG-CAM related cell adhesion molecule precursor.<br>Homo sapiens cDNA FLJ45516 fis, clone BRTHA2022968, moderately similar to NG-CAM related cell adhesion molecule precursor. |
| 2377111 | 38126 | 2377094 | core | 205307480 | 205307606 | 1 | + | NM_001018053<br>ENSESTT00000007558<br>GENSCAN00000027730<br>ENST00000271749<br>ENST00000367079<br>ENST00000367080<br>NM_006212<br>ENST00000344831 | Homo sapiens 6-phosphofructo-2-kinase/fructose-2,6-biphosphatase 2 (PFKFB2), transcript variant 2, mRNA.<br><br>cdna:Genscan chromosome:NCBI36:1:205294686:205318004:1<br>cdna:known chromosome:NCBI36:1:205293243:205317785:1 gene:ENSG00000123836<br>cdna:known chromosome:NCBI36:1:205293243:205320991:1 gene:ENSG00000123836<br>cdna:known chromosome:NCBI36:1:205293228:205317791:1 gene:ENSG00000123836<br>Homo sapiens 6-phosphofructo-2-kinase/fructose-2,6-biphosphatase 2 (PFKFB2), transcript variant 1, mRNA.<br>cdna:known chromosome:NCBI36:1:205293279:205320992:1 gene:ENSG00000123836 |
| 2377456 | 38358 | 2377427 | core | 205992068 | 205992122 | 1 | + | NM_172351<br>ENSESTT00000057539<br>NM_172360<br>NM_172355<br>ENST00000357714<br>NM_172354<br>ENST00000358170<br>ENST00000360212<br>NM_172352<br>NM_172357<br>NM_153826<br>ENST00000322875<br>ENST00000354848<br>NM_002389<br>ENST00000361067<br>NM_172359<br>ENST00000322918<br>ENST00000367047<br>NM_172350 | Homo sapiens CD46 molecule, complement regulatory protein (CD46), transcript variant c, mRNA.<br><br>Homo sapiens CD46 molecule, complement regulatory protein (CD46), transcript variant h, mRNA.<br>Homo sapiens CD46 molecule, complement regulatory protein (CD46), transcript variant i, mRNA.<br>cdna:known-ccds chromosome:NCBI36:1:205992025:206035481:1 gene:ENSG00000117335 CCDS1481.1<br>Homo sapiens CD46 molecule, complement regulatory protein (CD46), transcript variant g, mRNA.<br>cdna:known-ccds chromosome:NCBI36:1:205992025:206035481:1 gene:ENSG00000117335 CCDS1485.1<br>cdna:known-ccds chromosome:NCBI36:1:205992025:206035481:1 gene:ENSG00000117335 CCDS1484.1<br>Homo sapiens CD46 molecule, complement regulatory protein (CD46), transcript variant e, mRNA.<br>Homo sapiens CD46 molecule, complement regulatory protein (CD46), transcript variant k, mRNA.<br>Homo sapiens CD46 molecule, complement regulatory protein (CD46), transcript variant d, mRNA.<br>cdna:known-ccds chromosome:NCBI36:1:205992025:206035481:1 gene:ENSG00000117335 CCDS1482.1<br>cdna:known-ccds chromosome:NCBI36:1:205992025:206035481:1 gene:ENSG00000117335 CCDS1480.1<br>Homo sapiens CD46 molecule, complement regulatory protein (CD46), transcript variant a, mRNA.<br>cdna:known-ccds chromosome:NCBI36:1:205992025:206035481:1 gene:ENSG00000117335 CCDS1483.1<br>Homo sapiens CD46 molecule, complement regulatory protein (CD46), transcript variant b, mRNA.<br>cdna:known-ccds chromosome:NCBI36:1:205992025:206035481:1 gene:ENSG00000117335 CCDS1479.1<br>cdna:known chromosome:NCBI36:1:205992025:206035481:1 gene:ENSG00000117335<br>Homo sapiens CD46 molecule, complement regulatory protein (CD46), transcript variant n, mRNA. |

| | | | | | | | | | |
|---|---|---|---|---|---|---|---|---|---|
| | | | | | | | | NM_172358<br>ENST00000367041<br>NM_172361<br>ENST00000367042<br>NM_172353<br>NM_172356<br>ENSESTT00000057541<br>ENSESTT00000057540<br>ENSESTT00000057542 | Homo sapiens CD46 molecule, complement regulatory protein (CD46), transcript variant m, mRNA.<br>cdna:known chromosome:NCBI36:1:205992025:206035481:1 gene:ENSG00000117335<br>Homo sapiens CD46 molecule, complement regulatory protein (CD46), transcript variant l, mRNA.<br>cdna:known chromosome:NCBI36:1:205992025:206035481:1 gene:ENSG00000117335<br>Homo sapiens CD46 molecule, complement regulatory protein (CD46), transcript variant f, mRNA.<br>Homo sapiens CD46 molecule, complement regulatory protein (CD46), transcript variant j, mRNA. |
| 2398720 | 51231 | 2398706 | core | 17175980 | 17176005 | 1 | - | NM_002403<br>BC028033<br>NM_017459<br>ENST00000375535<br>ENSESTT00000039937<br>ENST00000235772<br>ENST00000375534<br>ENSESTT00000039938<br>GENSCAN00000024160 | Homo sapiens microfibrillar-associated protein 2 (MFAP2), transcript variant 2, mRNA.<br>Homo sapiens microfibrillar-associated protein 2, mRNA (cDNA clone IMAGE:5242233), with apparent retained intron.<br>Homo sapiens microfibrillar-associated protein 2 (MFAP2), transcript variant 1, mRNA.<br>cdna:known-ccds chromosome:NCBI36:1:17173584:17179917:-1 gene:ENSG00000117122 CCDS174.1<br><br>cdna:known chromosome:NCBI36:1:17173590:17179760:-1 gene:ENSG00000117122<br>cdna:known chromosome:NCBI36:1:17173584:17177358:-1 gene:ENSG00000117122<br><br>cdna:Genscan chromosome:NCBI36:1:17173325:17210602:-1 |
| 2402945 | 53810 | 2402942 | core | 27298356 | 27298979 | 1 | - | AK124218<br>ENST00000374089<br>ENST00000374093<br>NM_003047<br>ENST00000263980 | Homo sapiens cDNA FLJ42224 fis, clone THYMU2040114, highly similar to SODIUM/HYDROGEN EXCHANGER 1.<br>cdna:known chromosome:NCBI36:1:27297893:27303100:-1 gene:ENSG00000090020<br>cdna:known-ccds chromosome:NCBI36:1:27297893:27353988:-1 gene:ENSG00000090020 CCDS295.1<br>Homo sapiens solute carrier family 9 (sodium/hydrogen exchanger), member 1 (antiporter, Na+/H+, amiloride sensitive) (SLC9A1), mRNA.<br>cdna:known chromosome:NCBI36:1:27297894:27353990:-1 gene:ENSG00000090020 |
| 2405001 | 55081 | 2404999 | core | 32572256 | 32572577 | 1 | - | NM_023009<br>ENST00000329421<br>ENST00000309458 | Homo sapiens MARCKS-like 1 (MARCKSL1), mRNA.<br>cdna:known-ccds chromosome:NCBI36:1:32572020:32574567:-1 gene:ENSG00000175130 CCDS361.1<br>cdna:known chromosome:NCBI36:1:32572031:32574435:-1 gene:ENSG00000175130 |
| 2409153 | 57608 | 2409104 | core | 43196925 | 43196974 | 1 | - | ENSESTT00000045954<br>ENSESTT00000045955<br>ENST00000270929<br>GENSCAN00000047316<br>ENST00000372501<br>AB208987<br>NM_006516<br>ENST00000372500 | <br><br>cdna:known chromosome:NCBI36:1:43164115:43197088:-1 gene:ENSG00000117394<br>cdna:Genscan chromosome:NCBI36:1:43165299:43233077:-1<br>cdna:known-ccds chromosome:NCBI36:1:43164106:43197088:-1 gene:ENSG00000117394 CCDS477.1<br>Homo sapiens mRNA for solute carrier family 2 (facilitated glucose transporter), member 1 variant protein.<br>Homo sapiens solute carrier family 2 (facilitated glucose transporter), member 1 (SLC2A1), mRNA.<br>cdna:novel chromosome:NCBI36:1:43168577:43197117:-1 gene:ENSG00000117394 |
| 2411240 | 58798 | 2411228 | core | 47510335 | 47510381 | 1 | - | NM_003035<br>GENSCAN00000054579<br>ENST00000337817<br>AK128406<br>ENST00000371874<br>ENST00000360380<br>ENST00000371877<br>ENSESTT00000005478<br>ENSESTT00000005480<br>ENST00000243182<br>ENSESTT00000005479<br>ENSESTT00000005476<br>ENSESTT00000005472<br>ENSESTT00000005471 | Homo sapiens SCL/TAL1 interrupting locus (STIL), mRNA.<br>cdna:Genscan chromosome:NCBI36:1:47489395:47510452:-1<br>cdna:known-ccds chromosome:NCBI36:1:47488401:47552382:-1 gene:ENSG00000123473 CCDS548.1<br>Homo sapiens cDNA FLJ46549 fis, clone THYMU3038375, highly similar to Homo sapiens TAL1 (SCL) interrupting locus (SIL).<br>cdna:known chromosome:NCBI36:1:47488401:47519315:-1 gene:ENSG00000123473<br>cdna:known chromosome:NCBI36:1:47488436:47552406:-1 gene:ENSG00000123473<br>cdna:known chromosome:NCBI36:1:47488401:47552382:-1 gene:ENSG00000123473<br><br><br>cdna:known chromosome:NCBI36:1:47488401:47552382:-1 gene:ENSG00000123473 |

| | | | | | | | | | |
|---|---|---|---|---|---|---|---|---|---|
| 2411267 | 58813 | 2411228 | core | 47548597 | 47548621 | 1 | - | ENST00000243182<br>ENST00000371877<br>ENSESTT00000005471<br>ENST00000337817<br>ENST00000360380<br>NM_003035<br>GENSCAN00000064736 | cdna:known chromosome:NCBI36:1:47488401:47552382:-1 gene:ENSG00000123473<br>cdna:known chromosome:NCBI36:1:47488401:47552382:-1 gene:ENSG00000123473<br><br>cdna:known-ccds chromosome:NCBI36:1:47488401:47552382:-1 gene:ENSG00000123473 CCDS548.1<br>cdna:known chromosome:NCBI36:1:47488436:47552406:-1 gene:ENSG00000123473<br>Homo sapiens SCL/TAL1 interrupting locus (STIL), mRNA.<br>cdna:Genscan chromosome:NCBI36:1:47518479:47548578:-1 |
| 2413218 | 60080 | 2413203 | core | 53493377 | 53493421 | 1 | - | ENST00000371456<br>ENST00000371454<br>NM_001018054<br>ENST00000354412<br>ENST00000306052<br>NM_004631<br>NM_017522<br>ENSESTT00000008248<br>ENST00000357488<br>ENSESTT00000008249<br>GENSCAN00000034038<br>ENST00000371452<br>AK122887<br>NM_033300<br>ENST00000347547<br>AK096482 | cdna:known-ccds chromosome:NCBI36:1:53483805:53566274:-1 gene:ENSG00000157193 CCDS578.1<br>cdna:known chromosome:NCBI36:1:53483806:53566314:-1 gene:ENSG00000157193<br>Homo sapiens low density lipoprotein receptor-related protein 8, apolipoprotein e receptor (LRP8), transcript variant 4, mRNA.<br>cdna:known-ccds chromosome:NCBI36:1:53483806:53566314:-1 gene:ENSG00000157193 CCDS579.1<br>cdna:known chromosome:NCBI36:1:53483806:53566314:-1 gene:ENSG00000157193<br>Homo sapiens low density lipoprotein receptor-related protein 8, apolipoprotein e receptor (LRP8), transcript variant 1, mRNA.<br>Homo sapiens low density lipoprotein receptor-related protein 8, apolipoprotein e receptor (LRP8), transcript variant 3, mRNA.<br><br>cdna:known chromosome:NCBI36:1:53484813:53566314:-1 gene:ENSG00000157193<br><br>cdna:Genscan chromosome:NCBI36:1:53485278:53502656:-1<br>cdna:known chromosome:NCBI36:1:53484828:53566172:-1 gene:ENSG00000157193<br>Homo sapiens cDNA FLJ16536 fis, clone OCBBF2032152, highly similar to Homo sapiens low density lipoprotein receptor-related protein 8, apolipoprotein e receptor (LRP8).<br>Homo sapiens low density lipoprotein receptor-related protein 8, apolipoprotein e receptor (LRP8), transcript variant 2, mRNA.<br>cdna:known-ccds chromosome:NCBI36:1:53483800:53566409:-1 gene:ENSG00000157193 CCDS580.1<br>Homo sapiens cDNA FLJ39163 fis, clone OCBBF2002615, highly similar to Human mRNA for apolipoprotein E receptor 2. |
| 2419049 | 63834 | 2419046 | core | 77803658 | 77803788 | 1 | - | NM_015534<br>BC035079<br>BX640658<br>ENST00000370801<br>ENST00000370798<br>AK074119<br>ENST00000263190<br>GENSCAN00000000971<br>ENSESTT00000009675<br>ENSESTT00000009674<br>GENSCAN00000002078<br>ENSESTT00000009676<br>ENSESTT00000009673 | Homo sapiens zinc finger, ZZ-type containing 3 (ZZZ3), mRNA.<br>Homo sapiens zinc finger, ZZ-type containing 3, mRNA (cDNA clone IMAGE:5260515), complete cds.<br>Homo sapiens mRNA; cDNA DKFZp313N0119 (from clone DKFZp313N0119).<br>cdna:known-ccds chromosome:NCBI36:1:77802773:77920931:-1 gene:ENSG00000036549 CCDS677.1<br>cdna:known chromosome:NCBI36:1:77803161:77921692:-1 gene:ENSG00000036549<br>Homo sapiens mRNA for FLJ00191 protein.<br>cdna:known chromosome:NCBI36:1:77802779:77920931:-1 gene:ENSG00000036549<br>cdna:Genscan chromosome:NCBI36:1:77803913:77814493:-1<br><br><br><br>cdna:Genscan chromosome:NCBI36:1:77817800:77871627:-1 |
| 2424108 | 67041 | 2424102 | core | 95137535 | 95137632 | 1 | - | GENSCAN00000066593<br>NM_001839<br>ENST00000370206<br>ENST00000281863 | cdna:Genscan chromosome:NCBI36:1:95135886:95141668:-1<br>Homo sapiens calponin 3, acidic (CNN3), mRNA.<br>cdna:known chromosome:NCBI36:1:95135095:95165422:-1 gene:ENSG00000117519<br>cdna:known chromosome:NCBI36:1:95135097:95165289:-1 gene:ENSG00000117519 |
| 2425766 | 68112 | 2425756 | core | 103121352 | 103121437 | 1 | - | ENST00000353414<br>ENST00000358392<br>NM_080630<br>ENSESTT00000034471<br>NM_001854<br>ENST00000370096<br>GENSCAN00000063565 | cdna:known-ccds chromosome:NCBI36:1:103115583:103346640:-1 gene:ENSG00000060718 CCDS780.1<br>cdna:known-ccds chromosome:NCBI36:1:103115583:103346640:-1 gene:ENSG00000060718 CCDS779.1<br>Homo sapiens collagen, type XI, alpha 1 (COL11A1), transcript variant C, mRNA.<br><br>Homo sapiens collagen, type XI, alpha 1 (COL11A1), transcript variant A, mRNA.<br>cdna:known-ccds chromosome:NCBI36:1:103114611:103346635:-1 gene:ENSG00000060718 CCDS778.1<br>cdna:Genscan chromosome:NCBI36:1:103116163:103161533:-1 |

| | | | | | | | | | |
|---|---|---|---|---|---|---|---|---|---|
| | | | | | | | | NM_080629<br>ENST00000193186<br>ENST00000370090<br>ENSESTT00000034470<br>GENSCAN00000010468<br>ENSESTT00000034469 | Homo sapiens collagen, type XI, alpha 1 (COL11A1), transcript variant B, mRNA.<br>cdna:known chromosome:NCBI36:1:103115583:103346640:-1 gene:ENSG00000060718<br>cdna:known chromosome:NCBI36:1:103115629:103269357:-1 gene:ENSG00000060718<br><br>cdna:Genscan chromosome:NCBI36:1:103172593:103234186:-1 |
| 2425775 | 68118 | 2425756 | core | 103128613 | 103128643 | 1 | - | ENST00000353414<br>ENST00000358392<br>NM_080630<br>ENSESTT00000034471<br>NM_001854<br>ENST00000370096<br>GENSCAN00000063565<br>NM_080629<br>ENST00000193186<br>ENST00000370090<br>ENSESTT00000034470<br>GENSCAN00000010468<br>ENSESTT00000034469 | cdna:known-ccds chromosome:NCBI36:1:103115583:103346640:-1 gene:ENSG00000060718 CCDS780.1<br>cdna:known-ccds chromosome:NCBI36:1:103115583:103346640:-1 gene:ENSG00000060718 CCDS779.1<br>Homo sapiens collagen, type XI, alpha 1 (COL11A1), transcript variant C, mRNA.<br><br>Homo sapiens collagen, type XI, alpha 1 (COL11A1), transcript variant A, mRNA.<br>cdna:known-ccds chromosome:NCBI36:1:103114611:103346635:-1 gene:ENSG00000060718 CCDS778.1<br>cdna:Genscan chromosome:NCBI36:1:103116163:103161533:-1<br>Homo sapiens collagen, type XI, alpha 1 (COL11A1), transcript variant B, mRNA.<br>cdna:known chromosome:NCBI36:1:103115583:103346640:-1 gene:ENSG00000060718<br>cdna:known chromosome:NCBI36:1:103115629:103269357:-1 gene:ENSG00000060718<br><br>cdna:Genscan chromosome:NCBI36:1:103172593:103234186:-1 |
| 2425778 | 68120 | 2425756 | core | 103136815 | 103136882 | 1 | - | NM_080630<br>ENST00000370096<br>ENST00000370090<br>ENST00000353414<br>ENST00000358392<br>ENST00000193186<br>NM_080629<br>NM_001854<br>GENSCAN00000063565<br>ENSESTT00000034471<br>ENSESTT00000034470<br>GENSCAN00000010468<br>ENSESTT00000034469 | Homo sapiens collagen, type XI, alpha 1 (COL11A1), transcript variant C, mRNA.<br>cdna:known-ccds chromosome:NCBI36:1:103114611:103346635:-1 gene:ENSG00000060718 CCDS778.1<br>cdna:known chromosome:NCBI36:1:103115629:103269357:-1 gene:ENSG00000060718<br>cdna:known-ccds chromosome:NCBI36:1:103115583:103346640:-1 gene:ENSG00000060718 CCDS780.1<br>cdna:known-ccds chromosome:NCBI36:1:103115583:103346640:-1 gene:ENSG00000060718 CCDS779.1<br>cdna:known chromosome:NCBI36:1:103115583:103346640:-1 gene:ENSG00000060718<br>Homo sapiens collagen, type XI, alpha 1 (COL11A1), transcript variant B, mRNA.<br>Homo sapiens collagen, type XI, alpha 1 (COL11A1), transcript variant A, mRNA.<br>cdna:Genscan chromosome:NCBI36:1:103116163:103161533:-1<br><br><br>cdna:Genscan chromosome:NCBI36:1:103172593:103234186:-1 |
| 2425783 | 68125 | 2425756 | core | 103152848 | 103152927 | 1 | - | GENSCAN00000063565<br>ENST00000193186<br>NM_001854<br>ENST00000370090<br>ENST00000358392<br>ENST00000353414<br>ENST00000370096<br>NM_080629<br>ENSESTT00000034470<br>NM_080630 | cdna:Genscan chromosome:NCBI36:1:103116163:103161533:-1<br>cdna:known chromosome:NCBI36:1:103115583:103346640:-1 gene:ENSG00000060718<br>Homo sapiens collagen, type XI, alpha 1 (COL11A1), transcript variant A, mRNA.<br>cdna:known chromosome:NCBI36:1:103115629:103269357:-1 gene:ENSG00000060718<br>cdna:known-ccds chromosome:NCBI36:1:103115583:103346640:-1 gene:ENSG00000060718 CCDS779.1<br>cdna:known-ccds chromosome:NCBI36:1:103115583:103346640:-1 gene:ENSG00000060718 CCDS780.1<br>cdna:known-ccds chromosome:NCBI36:1:103114611:103346635:-1 gene:ENSG00000060718 CCDS778.1<br>Homo sapiens collagen, type XI, alpha 1 (COL11A1), transcript variant B, mRNA.<br><br>Homo sapiens collagen, type XI, alpha 1 (COL11A1), transcript variant C, mRNA. |
| 2425785 | 68127 | 2425756 | core | 103158456 | 103158496 | 1 | - | ENST00000353414<br>ENST00000358392<br>NM_080630<br>ENSESTT00000034471<br>NM_001854<br>ENST00000370096<br>GENSCAN00000063565<br>NM_080629<br>ENST00000193186<br>ENST00000370090<br>ENSESTT00000034470 | cdna:known-ccds chromosome:NCBI36:1:103115583:103346640:-1 gene:ENSG00000060718 CCDS780.1<br>cdna:known-ccds chromosome:NCBI36:1:103115583:103346640:-1 gene:ENSG00000060718 CCDS779.1<br>Homo sapiens collagen, type XI, alpha 1 (COL11A1), transcript variant C, mRNA.<br><br>Homo sapiens collagen, type XI, alpha 1 (COL11A1), transcript variant A, mRNA.<br>cdna:known-ccds chromosome:NCBI36:1:103114611:103346635:-1 gene:ENSG00000060718 CCDS778.1<br>cdna:Genscan chromosome:NCBI36:1:103116163:103161533:-1<br>Homo sapiens collagen, type XI, alpha 1 (COL11A1), transcript variant B, mRNA.<br>cdna:known chromosome:NCBI36:1:103115583:103346640:-1 gene:ENSG00000060718<br>cdna:known chromosome:NCBI36:1:103115629:103269357:-1 gene:ENSG00000060718 |

| | | | | | | | | | |
|---|---|---|---|---|---|---|---|---|---|
| | | | | | | | | GENSCAN00000010468<br>ENSESTT00000034469 | cdna:Genscan chromosome:NCBI36:1:103172593:103234186:-1 |
| 2425786 | 68128 | 2425756 | core | 103159662 | 103159694 | 1 | - | ENST00000353414<br>ENST00000358392<br>NM_080630<br>ENSESTT00000034471<br>NM_001854<br>ENST00000370096<br>GENSCAN00000063565<br>NM_080629<br>ENST00000193186<br>ENST00000370090<br>ENSESTT00000034470<br>GENSCAN00000010468<br>ENSESTT00000034469 | cdna:known-ccds chromosome:NCBI36:1:103115583:103346640:-1 gene:ENSG00000060718 CCDS780.1<br>cdna:known-ccds chromosome:NCBI36:1:103115583:103346640:-1 gene:ENSG00000060718 CCDS779.1<br>Homo sapiens collagen, type XI, alpha 1 (COL11A1), transcript variant C, mRNA.<br><br>Homo sapiens collagen, type XI, alpha 1 (COL11A1), transcript variant A, mRNA.<br>cdna:known-ccds chromosome:NCBI36:1:103114611:103346635:-1 gene:ENSG00000060718 CCDS778.1<br>cdna:Genscan chromosome:NCBI36:1:103116163:103161533:-1<br>Homo sapiens collagen, type XI, alpha 1 (COL11A1), transcript variant B, mRNA.<br>cdna:known chromosome:NCBI36:1:103115583:103346640:-1 gene:ENSG00000060718<br>cdna:known chromosome:NCBI36:1:103115629:103269357:-1 gene:ENSG00000060718<br><br>cdna:Genscan chromosome:NCBI36:1:103172593:103234186:-1 |
| 2425790 | 68131 | 2425756 | core | 103173213 | 103173243 | 1 | - | ENST00000193186<br>GENSCAN00000010468<br>GENSCAN00000063565<br>ENST00000353414<br>ENST00000358392<br>ENST00000370090<br>ENST00000370096<br>NM_001854<br>ENSESTT00000034471<br>NM_080629<br>NM_080630<br>ENSESTT00000034470<br>ENSESTT00000034469 | cdna:known chromosome:NCBI36:1:103115583:103346640:-1 gene:ENSG00000060718<br>cdna:Genscan chromosome:NCBI36:1:103172593:103234186:-1<br>cdna:Genscan chromosome:NCBI36:1:103116163:103161533:-1<br>cdna:known-ccds chromosome:NCBI36:1:103115583:103346640:-1 gene:ENSG00000060718 CCDS780.1<br>cdna:known-ccds chromosome:NCBI36:1:103115583:103346640:-1 gene:ENSG00000060718 CCDS779.1<br>cdna:known chromosome:NCBI36:1:103115629:103269357:-1 gene:ENSG00000060718<br>cdna:known-ccds chromosome:NCBI36:1:103114611:103346635:-1 gene:ENSG00000060718 CCDS778.1<br>Homo sapiens collagen, type XI, alpha 1 (COL11A1), transcript variant A, mRNA.<br><br>Homo sapiens collagen, type XI, alpha 1 (COL11A1), transcript variant B, mRNA.<br>Homo sapiens collagen, type XI, alpha 1 (COL11A1), transcript variant C, mRNA. |
| 2425793 | 68134 | 2425756 | core | 103185041 | 103185098 | 1 | - | ENST00000370096<br>NM_080629<br>ENSESTT00000034470<br>ENST00000358392<br>ENST00000193186<br>NM_001854<br>NM_080630<br>ENST00000353414<br>ENST00000370090<br>GENSCAN00000010468 | cdna:known-ccds chromosome:NCBI36:1:103114611:103346635:-1 gene:ENSG00000060718 CCDS778.1<br>Homo sapiens collagen, type XI, alpha 1 (COL11A1), transcript variant B, mRNA.<br><br>cdna:known-ccds chromosome:NCBI36:1:103115583:103346640:-1 gene:ENSG00000060718 CCDS779.1<br>cdna:known chromosome:NCBI36:1:103115583:103346640:-1 gene:ENSG00000060718<br>Homo sapiens collagen, type XI, alpha 1 (COL11A1), transcript variant A, mRNA.<br>Homo sapiens collagen, type XI, alpha 1 (COL11A1), transcript variant C, mRNA.<br>cdna:known-ccds chromosome:NCBI36:1:103115583:103346640:-1 gene:ENSG00000060718 CCDS780.1<br>cdna:known chromosome:NCBI36:1:103115629:103269357:-1 gene:ENSG00000060718<br>cdna:Genscan chromosome:NCBI36:1:103172593:103234186:-1 |
| 2425796 | 68136 | 2425756 | core | 103200352 | 103200397 | 1 | - | ENST00000358392<br>ENST00000353414<br>ENST00000193186<br>ENST00000370090<br>ENST00000370096<br>NM_080629<br>ENSESTT00000034470<br>NM_080630<br>NM_001854<br>GENSCAN00000010468<br>ENSESTT00000034469<br>ENSESTT00000034467 | cdna:known-ccds chromosome:NCBI36:1:103115583:103346640:-1 gene:ENSG00000060718 CCDS779.1<br>cdna:known-ccds chromosome:NCBI36:1:103115583:103346640:-1 gene:ENSG00000060718 CCDS780.1<br>cdna:known chromosome:NCBI36:1:103115583:103346640:-1 gene:ENSG00000060718<br>cdna:known chromosome:NCBI36:1:103115629:103269357:-1 gene:ENSG00000060718<br>cdna:known-ccds chromosome:NCBI36:1:103114611:103346635:-1 gene:ENSG00000060718 CCDS778.1<br>Homo sapiens collagen, type XI, alpha 1 (COL11A1), transcript variant B, mRNA.<br><br>Homo sapiens collagen, type XI, alpha 1 (COL11A1), transcript variant C, mRNA.<br>Homo sapiens collagen, type XI, alpha 1 (COL11A1), transcript variant A, mRNA.<br>cdna:Genscan chromosome:NCBI36:1:103172593:103234186:-1 |
| 2425801 | 68141 | 2425756 | core | 103216866 | 103216896 | 1 | - | NM_080629<br>NM_080630<br>NM_001854 | Homo sapiens collagen, type XI, alpha 1 (COL11A1), transcript variant B, mRNA.<br>Homo sapiens collagen, type XI, alpha 1 (COL11A1), transcript variant C, mRNA.<br>Homo sapiens collagen, type XI, alpha 1 (COL11A1), transcript variant A, mRNA. |

| | | | | | | | | | |
|---|---|---|---|---|---|---|---|---|---|
| | | | | | | | | ENST00000370096<br>ENSESTT00000034471<br>ENST00000193186<br>ENST00000353414<br>ENST00000370090<br>ENST00000358392<br>GENSCAN00000063565<br>ENSESTT00000034470<br>GENSCAN00000010468<br>ENSESTT00000034469<br>ENSESTT00000034468<br>ENSESTT00000034467 | cdna:known-ccds chromosome:NCBI36:1:103114611:103346635:-1 gene:ENSG00000060718 CCDS778.1<br><br>cdna:known chromosome:NCBI36:1:103115583:103346640:-1 gene:ENSG00000060718<br>cdna:known-ccds chromosome:NCBI36:1:103115583:103346640:-1 gene:ENSG00000060718 CCDS780.1<br>cdna:known chromosome:NCBI36:1:103115629:103269357:-1 gene:ENSG00000060718<br>cdna:known-ccds chromosome:NCBI36:1:103115583:103346640:-1 gene:ENSG00000060718 CCDS779.1<br>cdna:Genscan chromosome:NCBI36:1:103116163:103161533:-1<br><br>cdna:Genscan chromosome:NCBI36:1:103172593:103234186:-1 |
| 2425802 | 68142 | 2425756 | core | 103217012 | 103217036 | 1 | - | NM_080629<br>NM_080630<br>NM_001854<br>ENST00000370096<br>ENSESTT00000034471<br>ENST00000193186<br>ENST00000353414<br>ENST00000370090<br>ENST00000358392<br>GENSCAN00000063565<br>ENSESTT00000034470<br>GENSCAN00000010468<br>ENSESTT00000034469<br>ENSESTT00000034468<br>ENSESTT00000034467 | Homo sapiens collagen, type XI, alpha 1 (COL11A1), transcript variant B, mRNA.<br>Homo sapiens collagen, type XI, alpha 1 (COL11A1), transcript variant C, mRNA.<br>Homo sapiens collagen, type XI, alpha 1 (COL11A1), transcript variant A, mRNA.<br>cdna:known-ccds chromosome:NCBI36:1:103114611:103346635:-1 gene:ENSG00000060718 CCDS778.1<br><br>cdna:known chromosome:NCBI36:1:103115583:103346640:-1 gene:ENSG00000060718<br>cdna:known-ccds chromosome:NCBI36:1:103115583:103346640:-1 gene:ENSG00000060718 CCDS780.1<br>cdna:known chromosome:NCBI36:1:103115629:103269357:-1 gene:ENSG00000060718<br>cdna:known-ccds chromosome:NCBI36:1:103115583:103346640:-1 gene:ENSG00000060718 CCDS779.1<br>cdna:Genscan chromosome:NCBI36:1:103116163:103161533:-1<br><br>cdna:Genscan chromosome:NCBI36:1:103172593:103234186:-1 |
| 2425805 | 68145 | 2425756 | core | 103222302 | 103222327 | 1 | - | ENST00000193186<br>GENSCAN00000063565<br>ENST00000353414<br>ENST00000358392<br>ENST00000370090<br>ENST00000370096<br>NM_001854<br>ENSESTT00000034471<br>NM_080629<br>NM_080630<br>ENSESTT00000034470<br>ENSESTT00000034469 | cdna:known chromosome:NCBI36:1:103115583:103346640:-1 gene:ENSG00000060718<br>cdna:Genscan chromosome:NCBI36:1:103116163:103161533:-1<br>cdna:known-ccds chromosome:NCBI36:1:103115583:103346640:-1 gene:ENSG00000060718 CCDS780.1<br>cdna:known-ccds chromosome:NCBI36:1:103115583:103346640:-1 gene:ENSG00000060718 CCDS779.1<br>cdna:known chromosome:NCBI36:1:103115629:103269357:-1 gene:ENSG00000060718<br>cdna:known-ccds chromosome:NCBI36:1:103114611:103346635:-1 gene:ENSG00000060718 CCDS778.1<br>Homo sapiens collagen, type XI, alpha 1 (COL11A1), transcript variant A, mRNA.<br><br>Homo sapiens collagen, type XI, alpha 1 (COL11A1), transcript variant B, mRNA.<br>Homo sapiens collagen, type XI, alpha 1 (COL11A1), transcript variant C, mRNA. |
| 2425807 | 68146 | 2425756 | core | 103225782 | 103225883 | 1 | - | ENSESTT00000034469<br>ENST00000370096<br>ENST00000358392<br>NM_001854<br>NM_080630<br>GENSCAN00000010468<br>NM_080629<br>ENST00000370090<br>ENST00000193186<br>ENST00000353414 | <br>cdna:known-ccds chromosome:NCBI36:1:103114611:103346635:-1 gene:ENSG00000060718 CCDS778.1<br>cdna:known-ccds chromosome:NCBI36:1:103115583:103346640:-1 gene:ENSG00000060718 CCDS779.1<br>Homo sapiens collagen, type XI, alpha 1 (COL11A1), transcript variant A, mRNA.<br>Homo sapiens collagen, type XI, alpha 1 (COL11A1), transcript variant C, mRNA.<br>cdna:Genscan chromosome:NCBI36:1:103172593:103234186:-1<br>Homo sapiens collagen, type XI, alpha 1 (COL11A1), transcript variant B, mRNA.<br>cdna:known chromosome:NCBI36:1:103115629:103269357:-1 gene:ENSG00000060718<br>cdna:known chromosome:NCBI36:1:103115583:103346640:-1 gene:ENSG00000060718<br>cdna:known-ccds chromosome:NCBI36:1:103115583:103346640:-1 gene:ENSG00000060718 CCDS780.1 |
| 2425810 | 68149 | 2425756 | core | 103234151 | 103234181 | 1 | - | NM_080630<br>ENST00000370096<br>ENST00000370090<br>ENST00000353414 | Homo sapiens collagen, type XI, alpha 1 (COL11A1), transcript variant C, mRNA.<br>cdna:known-ccds chromosome:NCBI36:1:103114611:103346635:-1 gene:ENSG00000060718 CCDS778.1<br>cdna:known chromosome:NCBI36:1:103115629:103269357:-1 gene:ENSG00000060718<br>cdna:known-ccds chromosome:NCBI36:1:103115583:103346640:-1 gene:ENSG00000060718 CCDS780.1 |

| | | | | | | | | | |
|---|---|---|---|---|---|---|---|---|---|
| | | | | | | | | ENST00000358392<br>ENST00000193186<br>NM_080629<br>NM_001854<br>GENSCAN00000063565<br>ENSESTT00000034471<br>ENSESTT00000034470<br>GENSCAN00000010468<br>ENSESTT00000034469 | cdna:known-ccds chromosome:NCBI36:1:103115583:103346640:-1 gene:ENSG00000060718 CCDS779.1<br>cdna:known chromosome:NCBI36:1:103115583:103346640:-1 gene:ENSG00000060718<br>Homo sapiens collagen, type XI, alpha 1 (COL11A1), transcript variant B, mRNA.<br>Homo sapiens collagen, type XI, alpha 1 (COL11A1), transcript variant A, mRNA.<br>cdna:Genscan chromosome:NCBI36:1:103116163:103161533:-1<br><br><br>cdna:Genscan chromosome:NCBI36:1:103172593:103234186:-1 |
| 2425819 | 68157 | 2425756 | core | 103242598 | 103242623 | 1 | - | NM_080629<br>NM_080630<br>NM_001854<br>ENST00000370096<br>ENSESTT00000034471<br>ENST00000193186<br>ENST00000353414<br>ENST00000370090<br>ENST00000358392<br>GENSCAN00000063565<br>ENSESTT00000034470<br>GENSCAN00000010468<br>ENSESTT00000034469<br>ENSESTT00000034468<br>ENSESTT00000034467 | Homo sapiens collagen, type XI, alpha 1 (COL11A1), transcript variant B, mRNA.<br>Homo sapiens collagen, type XI, alpha 1 (COL11A1), transcript variant C, mRNA.<br>Homo sapiens collagen, type XI, alpha 1 (COL11A1), transcript variant A, mRNA.<br>cdna:known-ccds chromosome:NCBI36:1:103114611:103346635:-1 gene:ENSG00000060718 CCDS778.1<br><br>cdna:known chromosome:NCBI36:1:103115583:103346640:-1 gene:ENSG00000060718<br>cdna:known-ccds chromosome:NCBI36:1:103115583:103346640:-1 gene:ENSG00000060718 CCDS780.1<br>cdna:known chromosome:NCBI36:1:103115629:103269357:-1 gene:ENSG00000060718<br>cdna:known-ccds chromosome:NCBI36:1:103115583:103346640:-1 gene:ENSG00000060718 CCDS779.1<br>cdna:Genscan chromosome:NCBI36:1:103116163:103161533:-1<br><br>cdna:Genscan chromosome:NCBI36:1:103172593:103234186:-1 |
| 2425821 | 68159 | 2425756 | core | 103243987 | 103244034 | 1 | - | NM_001854<br>ENST00000370096<br>ENST00000193186<br>NM_080629<br>ENST00000353414<br>ENST00000358392<br>ENST00000370090<br>ENSESTT00000034471<br>NM_080630<br>ENSESTT00000034470<br>GENSCAN00000063565<br>ENSESTT00000034468<br>ENSESTT00000034467 | Homo sapiens collagen, type XI, alpha 1 (COL11A1), transcript variant A, mRNA.<br>cdna:known-ccds chromosome:NCBI36:1:103114611:103346635:-1 gene:ENSG00000060718 CCDS778.1<br>cdna:known chromosome:NCBI36:1:103115583:103346640:-1 gene:ENSG00000060718<br>Homo sapiens collagen, type XI, alpha 1 (COL11A1), transcript variant B, mRNA.<br>cdna:known-ccds chromosome:NCBI36:1:103115583:103346640:-1 gene:ENSG00000060718 CCDS780.1<br>cdna:known-ccds chromosome:NCBI36:1:103115583:103346640:-1 gene:ENSG00000060718 CCDS779.1<br>cdna:known chromosome:NCBI36:1:103115629:103269357:-1 gene:ENSG00000060718<br><br>Homo sapiens collagen, type XI, alpha 1 (COL11A1), transcript variant C, mRNA.<br><br>cdna:Genscan chromosome:NCBI36:1:103116163:103161533:-1 |
| 2425825 | 68163 | 2425756 | core | 103250558 | 103250598 | 1 | - | ENST00000370096<br>NM_080629<br>ENSESTT00000034470<br>ENST00000358392<br>ENST00000193186<br>NM_001854<br>NM_080630<br>ENST00000353414<br>ENST00000370090<br>GENSCAN00000010468 | cdna:known-ccds chromosome:NCBI36:1:103114611:103346635:-1 gene:ENSG00000060718 CCDS778.1<br>Homo sapiens collagen, type XI, alpha 1 (COL11A1), transcript variant B, mRNA.<br><br>cdna:known-ccds chromosome:NCBI36:1:103115583:103346640:-1 gene:ENSG00000060718 CCDS779.1<br>cdna:known chromosome:NCBI36:1:103115583:103346640:-1 gene:ENSG00000060718<br>Homo sapiens collagen, type XI, alpha 1 (COL11A1), transcript variant A, mRNA.<br>Homo sapiens collagen, type XI, alpha 1 (COL11A1), transcript variant C, mRNA.<br>cdna:known-ccds chromosome:NCBI36:1:103115583:103346640:-1 gene:ENSG00000060718 CCDS780.1<br>cdna:known chromosome:NCBI36:1:103115629:103269357:-1 gene:ENSG00000060718<br>cdna:Genscan chromosome:NCBI36:1:103172593:103234186:-1 |
| 2425826 | 68164 | 2425756 | core | 103252697 | 103252730 | 1 | - | ENSESTT00000034467<br>ENST00000358392<br>ENST00000370096<br>NM_080630<br>ENST00000353414<br>ENST00000370090 | <br>cdna:known-ccds chromosome:NCBI36:1:103115583:103346640:-1 gene:ENSG00000060718 CCDS779.1<br>cdna:known-ccds chromosome:NCBI36:1:103114611:103346635:-1 gene:ENSG00000060718 CCDS778.1<br>Homo sapiens collagen, type XI, alpha 1 (COL11A1), transcript variant C, mRNA.<br>cdna:known-ccds chromosome:NCBI36:1:103115583:103346640:-1 gene:ENSG00000060718 CCDS780.1<br>cdna:known chromosome:NCBI36:1:103115629:103269357:-1 gene:ENSG00000060718 |

| | | | | | | | | | |
|---|---|---|---|---|---|---|---|---|---|
| | | | | | | | | NM_001854<br>NM_080629<br>ENST00000193186<br>ENSESTT00000034468<br>ENSESTT00000034466 | Homo sapiens collagen, type XI, alpha 1 (COL11A1), transcript variant A, mRNA.<br>Homo sapiens collagen, type XI, alpha 1 (COL11A1), transcript variant B, mRNA.<br>cdna:known chromosome:NCBI36:1:103115583:103346640:-1 gene:ENSG00000060718 |
| 2425849 | 68182 | 2425756 | core | 103346240 | 103346285 | 1 | - | ENSESTT00000034465<br>ENST00000370096<br>ENST00000358392<br>ENST00000353414<br>ENST00000193186<br>NM_080629<br>ENSESTT00000034470<br>NM_001854<br>NM_080630<br>ENST00000370090<br>GENSCAN00000010468<br>ENSESTT00000034469<br>ENSESTT00000034467 | <br>cdna:known-ccds chromosome:NCBI36:1:103114611:103346635:-1 gene:ENSG00000060718 CCDS778.1<br>cdna:known-ccds chromosome:NCBI36:1:103115583:103346640:-1 gene:ENSG00000060718 CCDS779.1<br>cdna:known-ccds chromosome:NCBI36:1:103115583:103346640:-1 gene:ENSG00000060718 CCDS780.1<br>cdna:known chromosome:NCBI36:1:103115583:103346640:-1 gene:ENSG00000060718<br>Homo sapiens collagen, type XI, alpha 1 (COL11A1), transcript variant B, mRNA.<br><br>Homo sapiens collagen, type XI, alpha 1 (COL11A1), transcript variant A, mRNA.<br>Homo sapiens collagen, type XI, alpha 1 (COL11A1), transcript variant C, mRNA.<br>cdna:known chromosome:NCBI36:1:103115629:103269357:-1 gene:ENSG00000060718<br>cdna:Genscan chromosome:NCBI36:1:103172593:103234186:-1 |
| 2425850 | 68183 | 2425756 | core | 103346352 | 103346504 | 1 | - | ENSESTT00000034465<br>ENST00000370096<br>ENST00000358392<br>ENST00000353414<br>ENST00000193186<br>NM_080629<br>ENSESTT00000034470<br>NM_001854<br>NM_080630<br>ENST00000370090<br>GENSCAN00000010468<br>ENSESTT00000034469<br>ENSESTT00000034467 | <br>cdna:known-ccds chromosome:NCBI36:1:103114611:103346635:-1 gene:ENSG00000060718 CCDS778.1<br>cdna:known-ccds chromosome:NCBI36:1:103115583:103346640:-1 gene:ENSG00000060718 CCDS779.1<br>cdna:known-ccds chromosome:NCBI36:1:103115583:103346640:-1 gene:ENSG00000060718 CCDS780.1<br>cdna:known chromosome:NCBI36:1:103115583:103346640:-1 gene:ENSG00000060718<br>Homo sapiens collagen, type XI, alpha 1 (COL11A1), transcript variant B, mRNA.<br><br>Homo sapiens collagen, type XI, alpha 1 (COL11A1), transcript variant A, mRNA.<br>Homo sapiens collagen, type XI, alpha 1 (COL11A1), transcript variant C, mRNA.<br>cdna:known chromosome:NCBI36:1:103115629:103269357:-1 gene:ENSG00000060718<br>cdna:Genscan chromosome:NCBI36:1:103172593:103234186:-1 |
| 2434627 | 73349 | 2434609 | core | 149047325 | 149047414 | 1 | - | ENST00000368980<br>ENST00000271651<br>ENST00000368978<br>NM_000396<br>ENST00000368976<br>X82153<br>S79895<br>ENSESTT00000021274<br>AY429530 | cdna:known-ccds chromosome:NCBI36:1:149035308:149047423:-1 gene:ENSG00000143387 CCDS969.1<br>cdna:known chromosome:NCBI36:1:149035311:149047436:-1 gene:ENSG00000143387<br>cdna:known chromosome:NCBI36:1:149035311:149047436:-1 gene:ENSG00000143387<br>Homo sapiens cathepsin K (pycnodysostosis) (CTSK), mRNA.<br>cdna:known chromosome:NCBI36:1:149035311:149046524:-1 gene:ENSG00000143387<br>H.sapiens mRNA for cathepsin O.<br>OC2=cathepsin O2 [human, spleen, mRNA, 1482 nt].<br><br>Homo sapiens CTSKv_1 mRNA sequence; alternatively spliced. |
| 2438918 | 75785 | 2438892 | core | 155769993 | 155770892 | 1 | - | AF343663<br>ENST00000368189<br>ENST00000368190<br>NM_031281<br>ENST00000361835<br>GENSCAN00000004525<br>ENST00000356953<br>AF343662<br>AL834187 | Homo sapiens immunoglobulin superfamily receptor translocation associated protein 2b (IRTA2) mRNA, complete cds, alternatively spliced.<br>cdna:known chromosome:NCBI36:1:155768378:155788869:-1 gene:ENSG00000143297<br>cdna:known chromosome:NCBI36:1:155760225:155788869:-1 gene:ENSG00000143297<br>Homo sapiens Fc receptor-like 5 (FCRL5), mRNA.<br>cdna:known-ccds chromosome:NCBI36:1:155749791:155788934:-1 gene:ENSG00000143297 CCDS1165.1<br>cdna:Genscan chromosome:NCBI36:1:155752089:155785994:-1<br>cdna:known chromosome:NCBI36:1:155749791:155788934:-1 gene:ENSG00000143297<br>Homo sapiens immunoglobulin superfamily receptor translocation associated protein 2a (IRTA2) mRNA, complete cds, alternatively spliced.<br>Homo sapiens mRNA; cDNA DKFZp667F216 (from clone DKFZp667F216). |
| 2443143 | 78265 | 2443120 | core | 166931445 | 166931473 | 1 | - | ENST00000367817<br>ENST00000271418 | cdna:known-ccds chromosome:NCBI36:1:166931321:166965126:-1 gene:ENSG00000143196 CCDS1275.1<br>cdna:known chromosome:NCBI36:1:166931331:166965052:-1 gene:ENSG00000143196 |

| | | | | | | | | | |
|---|---|---|---|---|---|---|---|---|---|
| | | | | | | | | NM_001937 | Homo sapiens dermatopontin (DPT), mRNA. |
| 2446620 | 80427 | 2446567 | core | 179238372 | 179238434 | 1 | - | ENST00000258301<br>ENST00000362024<br>NM_005819<br>GENSCAN00000036701 | cdna:known-ccds chromosome:NCBI36:1:179208484:179258670:-1 gene:ENSG00000135823 CCDS1341.1<br>cdna:known chromosome:NCBI36:1:179208801:179258669:-1 gene:ENSG00000135823<br>Homo sapiens syntaxin 6 (STX6), mRNA.<br>cdna:Genscan chromosome:NCBI36:1:179187749:179225868:-1 |
| 2450408 | 82687 | 2450345 | core | 198854308 | 198854386 | 1 | - | ENST00000367350<br>ENST00000236917<br>GENSCAN00000051257<br>NM_014875<br>BC098582<br>ENSESTT00000045611<br>ENSESTT00000045610<br>ENSESTT00000045609 | cdna:known chromosome:NCBI36:1:198787251:198856485:-1 gene:ENSG00000118193<br>cdna:novel chromosome:NCBI36:1:198787248:198854474:-1 gene:ENSG00000118193<br>cdna:Genscan chromosome:NCBI36:1:198789139:198902491:-1<br>Homo sapiens kinesin family member 14 (KIF14), mRNA.<br>Homo sapiens kinesin family member 14, mRNA (cDNA clone IMAGE:6470912), partial cds. |
| 2451615 | 83403 | 2451593 | core | 201422392 | 201422454 | 1 | - | ENSESTT00000019594<br>ENST00000367232<br>BC039132<br>NM_001276<br>GENSCAN00000038022<br>ENST00000255409 | <br>cdna:known chromosome:NCBI36:1:201414682:201422500:-1 gene:ENSG00000133048<br>Homo sapiens chitinase 3-like 1 (cartilage glycoprotein-39), mRNA (cDNA clone MGC:17246 IMAGE:4183798), complete cds.<br>Homo sapiens chitinase 3-like 1 (cartilage glycoprotein-39) (CHI3L1), mRNA.<br>cdna:Genscan chromosome:NCBI36:1:201403828:201421669:-1<br>cdna:known-ccds chromosome:NCBI36:1:201414553:201422500:-1 gene:ENSG00000133048 CCDS1435.1 |
| 2453461 | 84541 | 2453370 | core | 206457983 | 206458009 | 1 | - | AY358496<br>ENSESTT00000019919 | Homo sapiens clone DNA35558 PLXNA2 (UNQ209) mRNA, complete cds. |
| 2459095 | 88091 | 2459042 | core | 225324102 | 225324165 | 1 | - | ENSESTT00000009548<br>AK098391<br>ENST00000366762<br>ENST00000366766<br>ENST00000366765<br>ENST00000366769<br>ENST00000348945<br>AB007920<br>ENST00000334218<br>CR933723<br>ENST00000366764<br>ENST00000366763<br>NM_003607<br>GENSCAN00000008118<br>ENSESTT00000009549<br>ENST00000295191<br>ENST00000366767<br>NM_014826 | <br>Homo sapiens cDNA FLJ25525 fis, clone CBR07120, highly similar to Mytonic dystrophy kinase-related Cdc42-binding kinase.<br>cdna:known chromosome:NCBI36:1:225277582:225335230:-1 gene:ENSG00000143776<br>cdna:known chromosome:NCBI36:1:225246676:225572449:-1 gene:ENSG00000143776<br>cdna:known chromosome:NCBI36:1:225246676:225572449:-1 gene:ENSG00000143776<br>cdna:known-ccds chromosome:NCBI36:1:225244189:225572798:-1 gene:ENSG00000143776 CCDS1558.1<br>cdna:known chromosome:NCBI36:1:225246676:225572449:-1 gene:ENSG00000143776<br>Homo sapiens mRNA for KIAA0451 protein, partial cds.<br>cdna:known chromosome:NCBI36:1:225244190:225335303:-1 gene:ENSG00000143776<br>Homo sapiens mRNA; cDNA DKFZp686L1738 (from clone DKFZp686L1738).<br>cdna:known chromosome:NCBI36:1:225246676:225572449:-1 gene:ENSG00000143776<br>cdna:known chromosome:NCBI36:1:225248591:225572038:-1 gene:ENSG00000143776<br>Homo sapiens CDC42 binding protein kinase alpha (DMPK-like) (CDC42BPA), transcript variant B, mRNA.<br>cdna:Genscan chromosome:NCBI36:1:225241856:225383555:-1<br><br>cdna:known chromosome:NCBI36:1:225246676:225572449:-1 gene:ENSG00000143776<br>cdna:known-ccds chromosome:NCBI36:1:225244189:225571506:-1 gene:ENSG00000143776 CCDS1559.1<br>Homo sapiens CDC42 binding protein kinase alpha (DMPK-like) (CDC42BPA), transcript variant A, mRNA. |
| 2479493 | 101023 | 2479433 | core | 43823401 | 43823546 | 2 | + | ENST00000378613<br>NM_172069<br>AL833546<br>ENST00000282406<br>AK126308<br>GENSCAN00000054769<br>ENSESTT00000005903<br>ENSESTT00000005904 | cdna:known-ccds chromosome:NCBI36:2:43717916:43848630:1 gene:ENSG00000152527 CCDS1812.1<br>Homo sapiens pleckstrin homology domain containing, family H (with MyTH4 domain) member 2 (PLEKHH2), mRNA.<br>Homo sapiens mRNA; cDNA DKFZp686K083 (from clone DKFZp686K083).<br>cdna:known chromosome:NCBI36:2:43717951:43848629:1 gene:ENSG00000152527<br>Homo sapiens cDNA FLJ44329 fis, clone TRACH3003379.<br>cdna:Genscan chromosome:NCBI36:2:43777945:43885901:1 |
| 2489144 | 107213 | 2489140 | core | 74215319 | 74215351 | 2 | + | BC004960<br>ENST00000313968 | Homo sapiens hypothetical protein MGC10955, mRNA (cDNA clone MGC:10955 IMAGE:3632495), complete cds. |

| | | | | | | | | | |
|---|---|---|---|---|---|---|---|---|---|
| | | | | | | | | | cdna:known chromosome:NCBI36:2:74215107:74216029:1 gene:ENSG00000177620 |
| 2494507 | 110436 | 2494484 | core | 96388547 | 96388656 | 2 | + | ENSESTT00000043917<br>ENSESTT00000043918<br>NM_015341<br>CR592757<br>GENSCAN00000003203<br>ENST00000240423 | <br><br>Homo sapiens barren homolog 1 (Drosophila) (BRRN1), mRNA.<br>full-length cDNA clone CS0DI074YB24 of Placenta Cot 25-normalized of Homo sapiens (human).<br>cdna:Genscan chromosome:NCBI36:2:96365295:96481903:1<br>cdna:known-ccds chromosome:NCBI36:2:96365211:96405001:1 gene:ENSG00000121152 CCDS2021.1 |
| 2497305 | 112184 | 2497301 | core | 102745113 | 102745225 | 2 | + | NM_144632<br>ENST00000302042<br>GENSCAN00000051755<br>AY358262 | Homo sapiens hypothetical protein FLJ30294 (FLJ30294), mRNA.<br>cdna:known-ccds chromosome:NCBI36:2:102744922:102800310:1 gene:ENSG00000170417 CCDS2064.1<br>cdna:Genscan chromosome:NCBI36:2:102719138:102755122:1<br>Homo sapiens clone DNA179765 RLNI6974 (UNQ6974) mRNA, complete cds. |
| 2500243 | 114018 | 2500165 | core | 111505689 | 111505739 | 2 | + | ENST00000283065<br>ENST00000389810<br>ENST00000389811<br>BC022268<br>ENST00000340561<br>ENSESTT00000019174<br>NM_018308<br>GENSCAN00000046063<br>ENSESTT00000019175 | cdna:known-ccds chromosome:NCBI36:2:111273085:111568392:1 gene:ENSG00000153093 CCDS2088.1<br>cdna:known chromosome:NCBI36:2:111248017:111591849:1 gene:ENSG00000153093<br>cdna:known chromosome:NCBI36:2:111247895:111591849:1 gene:ENSG00000153093<br>Homo sapiens acyl-Coenzyme A oxidase-like, mRNA (cDNA clone IMAGE:4730078), partial cds.<br>cdna:known chromosome:NCBI36:2:111206682:111362061:1 gene:ENSG00000153093<br><br>Homo sapiens acyl-Coenzyme A oxidase-like (ACOXL), mRNA.<br>cdna:Genscan chromosome:NCBI36:2:111339284:111442637:1 |
| 2502853 | 115645 | 2502842 | core | 119910786 | 119910928 | 2 | + | BC046362 | Homo sapiens transmembrane protein 37, mRNA (cDNA clone MGC:50757 IMAGE:5221396), complete cds. |
| 2516011 | 124114 | 2515933 | core | 173831701 | 173831776 | 2 | + | AB208974<br>ENSESTT00000013608<br>ENSESTT00000054532<br>CR598198<br>NM_016653<br>ENSESTT00000054534<br>GENSCAN00000004794<br>ENST00000375213<br>NM_133646<br>ENST00000338983<br>ENSESTT00000054531<br>GENSCAN00000036477 | Homo sapiens mRNA for Plaucible mixed-lineage kinase protein variant protein.<br><br><br>full-length cDNA clone CS0DJ010YE16 of T cells (Jurkat cell line) Cot 10-normalized of Homo sapiens (human).<br>Homo sapiens sterile alpha motif and leucine zipper containing kinase AZK (ZAK), transcript variant 1, mRNA.<br><br>cdna:Genscan chromosome:NCBI36:2:173734756:173824155:1<br>cdna:known chromosome:NCBI36:2:173648816:173840986:1 gene:ENSG00000091436<br>Homo sapiens sterile alpha motif and leucine zipper containing kinase AZK (ZAK), transcript variant 2, mRNA.<br>cdna:known-ccds chromosome:NCBI36:2:173648811:173800117:1 gene:ENSG00000091436 CCDS2251.1<br><br>cdna:Genscan chromosome:NCBI36:2:173831673:173839724:1 |
| 2531590 | 133765 | 2531589 | core | 231437873 | 231437907 | 2 | + | NM_030926<br>ENST00000326407<br>NM_001012514<br>ENST00000335005<br>NM_001012516<br>ENST00000326427<br>ENSESTT00000043428<br>GENSCAN00000048898 | Homo sapiens integral membrane protein 2C (ITM2C), transcript variant 1, mRNA.<br>cdna:known chromosome:NCBI36:2:231437865:231452206:1 gene:ENSG00000135916<br>Homo sapiens integral membrane protein 2C (ITM2C), transcript variant 3, mRNA.<br>cdna:known chromosome:NCBI36:2:231437865:231452206:1 gene:ENSG00000135916<br>Homo sapiens integral membrane protein 2C (ITM2C), transcript variant 2, mRNA.<br>cdna:known-ccds chromosome:NCBI36:2:231437865:231452206:1 gene:ENSG00000135916 CCDS2479.1<br><br>cdna:Genscan chromosome:NCBI36:2:231437985:231451058:1 |
| 2532483 | 134340 | 2532480 | core | 233206663 | 233206922 | 2 | + | NM_025202<br>BC035476<br>ENSESTT00000030828<br>GENSCAN00000033847<br>ENST00000264059<br>GENSCAN00000029857<br>CR627385<br>AF193044<br>ENSESTT00000030830 | Homo sapiens EF-hand domain family, member D1 (EFHD1), mRNA.<br>Homo sapiens EF-hand domain family, member D1, mRNA (cDNA clone IMAGE:5192690).<br><br>cdna:Genscan chromosome:NCBI36:2:233206659:233214956:1<br>cdna:known-ccds chromosome:NCBI36:2:233206182:233255703:1 gene:ENSG00000115468 CCDS2497.1<br>cdna:Genscan chromosome:NCBI36:2:233235756:233254673:1<br>Homo sapiens mRNA; cDNA DKFZp781H0842 (from clone DKFZp781H0842).<br>Homo sapiens PP1187 mRNA, complete cds. |
| 2535846 | 136492 | 2535830 | core | 241163161 | 241163228 | 2 | + | AK130805<br>ENSESTT00000022995 | Homo sapiens cDNA FLJ27295 fis, clone TMS03537, highly similar to Homo sapiens arginyl aminopeptidase (aminopeptidase B)-like 1 (RNPEPL1). |

| | | | | | | | | | |
|---|---|---|---|---|---|---|---|---|---|
| | | | | | | | | ENST00000270357<br>GENSCAN00000039199<br>ENSESTT00000022994<br>NM_018226<br>AL512754<br>ENSESTT00000022996 | cdna:known-ccds chromosome:NCBI36:2:241156777:241166814:1 gene:ENSG00000142327 CCDS2537.1<br>cdna:Genscan chromosome:NCBI36:2:241156677:241165982:1<br>Homo sapiens arginyl aminopeptidase (aminopeptidase B)-like 1 (RNPEPL1), mRNA.<br>Homo sapiens mRNA; cDNA DKFZp667K156 (from clone DKFZp667K156). |
| 2549569 | 145127 | 2549565 | core | 40195937 | 40195967 | 2 | - | NM_021097<br>BX648299<br>ENST00000378715<br>ENST00000346579<br>ENST00000356676<br>ENST00000332839<br>ENST00000340668<br>GENSCAN00000044339<br>ENSESTT00000014704<br>GENSCAN00000004423 | Homo sapiens solute carrier family 8 (sodium/calcium exchanger), member 1 (SLC8A1), mRNA.<br>Homo sapiens mRNA; cDNA DKFZp686J04125 (from clone DKFZp686J04125).<br>cdna:known-ccds chromosome:NCBI36:2:40192790:40534188:-1 gene:ENSG00000183023 CCDS1806.1<br>cdna:known chromosome:NCBI36:2:40195897:40510924:-1 gene:ENSG00000183023<br>cdna:known chromosome:NCBI36:2:40195897:40510924:-1 gene:ENSG00000183023<br>cdna:known chromosome:NCBI36:2:40195897:40510924:-1 gene:ENSG00000183023<br>cdna:known chromosome:NCBI36:2:40195897:40510924:-1 gene:ENSG00000183023<br>cdna:Genscan chromosome:NCBI36:2:40206577:40262753:-1<br>cdna:Genscan chromosome:NCBI36:2:40506350:40511917:-1 |
| 2559646 | 151546 | 2559637 | core | 73781910 | 73781936 | 2 | - | NM_016347<br>GENSCAN00000000304 | Homo sapiens putative N-acetyltransferase Camello 2 (CML2), mRNA.<br>cdna:Genscan chromosome:NCBI36:2:73781437:73785156:-1 |
| 2560100 | 151821 | 2560076 | core | 74511274 | 74511301 | 2 | - | ENSESTT00000037318<br>ENSESTT00000037315<br>ENSESTT00000037317<br>ENSESTT00000037314<br>ENST00000233330<br>ENST00000272430<br>NM_033046<br>NM_001015056<br>GENSCAN00000039288<br>NM_001015055<br>ENST00000305557 | cdna:known chromosome:NCBI36:2:74506497:74521218:-1 gene:ENSG00000114993<br>cdna:known chromosome:NCBI36:2:74506497:74522568:-1 gene:ENSG00000114993<br>Homo sapiens rhotekin (RTKN), transcript variant 2, mRNA.<br>Homo sapiens rhotekin (RTKN), transcript variant 3, mRNA.<br>cdna:Genscan chromosome:NCBI36:2:74506834:74522451:-1<br>Homo sapiens rhotekin (RTKN), transcript variant 1, mRNA.<br>cdna:known-ccds chromosome:NCBI36:2:74506497:74521218:-1 gene:ENSG00000114993 CCDS1941.1 |
| 2566934 | 156030 | 2566848 | core | 99709971 | 99709999 | 2 | - | AK092327<br>NM_002285<br>NM_001025108<br>ENST00000356421<br>ENST00000317233<br>ENSESTT00000045865<br>ENSESTT00000045864<br>ENSESTT00000045866<br>ENSESTT00000008775<br>GENSCAN00000053837 | Homo sapiens cDNA FLJ35008 fis, clone OCBBF2012191, highly similar to LAF-4 PROTEIN.<br>Homo sapiens AF4/FMR2 family, member 3 (AFF3), transcript variant 1, mRNA.<br>Homo sapiens AF4/FMR2 family, member 3 (AFF3), transcript variant 2, mRNA.<br>cdna:known chromosome:NCBI36:2:99530150:100088477:-1 gene:ENSG00000144218<br>cdna:known chromosome:NCBI36:2:99576283:100088477:-1 gene:ENSG00000144218<br>cdna:Genscan chromosome:NCBI36:2:99984727:99995254:-1 |
| 2566938 | 156034 | 2566848 | core | 99727003 | 99727027 | 2 | - | ENSESTT00000045864<br>ENSESTT00000045865<br>AK092327<br>NM_002285<br>ENST00000356421<br>NM_001025108<br>ENST00000317233 | Homo sapiens cDNA FLJ35008 fis, clone OCBBF2012191, highly similar to LAF-4 PROTEIN.<br>Homo sapiens AF4/FMR2 family, member 3 (AFF3), transcript variant 1, mRNA.<br>cdna:known chromosome:NCBI36:2:99530150:100088477:-1 gene:ENSG00000144218<br>Homo sapiens AF4/FMR2 family, member 3 (AFF3), transcript variant 2, mRNA.<br>cdna:known chromosome:NCBI36:2:99576283:100088477:-1 gene:ENSG00000144218 |
| 2570194 | 158069 | 2570193 | core | 110198762 | 110199020 | 2 | - | NM_005434<br>ENST00000272462 | Homo sapiens mal, T-cell differentiation protein-like (MALL), mRNA.<br>cdna:known-ccds chromosome:NCBI36:2:110198738:110231432:-1 gene:ENSG00000144063 CCDS2085.1 |
| 2570630 | 158337 | 2570616 | core | 111112034 | 111112168 | 2 | - | NM_004336<br>ENST00000389944<br>ENST00000302759 | Homo sapiens BUB1 budding uninhibited by benzimidazoles 1 homolog (yeast) (BUB1), mRNA.<br>cdna:known chromosome:NCBI36:2:111112013:111152043:-1 gene:ENSG00000169679<br>cdna:known chromosome:NCBI36:2:111111883:111152135:-1 gene:ENSG00000169679 |

| | | | | | | | | | |
|---|---|---|---|---|---|---|---|---|---|
| | | | | | | | | ENST00000389945<br>GENSCAN00000013627<br>ENSESTT00000019182<br>AK023540 | cdna:known chromosome:NCBI36:2:111112013:111152043:-1 gene:ENSG00000169679<br>cdna:Genscan chromosome:NCBI36:2:111112013:111194776:-1<br><br>Homo sapiens cDNA FLJ13478 fis, clone PLACE1003709, highly similar to Homo sapiens mitotic checkpoint kinase Bub1 (BUB1) mRNA. |
| 2570676 | 158364 | 2570616 | core | 111148156 | 111148237 | 2 | - | NM_004336<br>ENST00000302759<br>GENSCAN00000013627<br>ENST00000389945<br>ENST00000389944<br>ENSESTT00000019182<br>AK023540 | Homo sapiens BUB1 budding uninhibited by benzimidazoles 1 homolog (yeast) (BUB1), mRNA.<br>cdna:known chromosome:NCBI36:2:111111883:111152135:-1 gene:ENSG00000169679<br>cdna:Genscan chromosome:NCBI36:2:111112013:111194776:-1<br>cdna:known chromosome:NCBI36:2:111112013:111152043:-1 gene:ENSG00000169679<br>cdna:known chromosome:NCBI36:2:111112013:111152043:-1 gene:ENSG00000169679<br><br>Homo sapiens cDNA FLJ13478 fis, clone PLACE1003709, highly similar to Homo sapiens mitotic checkpoint kinase Bub1 (BUB1) mRNA. |
| 2570677 | 158365 | 2570616 | core | 111148362 | 111148392 | 2 | - | ENST00000389944<br>ENST00000389945<br>GENSCAN00000013627<br>NM_004336<br>ENST00000302759<br>ENSESTT00000019182<br>AK023540 | cdna:known chromosome:NCBI36:2:111112013:111152043:-1 gene:ENSG00000169679<br>cdna:known chromosome:NCBI36:2:111112013:111152043:-1 gene:ENSG00000169679<br>cdna:Genscan chromosome:NCBI36:2:111112013:111194776:-1<br>Homo sapiens BUB1 budding uninhibited by benzimidazoles 1 homolog (yeast) (BUB1), mRNA.<br>cdna:known chromosome:NCBI36:2:111111883:111152135:-1 gene:ENSG00000169679<br><br>Homo sapiens cDNA FLJ13478 fis, clone PLACE1003709, highly similar to Homo sapiens mitotic checkpoint kinase Bub1 (BUB1) mRNA. |
| 2575021 | 161036 | 2574984 | core | 128128876 | 128128940 | 2 | - | AF527767<br>AK123014<br>ENST00000324864<br>AF527769<br>AF527768<br>NM_017980<br>AK055363<br>AF527766<br>AF527770<br>AK095790<br>AK094954<br>ENSESTT00000037034<br>ENST00000324938<br>GENSCAN00000038207<br>ENST00000342067<br>ENST00000355119<br>AK091652<br>CR592921<br>ENSESTT00000037036<br>ENSESTT00000037035<br>GENSCAN00000008986<br>ENSESTT00000037033 | Homo sapiens tissue-type uterus LIM-like protein 2D mRNA, complete cds.<br>Homo sapiens cDNA FLJ16826 fis, clone UTERU3006687, highly similar to Homo sapiens LIM and senescent cell antigen-like domains 1 (LIMS1).<br>cdna:known chromosome:NCBI36:2:128112473:128132267:-1 gene:ENSG00000072163<br>Homo sapiens tissue-type brain LIM-like protein 2F mRNA, complete cds.<br>Homo sapiens tissue-type brain LIM-like protein 2E mRNA, complete cds.<br>Homo sapiens LIM and senescent cell antigen-like domains 2 (LIMS2), mRNA.<br>Homo sapiens cDNA FLJ30801 fis, clone FEBRA2001217, highly similar to PINCH PROTEIN.<br>Homo sapiens tissue-type spleen LIM-like protein 2C mRNA, complete cds.<br>Homo sapiens tissue-type brain LIM-like protein 2G mRNA, complete cds.<br>Homo sapiens cDNA FLJ38471 fis, clone FEBRA2022055, highly similar to PINCH PROTEIN.<br>Homo sapiens cDNA FLJ37635 fis, clone BRCOC2017856, highly similar to PINCH PROTEIN.<br><br>cdna:known-ccds chromosome:NCBI36:2:128112475:128138590:-1 gene:ENSG00000072163 CCDS2147.1<br>cdna:Genscan chromosome:NCBI36:2:128113326:128119675:-1<br>cdna:known chromosome:NCBI36:2:128112475:128118157:-1 gene:ENSG00000072163<br>cdna:known chromosome:NCBI36:2:128113093:128155507:-1 gene:ENSG00000072163<br>Homo sapiens cDNA FLJ34333 fis, clone FEBRA2009276, highly similar to PINCH PROTEIN.<br>full-length cDNA clone CS0DJ014YK07 of T cells (Jurkat cell line) Cot 10-normalized of Homo sapiens (human).<br><br><br>cdna:Genscan chromosome:NCBI36:2:128127128:128155062:-1 |
| 2575027 | 161037 | 2574984 | core | 128131488 | 128131602 | 2 | - | AF527767<br>AK091652<br>ENST00000324864<br>AF527769<br>AF527768<br>ENSESTT00000037034<br>AK055363<br>ENST00000324938<br>GENSCAN00000038207 | Homo sapiens tissue-type uterus LIM-like protein 2D mRNA, complete cds.<br>Homo sapiens cDNA FLJ34333 fis, clone FEBRA2009276, highly similar to PINCH PROTEIN.<br>cdna:known chromosome:NCBI36:2:128112473:128132267:-1 gene:ENSG00000072163<br>Homo sapiens tissue-type brain LIM-like protein 2F mRNA, complete cds.<br>Homo sapiens tissue-type brain LIM-like protein 2E mRNA, complete cds.<br><br>Homo sapiens cDNA FLJ30801 fis, clone FEBRA2001217, highly similar to PINCH PROTEIN.<br>cdna:known-ccds chromosome:NCBI36:2:128112475:128138590:-1 gene:ENSG00000072163 CCDS2147.1<br>cdna:Genscan chromosome:NCBI36:2:128113326:128119675:-1 |

| | | | | | | | | | |
|---|---|---|---|---|---|---|---|---|---|
| | | | | | | | | ENST00000342067<br>ENST00000355119<br>AF527766<br>NM_017980<br>AF527770<br>AK095790<br>CR592921<br>AK094954<br>ENSESTT00000037036<br>ENSESTT00000037035<br>GENSCAN00000008986<br>ENSESTT00000037033 | cdna:known chromosome:NCBI36:2:128112475:128118157:-1 gene:ENSG00000072163<br>cdna:known chromosome:NCBI36:2:128113093:128155507:-1 gene:ENSG00000072163<br>Homo sapiens tissue-type spleen LIM-like protein 2C mRNA, complete cds.<br>Homo sapiens LIM and senescent cell antigen-like domains 2 (LIMS2), mRNA.<br>Homo sapiens tissue-type brain LIM-like protein 2G mRNA, complete cds.<br>Homo sapiens cDNA FLJ38471 fis, clone FEBRA2022055, highly similar to PINCH PROTEIN.<br>full-length cDNA clone CS0DJ014YK07 of T cells (Jurkat cell line) Cot 10-normalized of Homo sapiens (human).<br>Homo sapiens cDNA FLJ37635 fis, clone BRCOC2017856, highly similar to PINCH PROTEIN.<br><br><br>cdna:Genscan chromosome:NCBI36:2:128127128:128155062:-1 |
| 2575036 | 161043 | 2574984 | core | 128147951 | 128148161 | 2 | - | AK055363<br>ENSESTT00000037037<br>ENSESTT00000037036<br>AK095790<br>ENSESTT00000037034<br>AK091652<br>AK123014<br>AF527766<br>GENSCAN00000008986<br>ENST00000324938<br>ENSESTT00000037035<br>NM_017980<br>AK094954<br>ENST00000355119<br>ENSESTT00000037033 | Homo sapiens cDNA FLJ30801 fis, clone FEBRA2001217, highly similar to PINCH PROTEIN.<br><br><br>Homo sapiens cDNA FLJ38471 fis, clone FEBRA2022055, highly similar to PINCH PROTEIN.<br><br>Homo sapiens cDNA FLJ34333 fis, clone FEBRA2009276, highly similar to PINCH PROTEIN.<br>Homo sapiens cDNA FLJ16826 fis, clone UTERU3006687, highly similar to Homo sapiens LIM and senescent cell antigen-like domains 1 (LIMS1).<br>Homo sapiens tissue-type spleen LIM-like protein 2C mRNA, complete cds.<br>cdna:Genscan chromosome:NCBI36:2:128127128:128155062:-1<br>cdna:known-ccds chromosome:NCBI36:2:128112475:128138590:-1 gene:ENSG00000072163 CCDS2147.1<br><br>Homo sapiens LIM and senescent cell antigen-like domains 2 (LIMS2), mRNA.<br>Homo sapiens cDNA FLJ37635 fis, clone BRCOC2017856, highly similar to PINCH PROTEIN.<br>cdna:known chromosome:NCBI36:2:128113093:128155507:-1 gene:ENSG00000072163 |
| 2583008 | 166066 | 2582979 | core | 159847700 | 159847791 | 2 | - | CR607851<br>ENSESTT00000054678<br>ENST00000359774<br>NM_152528<br>ENST00000358147<br>BC029520 | full-length cDNA clone CS0DN004YJ15 of Adult brain of Homo sapiens (human).<br><br>cdna:known-ccds chromosome:NCBI36:2:159800618:159851482:-1 gene:ENSG00000196151 CCDS2208.1<br>Homo sapiens WD repeat, sterile alpha motif and U-box domain containing 1 (WDSUB1), mRNA.<br>cdna:known chromosome:NCBI36:2:159800558:159851460:-1 gene:ENSG00000196151<br>Homo sapiens WD repeat, sterile alpha motif and U-box domain containing 1, mRNA (cDNA clone MGC:33855 IMAGE:5301559), complete cds. |
| 2584139 | 166744 | 2584134 | core | 162738479 | 162738513 | 2 | - | NM_004460<br>ENSESTT00000050863<br>AL832166<br>ENSESTT00000050861<br>ENST00000188790<br>AK055327<br>GENSCAN00000033018<br>ENSESTT00000050859<br>ENSESTT00000050860 | Homo sapiens fibroblast activation protein, alpha (FAP), mRNA.<br><br>Homo sapiens mRNA; cDNA DKFZp686G13158 (from clone DKFZp686G13158).<br><br>cdna:known chromosome:NCBI36:2:162735446:162808291:-1 gene:ENSG00000078098<br>Homo sapiens cDNA FLJ30765 fis, clone FEBRA2000659, highly similar to Human fibroblast activation protein mRNA.<br>cdna:Genscan chromosome:NCBI36:2:162692879:162846301:-1 |
| 2584166 | 166766 | 2584134 | core | 162765334 | 162765361 | 2 | - | ENSESTT00000050861<br>AL832166<br>AK055327<br>ENSESTT00000050863<br>NM_004460<br>ENST00000188790<br>GENSCAN00000033018 | <br>Homo sapiens mRNA; cDNA DKFZp686G13158 (from clone DKFZp686G13158).<br>Homo sapiens cDNA FLJ30765 fis, clone FEBRA2000659, highly similar to Human fibroblast activation protein mRNA.<br><br>Homo sapiens fibroblast activation protein, alpha (FAP), mRNA.<br>cdna:known chromosome:NCBI36:2:162735446:162808291:-1 gene:ENSG00000078098<br>cdna:Genscan chromosome:NCBI36:2:162692879:162846301:-1 |

| | | | | | | | | | |
|---|---|---|---|---|---|---|---|---|---|
| 2584187 | 166780 | 2584134 | core | 162790242 | 162790321 | 2 | - | ENSESTT00000050860<br>ENSESTT00000050859<br>NM_004460<br>GENSCAN00000033018<br>ENST00000188790 | <br><br>Homo sapiens fibroblast activation protein, alpha (FAP), mRNA.<br>cdna:Genscan chromosome:NCBI36:2:162692879:162846301:-1<br>cdna:known chromosome:NCBI36:2:162735446:162808291:-1 gene:ENSG00000078098 |
| 2585429 | 167616 | 2585400 | core | 166846440 | 166846564 | 2 | - | NM_002977<br>GENSCAN00000052247<br>ENST00000303354<br>ENST00000375387<br>GENSCAN00000062512<br>AY682086 | Homo sapiens sodium channel, voltage-gated, type IX, alpha (SCN9A), mRNA.<br>cdna:Genscan chromosome:NCBI36:2:166763428:166807412:-1<br>cdna:known chromosome:NCBI36:2:166763060:166876560:-1 gene:ENSG00000169432<br>cdna:known chromosome:NCBI36:2:166763060:166876560:-1 gene:ENSG00000169432<br>cdna:Genscan chromosome:NCBI36:2:166816521:166892878:-1<br>Homo sapiens voltage-gated sodium channel Nav1.7 (SCN9A) mRNA, partial cds, alternatively spliced. |
| 2595450 | 174094 | 2595443 | core | 203453583 | 203453727 | 2 | - | NM_018256<br>ENST00000261015<br>ENSESTT00000001877<br>GENSCAN00000032179 | Homo sapiens WD repeat domain 12 (WDR12), mRNA.<br>cdna:known-ccds chromosome:NCBI36:2:203453575:203485194:-1 gene:ENSG00000138442 CCDS2356.1<br><br>cdna:Genscan chromosome:NCBI36:2:203453828:203485586:-1 |
| 2602669 | 178753 | 2602653 | core | 229598610 | 229598969 | 2 | - | NM_017933<br>AK000708<br>BC040164<br>GENSCAN00000053188<br>ENST00000354069<br>ENST00000343451<br>ENSESTT00000019715<br>GENSCAN00000064509 | Homo sapiens hypothetical protein FLJ20701 (FLJ20701), mRNA.<br>Homo sapiens cDNA FLJ20701 fis, clone KAIA2204.<br>Homo sapiens hypothetical protein FLJ20701, mRNA (cDNA clone MGC:48969 IMAGE:5589172), complete cds.<br>cdna:Genscan chromosome:NCBI36:2:229598592:229600932:-1<br>cdna:known chromosome:NCBI36:2:229596955:229805014:-1 gene:ENSG00000153823<br>cdna:known-ccds chromosome:NCBI36:2:229596955:229835765:-1 gene:ENSG00000153823 CCDS2471.1<br><br>cdna:Genscan chromosome:NCBI36:2:229721109:229752241:-1 |
| 2604261 | 179732 | 2604254 | core | 234411011 | 234411037 | 2 | - | ENSESTT00000035628<br>ENST00000373395<br>ENST00000243201<br>NM_018410<br>ENSESTT00000035627 | <br>cdna:known chromosome:NCBI36:2:234410225:234427951:-1 gene:ENSG00000123485<br>cdna:known chromosome:NCBI36:2:234410746:234427917:-1 gene:ENSG00000123485<br>Homo sapiens hypothetical protein DKFZp762E1312 (DKFZp762E1312), mRNA. |
| 2605035 | 180201 | 2604998 | core | 236911592 | 236911716 | 2 | - | ENSESTT00000005745<br>ENSESTT00000005746<br>ENST00000254653<br>GENSCAN00000047931<br>NM_024726<br>ENST00000309507 | <br><br>cdna:known chromosome:NCBI36:2:236897535:237080914:-1 gene:ENSG00000132321<br>cdna:Genscan chromosome:NCBI36:2:236850165:236919104:-1<br>Homo sapiens IQ motif containing with AAA domain (IQCA), mRNA.<br>cdna:known chromosome:NCBI36:2:236897535:237080914:-1 gene:ENSG00000132321 |
| 2605390 | 180448 | 2605321 | core | 237961040 | 237961308 | 2 | - | ENST00000346358<br>NM_057166<br>ENSESTT00000018429<br>ENST00000347401<br>BX641155<br>NM_057165<br>NM_057167<br>ENST00000353578<br>NM_004369<br>ENST00000295550<br>NM_057164<br>ENSESTT00000018426<br>ENSESTT00000018425<br>GENSCAN00000024616<br>BX647500 | cdna:known chromosome:NCBI36:2:237897401:237987559:-1 gene:ENSG00000163359<br>Homo sapiens collagen, type VI, alpha 3 (COL6A3), transcript variant 4, mRNA.<br><br>cdna:known chromosome:NCBI36:2:237897401:237987559:-1 gene:ENSG00000163359<br>Homo sapiens mRNA; cDNA DKFZp686D23123 (from clone DKFZp686D23123).<br>Homo sapiens collagen, type VI, alpha 3 (COL6A3), transcript variant 3, mRNA.<br>Homo sapiens collagen, type VI, alpha 3 (COL6A3), transcript variant 5, mRNA.<br>cdna:known chromosome:NCBI36:2:237897401:237987559:-1 gene:ENSG00000163359<br>Homo sapiens collagen, type VI, alpha 3 (COL6A3), transcript variant 1, mRNA.<br>cdna:known chromosome:NCBI36:2:237897401:237987559:-1 gene:ENSG00000163359<br>Homo sapiens collagen, type VI, alpha 3 (COL6A3), transcript variant 2, mRNA.<br><br><br>cdna:Genscan chromosome:NCBI36:2:237914246:238019586:-1<br>Homo sapiens mRNA; cDNA DKFZp686K04147 (from clone DKFZp686K04147). |
| 2611945 | 184599 | 2611848 | core | 14501401 | 14501492 | 3 | + | NM_003043<br>U16120 | Homo sapiens solute carrier family 6 (neurotransmitter transporter, taurine), member 6 (SLC6A6), mRNA.<br>Human placental taurine transporter mRNA, complete cds. |

| | | | | | | | | | |
|---|---|---|---|---|---|---|---|---|---|
| | | | | | | | | ENST00000253707<br>ENST00000388984<br>BC111489<br>ENSESTT00000045726<br>ENST00000360861<br>ENST00000388983<br>ENSESTT00000045727<br>ENSESTT00000045728 | cdna:known chromosome:NCBI36:3:14419110:14503971:1 gene:ENSG00000131389<br>cdna:known chromosome:NCBI36:3:14460147:14501519:1 gene:ENSG00000131389<br>Homo sapiens solute carrier family 6 (neurotransmitter transporter, taurine), member 6, mRNA (cDNA clone IMAGE:5755891), complete cds.<br><br>cdna:known chromosome:NCBI36:3:14419154:14505859:1 gene:ENSG00000131389<br>cdna:known chromosome:NCBI36:3:14460147:14501519:1 gene:ENSG00000131389 |
| 2625820 | 193086 | 2625793 | core | 57807965 | 57808000 | 3 | + | ENSESTT00000002086<br>NM_007159<br>AK124200<br>AY358410<br>ENSESTT00000002084<br>ENSESTT00000002085<br>ENST00000383719<br>ENST00000295952<br>ENST00000383718 | <br>Homo sapiens sarcolemma associated protein (SLMAP), mRNA.<br>Homo sapiens cDNA FLJ42206 fis, clone THYMU2035735, highly similar to Oryctolagus cuniculus sarcolemmal associated protein-3 mRNA.<br>Homo sapiens clone DNA53991 SLAP (UNQ1847) mRNA, complete cds.<br><br><br>cdna:known chromosome:NCBI36:3:57718068:57851973:1 gene:ENSG00000163681<br>cdna:known chromosome:NCBI36:3:57718214:57889934:1 gene:ENSG00000163681<br>cdna:known chromosome:NCBI36:3:57718321:57858460:1 gene:ENSG00000163681 |
| 2625821 | 193087 | 2625793 | core | 57810522 | 57810567 | 3 | + | NM_007159<br>ENSESTT00000002083<br>ENST00000295952<br>AK124200<br>AY358410<br>ENSESTT00000002084<br>ENSESTT00000002085<br>ENST00000383719<br>ENST00000383718<br>ENSESTT00000002086<br>GENSCAN00000032566<br>AF304450<br>ENST00000383717<br>CR627321<br>AF100750<br>ENSESTT00000038019<br>ENSESTT00000038020<br>ENST00000295951<br>AK022561<br>GENSCAN00000032567 | Homo sapiens sarcolemma associated protein (SLMAP), mRNA.<br><br>cdna:known chromosome:NCBI36:3:57718214:57889934:1 gene:ENSG00000163681<br>Homo sapiens cDNA FLJ42206 fis, clone THYMU2035735, highly similar to Oryctolagus cuniculus sarcolemmal associated protein-3 mRNA.<br>Homo sapiens clone DNA53991 SLAP (UNQ1847) mRNA, complete cds.<br><br><br>cdna:known chromosome:NCBI36:3:57718068:57851973:1 gene:ENSG00000163681<br>cdna:known chromosome:NCBI36:3:57718321:57858460:1 gene:ENSG00000163681<br><br>cdna:Genscan chromosome:NCBI36:3:57818521:57869943:1<br>Homo sapiens sarcolemmal associated protein 1 mRNA, complete cds, alternatively spliced.<br>cdna:known chromosome:NCBI36:3:57850808:57888160:1 gene:ENSG00000163681<br>Homo sapiens mRNA; cDNA DKFZp779I1058 (from clone DKFZp779I1058).<br>Homo sapiens SLAP-2 homolog mRNA, complete cds.<br><br><br>cdna:known chromosome:NCBI36:3:57825353:57888326:1 gene:ENSG00000163681<br>Homo sapiens cDNA FLJ12499 fis, clone NT2RM2001671, highly similar to Oryctolagus cuniculus sarcolemmal associated protein (SLAP1) mRNA.<br>cdna:Genscan chromosome:NCBI36:3:57873114:57888155:1 |
| 2635193 | 199046 | 2635184 | core | 109553325 | 109553350 | 3 | + | NM_007072<br>ENSESTT00000001698<br>ENSESTT00000001700<br>ENSESTT00000001701<br>AK000692<br>ENSESTT00000001699<br>ENST00000357759<br>GENSCAN00000067102<br>AK027132<br>GENSCAN00000017540 | Homo sapiens HERV-H LTR-associating 2 (HHLA2), mRNA.<br><br><br><br>Homo sapiens cDNA FLJ20685 fis, clone KAIA3109.<br><br>cdna:known chromosome:NCBI36:3:109553351:109578094:1 gene:ENSG00000114455<br>cdna:Genscan chromosome:NCBI36:3:109547159:109559976:1<br>Homo sapiens cDNA: FLJ23479 fis, clone KAIA02239, highly similar to AF126162 Homo sapiens HERV-H LTR associated protein 2 (HHLA2) mRNA.<br>cdna:Genscan chromosome:NCBI36:3:109563879:109578094:1 |
| 2652528 | 209979 | 2652410 | core | 173538232 | 173538541 | 3 | + | AY358367 | Homo sapiens clone DNA77645 YVTM2421 (UNQ2421) mRNA, complete cds. |

| | | | | | | | | | |
|---|---|---|---|---|---|---|---|---|---|
| 2652679 | 210067 | 2652675 | core | 173952634 | 173952663 | 3 | + | | |
| 2652714 | 210094 | 2652675 | core | 174016094 | 174016160 | 3 | + | NM_018098<br>ENSESTT00000046493<br>ENSESTT00000046494<br>ENSESTT00000046495<br>ENSESTT00000046496<br>ENST00000232458<br>DQ847274<br>GENSCAN00000044983<br>ENSESTT00000046497<br>ENSESTT00000046498<br>ENSESTT00000046499<br>AL137710<br>ENSESTT00000046500 | Homo sapiens epithelial cell transforming sequence 2 oncogene (ECT2), mRNA.<br>cdna:known-ccds chromosome:NCBI36:3:173951207:174021957:1 gene:ENSG00000114346 CCDS3220.1<br>Homo sapiens epithelial cell transforming sequence 2 oncogene protein splice variant b (ECT2) mRNA, complete cds, alternatively spliced.<br>cdna:Genscan chromosome:NCBI36:3:173955784:174019253:1<br>Homo sapiens mRNA; cDNA DKFZp434C0523 (from clone DKFZp434C0523); partial cds. |
| 2652721 | 210098 | 2652675 | core | 174020676 | 174020715 | 3 | + | NM_018098<br>DQ847274<br>ENSESTT00000046500<br>ENSESTT00000046493<br>ENSESTT00000046494<br>ENSESTT00000046495<br>ENSESTT00000046496<br>ENST00000232458<br>GENSCAN00000044983<br>ENSESTT00000046497<br>ENSESTT00000046498<br>ENSESTT00000046499<br>AL137710 | Homo sapiens epithelial cell transforming sequence 2 oncogene (ECT2), mRNA.<br>Homo sapiens epithelial cell transforming sequence 2 oncogene protein splice variant b (ECT2) mRNA, complete cds, alternatively spliced.<br>cdna:known-ccds chromosome:NCBI36:3:173951207:174021957:1 gene:ENSG00000114346 CCDS3220.1<br>cdna:Genscan chromosome:NCBI36:3:173955784:174019253:1<br>Homo sapiens mRNA; cDNA DKFZp434C0523 (from clone DKFZp434C0523); partial cds. |
| 2669508 | 220716 | 2669488 | core | 38026925 | 38027035 | 3 | - | NM_006225<br>GENSCAN00000063745<br>BX647927<br>BC056157<br>ENST00000334661<br>ENST00000383758<br>ENSESTT00000031808<br>ENSESTT00000031809<br>ENSESTT00000031810 | Homo sapiens phospholipase C, delta 1 (PLCD1), mRNA.<br>cdna:Genscan chromosome:NCBI36:3:38024268:38040928:-1<br>Homo sapiens mRNA; cDNA DKFZp686K101 (from clone DKFZp686K101).<br>Homo sapiens cDNA clone IMAGE:6188165, **** WARNING: chimeric clone ****.<br>cdna:known-ccds chromosome:NCBI36:3:38023993:38046137:-1 gene:ENSG00000187091 CCDS2671.1<br>cdna:known chromosome:NCBI36:3:38023993:38041071:-1 gene:ENSG00000187091 |
| 2671165 | 221714 | 2671101 | core | 43596931 | 43596959 | 3 | - | ENSESTT00000030528<br>NM_018075<br>AK001237<br>ENSESTT00000030527<br>ENST00000350459<br>ENST00000292246<br>GENSCAN00000040656 | Homo sapiens transmembrane protein 16K (TMEM16K), mRNA.<br>Homo sapiens cDNA FLJ10375 fis, clone NT2RM2001950.<br>cdna:known-ccds chromosome:NCBI36:3:43382834:43638564:-1 gene:ENSG00000160746 CCDS2710.1<br>cdna:known chromosome:NCBI36:3:43382834:43638564:-1 gene:ENSG00000160746<br>cdna:Genscan chromosome:NCBI36:3:43593177:43647687:-1 |
| 2676011 | 224522 | 2676009 | core | 52237759 | 52237969 | 3 | - | NM_007284<br>ENST00000305533<br>ENSESTT00000004508<br>AF246973<br>ENST00000310209<br>GENSCAN00000007343 | Homo sapiens PTK9L protein tyrosine kinase 9-like (A6-related protein) (PTK9L), mRNA.<br>cdna:known-ccds chromosome:NCBI36:3:52237670:52248223:-1 gene:ENSG00000173366 CCDS2849.1<br>Homo sapiens toll-like receptor 9 (TLR9) mRNA, partial cds, alternatively spliced.<br>cdna:known chromosome:NCBI36:3:52230273:52238999:-1 gene:ENSG00000173366<br>cdna:Genscan chromosome:NCBI36:3:52238090:52241178:-1 |
| 2678303 | 226007 | 2678298 | core | 58154156 | 58154197 | 3 | - | NM_004944 | Homo sapiens deoxyribonuclease I-like 3 (DNASE1L3), mRNA. |

| | | | | | | | | | |
|---|---|---|---|---|---|---|---|---|---|
| | | | | | | | | AB209613<br>AK097120<br>AK131323<br>AF047354<br>U75744<br>ENSESTT00000038059<br>ENST00000318316<br>GENSCAN00000056417 | Homo sapiens mRNA for deoxyribonuclease I-like 3 precursor variant protein.<br>Homo sapiens cDNA FLJ39801 fis, clone SPLEN2007653.<br>Homo sapiens cDNA FLJ16325 fis, clone STOMA2003158, highly similar to DEOXYRIBONUCLEASE GAMMA PRECURSOR (EC 3.1.21.-).<br>Homo sapiens liver and spleen DNase precursor (LSD) mRNA, complete cds.<br>Homo sapiens DNase gamma mRNA, complete cds.<br><br>cdna:known-ccds chromosome:NCBI36:3:58153454:58175893:-1 gene:ENSG00000163687 CCDS2886.1<br>cdna:Genscan chromosome:NCBI36:3:58153454:58171673:-1 |
| 2686559 | 231404 | 2686458 | core | 102100307 | 102100427 | 3 | - | NM_015429<br>BX648726<br>AB056106<br>AK123737<br>AK123748<br>ENST00000284322<br>ENST00000383692<br>ENST00000383691<br>GENSCAN00000034994<br>ENSESTT00000007186<br>ENSESTT00000007184<br>ENSESTT00000007185<br>AL833204<br>ENST00000273339<br>ENSESTT00000007183<br>AK025204<br>GENSCAN00000036759<br>ENSESTT00000007182<br>GENSCAN00000042526 | Homo sapiens ABI gene family, member 3 (NESH) binding protein (ABI3BP), mRNA.<br>Homo sapiens mRNA; cDNA DKFZp686A12242 (from clone DKFZp686A12242).<br>Homo sapiens mRNA for NeshBP, complete cds.<br>Homo sapiens cDNA FLJ41743 fis, clone HSYRA2005456.<br>Homo sapiens cDNA FLJ41754 fis, clone HSYRA2009075.<br>cdna:known chromosome:NCBI36:3:101950871:102194939:-1 gene:ENSG00000154175<br>cdna:known chromosome:NCBI36:3:101951990:102038712:-1 gene:ENSG00000154175<br>cdna:known chromosome:NCBI36:3:101951990:102019008:-1 gene:ENSG00000154175<br>cdna:Genscan chromosome:NCBI36:3:101952029:101967675:-1<br><br><br><br>Homo sapiens mRNA; cDNA DKFZp667H216 (from clone DKFZp667H216).<br>cdna:known chromosome:NCBI36:3:102017553:102194939:-1 gene:ENSG00000154175<br><br>Homo sapiens cDNA: FLJ21551 fis, clone COL06266.<br>cdna:Genscan chromosome:NCBI36:3:102066135:102068490:-1<br><br>cdna:Genscan chromosome:NCBI36:3:102100296:102153390:-1 |
| 2690978 | 234179 | 2690956 | core | 120866794 | 120866860 | 3 | - | | |
| 2692329 | 234974 | 2692319 | core | 124491322 | 124491405 | 3 | - | NM_183357<br>GENSCAN00000030501<br>ENST00000309879<br>AK124691<br>ENSESTT00000001512<br>ENSESTT00000001513<br>AK093840 | Homo sapiens adenylate cyclase 5 (ADCY5), mRNA.<br>cdna:Genscan chromosome:NCBI36:3:124486145:124554362:-1<br>cdna:known-ccds chromosome:NCBI36:3:124486089:124650082:-1 gene:ENSG00000173175 CCDS3022.1<br>Homo sapiens cDNA FLJ42701 fis, clone BRAMY3004919, highly similar to Adenylate cyclase, type V (EC 4.6.1.1).<br><br><br>Homo sapiens cDNA FLJ36521 fis, clone TRACH2002138, highly similar to ADENYLATE CYCLASE, TYPE V (EC 4.6.1.1). |
| 2692358 | 234996 | 2692319 | core | 124530203 | 124530292 | 3 | - | NM_183357<br>ENST00000309879<br>AK124691<br>ENSESTT00000001512<br>ENSESTT00000001513<br>GENSCAN00000030501<br>AK093840 | Homo sapiens adenylate cyclase 5 (ADCY5), mRNA.<br>cdna:known-ccds chromosome:NCBI36:3:124486089:124650082:-1 gene:ENSG00000173175 CCDS3022.1<br>Homo sapiens cDNA FLJ42701 fis, clone BRAMY3004919, highly similar to Adenylate cyclase, type V (EC 4.6.1.1).<br><br><br>cdna:Genscan chromosome:NCBI36:3:124486145:124554362:-1<br>Homo sapiens cDNA FLJ36521 fis, clone TRACH2002138, highly similar to ADENYLATE CYCLASE, TYPE V (EC 4.6.1.1). |
| 2692359 | 234997 | 2692319 | core | 124532461 | 124532545 | 3 | - | NM_183357<br>ENST00000309879<br>AK124691<br>ENSESTT00000001512<br>ENSESTT00000001513<br>GENSCAN00000030501 | Homo sapiens adenylate cyclase 5 (ADCY5), mRNA.<br>cdna:known-ccds chromosome:NCBI36:3:124486089:124650082:-1 gene:ENSG00000173175 CCDS3022.1<br>Homo sapiens cDNA FLJ42701 fis, clone BRAMY3004919, highly similar to Adenylate cyclase, type V (EC 4.6.1.1). |

| | | | | | | | | | |
|---|---|---|---|---|---|---|---|---|---|
| | | | | | | | | AK093840 | cdna:Genscan chromosome:NCBI36:3:124486145:124554362:-1<br>Homo sapiens cDNA FLJ36521 fis, clone TRACH2002138, highly similar to ADENYLATE CYCLASE, TYPE V (EC 4.6.1.1). |
| 2693564 | 235778 | 2693563 | core | 127634468 | 127634730 | 3 | - | AK000130 | Homo sapiens cDNA FLJ20123 fis, clone COL06041. |
| 2693566 | 235778 | 2693563 | core | 127635735 | 127635881 | 3 | - | AK000130 | Homo sapiens cDNA FLJ20123 fis, clone COL06041. |
| 2710544 | 246404 | 2710474 | core | 191321217 | 191321277 | 3 | - | NM_018192<br>ENST00000319332<br>ENSESTT00000046375<br>ENSESTT00000046378<br>ENSESTT00000046380<br>ENSESTT00000046381<br>ENSESTT00000046376<br>ENSESTT00000046379<br>GENSCAN00000031374<br>ENSESTT00000046377 | Homo sapiens leprecan-like 1 (LEPREL1), mRNA.<br>cdna:known-ccds chromosome:NCBI36:3:191157213:191321407:-1 gene:ENSG00000090530 CCDS3294.1<br><br><br><br><br><br><br>cdna:Genscan chromosome:NCBI36:3:191162190:191201552:-1 |
| 2710603 | 246437 | 2710599 | core | 191506513 | 191506657 | 3 | - | NM_021101<br>AK225963<br>ENST00000295522<br>AF134160<br>ENSESTT00000014978<br>ENSESTT00000014979<br>GENSCAN00000062238 | Homo sapiens claudin 1 (CLDN1), mRNA.<br>Homo sapiens mRNA for claudin 1 variant, clone: FCC114C08.<br>cdna:known-ccds chromosome:NCBI36:3:191506197:191522909:-1 gene:ENSG00000163347 CCDS3295.1<br>Homo sapiens claudin-1 (CLDN1) mRNA, complete cds.<br><br><br>cdna:Genscan chromosome:NCBI36:3:191508760:191522689:-1 |
| 2712385 | 247572 | 2712236 | core | 197023146 | 197023210 | 3 | - | NM_138297<br>NM_018406<br>AJ000281<br>ENSESTT00000006041<br>GENSCAN00000034839<br>ENST00000346145<br>ENST00000349607<br>ENST00000339251<br>ENST00000308466<br>ENST00000333177<br>NM_004532<br>AJ242541<br>GENSCAN00000034842 | Homo sapiens mucin 4, cell surface associated (MUC4), transcript variant 5, mRNA.<br>Homo sapiens mucin 4, cell surface associated (MUC4), transcript variant 1, mRNA.<br>Homo sapiens mRNA for mucin protein, MUC4.<br><br>cdna:Genscan chromosome:NCBI36:3:196981259:197036867:-1<br>cdna:known-ccds chromosome:NCBI36:3:196959311:197023545:-1 gene:ENSG00000145113 CCDS3310.1<br>cdna:known-ccds chromosome:NCBI36:3:196959311:197023545:-1 gene:ENSG00000145113 CCDS3311.1<br>cdna:known chromosome:NCBI36:3:196959311:197023545:-1 gene:ENSG00000145113<br>cdna:known chromosome:NCBI36:3:196959311:197002767:-1 gene:ENSG00000145113<br>cdna:known chromosome:NCBI36:3:196959311:197002767:-1 gene:ENSG00000145113<br>Homo sapiens mucin 4, cell surface associated (MUC4), transcript variant 4, mRNA.<br>Homo sapiens partial mRNA for sv1-MUC4 apomucin.<br>cdna:Genscan chromosome:NCBI36:3:196959718:196976984:-1 |
| 2727236 | 256765 | 2727226 | core | 53944697 | 53944800 | 4 | + | NM_030917<br>ENST00000337488<br>ENST00000306932<br>ENST00000358575<br>AY229892<br>ENSESTT00000016271<br>ENSESTT00000016272<br>ENSESTT00000016273<br>BC017724<br>ENSESTT00000016270<br>GENSCAN00000023418<br>BC024016<br>GENSCAN00000010665<br>AK123992<br>CR595922<br>AK090938<br>ENSESTT00000016275 | Homo sapiens FIP1 like 1 (S. cerevisiae) (FIP1L1), mRNA.<br>cdna:known-ccds chromosome:NCBI36:4:53938620:54020599:1 gene:ENSG00000145216 CCDS3491.1<br>cdna:known chromosome:NCBI36:4:53938620:54020599:1 gene:ENSG00000145216<br>cdna:known chromosome:NCBI36:4:53938620:54020545:1 gene:ENSG00000145216<br>Homo sapiens FIP1L1/PDGFRA fusion protein (FIP1L1/PDGFRA fusion) mRNA, complete cds; alternatively spliced.<br><br><br><br>Homo sapiens FIP1 like 1 (S. cerevisiae), mRNA (cDNA clone MGC:21370 IMAGE:3452247), complete cds.<br><br>cdna:Genscan chromosome:NCBI36:4:53938763:53944800:1<br>Homo sapiens FIP1 like 1 (S. cerevisiae), mRNA (cDNA clone MGC:26986 IMAGE:4826276), complete cds.<br>cdna:Genscan chromosome:NCBI36:4:53950733:54020373:1<br>Homo sapiens cDNA FLJ41998 fis, clone SPLEN2029522.<br>full-length cDNA clone CS0DD005YK11 of Neuroblastoma Cot 50-normalized of Homo sapiens (human).<br>Homo sapiens cDNA FLJ33619 fis, clone BRAMY2020427. |

| | | | | | | | | | |
|---|---|---|---|---|---|---|---|---|---|
| | | | | | | | | ENSESTT00000016276<br>ENSESTT00000016274 | |
| 2729063 | 257948 | 2728938 | core | 62618509 | 62619146 | 4 | + | NM_015236<br>XM_937110<br>GENSCAN00000023734<br>ENSESTT00000013498<br>ENST00000280009<br>ENST00000295349<br>ENST00000355061<br>GENSCAN00000040521<br>ENSESTT00000022383<br>ENSESTT00000022384<br>ENSESTT00000022385<br>ENSESTT00000022386<br>GENSCAN00000001109<br>GENSCAN00000045466<br>ENSESTT00000022387<br>ENSESTT00000022388 | Homo sapiens latrophilin 3 (LPHN3), mRNA.<br>PREDICTED: Homo sapiens similar to Latrophilin-3 precursor (Calcium-independent alpha-latrotoxin receptor 3) (Lectomedin-3) (LOC653950), mRNA.<br>cdna:Genscan chromosome:NCBI36:4:62567041:62619221:1<br><br>cdna:known chromosome:NCBI36:4:62045434:62620762:1 gene:ENSG00000150471<br>cdna:known chromosome:NCBI36:4:62045434:62620762:1 gene:ENSG00000150471<br>cdna:known chromosome:NCBI36:4:62045434:62620110:1 gene:ENSG00000150471<br>cdna:Genscan chromosome:NCBI36:4:62281067:62289968:1<br><br><br><br><br>cdna:Genscan chromosome:NCBI36:4:62434820:62445414:1<br>cdna:Genscan chromosome:NCBI36:4:62457873:62544670:1 |
| 2730769 | 258958 | 2730746 | core | 72339899 | 72339932 | 4 | + | AF004813<br>AF011390<br>ENSESTT00000014873<br>ENST00000264485<br>ENST00000351898<br>GENSCAN00000042650 | Homo sapiens electrogenic Na+ bicarbonate cotransporter (NBC) mRNA, partial cds.<br>Homo sapiens pancreas sodium bicarbonate cotransporter mRNA, complete cds.<br><br>cdna:known chromosome:NCBI36:4:72271867:72654298:1 gene:ENSG00000080493<br>cdna:known chromosome:NCBI36:4:72271867:72654298:1 gene:ENSG00000080493<br>cdna:Genscan chromosome:NCBI36:4:72271857:72344483:1 |
| 2730780 | 258967 | 2730746 | core | 72423654 | 72423772 | 4 | + | NM_003759<br>BC030977<br>ENST00000340595<br>AF004813<br>AF011390<br>ENST00000264485<br>ENST00000351898<br>GENSCAN00000050788<br>ENSESTT00000014875<br>ENSESTT00000014876<br>ENSESTT00000014877<br>ENSESTT00000014878<br>GENSCAN00000043402<br>ENSESTT00000014879 | Homo sapiens solute carrier family 4, sodium bicarbonate cotransporter, member 4 (SLC4A4), mRNA.<br>Homo sapiens solute carrier family 4, sodium bicarbonate cotransporter, member 4, mRNA (cDNA clone MGC:32627 IMAGE:4610968), complete cds.<br>cdna:known-ccds chromosome:NCBI36:4:72423681:72656663:1 gene:ENSG00000080493 CCDS3549.1<br>Homo sapiens electrogenic Na+ bicarbonate cotransporter (NBC) mRNA, partial cds.<br>Homo sapiens pancreas sodium bicarbonate cotransporter mRNA, complete cds.<br>cdna:known chromosome:NCBI36:4:72271867:72654298:1 gene:ENSG00000080493<br>cdna:known chromosome:NCBI36:4:72271867:72654298:1 gene:ENSG00000080493<br>cdna:Genscan chromosome:NCBI36:4:72434493:72566441:1<br><br><br><br><br>cdna:Genscan chromosome:NCBI36:4:72614104:72692244:1 |
| 2730781 | 258967 | 2730746 | core | 72423893 | 72423940 | 4 | + | NM_003759<br>BC030977<br>GENSCAN00000046032<br>ENST00000340595<br>AF004813<br>AF011390<br>ENST00000264485<br>ENST00000351898<br>GENSCAN00000050788<br>ENSESTT00000014875<br>ENSESTT00000014876<br>ENSESTT00000014877 | Homo sapiens solute carrier family 4, sodium bicarbonate cotransporter, member 4 (SLC4A4), mRNA.<br>Homo sapiens solute carrier family 4, sodium bicarbonate cotransporter, member 4, mRNA (cDNA clone MGC:32627 IMAGE:4610968), complete cds.<br>cdna:Genscan chromosome:NCBI36:4:72423830:72424086:1<br>cdna:known-ccds chromosome:NCBI36:4:72423681:72656663:1 gene:ENSG00000080493 CCDS3549.1<br>Homo sapiens electrogenic Na+ bicarbonate cotransporter (NBC) mRNA, partial cds.<br>Homo sapiens pancreas sodium bicarbonate cotransporter mRNA, complete cds.<br>cdna:known chromosome:NCBI36:4:72271867:72654298:1 gene:ENSG00000080493<br>cdna:known chromosome:NCBI36:4:72271867:72654298:1 gene:ENSG00000080493<br>cdna:Genscan chromosome:NCBI36:4:72434493:72566441:1 |

| | | | | | | | | | |
|---|---|---|---|---|---|---|---|---|---|
| | | | | | | | | ENSESTT00000014878<br>GENSCAN00000043402<br>ENSESTT00000014879 | cdna:Genscan chromosome:NCBI36:4:72614104:72692244:1 |
| 2730794 | 258978 | 2730746 | core | 72535007 | 72535110 | 4 | + | NM_003759<br>ENST00000340595<br>AF004813<br>AF011390<br>BC030977<br>ENST00000264485<br>ENST00000351898<br>GENSCAN00000050788<br>ENSESTT00000014875<br>ENSESTT00000014876<br>ENSESTT00000014877<br>ENSESTT00000014878<br>GENSCAN00000043402<br>ENSESTT00000014879 | Homo sapiens solute carrier family 4, sodium bicarbonate cotransporter, member 4 (SLC4A4), mRNA.<br>cdna:known-ccds chromosome:NCBI36:4:72423681:72656663:1 gene:ENSG00000080493 CCDS3549.1<br>Homo sapiens electrogenic Na+ bicarbonate cotransporter (NBC) mRNA, partial cds.<br>Homo sapiens pancreas sodium bicarbonate cotransporter mRNA, complete cds.<br>Homo sapiens solute carrier family 4, sodium bicarbonate cotransporter, member 4, mRNA (cDNA clone MGC:32627 IMAGE:4610968), complete cds.<br>cdna:known chromosome:NCBI36:4:72271867:72654298:1 gene:ENSG00000080493<br>cdna:known chromosome:NCBI36:4:72271867:72654298:1 gene:ENSG00000080493<br>cdna:Genscan chromosome:NCBI36:4:72434493:72566441:1<br>cdna:Genscan chromosome:NCBI36:4:72614104:72692244:1 |
| 2734077 | 261021 | 2734047 | core | 84737035 | 84737090 | 4 | + | NM_032717<br>AK055749<br>ENSESTT00000000138<br>ENSESTT00000000139<br>GENSCAN00000067582<br>ENST00000264409<br>GENSCAN00000021363<br>ENSESTT00000000140<br>ENSESTT00000000141 | Homo sapiens lysophosphatidic acid acyltransferase theta (LPAAT-THETA), mRNA.<br>Homo sapiens cDNA FLJ31187 fis, clone KIDNE2000349, moderately similar to Mus musculus putative lysophosphatidic acid acyltransferase mRNA.<br>cdna:Genscan chromosome:NCBI36:4:84675910:84693775:1<br>cdna:known-ccds chromosome:NCBI36:4:84676588:84746049:1 gene:ENSG00000138678 CCDS3606.1<br>cdna:Genscan chromosome:NCBI36:4:84702894:84744944:1 |
| 2735071 | 261685 | 2735027 | core | 89123092 | 89123291 | 4 | + | NM_001040058<br>BC007016<br>ENSESTT00000042535<br>ENST00000237623<br>ENST00000359072<br>NM_001040060<br>NM_000582<br>ENSESTT00000042536<br>ENST00000360804<br>AY956318<br>BX648003<br>GENSCAN00000030225 | Homo sapiens secreted phosphoprotein 1 (osteopontin, bone sialoprotein I, early T-lymphocyte activation 1) (SPP1), transcript variant 1, mRNA.<br>Homo sapiens secreted phosphoprotein 1 (osteopontin, bone sialoprotein I, early T-lymphocyte activation 1), mRNA (cDNA clone MGC:12351 IMAGE:4052438), complete cds.<br>cdna:known-ccds chromosome:NCBI36:4:89115890:89123502:1 gene:ENSG00000118785 CCDS3626.1<br>cdna:known chromosome:NCBI36:4:89115905:89123592:1 gene:ENSG00000118785<br>Homo sapiens secreted phosphoprotein 1 (osteopontin, bone sialoprotein I, early T-lymphocyte activation 1) (SPP1), transcript variant 3, mRNA.<br>Homo sapiens secreted phosphoprotein 1 (osteopontin, bone sialoprotein I, early T-lymphocyte activation 1) (SPP1), transcript variant 2, mRNA.<br>cdna:known chromosome:NCBI36:4:89115890:89123502:1 gene:ENSG00000118785<br>Homo sapiens osteopontin/immunoglobulin alpha 1 heavy chain constant region fusion protein (SPP1/CALPHA1 fusion) mRNA, partial cds.<br>Homo sapiens mRNA; cDNA DKFZp686G0159 (from clone DKFZp686G0159).<br>cdna:Genscan chromosome:NCBI36:4:89115845:89123072:1 |
| 2740243 | 265073 | 2740067 | core | 114494644 | 114496079 | 4 | + | NM_001148<br>AK095596<br>BX537758<br>ENST00000357077<br>ENST00000361149<br>GENSCAN00000013660 | Homo sapiens ankyrin 2, neuronal (ANK2), transcript variant 1, mRNA.<br>Homo sapiens cDNA FLJ38277 fis, clone FCBBF3004955, highly similar to ANKYRIN 2.<br>Homo sapiens mRNA; cDNA DKFZp686H0688 (from clone DKFZp686H0688); complete cds.<br>cdna:known-ccds chromosome:NCBI36:4:114190319:114524334:1 gene:ENSG00000145362 CCDS3702.1<br>cdna:known chromosome:NCBI36:4:114190319:114524334:1 gene:ENSG00000145362<br>cdna:Genscan chromosome:NCBI36:4:114451860:114577680:1 |
| 2740248 | 265073 | 2740067 | core | 114498366 | 114498776 | 4 | + | NM_001148 | Homo sapiens ankyrin 2, neuronal (ANK2), transcript variant 1, mRNA. |

| | | | | | | | | | |
|---|---|---|---|---|---|---|---|---|---|
| | | | | | | | | ENST00000357077<br>ENST00000361149<br>GENSCAN00000013660 | cdna:known-ccds chromosome:NCBI36:4:114190319:114524334:1 gene:ENSG00000145362 CCDS3702.1<br>cdna:known chromosome:NCBI36:4:114190319:114524334:1 gene:ENSG00000145362<br>cdna:Genscan chromosome:NCBI36:4:114451860:114577680:1 |
| 2746627 | 269027 | 2746591 | core | 148680983 | 148681021 | 4 | + | NM_001957<br>ENSESTT00000044921<br>S67127<br>ENSESTT00000044923<br>ENSESTT00000044924<br>ENST00000324300<br>GENSCAN00000005966<br>ENST00000339690<br>ENST00000358556 | Homo sapiens endothelin receptor type A (EDNRA), mRNA.<br><br>endothelin ETA receptor [human, placenta, mRNA, 1661 nt].<br><br><br>cdna:known-ccds chromosome:NCBI36:4:148621575:148685555:1 gene:ENSG00000151617 CCDS3769.1<br>cdna:Genscan chromosome:NCBI36:4:148599594:148683204:1<br>cdna:known chromosome:NCBI36:4:148621575:148685555:1 gene:ENSG00000151617<br>cdna:known chromosome:NCBI36:4:148621575:148685555:1 gene:ENSG00000151617 |
| 2746628 | 269027 | 2746591 | core | 148681031 | 148681057 | 4 | + | NM_001957<br>ENSESTT00000044921<br>S67127<br>ENSESTT00000044923<br>ENSESTT00000044924<br>ENST00000324300<br>GENSCAN00000005966<br>ENST00000339690<br>ENST00000358556 | Homo sapiens endothelin receptor type A (EDNRA), mRNA.<br><br>endothelin ETA receptor [human, placenta, mRNA, 1661 nt].<br><br><br>cdna:known-ccds chromosome:NCBI36:4:148621575:148685555:1 gene:ENSG00000151617 CCDS3769.1<br>cdna:Genscan chromosome:NCBI36:4:148599594:148683204:1<br>cdna:known chromosome:NCBI36:4:148621575:148685555:1 gene:ENSG00000151617<br>cdna:known chromosome:NCBI36:4:148621575:148685555:1 gene:ENSG00000151617 |
| 2746632 | 269029 | 2746591 | core | 148683112 | 148683204 | 4 | + | NM_001957<br>ENST00000324300<br>GENSCAN00000005966<br>ENST00000358556<br>ENST00000339690<br>ENSESTT00000044921<br>S67127<br>ENSESTT00000044923<br>ENSESTT00000044924 | Homo sapiens endothelin receptor type A (EDNRA), mRNA.<br>cdna:known-ccds chromosome:NCBI36:4:148621575:148685555:1 gene:ENSG00000151617 CCDS3769.1<br>cdna:Genscan chromosome:NCBI36:4:148599594:148683204:1<br>cdna:known chromosome:NCBI36:4:148621575:148685555:1 gene:ENSG00000151617<br>cdna:known chromosome:NCBI36:4:148621575:148685555:1 gene:ENSG00000151617<br><br>endothelin ETA receptor [human, placenta, mRNA, 1661 nt]. |
| 2746804 | 269142 | 2746693 | core | 149213265 | 149213357 | 4 | + | NM_024605<br>BC047914<br>GENSCAN00000016310<br>ENST00000336498<br>GENSCAN00000003834<br>AK123785<br>GENSCAN00000045084<br>BX647727<br>ENSESTT00000044927<br>ENSESTT00000044928<br>ENSESTT00000044929<br>ENSESTT00000044930<br>GENSCAN00000034801<br>GENSCAN00000016307<br>ENSESTT00000044931 | Homo sapiens Rho GTPase activating protein 10 (ARHGAP10), mRNA.<br>Homo sapiens Rho GTPase activating protein 10, mRNA (cDNA clone IMAGE:6188248), complete cds.<br>cdna:Genscan chromosome:NCBI36:4:149211409:149220969:1<br>cdna:known chromosome:NCBI36:4:148872903:149213376:1 gene:ENSG00000071205<br>cdna:Genscan chromosome:NCBI36:4:148872903:148888229:1<br>Homo sapiens cDNA FLJ41791 fis, clone NESOP2001694, moderately similar to H.sapiens graf gene.<br>cdna:Genscan chromosome:NCBI36:4:148956670:149095975:1<br>Homo sapiens mRNA; cDNA DKFZp686G2124 (from clone DKFZp686G2124).<br><br><br><br><br>cdna:Genscan chromosome:NCBI36:4:149105625:149110864:1<br>cdna:Genscan chromosome:NCBI36:4:149163864:149195615:1 |
| 2758105 | 276058 | 2758076 | core | 2922639 | 2922716 | 4 | - | NM_003703<br>CR612484<br>ENST00000314262<br>AK025692<br>ENSESTT00000003411<br>ENSESTT00000003410<br>GENSCAN00000068065<br>BC009760 | Homo sapiens chromosome 4 open reading frame 9 (C4orf9), mRNA.<br>full-length cDNA clone CS0DC011YA09 of Neuroblastoma Cot 25-normalized of Homo sapiens (human).<br>cdna:known chromosome:NCBI36:4:2909464:2934916:-1 gene:ENSG00000087269<br>Homo sapiens cDNA: FLJ22039 fis, clone HEP09009, highly similar to AB000467 Homo sapiens mRNA.<br><br><br>cdna:Genscan chromosome:NCBI36:4:2902363:2934844:-1<br>Homo sapiens chromosome 4 open reading frame 9, mRNA (cDNA clone IMAGE:3936655), partial cds. |

| | | | | | | | | | |
|---|---|---|---|---|---|---|---|---|---|
| | | | | | | | | ENSESTT00000003407<br>BC017358 | <br>Homo sapiens chromosome 4 open reading frame 9, mRNA (cDNA clone IMAGE:5093564), with apparent retained intron. |
| 2779200 | 289308 | 2779199 | core | 100416700 | 100416801 | 4 | - | NM_000667<br>ENST00000209668 | Homo sapiens alcohol dehydrogenase 1A (class I), alpha polypeptide (ADH1A), mRNA.<br>cdna:known-ccds chromosome:NCBI36:4:100416547:100431165:-1 gene:ENSG00000187758 CCDS3648.1 |
| 2786337 | 293942 | 2786322 | core | 139321245 | 139321323 | 4 | - | NM_014331<br>ENST00000280612<br>ENSESTT00000005138<br>ENSESTT00000005135<br>ENSESTT00000005136 | Homo sapiens solute carrier family 7, (cationic amino acid transporter, y+ system) member 11 (SLC7A11), mRNA.<br>cdna:known-ccds chromosome:NCBI36:4:139304698:139382953:-1 gene:ENSG00000151012 CCDS3742.1 |
| 2786361 | 293964 | 2786322 | core | 139382726 | 139382860 | 4 | - | NM_014331<br>ENSESTT00000005137<br>ENST00000280612<br>ENSESTT00000005138<br>ENSESTT00000005135<br>ENSESTT00000005136 | Homo sapiens solute carrier family 7, (cationic amino acid transporter, y+ system) member 11 (SLC7A11), mRNA.<br><br>cdna:known-ccds chromosome:NCBI36:4:139304698:139382953:-1 gene:ENSG00000151012 CCDS3742.1 |
| 2801699 | 303765 | 2801694 | core | 10495301 | 10495393 | 5 | + | ENSESTT00000046068<br>ENST00000382469<br>ENST00000274134<br>GENSCAN00000060610<br>NM_031916<br>ENSESTT00000046069 | <br>cdna:known-ccds chromosome:NCBI36:5:10494988:10518140:1 gene:ENSG00000145491 CCDS3879.1<br>cdna:known chromosome:NCBI36:5:10495013:10518135:1 gene:ENSG00000145491<br>cdna:Genscan chromosome:NCBI36:5:10495280:10506642:1<br>Homo sapiens ropporin 1-like (ROPN1L), mRNA. |
| 2810409 | 309178 | 2810395 | core | 56248406 | 56248480 | 5 | + | NM_153706<br>CR625068<br>ENSESTT00000050370<br>ENST00000381264<br>GENSCAN00000052998<br>ENST00000285947<br>ENSESTT00000050373 | Homo sapiens hypothetical protein MGC33648 (MGC33648), mRNA.<br>full-length cDNA clone CS0DC017YN09 of Neuroblastoma Cot 25-normalized of Homo sapiens (human).<br><br>cdna:known-ccds chromosome:NCBI36:5:56240844:56248819:1 gene:ENSG00000155542 CCDS3972.1<br>cdna:Genscan chromosome:NCBI36:5:56241230:56248486:1<br>cdna:known chromosome:NCBI36:5:56240857:56248765:1 gene:ENSG00000155542 |
| 2820948 | 315923 | 2820925 | core | 95116969 | 95117160 | 5 | + | NM_014899<br>AK023621<br>GENSCAN00000040613<br>CR596551<br>ENSESTT00000002391<br>ENST00000379982<br>ENST00000296731<br>ENSESTT00000002392 | Homo sapiens Rho-related BTB domain containing 3 (RHOBTB3), mRNA.<br>Homo sapiens cDNA FLJ13559 fis, clone PLACE1007852, highly similar to Homo sapiens mRNA for KIAA0878 protein.<br>cdna:Genscan chromosome:NCBI36:5:95044142:95131346:1<br>full-length cDNA clone CS0DA010YB13 of Neuroblastoma of Homo sapiens (human).<br><br>cdna:known-ccds chromosome:NCBI36:5:95092635:95157827:1 gene:ENSG00000164292 CCDS4077.1<br>cdna:known chromosome:NCBI36:5:95092799:95156560:1 gene:ENSG00000164292 |
| 2842662 | 329327 | 2842624 | core | 175945561 | 175945611 | 5 | + | NM_017675<br>ENST00000261944<br>ENSESTT00000006503<br>GENSCAN00000044009<br>ENST00000389947<br>ENST00000389946<br>ENSESTT00000006504<br>ENSESTT00000006505<br>ENSESTT00000006506<br>ENSESTT00000006507 | Homo sapiens protocadherin LKC (PCLKC), mRNA.<br>cdna:novel chromosome:NCBI36:5:175908971:175955279:1 gene:ENSG00000074276<br><br>cdna:Genscan chromosome:NCBI36:5:175902503:175950934:1<br>cdna:known chromosome:NCBI36:5:175924960:175955279:1 gene:ENSG00000074276<br>cdna:known chromosome:NCBI36:5:175924960:175955279:1 gene:ENSG00000074276 |
| 2853654 | 336173 | 2853642 | core | 37144224 | 37144248 | 5 | - | NM_023073<br>ENSESTT00000056646 | Homo sapiens hypothetical protein FLJ13231 (FLJ13231), mRNA. |

| | | | | | | | | | |
|---|---|---|---|---|---|---|---|---|---|
| | | | | | | | | ENST00000274258<br>ENST00000335189<br>AK096581<br>ENSESTT00000056644<br>ENSESTT00000056645<br>ENST00000388739<br>GENSCAN00000052139<br>BX649121<br>ENSESTT00000056642<br>ENSESTT00000056643<br>BC028410<br>AL832176<br>ENSESTT00000056641<br>ENSESTT00000056640 | cdna:known chromosome:NCBI36:5:37142087:37237668:-1 gene:ENSG00000197603<br>cdna:known chromosome:NCBI36:5:37143141:37194207:-1 gene:ENSG00000197603<br>Homo sapiens cDNA FLJ39262 fis, clone OCBBF2009424.<br><br><br>cdna:known chromosome:NCBI36:5:37143461:37237599:-1 gene:ENSG00000197603<br>cdna:Genscan chromosome:NCBI36:5:37115660:37203072:-1<br>Homo sapiens mRNA; cDNA DKFZp686H2337 (from clone DKFZp686H2337).<br><br><br>Homo sapiens hypothetical protein FLJ13231, mRNA (cDNA clone IMAGE:4822016), partial cds.<br>Homo sapiens mRNA; cDNA DKFZp686M1016 (from clone DKFZp686M1016). |
| 2853812 | 336293 | 2853768 | core | 37387091 | 37387205 | 5 | - | NM_004298<br>NM_153485<br>AL117585<br>ENSESTT00000056639<br>ENST00000381843<br>ENST00000231498<br>GENSCAN00000013321<br>ENSESTT00000056638<br>ENSESTT00000056637<br>ENSESTT00000056636<br>GENSCAN00000018439 | Homo sapiens nucleoporin 155kDa (NUP155), transcript variant 2, mRNA.<br>Homo sapiens nucleoporin 155kDa (NUP155), transcript variant 1, mRNA.<br>Homo sapiens mRNA; cDNA DKFZp434L038 (from clone DKFZp434L038).<br><br>cdna:known-ccds chromosome:NCBI36:5:37323996:37406954:-1 gene:ENSG00000113569 CCDS3921.1<br>cdna:known chromosome:NCBI36:5:37327698:37406954:-1 gene:ENSG00000113569<br>cdna:Genscan chromosome:NCBI36:5:37249326:37378505:-1<br><br><br><br>cdna:Genscan chromosome:NCBI36:5:37400106:37406836:-1 |
| 2854488 | 336758 | 2854445 | core | 39428263 | 39428290 | 5 | - | ENSESTT00000031511<br>ENSESTT00000031512<br>NM_001343<br>L16886<br>ENSESTT00000031508<br>ENSESTT00000031509<br>ENSESTT00000031510<br>ENST00000339788<br>ENST00000388868<br>ENST00000388867<br>ENST00000320816<br>GENSCAN00000021653 | <br><br>Homo sapiens disabled homolog 2, mitogen-responsive phosphoprotein (Drosophila) (DAB2), mRNA.<br>Human differentially expressed protein mRNA, partial cds.<br><br><br><br>cdna:known chromosome:NCBI36:5:39408503:39460703:-1 gene:ENSG00000153071<br>cdna:known chromosome:NCBI36:5:39410878:39430179:-1 gene:ENSG00000153071<br>cdna:known chromosome:NCBI36:5:39410878:39430179:-1 gene:ENSG00000153071<br>cdna:novel chromosome:NCBI36:5:39408503:39460703:-1 gene:ENSG00000153071<br>cdna:Genscan chromosome:NCBI36:5:39364144:39444342:-1 |
| 2866232 | 343972 | 2866225 | core | 88054471 | 88054498 | 5 | - | NM_002397<br>AL833268<br>ENST00000357034<br>GENSCAN00000017836<br>ENST00000340208<br>ENSESTT00000035256<br>ENSESTT00000035257<br>ENSESTT00000035254<br>ENSESTT00000035255 | Homo sapiens MADS box transcription enhancer factor 2, polypeptide C (myocyte enhancer factor 2C) (MEF2C), mRNA.<br>Homo sapiens mRNA; cDNA DKFZp451I0810 (from clone DKFZp451I0810).<br>cdna:known chromosome:NCBI36:5:88051922:88214818:-1 gene:ENSG00000081189<br>cdna:Genscan chromosome:NCBI36:5:88054177:88108581:-1<br>cdna:known chromosome:NCBI36:5:88051922:88214818:-1 gene:ENSG00000081189 |
| 2886683 | 356792 | 2886679 | core | 169743184 | 169743215 | 5 | - | BC025707 | Homo sapiens potassium large conductance calcium-activated channel, subfamily M, beta member 1, mRNA (cDNA clone MGC:34483 IMAGE:5224514), complete cds. |
| 2889917 | 358697 | 2889916 | core | 178473758 | 178473924 | 5 | - | NM_014244<br>ENSESTT00000017819<br>ENSESTT00000017820<br>ENST00000251582 | Homo sapiens ADAM metallopeptidase with thrombospondin type 1 motif, 2 (ADAMTS2), transcript variant 1, mRNA. |

| | | | | | | | | | |
|---|---|---|---|---|---|---|---|---|---|
| | | | | | | | | GENSCAN00000011848 | cdna:known-ccds chromosome:NCBI36:5:178473474:178704935:-1 gene:ENSG00000087116 CCDS4444.1<br>cdna:Genscan chromosome:NCBI36:5:178473474:178541885:-1 |
| 2891610 | 359696 | 2891556 | core | 1258246 | 1258271 | 6 | + | NM_033260<br>ENST00000380899<br>GENSCAN00000058908<br>ENST00000296839 | Homo sapiens forkhead box Q1 (FOXQ1), mRNA.<br>cdna:known chromosome:NCBI36:6:1257708:1259422:1 gene:ENSG00000164379<br>cdna:Genscan chromosome:NCBI36:6:1257940:1259151:1<br>cdna:known-ccds chromosome:NCBI36:6:1257675:1259981:1 gene:ENSG00000164379 CCDS4471.1 |
| 2891616 | 359697 | 2891556 | core | 1259213 | 1259600 | 6 | + | NM_033260<br>ENST00000380899<br>ENST00000296839 | Homo sapiens forkhead box Q1 (FOXQ1), mRNA.<br>cdna:known chromosome:NCBI36:6:1257708:1259422:1 gene:ENSG00000164379<br>cdna:known-ccds chromosome:NCBI36:6:1257675:1259981:1 gene:ENSG00000164379 CCDS4471.1 |
| 2891617 | 359698 | 2891556 | core | 1259651 | 1259869 | 6 | + | NM_033260<br>ENST00000296839 | Homo sapiens forkhead box Q1 (FOXQ1), mRNA.<br>cdna:known-ccds chromosome:NCBI36:6:1257675:1259981:1 gene:ENSG00000164379 CCDS4471.1 |
| 2897904 | 363652 | 2897899 | core | 21703280 | 21703337 | 6 | + | ENST00000378570<br>ENST00000244745<br>GENSCAN00000002043<br>NM_003107 | cdna:known-ccds chromosome:NCBI36:6:21700979:21706829:1 gene:ENSG00000124766 CCDS4547.1<br>cdna:known chromosome:NCBI36:6:21701951:21706826:1 gene:ENSG00000124766<br>cdna:Genscan chromosome:NCBI36:6:21702745:21704169:1<br>Homo sapiens SRY (sex determining region Y)-box 4 (SOX4), mRNA. |
| 2907687 | 369280 | 2907671 | core | 43204795 | 43204917 | 6 | + | GENSCAN00000029527<br>NM_152882<br>ENST00000359792<br>NM_152880<br>BC046109<br>ENST00000325774<br>NM_152881<br>NM_002821<br>ENST00000345201<br>ENST00000230419<br>ENST00000352931<br>ENSESTT00000056720<br>ENST00000230418<br>ENSESTT00000056719<br>ENST00000349241 | cdna:Genscan chromosome:NCBI36:6:43152205:43236597:1<br>Homo sapiens PTK7 protein tyrosine kinase 7 (PTK7), transcript variant PTK7-4, mRNA.<br>cdna:novel chromosome:NCBI36:6:43152007:43237435:1 gene:ENSG00000112655<br>Homo sapiens PTK7 protein tyrosine kinase 7 (PTK7), transcript variant PTK7-2, mRNA.<br>Homo sapiens PTK7 protein tyrosine kinase 7, mRNA (cDNA clone IMAGE:5551146), complete cds.<br>cdna:known chromosome:NCBI36:6:43152007:43237435:1 gene:ENSG00000112655<br>Homo sapiens PTK7 protein tyrosine kinase 7 (PTK7), transcript variant PTK7-3, mRNA.<br>Homo sapiens PTK7 protein tyrosine kinase 7 (PTK7), transcript variant PTK7-1, mRNA.<br>cdna:known-ccds chromosome:NCBI36:6:43152007:43237435:1 gene:ENSG00000112655 CCDS4885.1<br>cdna:known-ccds chromosome:NCBI36:6:43152007:43237435:1 gene:ENSG00000112655 CCDS4884.1<br>cdna:known-ccds chromosome:NCBI36:6:43152007:43237435:1 gene:ENSG00000112655 CCDS4887.1<br><br>cdna:known-ccds chromosome:NCBI36:6:43152007:43237435:1 gene:ENSG00000112655 CCDS4888.1<br><br>cdna:known-ccds chromosome:NCBI36:6:43152007:43237435:1 gene:ENSG00000112655 CCDS4886.1 |
| 2923869 | 379326 | 2923868 | core | 122834785 | 122834877 | 6 | + | NM_181794<br>AJ420562<br>CR749456<br>GENSCAN00000056024<br>NM_032471<br>NM_181795<br>ENST00000368452<br>ENSESTT00000010766<br>ENST00000368451<br>ENST00000368448<br>AK074397 | Homo sapiens protein kinase (cAMP-dependent, catalytic) inhibitor beta (PKIB), transcript variant 2, mRNA.<br>Homo sapiens mRNA full length insert cDNA clone EUROIMAGE 1525273.<br>Homo sapiens mRNA; cDNA DKFZp781K1114 (from clone DKFZp781K1114).<br>cdna:Genscan chromosome:NCBI36:6:122972795:123005538:1<br>Homo sapiens protein kinase (cAMP-dependent, catalytic) inhibitor beta (PKIB), transcript variant 3, mRNA.<br>Homo sapiens protein kinase (cAMP-dependent, catalytic) inhibitor beta (PKIB), transcript variant 1, mRNA.<br>cdna:known-ccds chromosome:NCBI36:6:122973076:123089216:1 gene:ENSG00000135549 CCDS5126.1<br><br>cdna:known chromosome:NCBI36:6:122973076:123088736:1 gene:ENSG00000135549<br>cdna:known chromosome:NCBI36:6:122973076:123088736:1 gene:ENSG00000135549<br>Homo sapiens cDNA FLJ23817 fis, clone HSI07950. |
| 2924555 | 379794 | 2924514 | core | 126245267 | 126245386 | 6 | + | AB074157<br>ENSESTT00000024650<br>AK127512<br>NM_181782<br>ENST00000355470<br>ENST00000368357<br>GENSCAN00000062579<br>ENSESTT00000024649<br>ENSESTT00000024651<br>ENSESTT00000024648<br>AL834442 | Homo sapiens primary neuroblastoma cDNA, clone:Nbla10993, full insert sequence.<br><br>Homo sapiens cDNA FLJ45605 fis, clone BRTHA3021971, moderately similar to Homo sapiens oxidation resistance 1 (OXR1).<br>Homo sapiens nuclear receptor coactivator 7 (NCOA7), mRNA.<br>cdna:known chromosome:NCBI36:6:126144000:126293949:1 gene:ENSG00000111912<br>cdna:known-ccds chromosome:NCBI36:6:126144000:126293950:1 gene:ENSG00000111912 CCDS5132.1<br>cdna:Genscan chromosome:NCBI36:6:126217859:126285673:1 |

| | | | | | | | | | |
|---|---|---|---|---|---|---|---|---|---|
| | | | | | | | | | Homo sapiens mRNA; cDNA DKFZp761B2210 (from clone DKFZp761B2210). |
| 2924557 | 379795 | 2924514 | core | 126248007 | 126248142 | 6 | + | AB074157<br>ENSESTT00000024649<br>ENSESTT00000024650<br>AK127512<br>NM_181782<br>ENST00000355470<br>ENST00000368357<br>GENSCAN00000062579<br>ENSESTT00000024651<br>ENSESTT00000024648<br>AL834442 | Homo sapiens primary neuroblastoma cDNA, clone:Nbla10993, full insert sequence.<br><br><br>Homo sapiens cDNA FLJ45605 fis, clone BRTHA3021971, moderately similar to Homo sapiens oxidation resistance 1 (OXR1).<br>Homo sapiens nuclear receptor coactivator 7 (NCOA7), mRNA.<br>cdna:known chromosome:NCBI36:6:126144000:126293949:1 gene:ENSG00000111912<br>cdna:known-ccds chromosome:NCBI36:6:126144000:126293950:1 gene:ENSG00000111912 CCDS5132.1<br>cdna:Genscan chromosome:NCBI36:6:126217859:126285673:1<br><br><br>Homo sapiens mRNA; cDNA DKFZp761B2210 (from clone DKFZp761B2210). |
| 2924567 | 379799 | 2924514 | core | 126253521 | 126253642 | 6 | + | NM_181782<br>ENST00000355470<br>GENSCAN00000062579<br>AL834442<br>AK127512<br>ENST00000368357<br>ENSESTT00000024652<br>ENSESTT00000024653<br>AK094706<br>ENST00000368353<br>ENST00000229634<br>GENSCAN00000001622 | Homo sapiens nuclear receptor coactivator 7 (NCOA7), mRNA.<br>cdna:known chromosome:NCBI36:6:126144000:126293949:1 gene:ENSG00000111912<br>cdna:Genscan chromosome:NCBI36:6:126217859:126285673:1<br>Homo sapiens mRNA; cDNA DKFZp761B2210 (from clone DKFZp761B2210).<br>Homo sapiens cDNA FLJ45605 fis, clone BRTHA3021971, moderately similar to Homo sapiens oxidation resistance 1 (OXR1).<br>cdna:known-ccds chromosome:NCBI36:6:126144000:126293950:1 gene:ENSG00000111912 CCDS5132.1<br><br><br>Homo sapiens cDNA FLJ37387 fis, clone BRAMY2026685, moderately similar to Homo sapiens oxidation protection protein (OXR1) mRNA.<br>cdna:known chromosome:NCBI36:6:126262835:126293949:1 gene:ENSG00000111912<br>cdna:known chromosome:NCBI36:6:126282171:126293947:1 gene:ENSG00000111912<br>cdna:Genscan chromosome:NCBI36:6:126290502:126291610:1 |
| 2925308 | 380256 | 2925237 | core | 129555532 | 129555632 | 6 | + | ENST00000368151<br>NM_000426<br>ENST00000355250<br>ENST00000354729<br>ENST00000358023<br>ENSESTT00000041984<br>GENSCAN00000015946<br>GENSCAN00000057316<br>GENSCAN00000053420<br>ENSESTT00000041989<br>ENSESTT00000041988<br>ENSESTT00000041992<br>ENSESTT00000041993 | cdna:novel chromosome:NCBI36:6:129246035:129879401:1 gene:ENSG00000196569<br>Homo sapiens laminin, alpha 2 (merosin, congenital muscular dystrophy) (LAMA2), mRNA.<br>cdna:known chromosome:NCBI36:6:129246035:129879401:1 gene:ENSG00000196569<br>cdna:known-ccds chromosome:NCBI36:6:129246035:129879407:1 gene:ENSG00000196569 CCDS5138.1<br>cdna:novel chromosome:NCBI36:6:129246035:129879401:1 gene:ENSG00000196569<br><br>cdna:Genscan chromosome:NCBI36:6:129539339:129555691:1<br>cdna:Genscan chromosome:NCBI36:6:129675699:129801600:1<br>cdna:Genscan chromosome:NCBI36:6:129803654:129879185:1 |
| 2929345 | 382825 | 2929168 | core | 145191649 | 145191675 | 6 | + | GENSCAN00000027507<br>ENST00000367529<br>ENST00000367524<br>ENST00000367545<br>ENST00000367526<br>ENST00000367528<br>NM_007124<br>ENSESTT00000008743<br>ENST00000367523<br>ENST00000282749<br>ENSESTT00000008745<br>ENSESTT00000008744<br>ENSESTT00000008746 | cdna:Genscan chromosome:NCBI36:6:145173381:145245447:1<br>cdna:known chromosome:NCBI36:6:144707040:145209660:1 gene:ENSG00000152818<br>cdna:known chromosome:NCBI36:6:145152021:145209660:1 gene:ENSG00000152818<br>cdna:known chromosome:NCBI36:6:144654658:145215863:1 gene:ENSG00000152818<br>cdna:known chromosome:NCBI36:6:144946286:145209660:1 gene:ENSG00000152818<br>cdna:known chromosome:NCBI36:6:144946039:145213832:1 gene:ENSG00000152818<br>Homo sapiens utrophin (homologous to dystrophin) (UTRN), mRNA.<br><br>cdna:known chromosome:NCBI36:6:145162283:145209660:1 gene:ENSG00000152818<br>cdna:known chromosome:NCBI36:6:144654658:145209662:1 gene:ENSG00000152818 |

| | | | | | | | | | |
|---|---|---|---|---|---|---|---|---|---|
| 2931416 | 384154 | 2931391 | core | 151261795 | 151261825 | 6 | + | AY374131<br>ENSESTT00000036222<br>ENSESTT00000036221<br>ENST00000367308<br>ENSESTT00000036224<br>ENSESTT00000036223<br>ENST00000367307<br>ENST00000367321<br>ENST00000367310<br>ENST00000265365<br>NM_015440<br>GENSCAN00000056713<br>AK127089 | Homo sapiens truncated C1-tetrahydrofolate synthase mRNA, complete cds; nuclear gene for mitochondrial product; alternatively spliced.<br><br><br>cdna:known chromosome:NCBI36:6:151228384:151275534:1 gene:ENSG00000120254<br><br><br>cdna:known chromosome:NCBI36:6:151228545:151261850:1 gene:ENSG00000120254<br>cdna:known-ccds chromosome:NCBI36:6:151228378:151464716:1 gene:ENSG00000120254 CCDS5228.1<br>cdna:known chromosome:NCBI36:6:151228384:151404669:1 gene:ENSG00000120254<br>cdna:known chromosome:NCBI36:6:151228384:151464714:1 gene:ENSG00000120254<br>Homo sapiens methylenetetrahydrofolate dehydrogenase (NADP+ dependent) 1-like (MTHFD1L), mRNA.<br>cdna:Genscan chromosome:NCBI36:6:151240481:151281497:1<br>Homo sapiens cDNA FLJ45146 fis, clone BRAWH3041556, moderately similar to C-1-tetrahydrofolate synthase, cytoplasmic. |
| 2946117 | 393535 | 2946106 | core | 25958065 | 25958149 | 6 | - | BC017952<br>ENST00000308453<br>NM_006632<br>GENSCAN00000040440<br>ENST00000361703<br>ENST00000360657<br>ENST00000362070 | Homo sapiens solute carrier family 17 (sodium phosphate), member 3, mRNA (cDNA clone MGC:24061 IMAGE:4557795), complete cds.<br>cdna:known chromosome:NCBI36:6:25953312:25970212:-1 gene:ENSG00000124564<br>Homo sapiens solute carrier family 17 (sodium phosphate), member 3 (SLC17A3), mRNA.<br>cdna:Genscan chromosome:NCBI36:6:25906992:25976594:-1<br>cdna:known chromosome:NCBI36:6:25953312:25990501:-1 gene:ENSG00000124564<br>cdna:known chromosome:NCBI36:6:25953307:25990493:-1 gene:ENSG00000124564<br>cdna:known-ccds chromosome:NCBI36:6:25953312:25990501:-1 gene:ENSG00000124564 CCDS4566.1 |
| 2949627 | 395415 | 2949622 | core | 32117529 | 32117663 | 6 | - | ENSESTT00000021528<br>ENST00000342991<br>ENST00000383317<br>NM_019105<br>NM_032470<br>ENST00000375247<br>ENST00000375244<br>ENSESTT00000021529<br>BC071883<br>ENSESTT00000021530<br>GENSCAN00000024674<br>NR_001284<br>ENST00000299669<br>GENSCAN00000045300 | <br>cdna:known-ccds chromosome:NCBI36:6:32116909:32121883:-1 gene:ENSG00000168477 CCDS4736.1<br>cdna:known chromosome:NCBI36:c6_QBL:32109441:32116266:-1 gene:ENSG00000206336<br>Homo sapiens tenascin XB (TNXB), transcript variant XB, mRNA.<br>Homo sapiens tenascin XB (TNXB), transcript variant XB-S, mRNA.<br>cdna:known chromosome:NCBI36:6:32084175:32185131:-1 gene:ENSG00000168477<br>cdna:known chromosome:NCBI36:6:32116912:32185129:-1 gene:ENSG00000168477<br><br>Homo sapiens tenascin XB, mRNA (cDNA clone IMAGE:6571962), partial cds.<br><br>cdna:Genscan chromosome:NCBI36:6:32117105:32158081:-1<br>Homo sapiens tenascin XA pseudogene (TNXA) on chromosome 6.<br>cdna:known chromosome:NCBI36:6:32117105:32185131:-1 gene:ENSG00000168477<br>cdna:Genscan chromosome:NCBI36:c6_QBL:32109636:32155653:-1 |
| 2949629 | 395417 | 2949622 | core | 32118089 | 32118118 | 6 | - | ENST00000375244<br>ENST00000299669<br>BC071883<br>GENSCAN00000024674<br>ENSESTT00000021528<br>ENST00000383317<br>GENSCAN00000045300<br>ENST00000342991<br>NM_019105<br>ENST00000375247<br>NR_001284<br>NM_032470<br>ENST00000383160<br>ENST00000383158<br>ENST00000308994<br>ENST00000383159 | cdna:known chromosome:NCBI36:6:32116912:32185129:-1 gene:ENSG00000168477<br>cdna:known chromosome:NCBI36:6:32117105:32185131:-1 gene:ENSG00000168477<br>Homo sapiens tenascin XB, mRNA (cDNA clone IMAGE:6571962), partial cds.<br>cdna:Genscan chromosome:NCBI36:6:32117105:32158081:-1<br><br>cdna:known chromosome:NCBI36:c6_QBL:32109441:32116266:-1 gene:ENSG00000206336<br>cdna:Genscan chromosome:NCBI36:c6_QBL:32109636:32155653:-1<br>cdna:known-ccds chromosome:NCBI36:6:32116909:32121883:-1 gene:ENSG00000168477 CCDS4736.1<br>Homo sapiens tenascin XB (TNXB), transcript variant XB, mRNA.<br>cdna:known chromosome:NCBI36:6:32084175:32185131:-1 gene:ENSG00000168477<br>Homo sapiens tenascin XA pseudogene (TNXA) on chromosome 6.<br>Homo sapiens tenascin XB (TNXB), transcript variant XB-S, mRNA.<br>cdna:known chromosome:NCBI36:c6_COX:32113049:32137454:-1 gene:ENSG00000206258<br>cdna:known chromosome:NCBI36:c6_COX:32121537:32152842:-1 gene:ENSG00000206258<br>cdna:known chromosome:NCBI36:6:32133814:32165140:-1 gene:ENSG00000168477<br>cdna:known chromosome:NCBI36:c6_COX:32121537:32152869:-1 gene:ENSG00000206258 |

| | | | | | | | | | |
|---|---|---|---|---|---|---|---|---|---|
| | | | | | | | | ENST00000383313<br>Y17865<br>GENSCAN00000042608 | cdna:known chromosome:NCBI36:c6_QBL:32133367:32149681:-1 gene:ENSG00000206332<br>Homo sapiens mRNA for tenascin-X (partial), fibronectin type III repeats ho-h4.<br>cdna:Genscan chromosome:NCBI36:6:32160168:32164803:-1 |
| 2949636 | 395422 | 2949622 | core | 32119212 | 32119310 | 6 | - | NR_001284<br>ENST00000299669<br>GENSCAN00000045300<br>GENSCAN00000024658<br>GENSCAN00000024674<br>NM_019105<br>NM_032470<br>BC071883<br>ENSESTT00000021527<br>ENST00000342991<br>ENST00000383317<br>ENST00000375247<br>ENST00000375244<br>ENSESTT00000021528<br>ENSESTT00000021526 | Homo sapiens tenascin XA pseudogene (TNXA) on chromosome 6.<br>cdna:known chromosome:NCBI36:6:32117105:32185131:-1 gene:ENSG00000168477<br>cdna:Genscan chromosome:NCBI36:c6_QBL:32109636:32155653:-1<br>cdna:Genscan chromosome:NCBI36:6:32084369:32088120:-1<br>cdna:Genscan chromosome:NCBI36:6:32117105:32158081:-1<br>Homo sapiens tenascin XB (TNXB), transcript variant XB, mRNA.<br>Homo sapiens tenascin XB (TNXB), transcript variant XB-S, mRNA.<br>Homo sapiens tenascin XB, mRNA (cDNA clone IMAGE:6571962), partial cds.<br><br>cdna:known-ccds chromosome:NCBI36:6:32116909:32121883:-1 gene:ENSG00000168477 CCDS4736.1<br>cdna:known chromosome:NCBI36:c6_QBL:32109441:32116266:-1 gene:ENSG00000206336<br>cdna:known chromosome:NCBI36:6:32084175:32185131:-1 gene:ENSG00000168477<br>cdna:known chromosome:NCBI36:6:32116912:32185129:-1 gene:ENSG00000168477 |
| 2949643 | 395425 | 2949622 | core | 32120983 | 32121025 | 6 | - | NM_032470<br>ENSESTT00000021525<br>ENST00000342991<br>NM_019105<br>BC071883<br>ENST00000383317<br>ENST00000375247<br>ENST00000375244<br>ENST00000299669<br>GENSCAN00000045300<br>GENSCAN00000024674<br>NR_001284<br>ENSESTT00000021529<br>ENSESTT00000021530<br>GENSCAN00000024658<br>ENSESTT00000021528<br>ENSESTT00000021526<br>ENSESTT00000021527<br>ENST00000359300<br>ENST00000383161 | Homo sapiens tenascin XB (TNXB), transcript variant XB-S, mRNA.<br><br>cdna:known-ccds chromosome:NCBI36:6:32116909:32121883:-1 gene:ENSG00000168477 CCDS4736.1<br>Homo sapiens tenascin XB (TNXB), transcript variant XB, mRNA.<br>Homo sapiens tenascin XB, mRNA (cDNA clone IMAGE:6571962), partial cds.<br>cdna:known chromosome:NCBI36:c6_QBL:32109441:32116266:-1 gene:ENSG00000206336<br>cdna:known chromosome:NCBI36:6:32084175:32185131:-1 gene:ENSG00000168477<br>cdna:known chromosome:NCBI36:6:32116912:32185129:-1 gene:ENSG00000168477<br>cdna:known chromosome:NCBI36:6:32117105:32185131:-1 gene:ENSG00000168477<br>cdna:Genscan chromosome:NCBI36:c6_QBL:32109636:32155653:-1<br>cdna:Genscan chromosome:NCBI36:6:32117105:32158081:-1<br>Homo sapiens tenascin XA pseudogene (TNXA) on chromosome 6.<br><br><br>cdna:Genscan chromosome:NCBI36:6:32084369:32088120:-1<br><br><br><br>cdna:known chromosome:NCBI36:6:32086545:32088297:-1 gene:ENSG00000198493<br>cdna:novel chromosome:NCBI36:c6_COX:32107318:32108874:-1 gene:ENSG00000206259 |
| 2949684 | 395445 | 2949622 | core | 32145900 | 32146043 | 6 | - | ENSESTT00000021523<br>ENST00000383160<br>ENST00000375247<br>ENST00000375244<br>GENSCAN00000045300<br>GENSCAN00000024674<br>ENST00000383159<br>ENST00000383313<br>ENST00000308994<br>ENST00000383158<br>ENST00000299669<br>NM_019105 | <br>cdna:known chromosome:NCBI36:c6_COX:32113049:32137454:-1 gene:ENSG00000206258<br>cdna:known chromosome:NCBI36:6:32084175:32185131:-1 gene:ENSG00000168477<br>cdna:known chromosome:NCBI36:6:32116912:32185129:-1 gene:ENSG00000168477<br>cdna:Genscan chromosome:NCBI36:c6_QBL:32109636:32155653:-1<br>cdna:Genscan chromosome:NCBI36:6:32117105:32158081:-1<br>cdna:known chromosome:NCBI36:c6_COX:32121537:32152869:-1 gene:ENSG00000206258<br>cdna:known chromosome:NCBI36:c6_QBL:32133367:32149681:-1 gene:ENSG00000206332<br>cdna:known chromosome:NCBI36:6:32133814:32165140:-1 gene:ENSG00000168477<br>cdna:known chromosome:NCBI36:c6_COX:32121537:32152842:-1 gene:ENSG00000206258<br>cdna:known chromosome:NCBI36:6:32117105:32185131:-1 gene:ENSG00000168477<br>Homo sapiens tenascin XB (TNXB), transcript variant XB, mRNA. |
| 2949688 | 395448 | 2949622 | core | 32149432 | 32149580 | 6 | - | ENSESTT00000021523<br>ENST00000383160<br>GENSCAN00000045300 | <br>cdna:known chromosome:NCBI36:c6_COX:32113049:32137454:-1 gene:ENSG00000206258<br>cdna:Genscan chromosome:NCBI36:c6_QBL:32109636:32155653:-1 |

| | | | | | | | | | |
|---|---|---|---|---|---|---|---|---|---|
| | | | | | | | | ENST00000375247<br>ENST00000375244<br>GENSCAN00000024674<br>ENST00000383159<br>ENST00000383313<br>ENST00000308994<br>ENST00000383158<br>NM_019105<br>ENST00000299669<br>Y17867<br>ENST00000383316 | cdna:known chromosome:NCBI36:6:32084175:32185131:-1 gene:ENSG00000168477<br>cdna:known chromosome:NCBI36:6:32116912:32185129:-1 gene:ENSG00000168477<br>cdna:Genscan chromosome:NCBI36:6:32117105:32158081:-1<br>cdna:known chromosome:NCBI36:c6_COX:32121537:32152869:-1 gene:ENSG00000206258<br>cdna:known chromosome:NCBI36:c6_QBL:32133367:32149681:-1 gene:ENSG00000206332<br>cdna:known chromosome:NCBI36:6:32133814:32165140:-1 gene:ENSG00000168477<br>cdna:known chromosome:NCBI36:c6_COX:32121537:32152842:-1 gene:ENSG00000206258<br>Homo sapiens tenascin XB (TNXB), transcript variant XB, mRNA.<br>cdna:known chromosome:NCBI36:6:32117105:32185131:-1 gene:ENSG00000168477<br>Homo sapiens mRNA for tenascin-X (partial), fibronectin type III repeats h13-h16 and h13a.<br>cdna:known chromosome:NCBI36:c6_QBL:32125144:32133346:-1 gene:ENSG00000206333 |
| 2949695 | 395453 | 2949622 | core | 32157814 | 32158047 | 6 | - | ENST00000299669<br>NR_001284<br>GENSCAN00000024674<br>GENSCAN00000045300<br>NM_019105<br>ENST00000383158<br>ENST00000375244<br>ENST00000375247<br>ENST00000308994<br>ENST00000383159 | cdna:known chromosome:NCBI36:6:32117105:32185131:-1 gene:ENSG00000168477<br>Homo sapiens tenascin XA pseudogene (TNXA) on chromosome 6.<br>cdna:Genscan chromosome:NCBI36:6:32117105:32158081:-1<br>cdna:Genscan chromosome:NCBI36:c6_QBL:32109636:32155653:-1<br>Homo sapiens tenascin XB (TNXB), transcript variant XB, mRNA.<br>cdna:known chromosome:NCBI36:c6_COX:32121537:32152842:-1 gene:ENSG00000206258<br>cdna:known chromosome:NCBI36:6:32116912:32185129:-1 gene:ENSG00000168477<br>cdna:known chromosome:NCBI36:6:32084175:32185131:-1 gene:ENSG00000168477<br>cdna:known chromosome:NCBI36:6:32133814:32165140:-1 gene:ENSG00000168477<br>cdna:known chromosome:NCBI36:c6_COX:32121537:32152869:-1 gene:ENSG00000206258 |
| 2949696 | 395454 | 2949622 | core | 32160204 | 32160403 | 6 | - | GENSCAN00000042608<br>GENSCAN00000045300<br>ENST00000308994<br>ENST00000375244<br>Y17865<br>ENST00000383159<br>NM_019105<br>ENST00000383158<br>ENST00000375247<br>ENST00000299669 | cdna:Genscan chromosome:NCBI36:6:32160168:32164803:-1<br>cdna:Genscan chromosome:NCBI36:c6_QBL:32109636:32155653:-1<br>cdna:known chromosome:NCBI36:6:32133814:32165140:-1 gene:ENSG00000168477<br>cdna:known chromosome:NCBI36:6:32116912:32185129:-1 gene:ENSG00000168477<br>Homo sapiens mRNA for tenascin-X (partial), fibronectin type III repeats ho-h4.<br>cdna:known chromosome:NCBI36:c6_COX:32121537:32152869:-1 gene:ENSG00000206258<br>Homo sapiens tenascin XB (TNXB), transcript variant XB, mRNA.<br>cdna:known chromosome:NCBI36:c6_COX:32121537:32152842:-1 gene:ENSG00000206258<br>cdna:known chromosome:NCBI36:6:32084175:32185131:-1 gene:ENSG00000168477<br>cdna:known chromosome:NCBI36:6:32117105:32185131:-1 gene:ENSG00000168477 |
| 2949723 | 395466 | 2949622 | core | 32173922 | 32173950 | 6 | - | X71923<br>U52696<br>ENST00000375247<br>ENST00000375244<br>ENST00000383160<br>NM_019105<br>ENST00000299669<br>ENST00000308994<br>ENST00000383159<br>GENSCAN00000024674<br>GENSCAN00000045300<br>ENST00000383316<br>Y17867<br>ENST00000383158<br>ENSESTT00000021521<br>ENST00000375192<br>ENSESTT00000021520<br>ENSESTT00000021519<br>ENSESTT00000021515<br>ENSESTT00000021518<br>ENST00000375201<br>ENST00000375203 | H.sapiens XB gene for tenascin-X, exons 1 & 2.<br>Human adrenal Creb-rp homolog (Creb-rp), complete cds, and tenascin-X (XB), partial cds, mRNA.<br>cdna:known chromosome:NCBI36:6:32084175:32185131:-1 gene:ENSG00000168477<br>cdna:known chromosome:NCBI36:6:32116912:32185129:-1 gene:ENSG00000168477<br>cdna:known chromosome:NCBI36:c6_COX:32113049:32137454:-1 gene:ENSG00000206258<br>Homo sapiens tenascin XB (TNXB), transcript variant XB, mRNA.<br>cdna:known chromosome:NCBI36:6:32117105:32185131:-1 gene:ENSG00000168477<br>cdna:known chromosome:NCBI36:6:32133814:32165140:-1 gene:ENSG00000168477<br>cdna:known chromosome:NCBI36:c6_COX:32121537:32152869:-1 gene:ENSG00000206258<br>cdna:Genscan chromosome:NCBI36:6:32117105:32158081:-1<br>cdna:Genscan chromosome:NCBI36:c6_QBL:32109636:32155653:-1<br>cdna:known chromosome:NCBI36:c6_QBL:32125144:32133346:-1 gene:ENSG00000206333<br>Homo sapiens mRNA for tenascin-X (partial), fibronectin type III repeats h13-h16 and h13a.<br>cdna:known chromosome:NCBI36:c6_COX:32121537:32152842:-1 gene:ENSG00000206258<br><br>cdna:known chromosome:NCBI36:6:32191025:32203995:-1 gene:ENSG00000168477<br><br><br><br><br>cdna:known chromosome:NCBI36:6:32191023:32204008:-1 gene:ENSG00000168477<br>cdna:known-ccds chromosome:NCBI36:6:32191023:32204008:-1 gene:ENSG00000168477 CCDS4737.1 |

| | | | | | | | | | |
|---|---|---|---|---|---|---|---|---|---|
| | | | | | | | | ENST00000293709<br>ENST00000375195<br>ENSESTT00000021517<br>ENST00000383156<br>ENSESTT00000021516<br>NM_004381 | cdna:known chromosome:NCBI36:c6_COX:32178759:32191727:-1 gene:ENSG00000168468<br>cdna:known chromosome:NCBI36:6:32191025:32203995:-1 gene:ENSG00000168477<br><br>cdna:known chromosome:NCBI36:c6_COX:32178759:32191727:-1 gene:ENSG00000168468<br><br>Homo sapiens cAMP responsive element binding protein-like 1 (CREBL1), mRNA. |
| 2949734 | 395474 | 2949622 | core | 32192815 | 32192845 | 6 | - | NM_004381<br>ENSESTT00000021520<br>ENST00000375203<br>ENST00000293709<br>ENST00000383156<br>ENST00000375201<br>ENST00000375195<br>ENST00000375192<br>U52696<br>ENSESTT00000021515<br>ENSESTT00000021516<br>ENSESTT00000021517<br>ENSESTT00000021518<br>ENSESTT00000021519<br>BC008394<br>ENST00000375190 | Homo sapiens cAMP responsive element binding protein-like 1 (CREBL1), mRNA.<br><br>cdna:known-ccds chromosome:NCBI36:6:32191023:32204008:-1 gene:ENSG00000168477 CCDS4737.1<br>cdna:known chromosome:NCBI36:c6_COX:32178759:32191727:-1 gene:ENSG00000168468<br>cdna:known chromosome:NCBI36:c6_COX:32178759:32191727:-1 gene:ENSG00000168468<br>cdna:known chromosome:NCBI36:6:32191023:32204008:-1 gene:ENSG00000168477<br>cdna:known chromosome:NCBI36:6:32191025:32203995:-1 gene:ENSG00000168477<br>cdna:known chromosome:NCBI36:6:32191025:32203995:-1 gene:ENSG00000168477<br>Human adrenal Creb-rp homolog (Creb-rp), complete cds, and tenascin-X (XB), partial cds, mRNA.<br><br><br><br><br><br>Homo sapiens cAMP responsive element binding protein-like 1, mRNA (cDNA clone IMAGE:4296455),<br>complete cds.<br>cdna:known chromosome:NCBI36:6:32196197:32204008:-1 gene:ENSG00000168477 |
| 2949752 | 395484 | 2949622 | core | 32201892 | 32201948 | 6 | - | ENSESTT00000021518<br>ENST00000375203<br>ENST00000375201<br>NM_004381<br>ENST00000293709<br>ENST00000383156<br>ENST00000375195<br>ENST00000375192<br>ENST00000375190<br>ENSESTT00000021519<br>U52696<br>ENSESTT00000021520<br>ENSESTT00000021517<br>ENSESTT00000021516<br>ENSESTT00000021515<br>BC008394 | <br>cdna:known-ccds chromosome:NCBI36:6:32191023:32204008:-1 gene:ENSG00000168477 CCDS4737.1<br>cdna:known chromosome:NCBI36:6:32191023:32204008:-1 gene:ENSG00000168477<br>Homo sapiens cAMP responsive element binding protein-like 1 (CREBL1), mRNA.<br>cdna:known chromosome:NCBI36:c6_COX:32178759:32191727:-1 gene:ENSG00000168468<br>cdna:known chromosome:NCBI36:c6_COX:32178759:32191727:-1 gene:ENSG00000168468<br>cdna:known chromosome:NCBI36:6:32191025:32203995:-1 gene:ENSG00000168477<br>cdna:known chromosome:NCBI36:6:32191025:32203995:-1 gene:ENSG00000168477<br>cdna:known chromosome:NCBI36:6:32196197:32204008:-1 gene:ENSG00000168477<br><br>Human adrenal Creb-rp homolog (Creb-rp), complete cds, and tenascin-X (XB), partial cds, mRNA.<br><br><br><br><br>Homo sapiens cAMP responsive element binding protein-like 1, mRNA (cDNA clone IMAGE:4296455),<br>complete cds. |
| 2949754 | 395486 | 2949622 | core | 32203201 | 32203228 | 6 | - | ENST00000375190<br>ENSESTT00000021516<br>ENST00000375195<br>BC008394<br>ENST00000375201<br>ENST00000375203<br>ENST00000293709<br>NM_004381<br>ENSESTT00000021515 | cdna:known chromosome:NCBI36:6:32196197:32204008:-1 gene:ENSG00000168477<br><br>cdna:known chromosome:NCBI36:6:32191025:32203995:-1 gene:ENSG00000168477<br>Homo sapiens cAMP responsive element binding protein-like 1, mRNA (cDNA clone IMAGE:4296455),<br>complete cds.<br>cdna:known chromosome:NCBI36:6:32191023:32204008:-1 gene:ENSG00000168477<br>cdna:known-ccds chromosome:NCBI36:6:32191023:32204008:-1 gene:ENSG00000168477 CCDS4737.1<br>cdna:known chromosome:NCBI36:c6_COX:32178759:32191727:-1 gene:ENSG00000168468<br>Homo sapiens cAMP responsive element binding protein-like 1 (CREBL1), mRNA. |
| 2949758 | 395489 | 2949622 | core | 32203888 | 32203953 | 6 | - | BC008394<br>U52696<br>ENSESTT00000021515 | Homo sapiens cAMP responsive element binding protein-like 1, mRNA (cDNA clone IMAGE:4296455),<br>complete cds.<br>Human adrenal Creb-rp homolog (Creb-rp), complete cds, and tenascin-X (XB), partial cds, mRNA. |

| | | | | | | | | | |
|---|---|---|---|---|---|---|---|---|---|
| | | | | | | | | ENST00000375203<br>ENST00000375201<br>ENST00000375195<br>NM_004381<br>ENST00000293709<br>ENST00000383156<br>ENST00000375192<br>ENSESTT00000021516<br>ENSESTT00000021517<br>ENSESTT00000021518<br>ENST00000375190<br>ENSESTT00000021520<br>ENSESTT00000021519 | cdna:known-ccds chromosome:NCBI36:6:32191023:32204008:-1 gene:ENSG00000168477 CCDS4737.1<br>cdna:known chromosome:NCBI36:6:32191023:32204008:-1 gene:ENSG00000168477<br>cdna:known chromosome:NCBI36:6:32191025:32203995:-1 gene:ENSG00000168477<br>Homo sapiens cAMP responsive element binding protein-like 1 (CREBL1), mRNA.<br>cdna:known chromosome:NCBI36:c6_COX:32178759:32191727:-1 gene:ENSG00000168468<br>cdna:known chromosome:NCBI36:c6_COX:32178759:32191727:-1 gene:ENSG00000168468<br>cdna:known chromosome:NCBI36:6:32191025:32203995:-1 gene:ENSG00000168477<br>cdna:known chromosome:NCBI36:6:32196197:32204008:-1 gene:ENSG00000168477 |
| 2955707 | 399061 | 2955691 | core | 46322572 | 46322597 | 6 | - | ENST00000306764<br>NM_005822<br>AK090990<br>ENST00000371373<br>ENST00000330430<br>ENST00000371374<br>BC038509<br>ENST00000371371<br>GENSCAN00000000877<br>ENSESTT00000022983<br>ENSESTT00000022984<br>AY034085 | cdna:known chromosome:NCBI36:6:46296434:46567668:-1 gene:ENSG00000172348<br>Homo sapiens Down syndrome critical region gene 1-like 1 (DSCR1L1), mRNA.<br>Homo sapiens cDNA FLJ33671 fis, clone BRAWH2001459, highly similar to ZAKI-4 PROTEIN.<br>cdna:known chromosome:NCBI36:6:46296434:46401364:-1 gene:ENSG00000172348<br>cdna:known chromosome:NCBI36:6:46296434:46401364:-1 gene:ENSG00000172348<br>cdna:known chromosome:NCBI36:6:46296434:46567058:-1 gene:ENSG00000172348<br>Homo sapiens Down syndrome critical region gene 1-like 1, mRNA (cDNA clone MGC:41855 IMAGE:5259147), complete cds.<br>cdna:known chromosome:NCBI36:6:46298837:46532672:-1 gene:ENSG00000172348<br>cdna:Genscan chromosome:NCBI36:6:46298837:46326599:-1<br>Homo sapiens calcineurin inhibitor ZAKI-4 beta splice variant 1 mRNA, complete cds, alternatively spliced. |
| 2957058 | 399918 | 2956904 | core | 52026806 | 52026890 | 6 | - | NM_170724<br>ENST00000371111<br>ENST00000340994<br>AK128031<br>ENST00000304707<br>GENSCAN00000043267<br>NM_138694<br>ENST00000371117<br>GENSCAN00000067896 | Homo sapiens polycystic kidney and hepatic disease 1 (autosomal recessive) (PKHD1), transcript variant 2, mRNA.<br>cdna:known chromosome:NCBI36:6:51693606:52060367:-1 gene:ENSG00000170927<br>cdna:known-ccds chromosome:NCBI36:6:51693606:52060382:-1 gene:ENSG00000170927 CCDS4936.1<br>Homo sapiens cDNA FLJ46150 fis, clone TESTI4000703.<br>cdna:known chromosome:NCBI36:6:51588104:52060382:-1 gene:ENSG00000170927<br>cdna:Genscan chromosome:NCBI36:6:51832989:51945708:-1<br>Homo sapiens polycystic kidney and hepatic disease 1 (autosomal recessive) (PKHD1), transcript variant 1, mRNA.<br>cdna:known-ccds chromosome:NCBI36:6:51588057:52060382:-1 gene:ENSG00000170927 CCDS4935.1<br>cdna:Genscan chromosome:NCBI36:6:51975268:52038834:-1 |
| 2958257 | 400654 | 2958232 | core | 56050876 | 56050944 | 6 | - | ENST00000370808<br>AF330693<br>ENST00000370817<br>AF370383<br>GENSCAN00000048286<br>ENST00000244728<br>AK096444<br>ENSESTT00000016320<br>ENSESTT00000016321<br>NM_030820<br>ENST00000370819<br>ENST00000370811 | cdna:known chromosome:NCBI36:6:56029348:56098365:-1 gene:ENSG00000124749<br>Homo sapiens alpha 1 chain-like collagen COLA1L precursor (COLA1L) mRNA, complete cds.<br>cdna:known chromosome:NCBI36:6:56029347:56220301:-1 gene:ENSG00000124749<br>Homo sapiens FP633 mRNA, complete cds.<br>cdna:Genscan chromosome:NCBI36:6:56030414:56050330:-1<br>cdna:novel chromosome:NCBI36:6:56029348:56220303:-1 gene:ENSG00000124749<br>Homo sapiens cDNA FLJ39125 fis, clone NTONG2007034.<br>Homo sapiens collagen, type XXI, alpha 1 (COL21A1), mRNA.<br>cdna:known chromosome:NCBI36:6:56029347:56366851:-1 gene:ENSG00000124749<br>cdna:novel chromosome:NCBI36:6:56029348:56220303:-1 gene:ENSG00000124749 |
| 2960175 | 401857 | 2960146 | core | 71019810 | 71019845 | 6 | - | AK125738<br>ENST00000358737<br>ENSESTT00000031688 | Homo sapiens cDNA FLJ43750 fis, clone TESTI2034767, moderately similar to Homo sapiens collagen type IX alpha 1 chain (COL9A1) gene.<br>cdna:known chromosome:NCBI36:6:70982529:71069494:-1 gene:ENSG00000112280 |

| | | | | | | | | | |
|---|---|---|---|---|---|---|---|---|---|
| | | | | | | | | ENST00000370499<br>AK097582<br>ENST00000357250<br>ENSESTT00000031686<br>ENST00000356945<br>ENST00000320755<br>NM_001851<br>NM_078485<br>ENSESTT00000031687<br>GENSCAN00000034818<br>ENSESTT00000031683 | cdna:novel chromosome:NCBI36:6:70982529:71069494:-1 gene:ENSG00000112280<br>Homo sapiens cDNA FLJ40263 fis, clone TESTI2026218, highly similar to Homo sapiens collagen type IX alpha 1 chain (COL9A1) gene.<br>cdna:novel chromosome:NCBI36:6:70982529:71069494:-1 gene:ENSG00000112280<br>cdna:known-ccds chromosome:NCBI36:6:70982464:71069494:-1 gene:ENSG00000112280 CCDS4971.1<br>cdna:known chromosome:NCBI36:6:70982529:71049632:-1 gene:ENSG00000112280<br>Homo sapiens collagen, type IX, alpha 1 (COL9A1), transcript variant 1, mRNA.<br>Homo sapiens collagen, type IX, alpha 1 (COL9A1), transcript variant 2, mRNA.<br>cdna:Genscan chromosome:NCBI36:6:71008388:71049476:-1 |
| 2961188 | 402453 | 2961177 | core | 75854027 | 75854174 | 6 | - | ENSESTT00000016598<br>ENSESTT00000016601<br>ENST00000265379<br>ENST00000322507<br>NM_004370<br>ENST00000370134<br>NM_080645<br>ENST00000345356<br>ENST00000370099<br>GENSCAN00000020472<br>ENSESTT00000016600<br>ENSESTT00000016599<br>ENSESTT00000016597<br>ENSESTT00000016591<br>ENSESTT00000016589 | cdna:known chromosome:NCBI36:6:75853714:75972258:-1 gene:ENSG00000111799<br>cdna:known chromosome:NCBI36:6:75850763:75972290:-1 gene:ENSG00000111799<br>Homo sapiens collagen, type XII, alpha 1 (COL12A1), transcript variant long, mRNA.<br>cdna:known chromosome:NCBI36:6:75850762:75972290:-1 gene:ENSG00000111799<br>Homo sapiens collagen, type XII, alpha 1 (COL12A1), transcript variant short, mRNA.<br>cdna:known chromosome:NCBI36:6:75850763:75972290:-1 gene:ENSG00000111799<br>cdna:known chromosome:NCBI36:6:75850763:75972290:-1 gene:ENSG00000111799<br>cdna:Genscan chromosome:NCBI36:6:75851626:75932215:-1 |
| 2961190 | 402455 | 2961177 | core | 75856620 | 75856703 | 6 | - | ENST00000370134<br>NM_080645<br>ENST00000345356<br>NM_004370<br>ENSESTT00000016598<br>ENST00000265379<br>ENST00000370099<br>ENST00000322507<br>GENSCAN00000020472<br>ENSESTT00000016599<br>ENSESTT00000016600<br>ENSESTT00000016592<br>GENSCAN00000060548<br>ENSESTT00000016589<br>ENSESTT00000012127<br>GENSCAN00000047938 | cdna:known chromosome:NCBI36:6:75850762:75972290:-1 gene:ENSG00000111799<br>Homo sapiens collagen, type XII, alpha 1 (COL12A1), transcript variant short, mRNA.<br>cdna:known chromosome:NCBI36:6:75850763:75972290:-1 gene:ENSG00000111799<br>Homo sapiens collagen, type XII, alpha 1 (COL12A1), transcript variant long, mRNA.<br>cdna:known chromosome:NCBI36:6:75853714:75972258:-1 gene:ENSG00000111799<br>cdna:known chromosome:NCBI36:6:75850763:75972290:-1 gene:ENSG00000111799<br>cdna:known chromosome:NCBI36:6:75850763:75972290:-1 gene:ENSG00000111799<br>cdna:Genscan chromosome:NCBI36:6:75851626:75932215:-1<br>cdna:Genscan chromosome:NCBI36:6:75941474:75947647:-1<br>cdna:Genscan chromosome:NCBI36:6:75949486:76072331:-1 |
| 2961268 | 402519 | 2961177 | core | 75954805 | 75954913 | 6 | - | ENST00000265379<br>GENSCAN00000047938<br>ENST00000370099<br>ENSESTT00000016598<br>GENSCAN00000020472<br>NM_004370<br>ENST00000345356<br>ENST00000322507<br>ENST00000370134 | cdna:known chromosome:NCBI36:6:75853714:75972258:-1 gene:ENSG00000111799<br>cdna:Genscan chromosome:NCBI36:6:75949486:76072331:-1<br>cdna:known chromosome:NCBI36:6:75850763:75972290:-1 gene:ENSG00000111799<br>cdna:Genscan chromosome:NCBI36:6:75851626:75932215:-1<br>Homo sapiens collagen, type XII, alpha 1 (COL12A1), transcript variant long, mRNA.<br>cdna:known chromosome:NCBI36:6:75850763:75972290:-1 gene:ENSG00000111799<br>cdna:known chromosome:NCBI36:6:75850763:75972290:-1 gene:ENSG00000111799<br>cdna:known chromosome:NCBI36:6:75850762:75972290:-1 gene:ENSG00000111799 |

| | | | | | | | | | |
|---|---|---|---|---|---|---|---|---|---|
| | | | | | | | | NM_080645<br>ENSESTT00000016600<br>ENSESTT00000016599<br>ENSESTT00000016597<br>ENSESTT00000016591<br>ENSESTT00000016589<br>ENST00000370089<br>ENST00000370081<br>ENST00000230459<br>NM_001865<br>ENSESTT00000012126<br>ENSESTT00000012125<br>ENST00000316076<br>ENST00000370050<br>NM_018247<br>AL832490<br>ENST00000230461 | Homo sapiens collagen, type XII, alpha 1 (COL12A1), transcript variant short, mRNA.<br><br><br><br><br><br>cdna:known chromosome:NCBI36:6:76004111:76010442:-1 gene:ENSG00000112695<br>cdna:known chromosome:NCBI36:6:76004247:76016759:-1 gene:ENSG00000112695<br>cdna:known chromosome:NCBI36:6:76004223:76010245:-1 gene:ENSG00000112695<br>Homo sapiens cytochrome c oxidase subunit VIIa polypeptide 2 (liver) (COX7A2), mRNA.<br><br><br>cdna:known chromosome:NCBI36:6:76019369:76051212:-1 gene:ENSG00000112697<br>cdna:known-ccds chromosome:NCBI36:6:76019360:76051221:-1 gene:ENSG00000112697 CCDS4983.1<br>Homo sapiens transmembrane protein 30A (TMEM30A), mRNA.<br>Homo sapiens mRNA; cDNA DKFZp686K1722 (from clone DKFZp686K1722).<br>cdna:known chromosome:NCBI36:6:76019369:76051212:-1 gene:ENSG00000112697 |
| 2976045 | 411859 | 2976041 | core | 137364584 | 137364640 | 6 | - | NM_014432<br>AK098312<br>GENSCAN00000040915<br>ENST00000367748<br>ENST00000316649<br>ENST00000367747 | Homo sapiens interleukin 20 receptor, alpha (IL20RA), mRNA.<br>Homo sapiens cDNA FLJ40993 fis, clone UTERU2015405, highly similar to Homo sapiens class II cytokine receptor ZCYTOR7 (ZCYTOR7) mRNA.<br>cdna:Genscan chromosome:NCBI36:6:137364388:137407558:-1<br>cdna:known chromosome:NCBI36:6:137362801:137407991:-1 gene:ENSG00000016402<br>cdna:known-ccds chromosome:NCBI36:6:137362801:137407991:-1 gene:ENSG00000016402 CCDS5181.1<br>cdna:known chromosome:NCBI36:6:137362801:137407794:-1 gene:ENSG00000016402 |
| 2978002 | 413044 | 2977949 | core | 146098197 | 146098224 | 6 | - | NM_001018041<br>AF454493<br>AF454494<br>ENSESTT00000001081<br>ENST00000367519<br>ENST00000324857<br>GENSCAN00000035160<br>AF454492<br>AK022721<br>ENST00000262907<br>NM_005670 | Homo sapiens epilepsy, progressive myoclonus type 2A, Lafora disease (laforin) (EPM2A), transcript variant 2, mRNA.<br>Homo sapiens clone FB4 laforin (EPM2A) mRNA, complete cds, alternatively spliced.<br>Homo sapiens truncated laforin isoform (EPM2A) mRNA, partial cds, alternatively spliced.<br><br>cdna:known-ccds chromosome:NCBI36:6:145988134:146098853:-1 gene:ENSG00000112425 CCDS5206.1<br>cdna:known chromosome:NCBI36:6:145988141:146098327:-1 gene:ENSG00000112425<br>cdna:Genscan chromosome:NCBI36:6:146097869:146098327:-1<br>Homo sapiens clone FB1 laforin (EPM2A) mRNA, complete cds, alternatively spliced.<br>Homo sapiens cDNA FLJ12659 fis, clone NT2RM4002161, highly similar to Homo sapiens laforin (EPM2A) mRNA.<br>cdna:known chromosome:NCBI36:6:145988141:146098684:-1 gene:ENSG00000112425<br>Homo sapiens epilepsy, progressive myoclonus type 2A, Lafora disease (laforin) (EPM2A), transcript variant 1, mRNA. |
| 2985828 | 417807 | 2985781 | core | 169371667 | 169371699 | 6 | - | ENSESTT00000047990<br>ENST00000366787<br>ENST00000335061<br>GENSCAN00000058308<br>NM_003247 | <br>cdna:known chromosome:NCBI36:6:169357800:169396064:-1 gene:ENSG00000186340<br>cdna:known chromosome:NCBI36:6:169357801:169396062:-1 gene:ENSG00000186340<br>cdna:Genscan chromosome:NCBI36:6:169359842:169392804:-1<br>Homo sapiens thrombospondin 2 (THBS2), mRNA. |
| 2991564 | 421420 | 2991395 | core | 18799549 | 18799582 | 7 | + | NM_178425<br>AK095820<br>ENST00000262069<br>NM_178423<br>NM_014707<br>NM_058176<br>NM_058177<br>AK097178<br>AY429540<br>ENSESTT00000047317 | Homo sapiens histone deacetylase 9 (HDAC9), transcript variant 5, mRNA.<br>Homo sapiens cDNA FLJ38501 fis, clone HCHON1000176, moderately similar to Histone deacetylase 5.<br>cdna:known chromosome:NCBI36:7:18502451:19002210:1 gene:ENSG00000048052<br>Homo sapiens histone deacetylase 9 (HDAC9), transcript variant 4, mRNA.<br>Homo sapiens histone deacetylase 9 (HDAC9), transcript variant 3, mRNA.<br>Homo sapiens histone deacetylase 9 (HDAC9), transcript variant 1, mRNA.<br>Homo sapiens histone deacetylase 9 (HDAC9), transcript variant 2, mRNA.<br>Homo sapiens cDNA FLJ39859 fis, clone SPLEN2015094, moderately similar to Mus musculus histone deacetylase mHDA1 mRNA.<br>Homo sapiens HDAC9v_1 mRNA sequence; alternatively spliced. |

| | | | | | | | | | |
|---|---|---|---|---|---|---|---|---|---|
| | | | | | | | | ENSESTT00000047318<br>ENSESTT00000047319<br>ENSESTT00000047320<br>ENSESTT00000047321<br>ENST00000341009<br>GENSCAN00000056913<br>GENSCAN00000054378<br>ENSESTT00000047323<br>GENSCAN00000062439<br>GENSCAN00000051403<br>ENSESTT00000047324<br>ENSESTT00000047325<br>GENSCAN00000053692 | cdna:known chromosome:NCBI36:7:18501894:18960401:1 gene:ENSG00000048052<br>cdna:Genscan chromosome:NCBI36:7:18584313:18596681:1<br>cdna:Genscan chromosome:NCBI36:7:18600056:18686489:1<br>cdna:Genscan chromosome:NCBI36:7:18708514:18776777:1<br>cdna:Genscan chromosome:NCBI36:7:18785118:18844595:1<br>cdna:Genscan chromosome:NCBI36:7:18880626:18955765:1 |
| 2992815 | 422225 | 2992814 | core | 23252964 | 23253001 | 7 | + | NM_002510<br>BC011595<br>BC032783<br>ENSESTT00000020407<br>ENSESTT00000020411<br>NM_001005340<br>ENST00000258733<br>ENST00000381990<br>ENSESTT00000020408<br>ENSESTT00000020409<br>ENSESTT00000020410<br>GENSCAN00000018869<br>ENSESTT00000020412 | Homo sapiens glycoprotein (transmembrane) nmb (GPNMB), transcript variant 2, mRNA.<br>Homo sapiens glycoprotein (transmembrane) nmb, mRNA (cDNA clone IMAGE:3345861), complete cds.<br>Homo sapiens glycoprotein (transmembrane) nmb, mRNA (cDNA clone MGC:45334 IMAGE:5177095), complete cds.<br>Homo sapiens glycoprotein (transmembrane) nmb (GPNMB), transcript variant 1, mRNA.<br>cdna:known-ccds chromosome:NCBI36:7:23252841:23281248:1 gene:ENSG00000136235 CCDS5380.1<br>cdna:known chromosome:NCBI36:7:23252841:23281248:1 gene:ENSG00000136235<br>cdna:Genscan chromosome:NCBI36:7:23253002:23300169:1 |
| 2993053 | 422365 | 2993029 | core | 23775785 | 23775866 | 7 | + | NM_031414<br>NM_032944<br>BC047506<br>ENSESTT00000020421<br>ENSESTT00000020422<br>ENST00000355870<br>ENST00000381965<br>ENST00000354639<br>BC033018<br>ENSESTT00000020423<br>GENSCAN00000038910<br>ENSESTT00000020424<br>ENSESTT00000020425 | Homo sapiens serine/threonine kinase 31 (STK31), transcript variant 1, mRNA.<br>Homo sapiens serine/threonine kinase 31 (STK31), transcript variant 2, mRNA.<br>Homo sapiens cDNA clone IMAGE:5295088, containing frame-shift errors.<br>cdna:known-ccds chromosome:NCBI36:7:23716404:23838651:1 gene:ENSG00000196335 CCDS5386.1<br>cdna:known chromosome:NCBI36:7:23716404:23838843:1 gene:ENSG00000196335<br>cdna:known chromosome:NCBI36:7:23716404:23838651:1 gene:ENSG00000196335<br>Homo sapiens serine/threonine kinase 31, mRNA (cDNA clone MGC:27039 IMAGE:4839162), complete cds.<br>cdna:Genscan chromosome:NCBI36:7:23741727:23797090:1 |
| 2999313 | 426408 | 2999303 | core | 42943844 | 42943907 | 7 | + | NM_031903<br>ENSESTT00000032212<br>ENST00000223324<br>GENSCAN00000039852 | Homo sapiens mitochondrial ribosomal protein L32 (MRPL32), nuclear gene encoding mitochondrial protein, mRNA.<br>cdna:known-ccds chromosome:NCBI36:7:42938464:42943976:1 gene:ENSG00000106591 CCDS5468.1<br>cdna:Genscan chromosome:NCBI36:7:42938511:42943700:1 |
| 2999401 | 426470 | 2999334 | core | 43485742 | 43485780 | 7 | + | ENSESTT00000039868<br>ENSESTT00000039869<br>NM_015052<br>ENST00000265522<br>GENSCAN00000052109 | Homo sapiens HECT, C2 and WW domain containing E3 ubiquitin protein ligase 1 (HECW1), mRNA.<br>cdna:known-ccds chromosome:NCBI36:7:43118723:43569463:1 gene:ENSG00000002746 CCDS5469.1<br>cdna:Genscan chromosome:NCBI36:7:43453641:43535377:1 |
| 3009402 | 432552 | 3009399 | core | 75769866 | 75769961 | 7 | + | NM_001540<br>AF086135 | Homo sapiens heat shock 27kDa protein 1 (HSPB1), mRNA.<br>Homo sapiens full length insert cDNA clone ZA89B11. |

| | | | | | | | | | |
|---|---|---|---|---|---|---|---|---|---|
| | | | | | | | | ENSESTT00000048348<br>CR606208<br>ENST00000248553<br>GENSCAN00000032320 | full-length cDNA clone CS0DI068YA24 of Placenta Cot 25-normalized of Homo sapiens (human).<br>cdna:known-ccds chromosome:NCBI36:7:75769966:75771545:1 gene:ENSG00000106211 CCDS5583.1<br>cdna:Genscan chromosome:NCBI36:7:75769966:75771426:1 |
| 3011936 | 434117 | 3011911 | core | 89746960 | 89747013 | 7 | + | NM_001039706<br>BC036351<br>AK093991<br>ENSESTT00000047079<br>ENSESTT00000047081<br>ENST00000333944<br>ENST00000316089<br>ENST00000344025<br>ENST00000389297<br>GENSCAN00000003416<br>AL833446<br>ENSESTT00000047084<br>ENSESTT00000047086<br>ENSESTT00000047088<br>ENSESTT00000047090 | Homo sapiens hypothetical protein FLJ21062 (FLJ21062), mRNA.<br>Homo sapiens hypothetical protein FLJ21062, mRNA (cDNA clone IMAGE:5171041), complete cds.<br>Homo sapiens cDNA FLJ36672 fis, clone UTERU2004073.<br>cdna:known-ccds chromosome:NCBI36:7:89712445:89777637:1 gene:ENSG00000105792 CCDS5616.1<br>cdna:known chromosome:NCBI36:7:89712445:89777637:1 gene:ENSG00000105792<br>cdna:known chromosome:NCBI36:7:89712675:89738930:1 gene:ENSG00000105792<br>cdna:novel chromosome:NCBI36:7:89722676:89777485:1 gene:ENSG00000105792<br>cdna:Genscan chromosome:NCBI36:7:89678212:89777488:1<br>Homo sapiens mRNA; cDNA DKFZp686F18109 (from clone DKFZp686F18109). |
| 3020408 | 439380 | 3020343 | core | 116223701 | 116223759 | 7 | + | NM_000245<br>ENST00000318493<br>ENSESTT00000028319<br>GENSCAN00000065557<br>ENSESTT00000028320<br>U08818<br>ENSESTT00000028321<br>GENSCAN00000040592 | Homo sapiens met proto-oncogene (hepatocyte growth factor receptor) (MET), mRNA.<br>cdna:known chromosome:NCBI36:7:116099695:116223632:1 gene:ENSG00000105976<br>cdna:Genscan chromosome:NCBI36:7:116185784:116210759:1<br>Human activated met oncogene mRNA, partial cds.<br>cdna:Genscan chromosome:NCBI36:7:116222945:116230307:1 |
| 3020409 | 439380 | 3020343 | core | 116223845 | 116224277 | 7 | + | NM_000245<br>ENST00000318493<br>ENSESTT00000028319<br>GENSCAN00000065557<br>ENSESTT00000028320<br>U08818<br>ENSESTT00000028321<br>GENSCAN00000040592 | Homo sapiens met proto-oncogene (hepatocyte growth factor receptor) (MET), mRNA.<br>cdna:known chromosome:NCBI36:7:116099695:116223632:1 gene:ENSG00000105976<br>cdna:Genscan chromosome:NCBI36:7:116185784:116210759:1<br>Human activated met oncogene mRNA, partial cds.<br>cdna:Genscan chromosome:NCBI36:7:116222945:116230307:1 |
| 3020410 | 439380 | 3020343 | core | 116224381 | 116224420 | 7 | + | NM_000245<br>ENST00000318493<br>ENSESTT00000028319<br>GENSCAN00000065557<br>ENSESTT00000028320<br>U08818<br>ENSESTT00000028321<br>GENSCAN00000040592 | Homo sapiens met proto-oncogene (hepatocyte growth factor receptor) (MET), mRNA.<br>cdna:known chromosome:NCBI36:7:116099695:116223632:1 gene:ENSG00000105976<br>cdna:Genscan chromosome:NCBI36:7:116185784:116210759:1<br>Human activated met oncogene mRNA, partial cds.<br>cdna:Genscan chromosome:NCBI36:7:116222945:116230307:1 |
| 3023526 | 441403 | 3023483 | core | 128907917 | 128907944 | 7 | + | NM_020704<br>ENST00000249344<br>BC019064<br>ENSESTT00000008384<br>GENSCAN00000062922<br>ENSESTT00000008385<br>ENSESTT00000008386<br>ENSESTT00000008387<br>ENSESTT00000008388 | Homo sapiens family with sequence similarity 40, member B (FAM40B), mRNA.<br>cdna:known chromosome:NCBI36:7:128861536:128915473:1 gene:ENSG00000128578<br>Homo sapiens family with sequence similarity 40, member B, mRNA (cDNA clone MGC:29781 IMAGE:4590587), complete cds.<br>cdna:Genscan chromosome:NCBI36:7:128861550:128923295:1 |

| | | | | | | | | | |
|---|---|---|---|---|---|---|---|---|---|
| 3025456 | 442640 | 3025433 | core | 133876431 | 133876526 | 7 | + | ENSESTT00000042742<br>ENST00000359579<br>NM_020299 | cdna:known-ccds chromosome:NCBI36:7:133862884:133876693:1 gene:ENSG00000198074 CCDS5832.1<br>Homo sapiens aldo-keto reductase family 1, member B10 (aldose reductase) (AKR1B10), mRNA. |
| 3025632 | 442755 | 3025545 | core | 134268889 | 134268951 | 7 | + | NM_033138<br>ENST00000361675<br>GENSCAN00000031243 | Homo sapiens caldesmon 1 (CALD1), transcript variant 1, mRNA.<br>cdna:known-ccds chromosome:NCBI36:7:134114711:134306010:1 gene:ENSG00000122786 CCDS5835.1<br>cdna:Genscan chromosome:NCBI36:7:134255820:134323287:1 |
| 3026644 | 443374 | 3026599 | core | 137890153 | 137890250 | 7 | + | NM_015905<br>ENSESTT00000038860<br>NM_003852<br>ENST00000343526<br>ENST00000378381<br>GENSCAN00000024892 | Homo sapiens tripartite motif-containing 24 (TRIM24), transcript variant 1, mRNA.<br><br>Homo sapiens tripartite motif-containing 24 (TRIM24), transcript variant 2, mRNA.<br>cdna:known-ccds chromosome:NCBI36:7:137795619:137920851:1 gene:ENSG00000122779 CCDS5847.1<br>cdna:known chromosome:NCBI36:7:137796350:137921233:1 gene:ENSG00000122779<br>cdna:Genscan chromosome:NCBI36:7:137826030:137920236:1 |
| 3032259 | 446760 | 3032243 | core | 151422357 | 151422511 | 7 | + | NM_022087<br>GENSCAN00000033935<br>ENST00000320311<br>AK124934<br>BC059377<br>ENSESTT00000042284<br>AK128545<br>ENSESTT00000042286 | Homo sapiens UDP-N-acetyl-alpha-D-galactosamine:polypeptide N-acetylgalactosaminyltransferase 11 (GalNAc-T11) (GALNT11), mRNA.<br>cdna:Genscan chromosome:NCBI36:7:151422246:151457768:1<br>cdna:known-ccds chromosome:NCBI36:7:151353797:151450357:1 gene:ENSG00000178234 CCDS5930.1<br>Homo sapiens cDNA FLJ42944 fis, clone BRSTN2004863, weakly similar to Drosophila melanogaster polypeptide N-acetylgalactosaminyltransferase mRNA.<br>Homo sapiens UDP-N-acetyl-alpha-D-galactosamine:polypeptide N-acetylgalactosaminyltransferase 11 (GalNAc-T11), mRNA (cDNA clone IMAGE:30346967), complete cds.<br><br>Homo sapiens cDNA FLJ46704 fis, clone TRACH3015136, moderately similar to Polypeptide N-acetylgalactosaminyltransferase (EC 2.4.1.41). |
| 3041902 | 452802 | 3041875 | core | 24840451 | 24840482 | 7 | - | AF491785<br>ENSESTT00000015952 | Homo sapiens oxysterol binding protein-related protein 3 isoform 2a mRNA, complete cds; alternatively spliced. |
| 3046447 | 455640 | 3046444 | core | 37912212 | 37912839 | 7 | - | NM_003014<br>BC032828<br>BC058911<br>ENSESTT00000014891<br>ENST00000223214<br>GENSCAN00000040951 | Homo sapiens secreted frizzled-related protein 4 (SFRP4), mRNA.<br>Homo sapiens secreted frizzled-related protein 4, mRNA (cDNA clone MGC:26498 IMAGE:4828181), complete cds.<br>Homo sapiens secreted frizzled-related protein 4, mRNA (cDNA clone MGC:65015 IMAGE:5228231), complete cds.<br><br>cdna:known-ccds chromosome:NCBI36:7:37912247:37922903:-1 gene:ENSG00000106483 CCDS5453.1<br>cdna:Genscan chromosome:NCBI36:7:37907848:37922664:-1 |
| 3047582 | 456330 | 3047581 | core | 41695321 | 41695588 | 7 | - | NM_002192<br>ENST00000242208<br>ENSESTT00000021044<br>ENSESTT00000021045 | Homo sapiens inhibin, beta A (activin A, activin AB alpha polypeptide) (INHBA), mRNA.<br>cdna:known-ccds chromosome:NCBI36:7:41695126:41709231:-1 gene:ENSG00000122641 CCDS5464.1 |
| 3047583 | 456330 | 3047581 | core | 41695857 | 41696021 | 7 | - | NM_002192<br>M13436<br>GENSCAN00000013491<br>ENST00000242208<br>BX648811<br>ENSESTT00000021044<br>ENSESTT00000021045 | Homo sapiens inhibin, beta A (activin A, activin AB alpha polypeptide) (INHBA), mRNA.<br>Human ovarian beta-A inhibin mRNA, complete cds.<br>cdna:Genscan chromosome:NCBI36:7:41663877:41729204:-1<br>cdna:known-ccds chromosome:NCBI36:7:41695126:41709231:-1 gene:ENSG00000122641 CCDS5464.1<br>Homo sapiens mRNA; cDNA DKFZp686A06204 (from clone DKFZp686A06204). |
| 3047584 | 456331 | 3047581 | core | 41696175 | 41696205 | 7 | - | NM_002192<br>M13436<br>GENSCAN00000013491<br>ENST00000242208<br>BX648811<br>ENSESTT00000021044<br>ENSESTT00000021045 | Homo sapiens inhibin, beta A (activin A, activin AB alpha polypeptide) (INHBA), mRNA.<br>Human ovarian beta-A inhibin mRNA, complete cds.<br>cdna:Genscan chromosome:NCBI36:7:41663877:41729204:-1<br>cdna:known-ccds chromosome:NCBI36:7:41695126:41709231:-1 gene:ENSG00000122641 CCDS5464.1<br>Homo sapiens mRNA; cDNA DKFZp686A06204 (from clone DKFZp686A06204). |

| | | | | | | | | | |
|---|---|---|---|---|---|---|---|---|---|
| 3047597 | 456335 | 3047581 | core | 41706453 | 41706490 | 7 | - | NM_002192<br>M13436<br>ENST00000242208<br>BX648811<br>ENSESTT00000021044<br>ENSESTT00000021045 | Homo sapiens inhibin, beta A (activin A, activin AB alpha polypeptide) (INHBA), mRNA.<br>Human ovarian beta-A inhibin mRNA, complete cds.<br>cdna:known-ccds chromosome:NCBI36:7:41695126:41709231:-1 gene:ENSG00000122641 CCDS5464.1<br>Homo sapiens mRNA; cDNA DKFZp686A06204 (from clone DKFZp686A06204). |
| 3049538 | 457518 | 3049522 | core | 47281297 | 47281321 | 7 | - | NM_022748<br>AK092864<br>BX648770<br>BX649002<br>ENST00000355730<br>ENST00000311160<br>ENSESTT00000030482<br>GENSCAN00000000239<br>GENSCAN00000000241<br>ENSESTT00000030478<br>ENSESTT00000030479<br>BC071791<br>ENSESTT00000030477<br>ENSESTT00000030475<br>ENSESTT00000030473<br>ENSESTT00000030476<br>ENSESTT00000030474<br>ENSESTT00000030472 | Homo sapiens tensin 3 (TNS3), mRNA.<br>Homo sapiens cDNA FLJ35545 fis, clone SPLEN2003918, moderately similar to TENSIN.<br>Homo sapiens mRNA; cDNA DKFZp686K12123 (from clone DKFZp686K12123).<br>Homo sapiens mRNA; cDNA DKFZp686G2290 (from clone DKFZp686G2290).<br>cdna:known-ccds chromosome:NCBI36:7:47281279:47588216:-1 gene:ENSG00000136205 CCDS5506.1<br>cdna:known chromosome:NCBI36:7:47281279:47588216:-1 gene:ENSG00000136205<br><br>cdna:Genscan chromosome:NCBI36:7:47284199:47353643:-1<br>cdna:Genscan chromosome:NCBI36:7:47372814:47380168:-1<br><br><br>Homo sapiens tensin 3, mRNA (cDNA clone IMAGE:4611842), complete cds. |
| 3056323 | 461717 | 3056320 | core | 72887009 | 72887074 | 7 | - | NM_152559<br>ENSESTT00000038207<br>GENSCAN00000020981<br>ENST00000297873 | Homo sapiens Williams Beuren syndrome chromosome region 27 (WBSCR27), mRNA.<br><br>cdna:Genscan chromosome:NCBI36:7:72886977:72894406:-1<br>cdna:known-ccds chromosome:NCBI36:7:72886856:72894800:-1 gene:ENSG00000165171 CCDS5561.1 |
| 3057662 | 462513 | 3057650 | core | 75826111 | 75826181 | 7 | - | NM_012479<br>ENSESTT00000048353<br>ENST00000307630 | Homo sapiens tyrosine 3-monooxygenase/tryptophan 5-monooxygenase activation protein, gamma polypeptide (YWHAG), mRNA.<br><br>cdna:known-ccds chromosome:NCBI36:7:75794053:75826252:-1 gene:ENSG00000170027 CCDS5584.1 |
| 3063757 | 466365 | 3063727 | core | 99554799 | 99554887 | 7 | - | NM_139122<br>NM_005641<br>ENSESTT00000011743<br>ENSESTT00000011744<br>NM_139315<br>NM_139123<br>AK097781<br>U31659<br>ENST00000344095<br>GENSCAN00000041181 | Homo sapiens TAF6 RNA polymerase II, TATA box binding protein (TBP)-associated factor, 80kDa (TAF6), transcript variant 3, mRNA.<br>Homo sapiens TAF6 RNA polymerase II, TATA box binding protein (TBP)-associated factor, 80kDa (TAF6), transcript variant 1, mRNA.<br><br><br>Homo sapiens TAF6 RNA polymerase II, TATA box binding protein (TBP)-associated factor, 80kDa (TAF6), transcript variant 2, mRNA.<br>Homo sapiens TAF6 RNA polymerase II, TATA box binding protein (TBP)-associated factor, 80kDa (TAF6), transcript variant 4, mRNA.<br>Homo sapiens cDNA FLJ40462 fis, clone TESTI2042062, highly similar to TRANSCRIPTION INITIATION FACTOR TFIID 70 KDA SUBUNIT.<br>Human TBP-associated factor TAFII80 mRNA, complete cds.<br>cdna:known-ccds chromosome:NCBI36:7:99542630:99554915:-1 gene:ENSG00000106290 CCDS5686.1<br>cdna:Genscan chromosome:NCBI36:7:99542805:99549768:-1 |
| 3069370 | 469894 | 3069366 | core | 116704984 | 116705060 | 7 | - | NM_003391<br>ENST00000265441<br>ENSESTT00000028337<br>ENSESTT00000028338<br>GENSCAN00000053585 | Homo sapiens wingless-type MMTV integration site family member 2 (WNT2), mRNA.<br>cdna:known-ccds chromosome:NCBI36:7:116704518:116750579:-1 gene:ENSG00000105989 CCDS5771.1<br><br><br>cdna:Genscan chromosome:NCBI36:7:116723946:116854750:-1 |

| | | | | | | | | | |
|---|---|---|---|---|---|---|---|---|---|
| 3079969 | 476617 | 3079803 | core | 151204528 | 151204638 | 7 | - | NM_016203<br>AK074675<br>ENSESTT00000042310<br>ENST00000287878<br>GENSCAN00000006331<br>NM_001040633<br>GENSCAN00000061091<br>ENSESTT00000042311<br>GENSCAN00000068126 | Homo sapiens protein kinase, AMP-activated, gamma 2 non-catalytic subunit (PRKAG2), transcript variant a, mRNA.<br>Homo sapiens cDNA FLJ90194 fis, clone MAMMA1001284.<br><br>cdna:known-ccds chromosome:NCBI36:7:150884960:151204728:-1 gene:ENSG00000106617 CCDS5928.1<br>cdna:Genscan chromosome:NCBI36:7:151190838:151204623:-1<br>Homo sapiens protein kinase, AMP-activated, gamma 2 non-catalytic subunit (PRKAG2), transcript variant c, mRNA.<br>cdna:Genscan chromosome:NCBI36:7:150953970:151072932:-1<br><br>cdna:Genscan chromosome:NCBI36:7:151107627:151123417:-1 |
| 3089375 | 482571 | 3089360 | core | 22323484 | 22323567 | 8 | + | NM_015359<br>ENST00000359741<br>BC015770<br>ENSESTT00000016977<br>ENSESTT00000016978<br>ENSESTT00000016979<br>ENSESTT00000016980<br>ENST00000381240<br>ENST00000240095<br>ENST00000289952<br>ENST00000381234<br>ENST00000381237 | Homo sapiens solute carrier family 39 (zinc transporter), member 14 (SLC39A14), mRNA.<br>cdna:known-ccds chromosome:NCBI36:8:22280737:22336139:1 gene:ENSG00000104635 CCDS6030.1<br>Homo sapiens solute carrier family 39 (zinc transporter), member 14, mRNA (cDNA clone MGC:23235 IMAGE:4865469), complete cds.<br><br><br><br><br>cdna:known chromosome:NCBI36:8:22280745:22347587:1 gene:ENSG00000104635<br>cdna:known chromosome:NCBI36:8:22280745:22347583:1 gene:ENSG00000104635<br>cdna:known chromosome:NCBI36:8:22280763:22336131:1 gene:ENSG00000104635<br>cdna:known chromosome:NCBI36:8:22280763:22336131:1 gene:ENSG00000104635<br>cdna:novel chromosome:NCBI36:8:22280763:22336199:1 gene:ENSG00000104635 |
| 3090726 | 483390 | 3090697 | core | 25402006 | 25402113 | 8 | + | NM_152562<br>ENSESTT00000019032<br>ENST00000330560<br>ENST00000380665<br>GENSCAN00000014721<br>ENSESTT00000019033<br>BX649008 | Homo sapiens cell division cycle associated 2 (CDCA2), mRNA.<br><br>cdna:known-ccds chromosome:NCBI36:8:25372428:25421342:1 gene:ENSG00000184661 CCDS6049.1<br>cdna:known chromosome:NCBI36:8:25372430:25421353:1 gene:ENSG00000184661<br>cdna:Genscan chromosome:NCBI36:8:25371133:25421171:1<br><br>Homo sapiens mRNA; cDNA DKFZp779I0613 (from clone DKFZp779I0613). |
| 3095087 | 486147 | 3095057 | core | 39208743 | 39208786 | 8 | + | NM_145004<br>ENST00000379907<br>ENST00000379912<br>ENSESTT00000009620<br>ENSESTT00000009621<br>GENSCAN00000018453<br>AB209690<br>ENST00000356164<br>ENSESTT00000009622<br>AK129810<br>ENSESTT00000009623<br>ENSESTT00000009624<br>ENSESTT00000009625 | Homo sapiens ADAM metallopeptidase domain 32 (ADAM32), mRNA.<br>cdna:known chromosome:NCBI36:8:39084325:39261585:1 gene:ENSG00000197140<br>cdna:novel chromosome:NCBI36:8:39084325:39261592:1 gene:ENSG00000197140<br><br><br>cdna:Genscan chromosome:NCBI36:8:39084452:39210758:1<br>Homo sapiens mRNA for ADAM 32 precursor (A disintegrin and metalloprotease domain 32) variant protein.<br>cdna:known chromosome:NCBI36:8:39106274:39200831:1 gene:ENSG00000197140<br><br>Homo sapiens cDNA FLJ26299 fis, clone DMC07412. |
| 3102465 | 490762 | 3102372 | core | 70735582 | 70735653 | 8 | + | NM_015170<br>ENSESTT00000052664<br>ENST00000260128<br>BX571746<br>ENSESTT00000052667<br>ENSESTT00000052665<br>ENSESTT00000052668<br>ENSESTT00000023351<br>ENSESTT00000023352 | Homo sapiens sulfatase 1 (SULF1), mRNA.<br><br>cdna:known-ccds chromosome:NCBI36:8:70541427:70735701:1 gene:ENSG00000137573 CCDS6204.1<br>Homo sapiens mRNA; cDNA DKFZp686F13142 (from clone DKFZp686F13142). |

| | | | | | | | | | |
|---|---|---|---|---|---|---|---|---|---|
| | | | | | | | | GENSCAN00000056157<br>BC068565<br>ENSESTT00000023353<br>ENSESTT00000023354<br>AK055467 | cdna:Genscan chromosome:NCBI36:8:70650737:70682774:1<br>Homo sapiens sulfatase 1, mRNA (cDNA clone MGC:87514 IMAGE:4831138), complete cds.<br><br><br>Homo sapiens cDNA FLJ30905 fis, clone FEBRA2005998. |
| 3105591 | 492784 | 3105581 | core | 86543521 | 86543604 | 8 | + | NM_005181<br>ENSESTT00000035286<br>ENSESTT00000035287<br>ENST00000285381<br>GENSCAN00000049622 | Homo sapiens carbonic anhydrase III, muscle specific (CA3), mRNA.<br><br><br>cdna:known-ccds chromosome:NCBI36:8:86537710:86548526:1 gene:ENSG00000164879 CCDS6238.1<br>cdna:Genscan chromosome:NCBI36:8:86538391:86547752:1 |
| 3105629 | 492810 | 3105600 | core | 86580823 | 86580923 | 8 | + | NM_000067<br>AK123309<br>ENST00000285379<br>BC011949<br>ENSESTT00000035288<br>ENSESTT00000035289<br>GENSCAN00000049625 | Homo sapiens carbonic anhydrase II (CA2), mRNA.<br>Homo sapiens cDNA FLJ41315 fis, clone BRAMY2043069.<br>cdna:known-ccds chromosome:NCBI36:8:86563433:86580945:1 gene:ENSG00000104267 CCDS6239.1<br>Homo sapiens carbonic anhydrase II, mRNA (cDNA clone MGC:9006 IMAGE:3863603), complete cds.<br><br><br>cdna:Genscan chromosome:NCBI36:8:86563019:86580270:1 |
| 3108614 | 494741 | 3108526 | core | 99114447 | 99114486 | 8 | + | NM_002380<br>NM_030583<br>ENST00000254898<br>ENST00000378716<br>AK075489<br>GENSCAN00000015958<br>BC016394<br>ENSESTT00000051989<br>ENSESTT00000051990<br>ENSESTT00000051991<br>ENSESTT00000051992 | Homo sapiens matrilin 2 (MATN2), transcript variant 1, mRNA.<br>Homo sapiens matrilin 2 (MATN2), transcript variant 2, mRNA.<br>cdna:known chromosome:NCBI36:8:98950487:99117116:1 gene:ENSG00000132561<br>cdna:known chromosome:NCBI36:8:98950487:99117116:1 gene:ENSG00000132561<br>Homo sapiens cDNA PSEC0183 fis, clone OVARC1001849, highly similar to Matrilin-2 precursor.<br>cdna:Genscan chromosome:NCBI36:8:98969505:99060412:1<br>Homo sapiens matrilin 2, mRNA (cDNA clone IMAGE:3863870), partial cds. |
| 3110320 | 495836 | 3110317 | core | 104453129 | 104453161 | 8 | + | NM_138455<br>ENST00000330295<br>GENSCAN00000004992<br>ENSESTT00000053027<br>ENSESTT00000053028<br>AF395488<br>ENST00000297577 | Homo sapiens collagen triple helix repeat containing 1 (CTHRC1), mRNA.<br>cdna:known-ccds chromosome:NCBI36:8:104453061:104464397:1 gene:ENSG00000164932 CCDS6299.1<br>cdna:Genscan chromosome:NCBI36:8:104453061:104459647:1<br><br><br>Homo sapiens NTMC1 mRNA, complete cds.<br>cdna:known chromosome:NCBI36:8:104453837:104464371:1 gene:ENSG00000164932 |
| 3110328 | 495838 | 3110317 | core | 104453973 | 104454047 | 8 | + | AF395488<br>GENSCAN00000004992<br>ENST00000297577 | Homo sapiens NTMC1 mRNA, complete cds.<br>cdna:Genscan chromosome:NCBI36:8:104453061:104459647:1<br>cdna:known chromosome:NCBI36:8:104453837:104464371:1 gene:ENSG00000164932 |
| 3110331 | 495841 | 3110317 | core | 104459447 | 104459578 | 8 | + | NM_138455<br>ENSESTT00000053028<br>ENSESTT00000053027<br>AF395488<br>ENST00000330295<br>ENST00000297577<br>GENSCAN00000004992 | Homo sapiens collagen triple helix repeat containing 1 (CTHRC1), mRNA.<br><br><br>Homo sapiens NTMC1 mRNA, complete cds.<br>cdna:known-ccds chromosome:NCBI36:8:104453061:104464397:1 gene:ENSG00000164932 CCDS6299.1<br>cdna:known chromosome:NCBI36:8:104453837:104464371:1 gene:ENSG00000164932<br>cdna:Genscan chromosome:NCBI36:8:104453061:104459647:1 |
| 3110336 | 495845 | 3110317 | core | 104464042 | 104464343 | 8 | + | NM_138455<br>ENSESTT00000053027<br>ENST00000297577<br>ENSESTT00000053028<br>AF395488<br>ENST00000330295<br>GENSCAN00000004992 | Homo sapiens collagen triple helix repeat containing 1 (CTHRC1), mRNA.<br><br>cdna:known chromosome:NCBI36:8:104453837:104464371:1 gene:ENSG00000164932<br><br>Homo sapiens NTMC1 mRNA, complete cds.<br>cdna:known-ccds chromosome:NCBI36:8:104453061:104464397:1 gene:ENSG00000164932 CCDS6299.1<br>cdna:Genscan chromosome:NCBI36:8:104453061:104459647:1 |

| | | | | | | | | | |
|---|---|---|---|---|---|---|---|---|---|
| 3121865 | 503012 | 3121751 | core | 3192946 | 3193063 | 8 | - | NM_033225<br>ENST00000335551<br>ENST00000318252<br>AB209502<br>GENSCAN00000005356<br>ENSESTT00000014064<br>ENSESTT00000014063<br>ENSESTT00000014062<br>GENSCAN00000004242<br>ENSESTT00000014061<br>AK126936<br>ENSESTT00000014060<br>ENST00000339701<br>AY358174<br>ENSESTT00000026098<br>ENSESTT00000026096<br>ENSESTT00000026097<br>GENSCAN00000000187<br>AK126857<br>ENSESTT00000026093<br>ENSESTT00000026094<br>GENSCAN00000000186<br>GENSCAN00000050780<br>GENSCAN00000009040<br>GENSCAN00000063191<br>GENSCAN00000057511<br>GENSCAN00000035921 | Homo sapiens CUB and Sushi multiple domains 1 (CSMD1), mRNA.<br>cdna:known chromosome:NCBI36:8:2782790:3254536:-1 gene:ENSG00000183117<br>cdna:known chromosome:NCBI36:8:2782790:3254536:-1 gene:ENSG00000183117<br>Homo sapiens mRNA for CUB and Sushi multiple domains 1 variant protein.<br>cdna:Genscan chromosome:NCBI36:8:2772604:2874442:-1<br><br><br><br>cdna:Genscan chromosome:NCBI36:8:2930851:3068796:-1<br><br>Homo sapiens cDNA FLJ44989 fis, clone BRAWH3008167, highly similar to Homo sapiens CUB and Sushi multiple domains 1 (CSMD1).<br><br>cdna:known chromosome:NCBI36:8:2972315:3254536:-1 gene:ENSG00000183117<br>Homo sapiens clone DNA41384 CSMD1 (UNQ5952) mRNA, complete cds.<br><br><br><br>cdna:Genscan chromosome:NCBI36:8:3112219:3177815:-1<br>Homo sapiens cDNA FLJ44909 fis, clone BRAMY3007968, highly similar to Homo sapiens CUB and Sushi multiple domains 1 (CSMD1).<br><br><br>cdna:Genscan chromosome:NCBI36:8:3184543:3193090:-1<br>cdna:Genscan chromosome:NCBI36:8:3210995:3263610:-1<br>cdna:Genscan chromosome:NCBI36:8:3312226:3338656:-1<br>cdna:Genscan chromosome:NCBI36:8:3414738:3423104:-1<br>cdna:Genscan chromosome:NCBI36:8:3835963:3921716:-1<br>cdna:Genscan chromosome:NCBI36:8:4477993:4507782:-1 |
| 3125616 | 505409 | 3125571 | core | 16094478 | 16094518 | 8 | - | NM_138715<br>ENSESTT00000010844<br>ENST00000262101<br>ENST00000381998<br>NM_138716<br>ENST00000350896<br>ENST00000382001<br>BC063878<br>ENSESTT00000010847<br>NM_002445<br>ENSESTT00000010845<br>ENST00000355282<br>ENST00000308443<br>ENSESTT00000010846 | Homo sapiens macrophage scavenger receptor 1 (MSR1), transcript variant SR-AI, mRNA.<br><br>cdna:known-ccds chromosome:NCBI36:8:16009761:16094595:-1 gene:ENSG00000038945 CCDS5995.1<br>cdna:known-ccds chromosome:NCBI36:8:16041197:16094539:-1 gene:ENSG00000038945 CCDS5997.1<br>Homo sapiens macrophage scavenger receptor 1 (MSR1), transcript variant SR-AIII, mRNA.<br>cdna:known-ccds chromosome:NCBI36:8:16009761:16094595:-1 gene:ENSG00000038945 CCDS5996.1<br>cdna:known chromosome:NCBI36:8:16009761:16094595:-1 gene:ENSG00000038945<br>Homo sapiens macrophage scavenger receptor 1, mRNA (cDNA clone MGC:75441 IMAGE:30378951), complete cds.<br><br>Homo sapiens macrophage scavenger receptor 1 (MSR1), transcript variant SR-AII, mRNA.<br><br>cdna:known chromosome:NCBI36:8:16011965:16079868:-1 gene:ENSG00000038945<br>cdna:known chromosome:NCBI36:8:16041199:16094595:-1 gene:ENSG00000038945 |
| 3127797 | 506811 | 3127775 | core | 23138524 | 23138584 | 8 | - | NM_003844<br>ENST00000221132<br>ENST00000380928<br>GENSCAN00000033278<br>ENSESTT00000002378 | Homo sapiens tumor necrosis factor receptor superfamily, member 10a (TNFRSF10A), mRNA.<br>cdna:known chromosome:NCBI36:8:23104916:23138584:-1 gene:ENSG00000104689<br>cdna:known-ccds chromosome:NCBI36:8:23104009:23138584:-1 gene:ENSG00000104689 CCDS6039.1<br>cdna:Genscan chromosome:NCBI36:8:23105152:23138345:-1 |
| 3147294 | 518869 | 3147286 | core | 103295468 | 103295544 | 8 | - | NM_015713<br>ENST00000251810<br>AB166670<br>CR627376 | Homo sapiens ribonucleotide reductase M2 B (TP53 inducible) (RRM2B), mRNA.<br>cdna:known chromosome:NCBI36:8:103285908:103320522:-1 gene:ENSG00000048392<br>Homo sapiens p53R2 mRNA for p53-inducible ribonucleotide reductase small subunit 2 short form gamma, complete cds. |

| | | | | | | | | | |
|---|---|---|---|---|---|---|---|---|---|
| | | | | | | | | ENSESTT00000019224<br>ENSESTT00000019225 | Homo sapiens mRNA; cDNA DKFZp686M05248 (from clone DKFZp686M05248). |
| 3150949 | 521343 | 3150844 | core | 121893848 | 121893910 | 8 | - | NM_021021<br>AK026095<br>ENSESTT00000031968<br>ENST00000346808<br>GENSCAN00000068873<br>ENSESTT00000031966<br>AF028828<br>ENSESTT00000031967 | Homo sapiens syntrophin, beta 1 (dystrophin-associated protein A1, 59kDa, basic component 1) (SNTB1), mRNA.<br>Homo sapiens cDNA: FLJ22442 fis, clone HRC09342, highly similar to AF028828 Homo sapiens Tax interaction protein 43 mRNA.<br><br>cdna:known-ccds chromosome:NCBI36:8:121619297:121893264:-1 gene:ENSG00000172164 CCDS6334.1<br>cdna:Genscan chromosome:NCBI36:8:121622131:121674434:-1<br><br>Homo sapiens Tax interaction protein 43 mRNA, partial cds. |
| 3151559 | 521724 | 3151534 | core | 124426236 | 124426299 | 8 | - | BC007123 | Homo sapiens ATPase family, AAA domain containing 2, mRNA (cDNA clone IMAGE:3454033), complete cds. |
| 3156918 | 525294 | 3156848 | core | 143425072 | 143425158 | 8 | - | NM_145003<br>ENST00000307180<br>BC065226<br>ENST00000377711<br>GENSCAN00000018989<br>GENSCAN00000018984<br>ENSESTT00000014621 | Homo sapiens t-SNARE domain containing 1 (TSNARE1), mRNA.<br>cdna:known-ccds chromosome:NCBI36:8:143291349:143482444:-1 gene:ENSG00000171045 CCDS6384.1<br>Homo sapiens cDNA clone IMAGE:5768818, partial cds.<br>cdna:known chromosome:NCBI36:8:143349756:143482434:-1 gene:ENSG00000171045<br>cdna:Genscan chromosome:NCBI36:8:143350355:143367364:-1<br>cdna:Genscan chromosome:NCBI36:8:143375414:143411408:-1 |
| 3157389 | 525559 | 3157385 | core | 144463004 | 144463031 | 8 | - | CR602435<br>ENSESTT00000009162<br>ENST00000329245<br>NM_052963 | full-length cDNA clone CS0DC018YN15 of Neuroblastoma Cot 25-normalized of Homo sapiens (human).<br><br>cdna:known-ccds chromosome:NCBI36:8:144462910:144488425:-1 gene:ENSG00000184428 CCDS6400.1<br>Homo sapiens topoisomerase (DNA) I, mitochondrial (TOP1MT), nuclear gene encoding mitochondrial protein, mRNA. |
| 3157401 | 525568 | 3157385 | core | 144474855 | 144474905 | 8 | - | ENSESTT00000009163<br>NM_052963<br>CR602435<br>ENSESTT00000009162<br>ENST00000329245<br>GENSCAN00000060305 | <br>Homo sapiens topoisomerase (DNA) I, mitochondrial (TOP1MT), nuclear gene encoding mitochondrial protein, mRNA.<br>full-length cDNA clone CS0DC018YN15 of Neuroblastoma Cot 25-normalized of Homo sapiens (human).<br><br>cdna:known-ccds chromosome:NCBI36:8:144462910:144488425:-1 gene:ENSG00000184428 CCDS6400.1<br>cdna:Genscan chromosome:NCBI36:8:144459023:144498845:-1 |
| 3158502 | 526178 | 3158478 | core | 145552716 | 145552741 | 8 | - | NM_024555<br>ENST00000331890<br>GENSCAN00000061978<br>NM_012162<br>AL713783 | Homo sapiens F-box and leucine-rich repeat protein 6 (FBXL6), transcript variant 2, mRNA.<br>cdna:known-ccds chromosome:NCBI36:8:145549900:145552915:-1 gene:ENSG00000182325 CCDS6422.1<br>cdna:Genscan chromosome:NCBI36:8:145527655:145553624:-1<br>Homo sapiens F-box and leucine-rich repeat protein 6 (FBXL6), transcript variant 1, mRNA.<br>Homo sapiens mRNA; cDNA DKFZp547A019 (from clone DKFZp547A019). |
| 3168530 | 532340 | 3168508 | core | 36659310 | 36659394 | 9 | + | NM_014791<br>ENSESTT00000032490<br>ENSESTT00000032491<br>ENST00000298048<br>GENSCAN00000059764 | Homo sapiens maternal embryonic leucine zipper kinase (MELK), mRNA.<br><br><br>cdna:known-ccds chromosome:NCBI36:9:36562873:36667678:1 gene:ENSG00000165304 CCDS6606.1<br>cdna:Genscan chromosome:NCBI36:9:36571679:36633080:1 |
| 3173982 | 535887 | 3173974 | core | 71134120 | 71134435 | 9 | + | NM_004816<br>ENST00000257515<br>ENST00000377218<br>ENSESTT00000027844<br>ENSESTT00000027845<br>ENST00000377225<br>GENSCAN00000000819<br>ENST00000303068<br>BX641153<br>ENSESTT00000027846 | Homo sapiens chromosome 9 open reading frame 61 (C9orf61), mRNA.<br>cdna:known-ccds chromosome:NCBI36:9:71134061:71197188:1 gene:ENSG00000135063 CCDS6629.1<br>cdna:known chromosome:NCBI36:9:71134061:71197188:1 gene:ENSG00000135063<br><br><br>cdna:known chromosome:NCBI36:9:71130168:71197190:1 gene:ENSG00000135063<br>cdna:Genscan chromosome:NCBI36:9:71130046:71196540:1<br>cdna:novel chromosome:NCBI36:9:71176002:71197191:1 gene:ENSG00000135063<br>Homo sapiens mRNA; cDNA DKFZp686J24109 (from clone DKFZp686J24109). |

| | | | | | | | | | |
|---|---|---|---|---|---|---|---|---|---|
| | | | | | | | | ENSESTT00000027847<br>ENSESTT00000027848 | |
| 3174122 | 535962 | 3174121 | core | 71848392 | 71848876 | 9 | + | NM_153267<br>ENSESTT00000015668<br>ENST00000377182<br>ENST00000324072<br>ENSESTT00000015669<br>GENSCAN00000030326<br>BX538309<br>GENSCAN00000035643 | Homo sapiens MAM domain containing 2 (MAMDC2), mRNA.<br><br>cdna:known-ccds chromosome:NCBI36:9:71848317:72031706:1 gene:ENSG00000165072 CCDS6631.1<br>cdna:known chromosome:NCBI36:9:71848340:72031708:1 gene:ENSG00000165072<br><br>cdna:Genscan chromosome:NCBI36:9:71880693:71976979:1<br>Homo sapiens mRNA; cDNA DKFZp686C09130 (from clone DKFZp686C09130).<br>cdna:Genscan chromosome:NCBI36:9:72023186:72030768:1 |
| 3175980 | 537043 | 3175971 | core | 80109478 | 80109503 | 9 | + | NM_021154<br>NM_058179<br>ENST00000376588<br>ENST00000347159<br>ENST00000277070<br>ENST00000342478<br>ENSESTT00000001673<br>ENSESTT00000001674<br>GENSCAN00000044543<br>GENSCAN00000054586 | Homo sapiens phosphoserine aminotransferase 1 (PSAT1), transcript variant 2, mRNA.<br>Homo sapiens phosphoserine aminotransferase 1 (PSAT1), transcript variant 1, mRNA.<br>cdna:known-ccds chromosome:NCBI36:9:80101879:80134829:1 gene:ENSG00000135069 CCDS6660.1<br>cdna:known-ccds chromosome:NCBI36:9:80101879:80134827:1 gene:ENSG00000135069 CCDS6659.1<br>cdna:known chromosome:NCBI36:9:80101879:80134827:1 gene:ENSG00000135069<br>cdna:known chromosome:NCBI36:9:80101879:80134190:1 gene:ENSG00000135069<br><br><br>cdna:Genscan chromosome:NCBI36:9:80101686:80122544:1<br>cdna:Genscan chromosome:NCBI36:1:79293218:79294328:-1 |
| 3175990 | 537051 | 3175971 | core | 80133986 | 80134320 | 9 | + | ENST00000376588<br>NM_021154<br>NM_058179<br>ENSESTT00000001673<br>ENSESTT00000001674<br>ENSESTT00000001675<br>ENST00000347159<br>ENST00000277070<br>ENST00000342478 | cdna:known-ccds chromosome:NCBI36:9:80101879:80134829:1 gene:ENSG00000135069 CCDS6660.1<br>Homo sapiens phosphoserine aminotransferase 1 (PSAT1), transcript variant 2, mRNA.<br>Homo sapiens phosphoserine aminotransferase 1 (PSAT1), transcript variant 1, mRNA.<br><br><br><br>cdna:known-ccds chromosome:NCBI36:9:80101879:80134827:1 gene:ENSG00000135069 CCDS6659.1<br>cdna:known chromosome:NCBI36:9:80101879:80134827:1 gene:ENSG00000135069<br>cdna:known chromosome:NCBI36:9:80101879:80134190:1 gene:ENSG00000135069 |
| 3189493 | 545095 | 3189422 | core | 128307228 | 128307719 | 9 | + | AK000001<br>ENST00000360044<br>NM_033446<br>ENST00000361171 | Homo sapiens mRNA for FLJ00001 protein, partial cds.<br>cdna:known chromosome:NCBI36:9:128128949:128309141:1 gene:ENSG00000196814<br>Homo sapiens chromosome 9 open reading frame 28 (C9orf28), transcript variant 1, mRNA.<br>cdna:known chromosome:NCBI36:9:128128949:128309140:1 gene:ENSG00000196814 |
| 3201324 | 552449 | 3201319 | core | 21375293 | 21375317 | 9 | - | NM_000605<br>ENST00000380206<br>ENST00000315719<br>GENSCAN00000028810 | Homo sapiens interferon, alpha 2 (IFNA2), mRNA.<br>cdna:known-ccds chromosome:NCBI36:9:21374253:21375387:-1 gene:ENSG00000188379 CCDS6506.1<br>cdna:known chromosome:NCBI36:9:21374762:21375328:-1 gene:ENSG00000188379<br>cdna:Genscan chromosome:NCBI36:9:21374762:21375328:-1 |
| 3204253 | 554207 | 3204243 | core | 34627274 | 34627336 | 9 | - | NM_147158<br>NM_147157<br>ENST00000378899<br>ENST00000378892<br>NM_005866<br>AK130502<br>ENSESTT00000056386<br>ENSESTT00000056387<br>ENST00000277010<br>ENST00000360710<br>ENST00000378893<br>ENST00000341240<br>GENSCAN00000019587<br>ENST00000353468 | Homo sapiens opioid receptor, sigma 1 (OPRS1), transcript variant 3, mRNA.<br>Homo sapiens opioid receptor, sigma 1 (OPRS1), transcript variant 2, mRNA.<br>cdna:known-ccds chromosome:NCBI36:9:34624717:34627806:-1 gene:ENSG00000147955 CCDS6564.1<br>cdna:known chromosome:NCBI36:9:34624719:34627729:-1 gene:ENSG00000147955<br>Homo sapiens opioid receptor, sigma 1 (OPRS1), transcript variant 1, mRNA.<br>Homo sapiens cDNA FLJ26992 fis, clone SLV03821, highly similar to Homo sapiens sigma receptor (SR31747 binding protein 1) (SR-BP1).<br><br><br>cdna:known-ccds chromosome:NCBI36:9:34624717:34627806:-1 gene:ENSG00000147955 CCDS6562.1<br>cdna:known chromosome:NCBI36:9:34624717:34627694:-1 gene:ENSG00000147955<br>cdna:known chromosome:NCBI36:9:34624719:34627768:-1 gene:ENSG00000147955<br>cdna:known chromosome:NCBI36:9:34624719:34627741:-1 gene:ENSG00000147955<br>cdna:Genscan chromosome:NCBI36:9:34625629:34627694:-1<br>cdna:known-ccds chromosome:NCBI36:9:34624717:34627806:-1 gene:ENSG00000147955 CCDS6563.1 |

| | | | | | | | | | |
|---|---|---|---|---|---|---|---|---|---|
| 3213228 | 559708 | 3213219 | core | 88889592 | 88889627 | 9 | - | BC068978<br>GENSCAN00000057098 | Homo sapiens NFYC pseudogene, mRNA (cDNA clone MGC:70842 IMAGE:6169268), complete cds.<br>cdna:Genscan chromosome:NCBI36:9:88888890:88894085:-1 |
| 3220182 | 564011 | 3220180 | core | 112105718 | 112105801 | 9 | - | NM_001003936<br>ENSESTT00000037202<br>ENST00000374510<br>ENST00000374511<br>ENST00000389181<br>ENST00000389180<br>BC035743<br>ENST00000374509 | Homo sapiens thioredoxin domain containing 8 (TXNDC8), mRNA.<br><br>cdna:novel chromosome:NCBI36:9:112105690:112139897:-1 gene:ENSG00000204193<br>cdna:known chromosome:NCBI36:9:112105622:112139897:-1 gene:ENSG00000204193<br>cdna:known chromosome:NCBI36:9:112105783:112139897:-1 gene:ENSG00000204193<br>cdna:known chromosome:NCBI36:9:112105783:112139897:-1 gene:ENSG00000204193<br>Homo sapiens thioredoxin domain containing 8, mRNA (cDNA clone IMAGE:5744423).<br>cdna:known chromosome:NCBI36:9:112124562:112139948:-1 gene:ENSG00000204193 |
| 3222198 | 565230 | 3222170 | core | 116848524 | 116848778 | 9 | - | NM_002160<br>AK024586<br>ENSESTT00000038174<br>ENSESTT00000038178<br>ENSESTT00000038184<br>ENST00000350763<br>ENST00000341037<br>ENST00000340094<br>ENST00000346706<br>ENST00000345230<br>GENSCAN00000048533<br>ENSESTT00000038186<br>ENSESTT00000038167<br>ENSESTT00000038163 | Homo sapiens tenascin C (hexabrachion) (TNC), mRNA.<br>Homo sapiens cDNA: FLJ20933 fis, clone ADSE01388.<br><br><br><br>cdna:known-ccds chromosome:NCBI36:9:116822634:116920260:-1 gene:ENSG00000041982 CCDS6811.1<br>cdna:known chromosome:NCBI36:9:116822634:116920260:-1 gene:ENSG00000041982<br>cdna:known chromosome:NCBI36:9:116822634:116920260:-1 gene:ENSG00000041982<br>cdna:known chromosome:NCBI36:9:116822634:116920260:-1 gene:ENSG00000041982<br>cdna:known chromosome:NCBI36:9:116822634:116920260:-1 gene:ENSG00000041982<br>cdna:Genscan chromosome:NCBI36:9:116823257:116893118:-1 |
| 3229770 | 570027 | 3229741 | core | 138230322 | 138230422 | 9 | - | NM_014564<br>NM_178138<br>ENST00000371748<br>ENST00000371746<br>ENST00000336264<br>ENST00000241587<br>ENST00000325195<br>GENSCAN00000033903 | Homo sapiens LIM homeobox 3 (LHX3), transcript variant 2, mRNA.<br>Homo sapiens LIM homeobox 3 (LHX3), transcript variant 1, mRNA.<br>cdna:known-ccds chromosome:NCBI36:9:138227917:138236776:-1 gene:ENSG00000107187 CCDS6994.1<br>cdna:known-ccds chromosome:NCBI36:9:138227917:138234825:-1 gene:ENSG00000107187 CCDS6995.1<br>cdna:known chromosome:NCBI36:9:138227919:138236776:-1 gene:ENSG00000107187<br>cdna:known chromosome:NCBI36:9:138227919:138234825:-1 gene:ENSG00000107187<br>cdna:known chromosome:NCBI36:9:138228992:138234825:-1 gene:ENSG00000107187<br>cdna:Genscan chromosome:NCBI36:9:138212602:138236679:-1 |
| 3230761 | 570545 | 3230760 | core | 139076805 | 139077197 | 9 | - | NM_178448<br>ENST00000359018<br>ENST00000334240<br>ENSESTT00000056169<br>GENSCAN00000017028 | Homo sapiens chromosome 9 open reading frame 140 (C9orf140), mRNA.<br>cdna:known-ccds chromosome:NCBI36:9:139076402:139084805:-1 gene:ENSG00000186193 CCDS7027.1<br>cdna:known chromosome:NCBI36:9:139076599:139084849:-1 gene:ENSG00000186193<br><br>cdna:Genscan chromosome:NCBI36:9:139078932:139084718:-1 |
| 3235798 | 573675 | 3235789 | core | 13254676 | 13254764 | 10 | + | NM_182751<br>ENST00000361282<br>NM_018518<br>ENSESTT00000002001<br>ENST00000378714<br>ENST00000357807<br>ENST00000378694 | Homo sapiens MCM10 minichromosome maintenance deficient 10 (S. cerevisiae) (MCM10), transcript variant 1, mRNA.<br>cdna:known-ccds chromosome:NCBI36:10:13243587:13293106:1 gene:ENSG00000065328 CCDS7096.1<br>Homo sapiens MCM10 minichromosome maintenance deficient 10 (S. cerevisiae) (MCM10), transcript variant 2, mRNA.<br><br>cdna:known-ccds chromosome:NCBI36:10:13243587:13293110:1 gene:ENSG00000065328 CCDS7095.1<br>cdna:known chromosome:NCBI36:10:13243587:13293106:1 gene:ENSG00000065328<br>cdna:known chromosome:NCBI36:10:13246134:13293110:1 gene:ENSG00000065328 |
| 3236548 | 574131 | 3236538 | core | 15186183 | 15186233 | 10 | + | NM_006414<br>ENST00000378197<br>ENST00000282318<br>ENSESTT00000024451<br>ENST00000378202<br>ENST00000378203 | Homo sapiens ribonuclease P/MRP 38kDa subunit (RPP38), transcript variant 2, mRNA.<br>cdna:known chromosome:NCBI36:10:15179387:15186262:1 gene:ENSG00000152464<br>cdna:known chromosome:NCBI36:10:15179336:15186260:1 gene:ENSG00000152464<br><br>cdna:known chromosome:NCBI36:10:15179208:15186257:1 gene:ENSG00000152464<br>cdna:known-ccds chromosome:NCBI36:10:15179185:15186256:1 gene:ENSG00000152464 CCDS7108.1 |

| | | | | | | | | | |
|---|---|---|---|---|---|---|---|---|---|
| | | | | | | | | NM_183005 | Homo sapiens ribonuclease P/MRP 38kDa subunit (RPP38), transcript variant 1, mRNA. |
| 3237511 | 574759 | 3237396 | core | 18868182 | 18868588 | 10 | + | NM_201572<br>NM_201597<br>AF423192<br>ENST00000324631<br>ENST00000377319<br>ENST00000340232<br>NM_201571<br>ENST00000362084<br>NM_201593<br>NM_201596<br>AY393860<br>ENST00000377331<br>ENST00000282343<br>ENST00000359991<br>ENST00000377329<br>ENST00000377328<br>GENSCAN00000057423<br>NM_201590<br>NM_201570<br>NM_000724<br>ENSESTT00000052764<br>ENSESTT00000052766<br>ENSESTT00000052767<br>ENST00000352115<br>ENST00000377318<br>ENST00000340194<br>ENST00000377315<br>GENSCAN00000041685<br>GENSCAN00000022946<br>ENSESTT00000052768 | Homo sapiens calcium channel, voltage-dependent, beta 2 subunit (CACNB2), transcript variant 8, mRNA.<br>Homo sapiens calcium channel, voltage-dependent, beta 2 subunit (CACNB2), transcript variant 4, mRNA.<br>Homo sapiens calcium channel beta 2e subunit (CACNB2) mRNA, complete cds.<br>cdna:known-ccds chromosome:NCBI36:10:18469612:18870062:1 gene:ENSG00000165995 CCDS7125.1<br>cdna:known-ccds chromosome:NCBI36:10:18589704:18870797:1 gene:ENSG00000165995 CCDS7128.1<br>cdna:known chromosome:NCBI36:10:18729967:18870044:1 gene:ENSG00000165995<br>Homo sapiens calcium channel, voltage-dependent, beta 2 subunit (CACNB2), transcript variant 6, mRNA.<br>cdna:known chromosome:NCBI36:10:18470115:18868659:1 gene:ENSG00000165995<br>Homo sapiens calcium channel, voltage-dependent, beta 2 subunit (CACNB2), transcript variant 5, mRNA.<br>Homo sapiens calcium channel, voltage-dependent, beta 2 subunit (CACNB2), transcript variant 2, mRNA.<br>Homo sapiens voltage-gated calcium channel beta 2 subunit splice variant CavB2cN1 (CACNB2) mRNA, complete cds, alternatively spliced.<br>cdna:known-ccds chromosome:NCBI36:10:18469672:18868659:1 gene:ENSG00000165995 CCDS7126.1<br>cdna:known-ccds chromosome:NCBI36:10:18469893:18870062:1 gene:ENSG00000165995 CCDS7127.1<br>cdna:known chromosome:NCBI36:10:18469672:18868659:1 gene:ENSG00000165995<br>cdna:known chromosome:NCBI36:10:18469672:18868659:1 gene:ENSG00000165995<br>cdna:known chromosome:NCBI36:10:18469672:18868659:1 gene:ENSG00000165995<br>cdna:Genscan chromosome:NCBI36:10:18469687:18479910:1<br>Homo sapiens calcium channel, voltage-dependent, beta 2 subunit (CACNB2), transcript variant 3, mRNA.<br>Homo sapiens calcium channel, voltage-dependent, beta 2 subunit (CACNB2), transcript variant 7, mRNA.<br>Homo sapiens calcium channel, voltage-dependent, beta 2 subunit (CACNB2), transcript variant 1, mRNA.<br><br><br><br>cdna:known-ccds chromosome:NCBI36:10:18729748:18870062:1 gene:ENSG00000165995 CCDS7129.1<br>cdna:known chromosome:NCBI36:10:18589704:18870062:1 gene:ENSG00000165995<br>cdna:known chromosome:NCBI36:10:18590196:18870062:1 gene:ENSG00000165995<br>cdna:known chromosome:NCBI36:10:18669660:18827728:1 gene:ENSG00000165995<br>cdna:Genscan chromosome:NCBI36:10:18695988:18743934:1<br>cdna:Genscan chromosome:NCBI36:10:18827290:18843304:1 |
| 3239178 | 575835 | 3238962 | core | 24874077 | 24874163 | 10 | + | BC018764<br>BX640796<br>ENSESTT00000016154<br>ENSESTT00000016156<br>ENSESTT00000016158<br>ENST00000307544 | Homo sapiens KIAA1217, mRNA (cDNA clone IMAGE:3604428), complete cds.<br>Homo sapiens mRNA; cDNA DKFZp686E0469 (from clone DKFZp686E0469); complete cds.<br><br><br><br>cdna:known chromosome:NCBI36:10:24778372:24874203:1 gene:ENSG00000120549 |
| 3250065 | 582524 | 3250055 | core | 70389580 | 70389620 | 10 | + | NM_004728<br>BC104671<br>BX648405<br>ENSESTT00000040952<br>ENST00000354185<br>ENST00000277806<br>CR749598<br>GENSCAN00000035956<br>ENSESTT00000040953<br>ENSESTT00000040954 | Homo sapiens DEAD (Asp-Glu-Ala-Asp) box polypeptide 21 (DDX21), mRNA.<br>Homo sapiens DEAD (Asp-Glu-Ala-Asp) box polypeptide 21, mRNA (cDNA clone IMAGE:6744750), partial cds.<br>Homo sapiens mRNA; cDNA DKFZp686A04240 (from clone DKFZp686A04240).<br><br>cdna:known chromosome:NCBI36:10:70385890:70414835:1 gene:ENSG00000165732<br>cdna:known chromosome:NCBI36:10:70385898:70414285:1 gene:ENSG00000165732<br>Homo sapiens mRNA; cDNA DKFZp686F21172 (from clone DKFZp686F21172).<br>cdna:Genscan chromosome:NCBI36:10:70380584:70412578:1 |
| 3252054 | 583822 | 3252036 | core | 75343760 | 75343892 | 10 | + | NM_002658<br>ENSESTT00000058212<br>GENSCAN00000010635<br>ENSESTT00000058209 | Homo sapiens plasminogen activator, urokinase (PLAU), mRNA.<br><br>cdna:Genscan chromosome:NCBI36:10:75341320:75346329:1 |

| | | | | | | | | | |
|---|---|---|---|---|---|---|---|---|---|
| | | | | | | | | ENST00000372764<br>ENST00000242464<br>ENST00000372762<br>ENST00000372761<br>ENSESTT00000058211 | cdna:known-ccds chromosome:NCBI36:10:75340896:75347260:1 gene:ENSG00000122861 CCDS7339.1<br>cdna:known chromosome:NCBI36:10:75340921:75347261:1 gene:ENSG00000122861<br>cdna:known chromosome:NCBI36:10:75340921:75347258:1 gene:ENSG00000122861<br>cdna:known chromosome:NCBI36:10:75341320:75346329:1 gene:ENSG00000122861 |
| 3258459 | 587865 | 3258444 | core | 95268633 | 95268695 | 10 | + | ENSESTT00000025833<br>ENSESTT00000025834<br>GENSCAN00000019331<br>ENSESTT00000025836<br>ENSESTT00000025837<br>NM_018131<br>BX640718<br>ENSESTT00000025832<br>ENSESTT00000025835<br>ENST00000371485<br>ENST00000260751<br>ENST00000358339 | <br><br>cdna:Genscan chromosome:NCBI36:10:95244402:95309512:1<br><br><br>Homo sapiens centrosomal protein 55kDa (CEP55), mRNA.<br>Homo sapiens mRNA; cDNA DKFZp686P16192 (from clone DKFZp686P16192).<br><br><br>cdna:known-ccds chromosome:NCBI36:10:95246379:95278839:1 gene:ENSG00000138180 CCDS7428.1<br>cdna:known chromosome:NCBI36:10:95246399:95278837:1 gene:ENSG00000138180<br>cdna:known chromosome:NCBI36:10:95246399:95278837:1 gene:ENSG00000138180 |
| 3265254 | 592158 | 3265224 | core | 116028510 | 116028586 | 10 | + | NM_198496<br>ENST00000298715<br>AY572972<br>AJ536328<br>ENST00000369277<br>ENST00000369276<br>ENST00000369275<br>AK127756 | Homo sapiens von Willebrand factor A domain containing 2 (VWA2), mRNA.<br>cdna:known-ccds chromosome:NCBI36:10:115989008:116039741:1 gene:ENSG00000165816 CCDS7589.1<br>Homo sapiens colon cancer secreted protein-2 (CCSP-2) mRNA, complete cds.<br>Homo sapiens mRNA for AMACO.<br>cdna:known chromosome:NCBI36:10:115989079:116041262:1 gene:ENSG00000165816<br>cdna:known chromosome:NCBI36:10:115989079:116039741:1 gene:ENSG00000165816<br>cdna:known chromosome:NCBI36:10:115989241:116041727:1 gene:ENSG00000165816<br>Homo sapiens cDNA FLJ45857 fis, clone OCBBF2030927, weakly similar to Homo sapiens matrilin 4 (MATN4). |
| 3265582 | 592351 | 3265565 | core | 116879086 | 116879144 | 10 | + | ENSESTT00000011053<br>NM_207303<br>BC035157<br>BC029592<br>ENSESTT00000011051<br>ENST00000355044<br>ENST00000303745 | <br>Homo sapiens attractin-like 1 (ATRNL1), mRNA.<br>Homo sapiens attractin-like 1, mRNA (cDNA clone IMAGE:5265311), complete cds.<br>Homo sapiens attractin-like 1, mRNA (cDNA clone IMAGE:5272894), with apparent retained intron.<br><br>cdna:known-ccds chromosome:NCBI36:10:116843114:117698484:1 gene:ENSG00000107518 CCDS7592.1<br>cdna:known chromosome:NCBI36:10:116843114:117347296:1 gene:ENSG00000107518 |
| 3290654 | 608131 | 3290649 | core | 60677383 | 60677480 | 10 | - | NM_198215<br>ENSESTT00000017406<br>ENST00000373868<br>ENST00000373870<br>GENSCAN00000005261<br>ENST00000277705<br>ENST00000373867<br>NM_001001971<br>ENSESTT00000017414<br>ENSESTT00000017416<br>ENSESTT00000017419<br>ENSESTT00000017424<br>ENSESTT00000017425<br>ENSESTT00000017409<br>ENSESTT00000017418<br>ENSESTT00000017421<br>U79304<br>ENSESTT00000017423<br>ENSESTT00000045771<br>ENSESTT00000045773<br>ENSESTT00000045772 | Homo sapiens family with sequence similarity 13, member C1 (FAM13C1), transcript variant 1, mRNA.<br><br>cdna:known-ccds chromosome:NCBI36:10:60675902:60792358:-1 gene:ENSG00000148541 CCDS7255.1<br>cdna:known chromosome:NCBI36:10:60675899:60792314:-1 gene:ENSG00000148541<br>cdna:Genscan chromosome:NCBI36:10:60677380:60701560:-1<br>cdna:known chromosome:NCBI36:10:60675902:60792358:-1 gene:ENSG00000148541<br>cdna:known chromosome:NCBI36:10:60675902:60792358:-1 gene:ENSG00000148541<br>Homo sapiens family with sequence similarity 13, member C1 (FAM13C1), transcript variant 2, mRNA.<br><br><br><br><br><br><br><br><br>Human clone 23909 mRNA, partial cds. |

| | | | | | | | | | |
|---|---|---|---|---|---|---|---|---|---|
| 3292953 | 609669 | 3292946 | core | 70834703 | 70834732 | 10 | - | NM_001057<br>AK096906<br>ENST00000373306<br>ENST00000352020<br>ENST00000373307<br>ENSESTT00000015285<br>GENSCAN00000003211 | Homo sapiens tachykinin receptor 2 (TACR2), mRNA.<br>Homo sapiens cDNA FLJ39587 fis, clone SKMUS2008407, highly similar to SUBSTANCE-K RECEPTOR.<br>cdna:known-ccds chromosome:NCBI36:10:70834246:70846629:-1 gene:ENSG00000075073 CCDS7293.1<br>cdna:known chromosome:NCBI36:10:70834429:70846607:-1 gene:ENSG00000075073<br>cdna:novel chromosome:NCBI36:10:70833665:70839044:-1 gene:ENSG00000075073<br><br>cdna:Genscan chromosome:NCBI36:10:70832754:70869113:-1 |
| 3296059 | 611553 | 3296046 | core | 78314677 | 78314749 | 10 | - | NM_002247<br>ENST00000286628 | Homo sapiens potassium large conductance calcium-activated channel, subfamily M, alpha member 1 (KCNMA1), transcript variant 2, mRNA.<br>cdna:known-ccds chromosome:NCBI36:10:78314645:79068359:-1 gene:ENSG00000156113 CCDS7352.1 |
| 3296279 | 611728 | 3296046 | core | 79066239 | 79066269 | 10 | - | BC062659<br>CR627384<br>BC009695<br>ENST00000354353 | Homo sapiens potassium large conductance calcium-activated channel, subfamily M, alpha member 1, mRNA (cDNA clone IMAGE:4277048), complete cds.<br>Homo sapiens mRNA; cDNA DKFZp781N1049 (from clone DKFZp781N1049).<br>Homo sapiens potassium large conductance calcium-activated channel, subfamily M, alpha member 1, mRNA (cDNA clone IMAGE:3900695), with apparent retained intron.<br>cdna:known chromosome:NCBI36:10:79065969:79067489:-1 gene:ENSG00000156113 |
| 3304306 | 616724 | 3304301 | core | 104154357 | 104154458 | 10 | - | NM_002779<br>ENST00000020673<br>CR616163<br>GENSCAN00000036199<br>ENSESTT00000007154<br>ENSESTT00000007153<br>ENSESTT00000007152 | Homo sapiens pleckstrin and Sec7 domain containing (PSD), mRNA.<br>cdna:known chromosome:NCBI36:10:104152366:104168891:-1 gene:ENSG00000059915<br>full-length cDNA clone CS0DF031YD08 of Fetal brain of Homo sapiens (human).<br>cdna:Genscan chromosome:NCBI36:10:104152947:104166785:-1 |
| 3305230 | 617297 | 3305198 | core | 105917369 | 105917477 | 10 | - | NM_025145<br>ENST00000278064<br>ENST00000357060<br>AL833836<br>ENST00000389588<br>GENSCAN00000016988<br>ENSESTT00000032785<br>ENSESTT00000032784<br>ENSESTT00000032782<br>GENSCAN00000017350<br>AL136901<br>ENST00000369720<br>AK026597<br>ENSESTT00000032780<br>ENSESTT00000032781<br>ENST00000369719 | Homo sapiens chromosome 10 open reading frame 79 (C10orf79), mRNA.<br>cdna:known chromosome:NCBI36:10:105917333:105982110:-1 gene:ENSG00000197748<br>cdna:known chromosome:NCBI36:10:105879637:105982089:-1 gene:ENSG00000197748<br>Homo sapiens mRNA; cDNA DKFZp434L086 (from clone DKFZp434L086).<br>cdna:known chromosome:NCBI36:10:105879887:105981994:-1 gene:ENSG00000197748<br>cdna:Genscan chromosome:NCBI36:10:105879887:105918596:-1<br><br><br><br>cdna:Genscan chromosome:NCBI36:10:105922158:105963228:-1<br>Homo sapiens mRNA; cDNA DKFZp434P078 (from clone DKFZp434P078).<br>cdna:known chromosome:NCBI36:10:105942242:105982053:-1 gene:ENSG00000197748<br>Homo sapiens cDNA: FLJ22944 fis, clone KAT08974.<br><br><br>cdna:known chromosome:NCBI36:10:105953178:105982088:-1 gene:ENSG00000197748 |
| 3320347 | 626583 | 3320301 | core | 10748342 | 10748418 | 11 | + | NM_014633<br>ENSESTT00000054867<br>ENSESTT00000054869<br>ENST00000361367<br>ENST00000361944<br>ENSESTT00000054870<br>ENSESTT00000054871<br>ENSESTT00000054872<br>ENSESTT00000054873 | Homo sapiens Ctr9, Paf1/RNA polymerase II complex component, homolog (S. cerevisiae) (CTR9), mRNA.<br><br><br>cdna:known-ccds chromosome:NCBI36:11:10729379:10757863:1 gene:ENSG00000198730 CCDS7805.1<br>cdna:known chromosome:NCBI36:11:10729379:10757863:1 gene:ENSG00000198730 |
| 3326260 | 630422 | 3326252 | core | 34091843 | 34091882 | 11 | + | NM_024662<br>ENST00000257829<br>ENSESTT00000023550<br>GENSCAN00000011468 | Homo sapiens N-acetyltransferase 10 (NAT10), mRNA.<br>cdna:known-ccds chromosome:NCBI36:11:34083725:34125033:1 gene:ENSG00000135372 CCDS7889.1<br><br>cdna:Genscan chromosome:NCBI36:11:34086349:34124315:1 |

| | | | | | | | | | |
|---|---|---|---|---|---|---|---|---|---|
| | | | | | | | | ENSESTT00000023551 | |
| 3330900 | 633161 | 3330897 | core | 55629502 | 55629621 | 11 | + | NM_001005200 | Homo sapiens olfactory receptor, family 8, subfamily H, member 2 (OR8H2), mRNA. |
| 3331572 | 633533 | 3331487 | core | 57333415 | 57333486 | 11 | + | NM_001331<br>ENST00000361332<br>ENST00000361796<br>ENST00000361391<br>AB002382<br>ENSESTT00000015549<br>ENSESTT00000015550<br>ENSESTT00000015554<br>ENSESTT00000015555<br>ENST00000360682<br>ENSESTT00000015552<br>ENSESTT00000015553<br>ENST00000358694<br>GENSCAN00000043817<br>ENSESTT00000015556 | Homo sapiens catenin (cadherin-associated protein), delta 1 (CTNND1), mRNA.<br>cdna:known chromosome:NCBI36:11:57285845:57343226:1 gene:ENSG00000198561<br>cdna:known chromosome:NCBI36:11:57285845:57343226:1 gene:ENSG00000198561<br>cdna:known chromosome:NCBI36:11:57285845:57343226:1 gene:ENSG00000198561<br>Homo sapiens mRNA for KIAA0384 gene, partial cds.<br><br><br><br><br>cdna:known chromosome:NCBI36:11:57236665:57342494:1 gene:ENSG00000198561<br><br><br>cdna:known chromosome:NCBI36:11:57315527:57339644:1 gene:ENSG00000198561<br>cdna:Genscan chromosome:NCBI36:11:57315527:57340100:1 |
| 3332658 | 634180 | 3332626 | core | 60460448 | 60460532 | 11 | + | NM_017870<br>CR613429<br>GENSCAN00000004748<br>NM_178031<br>BC028106<br>ENSESTT00000011406<br>ENSESTT00000011407<br>ENST00000005286<br>AB046803<br>ENST00000345586<br>ENSESTT00000011408<br>ENSESTT00000011409<br>ENSESTT00000011410 | Homo sapiens transmembrane protein 132A (TMEM132A), transcript variant 1, mRNA.<br>full-length cDNA clone CS0DB001YG10 of Neuroblastoma Cot 10-normalized of Homo sapiens (human).<br>cdna:Genscan chromosome:NCBI36:11:60448694:60460955:1<br>Homo sapiens transmembrane protein 132A (TMEM132A), transcript variant 2, mRNA.<br>Homo sapiens transmembrane protein 132A, mRNA (cDNA clone IMAGE:5247458), partial cds.<br><br><br>cdna:known-ccds chromosome:NCBI36:11:60448522:60461206:1 gene:ENSG00000006118 CCDS7997.1<br>Homo sapiens mRNA for KIAA1583 protein, partial cds.<br>cdna:known chromosome:NCBI36:11:60448522:60461206:1 gene:ENSG00000006118 |
| 3335920 | 636016 | 3335907 | core | 65579148 | 65579173 | 11 | + | NM_006842<br>ENSESTT00000058967<br>ENST00000322535<br>ENST00000355456<br>ENSESTT00000058965<br>GENSCAN00000037050<br>BC051237 | Homo sapiens splicing factor 3b, subunit 2, 145kDa (SF3B2), mRNA.<br><br>cdna:known chromosome:NCBI36:11:65576392:65592958:1 gene:ENSG00000087365<br>cdna:known chromosome:NCBI36:11:65576392:65592958:1 gene:ENSG00000087365<br><br>cdna:Genscan chromosome:NCBI36:11:65573035:65592792:1<br>Homo sapiens cDNA clone IMAGE:6082816, **** WARNING: chimeric clone ****. |
| 3343835 | 640688 | 3343832 | core | 88550547 | 88550766 | 11 | + | NM_000372<br>BC027179<br>ENST00000263321<br>ENSESTT00000056621<br>GENSCAN00000064177 | Homo sapiens tyrosinase (oculocutaneous albinism IA) (TYR), mRNA.<br>Homo sapiens tyrosinase (oculocutaneous albinism IA), mRNA (cDNA clone MGC:9191 IMAGE:3923096), complete cds.<br>cdna:known-ccds chromosome:NCBI36:11:88550268:88668474:1 gene:ENSG00000077498 CCDS8284.1<br><br>cdna:Genscan chromosome:NCBI36:11:88550770:88661337:1 |
| 3349368 | 644191 | 3349293 | core | 112610970 | 112611081 | 11 | + | ENST00000316851<br>NM_000615<br>NM_181351<br>AB209443<br>AK057509<br>BC014205<br>X16841<br>M22094<br>ENSESTT00000026747 | cdna:known chromosome:NCBI36:11:112578307:112653781:1 gene:ENSG00000149294<br>Homo sapiens neural cell adhesion molecule 1 (NCAM1), transcript variant 1, mRNA.<br>Homo sapiens neural cell adhesion molecule 1 (NCAM1), transcript variant 2, mRNA.<br>Homo sapiens mRNA for Neural cell adhesion molecule 1, 120 kDa isoform precursor variant protein.<br>Homo sapiens cDNA FLJ32947 fis, clone TESTI2007951, highly similar to NEURAL CELL ADHESION MOLECULE, 140 KDA ISOFORM PRECURSOR.<br>Homo sapiens neural cell adhesion molecule 1, mRNA (cDNA clone IMAGE:4584893), partial cds.<br>Human mRNA for a nontransmembrane isoform of N-CAM from skeletal muscle.<br>Human neural cell adhesion molecule (N-CAM) secreted isoform mRNA, 3' end. |

| | | | | | | | | | |
|---|---|---|---|---|---|---|---|---|---|
| | | | | | | | | ENSESTT00000026748<br>ENSESTT00000026749<br>ENSESTT00000026750<br>ENSESTT00000026751<br>ENSESTT00000026752<br>GENSCAN00000004978 | cdna:Genscan chromosome:NCBI36:11:112590301:112651319:1 |
| 3358234 | 649646 | 3358201 | core | 614384 | 614411 | 11 | - | NM_031265<br>NM_031264<br>BC038405<br>NM_021924<br>AY358368<br>ENST00000358353<br>ENST00000349570<br>GENSCAN00000068250 | Homo sapiens mucin and cadherin-like (MUCDHL), transcript variant 4, mRNA.<br>Homo sapiens mucin and cadherin-like (MUCDHL), transcript variant 3, mRNA.<br>Homo sapiens mucin and cadherin-like, mRNA (cDNA clone MGC:34114 IMAGE:5185584), complete cds.<br>Homo sapiens mucin and cadherin-like (MUCDHL), transcript variant 1, mRNA.<br>Homo sapiens clone DNA102846 MUCDHL (UNQ2781) mRNA, complete cds.<br>cdna:known-ccds chromosome:NCBI36:11:607253:616078:-1 gene:ENSG00000099834 CCDS7707.1<br>cdna:known-ccds chromosome:NCBI36:11:607253:616078:-1 gene:ENSG00000099834 CCDS7708.1<br>cdna:Genscan chromosome:NCBI36:11:602645:614902:-1 |
| 3358383 | 649730 | 3358361 | core | 764008 | 764081 | 11 | - | NM_182612<br>AK128653<br>BC110868<br>ENSESTT00000022238<br>ENSESTT00000022239<br>ENST00000319863<br>ENST00000354286<br>GENSCAN00000021893 | Homo sapiens Parkinson disease 7 domain containing 1 (PDDC1), mRNA.<br>Homo sapiens cDNA FLJ46812 fis, clone TRACH3036278.<br>Homo sapiens Parkinson disease 7 domain containing 1, mRNA (cDNA clone MGC:131881 IMAGE:5726838), complete cds.<br>cdna:known-ccds chromosome:NCBI36:11:757225:767484:-1 gene:ENSG00000177225 CCDS7713.1<br>cdna:known chromosome:NCBI36:11:757225:767487:-1 gene:ENSG00000177225<br>cdna:Genscan chromosome:NCBI36:11:760989:778576:-1 |
| 3381183 | 663285 | 3381150 | core | 71978608 | 71978664 | 11 | - | NM_002599<br>AK095024<br>AK131525<br>AY495087<br>ENST00000334456<br>ENST00000376450<br>GENSCAN00000063350<br>AK092278<br>ENSESTT00000049260<br>ENSESTT00000049255<br>ENSESTT00000049258<br>ENSESTT00000049257 | Homo sapiens phosphodiesterase 2A, cGMP-stimulated (PDE2A), mRNA.<br>Homo sapiens cDNA FLJ37705 fis, clone BRHIP2017404, highly similar to CGMP-DEPENDENT 3',5'-CYCLIC PHOSPHODIESTERASE (EC 3.1.4.17).<br>Homo sapiens cDNA FLJ16750 fis, clone ADRGL2011190, highly similar to cGMP-dependent 3',5'-cyclic phosphodiesterase (EC 3.1.4.17).<br>Homo sapiens cGMP-stimulated phosphodiesterase 4 (PDE2A) mRNA, complete cds, alternatively spliced.<br>cdna:known-ccds chromosome:NCBI36:11:71964834:72063113:-1 gene:ENSG00000186642 CCDS8216.1<br>cdna:known chromosome:NCBI36:11:71964834:72031123:-1 gene:ENSG00000186642<br>cdna:Genscan chromosome:NCBI36:11:71966076:71997443:-1<br>Homo sapiens cDNA FLJ34959 fis, clone NTONG2003454, highly similar to CGMP-DEPENDENT 3',5'-CYCLIC PHOSPHODIESTERASE (EC 3.1.4.17). |
| 3388674 | 667944 | 3388673 | core | 101896496 | 101896563 | 11 | - | NM_002423<br>ENSESTT00000038173<br>ENST00000260227<br>Z11887<br>GENSCAN00000041572 | Homo sapiens matrix metallopeptidase 7 (matrilysin, uterine) (MMP7), mRNA.<br>cdna:known-ccds chromosome:NCBI36:11:101896450:101906688:-1 gene:ENSG00000137673 CCDS8317.1<br>H.sapiens PUMP-1 gene encoding PUMP.<br>cdna:Genscan chromosome:NCBI36:11:101899894:101906641:-1 |
| 3388842 | 668049 | 3388830 | core | 102213248 | 102213311 | 11 | - | NM_002422<br>ENST00000299855<br>ENSESTT00000038145<br>GENSCAN00000016483<br>ENSESTT00000038141<br>ENSESTT00000038143 | Homo sapiens matrix metallopeptidase 3 (stromelysin 1, progelatinase) (MMP3), mRNA.<br>cdna:known-ccds chromosome:NCBI36:11:102211738:102219552:-1 gene:ENSG00000149968 CCDS8323.1<br>cdna:Genscan chromosome:NCBI36:11:102212067:102219487:-1 |
| 3388938 | 668108 | 3388914 | core | 102440229 | 102440316 | 11 | - | NM_032299<br>ENSESTT00000038122 | Homo sapiens DCN1, defective in cullin neddylation 1, domain containing 5 (S. cerevisiae) (DCUN1D5), mRNA. |

| | | | | | | | | | |
|---|---|---|---|---|---|---|---|---|---|
| | | | | | | | | CR619246<br>ENST00000260247<br>GENSCAN00000020915<br>ENSESTT00000038117<br>ENSESTT00000038115<br>ENSESTT00000038118<br>ENSESTT00000038120 | full-length cDNA clone CS0DJ013YA04 of T cells (Jurkat cell line) Cot 10-normalized of Homo sapiens (human).<br>cdna:known-ccds chromosome:NCBI36:11:102438037:102467811:-1 gene:ENSG00000137692 CCDS8325.1<br>cdna:Genscan chromosome:NCBI36:11:102438298:102474263:-1 |
| 3394663 | 671650 | 3394660 | core | 119487484 | 119487662 | 11 | - | NM_012101<br>CR591524<br>CR612298<br>BX648072<br>AK093404<br>AL832678<br>ENST00000341846<br>AF230389<br>ENSESTT00000026735<br>ENSESTT00000026736<br>ENST00000350242<br>GENSCAN00000045646 | Homo sapiens tripartite motif-containing 29 (TRIM29), mRNA.<br>full-length cDNA clone CS0DI004YC15 of Placenta Cot 25-normalized of Homo sapiens (human).<br>full-length cDNA clone CS0DK004YE16 of HeLa cells Cot 25-normalized of Homo sapiens (human).<br>Homo sapiens mRNA; cDNA DKFZp686I03210 (from clone DKFZp686I03210).<br>Homo sapiens cDNA FLJ36085 fis, clone TESTI2020084.<br>Homo sapiens mRNA; cDNA DKFZp313K2117 (from clone DKFZp313K2117).<br>cdna:known-ccds chromosome:NCBI36:11:119487205:119513949:-1 gene:ENSG00000137699 CCDS8428.1<br>Homo sapiens tripartite motif protein TRIM29 beta mRNA, complete cds.<br><br><br>cdna:known-ccds chromosome:NCBI36:11:119487205:119514073:-1 gene:ENSG00000137699 CCDS8429.1<br>cdna:Genscan chromosome:NCBI36:11:119488332:119513949:-1 |
| 3396092 | 672484 | 3396084 | core | 124123807 | 124123869 | 11 | - | NM_014312<br>BC012567<br>ENSESTT00000013808<br>ENSESTT00000013809<br>ENST00000326621<br>GENSCAN00000029044<br>AK000460 | Homo sapiens V-set and immunoglobulin domain containing 2 (VSIG2), mRNA.<br>Homo sapiens V-set and immunoglobulin domain containing 2, mRNA (cDNA clone IMAGE:4273825).<br><br><br>cdna:known-ccds chromosome:NCBI36:11:124122581:124127378:-1 gene:ENSG00000019102 CCDS8452.1<br>cdna:Genscan chromosome:NCBI36:11:124122641:124137260:-1<br>Homo sapiens cDNA FLJ20453 fis, clone KAT05713. |
| 3399071 | 674311 | 3399004 | core | 132032297 | 132032418 | 11 | - | NM_002545<br>NM_001012393<br>BC117254<br>ENSESTT00000011829<br>ENST00000331898<br>ENST00000374778<br>GENSCAN00000038183<br>GENSCAN00000000545 | Homo sapiens opioid binding protein/cell adhesion molecule-like (OPCML), transcript variant 1, mRNA.<br>Homo sapiens opioid binding protein/cell adhesion molecule-like (OPCML), transcript variant 2, mRNA.<br>Homo sapiens opioid binding protein/cell adhesion molecule-like, mRNA (cDNA clone MGC:150863 IMAGE:40125805), complete cds.<br><br>cdna:known-ccds chromosome:NCBI36:11:131795297:132318197:-1 gene:ENSG00000183715 CCDS8492.1<br>cdna:known chromosome:NCBI36:11:131795297:132907429:-1 gene:ENSG00000183715<br>cdna:Genscan chromosome:NCBI36:11:131780561:131904290:-1<br>cdna:Genscan chromosome:NCBI36:11:132030603:132040397:-1 |
| 3416293 | 684999 | 3416290 | core | 52665556 | 52665882 | 12 | + | NM_017409<br>ENSESTT00000038754<br>ENST00000303460<br>GENSCAN00000002166 | Homo sapiens homeobox C10 (HOXC10), mRNA.<br><br>cdna:known-ccds chromosome:NCBI36:12:52665221:52670327:1 gene:ENSG00000180818 CCDS8868.1<br>cdna:Genscan chromosome:NCBI36:12:52665311:52669497:1 |
| 3418272 | 686094 | 3418249 | core | 56251384 | 56251415 | 12 | + | NM_004984<br>AB210045<br>ENSESTT00000030259<br>ENSESTT00000030260<br>ENST00000286452<br>GENSCAN00000024756 | Homo sapiens kinesin family member 5A (KIF5A), mRNA.<br>Homo sapiens mRNA for KIF5A variant protein, partial cds, clone: ph00435.<br><br><br>cdna:known-ccds chromosome:NCBI36:12:56230289:56266682:1 gene:ENSG00000155980 CCDS8945.1<br>cdna:Genscan chromosome:NCBI36:12:56230322:56264284:1 |
| 3422189 | 688516 | 3422144 | core | 70251571 | 70251596 | 12 | + | NM_003667<br>ENSESTT00000044683<br>AK075399<br>ENSESTT00000044682<br>ENST00000266674<br>GENSCAN00000063089 | Homo sapiens leucine-rich repeat-containing G protein-coupled receptor 5 (LGR5), mRNA.<br><br>Homo sapiens cDNA PSEC0089 fis, clone PLACE1001148, highly similar to Homo sapiens orphan G protein-coupled receptor HG38 mRNA.<br><br>cdna:known-ccds chromosome:NCBI36:12:70120031:70264781:1 gene:ENSG00000139292 CCDS9000.1<br>cdna:Genscan chromosome:NCBI36:12:70204453:70264781:1 |

| | | | | | | | | | |
|---|---|---|---|---|---|---|---|---|---|
| 3427285 | 691722 | 3427282 | core | 95567866 | 95567944 | 12 | + | NM_198520<br>ENST00000342887<br>GENSCAN00000000453 | Homo sapiens chromosome 12 open reading frame 63 (C12orf63), mRNA.<br>cdna:known-ccds chromosome:NCBI36:12:95565884:95683179:1 gene:ENSG00000188596 CCDS9062.1<br>cdna:Genscan chromosome:NCBI36:12:95674367:95684386:1 |
| 3428850 | 692710 | 3428845 | core | 101041852 | 101041945 | 12 | + | NM_017915<br>AF274940<br>BC018903<br>BC014313<br>ENSESTT00000002234<br>ENSESTT00000002235<br>ENSESTT00000002237<br>ENST00000327680<br>ENST00000358383<br>ENST00000378128<br>ENSESTT00000002239 | Homo sapiens chromosome 12 open reading frame 48 (C12orf48), mRNA.<br>Homo sapiens PNAS-15 mRNA, complete cds.<br>Homo sapiens chromosome 12 open reading frame 48, mRNA (cDNA clone IMAGE:3951688), complete cds.<br>Homo sapiens chromosome 12 open reading frame 48, mRNA (cDNA clone IMAGE:3681336), with apparent retained intron.<br>cdna:known-ccds chromosome:NCBI36:12:101038155:101115421:1 gene:ENSG00000185480 CCDS9090.1<br>cdna:known chromosome:NCBI36:12:101038155:101115421:1 gene:ENSG00000185480<br>cdna:known chromosome:NCBI36:12:101038155:101115421:1 gene:ENSG00000185480 |
| 3432061 | 694815 | 3432030 | core | 110667111 | 110667156 | 12 | + | NM_025247<br>ENST00000313698<br>AK092356<br>BC015056<br>ENSESTT00000020608<br>ENSESTT00000020609<br>ENSESTT00000020610<br>ENSESTT00000020611<br>ENSESTT00000020612<br>ENSESTT00000020613<br>ENSESTT00000020614<br>GENSCAN00000017682<br>AK097425<br>BC054886<br>ENSESTT00000020617<br>CR591627<br>ENSESTT00000020616<br>ENSESTT00000020619<br>ENSESTT00000020620<br>ENSESTT00000020621<br>ENSESTT00000020622<br>ENSESTT00000020623<br>ENSESTT00000020624 | Homo sapiens acyl-Coenzyme A dehydrogenase family, member 10 (ACAD10), mRNA.<br>cdna:known chromosome:NCBI36:12:110608285:110679284:1 gene:ENSG00000111271<br>Homo sapiens cDNA FLJ35037 fis, clone OCBBF2016729, moderately similar to Probable acyl-CoA dehydrogenase - Deinococcus radiodurans.<br>Homo sapiens acyl-Coenzyme A dehydrogenase family, member 10, mRNA (cDNA clone IMAGE:3922910), complete cds.<br>cdna:Genscan chromosome:NCBI36:12:110614936:110678660:1<br>Homo sapiens cDNA FLJ40106 fis, clone TESTI2006667, moderately similar to Probable acyl-CoA dehydrogenase.<br>Homo sapiens cDNA clone IMAGE:6597672, partial cds.<br>full-length cDNA clone CS0DK002YE05 of HeLa cells Cot 25-normalized of Homo sapiens (human). |
| 3436240 | 697336 | 3436236 | core | 123024036 | 123024288 | 12 | + | NM_152437<br>ENSESTT00000042891<br>ENSESTT00000042892<br>ENSESTT00000042893<br>ENST00000337815 | Homo sapiens zinc finger protein 664 (ZNF664), mRNA.<br>cdna:known-ccds chromosome:NCBI36:12:123023733:123065926:1 gene:ENSG00000179195 CCDS9257.1 |
| 3441868 | 700888 | 3441849 | core | 6312797 | 6312921 | 12 | - | NM_001065<br>ENST00000162749<br>X55313<br>ENSESTT00000024051<br>ENSESTT00000024052<br>GENSCAN00000031724 | Homo sapiens tumor necrosis factor receptor superfamily, member 1A (TNFRSF1A), mRNA.<br>cdna:known-ccds chromosome:NCBI36:12:6308185:6321522:-1 gene:ENSG00000067182 CCDS8542.1<br>H.sapiens TNF-R mRNA for tumor necrosis factor receptor type 1.<br>cdna:Genscan chromosome:NCBI36:12:6308739:6343137:-1 |
| 3442436 | 701195 | 3442427 | core | 6958051 | 6958100 | 12 | - | NM_005768 | Homo sapiens membrane bound O-acyltransferase domain containing 5 (MBOAT5), mRNA. |

| | | | | | | | | | |
|---|---|---|---|---|---|---|---|---|---|
| | | | | | | | | U72517<br>ENST00000261407<br>AK058063<br>AK096775<br>ENSESTT00000024010<br>ENSESTT00000024011<br>ENSESTT00000024012<br>ENSESTT00000024013<br>GENSCAN00000037615<br>ENSESTT00000024014 | Human alternatively spliced variant C7f (C3f) mRNA, partial 3'UTR.<br>cdna:known-ccds chromosome:NCBI36:12:6955608:6996103:-1 gene:ENSG00000111684 CCDS8572.1<br>Homo sapiens cDNA FLJ25334 fis, clone TST00701.<br>Homo sapiens cDNA FLJ39456 fis, clone PROST2010782, highly similar to Human C3f mRNA.<br><br><br><br><br>cdna:Genscan chromosome:NCBI36:12:6956569:6962961:-1 |
| 3442673 | 701323 | 3442641 | core | 7440215 | 7440247 | 12 | - | NM_174941<br>ENSESTT00000006078<br>ENSESTT00000006079<br>ENST00000313599<br>GENSCAN00000000489<br>ENSESTT00000006077<br>AY358856 | Homo sapiens CD163 molecule-like 1 (CD163L1), mRNA.<br><br><br>cdna:known-ccds chromosome:NCBI36:12:7398826:7488015:-1 gene:ENSG00000177675 CCDS8577.1<br>cdna:Genscan chromosome:NCBI36:12:7385475:7417300:-1<br><br>Homo sapiens clone DNA176911 scavenger receptor hlg (UNQ6434) mRNA, complete cds. |
| 3446145 | 703347 | 3446137 | core | 16604651 | 16604739 | 12 | - | NM_018640<br>ENSESTT00000039638<br>NM_001001395<br>ENST00000354662<br>ENST00000320122<br>AB044745<br>AB044746<br>AF258348<br>ENSESTT00000039630<br>ENSESTT00000039631<br>ENSESTT00000039633<br>ENSESTT00000039634<br>ENSESTT00000039636<br>ENSESTT00000039637<br>ENST00000261169<br>ENSESTT00000039632<br>GENSCAN00000047264 | Homo sapiens LIM domain only 3 (rhombotin-like 2) (LMO3), transcript variant 1, mRNA.<br><br>Homo sapiens LIM domain only 3 (rhombotin-like 2) (LMO3), transcript variant 2, mRNA.<br>cdna:known-ccds chromosome:NCBI36:12:16592574:16652291:-1 gene:ENSG00000048540 CCDS8678.1<br>cdna:known chromosome:NCBI36:12:16592574:16645061:-1 gene:ENSG00000048540<br>Homo sapiens Nbla03267 mRNA, complete cds.<br>Homo sapiens Nbla03267 mRNA, complete cds.<br>Homo sapiens neuronal specific transcription factor DAT1 mRNA, complete cds.<br><br><br><br><br><br><br>cdna:known chromosome:NCBI36:12:16592626:16649348:-1 gene:ENSG00000048540<br><br>cdna:Genscan chromosome:NCBI36:12:16644541:16722872:-1 |
| 3454924 | 708744 | 3454892 | core | 50071364 | 50071393 | 12 | - | ENST00000356317<br>GENSCAN00000050354<br>BC035822 | cdna:known-ccds chromosome:NCBI36:12:50033954:50071464:-1 gene:ENSG00000139629 CCDS8813.1<br>cdna:Genscan chromosome:NCBI36:12:50068284:50071481:-1<br>Homo sapiens UDP-N-acetyl-alpha-D-galactosamine:polypeptide N-acetylgalactosaminyltransferase 6 (GalNAc-T6), mRNA (cDNA clone MGC:46032 IMAGE:5552059), complete cds. |
| 3456094 | 709405 | 3456081 | core | 51895740 | 51895776 | 12 | - | NM_000966<br>XM_944827<br>XM_940838<br>XM_944834<br>ENST00000327550<br>ENST00000338561<br>GENSCAN00000055537<br>XM_944829<br>ENSESTT00000038791<br>ENSESTT00000038789<br>ENSESTT00000038790 | Homo sapiens retinoic acid receptor, gamma (RARG), mRNA.<br>PREDICTED: Homo sapiens retinoic acid receptor, gamma, transcript variant 3 (RARG), mRNA.<br>PREDICTED: Homo sapiens retinoic acid receptor, gamma, transcript variant 2 (RARG), mRNA.<br>PREDICTED: Homo sapiens retinoic acid receptor, gamma, transcript variant 5 (RARG), mRNA.<br>cdna:known-ccds chromosome:NCBI36:12:51890621:51912253:-1 gene:ENSG00000172819 CCDS8850.1<br>cdna:known chromosome:NCBI36:12:51890621:51900151:-1 gene:ENSG00000172819<br>cdna:Genscan chromosome:NCBI36:12:51871607:51900151:-1<br>PREDICTED: Homo sapiens retinoic acid receptor, gamma, transcript variant 4 (RARG), mRNA. |
| 3457139 | 710001 | 3457101 | core | 54378878 | 54378904 | 12 | - | NM_002206<br>AY358882<br>ENSESTT00000043167<br>ENSESTT00000043168 | Homo sapiens integrin, alpha 7 (ITGA7), mRNA.<br>Homo sapiens clone DNA55737 ITGA7 (UNQ406) mRNA, complete cds. |

| | | | | | | | | | |
|---|---|---|---|---|---|---|---|---|---|
| | | | | | | | | ENST00000257879<br>ENST00000257880<br>ENST00000347027<br>ENST00000353687<br>GENSCAN00000028867<br>ENSESTT00000043163<br>ENSESTT00000043164<br>ENSESTT00000043165<br>ENSESTT00000043166 | cdna:known-ccds chromosome:NCBI36:12:54364640:54387949:-1 gene:ENSG00000135424 CCDS8888.1<br>cdna:known chromosome:NCBI36:12:54364640:54387752:-1 gene:ENSG00000135424<br>cdna:known chromosome:NCBI36:12:54364640:54387752:-1 gene:ENSG00000135424<br>cdna:known chromosome:NCBI36:12:54364640:54387752:-1 gene:ENSG00000135424<br>cdna:Genscan chromosome:NCBI36:12:54365109:54387733:-1 |
| 3457141 | 710003 | 3457101 | core | 54379921 | 54380036 | 12 | - | NM_002206<br>ENSESTT00000043163<br>AY358882<br>ENSESTT00000043167<br>ENSESTT00000043168<br>ENST00000257879<br>ENST00000257880<br>ENST00000347027<br>ENST00000353687<br>GENSCAN00000028867<br>ENSESTT00000043165<br>ENSESTT00000043164<br>ENSESTT00000043166 | Homo sapiens integrin, alpha 7 (ITGA7), mRNA.<br><br>Homo sapiens clone DNA55737 ITGA7 (UNQ406) mRNA, complete cds.<br><br><br>cdna:known-ccds chromosome:NCBI36:12:54364640:54387949:-1 gene:ENSG00000135424 CCDS8888.1<br>cdna:known chromosome:NCBI36:12:54364640:54387752:-1 gene:ENSG00000135424<br>cdna:known chromosome:NCBI36:12:54364640:54387752:-1 gene:ENSG00000135424<br>cdna:known chromosome:NCBI36:12:54364640:54387752:-1 gene:ENSG00000135424<br>cdna:Genscan chromosome:NCBI36:12:54365109:54387733:-1 |
| 3457678 | 710289 | 3457667 | core | 54991264 | 54991370 | 12 | - | NM_014255<br>CR625337<br>BC065015<br>ENSESTT00000051237<br>ENST00000273308<br>GENSCAN00000024722<br>ENSESTT00000051236<br>BC001027<br>CR596106 | Homo sapiens transmembrane protein 4 (TMEM4), mRNA.<br>full-length cDNA clone CS0DI027YB14 of Placenta Cot 25-normalized of Homo sapiens (human).<br>Homo sapiens transmembrane protein 4, mRNA (cDNA clone MGC:74660 IMAGE:6169229), complete cds.<br><br>cdna:known-ccds chromosome:NCBI36:12:54990484:54996387:-1 gene:ENSG00000144785 CCDS8914.1<br>cdna:Genscan chromosome:NCBI36:12:54953135:55013145:-1<br><br>Homo sapiens transmembrane protein 4, mRNA (cDNA clone IMAGE:3344788), complete cds.<br>full-length cDNA clone CS0DI062YO15 of Placenta Cot 25-normalized of Homo sapiens (human). |
| 3470604 | 718347 | 3470597 | core | 107710006 | 107710150 | 12 | - | AK095421<br>CR623842<br>ENST00000326470 | Homo sapiens cDNA FLJ38102 fis, clone D3OST2000618, moderately similar to Drosophila melanogaster slingshot mRNA.<br>full-length cDNA clone CS0DC021YJ15 of Neuroblastoma Cot 25-normalized of Homo sapiens (human).<br>cdna:known chromosome:NCBI36:12:107709831:107745451:-1 gene:ENSG00000084112 |
| 3486907 | 728606 | 3486883 | core | 40830459 | 40830573 | 13 | + | NM_024561<br>ENSESTT00000005191<br>ENST00000379372<br>NM_018527<br>ENSESTT00000005190<br>ENSESTT00000005189<br>AK097224<br>BX640665<br>ENST00000379406<br>ENST00000325763<br>ENST00000311541<br>ENST00000379367<br>ENST00000347166<br>BC017099<br>ENSESTT00000005192<br>ENST00000379366<br>ENSESTT00000005193<br>ENSESTT00000005194 | Homo sapiens NMDA receptor regulated 1-like (NARG1L), transcript variant 1, mRNA.<br><br>cdna:known chromosome:NCBI36:13:40783418:40830854:1 gene:ENSG00000172766<br>Homo sapiens NMDA receptor regulated 1-like (NARG1L), transcript variant 2, mRNA.<br><br><br>Homo sapiens cDNA FLJ39905 fis, clone SPLEN2017351, moderately similar to Xenopus laevis N-terminal acetyltransferase mRNA.<br>Homo sapiens mRNA; cDNA DKFZp686O08147 (from clone DKFZp686O08147).<br>cdna:known-ccds chromosome:NCBI36:13:40783416:40849166:1 gene:ENSG00000172766 CCDS9379.1<br>cdna:known-ccds chromosome:NCBI36:13:40783418:40849162:1 gene:ENSG00000172766 CCDS9380.1<br>cdna:known chromosome:NCBI36:13:40783418:40849162:1 gene:ENSG00000172766<br>cdna:known chromosome:NCBI36:13:40783431:40841340:1 gene:ENSG00000172766<br>cdna:known chromosome:NCBI36:13:40783447:40849166:1 gene:ENSG00000172766<br>Homo sapiens NMDA receptor regulated 1-like, mRNA (cDNA clone IMAGE:3357835), partial cds.<br><br>cdna:known chromosome:NCBI36:13:40834591:40849166:1 gene:ENSG00000172766 |

| | | | | | | | | | |
|---|---|---|---|---|---|---|---|---|---|
| 3490674 | 730939 | 3490655 | core | 51937634 | 51937893 | 13 | + | AY062262<br>ENST00000378034<br>BC010901 | Homo sapiens tumor-associated microtubule-associated protein splice variant (TMAP) mRNA, complete cds; alternatively spliced.<br>cdna:known chromosome:NCBI36:13:51933609:51937946:1 gene:ENSG00000136108<br>Homo sapiens cytoskeleton associated protein 2, mRNA (cDNA clone IMAGE:4096950), complete cds. |
| 3504635 | 739738 | 3504617 | core | 20644573 | 20644642 | 13 | - | NM_145061<br>ENST00000298260<br>ENST00000314759<br>GENSCAN00000002709 | Homo sapiens chromosome 13 open reading frame 3 (C13orf3), mRNA.<br>cdna:known chromosome:NCBI36:13:20625734:20648687:-1 gene:ENSG00000165480<br>cdna:known chromosome:NCBI36:13:20625735:20648710:-1 gene:ENSG00000165480<br>cdna:Genscan chromosome:NCBI36:13:20626798:20649337:-1 |
| 3505958 | 740565 | 3505937 | core | 24361511 | 24361538 | 13 | - | NM_018451<br>AF141343<br>ENSESTT00000016067<br>AK123551<br>BC113111<br>ENST00000381884<br>ENST00000281593<br>GENSCAN00000036673<br>ENSESTT00000016066 | Homo sapiens centromere protein J (CENPJ), mRNA.<br>Homo sapiens LYST-interacting protein LIP7 mRNA, partial cds.<br><br>Homo sapiens cDNA FLJ41557 fis, clone COLON2008105, highly similar to Homo sapiens LYST-interacting protein LIP1 mRNA.<br>Homo sapiens centromere protein J, mRNA (cDNA clone MGC:131582 IMAGE:7961390), complete cds.<br>cdna:known-ccds chromosome:NCBI36:13:24355171:24395018:-1 gene:ENSG00000151849 CCDS9310.1<br>cdna:known chromosome:NCBI36:13:24355172:24395018:-1 gene:ENSG00000151849<br>cdna:Genscan chromosome:NCBI36:13:24355315:24454642:-1 |
| 3510089 | 743163 | 3510066 | core | 37046714 | 37046754 | 13 | - | NM_006475<br>ENSESTT00000017845<br>ENSESTT00000017846<br>ENST00000379747<br>ENST00000379749<br>ENST00000379743<br>ENST00000379742<br>ENST00000350926<br>ENST00000255475<br>ENST00000379733<br>ENST00000349737<br>GENSCAN00000025140<br>AK021444<br>ENSESTT00000017843<br>ENSESTT00000017844 | Homo sapiens periostin, osteoblast specific factor (POSTN), mRNA.<br><br><br>cdna:known-ccds chromosome:NCBI36:13:37034726:37070893:-1 gene:ENSG00000133110 CCDS9364.1<br>cdna:known chromosome:NCBI36:13:37034722:37070898:-1 gene:ENSG00000133110<br>cdna:known chromosome:NCBI36:13:37034728:37070887:-1 gene:ENSG00000133110<br>cdna:known chromosome:NCBI36:13:37034771:37070895:-1 gene:ENSG00000133110<br>cdna:known chromosome:NCBI36:13:37034776:37070885:-1 gene:ENSG00000133110<br>cdna:known chromosome:NCBI36:13:37034779:37070874:-1 gene:ENSG00000133110<br>cdna:known chromosome:NCBI36:13:37034779:37070874:-1 gene:ENSG00000133110<br>cdna:known chromosome:NCBI36:13:37034779:37070874:-1 gene:ENSG00000133110<br>cdna:Genscan chromosome:NCBI36:13:37038790:37052834:-1<br>Homo sapiens cDNA FLJ11382 fis, clone HEMBA1000504. |
| 3510363 | 743342 | 3510362 | core | 38482055 | 38482272 | 13 | - | ENST00000358672<br>NM_025138<br>NM_170719<br>ENST00000350125<br>ENST00000352251<br>ENST00000379609 | cdna:known chromosome:NCBI36:13:38482002:38510186:-1 gene:ENSG00000120685<br>Homo sapiens chromosome 13 open reading frame 23 (C13orf23), transcript variant 1, mRNA.<br>Homo sapiens chromosome 13 open reading frame 23 (C13orf23), transcript variant 2, mRNA.<br>cdna:known-ccds chromosome:NCBI36:13:38482003:38510213:-1 gene:ENSG00000120685 CCDS9368.1<br>cdna:known chromosome:NCBI36:13:38482003:38510213:-1 gene:ENSG00000120685<br>cdna:known chromosome:NCBI36:13:38482003:38510213:-1 gene:ENSG00000120685 |
| 3516014 | 746922 | 3515965 | core | 59383887 | 59383953 | 13 | - | NM_030932<br>ENSESTT00000005232<br>ENST00000377908<br>ENST00000267215<br>NM_001042517<br>ENSESTT00000005231<br>ENST00000267214<br>GENSCAN00000013585<br>ENSESTT00000005230<br>AL137718<br>GENSCAN00000057766<br>ENSESTT00000005229 | Homo sapiens diaphanous homolog 3 (Drosophila) (DIAPH3), transcript variant 2, mRNA.<br><br>cdna:known chromosome:NCBI36:13:59246299:59635901:-1 gene:ENSG00000139734<br>cdna:known chromosome:NCBI36:13:59246299:59635901:-1 gene:ENSG00000139734<br>Homo sapiens diaphanous homolog 3 (Drosophila) (DIAPH3), transcript variant 1, mRNA.<br><br>cdna:known chromosome:NCBI36:13:59138719:59485348:-1 gene:ENSG00000139734<br>cdna:Genscan chromosome:NCBI36:13:59272779:59388384:-1<br><br>Homo sapiens mRNA; cDNA DKFZp434C0931 (from clone DKFZp434C0931).<br>cdna:Genscan chromosome:NCBI36:13:59396910:59534042:-1 |

| | | | | | | | | | |
|---|---|---|---|---|---|---|---|---|---|
| 3516031 | 746936 | 3515965 | core | 59446497 | 59446540 | 13 | - | NM_030932<br>ENSESTT00000005232<br>ENST00000377908<br>ENST00000267215<br>NM_001042517<br>ENSESTT00000005231<br>ENST00000267214<br>GENSCAN00000013585<br>ENSESTT00000005230<br>AL137718<br>GENSCAN00000057766<br>ENSESTT00000005229 | Homo sapiens diaphanous homolog 3 (Drosophila) (DIAPH3), transcript variant 2, mRNA.<br><br>cdna:known chromosome:NCBI36:13:59246299:59635901:-1 gene:ENSG00000139734<br>cdna:known chromosome:NCBI36:13:59246299:59635901:-1 gene:ENSG00000139734<br>Homo sapiens diaphanous homolog 3 (Drosophila) (DIAPH3), transcript variant 1, mRNA.<br><br>cdna:known chromosome:NCBI36:13:59138719:59485348:-1 gene:ENSG00000139734<br>cdna:Genscan chromosome:NCBI36:13:59272779:59388384:-1<br><br>Homo sapiens mRNA; cDNA DKFZp434C0931 (from clone DKFZp434C0931).<br>cdna:Genscan chromosome:NCBI36:13:59396910:59534042:-1 |
| 3547382 | 766249 | 3547375 | core | 87548112 | 87548148 | 14 | + | NM_003608<br>ENST00000267549 | Homo sapiens G protein-coupled receptor 65 (GPR65), mRNA.<br>cdna:known-ccds chromosome:NCBI36:14:87541249:87548164:1 gene:ENSG00000140030 CCDS9879.1 |
| 3555425 | 771230 | 3555340 | core | 19946406 | 19946436 | 14 | - | NM_007110<br>ENSESTT00000007014<br>BX640983<br>ENSESTT00000007019<br>ENST00000262715<br>ENST00000359243<br>GENSCAN00000012529<br>ENSESTT00000007020<br>ENSESTT00000007021<br>ENSESTT00000007018<br>ENSESTT00000007016<br>ENSESTT00000007017 | Homo sapiens telomerase-associated protein 1 (TEP1), mRNA.<br><br>Homo sapiens mRNA; cDNA DKFZp686A04252 (from clone DKFZp686A04252).<br><br>cdna:known-ccds chromosome:NCBI36:14:19905755:19951426:-1 gene:ENSG00000129566 CCDS9548.1<br>cdna:known chromosome:NCBI36:14:19905755:19951426:-1 gene:ENSG00000129566<br>cdna:Genscan chromosome:NCBI36:14:19906436:19973810:-1 |
| 3557012 | 772062 | 3556990 | core | 22521432 | 22521458 | 14 | - | NM_032876<br>GENSCAN00000022101<br>NM_198086<br>ENST00000262713<br>ENST00000361265<br>ENSESTT00000058313 | Homo sapiens jub, ajuba homolog (Xenopus laevis) (JUB), transcript variant 1, mRNA.<br>cdna:Genscan chromosome:NCBI36:14:22485574:22538250:-1<br>Homo sapiens jub, ajuba homolog (Xenopus laevis) (JUB), transcript variant 2, mRNA.<br>cdna:known-ccds chromosome:NCBI36:14:22510249:22521691:-1 gene:ENSG00000129474 CCDS9581.1<br>cdna:known-ccds chromosome:NCBI36:14:22510249:22521691:-1 gene:ENSG00000129474 CCDS9582.1 |
| 3557013 | 772062 | 3556990 | core | 22521460 | 22521520 | 14 | - | NM_032876<br>NM_198086<br>ENST00000262713<br>ENST00000361265<br>GENSCAN00000022101<br>ENSESTT00000058313 | Homo sapiens jub, ajuba homolog (Xenopus laevis) (JUB), transcript variant 1, mRNA.<br>Homo sapiens jub, ajuba homolog (Xenopus laevis) (JUB), transcript variant 2, mRNA.<br>cdna:known-ccds chromosome:NCBI36:14:22510249:22521691:-1 gene:ENSG00000129474 CCDS9581.1<br>cdna:known-ccds chromosome:NCBI36:14:22510249:22521691:-1 gene:ENSG00000129474 CCDS9582.1<br>cdna:Genscan chromosome:NCBI36:14:22485574:22538250:-1 |
| 3557859 | 772515 | 3557851 | core | 23721027 | 23721065 | 14 | - | NM_024658<br>AB209476<br>AK094897<br>ENSESTT00000007467<br>ENSESTT00000007469<br>GENSCAN00000004003<br>ENST00000354464<br>ENSESTT00000007468 | Homo sapiens importin 4 (IPO4), mRNA.<br>Homo sapiens mRNA for importin 4 variant protein.<br>Homo sapiens cDNA FLJ37578 fis, clone BRCOC2003732.<br><br><br>cdna:Genscan chromosome:NCBI36:14:23719488:23744036:-1<br>cdna:known chromosome:NCBI36:14:23719488:23727833:-1 gene:ENSG00000196497 |
| 3569228 | 779462 | 3569200 | core | 66879842 | 66879902 | 14 | - | NM_015994<br>AF100741<br>AK001155<br>BC025373<br>CR612882<br>CR619369 | Homo sapiens ATPase, H+ transporting, lysosomal 34kDa, V1 subunit D (ATP6V1D), mRNA.<br>Homo sapiens vacuolar H-ATPase subunit D mRNA, complete cds.<br>Homo sapiens cDNA FLJ10293 fis, clone NT2RM1000280, highly similar to VACUOLAR ATP SYNTHASE SUBUNIT D (EC 3.6.1.34).<br>Homo sapiens ATPase, H+ transporting, lysosomal 34kDa, V1 subunit D, mRNA (cDNA clone MGC:22422 IMAGE:4108457), complete cds. |

| | | | | | | | | | |
|---|---|---|---|---|---|---|---|---|---|
| | | | | | | | | ENSESTT00000003300<br>ENSESTT00000003301<br>ENST00000216442<br>GENSCAN00000066247<br>ENSESTT00000003299<br>ENSESTT00000003302 | full-length cDNA clone CS0DA008YH16 of Neuroblastoma of Homo sapiens (human).<br>full-length cDNA clone CS0DG004YJ05 of B cells (Ramos cell line) of Homo sapiens (human).<br><br><br>cdna:known-ccds chromosome:NCBI36:14:66830841:66896261:-1 gene:ENSG00000100554 CCDS9780.1<br>cdna:Genscan chromosome:NCBI36:14:66875091:66897217:-1 |
| 3569830 | 779807 | 3569814 | core | 68415464 | 68415535 | 14 | - | NM_001102<br>CR620987<br>AK124378<br>ENST00000193403<br>AK098203<br>BX641076<br>M95178<br>ENSESTT00000029087<br>ENSESTT00000029088<br>ENSESTT00000029089<br>GENSCAN00000045145<br>BX248020 | Homo sapiens actinin, alpha 1 (ACTN1), mRNA.<br>full-length cDNA clone CS0DM010YM22 of Fetal liver of Homo sapiens (human).<br>Homo sapiens cDNA FLJ42387 fis, clone UTERU2037791, highly similar to Human mRNA for alpha-actinin.<br>cdna:known-ccds chromosome:NCBI36:14:68410793:68515747:-1 gene:ENSG00000072110 CCDS9792.1<br>Homo sapiens cDNA FLJ40884 fis, clone UTERU2000607, highly similar to ALPHA-ACTININ, SMOOTH MUSCLE ISOFORM.<br>Homo sapiens mRNA; cDNA DKFZp686J22226 (from clone DKFZp686J22226).<br>Human non-muscle alpha-actinin mRNA, complete cds.<br><br><br><br>cdna:Genscan chromosome:NCBI36:14:68411329:68542331:-1<br>human full-length cDNA clone CS0DB009YA03 of Neuroblastoma of Homo sapiens (human). |
| 3573884 | 782351 | 3573870 | core | 79738833 | 79739046 | 14 | - | NM_013989<br>AB041843<br>AB041844<br>BC063118<br>ENSESTT00000052738<br>ENSESTT00000052739<br>ENSESTT00000052740<br>ENSESTT00000052742<br>ENST00000388841<br>ENST00000388838<br>GENSCAN00000064922<br>NM_000793<br>NM_001007023<br>ENST00000388840<br>ENSESTT00000052741<br>ENST00000388839 | Homo sapiens deiodinase, iodothyronine, type II (DIO2), transcript variant 1, mRNA.<br>Homo sapiens mRNA for type II iodothyronine deiodinase, complete cds.<br>Homo sapiens mRNA for type II iodothyronine deiodinase, complete cds.<br>Homo sapiens deiodinase, iodothyronine, type II, mRNA (cDNA clone IMAGE:30351753), complete cds.<br><br><br><br><br>cdna:known chromosome:NCBI36:14:79736595:79747604:-1 gene:ENSG00000211448<br>cdna:known chromosome:NCBI36:14:79738809:79747568:-1 gene:ENSG00000211448<br>cdna:Genscan chromosome:NCBI36:14:79738809:79747568:-1<br>Homo sapiens deiodinase, iodothyronine, type II (DIO2), transcript variant 2, mRNA.<br>Homo sapiens deiodinase, iodothyronine, type II (DIO2), transcript variant 3, mRNA.<br>cdna:known chromosome:NCBI36:14:79738694:79747596:-1 gene:ENSG00000211448<br><br>cdna:known chromosome:NCBI36:14:79738915:79747409:-1 gene:ENSG00000211448 |
| 3577465 | 784698 | 3577443 | core | 93493095 | 93493316 | 14 | - | NM_016150<br>AK093271<br>ENST00000315988<br>AK057078<br>AK002049<br>ENSESTT00000021180<br>ENSESTT00000021178 | Homo sapiens ankyrin repeat and SOCS box-containing 2 (ASB2), mRNA.<br>Homo sapiens cDNA FLJ35952 fis, clone TESTI2012152, highly similar to Homo sapiens ankyrin repeat-containing protein ASB-2 mRNA.<br>cdna:known-ccds chromosome:NCBI36:14:93470266:93493520:-1 gene:ENSG00000100628 CCDS9915.1<br>Homo sapiens cDNA FLJ32516 fis, clone SMINT1000103, highly similar to Homo sapiens ankyrin repeat-containing protein ASB-2 mRNA.<br>Homo sapiens cDNA FLJ11187 fis, clone PLACE1007537, highly similar to Homo sapiens ankyrin repeat-containing protein ASB-2 mRNA. |
| 3581443 | 787069 | 3581442 | core | 104679281 | 104679842 | 14 | - | NM_002226<br>AB209370<br>NM_145159<br>ENST00000331782<br>ENST00000347004<br>ENSESTT00000049527 | Homo sapiens jagged 2 (JAG2), transcript variant 1, mRNA.<br>Homo sapiens mRNA for jagged 2 isoform b precursor variant protein.<br>Homo sapiens jagged 2 (JAG2), transcript variant 2, mRNA.<br>cdna:known-ccds chromosome:NCBI36:14:104679121:104706206:-1 gene:ENSG00000184916 CCDS9998.1<br>cdna:known-ccds chromosome:NCBI36:14:104679121:104706206:-1 gene:ENSG00000184916 CCDS9999.1 |

| | | | | | | | | | |
|---|---|---|---|---|---|---|---|---|---|
| | | | | | | | | GENSCAN00000010972<br>ENSESTT00000049526<br>ENSESTT00000049524<br>ENSESTT00000049521<br>ENSESTT00000049520 | cdna:Genscan chromosome:NCBI36:14:104680077:104705802:-1 |
| 3581693 | 787210 | 3581637 | core | 105161956 | 105161980 | 14 | - | XM_940985<br>AK128477<br>BC111019<br>GENSCAN00000064310<br>BX640824<br>ENST00000379913<br>BC025985<br>ENSESTT00000049495<br>GENSCAN00000064300<br>BC037361 | PREDICTED: Homo sapiens similar to Ig gamma-4 chain C region (LOC651766), mRNA.<br>Homo sapiens cDNA FLJ46622 fis, clone TLUNG2001600, highly similar to Ig gamma-4 chain C region.<br>Homo sapiens immunoglobulin heavy constant gamma 4 (G4m marker), mRNA (cDNA clone MGC:117419 IMAGE:5211387), complete cds.<br>cdna:Genscan chromosome:NCBI36:14:105159294:105175324:-1<br>Homo sapiens mRNA; cDNA DKFZp686M24218 (from clone DKFZp686M24218).<br>cdna:known chromosome:NCBI36:14:105161725:105163451:-1 gene:ENSG00000205402<br>Homo sapiens immunoglobulin heavy constant gamma 4 (G4m marker), mRNA (cDNA clone MGC:22866 IMAGE:4042623), complete cds.<br><br>cdna:Genscan chromosome:NCBI36:14:105178347:105182529:-1<br>Homo sapiens immunoglobulin heavy constant gamma 1 (G1m marker), mRNA (cDNA clone MGC:45938 IMAGE:5440797), complete cds. |
| 3581841 | 787296 | 3581637 | core | 105280186 | 105280216 | 14 | - | AK128394<br>AK129777<br>AY515004<br>AY515010<br>DQ497189<br>U07985<br>L13487<br>CR592363<br>CR594000<br>ENSESTT00000049487<br>XM_939003<br>BC019337<br>ENSESTT00000049488<br>BC073789<br>BX640947<br>AK092976<br>AK093636<br>AK097206<br>AK097350<br>AK097354<br>AK097357<br>AK097360<br>AK097361<br>AK097365<br>AK097366<br>AK097367<br>AK097950<br>AK126281<br>AK127415<br>AK129508<br>AK129510<br>AK129512<br>AK129514<br>AK129517<br>AK129622 | Homo sapiens cDNA FLJ46537 fis, clone THYMU3037772, highly similar to Ig gamma-1 chain C region.<br>Homo sapiens cDNA FLJ26266 fis, clone DMC05613.<br>Homo sapiens anti-HIV-1 gp120 immunoglobulin 16c heavy chain mRNA, partial cds.<br>Homo sapiens anti-HIV-1 gp120 immunoglobulin 412d heavy chain mRNA, partial cds.<br>Homo sapiens nonfunctional anti-SARS-CoV S protein immunoglobulin heavy chain Fd fragment mRNA, partial sequence.<br>Human myeloma immunoglobulin heavy chain Fd region V-D-J-CH1 mRNA, partial cds.<br>Human IgG4 chain V-region CH1 domain mRNA.<br>full-length cDNA clone CS0DE006YK04 of Placenta of Homo sapiens (human).<br>full-length cDNA clone CS0DI017YH15 of Placenta Cot 25-normalized of Homo sapiens (human).<br><br>PREDICTED: Homo sapiens similar to Ig gamma-2 chain C region (LOC649923), mRNA.<br>Homo sapiens immunoglobulin heavy constant gamma 1 (G1m marker), mRNA (cDNA clone MGC:12853 IMAGE:4054679), complete cds.<br><br>Homo sapiens immunoglobulin heavy constant gamma 1 (G1m marker), mRNA (cDNA clone MGC:88807 IMAGE:5480220), complete cds.<br>Homo sapiens mRNA; cDNA DKFZp686G11190 (from clone DKFZp686G11190).<br>Homo sapiens cDNA FLJ35657 fis, clone SPLEN2013910, highly similar to Human (hybridoma H210) anti-hepatitis A IgG.<br>Homo sapiens cDNA FLJ36317 fis, clone THYMU2005283, highly similar to Homo sapiens mRNA for immunoglobulin lambda heavy chain.<br>Homo sapiens cDNA FLJ39887 fis, clone SPLEN2016531, highly similar to Homo sapiens mRNA for immunoglobulin lambda heavy chain.<br>Homo sapiens cDNA FLJ40031 fis, clone STOMA2009253, highly similar to Homo sapiens mRNA for immunoglobulin lambda heavy chain.<br>Homo sapiens cDNA FLJ40035 fis, clone SYNOV2000173, highly similar to Human (hybridoma H210) anti-hepatitis A IgG variable region, constant region, complementarity-determining regions mRNA.<br>Homo sapiens cDNA FLJ40038 fis, clone SYNOV2000297, highly similar to Human (hybridoma H210) anti-hepatitis A IgG variable region, constant region, complementarity-determining regions mRNA.<br>Homo sapiens cDNA FLJ40041 fis, clone SYNOV2000700, highly similar to Human (hybridoma H210) anti-hepatitis A IgG variable region, constant region, complementarity-determining regions mRNA.<br>Homo sapiens cDNA FLJ40042 fis, clone SYNOV2000824, highly similar to Human (hybridoma H210) anti-hepatitis A IgG variable region, constant region, complementarity-determining regions mRNA.<br>Homo sapiens cDNA FLJ40046 fis, clone SYNOV2001300, highly similar to Ig gamma =immunoglobulin heavy |

| | | | | | | | | | |
|---|---|---|---|---|---|---|---|---|---|
| | | | | | | | | AK129637 | chain. |
| | | | | | | | | AK130577 | Homo sapiens cDNA FLJ40047 fis, clone SYNOV2001356, highly similar to Homo sapiens mRNA for |
| | | | | | | | | AK130589 | immunoglobulin lambda heavy chain. |
| | | | | | | | | AK130611 | Homo sapiens cDNA FLJ40048 fis, clone SYNOV2001390, highly similar to Homo sapiens mRNA for |
| | | | | | | | | AK130642 | immunoglobulin kappa heavy chain. |
| | | | | | | | | BC014667 | Homo sapiens cDNA FLJ40631 fis, clone THYMU2014777, highly similar to Homo sapiens mRNA for |
| | | | | | | | | BC073782 | immunoglobulin lambda heavy chain. |
| | | | | | | | | BC078671 | Homo sapiens cDNA FLJ44295 fis, clone TRACH2022649, highly similar to Ig gamma-1 chain C region. |
| | | | | | | | | BC090940 | Homo sapiens cDNA FLJ45507 fis, clone BRTHA2020721, highly similar to Ig gamma-1 chain C region. |
| | | | | | | | | Y14735 | Homo sapiens cDNA FLJ25997 fis, clone DMC06923, highly similar to Ig gamma-1 chain C region. |
| | | | | | | | | BX538118 | Homo sapiens cDNA FLJ25999 fis, clone DMC07355, highly similar to Ig gamma-1 chain C region. |
| | | | | | | | | BX640619 | Homo sapiens cDNA FLJ26001 fis, clone DMC07585, highly similar to Ig gamma-1 chain C region. |
| | | | | | | | | AK130585 | Homo sapiens cDNA FLJ26003 fis, clone DMC07928, highly similar to Ig gamma-1 chain C region. |
| | | | | | | | | BC064496 | Homo sapiens cDNA FLJ26006 fis, clone DMC08725, highly similar to Ig gamma-1 chain C region. |
| | | | | | | | | AK057754 | Homo sapiens cDNA FLJ26111 fis, clone SPL06037, highly similar to Ig gamma-1 chain C region. |
| | | | | | | | | AK097359 | Homo sapiens cDNA FLJ26126 fis, clone TMS02464, highly similar to Ig gamma-1 chain C region. |
| | | | | | | | | AK097363 | Homo sapiens cDNA FLJ27067 fis, clone SPL01356, highly similar to Ig gamma-1 chain C region. |
| | | | | | | | | AK125632 | Homo sapiens cDNA FLJ27079 fis, clone SPL02746, highly similar to Ig gamma-1 chain C region. |
| | | | | | | | | AK130586 | Homo sapiens cDNA FLJ27101 fis, clone SPL04668, highly similar to Ig gamma-1 chain C region. |
| | | | | | | | | AY172959 | Homo sapiens cDNA FLJ27132 fis, clone SPL08253, highly similar to Ig gamma-1 chain C region. |
| | | | | | | | | AK128313 | Homo sapiens immunoglobulin heavy constant gamma 1 (G1m marker), mRNA (cDNA clone MGC:23153 |
| | | | | | | | | AK129776 | IMAGE:4850078), complete cds. |
| | | | | | | | | AB004303 | Homo sapiens cDNA clone MGC:88796 IMAGE:6295732, complete cds. |
| | | | | | | | | AB022655 | Homo sapiens immunoglobulin heavy constant gamma 1 (G1m marker), mRNA (cDNA clone MGC:88811 |
| | | | | | | | | AB027435 | IMAGE:6281248), complete cds. |
| | | | | | | | | AB027437 | Homo sapiens immunoglobulin heavy constant gamma 1 (G1m marker), mRNA (cDNA clone MGC:105011 |
| | | | | | | | | AB027441 | IMAGE:3059913), complete cds. |
| | | | | | | | | AB027443 | Homo sapiens mRNA for immunoglobulin kappa heavy chain. |
| | | | | | | | | AB027445 | Homo sapiens mRNA; cDNA DKFZp686N02209 (from clone DKFZp686N02209). |
| | | | | | | | | AB027447 | Homo sapiens mRNA; cDNA DKFZp686H20196 (from clone DKFZp686H20196). |
| | | | | | | | | AB030639 | Homo sapiens cDNA FLJ27075 fis, clone SPL02093, highly similar to Ig gamma-1 chain C region. |
| | | | | | | | | AB245095 | Homo sapiens immunoglobulin heavy constant gamma 1 (G1m marker), mRNA (cDNA clone MGC:71313 |
| | | | | | | | | AF184764 | IMAGE:6280923), complete cds. |
| | | | | | | | | AF400158 | Homo sapiens cDNA FLJ25025 fis, clone CBL01928, highly similar to Ig gamma immunoglobulin heavy chain. |
| | | | | | | | | AK057775 | Homo sapiens cDNA FLJ40040 fis, clone SYNOV2000426, highly similar to Homo sapiens mRNA for |
| | | | | | | | | AK093806 | immunoglobulin lambda heavy chain. |
| | | | | | | | | AK097010 | Homo sapiens cDNA FLJ40044 fis, clone SYNOV2001088, highly similar to Human (hybridoma H210) |
| | | | | | | | | AK097356 | anti-hepatitis A IgG variable region, constant region, complementarity-determining regions mRNA. |
| | | | | | | | | AK097364 | Homo sapiens cDNA FLJ43644 fis, clone SYNOV3000231, highly similar to Ig gamma-1 chain C region. |
| | | | | | | | | AK123070 | Homo sapiens cDNA FLJ27076 fis, clone SPL02374, highly similar to Ig gamma-1 chain C region. |
| | | | | | | | | AK123800 | Homo sapiens anti-rabies SOJA immunoglobulin heavy chain mRNA, complete cds. |
| | | | | | | | | AK127409 | Homo sapiens cDNA FLJ46454 fis, clone THYMU3020221, highly similar to Ig gamma-1 chain C region. |
| | | | | | | | | AK129809 | Homo sapiens cDNA FLJ26265 fis, clone DMC05516, highly similar to Ig gamma-1 chain C region. |
| | | | | | | | | AK130434 | Homo sapiens mRNA for IgG heavy chain, partial cds. |
| | | | | | | | | AK130539 | Homo sapiens mRNA for anti-Entamoeba histolytica immunoglobulin gamma heavy chain (V-CH1 region), partial |
| | | | | | | | | AM183975 | cds, clone:E244-H1. |
| | | | | | | | | AY623427 | Homo sapiens mRNA for anti TNF-alpha antibody heavy-chain Fab fragment, partial cds, clone:1F8-gk10H. |
| | | | | | | | | AY885219 | Homo sapiens mRNA for anti TNF-alpha antibody heavy-chain Fab fragment, partial cds, clone:1F8-gk20H. |
| | | | | | | | | AY894992 | Homo sapiens mRNA for anti HBs antibody heavy-chain Fab fragment, partial cds, clone:303-15H. |
| | | | | | | | | BC016381 | Homo sapiens mRNA for anti HBs antibody heavy-chain Fab fragment, partial cds, clone:305-51H. |
| | | | | | | | | BC018747 | Homo sapiens mRNA for anti HBs antibody heavy-chain Fab fragment, partial cds, clone:308-09H. |
| | | | | | | | | BC025314 | Homo sapiens mRNA for anti HBs antibody heavy-chain Fab fragment, partial cds, clone:308-19H. |
| | | | | | | | | BC026038 | Homo sapiens mRNA for immunoglobulin gamma heavy chain, partial cds, V and CH1 region, specific for |
| | | | | | | | | BC041037 | Entamoeba histolytica. |

| | | | | | | | | | |
|---|---|---|---|---|---|---|---|---|---|
| | | | | | | | | BC053984<br>BC062336<br>BC065820<br>BC066642<br>BC069016<br>BC069020<br>BC075840<br>BC075846<br>BC078670<br>BC080557<br>BC089417<br>BC090939<br>BC092518<br>AJ309318<br>U92452<br>L01410<br>CR933690<br>BX640620<br>BX640621<br>BX640622<br>BX640627<br>BX640853<br>NM_001040077<br>AB159728<br>AK092847<br>AK097859<br>AK098516<br>AK126132<br>AK128301<br>AK129812<br>AK130786<br>AK130813<br>AY172957<br>BC006402<br>BC024289<br>BC037361<br>BC051328<br>BC072419<br>BC073766<br>BC075842<br>BC090938<br>Y14737<br>M87789<br>ENSESTT00000049486<br>ENST00000383048<br>GENSCAN00000029220<br>U63211<br>AY515006<br>AY515002<br>AY515008 | Homo sapiens mRNA for immunoglobulin heavy chain, partial cds, anti-SARS coronavirus spike protein.<br>Homo sapiens IgG1 heavy chain mRNA, partial cds.<br>Homo sapiens anti-hepatitis B surface antigen immunoglobulin heavy chain mRNA, partial cds.<br>Homo sapiens cDNA FLJ25046 fis, clone CBL03624, highly similar to Ig gamma immunoglobulin heavy chain.<br>Homo sapiens cDNA FLJ36487 fis, clone THYMU2017844, highly similar to Homo sapiens mRNA for<br>immunoglobulin lambda heavy chain.<br>Homo sapiens cDNA FLJ39691 fis, clone SMINT2010672, highly similar to Homo sapiens mRNA for<br>immunoglobulin lambda heavy chain.<br>Homo sapiens cDNA FLJ40037 fis, clone SYNOV2000279, highly similar to Human (hybridoma H210)<br>anti-hepatitis A IgG variable region, constant region, complementarity-determining regions mRNA.<br>Homo sapiens cDNA FLJ40045 fis, clone SYNOV2001111, highly similar to Human (hybridoma H210)<br>anti-hepatitis A IgG variable region, constant region, complementarity-determining regions mRNA.<br>Homo sapiens cDNA FLJ41075 fis, clone ADIPS2000088, highly similar to Human (hybridoma H210)<br>anti-hepatitis A IgG.<br>Homo sapiens cDNA FLJ41806 fis, clone NOVAR2001108, highly similar to Human (hybridoma H210)<br>anti-hepatitis A IgG variable region, constant region, complementarity-determining regions mRNA, complete cds.<br>Homo sapiens cDNA FLJ45501 fis, clone BRTHA2019726, highly similar to Ig gamma-1 chain C region.<br>Homo sapiens cDNA FLJ26298 fis, clone DMC07404, highly similar to Ig gamma-1 chain C region.<br>Homo sapiens cDNA FLJ26924 fis, clone RCT05096, highly similar to Ig gamma-1 chain C region.<br>Homo sapiens cDNA FLJ27029 fis, clone SLV07518, highly similar to Ig gamma-1 chain C region.<br>Homo sapiens partial mRNA for immunoglobulin heavy chain (IGHV gene), cell line SP53.<br>Homo sapiens immunoglobulin heavy constant gamma 1-like protein (IGHG1) mRNA, complete cds.<br>Homo sapiens isolate BW1-MT-IgG1 immunoglobulin gamma 1 heavy chain constant region mRNA, partial cds.<br>Homo sapiens anti-RhD monoclonal T125 gamma1 heavy chain precursor, mRNA, complete cds.<br>Homo sapiens cDNA clone MGC:27378 IMAGE:4688865, complete cds.<br>Homo sapiens immunoglobulin heavy constant gamma 1 (G1m marker), mRNA (cDNA clone MGC:31937<br>IMAGE:4851063), complete cds.<br>Homo sapiens immunoglobulin heavy constant gamma 1 (G1m marker), mRNA (cDNA clone MGC:39308<br>IMAGE:5450493), complete cds.<br>Homo sapiens immunoglobulin heavy constant gamma 1 (G1m marker), mRNA (cDNA clone MGC:39303<br>IMAGE:5440392), complete cds.<br>Homo sapiens immunoglobulin heavy constant mu, mRNA (cDNA clone MGC:52291 IMAGE:4765763),<br>complete cds.<br>Homo sapiens anti-rabies SO57 immunoglobulin heavy chain, mRNA (cDNA clone MGC:59926<br>IMAGE:5480266), complete cds.<br>Homo sapiens immunoglobulin heavy constant gamma 1 (G1m marker), mRNA (cDNA clone MGC:71315<br>IMAGE:6300554), complete cds.<br>Homo sapiens cDNA clone MGC:75197 IMAGE:6178926, complete cds.<br>Homo sapiens immunoglobulin heavy constant gamma 1 (G1m marker), mRNA (cDNA clone MGC:71306<br>IMAGE:5451018), complete cds.<br>Homo sapiens immunoglobulin heavy constant gamma 1 (G1m marker), mRNA (cDNA clone MGC:78595<br>IMAGE:6302910), complete cds.<br>Homo sapiens immunoglobulin heavy constant gamma 1 (G1m marker), mRNA (cDNA clone MGC:78608<br>IMAGE:6214622), complete cds.<br>Homo sapiens immunoglobulin heavy constant gamma 1 (G1m marker), mRNA (cDNA clone MGC:88762<br>IMAGE:4575935), complete cds.<br>Homo sapiens immunoglobulin heavy constant gamma 1 (G1m marker), mRNA (cDNA clone MGC:88812<br>IMAGE:6281279), complete cds.<br>Homo sapiens immunoglobulin heavy constant gamma 1 (G1m marker), mRNA (cDNA clone MGC:88797<br>IMAGE:6295788), complete cds.<br>Homo sapiens immunoglobulin heavy constant gamma 1 (G1m marker), mRNA (cDNA clone MGC:99684<br>IMAGE:6302293), complete cds.<br>Homo sapiens immunoglobulin heavy constant gamma 1 (G1m marker), mRNA (cDNA clone MGC:105003<br>IMAGE:3054816), complete cds. |

| | | | | | | | | | |
|---|---|---|---|---|---|---|---|---|---|
| | | | | | | | | | Homo sapiens immunoglobulin heavy constant gamma 1 (G1m marker), mRNA (cDNA clone IMAGE:3056020), partial cds.<br>Homo sapiens immunoglobulin heavy constant gamma 1 (G1m marker), mRNA (cDNA clone MGC:105004 IMAGE:3056327), complete cds.<br>Homo sapiens partial mRNA for anti-peptide/MHC complex HLA-A1/MAGE-A1 monoclonal antibody heavy chain.<br>Human anti-HBsAg immunoglobulin Fd chain mRNA, partial cds.<br>Homo sapiens Ig rearranged gamma-1 chain (IGHG1) mRNA subgroup VH-III hybridoma, partial cds.<br>Homo sapiens mRNA; cDNA DKFZp686F17185 (from clone DKFZp686F17185).<br>Homo sapiens mRNA; cDNA DKFZp686I15196 (from clone DKFZp686I15196).<br>Homo sapiens mRNA; cDNA DKFZp686K03196 (from clone DKFZp686K03196).<br>Homo sapiens mRNA; cDNA DKFZp686O01196 (from clone DKFZp686O01196).<br>Homo sapiens mRNA; cDNA DKFZp686P15220 (from clone DKFZp686P15220).<br>Homo sapiens mRNA; cDNA DKFZp686C11235 (from clone DKFZp686C11235).<br>Homo sapiens anti-rabies SO57 immunoglobulin heavy chain (IGHG1), mRNA.<br>Homo sapiens IgH mRNA for anti-HBs antibody heavy chain, partial cds.<br>Homo sapiens cDNA FLJ35528 fis, clone SPLEN2001881, highly similar to IG GAMMA-1 CHAIN C REGION.<br>Homo sapiens cDNA FLJ40540 fis, clone THYMU1000554, highly similar to Homo sapiens mRNA for immunoglobulin lambda heavy chain.<br>Homo sapiens cDNA FLJ25650 fis, clone SYN01104, highly similar to Ig gamma =immunoglobulin heavy chain.<br>Homo sapiens cDNA FLJ44144 fis, clone THYMU2023711, highly similar to Homo sapiens mRNA for immunoglobulin lambda heavy chain.<br>Homo sapiens cDNA FLJ46441 fis, clone THYMU3016518, highly similar to Ig gamma-1 chain C region.<br>Homo sapiens cDNA FLJ26301 fis, clone DMC07540.<br>Homo sapiens cDNA FLJ27276 fis, clone TMS01151, highly similar to Ig gamma-1 chain C region.<br>Homo sapiens cDNA FLJ27303 fis, clone TMS04901, highly similar to Ig gamma-1 chain C region.<br>Homo sapiens anti-rabies SO57 immunoglobulin heavy chain mRNA, complete cds.<br>Homo sapiens immunoglobulin heavy constant gamma 1 (G1m marker), mRNA (cDNA clone MGC:12848 IMAGE:4308411), complete cds.<br>Homo sapiens cDNA clone MGC:39273 IMAGE:5440834, complete cds.<br>Homo sapiens immunoglobulin heavy constant gamma 1 (G1m marker), mRNA (cDNA clone MGC:45938 IMAGE:5440797), complete cds.<br>Homo sapiens immunoglobulin heavy constant gamma 1 (G1m marker), mRNA (cDNA clone MGC:59934 IMAGE:6278990), complete cds.<br>Homo sapiens immunoglobulin heavy constant gamma 1 (G1m marker), mRNA (cDNA clone MGC:88064 IMAGE:6182140), complete cds.<br>Homo sapiens anti-rabies SO57 immunoglobulin heavy chain, mRNA (cDNA clone MGC:88774 IMAGE:4855124), complete cds.<br>Homo sapiens immunoglobulin heavy constant gamma 1 (G1m marker), mRNA (cDNA clone MGC:88778 IMAGE:6215815), complete cds.<br>Homo sapiens immunoglobulin heavy constant gamma 1 (G1m marker), mRNA (cDNA clone MGC:104997 IMAGE:3057210), complete cds.<br>Homo sapiens mRNA for immunoglobulin lambda heavy chain.<br>Human (hybridoma H210) anti-hepatitis A IgG variable region, constant region, complementarity-determining regions mRNA, complete cds.<br><br>cdna:known chromosome:NCBI36:14:105273726:105796730:-1 gene:ENSG00000130076<br>cdna:Genscan chromosome:NCBI36:14:105275144:105284872:-1<br>Human Ig heavy chain variable region (humha311) mRNA, partial cds.<br>Homo sapiens anti-HIV-1 gp120 immunoglobulin 23e heavy chain mRNA, partial cds.<br>Homo sapiens anti-HIV-1 gp120 immunoglobulin 411g heavy chain mRNA, partial cds.<br>Homo sapiens anti-HIV-1 gp120 immunoglobulin 47e heavy chain mRNA, partial cds. |
| 3581858 | 787305 | 3581637 | core | 105306675 | 105306699 | 14 | - | XM_941366<br>AK097906 | PREDICTED: Homo sapiens similar to Ig gamma-1 chain C region (LOC652050), mRNA.<br>Homo sapiens cDNA FLJ40587 fis, clone THYMU2008928, highly similar to IG GAMMA-2 CHAIN C |

| | | | | | | | | | |
|---|---|---|---|---|---|---|---|---|---|
| | | | | | | | | AK123981<br>AY172958<br>ENSESTT00000049475<br>ENSESTT00000049483<br>GENSCAN00000029205<br>AK090464<br>AK128579<br>ENST00000337025<br>AK126133<br>AK097307<br>AK097355<br>AK098108<br>BC033178<br>BX640724<br>AK097572<br>AK098153<br>AK126358<br>BX538126<br>ENST00000251006<br>ENST00000383047<br>ENST00000361266<br>J00231<br>BC089421<br>ENSESTT00000049480<br>ENSESTT00000049481<br>AY393143<br>AK097354<br>AY392909<br>X56524<br>X56523 | REGION.<br>Homo sapiens cDNA FLJ41987 fis, clone SPLEN2015267, highly similar to Homo sapiens IGHG3 gene for immunoglobulin heavy chain gamma 3 constant region.<br>Homo sapiens anti-rabies SOJB immunoglobulin heavy chain mRNA, complete cds.<br><br><br>cdna:Genscan chromosome:NCBI36:14:105303296:105323213:-1<br>Homo sapiens mRNA for FLJ00385 protein.<br>Homo sapiens cDNA FLJ46738 fis, clone TRACH3020930, highly similar to Ig gamma-1 chain C region.<br>cdna:known chromosome:NCBI36:14:105301886:105310425:-1 gene:ENSG00000130076<br>Homo sapiens cDNA FLJ44145 fis, clone THYMU2027695, moderately similar to Ig gamma-1 chain C region.<br>Homo sapiens cDNA FLJ39988 fis, clone STOMA2000640, highly similar to Homo sapiens IGHG3 gene for immunoglobulin heavy chain gamma 3 constant region.<br>Homo sapiens cDNA FLJ40036 fis, clone SYNOV2000251, highly similar to Homo sapiens IGHG3 gene for immunoglobulin heavy chain gamma 3 constant region.<br>Homo sapiens cDNA FLJ40789 fis, clone TRACH2006918, highly similar to Homo sapiens IGHG3 gene for immunoglobulin heavy chain gamma 3 constant region.<br>Homo sapiens immunoglobulin heavy constant gamma 3 (G3m marker), mRNA (cDNA clone MGC:45809 IMAGE:4566267), complete cds.<br>Homo sapiens mRNA; cDNA DKFZp686I15212 (from clone DKFZp686I15212).<br>Homo sapiens cDNA FLJ40253 fis, clone TESTI2024419, highly similar to Homo sapiens IGHG3 gene for immunoglobulin heavy chain gamma 3 constant region.<br>Homo sapiens cDNA FLJ40834 fis, clone TRACH2012823, highly similar to Homo sapiens IGHG3 gene for immunoglobulin heavy chain gamma 3 constant region.<br>Homo sapiens cDNA FLJ44394 fis, clone TSTOM2000442, moderately similar to Ig gamma-1 chain C region.<br>Homo sapiens mRNA; cDNA DKFZp686H11213 (from clone DKFZp686H11213).<br>cdna:known chromosome:NCBI36:14:105306486:105589977:-1 gene:ENSG00000130076<br>cdna:known chromosome:NCBI36:14:105306486:105589977:-1 gene:ENSG00000130076<br>cdna:known chromosome:NCBI36:14:105306493:105524151:-1 gene:ENSG00000130076<br>Human Ig gamma3 heavy chain disease OMM protein mRNA.<br>Homo sapiens cDNA clone MGC:105008 IMAGE:3055541, complete cds.<br><br><br>Homo sapiens clone RA702-M2-16.fa immunoglobulin heavy chain mRNA, partial cds.<br>Homo sapiens cDNA FLJ40035 fis, clone SYNOV2000173, highly similar to Human (hybridoma H210) anti-hepatitis A IgG variable region, constant region, complementarity-determining regions mRNA.<br>Homo sapiens clone RA702-A3-11.fa immunoglobulin heavy chain mRNA, partial cds.<br>Human mRNA for Ig heavy chain variable region (hybridoma Ray 4).<br>Human mRNA for Ig heavy chain variable region (hybridoma SB5/D6). |
| 3581862 | 787307 | 3581637 | core | 105307332 | 105307356 | 14 | - | XM_941366<br>AK097906<br>AK126133<br>AK097307<br>AK097355<br>AK098108<br>BC033178<br>BX640724<br>AK097572<br>AK098153<br>AK126358<br>BX538126<br>ENST00000251006<br>ENST00000383047<br>AK123981 | PREDICTED: Homo sapiens similar to Ig gamma-1 chain C region (LOC652050), mRNA.<br>Homo sapiens cDNA FLJ40587 fis, clone THYMU2008928, highly similar to IG GAMMA-2 CHAIN C REGION.<br>Homo sapiens cDNA FLJ44145 fis, clone THYMU2027695, moderately similar to Ig gamma-1 chain C region.<br>Homo sapiens cDNA FLJ39988 fis, clone STOMA2000640, highly similar to Homo sapiens IGHG3 gene for immunoglobulin heavy chain gamma 3 constant region.<br>Homo sapiens cDNA FLJ40036 fis, clone SYNOV2000251, highly similar to Homo sapiens IGHG3 gene for immunoglobulin heavy chain gamma 3 constant region.<br>Homo sapiens cDNA FLJ40789 fis, clone TRACH2006918, highly similar to Homo sapiens IGHG3 gene for immunoglobulin heavy chain gamma 3 constant region.<br>Homo sapiens immunoglobulin heavy constant gamma 3 (G3m marker), mRNA (cDNA clone MGC:45809 IMAGE:4566267), complete cds.<br>Homo sapiens mRNA; cDNA DKFZp686I15212 (from clone DKFZp686I15212).<br>Homo sapiens cDNA FLJ40253 fis, clone TESTI2024419, highly similar to Homo sapiens IGHG3 gene for immunoglobulin heavy chain gamma 3 constant region. |

| | | | | | | | | | |
|---|---|---|---|---|---|---|---|---|---|
| | | | | | | | | AY172958<br>BC089421<br>ENSESTT00000049475<br>ENSESTT00000049480<br>ENSESTT00000049481<br>ENST00000361266<br>GENSCAN00000029205<br>AK090464<br>AK128579<br>ENSESTT00000049483<br>ENST00000337025<br>J00231<br>AK097354 | Homo sapiens cDNA FLJ40834 fis, clone TRACH2012823, highly similar to Homo sapiens IGHG3 gene for immunoglobulin heavy chain gamma 3 constant region.<br>Homo sapiens cDNA FLJ44394 fis, clone TSTOM2000442, moderately similar to Ig gamma-1 chain C region.<br>Homo sapiens mRNA; cDNA DKFZp686H11213 (from clone DKFZp686H11213).<br>cdna:known chromosome:NCBI36:14:105306486:105589977:-1 gene:ENSG00000130076<br>cdna:known chromosome:NCBI36:14:105306486:105589977:-1 gene:ENSG00000130076<br>Homo sapiens cDNA FLJ41987 fis, clone SPLEN2015267, highly similar to Homo sapiens IGHG3 gene for immunoglobulin heavy chain gamma 3 constant region.<br>Homo sapiens anti-rabies SOJB immunoglobulin heavy chain mRNA, complete cds.<br>Homo sapiens cDNA clone MGC:105008 IMAGE:3055541, complete cds.<br>cdna:known chromosome:NCBI36:14:105306493:105524151:-1 gene:ENSG00000130076<br>cdna:Genscan chromosome:NCBI36:14:105303296:105323213:-1<br>Homo sapiens mRNA for FLJ00385 protein.<br>Homo sapiens cDNA FLJ46738 fis, clone TRACH3020930, highly similar to Ig gamma-1 chain C region.<br>cdna:known chromosome:NCBI36:14:105301886:105310425:-1 gene:ENSG00000130076<br>Human Ig gamma3 heavy chain disease OMM protein mRNA.<br>Homo sapiens cDNA FLJ40035 fis, clone SYNOV2000173, highly similar to Human (hybridoma H210) anti-hepatitis A IgG variable region, constant region, complementarity-determining regions mRNA. |
| 3581869 | 787309 | 3581637 | core | 105308066 | 105308099 | 14 | - | J00231<br>XM_941366<br>AK097307<br>AK097355<br>AK097572<br>AK097906<br>AK098108<br>AK098153<br>AK123981<br>AK126133<br>AK126358<br>AY172958<br>BC033178<br>BC089421<br>BX538126<br>BX640724<br>ENSESTT00000049475<br>ENSESTT00000049477<br>ENSESTT00000049478<br>ENSESTT00000049480<br>ENSESTT00000049481<br>ENST00000251006<br>ENST00000361266<br>GENSCAN00000029205<br>AK090464<br>AK128579<br>ENST00000337025 | Human Ig gamma3 heavy chain disease OMM protein mRNA.<br>PREDICTED: Homo sapiens similar to Ig gamma-1 chain C region (LOC652050), mRNA.<br>Homo sapiens cDNA FLJ39988 fis, clone STOMA2000640, highly similar to Homo sapiens IGHG3 gene for immunoglobulin heavy chain gamma 3 constant region.<br>Homo sapiens cDNA FLJ40036 fis, clone SYNOV2000251, highly similar to Homo sapiens IGHG3 gene for immunoglobulin heavy chain gamma 3 constant region.<br>Homo sapiens cDNA FLJ40253 fis, clone TESTI2024419, highly similar to Homo sapiens IGHG3 gene for immunoglobulin heavy chain gamma 3 constant region.<br>Homo sapiens cDNA FLJ40587 fis, clone THYMU2008928, highly similar to IG GAMMA-2 CHAIN C REGION.<br>Homo sapiens cDNA FLJ40789 fis, clone TRACH2006918, highly similar to Homo sapiens IGHG3 gene for immunoglobulin heavy chain gamma 3 constant region.<br>Homo sapiens cDNA FLJ40834 fis, clone TRACH2012823, highly similar to Homo sapiens IGHG3 gene for immunoglobulin heavy chain gamma 3 constant region.<br>Homo sapiens cDNA FLJ41987 fis, clone SPLEN2015267, highly similar to Homo sapiens IGHG3 gene for immunoglobulin heavy chain gamma 3 constant region.<br>Homo sapiens cDNA FLJ44145 fis, clone THYMU2027695, moderately similar to Ig gamma-1 chain C region.<br>Homo sapiens cDNA FLJ44394 fis, clone TSTOM2000442, moderately similar to Ig gamma-1 chain C region.<br>Homo sapiens anti-rabies SOJB immunoglobulin heavy chain mRNA, complete cds.<br>Homo sapiens immunoglobulin heavy constant gamma 3 (G3m marker), mRNA (cDNA clone MGC:45809 IMAGE:4566267), complete cds.<br>Homo sapiens cDNA clone MGC:105008 IMAGE:3055541, complete cds.<br>Homo sapiens mRNA; cDNA DKFZp686H11213 (from clone DKFZp686H11213).<br>Homo sapiens mRNA; cDNA DKFZp686I15212 (from clone DKFZp686I15212).<br>cdna:known chromosome:NCBI36:14:105306486:105589977:-1 gene:ENSG00000130076<br>cdna:known chromosome:NCBI36:14:105306493:105524151:-1 gene:ENSG00000130076<br>cdna:Genscan chromosome:NCBI36:14:105303296:105323213:-1 |

| | | | | | | | | | |
|---|---|---|---|---|---|---|---|---|---|
| | | | | | | | | | Homo sapiens mRNA for FLJ00385 protein.<br>Homo sapiens cDNA FLJ46738 fis, clone TRACH3020930, highly similar to Ig gamma-1 chain C region.<br>cdna:known chromosome:NCBI36:14:105301886:105310425:-1 gene:ENSG00000130076 |
| 3589720 | 792102 | 3589697 | core | 38279759 | 38279851 | 15 | + | NM_001211<br>ENST00000287598<br>ENSESTT00000034777<br>ENSESTT00000034778<br>ENSESTT00000034779<br>GENSCAN00000007743<br>ENSESTT00000034774<br>ENSESTT00000034775<br>ENSESTT00000034776<br>ENSESTT00000034780 | Homo sapiens BUB1 budding uninhibited by benzimidazoles 1 homolog beta (yeast) (BUB1B), mRNA.<br>cdna:known-ccds chromosome:NCBI36:15:38240530:38300627:1 gene:ENSG00000156970 CCDS10053.1<br><br><br><br>cdna:Genscan chromosome:NCBI36:15:38225302:38300210:1 |
| 3590094 | 792319 | 3590086 | core | 38798299 | 38798356 | 15 | + | NM_002875<br>NM_133487<br>AK131299<br>ENSESTT00000041064<br>ENST00000267868<br>ENST00000382643<br>ENST00000382642<br>AY425955<br>ENSESTT00000041065 | Homo sapiens RAD51 homolog (RecA homolog, E. coli) (S. cerevisiae) (RAD51), transcript variant 1, mRNA.<br>Homo sapiens RAD51 homolog (RecA homolog, E. coli) (S. cerevisiae) (RAD51), transcript variant 2, mRNA.<br>Homo sapiens cDNA FLJ16262 fis, clone IMR322008651, highly similar to DNA REPAIR PROTEIN RAD51.<br><br>cdna:known-ccds chromosome:NCBI36:15:38774661:38811646:1 gene:ENSG00000051180 CCDS10062.1<br>cdna:known chromosome:NCBI36:15:38774661:38811646:1 gene:ENSG00000051180<br>cdna:known chromosome:NCBI36:15:38774661:38811646:1 gene:ENSG00000051180<br>Homo sapiens Rad51 mRNA, partial cds, alternatively spliced. |
| 3591477 | 793163 | 3591459 | core | 41607937 | 41608262 | 15 | + | NM_002373<br>ENSESTT00000003516<br>ENST00000382031<br>ENST00000300231 | Homo sapiens microtubule-associated protein 1A (MAP1A), mRNA.<br><br>cdna:known chromosome:NCBI36:15:41590448:41611110:1 gene:ENSG00000166963<br>cdna:known chromosome:NCBI36:15:41597133:41611109:1 gene:ENSG00000166963 |
| 3595997 | 795980 | 3595979 | core | 57204410 | 57204478 | 15 | + | NM_004701<br>ENST00000288207<br>ENSESTT00000027806<br>GENSCAN00000065620 | Homo sapiens cyclin B2 (CCNB2), mRNA.<br>cdna:known-ccds chromosome:NCBI36:15:57184612:57204535:1 gene:ENSG00000157456 CCDS10170.1<br><br>cdna:Genscan chromosome:NCBI36:15:57092573:57204368:1 |
| 3597384 | 796825 | 3597338 | core | 61140462 | 61140501 | 15 | + | NM_001018006<br>AK092051<br>NM_000366<br>NM_001018020<br>AF474156<br>AK092573<br>DQ424903<br>M19267<br>ENSESTT00000056493<br>ENST00000288398<br>ENST00000358278<br>ENST00000317516<br>GENSCAN00000013115<br>AK131384<br>X12369 | Homo sapiens tropomyosin 1 (alpha) (TPM1), transcript variant 4, mRNA.<br>Homo sapiens cDNA FLJ34732 fis, clone MESAN2006743, moderately similar to TROPOMYOSIN, FIBROBLAST ISOFORM TM3.<br>Homo sapiens tropomyosin 1 (alpha) (TPM1), transcript variant 5, mRNA.<br>Homo sapiens tropomyosin 1 (alpha) (TPM1), transcript variant 7, mRNA.<br>Homo sapiens TPMsk1 (TPM1) mRNA, partial cds.<br>Homo sapiens cDNA FLJ35254 fis, clone PROST2003775, highly similar to TROPOMYOSIN, FIBROBLAST ISOFORM TM3.<br>Homo sapiens clone FG060306 tropomyosin 1 alpha variant 6 mRNA, complete cds, alternatively spliced.<br>Human tropomyosin mRNA, complete cds.<br><br>cdna:known-ccds chromosome:NCBI36:15:61121891:61145344:1 gene:ENSG00000140416 CCDS10181.1<br>cdna:known chromosome:NCBI36:15:61121891:61151164:1 gene:ENSG00000140416<br>cdna:known chromosome:NCBI36:15:61121891:61150413:1 gene:ENSG00000140416<br>cdna:Genscan chromosome:NCBI36:15:61121422:61143398:1<br>Homo sapiens cDNA FLJ16459 fis, clone BRCAN2002473, moderately similar to Tropomyosin, fibroblast isoform 2.<br>Human mRNA (exon 6-9 part.) for smooth muscle tropomyosin. |
| 3601095 | 799202 | 3601051 | core | 71328468 | 71328495 | 15 | + | ENSESTT00000024371<br>NM_002499<br>AB209412<br>ENSESTT00000024370 | <br>Homo sapiens neogenin homolog 1 (chicken) (NEO1), mRNA.<br>Homo sapiens mRNA for neogenin homolog 1 variant protein. |

| | | | | | | | | | |
|---|---|---|---|---|---|---|---|---|---|
| | | | | | | | | ENST00000261908<br>ENST00000339362<br>ENST00000379842 | cdna:known-ccds chromosome:NCBI36:15:71131928:71382835:1 gene:ENSG00000067141 CCDS10247.1<br>cdna:known chromosome:NCBI36:15:71131928:71382835:1 gene:ENSG00000067141<br>cdna:known chromosome:NCBI36:15:71215379:71384592:1 gene:ENSG00000067141 |
| 3604184 | 801035 | 3604147 | core | 78968876 | 78968988 | 15 | + | ENSESTT00000056273<br>NM_018689<br>ENST00000220244<br>ENST00000356249<br>GENSCAN00000029643 | <br>Homo sapiens KIAA1199 (KIAA1199), mRNA.<br>cdna:known-ccds chromosome:NCBI36:15:78858767:79031054:1 gene:ENSG00000103888 CCDS10315.1<br>cdna:known chromosome:NCBI36:15:78858767:79031054:1 gene:ENSG00000103888<br>cdna:Genscan chromosome:NCBI36:15:78916631:79028320:1 |
| 3604218 | 801064 | 3604147 | core | 79028263 | 79028298 | 15 | + | NM_018689<br>ENSESTT00000056274<br>ENSESTT00000056278<br>GENSCAN00000029643<br>ENST00000220244<br>ENST00000356249<br>ENSESTT00000056272<br>ENSESTT00000056273<br>ENSESTT00000056275<br>ENSESTT00000056276 | Homo sapiens KIAA1199 (KIAA1199), mRNA.<br><br><br>cdna:Genscan chromosome:NCBI36:15:78916631:79028320:1<br>cdna:known-ccds chromosome:NCBI36:15:78858767:79031054:1 gene:ENSG00000103888 CCDS10315.1<br>cdna:known chromosome:NCBI36:15:78858767:79031054:1 gene:ENSG00000103888 |
| 3605495 | 801853 | 3605395 | core | 82497456 | 82497493 | 15 | + | NM_207517<br>ENST00000286744<br>ENST00000286745<br>GENSCAN00000025268<br>ENSESTT00000002568<br>GENSCAN00000025272<br>ENSESTT00000002569<br>GENSCAN00000025265<br>ENSESTT00000002570<br>GENSCAN00000035975 | Homo sapiens ADAMTS-like 3 (ADAMTSL3), mRNA.<br>cdna:known-ccds chromosome:NCBI36:15:82113842:82499595:1 gene:ENSG00000156218 CCDS10326.1<br>cdna:known chromosome:NCBI36:15:82113842:82321329:1 gene:ENSG00000156218<br>cdna:Genscan chromosome:NCBI36:15:82199704:82279905:1<br><br>cdna:Genscan chromosome:NCBI36:15:82297783:82346678:1<br><br>cdna:Genscan chromosome:NCBI36:15:82352759:82373928:1<br><br>cdna:Genscan chromosome:NCBI36:15:82442043:82491192:1 |
| 3607351 | 802985 | 3607332 | core | 87180467 | 87180511 | 15 | + | NM_013227<br>NM_001135<br>XM_938439<br>BC036445<br>ENST00000268134<br>ENST00000338824<br>ENST00000352105<br>ENSESTT00000024257<br>ENSESTT00000024258<br>ENSESTT00000024259 | Homo sapiens aggrecan 1 (chondroitin sulfate proteoglycan 1, large aggregating proteoglycan, antigen identified by monoclonal antibody A0122) (AGC1), transcript variant 2, mRNA.<br>Homo sapiens aggrecan 1 (chondroitin sulfate proteoglycan 1, large aggregating proteoglycan, antigen identified by monoclonal antibody A0122) (AGC1), transcript variant 1, mRNA.<br>PREDICTED: Homo sapiens similar to aggrecan 1 isoform 2 precursor (LOC649366), mRNA.<br>Homo sapiens aggrecan 1 (chondroitin sulfate proteoglycan 1, large aggregating proteoglycan, antigen identified by monoclonal antibody A0122), mRNA (cDNA clone IMAGE:4823544), complete cds.<br>cdna:known chromosome:NCBI36:15:87147709:87218716:1 gene:ENSG00000157766<br>cdna:known chromosome:NCBI36:15:87147709:87218716:1 gene:ENSG00000157766<br>cdna:known chromosome:NCBI36:15:87147709:87218716:1 gene:ENSG00000157766 |
| 3607589 | 803133 | 3607537 | core | 87660721 | 87661108 | 15 | + | NM_018193<br>AB058697<br>ENST00000310775<br>ENST00000300027<br>BC004277<br>ENSESTT00000019518<br>ENSESTT00000019519<br>ENSESTT00000019520<br>ENSESTT00000019524<br>ENSESTT00000019515<br>ENSESTT00000019516<br>ENSESTT00000019517 | Homo sapiens KIAA1794 (KIAA1794), mRNA.<br>Homo sapiens mRNA for KIAA1794 protein, partial cds.<br>cdna:known-ccds chromosome:NCBI36:15:87588198:87661366:1 gene:ENSG00000140525 CCDS10349.1<br>cdna:known chromosome:NCBI36:15:87588198:87661366:1 gene:ENSG00000140525<br>Homo sapiens KIAA1794, mRNA (cDNA clone IMAGE:3615489), complete cds. |

| | | | | | | | | | |
|---|---|---|---|---|---|---|---|---|---|
| | | | | | | | | ENSESTT00000019521<br>ENSESTT00000019522<br>ENSESTT00000019523<br>ENSESTT00000019525<br>GENSCAN00000000357<br>ENSESTT00000019526<br>ENSESTT00000019527<br>ENSESTT00000019528<br>ENSESTT00000019529<br>ENST00000345047<br>ENSESTT00000019531<br>ENSESTT00000019532 | <br><br><br><br>cdna:Genscan chromosome:NCBI36:15:87591883:87659666:1<br><br><br><br><br>cdna:known chromosome:NCBI36:15:87622915:87661363:1 gene:ENSG00000140525 |
| 3610996 | 805298 | 3610982 | core | 97462813 | 97462885 | 15 | + | NM_015286<br>ENST00000328642<br>GENSCAN00000002866<br>NM_145728<br>ENST00000336292<br>AL833262<br>ENSESTT00000032557 | Homo sapiens desmuslin (DMN), transcript variant B, mRNA.<br>cdna:known chromosome:NCBI36:15:97462771:97490786:1 gene:ENSG00000182253<br>cdna:Genscan chromosome:NCBI36:15:97424857:97490786:1<br>Homo sapiens desmuslin (DMN), transcript variant A, mRNA.<br>cdna:known chromosome:NCBI36:15:97462809:97490786:1 gene:ENSG00000182253<br>Homo sapiens mRNA; cDNA DKFZp451C2210 (from clone DKFZp451C2210). |
| 3618775 | 810061 | 3618736 | core | 36639326 | 36639466 | 15 | - | NM_005739<br>AY634315<br>ENSESTT00000021649<br>ENSESTT00000021651<br>ENST00000310803<br>GENSCAN00000025583<br>BC067298<br>ENSESTT00000021650<br>ENSESTT00000021646<br>ENSESTT00000021647<br>ENSESTT00000021648<br>ENSESTT00000021644<br>ENSESTT00000021645<br>GENSCAN00000004246 | Homo sapiens RAS guanyl releasing protein 1 (calcium and DAG-regulated) (RASGRP1), mRNA.<br>Homo sapiens RAS guanyl releasing protein 1 splice variant D mRNA, complete cds, alternatively spliced.<br><br><br>cdna:known chromosome:NCBI36:15:36567590:36644224:-1 gene:ENSG00000172575<br>cdna:Genscan chromosome:NCBI36:15:36570047:36598923:-1<br>Homo sapiens RAS guanyl releasing protein 1 (calcium and DAG-regulated), mRNA (cDNA clone IMAGE:30377671), partial cds.<br><br><br><br><br><br><br>cdna:Genscan chromosome:NCBI36:15:36636650:36743555:-1 |
| 3630757 | 817644 | 3630736 | core | 66390707 | 66390788 | 15 | - | ENSESTT00000019388<br>ENST00000315757<br>NM_012211<br>NM_001004439<br>AL359064<br>ENSESTT00000019389<br>GENSCAN00000028557 | <br>cdna:known chromosome:NCBI36:15:66381469:66511531:-1 gene:ENSG00000137809<br>Homo sapiens integrin, alpha 11 (ITGA11), transcript variant 2, mRNA.<br>Homo sapiens integrin, alpha 11 (ITGA11), transcript variant 1, mRNA.<br>Homo sapiens mRNA full length insert cDNA clone EUROIMAGE 322987.<br><br>cdna:Genscan chromosome:NCBI36:15:66381546:66487450:-1 |
| 3630758 | 817645 | 3630736 | core | 66392173 | 66392251 | 15 | - | NM_012211<br>NM_001004439<br>AL359064<br>ENSESTT00000019389<br>ENST00000315757<br>GENSCAN00000028557<br>ENSESTT00000019388<br>ENSESTT00000019387<br>ENSESTT00000019386<br>ENSESTT00000019384<br>ENSESTT00000019385 | Homo sapiens integrin, alpha 11 (ITGA11), transcript variant 2, mRNA.<br>Homo sapiens integrin, alpha 11 (ITGA11), transcript variant 1, mRNA.<br>Homo sapiens mRNA full length insert cDNA clone EUROIMAGE 322987.<br><br>cdna:known chromosome:NCBI36:15:66381469:66511531:-1 gene:ENSG00000137809<br>cdna:Genscan chromosome:NCBI36:15:66381546:66487450:-1 |
| 3632816 | 818898 | 3632806 | core | 72268562 | 72268655 | 15 | - | NM_022369 | Homo sapiens stimulated by retinoic acid gene 6 homolog (mouse) (STRA6), mRNA. |

| | | | | | | | | | |
|---|---|---|---|---|---|---|---|---|---|
| | | | | | | | | GENSCAN00000056778<br>AK056125<br>AK091152<br>AK092227<br>BX537413<br>ENST00000323940<br>ENSESTT00000022353<br>ENSESTT00000022354<br>BC015881<br>ENSESTT00000022349<br>ENSESTT00000022350<br>ENSESTT00000022351<br>ENSESTT00000022352 | cdna:Genscan chromosome:NCBI36:15:72259474:72281661:-1<br>Homo sapiens cDNA FLJ31563 fis, clone NT2RI2001449, moderately similar to Mus musculus retinoic acid-responsive protein (Stra6) mRNA.<br>Homo sapiens cDNA FLJ33833 fis, clone CTONG2004126, moderately similar to Mus musculus retinoic acid-responsive protein (Stra6) mRNA.<br>Homo sapiens cDNA FLJ34908 fis, clone NT2RI2006294, moderately similar to Mus musculus retinoic acid-responsive protein (Stra6) mRNA.<br>Homo sapiens mRNA; cDNA DKFZp686P1959 (from clone DKFZp686P1959); complete cds.<br>cdna:known-ccds chromosome:NCBI36:15:72258863:72288419:-1 gene:ENSG00000137868 CCDS10261.1<br>Homo sapiens stimulated by retinoic acid gene 6 homolog (mouse), mRNA (cDNA clone IMAGE:3530901), complete cds. |
| 3632839 | 818912 | 3632806 | core | 72281826 | 72281901 | 15 | - | BX537413<br>ENSESTT00000022352 | Homo sapiens mRNA; cDNA DKFZp686P1959 (from clone DKFZp686P1959); complete cds. |
| 3638338 | 822280 | 3638337 | core | 87660586 | 87660976 | 15 | - | NM_002693<br>ENST00000268124<br>GENSCAN00000000360 | Homo sapiens polymerase (DNA directed), gamma (POLG), mRNA.<br>cdna:known-ccds chromosome:NCBI36:15:87660577:87679030:-1 gene:ENSG00000140521 CCDS10350.1<br>cdna:Genscan chromosome:NCBI36:15:87660986:87677989:-1 |
| 3642784 | 825065 | 3642765 | core | 277124 | 277199 | 16 | + | NM_006849<br>ENSESTT00000033299<br>AB127078<br>ENSESTT00000033296<br>ENST00000219406<br>GENSCAN00000061774<br>Z84717<br>ENSESTT00000033297<br>ENSESTT00000033298 | Homo sapiens protein disulfide isomerase family A, member 2 (PDIA2), mRNA.<br>Homo sapiens ARHGDIG, PDIP polycistronic mRNA for Rho GDP dissociation inhibitor gamma, pancreatic protein disulfide isomerase, complete cds.<br>cdna:known chromosome:NCBI36:16:273171:277209:1 gene:ENSG00000185615<br>cdna:Genscan chromosome:NCBI36:16:273171:277114:1<br>Human DNA sequence from cDNA 16pHQG;19 from chromosome 16p13.3. |
| 3643592 | 825508 | 3643580 | core | 1143864 | 1143894 | 16 | + | NM_001005407<br>ENST00000358590<br>GENSCAN00000065756<br>NM_021098<br>ENST00000356546<br>ENSESTT00000030137<br>ENSESTT00000030138<br>GENSCAN00000065769<br>ENSESTT00000030140<br>ENSESTT00000030141 | Homo sapiens calcium channel, voltage-dependent, alpha 1H subunit (CACNA1H), transcript variant 2, mRNA.<br>cdna:known chromosome:NCBI36:16:1143739:1211772:1 gene:ENSG00000196557<br>cdna:Genscan chromosome:NCBI36:16:1135479:1146660:1<br>Homo sapiens calcium channel, voltage-dependent, alpha 1H subunit (CACNA1H), transcript variant 1, mRNA.<br>cdna:known chromosome:NCBI36:16:1143739:1211772:1 gene:ENSG00000196557<br>cdna:Genscan chromosome:NCBI36:16:1183205:1210995:1 |
| 3643594 | 825508 | 3643580 | core | 1143989 | 1144014 | 16 | + | NM_001005407<br>ENST00000358590<br>NM_021098<br>ENST00000356546<br>ENSESTT00000030137<br>ENSESTT00000030138<br>GENSCAN00000065769<br>ENSESTT00000030140<br>ENSESTT00000030141 | Homo sapiens calcium channel, voltage-dependent, alpha 1H subunit (CACNA1H), transcript variant 2, mRNA.<br>cdna:known chromosome:NCBI36:16:1143739:1211772:1 gene:ENSG00000196557<br>Homo sapiens calcium channel, voltage-dependent, alpha 1H subunit (CACNA1H), transcript variant 1, mRNA.<br>cdna:known chromosome:NCBI36:16:1143739:1211772:1 gene:ENSG00000196557<br>cdna:Genscan chromosome:NCBI36:16:1183205:1210995:1 |
| 3643783 | 825629 | 3643752 | core | 1335271 | 1335373 | 16 | + | NM_003933 | Homo sapiens BAI1-associated protein 3 (BAIAP3), mRNA. |

| | | | | | | | | | |
|---|---|---|---|---|---|---|---|---|---|
| | | | | | | | | BC112129<br>ENSESTT00000030147<br>ENSESTT00000030148<br>ENST00000324385<br>GENSCAN00000006963<br>ENSESTT00000030149<br>ENSESTT00000030150<br>ENSESTT00000030151<br>ENSESTT00000030152<br>ENSESTT00000030153 | Homo sapiens BAI1-associated protein 3, mRNA (cDNA clone MGC:138334 IMAGE:8327597), complete cds.<br><br><br>cdna:known-ccds chromosome:NCBI36:16:1324654:1339440:1 gene:ENSG00000007516 CCDS10434.1<br>cdna:Genscan chromosome:NCBI36:16:1322440:1338484:1 |
| 3644590 | 826084 | 3644541 | core | 2168075 | 2168112 | 16 | + | NM_206835<br>NM_032271<br>ENST00000326181<br>ENST00000357892<br>ENSESTT00000027991<br>ENSESTT00000027993<br>GENSCAN00000008762<br>ENSESTT00000027995<br>ENSESTT00000027996 | Homo sapiens TNF receptor-associated factor 7 (TRAF7), transcript variant 2, mRNA.<br>Homo sapiens TNF receptor-associated factor 7 (TRAF7), transcript variant 1, mRNA.<br>cdna:known-ccds chromosome:NCBI36:16:2145800:2168130:1 gene:ENSG00000131653 CCDS10461.1<br>cdna:novel chromosome:NCBI36:16:2145800:2168130:1 gene:ENSG00000131653<br><br><br>cdna:Genscan chromosome:NCBI36:16:2138650:2167960:1 |
| 3645791 | 826713 | 3645779 | core | 3278501 | 3278532 | 16 | + | NM_005741<br>ENST00000219069<br>GENSCAN00000015448<br>CR602060 | Homo sapiens zinc finger protein 263 (ZNF263), mRNA.<br>cdna:known-ccds chromosome:NCBI36:16:3273488:3281456:1 gene:ENSG00000006194 CCDS10499.1<br>cdna:Genscan chromosome:NCBI36:16:3273820:3286516:1<br>full-length cDNA clone CS0DK009YN15 of HeLa cells Cot 25-normalized of Homo sapiens (human). |
| 3646194 | 826946 | 3646164 | core | 4446732 | 4446767 | 16 | + | NM_005147<br>ENSESTT00000003095<br>ENST00000262375<br>GENSCAN00000045710<br>ENSESTT00000003090<br>ENSESTT00000003091<br>ENSESTT00000003092<br>ENST00000355296<br>ENSESTT00000003093<br>AK127660<br>ENSESTT00000003094 | Homo sapiens DnaJ (Hsp40) homolog, subfamily A, member 3 (DNAJA3), mRNA.<br><br>cdna:known-ccds chromosome:NCBI36:16:4415883:4446774:1 gene:ENSG00000103423 CCDS10515.1<br>cdna:Genscan chromosome:NCBI36:16:4415884:4445742:1<br><br><br><br>cdna:known chromosome:NCBI36:16:4415854:4446774:1 gene:ENSG00000103423<br><br>Homo sapiens cDNA FLJ45758 fis, clone MESAN2016304. |
| 3651532 | 830323 | 3651509 | core | 20743290 | 20743331 | 16 | + | ENST00000381320<br>ENST00000319757<br>GENSCAN00000027818<br>ENST00000348433<br>NM_030941<br>BC068503<br>ENSESTT00000031557<br>ENST00000261377 | cdna:known chromosome:NCBI36:16:20725322:20768485:1 gene:ENSG00000005189<br>cdna:known chromosome:NCBI36:16:20725322:20768485:1 gene:ENSG00000005189<br>cdna:Genscan chromosome:NCBI36:16:20719102:20770779:1<br>cdna:known chromosome:NCBI36:16:20725322:20768485:1 gene:ENSG00000005189<br>Homo sapiens exonuclease NEF-sp (LOC81691), mRNA.<br>Homo sapiens exonuclease NEF-sp, mRNA (cDNA clone MGC:87245 IMAGE:5298867), complete cds.<br><br>cdna:known-ccds chromosome:NCBI36:16:20725322:20768485:1 gene:ENSG00000005189 CCDS10591.1 |
| 3653680 | 831562 | 3653677 | core | 25134563 | 25134594 | 16 | + | BC040630 | Homo sapiens aquaporin 8, mRNA (cDNA clone MGC:50607 IMAGE:5759105), complete cds. |
| 3653691 | 831569 | 3653677 | core | 25147694 | 25147746 | 16 | + | NM_001169<br>BC040630<br>ENSESTT00000042207<br>ENST00000219660 | Homo sapiens aquaporin 8 (AQP8), mRNA.<br>Homo sapiens aquaporin 8, mRNA (cDNA clone MGC:50607 IMAGE:5759105), complete cds.<br><br>cdna:known-ccds chromosome:NCBI36:16:25135784:25147752:1 gene:ENSG00000103375 CCDS10626.1 |
| 3654628 | 832160 | 3654614 | core | 28527931 | 28528025 | 16 | - | NM_177534<br>NM_177530 | Homo sapiens sulfotransferase family, cytosolic, 1A, phenol-preferring, member 1 (SULT1A1), transcript variant 4, mRNA.<br>Homo sapiens sulfotransferase family, cytosolic, 1A, phenol-preferring, member 1 (SULT1A1), transcript variant 3, mRNA. |
| 3662813 | 837010 | 3662808 | core | 56220058 | 56220181 | 16 | + | NM_201525 | Homo sapiens G protein-coupled receptor 56 (GPR56), transcript variant 3, mRNA. |

| | | | | | | | | | |
|---|---|---|---|---|---|---|---|---|---|
| | | | | | | | | NM_201524<br>ENSESTT00000006101<br>ENSESTT00000006102<br>NM_005682<br>BC013207<br>ENSESTT00000006100<br>ENST00000388815<br>ENST00000388814<br>ENST00000388813<br>ENST00000388812<br>ENST00000379696<br>CR936747<br>GENSCAN00000035943<br>AK131550<br>ENSESTT00000006105<br>ENST00000379694<br>ENSESTT00000006106 | Homo sapiens G protein-coupled receptor 56 (GPR56), transcript variant 2, mRNA.<br><br><br>Homo sapiens G protein-coupled receptor 56 (GPR56), transcript variant 1, mRNA.<br>Homo sapiens G protein-coupled receptor 56, mRNA (cDNA clone IMAGE:4157209).<br><br>cdna:known chromosome:NCBI36:16:56241701:56254995:1 gene:ENSG00000205336<br>cdna:known chromosome:NCBI36:16:56241701:56254995:1 gene:ENSG00000205336<br>cdna:known chromosome:NCBI36:16:56241701:56254995:1 gene:ENSG00000205336<br>cdna:known chromosome:NCBI36:16:56241701:56254995:1 gene:ENSG00000205336<br>cdna:novel chromosome:NCBI36:16:56241701:56256445:1 gene:ENSG00000205336<br>Homo sapiens mRNA; cDNA DKFZp781L1398 (from clone DKFZp781L1398).<br>cdna:Genscan chromosome:NCBI36:16:56241701:56254995:1<br>Homo sapiens cDNA FLJ16789 fis, clone PROST2010326, highly similar to Homo sapiens G protein-coupled receptor 56 (GPR56).<br><br>cdna:known chromosome:NCBI36:16:56241701:56256445:1 gene:ENSG00000205336 |
| 3673695 | 843640 | 3673684 | core | 87399364 | 87399515 | 16 | + | NM_030928<br>ENST00000301019<br>ENSESTT00000009797<br>ENSESTT00000009798<br>GENSCAN00000045700<br>ENSESTT00000009799 | Homo sapiens chromatin licensing and DNA replication factor 1 (CDT1), mRNA.<br>cdna:known chromosome:NCBI36:16:87397687:87403166:1 gene:ENSG00000167513<br><br><br>cdna:Genscan chromosome:NCBI36:16:87397741:87402187:1 |
| 3674216 | 843986 | 3674199 | core | 88179596 | 88179659 | 16 | + | NM_014427<br>ENSESTT00000047135<br>NM_153636<br>ENST00000268720<br>ENST00000319518<br>GENSCAN00000003788<br>GENSCAN00000003787 | Homo sapiens copine VII (CPNE7), transcript variant 2, mRNA.<br><br>Homo sapiens copine VII (CPNE7), transcript variant 1, mRNA.<br>cdna:known-ccds chromosome:NCBI36:16:88169677:88191155:1 gene:ENSG00000178773 CCDS10980.1<br>cdna:known-ccds chromosome:NCBI36:16:88169677:88191155:1 gene:ENSG00000178773 CCDS10981.1<br>cdna:Genscan chromosome:NCBI36:16:88166838:88173838:1<br>cdna:Genscan chromosome:NCBI36:16:88177154:88179462:1 |
| 3674229 | 843995 | 3674199 | core | 88185050 | 88185165 | 16 | + | NM_014427<br>NM_153636<br>ENST00000268720<br>ENST00000319518<br>GENSCAN00000003788<br>GENSCAN00000003787<br>ENSESTT00000047135 | Homo sapiens copine VII (CPNE7), transcript variant 2, mRNA.<br>Homo sapiens copine VII (CPNE7), transcript variant 1, mRNA.<br>cdna:known-ccds chromosome:NCBI36:16:88169677:88191155:1 gene:ENSG00000178773 CCDS10980.1<br>cdna:known-ccds chromosome:NCBI36:16:88169677:88191155:1 gene:ENSG00000178773 CCDS10981.1<br>cdna:Genscan chromosome:NCBI36:16:88166838:88173838:1<br>cdna:Genscan chromosome:NCBI36:16:88177154:88179462:1 |
| 3676133 | 845091 | 3676127 | core | 1762863 | 1763054 | 16 | - | NM_023936<br>CR623189<br>ENST00000177742<br>GENSCAN00000031550 | Homo sapiens mitochondrial ribosomal protein S34 (MRPS34), nuclear gene encoding mitochondrial protein, mRNA.<br>full-length cDNA clone CS0DD006YK13 of Neuroblastoma Cot 50-normalized of Homo sapiens (human).<br>cdna:known-ccds chromosome:NCBI36:16:1762223:1763152:-1 gene:ENSG00000074071 CCDS10444.1<br>cdna:Genscan chromosome:NCBI36:16:1762223:1763121:-1 |
| 3685350 | 850517 | 3685329 | core | 23554747 | 23554883 | 16 | - | NM_024675<br>ENST00000261584<br>ENSESTT00000039401<br>ENST00000388994<br>ENST00000388993<br>ENSESTT00000039402<br>GENSCAN00000033635 | Homo sapiens hypothetical protein FLJ21816 (FLJ21816), mRNA.<br>cdna:known chromosome:NCBI36:16:23521984:23560128:-1 gene:ENSG00000083093<br><br>cdna:known chromosome:NCBI36:16:23522281:23559979:-1 gene:ENSG00000083093<br>cdna:known chromosome:NCBI36:16:23522281:23559979:-1 gene:ENSG00000083093<br><br>cdna:Genscan chromosome:NCBI36:16:23501106:23559127:-1 |
| 3694667 | 856161 | 3694657 | core | 63540018 | 63540147 | 16 | - | ENST00000268602 | cdna:known chromosome:NCBI36:16:63538186:63713431:-1 gene:ENSG00000140937 |
| 3697103 | 857568 | 3697090 | core | 68989938 | 68989996 | 16 | - | NM_006927 | Homo sapiens ST3 beta-galactoside alpha-2,3-sialyltransferase 2 (ST3GAL2), mRNA. |

| | | | | | | | | | |
|---|---|---|---|---|---|---|---|---|---|
| | | | | | | | | AK127322<br>ENST00000342907<br>ENSESTT00000024552<br>GENSCAN00000069038<br>ENSESTT00000024551 | Homo sapiens cDNA FLJ45393 fis, clone BRHIP3027105, highly similar to Homo sapiens sialyltransferase 4B (beta-galactoside alpha-2,3-sialyltransferase) (SIAT4B).<br>cdna:known-ccds chromosome:NCBI36:16:68972810:69030492:-1 gene:ENSG00000157350 CCDS10890.1<br>cdna:Genscan chromosome:NCBI36:16:68973093:68989934:-1 |
| 3703931 | 861828 | 3703885 | core | 86454697 | 86454736 | 16 | - | | |
| 3704449 | 862121 | 3704376 | core | 87329055 | 87329136 | 16 | - | NM_014745<br>ENST00000327397<br>BC008073<br>ENST00000388846<br>ENST00000388844<br>ENST00000301015<br>GENSCAN00000014783<br>ENSESTT00000009827 | Homo sapiens family with sequence similarity 38, member A (FAM38A), mRNA.<br>cdna:known chromosome:NCBI36:16:87309256:87311520:-1 gene:ENSG00000103335<br>Homo sapiens family with sequence similarity 38, member A, mRNA (cDNA clone IMAGE:3029644), complete cds.<br>cdna:known chromosome:NCBI36:16:87309514:87330158:-1 gene:ENSG00000103335<br>cdna:known chromosome:NCBI36:16:87309514:87330158:-1 gene:ENSG00000103335<br>cdna:novel chromosome:NCBI36:16:87309267:87330158:-1 gene:ENSG00000103335<br>cdna:Genscan chromosome:NCBI36:16:87309514:87372559:-1 |
| 3705153 | 862546 | 3705151 | core | 88599379 | 88599972 | 16 | - | NM_024043<br>AK090696<br>AK096507<br>CR594535<br>ENST00000304733<br>ENST00000002501<br>ENSESTT00000047198<br>GENSCAN00000064753 | Homo sapiens dysbindin (dystrobrevin binding protein 1) domain containing 1 (DBNDD1), mRNA.<br>Homo sapiens cDNA FLJ33377 fis, clone BRACE2006258.<br>Homo sapiens cDNA FLJ39188 fis, clone OCBBF2004567.<br>full-length cDNA clone CS0DD007YI18 of Neuroblastoma Cot 50-normalized of Homo sapiens (human).<br>cdna:known-ccds chromosome:NCBI36:16:88598782:88604030:-1 gene:ENSG00000003249 CCDS10991.1<br>cdna:known chromosome:NCBI36:16:88598804:88613382:-1 gene:ENSG00000003249<br>cdna:Genscan chromosome:NCBI36:16:88600244:88613356:-1 |
| 3707281 | 863733 | 3707258 | core | 4731195 | 4731219 | 17 | + | ENSESTT00000058590<br>NM_015716<br>NM_170663<br>NM_153827<br>NM_001024937<br>AB209450<br>AL157418<br>ENSESTT00000058591<br>ENSESTT00000058592<br>ENSESTT00000058593<br>ENSESTT00000058594<br>ENSESTT00000058595<br>ENST00000347992<br>ENST00000355280<br>GENSCAN00000015310 | Homo sapiens misshapen-like kinase 1 (zebrafish) (MINK1), transcript variant 1, mRNA.<br>Homo sapiens misshapen-like kinase 1 (zebrafish) (MINK1), transcript variant 2, mRNA.<br>Homo sapiens misshapen-like kinase 1 (zebrafish) (MINK1), transcript variant 3, mRNA.<br>Homo sapiens misshapen-like kinase 1 (zebrafish) (MINK1), transcript variant 4, mRNA.<br>Homo sapiens mRNA for misshapen/NIK-related kinase isoform 2 variant protein.<br>Homo sapiens mRNA; cDNA DKFZp761K18121 (from clone DKFZp761K18121).<br>cdna:known chromosome:NCBI36:17:4728437:4742128:1 gene:ENSG00000141503<br>cdna:known chromosome:NCBI36:17:4728437:4741358:1 gene:ENSG00000141503<br>cdna:Genscan chromosome:NCBI36:17:4728447:4741358:1 |
| 3708405 | 864390 | 3708399 | core | 7127608 | 7127676 | 17 | + | NM_001042<br>CR603611<br>BC034387<br>ENSESTT00000004661<br>ENSESTT00000004662<br>ENST00000380786<br>ENST00000317370<br>GENSCAN00000038238 | Homo sapiens solute carrier family 2 (facilitated glucose transporter), member 4 (SLC2A4), mRNA.<br>full-length cDNA clone CS0DB001YC20 of Neuroblastoma Cot 10-normalized of Homo sapiens (human).<br>Homo sapiens solute carrier family 2 (facilitated glucose transporter), member 4, mRNA (cDNA clone IMAGE:5187454), containing frame-shift errors.<br>cdna:known-ccds chromosome:NCBI36:17:7125777:7132300:1 gene:ENSG00000181856 CCDS11097.1<br>cdna:known chromosome:NCBI36:17:7125778:7131872:1 gene:ENSG00000181856<br>cdna:Genscan chromosome:NCBI36:17:7126023:7130672:1 |
| 3710825 | 865856 | 3710823 | core | 12510240 | 12510284 | 17 | + | NM_153604<br>AK128608<br>ENST00000343344<br>ENST00000356106 | Homo sapiens myocardin (MYOCD), mRNA.<br>Homo sapiens cDNA FLJ46767 fis, clone TRACH3024823.<br>cdna:known-ccds chromosome:NCBI36:17:12509939:12607724:1 gene:ENSG00000141052 CCDS11163.1<br>cdna:known chromosome:NCBI36:17:12509939:12607724:1 gene:ENSG00000141052 |

| | | | | | | | | | |
|---|---|---|---|---|---|---|---|---|---|
| | | | | | | | | GENSCAN00000003376<br>AK097821<br>BX640673<br>ENSESTT00000010169 | cdna:Genscan chromosome:NCBI36:17:12538154:12607686:1<br>Homo sapiens cDNA FLJ40502 fis, clone TESTI2045199.<br>Homo sapiens mRNA; cDNA DKFZp686O15128 (from clone DKFZp686O15128). |
| 3712740 | 867062 | 3712675 | core | 17653429 | 17653459 | 17 | + | NM_030665<br>ENST00000353383<br>ENSESTT00000030588<br>GENSCAN00000006007 | Homo sapiens retinoic acid induced 1 (RAI1), mRNA.<br>cdna:known-ccds chromosome:NCBI36:17:17525512:17655492:1 gene:ENSG00000108557 CCDS11188.1<br><br>cdna:Genscan chromosome:NCBI36:17:17636988:17654520:1 |
| 3716126 | 869097 | 3716113 | core | 24920463 | 24920491 | 17 | + | NM_138349<br>ENSESTT00000002787<br>ENSESTT00000002788<br>ENST00000301057<br>ENSESTT00000002789<br>GENSCAN00000055600 | Homo sapiens tumor protein p53 inducible protein 13 (TP53I13), mRNA.<br><br><br>cdna:known chromosome:NCBI36:17:24919788:24924301:1 gene:ENSG00000167543<br><br>cdna:Genscan chromosome:NCBI36:17:24919903:24924068:1 |
| 3719158 | 870903 | 3719150 | core | 31968579 | 31969087 | 17 | + | NM_178517<br>ENST00000328396 | Homo sapiens phosphatidylinositol glycan anchor biosynthesis, class W (PIGW), mRNA.<br>cdna:known-ccds chromosome:NCBI36:17:31965516:31969263:1 gene:ENSG00000184886 CCDS11313.1 |
| 3724236 | 873795 | 3724197 | core | 42125581 | 42125610 | 17 | + | NM_006178<br>ENSESTT00000019282<br>ENST00000225282<br>ENSESTT00000019288<br>XM_941172<br>ENSESTT00000019283<br>ENSESTT00000019290<br>ENSESTT00000019291<br>GENSCAN00000009366 | Homo sapiens N-ethylmaleimide-sensitive factor (NSF), mRNA.<br><br>cdna:known chromosome:NCBI36:17:42056760:42189993:1 gene:ENSG00000073969<br><br>PREDICTED: Homo sapiens similar to Vesicle-fusing ATPase (Vesicular-fusion protein NSF) (N-ethylmaleimide sensitive fusion protein) (NEM-sensitive fusion protein) (SKD2 protein) (LOC651907), mRNA.<br><br><br><br>cdna:Genscan chromosome:NCBI36:17:42158049:42186368:1 |
| 3726377 | 875107 | 3726375 | core | 45807620 | 45807824 | 17 | + | NM_152463<br>ENSESTT00000004031<br>GENSCAN00000005263<br>ENST00000338165<br>ENSESTT00000004032<br>AK021607 | Homo sapiens essential meiotic endonuclease 1 homolog 1 (S. pombe) (EME1), mRNA.<br><br>cdna:Genscan chromosome:NCBI36:17:45807569:45813299:1<br>cdna:known-ccds chromosome:NCBI36:17:45805589:45813817:1 gene:ENSG00000154920 CCDS11565.1<br><br>Homo sapiens cDNA FLJ11545 fis, clone HEMBA1002833. |
| 3728778 | 876615 | 3728776 | core | 54125006 | 54125030 | 17 | + | NM_058216<br>BC073161<br>ENSESTT00000033921<br>ENSESTT00000033922<br>ENSESTT00000033923<br>ENST00000308321<br>NM_002876<br>ENST00000337432<br>CR623930<br>GENSCAN00000067746<br>ENSESTT00000033924 | Homo sapiens RAD51 homolog C (S. cerevisiae) (RAD51C), transcript variant 1, mRNA.<br>Homo sapiens cDNA clone IMAGE:6156549, containing frame-shift errors.<br><br><br><br>cdna:known-ccds chromosome:NCBI36:17:54125004:54166689:1 gene:ENSG00000108384 CCDS11612.1<br>Homo sapiens RAD51 homolog C (S. cerevisiae) (RAD51C), transcript variant 2, mRNA.<br>cdna:known-ccds chromosome:NCBI36:17:54124962:54166689:1 gene:ENSG00000108384 CCDS11611.1<br>full-length cDNA clone CS0DN003YG15 of Adult brain of Homo sapiens (human).<br>cdna:Genscan chromosome:NCBI36:17:54125004:54142350:1 |
| 3728909 | 876707 | 3728889 | core | 55169596 | 55169628 | 17 | + | NM_030938<br>ENST00000262291<br>ENSESTT00000016238<br>ENSESTT00000016239<br>ENSESTT00000016240<br>GENSCAN00000057250<br>ENSESTT00000016241 | Homo sapiens transmembrane protein 49 (TMEM49), mRNA.<br>cdna:known-ccds chromosome:NCBI36:17:55139811:55273235:1 gene:ENSG00000062716 CCDS11619.1<br><br><br><br>cdna:Genscan chromosome:NCBI36:17:55163590:55243986:1 |
| 3728991 | 876771 | 3728964 | core | 54635664 | 54635739 | 17 | + | AK000296<br>BC008669 | Homo sapiens cDNA FLJ20289 fis, clone HEP04492.<br>Homo sapiens proline rich 11, mRNA (cDNA clone MGC:8798 IMAGE:3867293), complete cds. |

| | | | | | | | | | |
|---|---|---|---|---|---|---|---|---|---|
| | | | | | | | | ENST00000262293<br>ENSESTT00000033928<br>NM_018304<br>AK225380<br>GENSCAN00000046107 | cdna:known-ccds chromosome:NCBI36:17:54587900:54635977:1 gene:ENSG00000068489 CCDS11614.1<br><br>Homo sapiens proline rich 11 (PRR11), mRNA.<br>Homo sapiens mRNA for hypothetical protein LOC55771 variant, clone: HEP19570.<br>cdna:Genscan chromosome:NCBI36:17:54601896:54633774:1 |
| 3730618 | 877779 | 3730601 | core | 58912659 | 58912826 | 17 | + | NM_000789<br>ENSESTT00000004908<br>ENST00000290866<br>GENSCAN00000050147<br>NM_152830<br>NM_152831<br>ENSESTT00000004909<br>ENSESTT00000004910<br>ENSESTT00000004911<br>ENSESTT00000004912<br>ENST00000290863<br>M26658<br>ENSESTT00000004913<br>ENSESTT00000004914 | Homo sapiens angiotensin I converting enzyme (peptidyl-dipeptidase A) 1 (ACE), transcript variant 1, mRNA.<br><br>cdna:known-ccds chromosome:NCBI36:17:58908166:58928711:1 gene:ENSG00000159640 CCDS11637.1<br>cdna:Genscan chromosome:NCBI36:17:58908188:58976995:1<br>Homo sapiens angiotensin I converting enzyme (peptidyl-dipeptidase A) 1 (ACE), transcript variant 2, mRNA.<br>Homo sapiens angiotensin I converting enzyme (peptidyl-dipeptidase A) 1 (ACE), transcript variant 3, mRNA.<br><br><br><br><br>cdna:known chromosome:NCBI36:17:58915909:58938721:1 gene:ENSG00000159640<br>Human testicular angiotensin converting enzyme mRNA (5' variant), complete cds. |
| 3733601 | 879595 | 3733590 | core | 67628772 | 67628986 | 17 | + | NM_000346<br>BC056420<br>ENST00000245479<br>ENSESTT00000042914<br>GENSCAN00000059056 | Homo sapiens SRY (sex determining region Y)-box 9 (campomelic dysplasia, autosomal sex-reversal) (SOX9), mRNA.<br>Homo sapiens SRY (sex determining region Y)-box 9 (campomelic dysplasia, autosomal sex-reversal), mRNA (cDNA clone MGC:65106 IMAGE:6200521), complete cds.<br>cdna:known-ccds chromosome:NCBI36:17:67628756:67634147:1 gene:ENSG00000125398 CCDS11689.1<br><br>cdna:Genscan chromosome:NCBI36:17:67611413:67632123:1 |
| 3733603 | 879595 | 3733590 | core | 67629042 | 67629073 | 17 | + | NM_000346<br>BC056420<br>GENSCAN00000059056<br>ENST00000245479<br>ENSESTT00000042914 | Homo sapiens SRY (sex determining region Y)-box 9 (campomelic dysplasia, autosomal sex-reversal) (SOX9), mRNA.<br>Homo sapiens SRY (sex determining region Y)-box 9 (campomelic dysplasia, autosomal sex-reversal), mRNA (cDNA clone MGC:65106 IMAGE:6200521), complete cds.<br>cdna:Genscan chromosome:NCBI36:17:67611413:67632123:1<br>cdna:known-ccds chromosome:NCBI36:17:67628756:67634147:1 gene:ENSG00000125398 CCDS11689.1 |
| 3733605 | 879596 | 3733590 | core | 67630557 | 67630700 | 17 | + | NM_000346<br>ENST00000245479<br>BC056420<br>ENSESTT00000042914<br>GENSCAN00000059056 | Homo sapiens SRY (sex determining region Y)-box 9 (campomelic dysplasia, autosomal sex-reversal) (SOX9), mRNA.<br>cdna:known-ccds chromosome:NCBI36:17:67628756:67634147:1 gene:ENSG00000125398 CCDS11689.1<br>Homo sapiens SRY (sex determining region Y)-box 9 (campomelic dysplasia, autosomal sex-reversal), mRNA (cDNA clone MGC:65106 IMAGE:6200521), complete cds.<br><br>cdna:Genscan chromosome:NCBI36:17:67611413:67632123:1 |
| 3735489 | 880704 | 3735478 | core | 71892493 | 71892720 | 17 | + | NM_182965<br>ENSESTT00000056515<br>ENST00000323374<br>NM_021972<br>AK122781<br>ENSESTT00000056513<br>ENSESTT00000056514<br>GENSCAN00000007977<br>AK023393<br>ENSESTT00000056516<br>AK095578<br>ENSESTT00000056517 | Homo sapiens sphingosine kinase 1 (SPHK1), transcript variant 2, mRNA.<br><br>cdna:known-ccds chromosome:NCBI36:17:71892326:71895530:1 gene:ENSG00000176170 CCDS11744.1<br>Homo sapiens sphingosine kinase 1 (SPHK1), transcript variant 1, mRNA.<br>Homo sapiens cDNA FLJ16332 fis, clone SYNOV2016837, highly similar to Homo sapiens sphingosine kinase (SPHK1) mRNA.<br><br><br>cdna:Genscan chromosome:NCBI36:17:71884483:71895262:1<br>Homo sapiens cDNA FLJ13331 fis, clone OVARC1001809, moderately similar to Mus musculus sphingosine kinase (SPHK1a) mRNA.<br><br>Homo sapiens cDNA FLJ38259 fis, clone FCBBF3001302, highly similar to Homo sapiens sphingosine kinase |

| | | | | | | | | | |
|---|---|---|---|---|---|---|---|---|---|
| | | | | | | | | | (SPHK1) mRNA. |
| 3738889 | 882768 | 3738842 | core | 77993602 | 77993677 | 17 | + | ENSESTT00000053634<br>ENST00000337014<br>ENSESTT00000053632<br>ENSESTT00000053633<br>ENST00000327949<br>NM_173620 | <br>cdna:known chromosome:NCBI36:17:77969862:77993806:1 gene:ENSG00000169660<br><br><br>cdna:known chromosome:NCBI36:17:77969860:77993804:1 gene:ENSG00000169660<br>Homo sapiens hexosaminidase (glycosyl hydrolase family 20, catalytic domain) containing (HEXDC), mRNA. |
| 3742296 | 884827 | 3742285 | core | 4589384 | 4589420 | 17 | - | NM_022059<br>CR623711<br>ENSESTT00000058624<br>ENST00000381478<br>ENST00000293778<br>CR611932<br>GENSCAN00000052915 | Homo sapiens chemokine (C-X-C motif) ligand 16 (CXCL16), mRNA.<br>full-length cDNA clone CS0DJ002YJ19 of T cells (Jurkat cell line) Cot 10-normalized of Homo sapiens (human).<br><br>cdna:known-ccds chromosome:NCBI36:17:4583577:4589972:-1 gene:ENSG00000161921 CCDS11052.1<br>cdna:known chromosome:NCBI36:17:4583579:4589890:-1 gene:ENSG00000161921<br>full-length cDNA clone CS0DI028YA08 of Placenta Cot 25-normalized of Homo sapiens (human).<br>cdna:Genscan chromosome:NCBI36:17:4585119:4589383:-1 |
| 3750673 | 889764 | 3750662 | core | 23720661 | 23720719 | 17 | - | NM_000638<br>GENSCAN00000050503<br>ENST00000226218<br>ENSESTT00000018072 | Homo sapiens vitronectin (VTN), mRNA.<br>cdna:Genscan chromosome:NCBI36:17:23718517:23721351:-1<br>cdna:known-ccds chromosome:NCBI36:17:23718425:23721844:-1 gene:ENSG00000109072 CCDS11229.1 |
| 3751864 | 890458 | 3751859 | core | 25669970 | 25670022 | 17 | - | NM_206832<br>ENST00000328886<br>ENSESTT00000002811 | Homo sapiens transmembrane and immunoglobulin domain containing 1 (TMIGD1), mRNA.<br>cdna:known chromosome:NCBI36:17:25667492:25685191:-1 gene:ENSG00000182271 |
| 3751869 | 890463 | 3751859 | core | 25680573 | 25680644 | 17 | - | NM_206832<br>ENST00000328886<br>ENSESTT00000002811 | Homo sapiens transmembrane and immunoglobulin domain containing 1 (TMIGD1), mRNA.<br>cdna:known chromosome:NCBI36:17:25667492:25685191:-1 gene:ENSG00000182271 |
| 3756205 | 893088 | 3756193 | core | 35808368 | 35808419 | 17 | - | NM_001067<br>ENST00000269577<br>ENST00000357601<br>ENST00000348049<br>ENSESTT00000026174<br>GENSCAN00000049618<br>ENSESTT00000026172<br>ENSESTT00000026173<br>AF285157<br>AF285158<br>AF285159 | Homo sapiens topoisomerase (DNA) II alpha 170kDa (TOP2A), mRNA.<br>cdna:known chromosome:NCBI36:17:35798321:35827695:-1 gene:ENSG00000131747<br>cdna:known chromosome:NCBI36:17:35798321:35827695:-1 gene:ENSG00000131747<br>cdna:known chromosome:NCBI36:17:35798321:35827695:-1 gene:ENSG00000131747<br><br>cdna:Genscan chromosome:NCBI36:17:35799297:35827598:-1<br><br><br>Homo sapiens topoisomerase II alpha-2 (TOP2A) mRNA, partial cds.<br>Homo sapiens topoisomerase II alpha-3 (TOP2A) mRNA, partial cds.<br>Homo sapiens topoisomerase II alpha-4 (TOP2A) mRNA, partial cds. |
| 3758526 | 894377 | 3758510 | core | 38978211 | 38978261 | 17 | - | NM_001986<br>DQ396625<br>GENSCAN00000064255<br>BC007242<br>ENSESTT00000014413<br>ENST00000319349<br>ENSESTT00000014412 | Homo sapiens ets variant gene 4 (E1A enhancer binding protein, E1AF) (ETV4), mRNA.<br>Homo sapiens TMPRSS2/ETV4a fusion transcript.<br>cdna:Genscan chromosome:NCBI36:17:38961413:38980118:-1<br>Homo sapiens ets variant gene 4 (E1A enhancer binding protein, E1AF), mRNA (cDNA clone IMAGE:2964546), complete cds.<br><br>cdna:known-ccds chromosome:NCBI36:17:38960738:38979230:-1 gene:ENSG00000175832 CCDS11465.1 |
| 3758864 | 894585 | 3758845 | core | 39515660 | 39515714 | 17 | - | NM_005474<br>NM_001015053<br>ENST00000225983<br>ENST00000336057<br>ENSESTT00000014394<br>ENSESTT00000014395<br>ENSESTT00000014396<br>GENSCAN00000056331 | Homo sapiens histone deacetylase 5 (HDAC5), transcript variant 1, mRNA.<br>Homo sapiens histone deacetylase 5 (HDAC5), transcript variant 3, mRNA.<br>cdna:known chromosome:NCBI36:17:39509647:39556540:-1 gene:ENSG00000108840<br>cdna:known chromosome:NCBI36:17:39509647:39556540:-1 gene:ENSG00000108840<br><br><br><br>cdna:Genscan chromosome:NCBI36:17:39511270:39567854:-1 |

| | | | | | | | | | |
|---|---|---|---|---|---|---|---|---|---|
| | | | | | | | | AK027734<br>AK075095<br>ENSESTT00000014391 | Homo sapiens cDNA FLJ14828 fis, clone OVARC1000915, highly similar to Homo sapiens histone deacetylase 5 mRNA.<br>Homo sapiens cDNA FLJ90614 fis, clone PLACE1002080, highly similar to Homo sapiens antigen NY-CO-9 (NY-CO-9) mRNA. |
| 3765182 | 898401 | 3765167 | core | 55622741 | 55622770 | 17 | - | NM_032582<br>ENST00000300896<br>ENSESTT00000016248<br>ENSESTT00000016249<br>ENSESTT00000016250 | Homo sapiens ubiquitin specific peptidase 32 (USP32), mRNA.<br>cdna:known chromosome:NCBI36:17:55609473:55824368:-1 gene:ENSG00000170832 |
| 3768666 | 900523 | 3768627 | core | 64401933 | 64402041 | 17 | - | NM_007168<br>BC037309<br>ENST00000269080<br>ENST00000356282<br>ENSESTT00000032158<br>GENSCAN00000039049<br>ENSESTT00000032157<br>ENST00000375538<br>BC024003<br>BC047765<br>ENSESTT00000032152<br>ENSESTT00000032154 | Homo sapiens ATP-binding cassette, sub-family A (ABC1), member 8 (ABCA8), mRNA.<br>Homo sapiens ATP-binding cassette, sub-family A (ABC1), member 8, mRNA (cDNA clone IMAGE:5262251), complete cds.<br>cdna:known-ccds chromosome:NCBI36:17:64375028:64463128:-1 gene:ENSG00000141338 CCDS11680.1<br>cdna:known chromosome:NCBI36:17:64375028:64463128:-1 gene:ENSG00000141338<br>cdna:Genscan chromosome:NCBI36:17:64375821:64448611:-1<br>cdna:known chromosome:NCBI36:17:64429151:64463128:-1 gene:ENSG00000141338<br>Homo sapiens ATP-binding cassette, sub-family A (ABC1), member 8, mRNA (cDNA clone IMAGE:4821839), complete cds.<br>Homo sapiens ATP-binding cassette, sub-family A (ABC1), member 8, mRNA (cDNA clone IMAGE:5761384), complete cds. |
| 3771188 | 902097 | 3771160 | core | 71429205 | 71429231 | 17 | - | XM_946191<br>XM_932831<br>AB058766<br>BC007570<br>BC012332<br>ENST00000389570<br>ENSESTT00000053299<br>ENST00000354315<br>ENSESTT00000053300<br>ENST00000319129<br>GENSCAN00000036029<br>XM_942657<br>XM_946192<br>XM_946193<br>XM_371079<br>XM_932838<br>XM_932840<br>BC023549<br>ENST00000337792<br>ENSESTT00000053297<br>ENSESTT00000053298<br>ENST00000375160<br>ENSESTT00000053293<br>ENSESTT00000053294<br>ENSESTT00000053295 | PREDICTED: Homo sapiens Fas (TNFRSF6) binding factor 1, transcript variant 8 (FBF1), mRNA.<br>PREDICTED: Homo sapiens Fas (TNFRSF6) binding factor 1, transcript variant 3 (FBF1), mRNA.<br>Homo sapiens mRNA for KIAA1863 protein, partial cds.<br>Homo sapiens Fas (TNFRSF6) binding factor 1, mRNA (cDNA clone IMAGE:3029289), partial cds.<br>Homo sapiens Fas (TNFRSF6) binding factor 1, mRNA (cDNA clone IMAGE:4562904), partial cds.<br>cdna:known chromosome:NCBI36:17:71418402:71445873:-1 gene:ENSG00000188878<br>cdna:known chromosome:NCBI36:17:71422472:71431308:-1 gene:ENSG00000188878<br>cdna:novel chromosome:NCBI36:17:71417523:71431308:-1 gene:ENSG00000188878<br>cdna:Genscan chromosome:NCBI36:17:71406526:71459253:-1<br>PREDICTED: Homo sapiens Fas (TNFRSF6) binding factor 1, transcript variant 6 (FBF1), mRNA.<br>PREDICTED: Homo sapiens Fas (TNFRSF6) binding factor 1, transcript variant 9 (FBF1), mRNA.<br>PREDICTED: Homo sapiens Fas (TNFRSF6) binding factor 1, transcript variant 10 (FBF1), mRNA.<br>PREDICTED: Homo sapiens Fas (TNFRSF6) binding factor 1, transcript variant 1 (FBF1), mRNA.<br>PREDICTED: Homo sapiens Fas (TNFRSF6) binding factor 1, transcript variant 4 (FBF1), mRNA.<br>PREDICTED: Homo sapiens Fas (TNFRSF6) binding factor 1, transcript variant 5 (FBF1), mRNA.<br>Homo sapiens Fas (TNFRSF6) binding factor 1, mRNA (cDNA clone MGC:16590 IMAGE:4109753), complete cds.<br>cdna:known chromosome:NCBI36:17:71417582:71448816:-1 gene:ENSG00000188878<br>cdna:known chromosome:NCBI36:17:71427749:71448701:-1 gene:ENSG00000188878 |
| 3771265 | 902148 | 3771259 | core | 71515240 | 71516370 | 17 | - | NM_001988<br>GENSCAN00000044500 | Homo sapiens envoplakin (EVPL), mRNA.<br>cdna:Genscan chromosome:NCBI36:17:71514779:71580167:-1 |

| | | | | | | | | | |
|---|---|---|---|---|---|---|---|---|---|
| | | | | | | | | ENST00000301607<br>ENSESTT00000053288<br>ENSESTT00000053287 | cdna:known-ccds chromosome:NCBI36:17:71514522:71534972:-1 gene:ENSG00000167880 CCDS11737.1 |
| 3773253 | 903380 | 3773244 | core | 75536078 | 75536204 | 17 | - | NM_019020<br>ENST00000310924<br>CR936670<br>ENSESTT00000044719<br>ENSESTT00000044721<br>ENSESTT00000044723<br>ENST00000340848<br>GENSCAN00000040687<br>BC001525<br>ENSESTT00000044720<br>ENSESTT00000044722<br>ENSESTT00000044724<br>ENSESTT00000044718<br>GENSCAN00000029393<br>ENSESTT00000044716 | Homo sapiens TBC1 domain family, member 16 (TBC1D16), mRNA.<br>cdna:known-ccds chromosome:NCBI36:17:75528416:75624242:-1 gene:ENSG00000167291 CCDS11766.1<br>Homo sapiens mRNA; cDNA DKFZp451F1311 (from clone DKFZp451F1311).<br>cdna:known chromosome:NCBI36:17:75526303:75539232:-1 gene:ENSG00000167291<br>cdna:Genscan chromosome:NCBI36:17:75529253:75542981:-1<br>Homo sapiens TBC1 domain family, member 16, mRNA (cDNA clone IMAGE:3050107), partial cds.<br>cdna:Genscan chromosome:NCBI36:17:75589304:75601941:-1 |
| 3781483 | 908350 | 3781429 | core | 18826739 | 18826804 | 18 | + | NM_203292<br>BC001170<br>NM_002894<br>NM_203291<br>ENSESTT00000044675<br>ENST00000327155<br>ENST00000327174<br>ENST00000360790<br>ENSESTT00000053553<br>ENSESTT00000053554<br>ENSESTT00000053555 | Homo sapiens retinoblastoma binding protein 8 (RBBP8), transcript variant 3, mRNA.<br>Homo sapiens, Similar to retinoblastoma-binding protein 8, clone IMAGE:3357638, mRNA.<br>Homo sapiens retinoblastoma binding protein 8 (RBBP8), transcript variant 1, mRNA.<br>Homo sapiens retinoblastoma binding protein 8 (RBBP8), transcript variant 2, mRNA.<br>cdna:known-ccds chromosome:NCBI36:18:18767293:18860443:1 gene:ENSG00000101773 CCDS11875.1<br>cdna:known-ccds chromosome:NCBI36:18:18767293:18860443:1 gene:ENSG00000101773 CCDS11874.1<br>cdna:known chromosome:NCBI36:18:18768709:18860328:1 gene:ENSG00000101773 |
| 3783804 | 909788 | 3783788 | core | 28047203 | 28047421 | 18 | + | NM_005925<br>ENST00000269202<br>ENSESTT00000011881<br>GENSCAN00000056863 | Homo sapiens meprin A, beta (MEP1B), mRNA.<br>cdna:known chromosome:NCBI36:18:28023985:28054362:1 gene:ENSG00000141434<br>cdna:Genscan chromosome:NCBI36:18:28036856:28051078:1 |
| 3789456 | 913456 | 3789442 | core | 52512904 | 52512986 | 18 | + | NM_015285<br>ENSESTT00000002315<br>NM_052834<br>ENSESTT00000002313<br>ENST00000254442<br>ENST00000357574<br>GENSCAN00000030675<br>ENSESTT00000002320<br>ENSESTT00000002321<br>ENSESTT00000002325<br>ENSESTT00000002326<br>GENSCAN00000041379<br>GENSCAN00000041376 | Homo sapiens WD repeat domain 7 (WDR7), transcript variant 1, mRNA.<br>Homo sapiens WD repeat domain 7 (WDR7), transcript variant 2, mRNA.<br>cdna:known-ccds chromosome:NCBI36:18:52469614:52848034:1 gene:ENSG00000091157 CCDS11962.1<br>cdna:known-ccds chromosome:NCBI36:18:52469614:52848034:1 gene:ENSG00000091157 CCDS11963.1<br>cdna:Genscan chromosome:NCBI36:18:52490745:52514691:1<br>cdna:Genscan chromosome:NCBI36:18:52676017:52752864:1<br>cdna:Genscan chromosome:NCBI36:18:52780666:52845866:1 |
| 3790286 | 914015 | 3790259 | core | 54541310 | 54541424 | 18 | + | NM_173844<br>NM_006785<br>ENST00000348428<br>ENST00000345724<br>ENSESTT00000034293<br>ENSESTT00000034294<br>AK092004 | Homo sapiens mucosa associated lymphoid tissue lymphoma translocation gene 1 (MALT1), transcript variant 2, mRNA.<br>Homo sapiens mucosa associated lymphoid tissue lymphoma translocation gene 1 (MALT1), transcript variant 1, mRNA.<br>cdna:known-ccds chromosome:NCBI36:18:54489598:54568350:1 gene:ENSG00000172175 CCDS11967.1<br>cdna:known-ccds chromosome:NCBI36:18:54489598:54568350:1 gene:ENSG00000172175 CCDS11968.1 |

| | | | | | | | | | |
|---|---|---|---|---|---|---|---|---|---|
| | | | | | | | | ENSESTT00000034295 | Homo sapiens cDNA FLJ34685 fis, clone LIVER2009110, highly similar to Homo sapiens API2-MLT fusion protein (API2-MLT) mRNA. |
| 3791024 | 914465 | 3790982 | core | 57368197 | 57368442 | 18 | + | NM_031891<br>ENST00000262717<br>GENSCAN00000036120<br>AK094366 | Homo sapiens cadherin 20, type 2 (CDH20), mRNA.<br>cdna:known-ccds chromosome:NCBI36:18:57308755:57373345:1 gene:ENSG00000101542 CCDS11977.1<br>cdna:Genscan chromosome:NCBI36:18:57308785:57363357:1<br>Homo sapiens cDNA FLJ37047 fis, clone BRACE2012232, highly similar to Homo sapiens cadherin 20 (CDH20) mRNA. |
| 3793781 | 916156 | 3793760 | core | 70318102 | 70318146 | 18 | + | NM_018235<br>ENSESTT00000002307<br>ENST00000324262<br>ENST00000324301<br>ENST00000382661<br>ENSESTT00000002308<br>AK097155<br>ENSESTT00000002303<br>ENSESTT00000002304<br>CR590069<br>ENSESTT00000002306<br>GENSCAN00000027024<br>AK024471<br>ENSESTT00000002309 | Homo sapiens CNDP dipeptidase 2 (metallopeptidase M20 family) (CNDP2), mRNA.<br><br>cdna:known-ccds chromosome:NCBI36:18:70314577:70339336:1 gene:ENSG00000133313 CCDS12006.1<br>cdna:known chromosome:NCBI36:18:70314577:70339336:1 gene:ENSG00000133313<br>cdna:novel chromosome:NCBI36:18:70314577:70339336:1 gene:ENSG00000133313<br><br>Homo sapiens cDNA FLJ39836 fis, clone SPLEN2014073.<br><br><br>full-length cDNA clone CS0DC010YD11 of Neuroblastoma Cot 25-normalized of Homo sapiens (human).<br><br>cdna:Genscan chromosome:NCBI36:18:70318189:70338283:1<br>Homo sapiens mRNA for FLJ00064 protein, partial cds. |
| 3795922 | 917539 | 3795866 | core | 687245 | 687334 | 18 | - | NM_017512<br>BX648215<br>ENSESTT00000002160<br>ENST00000251101<br>ENST00000383578<br>AK093873<br>AK127219<br>AK127818<br>X67098<br>ENSESTT00000002146<br>ENSESTT00000002147<br>ENSESTT00000002149<br>ENSESTT00000002151<br>ENSESTT00000002155<br>ENSESTT00000002158<br>ENST00000340116<br>ENST00000319815<br>GENSCAN00000027512<br>ENSESTT00000002153 | Homo sapiens enolase superfamily member 1 (ENOSF1), mRNA.<br>Homo sapiens mRNA; cDNA DKFZp779G0642 (from clone DKFZp779G0642).<br><br>cdna:known-ccds chromosome:NCBI36:18:663918:702642:-1 gene:ENSG00000132199 CCDS11822.1<br>cdna:known chromosome:NCBI36:18:663918:702642:-1 gene:ENSG00000132199<br>Homo sapiens cDNA FLJ36554 fis, clone TRACH2008597, highly similar to H.sapiens rTS alpha mRNA.<br>Homo sapiens cDNA FLJ45286 fis, clone BRHIP3002114, highly similar to Homo sapiens rTS beta protein (HSRTSBETA).<br>Homo sapiens cDNA FLJ45920 fis, clone PLACE6003004, highly similar to Homo sapiens rTS beta protein (HSRTSBETA).<br>H.sapiens rTS alpha mRNA containing four open reading frames.<br><br><br><br><br><br><br>cdna:known-ccds chromosome:NCBI36:18:663900:702506:-1 gene:ENSG00000132199 CCDS11823.1<br>cdna:known chromosome:NCBI36:18:663900:702515:-1 gene:ENSG00000132199<br>cdna:Genscan chromosome:NCBI36:18:664305:696638:-1 |
| 3815413 | 929924 | 3815399 | core | 988820 | 988846 | 19 | + | NM_004368<br>AK093630<br>ENSESTT00000021874<br>GENSCAN00000009502<br>NM_201277<br>ENST00000263097<br>ENST00000348419<br>AY927514 | Homo sapiens calponin 2 (CNN2), transcript variant 1, mRNA.<br>Homo sapiens cDNA FLJ36311 fis, clone THYMU2005046, highly similar to CALPONIN H2, SMOOTH MUSCLE.<br><br>cdna:Genscan chromosome:NCBI36:19:986955:988899:1<br>Homo sapiens calponin 2 (CNN2), transcript variant 2, mRNA.<br>cdna:known-ccds chromosome:NCBI36:19:977298:990060:1 gene:ENSG00000064666 CCDS12053.1<br>cdna:known-ccds chromosome:NCBI36:19:977298:990060:1 gene:ENSG00000064666 CCDS12054.1<br>Homo sapiens mRNA sequence. |

| | | | | | | | | | |
|---|---|---|---|---|---|---|---|---|---|
| 3816655 | 930613 | 3816645 | core | 2783357 | 2783427 | 19 | + | NM_152303<br>ENSESTT00000017061<br>ENSESTT00000017059<br>ENST00000317243<br>GENSCAN00000047607 | Homo sapiens zinc finger protein 554 (ZNF554), mRNA.<br><br><br>cdna:known chromosome:NCBI36:19:2770961:2786469:1 gene:ENSG00000172006<br>cdna:Genscan chromosome:NCBI36:19:2774078:2785850:1 |
| 3820546 | 932866 | 3820501 | core | 10433208 | 10433348 | 19 | + | NM_006202<br>CR604925<br>ENST00000344979<br>AF073745<br>AY593872<br>AY618547<br>BC038234<br>L20965<br>ENSESTT00000043701<br>ENST00000380702<br>ENST00000270478<br>ENST00000293683<br>ENST00000309641<br>ENST00000352831<br>ENSESTT00000043702<br>U18088<br>ENST00000380686<br>AY266362<br>ENSESTT00000043703 | Homo sapiens phosphodiesterase 4A, cAMP-specific (phosphodiesterase E2 dunce homolog, Drosophila) (PDE4A), mRNA.<br>full-length cDNA clone CS0DF038YH04 of Fetal brain of Homo sapiens (human).<br>cdna:known-ccds chromosome:NCBI36:19:10424637:10441306:1 gene:ENSG00000065989 CCDS12238.1<br>Homo sapiens cyclic AMP-specific phosphodiesterase HSPDE4A10 (PDE4A) mRNA, complete cds.<br>Homo sapiens cAMP-specific phosphodiesterase (PDE4A) mRNA, complete cds.<br>Homo sapiens cyclic AMP phosphodiesterase PDE4A11 (PDE4A) mRNA, partial cds, alternatively spliced.<br>Homo sapiens phosphodiesterase 4A, cAMP-specific (phosphodiesterase E2 dunce homolog, Drosophila), mRNA (cDNA clone MGC:46146 IMAGE:5732341), complete cds.<br>Human phosphodiesterase mRNA, complete cds.<br><br>cdna:known chromosome:NCBI36:19:10388449:10439325:1 gene:ENSG00000065989<br>cdna:known chromosome:NCBI36:19:10392331:10440169:1 gene:ENSG00000065989<br>cdna:known chromosome:NCBI36:19:10402521:10439297:1 gene:ENSG00000065989<br>cdna:known chromosome:NCBI36:19:10402521:10439297:1 gene:ENSG00000065989<br>cdna:known chromosome:NCBI36:19:10404111:10439297:1 gene:ENSG00000065989<br><br>Human 3',5'-cyclic AMP phosphodiesterase inactive splice variant HSPDE4A7A mRNA, complete cds.<br>cdna:known chromosome:NCBI36:19:10430645:10436099:1 gene:ENSG00000065989<br>Homo sapiens phosphodiesterase isozyme 4 (PDE4) mRNA, partial cds. |
| 3821280 | 933297 | 3821263 | core | 11521987 | 11522064 | 19 | + | NM_001299<br>ENSESTT00000055953<br>ENST00000252456<br>ENSESTT00000055954<br>ENSESTT00000055955<br>GENSCAN00000044395 | Homo sapiens calponin 1, basic, smooth muscle (CNN1), mRNA.<br><br>cdna:known-ccds chromosome:NCBI36:19:11510579:11522138:1 gene:ENSG00000130176 CCDS12263.1<br><br><br>cdna:Genscan chromosome:NCBI36:19:11495251:11523578:1 |
| 3825620 | 935778 | 3825609 | core | 19198298 | 19198392 | 19 | + | NM_004386<br>GENSCAN00000042126<br>ENSESTT00000003696<br>ENST00000252575<br>ENSESTT00000003698<br>ENSESTT00000003699<br>AK126639<br>ENSESTT00000003700 | Homo sapiens chondroitin sulfate proteoglycan 3 (neurocan) (CSPG3), mRNA.<br>cdna:Genscan chromosome:NCBI36:19:19188763:19200448:1<br><br>cdna:known-ccds chromosome:NCBI36:19:19183838:19224040:1 gene:ENSG00000130287 CCDS12397.1<br><br><br>Homo sapiens cDNA FLJ44681 fis, clone BRACE3010086, moderately similar to Homo sapiens chondroitin sulfate proteoglycan 3 (neurocan) (CSPG3). |
| 3826071 | 936035 | 3826041 | core | 19865375 | 19865403 | 19 | + | CR590630<br>BC022891<br>BC053607<br>ENST00000355650 | full-length cDNA clone CS0DF018YD14 of Fetal brain of Homo sapiens (human).<br>Homo sapiens zinc finger protein 253, mRNA (cDNA clone IMAGE:4658943), with apparent retained intron.<br>Homo sapiens zinc finger protein 253, mRNA (cDNA clone IMAGE:6012910), with apparent retained intron.<br>cdna:known chromosome:NCBI36:19:19837709:20276411:1 gene:ENSG00000081665 |
| 3828304 | 937339 | 3828278 | core | 35555180 | 35555267 | 19 | + | NM_014717<br>ENST00000355537<br>GENSCAN00000066451<br>GENSCAN00000022205 | Homo sapiens zinc finger protein 536 (ZNF536), mRNA.<br>cdna:known chromosome:NCBI36:19:35555168:35740805:1 gene:ENSG00000198597<br>cdna:Genscan chromosome:NCBI36:19:35622104:35629361:1<br>cdna:Genscan chromosome:NCBI36:19:35694309:35732999:1 |
| 3832767 | 940012 | 3832760 | core | 44087513 | 44087543 | 19 | + | NM_002503<br>AB209784<br>ENSESTT00000033695 | Homo sapiens nuclear factor of kappa light polypeptide gene enhancer in B-cells inhibitor, beta (NFKBIB), transcript variant 1, mRNA.<br>Homo sapiens mRNA for NF-kappaB inhibitor beta variant protein. |

| | | | | | | | | | |
|---|---|---|---|---|---|---|---|---|---|
| | | | | | | | | ENSESTT00000033696<br>ENST00000313582<br>GENSCAN00000019479 | cdna:known-ccds chromosome:NCBI36:19:44082455:44091371:1 gene:ENSG00000104825 CCDS12524.1<br>cdna:Genscan chromosome:NCBI36:19:44082000:44091312:1 |
| 3837815 | 942927 | 3837796 | core | 53646262 | 53646297 | 19 | + | NM_031485<br>AK131055<br>ENST00000253237<br>AB075822<br>ENSESTT00000016491<br>GENSCAN00000046121 | Homo sapiens glutamate-rich WD repeat containing 1 (GRWD1), mRNA.<br>Homo sapiens cDNA FLJ29021 fis, clone PNC06750, highly similar to Glutamate-rich WD repeat protein.<br>cdna:known-ccds chromosome:NCBI36:19:53640874:53648966:1 gene:ENSG00000105447 CCDS12720.1<br>Homo sapiens mRNA for KIAA1942 protein.<br>cdna:Genscan chromosome:NCBI36:19:53641075:53648094:1 |
| 3838431 | 943250 | 3838425 | core | 54559666 | 54559766 | 19 | + | NM_014419<br>ENST00000221498<br>AF177398<br>ENSESTT00000010393<br>ENSESTT00000010394<br>ENSESTT00000010395<br>GENSCAN00000036904 | Homo sapiens dickkopf-like 1 (soggy) (DKKL1), mRNA.<br>cdna:known-ccds chromosome:NCBI36:19:54558854:54570183:1 gene:ENSG00000104901 CCDS12762.1<br>Homo sapiens soggy-1 protein (SGY-1) mRNA, complete cds.<br>cdna:Genscan chromosome:NCBI36:19:54556945:54565532:1 |
| 3839352 | 943766 | 3839346 | core | 55617938 | 55618101 | 19 | + | ENSESTT00000004336<br>NM_003121<br>ENSESTT00000004335<br>ENST00000270632<br>GENSCAN00000031985 | Homo sapiens Spi-B transcription factor (Spi-1/PU.1 related) (SPIB), mRNA.<br>cdna:known chromosome:NCBI36:19:55614028:55624058:1 gene:ENSG00000142539<br>cdna:Genscan chromosome:NCBI36:19:55614032:55623405:1 |
| 3839355 | 943769 | 3839346 | core | 55623109 | 55623138 | 19 | + | NM_003121<br>ENSESTT00000004335<br>ENSESTT00000004336<br>ENST00000270632<br>GENSCAN00000031985 | Homo sapiens Spi-B transcription factor (Spi-1/PU.1 related) (SPIB), mRNA.<br>cdna:known chromosome:NCBI36:19:55614028:55624058:1 gene:ENSG00000142539<br>cdna:Genscan chromosome:NCBI36:19:55614032:55623405:1 |
| 3841091 | 944684 | 3841076 | core | 59069406 | 59069511 | 19 | + | NM_001020818<br>BC013995<br>ENST00000336967<br>GENSCAN00000037427<br>NM_001020819<br>NM_001020821<br>NM_001020820<br>NM_138373 | Homo sapiens myeloid-associated differentiation marker (MYADM), transcript variant 1, mRNA.<br>Homo sapiens myeloid-associated differentiation marker, mRNA (cDNA clone MGC:20205 IMAGE:3627858), complete cds.<br>cdna:known-ccds chromosome:NCBI36:19:59061423:59071494:1 gene:ENSG00000179820 CCDS12866.1<br>cdna:Genscan chromosome:NCBI36:19:59061332:59069564:1<br>Homo sapiens myeloid-associated differentiation marker (MYADM), transcript variant 3, mRNA.<br>Homo sapiens myeloid-associated differentiation marker (MYADM), transcript variant 5, mRNA.<br>Homo sapiens myeloid-associated differentiation marker (MYADM), transcript variant 4, mRNA.<br>Homo sapiens myeloid-associated differentiation marker (MYADM), transcript variant 2, mRNA. |
| 3843716 | 946133 | 3843690 | core | 63256807 | 63257161 | 19 | + | NM_182572<br>ENST00000282326<br>BC107729<br>GENSCAN00000049884 | Homo sapiens zinc finger and SCAN domain containing 1 (ZSCAN1), mRNA.<br>cdna:known-ccds chromosome:NCBI36:19:63237246:63257811:1 gene:ENSG00000152467 CCDS12969.1<br>Homo sapiens zinc finger and SCAN domain containing 1, mRNA (cDNA clone IMAGE:4688272), complete cds.<br>cdna:Genscan chromosome:NCBI36:19:63237221:63243724:1 |
| 3846394 | 947630 | 3846390 | core | 3721635 | 3721742 | 19 | - | NM_032753<br>ENST00000328906<br>GENSCAN00000068855 | Homo sapiens retina and anterior neural fold homeobox like 1 (RAXL1), mRNA.<br>cdna:known-ccds chromosome:NCBI36:19:3448813:3723221:-1 gene:ENSG00000173976 CCDS12112.1<br>cdna:Genscan chromosome:NCBI36:19:3721619:3757123:-1 |
| 3851708 | 950736 | 3851703 | core | 12703172 | 12703232 | 19 | - | NM_024038<br>CR610021<br>ENSESTT00000046111<br>ENST00000242784<br>GENSCAN00000043254 | Homo sapiens chromosome 19 open reading frame 43 (C19orf43), mRNA.<br>full-length cDNA clone CS0DJ013YJ18 of T cells (Jurkat cell line) Cot 10-normalized of Homo sapiens (human).<br>cdna:known-ccds chromosome:NCBI36:19:12702487:12706589:-1 gene:ENSG00000123144 CCDS12279.1<br>cdna:Genscan chromosome:NCBI36:19:12702366:12706471:-1 |
| 3853151 | 951536 | 3853108 | core | 15156752 | 15156826 | 19 | - | NM_000435<br>ENST00000263388<br>ENSESTT00000032951 | Homo sapiens Notch homolog 3 (Drosophila) (NOTCH3), mRNA.<br>cdna:known-ccds chromosome:NCBI36:19:15131445:15172792:-1 gene:ENSG00000074181 CCDS12326.1 |

| | | | | | | | | | |
|---|---|---|---|---|---|---|---|---|---|
| | | | | | | | | ENSESTT00000032952<br>GENSCAN00000015517<br>ENSESTT00000032948<br>ENSESTT00000032949<br>ENSESTT00000032947 | <br>cdna:Genscan chromosome:NCBI36:19:15132473:15168156:-1 |
| 3855837 | 953053 | 3855818 | core | 19542593 | 19542642 | 19 | - | NM_025245<br>ENSESTT00000003734<br>ENST00000251203<br>ENSESTT00000003733<br>GENSCAN00000032822 | Homo sapiens pre-B-cell leukemia transcription factor 4 (PBX4), mRNA.<br><br>cdna:known-ccds chromosome:NCBI36:19:19533524:19590462:-1 gene:ENSG00000105717 CCDS12406.1<br><br>cdna:Genscan chromosome:NCBI36:19:19534179:19571174:-1 |
| 3859781 | 955445 | 3859761 | core | 40686051 | 40686154 | 19 | - | AK125695<br>ENSESTT00000029534<br>AY358412<br>ENST00000379073 | Homo sapiens cDNA FLJ43707 fis, clone TESOP2001865.<br><br>Homo sapiens clone DNA59212 KFQG729 (UNQ729) mRNA, complete cds.<br>cdna:known chromosome:NCBI36:19:40686049:40696400:-1 gene:ENSG00000161249 |
| 3859783 | 955446 | 3859761 | core | 40688472 | 40688496 | 19 | - | NM_033317<br>NM_001035516<br>AK096215<br>BC004493<br>ENSESTT00000029536<br>ENSESTT00000029539<br>ENST00000339686<br>ENST00000379075<br>ENST00000379074<br>AY789699<br>AY789701<br>ENSESTT00000029533<br>ENSESTT00000029538<br>GENSCAN00000027868<br>ENSESTT00000029532<br>ENSESTT00000029537<br>ENSESTT00000029535<br>AK125695<br>AY358412<br>ENSESTT00000029534<br>ENST00000379073 | Homo sapiens dermokine (DMKN), transcript variant 2, mRNA.<br>Homo sapiens dermokine (DMKN), transcript variant 1, mRNA.<br>Homo sapiens cDNA FLJ38896 fis, clone NOVAR2000352.<br>Homo sapiens dermokine, mRNA (cDNA clone IMAGE:3690018), complete cds.<br><br><br>cdna:known-ccds chromosome:NCBI36:19:40679963:40696394:-1 gene:ENSG00000161249 CCDS12463.1<br>cdna:known chromosome:NCBI36:19:40679963:40693212:-1 gene:ENSG00000161249<br>cdna:known chromosome:NCBI36:19:40679963:40684653:-1 gene:ENSG00000161249<br>Homo sapiens dermokine-delta 2 mRNA, complete cds, alternatively spliced.<br>Homo sapiens dermokine-delta 4 mRNA, complete cds, alternatively spliced.<br><br><br>cdna:Genscan chromosome:NCBI36:19:40681458:40692994:-1<br><br><br><br>Homo sapiens cDNA FLJ43707 fis, clone TESOP2001865.<br>Homo sapiens clone DNA59212 KFQG729 (UNQ729) mRNA, complete cds.<br><br>cdna:known chromosome:NCBI36:19:40686049:40696400:-1 gene:ENSG00000161249 |
| 3871205 | 961798 | 3871192 | core | 60394865 | 60394921 | 19 | - | NM_002842<br>ENST00000376350<br>ENST00000263434<br>GENSCAN00000017481<br>ENSESTT00000007332<br>AK097257<br>ENSESTT00000007331 | Homo sapiens protein tyrosine phosphatase, receptor type, H (PTPRH), mRNA.<br>cdna:known chromosome:NCBI36:19:60384428:60412654:-1 gene:ENSG00000080031<br>cdna:known chromosome:NCBI36:19:60384428:60412654:-1 gene:ENSG00000080031<br>cdna:Genscan chromosome:NCBI36:19:60355014:60412613:-1<br><br>Homo sapiens cDNA FLJ39938 fis, clone SPLEN2022162, highly similar to Homo sapiens transmembrane-type protein tyrosine phosphatase H (PTPRH) gene. |
| 3873670 | 963142 | 3873629 | core | 1866236 | 1866896 | 20 | + | BC033092<br>ENST00000356025<br>ENST00000358771<br>NM_080792<br>NM_001040022<br>GENSCAN00000003222<br>NM_001040023 | Homo sapiens signal-regulatory protein alpha, mRNA (cDNA clone MGC:45658 IMAGE:4589298), complete cds.<br>cdna:known-ccds chromosome:NCBI36:20:1823425:1868532:1 gene:ENSG00000198053 CCDS13022.1<br>cdna:known chromosome:NCBI36:20:1823942:1868543:1 gene:ENSG00000198053<br>Homo sapiens signal-regulatory protein alpha (SIRPA), transcript variant 3, mRNA.<br>Homo sapiens signal-regulatory protein alpha (SIRPA), transcript variant 1, mRNA.<br>cdna:Genscan chromosome:NCBI36:20:1806178:1844101:1<br>Homo sapiens signal-regulatory protein alpha (SIRPA), transcript variant 2, mRNA. |
| 3874442 | 963638 | 3874438 | core | 3724530 | 3724719 | 20 | + | NM_004358 | Homo sapiens cell division cycle 25B (CDC25B), transcript variant 2, mRNA. |

| | | | | | | | | | |
|---|---|---|---|---|---|---|---|---|---|
| | | | | | | | | ENST00000379590<br>NM_021873<br>NM_021872<br>ENST00000245959<br>ENST00000245960<br>ENST00000344256<br>ENST00000340833<br>ENST00000361765<br>ENST00000379598<br>ENST00000379588<br>ENSESTT00000037063<br>ENST00000379647<br>ENST00000379584<br>ENST00000245958<br>GENSCAN00000060710<br>BX640836<br>ENSESTT00000037064 | cdna:known chromosome:NCBI36:20:3724407:3734762:1 gene:ENSG00000101224<br>Homo sapiens cell division cycle 25B (CDC25B), transcript variant 1, mRNA.<br>Homo sapiens cell division cycle 25B (CDC25B), transcript variant 3, mRNA.<br>cdna:known-ccds chromosome:NCBI36:20:3724401:3734757:1 gene:ENSG00000101224 CCDS13068.1<br>cdna:known-ccds chromosome:NCBI36:20:3724401:3734757:1 gene:ENSG00000101224 CCDS13067.1<br>cdna:known-ccds chromosome:NCBI36:20:3724401:3734757:1 gene:ENSG00000101224 CCDS13065.1<br>cdna:known-ccds chromosome:NCBI36:20:3724401:3734757:1 gene:ENSG00000101224 CCDS13066.1<br>cdna:known chromosome:NCBI36:20:3724401:3734757:1 gene:ENSG00000101224<br>cdna:known chromosome:NCBI36:20:3724401:3734757:1 gene:ENSG00000101224<br>cdna:known chromosome:NCBI36:20:3724938:3734762:1 gene:ENSG00000101224<br><br>cdna:known-ccds chromosome:NCBI36:20:3724386:3733884:1 gene:ENSG00000101224 CCDS13069.1<br>cdna:known chromosome:NCBI36:20:3724956:3734762:1 gene:ENSG00000101224<br>cdna:known chromosome:NCBI36:20:3725078:3733865:1 gene:ENSG00000101224<br>cdna:Genscan chromosome:NCBI36:20:3725179:3733608:1<br>Homo sapiens mRNA; cDNA DKFZp686G14213 (from clone DKFZp686G14213). |
| 3878856 | 966399 | 3878836 | core | 19818264 | 19818301 | 20 | + | NM_018993<br>ENSESTT00000044418<br>ENSESTT00000044420<br>AK094884<br>ENST00000255006<br>ENST00000377373<br>GENSCAN00000047733<br>ENSESTT00000014463<br>ENSESTT00000014464<br>ENSESTT00000014465 | Homo sapiens Ras and Rab interactor 2 (RIN2), mRNA.<br><br><br>Homo sapiens cDNA FLJ37565 fis, clone BRCOC2000850, highly similar to Human ras inhibitor mRNA.<br>cdna:known chromosome:NCBI36:20:19815165:19931101:1 gene:ENSG00000132669<br>cdna:known chromosome:NCBI36:20:19815165:19929580:1 gene:ENSG00000132669<br>cdna:Genscan chromosome:NCBI36:20:19857311:19929580:1 |
| 3881289 | 967907 | 3881282 | core | 29565948 | 29565985 | 20 | + | NM_178581<br>ENST00000340852<br>ENST00000376118<br>GENSCAN00000062391<br>NM_178582<br>NM_178580<br>NM_030789<br>ENST00000335574<br>ENST00000344042<br>ENST00000262657<br>ENST00000376127<br>BC062595<br>ENSESTT00000040696<br>ENSESTT00000040697<br>GENSCAN00000057053<br>BC053868<br>ENSESTT00000040698 | Homo sapiens histocompatibility (minor) 13 (HM13), transcript variant 3, mRNA.<br>cdna:known-ccds chromosome:NCBI36:20:29565892:29621031:1 gene:ENSG00000101294 CCDS13182.1<br>cdna:known chromosome:NCBI36:20:29565921:29590079:1 gene:ENSG00000101294<br>cdna:Genscan chromosome:NCBI36:20:29548613:29566198:1<br>Homo sapiens histocompatibility (minor) 13 (HM13), transcript variant 4, mRNA.<br>Homo sapiens histocompatibility (minor) 13 (HM13), transcript variant 2, mRNA.<br>Homo sapiens histocompatibility (minor) 13 (HM13), transcript variant 1, mRNA.<br>cdna:known-ccds chromosome:NCBI36:20:29565902:29621029:1 gene:ENSG00000101294 CCDS13183.1<br>cdna:known-ccds chromosome:NCBI36:20:29565902:29591257:1 gene:ENSG00000101294 CCDS13184.1<br>cdna:known chromosome:NCBI36:20:29565902:29621029:1 gene:ENSG00000101294<br>cdna:known chromosome:NCBI36:20:29565902:29621029:1 gene:ENSG00000101294<br>Homo sapiens histocompatibility (minor) 13, mRNA (cDNA clone MGC:74542 IMAGE:5245982), complete cds.<br><br><br>cdna:Genscan chromosome:NCBI36:20:29578948:29657277:1<br>Homo sapiens cDNA clone IMAGE:5194843, **** WARNING: chimeric clone ****. |
| 3881464 | 968010 | 3881443 | core | 29828967 | 29829095 | 20 | + | NM_012112<br>ENST00000300403<br>ENST00000340513<br>AF287265<br>ENSESTT00000017907<br>ENST00000376017<br>ENST00000376026<br>GENSCAN00000000996<br>ENSESTT00000017908 | Homo sapiens TPX2, microtubule-associated, homolog (Xenopus laevis) (TPX2), mRNA.<br>cdna:known-ccds chromosome:NCBI36:20:29790565:29853264:1 gene:ENSG00000088325 CCDS13190.1<br>cdna:known chromosome:NCBI36:20:29790565:29853264:1 gene:ENSG00000088325<br>Homo sapiens hepatocellular carcinoma-associated antigen 90 (HCA90) mRNA, complete cds.<br><br>cdna:known chromosome:NCBI36:20:29790754:29853264:1 gene:ENSG00000088325<br>cdna:known chromosome:NCBI36:20:29790754:29853264:1 gene:ENSG00000088325<br>cdna:Genscan chromosome:NCBI36:20:29808941:29852544:1 |

| | | | | | | | | | |
|---|---|---|---|---|---|---|---|---|---|
| | | | | | | | | AK093379 | Homo sapiens cDNA FLJ36060 fis, clone TESTI2018521, moderately similar to Targeting protein for Xklp2; TPX2. |
| 3881787 | 968215 | 3881786 | core | 30259356 | 30259385 | 20 | + | ENST00000375749<br>NM_172236<br>ENST00000375730<br>ENST00000278989<br>ENSESTT00000017919<br>ENSESTT00000017921<br>GENSCAN00000064538<br>ENSESTT00000017920<br>NM_015352<br>ENST00000217326 | cdna:known-ccds chromosome:NCBI36:20:30259344:30290131:1 gene:ENSG00000101346 CCDS13198.1<br>Homo sapiens protein O-fucosyltransferase 1 (POFUT1), transcript variant 2, mRNA.<br>cdna:known-ccds chromosome:NCBI36:20:30259357:30269387:1 gene:ENSG00000101346 CCDS13199.1<br>cdna:known chromosome:NCBI36:20:30259357:30269386:1 gene:ENSG00000101346<br><br><br>cdna:Genscan chromosome:NCBI36:20:30259406:30286125:1<br><br>Homo sapiens protein O-fucosyltransferase 1 (POFUT1), transcript variant 1, mRNA.<br>cdna:known chromosome:NCBI36:20:30259357:30290125:1 gene:ENSG00000101346 |
| 3882057 | 968362 | 3882012 | core | 30852810 | 30852877 | 20 | + | NM_175850<br>GENSCAN00000021619<br>ENST00000201963<br>NM_175848<br>NM_175849<br>NM_006892<br>AB208880<br>ENSESTT00000026305<br>ENSESTT00000026306<br>ENSESTT00000026307<br>ENSESTT00000026308<br>ENSESTT00000026309<br>ENSESTT00000026310<br>ENSESTT00000026311<br>ENSESTT00000026312<br>ENST00000328111<br>ENST00000353855<br>ENST00000348286<br>ENST00000344505<br>ENST00000375621<br>ENST00000375624<br>ENST00000375623<br>ENSESTT00000026313<br>ENSESTT00000026314<br>ENSESTT00000026315<br>ENSESTT00000026316<br>ENSESTT00000026317<br>ENSESTT00000026318<br>ENSESTT00000026319<br>ENSESTT00000026320<br>ENSESTT00000026321<br>ENSESTT00000026322<br>ENSESTT00000026323<br>ENSESTT00000026324<br>ENSESTT00000026325<br>ENSESTT00000026326<br>ENSESTT00000026327 | Homo sapiens DNA (cytosine-5-)-methyltransferase 3 beta (DNMT3B), transcript variant 6, mRNA.<br>cdna:Genscan chromosome:NCBI36:20:30827013:30859370:1<br>cdna:known-ccds chromosome:NCBI36:20:30831319:30860822:1 gene:ENSG00000088305 CCDS13204.1<br>Homo sapiens DNA (cytosine-5-)-methyltransferase 3 beta (DNMT3B), transcript variant 2, mRNA.<br>Homo sapiens DNA (cytosine-5-)-methyltransferase 3 beta (DNMT3B), transcript variant 3, mRNA.<br>Homo sapiens DNA (cytosine-5-)-methyltransferase 3 beta (DNMT3B), transcript variant 1, mRNA.<br>Homo sapiens mRNA for DNA cytosine-5 methyltransferase 3 beta isoform 6 variant protein.<br><br><br><br><br><br><br><br><br>cdna:known-ccds chromosome:NCBI36:20:30813852:30860822:1 gene:ENSG00000088305 CCDS13205.1<br>cdna:known-ccds chromosome:NCBI36:20:30813852:30860822:1 gene:ENSG00000088305 CCDS13206.1<br>cdna:known-ccds chromosome:NCBI36:20:30813852:30860822:1 gene:ENSG00000088305 CCDS13207.1<br>cdna:known chromosome:NCBI36:20:30813852:30860822:1 gene:ENSG00000088305<br>cdna:known chromosome:NCBI36:20:30814012:30860823:1 gene:ENSG00000088305<br>cdna:known chromosome:NCBI36:20:30814012:30860823:1 gene:ENSG00000088305<br>cdna:known chromosome:NCBI36:20:30814012:30860823:1 gene:ENSG00000088305 |
| 3883068 | 968996 | 3883064 | core | 32934273 | 32934346 | 20 | + | ENSESTT00000052312<br>NM_139274<br>NM_018677<br>ENSESTT00000052308 | <br>Homo sapiens acyl-CoA synthetase short-chain family member 2 (ACSS2), transcript variant 2, mRNA.<br>Homo sapiens acyl-CoA synthetase short-chain family member 2 (ACSS2), transcript variant 1, mRNA. |

| | | | | | | | | | |
|---|---|---|---|---|---|---|---|---|---|
| | | | | | | | | ENSESTT00000052309<br>ENSESTT00000052310<br>ENSESTT00000052311<br>ENST00000336325<br>ENST00000360596<br>ENST00000357483<br>ENST00000374703<br>ENST00000374698<br>ENST00000374691<br>ENST00000253382<br>ENST00000374693<br>GENSCAN00000066597 | <br><br><br>cdna:known-ccds chromosome:NCBI36:20:32926502:32979426:1 gene:ENSG00000131069 CCDS13244.1<br>cdna:known-ccds chromosome:NCBI36:20:32928036:32979423:1 gene:ENSG00000131069 CCDS13243.1<br>cdna:known chromosome:NCBI36:20:32926502:32979423:1 gene:ENSG00000131069<br>cdna:known chromosome:NCBI36:20:32928054:32979426:1 gene:ENSG00000131069<br>cdna:known chromosome:NCBI36:20:32928078:32979426:1 gene:ENSG00000131069<br>cdna:known chromosome:NCBI36:20:32928082:32979426:1 gene:ENSG00000131069<br>cdna:novel chromosome:NCBI36:20:32928036:32979423:1 gene:ENSG00000131069<br>cdna:novel chromosome:NCBI36:20:32928078:32979424:1 gene:ENSG00000131069<br>cdna:Genscan chromosome:NCBI36:20:32928110:32972108:1 |
| 3883326 | 969162 | 3883309 | core | 33517292 | 33517367 | 20 | + | NM_007186<br>ENST00000358645<br>NM_001035518<br>ENST00000356095<br>ENST00000342580<br>ENSESTT00000052321<br>ENSESTT00000052322<br>BC001433<br>BC071869<br>ENSESTT00000052320<br>GENSCAN00000042782<br>ENSESTT00000052324<br>ENSESTT00000052325<br>ENSESTT00000052326<br>ENSESTT00000052327<br>ENSESTT00000052328 | Homo sapiens centrosomal protein 250kDa (CEP250), transcript variant 1, mRNA.<br>cdna:known chromosome:NCBI36:20:33506564:33563218:1 gene:ENSG00000126001<br>Homo sapiens centrosomal protein 250kDa (CEP250), transcript variant 2, mRNA.<br>cdna:known-ccds chromosome:NCBI36:20:33506564:33563216:1 gene:ENSG00000126001 CCDS13255.1<br>cdna:known chromosome:NCBI36:20:33506564:33563216:1 gene:ENSG00000126001<br><br><br>Homo sapiens centrosomal protein 250kDa, mRNA (cDNA clone IMAGE:3138798), complete cds.<br>Homo sapiens centrosomal protein 250kDa, mRNA (cDNA clone IMAGE:4892243), complete cds.<br><br>cdna:Genscan chromosome:NCBI36:20:33494488:33562869:1 |
| 3886210 | 970941 | 3886179 | core | 41708990 | 41709023 | 20 | + | ENSESTT00000049597<br>ENST00000373039<br>GENSCAN00000035656<br>NM_016004<br>ENSESTT00000049599<br>ENST00000373030<br>ENST00000262601 | <br>cdna:known chromosome:NCBI36:20:41652985:41709350:1 gene:ENSG00000101052<br>cdna:Genscan chromosome:NCBI36:20:41675904:41709037:1<br>Homo sapiens intraflagellar transport 52 homolog (Chlamydomonas) (IFT52), mRNA.<br><br>cdna:known chromosome:NCBI36:20:41653044:41709276:1 gene:ENSG00000101052<br>cdna:known chromosome:NCBI36:20:41652991:41709275:1 gene:ENSG00000101052 |
| 3887052 | 971452 | 3887049 | core | 43874845 | 43874883 | 20 | + | NM_181802<br>ENST00000372579<br>NM_007019<br>NM_181800<br>NM_181801<br>ENSESTT00000048448<br>ENSESTT00000048449<br>ENST00000356455<br>ENST00000352551<br>ENST00000343198<br>ENST00000372568<br>GENSCAN00000027336 | Homo sapiens ubiquitin-conjugating enzyme E2C (UBE2C), transcript variant 5, mRNA.<br>cdna:known-ccds chromosome:NCBI36:20:43874662:43878999:1 gene:ENSG00000175063 CCDS13374.1<br>Homo sapiens ubiquitin-conjugating enzyme E2C (UBE2C), transcript variant 1, mRNA.<br>Homo sapiens ubiquitin-conjugating enzyme E2C (UBE2C), transcript variant 3, mRNA.<br>Homo sapiens ubiquitin-conjugating enzyme E2C (UBE2C), transcript variant 4, mRNA.<br><br><br>cdna:known-ccds chromosome:NCBI36:20:43874710:43879003:1 gene:ENSG00000175063 CCDS13370.1<br>cdna:known-ccds chromosome:NCBI36:20:43874742:43878999:1 gene:ENSG00000175063 CCDS13372.1<br>cdna:known chromosome:NCBI36:20:43874742:43878999:1 gene:ENSG00000175063<br>cdna:known chromosome:NCBI36:20:43875098:43878884:1 gene:ENSG00000175063<br>cdna:Genscan chromosome:NCBI36:20:43854051:43878813:1 |
| 3887066 | 971457 | 3887049 | core | 43877924 | 43877956 | 20 | + | NM_181803<br>NM_181799<br>NM_007019<br>NM_181800<br>ENST00000243893<br>ENST00000335046 | Homo sapiens ubiquitin-conjugating enzyme E2C (UBE2C), transcript variant 6, mRNA.<br>Homo sapiens ubiquitin-conjugating enzyme E2C (UBE2C), transcript variant 2, mRNA.<br>Homo sapiens ubiquitin-conjugating enzyme E2C (UBE2C), transcript variant 1, mRNA.<br>Homo sapiens ubiquitin-conjugating enzyme E2C (UBE2C), transcript variant 3, mRNA.<br>cdna:known-ccds chromosome:NCBI36:20:43874662:43879003:1 gene:ENSG00000175063 CCDS13373.1<br>cdna:known-ccds chromosome:NCBI36:20:43874662:43878999:1 gene:ENSG00000175063 CCDS13371.1 |

| | | | | | | | | | |
|---|---|---|---|---|---|---|---|---|---|
| | | | | | | | | NM_181801<br>ENSESTT00000048448<br>ENSESTT00000048449<br>ENST00000356455<br>ENST00000352551<br>ENST00000343198<br>ENST00000372568<br>NM_181802<br>BC032677<br>ENST00000372579<br>GENSCAN00000027336 | Homo sapiens ubiquitin-conjugating enzyme E2C (UBE2C), transcript variant 4, mRNA.<br><br><br>cdna:known-ccds chromosome:NCBI36:20:43874710:43879003:1 gene:ENSG00000175063 CCDS13370.1<br>cdna:known-ccds chromosome:NCBI36:20:43874742:43878999:1 gene:ENSG00000175063 CCDS13372.1<br>cdna:known chromosome:NCBI36:20:43874742:43878999:1 gene:ENSG00000175063<br>cdna:known chromosome:NCBI36:20:43875098:43878884:1 gene:ENSG00000175063<br>Homo sapiens ubiquitin-conjugating enzyme E2C (UBE2C), transcript variant 5, mRNA.<br>Homo sapiens ubiquitin-conjugating enzyme E2C, mRNA (cDNA clone IMAGE:5574059), partial cds.<br>cdna:known-ccds chromosome:NCBI36:20:43874662:43878999:1 gene:ENSG00000175063 CCDS13374.1<br>cdna:Genscan chromosome:NCBI36:20:43854051:43878813:1 |
| 3891321 | 974030 | 3891278 | core | 57003398 | 57003434 | 20 | + | AK127431<br>ENSESTT00000024233<br>ENST00000371062<br>ENST00000217129<br>AJ238374<br>NM_198976<br>ENST00000344018<br>AJ238376<br>ENSESTT00000024237<br>ENSESTT00000024239 | Homo sapiens cDNA FLJ45523 fis, clone BRTHA2026071.<br><br>cdna:known chromosome:NCBI36:20:56989725:57003583:1 gene:ENSG00000101158<br>cdna:known chromosome:NCBI36:20:56989725:57003583:1 gene:ENSG00000101158<br>Homo sapiens mRNA for putative protein TH1, partial, clone IMAGE ID 785447.<br>Homo sapiens TH1-like (Drosophila) (TH1L), transcript variant 1, mRNA.<br>cdna:known-ccds chromosome:NCBI36:20:56989706:57003581:1 gene:ENSG00000101158 CCDS13473.1<br>Homo sapiens mRNA for putative protein TH1, partial, clone IMAGE ID 255931. |
| 3893541 | 975418 | 3893520 | core | 61769300 | 61769345 | 20 | + | NM_032957<br>NM_016434<br>ENSESTT00000057010<br>ENST00000318100<br>ENST00000370006<br>ENST00000355223<br>ENST00000342852<br>ENSESTT00000057011<br>ENSESTT00000057012<br>ENST00000370018<br>AB029011<br>ENST00000356810<br>GENSCAN00000045781<br>ENSESTT00000057013<br>ENST00000360203<br>AK131105<br>AL080127<br>ENST00000370003<br>ENSESTT00000057014<br>ENSESTT00000057015 | Homo sapiens regulator of telomere elongation helicase 1 (RTEL1), transcript variant 2, mRNA.<br>Homo sapiens regulator of telomere elongation helicase 1 (RTEL1), transcript variant 1, mRNA.<br><br>cdna:known-ccds chromosome:NCBI36:20:61760091:61800495:1 gene:ENSG00000026036 CCDS13530.1<br>cdna:known-ccds chromosome:NCBI36:20:61760091:61800495:1 gene:ENSG00000026036 CCDS13532.1<br>cdna:known chromosome:NCBI36:20:61760091:61800495:1 gene:ENSG00000026036<br>cdna:known chromosome:NCBI36:20:61760091:61800495:1 gene:ENSG00000026036<br><br><br>cdna:known-ccds chromosome:NCBI36:20:61759607:61798050:1 gene:ENSG00000026036 CCDS13531.1<br>Homo sapiens mRNA for KIAA1088 protein, partial cds.<br>cdna:known chromosome:NCBI36:20:61761200:61800481:1 gene:ENSG00000026036<br>cdna:Genscan chromosome:NCBI36:20:61755129:61797490:1<br><br>cdna:known chromosome:NCBI36:20:61788168:61800481:1 gene:ENSG00000026036<br>Homo sapiens mRNA for FLJ00362 protein.<br>Homo sapiens mRNA; cDNA DKFZp434C013 (from clone DKFZp434C013).<br>cdna:novel chromosome:NCBI36:20:61791763:61798045:1 gene:ENSG00000026036 |
| 3901094 | 979950 | 3901085 | core | 23054639 | 23055151 | 20 | - | NM_182535 | Homo sapiens hypothetical protein LOC200261 (LOC200261), mRNA. |
| 3901398 | 980116 | 3901387 | core | 23753895 | 23753960 | 20 | - | NM_001322<br>ENST00000304725<br>ENST00000376882<br>ENSESTT00000048274<br>GENSCAN00000012616 | Homo sapiens cystatin SA (CST2), mRNA.<br>cdna:known-ccds chromosome:NCBI36:20:23752406:23755368:-1 gene:ENSG00000170369 CCDS13161.1<br>cdna:known chromosome:NCBI36:20:23752409:23755312:-1 gene:ENSG00000170369<br><br>cdna:Genscan chromosome:NCBI36:20:23752657:23755297:-1 |
| 3901399 | 980117 | 3901387 | core | 23755084 | 23755218 | 20 | - | NM_001322<br>ENST00000304725<br>GENSCAN00000012616<br>ENST00000376882<br>ENSESTT00000048274 | Homo sapiens cystatin SA (CST2), mRNA.<br>cdna:known-ccds chromosome:NCBI36:20:23752406:23755368:-1 gene:ENSG00000170369 CCDS13161.1<br>cdna:Genscan chromosome:NCBI36:20:23752657:23755297:-1<br>cdna:known chromosome:NCBI36:20:23752409:23755312:-1 gene:ENSG00000170369 |

| | | | | | | | | | |
|---|---|---|---|---|---|---|---|---|---|
| 3904586 | 982002 | 3904566 | core | 34833249 | 34833275 | 20 | - | NM_024918<br>BC026011<br>ENST00000373734<br>ENST00000339439<br>ENST00000373733<br>BC035821<br>ENSESTT00000008378<br>ENST00000373750<br>ENST00000373752<br>ENST00000373745<br>ENST00000373743<br>ENST00000373740<br>GENSCAN00000005256<br>ENSESTT00000008380<br>ENSESTT00000008379 | Homo sapiens chromosome 20 open reading frame 172 (C20orf172), mRNA.<br>Homo sapiens chromosome 20 open reading frame 172, mRNA (cDNA clone MGC:32987 IMAGE:4827570), complete cds.<br>cdna:known chromosome:NCBI36:20:34813890:34833275:-1 gene:ENSG00000149636<br>cdna:known chromosome:NCBI36:20:34813890:34835614:-1 gene:ENSG00000149636<br>cdna:known chromosome:NCBI36:20:34813890:34832983:-1 gene:ENSG00000149636<br>Homo sapiens chromosome 20 open reading frame 172, mRNA (cDNA clone IMAGE:5553020), with apparent retained intron.<br><br>cdna:known-ccds chromosome:NCBI36:20:34813612:34835614:-1 gene:ENSG00000149636 CCDS13286.1<br>cdna:known chromosome:NCBI36:20:34813612:34835615:-1 gene:ENSG00000149636<br>cdna:known chromosome:NCBI36:20:34813612:34835614:-1 gene:ENSG00000149636<br>cdna:known chromosome:NCBI36:20:34813612:34835563:-1 gene:ENSG00000149636<br>cdna:known chromosome:NCBI36:20:34813612:34835537:-1 gene:ENSG00000149636<br>cdna:Genscan chromosome:NCBI36:20:34798724:34835718:-1 |
| 3907112 | 983589 | 3907111 | core | 43004185 | 43004238 | 20 | - | ENST00000372813<br>NM_006809<br>ENST00000216891<br>ENST00000372810 | cdna:known-ccds chromosome:NCBI36:20:43004185:43022541:-1 gene:ENSG00000025772 CCDS13340.1<br>Homo sapiens translocase of outer mitochondrial membrane 34 (TOMM34), nuclear gene encoding mitochondrial protein, mRNA.<br>cdna:known chromosome:NCBI36:20:43004186:43022528:-1 gene:ENSG00000025772<br>cdna:known chromosome:NCBI36:20:43004186:43022528:-1 gene:ENSG00000025772 |
| 3907126 | 983599 | 3907111 | core | 43022401 | 43022509 | 20 | - | NM_006809<br>ENST00000372813<br>GENSCAN00000052376<br>ENST00000216891<br>ENST00000372810<br>ENSESTT00000012877<br>ENSESTT00000012876 | Homo sapiens translocase of outer mitochondrial membrane 34 (TOMM34), nuclear gene encoding mitochondrial protein, mRNA.<br>cdna:known-ccds chromosome:NCBI36:20:43004185:43022541:-1 gene:ENSG00000025772 CCDS13340.1<br>cdna:Genscan chromosome:NCBI36:20:43005164:43022604:-1<br>cdna:known chromosome:NCBI36:20:43004186:43022528:-1 gene:ENSG00000025772<br>cdna:known chromosome:NCBI36:20:43004186:43022528:-1 gene:ENSG00000025772 |
| 3909778 | 985242 | 3909777 | core | 49834038 | 49834189 | 20 | - | NM_020436<br>ENST00000217086<br>ENST00000371539<br>GENSCAN00000034662 | Homo sapiens sal-like 4 (Drosophila) (SALL4), mRNA.<br>cdna:known-ccds chromosome:NCBI36:20:49833988:49852421:-1 gene:ENSG00000101115 CCDS13438.1<br>cdna:known chromosome:NCBI36:20:49833988:49852421:-1 gene:ENSG00000101115<br>cdna:Genscan chromosome:NCBI36:20:49834211:49852354:-1 |
| 3910796 | 985851 | 3910785 | core | 54381958 | 54381982 | 20 | - | NM_198436<br>NM_198434<br>NM_198437<br>NM_198435<br>NM_198433<br>NM_003600<br>ENST00000347343<br>ENST00000371356<br>ENST00000371353<br>ENST00000312783<br>NR_001587<br>ENSESTT00000006498<br>ENSESTT00000006499<br>GENSCAN00000011605 | Homo sapiens aurora kinase A (AURKA), transcript variant 5, mRNA.<br>Homo sapiens aurora kinase A (AURKA), transcript variant 3, mRNA.<br>Homo sapiens aurora kinase A (AURKA), transcript variant 6, mRNA.<br>Homo sapiens aurora kinase A (AURKA), transcript variant 4, mRNA.<br>Homo sapiens aurora kinase A (AURKA), transcript variant 1, mRNA.<br>Homo sapiens aurora kinase A (AURKA), transcript variant 2, mRNA.<br>cdna:known-ccds chromosome:NCBI36:20:54377852:54400758:-1 gene:ENSG00000087586 CCDS13451.1<br>cdna:known chromosome:NCBI36:20:54377852:54400659:-1 gene:ENSG00000087586<br>cdna:known chromosome:NCBI36:20:54377852:54400656:-1 gene:ENSG00000087586<br>cdna:known chromosome:NCBI36:20:54377853:54400682:-1 gene:ENSG00000087586<br>Homo sapiens serine/threonine kinase 6 pseudogene (STK6P) on chromosome 1.<br><br><br>cdna:Genscan chromosome:NCBI36:20:54378256:54402553:-1 |
| 3910807 | 985861 | 3910785 | core | 54400430 | 54400529 | 20 | - | NM_198433<br>ENST00000371353<br>ENST00000371356<br>NM_003600<br>ENST00000347343 | Homo sapiens aurora kinase A (AURKA), transcript variant 1, mRNA.<br>cdna:known chromosome:NCBI36:20:54377852:54400656:-1 gene:ENSG00000087586<br>cdna:known chromosome:NCBI36:20:54377852:54400659:-1 gene:ENSG00000087586<br>Homo sapiens aurora kinase A (AURKA), transcript variant 2, mRNA.<br>cdna:known-ccds chromosome:NCBI36:20:54377852:54400758:-1 gene:ENSG00000087586 CCDS13451.1 |

| | | | | | | | | | |
|---|---|---|---|---|---|---|---|---|---|
| 3910808 | 985862 | 3910785 | core | 54400659 | 54400758 | 20 | - | NM_198436<br>NM_198434<br>NM_198433<br>NM_198437<br>NM_198435<br>NM_003600<br>ENST00000347343<br>ENST00000371356<br>ENST00000371353<br>ENST00000312783<br>NR_001587<br>ENSESTT00000006498<br>ENSESTT00000006499<br>GENSCAN00000011605 | Homo sapiens aurora kinase A (AURKA), transcript variant 5, mRNA.<br>Homo sapiens aurora kinase A (AURKA), transcript variant 3, mRNA.<br>Homo sapiens aurora kinase A (AURKA), transcript variant 1, mRNA.<br>Homo sapiens aurora kinase A (AURKA), transcript variant 6, mRNA.<br>Homo sapiens aurora kinase A (AURKA), transcript variant 4, mRNA.<br>Homo sapiens aurora kinase A (AURKA), transcript variant 2, mRNA.<br>cdna:known-ccds chromosome:NCBI36:20:54377852:54400758:-1 gene:ENSG00000087586 CCDS13451.1<br>cdna:known chromosome:NCBI36:20:54377852:54400659:-1 gene:ENSG00000087586<br>cdna:known chromosome:NCBI36:20:54377852:54400656:-1 gene:ENSG00000087586<br>cdna:known chromosome:NCBI36:20:54377853:54400682:-1 gene:ENSG00000087586<br>Homo sapiens serine/threonine kinase 6 pseudogene (STK6P) on chromosome 1.<br><br><br>cdna:Genscan chromosome:NCBI36:20:54378256:54402553:-1 |
| 3913983 | 987787 | 3913960 | core | 61665614 | 61666117 | 20 | - | NM_001037335<br>NM_033405<br>AF517673<br>ENST00000370091<br>ENST00000252889<br>ENST00000358289<br>ENST00000338175<br>GENSCAN00000021520<br>ENST00000370082 | Homo sapiens peroxisomal proliferator-activated receptor A interacting complex 285 (PRIC285), transcript variant 1, mRNA.<br>Homo sapiens peroxisomal proliferator-activated receptor A interacting complex 285 (PRIC285), transcript variant 2, mRNA.<br>Homo sapiens peroxisomal proliferator-activated receptor A interacting complex-285 peptide (PRIC285) mRNA, complete cds.<br>cdna:known-ccds chromosome:NCBI36:20:61659883:61669871:-1 gene:ENSG00000130589 CCDS13527.1<br>cdna:known chromosome:NCBI36:20:61659884:61676033:-1 gene:ENSG00000130589<br>cdna:known chromosome:NCBI36:20:61659884:61676033:-1 gene:ENSG00000130589<br>cdna:known chromosome:NCBI36:20:61659908:61669856:-1 gene:ENSG00000130589<br>cdna:Genscan chromosome:NCBI36:20:61659170:61680020:-1<br>cdna:known chromosome:NCBI36:20:61671936:61676036:-1 gene:ENSG00000130589 |
| 3923244 | 993556 | 3923218 | core | 43932345 | 43932543 | 21 | + | NM_015056<br>ENST00000340648<br>ENSESTT00000018442<br>GENSCAN00000014033<br>AK124620<br>ENSESTT00000018443 | Homo sapiens KIAA0179 (KIAA0179), mRNA.<br>cdna:known chromosome:NCBI36:21:43903860:43940388:1 gene:ENSG00000160208<br><br>cdna:Genscan chromosome:NCBI36:21:43903974:43937692:1<br>Homo sapiens cDNA FLJ42629 fis, clone BRACE3019055. |
| 3923258 | 993566 | 3923257 | core | 43963420 | 43963506 | 21 | + | NM_003681<br>BC005825<br>ENST00000291565<br>ENST00000343528<br>ENSESTT00000033512<br>ENSESTT00000033514<br>ENSESTT00000033515<br>ENSESTT00000033516<br>ENST00000327574<br>AY303972<br>CR613269 | Homo sapiens pyridoxal (pyridoxine, vitamin B6) kinase (PDXK), mRNA.<br>Homo sapiens pyridoxal (pyridoxine, vitamin B6) kinase, mRNA (cDNA clone MGC:1687 IMAGE:2989843), complete cds.<br>cdna:known-ccds chromosome:NCBI36:21:43963406:44006608:1 gene:ENSG00000160209 CCDS13699.1<br>cdna:known chromosome:NCBI36:21:43963406:44006608:1 gene:ENSG00000160209<br><br><br><br><br>cdna:known-ccds chromosome:NCBI36:21:43973209:43983167:1 gene:ENSG00000160209 CCDS13700.1<br>Homo sapiens pyridoxal kinase mRNA, complete cds.<br>full-length cDNA clone CS0DI073YB04 of Placenta Cot 25-normalized of Homo sapiens (human). |
| 3924659 | 994465 | 3924573 | core | 46680272 | 46680407 | 21 | + | NM_006031<br>ENST00000359568<br>ENST00000337772<br>ENSESTT00000045653<br>AB007862<br>GENSCAN00000065183<br>GENSCAN00000030482<br>ENSESTT00000045654<br>GENSCAN00000019008 | Homo sapiens pericentrin (kendrin) (PCNT), mRNA.<br>cdna:known chromosome:NCBI36:21:46568483:46690106:1 gene:ENSG00000160299<br>cdna:known chromosome:NCBI36:21:46568483:46690106:1 gene:ENSG00000160299<br><br>Homo sapiens KIAA0402 mRNA, partial cds.<br>cdna:Genscan chromosome:NCBI36:21:46568571:46579110:1<br>cdna:Genscan chromosome:NCBI36:21:46590470:46621706:1<br><br>cdna:Genscan chromosome:NCBI36:21:46626037:46646825:1 |

| | | | | | | | | | |
|---|---|---|---|---|---|---|---|---|---|
| | | | | | | | | ENSESTT00000045656<br>GENSCAN00000039829<br>ENSESTT00000045657<br>ENSESTT00000045658<br>ENSESTT00000045659<br>ENSESTT00000045660<br>ENSESTT00000045661<br>ENSESTT00000045662 | cdna:Genscan chromosome:NCBI36:21:46655287:46687025:1 |
| 3930433 | 997986 | 3930360 | core | 35181149 | 35181263 | 21 | - | NM_001754<br>NM_001001890<br>S60998<br>ENSESTT00000044878<br>GENSCAN00000019438<br>ENSESTT00000044879<br>ENSESTT00000044881<br>ENST00000300305<br>ENST00000325074<br>ENST00000344691<br>GENSCAN00000065028<br>D43967<br>ENSESTT00000044880<br>ENST00000358356<br>ENST00000342083<br>ENSESTT00000044875<br>ENSESTT00000044876 | Homo sapiens runt-related transcription factor 1 (acute myeloid leukemia 1; aml1 oncogene) (RUNX1), transcript variant 1, mRNA.<br>Homo sapiens runt-related transcription factor 1 (acute myeloid leukemia 1; aml1 oncogene) (RUNX1), transcript variant 2, mRNA.<br>AML1=acute myeloid leukemia {alternatively spliced} [human, mRNA Partial, 894 nt].<br><br>cdna:Genscan chromosome:NCBI36:21:35128577:35181279:-1<br><br><br>cdna:known-ccds chromosome:NCBI36:21:35081975:35343511:-1 gene:ENSG00000159216 CCDS13639.1<br>cdna:known chromosome:NCBI36:21:35081975:35183904:-1 gene:ENSG00000159216<br>cdna:known chromosome:NCBI36:21:35086146:35182857:-1 gene:ENSG00000159216<br>cdna:Genscan chromosome:NCBI36:21:35086302:35113505:-1<br>Homo sapiens mRNA for AML1a protein, complete cds.<br><br>cdna:known chromosome:NCBI36:21:35115444:35182857:-1 gene:ENSG00000159216<br>cdna:known chromosome:NCBI36:21:35150580:35182857:-1 gene:ENSG00000159216 |
| 3931206 | 998466 | 3931112 | core | 37241521 | 37241951 | 21 | - | D87328<br>GENSCAN00000060242<br>NM_000411<br>ENSESTT00000025942<br>ENSESTT00000025943<br>ENST00000336648 | Homo sapiens mRNA for HCS, complete cds.<br>cdna:Genscan chromosome:NCBI36:21:37241522:37260808:-1<br>Homo sapiens holocarboxylase synthetase (biotin-(proprionyl-Coenzyme A-carboxylase (ATP-hydrolysing)) ligase) (HLCS), mRNA.<br><br><br>cdna:known-ccds chromosome:NCBI36:21:37045066:37284373:-1 gene:ENSG00000159267 CCDS13647.1 |
| 3933551 | 999946 | 3933550 | core | 42639608 | 42639700 | 21 | - | NM_005423<br>X51698<br>ENST00000291526<br>BC032820<br>ENSESTT00000046189<br>GENSCAN00000054755 | Homo sapiens trefoil factor 2 (spasmolytic protein 1) (TFF2), mRNA.<br>H.sapiens spasmolytic polypeptide (SP) mRNA.<br>cdna:known-ccds chromosome:NCBI36:21:42639538:42644176:-1 gene:ENSG00000160181 CCDS13684.1<br>Homo sapiens trefoil factor 2 (spasmolytic protein 1), mRNA (cDNA clone MGC:45237 IMAGE:5184770), complete cds.<br><br>cdna:Genscan chromosome:NCBI36:21:42640332:42644135:-1 |
| 3934252 | 1000338 | 3934245 | core | 44018516 | 44018615 | 21 | - | NM_000100<br>CR591371<br>ENSESTT00000033557<br>GENSCAN00000025063<br>ENST00000291568 | Homo sapiens cystatin B (stefin B) (CSTB), mRNA.<br>full-length cDNA clone CS0DF018YI07 of Fetal brain of Homo sapiens (human).<br><br>cdna:Genscan chromosome:NCBI36:21:44018511:44020578:-1<br>cdna:known-ccds chromosome:NCBI36:21:44016826:44020585:-1 gene:ENSG00000160213 CCDS13701.1 |
| 3939212 | 1003160 | 3939183 | core | 21956216 | 21956249 | 22 | + | NM_021574<br>NM_004327<br>ENST00000305877<br>ENST00000359540<br>ENST00000292697<br>ENST00000290956<br>AJ298916 | Homo sapiens breakpoint cluster region (BCR), transcript variant 2, mRNA.<br>Homo sapiens breakpoint cluster region (BCR), transcript variant 1, mRNA.<br>cdna:known-ccds chromosome:NCBI36:22:21852552:21990224:1 gene:ENSG00000186716 CCDS13806.1<br>cdna:known-ccds chromosome:NCBI36:22:21852552:21990224:1 gene:ENSG00000186716 CCDS13807.1<br>cdna:known chromosome:NCBI36:22:21852552:21990224:1 gene:ENSG00000186716<br>cdna:known chromosome:NCBI36:22:21852552:21990224:1 gene:ENSG00000186716<br>Homo sapiens partial mRNA for BCR/FGFR1 chimaeric fusion protein. |

| | | | | | | | | | |
|---|---|---|---|---|---|---|---|---|---|
| | | | | | | | | ENSESTT00000003871<br>ENSESTT00000003872<br>ENST00000334149<br>ENST00000347173<br>GENSCAN00000013511<br>AY536249<br>AF192533<br>AF487522<br>BC031568<br>AK122842 | <br><br>cdna:known chromosome:NCBI36:22:21870438:21990224:1 gene:ENSG00000186716<br>cdna:known chromosome:NCBI36:22:21926725:21990224:1 gene:ENSG00000186716<br>cdna:Genscan chromosome:NCBI36:22:21922436:21987709:1<br>Homo sapiens isolate BCRcml2K23I072001 breakpoint cluster region protein (BCR) mRNA, partial cds.<br>Homo sapiens BCR-ABL fusion protein (BCR-ABL fusion) mRNA, partial cds.<br>Homo sapiens BCRe18/ABL1e3 fusion protein (BCR/ABL fusion) mRNA, partial cds.<br>Homo sapiens breakpoint cluster region, mRNA (cDNA clone IMAGE:5192593), with apparent retained intron.<br>Homo sapiens cDNA FLJ16453 fis, clone BRAWH3003019, highly similar to Breakpoint cluster region protein (EC 2.7.1.-). |
| 3939477 | 1003307 | 3939470 | core | 22451502 | 22451555 | 22 | + | NM_005940<br>AK125911<br>GENSCAN00000024043<br>ENSESTT00000003878<br>ENSESTT00000003879<br>ENST00000215743 | Homo sapiens matrix metallopeptidase 11 (stromelysin 3) (MMP11), mRNA.<br>Homo sapiens cDNA FLJ43923 fis, clone TESTI4012448, highly similar to Stromelysin-3 precursor (EC 3.4.24.-).<br>cdna:Genscan chromosome:NCBI36:22:22445058:22475609:1<br><br><br>cdna:known-ccds chromosome:NCBI36:22:22445036:22456502:1 gene:ENSG00000099953 CCDS13816.1 |
| 3939491 | 1003311 | 3939470 | core | 22455598 | 22455653 | 22 | + | NM_005940<br>AK125911<br>CR626443<br>ENSESTT00000003878<br>GENSCAN00000024043<br>ENSESTT00000003879<br>ENST00000215743 | Homo sapiens matrix metallopeptidase 11 (stromelysin 3) (MMP11), mRNA.<br>Homo sapiens cDNA FLJ43923 fis, clone TESTI4012448, highly similar to Stromelysin-3 precursor (EC 3.4.24.-).<br>full-length cDNA clone CS0DI013YL21 of Placenta Cot 25-normalized of Homo sapiens (human).<br><br>cdna:Genscan chromosome:NCBI36:22:22445058:22475609:1<br><br>cdna:known-ccds chromosome:NCBI36:22:22445036:22456502:1 gene:ENSG00000099953 CCDS13816.1 |
| 3950876 | 1010173 | 3950872 | core | 49301754 | 49301783 | 22 | + | NM_152299<br>NM_014551<br>ENST00000380768<br>ENST00000299821<br>ENST00000361482<br>GENSCAN00000046424<br>ENSESTT00000044456<br>ENSESTT00000044457<br>ENSESTT00000044458 | Homo sapiens kleisin beta (hCAP-H2), transcript variant 2, mRNA.<br>Homo sapiens kleisin beta (hCAP-H2), transcript variant 1, mRNA.<br>cdna:known-ccds chromosome:NCBI36:22:49293539:49308670:1 gene:ENSG00000025770 CCDS14094.1<br>cdna:known chromosome:NCBI36:22:49293532:49308753:1 gene:ENSG00000025770<br>cdna:known chromosome:NCBI36:22:49293532:49308753:1 gene:ENSG00000025770<br>cdna:Genscan chromosome:NCBI36:22:49293633:49308670:1 |
| 3952847 | 1011325 | 3952825 | core | 18188752 | 18188841 | 22 | - | NM_053004<br>AB051432<br>CR604918<br>ENSESTT00000054229<br>ENST00000329517<br>GENSCAN00000059338 | Homo sapiens guanine nucleotide binding protein (G protein), beta polypeptide 1-like (GNB1L), mRNA.<br>Homo sapiens mRNA for KIAA1645 protein, partial cds.<br>full-length cDNA clone CS0DI021YF08 of Placenta Cot 25-normalized of Homo sapiens (human).<br><br>cdna:known-ccds chromosome:NCBI36:22:18150747:18222403:-1 gene:ENSG00000185838 CCDS13768.1<br>cdna:Genscan chromosome:NCBI36:22:18166274:18188898:-1 |
| 3952863 | 1011332 | 3952825 | core | 18219249 | 18219428 | 22 | - | NM_024627<br>ENST00000328554<br>NM_053004<br>AB051432<br>BC008696<br>ENSESTT00000054227<br>ENST00000329517 | Homo sapiens hypothetical protein FLJ21125 (FLJ21125), mRNA.<br>cdna:known-ccds chromosome:NCBI36:22:18213671:18222339:-1 gene:ENSG00000185838 CCDS13769.1<br>Homo sapiens guanine nucleotide binding protein (G protein), beta polypeptide 1-like (GNB1L), mRNA.<br>Homo sapiens mRNA for KIAA1645 protein, partial cds.<br>Homo sapiens hypothetical protein FLJ21125, mRNA (cDNA clone IMAGE:2820627), with apparent retained intron.<br><br>cdna:known-ccds chromosome:NCBI36:22:18150747:18222403:-1 gene:ENSG00000185838 CCDS13768.1 |
| 3959404 | 1015234 | 3959388 | core | 34930680 | 34930818 | 22 | - | NM_145660<br>NM_030643<br>ENSESTT00000045170<br>ENST00000332987<br>ENST00000352371 | Homo sapiens apolipoprotein L, 4 (APOL4), transcript variant b, mRNA.<br>Homo sapiens apolipoprotein L, 4 (APOL4), transcript variant a, mRNA.<br><br>cdna:known chromosome:NCBI36:22:34917069:34930825:-1 gene:ENSG00000100336<br>cdna:known chromosome:NCBI36:22:34917069:34930825:-1 gene:ENSG00000100336 |

| | | | | | | | | | |
|---|---|---|---|---|---|---|---|---|---|
| | | | | | | | | GENSCAN00000024540<br>BC006276<br>ENST00000328429 | cdna:Genscan chromosome:NCBI36:22:34917069:34928028:-1<br>Homo sapiens apolipoprotein L, 4, mRNA (cDNA clone IMAGE:3956995), complete cds.<br>cdna:known chromosome:NCBI36:22:34923448:34930735:-1 gene:ENSG00000100336 |
| 3963026 | 1017383 | 3962997 | core | 42303510 | 42303664 | 22 | - | NM_022785<br>NM_198856<br>ENST00000262726<br>ENST00000358439<br>ENSESTT00000034755<br>AL355841<br>GENSCAN00000062211<br>ENSESTT00000034754<br>GENSCAN00000042351<br>AB051459<br>GENSCAN00000067168<br>ENSESTT00000034753<br>AK058069<br>GENSCAN00000059584<br>Y10776<br>ENSESTT00000034752<br>ENST00000356087<br>GENSCAN00000027033<br>ENSESTT00000034751 | Homo sapiens CAP-binding protein complex interacting protein 1 (FLJ23588), transcript variant 1, mRNA.<br>Homo sapiens CAP-binding protein complex interacting protein 1 (FLJ23588), transcript variant 2, mRNA.<br>cdna:known-ccds chromosome:NCBI36:22:42255987:42539451:-1 gene:ENSG00000186976 CCDS14049.1<br>cdna:known-ccds chromosome:NCBI36:22:42255987:42539451:-1 gene:ENSG00000186976 CCDS14050.1<br><br>Novel human gene mapping to chomosome 22.<br>cdna:Genscan chromosome:NCBI36:22:42256066:42269330:-1<br><br>cdna:Genscan chromosome:NCBI36:22:42282082:42288815:-1<br>Homo sapiens mRNA for KIAA1672 protein, partial cds.<br>cdna:Genscan chromosome:NCBI36:22:42303510:42411068:-1<br><br>Homo sapiens cDNA FLJ25340 fis, clone TST00961, highly similar to H.sapiens mRNA for CAP-binding protein complex interacting protein 1.<br>cdna:Genscan chromosome:NCBI36:22:42438688:42472230:-1<br>H.sapiens mRNA for CAP-binding protein complex interacting protein 1.<br><br>cdna:known chromosome:NCBI36:22:42444022:42539451:-1 gene:ENSG00000186976<br>cdna:Genscan chromosome:NCBI36:22:42500105:42505568:-1 |
| 3965891 | 1019166 | 3965833 | core | 49249896 | 49250032 | 22 | - | NM_002972<br>ENSESTT00000044503<br>ENSESTT00000044507<br>CR610158<br>ENST00000337034<br>ENST00000380817<br>ENST00000348911<br>GENSCAN00000050155<br>ENSESTT00000044505<br>ENSESTT00000044506<br>ENSESTT00000044504<br>ENST00000356279<br>ENSESTT00000044502 | Homo sapiens SET binding factor 1 (SBF1), transcript variant 1, mRNA.<br><br><br>full-length cDNA clone CS0DF005YO14 of Fetal brain of Homo sapiens (human).<br>cdna:known chromosome:NCBI36:22:49232437:49260330:-1 gene:ENSG00000100241<br>cdna:known chromosome:NCBI36:22:49232437:49260135:-1 gene:ENSG00000100241<br>cdna:known chromosome:NCBI36:22:49232437:49260135:-1 gene:ENSG00000100241<br>cdna:Genscan chromosome:NCBI36:22:49232437:49260307:-1<br><br><br><br>cdna:known chromosome:NCBI36:22:49232118:49260135:-1 gene:ENSG00000100241 |
| 3970711 | 1022164 | 3970642 | core | 18578452 | 18578612 | X | + | NM_001037343<br>ENSESTT00000012130<br>ENST00000379989<br>NM_003159<br>BC010966<br>ENSESTT00000012128<br>ENST00000379996<br>ENST00000262697<br>ENST00000379991<br>GENSCAN00000038339<br>ENSESTT00000012129 | Homo sapiens cyclin-dependent kinase-like 5 (CDKL5), transcript variant II, mRNA.<br><br>cdna:known chromosome:NCBI36:X:18370229:18581666:1 gene:ENSG00000008086<br>Homo sapiens cyclin-dependent kinase-like 5 (CDKL5), transcript variant I, mRNA.<br>Homo sapiens cyclin-dependent kinase-like 5, mRNA (cDNA clone IMAGE:4294753), with apparent retained intron.<br><br>cdna:known-ccds chromosome:NCBI36:X:18353624:18581670:1 gene:ENSG00000008086 CCDS14186.1<br>cdna:known chromosome:NCBI36:X:18353624:18581585:1 gene:ENSG00000008086<br>cdna:novel chromosome:NCBI36:X:18353624:18581585:1 gene:ENSG00000008086<br>cdna:Genscan chromosome:NCBI36:X:18478180:18556798:1 |
| 3973512 | 1023911 | 3973505 | core | 35869321 | 35869418 | X | + | NM_152632<br>ENSESTT00000034974<br>ENST00000361984<br>ENST00000297866<br>ENST00000378663 | Homo sapiens chromosome X open reading frame 22 (CXorf22), mRNA.<br><br>cdna:known-ccds chromosome:NCBI36:X:35847806:35903293:1 gene:ENSG00000165164 CCDS14237.1<br>cdna:known chromosome:NCBI36:X:35847806:35918185:1 gene:ENSG00000165164<br>cdna:known chromosome:NCBI36:X:35847819:35918190:1 gene:ENSG00000165164 |

| | | | | | | | | | |
|---|---|---|---|---|---|---|---|---|---|
| | | | | | | | | GENSCAN00000024815<br>ENSESTT00000034975 | cdna:Genscan chromosome:NCBI36:X:35847838:35966298:1 |
| 3980607 | 1028096 | 3980560 | core | 69552055 | 69552080 | X | + | | |
| 3982251 | 1029057 | 3982242 | core | 75311715 | 75311818 | X | + | NM_016500<br>ENST00000373358<br>ENST00000246127<br>AF151079<br>ENSESTT00000014103<br>ENST00000373357<br>GENSCAN00000065155 | Homo sapiens chromosome X open reading frame 26 (CXorf26), mRNA.<br>cdna:known-ccds chromosome:NCBI36:X:75309173:75314442:1 gene:ENSG00000102390 CCDS14432.1<br>cdna:known chromosome:NCBI36:X:75309173:75314434:1 gene:ENSG00000102390<br>Homo sapiens HSPC245 mRNA, complete cds.<br><br>cdna:novel chromosome:NCBI36:X:75309277:75314434:1 gene:ENSG00000102390<br>cdna:Genscan chromosome:NCBI36:X:75309376:75314146:1 |
| 3984661 | 1030564 | 3984655 | core | 100241573 | 100241682 | X | + | | |
| 3985763 | 1031200 | 3985717 | core | 102932278 | 102933611 | X | + | ENSESTT00000034958<br>ENST00000372608<br>ENSESTT00000034959<br>ENST00000372606<br>ENSESTT00000034960<br>ENSESTT00000034961<br>NM_199478<br>NM_000533<br>ENST00000303958<br>ENST00000361621<br>ENSESTT00000034962<br>ENSESTT00000034964<br>ENSESTT00000034966 | <br>cdna:known chromosome:NCBI36:X:102918434:102934204:1 gene:ENSG00000123560<br><br>cdna:known chromosome:NCBI36:X:102918434:102934204:1 gene:ENSG00000123560<br><br><br>Homo sapiens proteolipid protein 1 (Pelizaeus-Merzbacher disease, spastic paraplegia 2, uncomplicated) (PLP1), transcript variant 2, mRNA.<br>Homo sapiens proteolipid protein 1 (Pelizaeus-Merzbacher disease, spastic paraplegia 2, uncomplicated) (PLP1), transcript variant 1, mRNA.<br>cdna:known-ccds chromosome:NCBI36:X:102918410:102934201:1 gene:ENSG00000123560 CCDS14513.1<br>cdna:known-ccds chromosome:NCBI36:X:102918410:102934201:1 gene:ENSG00000123560 CCDS14514.1 |
| 3988181 | 1032727 | 3988165 | core | 115486162 | 115486267 | X | + | NM_007231<br>ENST00000371900<br>ENST00000262822<br>ENSESTT00000049006<br>GENSCAN00000023156<br>ENSESTT00000049007<br>ENSESTT00000049008 | Homo sapiens solute carrier family 6 (amino acid transporter), member 14 (SLC6A14), mRNA.<br>cdna:known-ccds chromosome:NCBI36:X:115481818:115506653:1 gene:ENSG00000087916 CCDS14570.1<br>cdna:known chromosome:NCBI36:X:115481818:115506651:1 gene:ENSG00000087916<br><br>cdna:Genscan chromosome:NCBI36:X:115429624:115492075:1 |
| 3996693 | 1037768 | 3996667 | core | 153658268 | 153658336 | X | + | NM_001363<br>ENSESTT00000045529<br>ENST00000369550<br>GENSCAN00000045573<br>ENST00000253127<br>ENSESTT00000045530<br>ENSESTT00000045531<br>ENSESTT00000045532 | Homo sapiens dyskeratosis congenita 1, dyskerin (DKC1), mRNA.<br><br>cdna:known-ccds chromosome:NCBI36:X:153644229:153659158:1 gene:ENSG00000130826 CCDS14761.1<br>cdna:Genscan chromosome:NCBI36:X:153644435:153658336:1<br>cdna:known chromosome:NCBI36:X:153644233:153659150:1 gene:ENSG00000130826 |
| 3998804 | 1039067 | 3998766 | core | 8515843 | 8516006 | X | - | NM_000216<br>ENSESTT00000052686<br>ENST00000262648<br>GENSCAN00000024172<br>ENSESTT00000052687<br>ENSESTT00000052688<br>GENSCAN00000024169<br>GENSCAN00000067774<br>ENSESTT00000052689<br>GENSCAN00000062955 | Homo sapiens Kallmann syndrome 1 sequence (KAL1), mRNA.<br><br>cdna:known-ccds chromosome:NCBI36:X:8456915:8660227:-1 gene:ENSG00000011201 CCDS14130.1<br>cdna:Genscan chromosome:NCBI36:X:8448039:8475422:-1<br><br><br>cdna:Genscan chromosome:NCBI36:X:8494053:8498745:-1<br>cdna:Genscan chromosome:NCBI36:X:8513308:8527270:-1<br><br>cdna:Genscan chromosome:NCBI36:X:8625709:8660077:-1 |
| 4004319 | 1042562 | 4004044 | core | 32376510 | 32376667 | X | - | ENSESTT00000034371 | |

| | | | | | | | | | |
|---|---|---|---|---|---|---|---|---|---|
| | | | | | | | | NM_004006<br>NM_000109<br>NM_004010<br>NM_004009<br>NM_004007<br>ENST00000357033<br>ENST00000378690<br>ENST00000378687<br>GENSCAN00000023576 | Homo sapiens dystrophin (muscular dystrophy, Duchenne and Becker types) (DMD), transcript variant Dp427m, mRNA.<br>Homo sapiens dystrophin (muscular dystrophy, Duchenne and Becker types) (DMD), transcript variant Dp427c, mRNA.<br>Homo sapiens dystrophin (muscular dystrophy, Duchenne and Becker types) (DMD), transcript variant Dp427p2, mRNA.<br>Homo sapiens dystrophin (muscular dystrophy, Duchenne and Becker types) (DMD), transcript variant Dp427p1, mRNA.<br>Homo sapiens dystrophin (muscular dystrophy, Duchenne and Becker types) (DMD), transcript variant Dp427l, mRNA.<br>cdna:known-ccds chromosome:NCBI36:X:31047257:33139632:-1 gene:ENSG00000198947 CCDS14233.1<br>cdna:known chromosome:NCBI36:X:31049957:33139350:-1 gene:ENSG00000198947<br>cdna:known chromosome:NCBI36:X:31054671:32948245:-1 gene:ENSG00000198947<br>cdna:Genscan chromosome:NCBI36:X:32366273:32474136:-1 |
| 4004323 | 1042566 | 4004044 | core | 32396645 | 32396736 | X | - | NM_004006<br>NM_000109<br>NM_004010<br>NM_004009<br>NM_004007<br>ENST00000357033<br>ENST00000378690<br>ENST00000378687<br>ENSESTT00000034371<br>GENSCAN00000023576<br>ENSESTT00000034370<br>ENSESTT00000034369<br>BC036103<br>ENST00000288447<br>ENSESTT00000034368<br>ENST00000378677<br>GENSCAN00000066631<br>GENSCAN00000028533<br>BC010932 | Homo sapiens dystrophin (muscular dystrophy, Duchenne and Becker types) (DMD), transcript variant Dp427m, mRNA.<br>Homo sapiens dystrophin (muscular dystrophy, Duchenne and Becker types) (DMD), transcript variant Dp427c, mRNA.<br>Homo sapiens dystrophin (muscular dystrophy, Duchenne and Becker types) (DMD), transcript variant Dp427p2, mRNA.<br>Homo sapiens dystrophin (muscular dystrophy, Duchenne and Becker types) (DMD), transcript variant Dp427p1, mRNA.<br>Homo sapiens dystrophin (muscular dystrophy, Duchenne and Becker types) (DMD), transcript variant Dp427l, mRNA.<br>cdna:known-ccds chromosome:NCBI36:X:31047257:33139632:-1 gene:ENSG00000198947 CCDS14233.1<br>cdna:known chromosome:NCBI36:X:31049957:33139350:-1 gene:ENSG00000198947<br>cdna:known chromosome:NCBI36:X:31054671:32948245:-1 gene:ENSG00000198947<br><br>cdna:Genscan chromosome:NCBI36:X:32366273:32474136:-1<br><br><br>Homo sapiens dystrophin (muscular dystrophy, Duchenne and Becker types), mRNA (cDNA clone IMAGE:5274415), complete cds.<br>cdna:known chromosome:NCBI36:X:32444634:33267479:-1 gene:ENSG00000198947<br><br>cdna:known chromosome:NCBI36:X:32472327:33056468:-1 gene:ENSG00000198947<br>cdna:Genscan chromosome:NCBI36:X:32493740:32573190:-1<br>cdna:Genscan chromosome:NCBI36:X:32625908:32777858:-1<br>Homo sapiens dystrophin (muscular dystrophy, Duchenne and Becker types), mRNA (cDNA clone IMAGE:4273908), with apparent retained intron. |
| 4004875 | 1042892 | 4004853 | core | 37964899 | 37964985 | X | - | NM_006307<br>ENSESTT00000005993<br>ENST00000378533<br>GENSCAN00000055483<br>ENST00000343800<br>ENST00000218072<br>GENSCAN00000033697<br>GENSCAN00000036964 | Homo sapiens sushi-repeat-containing protein, X-linked (SRPX), mRNA.<br><br>cdna:known-ccds chromosome:NCBI36:X:37893536:37965096:-1 gene:ENSG00000101955 CCDS14245.1<br>cdna:Genscan chromosome:NCBI36:X:37964332:37964989:-1<br>cdna:known chromosome:NCBI36:X:37893539:37965640:-1 gene:ENSG00000101955<br>cdna:known chromosome:NCBI36:X:37893539:37965075:-1 gene:ENSG00000101955<br>cdna:Genscan chromosome:NCBI36:X:37893908:37909092:-1<br>cdna:Genscan chromosome:NCBI36:X:37916078:37918548:-1 |
| 4005674 | 1043399 | 4005644 | core | 40426054 | 40426121 | X | - | NM_004229<br>ENST00000324817<br>AK023368<br>ENSESTT00000002347<br>ENSESTT00000002348 | Homo sapiens cofactor required for Sp1 transcriptional activation, subunit 2, 150kDa (CRSP2), mRNA.<br>cdna:known chromosome:NCBI36:X:40392502:40479727:-1 gene:ENSG00000180182<br>Homo sapiens cDNA FLJ13306 fis, clone OVARC1001417, highly similar to Homo sapiens thyroid hormone receptor-associated protein complex component TRAP170 mRNA. |

| | | | | | | | | | |
|---|---|---|---|---|---|---|---|---|---|
| | | | | | | | | AJ012077<br>ENSESTT00000002346<br>GENSCAN00000027468<br>ENSESTT00000002345<br>ENSESTT00000002344<br>ENSESTT00000002343 | Homo sapiens mRNA for EXLM1 protein, 3' end.<br>cdna:Genscan chromosome:NCBI36:X:40419417:40480234:-1 |
| 4007881 | 1044679 | 4007865 | core | 48921310 | 48921435 | X | - | NM_006150<br>ENST00000376317<br>ENST00000012407<br>GENSCAN00000042919<br>ENSESTT00000039543<br>ENST00000376310 | Homo sapiens LIM domain only 6 (LMO6), mRNA.<br>cdna:known chromosome:NCBI36:X:48918095:48929789:-1 gene:ENSG00000012211<br>cdna:known chromosome:NCBI36:X:48918847:48929766:-1 gene:ENSG00000012211<br>cdna:Genscan chromosome:NCBI36:X:48918966:48929694:-1<br>cdna:novel chromosome:NCBI36:X:48922698:48929786:-1 gene:ENSG00000012211 |
| 4009714 | 1045783 | 4009667 | core | 54538473 | 54538554 | X | - | NM_004463<br>GENSCAN00000038822<br>ENST00000375135<br>ENST00000218425<br>ENSESTT00000007975<br>ENSESTT00000007974 | Homo sapiens FYVE, RhoGEF and PH domain containing 1 (faciogenital dysplasia) (FGD1), mRNA.<br>cdna:Genscan chromosome:NCBI36:X:54489267:54538590:-1<br>cdna:known-ccds chromosome:NCBI36:X:54488612:54539324:-1 gene:ENSG00000102302 CCDS14359.1<br>cdna:known chromosome:NCBI36:X:54488614:54539324:-1 gene:ENSG00000102302 |
| 4019469 | 1051554 | 4019465 | core | 118606878 | 118606955 | X | - | NM_017544<br>AJ011812<br>ENST00000304449<br>ENST00000371527<br>ENST00000335357<br>BC040379<br>ENSESTT00000003589 | Homo sapiens NF-kappaB repressing factor (NKRF), mRNA.<br>Homo sapiens mRNA for transcription factor NRF, partial.<br>cdna:known chromosome:NCBI36:X:118606328:118623886:-1 gene:ENSG00000186416<br>cdna:known chromosome:NCBI36:X:118606328:118611141:-1 gene:ENSG00000186416<br>cdna:known chromosome:NCBI36:X:118606338:118623874:-1 gene:ENSG00000186416<br>Homo sapiens NF-kappaB repressing factor, mRNA (cDNA clone MGC:24866 IMAGE:4777539), complete cds. |
| 4026633 | 1055914 | 4026624 | core | 152589846 | 152589872 | X | - | BC033746<br>ENST00000253123 | Homo sapiens pregnancy upregulated non-ubiquitously expressed CaM kinase, mRNA (cDNA clone IMAGE:5194910), complete cds.<br>cdna:known-ccds chromosome:NCBI36:X:152588384:152592451:-1 gene:ENSG00000130822 CCDS14725.1 |
| 4026975 | 1056119 | 4026956 | core | 152873924 | 152873971 | X | - | NM_005334<br>ENST00000310441<br>ENST00000354233<br>ENST00000369984<br>ENST00000369988<br>GENSCAN00000038710<br>BC063435<br>ENSESTT00000038563 | Homo sapiens host cell factor C1 (VP16-accessory protein) (HCFC1), mRNA.<br>cdna:known chromosome:NCBI36:X:152866204:152889829:-1 gene:ENSG00000172534<br>cdna:known chromosome:NCBI36:X:152866204:152889829:-1 gene:ENSG00000172534<br>cdna:novel chromosome:NCBI36:X:152866204:152889829:-1 gene:ENSG00000172534<br>cdna:novel chromosome:NCBI36:X:152866198:152890452:-1 gene:ENSG00000172534<br>cdna:Genscan chromosome:NCBI36:X:152848634:152889485:-1<br>Homo sapiens host cell factor C1 (VP16-accessory protein), mRNA (cDNA clone IMAGE:5395387), complete cds. |
| 4027241 | 1056267 | 4027176 | core | 153246388 | 153246519 | X | - | NM_001456<br>ENST00000369863<br>ENST00000356744<br>ENST00000369860<br>ENST00000369856<br>ENST00000369854<br>ENST00000369852<br>BC041179<br>AL157419<br>ENSESTT00000045567<br>ENSESTT00000045568<br>ENST00000360319<br>ENST00000369850<br>ENST00000344736 | Homo sapiens filamin A, alpha (actin binding protein 280) (FLNA), mRNA.<br>cdna:known chromosome:NCBI36:X:153230159:153256123:-1 gene:ENSG00000196924<br>cdna:known chromosome:NCBI36:X:153230159:153256123:-1 gene:ENSG00000196924<br>cdna:known chromosome:NCBI36:X:153230159:153256123:-1 gene:ENSG00000196924<br>cdna:known chromosome:NCBI36:X:153230159:153256123:-1 gene:ENSG00000196924<br>cdna:known chromosome:NCBI36:X:153230159:153256123:-1 gene:ENSG00000196924<br>cdna:known chromosome:NCBI36:X:153230159:153256123:-1 gene:ENSG00000196924<br>Homo sapiens, Similar to filamin C, gamma (actin binding protein 280), clone IMAGE:4152096, mRNA, partial cds.<br>Homo sapiens mRNA; cDNA DKFZp434P031 (from clone DKFZp434P031).<br>cdna:known chromosome:NCBI36:X:153230088:153252845:-1 gene:ENSG00000196924<br>cdna:known chromosome:NCBI36:X:153230210:153256188:-1 gene:ENSG00000196924 |

| | | | | | | | | | |
|---|---|---|---|---|---|---|---|---|---|
| | | | | | | | | GENSCAN00000057448<br>ENSESTT00000045566<br>ENSESTT00000045565<br>ENSESTT00000045564 | cdna:known chromosome:NCBI36:X:153230346:153252850:-1 gene:ENSG00000196924<br>cdna:Genscan chromosome:NCBI36:X:153230411:153242149:-1 |
