# Supplementary material for: Comparison of Two Methods for Detecting Alternative Splice Variants Using GeneChip® Exon Arrays
Source: Int J Biomed Sci. 2011 Sep;7(3):172–80. (PMC3614835)
Supplement: Supplementary file 7 [file IJBS-7-172_SD9.pdf]

Table OL3. Affymetrix Human Exon 1.0 ST Transcription ID for alternative spice variants detected by both Partek GS and MIDAS

2328868	2371139	2376168	2398706	2409104	2411228	2413203	2425756
2443120	2446567	2450345	2451593	2531589	2560076	2570193	2570616
2574984	2584134	2604254	2605321	2611848	2625793	2652675	2676009
2686458	2690956	2692319	2710474	2710599	2712236	2727226	2728938
2730746	2734047	2735027	2740067	2746591	2779199	2786322	2842624
2886679	2889916	2891556	2897899	2907671	2923868	2924514	2931391
2946106	2949622	2961177	2976041	2985781	3020343	3025545	3026599
3046444	3047581	3049522	3069366	3079803	3102372	3105600	3110317
3125571	3150844	3151534	3157385	3168508	3173974	3174121	3201319
3222170	3230760	3238962	3252036	3265224	3265565	3292946	3296046
3304301	3332626	3358201	3388673	3388830	3394660	3422144	3428845
3442641	3454892	3457101	3490655	3510066	3556990	3557851	3569814
3573870	3577443	3581637	3595979	3597338	3604147	3605395	3607537
3610982	3630736	3632806	3643580	3644541	3653677	3662808	3674199
3685329	3694657	3703885	3728776	3728964	3733590	3742285	3750662
3751859	3756193	3758510	3768627	3773244	3815399	3821263	3838425
3839346	3853108	3859761	3871192	3881282	3881443	3881786	3887049
3891278	3893520	3907111	3910785	3913960	3923218	3923257	3930360
3939470	3950872	3952825	3959388	3985717	3996667	3998766	4004044
