# Supplementary material for: Comparison of Two Methods for Detecting Alternative Splice Variants Using GeneChip® Exon Arrays
Source: Int J Biomed Sci. 2011 Sep;7(3):172–80. (PMC3614835)
Supplement: Supplementary file 8 [file IJBS-7-172_SD5.pdf]

**Additional file 5.** Transcript cluster IDs for the those selected by both Partek® GS and MIDAS
**Transcript IDs**
2328868
2371139
2376168
2398706
2409104
2411228
2413203
2425756
2443120
2446567
2450345
2451593
2531589
2560076
2570193
2570616
2574984
2584134
2604254
2605321
2611848
2625793
2652675
2676009
2686458
2690956
2692319
2710474
2710599
2712236
2727226
2728938
2730746
2734047
2735027
2740067
2746591
2779199
2786322
2842624
2886679

2889916
2891556
2897899
2907671
2923868
2924514
2931391
2946106
2949622
2961177
2976041
2985781
3020343
3025545
3026599
3046444
3047581
3049522
3069366
3079803
3102372
3105600
3110317
3125571
3150844
3151534
3157385
3168508
3173974
3174121
3201319
3222170
3230760
3238962
3252036
3265224
3265565
3292946
3296046
3304301
3332626
3358201
3388673

3388830
3394660
3422144
3428845
3442641
3454892
3457101
3490655
3510066
3556990
3557851
3569814
3573870
3577443
3581637
3595979
3597338
3604147
3605395
3607537
3610982
3630736
3632806
3643580
3644541
3653677
3662808
3674199
3685329
3694657
3703885
3728776
3728964
3733590
3742285
3750662
3751859
3756193
3758510
3768627
3773244
3815399
3821263

3838425
3839346
3853108
3859761
3871192
3881282
3881443
3881786
3887049
3891278
3893520
3907111
3910785
3913960
3923218
3923257
3930360
3939470
3950872
3952825
3959388
3985717
3996667
3998766
4004044
