# Supplementary material for: Comparison of Two Methods for Detecting Alternative Splice Variants Using GeneChip® Exon Arrays
Source: Int J Biomed Sci. 2011 Sep;7(3):172–80. (PMC3614835)
Supplement: Supplementary file 9 [file IJBS-7-172_SD6.pdf]

Additional file 6. Annotation for those transcript clusters selected by both Partek® GS and MIDAS

Transcript Cluster ID	Exon Cluster ID	Probe Set ID	Probe Set Grade	Current Genome Chromosome	Current Genome Transcript Start	Current Genome Transcript Stop	Current Genome Strand	Transcript ID (Annotated)	Representative Transcript Description
2328868	8406	2328869	core	1	32530294	32572813	+	NM_004964	Homo sapiens histone deacetylase 1 (HDAC1), mRNA.
	8407	2328870	core					AK225555	Homo sapiens mRNA for histone deacetylase 1 variant, clone: KDN05801.
	8408	2328871	extended					U50079	Human histone deacetylase HD1 mRNA, complete cds.
	8409	2328872	core					BX648055	Homo sapiens mRNA; cDNA DKFZp686H12203 (from clone DKFZp686H12203).
	8410	2328873	core					ENSESTT00000029616	
	8411	2328874	extended					ENSESTT00000029617	
	8412	2328875	extended					ENSESTT00000029618	
	8413	2328876	full					ENST00000373548	cdna:known-ccds chromosome:NCBI36:1:32530274:32571823:1 gene:ENSG00000116478 CCDS360.1
	8414	2328877	extended					ENST00000271095	cdna:known chromosome:NCBI36:1:32530295:32571811:1 gene:ENSG00000116478
	8415	2328878	core					ENST00000373541	cdna:known chromosome:NCBI36:1:32530295:32571811:1 gene:ENSG00000116478
	8416	2328879	core					GENSCAN00000018653	cdna:Genscan chromosome:NCBI36:1:32530358:32571232:1
	8417	2328880	extended						
	8418	2328881	extended						
	8419	2328882	core						
	8420	2328883	extended						
	8421	2328884	extended						
	8422	2328885	core						
	8423	2328886	extended						
	8424	2328887	extended						
	8425	2328888	extended						
	8426	2328889	core						
	8427	2328890	core						
	8428	2328891	core						
		2328892	core						
		2328893	full						
		2328894	core						
		2328895	extended						
		2328896	core						
		2328897	extended						
		2328898	core						
		2328899	extended						
		2328900	core						
		2328901	extended						
2371139	34337	2371140	full	1	181400862	181494723	+	NM_005562	Homo sapiens laminin, gamma 2 (LAMC2), transcript variant 1, mRNA.
	34338	2371141	full					NM_018891	Homo sapiens laminin, gamma 2 (LAMC2), transcript variant 2, mRNA.
	34339	2371142	full					ENSESTT00000017771	
	34340	2371143	full					ENSESTT00000017772	
	34341	2371144	full					ENSESTT00000017773	
	34342	2371145	full					ENST00000264144	cdna:known-ccds chromosome:NCBI36:1:181422022:181480662:1 gene:ENSG00000058085 CCDS1352.1
	34343	2371146	core					ENST00000367544	cdna:known chromosome:NCBI36:1:181422046:181480658:1 gene:ENSG00000058085
	34344	2371147	full					GENSCAN00000000951	cdna:Genscan chromosome:NCBI36:1:181400862:181479158:1
	34345	2371148	full						
	34346	2371149	full						
	34347	2371150	core						
	34348	2371151	extended						
	34349	2371152	core						
	34350	2371153	core						
	34351	2371154	extended						
	34352	2371155	core						
	34353	2371156	full						

	34354<br>34355<br>34356<br>34357<br>34358<br>34359<br>34360<br>34361<br>34362<br>34363<br>34364<br>34365<br>34366<br>34367<br>34368<br>34369<br>34370<br>34371<br>34372<br>34373<br>34374<br>34375<br>34376<br>34377	2371157<br>2371158<br>2371159<br>2371160<br>2371161<br>2371162<br>2371163<br>2371164<br>2371165<br>2371166<br>2371167<br>2371168<br>2371169<br>2371170<br>2371171<br>2371172<br>2371173<br>2371174<br>2371175<br>2371176<br>2371177<br>2371178<br>2371179<br>2371180<br>2371181<br>2371182<br>2371183<br>2371184<br>2371185<br>2371186<br>2371187<br>2371188<br>2371189<br>2371190<br>2371191<br>2371192	core<br>core<br>core<br>core<br>core<br>core<br>full<br>core<br>core<br>core<br>core<br>extended<br>core<br>core<br>core<br>core<br>core<br>core<br>core<br>extended<br>core<br>core<br>core<br>extended<br>full<br>core<br>core<br>core<br>core<br>core<br>core<br>core<br>core<br>extended<br>extended<br>extended						
2376168	37558<br>37559<br>37560<br>37561<br>37562<br>37563<br>37564<br>37565<br>37566<br>37567<br>37568<br>37569<br>37570<br>37571<br>37572<br>37573<br>37574<br>37575	2376169<br>2376170<br>2376171<br>2376172<br>2376173<br>2376174<br>2376175<br>2376176<br>2376177<br>2376178<br>2376179<br>2376180<br>2376181<br>2376182<br>2376183<br>2376184<br>2376185<br>2376186	extended<br>full<br>full<br>extended<br>extended<br>extended<br>extended<br>full<br>full<br>full<br>full<br>full<br>full<br>full<br>full<br>full<br>extended<br>extended	1	203063774	203258569	+	NM_015090<br>AB177861<br>AK090639<br>AK127424<br>AK128699<br>AK172728<br>BC040674<br>BX537841<br>BX641048<br>ENSESTT00000016402<br>ENSESTT00000016403<br>ENSESTT00000016404<br>ENSESTT00000016405<br>ENSESTT00000016406<br>ENSESTT00000016407<br>ENST00000367172<br>ENST00000367171<br>ENST00000367170	Homo sapiens neurofascin homolog (chicken) (NFASC), mRNA.<br>Homo sapiens mRNA for KIAA0756 splice variant 1.<br>Homo sapiens cDNA FLJ33320 fis, clone BNGH42007798, highly similar to Rattus norvegicus ankyrin binding cell adhesion molecule neurofascin mRNA.<br>Homo sapiens cDNA FLJ45516 fis, clone BRTHA2022968, moderately similar to NG-CAM related cell adhesion molecule precursor.<br>Homo sapiens cDNA FLJ46866 fis, clone UTERU3011837, moderately similar to NG-CAM related cell adhesion molecule precursor.<br>Homo sapiens cDNA PSEC0044 fis, clone: NT2RP1001007.<br>Homo sapiens neurofascin, mRNA (cDNA clone IMAGE:4817018).<br>Homo sapiens mRNA; cDNA DKFZp686E03196 (from clone DKFZp686E03196).<br>Homo sapiens mRNA; cDNA DKFZp686J0597 (from clone DKFZp686J0597).

	37576	2376187	core					ENST00000339876	cdna:known chromosome:NCBI36:1:203064405:203258587:1 gene:ENSG00000163531
	37577	2376188	core					ENST00000367169	cdna:known chromosome:NCBI36:1:203064405:203258587:1 gene:ENSG00000163531
	37578	2376189	full					ENST00000338515	cdna:known chromosome:NCBI36:1:203064405:203258587:1 gene:ENSG00000163531
	37579	2376190	full					ENST00000338586	cdna:known chromosome:NCBI36:1:203064405:203258587:1 gene:ENSG00000163531
	37580	2376191	full					ENST00000295776	cdna:known chromosome:NCBI36:1:203064405:203212895:1 gene:ENSG00000163531
	37581	2376192	full					ENST00000360049	cdna:known chromosome:NCBI36:1:203156423:203258572:1 gene:ENSG00000163531
	37582	2376193	core					ENST00000367173	cdna:known chromosome:NCBI36:1:203156423:203258572:1 gene:ENSG00000163531
	37583	2376194	core					GENSCAN00000038264	cdna:known chromosome:NCBI36:1:203156423:203258572:1 gene:ENSG00000163531
	37584	2376195	core					GENSCAN00000017937	cdna:known chromosome:NCBI36:1:203156423:203258572:1 gene:ENSG00000163531
	37585	2376196	extended						cdna:novel chromosome:NCBI36:1:203064402:203246653:1 gene:ENSG00000163531
	37586	2376197	full						cdna:Genscan chromosome:NCBI36:1:203064427:203126117:1
	37587	2376198	full						cdna:Genscan chromosome:NCBI36:1:203173804:203252290:1
	37588	2376199	full						
	37589	2376200	full						
	37590	2376201	full						
	37591	2376202	extended						
	37592	2376203	extended						
	37593	2376204	extended						
	37594	2376205	full						
	37595	2376206	full						
	37596	2376207	full						
	37597	2376208	extended						
	37598	2376209	core						
	37599	2376210	core						
	37600	2376211	full						
	37601	2376212	core						
	37602	2376213	core						
	37603	2376214	core						
	37604	2376215	full						
	37605	2376216	full						
	37606	2376217	core						
	37607	2376218	core						
	37608	2376219	full						
	37609	2376220	core						
	37610	2376221	full						
	37611	2376222	core						
	37612	2376223	core						
	37613	2376224	core						
	37614	2376225	full						
	37615	2376226	core						
	37616	2376227	core						
	37617	2376228	core						
	37618	2376229	core						
	37619	2376230	core						
	37620	2376231	core						
	37621	2376232	full						
	37622	2376233	core						
	37623	2376234	core						
	37624	2376235	core						
	37625	2376236	core						
	37626	2376237	core						
	37627	2376238	full						
	37628	2376239	full						
	37629	2376240	extended						

	37630<br>37631<br>37632<br>37633<br>37634<br>37635	2376241<br>2376242<br>2376243<br>2376244<br>2376245<br>2376246<br>2376247<br>2376248<br>2376249<br>2376250<br>2376251<br>2376252<br>2376253<br>2376254<br>2376255<br>2376256<br>2376257<br>2376258<br>2376259<br>2376260<br>2376261<br>2376262<br>2376263<br>2376264<br>2376265<br>2376266<br>2376267<br>2376268<br>2376269<br>2376270<br>2376271<br>2376272<br>2376273<br>2376274<br>2376275<br>2376276<br>2376277<br>2376278<br>2376279<br>2376280<br>2376281<br>2376282<br>2376283<br>2376284	extended<br>extended<br>core<br>extended<br>extended<br>extended<br>extended<br>extended<br>extended<br>core<br>core<br>extended<br>full<br>free<br>full<br>core<br>core<br>extended<br>extended<br>extended<br>extended<br>extended<br>extended<br>extended<br>extended<br>extended<br>extended<br>core<br>core<br>core<br>full<br>full<br>full<br>core<br>core<br>core<br>core<br>full<br>full<br>core<br>core<br>core<br>core<br>core						
2398706	51223<br>51224<br>51225<br>51226<br>51227<br>51228<br>51229<br>51230<br>51231<br>51232	2398707<br>2398708<br>2398709<br>2398710<br>2398711<br>2398712<br>2398713<br>2398714<br>2398715<br>2398716	full<br>full<br>full<br>core<br>core<br>core<br>core<br>core<br>full<br>extended	1	17173325	17180668	-	NM_022089<br>NM_017459<br>NM_002403<br>BC030267<br>BC028033<br>ENSESTT00000039935<br>ENSESTT00000039936<br>ENSESTT00000039937<br>ENSESTT00000039938<br>ENST00000375535	Homo sapiens ATPase type 13A2 (ATP13A2), mRNA.<br>Homo sapiens microfibrillar-associated protein 2 (MFAP2), transcript variant 1, mRNA.<br>Homo sapiens microfibrillar-associated protein 2 (MFAP2), transcript variant 2, mRNA.<br>Homo sapiens ATPase type 13A2, mRNA (cDNA clone MGC:40082 IMAGE:5240813), complete cds.<br>Homo sapiens microfibrillar-associated protein 2, mRNA (cDNA clone IMAGE:5242233), with apparent retained intron.<br><br><br><br><br>cdna:known-ccds chromosome:NCBI36:1:17173584:17179917:-1 gene:ENSG00000117122 CCDS174.1

	51233<br>51234<br>51235<br>51236	2398717<br>2398718<br>2398719<br>2398720<br>2398721<br>2398722<br>2398723<br>2398724<br>2398725<br>2398726<br>2398727<br>2398728<br>2398729<br>2398730<br>2398731	core<br>core<br>extended<br>core<br>extended<br>core<br>extended<br>core<br>core<br>full<br>full<br>core<br>extended<br>extended<br>core					ENST00000326735<br>ENST00000375534<br>ENST00000235772<br>ENST00000341676<br>ENST00000375508<br>GENSCAN00000024160	cdna:known-ccds chromosome:NCBI36:1:17185040:17210854:-1 gene:ENSG00000159363 CCDS175.1<br>cdna:known chromosome:NCBI36:1:17173584:17177358:-1 gene:ENSG00000117122<br>cdna:known chromosome:NCBI36:1:17173590:17179760:-1 gene:ENSG00000117122<br>cdna:known chromosome:NCBI36:1:17185040:17210997:-1 gene:ENSG00000159363<br>cdna:known chromosome:NCBI36:1:17194941:17199373:-1 gene:ENSG00000159363<br>cdna:Genscan chromosome:NCBI36:1:17173325:17210602:-1
2409104	57577<br>57578<br>57579<br>57580<br>57581<br>57582<br>57583<br>57584<br>57585<br>57586<br>57587<br>57588<br>57589<br>57590<br>57591<br>57592<br>57593<br>57594<br>57595<br>57596<br>57597<br>57598<br>57599<br>57600<br>57601<br>57602<br>57603<br>57604<br>57605<br>57606<br>57607<br>57608<br>57609<br>57610<br>57611<br>57612<br>57613<br>57614<br>57615	2409105<br>2409106<br>2409107<br>2409108<br>2409109<br>2409110<br>2409111<br>2409112<br>2409113<br>2409114<br>2409115<br>2409116<br>2409117<br>2409118<br>2409119<br>2409120<br>2409121<br>2409122<br>2409123<br>2409124<br>2409125<br>2409126<br>2409127<br>2409128<br>2409129<br>2409130<br>2409131<br>2409132<br>2409133<br>2409134<br>2409135<br>2409136<br>2409137<br>2409138<br>2409139<br>2409140<br>2409141<br>2409142<br>2409143	full<br>full<br>extended<br>full<br>full<br>full<br>full<br>full<br>full<br>full<br>extended<br>core<br>core<br>core<br>core<br>core<br>extended<br>core<br>core<br>core<br>extended<br>full<br>core<br>core<br>extended<br>core<br>extended<br>extended<br>core<br>full<br>extended<br>full<br>extended<br>extended<br>core<br>full<br>extended<br>extended<br>extended	1	43124794	43338329	-	NM_006516<br>XM_941326<br>XM_927705<br>AB208987<br>ENSESTT00000045954<br>ENSESTT00000045955<br>ENST00000372501<br>ENST00000270929<br>ENST00000372500<br>GENSCAN00000054874<br>GENSCAN00000047316<br>GENSCAN00000031170<br>GENSCAN00000007378	Homo sapiens solute carrier family 2 (facilitated glucose transporter), member 1 (SLC2A1), mRNA.<br>PREDICTED: Homo sapiens similar to EVIN1 (LOC652027), mRNA.<br>PREDICTED: Homo sapiens similar to EVIN1 (LOC644590), mRNA.<br>Homo sapiens mRNA for solute carrier family 2 (facilitated glucose transporter), member 1 variant protein.<br><br><br>cdna:known-ccds chromosome:NCBI36:1:43164106:43197088:-1 gene:ENSG00000117394 CCDS477.1<br>cdna:known chromosome:NCBI36:1:43164115:43197088:-1 gene:ENSG00000117394<br>cdna:novel chromosome:NCBI36:1:43168577:43197117:-1 gene:ENSG00000117394<br>cdna:Genscan supercontig::NT_113873:10431:21686:1<br>cdna:Genscan chromosome:NCBI36:1:43165299:43233077:-1<br>cdna:Genscan chromosome:NCBI36:1:43279531:43338329:-1<br>cdna:Genscan chromosome:NCBI36:1:43124794:43129769:-1

	57616<br>57617<br>57618<br>57619<br>57620<br>57621<br>57622<br>57623<br>57624<br>57625<br>57626	2409144<br>2409145<br>2409146<br>2409147<br>2409148<br>2409149<br>2409150<br>2409151<br>2409152<br>2409153<br>2409154<br>2409155<br>2409156<br>2409157<br>2409158<br>2409159<br>2409160<br>2409161<br>2409162<br>2409163<br>2409164<br>2409165<br>2409166<br>2409167<br>2409168<br>2409169<br>2409170<br>2409171<br>2409172<br>2409173<br>2409174<br>2409175<br>2409176<br>2409177<br>2409178	extended<br>extended<br>full<br>full<br>full<br>full<br>full<br>full<br>full<br>core<br>extended<br>full<br>full<br>full<br>full<br>full<br>full<br>full<br>full<br>full<br>full<br>full<br>full<br>full<br>full<br>full<br>full<br>full<br>full<br>full<br>full<br>full<br>full<br>full<br>full						
2411228	58794<br>58795<br>58796<br>58797<br>58798<br>58799<br>58800<br>58801<br>58802<br>58803<br>58804<br>58805<br>58806<br>58807<br>58808<br>58809<br>58810<br>58811<br>58812	2411229<br>2411230<br>2411231<br>2411232<br>2411233<br>2411234<br>2411235<br>2411236<br>2411237<br>2411238<br>2411239<br>2411240<br>2411241<br>2411242<br>2411243<br>2411244<br>2411245<br>2411246<br>2411247	core<br>core<br>core<br>core<br>core<br>core<br>core<br>core<br>core<br>core<br>core<br>core<br>core<br>core<br>core<br>core<br>core<br>core<br>extended	1	47488403	47552432	-	NM_003035<br>AK128406<br>ENSESTT00000005471<br>ENSESTT00000005472<br>ENSESTT00000005476<br>ENSESTT00000005478<br>ENSESTT00000005479<br>ENSESTT00000005480<br>ENST00000337817<br>ENST00000371877<br>ENST00000243182<br>ENST00000371874<br>ENST00000360380<br>GENSCAN00000054579<br>GENSCAN00000064736	Homo sapiens SCL/TAL1 interrupting locus (STIL), mRNA.<br>Homo sapiens cDNA FLJ46549 fis, clone THYMU3038375, highly similar to Homo sapiens TAL1 (SCL) interrupting locus (SIL).<br><br><br><br><br><br><br>cdna:known-ccds chromosome:NCBI36:1:47488401:47552382:-1 gene:ENSG00000123473 CCDS548.1<br>cdna:known chromosome:NCBI36:1:47488401:47552382:-1 gene:ENSG00000123473<br>cdna:known chromosome:NCBI36:1:47488401:47552382:-1 gene:ENSG00000123473<br>cdna:known chromosome:NCBI36:1:47488401:47519315:-1 gene:ENSG00000123473<br>cdna:known chromosome:NCBI36:1:47488436:47552406:-1 gene:ENSG00000123473<br>cdna:Genscan chromosome:NCBI36:1:47489395:47510452:-1<br>cdna:Genscan chromosome:NCBI36:1:47518479:47548578:-1

	58813<br>58814<br>58815	2411248<br>2411249<br>2411250<br>2411251<br>2411252<br>2411253<br>2411254<br>2411255<br>2411256<br>2411257<br>2411258<br>2411259<br>2411260<br>2411261<br>2411262<br>2411263<br>2411264<br>2411265<br>2411266<br>2411267<br>2411268<br>2411269<br>2411270<br>2411271	core<br>core<br>extended<br>extended<br>core<br>core<br>extended<br>core<br>core<br>core<br>core<br>full<br>core<br>core<br>extended<br>core<br>extended<br>extended<br>core<br>core<br>core<br>core<br>extended<br>full						
2413203	60072<br>60073<br>60074<br>60075<br>60076<br>60077<br>60078<br>60079<br>60080<br>60081<br>60082<br>60083<br>60084<br>60085<br>60086<br>60087<br>60088<br>60089<br>60090<br>60091<br>60092<br>60093<br>60094<br>60095<br>60096<br>60097<br>60098<br>60099<br>60100<br>60101	2413204<br>2413205<br>2413206<br>2413207<br>2413208<br>2413209<br>2413210<br>2413211<br>2413212<br>2413213<br>2413214<br>2413215<br>2413216<br>2413217<br>2413218<br>2413219<br>2413220<br>2413221<br>2413222<br>2413223<br>2413224<br>2413225<br>2413226<br>2413227<br>2413228<br>2413229<br>2413230<br>2413231<br>2413232<br>2413233	free<br>free<br>extended<br>extended<br>extended<br>core<br>core<br>core<br>core<br>core<br>core<br>extended<br>extended<br>extended<br>core<br>core<br>core<br>core<br>extended<br>extended<br>core<br>core<br>core<br>full<br>core<br>core<br>extended<br>core<br>core<br>extended	1	53482138	53632680	-	NM_017522<br>NM_004631<br>NM_001018054<br>NM_033300<br>AK096482<br>AK122887<br>BC014162<br>ENSESTT00000008245<br>ENSESTT00000008246<br>ENSESTT00000008247<br>ENSESTT00000008248<br>ENSESTT00000008249<br>ENST00000347547<br>ENST00000371456<br>ENST00000354412<br>ENST00000306052<br>ENST00000371454<br>ENST00000357488<br>ENST00000371452<br>GENSCAN00000034038	Homo sapiens low density lipoprotein receptor-related protein 8, apolipoprotein e receptor (LRP8), transcript variant 3, mRNA.<br>Homo sapiens low density lipoprotein receptor-related protein 8, apolipoprotein e receptor (LRP8), transcript variant 1, mRNA.<br>Homo sapiens low density lipoprotein receptor-related protein 8, apolipoprotein e receptor (LRP8), transcript variant 4, mRNA.<br>Homo sapiens low density lipoprotein receptor-related protein 8, apolipoprotein e receptor (LRP8), transcript variant 2, mRNA.<br>Homo sapiens cDNA FLJ39163 fis, clone OCBBF2002615, highly similar to Human mRNA for apolipoprotein E receptor 2.<br>Homo sapiens cDNA FLJ16536 fis, clone OCBBF2032152, highly similar to Homo sapiens low density lipoprotein receptor-related protein 8, apolipoprotein e receptor (LRP8).<br>Homo sapiens cDNA clone IMAGE:4547814, partial cds.<br>cdna:known-ccds chromosome:NCBI36:1:53483800:53566409:-1 gene:ENSG00000157193 CCDS580.1<br>cdna:known-ccds chromosome:NCBI36:1:53483805:53566274:-1 gene:ENSG00000157193 CCDS578.1<br>cdna:known-ccds chromosome:NCBI36:1:53483806:53566314:-1 gene:ENSG00000157193 CCDS579.1<br>cdna:known chromosome:NCBI36:1:53483806:53566314:-1 gene:ENSG00000157193<br>cdna:known chromosome:NCBI36:1:53483806:53566314:-1 gene:ENSG00000157193<br>cdna:known chromosome:NCBI36:1:53484813:53566314:-1 gene:ENSG00000157193<br>cdna:known chromosome:NCBI36:1:53484828:53566172:-1 gene:ENSG00000157193<br>cdna:Genscan chromosome:NCBI36:1:53485278:53502656:-1

	60102	2413234	extended						
	60103	2413235	full						
	60104	2413236	full						
	60105	2413237	full						
	60106	2413238	extended						
	60107	2413239	full						
	60108	2413240	full						
	60109	2413241	core						
	60110	2413242	core						
	60111	2413243	full						
	60112	2413244	full						
	60113	2413245	full						
	60114	2413246	core						
	60115	2413247	extended						
	60116	2413248	extended						
	60117	2413249	extended						
	60118	2413250	extended						
	60119	2413251	extended						
	60120	2413252	extended						
	60121	2413253	core						
	60122	2413254	full						
	60123	2413255	full						
	60124	2413256	full						
	60125	2413257	extended						
	60126	2413258	extended						
	60127	2413259	extended						
	60128	2413260	full						
	60129	2413261	extended						
	60130	2413262	extended						
	60131	2413263	extended						
	60132	2413264	full						
	60133	2413265	extended						
	60134	2413266	extended						
		2413267	extended						
		2413268	full						
		2413269	full						
		2413270	extended						
		2413271	extended						
		2413272	full						
		2413273	extended						
		2413274	full						
		2413275	full						
		2413276	full						
		2413277	core						
		2413278	full						
		2413279	full						
		2413280	extended						
		2413281	extended						
		2413282	core						
		2413283	core						
		2413284	core						
		2413285	full						
		2413286	full						
		2413287	full						

		2413288	full						
		2413289	full						
		2413290	full						
2425756	68105	2425757	extended	1	103114282	103347204	-	NM_001854	Homo sapiens collagen, type XI, alpha 1 (COL11A1), transcript variant A, mRNA.
	68106	2425758	extended					NM_080630	Homo sapiens collagen, type XI, alpha 1 (COL11A1), transcript variant C, mRNA.
	68107	2425759	core					NM_080629	Homo sapiens collagen, type XI, alpha 1 (COL11A1), transcript variant B, mRNA.
	68108	2425760	core					ENSESTT00000034465	
	68109	2425761	core					ENSESTT00000034466	
	68110	2425762	core					ENSESTT00000034467	
	68111	2425763	core					ENSESTT00000034468	
	68112	2425764	extended					ENSESTT00000034469	
	68113	2425765	core					ENSESTT00000034470	
	68114	2425766	core					ENSESTT00000034471	
	68115	2425767	core					ENST00000370096	cdna:known-ccds chromosome:NCBI36:1:103114611:103346635:-1 gene:ENSG00000060718 CCDS778.1
	68116	2425768	core					ENST00000358392	cdna:known-ccds chromosome:NCBI36:1:103115583:103346640:-1 gene:ENSG00000060718 CCDS779.1
	68117	2425769	extended					ENST00000353414	cdna:known-ccds chromosome:NCBI36:1:103115583:103346640:-1 gene:ENSG00000060718 CCDS780.1
	68118	2425770	core					ENST00000193186	cdna:known chromosome:NCBI36:1:103115583:103346640:-1 gene:ENSG00000060718
	68119	2425771	core					ENST00000370090	cdna:known chromosome:NCBI36:1:103115629:103269357:-1 gene:ENSG00000060718
	68120	2425772	extended					GENSCAN00000063565	cdna:Genscan chromosome:NCBI36:1:103116163:103161533:-1
	68121	2425773	core					GENSCAN00000010468	cdna:Genscan chromosome:NCBI36:1:103172593:103234186:-1
	68122	2425774	core						
	68123	2425775	core						
	68124	2425776	extended						
	68125	2425777	core						
	68126	2425778	core						
	68127	2425779	core						
	68128	2425780	core						
	68129	2425781	core						
	68130	2425782	core						
	68131	2425783	core						
	68132	2425784	core						
	68133	2425785	core						
	68134	2425786	core						
	68135	2425787	core						
	68136	2425788	core						
	68137	2425789	full						
	68138	2425790	core						
	68139	2425791	core						
	68140	2425792	core						
	68141	2425793	core						
	68142	2425794	extended						
	68143	2425795	core						
	68144	2425796	core						
	68145	2425797	core						
	68146	2425798	core						
	68147	2425799	core						
	68148	2425800	core						
	68149	2425801	core						
	68150	2425802	core						
	68151	2425803	core						
	68152	2425804	core						
	68153	2425805	core						
	68154	2425806	extended						
	68155	2425807	core						

	68156<br>68157<br>68158<br>68159<br>68160<br>68161<br>68162<br>68163<br>68164<br>68165<br>68166<br>68167<br>68168<br>68169<br>68170<br>68171<br>68172<br>68173<br>68174<br>68175<br>68176<br>68177<br>68178<br>68179<br>68180<br>68181<br>68182<br>68183<br>68184	2425808<br>2425809<br>2425810<br>2425811<br>2425812<br>2425813<br>2425814<br>2425815<br>2425816<br>2425817<br>2425818<br>2425819<br>2425820<br>2425821<br>2425822<br>2425823<br>2425824<br>2425825<br>2425826<br>2425827<br>2425828<br>2425829<br>2425830<br>2425831<br>2425832<br>2425833<br>2425834<br>2425835<br>2425836<br>2425837<br>2425838<br>2425839<br>2425840<br>2425841<br>2425842<br>2425843<br>2425844<br>2425845<br>2425846<br>2425847<br>2425848<br>2425849<br>2425850<br>2425851	core<br>core<br>core<br>core<br>core<br>extended<br>extended<br>core<br>core<br>core<br>core<br>core<br>core<br>core<br>core<br>core<br>core<br>core<br>core<br>core<br>core<br>core<br>core<br>extended<br>core<br>core<br>extended<br>core<br>core<br>core<br>extended<br>core<br>full<br>full<br>core<br>full<br>core<br>core<br>full<br>full<br>full<br>core<br>core<br>full						
2443120	78246<br>78247<br>78248<br>78249<br>78250<br>78251<br>78252<br>78253<br>78254<br>78255	2443121<br>2443122<br>2443123<br>2443124<br>2443125<br>2443126<br>2443127<br>2443128<br>2443129<br>2443130	full<br>full<br>full<br>full<br>full<br>full<br>full<br>full<br>full<br>full	1	166827378	166965116	-	NM_001937<br>Z22865<br>ENSESTT00000055262<br>ENST00000367817<br>ENST00000271418<br>GENSCAN00000052263<br>GENSCAN00000052264	Homo sapiens dermatopontin (DPT), mRNA.<br>H.sapiens dermatopontin mRNA, complete CDS.<br><br>cdna:known-ccds chromosome:NCBI36:1:166931321:166965126:-1 gene:ENSG00000143196 CCDS1275.1<br>cdna:known chromosome:NCBI36:1:166931331:166965052:-1 gene:ENSG00000143196<br>cdna:Genscan chromosome:NCBI36:1:166924151:166931665:-1<br>cdna:Genscan chromosome:NCBI36:1:166932411:166965036:-1

	78256<br>78257<br>78258<br>78259<br>78260<br>78261<br>78262<br>78263<br>78264<br>78265<br>78266<br>78267<br>78268<br>78269<br>78270<br>78271<br>78272<br>78273<br>78274<br>78275<br>78276<br>78277<br>78278	2443131<br>2443132<br>2443133<br>2443134<br>2443135<br>2443136<br>2443137<br>2443138<br>2443139<br>2443140<br>2443141<br>2443142<br>2443143<br>2443144<br>2443145<br>2443146<br>2443147<br>2443148<br>2443149<br>2443150<br>2443151<br>2443152<br>2443153<br>2443154<br>2443155<br>2443156<br>2443157<br>2443158<br>2443159<br>2443160<br>2443161<br>2443162<br>2443163<br>2443164<br>2443165<br>2443166<br>2443167	full<br>full<br>full<br>full<br>full<br>full<br>full<br>full<br>full<br>full<br>full<br>core<br>core<br>core<br>core<br>core<br>core<br>core<br>core<br>core<br>extended<br>core<br>extended<br>full<br>core<br>full<br>full<br>full<br>full<br>full<br>full<br>full<br>full<br>core<br>core<br>core<br>extended						
2446567	80388<br>80389<br>80390<br>80391<br>80392<br>80393<br>80394<br>80395<br>80396<br>80397<br>80398<br>80399<br>80400<br>80401<br>80402<br>80403<br>80404	2446568<br>2446569<br>2446570<br>2446571<br>2446572<br>2446573<br>2446574<br>2446575<br>2446576<br>2446577<br>2446578<br>2446579<br>2446580<br>2446581<br>2446582<br>2446583<br>2446584	extended<br>extended<br>extended<br>extended<br>extended<br>extended<br>full<br>full<br>full<br>full<br>full<br>full<br>extended<br>full<br>full<br>full<br>extended	1	179095658	179258656	-	NM_005819<br>AK056657<br>BC039118<br>ENST00000258301<br>ENST00000358073<br>ENST00000362024<br>GENSCAN00000036701<br>GENSCAN00000051836	Homo sapiens syntaxin 6 (STX6), mRNA.<br>Homo sapiens cDNA FLJ32095 fis, clone OCBBF2000998.<br>Homo sapiens syntaxin 6, mRNA (cDNA clone IMAGE:4831269), complete cds.<br>cdna:known-ccds chromosome:NCBI36:1:179208484:179258670:-1 gene:ENSG00000135823 CCDS1341.1<br>cdna:known chromosome:NCBI36:1:179179798:179212241:-1 gene:ENSG00000135823<br>cdna:known chromosome:NCBI36:1:179208801:179258669:-1 gene:ENSG00000135823<br>cdna:Genscan chromosome:NCBI36:1:179187749:179225868:-1<br>cdna:Genscan chromosome:NCBI36:1:179241012:179258432:-1

	80405<br>80406<br>80407<br>80408<br>80409<br>80410<br>80411<br>80412<br>80413<br>80414<br>80415<br>80416<br>80417<br>80418<br>80419<br>80420<br>80421<br>80422<br>80423<br>80424<br>80425<br>80426<br>80427<br>80428<br>80429<br>80430<br>80431<br>80432<br>80433<br>80434<br>80435<br>80436<br>80437<br>80438<br>80439<br>80440	2446585<br>2446586<br>2446587<br>2446588<br>2446589<br>2446590<br>2446591<br>2446592<br>2446593<br>2446594<br>2446595<br>2446596<br>2446597<br>2446598<br>2446599<br>2446600<br>2446601<br>2446602<br>2446603<br>2446604<br>2446605<br>2446606<br>2446607<br>2446608<br>2446609<br>2446610<br>2446611<br>2446612<br>2446613<br>2446614<br>2446615<br>2446616<br>2446617<br>2446618<br>2446619<br>2446620<br>2446621<br>2446622<br>2446623<br>2446624<br>2446625<br>2446626<br>2446627<br>2446628<br>2446629<br>2446630<br>2446631<br>2446632<br>2446633<br>2446634<br>2446635	full<br>full<br>full<br>full<br>full<br>extended<br>extended<br>extended<br>extended<br>full<br>extended<br>extended<br>full<br>full<br>full<br>extended<br>extended<br>extended<br>extended<br>extended<br>extended<br>core<br>core<br>core<br>extended<br>core<br>extended<br>core<br>extended<br>core<br>full<br>full<br>full<br>full<br>core<br>core<br>full<br>extended<br>extended<br>core<br>core<br>full<br>extended<br>extended<br>full<br>full<br>extended<br>extended<br>full<br>core<br>extended						
2450345	82639<br>82640<br>82641	2450346<br>2450347<br>2450348	full<br>extended<br>full	1	198699087	198856743	-	NM_014875<br>BC098582<br>ENSESTT00000045609	Homo sapiens kinesin family member 14 (KIF14), mRNA.<br>Homo sapiens kinesin family member 14, mRNA (cDNA clone IMAGE:6470912), partial cds.

	82642	2450349	full					ENSESTT00000045610	
	82643	2450350	full					ENSESTT00000045611	
	82644	2450351	full					ENST00000367350	cdna:known chromosome:NCBI36:1:198787251:198856485:-1 gene:ENSG00000118193
	82645	2450352	full					ENST00000236917	cdna:novel chromosome:NCBI36:1:198787248:198854474:-1 gene:ENSG00000118193
	82646	2450353	full					GENSCAN00000032460	cdna:Genscan chromosome:NCBI36:1:198746100:198762905:-1
	82647	2450354	full					GENSCAN00000051257	cdna:Genscan chromosome:NCBI36:1:198789139:198902491:-1
	82648	2450355	full						
	82649	2450356	extended						
	82650	2450357	full						
	82651	2450358	full						
	82652	2450359	full						
	82653	2450360	full						
	82654	2450361	full						
	82655	2450362	extended						
	82656	2450363	core						
	82657	2450364	core						
	82658	2450365	core						
	82659	2450366	core						
	82660	2450367	core						
	82661	2450368	core						
	82662	2450369	core						
	82663	2450370	core						
	82664	2450371	extended						
	82665	2450372	full						
	82666	2450373	core						
	82667	2450374	core						
	82668	2450375	core						
	82669	2450376	core						
	82670	2450377	core						
	82671	2450378	core						
	82672	2450379	core						
	82673	2450380	core						
	82674	2450381	core						
	82675	2450382	core						
	82676	2450383	core						
	82677	2450384	core						
	82678	2450385	core						
	82679	2450386	core						
	82680	2450387	core						
	82681	2450388	full						
	82682	2450389	core						
	82683	2450390	core						
	82684	2450391	core						
	82685	2450392	full						
	82686	2450393	core						
	82687	2450394	core						
	82688	2450395	core						
		2450396	core						
		2450397	core						
		2450398	core						
		2450399	core						
		2450400	core						
		2450401	core						
		2450402	core						

		2450403<br>2450404<br>2450405<br>2450406<br>2450407<br>2450408<br>2450409<br>2450410<br>2450411<br>2450412<br>2450413<br>2450414<br>2450415	core<br>core<br>core<br>core<br>core<br>core<br>core<br>core<br>core<br>core<br>core<br>full<br>full						
2451593	83393<br>83394<br>83395<br>83396<br>83397<br>83398<br>83399<br>83400<br>83401<br>83402<br>83403	2451594<br>2451595<br>2451596<br>2451597<br>2451598<br>2451599<br>2451600<br>2451601<br>2451602<br>2451603<br>2451604<br>2451605<br>2451606<br>2451607<br>2451608<br>2451609<br>2451610<br>2451611<br>2451612<br>2451613<br>2451614<br>2451615	core<br>core<br>core<br>core<br>core<br>extended<br>core<br>extended<br>core<br>extended<br>core<br>extended<br>core<br>core<br>extended<br>core<br>core<br>full<br>full<br>core<br>core<br>core	1	201414553	201422500	-	NM_001276<br>NM_004997<br>AB209459<br>BC039132<br>ENSESTT00000019594<br>ENST00000255409<br>ENST00000255416<br>ENST00000367234<br>ENST00000367232<br>GENSCAN00000038022	Homo sapiens chitinase 3-like 1 (cartilage glycoprotein-39) (CHI3L1), mRNA.<br>Homo sapiens myosin binding protein H (MYBPH), mRNA.<br>Homo sapiens mRNA for chitinase 3-like 1 variant protein.<br>Homo sapiens chitinase 3-like 1 (cartilage glycoprotein-39), mRNA (cDNA clone MGC:17246 IMAGE:4183798), complete cds.<br><br>cdna:known-ccds chromosome:NCBI36:1:201414553:201422500:-1 gene:ENSG00000133048 CCDS1435.1<br>cdna:known chromosome:NCBI36:1:201403562:201411565:-1 gene:ENSG00000133055<br>cdna:known chromosome:NCBI36:1:201403562:201411564:-1 gene:ENSG00000133055<br>cdna:known chromosome:NCBI36:1:201414682:201422500:-1 gene:ENSG00000133048<br>cdna:Genscan chromosome:NCBI36:1:201403828:201421669:-1
2531589	133765<br>133766<br>133767<br>133768<br>133769<br>133770<br>133771<br>133772<br>133773<br>133774<br>133775<br>133776<br>133777	2531590<br>2531591<br>2531592<br>2531593<br>2531594<br>2531595<br>2531596<br>2531597<br>2531598<br>2531599<br>2531600<br>2531601<br>2531602<br>2531603<br>2531604<br>2531605<br>2531606<br>2531607	core<br>core<br>core<br>core<br>extended<br>full<br>full<br>full<br>full<br>full<br>full<br>core<br>full<br>full<br>full<br>full<br>core<br>full	2	231437872	231452176	+	NM_001012516<br>NM_030926<br>NM_001012514<br>CR749389<br>ENSESTT00000043428<br>ENST00000326427<br>ENST00000326407<br>ENST00000335005<br>GENSCAN00000048898	Homo sapiens integral membrane protein 2C (ITM2C), transcript variant 2, mRNA.<br>Homo sapiens integral membrane protein 2C (ITM2C), transcript variant 1, mRNA.<br>Homo sapiens integral membrane protein 2C (ITM2C), transcript variant 3, mRNA.<br>Homo sapiens mRNA; cDNA DKFZp686L07102 (from clone DKFZp686L07102).<br><br>cdna:known-ccds chromosome:NCBI36:2:231437865:231452206:1 gene:ENSG00000135916 CCDS2479.1<br>cdna:known chromosome:NCBI36:2:231437865:231452206:1 gene:ENSG00000135916<br>cdna:known chromosome:NCBI36:2:231437865:231452206:1 gene:ENSG00000135916<br>cdna:Genscan chromosome:NCBI36:2:231437985:231451058:1

		2531608<br>2531609<br>2531610<br>2531611<br>2531612<br>2531613<br>2531614<br>2531615	core<br>core<br>core<br>core<br>core<br>core<br>core<br>core						
2560076	151809<br>151810<br>151811<br>151812<br>151813<br>151814<br>151815<br>151816<br>151817<br>151818<br>151819<br>151820<br>151821<br>151822<br>151823<br>151824<br>151825<br>151826<br>151827<br>151828<br>151829<br>151830<br>151831	2560077<br>2560078<br>2560079<br>2560080<br>2560081<br>2560082<br>2560083<br>2560084<br>2560085<br>2560086<br>2560087<br>2560088<br>2560089<br>2560090<br>2560091<br>2560092<br>2560093<br>2560094<br>2560095<br>2560096<br>2560097<br>2560098<br>2560099<br>2560100<br>2560101<br>2560102<br>2560103<br>2560104<br>2560105<br>2560106<br>2560107<br>2560108<br>2560109<br>2560110<br>2560111<br>2560112<br>2560113<br>2560114<br>2560115<br>2560116<br>2560117<br>2560118<br>2560119<br>2560120<br>2560121	extended<br>full<br>core<br>core<br>core<br>core<br>core<br>extended<br>extended<br>full<br>extended<br>core<br>core<br>core<br>extended<br>core<br>core<br>core<br>core<br>extended<br>core<br>core<br>core<br>core<br>extended<br>extended<br>extended<br>extended<br>extended<br>core<br>extended<br>full<br>full<br>core<br>core<br>extended<br>extended<br>extended<br>full<br>full<br>core<br>core<br>extended<br>extended<br>extended	2	74504785	74523034	-	NM_033046<br>NM_001015055<br>NM_001015056<br>ENSESTT00000037314<br>ENSESTT00000037315<br>ENSESTT00000037316<br>ENSESTT00000037317<br>ENSESTT00000037318<br>ENST00000305557<br>ENST00000272430<br>ENST00000233330<br>GENSCAN00000039288	Homo sapiens rhotekin (RTKN), transcript variant 2, mRNA.<br>Homo sapiens rhotekin (RTKN), transcript variant 1, mRNA.<br>Homo sapiens rhotekin (RTKN), transcript variant 3, mRNA.<br><br><br><br><br><br>cdna:known-ccds chromosome:NCBI36:2:74506497:74521218:-1 gene:ENSG00000114993 CCDS1941.1<br>cdna:known chromosome:NCBI36:2:74506497:74522568:-1 gene:ENSG00000114993<br>cdna:known chromosome:NCBI36:2:74506497:74521218:-1 gene:ENSG00000114993<br>cdna:Genscan chromosome:NCBI36:2:74506834:74522451:-1

2570193	158069<br>158070<br>158071<br>158072<br>158073<br>158074<br>158075<br>158076<br>158077<br>158078<br>158079<br>158080<br>158081<br>158082<br>158083<br>158084<br>158085<br>158086<br>158087<br>158088<br>158089<br>158090<br>158091<br>158092<br>158093<br>158094<br>158095<br>158096<br>158097<br>158098<br>158099<br>158100	2570194<br>2570195<br>2570196<br>2570197<br>2570198<br>2570199<br>2570200<br>2570201<br>2570202<br>2570203<br>2570204<br>2570205<br>2570206<br>2570207<br>2570208<br>2570209<br>2570210<br>2570211<br>2570212<br>2570213<br>2570214<br>2570215<br>2570216<br>2570217<br>2570218<br>2570219<br>2570220<br>2570221<br>2570222<br>2570223<br>2570224<br>2570225<br>2570226<br>2570227<br>2570228<br>2570229<br>2570230<br>2570231<br>2570232<br>2570233	core<br>core<br>core<br>core<br>extended<br>extended<br>core<br>full<br>full<br>full<br>core<br>extended<br>extended<br>full<br>full<br>extended<br>full<br>extended<br>full<br>full<br>extended<br>extended<br>full<br>extended<br>extended<br>full<br>extended<br>extended<br>extended<br>extended<br>extended<br>extended<br>extended<br>extended<br>full<br>full<br>extended<br>extended<br>extended<br>extended	2	110198740	110616256	-	NM_005434<br>ENSESTT00000036525<br>ENSESTT00000036526<br>ENST00000272462<br>GENSCAN00000054913	Homo sapiens mal, T-cell differentiation protein-like (MALL), mRNA.<br><br><br>cdna:known-ccds chromosome:NCBI36:2:110198738:110231432:-1 gene:ENSG00000144063 CCDS2085.1<br>cdna:Genscan chromosome:NCBI36:2:110200561:110230658:-1
2570616	158325<br>158326<br>158327<br>158328<br>158329<br>158330<br>158331<br>158332<br>158333<br>158334<br>158335<br>158336<br>158337<br>158338	2570617<br>2570618<br>2570619<br>2570620<br>2570621<br>2570622<br>2570623<br>2570624<br>2570625<br>2570626<br>2570627<br>2570628<br>2570629<br>2570630	full<br>full<br>extended<br>extended<br>extended<br>extended<br>full<br>extended<br>extended<br>extended<br>extended<br>core<br>core<br>core	2	111088456	111174950	-	NM_004336<br>AK023540<br>ENSESTT00000019182<br>ENST00000302759<br>ENST00000389945<br>ENST00000389944<br>GENSCAN00000013627	Homo sapiens BUB1 budding uninhibited by benzimidazoles 1 homolog (yeast) (BUB1), mRNA.<br>Homo sapiens cDNA FLJ13478 fis, clone PLACE1003709, highly similar to Homo sapiens mitotic checkpoint kinase Bub1 (BUB1) mRNA.<br><br>cdna:known chromosome:NCBI36:2:111111883:111152135:-1 gene:ENSG00000169679<br>cdna:known chromosome:NCBI36:2:111112013:111152043:-1 gene:ENSG00000169679<br>cdna:known chromosome:NCBI36:2:111112013:111152043:-1 gene:ENSG00000169679<br>cdna:Genscan chromosome:NCBI36:2:111112013:111194776:-1

	158339	2570631	extended						
	158340	2570632	extended						
	158341	2570633	core						
	158342	2570634	core						
	158343	2570635	core						
	158344	2570636	full						
	158345	2570637	core						
	158346	2570638	full						
	158347	2570639	full						
	158348	2570640	core						
	158349	2570641	extended						
	158350	2570642	extended						
	158351	2570643	core						
	158352	2570644	extended						
	158353	2570645	extended						
	158354	2570646	core						
	158355	2570647	core						
	158356	2570648	extended						
	158357	2570649	core						
	158358	2570650	core						
	158359	2570651	core						
	158360	2570652	core						
	158361	2570653	core						
	158362	2570654	extended						
	158363	2570655	core						
	158364	2570656	extended						
	158365	2570657	core						
	158366	2570658	extended						
	158367	2570659	core						
	158368	2570660	extended						
	158369	2570661	extended						
	158370	2570662	extended						
	158371	2570663	core						
	158372	2570664	core						
		2570665	core						
		2570666	extended						
		2570667	extended						
		2570668	core						
		2570669	core						
		2570670	core						
		2570671	extended						
		2570672	extended						
		2570673	core						
		2570674	core						
		2570675	core						
		2570676	core						
		2570677	core						
		2570678	core						
		2570679	full						
		2570680	full						
		2570681	full						
		2570682	full						
		2570683	full						
		2570684	full						

		2570685<br>2570686	full<br>full						
2574984	161021<br>161022<br>161023<br>161024<br>161025<br>161026<br>161027<br>161028<br>161029<br>161030<br>161031<br>161032<br>161033<br>161034<br>161035<br>161036<br>161037<br>161038<br>161039<br>161040<br>161041<br>161042<br>161043<br>161044<br>161045<br>161046<br>161047<br>161048<br>161049	2574985<br>2574986<br>2574987<br>2574988<br>2574989<br>2574990<br>2574991<br>2574992<br>2574993<br>2574994<br>2574995<br>2574996<br>2574997<br>2574998<br>2574999<br>2575000<br>2575001<br>2575002<br>2575003<br>2575004<br>2575005<br>2575006<br>2575007<br>2575008<br>2575009<br>2575010<br>2575011<br>2575012<br>2575013<br>2575014<br>2575015<br>2575016<br>2575017<br>2575018<br>2575019<br>2575020<br>2575021<br>2575022<br>2575023<br>2575024<br>2575025<br>2575026<br>2575027<br>2575028<br>2575029<br>2575030<br>2575031<br>2575032<br>2575033<br>2575034<br>2575035<br>2575036	full<br>full<br>core<br>core<br>core<br>core<br>core<br>core<br>core<br>core<br>core<br>core<br>core<br>extended<br>core<br>core<br>extended<br>core<br>extended<br>extended<br>core<br>extended<br>core<br>core<br>core<br>core<br>core<br>extended<br>extended<br>full<br>core<br>extended<br>full<br>extended<br>core<br>core<br>core<br>extended<br>extended<br>extended<br>extended<br>full<br>core<br>extended<br>extended<br>core<br>core<br>extended<br>full<br>full<br>core<br>core	2	128112306	128155497	-	NM_017980<br>AF527766<br>AF527767<br>AF527768<br>AF527769<br>AF527770<br>AK055363<br>AK091652<br>AK093692<br>AK094954<br>AK095790<br>AK097026<br>AK123014<br>CR592921<br>ENSESTT00000037033<br>ENSESTT00000037034<br>ENSESTT00000037035<br>ENSESTT00000037036<br>ENSESTT00000037037<br>ENST00000324938<br>ENST00000324864<br>ENST00000342067<br>ENST00000355119<br>GENSCAN00000038207<br>GENSCAN00000008986	Homo sapiens LIM and senescent cell antigen-like domains 2 (LIMS2), mRNA.<br>Homo sapiens tissue-type spleen LIM-like protein 2C mRNA, complete cds.<br>Homo sapiens tissue-type uterus LIM-like protein 2D mRNA, complete cds.<br>Homo sapiens tissue-type brain LIM-like protein 2E mRNA, complete cds.<br>Homo sapiens tissue-type brain LIM-like protein 2F mRNA, complete cds.<br>Homo sapiens tissue-type brain LIM-like protein 2G mRNA, complete cds.<br>Homo sapiens cDNA FLJ30801 fis, clone FEBRA2001217, highly similar to PINCH PROTEIN.<br>Homo sapiens cDNA FLJ34333 fis, clone FEBRA2009276, highly similar to PINCH PROTEIN.<br>Homo sapiens cDNA FLJ36373 fis, clone THYMU2008111, moderately similar to PINCH PROTEIN.<br>Homo sapiens cDNA FLJ37635 fis, clone BRCOC2017856, highly similar to PINCH PROTEIN.<br>Homo sapiens cDNA FLJ38471 fis, clone FEBRA2022055, highly similar to PINCH PROTEIN.<br>Homo sapiens cDNA FLJ39707 fis, clone SMINT2012734.<br>Homo sapiens cDNA FLJ16826 fis, clone UTERU3006687, highly similar to Homo sapiens LIM and senescent cell antigen-like domains 1 (LIMS1).<br>full-length cDNA clone CS0DJ014YK07 of T cells (Jurkat cell line) Cot 10-normalized of Homo sapiens (human).<br><br><br><br><br><br>cdna:known-ccds chromosome:NCBI36:2:128112475:128138590:-1 gene:ENSG00000072163 CCDS2147.1<br>cdna:known chromosome:NCBI36:2:128112473:128132267:-1 gene:ENSG00000072163<br>cdna:known chromosome:NCBI36:2:128112475:128118157:-1 gene:ENSG00000072163<br>cdna:known chromosome:NCBI36:2:128113093:128155507:-1 gene:ENSG00000072163<br>cdna:Genscan chromosome:NCBI36:2:128113326:128119675:-1<br>cdna:Genscan chromosome:NCBI36:2:128127128:128155062:-1

		2575037<br>2575038<br>2575039<br>2575040<br>2575041<br>2575042<br>2575043<br>2575044<br>2575045	extended<br>extended<br>extended<br>full<br>full<br>extended<br>extended<br>full<br>core						
2584134	166741<br>166742<br>166743<br>166744<br>166745<br>166746<br>166747<br>166748<br>166749<br>166750<br>166751<br>166752<br>166753<br>166754<br>166755<br>166756<br>166757<br>166758<br>166759<br>166760<br>166761<br>166762<br>166763<br>166764<br>166765<br>166766<br>166767<br>166768<br>166769<br>166770<br>166771<br>166772<br>166773<br>166774<br>166775<br>166776<br>166777<br>166778<br>166779<br>166780<br>166781<br>166782<br>166783<br>166784<br>166785	2584135<br>2584136<br>2584137<br>2584138<br>2584139<br>2584140<br>2584141<br>2584142<br>2584143<br>2584144<br>2584145<br>2584146<br>2584147<br>2584148<br>2584149<br>2584150<br>2584151<br>2584152<br>2584153<br>2584154<br>2584155<br>2584156<br>2584157<br>2584158<br>2584159<br>2584160<br>2584161<br>2584162<br>2584163<br>2584164<br>2584165<br>2584166<br>2584167<br>2584168<br>2584169<br>2584170<br>2584171<br>2584172<br>2584173<br>2584174<br>2584175<br>2584176<br>2584177<br>2584178<br>2584179	core<br>core<br>core<br>core<br>core<br>core<br>extended<br>extended<br>extended<br>core<br>extended<br>full<br>full<br>core<br>core<br>core<br>extended<br>core<br>extended<br>extended<br>extended<br>full<br>core<br>full<br>extended<br>extended<br>extended<br>full<br>extended<br>core<br>full<br>core<br>full<br>core<br>full<br>core<br>extended<br>extended<br>extended<br>extended<br>extended<br>extended<br>core<br>core<br>core	2	162735446	162809835	-	NM_022168<br>NM_004460<br>NM_002054<br>AK055327<br>AL832166<br>ENSESTT00000006312<br>ENSESTT00000006315<br>ENSESTT00000050859<br>ENSESTT00000050860<br>ENSESTT00000050861<br>ENSESTT00000050862<br>ENSESTT00000050863<br>ENSESTT00000050864<br>ENST00000263642<br>ENST00000188790<br>GENSCAN00000033018	Homo sapiens interferon induced with helicase C domain 1 (IFIH1), mRNA.<br>Homo sapiens fibroblast activation protein, alpha (FAP), mRNA.<br>Homo sapiens glucagon (GCG), mRNA.<br>Homo sapiens cDNA FLJ30765 fis, clone FEBRA2000659, highly similar to Human fibroblast activation protein mRNA.<br>Homo sapiens mRNA; cDNA DKFZp686G13158 (from clone DKFZp686G13158).<br><br><br><br><br><br><br><br><br>cdna:known-ccds chromosome:NCBI36:2:162831836:162883285:-1 gene:ENSG00000115267 CCDS2217.1<br>cdna:known chromosome:NCBI36:2:162735446:162808291:-1 gene:ENSG00000078098<br>cdna:Genscan chromosome:NCBI36:2:162692879:162846301:-1

	166786<br>166787	2584180<br>2584181<br>2584182<br>2584183<br>2584184<br>2584185<br>2584186<br>2584187<br>2584188<br>2584189<br>2584190<br>2584191<br>2584192<br>2584193<br>2584194<br>2584195	core<br>core<br>extended<br>core<br>extended<br>core<br>core<br>core<br>full<br>core<br>extended<br>core<br>core<br>full<br>full<br>full						
2604254	179728<br>179729<br>179730<br>179731<br>179732<br>179733<br>179734<br>179735<br>179736<br>179737<br>179738<br>179739<br>179740<br>179741<br>179742<br>179743<br>179744<br>179745	2604255<br>2604256<br>2604257<br>2604258<br>2604259<br>2604260<br>2604261<br>2604262<br>2604263<br>2604264<br>2604265<br>2604266<br>2604267<br>2604268<br>2604269<br>2604270<br>2604271<br>2604272<br>2604273<br>2604274<br>2604275<br>2604276<br>2604277<br>2604278<br>2604279<br>2604280	extended<br>full<br>extended<br>core<br>core<br>core<br>core<br>full<br>core<br>core<br>core<br>core<br>core<br>core<br>core<br>core<br>extended<br>extended<br>core<br>core<br>extended<br>core<br>full<br>core<br>core<br>extended	2	234406876	234427931	-	NM_018410<br>ENSESTT00000035627<br>ENSESTT00000035628<br>ENST00000373395<br>ENST00000243201<br>GENSCAN00000015883<br>GENSCAN00000015881	Homo sapiens hypothetical protein DKFZp762E1312 (DKFZp762E1312), mRNA.<br><br><br>cdna:known chromosome:NCBI36:2:234410225:234427951:-1 gene:ENSG00000123485<br>cdna:known chromosome:NCBI36:2:234410746:234427917:-1 gene:ENSG00000123485<br>cdna:Genscan chromosome:NCBI36:2:234412902:234415492:-1<br>cdna:Genscan chromosome:NCBI36:2:234427149:234427864:-1
2605321	180399<br>180400<br>180401<br>180402<br>180403<br>180404<br>180405<br>180406<br>180407<br>180408<br>180409	2605322<br>2605323<br>2605324<br>2605325<br>2605326<br>2605327<br>2605328<br>2605329<br>2605330<br>2605331<br>2605332	full<br>full<br>full<br>core<br>core<br>core<br>core<br>core<br>core<br>extended<br>extended	2	237897089	238019668	-	NM_057164<br>NM_004369<br>NM_057165<br>NM_057167<br>NM_057166<br>BX641155<br>BX647361<br>S49432<br>BX647500<br>ENSESTT00000018422<br>ENSESTT00000018423	Homo sapiens collagen, type VI, alpha 3 (COL6A3), transcript variant 2, mRNA.<br>Homo sapiens collagen, type VI, alpha 3 (COL6A3), transcript variant 1, mRNA.<br>Homo sapiens collagen, type VI, alpha 3 (COL6A3), transcript variant 3, mRNA.<br>Homo sapiens collagen, type VI, alpha 3 (COL6A3), transcript variant 5, mRNA.<br>Homo sapiens collagen, type VI, alpha 3 (COL6A3), transcript variant 4, mRNA.<br>Homo sapiens mRNA; cDNA DKFZp686D23123 (from clone DKFZp686D23123).<br>Homo sapiens mRNA; cDNA DKFZp686N0262 (from clone DKFZp686N0262).<br>type VI collagen alpha 3 chain {5' region, alternatively spliced, exon A9/N10} [human, mRNA, 653 nt].<br>Homo sapiens mRNA; cDNA DKFZp686K04147 (from clone DKFZp686K04147).

	180410	2605333	core					ENSESTT00000018424	
	180411	2605334	core					ENSESTT00000018425	
	180412	2605335	core					ENSESTT00000018426	
	180413	2605336	core					ENSESTT00000018427	
	180414	2605337	core					ENSESTT00000018428	
	180415	2605338	core					ENSESTT00000018429	
	180416	2605339	core					ENST00000295550	cdna:known chromosome:NCBI36:2:237897401:237987559:-1 gene:ENSG00000163359
	180417	2605340	core					ENST00000346358	cdna:known chromosome:NCBI36:2:237897401:237987559:-1 gene:ENSG00000163359
	180418	2605341	full					ENST00000347401	cdna:known chromosome:NCBI36:2:237897401:237987559:-1 gene:ENSG00000163359
	180419	2605342	core					ENST00000353578	cdna:known chromosome:NCBI36:2:237897401:237987559:-1 gene:ENSG00000163359
	180420	2605343	extended					GENSCAN00000024616	cdna:Genscan chromosome:NCBI36:2:237914246:238019586:-1
	180421	2605344	extended						
	180422	2605345	extended						
	180423	2605346	core						
	180424	2605347	core						
	180425	2605348	extended						
	180426	2605349	full						
	180427	2605350	core						
	180428	2605351	core						
	180429	2605352	full						
	180430	2605353	core						
	180431	2605354	core						
	180432	2605355	core						
	180433	2605356	core						
	180434	2605357	full						
	180435	2605358	core						
	180436	2605359	core						
	180437	2605360	extended						
	180438	2605361	extended						
	180439	2605362	extended						
	180440	2605363	extended						
	180441	2605364	core						
	180442	2605365	extended						
	180443	2605366	core						
	180444	2605367	core						
	180445	2605368	core						
	180446	2605369	core						
	180447	2605370	core						
	180448	2605371	core						
	180449	2605372	full						
	180450	2605373	core						
	180451	2605374	core						
	180452	2605375	core						
	180453	2605376	core						
	180454	2605377	core						
	180455	2605378	core						
	180456	2605379	core						
	180457	2605380	core						
	180458	2605381	extended						
	180459	2605382	core						
	180460	2605383	core						
	180461	2605384	core						
	180462	2605385	core						
	180463	2605386	core						

	180464<br>180465<br>180466<br>180467<br>180468<br>180469	2605387<br>2605388<br>2605389<br>2605390<br>2605391<br>2605392<br>2605393<br>2605394<br>2605395<br>2605396<br>2605397<br>2605398<br>2605399<br>2605400<br>2605401<br>2605402<br>2605403<br>2605404<br>2605405<br>2605406<br>2605407<br>2605408<br>2605409<br>2605410<br>2605411<br>2605412<br>2605413<br>2605414<br>2605415<br>2605416<br>2605417<br>2605418<br>2605419	core<br>extended<br>extended<br>core<br>core<br>full<br>extended<br>full<br>core<br>core<br>extended<br>core<br>core<br>core<br>full<br>extended<br>extended<br>extended<br>extended<br>extended<br>extended<br>core<br>full<br>extended<br>full<br>full<br>full<br>full<br>full<br>full<br>full<br>full<br>full						
2611848	184517<br>184518<br>184519<br>184520<br>184521<br>184522<br>184523<br>184524<br>184525<br>184526<br>184527<br>184528<br>184529<br>184530<br>184531<br>184532<br>184533<br>184534<br>184535<br>184536<br>184537	2611849<br>2611850<br>2611851<br>2611852<br>2611853<br>2611854<br>2611855<br>2611856<br>2611857<br>2611858<br>2611859<br>2611860<br>2611861<br>2611862<br>2611863<br>2611864<br>2611865<br>2611866<br>2611867<br>2611868<br>2611869	full<br>full<br>extended<br>full<br>full<br>full<br>full<br>full<br>full<br>full<br>full<br>full<br>full<br>full<br>full<br>extended<br>extended<br>full<br>full<br>full<br>full	3	14249186	14505858	+	NM_003043<br>AK023516<br>BC038790<br>BC111489<br>U16120<br>ENSESTT00000045726<br>ENSESTT00000045727<br>ENSESTT00000045728<br>ENST00000253707<br>ENST00000360861<br>ENST00000388984<br>ENST00000388983<br>GENSCAN00000039280	Homo sapiens solute carrier family 6 (neurotransmitter transporter, taurine), member 6 (SLC6A6), mRNA.<br>Homo sapiens cDNA FLJ13454 fis, clone PLACE1003249.<br>Homo sapiens cDNA clone IMAGE:5271875.<br>Homo sapiens solute carrier family 6 (neurotransmitter transporter, taurine), member 6, mRNA (cDNA clone IMAGE:5755891), complete cds.<br>Human placental taurine transporter mRNA, complete cds.<br><br><br><br><br>cdna:known chromosome:NCBI36:3:14419110:14503971:1 gene:ENSG00000131389<br>cdna:known chromosome:NCBI36:3:14419154:14505859:1 gene:ENSG00000131389<br>cdna:known chromosome:NCBI36:3:14460147:14501519:1 gene:ENSG00000131389<br>cdna:known chromosome:NCBI36:3:14460147:14501519:1 gene:ENSG00000131389<br>cdna:Genscan chromosome:NCBI36:3:14198139:14263413:1

	184538	2611870	full						
	184539	2611871	full						
	184540	2611872	full						
	184541	2611873	full						
	184542	2611874	full						
	184543	2611875	full						
	184544	2611876	full						
	184545	2611877	full						
	184546	2611878	full						
	184547	2611879	full						
	184548	2611880	full						
	184549	2611881	extended						
	184550	2611882	extended						
	184551	2611883	full						
	184552	2611884	extended						
	184553	2611885	extended						
	184554	2611886	full						
	184555	2611887	full						
	184556	2611888	full						
	184557	2611889	extended						
	184558	2611890	full						
	184559	2611891	core						
	184560	2611892	full						
	184561	2611893	full						
	184562	2611894	extended						
	184563	2611895	extended						
	184564	2611896	extended						
	184565	2611897	extended						
	184566	2611898	extended						
	184567	2611899	extended						
	184568	2611900	extended						
	184569	2611901	extended						
	184570	2611902	full						
	184571	2611903	full						
	184572	2611904	full						
	184573	2611905	full						
	184574	2611906	full						
	184575	2611907	full						
	184576	2611908	full						
	184577	2611909	full						
	184578	2611910	extended						
	184579	2611911	extended						
	184580	2611912	extended						
	184581	2611913	full						
	184582	2611914	full						
	184583	2611915	full						
	184584	2611916	full						
	184585	2611917	core						
	184586	2611918	extended						
	184587	2611919	core						
	184588	2611920	extended						
	184589	2611921	core						
	184590	2611922	extended						
	184591	2611923	extended						

	184592<br>184593<br>184594<br>184595<br>184596<br>184597<br>184598<br>184599<br>184600	2611924<br>2611925<br>2611926<br>2611927<br>2611928<br>2611929<br>2611930<br>2611931<br>2611932<br>2611933<br>2611934<br>2611935<br>2611936<br>2611937<br>2611938<br>2611939<br>2611940<br>2611941<br>2611942<br>2611943<br>2611944<br>2611945<br>2611946	extended<br>full<br>core<br>full<br>full<br>extended<br>extended<br>extended<br>extended<br>core<br>core<br>full<br>core<br>core<br>core<br>full<br>core<br>core<br>core<br>full<br>extended<br>core<br>extended						
2625793	193063<br>193064<br>193065<br>193066<br>193067<br>193068<br>193069<br>193070<br>193071<br>193072<br>193073<br>193074<br>193075<br>193076<br>193077<br>193078<br>193079<br>193080<br>193081<br>193082<br>193083<br>193084<br>193085<br>193086<br>193087<br>193088<br>193089<br>193090<br>193091<br>193092<br>193093	2625794<br>2625795<br>2625796<br>2625797<br>2625798<br>2625799<br>2625800<br>2625801<br>2625802<br>2625803<br>2625804<br>2625805<br>2625806<br>2625807<br>2625808<br>2625809<br>2625810<br>2625811<br>2625812<br>2625813<br>2625814<br>2625815<br>2625816<br>2625817<br>2625818<br>2625819<br>2625820<br>2625821<br>2625822<br>2625823<br>2625824	full<br>extended<br>extended<br>core<br>core<br>core<br>extended<br>full<br>extended<br>extended<br>extended<br>full<br>extended<br>extended<br>full<br>extended<br>extended<br>full<br>full<br>full<br>core<br>extended<br>extended<br>extended<br>full<br>core<br>core<br>core<br>extended<br>extended<br>core	3	57716908	57933826	+	NM_007159<br>AF304450<br>AK022561<br>AK124200<br>AY358410<br>BC029462<br>CR627321<br>AF100750<br>ENSESTT00000002082<br>ENSESTT00000002083<br>ENSESTT00000002084<br>ENSESTT00000002085<br>ENSESTT00000002086<br>ENSESTT00000038019<br>ENSESTT00000038020<br>ENSESTT00000038021<br>ENST00000383719<br>ENST00000295952<br>ENST00000383718<br>ENST00000295951<br>ENST00000383717<br>GENSCAN00000032567<br>GENSCAN00000032566<br>GENSCAN00000032562	Homo sapiens sarcolemma associated protein (SLMAP), mRNA.<br>Homo sapiens sarcolemmal associated protein 1 mRNA, complete cds, alternatively spliced.<br>Homo sapiens cDNA FLJ12499 fis, clone NT2RM2001671, highly similar to Oryctolagus cuniculus sarcolemmal associated protein (SLAP1) mRNA.<br>Homo sapiens cDNA FLJ42206 fis, clone THYMU2035735, highly similar to Oryctolagus cuniculus sarcolemmal associated protein-3 mRNA.<br>Homo sapiens clone DNA53991 SLAP (UNQ1847) mRNA, complete cds.<br>Homo sapiens, clone IMAGE:4663772, mRNA.<br>Homo sapiens mRNA; cDNA DKFZp779I1058 (from clone DKFZp779I1058).<br>Homo sapiens SLAP-2 homolog mRNA, complete cds.<br>cdna:known chromosome:NCBI36:3:57718068:57851973:1 gene:ENSG00000163681<br>cdna:known chromosome:NCBI36:3:57718214:57889934:1 gene:ENSG00000163681<br>cdna:known chromosome:NCBI36:3:57718321:57858460:1 gene:ENSG00000163681<br>cdna:known chromosome:NCBI36:3:57825353:57888326:1 gene:ENSG00000163681<br>cdna:known chromosome:NCBI36:3:57850808:57888160:1 gene:ENSG00000163681<br>cdna:Genscan chromosome:NCBI36:3:57873114:57888155:1<br>cdna:Genscan chromosome:NCBI36:3:57818521:57869943:1<br>cdna:Genscan chromosome:NCBI36:3:57780491:57798832:1

	193094	2625825	core						
	193095	2625826	core						
	193096	2625827	extended						
	193097	2625828	core						
	193098	2625829	core						
	193099	2625830	core						
	193100	2625831	core						
	193101	2625832	extended						
	193102	2625833	extended						
	193103	2625834	extended						
	193104	2625835	extended						
	193105	2625836	extended						
	193106	2625837	extended						
	193107	2625838	extended						
	193108	2625839	extended						
	193109	2625840	extended						
	193110	2625841	core						
	193111	2625842	core						
	193112	2625843	core						
	193113	2625844	extended						
	193114	2625845	extended						
	193115	2625846	extended						
	193116	2625847	extended						
	193117	2625848	core						
	193118	2625849	extended						
	193119	2625850	core						
	193120	2625851	extended						
	193121	2625852	extended						
	193122	2625853	extended						
	193123	2625854	extended						
	193124	2625855	extended						
	193125	2625856	extended						
	193126	2625857	full						
	193127	2625858	core						
	193128	2625859	core						
	193129	2625860	full						
	193130	2625861	extended						
	193131	2625862	extended						
	193132	2625863	extended						
	193133	2625864	extended						
	193134	2625865	core						
	193135	2625866	core						
	193136	2625867	extended						
	193137	2625868	extended						
	193138	2625869	extended						
	193139	2625870	extended						
	193140	2625871	full						
	193141	2625872	core						
	193142	2625873	full						
	193143	2625874	extended						
	193144	2625875	full						
	193145	2625876	extended						
		2625877	core						
		2625878	extended						

		2625879<br>2625880<br>2625881<br>2625882<br>2625883<br>2625884<br>2625885<br>2625886<br>2625887<br>2625888<br>2625889<br>2625890<br>2625891<br>2625892<br>2625893<br>2625894<br>2625895<br>2625896<br>2625897<br>2625898<br>2625899<br>2625900<br>2625901<br>2625902<br>2625903<br>2625904	extended<br>extended<br>extended<br>extended<br>extended<br>extended<br>core<br>extended<br>extended<br>extended<br>core<br>core<br>extended<br>extended<br>extended<br>extended<br>extended<br>extended<br>full<br>extended<br>extended<br>extended<br>full<br>full<br>full<br>full						
2652675	210065<br>210066<br>210067<br>210068<br>210069<br>210070<br>210071<br>210072<br>210073<br>210074<br>210075<br>210076<br>210077<br>210078<br>210079<br>210080<br>210081<br>210082<br>210083<br>210084<br>210085<br>210086<br>210087<br>210088<br>210089<br>210090<br>210091<br>210092	2652676<br>2652677<br>2652678<br>2652679<br>2652680<br>2652681<br>2652682<br>2652683<br>2652684<br>2652685<br>2652686<br>2652687<br>2652688<br>2652689<br>2652690<br>2652691<br>2652692<br>2652693<br>2652694<br>2652695<br>2652696<br>2652697<br>2652698<br>2652699<br>2652700<br>2652701<br>2652702<br>2652703	full<br>extended<br>extended<br>core<br>core<br>core<br>core<br>extended<br>extended<br>core<br>core<br>core<br>extended<br>core<br>core<br>core<br>full<br>core<br>core<br>core<br>core<br>core<br>core<br>core<br>extended<br>core<br>core<br>core	3	173950943	174077327	+	NM_018098<br>DQ847274<br>AL137710<br>ENSESTT00000046493<br>ENSESTT00000046494<br>ENSESTT00000046495<br>ENSESTT00000046496<br>ENSESTT00000046497<br>ENSESTT00000046498<br>ENSESTT00000046499<br>ENSESTT00000046500<br>ENST00000232458<br>GENSCAN00000045148<br>GENSCAN00000044983	Homo sapiens epithelial cell transforming sequence 2 oncogene (ECT2), mRNA.<br>Homo sapiens epithelial cell transforming sequence 2 oncogene protein splice variant b (ECT2) mRNA, complete cds, alternatively spliced.<br>Homo sapiens mRNA; cDNA DKFZp434C0523 (from clone DKFZp434C0523); partial cds.<br><br><br><br><br><br><br><br><br>cdna:known-ccds chromosome:NCBI36:3:173951207:174021957:1 gene:ENSG00000114346 CCDS3220.1<br>cdna:Genscan chromosome:NCBI36:3:174043737:174077327:1<br>cdna:Genscan chromosome:NCBI36:3:173955784:174019253:1

	210093<br>210094<br>210095<br>210096<br>210097<br>210098<br>210099<br>210100<br>210101<br>210102<br>210103<br>210104<br>210105<br>210106<br>210107<br>210108<br>210109<br>210110<br>210111	2652704<br>2652705<br>2652706<br>2652707<br>2652708<br>2652709<br>2652710<br>2652711<br>2652712<br>2652713<br>2652714<br>2652715<br>2652716<br>2652717<br>2652718<br>2652719<br>2652720<br>2652721<br>2652722<br>2652723<br>2652724<br>2652725<br>2652726<br>2652727<br>2652728<br>2652729<br>2652730<br>2652731<br>2652732<br>2652733<br>2652734<br>2652735<br>2652736<br>2652737<br>2652738<br>2652739	core<br>core<br>extended<br>core<br>core<br>extended<br>full<br>extended<br>core<br>extended<br>core<br>core<br>extended<br>extended<br>extended<br>extended<br>extended<br>core<br>core<br>core<br>extended<br>extended<br>extended<br>extended<br>full<br>full<br>full<br>full<br>full<br>full<br>full<br>full<br>full<br>full<br>full<br>full						
2676009	224521<br>224522<br>224523<br>224524<br>224525<br>224526<br>224527<br>224528<br>224529<br>224530<br>224531<br>224532<br>224533<br>224534<br>224535<br>224536<br>224537	2676010<br>2676011<br>2676012<br>2676013<br>2676014<br>2676015<br>2676016<br>2676017<br>2676018<br>2676019<br>2676020<br>2676021<br>2676022<br>2676023<br>2676024<br>2676025<br>2676026<br>2676027	full<br>core<br>core<br>core<br>extended<br>extended<br>core<br>core<br>core<br>core<br>core<br>core<br>core<br>core<br>core<br>core<br>extended<br>extended	3	52237535	52254242	-	NM_007284<br>AF246973<br>AF246974<br>ENSESTT00000004508<br>ENST00000305533<br>ENST00000310209<br>GENSCAN00000007343	Homo sapiens PTK9L protein tyrosine kinase 9-like (A6-related protein) (PTK9L), mRNA.<br>Homo sapiens toll-like receptor 9 (TLR9) mRNA, partial cds, alternatively spliced.<br>Homo sapiens toll-like receptor 9 (TLR9) mRNA, partial cds, alternatively spliced.<br><br>cdna:known-ccds chromosome:NCBI36:3:52237670:52248223:-1 gene:ENSG00000173366 CCDS2849.1<br>cdna:known chromosome:NCBI36:3:52230273:52238999:-1 gene:ENSG00000173366<br>cdna:Genscan chromosome:NCBI36:3:52238090:52241178:-1

		2676028	extended						
		2676029	full						
		2676030	core						
		2676031	core						
		2676032	core						
		2676033	full						
		2676034	full						
2686458	231322	2686459	extended	3	101950692	102195002	-	NM_015429	Homo sapiens ABI gene family, member 3 (NESH) binding protein (ABI3BP), mRNA.
	231323	2686460	core					AB056106	Homo sapiens mRNA for NeshBP, complete cds.
	231324	2686461	core					AK025204	Homo sapiens cDNA: FLJ21551 fis, clone COL06266.
	231325	2686462	core					AK123737	Homo sapiens cDNA FLJ41743 fis, clone HSYRA2005456.
	231326	2686463	extended					AK123748	Homo sapiens cDNA FLJ41754 fis, clone HSYRA2009075.
	231327	2686464	core					BX648726	Homo sapiens mRNA; cDNA DKFZp686A12242 (from clone DKFZp686A12242).
	231328	2686465	core					AL833204	Homo sapiens mRNA; cDNA DKFZp667H216 (from clone DKFZp667H216).
	231329	2686466	core					ENSESTT00000007182	
	231330	2686467	extended					ENSESTT00000007183	
	231331	2686468	full					ENSESTT00000007184	
	231332	2686469	core					ENSESTT00000007185	
	231333	2686470	full					ENSESTT00000007186	
	231334	2686471	core					ENST00000284322	cdna:known chromosome:NCBI36:3:101950871:102194939:-1 gene:ENSG00000154175
	231335	2686472	core					ENST00000383692	cdna:known chromosome:NCBI36:3:101951990:102038712:-1 gene:ENSG00000154175
	231336	2686473	core					ENST00000383691	cdna:known chromosome:NCBI36:3:101951990:102019008:-1 gene:ENSG00000154175
	231337	2686474	core					ENST00000273339	cdna:known chromosome:NCBI36:3:102017553:102194939:-1 gene:ENSG00000154175
	231338	2686475	core					GENSCAN00000036759	cdna:Genscan chromosome:NCBI36:3:102066135:102068490:-1
	231339	2686476	core					GENSCAN00000034994	cdna:Genscan chromosome:NCBI36:3:101952029:101967675:-1
	231340	2686477	core					GENSCAN00000042526	cdna:Genscan chromosome:NCBI36:3:102100296:102153390:-1
	231341	2686478	extended						
	231342	2686479	core						
	231343	2686480	core						
	231344	2686481	core						
	231345	2686482	extended						
	231346	2686483	core						
	231347	2686484	extended						
	231348	2686485	full						
	231349	2686486	extended						
	231350	2686487	core						
	231351	2686488	full						
	231352	2686489	full						
	231353	2686490	extended						
	231354	2686491	core						
	231355	2686492	extended						
	231356	2686493	extended						
	231357	2686494	extended						
	231358	2686495	extended						
	231359	2686496	extended						
	231360	2686497	extended						
	231361	2686498	extended						
	231362	2686499	extended						
	231363	2686500	extended						
	231364	2686501	extended						
	231365	2686502	extended						
	231366	2686503	extended						
	231367	2686504	full						
	231368	2686505	full						

	231369	2686506	extended						
	231370	2686507	extended						
	231371	2686508	extended						
	231372	2686509	extended						
	231373	2686510	full						
	231374	2686511	extended						
	231375	2686512	extended						
	231376	2686513	extended						
	231377	2686514	extended						
	231378	2686515	extended						
	231379	2686516	full						
	231380	2686517	extended						
	231381	2686518	extended						
	231382	2686519	extended						
	231383	2686520	extended						
	231384	2686521	extended						
	231385	2686522	extended						
	231386	2686523	extended						
	231387	2686524	extended						
	231388	2686525	extended						
	231389	2686526	extended						
	231390	2686527	extended						
	231391	2686528	extended						
	231392	2686529	extended						
	231393	2686530	extended						
	231394	2686531	core						
	231395	2686532	core						
	231396	2686533	core						
	231397	2686534	core						
	231398	2686535	core						
	231399	2686536	extended						
	231400	2686537	core						
	231401	2686538	core						
	231402	2686539	full						
	231403	2686540	full						
	231404	2686541	core						
	231405	2686542	extended						
	231406	2686543	extended						
	231407	2686544	extended						
	231408	2686545	extended						
	231409	2686546	extended						
	231410	2686547	extended						
	231411	2686548	extended						
	231412	2686549	core						
	231413	2686550	extended						
	231414	2686551	core						
	231415	2686552	full						
	231416	2686553	core						
		2686554	core						
		2686555	core						
		2686556	full						
		2686557	core						
		2686558	core						
		2686559	core						

		2686560<br>2686561<br>2686562<br>2686563<br>2686564<br>2686565<br>2686566<br>2686567<br>2686568<br>2686569<br>2686570<br>2686571<br>2686572<br>2686573<br>2686574<br>2686575<br>2686576<br>2686577<br>2686578<br>2686579	core<br>full<br>extended<br>extended<br>extended<br>extended<br>extended<br>extended<br>full<br>full<br>full<br>full<br>full<br>core<br>full<br>full<br>extended<br>full<br>core<br>core						
2690956	234168<br>234169<br>234170<br>234171<br>234172<br>234173<br>234174<br>234175<br>234176<br>234177<br>234178<br>234179	2690957<br>2690958<br>2690959<br>2690960<br>2690961<br>2690962<br>2690963<br>2690964<br>2690965<br>2690966<br>2690967<br>2690968<br>2690969<br>2690970<br>2690971<br>2690972<br>2690973<br>2690974<br>2690975<br>2690976<br>2690977<br>2690978	extended<br>extended<br>core<br>core<br>core<br>extended<br>extended<br>extended<br>core<br>extended<br>extended<br>core<br>core<br>core<br>extended<br>extended<br>extended<br>core<br>core<br>core<br>extended<br>core	3	120838014	120866860	-	NM_022135<br>AK124602<br>BC026911<br>ENSESTT00000018414<br>ENST00000264231<br>ENST00000341124<br>GENSCAN00000017732	Homo sapiens popeye domain containing 2 (POPDC2), mRNA.<br>Homo sapiens cDNA FLJ42611 fis, clone BRACE3013740.<br>Homo sapiens popeye domain containing 2, mRNA (cDNA clone IMAGE:4517469), complete cds.<br><br>cdna:known-ccds chromosome:NCBI36:3:120843596:120862127:-1 gene:ENSG00000121577 CCDS2992.1<br>cdna:known chromosome:NCBI36:3:120843596:120861960:-1 gene:ENSG00000121577<br>cdna:Genscan chromosome:NCBI36:3:120849699:120861960:-1
2692319	234969<br>234970<br>234971<br>234972<br>234973<br>234974<br>234975<br>234976<br>234977<br>234978<br>234979	2692320<br>2692321<br>2692322<br>2692323<br>2692324<br>2692325<br>2692326<br>2692327<br>2692328<br>2692329<br>2692330	extended<br>core<br>core<br>full<br>full<br>full<br>full<br>core<br>extended<br>core<br>full	3	124483836	124650490	-	NM_183357<br>AK093840<br>AK098381<br>AK124691<br>ENSESTT00000001512<br>ENSESTT00000001513<br>ENST00000309879<br>GENSCAN00000030501<br>GENSCAN00000005047	Homo sapiens adenylate cyclase 5 (ADCY5), mRNA.<br>Homo sapiens cDNA FLJ36521 fis, clone TRACH2002138, highly similar to ADENYLATE CYCLASE, TYPE V (EC 4.6.1.1).<br>Homo sapiens cDNA FLJ25515 fis, clone CBR06479.<br>Homo sapiens cDNA FLJ42701 fis, clone BRAMY3004919, highly similar to Adenylate cyclase, type V (EC 4.6.1.1).<br><br><br>cdna:known-ccds chromosome:NCBI36:3:124486089:124650082:-1 gene:ENSG00000173175 CCDS3022.1<br>cdna:Genscan chromosome:NCBI36:3:124486145:124554362:-1<br>cdna:Genscan chromosome:NCBI36:3:124633806:124651441:-1

	234980	2692331	core						
	234981	2692332	full						
	234982	2692333	full						
	234983	2692334	full						
	234984	2692335	core						
	234985	2692336	extended						
	234986	2692337	extended						
	234987	2692338	extended						
	234988	2692339	core						
	234989	2692340	extended						
	234990	2692341	extended						
	234991	2692342	core						
	234992	2692343	core						
	234993	2692344	full						
	234994	2692345	core						
	234995	2692346	core						
	234996	2692347	core						
	234997	2692348	full						
	234998	2692349	full						
	234999	2692350	full						
	235000	2692351	core						
	235001	2692352	core						
	235002	2692353	core						
	235003	2692354	full						
	235004	2692355	core						
	235005	2692356	full						
	235006	2692357	core						
	235007	2692358	core						
	235008	2692359	core						
	235009	2692360	full						
	235010	2692361	core						
	235011	2692362	full						
	235012	2692363	extended						
	235013	2692364	core						
	235014	2692365	full						
	235015	2692366	core						
	235016	2692367	full						
	235017	2692368	full						
	235018	2692369	full						
	235019	2692370	full						
	235020	2692371	extended						
	235021	2692372	extended						
	235022	2692373	full						
	235023	2692374	full						
		2692375	extended						
		2692376	full						
		2692377	full						
		2692378	extended						
		2692379	extended						
		2692380	extended						
		2692381	extended						
		2692382	extended						
		2692383	full						
		2692384	full						

		2692385<br>2692386<br>2692387<br>2692388<br>2692389<br>2692390	full<br>full<br>core<br>core<br>extended<br>extended						
2710474	246357<br>246358<br>246359<br>246360<br>246361<br>246362<br>246363<br>246364<br>246365<br>246366<br>246367<br>246368<br>246369<br>246370<br>246371<br>246372<br>246373<br>246374<br>246375<br>246376<br>246377<br>246378<br>246379<br>246380<br>246381<br>246382<br>246383<br>246384<br>246385<br>246386<br>246387<br>246388<br>246389<br>246390<br>246391<br>246392<br>246393<br>246394<br>246395<br>246396<br>246397<br>246398<br>246399<br>246400<br>246401<br>246402<br>246403<br>246404	2710475<br>2710476<br>2710477<br>2710478<br>2710479<br>2710480<br>2710481<br>2710482<br>2710483<br>2710484<br>2710485<br>2710486<br>2710487<br>2710488<br>2710489<br>2710490<br>2710491<br>2710492<br>2710493<br>2710494<br>2710495<br>2710496<br>2710497<br>2710498<br>2710499<br>2710500<br>2710501<br>2710502<br>2710503<br>2710504<br>2710505<br>2710506<br>2710507<br>2710508<br>2710509<br>2710510<br>2710511<br>2710512<br>2710513<br>2710514<br>2710515<br>2710516<br>2710517<br>2710518<br>2710519<br>2710520<br>2710521<br>2710522	extended<br>core<br>core<br>extended<br>full<br>full<br>extended<br>extended<br>core<br>core<br>extended<br>extended<br>extended<br>extended<br>extended<br>full<br>full<br>core<br>extended<br>core<br>core<br>core<br>extended<br>core<br>full<br>full<br>full<br>core<br>core<br>core<br>core<br>core<br>full<br>extended<br>core<br>core<br>core<br>extended<br>extended<br>full<br>extended<br>full<br>full<br>full<br>extended<br>full<br>extended<br>free	3	191157213	191329336	-	NM_018192<br>ENSESTT00000046375<br>ENSESTT00000046376<br>ENSESTT00000046377<br>ENSESTT00000046378<br>ENSESTT00000046379<br>ENSESTT00000046380<br>ENSESTT00000046381<br>ENSESTT00000046382<br>ENST00000319332<br>GENSCAN00000003188<br>GENSCAN00000031374<br>GENSCAN00000045486	Homo sapiens leprecan-like 1 (LEPREL1), mRNA.<br><br><br><br><br><br><br><br><br>cdna:known-ccds chromosome:NCBI36:3:191157213:191321407:-1 gene:ENSG00000090530 CCDS3294.1<br>cdna:Genscan chromosome:NCBI36:3:191260068:191321214:-1<br>cdna:Genscan chromosome:NCBI36:3:191162190:191201552:-1<br>cdna:Genscan chromosome:NCBI36:3:191104112:191157788:-1

	246405<br>246406<br>246407<br>246408	2710523<br>2710524<br>2710525<br>2710526<br>2710527<br>2710528<br>2710529<br>2710530<br>2710531<br>2710532<br>2710533<br>2710534<br>2710535<br>2710536<br>2710537<br>2710538<br>2710539<br>2710540<br>2710541<br>2710542<br>2710543<br>2710544<br>2710545<br>2710546<br>2710547<br>2710548<br>2710549<br>2710550<br>2710551	full<br>full<br>full<br>extended<br>extended<br>extended<br>extended<br>extended<br>extended<br>extended<br>full<br>full<br>extended<br>extended<br>full<br>full<br>core<br>core<br>core<br>core<br>core<br>core<br>core<br>core<br>extended<br>full<br>full<br>full<br>full						
2710599	246434<br>246435<br>246436<br>246437<br>246438<br>246439<br>246440<br>246441<br>246442<br>246443<br>246444<br>246445	2710600<br>2710601<br>2710602<br>2710603<br>2710604<br>2710605<br>2710606<br>2710607<br>2710608<br>2710609<br>2710610<br>2710611<br>2710612<br>2710613<br>2710614<br>2710615<br>2710616<br>2710617<br>2710618<br>2710619	full<br>full<br>extended<br>core<br>core<br>core<br>core<br>core<br>core<br>core<br>extended<br>core<br>core<br>extended<br>extended<br>extended<br>full<br>full<br>core<br>extended	3	191483506	191522932	-	NM_021101<br>AF134160<br>AK225963<br>ENSESTT00000014978<br>ENSESTT00000014979<br>ENSESTT00000014980<br>ENST00000295522<br>GENSCAN00000062238<br>GENSCAN00000018618	Homo sapiens claudin 1 (CLDN1), mRNA.<br>Homo sapiens claudin-1 (CLDN1) mRNA, complete cds.<br>Homo sapiens mRNA for claudin 1 variant, clone: FCC114C08.<br><br><br><br>cdna:known-ccds chromosome:NCBI36:3:191506197:191522909:-1 gene:ENSG00000163347 CCDS3295.1<br>cdna:Genscan chromosome:NCBI36:3:191508760:191522689:-1<br>cdna:Genscan chromosome:NCBI36:3:191483506:191491064:-1
2712236	247509<br>247510<br>247511<br>247512	2712237<br>2712238<br>2712239<br>2712240	core<br>core<br>core<br>core	3	196959311	197036867	-	NM_138297<br>NM_004532<br>NM_018406<br>AF058804	Homo sapiens mucin 4, cell surface associated (MUC4), transcript variant 5, mRNA.<br>Homo sapiens mucin 4, cell surface associated (MUC4), transcript variant 4, mRNA.<br>Homo sapiens mucin 4, cell surface associated (MUC4), transcript variant 1, mRNA.<br>Homo sapiens clone G4-10-3 mucin 4 (MUC4) mRNA, partial cds.

	247513	2712241	core					AF177925	Homo sapiens mucin 4 (MUC4) mRNA, partial cds.
	247514	2712242	core					AK074437	Homo sapiens cDNA FLJ23857 fis, clone LNG07164.
	247515	2712243	extended					AJ242541	Homo sapiens partial mRNA for sv1-MUC4 apomucin.
	247516	2712244	core					AJ000281	Homo sapiens mRNA for mucin protein, MUC4.
	247517	2712245	core					M64594	Human tracheo-bronchial mucin (MUC4) mRNA, partial cds.
	247518	2712246	core					ENSESTT00000006041	
	247519	2712247	extended					ENST00000346145	cdna:known-ccds chromosome:NCBI36:3:196959311:197023545:-1 gene:ENSG00000145113 CCDS3310.1
	247520	2712248	core					ENST00000349607	cdna:known-ccds chromosome:NCBI36:3:196959311:197023545:-1 gene:ENSG00000145113 CCDS3311.1
	247521	2712249	core					ENST00000339251	cdna:known chromosome:NCBI36:3:196959311:197023545:-1 gene:ENSG00000145113
	247522	2712250	extended					ENST00000308466	cdna:known chromosome:NCBI36:3:196959311:197002767:-1 gene:ENSG00000145113
	247523	2712251	core					ENST00000333177	cdna:known chromosome:NCBI36:3:196959311:197002767:-1 gene:ENSG00000145113
	247524	2712252	core					ENST00000381931	cdna:novel chromosome:NCBI36:3:196991089:196993062:-1 gene:ENSG00000205811
	247525	2712253	core					ENST00000381929	cdna:novel chromosome:NCBI36:3:196991833:196999518:-1 gene:ENSG00000205811
	247526	2712254	extended					ENST00000381928	cdna:novel chromosome:NCBI36:3:196992865:196993734:-1 gene:ENSG00000205811
	247527	2712255	core					ENST00000381926	cdna:novel chromosome:NCBI36:3:196993105:196993734:-1 gene:ENSG00000205811
	247528	2712256	extended					ENST00000381925	cdna:novel chromosome:NCBI36:3:196995841:196997718:-1 gene:ENSG00000205811
	247529	2712257	core					ENST00000381924	cdna:novel chromosome:NCBI36:3:196995841:196996707:-1 gene:ENSG00000205811
	247530	2712258	full					GENSCAN00000034839	cdna:Genscan chromosome:NCBI36:3:196981259:197036867:-1
	247531	2712259	full					GENSCAN00000034842	cdna:Genscan chromosome:NCBI36:3:196959718:196976984:-1
	247532	2712260	full						
	247533	2712261	core						
	247534	2712262	core						
	247535	2712263	full						
	247536	2712264	core						
	247537	2712265	core						
	247538	2712266	core						
	247539	2712267	core						
	247540	2712268	core						
	247541	2712269	core						
	247542	2712270	core						
	247543	2712271	core						
	247544	2712272	full						
	247545	2712273	core						
	247546	2712274	core						
	247547	2712275	full						
	247548	2712276	core						
	247549	2712277	core						
	247550	2712278	core						
	247551	2712279	core						
	247552	2712280	extended						
	247553	2712281	core						
	247554	2712282	core						
	247555	2712283	full						
	247556	2712284	core						
	247557	2712285	extended						
	247558	2712286	full						
	247559	2712287	full						
	247560	2712288	full						
	247561	2712289	core						
	247562	2712290	core						
	247563	2712291	extended						
	247564	2712292	full						
	247565	2712293	full						
	247566	2712294	extended						

	247567	2712295	extended						
	247568	2712296	extended						
	247569	2712297	extended						
	247570	2712298	full						
	247571	2712299	full						
	247572	2712300	full						
	247573	2712301	extended						
	247574	2712302	extended						
	247575	2712303	full						
	247576	2712304	full						
	247577	2712305	full						
	247578	2712306	full						
	247579	2712307	full						
	247580	2712308	full						
	247581	2712309	extended						
	247582	2712310	extended						
	247583	2712311	full						
	247584	2712312	full						
		2712313	full						
		2712314	full						
		2712315	full						
		2712316	extended						
		2712317	full						
		2712318	extended						
		2712319	extended						
		2712320	full						
		2712321	extended						
		2712322	extended						
		2712323	extended						
		2712324	full						
		2712325	full						
		2712326	full						
		2712327	extended						
		2712328	extended						
		2712329	extended						
		2712330	full						
		2712331	full						
		2712332	full						
		2712333	full						
		2712334	extended						
		2712335	extended						
		2712336	extended						
		2712337	extended						
		2712338	extended						
		2712339	extended						
		2712340	full						
		2712341	extended						
		2712342	extended						
		2712343	extended						
		2712344	extended						
		2712345	extended						
		2712346	extended						
		2712347	extended						
		2712348	extended						

		2712349	full						
		2712350	extended						
		2712351	extended						
		2712352	full						
		2712353	full						
		2712354	full						
		2712355	extended						
		2712356	extended						
		2712357	extended						
		2712358	extended						
		2712359	extended						
		2712360	full						
		2712361	extended						
		2712362	full						
		2712363	full						
		2712364	extended						
		2712365	full						
		2712366	full						
		2712367	extended						
		2712368	extended						
		2712369	core						
		2712370	core						
		2712371	core						
		2712372	full						
		2712373	full						
		2712374	full						
		2712375	full						
		2712376	full						
		2712377	extended						
		2712378	full						
		2712379	full						
		2712380	full						
		2712381	full						
		2712382	extended						
		2712383	core						
		2712384	core						
		2712385	core						
		2712386	core						
		2712387	extended						
		2712388	extended						
		2712389	extended						
		2712390	full						
		2712391	extended						
		2712392	full						
		2712393	full						
		2712394	full						
		2712395	full						
		2712396	full						
		2712397	full						
		2712398	full						
		2712399	full						
		2712400	full						
		2712401	full						
		2712402	full						

2727226	256762	2727227	extended	4	53938569	54922990	+	NM_030917	Homo sapiens FIP1 like 1 (S. cerevisiae) (FIP1L1), mRNA.
	256763	2727228	core					XM_936464	PREDICTED: Homo sapiens similar to hypertension-related calcium-regulated gene (LOC441016), mRNA.
	256764	2727229	core					XM_496693	PREDICTED: Homo sapiens similar to hypertension-related calcium-regulated gene (LOC441016), mRNA.
	256765	2727230	extended					NM_006206	Homo sapiens platelet-derived growth factor receptor, alpha polypeptide (PDGFRA), mRNA.
	256766	2727231	extended					AF090902	Homo sapiens clone HQ0202 PRO0202 mRNA, partial cds.
	256767	2727232	core					AK090938	Homo sapiens cDNA FLJ33619 fis, clone BRAMY2020427.
	256768	2727233	extended					AK091371	Homo sapiens cDNA FLJ34052 fis, clone FCBBF3000175.
	256769	2727234	core					AK123992	Homo sapiens cDNA FLJ41998 fis, clone SPLEN2029522.
	256770	2727235	core					AY229892	Homo sapiens FIP1L1/PDGFRA fusion protein (FIP1L1/PDGFRA fusion) mRNA, complete cds; alternatively spliced.
	256771	2727236	core					BC015186	Homo sapiens platelet-derived growth factor receptor, alpha polypeptide, mRNA (cDNA clone IMAGE:4043984),
	256772	2727237	extended					BC017724	complete cds.
	256773	2727238	core					BC024016	Homo sapiens FIP1 like 1 (S. cerevisiae), mRNA (cDNA clone MGC:21370 IMAGE:3452247), complete cds.
	256774	2727239	core					BC063414	Homo sapiens FIP1 like 1 (S. cerevisiae), mRNA (cDNA clone MGC:26986 IMAGE:4826276), complete cds.
	256775	2727240	core					X76079	Homo sapiens platelet-derived growth factor receptor, alpha polypeptide, mRNA (cDNA clone MGC:74795
	256776	2727241	full					X95095	IMAGE:5205969), complete cds.
	256777	2727242	core					CR595922	H.sapiens mRNA for platelet derived growth factor alpha receptor.
	256778	2727243	extended					ENSESTT00000016270	H.sapiens mRNA for PDGFRalpha protein.
	256779	2727244	core					ENSESTT00000016271	full-length cDNA clone CS0DD005YK11 of Neuroblastoma Cot 50-normalized of Homo sapiens (human).
	256780	2727245	core					ENSESTT00000016272	
	256781	2727246	core					ENSESTT00000016273	
	256782	2727247	extended					ENSESTT00000016274	
	256783	2727248	extended					ENSESTT00000016275	
	256784	2727249	core					ENSESTT00000016276	
	256785	2727250	full					ENSESTT00000016280	
	256786	2727251	extended					ENSESTT00000054339	
	256787	2727252	core					ENSESTT00000054340	
	256788	2727253	core					ENSESTT00000054341	
	256789	2727254	extended					ENSESTT00000054342	
	256790	2727255	extended					ENSESTT00000054343	
	256791	2727256	extended					ENSESTT00000054344	
	256792	2727257	extended					ENSESTT00000054345	
	256793	2727258	extended					ENSESTT00000054346	
	256794	2727259	extended					ENSESTT00000054347	
	256795	2727260	extended					ENSESTT00000054348	
	256796	2727261	free					ENSESTT00000054349	
	256797	2727262	free					ENST00000337488	
	256798	2727263	core					ENST00000257290	
	256799	2727264	free					ENST00000306932	cdna:known-ccds chromosome:NCBI36:4:53938620:54020599:1 gene:ENSG00000145216 CCDS3491.1
	256800	2727265	core					ENST00000358575	cdna:known-ccds chromosome:NCBI36:4:54790204:54859168:1 gene:ENSG00000134853 CCDS3495.1
	256801	2727266	extended					ENST00000381354	cdna:known chromosome:NCBI36:4:53938620:54020599:1 gene:ENSG00000145216
	256802	2727267	extended					ENST00000312008	cdna:known chromosome:NCBI36:4:53938620:54020545:1 gene:ENSG00000145216
	256803	2727268	core					GENSCAN00000034232	cdna:known chromosome:NCBI36:4:54790214:54859171:1 gene:ENSG00000134853
	256804	2727269	core					GENSCAN00000005148	cdna:novel chromosome:NCBI36:4:54136637:54137311:1 gene:ENSG00000173966
	256805	2727270	extended					GENSCAN00000023418	cdna:Genscan chromosome:NCBI36:4:54133508:54137311:1
	256806	2727271	extended					GENSCAN00000013867	cdna:Genscan chromosome:NCBI36:4:54822150:54856196:1
	256807	2727272	extended					GENSCAN00000038257	cdna:Genscan chromosome:NCBI36:4:53938763:53944800:1
	256808	2727273	full					GENSCAN00000010665	cdna:Genscan chromosome:NCBI36:4:54648016:54648288:1
	256809	2727274	core					GENSCAN00000010666	cdna:Genscan chromosome:NCBI36:4:54773914:54795393:1
	256810	2727275	core						cdna:Genscan chromosome:NCBI36:4:53950733:54020373:1
	256811	2727276	core						cdna:Genscan chromosome:NCBI36:4:54090496:54111479:1
	256812	2727277	core						
	256813	2727278	extended						
	256814	2727279	extended						
	256815	2727280	extended						

	256816	2727281	extended						
	256817	2727282	extended						
	256818	2727283	extended						
	256819	2727284	extended						
	256820	2727285	full						
	256821	2727286	full						
	256822	2727287	full						
	256823	2727288	full						
	256824	2727289	full						
	256825	2727290	extended						
	256826	2727291	extended						
	256827	2727292	extended						
	256828	2727293	full						
	256829	2727294	full						
	256830	2727295	full						
	256831	2727296	extended						
	256832	2727297	extended						
	256833	2727298	extended						
	256834	2727299	full						
	256835	2727300	full						
	256836	2727301	full						
	256837	2727302	full						
	256838	2727303	extended						
	256839	2727304	full						
	256840	2727305	full						
	256841	2727306	full						
	256842	2727307	full						
	256843	2727308	full						
	256844	2727309	full						
	256845	2727310	full						
	256846	2727311	full						
	256847	2727312	full						
	256848	2727313	extended						
	256849	2727314	extended						
	256850	2727315	extended						
	256851	2727316	extended						
	256852	2727317	extended						
	256853	2727318	extended						
	256854	2727319	extended						
	256855	2727320	extended						
	256856	2727321	extended						
	256857	2727322	extended						
	256858	2727323	extended						
	256859	2727324	extended						
	256860	2727325	full						
	256861	2727326	full						
	256862	2727327	full						
	256863	2727328	extended						
	256864	2727329	full						
	256865	2727330	full						
	256866	2727331	full						
	256867	2727332	extended						
	256868	2727333	full						
	256869	2727334	full						

	256870	2727335	full						
	256871	2727336	full						
	256872	2727337	full						
	256873	2727338	full						
	256874	2727339	full						
	256875	2727340	full						
	256876	2727341	full						
	256877	2727342	extended						
	256878	2727343	full						
	256879	2727344	full						
	256880	2727345	full						
	256881	2727346	full						
	256882	2727347	full						
	256883	2727348	extended						
	256884	2727349	core						
	256885	2727350	core						
	256886	2727351	full						
	256887	2727352	full						
	256888	2727353	extended						
	256889	2727354	extended						
	256890	2727355	full						
	256891	2727356	full						
	256892	2727357	full						
	256893	2727358	full						
	256894	2727359	full						
	256895	2727360	full						
	256896	2727361	full						
	256897	2727362	full						
	256898	2727363	full						
	256899	2727364	full						
	256900	2727365	full						
	256901	2727366	full						
		2727367	extended						
		2727368	extended						
		2727369	extended						
		2727370	core						
		2727371	full						
		2727372	core						
		2727373	core						
		2727374	core						
		2727375	core						
		2727376	core						
		2727377	core						
		2727378	core						
		2727379	full						
		2727380	core						
		2727381	core						
		2727382	core						
		2727383	core						
		2727384	extended						
		2727385	full						
		2727386	core						
		2727387	core						
		2727388	core						

		2727389<br>2727390<br>2727391<br>2727392<br>2727393<br>2727394<br>2727395<br>2727396<br>2727397<br>2727398<br>2727399<br>2727400<br>2727401<br>2727402<br>2727403<br>2727404<br>2727405<br>2727406<br>2727407<br>2727408<br>2727409<br>2727410<br>2727411<br>2727412<br>2727413<br>2727414<br>2727415<br>2727416<br>2727417<br>2727418<br>2727419<br>2727420	core<br>extended<br>core<br>extended<br>core<br>extended<br>core<br>core<br>core<br>core<br>extended<br>extended<br>core<br>extended<br>core<br>core<br>full<br>core<br>core<br>core<br>full<br>full<br>core<br>extended<br>core<br>core<br>core<br>full<br>full<br>full<br>full<br>full						
2728938	257838<br>257839<br>257840<br>257841<br>257842<br>257843<br>257844<br>257845<br>257846<br>257847<br>257848<br>257849<br>257850<br>257851<br>257852<br>257853<br>257854<br>257855<br>257856<br>257857<br>257858<br>257859	2728939<br>2728940<br>2728941<br>2728942<br>2728943<br>2728944<br>2728945<br>2728946<br>2728947<br>2728948<br>2728949<br>2728950<br>2728951<br>2728952<br>2728953<br>2728954<br>2728955<br>2728956<br>2728957<br>2728958<br>2728959<br>2728960	core<br>extended<br>extended<br>extended<br>extended<br>full<br>full<br>extended<br>extended<br>extended<br>extended<br>core<br>full<br>extended<br>extended<br>extended<br>full<br>full<br>core<br>core<br>full<br>full	4	61749976	62622166	+	NM_015236<br>XM_937110<br>ENSESTT00000013498<br>ENSESTT00000022382<br>ENSESTT00000022383<br>ENSESTT00000022384<br>ENSESTT00000022385<br>ENSESTT00000022386<br>ENSESTT00000022387<br>ENSESTT00000022388<br>ENST00000280009<br>ENST00000295349<br>ENST00000355061<br>GENSCAN00000045466<br>GENSCAN00000048856<br>GENSCAN00000001109<br>GENSCAN00000040521<br>GENSCAN00000023734<br>GENSCAN00000012498	Homo sapiens latrophilin 3 (LPHN3), mRNA.<br>PREDICTED: Homo sapiens similar to Latrophilin-3 precursor (Calcium-independent alpha-latrotoxin receptor 3) (Lectomedin-3) (LOC653950), mRNA.<br>cdna:known chromosome:NCBI36:4:62045434:62620762:1 gene:ENSG00000150471<br>cdna:known chromosome:NCBI36:4:62045434:62620762:1 gene:ENSG00000150471<br>cdna:known chromosome:NCBI36:4:62045434:62620110:1 gene:ENSG00000150471<br>cdna:Genscan chromosome:NCBI36:4:62457873:62544670:1<br>cdna:Genscan chromosome:NCBI36:4:62119236:62232946:1<br>cdna:Genscan chromosome:NCBI36:4:62434820:62445414:1<br>cdna:Genscan chromosome:NCBI36:4:62281067:62289968:1<br>cdna:Genscan chromosome:NCBI36:4:62567041:62619221:1<br>cdna:Genscan chromosome:NCBI36:4:62060305:62068317:1

	257860	2728961	full							
	257861	2728962	extended							
	257862	2728963	full							
	257863	2728964	full							
	257864	2728965	full							
	257865	2728966	full							
	257866	2728967	extended							
	257867	2728968	extended							
	257868	2728969	extended							
	257869	2728970	full							
	257870	2728971	full							
	257871	2728972	extended							
	257872	2728973	full							
	257873	2728974	core							
	257874	2728975	full							
	257875	2728976	extended							
	257876	2728977	extended							
	257877	2728978	full							
	257878	2728979	full							
	257879	2728980	full							
	257880	2728981	extended							
	257881	2728982	full							
	257882	2728983	full							
	257883	2728984	full							
	257884	2728985	full							
	257885	2728986	full							
	257886	2728987	extended							
	257887	2728988	core							
	257888	2728989	extended							
	257889	2728990	full							
	257890	2728991	extended							
	257891	2728992	extended							
	257892	2728993	full							
	257893	2728994	full							
	257894	2728995	core							
	257895	2728996	core							
	257896	2728997	extended							
	257897	2728998	full							
	257898	2728999	full							
	257899	2729000	full							
	257900	2729001	full							
	257901	2729002	extended							
	257902	2729003	extended							
	257903	2729004	full							
	257904	2729005	extended							
	257905	2729006	full							
	257906	2729007	full							
	257907	2729008	full							
	257908	2729009	core							
	257909	2729010	extended							
	257910	2729011	extended							
	257911	2729012	extended							
	257912	2729013	extended							
	257913	2729014	extended							

	257914<br>257915<br>257916<br>257917<br>257918<br>257919<br>257920<br>257921<br>257922<br>257923<br>257924<br>257925<br>257926<br>257927<br>257928<br>257929<br>257930<br>257931<br>257932<br>257933<br>257934<br>257935<br>257936<br>257937<br>257938<br>257939<br>257940<br>257941<br>257942<br>257943<br>257944<br>257945<br>257946<br>257947<br>257948<br>257949	2729015<br>2729016<br>2729017<br>2729018<br>2729019<br>2729020<br>2729021<br>2729022<br>2729023<br>2729024<br>2729025<br>2729026<br>2729027<br>2729028<br>2729029<br>2729030<br>2729031<br>2729032<br>2729033<br>2729034<br>2729035<br>2729036<br>2729037<br>2729038<br>2729039<br>2729040<br>2729041<br>2729042<br>2729043<br>2729044<br>2729045<br>2729046<br>2729047<br>2729048<br>2729049<br>2729050<br>2729051<br>2729052<br>2729053<br>2729054<br>2729055<br>2729056<br>2729057<br>2729058<br>2729059<br>2729060<br>2729061<br>2729062<br>2729063<br>2729064<br>2729065<br>2729066	full<br>core<br>core<br>extended<br>extended<br>full<br>full<br>core<br>extended<br>full<br>extended<br>core<br>core<br>core<br>core<br>extended<br>core<br>extended<br>extended<br>extended<br>core<br>core<br>full<br>full<br>full<br>core<br>core<br>extended<br>full<br>core<br>core<br>full<br>full<br>full<br>core<br>core<br>core<br>core<br>extended<br>extended<br>extended<br>full<br>extended<br>core<br>extended<br>extended<br>extended<br>core<br>core<br>core<br>core<br>free						
2730746	258940<br>258941	2730747<br>2730748	full<br>full	4	72240703	72797108	+	NM_003759<br>AF004813	Homo sapiens solute carrier family 4, sodium bicarbonate cotransporter, member 4 (SLC4A4), mRNA.<br>Homo sapiens electrogenic Na+ bicarbonate cotransporter (NBC) mRNA, partial cds.

	258942	2730749	full					AF011390	Homo sapiens pancreas sodium bicarbonate cotransporter mRNA, complete cds.
	258943	2730750	full					BC030977	Homo sapiens solute carrier family 4, sodium bicarbonate cotransporter, member 4, mRNA (cDNA clone MGC:32627
	258944	2730751	full					ENSESTT00000014873	IMAGE:4610968), complete cds.
	258945	2730752	core					ENSESTT00000014874	
	258946	2730753	extended					ENSESTT00000014875	
	258947	2730754	extended					ENSESTT00000014876	
	258948	2730755	extended					ENSESTT00000014877	
	258949	2730756	extended					ENSESTT00000014878	
	258950	2730757	full					ENSESTT00000014879	
	258951	2730758	full					ENST00000340595	
	258952	2730759	full					ENST00000264485	cdna:known-ccds chromosome:NCBI36:4:72423681:72656663:1 gene:ENSG00000080493 CCDS3549.1
	258953	2730760	core					ENST00000351898	cdna:known chromosome:NCBI36:4:72271867:72654298:1 gene:ENSG00000080493
	258954	2730761	full					GENSCAN00000042650	cdna:known chromosome:NCBI36:4:72271867:72654298:1 gene:ENSG00000080493
	258955	2730762	full					GENSCAN00000046032	cdna:Genscan chromosome:NCBI36:4:72271857:72344483:1
	258956	2730763	full					GENSCAN00000043402	cdna:Genscan chromosome:NCBI36:4:72423830:72424086:1
	258957	2730764	full					GENSCAN00000050788	cdna:Genscan chromosome:NCBI36:4:72614104:72692244:1
	258958	2730765	full						cdna:Genscan chromosome:NCBI36:4:72434493:72566441:1
	258959	2730766	full						
	258960	2730767	full						
	258961	2730768	core						
	258962	2730769	core						
	258963	2730770	full						
	258964	2730771	full						
	258965	2730772	full						
	258966	2730773	full						
	258967	2730774	extended						
	258968	2730775	extended						
	258969	2730776	extended						
	258970	2730777	extended						
	258971	2730778	full						
	258972	2730779	extended						
	258973	2730780	core						
	258974	2730781	core						
	258975	2730782	core						
	258976	2730783	core						
	258977	2730784	core						
	258978	2730785	full						
	258979	2730786	full						
	258980	2730787	full						
	258981	2730788	full						
	258982	2730789	full						
	258983	2730790	core						
	258984	2730791	full						
	258985	2730792	core						
	258986	2730793	core						
	258987	2730794	core						
	258988	2730795	core						
	258989	2730796	core						
	258990	2730797	full						
	258991	2730798	full						
	258992	2730799	core						
	258993	2730800	core						
	258994	2730801	core						
	258995	2730802	extended						

	258996<br>258997<br>258998<br>258999<br>259000<br>259001<br>259002<br>259003<br>259004	2730803<br>2730804<br>2730805<br>2730806<br>2730807<br>2730808<br>2730809<br>2730810<br>2730811<br>2730812<br>2730813<br>2730814<br>2730815<br>2730816<br>2730817<br>2730818<br>2730819<br>2730820<br>2730821<br>2730822<br>2730823<br>2730824<br>2730825<br>2730826	core<br>core<br>core<br>core<br>core<br>core<br>full<br>core<br>core<br>full<br>core<br>core<br>extended<br>extended<br>core<br>core<br>core<br>core<br>core<br>full<br>full<br>full<br>full<br>full						
2734047	260998<br>260999<br>261000<br>261001<br>261002<br>261003<br>261004<br>261005<br>261006<br>261007<br>261008<br>261009<br>261010<br>261011<br>261012<br>261013<br>261014<br>261015<br>261016<br>261017<br>261018<br>261019<br>261020<br>261021<br>261022<br>261023<br>261024<br>261025<br>261026	2734048<br>2734049<br>2734050<br>2734051<br>2734052<br>2734053<br>2734054<br>2734055<br>2734056<br>2734057<br>2734058<br>2734059<br>2734060<br>2734061<br>2734062<br>2734063<br>2734064<br>2734065<br>2734066<br>2734067<br>2734068<br>2734069<br>2734070<br>2734071<br>2734072<br>2734073<br>2734074<br>2734075<br>2734076<br>2734077	full<br>full<br>extended<br>extended<br>core<br>core<br>full<br>full<br>extended<br>extended<br>extended<br>full<br>core<br>extended<br>full<br>full<br>full<br>extended<br>full<br>core<br>extended<br>core<br>core<br>full<br>full<br>core<br>core<br>extended<br>extended<br>core	4	84675910	84746050	+	NM_032717<br>AK055749<br>ENSESTT00000000138<br>ENSESTT00000000139<br>ENSESTT00000000140<br>ENSESTT00000000141<br>ENST00000264409<br>GENSCAN00000067582<br>GENSCAN00000021363	Homo sapiens lysophosphatidic acid acyltransferase theta (LPAAT-THETA), mRNA.<br>Homo sapiens cDNA FLJ31187 fis, clone KIDNE2000349, moderately similar to Mus musculus putative lysophosphatidic acid acyltransferase mRNA.<br><br><br><br><br>cdna:known-ccds chromosome:NCBI36:4:84676588:84746049:1 gene:ENSG00000138678 CCDS3606.1<br>cdna:Genscan chromosome:NCBI36:4:84675910:84693775:1<br>cdna:Genscan chromosome:NCBI36:4:84702894:84744944:1

		2734078<br>2734079<br>2734080<br>2734081<br>2734082	core<br>core<br>core<br>core<br>core						
2735027	261647<br>261648<br>261649<br>261650<br>261651<br>261652<br>261653<br>261654<br>261655<br>261656<br>261657<br>261658<br>261659<br>261660<br>261661<br>261662<br>261663<br>261664<br>261665<br>261666<br>261667<br>261668<br>261669<br>261670<br>261671<br>261672<br>261673<br>261674<br>261675<br>261676<br>261677<br>261678<br>261679<br>261680<br>261681<br>261682<br>261683<br>261684<br>261685<br>261686	2735028<br>2735029<br>2735030<br>2735031<br>2735032<br>2735033<br>2735034<br>2735035<br>2735036<br>2735037<br>2735038<br>2735039<br>2735040<br>2735041<br>2735042<br>2735043<br>2735044<br>2735045<br>2735046<br>2735047<br>2735048<br>2735049<br>2735050<br>2735051<br>2735052<br>2735053<br>2735054<br>2735055<br>2735056<br>2735057<br>2735058<br>2735059<br>2735060<br>2735061<br>2735062<br>2735063<br>2735064<br>2735065<br>2735066<br>2735067<br>2735068<br>2735069<br>2735070<br>2735071<br>2735072	extended<br>extended<br>extended<br>full<br>extended<br>extended<br>full<br>full<br>full<br>full<br>full<br>extended<br>extended<br>full<br>full<br>full<br>full<br>full<br>full<br>full<br>extended<br>extended<br>extended<br>full<br>full<br>full<br>extended<br>core<br>extended<br>core<br>core<br>extended<br>extended<br>extended<br>core<br>extended<br>core<br>extended<br>core<br>core<br>core<br>extended<br>core<br>core<br>core	4	88627267	89123585	+	NM_001040058<br>NM_001040060<br>NM_000582<br>AY956318<br>BC007016<br>BX648003<br>ENSESTT00000042535<br>ENSESTT00000042536<br>ENST00000237623<br>ENST00000360804<br>ENST00000359072<br>GENSCAN00000030225	Homo sapiens secreted phosphoprotein 1 (osteopontin, bone sialoprotein I, early T-lymphocyte activation 1) (SPP1), transcript variant 1, mRNA.<br>Homo sapiens secreted phosphoprotein 1 (osteopontin, bone sialoprotein I, early T-lymphocyte activation 1) (SPP1), transcript variant 3, mRNA.<br>Homo sapiens secreted phosphoprotein 1 (osteopontin, bone sialoprotein I, early T-lymphocyte activation 1) (SPP1), transcript variant 2, mRNA.<br>Homo sapiens osteopontin/immunoglobulin alpha 1 heavy chain constant region fusion protein (SPP1/CALPHA1 fusion) mRNA, partial cds.<br>Homo sapiens secreted phosphoprotein 1 (osteopontin, bone sialoprotein I, early T-lymphocyte activation 1), mRNA (cDNA clone MGC:12351 IMAGE:4052438), complete cds.<br>Homo sapiens mRNA; cDNA DKFZp686G0159 (from clone DKFZp686G0159).<br><br><br>cdna:known-ccds chromosome:NCBI36:4:89115890:89123502:1 gene:ENSG00000118785 CCDS3626.1<br>cdna:known chromosome:NCBI36:4:89115890:89123502:1 gene:ENSG00000118785<br>cdna:known chromosome:NCBI36:4:89115905:89123592:1 gene:ENSG00000118785<br>cdna:Genscan chromosome:NCBI36:4:89115845:89123072:1
2740067	264929<br>264930<br>264931	2740068<br>2740069<br>2740070	extended<br>extended<br>extended	4	113846291	114577680	+	NM_020977<br>NM_001148<br>AF131823	Homo sapiens ankyrin 2, neuronal (ANK2), transcript variant 2, mRNA.<br>Homo sapiens ankyrin 2, neuronal (ANK2), transcript variant 1, mRNA.<br>Homo sapiens clone 24997 mRNA sequence.

	264932	2740071	full					AK021894	Homo sapiens cDNA FLJ11832 fis, clone HEMBA1006566.
	264933	2740072	full					AK095596	Homo sapiens cDNA FLJ38277 fis, clone FCBBF3004955, highly similar to ANKYRIN 2.
	264934	2740073	full					BC030740	Homo sapiens cDNA clone IMAGE:4791521.
	264935	2740074	extended					BX537758	Homo sapiens mRNA; cDNA DKFZp686H0688 (from clone DKFZp686H0688); complete cds.
	264936	2740075	extended					BX538132	Homo sapiens mRNA; cDNA DKFZp686M09125 (from clone DKFZp686M09125).
	264937	2740076	full					ENSESTT00000010240	
	264938	2740077	full					ENSESTT00000010242	
	264939	2740078	full					ENSESTT00000010244	
	264940	2740079	full					ENSESTT00000010245	
	264941	2740080	extended					ENSESTT00000010246	
	264942	2740081	full					ENSESTT00000010249	
	264943	2740082	full					ENSESTT00000010250	
	264944	2740083	full					ENSESTT00000010253	
	264945	2740084	full					ENSESTT00000010254	
	264946	2740085	extended					ENSESTT00000010255	
	264947	2740086	extended					ENSESTT00000010258	
	264948	2740087	extended					ENSESTT00000010259	
	264949	2740088	full					ENST00000357077	cdna:known-ccds chromosome:NCBI36:4:114190319:114524334:1 gene:ENSG00000145362 CCDS3702.1
	264950	2740089	extended					ENST00000361149	cdna:known chromosome:NCBI36:4:114190319:114524334:1 gene:ENSG00000145362
	264951	2740090	full					ENST00000264366	cdna:known chromosome:NCBI36:4:114190319:114524334:1 gene:ENSG00000145362
	264952	2740091	full					ENST00000343056	cdna:known chromosome:NCBI36:4:114459190:114524333:1 gene:ENSG00000145362
	264953	2740092	full					GENSCAN00000066751	cdna:Genscan chromosome:NCBI36:4:114372787:114443444:1
	264954	2740093	core					GENSCAN00000066753	cdna:Genscan chromosome:NCBI36:4:114333556:114340486:1
	264955	2740094	full					GENSCAN00000066752	cdna:Genscan chromosome:NCBI36:4:114315021:114328201:1
	264956	2740095	full					GENSCAN00000038387	cdna:Genscan chromosome:NCBI36:4:114286474:114291756:1
	264957	2740096	full					GENSCAN00000001761	cdna:Genscan chromosome:NCBI36:4:114190334:114214445:1
	264958	2740097	full					GENSCAN00000001760	cdna:Genscan chromosome:NCBI36:4:114257201:114260384:1
	264959	2740098	extended					GENSCAN00000013660	cdna:Genscan chromosome:NCBI36:4:114451860:114577680:1
	264960	2740099	extended					GENSCAN00000020745	cdna:Genscan chromosome:NCBI36:4:113846751:113847162:1
	264961	2740100	full					GENSCAN00000058961	cdna:Genscan chromosome:NCBI36:4:113877427:113899559:1
	264962	2740101	extended					GENSCAN00000058963	cdna:Genscan chromosome:NCBI36:4:113958730:113968287:1
	264963	2740102	extended						
	264964	2740103	extended						
	264965	2740104	extended						
	264966	2740105	extended						
	264967	2740106	extended						
	264968	2740107	extended						
	264969	2740108	full						
	264970	2740109	full						
	264971	2740110	full						
	264972	2740111	full						
	264973	2740112	extended						
	264974	2740113	extended						
	264975	2740114	extended						
	264976	2740115	extended						
	264977	2740116	extended						
	264978	2740117	core						
	264979	2740118	full						
	264980	2740119	full						
	264981	2740120	extended						
	264982	2740121	full						
	264983	2740122	full						
	264984	2740123	full						
	264985	2740124	extended						

	264986	2740125	full						
	264987	2740126	extended						
	264988	2740127	extended						
	264989	2740128	extended						
	264990	2740129	extended						
	264991	2740130	extended						
	264992	2740131	full						
	264993	2740132	full						
	264994	2740133	extended						
	264995	2740134	extended						
	264996	2740135	extended						
	264997	2740136	extended						
	264998	2740137	extended						
	264999	2740138	full						
	265000	2740139	full						
	265001	2740140	full						
	265002	2740141	full						
	265003	2740142	extended						
	265004	2740143	full						
	265005	2740144	full						
	265006	2740145	extended						
	265007	2740146	extended						
	265008	2740147	extended						
	265009	2740148	extended						
	265010	2740149	extended						
	265011	2740150	full						
	265012	2740151	extended						
	265013	2740152	core						
	265014	2740153	full						
	265015	2740154	full						
	265016	2740155	core						
	265017	2740156	core						
	265018	2740157	full						
	265019	2740158	extended						
	265020	2740159	extended						
	265021	2740160	full						
	265022	2740161	extended						
	265023	2740162	extended						
	265024	2740163	extended						
	265025	2740164	extended						
	265026	2740165	extended						
	265027	2740166	extended						
	265028	2740167	extended						
	265029	2740168	core						
	265030	2740169	full						
	265031	2740170	core						
	265032	2740171	full						
	265033	2740172	core						
	265034	2740173	full						
	265035	2740174	core						
	265036	2740175	core						
	265037	2740176	extended						
	265038	2740177	extended						
	265039	2740178	core						

	265040	2740179	extended						
	265041	2740180	full						
	265042	2740181	core						
	265043	2740182	extended						
	265044	2740183	core						
	265045	2740184	core						
	265046	2740185	full						
	265047	2740186	extended						
	265048	2740187	extended						
	265049	2740188	core						
	265050	2740189	extended						
	265051	2740190	extended						
	265052	2740191	core						
	265053	2740192	core						
	265054	2740193	extended						
	265055	2740194	core						
	265056	2740195	core						
	265057	2740196	full						
	265058	2740197	core						
	265059	2740198	core						
	265060	2740199	core						
	265061	2740200	extended						
	265062	2740201	extended						
	265063	2740202	core						
	265064	2740203	extended						
	265065	2740204	core						
	265066	2740205	full						
	265067	2740206	full						
	265068	2740207	extended						
	265069	2740208	extended						
	265070	2740209	core						
	265071	2740210	full						
	265072	2740211	full						
	265073	2740212	extended						
	265074	2740213	core						
	265075	2740214	full						
	265076	2740215	full						
	265077	2740216	extended						
	265078	2740217	core						
	265079	2740218	core						
	265080	2740219	core						
	265081	2740220	core						
	265082	2740221	full						
	265083	2740222	core						
	265084	2740223	core						
	265085	2740224	core						
	265086	2740225	core						
	265087	2740226	extended						
	265088	2740227	core						
	265089	2740228	core						
	265090	2740229	core						
	265091	2740230	core						
	265092	2740231	full						
	265093	2740232	core						

	265094	2740233	extended						
	265095	2740234	extended						
	265096	2740235	extended						
	265097	2740236	core						
	265098	2740237	extended						
	265099	2740238	extended						
	265100	2740239	core						
	265101	2740240	core						
	265102	2740241	core						
	265103	2740242	core						
	265104	2740243	core						
	265105	2740244	core						
	265106	2740245	core						
	265107	2740246	core						
	265108	2740247	core						
		2740248	core						
		2740249	core						
		2740250	core						
		2740251	core						
		2740252	core						
		2740253	core						
		2740254	core						
		2740255	core						
		2740256	full						
		2740257	full						
		2740258	core						
		2740259	core						
		2740260	extended						
		2740261	core						
		2740262	core						
		2740263	core						
		2740264	extended						
		2740265	extended						
		2740266	core						
		2740267	core						
		2740268	extended						
		2740269	extended						
		2740270	extended						
		2740271	extended						
		2740272	extended						
		2740273	extended						
		2740274	extended						
		2740275	extended						
		2740276	extended						
		2740277	extended						
		2740278	extended						
		2740279	full						
		2740280	core						
		2740281	core						
		2740282	core						
		2740283	core						
		2740284	core						
		2740285	core						
		2740286	full						

		2740287<br>2740288<br>2740289<br>2740290<br>2740291<br>2740292<br>2740293<br>2740294<br>2740295<br>2740296<br>2740297<br>2740298<br>2740299<br>2740300<br>2740301<br>2740302<br>2740303<br>2740304	extended<br>full<br>full<br>full<br>full<br>full<br>full<br>full<br>full<br>full<br>full<br>full<br>full<br>extended<br>full<br>full<br>full<br>full						
2746591	269003<br>269004<br>269005<br>269006<br>269007<br>269008<br>269009<br>269010<br>269011<br>269012<br>269013<br>269014<br>269015<br>269016<br>269017<br>269018<br>269019<br>269020<br>269021<br>269022<br>269023<br>269024<br>269025<br>269026<br>269027<br>269028<br>269029<br>269030	2746592<br>2746593<br>2746594<br>2746595<br>2746596<br>2746597<br>2746598<br>2746599<br>2746600<br>2746601<br>2746602<br>2746603<br>2746604<br>2746605<br>2746606<br>2746607<br>2746608<br>2746609<br>2746610<br>2746611<br>2746612<br>2746613<br>2746614<br>2746615<br>2746616<br>2746617<br>2746618<br>2746619<br>2746620<br>2746621<br>2746622<br>2746623<br>2746624<br>2746625<br>2746626<br>2746627	full<br>full<br>full<br>full<br>extended<br>extended<br>core<br>full<br>full<br>core<br>core<br>core<br>full<br>extended<br>extended<br>extended<br>extended<br>extended<br>extended<br>extended<br>extended<br>extended<br>extended<br>extended<br>extended<br>full<br>core<br>extended<br>extended<br>extended<br>full<br>core<br>core<br>core<br>extended<br>core	4	148599594	148685542	+	NM_001957<br>AK123169<br>BX537573<br>S67127<br>ENSESTT00000044921<br>ENSESTT00000044922<br>ENSESTT00000044923<br>ENSESTT00000044924<br>ENST00000324300<br>ENST00000358556<br>ENST00000339690<br>GENSCAN00000005966	Homo sapiens endothelin receptor type A (EDNRA), mRNA.<br>Homo sapiens cDNA FLJ41174 fis, clone BRACE2042398.<br>Homo sapiens mRNA; cDNA DKFZp686O049 (from clone DKFZp686O049).<br>endothelin ETA receptor [human, placenta, mRNA, 1661 nt].<br><br><br><br><br>cdna:known-ccds chromosome:NCBI36:4:148621575:148685555:1 gene:ENSG00000151617 CCDS3769.1<br>cdna:known chromosome:NCBI36:4:148621575:148685555:1 gene:ENSG00000151617<br>cdna:known chromosome:NCBI36:4:148621575:148685555:1 gene:ENSG00000151617<br>cdna:Genscan chromosome:NCBI36:4:148599594:148683204:1

		2746628<br>2746629<br>2746630<br>2746631<br>2746632<br>2746633	core<br>extended<br>full<br>full<br>core<br>core						
2779199	289308<br>289309<br>289310<br>289311<br>289312<br>289313<br>289314<br>289315<br>289316<br>289317<br>289318<br>289319<br>289320<br>289321<br>289322<br>289323	2779200<br>2779201<br>2779202<br>2779203<br>2779204<br>2779205<br>2779206<br>2779207<br>2779208<br>2779209<br>2779210<br>2779211<br>2779212<br>2779213<br>2779214<br>2779215<br>2779216<br>2779217<br>2779218<br>2779219<br>2779220<br>2779221<br>2779222<br>2779223<br>2779224<br>2779225	core<br>core<br>extended<br>extended<br>core<br>extended<br>full<br>core<br>extended<br>full<br>extended<br>core<br>extended<br>core<br>core<br>core<br>full<br>core<br>extended<br>core<br>core<br>core<br>full<br>core<br>core<br>extended	4	100416547	100431188	-	NM_000667<br>ENSESTT00000025743<br>ENST00000209668<br>GENSCAN00000002246	Homo sapiens alcohol dehydrogenase 1A (class I), alpha polypeptide (ADH1A), mRNA.<br><br>cdna:known-ccds chromosome:NCBI36:4:100416547:100431165:-1 gene:ENSG00000187758 CCDS3648.1<br>cdna:Genscan chromosome:NCBI36:4:100419606:100483175:-1
2786322	293934<br>293935<br>293936<br>293937<br>293938<br>293939<br>293940<br>293941<br>293942<br>293943<br>293944<br>293945<br>293946<br>293947<br>293948<br>293949<br>293950<br>293951<br>293952<br>293953<br>293954	2786323<br>2786324<br>2786325<br>2786326<br>2786327<br>2786328<br>2786329<br>2786330<br>2786331<br>2786332<br>2786333<br>2786334<br>2786335<br>2786336<br>2786337<br>2786338<br>2786339<br>2786340<br>2786341<br>2786342<br>2786343	full<br>full<br>core<br>core<br>extended<br>extended<br>extended<br>core<br>core<br>extended<br>extended<br>full<br>core<br>full<br>core<br>full<br>core<br>core<br>core<br>full<br>full	4	139279856	139461590	-	NM_014331<br>ENSESTT00000005135<br>ENSESTT00000005136<br>ENSESTT00000005137<br>ENSESTT00000005138<br>ENST00000280612<br>GENSCAN00000041927	Homo sapiens solute carrier family 7, (cationic amino acid transporter, y+ system) member 11 (SLC7A11), mRNA.<br><br><br><br><br>cdna:known-ccds chromosome:NCBI36:4:139304698:139382953:-1 gene:ENSG00000151012 CCDS3742.1<br>cdna:Genscan chromosome:NCBI36:4:139428794:139468806:-1

	293955<br>293956<br>293957<br>293958<br>293959<br>293960<br>293961<br>293962<br>293963<br>293964<br>293965<br>293966<br>293967<br>293968<br>293969<br>293970<br>293971<br>293972<br>293973	2786344<br>2786345<br>2786346<br>2786347<br>2786348<br>2786349<br>2786350<br>2786351<br>2786352<br>2786353<br>2786354<br>2786355<br>2786356<br>2786357<br>2786358<br>2786359<br>2786360<br>2786361<br>2786362<br>2786363<br>2786364<br>2786365<br>2786366<br>2786367<br>2786368<br>2786369<br>2786370	extended<br>free<br>extended<br>extended<br>core<br>full<br>full<br>core<br>core<br>full<br>core<br>full<br>core<br>extended<br>extended<br>full<br>core<br>core<br>extended<br>full<br>extended<br>full<br>full<br>full<br>extended<br>extended<br>full						
2842624	329299<br>329300<br>329301<br>329302<br>329303<br>329304<br>329305<br>329306<br>329307<br>329308<br>329309<br>329310<br>329311<br>329312<br>329313<br>329314<br>329315<br>329316<br>329317<br>329318<br>329319<br>329320<br>329321<br>329322<br>329323<br>329324<br>329325	2842625<br>2842626<br>2842627<br>2842628<br>2842629<br>2842630<br>2842631<br>2842632<br>2842633<br>2842634<br>2842635<br>2842636<br>2842637<br>2842638<br>2842639<br>2842640<br>2842641<br>2842642<br>2842643<br>2842644<br>2842645<br>2842646<br>2842647<br>2842648<br>2842649<br>2842650<br>2842651	full<br>full<br>full<br>extended<br>extended<br>extended<br>full<br>full<br>extended<br>full<br>full<br>full<br>core<br>core<br>core<br>core<br>core<br>core<br>extended<br>core<br>core<br>core<br>core<br>core<br>core<br>full<br>full	5	175902503	175956759	+	NM_017675<br>AK128446<br>ENSESTT00000006503<br>ENSESTT00000006504<br>ENSESTT00000006505<br>ENSESTT00000006506<br>ENSESTT00000006507<br>ENST00000389947<br>ENST00000389946<br>ENST00000261944<br>GENSCAN00000044009	Homo sapiens protocadherin LKC (PCLKC), mRNA.<br>Homo sapiens cDNA FLJ46589 fis, clone THYMU3044188.<br><br><br><br><br><br>cdna:known chromosome:NCBI36:5:175924960:175955279:1 gene:ENSG00000074276<br>cdna:known chromosome:NCBI36:5:175924960:175955279:1 gene:ENSG00000074276<br>cdna:novel chromosome:NCBI36:5:175908971:175955279:1 gene:ENSG00000074276<br>cdna:Genscan chromosome:NCBI36:5:175902503:175950934:1

	329326<br>329327<br>329328<br>329329<br>329330<br>329331<br>329332<br>329333<br>329334<br>329335<br>329336<br>329337<br>329338<br>329339<br>329340	2842652<br>2842653<br>2842654<br>2842655<br>2842656<br>2842657<br>2842658<br>2842659<br>2842660<br>2842661<br>2842662<br>2842663<br>2842664<br>2842665<br>2842666<br>2842667<br>2842668<br>2842669<br>2842670<br>2842671<br>2842672<br>2842673<br>2842674<br>2842675<br>2842676<br>2842677<br>2842678<br>2842679<br>2842680<br>2842681<br>2842682	core<br>core<br>core<br>core<br>core<br>full<br>core<br>core<br>core<br>core<br>core<br>extended<br>core<br>core<br>core<br>full<br>core<br>core<br>core<br>core<br>full<br>core<br>core<br>core<br>core<br>extended<br>core<br>core<br>extended<br>extended<br>full						
2886679	356791<br>356792<br>356793<br>356794	2886680<br>2886681<br>2886682<br>2886683<br>2886684<br>2886685<br>2886686<br>2886687	core<br>core<br>core<br>core<br>core<br>core<br>core<br>core	5	169737753	169749216	-	NM_004137<br>BC025707<br>ENSESTT00000021376<br>ENST00000274629<br>GENSCAN00000006994	Homo sapiens potassium large conductance calcium-activated channel, subfamily M, beta member 1 (KCNMB1), mRNA.<br>Homo sapiens potassium large conductance calcium-activated channel, subfamily M, beta member 1, mRNA (cDNA clone MGC:34483 IMAGE:5224514), complete cds.<br><br>cdna:known-ccds chromosome:NCBI36:5:169737745:169749216:-1 gene:ENSG00000145936 CCDS4373.1<br>cdna:Genscan chromosome:NCBI36:5:169738286:169745011:-1
2889916	358697<br>358698<br>358699<br>358700<br>358701<br>358702<br>358703<br>358704<br>358705<br>358706<br>358707<br>358708<br>358709<br>358710	2889917<br>2889918<br>2889919<br>2889920<br>2889921<br>2889922<br>2889923<br>2889924<br>2889925<br>2889926<br>2889927<br>2889928<br>2889929<br>2889930	core<br>extended<br>extended<br>extended<br>extended<br>extended<br>extended<br>extended<br>core<br>extended<br>full<br>core<br>extended<br>extended	5	178473474	178717474	-	NM_021599<br>NM_014244<br>ENSESTT00000017818<br>ENSESTT00000017819<br>ENSESTT00000017820<br>ENST00000251582<br>ENST00000274609<br>GENSCAN00000030552<br>GENSCAN00000030553<br>GENSCAN00000016400<br>GENSCAN00000016398<br>GENSCAN00000011848	Homo sapiens ADAM metallopeptidase with thrombospondin type 1 motif, 2 (ADAMTS2), transcript variant 2, mRNA.<br>Homo sapiens ADAM metallopeptidase with thrombospondin type 1 motif, 2 (ADAMTS2), transcript variant 1, mRNA.<br><br><br><br>cdna:known-ccds chromosome:NCBI36:5:178473474:178704935:-1 gene:ENSG00000087116 CCDS4444.1<br>cdna:known chromosome:NCBI36:5:178510736:178704935:-1 gene:ENSG00000087116<br>cdna:Genscan chromosome:NCBI36:5:178702420:178717474:-1<br>cdna:Genscan chromosome:NCBI36:5:178631866:178639504:-1<br>cdna:Genscan chromosome:NCBI36:5:178579985:178580810:-1<br>cdna:Genscan chromosome:NCBI36:5:178564270:178577858:-1<br>cdna:Genscan chromosome:NCBI36:5:178473474:178541885:-1

	358711<br>358712<br>358713<br>358714<br>358715<br>358716<br>358717<br>358718<br>358719<br>358720<br>358721<br>358722<br>358723<br>358724<br>358725<br>358726<br>358727<br>358728<br>358729<br>358730<br>358731<br>358732<br>358733<br>358734<br>358735<br>358736<br>358737<br>358738<br>358739<br>358740<br>358741<br>358742<br>358743<br>358744<br>358745<br>358746<br>358747<br>358748<br>358749<br>358750<br>358751<br>358752<br>358753<br>358754<br>358755<br>358756<br>358757<br>358758<br>358759<br>358760<br>358761<br>358762<br>358763<br>358764	2889931<br>2889932<br>2889933<br>2889934<br>2889935<br>2889936<br>2889937<br>2889938<br>2889939<br>2889940<br>2889941<br>2889942<br>2889943<br>2889944<br>2889945<br>2889946<br>2889947<br>2889948<br>2889949<br>2889950<br>2889951<br>2889952<br>2889953<br>2889954<br>2889955<br>2889956<br>2889957<br>2889958<br>2889959<br>2889960<br>2889961<br>2889962<br>2889963<br>2889964<br>2889965<br>2889966<br>2889967<br>2889968<br>2889969<br>2889970<br>2889971<br>2889972<br>2889973<br>2889974<br>2889975<br>2889976<br>2889977<br>2889978<br>2889979<br>2889980<br>2889981<br>2889982<br>2889983<br>2889984	full<br>core<br>full<br>core<br>core<br>core<br>full<br>full<br>core<br>full<br>core<br>core<br>core<br>full<br>full<br>core<br>extended<br>extended<br>full<br>extended<br>core<br>core<br>core<br>full<br>core<br>core<br>core<br>full<br>extended<br>full<br>full<br>full<br>full<br>full<br>full<br>full<br>core<br>full<br>full<br>full<br>full<br>extended<br>extended<br>full<br>full<br>extended<br>full<br>full<br>full<br>core<br>full<br>full<br>full<br>full						

	358765<br>358766<br>358767<br>358768<br>358769<br>358770<br>358771<br>358772<br>358773<br>358774<br>358775<br>358776<br>358777<br>358778<br>358779<br>358780<br>358781<br>358782<br>358783<br>358784<br>358785	2889985<br>2889986<br>2889987<br>2889988<br>2889989<br>2889990<br>2889991<br>2889992<br>2889993<br>2889994<br>2889995<br>2889996<br>2889997<br>2889998<br>2889999<br>2890000<br>2890001<br>2890002<br>2890003<br>2890004<br>2890005<br>2890006<br>2890007<br>2890008<br>2890009<br>2890010<br>2890011<br>2890012<br>2890013<br>2890014<br>2890015<br>2890016<br>2890017<br>2890018<br>2890019<br>2890020<br>2890021<br>2890022<br>2890023	full<br>full<br>full<br>full<br>full<br>extended<br>full<br>full<br>full<br>extended<br>full<br>extended<br>extended<br>full<br>full<br>core<br>full<br>full<br>full<br>full<br>full<br>full<br>full<br>full<br>core<br>full<br>full<br>core<br>full<br>full<br>full<br>full<br>full<br>full<br>full<br>full<br>full<br>full<br>full						
2891556	359652<br>359653<br>359654<br>359655<br>359656<br>359657<br>359658<br>359659<br>359660<br>359661<br>359662<br>359663<br>359664<br>359665<br>359666	2891557<br>2891558<br>2891559<br>2891560<br>2891561<br>2891562<br>2891563<br>2891564<br>2891565<br>2891566<br>2891567<br>2891568<br>2891569<br>2891570<br>2891571	full<br>full<br>full<br>full<br>full<br>full<br>full<br>full<br>full<br>full<br>full<br>full<br>full<br>extended<br>full	6	1051225	1285512	+	NM_033260<br>ENST00000296839<br>ENST00000380899<br>GENSCAN00000058908<br>GENSCAN00000027934	Homo sapiens forkhead box Q1 (FOXQ1), mRNA.<br>cdna:known-ccds chromosome:NCBI36:6:1257675:1259981:1 gene:ENSG00000164379 CCDS4471.1<br>cdna:known chromosome:NCBI36:6:1257708:1259422:1 gene:ENSG00000164379<br>cdna:Genscan chromosome:NCBI36:6:1257940:1259151:1<br>cdna:Genscan chromosome:NCBI36:6:1211220:1229657:1

	359667<br>359668<br>359669<br>359670<br>359671<br>359672<br>359673<br>359674<br>359675<br>359676<br>359677<br>359678<br>359679<br>359680<br>359681<br>359682<br>359683<br>359684<br>359685<br>359686<br>359687<br>359688<br>359689<br>359690<br>359691<br>359692<br>359693<br>359694<br>359695<br>359696<br>359697<br>359698<br>359699<br>359700<br>359701	2891572<br>2891573<br>2891574<br>2891575<br>2891576<br>2891577<br>2891578<br>2891579<br>2891580<br>2891581<br>2891582<br>2891583<br>2891584<br>2891585<br>2891586<br>2891587<br>2891588<br>2891589<br>2891590<br>2891591<br>2891592<br>2891593<br>2891594<br>2891595<br>2891596<br>2891597<br>2891598<br>2891599<br>2891600<br>2891601<br>2891602<br>2891603<br>2891604<br>2891605<br>2891606<br>2891607<br>2891608<br>2891609<br>2891610<br>2891611<br>2891612<br>2891613<br>2891614<br>2891615<br>2891616<br>2891617<br>2891618<br>2891619<br>2891620<br>2891621<br>2891622	full<br>full<br>full<br>full<br>full<br>full<br>full<br>full<br>full<br>full<br>full<br>full<br>full<br>full<br>full<br>full<br>full<br>full<br>full<br>full<br>full<br>full<br>full<br>full<br>full<br>full<br>full<br>full<br>full<br>full<br>full<br>full<br>full<br>extended<br>core<br>core<br>core<br>core<br>core<br>core<br>core<br>core<br>core<br>core<br>core<br>core<br>full<br>full<br>full<br>full<br>full						
2897899	363651<br>363652<br>363653	2897900<br>2897901<br>2897902	extended<br>core<br>core	6	21700979	21706814	+	NM_003107<br>X65661<br>ENST00000378570	Homo sapiens SRY (sex determining region Y)-box 4 (SOX4), mRNA.<br>H.sapiens Sox-4 mRNA.<br>cdna:known-ccds chromosome:NCBI36:6:21700979:21706829:1 gene:ENSG00000124766 CCDS4547.1

		2897903<br>2897904<br>2897905<br>2897906<br>2897907<br>2897908<br>2897909<br>2897910<br>2897911<br>2897912<br>2897913<br>2897914<br>2897915<br>2897916<br>2897917<br>2897918	core<br>core<br>core<br>core<br>core<br>core<br>core<br>core<br>core<br>core<br>core<br>core<br>core<br>core<br>core<br>core					ENST00000244745<br>GENSCAN00000002043	cdna:known chromosome:NCBI36:6:21701951:21706826:1 gene:ENSG00000124766<br>cdna:Genscan chromosome:NCBI36:6:21702745:21704169:1
2907671	369267<br>369268<br>369269<br>369270<br>369271<br>369272<br>369273<br>369274<br>369275<br>369276<br>369277<br>369278<br>369279<br>369280<br>369281<br>369282<br>369283<br>369284<br>369285<br>369286<br>369287<br>369288<br>369289<br>369290<br>369291<br>369292<br>369293<br>369294<br>369295<br>369296<br>369297<br>369298<br>369299<br>369300<br>369301<br>369302<br>369303<br>369304	2907672<br>2907673<br>2907674<br>2907675<br>2907676<br>2907677<br>2907678<br>2907679<br>2907680<br>2907681<br>2907682<br>2907683<br>2907684<br>2907685<br>2907686<br>2907687<br>2907688<br>2907689<br>2907690<br>2907691<br>2907692<br>2907693<br>2907694<br>2907695<br>2907696<br>2907697<br>2907698<br>2907699<br>2907700<br>2907701<br>2907702<br>2907703<br>2907704<br>2907705<br>2907706<br>2907707<br>2907708<br>2907709	core<br>core<br>full<br>full<br>full<br>full<br>full<br>full<br>extended<br>full<br>extended<br>full<br>full<br>full<br>full<br>core<br>extended<br>core<br>core<br>core<br>full<br>core<br>core<br>core<br>core<br>core<br>core<br>full<br>core<br>core<br>core<br>core<br>core<br>core<br>extended<br>core<br>extended<br>core	6	43152004	43237427	+	NM_152880<br>NM_152881<br>NM_152882<br>NM_002821<br>AK093428<br>AK131487<br>BC046109<br>ENSESTT00000056719<br>ENSESTT00000056720<br>ENSESTT00000056721<br>ENSESTT00000056722<br>ENST00000230419<br>ENST00000345201<br>ENST00000352931<br>ENST00000230418<br>ENST00000349241<br>ENST00000325774<br>ENST00000359792<br>GENSCAN00000029527	Homo sapiens PTK7 protein tyrosine kinase 7 (PTK7), transcript variant PTK7-2, mRNA.<br>Homo sapiens PTK7 protein tyrosine kinase 7 (PTK7), transcript variant PTK7-3, mRNA.<br>Homo sapiens PTK7 protein tyrosine kinase 7 (PTK7), transcript variant PTK7-4, mRNA.<br>Homo sapiens PTK7 protein tyrosine kinase 7 (PTK7), transcript variant PTK7-1, mRNA.<br>Homo sapiens cDNA FLJ36109 fis, clone TESTI2021911, weakly similar to TYROSINE-PROTEIN KINASE-LIKE 7 PRECURSOR.<br>Homo sapiens cDNA FLJ16667 fis, clone THYMU2035710, highly similar to TYROSINE-PROTEIN KINASE-LIKE 7 PRECURSOR.<br>Homo sapiens PTK7 protein tyrosine kinase 7, mRNA (cDNA clone IMAGE:5551146), complete cds.<br><br><br><br><br>cdna:known-ccds chromosome:NCBI36:6:43152007:43237435:1 gene:ENSG00000112655 CCDS4884.1<br>cdna:known-ccds chromosome:NCBI36:6:43152007:43237435:1 gene:ENSG00000112655 CCDS4885.1<br>cdna:known-ccds chromosome:NCBI36:6:43152007:43237435:1 gene:ENSG00000112655 CCDS4887.1<br>cdna:known-ccds chromosome:NCBI36:6:43152007:43237435:1 gene:ENSG00000112655 CCDS4888.1<br>cdna:known-ccds chromosome:NCBI36:6:43152007:43237435:1 gene:ENSG00000112655 CCDS4886.1<br>cdna:known chromosome:NCBI36:6:43152007:43237435:1 gene:ENSG00000112655<br>cdna:novel chromosome:NCBI36:6:43152007:43237435:1 gene:ENSG00000112655<br>cdna:Genscan chromosome:NCBI36:6:43152205:43236597:1

	369305<br>369306<br>369307	2907710<br>2907711<br>2907712<br>2907713<br>2907714<br>2907715<br>2907716<br>2907717<br>2907718<br>2907719<br>2907720<br>2907721	extended<br>core<br>extended<br>extended<br>core<br>core<br>extended<br>core<br>core<br>core<br>core<br>core						
2923868	379326<br>379327<br>379328<br>379329<br>379330<br>379331<br>379332<br>379333<br>379334<br>379335<br>379336<br>379337<br>379338<br>379339<br>379340<br>379341<br>379342<br>379343<br>379344<br>379345<br>379346<br>379347<br>379348<br>379349<br>379350<br>379351<br>379352<br>379353<br>379354<br>379355<br>379356<br>379357<br>379358<br>379359<br>379360<br>379361<br>379362<br>379363<br>379364<br>379365<br>379366	2923869<br>2923870<br>2923871<br>2923872<br>2923873<br>2923874<br>2923875<br>2923876<br>2923877<br>2923878<br>2923879<br>2923880<br>2923881<br>2923882<br>2923883<br>2923884<br>2923885<br>2923886<br>2923887<br>2923888<br>2923889<br>2923890<br>2923891<br>2923892<br>2923893<br>2923894<br>2923895<br>2923896<br>2923897<br>2923898<br>2923899<br>2923900<br>2923901<br>2923902<br>2923903<br>2923904<br>2923905<br>2923906<br>2923907<br>2923908<br>2923909	core<br>core<br>extended<br>extended<br>extended<br>extended<br>full<br>extended<br>core<br>extended<br>extended<br>extended<br>full<br>core<br>full<br>full<br>extended<br>extended<br>full<br>core<br>extended<br>extended<br>extended<br>full<br>full<br>full<br>extended<br>extended<br>extended<br>extended<br>extended<br>extended<br>extended<br>extended<br>core<br>extended<br>extended<br>extended<br>extended<br>full<br>full	6	122834771	123120381	+	NM_181795<br>NM_181794<br>NM_032471<br>AF225513<br>AK026221<br>AK074397<br>AJ420562<br>CR749456<br>ENSESTT00000010766<br>ENSESTT00000010767<br>ENST00000368452<br>ENST00000368448<br>ENST00000368451<br>ENST00000258014<br>ENST00000354275<br>ENST00000368446<br>GENSCAN00000056024<br>GENSCAN00000056025	Homo sapiens protein kinase (cAMP-dependent, catalytic) inhibitor beta (PKIB), transcript variant 1, mRNA.<br>Homo sapiens protein kinase (cAMP-dependent, catalytic) inhibitor beta (PKIB), transcript variant 2, mRNA.<br>Homo sapiens protein kinase (cAMP-dependent, catalytic) inhibitor beta (PKIB), transcript variant 3, mRNA.<br>Homo sapiens cAMP-dependent protein kinase inhibitor beta mRNA, complete cds.<br>Homo sapiens cDNA: FLJ22568 fis, clone HSI02138.<br>Homo sapiens cDNA FLJ23817 fis, clone HSI07950.<br>Homo sapiens mRNA full length insert cDNA clone EUROIMAGE 1525273.<br>Homo sapiens mRNA; cDNA DKFZp781K1114 (from clone DKFZp781K1114).<br><br><br>cdna:known-ccds chromosome:NCBI36:6:122973076:123089216:1 gene:ENSG00000135549 CCDS5126.1<br>cdna:known chromosome:NCBI36:6:122973076:123088736:1 gene:ENSG00000135549<br>cdna:known chromosome:NCBI36:6:122973076:123088736:1 gene:ENSG00000135549<br>cdna:known chromosome:NCBI36:6:123015576:123089217:1 gene:ENSG00000135549<br>cdna:known chromosome:NCBI36:6:123080405:123088736:1 gene:ENSG00000135549<br>cdna:known chromosome:NCBI36:6:123080406:123088125:1 gene:ENSG00000135549<br>cdna:Gensc

	379367<br>379368<br>379369	2923910<br>2923911<br>2923912<br>2923913<br>2923914<br>2923915<br>2923916<br>2923917<br>2923918<br>2923919<br>2923920<br>2923921<br>2923922<br>2923923<br>2923924	full<br>extended<br>full<br>extended<br>extended<br>core<br>full<br>core<br>core<br>core<br>extended<br>extended<br>full<br>full<br>full						
